# In‐Depth Examination of TPBG as a New Predictive Indicator for Gastric Cancer

**DOI:** 10.1111/jcmm.70354

**Published:** 2025-01-17

**Authors:** Lianlei Yang, Chunyan Weng, Yaping Zhang, Yu Zhao, Kexin Chen, Guodong Li, Xueqing Zhong, Chenghai He

**Affiliations:** ^1^ Department of Gastroenterology The First People's Hospital of Linping District Hangzhou Zhejiang Province China; ^2^ The First Clinical Medical of Zhejiang Chinese Medical University Hangzhou Zhejiang China; ^3^ Department of Gastroenterology The Affiliated Hospital of Hangzhou Normal University Hangzhou Zhejiang Province China

**Keywords:** biomarker, gastric cancer, immune infiltration, PI3K/AKT signalling pathway, prognosis, TPBG

## Abstract

Trophoblast glycoprotein (TPBG) plays a significant part in the growth of specific cancers, yet its connection to gastric cancer (GC) remains uncertain. This research seeks to analyse the fluctuation in TPBG levels in GC and evaluate how TPBG expression relates to the prognosis of GC patients. TPBG expression in GC and normal gastric tissues was investigated in The Cancer Genome Atlas (TCGA) and Genotype‐Tissue Expression (GTEx) database, further extracting the immunohistochemistry images from HPA database and validating by Western blot. The connection between TPBG and GC patients' survival rates was investigated by Kaplan–Meier and COX regression analysis. Genes related to TPBG were enriched using GO and KEGG data. In vitro and in vivo tumour models were utilised to evaluate the function of TPBG in GC. Western blot analysis was performed to detect the expression of PI3K/AKT signalling pathway proteins following TPBG knockdown. Immune infiltration was analysed using the CIBERSOFT and ssGSEA methods. The association between TPBG and immune cells that infiltrate tumours was evaluated through the utilisation of GSVA. TPBG expression increased in several tumour tissues (including GC) more than in adjacent noncancerous tissues. Elevated TPBG level predicted worse outcomes, such as poorer overall survival, pathological stage, and therapy response in GC. Enrichment analysis primarily focused on biological processes like the organisation of external encapsulating structures, extracellular structure, and collagen metabolism. Biological experiments further demonstrated that TPBG knockdown successfully inhibits the progression, migration, and invasion of GC cells. Western blot analysis revealed that TPBG knockdown inhibits the PI3K/AKT signalling pathway. Furthermore, TPBG is associated with the infiltration of immune cells in GC, which correlates with the expression of macrophage cells. There is a positive relationship between TPBG and malignant behaviour of GC tissues and cells, suggesting that TPBG can be useful for diagnosing and prognosing GC.

## 1 Introduction

According to GLOBOCAN, gastric cancer (GC) ranked fifth in cancer incidence and mortality in 2022 [1]. According to statistics in 2022, there were 968,350 new instances of GC and 659,853 fatalities linked to GC. GC presents with clinical symptoms such as indigestion, loss of appetite, feeling full quickly after eating, vomiting, stomach discomfort, and weight loss [2]. Unfortunately, the disease frequently advances to a late stage with distant metastasis upon symptom presentation, placing a significant strain on public healthcare. Hence, discovering efficient diagnostic and prognostic indicators could enhance the existing treatment approach for individuals with GC, ultimately extending their lifespan.

Trophoblast glycoprotein (TPBG), also known as 5T4, is a transmembrane glycoprotein that has been the subject of thorough research as an oncofetal antigen [3]. Present in small quantities in typical adult tissues, TPBG is prevalent in various human cancers [4, 5]. Extensive research has indicated that the TPBG oncotrophoblast antigen is linked to numerous cell functions, including cell migration, enhanced motility, morphological changes, and membrane integrity [6]. In the early stages, TPBG conferred epithelial‐to‐mesenchymal transition (EMT) capability to tumour cells, followed by apoptosis and proliferation of tumour cells [5]. In addition, TPBG expression is to cause vascular remodelling in the tumour's microenvironment, which further accentuates tumour growth and invasion [7]. TPBG is suggested to have a dual function in various biological processes. However, the relationship between TPBG and GC has not been clearly elucidated yet.

This study examined the TPBG expression levels and the relationship between TPBG expression and clinicopathological characteristics of GC patients by utilising data from public databases. We determined that TPBG was an independent prognostic factor for GC by multiple analyses, such as KM, COX regression, and enrichment analyses. Knockdown of TPBG inhibits the progression and metastasis of GC cells, as well as the PI3K/AKT signalling pathway. Further investigation found that TPBG of GC influenced the immune cell infiltration rates in the tumour microenvironment. These results indicate that TPBG acts as a key in the development of GC and could potentially serve as a prognosticator and treatment guide for GC.

## 2 Materials and Methods

### 2.1 Downloading and Analysing Data

Clinical information concerning TPBG expression was obtained from The Cancer Genome Atlas (TCGA) and Genotype‐Tissue Expression (GTEx) databases. TPM values provided in the TCGA database are pre‐normalised to allow for cross‐sample comparisons. From the cBioPortal for Cancer Genomics, we obtained information about TPBG gene amplification and mutation status. A human protein Atlas (HPA) database was searched to verify the TPBG protein expression in multiple cancers and obtain images of the expression of TPBG in GC using immunofluorescence.

### 2.2 Survival Analysis

TPBG levels and patient clinical features in gastric adenocarcinoma (STAD) were analysed by data from the GEPIA and TCGA databases. The R package of ‘survival’ (version 3.6) was used to produce an overall survival (OS) map for TPBG. A 50% threshold value was selected as the dividing point to separate the samples into TPBG high or low expression groups. The results were visualised with ggplot2.

### 2.3 Development and Assessment of Predictive Nomogram

The OS probability of patients with STAD was assessed by constructing a line plot based on all independent clinicopathological prognostic parameters from a Cox regression analysis. Calibrating curves were used to determine the accuracy of the nomogram by comparing the predicted probabilities with the observed actual probabilities. There is overlap between the reference lines, indicating that the model is accurate. A nomogram was generated using RMS and survival packages. A bootstrap approach was used to determine the discrimination of the nomogram, which was calculated based on 1000 resamples of the nomogram.

### 2.4 Exploring the Relationship and Analysing the Enrichment of Gene Sets

Correlation analyses were conducted using data from TCGA to explore the biological functions in which TPBG can play a role by examining its relationship with other mRNAs in GC. Kyoto Encyclopaedia of Genes and Genomes (KEGG) pathway analysis was carried out using the clusterProfiler package in R language to analyse co‐expressed genes.

### 2.5 Cell Culture and Transfection

GC cell lines (NUGC‐3 and MNK‐74) and a normal gastric epithelial cell line (Ges‐1) were purchased from Hangzhou Jenker Biological Technology Co. Ltd. Cells were cultured with 10% FBS in RPMI‐1640 medium at 37°C with 5% CO_2_. Short hairpin RNAs (shRNAs) were utilised in TPBG knockdown experiments, with expression controlled by the human H1 promoter.siDirect version 2.0 was used to design three different sets of shRNAs that target the TPBG gene. Three different groups of shRNAs were inserted into the lentiviral vector as follows: sh1, GGTATCATTACAGATACGATTCAAGAGATCGTATCTGTAATGATACCCTTTTTT; sh2, GACTTTGATCCTTGTTATGTATTCAAGAGATACATAACAAGGATCAAAGTCTTTTT; and sh3, GCTCTTACATAGAACTTTGTATTCAAGAGATACAAAGTTCTATGTAAGAGCTTTTT.

### 2.6 Western Blot

Ten pairs of carcinoma and adjacent normal tissues were collected between July 2022 and November 2023 from GC patients at The Affiliated Hospital of Hangzhou Normal University. Patients were informed about the experiment and consented to it, which was approved by the Ethics Committee (2022‐E2‐HS‐097). Tissue protein extraction methods included tissue clipping, homogenisation with a tissue homogeniser, lysis, protein collecting, electrophoresis, gel cutting, membrane transfer, milk closure, addition of antibodies of TPBG (Proteintech, #29394‐1‐AP), PI3K (Proteintech, #60225‐1‐Ig), p‐PI3K (CST, #4228 T), AKT (Proteintech, #60203‐2‐Ig), p‐AKT (Proteintech, #28731‐1‐AP), and GAPDH (Proteintech, #60004‐1‐Ig), overnight incubation at 4°C, membrane washing, addition of goat anti‐rabbit IgG secondary antibody, 90 min incubation at 37°C, ECL luminescence kit development, and expression level analysis of TPBG, PI3K, p‐PI3K, AKT and p‐AKT with GAPDH as the control.

### 2.7 Colony Formation, Wound Healing, and Transwell Assay

For the colony formation assay, 1000 NUGC‐3 cells transfected with different shRNA plasmids were seeded into a 6‐well plate. Cells were fixed and stained 14 days later. In the wound‐healing assay, a total of 10,000 cells were initially seeded into culture inserts within a 6‐well plate. Once the cells had adhered to the plate, the culture inserts were carefully removed and replaced with a 1% FBS medium. Subsequently, images were captured using a microscope. For the transwell assay, 3 × 10^4^ cells were seeded in transwell chambers (Corning, USA) with 200 μL basic medium (pre‐spread Matrigel or not), whereas 600 μL medium with 10% FBS was added to the lower chamber. Cells in the transwell chambers were fixed and stained 24 h later. The cells on top of the chamber membrane should be wiped off, and invaded cells photographed under a microscope using a magnification of 100X.

### 2.8 Immune Infiltration Analysis

The relationship between TPBG and 24 types of immune cells was evaluated with the GSVA tool in R. Furthermore, TISIDB, an online platform for studying interactions between tumours and the immune system, was employed to exhibit the infiltration of immune cells and corresponding molecular subtypes in different TPBG expression levels [8].

### 2.9 Xenograft Tumour Growth

Mouse‐related procedures were in compliance with ethical regulations and approved by The Animal Ethical and Welfare Committee at Zhejiang Chinese Medical University. Nude mice (male, 5 weeks) were purchased from Shanghai BK Company. To examine the effects of TPBG, 4 × 10^6^ NUGC3 cells that stably expressed pLKO and sh TPBG were inoculated subcutaneously into the right armpit of mice. Longest dimensions (LD) and shortest dimensions (SD) of tumours were measured twice a week (tumour volume = LD × SD^2^/2). To further analyse the tumours, the mice were euthanised 4 weeks after feeding.

### 2.10 Statistical Analysis

The study utilised box line plots and scatter plots to assess TPBG gene expression in patients with GC. Median gene expression was employed to establish the cut‐off value for TPBG expression. To explore the relative TPBG‐related genes, Spearman correlation analysis was conducted. The Cox analysis has been used to identify the prognostic factors on both the univariate and multivariate levels. Statistical significance levels were denoted by *, **, and ***, indicating values less than 0.05, 0.01, and 0.001, respectively.

## 3 Results

### 3.1 TPBG Is Highly Expressed in GC

**FIGURE 1:**
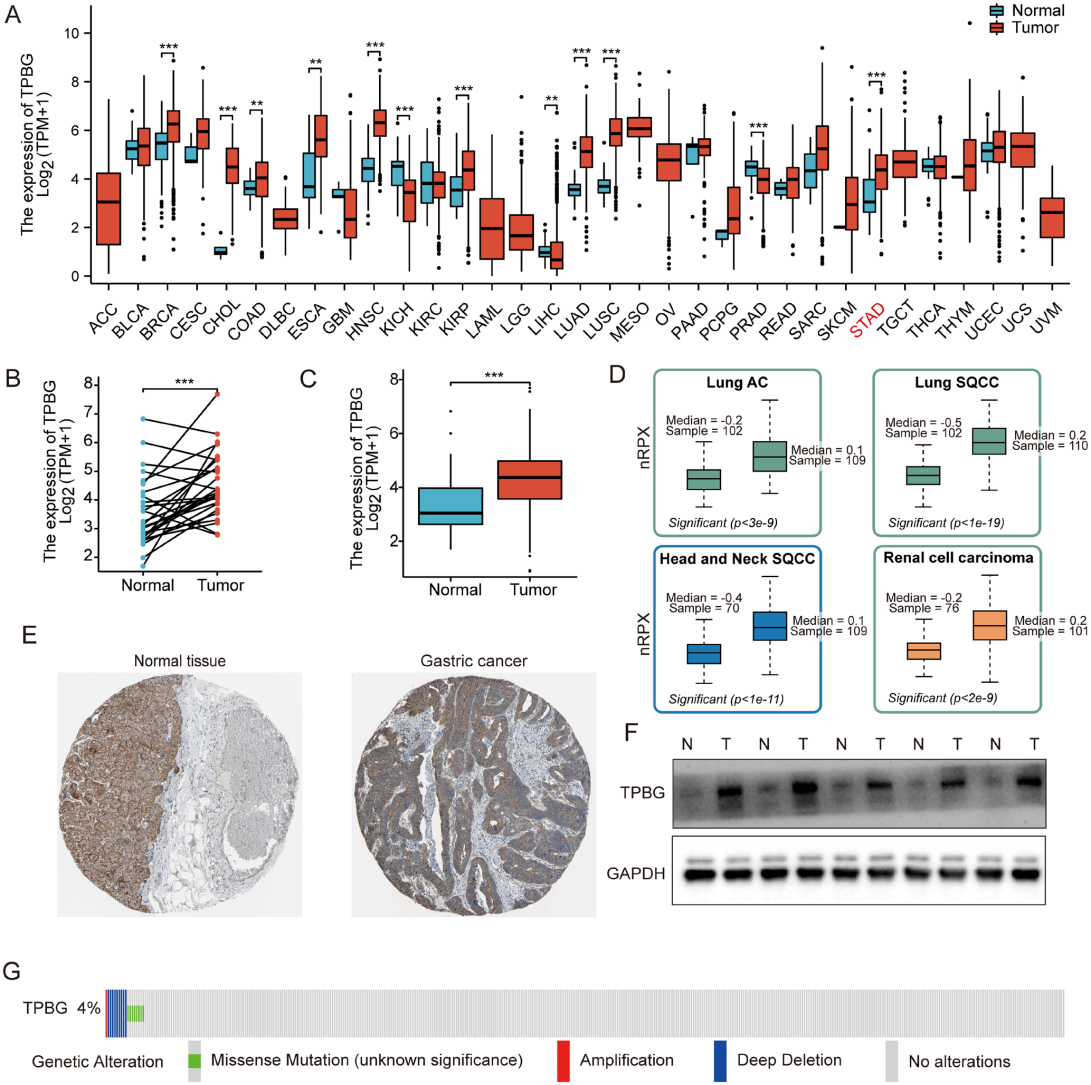
The expression of TPBG in GC. (A) TPBG expression in multiple tumour and normal tissues from TCGA and GTEx databases. (B, C) Comparison of TPBG expression between GC tissues and their matched or unmatched normal gastric tissues from the TCGA database. (D) The protein expression of TPBG in Lung AC, Lung SQCC, Head and Neck SQCC, and Renal cell carcinoma in the HPA database. (E) The HPA database provided immunohistochemistry stainings for TPBG in GC and normal tissues. (F) Protein expression of TPBG was detected in GC and normal tissues by western blot. (G) The OncoPrint graph of STAD patients shows the distribution of TPBG genomic changes. The meaning of cancer abbreviations is shown in Table 2. ** and *** indicate *p* < 0.01, and *p* < 0.001.

Data downloaded as previously described. Significant differences in TPBG expression were observed across different tumour types, as depicted in Figure 1A. Given TPBG's involvement in various cancers, we aimed to investigate its significance in GC as well. The TPBG mRNA from the database was compared between cancerous and adjacent samples of STAD patients, revealing an obvious increase in tumour samples (*p* < 0.001) (Figure 1B,C). After extracting the protein expression from the HPA database [9], we discovered that the levels of TPBG signals were increased in multiple cancers, including Lung AC, Lung SACC, Head and Neck SQCC, and Renal Cell Carcinoma (Figure 1D). Furthermore, TPBG protein levels in stomach tissues were lower compared to stomach cancer tissues (Figure 1E). The protein expressions of TPBG in 5 sets of cancerous and adjacent tissues were employed by western blot. Compared to the paracancerous tissues, the level of TPBG protein is significantly elevated in GC tissues, as shown in Figure 1F. To understand the mutation level, we examined the genome and copy number of TPBG in STAD through OncoPrint mapping using cBioPortal plots. As shown in Figure 1G, TPBG displayed mutations including less than 3% gene amplification, missense mutations, and deep deletions. Overall, these results uncovered the overexpression of TPBG in GC.

### 3.2 TPBG Expression Associated With Clinicopathological Features in GC

**FIGURE 2:**
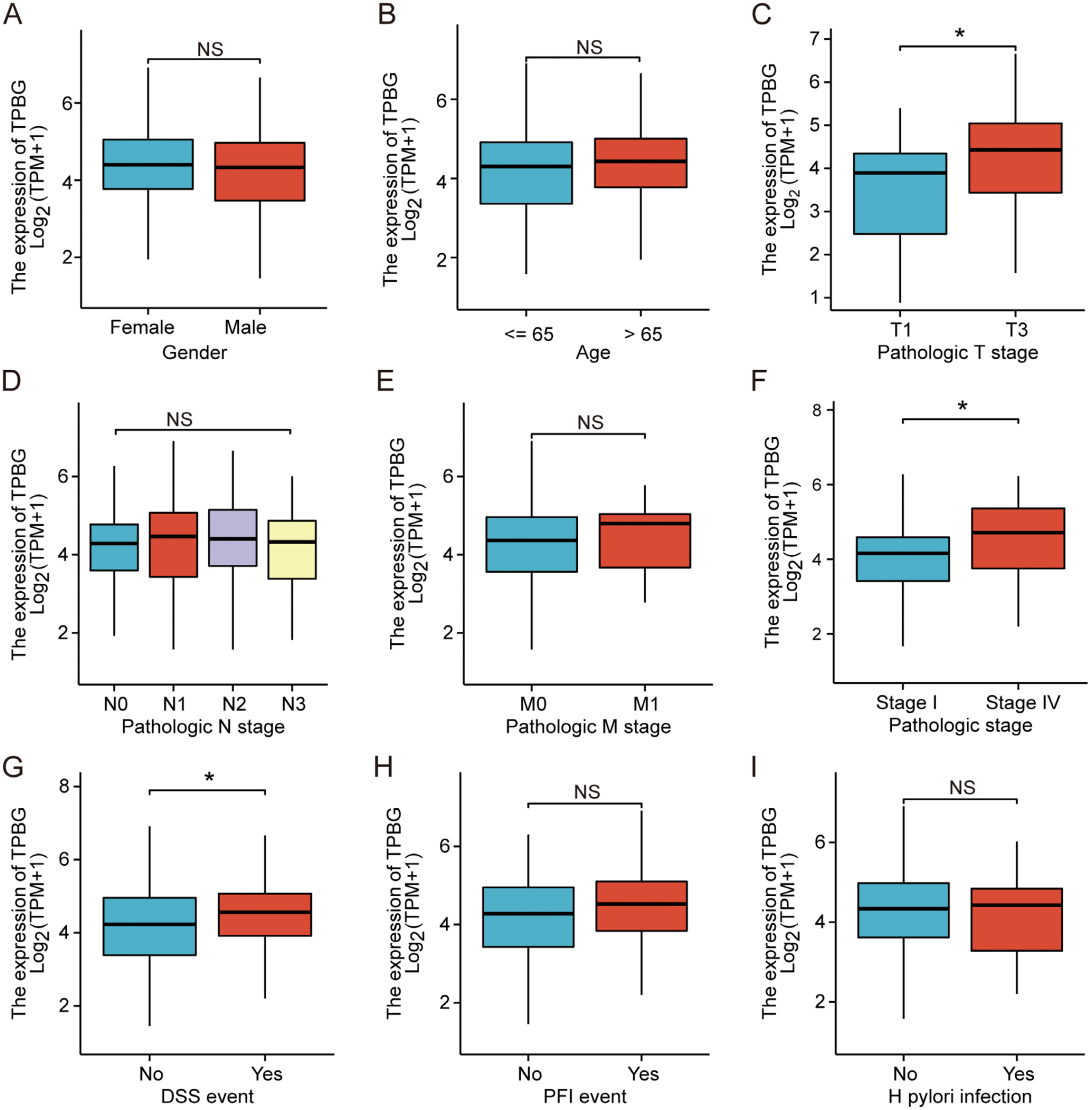
Expression levels of TPBG across different groups of various clinical characteristics. (A) Gender, (B) Age, (C) T‐stage, (D) N‐stage, (E) M‐stage, (F) Pathological stage, (G) DSS event, (H) PFI event, (I) *H. pylori* infection. *indicate *p* < 0.05.

The association between TPBG expression and clinical features was analysed using the TCGA database of STAD. This study examined how TPBG expression correlates with nine clinicopathological factors in GC patients, including gender, age, *H. pylori* infection, T or N or M stage, pathological stage, disease‐specific survival (DSS), and Progression Free Interval (PFI). As exhibited in Figure 2, a tight relationship between elevated TPBG expression and T stage (*p* < 0.05) (Figure 2C), pathological stage (*p* < 0.05) (Figure 2F), and DSS events (*p* < 0.05) (Figure 2G) was demonstrated in GC patients. However, no significant differences in TPBG expression were found across other variables such as gender (Figure 2A), age (Figure 2B), N (Figure 2D) and M (Figure 2E) stages, PFI outcomes (Figure 2H), or HP infection status (Figure 2I). These findings indicated a significant increase in TPBG expression in STAD and its correlation with various clinical characteristics.

### 3.3 Elevated TPBG Levels Impact the Outlook of GC Patients Across Various Clinicopathological Conditions

**FIGURE 3:**
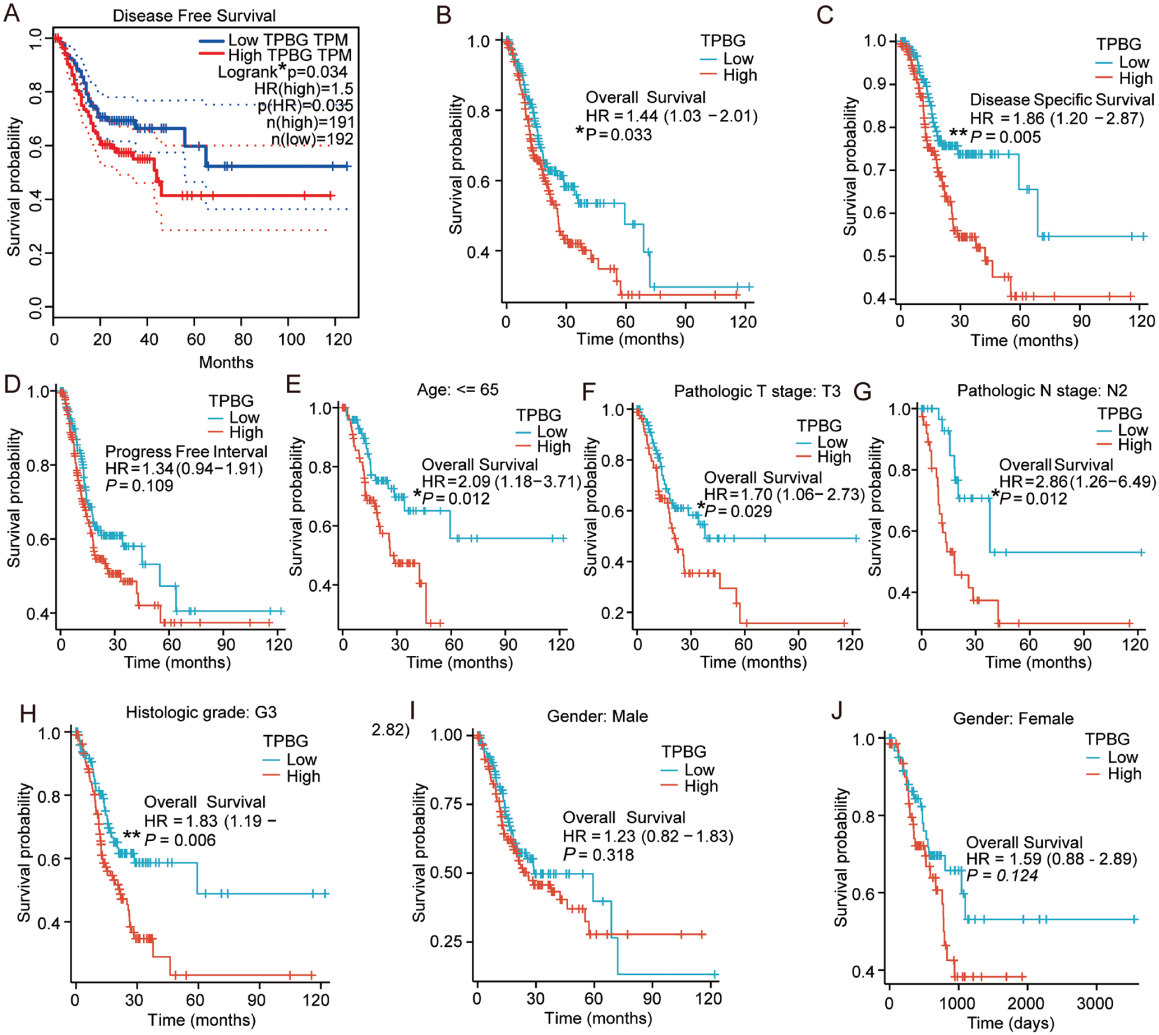
The association between TPBG and the prognosis of GC patients. (A, B) The DFS and OS analysis of STAD patients between high‐expressed and low‐expressed TPBG groups in the GEPIA database. (C, D) The DSS and PFI analysis of STAD patients between high‐expressed and low‐expressed TPBG groups in the TCGA database. (E–J) The OS of STAD patients with high and low TPBG expression levels is analysed across various clinically (E, I, and J) and pathologically distinct subgroups (F–H). * and ** indicate p < 0.05 and p < 0.01.

Based on the ranking of TPBG expression levels, we categorised the GC cases from the TCGA database into two groups: the high expression group (top 50% cases) and the low expression group (remaining 50% cases). Subsequent to this, survival analysis was conducted utilising the average TPBG expression level. In GC, high TPBG expression exhibited a worse prognosis in terms of disease‐free survival (DFS) according to Kaplan–Meier (KM) survival analysis (HR = 1.5, *p* = 0.035, Figure 3A). Furthermore, highly expressed TPBG predicted poorer overall survival (OS) (HR = 1.44, *p* = 0.033, Figure 3B) and DSS (HR = 1.86, *p* = 0.005, Figure 3C). However, no significant association was found with PFI (HR = 1.34, *p* = 0.109, Figure 3D). Subgroup analyses revealed a strong link between elevated TPBG levels and unfavourable outcomes in STAD patients younger than 65 years old (HR = 2.09, *p* = 0.012), with T3 tumours (HR = 1.70, *p* = 0.029), N2 lymph node status (HR = 2.86, *p* = 0.012), and histologic grade G3 (HR = 1.83, *p* = 0.006) (Figure 3E–H). Gender did not show a significant association with outcomes of different TPBG levels (Figure 3I,J).

### 3.4 Elevated Levels of TPBG Are a Separate Predictor of Survival Outcomes

STAD was evaluated with univariate and multivariate cox regression models using TPBG, age, gender, tumour size, lymph node, and distant metastasis as significant prognostic indicators. Both types of analysis showed that high TPBG levels were a strong predictor of survival in GC cases (HR = 1.493, 95% CI [1.003–2.064], *p* = 0.048). Furthermore, age (HR = 1.720, 95% CI [1.191–2.484], *p* = 0.004), N stage (HR = 1.661, 95% CI [1.053–2.621], *p* = 0.029), and M stage (HR = 2.368, 95% CI [1.324–4.237], *p* = 0.004) were identified as significant independent prognostic variables (Table 1).

**TABLE 1:** Univariate and multivariate Cox regression analyses of clinical characteristics associated with OS of STAD in TCGA.

Characteristics		Total (N)	Univariate analysis		Multivariate analysis	
			Hazard ratio (95% CI)	*p*	Hazard ratio (95% CI)	*p*
Gender		370				
	Female	133	Reference			
	Male	237	1.267 (0.891–1.804)	0.188		
Age		367				
	<= 65	163	Reference		Reference	
	> 65	204	1.620 (1.154–2.276)	0.005	1.720 (1.191–2.484)	0.004
Pathologic T stage		362				
	T1&T2	96	Reference		Reference	
	T3&T4	266	1.719 (1.131–2.612)	0.011	1.329 (0.839–2.106)	0.225
Pathologic N stage		352				
	N0	107	Reference		Reference	
	N1&N3&N2	245	1.925 (1.264–2.931)	0.002	1.661 (1.053–2.621)	0.029
Pathologic M stage		352				
	M0	327	Reference		Reference	
	M1	25	2.254 (1.295–3.924)	0.004	2.368 (1.324–4.237)	0.004
TPBG		370				
	Low	182	Reference		Reference	
	High	188	1.437 (1.030–2.005)	0.033	1.439 (1.003–2.064)	0.048

**TABLE 2:** The meaning of abbreviations for cancers.

Abbreviation	Amplify
ACC	Adrenocortical carcinoma
BLCA	Bladder urothelial carcinoma
BRCA	Breast invasive carcinoma
CESC	Cervical squamous cell carcinoma and endocervical adenocarcinoma
CHOL	Cholangiocarcinoma
COAD	Colon adenocarcinoma
DLBC	Lymphoid neoplasm diffuse large B‐cell lymphoma
ESCA	Oesophageal carcinoma
GBM	Glioblastoma multiforme
HNSC	Head and neck squamous cell carcinoma
KICH	Kidney chromophobe
KIRC	Kidney renal clear cell carcinoma
KIRP	Kidney renal papillary cell carcinoma
LAML	Acute myeloid leukaemia
LGG	Brain lower grade glioma
LIHC	Liver hepatocellular carcinoma
LUAD	Lung adenocarcinoma
LUSC	Lung squamous cell carcinoma
MESO	Mesothelioma
OV	Ovarian serous cystadenocarcinoma
PAAD	Pancreatic adenocarcinoma
PCPG	Pheochromocytoma and paraganglioma
PRAD	Prostate adenocarcinoma
READ	Rectum adenocarcinoma
SARC	Sarcoma
SKCM	Skin cutaneous melanoma
STAD	Stomach adenocarcinoma
TGCT	Testicular germ cell tumours
THCA	Thyroid carcinoma
THYM	Thymoma
UCEC	Uterine corpus endometrial carcinoma
UCS	Uterine carcinosarcoma
UVM	Uveal melanoma

### 3.5 Creating Predictive Line Charts Using Separate Predictive Variables

**FIGURE 4:**
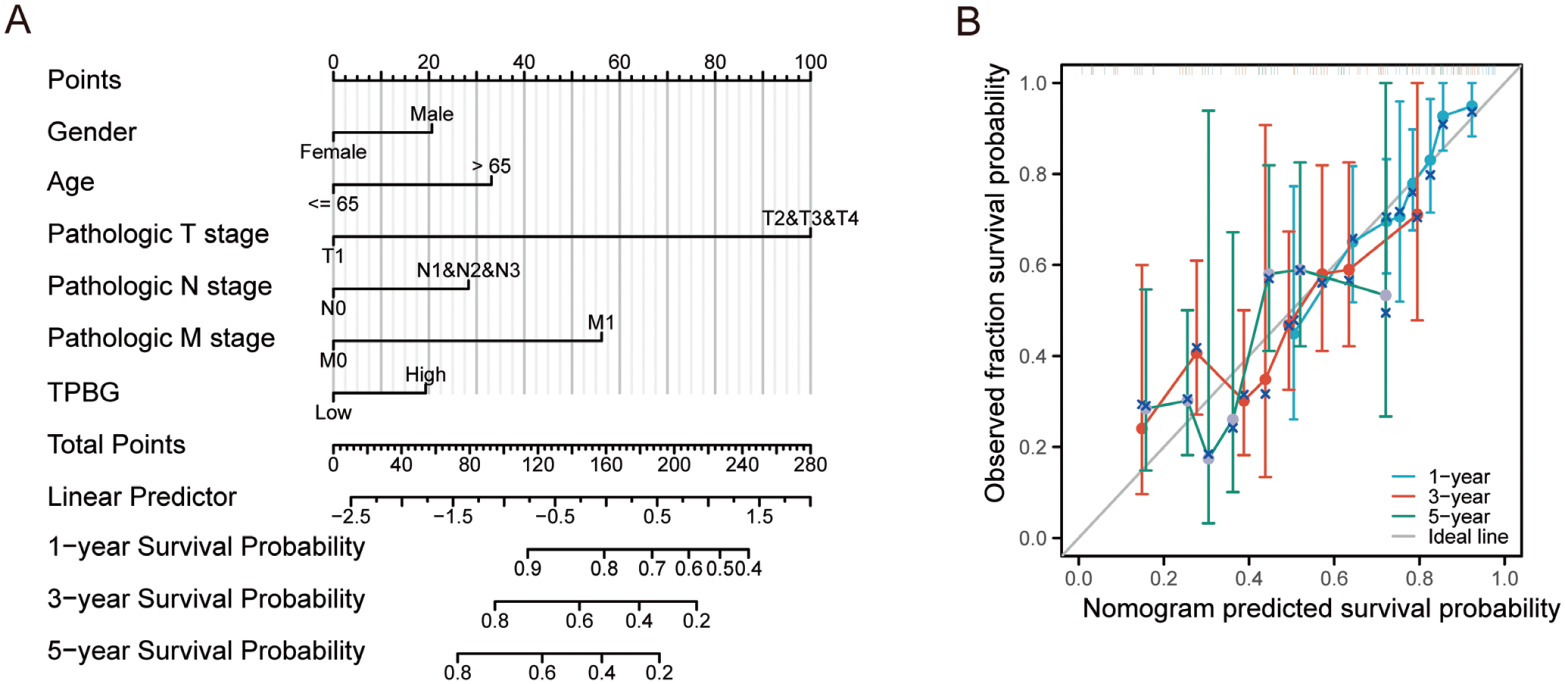
Construction of prognostic line graphs. (A) Nomogram for predicting OS at 1, 3 and 5 years. (B) Calibration curves for 1, 3 and 5 years.

Next, TPBG, age, gender, and T or N or M stage were analysed as separate prognostic variables in order to develop a prognostic nomogram, which accessed the accuracy by generating a calibration curve. Figure 4A visually displayed the correlation between TPBG, age, gender, N or M stage, and the OS at 1‐year, 3‐year, and 5‐year intervals. Furthermore, the calibration curves demonstrated the reliability and the value of our predictions, verifying the efficiency of the TPBG‐derived nomogram (Figure 4B). With a c statistic of 0.658, the predictive accuracy was moderate. Overall, this nomogram could serve as a more effective tool in forecasting the survival of patients with GC when compared to single prognostic indicators.

### 3.6 Functional Enrichment of TPBG‐Related Genes

**FIGURE 5:**
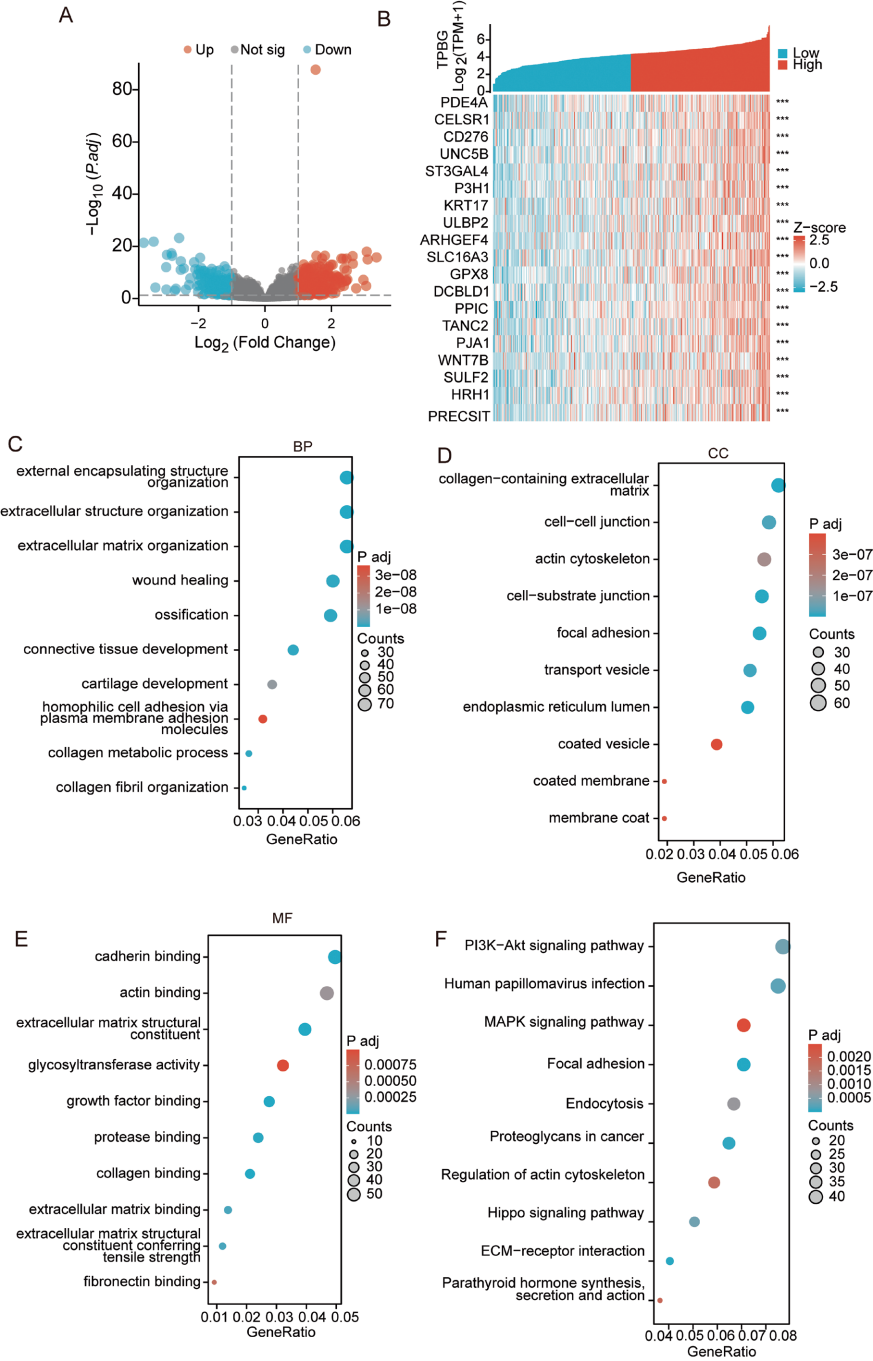
Gene interaction networks and functional clustering of genes related to the TPBG. (A) Volcano map of differential genes in the TPBG high‐expression group compared to the low‐expression group. (B) Heat map showing the top 50 genes positively associated with TPBG in STAD. (C–E) Enrichment analyses of BP (C), CC (D), and MF (E) of TPBG‐correlated genes. (F) KEGG enrichment analyses of correlated TPBG gene terms. *** indicate *p* < 0.001.

With the Limma package, we enriched genes that encode proteins in high‐ and low‐expressed TPBG samples in STAD. With |logFC| > 1 and *p* < 0.05, 797 genes were identified as differentially expressed, with 443 genes showing up‐regulation and 354 genes showing down‐regulation (Figure 5A, Table S1). The clusterProfiler package in R language was used to study the GO terms and KEGG pathway of 797 differentially expressed genes (DEGs). With statistical significance *p* < 0.05 and *q* < 0.05, the DEGs between TPBG high‐expression and low‐expression groups were associated with 143 different biological processes (BPs), 24 cellular components (CCs), 39 molecular functions (MFs), and 13 KEGGs (Table S2). GO and KEGG term assignments showed that DEGs participated in several immune and metabolism‐associated pathways, including humoral immune response, immunoglobulin complex, signalling receptor activator activity, neuroactive ligand−receptor interaction, and Protein digestion and absorption (Figure S1A–D). Furthermore, we examined the co‐expressed TPBG‐related genes in the TCGA database, as shown in Table S3. With Spearman correlation coefficient > 0.3 and *p* < 0.05, 1259 genes were acquired, and top 20 correlated genes were displayed in Figure 5B. Moreover, the genes that were co‐expressed with TPBG were associated with 420 different BPs, 98 CCs, 36 MFs, and 30 KEGGs (Table S4). GO term assignments showed that different genes primarily participated in the organisation of external encapsulating structures, extracellular structures, collagen metabolic processes, collagen‐containing cadherin binding, extracellular matrices, and glycosyltransferase activity (Figure 5C–E). The analysis of KEGG pathways indicated that these genes were primarily connected with multiple pathways like PI3K/Akt, MAPK, focal adhesion, regulation of actin cytoskeleton, and ECM‐receptor interaction (Figure 5F).

### 3.7 Knockdown of TPBG Suppresses GC Cell Progression and Metastasis In Vitro

**FIGURE 6:**
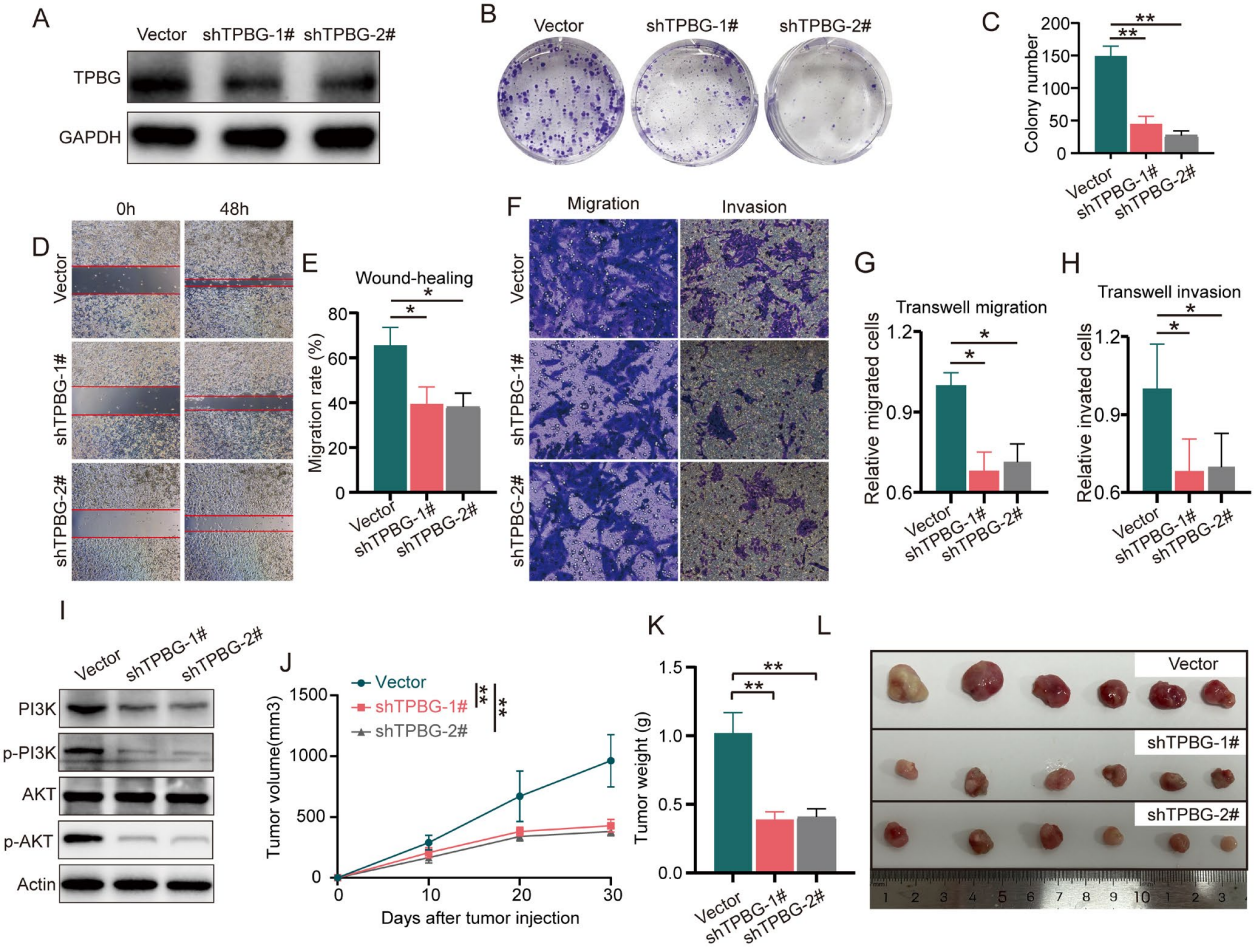
Knockdown of TPBG inhibits the growth and metastasis of GC both in vitro and in vivo. (A) The efficiency of TPBG shRNA lentivirus in NUGC‐3 cells was detected by western blot analysis. (B, C) Colony formation assays and corresponding statistical analyses of NUGC‐3 cells were conducted in vector control and TPBG shRNA groups. (D, E) Wound‐healing assay and corresponding statistical analyses of NUGC‐3 cells were conducted in vector control and TPBG shRNA groups. (F–H) Transwell assays (migration and invasion) and corresponding statistical analyses of NUGC‐3 cells were conducted in vector control and TPBG shRNA groups. (I) The protein expressions of PI3K, p‐PI3K, AKT, p‐AKT, and GAPDH in the vector control and TPBG shRNA groups were detected using western blot analysis. (J) The tumour volumes of xenograft models, both in the vector control and TPBG shRNA groups, were measured and calculated weekly. (K, L) The weights and images of the tumours were measured and recorded at the endpoint of the experiment. * and ** indicate *p* < 0.05 and *p* < 0.01.

To investigate the role of TPBG in GC cells, the efficacy of specific shRNA‐targeted TPBG was initially confirmed. The Western blot findings showed that the TPBG shRNA sequence in lentiviral vectors successfully reduced TPBG expression in NUGC‐3 cells (Figure 6A). In addition, knocking down TPBG led to a notable decrease in cell growth, as shown in the colony formation assay (Figure 6B,C). Furthermore, a wound‐healing assay demonstrated a significant inhibition of migration rate in NUGC‐3 cells transfected with TPBG shRNA compared to control cells (Figure 6D,E). As demonstrated in Figure 6F–H, transwell assays revealed that NUGC‐3 cells transfected with TPBG shRNA exhibited decreased migratory and invasive capabilities. Furthermore, based on the KEGG and GO analysis results above, we validated the effect of TPBG knockdown on the PI3K/AKT pathway. As demonstrated in Figure 6I, the expression of PI3K and the phosphorylation levels of PI3K and AKT were significantly reduced following TPBG knockdown, whereas the total AKT expression remained unchanged. These results suggest that TPBG shRNA may effectively inhibit TPBG‐induced NUGC‐3 cell migration and invasion via the PI3K/AKT signalling pathway.

### 3.8 Knockdown of TPBG Inhibits GC Xenograft Tumours In Vivo

In order to assess the impact of TPBG on the development of stomach cancer in living organisms, NUGC‐3 cells transfected with vector or TPBG shRNAs lentivirus were conducted in xenograft tumour models. As shown in Figure 6J–L, tumour dimensions were measured and tumour volumes were counted every 10 days after cell injection, and tumour tissues were collected 30 days post‐injection. As shown in Figure 6J, the tumour volume growth rates of TPBG shRNA groups were significantly slower than that of vector group. Furthermore, the inclusion of weights and images of tumour serves to corroborate the findings (Figure 6K,L). Taken together, knockdown TPBG expression can suppress xenograft tumour growth, suggesting that reducing TPBG levels could be advantageous in treating GC.

### 3.9 Association of TPBG Expression With Immune Characteristics in GC

**FIGURE 7:**
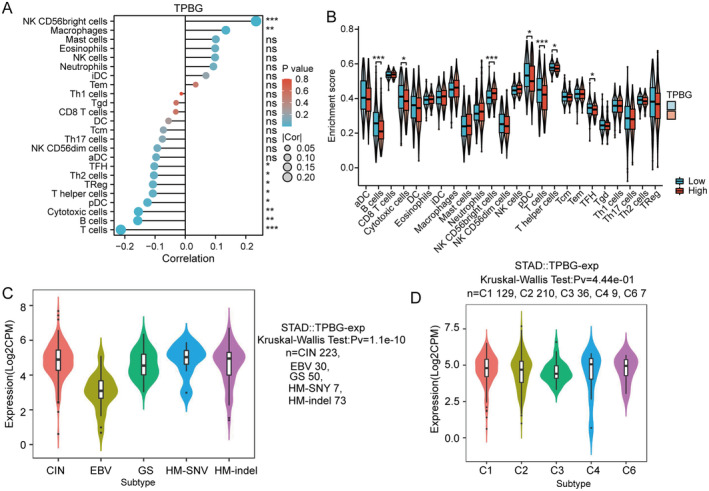
Analysis of the relationship between TPBG expression and immune infiltration in GC. (A) The correlation between TPBG and immune infiltrating cells in GC. (B) Differential distribution of immune cells in high TPBG expression and low TPBG expression tissues. (C, D) Correlation between TPBG expression and molecular subtypes (C) or immune subtypes (D). *, ** and *** indicate *p* < 0.05, *p* < 0.01 and *p* < 0.001, respectively.

For investigating the relationship between TPBG and immune response in GC tumours, immune infiltration analysis was performed using the TCGA database. The findings indicated a positive association between TPBG expression in individuals with stomach cancer and the presence of macrophages, mast cells, eosinophils, Natural Killer cells (NKs), CD56+ NKs, neutrophils, immature dendritic cells (iDCs), and effective memory T (Tem) cells, while a negative association with Cytotoxic T cells (CTLs), B cells, Plasmacytoid dendritic cells (pDCs), T helper 2 (Th2) cells, and regulatory T cells (Tregs) (Figure 7A). Furthermore, significant variations (*p* < 0.05) were observed in the infiltration of B cells, CTLs, CD56+ NKs, pDC, and helper T cells based on the categorisation of TPBG expression into high and low groups (Figure 7B).

There are five prognostic molecular subtypes of GC: chromosomal instability (CIN), Epstein–Barr virus (EBV), genomically stable (GS), HM‐SNY, and HM‐indel. Additionally, there are six immune subtypes of GC, which are categorised as wound healing (C1), IFN‐gamma dominant (C2), inflammatory (C3), lymphocyte depleted (C4), immunologically quiet (C5), and TGF‐β dominant (C6). The potential connection between TPBG levels and molecular or immune subtypes was investigated by TISIDB. Interestingly, TPBG expression was significantly associated with CIN, EBV, GS, HM‐SNY, and HM‐indel subgroups (*p* = 1.1e‐10), but showed no association with the immune subtypes (*p* = 0.444) (Figure 7C,D).

## 4 Discussion

GC (GC) remains a leading cause of cancer‐related mortality worldwide, with limited early detection and high rates of recurrence following treatment. Emerging therapies, including immune checkpoint inhibitors and targeted agents, are reshaping the treatment landscape, offering new avenues for personalised therapeutic approaches. Data from the TCGA database revealed a notable increase in TPBG expression levels in GC tissues when compared to normal tissues. Additional examination showed increased TPBG levels in GC samples or GC cell lines, both at the mRNA and protein levels. Those results indicate a possible role of TPBG in promoting GC development, suggesting that TPBG may serve as a potential therapeutic target to enhance treatment efficacy in advanced GC.

Accurate predictive biomarkers can offer crucial insights into the aggressiveness of cancer and/or the clinical results for individual untreated patients. They are crucial in personalised and precision medicine by guiding treatment decisions. Analysis of the TCGA database revealed a tight correlation between TPBG and tumour pathological stage, indicating the key function in cancer progression. Further examinations for several survival analyses among various subgroups indicated that individuals with elevated TPBG levels experienced decreased survival rates and that heightened TPBG expression independently posed a risk for OS in patients with stomach adenocarcinoma. Multiple researchers have consistently pointed out that higher expressed TPBG in tumours is linked to worse clinical outcomes [10, 11, 12, 13, 14]. A positive TPBG expression result is associated with a poor outcome in colorectal, gastric, ovarian, NSCLC, HNSCCs, and pancreatic cancer [4, 15, 16]. Consistent findings indicate that TPBG could be a dependable indicator of prognosis for patients with STAD.

The tumour microenvironment, comprising immune cells, mesenchymal stromal cells, lymphocytes, and extracellular matrix, is essential for tumour growth, chemotherapy resistance, and patient outcomes [17]. The infiltrative lymphocytes, such as CD8+ cytotoxic T lymphocytes (CTLs), CD4 + T lymphocytes (Th1, Th2, Th17, and Tregs), natural killer cells (NKs), dendritic cells (DCs), macrophages, and B cells, are involved in the progression of GC [18, 19, 20, 21, 22, 23]. Our study revealed a strong association between TPBG expression and the quantity of multiple immune cells, including CD56+ NKs, macrophages, mast cells, eosinophils, NKs, neutrophils and immature DCs (iDCs), as well as effective memory T (Tem) cells. The predominant infiltrative cells observed in cases of TPBG overexpression were CD56+ NKs and macrophages. NKs are crucial in the pathogenesis and progression of both acute allergic reactions and chronic inflammatory conditions. More importantly, NKs expressing CD56 brightly primarily facilitate the immune response against tumours as a promising approach for cancer immunotherapy. As another immune cell closely associated with TPBG, Macrophages are critical in the advancement. In other words, the presence of macrophages within tumours is strongly linked to poor outcomes. Our research suggested that TPBG could potentially impact the immunological stress in GC by controlling the infiltration of immune cells, which could ultimately impact the prognosis of patients.

Currently, targeted therapies play a crucial role in cancer treatment, with EGFR‐targeted therapy for lung cancer [24] and HER2‐targeted therapy for breast cancer [25] yielding promising results. However, therapeutic options for targeting GC remain limited. In this study, molecular biology experiments revealed that knockdown of TPBG significantly inhibited the proliferation, migration, and invasion of GC cells both in vitro and in vivo. Furthermore, we found that TPBG knockdown markedly suppressed the PI3K/AKT signalling pathway. These results suggest that TPBG may play an important role in the growth and metastasis of GC, and targeting TPBG could potentially improve treatment outcomes for certain GC patients.

While our findings may offer fresh perspectives on the relationship between TPBG and STAD, we did acknowledge some constraints in this research. Initially, sample bias could exist as a result of obtaining data directly from public databases. Increasing the sample size of GC specimens is the primary objective of the forthcoming research in order to improve the validity of the results. Furthermore, the investigation of the precise molecular mechanism of TPBG in tumorigenesis, such as the underlying reasons for TPBG‐induced PI3K‐AKT activation and whether TPBG induces other signalling pathways or molecular modifications, as well as the identification of potential TPBG inhibitors, is the focus of our upcoming research.

## 5 Conclusion

In this research, we conducted demonstration of the predictive significance of TPBG in GC. Our research indicates that TPBG might be a promising biomarker to predict treatment response and prognosis in GC patients. Exploring deeper molecular mechanisms and potential inhibitors represents a highly significant direction for future research in GC.

## Author Contributions

**Lianlei Yang:** investigation (equal), writing – original draft (equal). **Chunyan Weng:** writing – original draft (equal). **Yaping Zhang:** writing – original draft (equal). **Yu Zhao:** data curation (equal). **Kexin Chen:** investigation (equal). **Guodong Li:** supervision (equal). **Xueqing Zhong:** data curation (equal). **Chenghai He:** supervision (equal).

## Ethics Statement

The present study was approved by the Laboratory animal management and ethics committee of Zhejiang Chinese Medical University (Approval No. IACUC‐2022011‐02). All the procedures for the care of the rats were in accordance with the institutional guidelines for animal use in research.

## Conflicts of Interest

The authors declare no conflicts of interest.

## Supporting information


**Figure S1.** Gene interaction networks and functional clustering of genes related to the TPBG. (A–C) Enrichment analyses of BP (A), CC (B), and MF (C) of differentially expressed genes (DEGs) between TPBG high and low expression. (D) KEGG enrichment analyses of DEGs between TPBG high and low expression.

**Table S1.** Differentially expressed genes in STAD samples with high and low TPBG expression.

**Table S2.** GO and KEGG pathway enrichment analysis of 797 differentially expressed genes in STAD samples with high and low TPBG expression.

**Table S3.** Co‐expressed TPBG‐related genes were identified in the TCGA database.

**Table S4.** GO and KEGG pathway enrichment analysis of TPBG co‐expressed genes.

## Data Availability

Data will be made available on request.
