# Supplementary figures and images for: In‐Depth Examination of TPBG as a New Predictive Indicator for Gastric Cancer
Source: J Cell Mol Med. 2025 Jan 17;29(2):e70354. doi: 10.1111/jcmm.70354 (PMC11740983; doi:10.1111/jcmm.70354)

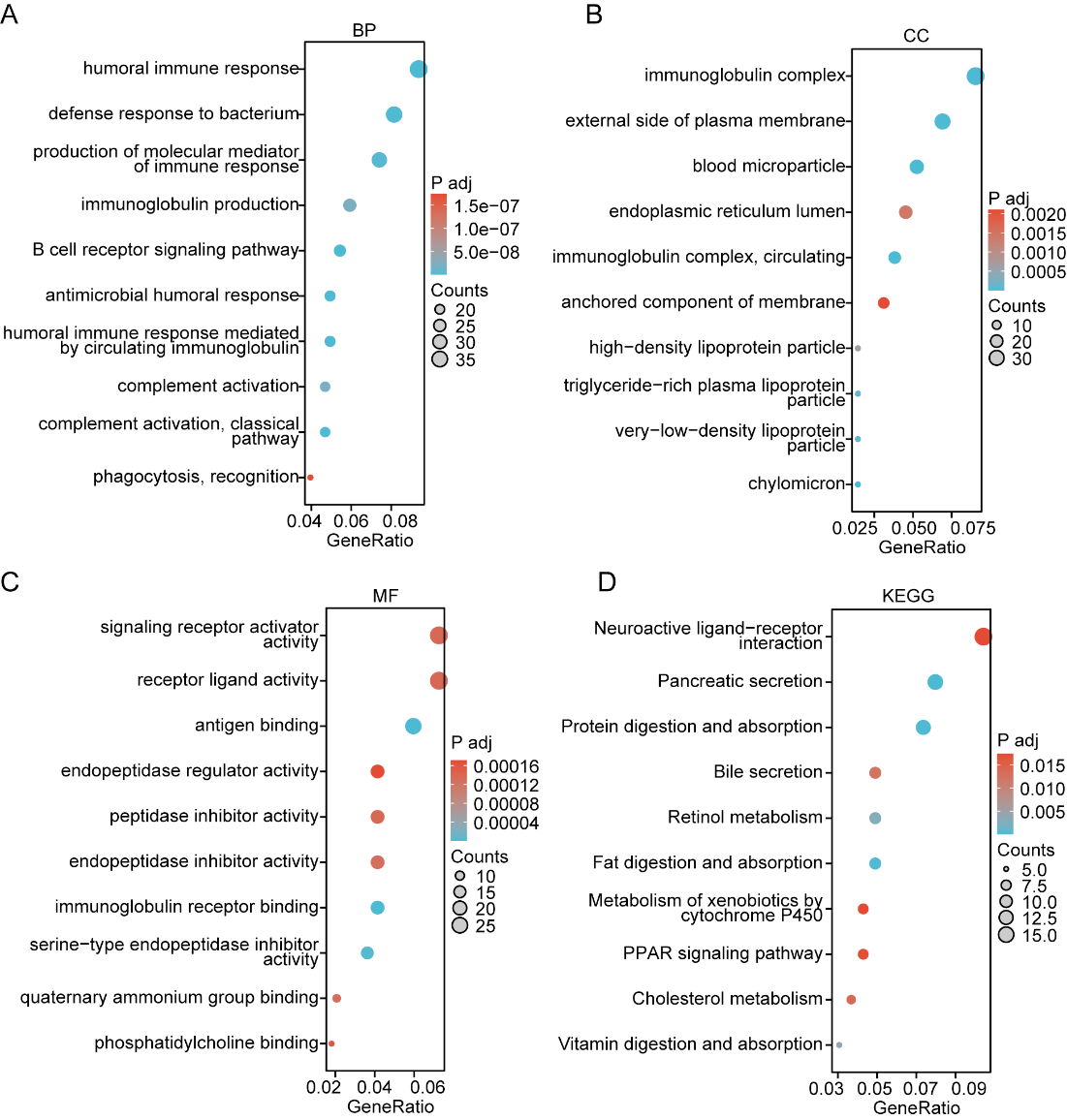

Supplement: Supplementary file 1 — Figure S1. Gene interaction networks and functional clustering of genes related to the TPBG. (A–C) Enrichment analyses of BP (A), CC (B), and MF (C) of differentially expressed genes (DEGs) between TPBG high and low expression. (D) KEGG enrichment analyses of DEGs between TPBG high and low expression. [file JCMM-29-e70354-s002.docx]
