# Supplementary material for: In‐Depth Examination of TPBG as a New Predictive Indicator for Gastric Cancer
Source: J Cell Mol Med. 2025 Jan 17;29(2):e70354. doi: 10.1111/jcmm.70354 (PMC11740983; doi:10.1111/jcmm.70354)
Supplement: Supplementary file 3 — Table S2. GO and KEGG pathway enrichment analysis of 797 differentially expressed genes in STAD samples with high and low TPBG expression. [file JCMM-29-e70354-s003.pdf]

id	correlation_pearson	pvalue_pearson	padj_pearson	correlation_spearman	pvalue_spearman	padj_spearman	gene_name	gene_type
ENSG00000002746.15	0.317032444	3.35147E-10	2.06254E-08	0.351012186	2.58616E-12	3.72676E-10	HECW1	protein_coding
ENSG00000003436.16	0.368778416	1.59222E-13	2.91497E-11	0.347152597	4.63241E-12	6.03333E-10	TFPI	protein_coding
ENSG00000004399.13	0.346180259	5.35844E-12	5.93075E-10	0.30765161	1.41409E-09	7.58761E-08	PLXND1	protein_coding
ENSG00000004700.16	0.321937918	1.72243E-10	1.15128E-08	0.307624531	1.41897E-09	7.59807E-08	RECQL	protein_coding
ENSG00000005238.20	0.407317684	2.02607E-16	9.66832E-14	0.418620596	2.41805E-17	1.54697E-14	FAM214B	protein_coding
ENSG00000005243.10	0.342965351	8.64074E-12	9.01517E-10	0.328823757	8.59234E-11	7.13593E-09	COPZ2	protein_coding
ENSG00000005700.15	0.296456679	4.8046E-09	2.01378E-07	0.302316987	2.76849E-09	1.32003E-07	IBTK	protein_coding
ENSG00000005884.18	0.392378899	2.98009E-15	1.00286E-12	0.380664012	2.64473E-14	8.27802E-12	ITGA3	protein_coding
ENSG00000006125.18	0.307706379	1.14952E-09	5.88076E-08	0.321654568	2.27363E-10	1.62015E-08	AP2B1	protein_coding
ENSG00000006468.14	0.383140807	1.46969E-14	3.8716E-12	0.419844147	1.91171E-17	1.26472E-14	ETV1	protein_coding
ENSG00000006534.16	0.377208852	3.98857E-14	9.17813E-12	0.358847423	1.06365E-12	1.78971E-10	ALDH3B1	protein_coding
ENSG00000007202.15	0.30542274	1.54441E-09	7.562E-08	0.310398015	8.08985E-10	4.82556E-08	KIAA0100	protein_coding
ENSG00000007384.15	0.388947313	5.42248E-15	1.6189E-12	0.407612015	0	0	RHBDF1	protein_coding
ENSG00000007866.22	0.326387961	9.31821E-11	6.85825E-09	0.333788599	4.31202E-11	3.96583E-09	TEAD3	protein_coding
ENSG00000008311.15	0.316192085	3.75177E-10	2.26342E-08	0.301637501	3.01297E-09	1.42147E-07	AASS	protein_coding
ENSG00000008405.12	0.31678463	3.46498E-10	2.11895E-08	0.31674798	4.36043E-10	2.8269E-08	CRY1	protein_coding
ENSG00000008513.16	0.373115613	7.85051E-14	1.62381E-11	0.333267949	4.63819E-11	4.20602E-09	ST3GAL1	protein_coding
ENSG00000009844.16	0.305332572	1.56244E-09	7.63744E-08	0.326067357	1.25296E-10	9.8044E-09	VTA1	protein_coding
ENSG00000010818.10	0.346248153	5.30432E-12	5.88204E-10	0.319858915	2.88945E-10	1.99547E-08	HIVEP2	protein_coding
ENSG00000011028.14	0.413858697	5.97947E-17	3.66434E-14	0.374910684	7.52387E-14	1.93816E-11	MRC2	protein_coding
ENSG00000011114.15	0.325395727	1.06955E-10	7.72544E-09	0.30746721	1.18577E-09	6.58715E-08	BTBD7	protein_coding
ENSG00000011198.10	0.338834924	1.58373E-11	1.51648E-09	0.355818657	1.23746E-12	2.02366E-10	ABHD5	protein_coding
ENSG00000011201.12	0.33542153	2.59542E-11	2.29287E-09	0.340924792	1.56344E-11	1.69814E-09	ANOS1	protein_coding
ENSG00000011243.18	0.30546364	1.53629E-09	7.52862E-08	0.319714793	2.33242E-10	1.65798E-08	AKAP8L	protein_coding
ENSG00000011422.12	0.359632902	6.83298E-13	1.00455E-10	0.362211628	6.25194E-13	1.13388E-10	PLAUR	protein_coding
ENSG00000012779.11	0.340239575	1.29013E-11	1.27303E-09	0.316967118	4.23651E-10	2.76503E-08	ALOX5	protein_coding
ENSG00000013288.9	0.361694238	4.94062E-13	7.54942E-11	0.347528046	5.95502E-12	7.48411E-10	MAN2B2	protein_coding
ENSG00000013375.16	0.364985135	2.9298E-13	4.91547E-11	0.372165889	1.21104E-13	2.9345E-11	PGM3	protein_coding
ENSG00000013563.14	0.341001373	1.15386E-11	1.1531E-09	0.331784048	5.70504E-11	5.05358E-09	DNASE1L1	protein_coding
ENSG00000013588.9	0.29713241	4.41668E-09	1.87413E-07	0.309999545	1.04768E-09	5.97394E-08	GPRC5A	protein_coding
ENSG00000013619.14	0.379923382	2.53218E-14	6.24656E-12	0.33183889	5.66167E-11	5.03993E-09	MAMLD1	protein_coding
ENSG00000016864.18	0.312057422	6.50287E-10	3.61589E-08	0.341485493	1.44188E-11	1.57941E-09	GLT8D1	protein_coding
ENSG00000017797.13	0.321664199	1.78818E-10	1.18705E-08	0.330726362	6.60744E-11	5.72428E-09	RALBP1	protein_coding
ENSG00000018280.17	0.329371219	6.13789E-11	4.75181E-09	0.337018091	2.73413E-11	2.68424E-09	SLC11A1	protein_coding
ENSG00000018408.15	0.336759947	2.13996E-11	1.93754E-09	0.312534532	7.5567E-10	4.55463E-08	WWTR1	protein_coding
ENSG00000019144.19	0.348010487	4.07227E-12	4.70396E-10	0.32021612	2.17876E-10	1.56018E-08	PHLDB1	protein_coding
ENSG00000019549.13	0.376961955	4.15598E-14	9.45129E-12	0.328215269	9.34163E-11	7.68741E-09	SNAI2	protein_coding
ENSG00000020129.16	0.357732514	9.19489E-13	1.28371E-10	0.356250012	1.15754E-12	1.91448E-10	NCDN	protein_coding
ENSG00000021762.20	0.398471736	1.01188E-15	3.98037E-13	0.421682785	0	0	OSBPL5	protein_coding
ENSG00000022567.10	0.310034392	8.48483E-10	4.56112E-08	0.309146205	1.16869E-09	6.50464E-08	SLC45A4	protein_coding
ENSG00000024422.12	0.392955116	2.69326E-15	9.38899E-13	0.344725225	8.99981E-12	1.05849E-09	EHD2	protein_coding
ENSG00000026652.15	0.43980399	3.60433E-19	5.15247E-16	0.416151875	3.87339E-17	2.42474E-14	AGPAT4	protein_coding
ENSG00000029559.7	0.276823956	5.04724E-08	1.48105E-06	0.306892462	1.27748E-09	7.00302E-08	IBSP	protein_coding
ENSG00000030582.18	0.394015464	2.23447E-15	7.98078E-13	0.396065309	6.4408E-16	3.17772E-13	GRN	protein_coding
ENSG00000035687.10	0.282985574	2.45995E-08	8.00075E-07	0.308388213	1.28747E-09	7.03793E-08	ADSS2	protein_coding
ENSG00000035862.12	0.450130029	4.15813E-20	8.64564E-17	0.401853908	0	0	TIMP2	protein_coding
ENSG00000037749.12	0.350838515	2.65535E-12	3.24767E-10	0.342822752	8.8247E-12	1.03999E-09	MFAP3	protein_coding
ENSG00000038382.20	0.347356465	4.49285E-12	5.07893E-10	0.313779952	6.42884E-10	3.99865E-08	TRIO	protein_coding
ENSG00000038427.16	0.416224822	3.82005E-17	2.47106E-14	0.385172375	1.09158E-14	3.80538E-12	VCAN	protein_coding
ENSG00000039523.20	0.352971735	1.91778E-12	2.43776E-10	0.311902054	6.63757E-10	4.10219E-08	RIPOR1	protein_coding
ENSG00000044115.21	0.275927477	5.59542E-08	1.61733E-06	0.326667198	1.1546E-10	9.13291E-09	CTNNA1	protein_coding
ENSG00000047932.14	0.314222916	4.88043E-10	2.81874E-08	0.316305837	4.62129E-10	2.98936E-08	GOPC	protein_coding
ENSG00000048544.6	0.316490999	3.60433E-10	2.19494E-08	0.325335078	1.38413E-10	1.07156E-08	MRPS10	protein_coding
ENSG00000050820.17	0.356911281	1.04472E-12	1.44469E-10	0.359905791	9.00874E-13	1.53354E-10	BCAR1	protein_coding
ENSG00000054277.14	0.31484731	4.49087E-10	2.62618E-08	0.32048441	2.10062E-10	1.51918E-08	OPN3	protein_coding
ENSG00000054392.13	0.331609918	4.47362E-11	3.65467E-09	0.334977131	3.64881E-11	3.43732E-09	HHAT	protein_coding
ENSG00000054598.9	0.335667392	2.50521E-11	2.21991E-09	0.342509045	1.24324E-11	1.40269E-09	FOXC1	protein_coding
ENSG00000055208.19	0.347888468	4.14771E-12	4.76275E-10	0.329601111	5.94245E-11	5.2201E-09	TAB2	protein_coding
ENSG00000057019.16	0.35334514	1.81114E-12	2.32761E-10	0.355124588	1.89395E-12	2.85653E-10	DCBLD2	protein_coding
ENSG00000057149.16	0.221770124	1.46216E-05	0.00019285	0.301362852	2.59439E-09	1.24827E-07	SERPINB3	protein_coding
ENSG00000058085.15	0.3529223	1.93235E-12	2.44745E-10	0.357324155	1.34867E-12	2.17499E-10	LAMC2	protein_coding
ENSG00000058262.10	0.37728028	3.94139E-14	9.14182E-12	0.367367391	2.71074E-13	5.82339E-11	SEC61A1	protein_coding
ENSG00000060558.4	0.350559934	2.77012E-12	3.36682E-10	0.331299579	6.10242E-11	5.34242E-09	GNA15	protein_coding
ENSG00000060656.20	0.45137252	3.19061E-20	7.14426E-17	0.44791899	0	0	PTPRU	protein_coding
ENSG00000060718.22	0.326508991	9.16252E-11	6.76076E-09	0.34033633	1.70172E-11	1.8245E-09	COL11A1	protein_coding
ENSG00000060982.15	0.289039543	1.19337E-08	4.33952E-07	0.335536826	2.55273E-11	2.53177E-09	BCAT1	protein_coding

ENSG00000061455.11	0.325156855	1.10556E-10	7.92652E-09	0.337350609	1.96468E-11	2.05719E-09	PRDM6	protein_coding
ENSG00000062038.14	0.34523937	6.16618E-12	6.69743E-10	0.351153942	3.46405E-12	4.70094E-10	CDH3	protein_coding
ENSG00000062725.10	0.30552862	1.52349E-09	7.4911E-08	0.304315201	1.78058E-09	9.15739E-08	APPBP2	protein_coding
ENSG00000063660.9	0.351320261	2.46774E-12	3.04415E-10	0.346021618	7.43952E-12	8.94863E-10	GPC1	protein_coding
ENSG00000064042.18	0.378154234	3.40648E-14	8.06172E-12	0.369066788	2.04479E-13	4.59628E-11	LIMCH1	protein_coding
ENSG00000064652.11	0.313568239	5.32412E-10	3.0418E-08	0.305444533	1.8702E-09	9.52573E-08	SNX24	protein_coding
ENSG00000064666.15	0.301181353	2.65476E-09	1.1981E-07	0.321858004	2.2125E-10	1.5824E-08	CNN2	protein_coding
ENSG00000064726.10	0.337924881	1.80773E-11	1.7002E-09	0.349909432	4.17615E-12	5.56355E-10	BTBD1	protein_coding
ENSG00000064932.16	0.319100975	2.53495E-10	1.61643E-08	0.33351189	4.48249E-11	4.09187E-09	SBNO2	protein_coding
ENSG00000065000.19	0.320935909	1.97524E-10	1.29207E-08	0.309573558	1.10648E-09	6.24197E-08	AP3D1	protein_coding
ENSG00000065491.8	0.302538146	2.23448E-09	1.03573E-07	0.312742292	7.35604E-10	4.46563E-08	TBC1D22B	protein_coding
ENSG00000065989.16	0.501434383	2.85659E-25	2.37578E-21	0.4585384	0	0	PDE4A	protein_coding
ENSG00000066455.13	0.383233939	1.4466E-14	3.82809E-12	0.362624893	4.26448E-13	8.4304E-11	GOLGA5	protein_coding
ENSG00000067225.18	0.318115418	2.89643E-10	1.821E-08	0.338766868	2.13122E-11	2.18827E-09	PKM	protein_coding
ENSG00000067445.21	0.29453051	6.10039E-09	2.46085E-07	0.309628434	8.94793E-10	5.22498E-08	TRO	protein_coding
ENSG00000067596.12	0.361655612	4.97083E-13	7.57571E-11	0.374115599	8.64729E-14	2.19837E-11	DHX8	protein_coding
ENSG00000068305.17	0.322870327	1.51565E-10	1.03082E-08	0.302166248	2.34278E-09	1.14423E-07	MEF2A	protein_coding
ENSG00000068912.14	0.362916384	4.07197E-13	6.43082E-11	0.339763341	1.84774E-11	1.94877E-09	ERLEC1	protein_coding
ENSG00000068971.14	0.410862204	1.0493E-16	5.60439E-14	0.368037774	2.42644E-13	5.23195E-11	PPP2R5B	protein_coding
ENSG00000069812.11	0.293058121	7.31324E-09	2.8422E-07	0.316826542	3.44553E-10	2.32435E-08	HES2	protein_coding
ENSG00000069956.12	0.333337228	3.49873E-11	2.99103E-09	0.350861304	3.62008E-12	4.88987E-10	MAPK6	protein_coding
ENSG00000070087.14	0.370109159	1.28312E-13	2.42534E-11	0.328546952	6.89149E-11	5.9111E-09	PFN2	protein_coding
ENSG00000070182.21	0.311560009	6.94375E-10	3.83176E-08	0.366884505	2.16106E-13	4.77253E-11	SPTB	protein_coding
ENSG00000070404.10	0.455324918	1.36405E-20	3.30885E-17	0.427532598	0	0	FSTL3	protein_coding
ENSG00000070444.15	0.362910125	4.07601E-13	6.43082E-11	0.380259415	2.85392E-14	8.83776E-12	MNT	protein_coding
ENSG00000070540.13	0.430947811	2.16838E-18	2.10398E-15	0.428848926	3.29232E-18	2.30931E-15	WIPI1	protein_coding
ENSG00000071242.12	0.361035522	5.48144E-13	8.24595E-11	0.330733644	6.60078E-11	5.72428E-09	RPS6KA2	protein_coding
ENSG00000072134.15	0.338433274	1.67904E-11	1.59723E-09	0.308028252	1.10244E-09	6.23443E-08	EPN2	protein_coding
ENSG00000072682.19	0.357574981	9.42315E-13	1.31243E-10	0.362443985	4.3884E-13	8.57329E-11	P4HA2	protein_coding
ENSG00000073150.14	0.284768713	1.99145E-08	6.68035E-07	0.30975598	8.79984E-10	5.16962E-08	PANX2	protein_coding
ENSG00000073711.11	0.315113506	4.33414E-10	2.54616E-08	0.307814541	1.38508E-09	7.48716E-08	PPP2R3A	protein_coding
ENSG00000073921.18	0.349745508	3.13415E-12	3.77772E-10	0.330653089	6.67486E-11	5.7741E-09	PICALM	protein_coding
ENSG00000074047.22	0.41275851	7.35563E-17	4.2399E-14	0.400523655	6.99775E-16	3.42349E-13	GLI2	protein_coding
ENSG00000074181.9	0.408959581	1.49523E-16	7.5695E-14	0.352404597	2.86763E-12	3.99397E-10	NOTCH3	protein_coding
ENSG00000074416.14	0.427433988	4.3557E-18	3.78478E-15	0.393543998	2.42813E-15	1.04712E-12	MGLL	protein_coding
ENSG00000074590.13	0.402554062	4.84588E-16	2.05925E-13	0.368250313	2.34242E-13	5.06956E-11	NUAK1	protein_coding
ENSG00000075275.18	0.470576831	4.62266E-22	1.79415E-18	0.468594834	0	0	CELSR1	protein_coding
ENSG00000075399.14	0.323816732	1.33053E-10	9.23253E-09	0.3230545	1.88401E-10	1.39015E-08	VPS9D1	protein_coding
ENSG00000075420.13	0.3655422	2.68018E-13	4.57579E-11	0.371385141	1.38362E-13	3.27446E-11	FNDC3B	protein_coding
ENSG00000075426.12	0.323152232	1.45803E-10	9.9512E-09	0.324624189	1.52419E-10	1.15994E-08	FOSL2	protein_coding
ENSG00000075618.18	0.431588024	1.90793E-18	1.9487E-15	0.377844578	4.4558E-14	1.2842E-11	FSCN1	protein_coding
ENSG00000075651.16	0.308660004	1.0154E-09	5.27809E-08	0.331737077	4.39363E-11	4.03451E-09	PLD1	protein_coding
ENSG00000075785.14	0.307379492	1.19934E-09	6.08747E-08	0.332585277	5.10241E-11	4.58501E-09	RAB7A	protein_coding
ENSG00000075945.13	0.304578452	1.72145E-09	8.30319E-08	0.341793606	1.37905E-11	1.52344E-09	KIFAP3	protein_coding
ENSG00000076108.12	0.310620112	7.85745E-10	4.26721E-08	0.315520537	5.12248E-10	3.26803E-08	BAZ2A	protein_coding
ENSG00000076201.15	0.325414898	1.06671E-10	7.7145E-09	0.319390374	3.07513E-10	2.11367E-08	PTPN23	protein_coding
ENSG00000076321.11	0.330168797	5.48542E-11	4.339E-09	0.321005575	2.47983E-10	1.74572E-08	KLHL20	protein_coding
ENSG00000076351.13	0.314267265	4.85171E-10	2.80493E-08	0.322871221	1.51546E-10	1.15592E-08	SLC46A1	protein_coding
ENSG00000077232.18	0.353326224	1.8164E-12	2.32923E-10	0.357745819	1.26313E-12	2.0541E-10	DNAJC10	protein_coding
ENSG00000077238.14	0.337332028	1.96998E-11	1.81469E-09	0.307389919	1.46192E-09	7.77974E-08	IL4R	protein_coding
ENSG00000078098.14	0.384667446	1.13293E-14	3.11119E-12	0.353697577	2.35602E-12	3.40354E-10	FAP	protein_coding
ENSG00000078369.18	0.292730369	7.61341E-09	2.94119E-07	0.316016839	4.79993E-10	3.0946E-08	GNB1	protein_coding
ENSG00000078804.13	0.334232609	3.07831E-11	2.66686E-09	0.328832859	8.58158E-11	7.13593E-09	TP53INP2	protein_coding
ENSG00000079150.18	0.324025639	1.29274E-10	9.00248E-09	0.312866993	7.2381E-10	4.41243E-08	FKBP7	protein_coding
ENSG00000079257.8	0.313017277	5.72769E-10	3.23115E-08	0.319267266	3.12581E-10	2.1384E-08	LXN	protein_coding
ENSG00000079432.7	0.3106059	7.87213E-10	4.2712E-08	0.309237701	9.4168E-10	5.43876E-08	CIC	protein_coding
ENSG00000079739.17	0.3642248	3.30752E-13	5.43947E-11	0.350259455	2.89934E-12	4.0285E-10	PGM1	protein_coding
ENSG00000080200.10	0.380887857	2.1525E-14	5.42486E-12	0.365450449	3.71063E-13	7.68773E-11	CRYBG3	protein_coding
ENSG00000080561.14	0.350726807	2.7008E-12	3.29634E-10	0.350275799	3.95296E-12	5.29042E-10	MID2	protein_coding
ENSG00000080815.19	0.34972051	3.14603E-12	3.78421E-10	0.344096845	7.30781E-12	8.82668E-10	PSEN1	protein_coding
ENSG00000082153.18	0.33479647	2.83927E-11	2.48193E-09	0.339140061	2.02042E-11	2.0967E-09	BZW1	protein_coding
ENSG00000082497.12	0.333278339	3.52826E-11	3.00744E-09	0.340676433	1.21018E-11	1.3707E-09	SERTAD4	protein_coding
ENSG00000082781.12	0.412478108	7.75344E-17	4.42539E-14	0.41325202	0	0	ITGB5	protein_coding
ENSG00000083099.11	0.316379269	3.65876E-10	2.21968E-08	0.325810919	1.00966E-10	8.16396E-09	LYRM2	protein_coding
ENSG00000083444.17	0.372597575	8.54715E-14	1.73379E-11	0.366113551	3.33027E-13	6.99933E-11	PLOD1	protein_coding
ENSG00000083817.9	0.381084842	2.08211E-14	5.2933E-12	0.332583912	5.10338E-11	4.58501E-09	ZNF416	protein_coding
ENSG00000083937.10	0.293354966	7.05129E-09	2.76191E-07	0.300094664	3.64836E-09	1.67508E-07	CHMP2B	protein_coding

ENSG00000084234.18	0.356359183	1.13812E-12	1.55538E-10	0.373401525	9.78952E-14	2.45658E-11	APLP2	protein_coding
ENSG00000085185.15	0.322420149	1.61227E-10	1.08951E-08	0.32638844	1.19934E-10	9.43618E-09	BCORL1	protein_coding
ENSG00000085511.20	0.327617307	7.84975E-11	5.87399E-09	0.316971232	3.37917E-10	2.28754E-08	MAP3K4	protein_coding
ENSG00000085733.16	0.33114193	4.78042E-11	3.86536E-09	0.353852543	2.30099E-12	3.34062E-10	CTTN	protein_coding
ENSG00000086062.13	0.381792946	1.84723E-14	4.75851E-12	0.364492661	4.33439E-13	8.52165E-11	B4GALT1	protein_coding
ENSG00000086991.13	0.319136689	2.52271E-10	1.61171E-08	0.303335454	2.01843E-09	1.01563E-07	NOX4	protein_coding
ENSG00000087111.21	0.354146577	1.60144E-12	2.09042E-10	0.364474229	4.34733E-13	8.52165E-11	PIGS	protein_coding
ENSG00000087116.16	0.362797845	4.14921E-13	6.49351E-11	0.34530504	8.26621E-12	9.80127E-10	ADAMTS2	protein_coding
ENSG00000087152.15	0.304392028	1.76312E-09	8.46193E-08	0.322557647	1.58214E-10	1.19467E-08	ATXN7L3	protein_coding
ENSG00000087245.13	0.412043338	8.41239E-17	4.62031E-14	0.373685744	9.31878E-14	2.35312E-11	MMP2	protein_coding
ENSG00000087253.13	0.33526435	2.65475E-11	2.33819E-09	0.310377876	8.11125E-10	4.83337E-08	LPCAT2	protein_coding
ENSG00000087303.18	0.351295935	2.4769E-12	3.04862E-10	0.322262829	2.09556E-10	1.51741E-08	NID2	protein_coding
ENSG00000087510.7	0.325196353	1.09952E-10	7.90272E-09	0.348609398	5.07115E-12	6.51727E-10	TFAP2C	protein_coding
ENSG00000088280.19	0.325821823	1.00813E-10	7.34562E-09	0.323587439	1.75344E-10	1.30775E-08	ASAP3	protein_coding
ENSG00000088882.8	0.340862021	1.17769E-11	1.17402E-09	0.311229036	8.94486E-10	5.22498E-08	CPXM1	protein_coding
ENSG00000089159.16	0.370376764	1.22847E-13	2.32961E-11	0.3305161	6.80269E-11	5.86725E-09	PXN	protein_coding
ENSG00000089597.18	0.277353126	4.7484E-08	1.40755E-06	0.300998976	3.26171E-09	1.52278E-07	GANAB	protein_coding
ENSG00000089723.10	0.290546379	9.9408E-09	3.6862E-07	0.305909108	1.45058E-09	7.72642E-08	OTUB2	protein_coding
ENSG00000089876.12	0.320431991	2.11567E-10	1.37774E-08	0.327682785	1.00492E-10	8.14282E-09	DHX32	protein_coding
ENSG00000090316.16	0.309704483	8.85934E-10	4.7232E-08	0.307953806	1.36075E-09	7.38992E-08	MAEA	protein_coding
ENSG00000090539.15	0.301235766	2.63652E-09	1.19171E-07	0.300885442	2.75612E-09	1.31595E-07	CHRD	protein_coding
ENSG00000090863.12	0.380729534	2.21075E-14	5.51566E-12	0.377401752	4.82788E-14	1.35939E-11	GLG1	protein_coding
ENSG00000090924.15	0.396868131	1.34751E-15	5.02881E-13	0.335338946	3.46741E-11	3.29308E-09	PLEKHG2	protein_coding
ENSG00000091317.8	0.277493994	4.67177E-08	1.38845E-06	0.30761998	1.41979E-09	7.59807E-08	CMTM6	protein_coding
ENSG00000092929.12	0.372330688	8.92931E-14	1.78757E-11	0.324750028	1.49843E-10	1.14482E-08	UNC13D	protein_coding
ENSG00000092969.12	0.345440333	5.98424E-12	6.52417E-10	0.36318398	5.35145E-13	1.01153E-10	TGFB2	protein_coding
ENSG00000095139.14	0.346878044	4.82707E-12	5.43563E-10	0.335274093	3.49927E-11	3.31792E-09	ARCN1	protein_coding
ENSG00000095383.20	0.407734592	1.87594E-16	9.02591E-14	0.389805439	3.9581E-15	1.56757E-12	TBC1D2	protein_coding
ENSG00000096401.8	0.29533323	5.52376E-09	2.26148E-07	0.315548335	4.08949E-10	2.69088E-08	CDC5L	protein_coding
ENSG00000099337.5	0.400673047	6.81167E-16	2.8125E-13	0.362779838	5.70943E-13	1.05521E-10	KCNK6	protein_coding
ENSG00000099904.16	0.324285548	1.24718E-10	8.7396E-09	0.356076687	1.63584E-12	2.53285E-10	ZDHHC8	protein_coding
ENSG00000099953.10	0.34824263	3.93244E-12	4.5566E-10	0.351963818	3.06549E-12	4.22906E-10	MMP11	protein_coding
ENSG00000099960.13	0.29948995	3.28694E-09	1.4464E-07	0.306215136	1.3944E-09	7.52356E-08	SLC7A4	protein_coding
ENSG00000100196.11	0.323063945	1.47584E-10	1.0061E-08	0.322828536	1.94217E-10	1.42047E-08	KDELR3	protein_coding
ENSG00000100221.10	0.325016169	1.12731E-10	8.0601E-09	0.331764706	5.72041E-11	5.05358E-09	JOSD1	protein_coding
ENSG00000100243.21	0.304043894	1.84357E-09	8.77588E-08	0.300693139	3.3878E-09	1.56906E-07	CYB5R3	protein_coding
ENSG00000100284.21	0.289690311	1.10296E-08	4.05125E-07	0.300719763	3.37664E-09	1.56638E-07	TOM1	protein_coding
ENSG00000100504.17	0.459085331	6.01492E-21	1.75088E-17	0.444168165	0	0	PYGL	protein_coding
ENSG00000100625.9	0.336501908	2.22123E-11	1.99253E-09	0.347557856	5.92876E-12	7.48411E-10	SIX4	protein_coding
ENSG00000100644.17	0.323873412	1.32017E-10	9.17158E-09	0.30523336	1.92067E-09	9.7148E-08	HIF1A	protein_coding
ENSG00000100711.14	0.290768854	9.67533E-09	3.60012E-07	0.302881329	2.58018E-09	1.24451E-07	ZFYVE21	protein_coding
ENSG00000100842.13	0.33850992	1.66043E-11	1.58211E-09	0.322925475	1.91701E-10	1.40737E-08	EFS	protein_coding
ENSG00000100934.15	0.301295746	2.61655E-09	1.18637E-07	0.311778832	6.74633E-10	4.16057E-08	SEC23A	protein_coding
ENSG00000100968.14	0.358047514	8.75455E-13	1.24008E-10	0.360627148	8.04E-13	1.37668E-10	NFATC4	protein_coding
ENSG00000101004.15	0.325009588	1.12834E-10	8.0601E-09	0.300235749	3.58524E-09	1.6514E-07	NINL	protein_coding
ENSG00000101198.15	0.250996945	8.50782E-07	1.67958E-05	0.337182335	2.01314E-11	2.09287E-09	NKAIN4	protein_coding
ENSG00000101443.18	0.379628085	2.66103E-14	6.455E-12	0.379499282	2.71922E-14	8.46566E-12	WFDC2	protein_coding
ENSG00000101752.12	0.311379123	7.11117E-10	3.91673E-08	0.323305419	1.42762E-10	1.10084E-08	MIB1	protein_coding
ENSG00000101825.8	0.389116114	5.266E-15	1.58029E-12	0.378648766	3.84751E-14	1.14869E-11	MXRA5	protein_coding
ENSG00000102024.18	0.345680133	5.77399E-12	6.31861E-10	0.355147116	1.88741E-12	2.85406E-10	PLS3	protein_coding
ENSG00000102038.16	0.399139879	8.97626E-16	3.60041E-13	0.36101536	7.56107E-13	1.33417E-10	SMARCA1	protein_coding
ENSG00000102302.8	0.353125403	1.87317E-12	2.3915E-10	0.337943149	1.80295E-11	1.90844E-09	FGD1	protein_coding
ENSG00000102316.17	0.343815593	7.61906E-12	8.07953E-10	0.329087723	8.28578E-11	6.95074E-09	MAGED2	protein_coding
ENSG00000102359.8	0.438845311	4.38824E-19	5.94127E-16	0.416420071	0	0	SRPX2	protein_coding
ENSG00000102362.15	0.432893163	1.4686E-18	1.58869E-15	0.434793225	1.00129E-18	7.37884E-16	SYTL4	protein_coding
ENSG00000102401.20	0.452061279	2.75364E-20	6.41246E-17	0.440158835	0	0	ARMCX3	protein_coding
ENSG00000102595.20	0.343238787	8.29839E-12	8.68913E-10	0.340398244	1.26051E-11	1.41943E-09	UGGT2	protein_coding
ENSG00000102710.20	0.29053754	9.95149E-09	3.68782E-07	0.300949596	3.28176E-09	1.52823E-07	SUPT20H	protein_coding
ENSG00000102802.10	0.339371914	1.46451E-11	1.4116E-09	0.316858819	3.43061E-10	2.31698E-08	MEDAG	protein_coding
ENSG00000103042.9	0.274302293	6.73908E-08	1.88532E-06	0.301776084	2.96146E-09	1.40285E-07	SLC38A7	protein_coding
ENSG00000103353.16	0.303214252	2.04991E-09	9.5934E-08	0.324050518	1.64718E-10	1.23896E-08	UBFD1	protein_coding
ENSG00000103528.17	0.327703266	7.75596E-11	5.81877E-09	0.345701257	5.75582E-12	7.31643E-10	SYT17	protein_coding
ENSG00000103549.22	0.301912183	2.41967E-09	1.11008E-07	0.303271817	2.45721E-09	1.19411E-07	RNF40	protein_coding
ENSG00000103591.13	0.326761852	8.84538E-11	6.54334E-09	0.324382751	1.57482E-10	1.19069E-08	AAGAB	protein_coding
ENSG00000103671.10	0.304818415	1.66922E-09	8.1021E-08	0.305799067	1.78835E-09	9.17303E-08	TRIP4	protein_coding
ENSG00000103855.18	0.494329154	1.67442E-24	8.86192E-21	0.50470793	0	0	CD276	protein_coding
ENSG00000103876.13	0.311240824	7.24181E-10	3.98491E-08	0.300533849	3.45533E-09	1.5978E-07	FAH	protein_coding

ENSG00000104067.17	0.368137577	1.766E-13	3.19294E-11	0.353880077	2.29134E-12	3.33493E-10	TJP1	protein_coding
ENSG00000104140.7	0.297239201	4.35822E-09	1.85473E-07	0.306758352	1.29984E-09	7.0989E-08	RHOV	protein_coding
ENSG00000104142.11	0.300611173	2.85341E-09	1.27588E-07	0.312034816	8.06138E-10	4.81351E-08	VPS18	protein_coding
ENSG00000104368.19	0.419226336	2.15277E-17	1.51E-14	0.391036068	2.93534E-15	1.22942E-12	PLAT	protein_coding
ENSG00000104427.12	0.33712076	2.03116E-11	1.86221E-09	0.322702697	1.97532E-10	1.44109E-08	ZC2HC1A	protein_coding
ENSG00000104447.13	0.347187563	4.60818E-12	5.1992E-10	0.322151326	2.12716E-10	1.53076E-08	TRPS1	protein_coding
ENSG00000104765.16	0.288662503	1.24899E-08	4.49683E-07	0.307230629	1.4918E-09	7.90983E-08	BNIP3L	protein_coding
ENSG00000104826.15	0.320094943	2.21497E-10	1.43959E-08	0.31030531	8.18882E-10	4.87461E-08	LHB	protein_coding
ENSG00000104881.16	0.316105313	3.79565E-10	2.28057E-08	0.306132001	1.40945E-09	7.56968E-08	PPP1R13L	protein_coding
ENSG00000105245.9	0.337584269	1.89928E-11	1.76352E-09	0.310783156	7.69099E-10	4.62078E-08	NUMBL	protein_coding
ENSG00000105355.9	0.354349406	1.55226E-12	2.03534E-10	0.343969052	1.00521E-11	1.16813E-09	PLIN3	protein_coding
ENSG00000105419.18	0.449246942	5.01608E-20	9.73421E-17	0.40283584	4.60404E-16	2.33077E-13	MEIS3	protein_coding
ENSG00000105443.15	0.325325042	1.08008E-10	7.7878E-09	0.330171351	7.13503E-11	6.08181E-09	CYTH2	protein_coding
ENSG00000105472.13	0.345553687	5.88394E-12	6.42685E-10	0.309495278	1.11763E-09	6.28842E-08	CLEC11A	protein_coding
ENSG00000105854.13	0.306458202	1.35129E-09	6.75856E-08	0.307836386	1.38124E-09	7.47332E-08	PON2	protein_coding
ENSG00000105856.14	0.33203513	4.21155E-11	3.48279E-09	0.330419615	6.89416E-11	5.9111E-09	HBP1	protein_coding
ENSG00000105971.15	0.385997967	9.02031E-15	2.52473E-12	0.36068654	7.96488E-13	1.36784E-10	CAV2	protein_coding
ENSG00000106080.11	0.386919467	7.69844E-15	2.22979E-12	0.366933802	2.144E-13	4.76409E-11	FKBP14	protein_coding
ENSG00000106089.12	0.28246656	2.61525E-08	8.43048E-07	0.315846626	4.90826E-10	3.16075E-08	STX1A	protein_coding
ENSG00000106366.9	0.358016973	8.79633E-13	1.24297E-10	0.306566845	1.62283E-09	8.48097E-08	SERPINE1	protein_coding
ENSG00000106624.11	0.404573695	3.35388E-16	1.51361E-13	0.352532484	2.81258E-12	3.94965E-10	AEBP1	protein_coding
ENSG00000107164.16	0.322267975	1.64627E-10	1.10929E-08	0.324053931	1.64642E-10	1.23896E-08	FUBP3	protein_coding
ENSG00000107175.12	0.331233725	4.71866E-11	3.82606E-09	0.33450586	3.89898E-11	3.622E-09	CREB3	protein_coding
ENSG00000107731.12	0.497783572	7.12381E-25	4.14734E-21	0.466864945	0	0	UNC5B	protein_coding
ENSG00000107862.6	0.32888066	6.57612E-11	5.05744E-09	0.300469906	3.48281E-09	1.60795E-07	GBF1	protein_coding
ENSG00000107957.16	0.430753099	2.25431E-18	2.1515E-15	0.391819228	3.28727E-15	1.35729E-12	SH3PXD2A	protein_coding
ENSG00000108256.9	0.366484198	2.3046E-13	4.0291E-11	0.369116168	2.028E-13	4.57621E-11	NUFIP2	protein_coding
ENSG00000108510.10	0.291982566	8.34361E-09	3.1769E-07	0.300924337	3.29206E-09	1.53081E-07	MED13	protein_coding
ENSG00000108557.19	0.307264349	1.21738E-09	6.15223E-08	0.304029355	2.23468E-09	1.10347E-07	RAI1	protein_coding
ENSG00000108587.16	0.324418474	1.22448E-10	8.64067E-09	0.314760041	5.65798E-10	3.57263E-08	GOSR1	protein_coding
ENSG00000108821.14	0.376900909	4.19842E-14	9.51066E-12	0.364391171	4.4061E-13	8.57908E-11	COL1A1	protein_coding
ENSG00000108828.16	0.299554275	3.26042E-09	1.43691E-07	0.309757652	1.08069E-09	6.13809E-08	VAT1	protein_coding
ENSG00000108947.5	0.322855083	1.51882E-10	1.03177E-08	0.345892236	5.59405E-12	7.17344E-10	EFNB3	protein_coding
ENSG00000109066.14	0.375575543	5.23191E-14	1.14508E-11	0.349618159	4.36227E-12	5.74577E-10	TMEM104	protein_coding
ENSG00000109079.10	0.414013318	5.80755E-17	3.63553E-14	0.379533735	3.26779E-14	9.90855E-12	TNFAIP1	protein_coding
ENSG00000109083.14	0.312176856	6.40113E-10	3.56272E-08	0.302521823	2.23913E-09	1.10473E-07	IFT20	protein_coding
ENSG00000109111.15	0.297747691	4.08999E-09	1.7521E-07	0.303239276	2.46724E-09	1.19798E-07	SUPT6H	protein_coding
ENSG00000109118.14	0.316417136	3.64023E-10	2.21449E-08	0.320132438	2.78614E-10	1.9287E-08	PHF12	protein_coding
ENSG00000109220.11	0.356790072	1.06456E-12	1.46517E-10	0.355273865	1.85102E-12	2.80695E-10	CHIC2	protein_coding
ENSG00000109255.11	0.27368838	7.22726E-08	2.00647E-06	0.312054614	8.04077E-10	4.80614E-08	NMU	protein_coding
ENSG00000109466.14	0.332824072	3.76438E-11	3.18104E-09	0.356823302	1.45757E-12	2.29964E-10	KLHL2	protein_coding
ENSG00000109625.19	0.377483505	3.8101E-14	8.87266E-12	0.375604232	5.20711E-14	1.43672E-11	CPZ	protein_coding
ENSG00000109680.11	0.297218135	4.36969E-09	1.85825E-07	0.319603841	2.36782E-10	1.68109E-08	TBC1D19	protein_coding
ENSG00000109736.15	0.324549121	1.20257E-10	8.51719E-09	0.305339857	1.89505E-09	9.63547E-08	MFSD10	protein_coding
ENSG00000109787.13	0.29886989	3.55344E-09	1.5473E-07	0.317576516	3.90961E-10	2.58941E-08	KLF3	protein_coding
ENSG00000109944.11	0.332224556	4.09969E-11	3.39994E-09	0.313068189	5.68919E-10	3.58068E-08	JHY	protein_coding
ENSG00000110057.8	0.337097219	2.03809E-11	1.86269E-09	0.327432928	1.03989E-10	8.33887E-09	UNC93B1	protein_coding
ENSG00000110080.20	0.498798583	5.53162E-25	3.57822E-21	0.479329189	6.13047E-23	5.09862E-20	ST3GAL4	protein_coding
ENSG00000110218.9	0.332495732	3.94456E-11	3.29949E-09	0.324878598	1.47255E-10	1.1295E-08	PANX1	protein_coding
ENSG00000110237.5	0.394042813	2.22372E-15	7.98078E-13	0.363397201	5.17139E-13	9.87108E-11	ARHGEF17	protein_coding
ENSG00000110318.15	0.310951777	7.52243E-10	4.12374E-08	0.302329651	2.29459E-09	1.12446E-07	CEP126	protein_coding
ENSG00000110321.19	0.285865784	1.74742E-08	5.98771E-07	0.30234839	2.75767E-09	1.31595E-07	EIF4G2	protein_coding
ENSG00000110330.8	0.320509316	2.09351E-10	1.36483E-08	0.336714662	2.15401E-11	2.20778E-09	BIRC2	protein_coding
ENSG00000110429.14	0.291044566	9.35585E-09	3.49826E-07	0.3038109	2.29676E-09	1.12458E-07	FBXO3	protein_coding
ENSG00000110811.20	0.417755616	2.85334E-17	1.93158E-14	0.39021982	3.58562E-15	1.45978E-12	P3H3	protein_coding
ENSG00000110841.14	0.296487287	4.78634E-09	2.00902E-07	0.315838662	4.91339E-10	3.16075E-08	PPFIBP1	protein_coding
ENSG00000110906.13	0.307604181	1.16488E-09	5.94364E-08	0.305468199	1.86462E-09	9.50565E-08	KCTD10	protein_coding
ENSG00000111199.12	0.447238388	7.66969E-20	1.39536E-16	0.42986397	2.69122E-18	1.9107E-15	TRPV4	protein_coding
ENSG00000111319.13	0.359914078	6.53823E-13	9.63653E-11	0.322864167	1.51693E-10	1.15592E-08	SCNN1A	protein_coding
ENSG00000111371.16	0.33217525	4.12852E-11	3.41898E-09	0.308402908	1.04998E-09	5.98121E-08	SLC38A1	protein_coding
ENSG00000111652.10	0.318174352	2.87347E-10	1.81047E-08	0.308893617	1.20703E-09	6.67976E-08	COPS7A	protein_coding
ENSG00000111711.10	0.288852288	1.22069E-08	4.42228E-07	0.307182842	1.50088E-09	7.95072E-08	GOLT1B	protein_coding
ENSG00000111799.21	0.444040612	1.49923E-19	2.56713E-16	0.424266925	0	0	COL12A1	protein_coding
ENSG00000111897.7	0.35547182	1.30561E-12	1.75138E-10	0.340439185	1.67672E-11	1.80102E-09	SERINC1	protein_coding
ENSG00000111907.21	0.365800929	2.57144E-13	4.45547E-11	0.362010013	6.45611E-13	1.1638E-10	TPD52L1	protein_coding
ENSG00000111981.5	0.317692341	3.06655E-10	1.91144E-08	0.355919944	1.21822E-12	2.00346E-10	ULBP1	protein_coding
ENSG00000112033.14	0.412416913	7.84303E-17	4.43306E-14	0.395220639	1.80568E-15	8.02467E-13	PPARD	protein_coding

ENSG00000112062.11	0.370710201	1.16356E-13	2.23564E-11	0.358893617	1.05599E-12	1.78196E-10	MAPK14	protein_coding
ENSG00000112078.14	0.346785949	4.89413E-12	5.48992E-10	0.326217772	1.22755E-10	9.61854E-09	KCTD20	protein_coding
ENSG00000112130.17	0.303243243	2.04234E-09	9.56564E-08	0.345462643	5.96437E-12	7.48411E-10	RNF8	protein_coding
ENSG00000112218.9	0.324327594	1.23996E-10	8.71833E-09	0.301296672	2.61625E-09	1.25567E-07	GPR63	protein_coding
ENSG00000112234.9	0.295979994	5.09792E-09	2.11993E-07	0.302200933	2.80884E-09	1.33708E-07	FBXL4	protein_coding
ENSG00000112319.19	0.381437325	1.96175E-14	5.00918E-12	0.393186451	2.58588E-15	1.09887E-12	EYA4	protein_coding
ENSG00000112378.12	0.373505778	7.36283E-14	1.53638E-11	0.382999659	1.68767E-14	5.61444E-12	PERP	protein_coding
ENSG00000112379.9	0.302083364	2.3676E-09	1.09394E-07	0.319299142	2.46776E-10	1.74143E-08	ARFGEF3	protein_coding
ENSG00000112473.18	0.345088647	6.30616E-12	6.83673E-10	0.352585505	2.79006E-12	3.94129E-10	SLC39A7	protein_coding
ENSG00000112541.17	0.270758057	1.00675E-07	2.68242E-06	0.306297574	1.37963E-09	7.47158E-08	PDE10A	protein_coding
ENSG00000112559.15	0.409482774	1.35678E-16	6.92887E-14	0.407760837	0	0	MDFI	protein_coding
ENSG00000112655.16	0.358212668	8.53199E-13	1.21744E-10	0.404228058	3.57254E-16	1.9258E-13	PTK7	protein_coding
ENSG00000112697.17	0.337627812	1.88733E-11	1.75803E-09	0.342119468	1.31549E-11	1.47279E-09	TMEM30A	protein_coding
ENSG00000112773.16	0.298370651	3.78313E-09	1.63752E-07	0.302302879	2.77337E-09	1.32128E-07	TENT5A	protein_coding
ENSG00000112972.15	0.295198805	5.6165E-09	2.2946E-07	0.30682444	1.57071E-09	8.26048E-08	HMGCS1	protein_coding
ENSG00000113083.15	0.398125851	1.07651E-15	4.18869E-13	0.381894321	1.81581E-14	5.97247E-12	LOX	protein_coding
ENSG00000113140.11	0.371027456	1.10492E-13	2.1442E-11	0.340856753	1.57885E-11	1.70533E-09	SPARC	protein_coding
ENSG00000113194.13	0.304151168	1.81841E-09	8.66318E-08	0.315689384	5.01045E-10	3.20547E-08	FAF2	protein_coding
ENSG00000113369.9	0.343360922	8.14978E-12	8.57982E-10	0.338910684	2.08784E-11	2.15591E-09	ARRDC3	protein_coding
ENSG00000113389.16	0.334094058	3.13998E-11	2.70419E-09	0.361300665	5.25712E-13	9.96936E-11	NPR3	protein_coding
ENSG00000113716.13	0.282777762	2.52103E-08	8.17224E-07	0.306135624	1.71387E-09	8.86127E-08	HMGXB3	protein_coding
ENSG00000113719.16	0.31232748	6.27502E-10	3.49923E-08	0.328872227	8.53523E-11	7.11897E-09	ERGIC1	protein_coding
ENSG00000113721.14	0.371702416	9.89621E-14	1.95301E-11	0.317121174	4.15144E-10	2.71866E-08	PDGFRB	protein_coding
ENSG00000113758.13	0.420411416	1.71384E-17	1.21678E-14	0.402211628	0	0	DBN1	protein_coding
ENSG00000114030.13	0.31637635	3.6602E-10	2.21968E-08	0.308460803	1.2756E-09	6.99936E-08	KPNA1	protein_coding
ENSG00000114251.15	0.390965454	3.81659E-15	1.21418E-12	0.377176151	4.01036E-14	1.18037E-11	WNT5A	protein_coding
ENSG00000114270.17	0.42776067	4.08361E-18	3.65753E-15	0.413505973	0	0	COL7A1	protein_coding
ENSG00000114316.13	0.326860187	8.72496E-11	6.47895E-09	0.317298214	4.05573E-10	2.68314E-08	USP4	protein_coding
ENSG00000114439.19	0.324065016	1.28573E-10	8.96588E-09	0.334811924	3.73468E-11	3.51253E-09	BBX	protein_coding
ENSG00000114480.13	0.342783083	8.87656E-12	9.21168E-10	0.382427352	1.88717E-14	6.13783E-12	GBE1	protein_coding
ENSG00000114544.17	0.327134297	8.39764E-11	6.24386E-09	0.341285698	1.4841E-11	1.61801E-09	SLC41A3	protein_coding
ENSG00000114554.11	0.31289363	5.82226E-10	3.27182E-08	0.316975765	4.23169E-10	2.76499E-08	PLXNA1	protein_coding
ENSG00000114626.18	0.403699163	3.93448E-16	1.73529E-13	0.389291864	5.10778E-15	1.9693E-12	ABTB1	protein_coding
ENSG00000114646.10	0.277701233	4.5612E-08	1.36107E-06	0.302266504	2.3131E-09	1.13163E-07	CSPG5	protein_coding
ENSG00000114648.12	0.286428463	1.63374E-08	5.65338E-07	0.319308681	3.10867E-10	2.12932E-08	KLHL18	protein_coding
ENSG00000114698.15	0.426298701	5.44724E-18	4.46659E-15	0.387514165	6.65426E-15	2.45309E-12	PLSCR4	protein_coding
ENSG00000114853.14	0.326829092	8.76287E-11	6.49881E-09	0.308640346	1.24671E-09	6.87321E-08	ZBTB47	protein_coding
ENSG00000114923.17	0.28314826	2.41313E-08	7.86606E-07	0.309606361	8.9738E-10	5.2296E-08	SLC4A3	protein_coding
ENSG00000115226.10	0.427536661	4.26832E-18	3.76504E-15	0.363414723	5.15685E-13	9.87108E-11	FNDC4	protein_coding
ENSG00000115310.18	0.310442336	8.04295E-10	4.34854E-08	0.306481056	1.64056E-09	8.55826E-08	RTN4	protein_coding
ENSG00000115339.14	0.303562598	1.96068E-09	9.26518E-08	0.337987731	1.79132E-11	1.90305E-09	GALNT3	protein_coding
ENSG00000115486.12	0.338024304	1.78183E-11	1.68128E-09	0.345895324	7.57915E-12	9.09779E-10	GGCX	protein_coding
ENSG00000115520.8	0.33796704	1.79671E-11	1.69257E-09	0.332633519	5.06817E-11	4.56748E-09	COQ10B	protein_coding
ENSG00000115598.10	0.331661589	4.44094E-11	3.63633E-09	0.342529431	9.21521E-12	1.07946E-09	IL1RL2	protein_coding
ENSG00000115806.13	0.353619856	1.7364E-12	2.24146E-10	0.354213449	2.17761E-12	3.20952E-10	GORASP2	protein_coding
ENSG00000115963.13	0.367010665	2.11766E-13	3.72465E-11	0.377636591	4.62709E-14	1.327E-11	RND3	protein_coding
ENSG00000116016.14	0.367667072	1.90527E-13	3.40248E-11	0.3269166	1.11595E-10	8.86332E-09	EPAS1	protein_coding
ENSG00000116260.17	0.283784949	2.23806E-08	7.36965E-07	0.310106952	1.03334E-09	5.92117E-08	QSOX1	protein_coding
ENSG00000116285.13	0.341387643	1.09023E-11	1.1077E-09	0.301637957	3.0128E-09	1.42147E-07	ERRFI1	protein_coding
ENSG00000116406.19	0.304385763	1.76454E-09	8.46193E-08	0.317875754	3.7582E-10	2.50911E-08	EDEM3	protein_coding
ENSG00000116525.14	0.363302539	3.82998E-13	6.09218E-11	0.328546497	6.89193E-11	5.9111E-09	TRIM62	protein_coding
ENSG00000116580.18	0.31273336	5.94711E-10	3.32912E-08	0.311043936	7.43183E-10	4.50225E-08	GON4L	protein_coding
ENSG00000116584.20	0.315844836	3.93042E-10	2.34448E-08	0.326810559	1.13222E-10	8.98035E-09	ARHGEF2	protein_coding
ENSG00000116604.18	0.392900788	2.7191E-15	9.42266E-13	0.380076004	2.95369E-14	9.0983E-12	MEF2D	protein_coding
ENSG00000117036.12	0.330681967	5.10189E-11	4.07437E-09	0.349637046	4.34997E-12	5.74255E-10	ETV3	protein_coding
ENSG00000117114.20	0.332596729	3.88826E-11	3.26177E-09	0.30051701	3.46255E-09	1.59986E-07	ADGRL2	protein_coding
ENSG00000117122.14	0.391901225	3.2404E-15	1.05983E-12	0.373689519	7.14356E-14	1.85662E-11	MFAP2	protein_coding
ENSG00000117139.18	0.424298741	8.06076E-18	6.25708E-15	0.431782025	1.83527E-18	1.33557E-15	KDM5B	protein_coding
ENSG00000117143.13	0.30710182	1.2433E-09	6.26146E-08	0.335233132	3.51954E-11	3.33171E-09	UAP1	protein_coding
ENSG00000117385.16	0.49953122	4.60604E-25	3.35193E-21	0.478296962	0	0	P3H1	protein_coding
ENSG00000117394.24	0.391983808	3.19385E-15	1.05647E-12	0.413599182	6.27931E-17	3.76875E-14	SLC2A1	protein_coding
ENSG00000117408.11	0.316104912	3.79586E-10	2.28057E-08	0.31493344	5.53129E-10	3.50785E-08	IPO13	protein_coding
ENSG00000117525.14	0.340270105	1.28438E-11	1.26951E-09	0.32175164	1.76691E-10	1.31374E-08	F3	protein_coding
ENSG00000117533.15	0.293636368	6.81138E-09	2.69575E-07	0.303707134	2.32684E-09	1.1374E-07	VAMP4	protein_coding
ENSG00000117899.11	0.291622342	8.71913E-09	3.29831E-07	0.317835931	3.77801E-10	2.51945E-08	MESD	protein_coding
ENSG00000117983.17	0.383271769	1.43732E-14	3.82091E-12	0.406762772	0	0	MUC5B	protein_coding
ENSG00000118200.14	0.327542635	7.93212E-11	5.92801E-09	0.334606212	3.84435E-11	3.59823E-09	CAMSAP2	protein_coding

ENSG00000118217.6	0.325319873	1.08086E-10	7.7878E-09	0.341985209	1.34132E-11	1.4931E-09	ATF6	protein_coding
ENSG00000118369.13	0.334449063	2.98431E-11	2.59315E-09	0.35104335	3.52223E-12	4.76878E-10	USP35	protein_coding
ENSG00000118454.13	0.328807029	6.64447E-11	5.07784E-09	0.323170686	1.45434E-10	1.11949E-08	ANKRD13C	protein_coding
ENSG00000118495.20	0.315998926	3.85014E-10	2.30842E-08	0.300105752	3.04149E-09	1.43144E-07	PLAGL1	protein_coding
ENSG00000118689.15	0.342226822	9.63574E-12	9.92874E-10	0.352514052	2.82046E-12	3.94965E-10	FOXO3	protein_coding
ENSG00000118849.10	0.396742005	1.37812E-15	5.1103E-13	0.39329389	1.60683E-15	7.30832E-13	RARRES1	protein_coding
ENSG00000118855.21	0.311781912	6.74359E-10	3.73192E-08	0.319783138	2.91872E-10	2.0133E-08	MFSD1	protein_coding
ENSG00000118898.16	0.394548277	2.03382E-15	7.35434E-13	0.37838935	4.03473E-14	1.18037E-11	PPL	protein_coding
ENSG00000119681.12	0.41601228	3.97752E-17	2.54465E-14	0.374279213	8.40392E-14	2.14587E-11	LTBP2	protein_coding
ENSG00000119771.15	0.32572457	1.02184E-10	7.42691E-09	0.326799181	1.13398E-10	8.98207E-09	KLHL29	protein_coding
ENSG00000119777.20	0.392093625	3.13297E-15	1.04503E-12	0.38748299	6.69966E-15	2.45309E-12	TMEM214	protein_coding
ENSG00000120093.11	0.297196141	4.3817E-09	1.862E-07	0.31044806	9.89054E-10	5.68418E-08	HOXB3	protein_coding
ENSG00000120129.6	0.320722703	2.03351E-10	1.3287E-08	0.31691478	4.26579E-10	2.78103E-08	DUSP1	protein_coding
ENSG00000120137.7	0.282258931	2.67999E-08	8.59634E-07	0.306930936	1.54964E-09	8.16442E-08	PANK3	protein_coding
ENSG00000120324.9	0.328648087	6.79439E-11	5.15718E-09	0.333896689	4.24716E-11	3.91856E-09	PCDHB10	protein_coding
ENSG00000120327.6	0.334185752	3.09903E-11	2.67443E-09	0.335047446	3.61285E-11	3.4145E-09	PCDHB14	protein_coding
ENSG00000120328.6	0.328841099	6.61276E-11	5.07222E-09	0.363547203	3.684E-13	7.65982E-11	PCDHB12	protein_coding
ENSG00000120820.12	0.33618321	2.32577E-11	2.07353E-09	0.302013426	2.87523E-09	1.36631E-07	GLT8D2	protein_coding
ENSG00000121005.9	0.312955986	5.77438E-10	3.24805E-08	0.306537035	1.62897E-09	8.50542E-08	CRISPLD1	protein_coding
ENSG00000121281.13	0.307295408	1.21249E-09	6.13283E-08	0.300046422	3.67019E-09	1.68378E-07	ADCY7	protein_coding
ENSG00000121481.11	0.310848072	7.62567E-10	4.17445E-08	0.343297303	1.10864E-11	1.27304E-09	RNF2	protein_coding
ENSG00000121742.19	0.223332797	1.26748E-05	0.000170692	0.301981912	2.39833E-09	1.16744E-07	GJB6	protein_coding
ENSG00000121753.12	0.28618922	1.68117E-08	5.78455E-07	0.303076021	2.0864E-09	1.04442E-07	ADGRB2	protein_coding
ENSG00000121964.14	0.283006692	2.45382E-08	7.98854E-07	0.30407238	1.83686E-09	9.38877E-08	GTDC1	protein_coding
ENSG00000122068.13	0.313058902	5.69619E-10	3.2165E-08	0.316773922	4.34558E-10	2.82356E-08	FYTTD1	protein_coding
ENSG00000122218.16	0.364649679	3.09094E-13	5.15611E-11	0.381966549	2.06313E-14	6.67284E-12	COPA	protein_coding
ENSG00000122417.15	0.326329307	9.39458E-11	6.90573E-09	0.310110269	8.40089E-10	4.97036E-08	ODF2L	protein_coding
ENSG00000122641.11	0.381470989	1.95062E-14	5.0027E-12	0.364869268	4.07797E-13	8.15847E-11	INHBA	protein_coding
ENSG00000122642.11	0.374911522	5.83957E-14	1.25449E-11	0.385542518	9.75337E-15	3.44134E-12	FKBP9	protein_coding
ENSG00000122778.10	0.351904954	2.25738E-12	2.80812E-10	0.378553647	3.9152E-14	1.16293E-11	KIAA1549	protein_coding
ENSG00000122861.16	0.406997674	2.14924E-16	1.01727E-13	0.375490044	6.79348E-14	1.78961E-11	PLAU	protein_coding
ENSG00000123094.15	0.32864816	6.79432E-11	5.15718E-09	0.307684833	1.40813E-09	7.56957E-08	RASSF8	protein_coding
ENSG00000123384.14	0.324863651	1.15137E-10	8.21452E-09	0.323464103	1.78285E-10	1.32222E-08	LRP1	protein_coding
ENSG00000123500.10	0.384372809	1.19142E-14	3.24121E-12	0.388518375	5.32628E-15	2.0267E-12	COL10A1	protein_coding
ENSG00000123562.18	0.341031359	1.14879E-11	1.15113E-09	0.365196041	3.86734E-13	7.89996E-11	MORF4L2	protein_coding
ENSG00000123983.14	0.310707471	7.76783E-10	4.23434E-08	0.332199568	5.38435E-11	4.8004E-09	ACSL3	protein_coding
ENSG00000123989.14	0.420776829	1.59719E-17	1.14796E-14	0.437161452	0	0	CHPF	protein_coding
ENSG00000123999.5	0.158690684	0.002053891	0.01288178	0.300161275	3.02025E-09	1.42375E-07	INHA	protein_coding
ENSG00000124006.15	0.348994459	3.511E-12	4.1517E-10	0.325206281	1.40854E-10	1.08756E-08	OBSL1	protein_coding
ENSG00000124067.17	0.373469848	7.40647E-14	1.53996E-11	0.30434839	2.14693E-09	1.06835E-07	SLC12A4	protein_coding
ENSG00000124107.5	0.355117605	1.37899E-12	1.83292E-10	0.371158266	1.438E-13	3.36488E-11	SLPI	protein_coding
ENSG00000124216.4	0.355901805	1.22164E-12	1.65016E-10	0.335466056	2.57885E-11	2.55333E-09	SNAI1	protein_coding
ENSG00000124225.16	0.349207671	3.39974E-12	4.03931E-10	0.350755717	3.67803E-12	4.9452E-10	PMEPA1	protein_coding
ENSG00000124356.16	0.310317999	8.1752E-10	4.41097E-08	0.319760155	2.92766E-10	2.01707E-08	STAMBP	protein_coding
ENSG00000124762.14	0.359394776	7.09273E-13	1.04011E-10	0.364128115	4.59731E-13	8.92154E-11	CDKN1A	protein_coding
ENSG00000124813.23	0.360521478	5.9433E-13	8.80425E-11	0.369386278	1.93846E-13	4.39118E-11	RUNX2	protein_coding
ENSG00000124831.19	0.35534095	1.33226E-12	1.77894E-10	0.33155103	5.89292E-11	5.19809E-09	LRRFIP1	protein_coding
ENSG00000125037.12	0.315658435	4.0297E-10	2.39389E-08	0.31608852	4.755E-10	3.06903E-08	EMC3	protein_coding
ENSG00000125266.8	0.343517773	7.96274E-12	8.41334E-10	0.321657299	2.2728E-10	1.62015E-08	EFNB2	protein_coding
ENSG00000125459.17	0.31592773	3.88703E-10	2.32575E-08	0.320359786	2.13657E-10	1.53564E-08	MSTO1	protein_coding
ENSG00000125505.17	0.352368922	2.10302E-12	2.64435E-10	0.343548527	1.0688E-11	1.23581E-09	MBOAT7	protein_coding
ENSG00000125531.7	0.30522855	1.58349E-09	7.72739E-08	0.323599878	1.3709E-10	1.06273E-08	FNDC11	protein_coding
ENSG00000125733.18	0.341553994	1.0639E-11	1.08663E-09	0.328596427	8.86517E-11	7.34157E-09	TRIP10	protein_coding
ENSG00000125734.15	0.321366766	1.86239E-10	1.2321E-08	0.310089202	1.0357E-09	5.92301E-08	GPR108	protein_coding
ENSG00000125746.18	0.287452133	1.44501E-08	5.10161E-07	0.300834452	3.32896E-09	1.54673E-07	EML2	protein_coding
ENSG00000126603.9	0.398007661	1.09951E-15	4.23916E-13	0.336043729	2.37301E-11	2.38193E-09	GLIS2	protein_coding
ENSG00000126705.15	0.366400707	2.33569E-13	4.07123E-11	0.360900444	7.69989E-13	1.33813E-10	AHDC1	protein_coding
ENSG00000126822.17	0.411322635	9.62783E-17	5.23844E-14	0.403008556	4.46171E-16	2.27852E-13	PLEKHG3	protein_coding
ENSG00000127377.10	0.235489658	4.02657E-06	6.35799E-05	0.302833573	2.15193E-09	1.06987E-07	CRYGN	protein_coding
ENSG00000127418.15	0.289402146	1.14215E-08	4.17936E-07	0.362846513	5.64882E-13	1.05405E-10	FGFRL1	protein_coding
ENSG00000127946.17	0.318756709	2.65593E-10	1.68251E-08	0.310602116	9.69661E-10	5.58375E-08	HIP1	protein_coding
ENSG00000127954.13	0.341734364	1.03605E-11	1.06004E-09	0.306973945	1.54121E-09	8.12735E-08	STEAP4	protein_coding
ENSG00000127955.17	0.333119752	3.609E-11	3.06591E-09	0.319442485	3.05392E-10	2.10157E-08	GNAI1	protein_coding
ENSG00000128274.17	0.386835558	7.81049E-15	2.24949E-12	0.335838662	3.23123E-11	3.08893E-09	A4GALT	protein_coding
ENSG00000128342.5	0.410581225	1.10579E-16	5.78751E-14	0.412717488	0	0	LIF	protein_coding
ENSG00000128422.17	0.434735326	1.01308E-18	1.23825E-15	0.453771786	1.90732E-20	1.48053E-17	KRT17	protein_coding
ENSG00000128487.17	0.357777301	9.13099E-13	1.27786E-10	0.358800091	1.07156E-12	1.79265E-10	SPECC1	protein_coding

ENSG00000128567.17	0.309284625	9.35925E-10	4.93548E-08	0.309483673	1.11929E-09	6.28986E-08	PODXL	protein_coding
ENSG00000128595.17	0.372958767	8.05534E-14	1.65712E-11	0.360934805	7.65813E-13	1.33485E-10	CALU	protein_coding
ENSG00000128606.13	0.311672671	6.84142E-10	3.78246E-08	0.304889783	1.65399E-09	8.61286E-08	LRRC17	protein_coding
ENSG00000128641.19	0.335176976	2.6883E-11	2.36059E-09	0.338342701	2.26432E-11	2.29659E-09	MYO1B	protein_coding
ENSG00000128655.18	0.292735075	7.60901E-09	2.94119E-07	0.318750347	2.65821E-10	1.85336E-08	PDE11A	protein_coding
ENSG00000128791.12	0.349974893	3.02714E-12	3.65631E-10	0.347668677	5.83213E-12	7.3812E-10	TWSG1	protein_coding
ENSG00000128923.11	0.330392234	5.3151E-11	4.21573E-09	0.320017749	2.82901E-10	1.95605E-08	MINDY2	protein_coding
ENSG00000129009.13	0.351473047	2.41099E-12	2.98645E-10	0.334636705	3.82789E-11	3.5886E-09	ISLR	protein_coding
ENSG00000129038.16	0.322064175	1.6929E-10	1.13676E-08	0.303329617	2.4395E-09	1.18649E-07	LOXL1	protein_coding
ENSG00000129219.14	0.329505016	6.02339E-11	4.70067E-09	0.307892365	1.37143E-09	7.43408E-08	PLD2	protein_coding
ENSG00000129451.12	0.334919358	2.78963E-11	2.44283E-09	0.32635808	9.35704E-11	7.68741E-09	KLK10	protein_coding
ENSG00000130164.14	0.314755586	4.54614E-10	2.65464E-08	0.315225168	5.32434E-10	3.38398E-08	LDLR	protein_coding
ENSG00000130202.10	0.313650529	5.26627E-10	3.01171E-08	0.322315394	2.08083E-10	1.51049E-08	NECTIN2	protein_coding
ENSG00000130340.16	0.414484571	5.31299E-17	3.36209E-14	0.393494596	1.51611E-15	6.94997E-13	SNX9	protein_coding
ENSG00000130363.12	0.392484965	2.92513E-15	1.00045E-12	0.38163022	2.20086E-14	7.00163E-12	RSPH3	protein_coding
ENSG00000130449.6	0.302160482	2.3445E-09	1.08499E-07	0.302541814	2.69192E-09	1.28774E-07	ZSWIM6	protein_coding
ENSG00000130479.11	0.289905654	1.07454E-08	3.95682E-07	0.31188076	8.22346E-10	4.89023E-08	MAP1S	protein_coding
ENSG00000130508.11	0.358595812	8.03675E-13	1.16679E-10	0.332900444	4.88274E-11	4.40718E-09	PXDN	protein_coding
ENSG00000130635.16	0.412902915	7.15866E-17	4.16763E-14	0.394938446	9.66696E-16	4.61304E-13	COL5A1	protein_coding
ENSG00000130702.15	0.379641361	2.6551E-14	6.455E-12	0.357021504	1.4135E-12	2.2594E-10	LAMA5	protein_coding
ENSG00000130720.13	0.301830169	2.44501E-09	1.11818E-07	0.383756872	1.32339E-14	4.53207E-12	FIBCD1	protein_coding
ENSG00000130821.17	0.362807473	4.14288E-13	6.49351E-11	0.304394584	2.13451E-09	1.06393E-07	SLC6A8	protein_coding
ENSG00000130827.6	0.361318945	5.24199E-13	7.90618E-11	0.351229947	3.42461E-12	4.66918E-10	PLXNA3	protein_coding
ENSG00000130962.18	0.387603828	6.84154E-15	1.9915E-12	0.389756536	4.71158E-15	1.84093E-12	PRRG1	protein_coding
ENSG00000131015.5	0.461110791	3.85356E-21	1.18297E-17	0.480938901	0	0	ULBP2	protein_coding
ENSG00000131019.11	0.344060124	7.34773E-12	7.83462E-10	0.371550575	1.34521E-13	3.19655E-11	ULBP3	protein_coding
ENSG00000131236.18	0.337643475	1.88305E-11	1.75803E-09	0.32898464	8.40421E-11	7.03994E-09	CAP1	protein_coding
ENSG00000131386.19	0.357883788	8.98079E-13	1.25986E-10	0.360250488	6.20189E-13	1.12832E-10	GALNT15	protein_coding
ENSG00000131446.17	0.31727986	3.24175E-10	2.00877E-08	0.303021277	2.53544E-09	1.227E-07	MGAT1	protein_coding
ENSG00000131473.17	0.194235777	0.000153764	0.001442216	0.30449744	2.10709E-09	1.05297E-07	ACLY	protein_coding
ENSG00000131626.19	0.294397372	6.2015E-09	2.48866E-07	0.313398339	6.7558E-10	4.162E-08	PPFIA1	protein_coding
ENSG00000131788.16	0.394781134	1.95178E-15	7.14645E-13	0.378344088	3.30004E-14	9.95451E-12	PIAS3	protein_coding
ENSG00000131871.15	0.372375614	8.86383E-14	1.78559E-11	0.393123677	1.68684E-15	7.61275E-13	SELENOS	protein_coding
ENSG00000131873.7	0.328974582	6.48992E-11	4.99776E-09	0.327396309	8.09597E-11	6.83089E-09	CHSY1	protein_coding
ENSG00000132000.13	0.369754631	1.35919E-13	2.55725E-11	0.365169416	3.8841E-13	7.90646E-11	PODNL1	protein_coding
ENSG00000132003.9	0.417025094	3.28024E-17	2.1701E-14	0.402330641	0	0	ZSWIM4	protein_coding
ENSG00000132031.13	0.349608236	3.19994E-12	3.83322E-10	0.317350115	3.21124E-10	2.18657E-08	MATN3	protein_coding
ENSG00000132205.11	0.328841467	6.61242E-11	5.07222E-09	0.347714871	5.7923E-12	7.34676E-10	EMILIN2	protein_coding
ENSG00000132376.20	0.34828699	3.90626E-12	4.53922E-10	0.345178974	8.4206E-12	9.94382E-10	INPP5K	protein_coding
ENSG00000132471.12	0.318102178	2.90161E-10	1.82229E-08	0.31482717	5.6086E-10	3.54915E-08	WBP2	protein_coding
ENSG00000132669.13	0.336528123	2.21284E-11	1.98807E-09	0.312818978	7.28329E-10	4.43534E-08	RIN2	protein_coding
ENSG00000133026.13	0.365067517	2.8915E-13	4.87699E-11	0.339132799	1.51648E-11	1.65021E-09	MYH10	protein_coding
ENSG00000133030.22	0.345856726	5.62379E-12	6.16583E-10	0.320651269	2.59996E-10	1.81492E-08	MPRIP	protein_coding
ENSG00000133103.17	0.293165934	7.21703E-09	2.81044E-07	0.317138259	3.30411E-10	2.24194E-08	COG6	protein_coding
ENSG00000133422.14	0.364238774	3.30016E-13	5.43947E-11	0.356260232	1.15571E-12	1.91448E-10	MORC2	protein_coding
ENSG00000133466.14	0.416444339	3.66385E-17	2.39665E-14	0.387507339	6.66418E-15	2.45309E-12	C1QTNF6	protein_coding
ENSG00000133703.13	0.320972997	1.96527E-10	1.28699E-08	0.379035385	3.58342E-14	1.07536E-11	KRAS	protein_coding
ENSG00000133805.15	0.365668648	2.62648E-13	4.52392E-11	0.322584822	2.00687E-10	1.46045E-08	AMPD3	protein_coding
ENSG00000133816.17	0.309552787	9.0369E-10	4.7959E-08	0.315552623	5.101E-10	3.25983E-08	MICAL2	protein_coding
ENSG00000134013.16	0.33984379	1.36701E-11	1.33262E-09	0.347380856	4.47643E-12	5.86957E-10	LOXL2	protein_coding
ENSG00000134107.5	0.424181527	8.24737E-18	6.31771E-15	0.44227944	0	0	BHLHE40	protein_coding
ENSG00000134108.14	0.335558958	2.54461E-11	2.2514E-09	0.31112527	9.06525E-10	5.26708E-08	ARL8B	protein_coding
ENSG00000134250.20	0.379699537	2.62928E-14	6.43157E-12	0.356392536	1.55799E-12	2.44482E-10	NOTCH2	protein_coding
ENSG00000134278.15	0.413720471	6.13738E-17	3.72193E-14	0.399627034	4.26778E-17	2.61539E-14	SPIRE1	protein_coding
ENSG00000134294.14	0.312834217	5.86824E-10	3.29448E-08	0.312357947	7.73143E-10	4.64029E-08	SLC38A2	protein_coding
ENSG00000134369.15	0.457788388	7.98677E-21	2.21416E-17	0.429097053	0	0	NAV1	protein_coding
ENSG00000134504.14	0.314917276	4.44915E-10	2.60584E-08	0.339396216	1.45933E-11	1.59398E-09	KCTD1	protein_coding
ENSG00000134531.10	0.316937516	3.39452E-10	2.08023E-08	0.301214018	3.17579E-09	1.48505E-07	EMP1	protein_coding
ENSG00000134590.14	0.342243954	9.61144E-12	9.92874E-10	0.31723336	4.09054E-10	2.69088E-08	RTL8C	protein_coding
ENSG00000134668.12	0.319212585	2.49689E-10	1.59917E-08	0.352214265	2.15329E-12	3.18173E-10	SPOCD1	protein_coding
ENSG00000134686.18	0.351619485	2.3578E-12	2.92679E-10	0.347336443	6.1265E-12	7.67038E-10	PHC2	protein_coding
ENSG00000134755.17	0.340007139	1.33475E-11	1.30599E-09	0.309709882	8.85309E-10	5.1852E-08	DSC2	protein_coding
ENSG00000134824.14	0.355938905	1.21465E-12	1.64453E-10	0.357220617	1.37052E-12	2.20411E-10	FADS2	protein_coding
ENSG00000134851.13	0.31713787	3.30428E-10	2.0378E-08	0.347773259	4.22018E-12	5.60937E-10	TMEM165	protein_coding
ENSG00000134901.13	0.270588229	1.02615E-07	2.72291E-06	0.306192986	1.3984E-09	7.53813E-08	POGLUT2	protein_coding
ENSG00000134909.18	0.282691359	2.54685E-08	8.24654E-07	0.306090568	1.72366E-09	8.904E-08	ARHGAP32	protein_coding
ENSG00000135046.14	0.28848265	1.2764E-08	4.58416E-07	0.321161224	2.42878E-10	1.71809E-08	ANXA1	protein_coding

ENSG00000135241.17	0.343274766	8.25434E-12	8.65858E-10	0.363575833	5.02503E-13	9.65503E-11	PNPLA8	protein_coding
ENSG00000135253.15	0.315388379	4.17788E-10	2.46432E-08	0.340493838	1.24299E-11	1.40269E-09	KCP	protein_coding
ENSG00000135318.12	0.367064246	2.09949E-13	3.71514E-11	0.321027193	2.47268E-10	1.74279E-08	NT5E	protein_coding
ENSG00000135341.18	0.34832808	3.88216E-12	4.52929E-10	0.353994766	2.25158E-12	3.29153E-10	MAP3K7	protein_coding
ENSG00000135454.14	0.333232659	3.55134E-11	3.02269E-09	0.303975607	1.85976E-09	9.48919E-08	B4GALNT1	protein_coding
ENSG00000135477.11	0.364508583	3.16127E-13	5.25837E-11	0.351751309	2.3109E-12	3.34666E-10	KRT87P	transcribed_unprocessed_pseudogene
ENSG00000135480.16	0.432876302	1.47359E-18	1.58869E-15	0.422151326	0	0	KRT7	protein_coding
ENSG00000135631.17	0.469782337	5.53727E-22	2.0148E-18	0.447323017	0	0	RAB11FIP5	protein_coding
ENSG00000135750.15	0.304516367	1.73522E-09	8.35576E-08	0.312600523	7.4924E-10	4.52952E-08	KCNK1	protein_coding
ENSG00000135828.11	0.413596201	6.28284E-17	3.77087E-14	0.403981728	3.73685E-16	1.99052E-13	RNASEL	protein_coding
ENSG00000135862.6	0.364330109	3.25249E-13	5.39469E-11	0.333509159	4.4842E-11	4.09187E-09	LAMC1	protein_coding
ENSG00000135916.16	0.405172829	3.00556E-16	1.37778E-13	0.384106724	1.35496E-14	4.60602E-12	ITM2C	protein_coding
ENSG00000135956.9	0.371958757	9.48992E-14	1.8792E-11	0.337621117	2.50955E-11	2.50173E-09	TMEM127	protein_coding
ENSG00000136002.20	0.437618528	5.63981E-19	7.38811E-16	0.460339226	4.56746E-21	3.64258E-18	ARHGEF4	protein_coding
ENSG00000136068.15	0.372873206	8.16928E-14	1.67464E-11	0.343675731	1.04917E-11	1.21674E-09	FLNB	protein_coding
ENSG00000136153.20	0.344796118	6.58676E-12	7.11443E-10	0.343968825	1.00524E-11	1.16813E-09	LMO7	protein_coding
ENSG00000136155.17	0.2865888	1.60268E-08	5.5638E-07	0.327116495	8.41853E-11	7.04181E-09	SCEL	protein_coding
ENSG00000136193.17	0.424966137	7.07465E-18	5.56584E-15	0.362868131	5.62931E-13	1.05378E-10	SCRN1	protein_coding
ENSG00000136378.15	0.391940886	3.21796E-15	1.05844E-12	0.361068857	5.45273E-13	1.02402E-10	ADAMTS7	protein_coding
ENSG00000136381.13	0.294046216	6.47605E-09	2.57881E-07	0.30635863	1.66619E-09	8.66864E-08	IREB2	protein_coding
ENSG00000136448.13	0.337628803	1.88706E-11	1.75803E-09	0.337802935	2.44543E-11	2.4504E-09	NMT1	protein_coding
ENSG00000136542.9	0.30052868	2.88332E-09	1.28629E-07	0.312944135	7.16605E-10	4.37768E-08	GALNT5	protein_coding
ENSG00000136631.15	0.306190374	1.39887E-09	6.9369E-08	0.312280806	7.80899E-10	4.6772E-08	VPS45	protein_coding
ENSG00000136802.12	0.37607973	4.81232E-14	1.06526E-11	0.364582546	4.27182E-13	8.4304E-11	LRRC8A	protein_coding
ENSG00000136827.12	0.30194926	2.4083E-09	1.1066E-07	0.308911139	1.20434E-09	6.67117E-08	TOR1A	protein_coding
ENSG00000136830.12	0.380750868	2.20281E-14	5.51566E-12	0.356878143	1.44525E-12	2.29026E-10	NIBAN2	protein_coding
ENSG00000136944.19	0.272447909	8.31989E-08	2.26976E-06	0.338777593	1.59701E-11	1.72175E-09	LMX1B	protein_coding
ENSG00000136999.5	0.368872467	1.56817E-13	2.87999E-11	0.360998521	7.58126E-13	1.33417E-10	CCN3	protein_coding
ENSG00000137207.12	0.36241886	4.40589E-13	6.82187E-11	0.371610877	1.33146E-13	3.17685E-11	YIPF3	protein_coding
ENSG00000137312.15	0.331526476	4.52688E-11	3.69112E-09	0.320838776	2.5357E-10	1.77818E-08	FLOT1	protein_coding
ENSG00000137331.12	0.267974247	1.37436E-07	3.50164E-06	0.313411765	6.74403E-10	4.16057E-08	IER3	protein_coding
ENSG00000137440.5	0.298346967	3.79438E-09	1.63873E-07	0.339472002	1.44328E-11	1.57941E-09	FGFBP1	protein_coding
ENSG00000137573.14	0.381898969	1.81438E-14	4.69464E-12	0.357438844	1.32487E-12	2.14253E-10	SULF1	protein_coding
ENSG00000137691.13	0.32994305	5.66291E-11	4.45518E-09	0.335550992	2.54753E-11	2.53093E-09	CFAP300	protein_coding
ENSG00000137699.17	0.325725219	1.02175E-10	7.42691E-09	0.343036068	1.1516E-11	1.31458E-09	TRIM29	protein_coding
ENSG00000137745.12	0.266562723	1.60716E-07	4.00539E-06	0.334502353	2.96161E-11	2.87844E-09	MMP13	protein_coding
ENSG00000137801.11	0.340689529	1.20786E-11	1.19998E-09	0.300944362	3.28389E-09	1.52823E-07	THBS1	protein_coding
ENSG00000137809.17	0.402616091	4.7916E-16	2.05925E-13	0.38713551	7.2241E-15	2.59613E-12	ITGA11	protein_coding
ENSG00000137843.12	0.324183351	1.2649E-10	8.84036E-09	0.336227709	2.31089E-11	2.33568E-09	PAK6	protein_coding
ENSG00000137857.17	0.329427989	6.08905E-11	4.7419E-09	0.365040164	3.96648E-13	7.99033E-11	DUOX1	protein_coding
ENSG00000138069.18	0.342825741	8.82081E-12	9.17018E-10	0.338908181	2.08858E-11	2.15591E-09	RAB1A	protein_coding
ENSG00000138131.4	0.324712845	1.17565E-10	8.35702E-09	0.317276368	4.06743E-10	2.68478E-08	LOXL4	protein_coding
ENSG00000138311.18	0.25739357	4.34569E-07	9.46283E-06	0.38965388	4.79643E-15	1.86159E-12	ZNF365	protein_coding
ENSG00000138316.11	0.392078488	3.14129E-15	1.04503E-12	0.386638298	8.03636E-15	2.87031E-12	ADAMTS14	protein_coding
ENSG00000138386.17	0.371674527	9.94143E-14	1.95491E-11	0.412325862	0	0	NAB1	protein_coding
ENSG00000138448.12	0.321356899	1.8649E-10	1.23236E-08	0.309577426	1.10593E-09	6.24197E-08	ITGAV	protein_coding
ENSG00000138463.9	0.325599071	1.0398E-10	7.54801E-09	0.317758562	3.8168E-10	2.5366E-08	SLC49A4	protein_coding
ENSG00000138594.14	0.323782318	1.33686E-10	9.26539E-09	0.317247468	4.08295E-10	2.69088E-08	TMOD3	protein_coding
ENSG00000138674.17	0.314603649	4.63916E-10	2.70082E-08	0.338559127	1.64859E-11	1.77408E-09	SEC31A	protein_coding
ENSG00000138696.11	0.23368689	4.79185E-06	7.38411E-05	0.302458921	2.25714E-09	1.11079E-07	BMPR1B	protein_coding
ENSG00000138738.11	0.344744828	6.6372E-12	7.12923E-10	0.319529994	2.39167E-10	1.69596E-08	PRDM5	protein_coding
ENSG00000138758.12	0.290163072	1.04148E-08	3.84239E-07	0.320182273	2.76771E-10	1.92259E-08	SEPTIN11	protein_coding
ENSG00000138764.15	0.323670556	1.35761E-10	9.36463E-09	0.310423939	9.92124E-10	5.6962E-08	CCNG2	protein_coding
ENSG00000138802.11	0.299723439	3.19169E-09	1.40982E-07	0.311800887	8.30873E-10	4.93087E-08	SEC24B	protein_coding
ENSG00000138814.17	0.406175021	2.50064E-16	1.16466E-13	0.435953148	7.91576E-19	5.9082E-16	PPP3CA	protein_coding
ENSG00000139112.11	0.353714544	1.71135E-12	2.21896E-10	0.314077597	6.18454E-10	3.86736E-08	GABARAPL1	protein_coding
ENSG00000139117.14	0.383947957	1.28098E-14	3.45261E-12	0.329442257	7.89072E-11	6.67707E-09	CPNE8	protein_coding
ENSG00000139209.16	0.139361725	0.006873318	0.034896076	0.322749739	1.54096E-10	1.16964E-08	SLC38A4	protein_coding
ENSG00000139211.7	0.370509885	1.20214E-13	2.29463E-11	0.338504494	2.21262E-11	2.25595E-09	AMIGO2	protein_coding
ENSG00000139318.8	0.275432116	5.92252E-08	1.696E-06	0.309511662	1.11529E-09	6.28556E-08	DUSP6	protein_coding
ENSG00000139324.12	0.32180754	1.75345E-10	1.16933E-08	0.333011264	4.80771E-11	4.35296E-09	TMTC3	protein_coding
ENSG00000139329.5	0.356081663	1.18811E-12	1.61234E-10	0.319197861	3.15473E-10	2.15566E-08	LUM	protein_coding
ENSG00000139725.8	0.335898992	2.42303E-11	2.15365E-09	0.30977667	8.77605E-10	5.16085E-08	RHOF	protein_coding
ENSG00000139926.16	0.391615814	3.40644E-15	1.10791E-12	0.362647307	4.24937E-13	8.4304E-11	FRMD6	protein_coding
ENSG00000139971.15	0.380712928	2.21695E-14	5.51566E-12	0.37536759	5.41523E-14	1.48011E-11	ARMH4	protein_coding
ENSG00000139990.18	0.30883832	9.92068E-10	5.18458E-08	0.309718529	8.84308E-10	5.1852E-08	DCAF5	protein_coding
ENSG00000140254.12	0.292493241	7.83798E-09	3.01594E-07	0.349237041	3.38468E-12	4.62558E-10	DUOXA1	protein_coding

ENSG00000140367.12	0.389974538	4.53624E-15	1.3973E-12	0.40337217	0	0	UBE2Q2	protein_coding
ENSG00000140403.12	0.40279032	4.64229E-16	2.0169E-13	0.359993876	6.45687E-13	1.1638E-10	DNAJA4	protein_coding
ENSG00000140406.4	0.276947832	4.97569E-08	1.46301E-06	0.318372966	3.51913E-10	2.36578E-08	TLNRD1	protein_coding
ENSG00000140521.17	0.293112714	7.26437E-09	2.8251E-07	0.300166117	3.61626E-09	1.66165E-07	POLG	protein_coding
ENSG00000140553.18	0.319374581	2.44264E-10	1.56787E-08	0.311677324	8.44235E-10	4.98982E-08	UNC45A	protein_coding
ENSG00000140564.12	0.337524051	1.91593E-11	1.77332E-09	0.342009102	1.33669E-11	1.4931E-09	FURIN	protein_coding
ENSG00000140688.17	0.309463318	9.14323E-10	4.84791E-08	0.309646604	1.09618E-09	6.2079E-08	RUSF1	protein_coding
ENSG00000140743.8	0.314436858	4.74339E-10	2.75325E-08	0.346413016	7.02248E-12	8.55331E-10	CDR2	protein_coding
ENSG00000140848.17	0.326225055	9.53184E-11	6.97141E-09	0.307166231	1.50405E-09	7.96027E-08	CPNE2	protein_coding
ENSG00000140937.14	0.341526367	1.06823E-11	1.08914E-09	0.31147163	7.02506E-10	4.30729E-08	CDH11	protein_coding
ENSG00000140939.14	0.329423945	6.09252E-11	4.7419E-09	0.309493003	1.11796E-09	6.28842E-08	NOL3	protein_coding
ENSG00000140941.14	0.336893184	2.09914E-11	1.90651E-09	0.313204233	6.92825E-10	4.25474E-08	MAP1LC3B	protein_coding
ENSG00000140943.17	0.344066461	7.34083E-12	7.83462E-10	0.331350552	6.05937E-11	5.31272E-09	MBTPS1	protein_coding
ENSG00000140950.16	0.355028544	1.39807E-12	1.85405E-10	0.324533622	1.54299E-10	1.16966E-08	MEAK7	protein_coding
ENSG00000141012.13	0.331768425	4.37412E-11	3.59679E-09	0.335446581	3.41516E-11	3.24876E-09	GALNS	protein_coding
ENSG00000141279.17	0.30363901	1.94162E-09	9.18255E-08	0.302447508	2.26042E-09	1.11146E-07	NPEPPS	protein_coding
ENSG00000141367.12	0.336727045	2.15016E-11	1.94074E-09	0.33425805	4.03713E-11	3.73662E-09	CLTC	protein_coding
ENSG00000141526.17	0.52040677	2.06401E-27	3.00406E-23	0.511380589	0	0	SLC16A3	protein_coding
ENSG00000141551.14	0.321704726	1.77829E-10	1.18318E-08	0.310762317	9.49888E-10	5.48073E-08	CSNK1D	protein_coding
ENSG00000141696.13	0.261244298	2.87491E-07	6.61285E-06	0.379765389	3.13001E-14	9.5883E-12	P3H4	protein_coding
ENSG00000141756.19	0.35131949	2.46803E-12	3.04415E-10	0.379738537	3.1457E-14	9.5883E-12	FKBP10	protein_coding
ENSG00000142156.15	0.365725061	2.60287E-13	4.49655E-11	0.330665396	5.11386E-11	4.58734E-09	COL6A1	protein_coding
ENSG00000142186.17	0.37668405	4.35266E-14	9.82182E-12	0.381711913	2.16666E-14	6.93068E-12	SCYL1	protein_coding
ENSG00000142552.8	0.343978349	7.4374E-12	7.91573E-10	0.330122881	7.18301E-11	6.11375E-09	RCN3	protein_coding
ENSG00000142623.11	0.215659427	2.53175E-05	0.000308807	0.303307639	2.02561E-09	1.01837E-07	PADI1	protein_coding
ENSG00000142733.17	0.313841008	5.13469E-10	2.94224E-08	0.315475708	5.15263E-10	3.28201E-08	MAP3K6	protein_coding
ENSG00000143001.5	0.304376431	1.76665E-09	8.4651E-08	0.343477451	8.01042E-12	9.53405E-10	TMEM61	protein_coding
ENSG00000143079.15	0.347508877	4.3912E-12	4.98337E-10	0.333708746	3.3179E-11	3.16658E-09	CTTNBP2NL	protein_coding
ENSG00000143157.12	0.353108393	1.87806E-12	2.39249E-10	0.378001365	4.3306E-14	1.25432E-11	POGK	protein_coding
ENSG00000143195.13	0.316527552	3.58669E-10	2.18649E-08	0.369066737	1.51961E-13	3.53874E-11	ILDR2	protein_coding
ENSG00000143320.9	0.397668417	1.16824E-15	4.44527E-13	0.397172147	3.98832E-16	2.09182E-13	CRABP2	protein_coding
ENSG00000143322.21	0.326159097	9.61968E-11	7.02683E-09	0.31309946	5.66566E-10	3.5736E-08	ABL2	protein_coding
ENSG00000143341.12	0.336868339	2.10669E-11	1.91039E-09	0.330739561	6.59537E-11	5.72428E-09	HMCN1	protein_coding
ENSG00000143369.15	0.393974341	2.25075E-15	7.98987E-13	0.395470702	8.04116E-16	3.90117E-13	ECM1	protein_coding
ENSG00000143384.14	0.358538928	8.10846E-13	1.16846E-10	0.360789168	7.83666E-13	1.35381E-10	MCL1	protein_coding
ENSG00000143387.14	0.348462886	3.80411E-12	4.45609E-10	0.309820913	1.07196E-09	6.09447E-08	CTSK	protein_coding
ENSG00000143393.16	0.329922548	5.6793E-11	4.46205E-09	0.326929343	1.11401E-10	8.85999E-09	PI4KB	protein_coding
ENSG00000143398.20	0.28747497	1.44105E-08	5.0969E-07	0.305322562	1.89919E-09	9.64807E-08	PIP5K1A	protein_coding
ENSG00000143437.21	0.336423905	2.24638E-11	2.012E-09	0.338605985	2.18078E-11	2.2313E-09	ARNT	protein_coding
ENSG00000143457.11	0.337594925	1.89635E-11	1.76352E-09	0.329902833	7.40478E-11	6.2933E-09	GOLPH3L	protein_coding
ENSG00000143479.17	0.354786731	1.45117E-12	1.91574E-10	0.374889997	5.86038E-14	1.58688E-11	DYRK3	protein_coding
ENSG00000143507.18	0.332729181	3.81561E-11	3.21008E-09	0.344210718	9.70343E-12	1.13209E-09	DUSP10	protein_coding
ENSG00000143514.17	0.309437425	9.17423E-10	4.85992E-08	0.332554102	5.12465E-11	4.58996E-09	TP53BP2	protein_coding
ENSG00000143537.14	0.288383876	1.29169E-08	4.63053E-07	0.320367732	2.70015E-10	1.88035E-08	ADAM15	protein_coding
ENSG00000143569.19	0.337056819	2.05004E-11	1.86775E-09	0.356751621	1.47384E-12	2.31902E-10	UBAP2L	protein_coding
ENSG00000143612.21	0.3152724	4.24313E-10	2.49522E-08	0.307506201	1.44048E-09	7.68669E-08	C1orf43	protein_coding
ENSG00000143614.10	0.295681928	5.29006E-09	2.17805E-07	0.321658664	2.27238E-10	1.62015E-08	GATAD2B	protein_coding
ENSG00000143622.11	0.352773898	1.97674E-12	2.49636E-10	0.349821368	4.2316E-12	5.61173E-10	RIT1	protein_coding
ENSG00000143756.12	0.321206338	1.90366E-10	1.2537E-08	0.333585846	4.4363E-11	4.06089E-09	FBXO28	protein_coding
ENSG00000143761.16	0.295910567	5.14206E-09	2.13068E-07	0.306344522	1.66916E-09	8.67638E-08	ARF1	protein_coding
ENSG00000143776.18	0.327344728	8.15451E-11	6.07861E-09	0.303112072	2.50681E-09	1.21416E-07	CDC42BPA	protein_coding
ENSG00000143786.8	0.308794406	9.97765E-10	5.20968E-08	0.339602649	1.41602E-11	1.56132E-09	CNIH3	protein_coding
ENSG00000143797.12	0.365573828	2.66665E-13	4.56608E-11	0.368513731	1.66187E-13	3.82413E-11	MBOAT2	protein_coding
ENSG00000143816.8	0.267410635	1.46313E-07	3.69227E-06	0.320231653	2.74956E-10	1.91247E-08	WNT9A	protein_coding
ENSG00000144233.10	0.36064119	5.83246E-13	8.66216E-11	0.357629992	1.28609E-12	2.08562E-10	AMMECR1L	protein_coding
ENSG00000144306.15	0.300687195	2.82611E-09	1.26562E-07	0.306765047	1.58258E-09	8.30689E-08	SCRN3	protein_coding
ENSG00000144366.16	0.374000083	6.7874E-14	1.4317E-11	0.362651724	5.82764E-13	1.07365E-10	GULP1	protein_coding
ENSG00000144369.13	0.361373876	5.19678E-13	7.87881E-11	0.322889749	1.92625E-10	1.41059E-08	FAM171B	protein_coding
ENSG00000144455.14	0.303946579	1.86669E-09	8.87142E-08	0.309698942	1.08885E-09	6.17842E-08	SUMF1	protein_coding
ENSG00000144476.6	0.38092162	2.14027E-14	5.41749E-12	0.343397656	1.09256E-11	1.25705E-09	ACKR3	protein_coding
ENSG00000144560.15	0.376317684	4.62591E-14	1.03184E-11	0.387303447	6.96637E-15	2.51906E-12	VGLL4	protein_coding
ENSG00000144566.11	0.413920024	5.91069E-17	3.66073E-14	0.427115989	4.63765E-18	3.17641E-15	RAB5A	protein_coding
ENSG00000144597.14	0.354730896	1.46371E-12	1.92792E-10	0.374518965	6.23075E-14	1.67162E-11	EAF1	protein_coding
ENSG00000144645.14	0.302013894	2.3886E-09	1.09935E-07	0.333923313	4.23133E-11	3.91015E-09	OSBPL10	protein_coding
ENSG00000144724.20	0.294543098	6.09091E-09	2.45909E-07	0.311546934	8.58561E-10	5.05397E-08	PTPRG	protein_coding
ENSG00000144730.19	0.244447621	1.66136E-06	2.98205E-05	0.306580857	1.33003E-09	7.25018E-08	IL17RD	protein_coding
ENSG00000144810.16	0.345957736	5.53959E-12	6.10803E-10	0.312794175	7.30674E-10	4.44174E-08	COL8A1	protein_coding

ENSG00000145040.4	0.342862103	8.77356E-12	9.13737E-10	0.326354271	9.362E-11	7.68741E-09	UCN2	protein_coding
ENSG00000145103.15	0.295821365	5.19932E-09	2.14981E-07	0.300072835	3.05415E-09	1.43508E-07	ILDR1	protein_coding
ENSG00000145113.22	0.363860747	3.50485E-13	5.66793E-11	0.383969962	1.39258E-14	4.65938E-12	MUC4	protein_coding
ENSG00000145214.14	0.332823464	3.76471E-11	3.18104E-09	0.327973831	9.65623E-11	7.88452E-09	DGKQ	protein_coding
ENSG00000145244.12	0.321160542	1.9156E-10	1.25872E-08	0.327231122	8.28491E-11	6.95074E-09	CORIN	protein_coding
ENSG00000145391.13	0.387739187	6.68349E-15	1.95527E-12	0.393813176	1.37979E-15	6.39602E-13	SETD7	protein_coding
ENSG00000145431.11	0.447660912	7.01594E-20	1.31759E-16	0.429923313	0	0	PDGFC	protein_coding
ENSG00000145506.14	0.24814235	1.14156E-06	2.17117E-05	0.335701123	2.49307E-11	2.49385E-09	NKD2	protein_coding
ENSG00000145536.15	0.333112659	3.61266E-11	3.06591E-09	0.352304779	2.12372E-12	3.14603E-10	ADAMTS16	protein_coding
ENSG00000145623.13	0.432065289	1.73403E-18	1.80271E-15	0.394338833	1.17425E-15	5.5368E-13	OSMR	protein_coding
ENSG00000145632.15	0.335370715	2.61446E-11	2.30619E-09	0.354871999	1.96878E-12	2.95584E-10	PLK2	protein_coding
ENSG00000145730.20	0.314093627	4.96511E-10	2.85849E-08	0.322892479	1.92554E-10	1.41059E-08	PAM	protein_coding
ENSG00000145794.17	0.179945967	0.000462421	0.003654297	0.305327984	1.56336E-09	8.22927E-08	MEGF10	protein_coding
ENSG00000145817.17	0.3198058	2.30376E-10	1.48857E-08	0.309254978	1.15254E-09	6.43322E-08	YIPF5	protein_coding
ENSG00000145860.12	0.285417657	1.84339E-08	6.25601E-07	0.311792695	8.31753E-10	4.93106E-08	RNF145	protein_coding
ENSG00000145911.6	0.341435954	1.08252E-11	1.10179E-09	0.353712843	1.7118E-12	2.63644E-10	N4BP3	protein_coding
ENSG00000146054.18	0.276621416	5.16636E-08	1.50992E-06	0.30438472	1.76477E-09	9.08414E-08	TRIM7	protein_coding
ENSG00000146072.6	0.348418215	3.8298E-12	4.47718E-10	0.330743429	6.59183E-11	5.72428E-09	TNFRSF21	protein_coding
ENSG00000146242.9	1	0	0	1	0	0	TPBG	protein_coding
ENSG00000146282.18	0.288510337	1.27214E-08	4.57623E-07	0.309362612	1.13677E-09	6.36352E-08	RARS2	protein_coding
ENSG00000146433.9	0.340209968	1.29573E-11	1.2764E-09	0.345507111	8.02447E-12	9.53405E-10	TMEM181	protein_coding
ENSG00000146648.19	0.330268654	5.40866E-11	4.2841E-09	0.336889749	2.78439E-11	2.7244E-09	EGFR	protein_coding
ENSG00000146674.15	0.36212419	4.61614E-13	7.10959E-11	0.334135757	3.1213E-11	3.00102E-09	IGFBP3	protein_coding
ENSG00000147036.12	0.295945487	5.11981E-09	2.126E-07	0.317622627	3.0955E-10	2.12516E-08	LANCL3	protein_coding
ENSG00000147144.13	0.323734983	1.34561E-10	9.31496E-09	0.317809307	3.79132E-10	2.52543E-08	CCDC120	protein_coding
ENSG00000147394.18	0.377804813	3.61122E-14	8.51166E-12	0.370527022	1.6002E-13	3.71158E-11	ZNF185	protein_coding
ENSG00000147650.11	0.347581261	4.34372E-12	4.94878E-10	0.336324041	3.01684E-11	2.91267E-09	LRP12	protein_coding
ENSG00000147689.16	0.408398069	1.65926E-16	8.25629E-14	0.419750201	1.94657E-17	1.27332E-14	FAM83A	protein_coding
ENSG00000147852.16	0.33983466	1.36883E-11	1.33262E-09	0.338227102	2.30198E-11	2.33072E-09	VLDLR	protein_coding
ENSG00000147883.12	0.371655123	9.97301E-14	1.95491E-11	0.365063147	3.95171E-13	7.98822E-11	CDKN2B	protein_coding
ENSG00000148339.12	0.306974097	1.26404E-09	6.36042E-08	0.317578792	3.90843E-10	2.58941E-08	SLC25A25	protein_coding
ENSG00000148841.17	0.373103992	7.86551E-14	1.62381E-11	0.340002731	1.78531E-11	1.90013E-09	ITPRIP	protein_coding
ENSG00000148848.14	0.321028517	1.95043E-10	1.28016E-08	0.303413375	1.99844E-09	1.00644E-07	ADAM12	protein_coding
ENSG00000148926.10	0.368030329	1.79684E-13	3.22865E-11	0.347786324	5.73122E-12	7.3011E-10	ADM	protein_coding
ENSG00000149084.13	0.363904421	3.48058E-13	5.66793E-11	0.336324269	3.01674E-11	2.91267E-09	HSD17B12	protein_coding
ENSG00000149115.14	0.392801895	2.76677E-15	9.53112E-13	0.372227557	1.19831E-13	2.91897E-11	TNKS1BP1	protein_coding
ENSG00000149218.5	0.37722141	3.98024E-14	9.17813E-12	0.359364226	7.12675E-13	1.27272E-10	ENDOD1	protein_coding
ENSG00000149256.16	0.338573556	1.64514E-11	1.57011E-09	0.31168872	6.82696E-10	4.20139E-08	TENM4	protein_coding
ENSG00000149257.16	0.43469718	1.02092E-18	1.23825E-15	0.445944021	0	0	SERPINH1	protein_coding
ENSG00000149380.12	0.389272701	5.12481E-15	1.54589E-12	0.3711105	1.09005E-13	2.68901E-11	P4HA3	protein_coding
ENSG00000149485.19	0.38929074	5.10878E-15	1.54589E-12	0.361097094	5.42853E-13	1.02278E-10	FADS1	protein_coding
ENSG00000150093.20	0.322165852	1.66948E-10	1.12233E-08	0.312304375	6.29421E-10	3.92906E-08	ITGB1	protein_coding
ENSG00000150403.18	0.370409972	1.22185E-13	2.32463E-11	0.365296166	3.80492E-13	7.82738E-11	TMCO3	protein_coding
ENSG00000150457.9	0.43141414	1.97546E-18	1.98288E-15	0.402778928	0	0	LATS2	protein_coding
ENSG00000150907.10	0.347823829	4.18822E-12	4.7998E-10	0.343087268	1.14305E-11	1.30826E-09	FOXO1	protein_coding
ENSG00000150961.15	0.413233948	6.72645E-17	3.95556E-14	0.398096484	2.38321E-16	1.3341E-13	SEC24D	protein_coding
ENSG00000150991.15	0.363330663	3.81291E-13	6.08165E-11	0.336423484	2.97465E-11	2.88631E-09	UBC	protein_coding
ENSG00000151240.17	0.350594189	2.75575E-12	3.35637E-10	0.35427343	1.57051E-12	2.45784E-10	DIP2C	protein_coding
ENSG00000151348.15	0.319840566	2.2929E-10	1.48444E-08	0.338885197	2.09546E-11	2.15918E-09	EXT2	protein_coding
ENSG00000151388.11	0.379540741	2.70036E-14	6.52322E-12	0.37693617	5.25024E-14	1.44179E-11	ADAMTS12	protein_coding
ENSG00000151414.15	0.349535502	3.23535E-12	3.86767E-10	0.359456593	9.66788E-13	1.64095E-10	NEK7	protein_coding
ENSG00000151458.12	0.404060369	3.68361E-16	1.64963E-13	0.389868197	4.62095E-15	1.81772E-12	ANKRD50	protein_coding
ENSG00000151651.16	0.334917576	2.79034E-11	2.44283E-09	0.309008761	1.1894E-09	6.60102E-08	ADAM8	protein_coding
ENSG00000151690.15	0.359359149	7.13242E-13	1.0433E-10	0.367077711	2.84321E-13	6.06323E-11	MFSD6	protein_coding
ENSG00000151692.15	0.423487141	9.44325E-18	7.13984E-15	0.403946433	3.761E-16	1.99052E-13	RNF144A	protein_coding
ENSG00000151693.11	0.358562826	8.07826E-13	1.16846E-10	0.32358357	1.75436E-10	1.30775E-08	ASAP2	protein_coding
ENSG00000151694.14	0.374255751	6.5073E-14	1.37761E-11	0.35484735	1.43767E-12	2.28685E-10	ADAM17	protein_coding
ENSG00000151718.16	0.34797771	4.09241E-12	4.71785E-10	0.328336026	7.09837E-11	6.06833E-09	WWC2	protein_coding
ENSG00000151748.15	0.363779419	3.55048E-13	5.71629E-11	0.365961315	3.41413E-13	7.14979E-11	SAV1	protein_coding
ENSG00000151914.20	0.410782762	1.06498E-16	5.63643E-14	0.362508818	5.96229E-13	1.08813E-10	DST	protein_coding
ENSG00000151929.10	0.375814819	5.0285E-14	1.10471E-11	0.410554784	0	0	BAG3	protein_coding
ENSG00000152684.11	0.326690888	8.93329E-11	6.59998E-09	0.349317784	4.56263E-12	5.95577E-10	PELO	protein_coding
ENSG00000152749.8	0.340042196	1.32793E-11	1.3037E-09	0.350590417	2.75733E-12	3.92484E-10	GPR180	protein_coding
ENSG00000152767.17	0.323945277	1.30715E-10	9.09197E-09	0.347319376	6.142E-12	7.67329E-10	FARP1	protein_coding
ENSG00000152894.15	0.314164774	4.91833E-10	2.83782E-08	0.306813517	1.57289E-09	8.26446E-08	PTPRK	protein_coding
ENSG00000152952.12	0.440114576	3.38125E-19	5.04742E-16	0.408830129	0	0	PLOD2	protein_coding
ENSG00000153046.18	0.40548133	2.84032E-16	1.31236E-13	0.415844806	0	0	CDYL	protein_coding

ENSG00000153234.15	0.293465919	6.95574E-09	2.73284E-07	0.318443868	2.77072E-10	1.92259E-08	NR4A2	protein_coding
ENSG00000153246.13	0.347542659	4.36898E-12	4.96784E-10	0.350498771	2.79596E-12	3.94129E-10	PLA2R1	protein_coding
ENSG00000153395.10	0.277892156	4.46158E-08	1.33613E-06	0.307619069	1.41996E-09	7.59807E-08	LPCAT1	protein_coding
ENSG00000153558.16	0.357267402	9.88491E-13	1.37346E-10	0.335668013	2.50499E-11	2.50146E-09	FBXL2	protein_coding
ENSG00000153827.14	0.32741429	8.07566E-11	6.02755E-09	0.318472864	3.47291E-10	2.34011E-08	TRIP12	protein_coding
ENSG00000153885.14	0.346826562	4.86445E-12	5.46715E-10	0.322188645	2.11653E-10	1.525E-08	KCTD15	protein_coding
ENSG00000153976.3	0.309221166	9.43716E-10	4.96756E-08	0.305771795	1.47649E-09	7.85008E-08	HS3ST3A1	protein_coding
ENSG00000153989.8	0.291983551	8.34261E-09	3.1769E-07	0.309042361	9.66006E-10	5.56821E-08	NUS1	protein_coding
ENSG00000154319.16	0.311903727	6.63611E-10	3.67944E-08	0.339486631	1.92256E-11	2.01671E-09	FAM167A	protein_coding
ENSG00000154845.16	0.341278282	1.10789E-11	1.11784E-09	0.336954053	2.08074E-11	2.15545E-09	PPP4R1	protein_coding
ENSG00000155011.9	0.325437083	1.06343E-10	7.70036E-09	0.31302266	5.72361E-10	3.59846E-08	DKK2	protein_coding
ENSG00000155254.13	0.399107144	9.02917E-16	3.60041E-13	0.375525771	6.7507E-14	1.78642E-11	MARVELD1	protein_coding
ENSG00000155886.11	0.283773772	2.24103E-08	7.37107E-07	0.336468753	2.23189E-11	2.27161E-09	SLC24A2	protein_coding
ENSG00000155918.8	0.251762037	7.85839E-07	1.57108E-05	0.322646022	1.56306E-10	1.18334E-08	RAET1L	protein_coding
ENSG00000155974.12	0.333279349	3.52775E-11	3.00744E-09	0.340907917	1.16979E-11	1.32754E-09	GRIP1	protein_coding
ENSG00000156011.17	0.41881008	2.33179E-17	1.6161E-14	0.415387416	0	0	PSD3	protein_coding
ENSG00000156515.24	0.386810892	7.84373E-15	2.24949E-12	0.389895551	3.87451E-15	1.54497E-12	HK1	protein_coding
ENSG00000156535.15	0.472078704	3.28186E-22	1.36474E-18	0.433288884	0	0	CD109	protein_coding
ENSG00000156599.11	0.330120865	5.52265E-11	4.36252E-09	0.327409034	1.04329E-10	8.35468E-09	ZDHHC5	protein_coding
ENSG00000156639.12	0.348985546	3.51573E-12	4.1517E-10	0.317152577	4.13431E-10	2.71048E-08	ZFAND3	protein_coding
ENSG00000156642.17	0.354705346	1.46948E-12	1.93116E-10	0.342276254	1.28593E-11	1.44526E-09	NPTN	protein_coding
ENSG00000156966.7	0.309002436	9.71053E-10	5.08846E-08	0.304135852	2.20501E-09	1.09098E-07	B3GNT7	protein_coding
ENSG00000156983.16	0.292637225	7.70086E-09	2.97103E-07	0.308616907	1.25045E-09	6.88728E-08	BRPF1	protein_coding
ENSG00000157020.18	0.299177165	3.41885E-09	1.49766E-07	0.304689174	1.69716E-09	8.7905E-08	SEC13	protein_coding
ENSG00000157064.11	0.34829066	3.9041E-12	4.53922E-10	0.363042139	3.99156E-13	8.01313E-11	NMNAT2	protein_coding
ENSG00000157227.13	0.450708469	3.67627E-20	7.92685E-17	0.407185573	0	0	MMP14	protein_coding
ENSG00000157240.4	0.40207147	5.28935E-16	2.23142E-13	0.377395381	4.83344E-14	1.35939E-11	FZD1	protein_coding
ENSG00000157483.9	0.355545523	1.29083E-12	1.73555E-10	0.360937991	7.65427E-13	1.33485E-10	MYO1E	protein_coding
ENSG00000157593.19	0.379321554	2.80157E-14	6.7384E-12	0.38835021	5.53138E-15	2.09108E-12	SLC35B2	protein_coding
ENSG00000157637.13	0.321684112	1.78331E-10	1.18517E-08	0.309464103	1.1221E-09	6.29956E-08	SLC38A10	protein_coding
ENSG00000157833.13	0.348829323	3.59961E-12	4.23358E-10	0.327473015	8.00967E-11	6.76788E-09	GAREM2	protein_coding
ENSG00000157954.15	0.295599162	5.34465E-09	2.19587E-07	0.301536466	3.05106E-09	1.43479E-07	WIPI2	protein_coding
ENSG00000157978.12	0.305350435	1.55885E-09	7.62631E-08	0.323164087	1.45566E-10	1.11949E-08	LDLRAP1	protein_coding
ENSG00000158186.13	0.337499119	1.92287E-11	1.77691E-09	0.303985891	2.2469E-09	1.10668E-07	MRAS	protein_coding
ENSG00000158292.7	0.358172271	8.58592E-13	1.22214E-10	0.322216634	2.1086E-10	1.52173E-08	GPR153	protein_coding
ENSG00000158615.9	0.288786601	1.23041E-08	4.4492E-07	0.302007737	2.87727E-09	1.36631E-07	PPP1R15B	protein_coding
ENSG00000158769.18	0.292860128	7.49317E-09	2.90631E-07	0.313093412	7.02862E-10	4.30729E-08	F11R	protein_coding
ENSG00000158796.17	0.31939347	2.43639E-10	1.56558E-08	0.355510525	1.78489E-12	2.72871E-10	DEDD	protein_coding
ENSG00000158850.15	0.272154904	8.60034E-08	2.33642E-06	0.305817272	1.78424E-09	9.16003E-08	B4GALT3	protein_coding
ENSG00000159216.19	0.335886476	2.4274E-11	2.15425E-09	0.372147912	1.21477E-13	2.9345E-11	RUNX1	protein_coding
ENSG00000159346.13	0.349298937	3.35317E-12	3.99213E-10	0.380790534	2.58227E-14	8.1262E-12	ADIPOR1	protein_coding
ENSG00000159399.10	0.337804782	1.83951E-11	1.72452E-09	0.365405641	2.73937E-13	5.86326E-11	HK2	protein_coding
ENSG00000159640.17	0.329548687	5.98648E-11	4.67813E-09	0.318917283	3.27436E-10	2.22695E-08	ACE	protein_coding
ENSG00000159674.12	0.434933566	9.73262E-19	1.23177E-15	0.399548527	5.09051E-17	3.08708E-14	SPON2	protein_coding
ENSG00000159921.19	0.363398184	3.77225E-13	6.03332E-11	0.315703265	5.00134E-10	3.20317E-08	GNE	protein_coding
ENSG00000160161.9	0.306332657	1.37339E-09	6.84557E-08	0.34732833	4.51186E-12	5.90273E-10	CILP2	protein_coding
ENSG00000160360.13	0.353654333	1.72724E-12	2.23459E-10	0.326993343	8.56444E-11	7.13311E-09	GPSM1	protein_coding
ENSG00000160471.13	0.314685387	4.58889E-10	2.67423E-08	0.39464453	1.9995E-15	8.75242E-13	COX6B2	protein_coding
ENSG00000160551.12	0.338238666	1.72721E-11	1.63504E-09	0.313560587	6.61486E-10	4.09661E-08	TAOK1	protein_coding
ENSG00000160570.14	0.334049767	3.15995E-11	2.71737E-09	0.338091023	2.34708E-11	2.35997E-09	DEDD2	protein_coding
ENSG00000160685.13	0.313306435	5.51232E-10	3.13475E-08	0.328030493	9.58148E-11	7.83448E-09	ZBTB7B	protein_coding
ENSG00000160691.19	0.455832757	1.22198E-20	3.23369E-17	0.435623848	0	0	SHC1	protein_coding
ENSG00000160710.17	0.326236001	9.51734E-11	6.96956E-09	0.326968256	1.1081E-10	8.82508E-09	ADAR	protein_coding
ENSG00000160714.10	0.315554929	4.08588E-10	2.42232E-08	0.315516669	5.12507E-10	3.26803E-08	UBE2Q1	protein_coding
ENSG00000160741.17	0.367160219	2.06732E-13	3.66936E-11	0.378397087	4.02902E-14	1.18037E-11	CRTC2	protein_coding
ENSG00000160752.15	0.300327714	2.95746E-09	1.31533E-07	0.310962016	7.51231E-10	4.53685E-08	FDPS	protein_coding
ENSG00000160753.16	0.332402379	3.99731E-11	3.33442E-09	0.346719763	6.71159E-12	8.27829E-10	RUSC1	protein_coding
ENSG00000160789.22	0.364049296	3.40125E-13	5.56219E-11	0.357007623	1.41654E-12	2.2594E-10	LMNA	protein_coding
ENSG00000161647.19	0.328809529	6.64214E-11	5.07784E-09	0.346580859	5.04677E-12	6.50187E-10	MPP3	protein_coding
ENSG00000161671.17	0.396444667	1.45302E-15	5.35392E-13	0.332420753	5.22087E-11	4.66895E-09	EMC10	protein_coding
ENSG00000161682.15	0.28029549	3.37393E-08	1.05491E-06	0.322476255	1.59991E-10	1.20652E-08	FAM171A2	protein_coding
ENSG00000161714.12	0.327170726	8.35505E-11	6.22014E-09	0.333358744	4.57963E-11	4.16589E-09	PLCD3	protein_coding
ENSG00000162006.9	0.269909817	1.10732E-07	2.89995E-06	0.303227533	2.04644E-09	1.02618E-07	MSLNL	protein_coding
ENSG00000162236.13	0.384519058	1.16203E-14	3.1761E-12	0.365139151	3.90324E-13	7.91774E-11	STX5	protein_coding
ENSG00000162337.12	0.304393288	1.76284E-09	8.46193E-08	0.307968825	1.35815E-09	7.38268E-08	LRP5	protein_coding
ENSG00000162490.7	0.246979949	1.28543E-06	2.39626E-05	0.312876021	5.83585E-10	3.65718E-08	DRAXIN	protein_coding
ENSG00000162493.16	0.357201063	9.98736E-13	1.38439E-10	0.331234498	6.15781E-11	5.3667E-09	PDPN	protein_coding

ENSG00000162576.17	0.372573501	8.58095E-14	1.7346E-11	0.333300717	4.61698E-11	4.19331E-09	MXRA8	protein_coding
ENSG00000162591.16	0.36147894	5.11137E-13	7.76955E-11	0.35671455	1.07711E-12	1.79677E-10	MEGF6	protein_coding
ENSG00000162702.8	0.295916842	5.13805E-09	2.13054E-07	0.311260212	8.90899E-10	5.2127E-08	ZNF281	protein_coding
ENSG00000162736.17	0.358258079	8.47177E-13	1.21182E-10	0.368075911	1.78367E-13	4.08825E-11	NCSTN	protein_coding
ENSG00000162783.11	0.331606313	4.47591E-11	3.65467E-09	0.336473774	2.95354E-11	2.8754E-09	IER5	protein_coding
ENSG00000162804.14	0.377495479	3.8025E-14	8.87266E-12	0.346412789	7.02271E-12	8.55331E-10	SNED1	protein_coding
ENSG00000162849.16	0.330619891	5.14686E-11	4.10179E-09	0.358723994	7.87743E-13	1.35683E-10	KIF26B	protein_coding
ENSG00000162889.11	0.307485736	1.18293E-09	6.00938E-08	0.312732051	7.36581E-10	4.4669E-08	MAPKAPK2	protein_coding
ENSG00000162923.17	0.385539818	9.75789E-15	2.71316E-12	0.372112868	1.22208E-13	2.93995E-11	WDR26	protein_coding
ENSG00000163040.15	0.399293452	8.73211E-16	3.53032E-13	0.356928133	1.04199E-12	1.76345E-10	CCDC74A	protein_coding
ENSG00000163110.15	0.313493913	5.37691E-10	3.06594E-08	0.32568233	1.32034E-10	1.02764E-08	PDLIM5	protein_coding
ENSG00000163283.7	0.261303842	2.85645E-07	6.5808E-06	0.376880973	4.21238E-14	1.22618E-11	ALPP	protein_coding
ENSG00000163297.17	0.37757737	3.75092E-14	8.80528E-12	0.355507339	1.78577E-12	2.72871E-10	ANTXR2	protein_coding
ENSG00000163349.22	0.309918826	8.61424E-10	4.6179E-08	0.307048356	1.52672E-09	8.0662E-08	HIPK1	protein_coding
ENSG00000163359.16	0.379725853	2.61768E-14	6.43021E-12	0.351939242	3.0769E-12	4.23477E-10	COL6A3	protein_coding
ENSG00000163449.11	0.274135628	6.86837E-08	1.91873E-06	0.306394513	1.36246E-09	7.39234E-08	TMEM169	protein_coding
ENSG00000163512.14	0.386541246	8.21627E-15	2.32201E-12	0.365340767	3.77743E-13	7.79838E-11	AZI2	protein_coding
ENSG00000163520.14	0.356718731	1.07641E-12	1.47798E-10	0.307040619	1.52822E-09	8.0662E-08	FBLN2	protein_coding
ENSG00000163623.9	0.29406155	6.46382E-09	2.5757E-07	0.306855864	1.28354E-09	7.02966E-08	NKX6-1	protein_coding
ENSG00000163625.16	0.401912959	5.44352E-16	2.27879E-13	0.398026852	2.49182E-16	1.38161E-13	WDFY3	protein_coding
ENSG00000163659.13	0.352536868	2.04972E-12	2.58291E-10	0.344822866	6.5606E-12	8.16122E-10	TIPARP	protein_coding
ENSG00000163746.12	0.303463055	1.98579E-09	9.33834E-08	0.315191692	4.28912E-10	2.78999E-08	PLSCR2	protein_coding
ENSG00000163755.9	0.308606583	1.02249E-09	5.30549E-08	0.303873933	2.27868E-09	1.11855E-07	HPS3	protein_coding
ENSG00000163877.11	0.340017243	1.33278E-11	1.30599E-09	0.314600296	5.77718E-10	3.62822E-08	SNIP1	protein_coding
ENSG00000163902.12	0.312824266	5.87598E-10	3.29564E-08	0.311754466	8.35869E-10	4.95042E-08	RPN1	protein_coding
ENSG00000164023.14	0.337714037	1.86388E-11	1.74456E-09	0.347155763	6.29259E-12	7.84458E-10	SGMS2	protein_coding
ENSG00000164086.10	0.385522603	9.78673E-15	2.71316E-12	0.382587325	1.82935E-14	5.98322E-12	DUSP7	protein_coding
ENSG00000164099.3	0.391323339	3.58524E-15	1.15937E-12	0.395461281	1.73029E-15	7.74877E-13	PRSS12	protein_coding
ENSG00000164111.15	0.44327791	1.75729E-19	2.92303E-16	0.407247924	0	0	ANXA5	protein_coding
ENSG00000164164.17	0.315893905	3.90468E-10	2.33391E-08	0.31866606	3.3852E-10	2.28896E-08	OTUD4	protein_coding
ENSG00000164168.8	0.295124281	5.66856E-09	2.31425E-07	0.305982706	1.74733E-09	9.01026E-08	TMEM184C	protein_coding
ENSG00000164171.11	0.319756131	2.31936E-10	1.49533E-08	0.312652179	7.44243E-10	4.50399E-08	ITGA2	protein_coding
ENSG00000164185.6	0.228311578	7.98478E-06	0.000114412	0.309961408	8.56633E-10	5.04772E-08	ZNF474	protein_coding
ENSG00000164244.21	0.305590215	1.51145E-09	7.43818E-08	0.312525657	7.56539E-10	4.55473E-08	PRRC1	protein_coding
ENSG00000164294.14	0.492510336	2.61573E-24	1.17141E-20	0.470618641	4.57889E-22	3.75456E-19	GPX8	protein_coding
ENSG00000164465.19	0.469210652	6.30355E-22	2.15871E-18	0.460550399	4.36005E-21	3.52546E-18	DCBLD1	protein_coding
ENSG00000164520.11	0.220463419	1.64644E-05	0.000213814	0.315530836	4.09907E-10	2.69345E-08	RAET1E	protein_coding
ENSG00000164692.18	0.399793948	7.98078E-16	3.27099E-13	0.377265673	4.94792E-14	1.38489E-11	COL1A2	protein_coding
ENSG00000164694.17	0.362522883	4.33393E-13	6.72834E-11	0.346152463	7.29749E-12	8.82668E-10	FNDC1	protein_coding
ENSG00000164828.18	0.306279922	1.38278E-09	6.88059E-08	0.311848674	8.25761E-10	4.90553E-08	SUN1	protein_coding
ENSG00000164930.12	0.370644194	1.17614E-13	2.25238E-11	0.306240528	1.69128E-09	8.77468E-08	FZD6	protein_coding
ENSG00000164932.13	0.421826684	1.30374E-17	9.48767E-15	0.390882239	3.04996E-15	1.2683E-12	CTHRC1	protein_coding
ENSG00000164970.15	0.328098357	7.33874E-11	5.54866E-09	0.321241343	1.89458E-10	1.39618E-08	FAM219A	protein_coding
ENSG00000164983.7	0.293512131	6.91631E-09	2.72504E-07	0.303039988	2.09602E-09	1.04833E-07	TMEM65	protein_coding
ENSG00000165006.14	0.365578354	2.66472E-13	4.56608E-11	0.339956764	1.79714E-11	1.90575E-09	UBAP1	protein_coding
ENSG00000165030.4	0.317394518	3.1921E-10	1.98332E-08	0.302848333	2.59084E-09	1.24827E-07	NFIL3	protein_coding
ENSG00000165102.15	0.309013721	9.69624E-10	5.08554E-08	0.31291478	7.19339E-10	4.38977E-08	HGSNAT	protein_coding
ENSG00000165233.18	0.273591619	7.30725E-08	2.02674E-06	0.304347935	2.14705E-09	1.06835E-07	CARD19	protein_coding
ENSG00000165458.14	0.28723282	1.48358E-08	5.21564E-07	0.306730003	1.58963E-09	8.32987E-08	INPPL1	protein_coding
ENSG00000165861.14	0.364685329	3.07341E-13	5.1416E-11	0.348640118	5.048E-12	6.50187E-10	ZFYVE1	protein_coding
ENSG00000165886.5	0.354927594	1.42E-12	1.87885E-10	0.323796109	1.70476E-10	1.27732E-08	UBTD1	protein_coding
ENSG00000165895.19	0.312336986	6.26714E-10	3.49818E-08	0.301667539	3.00173E-09	1.41962E-07	ARHGAP42	protein_coding
ENSG00000165915.14	0.397208261	1.26824E-15	4.79445E-13	0.371153487	1.43917E-13	3.36488E-11	SLC39A13	protein_coding
ENSG00000166147.14	0.374573782	6.17462E-14	1.31428E-11	0.336857663	2.79709E-11	2.73223E-09	FBN1	protein_coding
ENSG00000166170.10	0.278453703	4.18059E-08	1.26237E-06	0.302390943	2.74307E-09	1.31114E-07	BAG5	protein_coding
ENSG00000166173.11	0.35291737	1.93381E-12	2.44745E-10	0.321171938	1.91262E-10	1.40592E-08	LARP6	protein_coding
ENSG00000166394.15	0.410999848	1.02266E-16	5.51272E-14	0.408410741	0	0	CYB5R2	protein_coding
ENSG00000166415.15	0.30508534	1.61293E-09	7.85787E-08	0.308504722	1.26848E-09	6.97339E-08	WDR72	protein_coding
ENSG00000166446.15	0.355396734	1.32084E-12	1.76774E-10	0.309611709	8.96752E-10	5.2296E-08	CDYL2	protein_coding
ENSG00000166484.20	0.314484191	4.71358E-10	2.73868E-08	0.330992623	4.88254E-11	4.40718E-09	MAPK7	protein_coding
ENSG00000166546.14	0.250441526	9.0112E-07	1.76136E-05	0.344543697	6.83865E-12	8.39942E-10	BEAN1	protein_coding
ENSG00000166557.14	0.287382739	1.45711E-08	5.13498E-07	0.315787689	4.94632E-10	3.17492E-08	TMED3	protein_coding
ENSG00000166579.16	0.432096059	1.72337E-18	1.80271E-15	0.422729548	0	0	NDEL1	protein_coding
ENSG00000166734.20	0.302664331	2.19886E-09	1.02247E-07	0.330612129	6.71283E-11	5.79833E-09	GOLM2	protein_coding
ENSG00000166741.8	0.351996896	2.22594E-12	2.77494E-10	0.31009785	1.03455E-09	5.92226E-08	NNMT	protein_coding
ENSG00000166813.16	0.322011428	1.70518E-10	1.14237E-08	0.323686122	1.3547E-10	1.05298E-08	KIF7	protein_coding
ENSG00000166908.18	0.341034693	1.14823E-11	1.15113E-09	0.343082717	1.14381E-11	1.30826E-09	PIP4K2C	protein_coding

ENSG00000166949.16	0.346476736	5.12603E-12	5.717E-10	0.337408579	2.58658E-11	2.55663E-09	SMAD3	protein_coding
ENSG00000167034.10	0.301393427	2.58435E-09	1.17379E-07	0.304639663	2.06974E-09	1.03697E-07	NKX3-1	protein_coding
ENSG00000167110.18	0.345415487	6.00645E-12	6.53614E-10	0.33455615	3.8715E-11	3.61204E-09	GOLGA2	protein_coding
ENSG00000167123.19	0.390237333	4.33336E-15	1.36104E-12	0.340165889	1.74394E-11	1.86329E-09	CERCAM	protein_coding
ENSG00000167157.11	0.331701978	4.41557E-11	3.62575E-09	0.327713733	1.00067E-10	8.12508E-09	PRRX2	protein_coding
ENSG00000167173.19	0.372057508	9.3378E-14	1.85539E-11	0.359524426	6.95014E-13	1.24499E-10	C15orf39	protein_coding
ENSG00000167193.8	0.334745394	2.86016E-11	2.49644E-09	0.359988395	8.89235E-13	1.51817E-10	CRK	protein_coding
ENSG00000167460.17	0.35658201	1.09948E-12	1.50611E-10	0.368394584	2.28699E-13	4.96805E-11	TPM4	protein_coding
ENSG00000167470.12	0.317276084	3.2434E-10	2.00877E-08	0.329624303	7.69505E-11	6.52097E-09	MIDN	protein_coding
ENSG00000167535.8	0.433178039	1.38686E-18	1.55535E-15	0.444639372	1.32312E-19	1.01354E-16	CACNB3	protein_coding
ENSG00000167543.16	0.35034642	2.86135E-12	3.47046E-10	0.345551257	7.97257E-12	9.51121E-10	TP53I13	protein_coding
ENSG00000167566.17	0.372837313	8.21754E-14	1.67863E-11	0.349468882	4.46076E-12	5.86222E-10	NCKAP5L	protein_coding
ENSG00000167693.17	0.339108759	1.5218E-11	1.4644E-09	0.3026474	2.65668E-09	1.27402E-07	NXN	protein_coding
ENSG00000167757.14	0.346637188	5.00439E-12	5.59205E-10	0.352685422	2.00368E-12	2.99872E-10	KLK11	protein_coding
ENSG00000167767.14	0.335192752	2.68221E-11	2.3588E-09	0.331768574	5.71733E-11	5.05358E-09	KRT80	protein_coding
ENSG00000167771.6	0.319002882	2.56886E-10	1.63559E-08	0.322615542	1.9986E-10	1.45625E-08	RCOR2	protein_coding
ENSG00000167772.12	0.322416107	1.61317E-10	1.08951E-08	0.324794553	1.16243E-10	9.18244E-09	ANGPTL4	protein_coding
ENSG00000167881.15	0.299393917	3.3269E-09	1.45958E-07	0.309179884	1.16366E-09	6.48288E-08	SRP68	protein_coding
ENSG00000167986.14	0.303145156	2.06808E-09	9.66286E-08	0.319145068	3.17691E-10	2.16735E-08	DDB1	protein_coding
ENSG00000167987.11	0.303530614	1.96872E-09	9.29117E-08	0.312256002	7.83408E-10	4.68741E-08	VPS37C	protein_coding
ENSG00000167994.13	0.327660432	7.80256E-11	5.84619E-09	0.335004665	3.63469E-11	3.42957E-09	RAB3IL1	protein_coding
ENSG00000168032.10	0.258801864	3.73911E-07	8.34356E-06	0.301536825	2.53777E-09	1.22711E-07	ENTPD3	protein_coding
ENSG00000168092.14	0.318238933	2.84852E-10	1.79864E-08	0.332368415	5.25911E-11	4.69594E-09	PAFAH1B2	protein_coding
ENSG00000168116.14	0.38659486	8.14084E-15	2.31192E-12	0.362641391	4.25335E-13	8.4304E-11	KIAA1586	protein_coding
ENSG00000168140.5	0.441532115	2.52392E-19	3.86677E-16	0.388233019	5.67825E-15	2.13275E-12	VASN	protein_coding
ENSG00000168209.5	0.287081966	1.51068E-08	5.29813E-07	0.308819433	1.21853E-09	6.73696E-08	DDIT4	protein_coding
ENSG00000168246.6	0.315533113	4.09782E-10	2.42693E-08	0.328412334	9.09226E-11	7.50828E-09	UBTD2	protein_coding
ENSG00000168256.18	0.364304201	3.26595E-13	5.40162E-11	0.392170895	2.19111E-15	9.51954E-13	NKIRAS2	protein_coding
ENSG00000168374.11	0.373842442	6.96596E-14	1.46406E-11	0.376078052	6.12072E-14	1.6497E-11	ARF4	protein_coding
ENSG00000168427.9	0.32131692	1.87512E-10	1.23771E-08	0.325678459	1.0284E-10	8.27594E-09	KLHL30	protein_coding
ENSG00000168487.20	0.424989109	7.04291E-18	5.56584E-15	0.419237683	0	0	BMP1	protein_coding
ENSG00000168542.16	0.388805877	5.5571E-15	1.65063E-12	0.348883832	4.86794E-12	6.31184E-10	COL3A1	protein_coding
ENSG00000168575.10	0.324665325	1.1834E-10	8.40187E-09	0.308709296	1.23579E-09	6.81944E-08	SLC20A2	protein_coding
ENSG00000168591.16	0.383040478	1.49497E-14	3.92046E-12	0.377130276	5.07012E-14	1.40962E-11	TMUB2	protein_coding
ENSG00000168610.16	0.286657429	1.58956E-08	5.52814E-07	0.305789054	1.79061E-09	9.17655E-08	STAT3	protein_coding
ENSG00000168758.11	0.346748311	4.9218E-12	5.51034E-10	0.335866196	3.21868E-11	3.08199E-09	SEMA4C	protein_coding
ENSG00000168779.20	0.321370067	1.86155E-10	1.2321E-08	0.339503794	1.4366E-11	1.57941E-09	SHOX2	protein_coding
ENSG00000168795.5	0.363772495	3.55439E-13	5.71629E-11	0.374035975	6.74738E-14	1.78642E-11	ZBTB5	protein_coding
ENSG00000168938.6	0.540671337	7.4827E-30	2.17814E-25	0.557614291	0	0	PPIC	protein_coding
ENSG00000169018.6	0.32334269	1.42032E-10	9.74382E-09	0.306310616	1.67634E-09	8.70591E-08	FEM1B	protein_coding
ENSG00000169032.10	0.281151441	3.05236E-08	9.63156E-07	0.317709409	3.84165E-10	2.5502E-08	MAP2K1	protein_coding
ENSG00000169047.5	0.367019473	2.11466E-13	3.72465E-11	0.345850722	7.62906E-12	9.13886E-10	IRS1	protein_coding
ENSG00000169067.4	0.185012447	0.000315859	0.002640916	0.321207903	1.90325E-10	1.4008E-08	ACTBL2	protein_coding
ENSG00000169083.18	0.237838276	3.20319E-06	5.2207E-05	0.302700376	2.18878E-09	1.08448E-07	AR	protein_coding
ENSG00000169105.8	0.306414791	1.3589E-09	6.78492E-08	0.303825236	2.29264E-09	1.12446E-07	CHST14	protein_coding
ENSG00000169129.15	0.428602636	3.45702E-18	3.19462E-15	0.416704972	0	0	AFAP1L2	protein_coding
ENSG00000169213.7	0.311586946	6.91915E-10	3.82181E-08	0.351999224	2.22515E-12	3.2713E-10	RAB3B	protein_coding
ENSG00000169231.13	0.323701844	1.35177E-10	9.34649E-09	0.318426346	2.77729E-10	1.92486E-08	THBS3	protein_coding
ENSG00000169242.12	0.36841957	1.68736E-13	3.06026E-11	0.366878826	2.93773E-13	6.21923E-11	EFNA1	protein_coding
ENSG00000169251.13	0.280277849	3.38089E-08	1.05651E-06	0.304165793	1.815E-09	9.28523E-08	NMD3	protein_coding
ENSG00000169255.15	0.427896432	3.97551E-18	3.61635E-15	0.412770987	7.33841E-17	4.35946E-14	B3GALNT1	protein_coding
ENSG00000169258.7	0.384883987	1.09176E-14	3.01232E-12	0.395319604	8.48325E-16	4.08163E-13	GPRIN1	protein_coding
ENSG00000169371.14	0.302029923	2.38374E-09	1.09879E-07	0.30931143	9.32654E-10	5.39198E-08	SNUPN	protein_coding
ENSG00000169379.16	0.399756672	8.03448E-16	3.27099E-13	0.397093185	4.14287E-16	2.13442E-13	ARL13B	protein_coding
ENSG00000169432.18	0.233808523	4.73613E-06	7.30986E-05	0.344610143	6.77145E-12	8.33446E-10	SCN9A	protein_coding
ENSG00000169604.20	0.455545518	1.30044E-20	3.2917E-17	0.419696666	0	0	ANTXR1	protein_coding
ENSG00000169826.8	0.354037141	1.62861E-12	2.1164E-10	0.301439754	3.08796E-09	1.44746E-07	CSGALNACT2	protein_coding
ENSG00000169902.15	0.422048037	1.24901E-17	9.20438E-15	0.401400614	0	0	TPST1	protein_coding
ENSG00000169905.13	0.389573666	4.86377E-15	1.49031E-12	0.404980089	0	0	TOR1AIP2	protein_coding
ENSG00000169908.12	0.310625428	7.85197E-10	4.26721E-08	0.318768688	3.33948E-10	2.26331E-08	TM4SF1	protein_coding
ENSG00000169946.14	0.285909804	1.73826E-08	5.95982E-07	0.311934307	6.60939E-10	4.09661E-08	ZFPM2	protein_coding
ENSG00000169967.16	0.354125197	1.60672E-12	2.09261E-10	0.307789965	1.38942E-09	7.50365E-08	MAP3K2	protein_coding
ENSG00000170017.12	0.361902742	4.78057E-13	7.34341E-11	0.343404028	1.09154E-11	1.25705E-09	ALCAM	protein_coding
ENSG00000170035.16	0.304543079	1.72928E-09	8.33406E-08	0.308485835	1.27154E-09	6.98362E-08	UBE2E3	protein_coding
ENSG00000170348.9	0.289560962	1.12039E-08	4.11266E-07	0.30402448	1.84816E-09	9.43826E-08	TMED10	protein_coding
ENSG00000170382.12	0.321013909	1.95432E-10	1.28127E-08	0.301520458	2.54305E-09	1.22864E-07	LRRN2	protein_coding
ENSG00000170775.3	0.317147883	3.29984E-10	2.03722E-08	0.348065616	4.03863E-12	5.39269E-10	GPR37	protein_coding

ENSG00000170776.22	0.39010598	4.43363E-15	1.3803E-12	0.350719308	3.69822E-12	4.96089E-10	AKAP13	protein_coding
ENSG00000170786.12	0.360641139	5.8325E-13	8.66216E-11	0.334526795	3.88752E-11	3.62117E-09	SDR16C5	protein_coding
ENSG00000170801.10	0.341362763	1.09423E-11	1.10907E-09	0.304133576	2.20564E-09	1.09098E-07	HTRA3	protein_coding
ENSG00000170832.13	0.310218051	8.28305E-10	4.46089E-08	0.312283081	7.80669E-10	4.6772E-08	USP32	protein_coding
ENSG00000170876.8	0.365052416	2.89848E-13	4.87699E-11	0.385481852	1.02418E-14	3.5919E-12	TMEM43	protein_coding
ENSG00000170921.15	0.508764536	4.41019E-26	4.27921E-22	0.511442712	0	0	TANC2	protein_coding
ENSG00000170955.10	0.369594158	1.39506E-13	2.59482E-11	0.300235294	3.58544E-09	1.6514E-07	CAVIN3	protein_coding
ENSG00000171017.11	0.310818827	7.65503E-10	4.1846E-08	0.314529818	4.68502E-10	3.02722E-08	LRRC8E	protein_coding
ENSG00000171067.11	0.369743344	1.36169E-13	2.55725E-11	0.403476619	0	0	C11orf24	protein_coding
ENSG00000171150.9	0.305517442	1.52569E-09	7.49556E-08	0.306000359	1.4336E-09	7.65701E-08	SOCS5	protein_coding
ENSG00000171502.15	0.29577613	5.22859E-09	2.16038E-07	0.319893594	2.27644E-10	1.62017E-08	COL24A1	protein_coding
ENSG00000171603.18	0.371074507	1.09647E-13	2.13493E-11	0.346754352	6.67738E-12	8.27829E-10	CLSTN1	protein_coding
ENSG00000172031.7	0.412181504	8.19723E-17	4.58872E-14	0.427750532	4.09179E-18	2.8359E-15	EPHX4	protein_coding
ENSG00000172037.14	0.363878947	3.49472E-13	5.66793E-11	0.338140175	2.33069E-11	2.34816E-09	LAMB2	protein_coding
ENSG00000172061.9	0.317106909	3.31807E-10	2.04414E-08	0.30447787	2.11228E-09	1.05465E-07	LRRC15	protein_coding
ENSG00000172380.6	0.32202863	1.70116E-10	1.14099E-08	0.300758448	3.36049E-09	1.56013E-07	GNG12	protein_coding
ENSG00000172432.19	0.407807847	1.85071E-16	8.97874E-14	0.411583594	9.16903E-17	5.33802E-14	GTPBP2	protein_coding
ENSG00000172476.4	0.308144942	1.08583E-09	5.58437E-08	0.321563665	1.81293E-10	1.34042E-08	RAB40A	protein_coding
ENSG00000172765.17	0.362840483	4.12126E-13	6.48464E-11	0.338351348	2.26153E-11	2.29659E-09	TMCC1	protein_coding
ENSG00000172830.13	0.338950159	1.55738E-11	1.49616E-09	0.360839686	7.77428E-13	1.34703E-10	SSH3	protein_coding
ENSG00000172936.16	0.337557155	1.90676E-11	1.76764E-09	0.341915121	1.355E-11	1.50545E-09	MYD88	protein_coding
ENSG00000172939.9	0.316987125	3.37195E-10	2.07295E-08	0.325672905	1.0292E-10	8.27594E-09	OXSR1	protein_coding
ENSG00000172965.17	0.356792526	1.06416E-12	1.46517E-10	0.331682804	4.4276E-11	4.0593E-09	MIR4435-2HG	lncRNA
ENSG00000173039.19	0.344686655	6.69486E-12	7.17793E-10	0.34656639	6.86533E-12	8.41443E-10	RELA	protein_coding
ENSG00000173210.20	0.384102445	1.24768E-14	3.37848E-12	0.350979085	2.59921E-12	3.73632E-10	ABLIM3	protein_coding
ENSG00000173218.15	0.297960922	3.98233E-09	1.71102E-07	0.304214359	2.18338E-09	1.08273E-07	VANGL1	protein_coding
ENSG00000173376.14	0.357953302	8.88405E-13	1.25233E-10	0.349734213	4.28717E-12	5.67251E-10	NDNF	protein_coding
ENSG00000173548.9	0.442392105	2.1122E-19	3.32347E-16	0.425355103	0	0	SNX33	protein_coding
ENSG00000173599.15	0.332791272	3.78201E-11	3.18641E-09	0.305297986	1.90508E-09	9.66116E-08	PC	protein_coding
ENSG00000173846.13	0.382556388	1.62306E-14	4.21837E-12	0.375382637	6.92364E-14	1.80754E-11	PLK3	protein_coding
ENSG00000174238.15	0.323318013	1.42515E-10	9.76112E-09	0.30723427	1.49112E-09	7.90983E-08	PITPNA	protein_coding
ENSG00000174332.5	0.329784859	5.7906E-11	4.54336E-09	0.31521794	4.27411E-10	2.78334E-08	GLIS1	protein_coding
ENSG00000174695.10	0.293621847	6.82356E-09	2.69609E-07	0.311165093	9.01887E-10	5.24536E-08	TMEM167A	protein_coding
ENSG00000174705.13	0.391292713	3.60449E-15	1.15937E-12	0.374932074	7.49564E-14	1.93816E-11	SH3PXD2B	protein_coding
ENSG00000174807.4	0.375192771	5.57419E-14	1.20192E-11	0.346266697	7.17566E-12	8.70317E-10	CD248	protein_coding
ENSG00000175220.12	0.309268082	9.3795E-10	4.94168E-08	0.312487427	7.60293E-10	4.5726E-08	ARHGAP1	protein_coding
ENSG00000175315.3	0.299473827	3.29361E-09	1.44824E-07	0.306839496	1.28626E-09	7.03793E-08	CST6	protein_coding
ENSG00000175544.14	0.371606873	1.0052E-13	1.96377E-11	0.328589789	6.8502E-11	5.89948E-09	CABP4	protein_coding
ENSG00000175556.17	0.402563296	4.83776E-16	2.05925E-13	0.384534781	1.15891E-14	4.01604E-12	LONRF3	protein_coding
ENSG00000175591.12	0.360980335	5.52929E-13	8.289E-11	0.349133636	3.43798E-12	4.67645E-10	P2RY2	protein_coding
ENSG00000175592.9	0.340056136	1.32522E-11	1.30324E-09	0.355894641	1.6824E-12	2.59804E-10	FOSL1	protein_coding
ENSG00000175745.14	0.342499536	9.25595E-12	9.5883E-10	0.300702924	3.3837E-09	1.56841E-07	NR2F1	protein_coding
ENSG00000175866.15	0.443127227	1.81322E-19	2.93228E-16	0.425269086	0	0	BAIAP2	protein_coding
ENSG00000175874.10	0.293886647	6.60466E-09	2.62464E-07	0.3235051	1.38891E-10	1.07383E-08	CREG2	protein_coding
ENSG00000175906.5	0.289746394	1.09549E-08	4.02634E-07	0.305234953	1.92028E-09	9.7148E-08	ARL4D	protein_coding
ENSG00000176142.13	0.34590207	5.58584E-12	6.14738E-10	0.329850495	7.4585E-11	6.32972E-09	TMEM39A	protein_coding
ENSG00000176170.14	0.413363568	6.56429E-17	3.89959E-14	0.381113892	2.42875E-14	7.6846E-12	SPHK1	protein_coding
ENSG00000176641.11	0.368649438	1.6258E-13	2.96007E-11	0.345265673	8.31413E-12	9.83805E-10	RNF152	protein_coding
ENSG00000176658.17	0.326474708	9.20636E-11	6.78451E-09	0.318892934	3.28494E-10	2.23154E-08	MYO1D	protein_coding
ENSG00000176678.6	0.357905376	8.95064E-13	1.25867E-10	0.335561289	2.54376E-11	2.53093E-09	FOXL1	protein_coding
ENSG00000176697.20	0.375257925	5.51442E-14	1.19791E-11	0.384327993	1.20057E-14	4.13578E-12	BDNF	protein_coding
ENSG00000176845.13	0.282665063	2.55476E-08	8.26755E-07	0.305982706	1.74733E-09	9.01026E-08	METRNL	protein_coding
ENSG00000177096.9	0.332380869	4.00956E-11	3.33946E-09	0.322213904	2.10937E-10	1.52173E-08	PHETA2	protein_coding
ENSG00000177169.10	0.330585334	5.17206E-11	4.11348E-09	0.304002276	2.24229E-09	1.10535E-07	ULK1	protein_coding
ENSG00000177234.7	0.270439943	1.04339E-07	2.75526E-06	0.311613729	6.89477E-10	4.23864E-08	LINC01561	lncRNA
ENSG00000177628.16	0.36520603	2.8282E-13	4.8144E-11	0.339856184	1.82328E-11	1.92646E-09	GBA	protein_coding
ENSG00000177663.14	0.329386615	6.12461E-11	4.75181E-09	0.307547161	1.433E-09	7.65701E-08	IL17RA	protein_coding
ENSG00000177666.17	0.410410494	1.14157E-16	5.88142E-14	0.419286836	0	0	PNPLA2	protein_coding
ENSG00000177674.16	0.312039535	6.51824E-10	3.62098E-08	0.316474229	4.5202E-10	2.92722E-08	AGTRAP	protein_coding
ENSG00000177679.16	0.294163652	6.38295E-09	2.54871E-07	0.366592601	2.26483E-13	4.93835E-11	SRRM3	protein_coding
ENSG00000177706.9	0.374563025	6.1856E-14	1.31428E-11	0.34013039	1.75286E-11	1.86901E-09	FAM20C	protein_coding
ENSG00000177839.7	0.310356451	8.13408E-10	4.39285E-08	0.350437123	2.82224E-12	3.94965E-10	PCDHB9	protein_coding
ENSG00000177875.5	0.339715604	1.39285E-11	1.35336E-09	0.325399172	1.06904E-10	8.53767E-09	CCDC184	protein_coding
ENSG00000177951.17	0.301212392	2.64434E-09	1.19432E-07	0.334502446	3.90085E-11	3.622E-09	BET1L	protein_coding
ENSG00000178038.17	0.408442911	1.64553E-16	8.25629E-14	0.384073501	1.36401E-14	4.60602E-12	ALS2CL	protein_coding
ENSG00000178104.19	0.337102775	2.03645E-11	1.86269E-09	0.314077729	4.97562E-10	3.1902E-08	PDE4DIP	protein_coding
ENSG00000178149.17	0.292342194	7.98436E-09	3.0541E-07	0.304667443	1.7019E-09	8.80722E-08	DALRD3	protein_coding

ENSG00000178184.16	0.288872002	1.21778E-08	4.4145E-07	0.304109227	2.21239E-09	1.09339E-07	PARD6G	protein_coding
ENSG00000178209.16	0.403443314	4.12226E-16	1.80443E-13	0.372704062	1.10418E-13	2.71238E-11	PLEC	protein_coding
ENSG00000178342.4	0.291446199	8.90866E-09	3.35692E-07	0.304941491	1.64303E-09	8.56348E-08	KCNG2	protein_coding
ENSG00000178752.16	0.348711699	3.66406E-12	4.30069E-10	0.393239002	2.56208E-15	1.09676E-12	ERFE	protein_coding
ENSG00000178764.8	0.370758451	1.15445E-13	2.22548E-11	0.350767323	3.67161E-12	4.9452E-10	ZHX2	protein_coding
ENSG00000179134.15	0.346388553	5.19411E-12	5.78185E-10	0.361703493	6.77887E-13	1.21806E-10	SAMD4B	protein_coding
ENSG00000179222.18	0.398111771	1.07923E-15	4.18869E-13	0.358818751	1.06844E-12	1.79257E-10	MAGED1	protein_coding
ENSG00000179241.13	0.324283769	1.24748E-10	8.7396E-09	0.30816953	1.32387E-09	7.22332E-08	LDLRAD3	protein_coding
ENSG00000179277.9	0.434404757	1.08306E-18	1.26107E-15	0.39506317	1.85675E-15	8.18911E-13	MEIS3P1	processed_pseudogene
ENSG00000179431.7	0.340455288	1.25003E-11	1.23976E-09	0.32083741	2.53616E-10	1.77818E-08	FJX1	protein_coding
ENSG00000180044.5	0.318997849	2.57062E-10	1.63559E-08	0.309198089	1.16096E-09	6.474E-08	C3orf80	protein_coding
ENSG00000180340.7	0.28823341	1.31534E-08	4.70661E-07	0.308713164	1.23518E-09	6.81944E-08	FZD2	protein_coding
ENSG00000180370.10	0.337395005	1.95209E-11	1.80106E-09	0.320753669	2.56467E-10	1.79459E-08	PAK2	protein_coding
ENSG00000180730.5	0.366651005	2.24368E-13	3.93442E-11	0.402485835	0	0	SHISA2	protein_coding
ENSG00000180773.15	0.461102346	3.86075E-21	1.18297E-17	0.419570282	2.0151E-17	1.3035E-14	SLC36A4	protein_coding
ENSG00000181004.10	0.338371355	1.69423E-11	1.60904E-09	0.343695191	7.75622E-12	9.27211E-10	BBS12	protein_coding
ENSG00000181191.12	0.449545846	4.70779E-20	9.45096E-17	0.458542496	0	0	PJA1	protein_coding
ENSG00000181458.10	0.439078552	4.18333E-19	5.79869E-16	0.443509071	1.67478E-19	1.26626E-16	TMEM45A	protein_coding
ENSG00000181788.4	0.245310934	1.52271E-06	2.76274E-05	0.304224144	2.1807E-09	1.08273E-07	SIAH2	protein_coding
ENSG00000181789.14	0.324316627	1.24184E-10	8.72101E-09	0.317786779	3.80261E-10	2.53006E-08	COPG1	protein_coding
ENSG00000181830.9	0.336542982	2.20809E-11	1.98687E-09	0.352168847	2.97186E-12	4.10964E-10	SLC35C1	protein_coding
ENSG00000181982.19	0.38603524	8.96276E-15	2.52074E-12	0.365110065	2.87191E-13	6.10207E-11	CCDC149	protein_coding
ENSG00000182095.14	0.313216323	5.57858E-10	3.16235E-08	0.308082052	1.09475E-09	6.20587E-08	TNRC18	protein_coding
ENSG00000182218.9	0.392431573	2.95267E-15	1.00045E-12	0.381093363	2.07912E-14	6.68742E-12	HHIPL1	protein_coding
ENSG00000182492.16	0.358316658	8.39471E-13	1.20375E-10	0.301736489	2.97609E-09	1.40863E-07	BGN	protein_coding
ENSG00000182667.14	0.426473111	5.26357E-18	4.37764E-15	0.417806349	0	0	NTM	protein_coding
ENSG00000182704.8	0.403985332	3.7344E-16	1.65961E-13	0.412987143	0	0	TSKU	protein_coding
ENSG00000182718.18	0.363236501	3.87034E-13	6.1396E-11	0.354496985	2.0852E-12	3.09684E-10	ANXA2	protein_coding
ENSG00000182827.9	0.299004208	3.49399E-09	1.52598E-07	0.313019001	7.09681E-10	4.33993E-08	ACBD3	protein_coding
ENSG00000182831.12	0.330989585	4.88464E-11	3.9224E-09	0.33553055	3.37493E-11	3.21574E-09	C16orf72	protein_coding
ENSG00000182934.12	0.312378886	6.23254E-10	3.48221E-08	0.303917397	2.26629E-09	1.11341E-07	SRPRA	protein_coding
ENSG00000183019.7	0.288078441	1.34013E-08	4.78649E-07	0.303593993	1.95283E-09	9.85179E-08	MCEMP1	protein_coding
ENSG00000183145.9	0.308662863	1.01502E-09	5.27809E-08	0.337236567	1.9974E-11	2.08395E-09	RIPPLY3	protein_coding
ENSG00000183196.10	0.397948244	1.11125E-15	4.25625E-13	0.421533466	1.3799E-17	9.23389E-15	CHST6	protein_coding
ENSG00000183255.12	0.407957689	1.80015E-16	8.8068E-14	0.41604096	0	0	PTTG1IP	protein_coding
ENSG00000183283.16	0.344773121	6.60933E-12	7.12559E-10	0.338522016	2.20709E-11	2.25426E-09	DAZAP2	protein_coding
ENSG00000183682.8	0.341804429	1.02542E-11	1.05102E-09	0.314566617	5.80262E-10	3.64027E-08	BMP8A	protein_coding
ENSG00000183696.14	0.336349977	2.27048E-11	2.03046E-09	0.301982154	2.39826E-09	1.16744E-07	UPP1	protein_coding
ENSG00000183853.18	0.369297418	1.46385E-13	2.70547E-11	0.310073501	1.03779E-09	5.92914E-08	KIRREL1	protein_coding
ENSG00000183876.9	0.373635918	7.20686E-14	1.50924E-11	0.356087837	1.63302E-12	2.53285E-10	ARSI	protein_coding
ENSG00000183914.14	0.232675941	5.27979E-06	8.03184E-05	0.344676378	6.7051E-12	8.27829E-10	DNAH2	protein_coding
ENSG00000183960.9	0.276902204	5.00193E-08	1.46924E-06	0.323109915	1.46654E-10	1.12637E-08	KCNH8	protein_coding
ENSG00000184009.12	0.288755754	1.23501E-08	4.46304E-07	0.328741381	8.69024E-11	7.20696E-09	ACTG1	protein_coding
ENSG00000184185.10	0.290724844	9.7273E-09	3.61394E-07	0.351909359	2.25586E-12	3.29153E-10	KCNJ12	protein_coding
ENSG00000184194.6	0.34792738	4.12351E-12	4.74431E-10	0.372058162	9.3368E-14	2.35312E-11	GPR173	protein_coding
ENSG00000184304.16	0.328708389	6.73713E-11	5.13573E-09	0.323996132	1.65932E-10	1.24648E-08	PRKD1	protein_coding
ENSG00000184349.13	0.323176296	1.45321E-10	9.92995E-09	0.303668011	1.93443E-09	9.77593E-08	EFNA5	protein_coding
ENSG00000184378.3	0.359928781	6.52317E-13	9.63653E-11	0.3677082	1.89267E-13	4.30421E-11	ACTRT3	protein_coding
ENSG00000184408.10	0.31808007	2.91028E-10	1.82576E-08	0.329881973	5.71188E-11	5.05358E-09	KCND2	protein_coding
ENSG00000184432.11	0.346357212	5.21852E-12	5.79793E-10	0.35463534	2.04149E-12	3.03968E-10	COPB2	protein_coding
ENSG00000184500.16	0.345880027	5.60425E-12	6.15601E-10	0.338868813	2.10038E-11	2.16042E-09	PROS1	protein_coding
ENSG00000184584.13	0.344759509	6.62272E-12	7.12683E-10	0.331487996	5.94477E-11	5.2201E-09	STING1	protein_coding
ENSG00000184731.6	0.372299715	8.97473E-14	1.78935E-11	0.341516005	1.06986E-11	1.23581E-09	FAM110C	protein_coding
ENSG00000184792.16	0.310844457	7.62929E-10	4.17445E-08	0.333495961	4.4925E-11	4.09303E-09	OSBP2	protein_coding
ENSG00000184867.14	0.340996246	1.15473E-11	1.1531E-09	0.310061668	1.03936E-09	5.93232E-08	ARMCX2	protein_coding
ENSG00000184985.16	0.330891599	4.95284E-11	3.96623E-09	0.301717653	2.4802E-09	1.20327E-07	SORCS2	protein_coding
ENSG00000185022.12	0.358126313	8.64767E-13	1.22793E-10	0.364068267	4.64191E-13	8.94844E-11	MAFF	protein_coding
ENSG00000185033.14	0.425899079	5.89217E-18	4.76431E-15	0.411548527	0	0	SEMA4B	protein_coding
ENSG00000185052.12	0.359172707	7.34366E-13	1.07151E-10	0.315185118	5.3523E-10	3.39804E-08	SLC24A3	protein_coding
ENSG00000185100.11	0.271008221	9.78811E-08	2.61277E-06	0.313130294	5.64256E-10	3.56676E-08	ADSS1	protein_coding
ENSG00000185168.5	0.329698458	5.86152E-11	4.59282E-09	0.3671074	2.08496E-13	4.65066E-11	LINC00482	lncRNA
ENSG00000185201.16	0.355130611	1.37623E-12	1.83292E-10	0.331265673	6.13122E-11	5.35957E-09	IFITM2	protein_coding
ENSG00000185332.8	0.228471461	7.86581E-06	0.00011293	0.301367648	2.59281E-09	1.24827E-07	TMEM105	lncRNA
ENSG00000185359.14	0.302460222	2.25677E-09	1.04522E-07	0.301600637	3.02681E-09	1.42569E-07	HGS	protein_coding
ENSG00000185442.13	0.408262343	1.70148E-16	8.39466E-14	0.390527705	3.3279E-15	1.36439E-12	FAM174B	protein_coding
ENSG00000185519.9	0.230124799	6.73057E-06	9.88747E-05	0.32392858	1.31017E-10	1.02109E-08	FAM131C	protein_coding
ENSG00000185567.7	0.391016668	3.78261E-15	1.20998E-12	0.372861076	1.07473E-13	2.66251E-11	AHNAK2	protein_coding

ENSG00000185634.12	0.288929518	1.20935E-08	4.38939E-07	0.327025484	8.52612E-11	7.11897E-09	SHC4	protein_coding
ENSG00000185787.15	0.349382659	3.311E-12	3.95E-10	0.358069405	1.20106E-12	1.98084E-10	MORF4L1	protein_coding
ENSG00000185900.11	0.294146652	6.39634E-09	2.55231E-07	0.316761634	4.35261E-10	2.82497E-08	POMK	protein_coding
ENSG00000185950.9	0.369701082	1.37106E-13	2.56401E-11	0.354868131	1.96995E-12	2.95584E-10	IRS2	protein_coding
ENSG00000185989.11	0.383574427	1.36516E-14	3.64573E-12	0.363318011	5.23757E-13	9.96473E-11	RASA3	protein_coding
ENSG00000186063.13	0.349683666	3.16362E-12	3.79752E-10	0.352606667	2.78112E-12	3.94129E-10	AIDA	protein_coding
ENSG00000186162.10	0.358548009	8.09697E-13	1.16846E-10	0.371351618	1.04799E-13	2.61854E-11	CIDECP1	transcribed_unprocessed_pseudogene
ENSG00000186174.12	0.360890942	5.60767E-13	8.37096E-11	0.32546433	1.36004E-10	1.05572E-08	BCL9L	protein_coding
ENSG00000186318.16	0.367872332	1.84324E-13	3.30183E-11	0.349845736	3.08695E-12	4.23858E-10	BACE1	protein_coding
ENSG00000186340.16	0.388208876	6.16233E-15	1.82111E-12	0.377114347	5.08468E-14	1.40962E-11	THBS2	protein_coding
ENSG00000186432.10	0.30273177	2.18004E-09	1.01453E-07	0.303773598	1.90848E-09	9.66995E-08	KPNA4	protein_coding
ENSG00000186716.21	0.315789759	3.9595E-10	2.35941E-08	0.331498464	5.93613E-11	5.2201E-09	BCR	protein_coding
ENSG00000186767.7	0.286316148	1.65585E-08	5.7109E-07	0.323334395	1.8143E-10	1.34042E-08	SPIN4	protein_coding
ENSG00000186831.11	0.199212843	0.000102811	0.001025262	0.317424391	3.17928E-10	2.16735E-08	KRT17P2	transcribed_unprocessed_pseudogene
ENSG00000186832.9	0.291391523	8.9683E-09	3.37721E-07	0.351923215	2.2511E-12	3.29153E-10	KRT16	protein_coding
ENSG00000186866.17	0.324790178	1.16314E-10	8.28832E-09	0.330302196	7.00708E-11	5.99909E-09	POFUT2	protein_coding
ENSG00000187013.4	0.278810157	4.01118E-08	1.22391E-06	0.339825977	1.37057E-11	1.51695E-09	LINC02875	lncRNA
ENSG00000187189.11	0.33047772	5.2513E-11	4.17081E-09	0.336805097	2.81803E-11	2.74807E-09	TSPYL4	protein_coding
ENSG00000187231.14	0.318885118	2.61016E-10	1.65712E-08	0.303791575	1.90409E-09	9.66116E-08	SESTD1	protein_coding
ENSG00000187260.16	0.332007892	4.22788E-11	3.49133E-09	0.337213867	2.00397E-11	2.08707E-09	WDR86	protein_coding
ENSG00000187676.8	0.394731523	1.96898E-15	7.16438E-13	0.390944726	3.83043E-15	1.53793E-12	B3GLCT	protein_coding
ENSG00000187720.14	0.390685524	4.00769E-15	1.26804E-12	0.382928661	1.71133E-14	5.66082E-12	THSD4	protein_coding
ENSG00000187800.13	0.370897916	1.1285E-13	2.1827E-11	0.35564979	1.74705E-12	2.68364E-10	PEAR1	protein_coding
ENSG00000188013.6	0.379298168	2.81259E-14	6.7384E-12	0.410777934	1.06594E-16	6.14422E-14	MEIS3P2	processed_pseudogene
ENSG00000188064.10	0.418152552	2.64478E-17	1.81146E-14	0.459836491	5.10115E-21	4.01322E-18	WNT7B	protein_coding
ENSG00000188157.15	0.36186116	4.81208E-13	7.37236E-11	0.346728866	6.70257E-12	8.27829E-10	AGRN	protein_coding
ENSG00000188306.6	0.283270522	2.37852E-08	7.75757E-07	0.322982333	1.49249E-10	1.14329E-08	LRRIQ4	protein_coding
ENSG00000188643.11	0.375221849	5.54744E-14	1.2006E-11	0.411031972	0	0	S100A16	protein_coding
ENSG00000188766.12	0.344346972	7.04146E-12	7.53566E-10	0.356272085	1.15359E-12	1.91448E-10	SPRED3	protein_coding
ENSG00000188833.10	0.321782771	1.7594E-10	1.17195E-08	0.317590661	3.10886E-10	2.12932E-08	ENTPD8	protein_coding
ENSG00000188910.8	0.362740789	4.18689E-13	6.53492E-11	0.368458755	2.26273E-13	4.93835E-11	GJB3	protein_coding
ENSG00000188997.8	0.315447305	4.1451E-10	2.44746E-08	0.334971574	2.76879E-11	2.7137E-09	KCTD21	protein_coding
ENSG00000189171.14	0.358599923	8.03159E-13	1.16679E-10	0.323779725	1.70853E-10	1.2785E-08	S100A13	protein_coding
ENSG00000189280.3	0.319834411	2.29482E-10	1.48444E-08	0.36947601	1.42206E-13	3.35181E-11	GJB5	protein_coding
ENSG00000189337.17	0.372326737	8.9351E-14	1.78757E-11	0.401324861	6.05502E-16	3.01292E-13	KAZN	protein_coding
ENSG00000189366.9	0.35626986	1.15398E-12	1.56969E-10	0.345872855	5.61026E-12	7.17842E-10	ALG1L	protein_coding
ENSG00000189420.8	0.343304865	8.21766E-12	8.63567E-10	0.302720094	2.18329E-09	1.08273E-07	ZFP92	protein_coding
ENSG00000189433.7	0.376997812	4.13125E-14	9.43189E-12	0.391114764	3.71835E-15	1.5033E-12	GJB4	protein_coding
ENSG00000196083.10	0.330615144	5.15031E-11	4.10179E-09	0.34406749	7.33971E-12	8.84686E-10	IL1RAP	protein_coding
ENSG00000196352.16	0.367257339	2.03526E-13	3.6235E-11	0.387385823	6.84289E-15	2.48987E-12	CD55	protein_coding
ENSG00000196428.12	0.309616713	8.96166E-10	4.76466E-08	0.304760041	2.03864E-09	1.02315E-07	TSC22D2	protein_coding
ENSG00000196526.10	0.358477643	8.18642E-13	1.17678E-10	0.337663709	1.87754E-11	1.97661E-09	AFAP1	protein_coding
ENSG00000196562.14	0.526785667	3.66525E-28	7.11278E-24	0.506467858	0	0	SULF2	protein_coding
ENSG00000196639.7	0.513466525	1.29885E-26	1.51233E-22	0.489773181	5.09289E-24	4.36026E-21	HRH1	protein_coding
ENSG00000196712.18	0.313058939	5.69616E-10	3.2165E-08	0.310915918	9.31296E-10	5.38948E-08	NF1	protein_coding
ENSG00000196878.15	0.32367282	1.35719E-10	9.36463E-09	0.318318352	3.54465E-10	2.38019E-08	LAMB3	protein_coding
ENSG00000196923.14	0.303030901	2.09845E-09	9.79692E-08	0.313687792	6.50636E-10	4.04145E-08	PDLIM7	protein_coding
ENSG00000196961.13	0.342228694	9.63309E-12	9.92874E-10	0.320681306	2.58956E-10	1.80983E-08	AP2A1	protein_coding
ENSG00000196968.11	0.332320443	4.04417E-11	3.36348E-09	0.303267056	2.03614E-09	1.02278E-07	FUT11	protein_coding
ENSG00000197081.16	0.296006417	5.08121E-09	2.11601E-07	0.30855524	1.26033E-09	6.93514E-08	IGF2R	protein_coding
ENSG00000197102.12	0.313304501	5.51373E-10	3.13475E-08	0.305959267	1.75251E-09	9.029E-08	DYNC1H1	protein_coding
ENSG00000197226.13	0.301439613	2.56926E-09	1.16857E-07	0.314254409	6.04372E-10	3.78337E-08	TBC1D9B	protein_coding
ENSG00000197324.9	0.348235154	3.93687E-12	4.5566E-10	0.350507814	2.79212E-12	3.94129E-10	LRP10	protein_coding
ENSG00000197479.7	0.308436509	1.0454E-09	5.41469E-08	0.348732279	4.97916E-12	6.44171E-10	PCDHB11	protein_coding
ENSG00000197496.6	0.390217485	4.34837E-15	1.36104E-12	0.36520264	3.8632E-13	7.89996E-11	SLC2A10	protein_coding
ENSG00000197548.12	0.332794169	3.78045E-11	3.18641E-09	0.340863124	1.5774E-11	1.70533E-09	ATG7	protein_coding
ENSG00000197608.11	0.331031292	4.85589E-11	3.9101E-09	0.310406417	9.9436E-10	5.70341E-08	ZNF841	protein_coding
ENSG00000197622.13	0.330938408	4.92015E-11	3.94547E-09	0.306590738	1.61793E-09	8.46294E-08	CDC42SE1	protein_coding
ENSG00000197694.18	0.328030429	7.40888E-11	5.59125E-09	0.323956669	1.3051E-10	1.0185E-08	SPTAN1	protein_coding
ENSG00000197696.10	0.325908169	9.96114E-11	7.26714E-09	0.303893634	1.87938E-09	9.56416E-08	NMB	protein_coding
ENSG00000197712.12	0.433169531	1.38923E-18	1.55535E-15	0.397882125	2.72366E-16	1.49591E-13	FAM114A1	protein_coding
ENSG00000197763.19	0.325488263	1.05591E-10	7.65541E-09	0.323379224	1.80337E-10	1.33573E-08	TXNRD3	protein_coding
ENSG00000197879.17	0.397162638	1.2786E-15	4.80243E-13	0.376577085	5.59934E-14	1.52328E-11	MYO1C	protein_coding
ENSG00000197965.12	0.353349244	1.81E-12	2.32761E-10	0.360975993	7.60835E-13	1.33417E-10	MPZL1	protein_coding
ENSG00000198108.4	0.389490188	4.93484E-15	1.50417E-12	0.37445616	6.29567E-14	1.68129E-11	CHSY3	protein_coding
ENSG00000198113.3	0.369687476	1.37409E-13	2.56401E-11	0.338138355	2.3313E-11	2.34816E-09	TOR4A	protein_coding
ENSG00000198121.13	0.34774522	4.23801E-12	4.84731E-10	0.325839591	1.00565E-10	8.14282E-09	LPAR1	protein_coding

ENSG00000198142.5	0.375409738	5.37757E-14	1.17255E-11	0.356181136	1.60968E-12	2.5124E-10	SOWAHC	protein_coding
ENSG00000198369.10	0.304740146	1.68609E-09	8.16076E-08	0.332819566	3.7668E-11	3.53702E-09	SPRED2	protein_coding
ENSG00000198406.7	0.324185674	1.2645E-10	8.84036E-09	0.324568115	1.19942E-10	9.43618E-09	BZW1P2	processed_pseudogene
ENSG00000198455.4	0.365110372	2.87177E-13	4.8743E-11	0.347516896	5.96487E-12	7.48411E-10	ZXDB	protein_coding
ENSG00000198464.14	0.350151203	2.94733E-12	3.56731E-10	0.303492548	2.39024E-09	1.16545E-07	ZNF480	protein_coding
ENSG00000198517.10	0.406605749	2.31015E-16	1.08462E-13	0.398689953	1.53115E-16	8.73928E-14	MAFK	protein_coding
ENSG00000198576.4	0.260077854	3.26047E-07	7.38733E-06	0.355097685	1.38323E-12	2.21843E-10	ARC	protein_coding
ENSG00000198585.12	0.31661291	3.54582E-10	2.16384E-08	0.305161679	1.9381E-09	9.78596E-08	NUDT16	protein_coding
ENSG00000198598.6	0.361351859	5.21486E-13	7.88568E-11	0.362101511	4.63272E-13	8.94844E-11	MMP17	protein_coding
ENSG00000198663.17	0.331667735	4.43707E-11	3.63633E-09	0.309417226	1.12885E-09	6.32528E-08	C6orf89	protein_coding
ENSG00000198715.13	0.347475307	4.4134E-12	4.99882E-10	0.322812152	1.94646E-10	1.42182E-08	GLMP	protein_coding
ENSG00000198729.5	0.269606948	1.14552E-07	2.98137E-06	0.306479839	1.34752E-09	7.33176E-08	PPP1R14C	protein_coding
ENSG00000198752.11	0.366145683	2.43322E-13	4.22858E-11	0.355375128	1.82244E-12	2.77746E-10	CDC42BPB	protein_coding
ENSG00000198753.12	0.34106984	1.14232E-11	1.1486E-09	0.32811833	9.46673E-11	7.76245E-09	PLXNB3	protein_coding
ENSG00000198792.13	0.372748378	8.33834E-14	1.69735E-11	0.368615315	2.20459E-13	4.84327E-11	TMEM184B	protein_coding
ENSG00000198863.7	0.305125191	1.60468E-09	7.82425E-08	0.318435772	3.49E-10	2.34891E-08	RUNDC1	protein_coding
ENSG00000198890.9	0.257691643	4.2099E-07	9.22091E-06	0.301921038	2.90851E-09	1.38001E-07	PRMT6	protein_coding
ENSG00000198892.7	0.434458876	1.07129E-18	1.26107E-15	0.397136421	4.05788E-16	2.1093E-13	SHISA4	protein_coding
ENSG00000198925.12	0.316271187	3.71219E-10	2.2442E-08	0.30429971	1.78412E-09	9.16003E-08	ATG9A	protein_coding
ENSG00000198945.8	0.439771305	3.62863E-19	5.15247E-16	0.417433909	3.03415E-17	1.92002E-14	L3MBTL3	protein_coding
ENSG00000198960.11	0.362355407	4.45036E-13	6.87244E-11	0.349377765	3.31345E-12	4.53888E-10	ARMCX6	protein_coding
ENSG00000203485.14	0.390008045	4.50986E-15	1.39657E-12	0.372939584	1.06029E-13	2.63795E-11	INF2	protein_coding
ENSG00000203722.8	0.329552025	5.98366E-11	4.67813E-09	0.360556758	5.91042E-13	1.08205E-10	RAET1G	protein_coding
ENSG00000203805.11	0.339457299	1.44638E-11	1.39644E-09	0.344375155	7.01205E-12	8.55331E-10	PLPP4	protein_coding
ENSG00000203879.12	0.321170526	1.91299E-10	1.25842E-08	0.309598134	1.103E-09	6.23443E-08	GDI1	protein_coding
ENSG00000204262.14	0.426970785	4.77229E-18	4.02657E-15	0.404711344	0	0	COL5A2	protein_coding
ENSG00000204272.13	0.310486385	7.99659E-10	4.33066E-08	0.31355285	6.62152E-10	4.09661E-08	NBDY	protein_coding
ENSG00000204463.13	0.291651781	8.68784E-09	3.28861E-07	0.303600865	2.35803E-09	1.15071E-07	BAG6	protein_coding
ENSG00000204560.10	0.331791933	4.35955E-11	3.59298E-09	0.326363409	1.20343E-10	9.455E-09	DHX16	protein_coding
ENSG00000204580.13	0.365056984	2.89637E-13	4.87699E-11	0.366825122	2.96376E-13	6.25161E-11	DDR1	protein_coding
ENSG00000204590.12	0.338840016	1.58256E-11	1.51648E-09	0.320831494	2.53816E-10	1.77818E-08	GNL1	protein_coding
ENSG00000204618.8	0.299216279	3.40208E-09	1.49143E-07	0.311180794	9.00064E-10	5.23999E-08	RNF39	protein_coding
ENSG00000204713.11	0.293630764	6.81608E-09	2.69578E-07	0.309940835	1.0556E-09	6.00733E-08	TRIM27	protein_coding
ENSG00000204949.9	0.326279869	9.45943E-11	6.93668E-09	0.358950222	7.60374E-13	1.33417E-10	FAM83A-AS1	lncRNA
ENSG00000205336.14	0.29325285	7.14036E-09	2.78618E-07	0.317184208	4.11712E-10	2.70226E-08	ADGRG1	protein_coding
ENSG00000205420.11	0.224326919	1.15673E-05	0.000157453	0.313070421	5.6875E-10	3.58068E-08	KRT6A	protein_coding
ENSG00000205517.13	0.333425699	3.45482E-11	2.95783E-09	0.324765521	1.16711E-10	9.20691E-09	RGL3	protein_coding
ENSG00000205730.6	0.446326566	9.29166E-20	1.63922E-16	0.417118899	0	0	ITPRIPL2	protein_coding
ENSG00000207340.1	0.388170347	6.20354E-15	1.82403E-12	0.38911537	5.26668E-15	2.01721E-12	RNVU1-1	snRNA
ENSG00000212747.5	0.350912814	2.62553E-12	3.21796E-10	0.31938129	2.44042E-10	1.72423E-08	RTL8B	protein_coding
ENSG00000213222.3	0.332401553	3.99778E-11	3.33442E-09	0.32795904	9.67583E-11	7.88946E-09	TOMM40P4	processed_pseudogene
ENSG00000213406.3	0.318016263	2.93546E-10	1.83957E-08	0.314156254	4.92391E-10	3.16402E-08	ANXA2P1	processed_pseudogene
ENSG00000213625.9	0.437557339	5.71069E-19	7.38811E-16	0.401803846	0	0	LEPROT	protein_coding
ENSG00000213859.7	0.431080134	2.11183E-18	2.08384E-15	0.387487086	6.69368E-15	2.45309E-12	KCTD11	protein_coding
ENSG00000215105.4	0.331785845	4.36332E-11	3.59298E-09	0.332467933	3.9602E-11	3.67125E-09	TTC3P1	processed_pseudogene
ENSG00000219438.9	0.369409427	1.43751E-13	2.66524E-11	0.362818068	5.6746E-13	1.05502E-10	TAFA5	protein_coding
ENSG00000219607.4	0.291537055	8.8104E-09	3.32496E-07	0.34265474	9.04636E-12	1.06182E-09	PPP1R3G	protein_coding
ENSG00000222009.8	0.353229038	1.84366E-12	2.35899E-10	0.31379133	6.41933E-10	3.99701E-08	BTBD19	protein_coding
ENSG00000222012.1	0.242186465	2.08415E-06	3.60687E-05	0.307544414	1.17395E-09	6.52771E-08	AC005481.1	lncRNA
ENSG00000223485.4	0.329379097	6.13109E-11	4.75181E-09	0.360570577	5.89759E-13	1.08205E-10	LINC01615	lncRNA
ENSG00000223652.2	0.401876168	5.47993E-16	2.27879E-13	0.407153383	2.08842E-16	1.18042E-13	AC106786.1	lncRNA
ENSG00000223829.6	0.252549121	7.24004E-07	1.46253E-05	0.321949152	1.71978E-10	1.28527E-08	AC004870.2	lncRNA
ENSG00000225190.11	0.35416213	1.59762E-12	2.09012E-10	0.32573243	1.02073E-10	8.24198E-09	PLEKHM1	protein_coding
ENSG00000225614.4	0.393680224	2.37055E-15	8.36415E-13	0.385551734	9.73798E-15	3.44134E-12	ZNF469	protein_coding
ENSG00000225950.8	0.280947838	3.12605E-08	9.84276E-07	0.304710127	1.6926E-09	8.77468E-08	NTF4	protein_coding
ENSG00000226137.5	0.339660972	1.40401E-11	1.36004E-09	0.327912846	9.73731E-11	7.92848E-09	BAIAP2-DT	lncRNA
ENSG00000226887.8	0.279255945	3.80863E-08	1.16885E-06	0.305505254	1.52808E-09	8.0662E-08	ERVMER34-1	protein_coding
ENSG00000227038.3	0.249986454	9.4448E-07	1.83592E-05	0.305075416	1.61499E-09	8.45517E-08	GTF2IP7	transcribed_unprocessed_pseudogene
ENSG00000228672.4	0.377156298	4.02365E-14	9.22239E-12	0.36128456	7.24523E-13	1.28992E-10	PROB1	protein_coding
ENSG00000229320.3	0.386732735	7.94997E-15	2.26878E-12	0.365611105	3.61482E-13	7.54293E-11	KRT8P12	transcribed_processed_pseudogene
ENSG00000229660.1	0.332269825	4.07338E-11	3.38294E-09	0.354019474	1.63304E-12	2.53285E-10	AC004975.2	lncRNA
ENSG00000230479.1	0.310659506	7.81691E-10	4.25711E-08	0.328315697	7.11863E-11	6.07672E-09	AP000695.1	lncRNA
ENSG00000230882.1	0.312988003	5.74995E-10	3.24056E-08	0.369979334	1.31048E-13	3.13965E-11	AC005077.4	processed_pseudogene
ENSG00000231274.5	0.364200296	3.32045E-13	5.44535E-11	0.415852044	4.10043E-17	2.53956E-14	SBK3	protein_coding
ENSG00000231870.4	0.220373827	1.65985E-05	0.000215219	0.353201569	1.85144E-12	2.80695E-10	KRT17P3	unprocessed_pseudogene
ENSG00000231991.4	0.362648896	4.2483E-13	6.61303E-11	0.336214131	3.06414E-11	2.95344E-09	ANXA2P2	processed_pseudogene
ENSG00000232004.1	0.323350599	1.41877E-10	9.74382E-09	0.300349807	2.94922E-09	1.39819E-07	CAP1P2	processed_pseudogene

ENSG00000232164.1	0.215202128	2.63629E-05	0.000319749	0.310018765	8.50221E-10	5.01501E-08	LINC01873	lncRNA
ENSG00000232679.2	0.271849585	8.90227E-08	2.40522E-06	0.309716585	8.84533E-10	5.1852E-08	LINC01705	lncRNA
ENSG00000233221.7	0.250502193	8.95486E-07	1.75237E-05	0.345797797	5.67348E-12	7.24339E-10	AC133785.1	lncRNA
ENSG00000233384.3	0.146749096	0.004403235	0.024192858	0.316369057	3.66378E-10	2.45452E-08	CNIH3-AS2	lncRNA
ENSG00000233532.6	0.255846951	5.12061E-07	1.08602E-05	0.340922496	1.16729E-11	1.32754E-09	LINC00460	lncRNA
ENSG00000233542.2	0.331310519	4.6676E-11	3.78994E-09	0.316260281	3.71762E-10	2.48487E-08	AL391845.2	lncRNA
ENSG00000233818.1	0.300725038	2.81262E-09	1.26138E-07	0.321761266	1.76459E-10	1.31369E-08	AP000695.2	lncRNA
ENSG00000233901.6	0.273280131	7.5706E-08	2.09181E-06	0.317091592	4.16765E-10	2.7262E-08	LINC01503	lncRNA
ENSG00000234405.2	0.324733541	1.17229E-10	8.34331E-09	0.331952873	4.26106E-11	3.92516E-09	Z69733.1	lncRNA
ENSG00000235437.8	0.300001126	3.0819E-09	1.36443E-07	0.324474703	1.21501E-10	9.53305E-09	LINC01278	lncRNA
ENSG00000235863.4	0.42735046	4.42808E-18	3.79109E-15	0.369033792	2.05609E-13	4.60389E-11	B3GALT4	protein_coding
ENSG00000236039.3	0.300146401	3.02593E-09	1.34271E-07	0.301315218	2.6101E-09	1.25375E-07	AC019117.1	lncRNA
ENSG00000236675.1	0.410544465	1.1134E-16	5.78751E-14	0.401329617	0	0	MTX1P1	unprocessed_pseudogene
ENSG00000237172.4	0.42992698	2.65769E-18	2.49557E-15	0.422700673	1.10043E-17	7.44939E-15	B3GNT9	protein_coding
ENSG00000237512.6	0.348866333	3.57957E-12	4.21852E-10	0.368603733	1.63786E-13	3.78384E-11	UNC5B-AS1	lncRNA
ENSG00000237523.2	0.392425632	2.95575E-15	1.00045E-12	0.352794857	2.70283E-12	3.8567E-10	LINC00857	lncRNA
ENSG00000237854.3	0.331502399	4.54236E-11	3.69856E-09	0.313196286	5.59341E-10	3.54339E-08	LINC00674	transcribed_unprocessed_pseudogene
ENSG00000239552.2	0.263609796	2.22316E-07	5.29575E-06	0.303545827	1.96489E-09	9.90407E-08	HOXB-AS2	lncRNA
ENSG00000240207.7	0.376644225	4.38158E-14	9.8489E-12	0.40249405	4.89898E-16	2.4587E-13	AC080013.1	lncRNA
ENSG00000240429.1	0.340363195	1.267E-11	1.25446E-09	0.337324194	1.97221E-11	2.06137E-09	LRRFIP1P1	processed_pseudogene
ENSG00000240764.4	0.341355544	1.09539E-11	1.10907E-09	0.301101487	2.68175E-09	1.28393E-07	PCDHGC5	protein_coding
ENSG00000242247.11	0.304704877	1.69374E-09	8.1763E-08	0.322278302	2.09122E-10	1.51614E-08	ARFGAP3	protein_coding
ENSG00000242265.6	0.282453338	2.61932E-08	8.43428E-07	0.302591421	2.67531E-09	1.2819E-07	PEG10	protein_coding
ENSG00000243232.6	0.314131914	4.93988E-10	2.84743E-08	0.355851065	1.23127E-12	2.01922E-10	PCDHAC2	protein_coding
ENSG00000244486.9	0.382794856	1.55867E-14	4.06919E-12	0.344332233	9.53258E-12	1.11439E-09	SCARF2	protein_coding
ENSG00000244586.1	0.417511783	2.98937E-17	2.00041E-14	0.397615679	1.1793E-15	5.5368E-13	WNT5A-AS1	lncRNA
ENSG00000246082.2	0.331016007	4.86641E-11	3.91316E-09	0.306758903	1.58381E-09	8.30689E-08	NUDT16P1	transcribed_unitary_pseudogene
ENSG00000246095.2	0.301268392	2.62564E-09	1.18844E-07	0.359140391	7.38088E-13	1.31006E-10	LINC01096	lncRNA
ENSG00000246640.1	0.303818793	1.89747E-09	9.01036E-08	0.30654737	1.33581E-09	7.27483E-08	PICART1	lncRNA
ENSG00000247095.3	0.291289487	9.08063E-09	3.41068E-07	0.316516118	3.59219E-10	2.40934E-08	MIR210HG	lncRNA
ENSG00000247596.10	0.307537348	1.17503E-09	5.97971E-08	0.308920259	9.81521E-10	5.64646E-08	TWF2	protein_coding
ENSG00000248383.5	0.253867549	6.30747E-07	1.29618E-05	0.307749441	1.14311E-09	6.39287E-08	PCDHAC1	protein_coding
ENSG00000248449.2	0.297001381	4.48944E-09	1.89809E-07	0.310916493	7.5574E-10	4.55463E-08	PCDHGB8P	transcribed_unitary_pseudogene
ENSG00000248846.3	0.233773653	4.75204E-06	7.33101E-05	0.307853798	1.12772E-09	6.32502E-08	LINC02065	lncRNA
ENSG00000249158.7	0.294609035	6.0415E-09	2.44253E-07	0.32419969	1.26205E-10	9.86231E-09	PCDHA11	protein_coding
ENSG00000249740.3	0.31322973	5.56867E-10	3.15981E-08	0.313776795	5.17869E-10	3.295E-08	OSMR-AS1	lncRNA
ENSG00000249992.2	0.378902217	3.00567E-14	7.1422E-12	0.344228942	7.16594E-12	8.70317E-10	TMEM158	protein_coding
ENSG00000249996.1	0.375821078	5.02328E-14	1.10471E-11	0.376056433	4.83096E-14	1.35939E-11	PPIC-AS1	lncRNA
ENSG00000251664.5	0.291345794	9.01847E-09	3.39171E-07	0.334130126	3.12381E-11	3.00102E-09	PCDHA12	protein_coding
ENSG00000253276.4	0.32996266	5.64727E-11	4.44889E-09	0.30898646	1.1928E-09	6.61356E-08	CCDC71L	protein_coding
ENSG00000253305.2	0.306007647	1.43225E-09	7.08436E-08	0.319429473	2.42452E-10	1.71716E-08	PCDHGB6	protein_coding
ENSG00000253352.10	0.352021788	2.2175E-12	2.77035E-10	0.352235067	2.94222E-12	4.07834E-10	TUG1	protein_coding
ENSG00000253485.2	0.328015979	7.42389E-11	5.59125E-09	0.326216936	9.54261E-11	7.81367E-09	PCDHGA5	protein_coding
ENSG00000253731.3	0.317470746	3.1595E-10	1.96517E-08	0.309368162	9.25765E-10	5.36815E-08	PCDHGA6	protein_coding
ENSG00000253846.2	0.341281983	1.10729E-11	1.11784E-09	0.335080157	2.72595E-11	2.68073E-09	PCDHGA10	protein_coding
ENSG00000253873.6	0.368086079	1.78074E-13	3.20963E-11	0.339885977	1.3586E-11	1.50658E-09	PCDHGA11	protein_coding
ENSG00000254221.2	0.284449189	2.06852E-08	6.88005E-07	0.3253989	1.06908E-10	8.53767E-09	PCDHGB1	protein_coding
ENSG00000254332.1	0.324337555	1.23825E-10	8.71687E-09	0.300001513	3.08175E-09	1.44688E-07	AF201337.1	processed_pseudogene
ENSG00000254369.6	0.250582104	8.88116E-07	1.7403E-05	0.322158031	1.67127E-10	1.25384E-08	HOXA-AS3	lncRNA
ENSG00000254531.1	0.319452339	2.41701E-10	1.55485E-08	0.34198976	1.34044E-11	1.4931E-09	FLJ20021	lncRNA
ENSG00000254585.5	0.254305604	6.02399E-07	1.24629E-05	0.309334356	9.29864E-10	5.38655E-08	MAGEL2	protein_coding
ENSG00000254615.3	0.376202633	4.71514E-14	1.04773E-11	0.405533168	2.81344E-16	1.53078E-13	AC027031.2	lncRNA
ENSG00000255112.3	0.306713414	1.30742E-09	6.56169E-08	0.313044487	7.07338E-10	4.33016E-08	CHMP1B	protein_coding
ENSG00000255438.2	0.346108148	5.4165E-12	5.98364E-10	0.367871484	1.84349E-13	4.20879E-11	AL354813.1	lncRNA
ENSG00000255508.7	0.322673671	1.55714E-10	1.05657E-08	0.322961539	1.49677E-10	1.14482E-08	AP002990.1	protein_coding
ENSG00000255874.2	0.493829437	1.89324E-24	9.18505E-21	0.486480115	1.12635E-23	9.50346E-21	PRECSIT	lncRNA
ENSG00000256806.6	0.33872541	1.60918E-11	1.53832E-09	0.322755185	1.53981E-10	1.16964E-08	C17orf100	protein_coding
ENSG00000257038.1	0.35563479	1.27314E-12	1.71574E-10	0.373214031	7.72459E-14	1.9811E-11	AP002761.3	lncRNA
ENSG00000257671.1	0.392967229	2.68753E-15	9.38899E-13	0.396716991	1.38428E-15	6.39602E-13	KRT7-AS	lncRNA
ENSG00000257718.1	0.329312064	6.18919E-11	4.78516E-09	0.301461074	2.56228E-09	1.2369E-07	CPNE8-AS1	lncRNA
ENSG00000259240.1	0.317893124	2.98464E-10	1.86437E-08	0.325988404	9.85069E-11	8.0096E-09	MIR4713HG	lncRNA
ENSG00000259341.2	0.307120427	1.24031E-09	6.25179E-08	0.327283929	8.22405E-11	6.92891E-09	AC015660.1	lncRNA
ENSG00000259439.2	0.284388427	2.0835E-08	6.91939E-07	0.320343347	2.14136E-10	1.53718E-08	LINC01833	lncRNA
ENSG00000259807.2	0.30370628	1.92498E-09	9.1187E-08	0.305947749	1.44337E-09	7.69504E-08	AC009093.1	lncRNA
ENSG00000260220.7	0.245299934	1.5244E-06	2.76473E-05	0.337584247	1.89929E-11	1.9959E-09	CCDC187	protein_coding
ENSG00000260597.1	0.273002262	7.81323E-08	2.15069E-06	0.300703173	2.82041E-09	1.34149E-07	AC012531.1	lncRNA
ENSG00000260643.2	0.298355788	3.79018E-09	1.63873E-07	0.306156523	1.40499E-09	7.5597E-08	AC092718.3	protein_coding

ENSG00000260976.2	0.230929163	6.23646E-06	9.25265E-05	0.312224673	6.36083E-10	3.96483E-08	LINC01633	lncRNA
ENSG00000261101.2	0.337084111	2.04196E-11	1.8633E-09	0.321735102	1.77092E-10	1.31504E-08	AC234775.2	lncRNA
ENSG00000261327.5	0.321267384	1.88785E-10	1.2447E-08	0.334427206	2.99368E-11	2.89993E-09	AC134312.5	lncRNA
ENSG00000261559.1	0.297597341	4.16758E-09	1.78141E-07	0.312301374	6.2967E-10	3.92906E-08	FSCN1P1	processed_pseudogene
ENSG00000261609.8	0.379966092	2.51406E-14	6.22824E-12	0.373896845	6.90382E-14	1.80754E-11	GAN	protein_coding
ENSG00000261934.2	0.383825761	1.30795E-14	3.50903E-12	0.38355913	1.36872E-14	4.60602E-12	PCDHGA9	protein_coding
ENSG00000262209.3	0.316325312	3.68534E-10	2.2326E-08	0.301674031	2.49397E-09	1.20894E-07	PCDHGB3	protein_coding
ENSG00000262576.3	0.318545507	2.7329E-10	1.72752E-08	0.350414066	2.83213E-12	3.95398E-10	PCDHGA4	protein_coding
ENSG00000263432.2	0.422277471	1.19465E-17	8.91671E-15	0.411983027	8.50804E-17	5.00324E-14	RN7SL689P	misc_RNA
ENSG00000264230.9	0.288704301	1.2427E-08	4.48047E-07	0.370683589	1.16861E-13	2.85858E-11	ANXA8L1	protein_coding
ENSG00000265190.7	0.308239005	1.07263E-09	5.54092E-08	0.36687549	2.16419E-13	4.77253E-11	ANXA8	protein_coding
ENSG00000265241.7	0.323191965	1.45009E-10	9.92022E-09	0.35686722	1.44769E-12	2.29026E-10	RBM8A	protein_coding
ENSG00000265519.1	0.412104718	8.31613E-17	4.61094E-14	0.377577199	4.67712E-14	1.33477E-11	AC015922.2	lncRNA
ENSG00000265808.4	0.309024848	9.68217E-10	5.08274E-08	0.3077308	1.39992E-09	7.53936E-08	SEC22B	protein_coding
ENSG00000266074.10	0.376539738	4.45835E-14	9.98293E-12	0.35279554	2.70255E-12	3.8567E-10	BAHCC1	protein_coding
ENSG00000266338.6	0.379038314	2.93788E-14	7.00974E-12	0.360798064	5.69026E-13	1.05502E-10	NBPF15	protein_coding
ENSG00000267493.4	0.320064784	2.22407E-10	1.44349E-08	0.335211363	2.67504E-11	2.63512E-09	CIRBP-AS1	lncRNA
ENSG00000268812.3	0.314843919	4.4929E-10	2.62618E-08	0.318041643	3.67673E-10	2.46037E-08	AC004264.1	lncRNA
ENSG00000268941.2	0.322995148	1.48987E-10	1.01447E-08	0.329351887	6.15461E-11	5.3667E-09	LINC01711	lncRNA
ENSG00000269378.1	0.337229917	1.99932E-11	1.83591E-09	0.326991467	1.10459E-10	8.80919E-09	ITGB1P1	processed_pseudogene
ENSG00000269556.8	0.363862814	3.5037E-13	5.66793E-11	0.348930026	4.83452E-12	6.2825E-10	TMEM185A	protein_coding
ENSG00000269934.3	0.3045029	1.73822E-09	8.3633E-08	0.310036454	8.48253E-10	5.00848E-08	AL353593.1	lncRNA
ENSG00000270362.1	0.307644076	1.15886E-09	5.91813E-08	0.300979861	3.26946E-09	1.52516E-07	HMGN3-AS1	lncRNA
ENSG00000271643.2	0.324649437	1.18601E-10	8.4101E-09	0.322086244	2.14581E-10	1.53848E-08	AC112220.2	lncRNA
ENSG00000272674.3	0.31277561	5.91395E-10	3.31374E-08	0.334051902	3.15899E-11	3.02982E-09	PCDHB16	protein_coding
ENSG00000272824.1	0.340776697	1.19252E-11	1.18677E-09	0.352623148	2.02286E-12	3.01966E-10	AC245100.6	lncRNA
ENSG00000272841.1	0.374663506	6.08382E-14	1.30216E-11	0.338170915	1.7443E-11	1.86329E-09	MAP3K4-AS1	lncRNA
ENSG00000273179.1	0.343142012	8.41801E-12	8.79856E-10	0.340887245	1.57193E-11	1.70418E-09	AC092535.5	lncRNA
ENSG00000273749.5	0.306299111	1.37936E-09	6.86942E-08	0.322425077	2.05041E-10	1.49027E-08	CYFIP1	protein_coding
ENSG00000273760.1	0.337254973	1.99208E-11	1.83215E-09	0.311170587	7.30905E-10	4.44174E-08	AC245041.1	lncRNA
ENSG00000275591.5	0.198923395	0.000105275	0.001046425	0.301335714	2.60333E-09	1.25153E-07	XKR5	protein_coding
ENSG00000275832.5	0.399036381	9.14457E-16	3.62162E-13	0.352910911	2.65562E-12	3.808E-10	ARHGAP23	protein_coding
ENSG00000275880.1	0.314936843	4.43755E-10	2.60166E-08	0.325615214	1.03747E-10	8.33097E-09	AL139385.1	lncRNA
ENSG00000276048.1	0.278696342	4.06454E-08	1.23566E-06	0.305701281	1.48997E-09	7.90983E-08	AC012354.4	misc_RNA
ENSG00000276855.1	0.339837951	1.36817E-11	1.33262E-09	0.31204735	6.51152E-10	4.04145E-08	AC015922.3	lncRNA
ENSG00000277013.1	0.321447248	1.84202E-10	1.2214E-08	0.309485713	9.1165E-10	5.29157E-08	AC008556.1	lncRNA
ENSG00000277304.1	0.287127219	1.5025E-08	5.27262E-07	0.304200806	1.80688E-09	9.25179E-08	AC142086.6	unprocessed_pseudogene
ENSG00000277311.1	0.282365456	2.64658E-08	8.49855E-07	0.326662225	8.96904E-11	7.41704E-09	AC012354.7	misc_RNA
ENSG00000278540.5	0.310760258	7.71416E-10	4.20902E-08	0.320883605	2.52056E-10	1.77225E-08	ACACA	protein_coding
ENSG00000278709.2	0.324409864	1.22594E-10	8.64067E-09	0.325702051	1.02504E-10	8.26536E-09	NKILA	lncRNA
ENSG00000278730.1	0.345000028	6.38991E-12	6.91464E-10	0.340171711	1.30301E-11	1.46163E-09	AC005332.6	lncRNA
ENSG00000279136.1	0.285461484	1.83378E-08	6.23229E-07	0.307696817	1.15095E-09	6.43054E-08	AL031258.1	TEC
ENSG00000279970.1	0.288738729	1.23755E-08	4.46875E-07	0.30020004	3.00551E-09	1.42025E-07	AC023024.2	TEC
ENSG00000280832.1	0.360969734	5.53853E-13	8.289E-11	0.335414647	2.59799E-11	2.56356E-09	GSEC	lncRNA
ENSG00000281091.3	0.336730189	2.14918E-11	1.94074E-09	0.355702187	1.25995E-12	2.0541E-10	AL117327.1	lncRNA
ENSG00000283538.3	0.304325648	1.77819E-09	8.5064E-08	0.430335363	2.45013E-18	1.76101E-15	AC005972.3	lncRNA
ENSG00000285517.1	0.291275168	9.0965E-09	3.41444E-07	0.32992553	5.67691E-11	5.04578E-09	AC010198.2	lncRNA
ENSG00000285867.1	0.405059107	3.06883E-16	1.39579E-13	0.392803435	2.76602E-15	1.1669E-12	BX470102.2	lncRNA
ENSG00000285906.2	0.33370486	3.31974E-11	2.84637E-09	0.341480942	1.44283E-11	1.57941E-09	AC083855.2	lncRNA
ENSG00000286156.1	0.317750412	3.04264E-10	1.89857E-08	0.317285698	4.06243E-10	2.68452E-08	AC026273.1	transcribed_unprocessed_pseudogene
ENSG00000286177.1	0.36887334	1.56795E-13	2.87999E-11	0.332660655	3.85303E-11	3.60057E-09	AC011462.5	lncRNA
ENSG00000286615.1	0.16982603	0.000960999	0.00679386	0.302933024	2.12482E-09	1.06E-07	AC011416.4	lncRNA
ENSG00000287331.1	0.261652918	2.75052E-07	6.37713E-06	0.326516118	9.15343E-11	7.54808E-09	AC106045.1	lncRNA
ENSG00000287929.1	0.318190543	2.86719E-10	1.80848E-08	0.327234705	8.28077E-11	6.95074E-09	AL354953.1	lncRNA
