# Supplementary material for: In‐Depth Examination of TPBG as a New Predictive Indicator for Gastric Cancer
Source: J Cell Mol Med. 2025 Jan 17;29(2):e70354. doi: 10.1111/jcmm.70354 (PMC11740983; doi:10.1111/jcmm.70354)
Supplement: Supplementary file 4 — Table S3. Co‐expressed TPBG‐related genes were identified in the TCGA database. [file JCMM-29-e70354-s005.pdf]

ONTOLOGY	ID	Description	GeneRatio	BgRatio	pvalue	p.adjust	qvalue	geneID	Count
BP	GO:0035987	endodermal cell differentiation	5/93	46/18800	3.10334E-06	0.00407202	0.003606796	ITGB5/COL12A1/COL7A1/MMP14/COL5A2	5
BP	GO:1903828	negative regulation of protein localization	8/93	199/18800	6.45994E-06	0.00407202	0.003606796	RHBDF1/SYTL4/RAB11FIP5/PPP3CA/LATS2/BAG3/SNX33/LEPROT	8
BP	GO:0001706	endoderm formation	5/93	55/18800	7.60435E-06	0.00407202	0.003606796	ITGB5/COL12A1/COL7A1/MMP14/COL5A2	5
BP	GO:0060350	endochondral bone morphogenesis	5/93	57/18800	9.08171E-06	0.00407202	0.003606796	TRPV4/NAB1/SERPINH1/MMP14/TSKU	5
BP	GO:0016055	Wnt signaling pathway	11/93	452/18800	1.4225E-05	0.00407202	0.003606796	PTPRU/CELSR1/MDFI/PTK7/RAB5A/TPBG/LATS2/SHISA2/TSKU/WNT7B/SULF2	11
BP	GO:0198738	cell-cell signaling by wnt	11/93	454/18800	1.48208E-05	0.00407202	0.003606796	PTPRU/CELSR1/MDFI/PTK7/RAB5A/TPBG/LATS2/SHISA2/TSKU/WNT7B/SULF2	11
BP	GO:0060348	bone development	8/93	225/18800	1.58005E-05	0.00407202	0.003606796	TRPV4/P3H1/NAB1/PDGFC/SERPINH1/MMP14/TSKU/SULF2	8
BP	GO:0007492	endoderm development	5/93	78/18800	4.21953E-05	0.009515051	0.008427966	ITGB5/COL12A1/COL7A1/MMP14/COL5A2	5
BP	GO:0010975	regulation of neuron projection development	10/93	431/18800	5.35113E-05	0.010726043	0.009500603	TRPV4/PTK7/DBN1/P3H1/PPP3CA/NDEL1/TANC2/BAIAP2/TSKU/SEMA4B	10
BP	GO:0060349	bone morphogenesis	5/93	98/18800	0.000125584	0.022578549	0.019998972	TRPV4/NAB1/SERPINH1/MMP14/TSKU	5
BP	GO:0048705	skeletal system morphogenesis	7/93	228/18800	0.000137674	0.022578549	0.019998972	TRPV4/MDFI/NAB1/SERPINH1/MMP14/BMP1/TSKU	7
BP	GO:0046888	negative regulation of hormone secretion	4/93	60/18800	0.000221387	0.027923977	0.02473369	SYTL4/LIF/RAB11FIP5/PPP3CA	4
BP	GO:0031532	actin cytoskeleton reorganization	5/93	111/18800	0.000225347	0.027923977	0.02473369	TRPV4/PTK7/SHC1/ANTXR1/BAIAP2	5
BP	GO:0030178	negative regulation of Wnt signaling pathway	6/93	174/18800	0.000225875	0.027923977	0.02473369	PTPRU/MDFI/TPBG/LATS2/SHISA2/TSKU	6
BP	GO:0030199	collagen fibril organization	4/93	62/18800	0.00025136	0.027923977	0.02473369	COL12A1/SERPINH1/BMP1/COL5A2	4
BP	GO:0030111	regulation of Wnt signaling pathway	8/93	336/18800	0.000260838	0.027923977	0.02473369	PTPRU/MDFI/PTK7/TPBG/LATS2/SHISA2/TSKU/SULF2	8
BP	GO:0030324	lung development	6/93	179/18800	0.000263142	0.027923977	0.02473369	GLI2/CELSR1/PTK7/LIF/MMP14/WNT7B	6
BP	GO:0030323	respiratory tube development	6/93	183/18800	0.000296303	0.029696146	0.02630339	GLI2/CELSR1/PTK7/LIF/MMP14/WNT7B	6
BP	GO:0051960	regulation of nervous system development	9/93	440/18800	0.000328702	0.029757999	0.026358177	SRPX2/DBN1/LIF/BHLHE40/PPP3CA/TPBG/NDEL1/BAIAP2/SEMA4B	9
BP	GO:0050709	negative regulation of protein secretion	4/93	68/18800	0.000358753	0.029757999	0.026358177	RHBDF1/SYTL4/RAB11FIP5/PPP3CA	4
BP	GO:0001704	formation of primary germ layer	5/93	123/18800	0.000362636	0.029757999	0.026358177	ITGB5/COL12A1/COL7A1/MMP14/COL5A2	5
BP	GO:0051224	negative regulation of protein transport	5/93	124/18800	0.000376411	0.029757999	0.026358177	RHBDF1/SYTL4/RAB11FIP5/PPP3CA/BAG3	5
BP	GO:0034394	protein localization to cell surface	4/93	69/18800	0.000379398	0.029757999	0.026358177	PTPRU/RAB11FIP5/SNX33/LEPROT	4
BP	GO:0051216	cartilage development	6/93	195/18800	0.000415832	0.031256718	0.027685668	TRPV4/SERPINH1/BMP1/TSKU/WNT7B/SULF2	6
BP	GO:1904950	negative regulation of establishment of protein localization	5/93	128/18800	0.000435506	0.031426115	0.027835711	RHBDF1/SYTL4/RAB11FIP5/PPP3CA/BAG3	5
BP	GO:0060351	cartilage development involved in endochondral bone morphogenesis	3/93	31/18800	0.000476442	0.033057729	0.029280915	TRPV4/SERPINH1/TSKU	3
BP	GO:0060541	respiratory system development	6/93	203/18800	0.000514438	0.033751009	0.029894989	GLI2/CELSR1/PTK7/LIF/MMP14/WNT7B	6
BP	GO:0002063	chondrocyte development	3/93	32/18800	0.000523852	0.033751009	0.029894989	SERPINH1/TSKU/SULF2	3
BP	GO:0048813	dendrite morphogenesis	5/93	139/18800	0.000634258	0.039455194	0.034947476	DBN1/PPP3CA/TPBG/TANC2/BAIAP2	5
BP	GO:0006023	aminoglycan biosynthetic process	4/93	80/18800	0.000665951	0.039797185	0.035250395	ST3GAL4/CHPF/CHST6/B3GNT9	4
BP	GO:0021955	central nervous system neuron axonogenesis	3/93	35/18800	0.000683876	0.039797185	0.035250395	GLI2/NDEL1/TSKU	3
BP	GO:1903531	negative regulation of secretion by cell	5/93	143/18800	0.000721177	0.040656351	0.036011402	RHBDF1/SYTL4/LIF/RAB11FIP5/PPP3CA	5
BP	GO:0030198	extracellular matrix organization	7/93	307/18800	0.000825975	0.044666272	0.039563194	COL12A1/SERPINH1/MMP14/BMP1/ANTXR1/SULF2/COL5A2	7
BP	GO:0043062	extracellular structure organization	7/93	308/18800	0.000841826	0.044666272	0.039563194	COL12A1/SERPINH1/MMP14/BMP1/ANTXR1/SULF2/COL5A2	7
BP	GO:0045229	external encapsulating structure organization	7/93	310/18800	0.000874249	0.044816623	0.039696367	COL12A1/SERPINH1/MMP14/BMP1/ANTXR1/SULF2/COL5A2	7
BP	GO:0010976	positive regulation of neuron projection development	5/93	150/18800	0.000894345	0.044816623	0.039696367	PTK7/DBN1/P3H1/NDEL1/BAIAP2	5
BP	GO:0060560	developmental growth involved in morphogenesis	6/93	234/18800	0.00107886	0.051757016	0.045843827	PTK7/DBN1/KDM5B/NDEL1/SEMA4B/WNT7B	6
BP	GO:2000008	regulation of protein localization to cell surface	3/93	41/18800	0.001090225	0.051757016	0.045843827	RAB11FIP5/SNX33/LEPROT	3
BP	GO:0007409	axonogenesis	8/93	430/18800	0.001311159	0.060437262	0.053532363	ETV1/GLI2/UNC5B/DBN1/NDEL1/BAIAP2/TSKU/SEMA4B	8
BP	GO:0061001	regulation of dendritic spine morphogenesis	3/93	44/18800	0.001340072	0.060437262	0.053532363	DBN1/TANC2/BAIAP2	3
BP	GO:0051048	negative regulation of secretion	5/93	167/18800	0.001442464	0.063468419	0.056217213	RHBDF1/SYTL4/LIF/RAB11FIP5/PPP3CA	5

BP	GO:2000009	negative regulation of protein localization to cell surface	2/93	12/18800	0.001547058	0.066449831	0.058858001	SNX33/LEPROT	2
BP	GO:0032535	regulation of cellular component size	7/93	351/18800	0.001786109	0.074933513	0.066372431	TRPV4/DBN1/RAB5A/NDEL1/BAIAP2/SEMA4B/WNT7B	7
BP	GO:0061448	connective tissue development	6/93	260/18800	0.001844787	0.075636287	0.066994914	TRPV4/SERPINH1/BMP1/TSKU/WNT7B/SULF2	6
BP	GO:0008544	epidermis development	7/93	355/18800	0.00190436	0.076129428	0.067431714	GLI2/COL7A1/KRT17/KRT7/NAB1/CD109/KAZN	7
BP	GO:0060425	lung morphogenesis	3/93	50/18800	0.001941216	0.076129428	0.067431714	CELSR1/LIF/WNT7B	3
BP	GO:0007173	epidermal growth factor receptor signaling pathway	4/93	108/18800	0.002031395	0.077970988	0.069062878	RHBDF1/FAM83A/SHC1/AFAP1L2	4
BP	GO:0050767	regulation of neurogenesis	7/93	361/18800	0.002093049	0.078663763	0.069676504	DBN1/LIF/BHLHE40/PPP3CA/NDEL1/BAIAP2/SEMA4B	7
BP	GO:0051962	positive regulation of nervous system development	6/93	270/18800	0.002229333	0.082075868	0.072698779	SRPX2/DBN1/LIF/TPBG/NDEL1/BAIAP2	6
BP	GO:0003413	chondrocyte differentiation involved in endochondral bone morphogenesis	2/93	15/18800	0.002437572	0.082858898	0.073392348	SERPINH1/TSKU	2
BP	GO:0060707	trophoblast giant cell differentiation	2/93	15/18800	0.002437572	0.082858898	0.073392348	MDFI/LIF	2
BP	GO:0042303	molting cycle	4/93	114/18800	0.002473182	0.082858898	0.073392348	GLI2/KRT17/CD109/TSKU	4
BP	GO:0042633	hair cycle	4/93	114/18800	0.002473182	0.082858898	0.073392348	GLI2/KRT17/CD109/TSKU	4
BP	GO:0007369	gastrulation	5/93	190/18800	0.002534893	0.082858898	0.073392348	ITGB5/COL12A1/COL7A1/MMP14/COL5A2	5
BP	GO:0060998	regulation of dendritic spine development	3/93	55/18800	0.002552496	0.082858898	0.073392348	DBN1/TANC2/BAIAP2	3
BP	GO:0061564	axon development	8/93	479/18800	0.002572117	0.082858898	0.073392348	ETV1/GLI2/UNC5B/DBN1/NDEL1/BAIAP2/TSKU/SEMA4B	8
BP	GO:0046879	hormone secretion	6/93	281/18800	0.002719188	0.084905678	0.075205286	SYTL4/TRPV4/KDM5B/LIF/RAB11FIP5/PPP3CA	6
BP	GO:0018146	keratan sulfate biosynthetic process	2/93	16/18800	0.002776849	0.084905678	0.075205286	ST3GAL4/CHST6	2
BP	GO:0042249	establishment of planar polarity of embryonic epithelium	2/93	16/18800	0.002776849	0.084905678	0.075205286	CELSR1/PTK7	2
BP	GO:0090066	regulation of anatomical structure size	8/93	487/18800	0.002846791	0.085593516	0.075814539	TRPV4/DBN1/RAB5A/NDEL1/BAIAP2/SEMA4B/WNT7B/HRH1	8
BP	GO:0060997	dendritic spine morphogenesis	3/93	58/18800	0.002970212	0.087840354	0.077804678	DBN1/TANC2/BAIAP2	3
BP	GO:0038127	ERBB signaling pathway	4/93	121/18800	0.003067262	0.089247426	0.079050994	RHBDF1/FAM83A/SHC1/AFAP1L2	4
BP	GO:0051223	regulation of protein transport	8/93	495/18800	0.003143925	0.089545652	0.079315148	RHBDF1/SYTL4/P3H1/RAB11FIP5/PPP3CA/BAG3/NDEL1/LEPROT	8
BP	GO:0009914	hormone transport	6/93	290/18800	0.003176786	0.089545652	0.079315148	SYTL4/TRPV4/KDM5B/LIF/RAB11FIP5/PPP3CA	6
BP	GO:0009612	response to mechanical stimulus	5/93	201/18800	0.003228802	0.089611664	0.079373618	ETV1/TRPV4/SLC2A1/BAG3/MMP14	5
BP	GO:0034504	protein localization to nucleus	6/93	294/18800	0.003397683	0.091972308	0.08146456	LIF/PPP3CA/LATS2/BAG3/TOR1AIP2/PTTG1IP	6
BP	GO:0043588	skin development	6/93	296/18800	0.003512336	0.091972308	0.08146456	GLI2/KRT17/KRT7/CD109/KAZN/COL5A2	6
BP	GO:0042339	keratan sulfate metabolic process	2/93	18/18800	0.003517788	0.091972308	0.08146456	ST3GAL4/CHST6	2
BP	GO:0072234	metanephric nephron tubule development	2/93	18/18800	0.003517788	0.091972308	0.08146456	LIF/WNT7B	2
BP	GO:0050807	regulation of synapse organization	5/93	209/18800	0.003812948	0.098265122	0.087038427	SRPX2/DBN1/TPBG/TANC2/BAIAP2	5
BP	GO:0061003	positive regulation of dendritic spine morphogenesis	2/93	19/18800	0.003919031	0.098366918	0.087128593	DBN1/BAIAP2	2
BP	GO:0048814	regulation of dendrite morphogenesis	3/93	64/18800	0.003925952	0.098366918	0.087128593	DBN1/PPP3CA/BAIAP2	3
CC	GO:0005788	endoplasmic reticulum lumen	10/100	311/19594	4.30681E-06	0.000925964	0.00080696	FSTL3/COL12A1/COL7A1/P3H1/PDGFC/SERPINH1/GPX8/PNPLA2/WNT7B/COL5A2	10
CC	GO:0031252	cell leading edge	9/100	416/19594	0.000278218	0.021132833	0.018416863	PDE4A/TRPV4/DBN1/ARHGEF4/RAB5A/PSD3/NDEL1/ANTXR1/BAIAP2	9
CC	GO:0001726	ruffle	6/100	177/19594	0.000294877	0.021132833	0.018416863	PDE4A/TRPV4/ARHGEF4/RAB5A/PSD3/BAIAP2	6
CC	GO:0032587	ruffle membrane	4/100	97/19594	0.001538974	0.058987584	0.051406561	PDE4A/TRPV4/ARHGEF4/PSD3	4
CC	GO:0062023	collagen-containing extracellular matrix	8/100	429/19594	0.001590881	0.058987584	0.051406561	TIMP2/SRPX2/COL12A1/COL7A1/P3H1/SERPINH1/ANXA5/COL5A2	8
CC	GO:0099524	postsynaptic cytosol	2/100	12/19594	0.001646165	0.058987584	0.051406561	DBN1/BAIAP2	2
CC	GO:0031256	leading edge membrane	5/100	175/19594	0.002039421	0.060439843	0.052672176	PDE4A/TRPV4/ARHGEF4/PSD3/ANTXR1	5
CC	GO:0060076	excitatory synapse	3/100	51/19594	0.002248924	0.060439843	0.052672176	SRPX2/SLC16A3/BAIAP2	3
CC	GO:0099522	cytosolic region	2/100	15/19594	0.002592896	0.061941404	0.053980758	DBN1/BAIAP2	2
CC	GO:0031092	platelet alpha granule membrane	2/100	17/19594	0.003336172	0.071727689	0.06250932	SYTL4/CD109	2
CC	GO:0005911	cell-cell junction	8/100	497/19594	0.003928335	0.076781101	0.066913273	PTPRU/TRPV4/PTK7/DBN1/SLC2A1/PPP3CA/BAIAP2/KAZN	8

CC	GO:0098644	complex of collagen trimers	2/100	22/19594	0.005573339	0.099855652	0.087022307	COL7A1/COL5A2	2
