# Supplementary material for: In‐Depth Examination of TPBG as a New Predictive Indicator for Gastric Cancer
Source: J Cell Mol Med. 2025 Jan 17;29(2):e70354. doi: 10.1111/jcmm.70354 (PMC11740983; doi:10.1111/jcmm.70354)
Supplement: Supplementary file 2 — Table S1. Differentially expressed genes in STAD samples with high and low TPBG expression. [file JCMM-29-e70354-s001.pdf]

id	baseMean	log2FoldChange	lfcSE	stat	pvalue	padj	gene_name	gene_type
ENSG00000000003.15	2685.29632	-0.000557521	0.107746616	-0.00517437	0.995871468	0.998070214	TSPAN6	protein_coding
ENSG00000000005.6	5.862450006	1.12028589	0.25407728	4.409232858	1.03737E-05	0.000335603	TNMD	protein_coding
ENSG00000000419.13	2936.332687	-0.16894859	0.060201407	-2.806389378	0.005010011	0.037243476	DPM1	protein_coding
ENSG00000000457.14	968.5105797	-0.003154195	0.047675	-0.066160347	0.947250166	0.975247377	SCYL3	protein_coding
ENSG00000000460.17	585.2195178	0.085480073	0.069729081	1.22588842	0.220240667	0.442198997	C1orf112	protein_coding
ENSG00000000938.13	674.9636643	0.077178668	0.101936364	0.75712597	0.448974396	0.66387151	FGR	protein_coding
ENSG00000000971.16	4594.115969	0.087331789	0.123415059	0.707626688	0.479177115	0.688589112	CFH	protein_coding
ENSG00000001036.14	3974.737435	-0.046795208	0.063687637	-0.734761249	0.462484911	0.674917283	FUCA2	protein_coding
ENSG00000001084.13	2980.597293	-0.107973257	0.065112686	-1.65825222	0.097266569	0.270888046	GCLC	protein_coding
ENSG00000001167.14	2184.900944	0.065831914	0.066184412	0.994674001	0.319894843	0.549556792	NFYA	protein_coding
ENSG00000001460.18	558.4903478	-0.108388888	0.059919666	-1.808903391	0.070466014	0.220722935	STPG1	protein_coding
ENSG00000001461.17	2535.799058	-0.17755999	0.060522568	-2.933781478	0.003348599	0.027874286	NIPAL3	protein_coding
ENSG00000001497.18	2327.712933	-0.072030702	0.051244781	-1.405620253	0.159836919	0.365641699	LAS1L	protein_coding
ENSG00000001561.7	1842.688033	0.260072415	0.089367131	2.910157363	0.003612468	0.029480879	ENPP4	protein_coding
ENSG00000001617.12	3529.326074	0.105180512	0.096842099	1.086103187	0.277433343	0.506314503	SEMA3F	protein_coding
ENSG00000001626.16	3702.616779	-0.554536488	0.188141699	-2.947440623	0.003204163	0.026991649	CFTR	protein_coding
ENSG00000001629.10	4969.571175	0.164443912	0.080604661	2.040129076	0.041337471	0.157237346	ANKIB1	protein_coding
ENSG00000001630.17	326.3695156	0.148497686	0.132468183	1.121006437	0.262285123	0.489804962	CYP51A1	protein_coding
ENSG00000001631.16	500.0964993	0.032978853	0.084047911	0.392381589	0.694776288	0.839049614	KRIT1	protein_coding
ENSG00000002016.18	591.891168	-0.118640749	0.061950237	-1.915097592	0.055480059	0.190141826	RAD52	protein_coding
ENSG00000002079.14	54.79927393	0.173237202	0.147286122	1.176195016	0.239516956	0.465043235	MYH16	transcribed_unitary_pseudogene
ENSG00000002330.14	732.4577952	-0.069157206	0.075111518	-0.920727047	0.357192951	0.584967388	BAD	protein_coding
ENSG00000002549.12	7914.93078	-0.160393947	0.084085445	-1.907511411	0.056454395	0.192301506	LAP3	protein_coding
ENSG00000002586.20	9288.501732	-0.162834016	0.077182362	-2.109730924	0.034881539	0.140424921	CD99	protein_coding
ENSG00000002586.20_PAR_Y	0						CD99	protein_coding
ENSG00000002587.10	2042.402755	0.094855297	0.124976464	0.758985282	0.447861361	0.663035656	HS3ST1	protein_coding
ENSG00000002726.21	10173.7587	0.025095776	0.153098544	0.163919105	0.869794827	0.936988262	AOC1	protein_coding
ENSG00000002745.13	33.57241237	0.64738084	0.179681303	3.602939366	0.000314639	0.004817646	WNT16	protein_coding
ENSG00000002746.15	90.94102788	0.424600488	0.125382284	3.386447239	0.000708039	0.0088281	HECW1	protein_coding
ENSG00000002822.15	53.07486732	0.372549676	0.109996447	3.386924642	0.000706808	0.008819314	MAD1L1	protein_coding
ENSG00000002834.18	21933.24153	0.071198397	0.071843601	0.991019335	0.321676139	0.551448179	LASP1	protein_coding
ENSG00000002919.15	1232.039686	0.054039581	0.038962139	1.386976746	0.165448854	0.373446841	SNX11	protein_coding
ENSG00000002933.9	10382.82509	-0.185416876	0.127481673	-1.454459075	0.145819072	0.346229919	TMEM176A	protein_coding
ENSG00000003056.8	3327.896221	-0.018728298	0.067796164	-0.276244218	0.782360497	0.889871577	M6PR	protein_coding
ENSG00000003096.14	385.2522934	-0.253917043	0.162100884	-1.566413688	0.117251801	0.302999568	KLHL13	protein_coding
ENSG00000003137.8	358.3123336	0.096132399	0.153908117	0.62460903	0.532227692	0.729795032	CYP26B1	protein_coding
ENSG00000003147.19	2460.52996	0.09870725	0.083107584	1.187704483	0.234949857	0.459657444	ICA1	protein_coding
ENSG00000003249.15	1923.388723	0.032235129	0.127970326	0.251895339	0.801121963	0.900894084	DBNDD1	protein_coding
ENSG00000003393.15	1844.300895	-0.0083206	0.047983258	-0.173406327	0.862332053	0.932938848	ALS2	protein_coding
ENSG00000003400.15	2873.648115	-0.129287557	0.086845941	-1.488700057	0.13656637	0.332503783	CASP10	protein_coding
ENSG00000003402.20	7493.938631	-0.074177486	0.056195484	-1.319990163	0.186838302	0.401808848	CFLAR	protein_coding

ENSG00000003436.16	3415.052809	0.504781443	0.119349951	4.229423119	2.34291E-05	0.00063095	TFPI	protein_coding
ENSG00000003509.16	730.5220165	-0.062935123	0.038027435	-1.654992595	0.097926	0.272029152	NDUFAF7	protein_coding
ENSG00000003756.17	3161.408002	-0.013135903	0.051801644	-0.253580816	0.799819419	0.900234786	RBM5	protein_coding
ENSG00000003987.14	110.542119	-0.021607934	0.101135455	-0.213653401	0.830817352	0.916687349	MTMR7	protein_coding
ENSG00000003989.18	1861.891317	0.806800004	0.183767411	4.390332321	1.13178E-05	0.000358165	SLC7A2	protein_coding
ENSG00000004059.11	4170.122697	-0.19517937	0.061842466	-3.15607354	0.001599085	0.016277352	ARF5	protein_coding
ENSG00000004139.14	488.8985697	0.10053355	0.077937144	1.289931164	0.197074559	0.414334832	SARM1	protein_coding
ENSG00000004142.12	6752.013314	-0.042236378	0.050032133	-0.84418503	0.398566019	0.621623424	POLDIP2	protein_coding
ENSG00000004399.13	8952.954156	0.392169712	0.085933696	4.563631401	5.02763E-06	0.000186936	PLXND1	protein_coding
ENSG00000004455.17	6404.836101	-0.10094808	0.045485776	-2.219332894	0.026464085	0.11728114	AK2	protein_coding
ENSG00000004468.13	1143.553077	-1.014463739	0.154489217	-6.566566652	5.14886E-11	1.56903E-08	CD38	protein_coding
ENSG00000004478.8	6887.71976	0.133165852	0.085304392	1.561066777	0.118507996	0.304976316	FKBP4	protein_coding
ENSG00000004487.17	5141.624911	0.096795056	0.055955958	1.729843594	0.083658223	0.246997895	KDM1A	protein_coding
ENSG00000004534.15	3172.891614	-0.145988739	0.053757403	-2.71569553	0.006613671	0.045236845	RBM6	protein_coding
ENSG00000004660.15	516.9517556	0.127201938	0.078619056	1.617952997	0.105672728	0.284586889	CAMKK1	protein_coding
ENSG00000004700.16	1142.428883	0.144287458	0.080730898	1.787264389	0.073894767	0.227779038	RECQL	protein_coding
ENSG00000004766.17	1720.778331	0.020176602	0.075416031	0.267537315	0.789055488	0.893990718	VPS50	protein_coding
ENSG00000004776.13	4515.558033	-0.643422348	0.243448393	-2.642951718	0.008218675	0.05273045	HSPB6	protein_coding
ENSG00000004777.18	588.9926787	-0.141690977	0.089696845	-1.579665121	0.114183577	0.298147148	ARHGAP33	protein_coding
ENSG00000004779.10	3925.433897	-0.130801362	0.063036301	-2.075016445	0.037985011	0.148801943	NDUFAB1	protein_coding
ENSG00000004799.8	5267.733471	-0.296598723	0.179790949	-1.649686621	0.099007048	0.273931881	PDK4	protein_coding
ENSG00000004809.14	11.69539653	0.247695533	0.182696999	1.355772311	0.175171624	0.386680201	SLC22A16	protein_coding
ENSG00000004838.14	33.08857705	0.31297635	0.10794201	2.899486018	0.00373775	0.030207704	ZMYND10	protein_coding
ENSG00000004846.16	26.54804599	-0.009767863	0.213149634	-0.04582632	0.96344868	0.98321265	ABCB5	protein_coding
ENSG00000004848.8	88.57577868	0.227151518	0.226194909	1.00422914	0.31526818	0.54443352	ARX	protein_coding
ENSG00000004864.14	2586.527879	-0.049220805	0.077894257	-0.631892615	0.527457049	0.725919181	SLC25A13	protein_coding
ENSG00000004866.22	1779.003938	-0.140904722	0.0653742	-2.155356709	0.031133931	0.130782996	ST7	protein_coding
ENSG00000004897.12	3762.682909	0.052788427	0.041889596	1.260179898	0.207604473	0.427541593	CDC27	protein_coding
ENSG00000004939.15	17.76315862	-0.417535733	0.189043967	-2.208669964	0.027197605	0.119522997	SLC4A1	protein_coding
ENSG00000004948.16	17.22406142	0.263525112	0.16161348	1.630588683	0.102977139	0.280412096	CALCR	protein_coding
ENSG00000004961.15	1914.555639	-0.106616336	0.050146941	-2.126078538	0.033496721	0.136867004	HCCS	protein_coding
ENSG00000004975.12	1860.765841	0.031462625	0.052516575	0.599098964	0.549106892	0.74205694	DVL2	protein_coding
ENSG00000005001.10	2155.889215	0.553683635	0.133947407	4.133589805	3.57141E-05	0.000875809	PRSS22	protein_coding
ENSG00000005007.13	9299.1035	0.052722334	0.054678852	0.964218014	0.334936635	0.564449445	UPF1	protein_coding
ENSG00000005020.13	3571.676942	-0.005046394	0.065308265	-0.07727038	0.938408454	0.970823446	SKAP2	protein_coding
ENSG00000005022.6	19568.50608	-0.214360145	0.071405738	-3.002001659	0.002682107	0.023640409	SLC25A5	protein_coding
ENSG00000005059.16	1015.253656	0.163220544	0.081173337	2.010765492	0.044350233	0.164733216	MCUB	protein_coding
ENSG00000005073.6	285.8080152	0.370005741	0.207262603	1.785202616	0.074228458	0.22850053	HOXA11	protein_coding
ENSG00000005075.15	2901.64802	-0.178774153	0.073733141	-2.424610574	0.015324816	0.081242031	POLR2J	protein_coding
ENSG00000005100.13	1769.082636	-0.021802596	0.052676523	-0.413895872	0.678950367	0.829927805	DHX33	protein_coding
ENSG00000005102.14	241.3495994	-0.003530847	0.143947467	-0.02452872	0.980430875	0.991327585	MEOX1	protein_coding
ENSG00000005108.16	383.1706452	0.339462828	0.11698286	2.901816786	0.003710055	0.030050752	THSD7A	protein_coding

ENSG00000005156.12	2032.280546	0.060948408	0.055469012	1.098783018	0.271862721	0.500061703	LIG3	protein_coding
ENSG00000005175.10	2024.563988	-0.09046297	0.049055475	-1.844095284	0.065169255	0.210361528	RPAP3	protein_coding
ENSG00000005187.12	908.3761483	-0.522452005	0.139498115	-3.745226279	0.000180231	0.003147871	ACSM3	protein_coding
ENSG00000005189.20	380.4210492	-0.274118519	0.078902266	-3.474152686	0.000512469	0.006922466	REXO5	protein_coding
ENSG00000005194.15	2437.755865	-0.031076256	0.052555234	-0.591306588	0.554315016	0.745458233	CIAPIN1	protein_coding
ENSG00000005206.17	3450.283096	-0.005460968	0.06264234	-0.08717695	0.930530861	0.967010903	SPPL2B	protein_coding
ENSG00000005238.20	1579.799968	0.208208049	0.078622892	2.648186076	0.008092497	0.052171764	FAM214B	protein_coding
ENSG00000005243.10	471.8535103	0.418415248	0.11887048	3.519925612	0.000431668	0.006095525	COPZ2	protein_coding
ENSG00000005249.13	926.9595445	-0.024577163	0.139339141	-0.176383768	0.859992456	0.931893212	PRKAR2B	protein_coding
ENSG00000005302.19	2096.082907	-0.080869689	0.045987311	-1.758521817	0.078658763	0.237680316	MSL3	protein_coding
ENSG00000005339.15	10600.42265	0.031210925	0.049825208	0.626408336	0.531047143	0.728903046	CREBBP	protein_coding
ENSG00000005379.17	584.651427	-0.30928001	0.131199191	-2.357331692	0.018406797	0.091898355	TSPOAP1	protein_coding
ENSG00000005381.8	14.53477054	0.159373258	0.13222649	1.205305069	0.228085554	0.451518838	MPO	protein_coding
ENSG00000005421.9	44.30104927	0.657758265	0.23629391	2.783644594	0.00537519	0.03911007	PON1	protein_coding
ENSG00000005436.14	1065.631327	-0.081278695	0.046525589	-1.746967575	0.080642963	0.241225864	GCFC2	protein_coding
ENSG00000005448.17	591.6065074	0.202605477	0.081526753	2.48514099	0.012950013	0.072376906	WDR54	protein_coding
ENSG00000005469.12	799.9340642	-0.429347479	0.094035091	-4.565821896	4.97541E-06	0.000185469	CROT	protein_coding
ENSG00000005471.19	98.79717101	-0.076496197	0.116236963	-0.658105605	0.510470275	0.713705183	ABCB4	protein_coding
ENSG00000005483.21	7766.747948	-0.016596466	0.055076462	-0.301335007	0.763159049	0.879069956	KMT2E	protein_coding
ENSG00000005486.17	7574.996355	-0.093473506	0.072868516	-1.282769451	0.19957286	0.417773286	RHBDD2	protein_coding
ENSG00000005513.10	278.7942403	0.002465467	0.187674849	0.013136907	0.989518566	0.995274104	SOX8	protein_coding
ENSG00000005700.15	5445.692077	0.200393541	0.069081333	2.900834885	0.003721699	0.03010755	IBTK	protein_coding
ENSG00000005801.18	2085.73019	-0.165966373	0.06977038	-2.378751167	0.017371398	0.088402827	ZNF195	protein_coding
ENSG00000005810.18	4654.734476	-0.067247496	0.059425602	-1.131624988	0.25779213	0.484817913	MYCBP2	protein_coding
ENSG00000005812.11	2821.851822	0.078021409	0.048898439	1.595580778	0.110582422	0.292246358	FBXL3	protein_coding
ENSG00000005844.19	1741.456291	-0.613244857	0.123621256	-4.960674849	7.02487E-07	3.92969E-05	ITGAL	protein_coding
ENSG00000005882.12	1950.756715	-0.120901743	0.073174858	-1.652230654	0.09848754	0.272962636	PDK2	protein_coding
ENSG00000005884.18	16434.05702	0.564193886	0.100051113	5.639056583	1.70984E-08	1.88633E-06	ITGA3	protein_coding
ENSG00000005889.16	2638.442616	-0.050161616	0.055815355	-0.898706382	0.368809076	0.595597136	ZFX	protein_coding
ENSG00000005893.16	11069.83952	-0.011019868	0.057485542	-0.191698086	0.847978705	0.925502472	LAMP2	protein_coding
ENSG00000005961.19	33.94866615	0.14814915	0.132324804	1.119587154	0.262889732	0.490513631	ITGA2B	protein_coding
ENSG00000005981.13	270.651054	2.181841938	0.325360543	6.705920503	2.00141E-11	6.98614E-09	ASB4	protein_coding
ENSG00000006007.12	5212.136994	0.060663615	0.052472019	1.156113603	0.247634693	0.474220927	GDE1	protein_coding
ENSG00000006015.18	1439.190614	-0.101287988	0.05814415	-1.742015125	0.081505798	0.242924671	REX1BD	protein_coding
ENSG00000006016.11	190.3755699	0.31228364	0.195206705	1.599758782	0.109652106	0.29054541	CRLF1	protein_coding
ENSG00000006025.12	2255.080861	-0.066606638	0.097328128	-0.684351369	0.493753317	0.700739736	OSBPL7	protein_coding
ENSG00000006042.12	3927.02532	-0.330887643	0.090145586	-3.670591717	0.00024199	0.003932524	TMEM98	protein_coding
ENSG00000006047.13	843.5506439	-0.135297784	0.170084636	-0.795473283	0.426338246	0.644998672	YBX2	protein_coding
ENSG00000006059.4	2.840081945	-0.243298793	0.421142063	-0.577711928	0.563458625	0.752262147	KRT33A	protein_coding
ENSG00000006062.17	1468.276548	-0.297036642	0.078285427	-3.79427759	0.000148074	0.002695329	MAP3K14	protein_coding
ENSG00000006071.15	61.30492009	-0.551370607	0.197922053	-2.785796731	0.005339634	0.038931922	ABCC8	protein_coding
ENSG00000006116.4	1.922997335	-0.537300149	0.305349908	-1.759621123	0.078472068	0.23730585	CACNG3	protein_coding

ENSG00000006118.15	2737.380191	0.308009842	0.102194708	3.013950985	0.002578695	0.022989316	TMEM132A	protein_coding
ENSG00000006125.18	21367.2249	0.200909407	0.058418068	3.439165571	0.00058351	0.007642809	AP2B1	protein_coding
ENSG00000006128.12	42.52410501	-0.191057873	0.313785105	-0.608881271	0.542603139	0.737554618	TAC1	protein_coding
ENSG00000006194.10	1584.639079	-0.078578665	0.043460834	-1.808034004	0.070601208	0.220971692	ZNF263	protein_coding
ENSG00000006210.7	2717.198933	-0.418267279	0.149608204	-2.795750945	0.005177929	0.038121067	CX3CL1	protein_coding
ENSG00000006282.21	3383.771979	-0.221689709	0.067487598	-3.284895506	0.001020202	0.011669316	SPATA20	protein_coding
ENSG00000006283.18	33.72822749	0.346554662	0.15093836	2.296001238	0.021675811	0.102592245	CACNA1G	protein_coding
ENSG00000006327.14	4400.639472	0.229732552	0.1074996	2.13705495	0.032593522	0.134589968	TNFRSF12A	protein_coding
ENSG00000006377.11	116.626517	0.505528538	0.263943712	1.915289189	0.055455634	0.190112779	DLX6	protein_coding
ENSG00000006432.15	1313.067349	0.218254476	0.087101394	2.505751805	0.012219136	0.069368588	MAP3K9	protein_coding
ENSG00000006451.8	4345.949865	-0.017698661	0.051552964	-0.343310253	0.731365068	0.860426833	RALA	protein_coding
ENSG00000006453.14	6763.8309	0.013220831	0.103259496	0.128035012	0.898121266	0.951577485	BAIAP2L1	protein_coding
ENSG00000006459.11	2924.704288	-0.061433287	0.075223041	-0.816681781	0.414110305	0.634651642	KDM7A	protein_coding
ENSG00000006468.14	863.52674	0.800507055	0.113403681	7.058915977	1.67806E-12	9.02778E-10	ETV1	protein_coding
ENSG00000006530.18	1551.704046	-0.177968382	0.051574728	-3.450689697	0.000559156	0.007399515	AGK	protein_coding
ENSG00000006534.16	3601.842034	0.44217357	0.113684944	3.889464649	0.000100466	0.001991806	ALDH3B1	protein_coding
ENSG00000006555.11	2678.546238	0.14052756	0.113819452	1.234653284	0.216959569	0.438253703	TTC22	protein_coding
ENSG00000006576.16	1453.812892	-0.041505397	0.055791925	-0.743931977	0.456917629	0.670803689	PHTF2	protein_coding
ENSG00000006606.9	82.70084625	-0.297415357	0.181814355	-1.635818896	0.101877496	0.278527308	CCL26	protein_coding
ENSG00000006607.14	4562.456338	-0.086537039	0.055360568	-1.563153012	0.118016609	0.304164288	FARP2	protein_coding
ENSG00000006611.17	4680.94112	-0.18665432	0.110934115	-1.682569147	0.092458529	0.262531133	USH1C	protein_coding
ENSG00000006625.18	1503.178056	-0.35811684	0.076184222	-4.700669411	2.5931E-06	0.000111467	GGCT	protein_coding
ENSG00000006634.8	1549.165989	-0.155460453	0.08030594	-1.935852481	0.052885771	0.184262805	DBF4	protein_coding
ENSG00000006638.13	253.3657906	0.052301184	0.093762089	0.557807365	0.576975933	0.761697726	TBXA2R	protein_coding
ENSG00000006652.15	580.5690994	-0.156769105	0.080458042	-1.948457873	0.051360201	0.18103401	IFRD1	protein_coding
ENSG00000006659.13	3.585532196	-0.00873432	0.467348222	-0.018689104	0.98508912	0.993217373	LGALS14	protein_coding
ENSG00000006695.12	1284.877307	-0.044951964	0.051139005	-0.879015243	0.379393011	0.605027641	COX10	protein_coding
ENSG00000006704.11	3001.994184	0.106078408	0.074276092	1.428163558	0.153244787	0.356780173	GTF2IRD1	protein_coding
ENSG00000006712.15	3027.296631	0.179433826	0.055561345	3.229472295	0.001240189	0.013527838	PAF1	protein_coding
ENSG00000006715.16	3692.151128	-0.015768683	0.049816396	-0.316536016	0.751595693	0.872375074	VPS41	protein_coding
ENSG00000006740.17	1017.294768	-0.235750343	0.106718056	-2.209095191	0.027168021	0.119453217	ARHGAP44	protein_coding
ENSG00000006744.19	4274.179979	-0.003998433	0.050079918	-0.079841045	0.936363679	0.969836238	ELAC2	protein_coding
ENSG00000006747.15	880.3090479	0.374538095	0.157148902	2.383332554	0.017156688	0.087663101	SCIN	protein_coding
ENSG00000006756.16	3333.680508	-0.0043904	0.076539032	-0.057361585	0.954257163	0.978600343	ARSD	protein_coding
ENSG00000006757.12	882.0559597	-0.239843979	0.099177587	-2.418328443	0.015591996	0.082246736	PNPLA4	protein_coding
ENSG00000006788.14	21.65172582	-0.280493581	0.181488175	-1.545519871	0.122220566	0.310971309	MYH13	protein_coding
ENSG00000006831.10	5083.588661	0.169117349	0.067061697	2.521817341	0.011675031	0.067280381	ADIPOR2	protein_coding
ENSG00000006837.12	29.04013449	0.03749823	0.080185751	0.467642058	0.640040583	0.80515942	CDKL3	protein_coding
ENSG00000007001.13	8.414137525	-0.154265962	0.145490064	-1.06031957	0.288999239	0.518704387	UPP2	protein_coding
ENSG00000007038.11	774.5183235	0.447604543	0.253249518	1.767444794	0.077153767	0.234638125	PRSS21	protein_coding
ENSG00000007047.15	2416.998405	0.11704572	0.057443095	2.037594247	0.041590528	0.157956287	MARK4	protein_coding
ENSG00000007062.12	5814.171695	0.037476585	0.169147566	0.221561477	0.824655274	0.913424534	PROM1	protein_coding

ENSG00000007080.11	2852.456699	-0.131660335	0.067361065	-1.95454652	0.050636619	0.179397719	CCDC124	protein_coding
ENSG00000007129.18	85.00082381	-0.303311366	0.120331819	-2.520624802	0.011714669	0.067435947	CEACAM21	protein_coding
ENSG00000007168.14	6182.655563	0.048922434	0.040862802	1.197236412	0.231214421	0.455171518	PAFAH1B1	protein_coding
ENSG00000007171.18	1972.742524	-0.56457841	0.237749485	-2.374677742	0.017564277	0.089031825	NOS2	protein_coding
ENSG00000007174.18	45.43129963	0.2390401	0.180023056	1.327830476	0.184234117	0.39833339	DNAH9	protein_coding
ENSG00000007202.15	8894.009327	0.139603659	0.056368638	2.476619351	0.013263327	0.073590019	KIAA0100	protein_coding
ENSG00000007216.15	659.5984402	-0.732212415	0.296820629	-2.466851502	0.013630687	0.07507387	SLC13A2	protein_coding
ENSG00000007237.19	2547.486389	0.108954773	0.120748551	0.902327794	0.366882765	0.593894884	GAS7	protein_coding
ENSG00000007255.10	853.7647648	-0.06764318	0.087951833	-0.769093467	0.441837826	0.658277786	TRAPPC6A	protein_coding
ENSG00000007264.15	162.6441851	-0.242293354	0.119955717	-2.01985666	0.043398258	0.162429433	MATK	protein_coding
ENSG00000007306.15	2253.765364	1.066969328	0.246224819	4.333313481	1.46882E-05	0.000439606	CEACAM7	protein_coding
ENSG00000007312.13	187.1985646	-0.90876509	0.176817013	-5.139579467	2.75354E-07	1.83192E-05	CD79B	protein_coding
ENSG00000007314.12	32.02761132	0.294850021	0.167894386	1.756163667	0.079060466	0.238406889	SCN4A	protein_coding
ENSG00000007341.19	691.9630479	0.01413292	0.035714476	0.395719653	0.692311856	0.837669572	ST7L	protein_coding
ENSG00000007350.17	178.3484397	2.638188425	0.283503769	9.305655559	1.33168E-20	5.27552E-17	TKTL1	protein_coding
ENSG00000007372.24	121.5859345	0.293629934	0.227848208	1.288708551	0.197499429	0.414787826	PAX6	protein_coding
ENSG00000007376.8	1778.487232	-0.01499616	0.073119363	-0.205091497	0.83750064	0.92008445	RPUSD1	protein_coding
ENSG00000007384.15	2454.38062	0.31615818	0.069042447	4.579185613	4.6679E-06	0.000176574	RHBDF1	protein_coding
ENSG00000007392.17	1479.736592	-0.068546647	0.052852338	-1.296946359	0.194649644	0.411499905	LUC7L	protein_coding
ENSG00000007402.12	236.1346605	0.180196221	0.143892513	1.252297405	0.210461514	0.43075906	CACNA2D2	protein_coding
ENSG00000007516.14	288.6689227	0.162814844	0.13836831	1.176677261	0.239324347	0.464752098	BAIAP3	protein_coding
ENSG00000007520.4	1559.771781	-0.026912717	0.068233307	-0.394421999	0.693269505	0.838038536	TSR3	protein_coding
ENSG00000007541.17	1774.709848	-0.017894094	0.05902897	-0.303140887	0.761782496	0.878159776	PIGQ	protein_coding
ENSG00000007545.16	839.3443371	-0.081604586	0.050827299	-1.605526703	0.108377941	0.28869647	CRAMP1	protein_coding
ENSG00000007866.22	3626.127901	0.143985794	0.069489804	2.072042007	0.038261524	0.149575888	TEAD3	protein_coding
ENSG00000007908.16	575.8688124	0.176697758	0.164757856	1.07246939	0.283509255	0.512336519	SELE	protein_coding
ENSG00000007923.16	2037.435166	-0.059912209	0.059788238	-1.002073506	0.316308093	0.545524113	DNAJC11	protein_coding
ENSG00000007933.13	352.469845	-0.532081152	0.121811085	-4.368084811	1.25341E-05	0.000387925	FMO3	protein_coding
ENSG00000007944.15	2118.460635	-0.092288811	0.066904822	-1.379404473	0.167770081	0.376860697	MYLIP	protein_coding
ENSG00000007952.18	631.4717066	0.054179405	0.227602314	0.238044175	0.811846832	0.907099176	NOX1	protein_coding
ENSG00000007968.7	966.4714562	-0.293507847	0.104662728	-2.804320635	0.005042273	0.037432223	E2F2	protein_coding
ENSG00000008018.9	7990.933766	-0.070903159	0.054470978	-1.301668543	0.193029718	0.409425943	PSMB1	protein_coding
ENSG00000008056.14	222.3512387	0.058428369	0.143904963	0.406020527	0.684727518	0.833613763	SYN1	protein_coding
ENSG00000008083.14	2045.594314	0.016860958	0.063926754	0.263754323	0.791969234	0.895592145	JARID2	protein_coding
ENSG00000008086.13	1307.508605	-0.005204318	0.081975785	-0.063486036	0.949379479	0.97630937	CDKL5	protein_coding
ENSG00000008118.10	44.61169399	0.041364644	0.096796978	0.427334044	0.669136039	0.823605841	CAMK1G	protein_coding
ENSG00000008128.23	63.10666755	-0.128180236	0.079058186	-1.621340465	0.104944641	0.283557211	CDK11A	protein_coding
ENSG00000008130.15	4703.754933	-0.055524818	0.053068418	-1.046287413	0.295428355	0.525263561	NADK	protein_coding
ENSG00000008196.13	7.038364811	-0.22077841	0.267344902	-0.825818663	0.408906965	0.629800263	TFAP2B	protein_coding
ENSG00000008197.5	3.725590561	-0.210828986	0.354075243	-0.595435546	0.551552371	0.743701843	TFAP2D	protein_coding
ENSG00000008226.20	69.79101944	0.420275058	0.094343922	4.454712586	8.40057E-06	0.000281593	DLEC1	protein_coding
ENSG00000008256.16	2749.897699	0.208835765	0.069030309	3.025276403	0.00248406	0.022337574	CYTH3	protein_coding

ENSG00000008277.14	423.041033	0.341678487	0.119551521	2.858002012	0.004263177	0.033156605	ADAM22	protein_coding
ENSG00000008282.9	9002.560495	-0.144869614	0.060456241	-2.396272286	0.016562781	0.085763751	SYPL1	protein_coding
ENSG00000008283.16	5891.10999	0.05146524	0.070629986	0.728659919	0.466209716	0.67779391	CYB561	protein_coding
ENSG00000008294.21	5485.953733	0.034605873	0.052702049	0.6566324	0.511417311	0.714273009	SPAG9	protein_coding
ENSG00000008300.17	1615.746781	0.472426548	0.129559785	3.646398052	0.000265942	0.004237276	CELSR3	protein_coding
ENSG00000008311.15	557.6008102	0.171393345	0.099239393	1.727069663	0.084155153	0.24781924	AASS	protein_coding
ENSG00000008323.15	2743.339133	-0.314396199	0.118780618	-2.64686449	0.00812419	0.052324539	PLEKHG6	protein_coding
ENSG00000008324.12	953.482101	-0.183890018	0.058903413	-3.121890722	0.001796936	0.017708569	SS18L2	protein_coding
ENSG00000008382.16	594.9652191	-0.270763739	0.071696649	-3.776518729	0.000159036	0.002851973	MPND	protein_coding
ENSG00000008394.13	7299.52975	-0.152460145	0.127214158	-1.198452648	0.230740847	0.4546685	MGST1	protein_coding
ENSG00000008405.12	1445.532169	0.080904137	0.049810523	1.624237831	0.10432506	0.282493825	CRY1	protein_coding
ENSG00000008438.5	13.21041846	0.202582921	0.129900158	1.559527902	0.118871486	0.305500279	PGLYRP1	protein_coding
ENSG00000008441.18	10044.43628	-0.067909731	0.090396524	-0.751242731	0.452506588	0.666944449	NFIX	protein_coding
ENSG00000008513.16	3799.73977	0.42313794	0.103746042	4.078593564	4.5309E-05	0.00106095	ST3GAL1	protein_coding
ENSG00000008516.17	228.5077339	0.076949944	0.111621911	0.689380282	0.490583988	0.697835105	MMP25	protein_coding
ENSG00000008517.18	10408.31534	-0.083306802	0.117674934	-0.707940077	0.47898247	0.688514189	IL32	protein_coding
ENSG00000008710.20	5099.109991	0.104552651	0.100007731	1.045445686	0.295817031	0.525523933	PKD1	protein_coding
ENSG00000008735.14	880.3070513	0.089568337	0.183659383	0.487687236	0.625771394	0.795903334	MAPK8IP2	protein_coding
ENSG00000008838.20	7494.584298	-0.250601645	0.091388656	-2.742152649	0.006103797	0.042823091	MED24	protein_coding
ENSG00000008853.17	1140.514571	0.160512674	0.074479877	2.155114661	0.031152863	0.13082281	RHOBTB2	protein_coding
ENSG00000008869.12	2389.383822	-0.04966057	0.047663319	-1.041903308	0.297456511	0.527392886	HEATR5B	protein_coding
ENSG00000008952.17	13972.35428	-0.067008859	0.064669808	-1.036169144	0.300123239	0.529740264	SEC62	protein_coding
ENSG00000008988.11	52787.25545	-0.298653767	0.070500603	-4.236187412	2.27347E-05	0.000615638	RPS20	protein_coding
ENSG00000009307.16	25361.30244	0.027015778	0.046986731	0.574966107	0.565314219	0.753746527	CSDE1	protein_coding
ENSG00000009335.18	6898.747837	-0.034153721	0.051576856	-0.662190829	0.507848931	0.711703305	UBE3C	protein_coding
ENSG00000009413.16	2349.797367	-0.146976319	0.069399679	-2.11782418	0.034189961	0.138646558	REV3L	protein_coding
ENSG00000009694.13	166.7313512	0.271761174	0.200266154	1.357000012	0.174781207	0.386154624	TENM1	protein_coding
ENSG00000009709.12	4.533603931	-0.487872688	0.293427667	-1.662667641	0.096378983	0.269086811	PAX7	protein_coding
ENSG00000009724.17	100.1507938	-0.278635674	0.112480395	-2.477193242	0.013242018	0.073518593	MASP2	protein_coding
ENSG00000009765.15	1707.727664	-0.068322671	0.17567319	-0.38891917	0.697335941	0.840491603	IYD	protein_coding
ENSG00000009780.16	792.326885	-0.165738096	0.045207224	-3.666186066	0.000246195	0.003988265	FAM76A	protein_coding
ENSG00000009790.15	395.8817372	-0.629457277	0.128685334	-4.891445354	1.00098E-06	5.19283E-05	TRAF3IP3	protein_coding
ENSG00000009830.12	1085.364917	0.179858271	0.052218957	3.444309912	0.000572519	0.007548763	POMT2	protein_coding
ENSG00000009844.16	3772.731033	0.156475975	0.053583385	2.920233129	0.003497696	0.028823584	VTA1	protein_coding
ENSG00000009950.16	1635.357847	-0.639876759	0.191907809	-3.334292447	0.000855167	0.01016797	MLXIPL	protein_coding
ENSG00000009954.11	8764.552545	-0.04266412	0.040842988	-1.0445886	0.296213151	0.525980216	BAZ1B	protein_coding
ENSG00000010017.13	3032.145865	0.12471881	0.051672841	2.413624011	0.015794752	0.082966237	RANBP9	protein_coding
ENSG00000010030.14	1142.285482	-0.154492155	0.115707406	-1.335196779	0.181811951	0.39519176	ETV7	protein_coding
ENSG00000010072.16	877.1373798	0.045066644	0.046126888	0.977014611	0.328561919	0.558043319	SPRTN	protein_coding
ENSG00000010165.20	2213.221444	0.096771674	0.048206662	2.007433627	0.044703511	0.165509338	EEF1AKNMT	protein_coding
ENSG00000010219.13	855.4015146	-0.013427049	0.069526132	-0.193122333	0.846863163	0.925031066	DYRK4	protein_coding
ENSG00000010244.19	9378.422204	0.064905967	0.035156648	1.846193256	0.06486415	0.209687319	ZNF207	protein_coding

ENSG00000010256.11	12294.97028	-0.067177396	0.06714905	-1.000422127	0.317106266	0.546532976	UQCRC1	protein_coding
ENSG00000010270.14	1609.159438	-0.016938694	0.059207227	-0.286091671	0.774807911	0.886035101	STARD3NL	protein_coding
ENSG00000010278.15	15320.00733	-0.0259866	0.088821419	-0.292571325	0.769849834	0.883509684	CD9	protein_coding
ENSG00000010282.15	1.782903953	0.426866136	0.293574602	1.454029517	0.145938123	0.346380479	HHATL	protein_coding
ENSG00000010292.13	8750.422412	0.040487597	0.070932339	0.570791791	0.568140788	0.755458047	NCAPD2	protein_coding
ENSG00000010295.20	493.4474168	-0.083919733	0.088912884	-0.943842209	0.345250288	0.574740107	IFFO1	protein_coding
ENSG00000010310.9	174.0292448	0.344546611	0.132566777	2.599041919	0.009348436	0.057628629	GIPR	protein_coding
ENSG00000010318.21	232.6018076	-0.031292659	0.067088881	-0.466435852	0.640903554	0.80560327	PHF7	protein_coding
ENSG00000010319.7	655.7626784	0.037741961	0.111720473	0.337824925	0.735495125	0.862811989	SEMA3G	protein_coding
ENSG00000010322.16	4516.821479	0.145996025	0.055057267	2.651712142	0.008008479	0.0518167	NISCH	protein_coding
ENSG00000010327.10	3959.283345	0.095555663	0.100128783	0.954327614	0.339917834	0.569759192	STAB1	protein_coding
ENSG00000010361.14	369.5098847	0.120931004	0.125015557	0.967327643	0.333380269	0.562936877	FUZ	protein_coding
ENSG00000010379.16	9.979930119	0.061337256	0.184284034	0.33284086	0.739254413	0.865166901	SLC6A13	protein_coding
ENSG00000010404.18	1051.670454	0.021438446	0.09467576	0.226440702	0.820858675	0.911886162	IDS	protein_coding
ENSG00000010438.17	3388.912756	-0.058396959	0.135438723	-0.431168853	0.666345599	0.821703044	PRSS3	protein_coding
ENSG00000010539.12	613.6653791	0.027281884	0.047538243	0.573893407	0.566039935	0.754275648	ZNF200	protein_coding
ENSG00000010610.10	3620.732032	-0.192526905	0.109228267	-1.762610631	0.077966183	0.236199866	CD4	protein_coding
ENSG00000010626.15	281.8221226	-0.012868657	0.073411195	-0.17529556	0.860847401	0.932371305	LRRC23	protein_coding
ENSG00000010671.16	453.5406895	-0.64701996	0.12931643	-5.00338556	5.63321E-07	3.26869E-05	BTK	protein_coding
ENSG00000010704.18	777.4119766	0.067870627	0.073765372	0.920087902	0.357526826	0.585294207	HFE	protein_coding
ENSG00000010803.16	2380.946441	-0.037365481	0.0617363	-0.605243287	0.545017358	0.739398568	SCMH1	protein_coding
ENSG00000010810.17	2563.737405	0.068805269	0.075749028	0.90833204	0.363702819	0.590987825	FYN	protein_coding
ENSG00000010818.10	3232.683641	0.180241132	0.071793021	2.510566191	0.012053772	0.068724952	HIVEP2	protein_coding
ENSG00000010932.17	229.9777654	0.251752025	0.150660802	1.670985564	0.094724529	0.266183026	FMO1	protein_coding
ENSG00000011007.12	3364.139	-0.009797675	0.043026801	-0.227710976	0.819870939	0.911299985	ELOA	protein_coding
ENSG00000011009.11	4602.712849	-0.068241468	0.064526444	-1.057573666	0.290249853	0.520053364	LYPLA2	protein_coding
ENSG00000011021.23	1255.478777	0.063124223	0.059369165	1.063249299	0.287668911	0.517343409	CLCN6	protein_coding
ENSG00000011028.14	5441.474026	0.595967358	0.106531653	5.59427496	2.21546E-08	2.30642E-06	MRC2	protein_coding
ENSG00000011052.21	0						NME1-NME2	protein_coding
ENSG00000011083.9	71.58556329	-0.339989424	0.208551817	-1.630239564	0.103050875	0.280472674	SLC6A7	protein_coding
ENSG00000011105.14	2026.637903	0.24956612	0.079990256	3.119956508	0.001808777	0.017776521	TSPAN9	protein_coding
ENSG00000011114.15	2814.699737	0.106235463	0.045139167	2.353509593	0.018597128	0.092429904	BTBD7	protein_coding
ENSG00000011132.12	1091.87894	-0.013573273	0.052259894	-0.259726379	0.795074843	0.897357587	APBA3	protein_coding
ENSG00000011143.19	680.7938621	-0.002722244	0.049629536	-0.054851294	0.956256935	0.979659781	MKS1	protein_coding
ENSG00000011198.10	1530.494682	0.161826957	0.057815354	2.799030783	0.005125625	0.037870357	ABHD5	protein_coding
ENSG00000011201.12	805.4675915	0.679471314	0.127557496	5.32678467	9.99666E-08	8.06712E-06	ANOS1	protein_coding
ENSG00000011243.18	1241.722991	0.102748217	0.041954266	2.44905287	0.014323242	0.077570473	AKAP8L	protein_coding
ENSG00000011258.16	1708.182952	-0.12810703	0.054568543	-2.347635149	0.018893018	0.093355373	MBTD1	protein_coding
ENSG00000011260.14	1840.498325	-0.088244212	0.048259644	-1.828530119	0.06747003	0.21489945	UTP18	protein_coding
ENSG00000011275.19	3795.589663	-0.019139661	0.047439178	-0.403456839	0.68661218	0.834570051	RNF216	protein_coding
ENSG00000011295.16	2805.91539	-0.042389232	0.060540144	-0.700183876	0.48381248	0.691838828	TTC19	protein_coding
ENSG00000011304.22	24965.50589	0.020118206	0.044434405	0.452761916	0.65072019	0.811807435	PTBP1	protein_coding

ENSG00000011332.19	81.43157682	0.002808799	0.119327119	0.023538645	0.981220613	0.991757913	DPF1	protein_coding
ENSG00000011347.10	1824.234783	0.425715034	0.203062334	2.096474637	0.036040108	0.143570139	SYT7	protein_coding
ENSG00000011376.12	1807.890344	0.024020792	0.060400002	0.397695222	0.69085486	0.837004621	LARS2	protein_coding
ENSG00000011405.13	6266.961374	-0.097931938	0.048630395	-2.01380101	0.04403043	0.163992652	PIK3C2A	protein_coding
ENSG00000011422.12	5368.475108	0.486618431	0.101053543	4.815451445	1.46867E-06	7.03511E-05	PLAUR	protein_coding
ENSG00000011426.11	4277.893104	0.120624652	0.091038949	1.324978528	0.185178273	0.399698529	ANLN	protein_coding
ENSG00000011451.21	5901.434418	0.08767057	0.053469648	1.639632452	0.101081611	0.277242786	WIZ	protein_coding
ENSG00000011454.18	3712.937875	-0.003877814	0.058458305	-0.0663347	0.947111358	0.975196627	RABGAP1	protein_coding
ENSG00000011465.18	22772.68699	0.356463312	0.131375432	2.713317897	0.006661317	0.045435374	DCN	protein_coding
ENSG00000011478.13	708.1269704	-0.04907251	0.069161497	-0.709535113	0.47799247	0.687645765	QPCTL	protein_coding
ENSG00000011485.15	3776.921513	0.000395418	0.054979146	0.007192142	0.994261551	0.997443151	PPP5C	protein_coding
ENSG00000011523.14	1638.984991	-0.124707004	0.066667255	-1.870588564	0.061402131	0.202798292	CEP68	protein_coding
ENSG00000011566.15	3053.190382	0.074775525	0.061196933	1.221883536	0.221751672	0.444280032	MAP4K3	protein_coding
ENSG00000011590.14	71.05644767	-0.348435163	0.113213359	-3.07768595	0.002086146	0.019758314	ZBTB32	protein_coding
ENSG00000011600.12	4321.329144	-0.072430019	0.120125969	-0.602950549	0.546541587	0.740383074	TYROBP	protein_coding
ENSG00000011638.11	1441.346147	-0.069817585	0.077913291	-0.896093383	0.370202891	0.596807437	TMEM159	protein_coding
ENSG00000011677.13	215.7256719	0.666418577	0.326655136	2.040128881	0.041337491	0.157237346	GABRA3	protein_coding
ENSG00000012048.23	1865.069583	-0.031842566	0.079166031	-0.402225119	0.687518357	0.835028497	BRCA1	protein_coding
ENSG00000012061.16	2190.664988	-0.003320739	0.053257769	-0.06235219	0.950282368	0.976776854	ERCC1	protein_coding
ENSG00000012124.17	713.4976218	-1.113527259	0.200860998	-5.543770425	2.96027E-08	2.96551E-06	CD22	protein_coding
ENSG00000012171.20	3417.932612	0.298028493	0.125090017	2.382512203	0.017194962	0.087786981	SEMA3B	protein_coding
ENSG00000012174.12	1752.053938	-0.040188654	0.060346332	-0.665966806	0.505432318	0.709506945	MBTPS2	protein_coding
ENSG00000012211.13	1343.374314	0.127127772	0.062184218	2.04437355	0.040916658	0.156159154	PRICKLE3	protein_coding
ENSG00000012223.13	6605.419579	-0.805858365	0.261169339	-3.08557799	0.002031569	0.019350754	LTF	protein_coding
ENSG00000012232.9	3494.265359	0.162751076	0.066420218	2.450324324	0.014272759	0.077405505	EXTL3	protein_coding
ENSG00000012504.15	139.0951347	-0.260388699	0.253691871	-1.026397487	0.304704262	0.534179412	NR1H4	protein_coding
ENSG00000012660.14	3125.032131	0.35278407	0.102531033	3.440754083	0.000580096	0.007615988	ELOVL5	protein_coding
ENSG00000012779.11	1846.747161	0.39452387	0.111831193	3.527851757	0.000418947	0.005950391	ALOX5	protein_coding
ENSG00000012817.16	1032.77715	-0.23455616	0.279673481	-0.838678587	0.401649697	0.623695175	KDM5D	protein_coding
ENSG00000012822.16	3765.020974	0.063983498	0.070755819	0.904286021	0.365843757	0.593096666	CALCOCO1	protein_coding
ENSG00000012963.15	1975.566239	0.025762507	0.044064667	0.584652255	0.558781564	0.748839813	UBR7	protein_coding
ENSG00000012983.11	2318.614153	0.131825106	0.046618851	2.827721064	0.004688064	0.03555992	MAP4K5	protein_coding
ENSG00000013016.16	917.2504138	0.228376592	0.104339283	2.188788195	0.028612238	0.123546919	EHD3	protein_coding
ENSG00000013275.8	6786.782206	-0.031956559	0.063932516	-0.49984829	0.617181906	0.79005158	PSMC4	protein_coding
ENSG00000013288.9	5022.477999	0.196458096	0.05997117	3.275875684	0.001053349	0.011934944	MAN2B2	protein_coding
ENSG00000013293.6	24.13844472	-0.597295228	0.233779266	-2.554953814	0.010620189	0.062939749	SLC7A14	protein_coding
ENSG00000013297.12	225.1414524	0.414269762	0.165869982	2.49755716	0.01250523	0.070586954	CLDN11	protein_coding
ENSG00000013306.16	11224.87437	0.049750076	0.065521396	0.759295117	0.447676038	0.662896418	SLC25A39	protein_coding
ENSG00000013364.19	16103.1473	0.043946092	0.068521983	0.641342962	0.521299882	0.721148772	MVP	protein_coding
ENSG00000013374.16	4832.738969	-0.161800466	0.055904238	-2.894243279	0.003800736	0.030574978	NUB1	protein_coding
ENSG00000013375.16	2657.413626	0.325724796	0.064654744	5.037910238	4.70642E-07	2.80948E-05	PGM3	protein_coding
ENSG00000013392.8	456.0126769	0.122558895	0.054872357	2.233527098	0.025514205	0.114492074	RWDD2A	protein_coding

ENSG00000013441.16	3654.966601	-0.034202635	0.067220023	-0.50881618	0.610881077	0.78619479	CLK1	protein_coding
ENSG00000013503.10	880.8125172	-0.069803301	0.057321467	-1.217751487	0.223318425	0.445894884	POLR3B	protein_coding
ENSG00000013523.11	2057.652306	-0.10429938	0.054869059	-1.900877877	0.05731801	0.194281262	ANGEL1	protein_coding
ENSG00000013561.18	1863.920468	-0.021349373	0.042902397	-0.497626573	0.61874727	0.791175604	RNF14	protein_coding
ENSG00000013563.14	647.9189124	0.141254759	0.069768634	2.024616956	0.042906712	0.161282307	DNASE1L1	protein_coding
ENSG00000013573.17	1947.481489	0.185008445	0.080444717	2.299820936	0.021458367	0.101917303	DDX11	protein_coding
ENSG00000013583.10	1863.253381	-0.155642486	0.07240472	-2.149617966	0.031585446	0.131954271	HEBP1	protein_coding
ENSG00000013588.9	40001.54553	0.482170634	0.126009431	3.826464662	0.000129997	0.002420885	GPRC5A	protein_coding
ENSG00000013619.14	466.5630848	0.557402268	0.109919346	5.071011507	3.95707E-07	2.46691E-05	MAMLD1	protein_coding
ENSG00000013725.14	851.7839573	-0.440792384	0.121704418	-3.621827314	0.000292529	0.004560003	CD6	protein_coding
ENSG00000013810.20	4007.53261	0.023582079	0.082459963	0.285982165	0.774891782	0.886049147	TACC3	protein_coding
ENSG00000014123.10	3579.684574	-0.01922323	0.046173898	-0.416322453	0.677174065	0.828988116	UFL1	protein_coding
ENSG00000014138.9	296.7234588	-0.181100494	0.068388828	-2.648100558	0.008094544	0.052171764	POLA2	protein_coding
ENSG00000014164.7	3484.201666	0.025351918	0.060781157	0.417101612	0.676604086	0.828655076	ZC3H3	protein_coding
ENSG00000014216.16	18353.94018	0.107562574	0.061030417	1.762442072	0.077994636	0.236239121	CAPN1	protein_coding
ENSG00000014257.16	583.1036885	-0.357060586	0.152274115	-2.344854125	0.019034525	0.093831166	ACP3	protein_coding
ENSG00000014641.21	5849.314309	-0.107111228	0.046446301	-2.306130428	0.021103346	0.100725631	MDH1	protein_coding
ENSG00000014824.14	3876.058416	0.053096958	0.048716707	1.089912703	0.275751601	0.504489168	SLC30A9	protein_coding
ENSG00000014914.21	3756.222726	0.172075205	0.106072104	1.622247499	0.104750365	0.283293123	MTMR11	protein_coding
ENSG00000014919.13	1915.847302	-0.162890855	0.050481983	-3.226712669	0.001252211	0.013617977	COX15	protein_coding
ENSG00000015133.19	4391.02054	-0.107938863	0.067980691	-1.587787077	0.112334495	0.295230856	CCDC88C	protein_coding
ENSG00000015153.15	1032.865629	-0.12861061	0.047323253	-2.717704323	0.006573655	0.045062164	YAF2	protein_coding
ENSG00000015171.20	5013.986653	-0.141019143	0.05138988	-2.744103375	0.006067643	0.042654814	ZMYND11	protein_coding
ENSG00000015285.11	1067.889325	-0.490564244	0.120855663	-4.059091914	4.92639E-05	0.001136461	WAS	protein_coding
ENSG00000015413.9	1906.196156	-0.733128627	0.264314664	-2.77369638	0.005542338	0.039999744	DPEP1	protein_coding
ENSG00000015475.19	2441.858126	0.107888788	0.064459702	1.673740104	0.094181688	0.265160579	BID	protein_coding
ENSG00000015479.20	232.0745613	-0.044349595	0.081701104	-0.542827363	0.587248672	0.769296575	MATR3	protein_coding
ENSG00000015520.15	1597.489991	-0.048456066	0.226811064	-0.213640664	0.830827286	0.916687349	NPC1L1	protein_coding
ENSG00000015532.10	2462.120856	-0.038292466	0.068683169	-0.557523282	0.577169956	0.761822537	XYLT2	protein_coding
ENSG00000015568.13	11.2499476	0.108463586	0.09514574	1.139973126	0.254297497	0.481078399	RGPD5	protein_coding
ENSG00000015592.16	6.284882235	-0.32173189	0.230903374	-1.393361582	0.163510477	0.370703712	STMN4	protein_coding
ENSG00000015676.18	5121.690074	-0.046827652	0.043910714	-1.06642885	0.286229826	0.515653592	NUDCD3	protein_coding
ENSG00000016082.15	302.3111958	0.07769544	0.147615707	0.526335856	0.598654874	0.777647604	ISL1	protein_coding
ENSG00000016391.11	2853.708693	-0.196280771	0.093590601	-2.097227373	0.035973452	0.143410039	CHDH	protein_coding
ENSG00000016402.13	1036.62965	0.112419343	0.122301303	0.919199878	0.357991035	0.585630456	IL20RA	protein_coding
ENSG00000016490.16	306.9850525	-0.239361378	0.342538618	-0.698786546	0.484685438	0.692591386	CLCA1	protein_coding
ENSG00000016602.9	125.8607825	1.492917423	0.289411477	5.158459631	2.4899E-07	1.6904E-05	CLCA4	protein_coding
ENSG00000016864.18	2210.668288	0.117094917	0.044414698	2.636400159	0.008379085	0.053366765	GLT8D1	protein_coding
ENSG00000017260.20	7179.562856	-0.113510178	0.056965191	-1.992623473	0.046302693	0.169329678	ATP2C1	protein_coding
ENSG00000017427.17	243.8232077	0.401754301	0.18983456	2.1163391	0.034315978	0.138989437	IGF1	protein_coding
ENSG00000017483.15	1545.093314	0.341785996	0.180630349	1.892184778	0.058466366	0.196527987	SLC38A5	protein_coding
ENSG00000017797.13	6547.659994	0.132513616	0.052029931	2.546872811	0.010869304	0.064015632	RALBP1	protein_coding

ENSG00000018189.13	1668.64757	0.03943846	0.049707655	0.793408177	0.427540048	0.646044667	RUFY3	protein_coding
ENSG00000018236.15	443.6972409	0.267349308	0.2117319	1.262678451	0.206704761	0.426555542	CNTN1	protein_coding
ENSG00000018280.17	870.0662756	0.693289574	0.124280376	5.578431564	2.42697E-08	2.48847E-06	SLC11A1	protein_coding
ENSG00000018408.15	5986.563692	0.231456914	0.109281785	2.117982559	0.034176545	0.138617953	WWTR1	protein_coding
ENSG00000018510.17	3994.153467	0.033566668	0.05098752	0.658331069	0.510325418	0.713571338	AGPS	protein_coding
ENSG00000018607.7	6.717206217	-0.389423923	0.213662776	-1.822610046	0.068362473	0.216646672	ZNF806	transcribed_unprocessed_pseudogene
ENSG00000018610.15	960.4681433	-0.019090001	0.046199497	-0.413207977	0.679454244	0.830185829	CXorf56	protein_coding
ENSG00000018625.15	565.6682487	-0.634598212	0.239778984	-2.646596463	0.008130631	0.052335084	ATP1A2	protein_coding
ENSG00000018699.13	1076.053237	-0.146910671	0.047325371	-3.104268783	0.0019075	0.018484125	TTC27	protein_coding
ENSG00000018869.17	45.64487051	-0.262776392	0.123850643	-2.12172004	0.033861255	0.137865262	ZNF582	protein_coding
ENSG00000019102.12	3341.438472	-0.182670445	0.200623549	-0.910513474	0.362551779	0.590034623	VSIG2	protein_coding
ENSG00000019144.19	3262.113955	0.299152034	0.088345532	3.386159169	0.000708783	0.00883326	PHLDB1	protein_coding
ENSG00000019169.10	242.0106117	0.72870069	0.206561	3.527774805	0.000419069	0.005950391	MARCO	protein_coding
ENSG00000019186.10	106.9388476	1.363616279	0.258450191	5.27612795	1.31942E-07	1.01127E-05	CYP24A1	protein_coding
ENSG00000019485.13	979.215152	-0.050844265	0.083535958	-0.608651245	0.54275563	0.737703809	PRDM11	protein_coding
ENSG00000019505.8	3728.478466	-0.395245767	0.188365075	-2.098296443	0.035878967	0.143151229	SYT13	protein_coding
ENSG00000019549.13	781.3887118	0.558629264	0.108479068	5.149650299	2.60973E-07	1.75771E-05	SNAI2	protein_coding
ENSG00000019582.15	187552.3264	-0.391314748	0.120332302	-3.251950991	0.001146158	0.012741357	CD74	protein_coding
ENSG00000019991.18	572.9293666	0.002530512	0.131474672	0.019247142	0.98464395	0.993017503	HGF	protein_coding
ENSG00000019995.6	890.9879651	0.047367936	0.062851496	0.753648506	0.45106031	0.665736771	ZRANB1	protein_coding
ENSG00000020129.16	2073.894594	0.151717531	0.050994379	2.975181464	0.002928153	0.025187547	NCDN	protein_coding
ENSG00000020181.18	4228.58188	0.396840586	0.108099683	3.671061509	0.000241545	0.003930476	ADGRA2	protein_coding
ENSG00000020219.9	3.849789538	-0.296343436	0.229104876	-1.293483761	0.195843797	0.412882687	CCT8L1P	processed_pseudogene
ENSG00000020256.20	2065.278538	0.073544131	0.051265976	1.434560244	0.15141245	0.353823485	ZFP64	protein_coding
ENSG00000020426.11	593.615841	-0.018492447	0.058143436	-0.318048755	0.750447954	0.871851302	MNAT1	protein_coding
ENSG00000020577.14	1626.116003	0.05404847	0.113287128	0.477092775	0.633296059	0.8010549	SAMD4A	protein_coding
ENSG00000020633.19	1856.50619	0.095954541	0.112683447	0.851540692	0.394469069	0.617937257	RUNX3	protein_coding
ENSG00000020922.13	1812.023059	-0.145264721	0.063085765	-2.302654494	0.02129829	0.101355859	MRE11	protein_coding
ENSG00000021300.14	3306.242765	0.083874765	0.154116425	0.544229887	0.586283288	0.768548287	PLEKHB1	protein_coding
ENSG00000021355.13	15065.89597	0.007389893	0.092477442	0.079910223	0.936308659	0.969825353	SERPINB1	protein_coding
ENSG00000021461.17	7.30672517	-0.386602834	0.188775212	-2.04795338	0.040564568	0.155327866	CYP3A43	protein_coding
ENSG00000021488.13	285.2847042	-0.677590615	0.22443689	-3.019069706	0.002535522	0.022674007	SLC7A9	protein_coding
ENSG00000021574.13	1875.961916	-0.032454781	0.04631264	-0.700775882	0.483442891	0.691514041	SPAST	protein_coding
ENSG00000021645.20	843.9422123	-0.415303157	0.195663725	-2.122535265	0.033792815	0.137638048	NRXN3	protein_coding
ENSG00000021762.20	1957.979348	0.430477678	0.085359355	5.043122427	4.57996E-07	2.75039E-05	OSBPL5	protein_coding
ENSG00000021776.11	4208.522684	-0.032613259	0.045781995	-0.712359931	0.476241921	0.686282877	AQR	protein_coding
ENSG00000021826.17	9866.256284	-0.042628151	0.264763911	-0.161004386	0.872089946	0.938226491	CPS1	protein_coding
ENSG00000021852.13	6.970686553	-1.04279478	0.288486644	-3.614707303	0.000300687	0.004651418	C8B	protein_coding
ENSG00000022267.19	7858.452915	-0.543720452	0.192831212	-2.819670348	0.004807301	0.036212229	FHL1	protein_coding
ENSG00000022277.13	3727.753576	-0.059792765	0.052864658	-1.131053669	0.258032503	0.485121456	RTF2	protein_coding
ENSG00000022355.18	6.177823049	-0.437217553	0.305728166	-1.430085945	0.152692353	0.355917341	GABRA1	protein_coding
ENSG00000022556.16	1949.703841	-0.427299449	0.242262169	-1.763789412	0.077767441	0.235731203	NLRP2	protein_coding

ENSG00000022567.10	3220.81255	0.244660086	0.074017651	3.305428942	0.000948311	0.011002281	SLC45A4	protein_coding
ENSG00000022840.16	8820.13215	-0.037397132	0.036389027	-1.027703548	0.304089298	0.533577301	RNF10	protein_coding
ENSG00000022976.15	279.3501493	-0.062075003	0.055649063	-1.11547257	0.264647967	0.492444786	ZNF839	protein_coding
ENSG00000023041.11	2497.269697	-0.100166322	0.046422422	-2.157714293	0.030950052	0.130222112	ZDHHC6	protein_coding
ENSG00000023171.18	1911.08308	0.564934707	0.146893199	3.845887436	0.000120117	0.002283741	GRAMD1B	protein_coding
ENSG00000023191.17	6781.58187	0.016487611	0.051961647	0.317303466	0.751013348	0.872229116	RNH1	protein_coding
ENSG00000023228.14	7212.871621	0.004934879	0.04813848	0.102514224	0.918348523	0.961414381	NDUFS1	protein_coding
ENSG00000023287.13	4391.307833	0.070870998	0.056425645	1.256006875	0.209113478	0.429128232	RB1CC1	protein_coding
ENSG00000023318.8	6759.396565	-0.077088921	0.049864261	-1.545975412	0.122110506	0.310800159	ERP44	protein_coding
ENSG00000023330.15	4750.608703	-0.053114961	0.067461557	-0.787336726	0.431084776	0.649119242	ALAS1	protein_coding
ENSG00000023445.16	7539.321282	-0.295801366	0.123787856	-2.389583071	0.016867509	0.086796722	BIRC3	protein_coding
ENSG00000023516.9	5276.990121	0.018512952	0.068299178	0.271056734	0.786347398	0.89198929	AKAP11	protein_coding
ENSG00000023572.10	1158.694847	0.036382971	0.046910219	0.775587333	0.437992682	0.654832645	GLRX2	protein_coding
ENSG00000023608.5	287.80645	0.10767079	0.057661242	1.867299184	0.061859819	0.203738595	SNAPC1	protein_coding
ENSG00000023697.13	1927.690967	-0.150236715	0.066683734	-2.252973941	0.024260792	0.110792759	DERA	protein_coding
ENSG00000023734.11	6122.641193	-0.080955077	0.059514727	-1.360252831	0.173749929	0.384906749	STRAP	protein_coding
ENSG00000023839.12	541.4650559	-0.450687869	0.206436837	-2.183175612	0.02902288	0.124813075	ABCC2	protein_coding
ENSG00000023892.11	1262.455851	-0.242233371	0.08442625	-2.869171284	0.004115489	0.032377803	DEF6	protein_coding
ENSG00000023902.14	2625.60084	-0.248843172	0.109497526	-2.272591727	0.023050795	0.107053659	PLEKHO1	protein_coding
ENSG00000023909.10	1919.641962	-0.080837149	0.063081502	-1.281471542	0.20002809	0.418387723	GCLM	protein_coding
ENSG00000024048.11	4495.111062	0.192783108	0.061760614	3.121457098	0.001799585	0.017718143	UBR2	protein_coding
ENSG00000024422.12	9629.942436	0.302298883	0.107898156	2.801705732	0.005083322	0.037640767	EHD2	protein_coding
ENSG00000024526.17	1177.056501	0.156183772	0.097369112	1.604038179	0.108705637	0.289182643	DEPDC1	protein_coding
ENSG00000024862.18	989.6847301	0.01367491	0.051000823	0.268131159	0.788598362	0.893633101	CCDC28A	protein_coding
ENSG00000025039.15	769.6379739	0.194134177	0.12071514	1.608200732	0.107791223	0.287731585	RRAGD	protein_coding
ENSG00000025156.13	684.4379119	-0.091745919	0.055119282	-1.664497734	0.096013004	0.268409043	HSF2	protein_coding
ENSG00000025293.17	3546.949647	-0.078660085	0.043190615	-1.821230969	0.068571755	0.217020216	PHF20	protein_coding
ENSG00000025423.11	130.1302139	0.039425413	0.117727274	0.334887677	0.737709818	0.864217989	HSD17B6	protein_coding
ENSG00000025434.19	1605.836724	-0.198355976	0.07331128	-2.705667889	0.006816718	0.046183477	NR1H3	protein_coding
ENSG00000025708.14	5609.167579	0.122583877	0.114721395	1.068535448	0.285279049	0.514577747	TYMP	protein_coding
ENSG00000025770.19	2248.322839	-0.077256831	0.065491422	-1.179648102	0.238140203	0.463567723	NCAPH2	protein_coding
ENSG00000025772.8	3914.99163	-0.095210943	0.075358493	-1.26344011	0.206431057	0.426176776	TOMM34	protein_coding
ENSG00000025796.14	5907.246177	0.083540693	0.059632738	1.400919963	0.161238008	0.367655732	SEC63	protein_coding
ENSG00000025800.14	6246.093031	-0.017770027	0.037414136	-0.47495488	0.634819133	0.802004777	KPNA6	protein_coding
ENSG00000026025.16	18484.32943	0.069520254	0.092559269	0.751089061	0.452599059	0.66701732	VIM	protein_coding
ENSG00000026036.22	188.8905343	0.287661619	0.09695546	2.966946066	0.003007737	0.025719812	RTEL1-TNFRSF6B	protein_coding
ENSG00000026103.22	1299.604169	-0.169618945	0.109909598	-1.543258719	0.122768015	0.311763509	FAS	protein_coding
ENSG00000026297.16	2903.575091	-0.017854417	0.079079322	-0.225778582	0.821373636	0.912155936	RNASET2	protein_coding
ENSG00000026508.20	25742.01667	-0.104217655	0.117325742	-0.888276124	0.374392239	0.600112208	CD44	protein_coding
ENSG00000026559.14	142.0582989	0.290965694	0.185089029	1.572031011	0.11594336	0.300924775	KCNG1	protein_coding
ENSG00000026652.15	570.1268093	0.429858548	0.093572093	4.593875507	4.35089E-06	0.000167638	AGPAT4	protein_coding
ENSG00000026751.17	1867.657036	-0.709410425	0.141044789	-5.029681911	4.91294E-07	2.90097E-05	SLAMF7	protein_coding

ENSG00000026950.17	3185.124185	-0.065543959	0.082374426	-0.795683349	0.426216107	0.644858666	BTN3A1	protein_coding
ENSG00000027001.10	1483.949109	0.001963375	0.069731742	0.028156121	0.977537634	0.989940824	MIPEP	protein_coding
ENSG00000027075.15	1359.578117	-0.031167582	0.078690689	-0.396077122	0.692048135	0.837635241	PRKCH	protein_coding
ENSG00000027644.5	15.05102215	-0.461241434	0.167559408	-2.752703882	0.005910533	0.041880211	INSRR	protein_coding
ENSG00000027697.15	6897.977386	-0.019889079	0.071601273	-0.277775497	0.781184702	0.889172516	IFNGR1	protein_coding
ENSG00000027847.14	1688.852524	0.02294989	0.05501324	0.4171703	0.676553847	0.828637227	B4GALT7	protein_coding
ENSG00000027869.12	669.7244011	-0.07999398	0.084986792	-0.941251914	0.346575779	0.575921707	SH2D2A	protein_coding
ENSG00000028116.18	996.4682065	-0.126072085	0.064073218	-1.967625311	0.049111165	0.175917994	VRK2	protein_coding
ENSG00000028137.19	4424.91254	-0.145309683	0.084723961	-1.715095492	0.086327721	0.251817597	TNFRSF1B	protein_coding
ENSG00000028203.18	3784.460393	-0.02856334	0.048061413	-0.594309201	0.552305326	0.744146866	VEZT	protein_coding
ENSG00000028277.21	1614.163079	-0.541391536	0.117008742	-4.626932385	3.71122E-06	0.000147155	POU2F2	protein_coding
ENSG00000028310.18	2219.169471	-0.01388806	0.055615757	-0.249714477	0.802808162	0.90204397	BRD9	protein_coding
ENSG00000028528.15	5178.241553	-0.020577121	0.040568995	-0.507212999	0.612005375	0.787008535	SNX1	protein_coding
ENSG00000028839.10	695.5922245	-0.020801364	0.053213029	-0.390907353	0.695865719	0.839507242	TBPL1	protein_coding
ENSG00000029153.14	3014.425313	0.187371243	0.097026544	1.931133841	0.053466503	0.185579167	ARNTL2	protein_coding
ENSG00000029363.17	9164.982665	-0.01858163	0.040035929	-0.464123851	0.642559014	0.806683592	BCLAF1	protein_coding
ENSG00000029364.12	6104.034038	0.111893224	0.047197149	2.370762331	0.017751442	0.089635255	SLC39A9	protein_coding
ENSG00000029534.21	714.6529092	0.374719652	0.179164797	2.091480346	0.036485026	0.144734456	ANK1	protein_coding
ENSG00000029559.7	77.94682094	0.827519537	0.224217153	3.690705758	0.000223633	0.003699788	IBSP	protein_coding
ENSG00000029639.11	631.4560087	-0.159850976	0.055385364	-2.886159146	0.003899749	0.031192982	TFB1M	protein_coding
ENSG00000029725.17	2202.212309	-0.079285355	0.053251302	-1.488890451	0.136516218	0.33247672	RABEP1	protein_coding
ENSG00000029993.15	3053.826493	0.081262791	0.093027374	0.873536333	0.382370811	0.60763715	HMGB3	protein_coding
ENSG00000030066.13	4172.247467	0.126759053	0.051677407	2.452891132	0.014171322	0.077038885	NUP160	protein_coding
ENSG00000030110.13	2530.272962	0.051157847	0.074454006	0.687106709	0.492015487	0.698981511	BAK1	protein_coding
ENSG00000030304.14	165.4814751	-0.195031717	0.197695434	-0.986526154	0.323874988	0.553606205	MUSK	protein_coding
ENSG00000030419.16	719.3077421	0.216419468	0.113102176	1.913486339	0.055685821	0.190562354	IKZF2	protein_coding
ENSG00000030582.18	34560.22144	0.281584635	0.063167556	4.457741465	8.28277E-06	0.000278287	GRN	protein_coding
ENSG00000031003.10	1534.114283	-0.114184685	0.069367936	-1.646072983	0.099748734	0.275006364	FAM13B	protein_coding
ENSG00000031081.11	2144.995729	0.076272706	0.091693488	0.831822495	0.405509145	0.627004648	ARHGAP31	protein_coding
ENSG00000031691.7	392.8421779	0.185914606	0.072256766	2.572971581	0.010082949	0.060831324	CENPQ	protein_coding
ENSG00000031698.13	6206.803293	-0.136023357	0.048097917	-2.828050899	0.004683236	0.035529487	SARS1	protein_coding
ENSG00000031823.14	3711.81023	-0.01655397	0.044900764	-0.368679017	0.712366991	0.849673364	RANBP3	protein_coding
ENSG00000032219.19	2414.850806	-0.142460726	0.055010463	-2.589702376	0.009605894	0.058709124	ARID4A	protein_coding
ENSG00000032389.13	1748.298576	-0.031134549	0.057548549	-0.541013618	0.588498196	0.770310791	EIPR1	protein_coding
ENSG00000032444.17	4018.048952	0.13051546	0.056921604	2.292898487	0.02185385	0.103078735	PNPLA6	protein_coding
ENSG00000032742.18	826.8476957	-0.096579399	0.063143036	-1.52953365	0.126132204	0.316744254	IFT88	protein_coding
ENSG00000033011.13	1416.112388	-0.027458913	0.061206535	-0.448627139	0.653700653	0.813908893	ALG1	protein_coding
ENSG00000033030.15	1566.200013	-0.088443339	0.040129625	-2.203941325	0.027528471	0.120430496	ZCCHC8	protein_coding
ENSG00000033050.9	975.3378262	-0.233298313	0.057087155	-4.086704127	4.37545E-05	0.001038579	ABCF2	protein_coding
ENSG00000033100.16	4115.109843	0.134601782	0.061822947	2.177213943	0.029464608	0.126052231	CHPF2	protein_coding
ENSG00000033122.21	91.0771268	-0.487291246	0.145137679	-3.357441367	0.000786674	0.009565363	LRRC7	protein_coding
ENSG00000033170.17	3936.626259	-0.125372939	0.071336504	-1.757486451	0.078834929	0.238039753	FUT8	protein_coding

ENSG00000033178.13	3363.340441	0.101168683	0.064146652	1.577146739	0.114761765	0.299017689	UBA6	protein_coding
ENSG00000033327.13	2394.506887	-0.038747133	0.076704679	-0.505146927	0.613455644	0.787757672	GAB2	protein_coding
ENSG00000033627.17	4474.208886	-0.047219324	0.053845337	-0.876943609	0.380517268	0.606059978	ATP6V0A1	protein_coding
ENSG00000033800.14	4307.817978	-0.0079343	0.041564754	-0.190890101	0.848611696	0.925761934	PIAS1	protein_coding
ENSG00000033867.16	2109.35669	-0.067815029	0.074105278	-0.915117393	0.360130005	0.588031521	SLC4A7	protein_coding
ENSG00000034053.14	393.2294696	0.389579859	0.108657146	3.585404854	0.000336556	0.005062097	APBA2	protein_coding
ENSG00000034152.19	4981.415006	0.053282198	0.0716987	0.743140363	0.457396705	0.670998953	MAP2K3	protein_coding
ENSG00000034239.11	14.27581241	0.227747862	0.175917849	1.294626229	0.1954492	0.412509797	EFCAB1	protein_coding
ENSG00000034510.6	53957.25985	-0.018739132	0.083531086	-0.224337228	0.822494911	0.912517458	TMSB10	protein_coding
ENSG00000034533.12	547.162502	-0.213821101	0.048810461	-4.380640869	1.18331E-05	0.000370971	ASTE1	protein_coding
ENSG00000034677.13	4024.214729	0.024726949	0.067640725	0.365563037	0.714691161	0.850914722	RNF19A	protein_coding
ENSG00000034693.15	828.5971245	-0.14416584	0.055860258	-2.580830194	0.009856305	0.059810673	PEX3	protein_coding
ENSG00000034713.8	3048.892936	-0.007719844	0.044889232	-0.171975417	0.863456856	0.933553142	GABARAPL2	protein_coding
ENSG00000034971.17	157.8325798	-0.224834025	0.308508319	-0.728777834	0.466137573	0.677751059	MYOC	protein_coding
ENSG00000035115.22	3367.934792	-0.165804997	0.078466868	-2.113057421	0.034595853	0.139720436	SH3YL1	protein_coding
ENSG00000035141.8	3329.419477	-0.011943972	0.056863987	-0.210044568	0.833632889	0.918304821	FAM136A	protein_coding
ENSG00000035403.18	18579.14284	0.003942828	0.086688675	0.045482622	0.963722626	0.983375587	VCL	protein_coding
ENSG00000035499.13	1077.709151	-0.323196046	0.096497195	-3.349279197	0.000810221	0.009777625	DEPDC1B	protein_coding
ENSG00000035664.11	651.2909345	-0.504441295	0.102453262	-4.923623583	8.49562E-07	4.59322E-05	DAPK2	protein_coding
ENSG00000035681.9	2232.721595	-0.152771703	0.05108343	-2.990631281	0.002784014	0.024244403	NSMAF	protein_coding
ENSG00000035687.10	2815.414638	0.100214372	0.043602432	2.298366561	0.021540935	0.102172748	ADSS2	protein_coding
ENSG00000035720.8	110.9007705	-1.361535285	0.177548571	-7.668522933	1.73988E-14	1.43054E-11	STAP1	protein_coding
ENSG00000035862.12	15939.80252	0.537894901	0.101859766	5.280739617	1.28663E-07	9.90595E-06	TIMP2	protein_coding
ENSG00000035928.16	2934.772563	-0.017631814	0.045770457	-0.385222576	0.700072518	0.842148481	RFC1	protein_coding
ENSG00000036054.13	2585.122841	0.095241548	0.042298385	2.251659232	0.024343815	0.110977133	TBC1D23	protein_coding
ENSG00000036257.13	3657.332792	0.070692944	0.044864479	1.575699631	0.115095043	0.299414761	CUL3	protein_coding
ENSG00000036448.10	99.71819946	-0.077871685	0.114031315	-0.682897376	0.494671692	0.701314648	MYOM2	protein_coding
ENSG00000036473.8	307.3434568	-1.038715895	0.286947926	-3.619875943	0.000294744	0.004587169	OTC	protein_coding
ENSG00000036530.9	53.75442451	0.029766197	0.119681915	0.248710904	0.803584414	0.902413155	CYP46A1	protein_coding
ENSG00000036549.13	2530.320563	0.048830139	0.042395192	1.151784835	0.249409501	0.476146405	ZZZ3	protein_coding
ENSG00000036565.15	41.4898025	-0.489834778	0.219462217	-2.231977715	0.025616436	0.114820759	SLC18A1	protein_coding
ENSG00000036672.16	251.1567274	-0.169702319	0.140282913	-1.209714819	0.226388342	0.449613888	USP2	protein_coding
ENSG00000036828.17	17.08106265	-0.166001659	0.212230853	-0.782174962	0.434111757	0.651825789	CASR	protein_coding
ENSG00000037042.9	677.9531124	0.088853504	0.076469048	1.161953832	0.245254219	0.471664376	TUBG2	protein_coding
ENSG00000037241.7	837.0042633	-0.114041117	0.063554736	-1.79437638	0.072753114	0.225451509	RPL26L1	protein_coding
ENSG00000037280.16	985.2639282	0.092359167	0.087802172	1.051900716	0.292845103	0.52272605	FLT4	protein_coding
ENSG00000037474.15	4318.921099	0.040109087	0.059439862	0.674784326	0.4998128	0.705093635	NSUN2	protein_coding
ENSG00000037637.11	2175.662468	-0.033073924	0.041249742	-0.801797117	0.422670327	0.641945658	FBXO42	protein_coding
ENSG00000037749.12	1074.238642	0.221949512	0.066608974	3.332126279	0.000861851	0.010239067	MFAP3	protein_coding
ENSG00000037757.14	875.3311834	0.026959918	0.07146541	0.377244298	0.705992063	0.845913171	MRI1	protein_coding
ENSG00000037897.17	749.6569943	-0.000840741	0.073333099	-0.011464689	0.990852702	0.995880439	METTL1	protein_coding
ENSG00000037965.6	214.6222841	-0.09807539	0.169339301	-0.579164964	0.562477869	0.751274999	HOXC8	protein_coding

ENSG00000038002.9	896.1340506	-0.0654692	0.068067556	-0.961826808	0.336136609	0.565684136	AGA	protein_coding
ENSG00000038210.14	2356.150428	-0.075077159	0.061825446	-1.214340758	0.224617628	0.447544117	PI4K2B	protein_coding
ENSG00000038219.13	4882.180809	0.020831697	0.052698273	0.395301328	0.69262052	0.837699816	BOD1L1	protein_coding
ENSG00000038274.17	4106.301222	-0.019442348	0.051778242	-0.375492632	0.707294125	0.846728279	MAT2B	protein_coding
ENSG00000038295.8	132.6796126	-0.031091653	0.146241103	-0.212605436	0.831634731	0.91705417	TLL1	protein_coding
ENSG00000038358.15	3167.541302	0.049640468	0.058061925	0.854957323	0.392574775	0.616610112	EDC4	protein_coding
ENSG00000038382.20	6423.689633	0.268066358	0.070914489	3.78013523	0.000156743	0.002825796	TRIO	protein_coding
ENSG00000038427.16	12439.49761	0.718390436	0.115840188	6.201564844	5.59045E-10	1.09244E-07	VCAN	protein_coding
ENSG00000038532.16	2710.120656	0.067168892	0.051377688	1.307355278	0.191092079	0.406920422	CLEC16A	protein_coding
ENSG00000038945.15	1780.03631	0.03621227	0.126334107	0.286638907	0.77438882	0.885856378	MSR1	protein_coding
ENSG00000039068.19	24494.68432	0.034563064	0.103790872	0.333006776	0.739129168	0.865113259	CDH1	protein_coding
ENSG00000039123.16	3188.344701	-0.089506711	0.051257245	-1.746225553	0.080771766	0.241459121	MTREX	protein_coding
ENSG00000039139.9	252.4806399	0.377714141	0.144079068	2.621575399	0.00875244	0.055039145	DNAH5	protein_coding
ENSG00000039319.17	1860.071582	0.073762773	0.058638067	1.257933235	0.208415903	0.428402821	ZFYVE16	protein_coding
ENSG00000039523.20	2906.514948	0.164167097	0.066014174	2.486846182	0.012888111	0.072123438	RIPOR1	protein_coding
ENSG00000039537.14	166.9345871	-0.283595987	0.21628591	-1.311208794	0.189787232	0.405300531	C6	protein_coding
ENSG00000039560.14	4283.113236	0.178013718	0.075485456	2.358251863	0.018361231	0.09170514	RAI14	protein_coding
ENSG00000039600.11	113.3902399	0.453255951	0.172436277	2.628541729	0.008575183	0.054250982	SOX30	protein_coding
ENSG00000039650.12	2034.111419	0.017271108	0.058687187	0.294290955	0.768535583	0.882594308	PNKP	protein_coding
ENSG00000039987.7	15.87499237	1.35541115	0.216213222	6.268863369	3.63693E-10	7.80722E-08	BEST2	protein_coding
ENSG00000040199.18	834.5371221	-0.059675486	0.081714099	-0.730296083	0.465209222	0.677056384	PHLPP2	protein_coding
ENSG00000040275.17	1056.915494	0.178126275	0.065018124	2.739640322	0.006150645	0.043001231	SPDL1	protein_coding
ENSG00000040341.18	2265.031777	-0.039999239	0.054912636	-0.728415926	0.466359017	0.677914701	STAU2	protein_coding
ENSG00000040487.13	1144.73185	-0.04572359	0.064719333	-0.706490438	0.479883197	0.689091841	SLC66A1	protein_coding
ENSG00000040531.16	1108.021418	0.082500723	0.049197864	1.67691677	0.093558758	0.264414684	CTNS	protein_coding
ENSG00000040608.14	695.2202432	0.249190484	0.110336112	2.258467141	0.023916551	0.109602644	RTN4R	protein_coding
ENSG00000040633.13	1755.027478	0.049065333	0.052697898	0.931068119	0.351818329	0.580599608	PHF23	protein_coding
ENSG00000040731.10	14.96621203	-0.37715329	0.267287673	-1.411038845	0.15823316	0.363581673	CDH10	protein_coding
ENSG00000040933.16	2162.694842	-0.092305324	0.059427837	-1.553233769	0.120367296	0.308126986	INPP4A	protein_coding
ENSG00000041353.10	3572.829417	0.019250064	0.139983345	0.137516814	0.890622304	0.947901515	RAB27B	protein_coding
ENSG00000041357.16	7382.176628	-0.055634563	0.056090515	-0.991871137	0.32126039	0.551098848	PSMA4	protein_coding
ENSG00000041515.16	64.75744421	0.141311295	0.140805737	1.003590465	0.315576053	0.544735894	MYO16	protein_coding
ENSG00000041802.11	3031.112461	0.088099757	0.052538772	1.676852229	0.093571382	0.26443321	LSG1	protein_coding
ENSG00000041880.14	1577.378793	-0.100224099	0.078879876	-1.270591493	0.203874015	0.423279716	PARP3	protein_coding
ENSG00000041982.16	14918.51423	-0.073564571	0.168035741	-0.437791217	0.661537639	0.818669971	TNC	protein_coding
ENSG00000041988.15	497.7235419	-0.115568984	0.053068502	-2.177732181	0.029425982	0.125925169	THAP3	protein_coding
ENSG00000042062.13	288.7068714	0.289706187	0.106191078	2.728159389	0.006368883	0.044060454	RIPOR3	protein_coding
ENSG00000042088.14	1139.355199	-0.074283079	0.04888694	-1.519487198	0.128639908	0.320566207	TDP1	protein_coding
ENSG00000042286.15	1716.731361	-0.00609732	0.076698369	-0.079497392	0.936637006	0.969894566	AIFM2	protein_coding
ENSG00000042304.12	6.416127243	-0.217292089	0.256350866	-0.847635479	0.396641009	0.619891162	C2orf83	protein_coding
ENSG00000042317.17	436.3484252	-0.054276653	0.05501849	-0.986516583	0.323879682	0.553606205	SPATA7	protein_coding
ENSG00000042429.12	318.5618846	-0.111130843	0.048652378	-2.284181123	0.022360889	0.104742603	MED17	protein_coding

ENSG00000042445.14	5038.865273	-0.125465327	0.074293538	-1.688778462	0.091261893	0.260320711	RETSAT	protein_coding
ENSG00000042493.16	7132.081271	0.035071817	0.089063363	0.393785002	0.693739779	0.838302248	CAPG	protein_coding
ENSG00000042753.12	4375.572899	-0.066898984	0.063779919	-1.048903562	0.294222506	0.524210192	AP2S1	protein_coding
ENSG00000042781.14	33.288559	-0.218636915	0.147327907	-1.484015614	0.137804778	0.334193951	USH2A	protein_coding
ENSG00000042813.8	2.75308386	-0.341096479	0.349440439	-0.97612194	0.32900404	0.55844697	ZPBP	protein_coding
ENSG00000042832.12	81.28125533	0.212922628	0.161676148	1.316969948	0.187848698	0.403007223	TG	protein_coding
ENSG00000042980.13	3258.404966	-0.416330195	0.154408825	-2.696284974	0.007011765	0.047087639	ADAM28	protein_coding
ENSG00000043039.7	876.9114605	-0.046668822	0.167548428	-0.278539299	0.780598402	0.88874039	BARX2	protein_coding
ENSG00000043093.15	2403.815351	0.051303951	0.047725529	1.074979185	0.282384048	0.511172253	DCUN1D1	protein_coding
ENSG00000043143.20	4348.646818	-0.052194774	0.083731744	-0.623357063	0.533049907	0.730485732	JADE2	protein_coding
ENSG00000043355.12	497.0704804	0.570091744	0.247500559	2.303395783	0.021256585	0.101212652	ZIC2	protein_coding
ENSG00000043462.13	2311.720458	-0.245102436	0.099817082	-2.455515934	0.014068252	0.07667945	LCP2	protein_coding
ENSG00000043514.17	798.9298743	0.024630066	0.04647416	0.529973342	0.596130414	0.77561207	TRIT1	protein_coding
ENSG00000043591.6	142.9472597	0.208300696	0.154477432	1.348421532	0.177522842	0.389797083	ADRB1	protein_coding
ENSG00000044012.4	27.83086141	0.071951694	0.271238067	0.26527137	0.790800418	0.894967143	GUCA2B	protein_coding
ENSG00000044090.10	2162.71247	0.267958331	0.070062913	3.824538828	0.000131017	0.002434683	CUL7	protein_coding
ENSG00000044115.21	24626.87619	0.146327789	0.058068994	2.519895381	0.011738972	0.067513419	CTNNA1	protein_coding
ENSG00000044446.12	1324.123368	-0.142562524	0.065982606	-2.160607655	0.03072566	0.129578251	PHKA2	protein_coding
ENSG00000044459.15	446.8038816	-0.112440279	0.102113771	-1.101127479	0.270841179	0.499004146	CNTLN	protein_coding
ENSG00000044524.11	967.4410066	-0.112137092	0.156969702	-0.714386854	0.474987999	0.685291368	EPHA3	protein_coding
ENSG00000044574.8	43169.59375	0.076861958	0.056486339	1.360717652	0.173602934	0.384718016	HSPA5	protein_coding
ENSG00000046604.13	22953.59896	0.173893458	0.105755777	1.644292764	0.100115743	0.275688698	DSG2	protein_coding
ENSG00000046647.13	775.5335816	-0.178052579	0.055214376	-3.224750372	0.001260826	0.013684432	GEMIN8	protein_coding
ENSG00000046651.15	1808.352798	-0.190857168	0.056717221	-3.365065574	0.000765255	0.009398958	OFD1	protein_coding
ENSG00000046653.15	399.6687224	-0.121684287	0.128787497	-0.944845496	0.344737761	0.574342812	GPM6B	protein_coding
ENSG00000046774.10	38.97513384	1.045591029	0.520616269	2.008371793	0.044603798	0.165335846	MAGEC2	protein_coding
ENSG00000046889.19	716.6887118	0.101588628	0.111903608	0.907822637	0.363971936	0.591159339	PREX2	protein_coding
ENSG00000047056.16	1220.307781	-0.016574444	0.05194115	-0.319100441	0.74965035	0.871407364	WDR37	protein_coding
ENSG00000047188.16	2149.288389	-0.050663777	0.061106565	-0.829105312	0.407044818	0.628185784	YTHDC2	protein_coding
ENSG00000047230.15	1826.756369	-0.167928126	0.058608399	-2.865257022	0.004166709	0.032633479	CTPS2	protein_coding
ENSG00000047249.18	2609.047935	0.024811637	0.050592673	0.490419574	0.623837029	0.794695574	ATP6V1H	protein_coding
ENSG00000047315.17	5405.130472	0.080918838	0.052695445	1.535594567	0.124637848	0.314511402	POLR2B	protein_coding
ENSG00000047346.12	1719.931164	-0.036983689	0.065082519	-0.568258418	0.56985951	0.756572668	FAM214A	protein_coding
ENSG00000047365.12	1727.410895	0.098780736	0.083078509	1.189004685	0.234437823	0.4589854	ARAP2	protein_coding
ENSG00000047410.14	8819.154242	0.1084062	0.048955274	2.214392685	0.026801778	0.118378033	TPR	protein_coding
ENSG00000047457.14	1348.204326	1.640557248	0.200694355	8.174406544	2.97326E-16	4.62734E-13	CP	protein_coding
ENSG00000047578.13	1913.629232	0.106149204	0.055647608	1.907525011	0.056452635	0.192301506	KATNIP	protein_coding
ENSG00000047579.20	999.7977532	-0.124667756	0.044838142	-2.780395221	0.005429278	0.039425368	DTNBP1	protein_coding
ENSG00000047597.7	1412.55385	-0.358217472	0.127110265	-2.818163201	0.004829925	0.036331451	XK	protein_coding
ENSG00000047617.15	56.25455432	-0.201037558	0.103269963	-1.946718612	0.051568479	0.181489227	ANO2	protein_coding
ENSG00000047621.12	999.0203051	-0.054271404	0.050103665	-1.083182314	0.278727508	0.507630739	C12orf4	protein_coding
ENSG00000047634.15	1833.541543	-0.401393745	0.09839733	-4.079315402	4.51685E-05	0.001060842	SCML1	protein_coding

ENSG00000047644.19	2934.726933	0.144507325	0.08187583	1.764957076	0.07757098	0.235367593	WWC3	protein_coding
ENSG00000047648.23	737.9607506	0.053128687	0.130536605	0.407002214	0.684006362	0.833144681	ARHGAP6	protein_coding
ENSG00000047662.5	27.20743851	0.19861429	0.105682895	1.879341872	0.060197828	0.200308548	FAM184B	protein_coding
ENSG00000047849.21	16260.14407	0.048524065	0.055615362	0.872493915	0.382938986	0.607948066	MAP4	protein_coding
ENSG00000047932.14	2239.247985	0.102132292	0.044198578	2.310759645	0.020846135	0.099957308	GOPC	protein_coding
ENSG00000047936.11	109.5626653	0.740876918	0.233266971	3.176090103	0.001492746	0.015487948	ROS1	protein_coding
ENSG00000048028.11	1976.625862	-0.065383275	0.054610612	-1.197263171	0.231203994	0.455171518	USP28	protein_coding
ENSG00000048052.22	1213.23938	-0.136372096	0.139154589	-0.980004309	0.327083992	0.556725964	HDAC9	protein_coding
ENSG00000048140.18	2252.128406	0.007014609	0.054251419	0.129298161	0.897121727	0.951119711	TSPAN17	protein_coding
ENSG00000048162.21	1059.2289	-0.12578794	0.071873925	-1.750119252	0.080097738	0.240158185	NOP16	protein_coding
ENSG00000048342.17	765.5969063	0.057226247	0.095208307	0.601063595	0.54779763	0.74113997	CC2D2A	protein_coding
ENSG00000048392.12	2522.610194	-0.040877359	0.070399907	-0.580645071	0.561479689	0.75067974	RRM2B	protein_coding
ENSG00000048405.10	2148.979401	-0.114575927	0.045595389	-2.51288409	0.011974867	0.068428969	ZNF800	protein_coding
ENSG00000048462.11	141.1186903	-1.025962679	0.172262641	-5.955804889	2.58795E-09	4.01804E-07	TNFRSF17	protein_coding
ENSG00000048471.14	1889.391352	0.042525525	0.074960276	0.567307474	0.5705053	0.756939151	SNX29	protein_coding
ENSG00000048540.15	325.017191	-0.081489693	0.166323095	-0.489948152	0.624170588	0.794924329	LMO3	protein_coding
ENSG00000048544.6	3353.534947	0.145417712	0.062685337	2.319804282	0.020351467	0.098397414	MRPS10	protein_coding
ENSG00000048545.16	3.407641659	-0.003221983	0.20652847	-0.01560067	0.987552971	0.994100158	GUCA1A	protein_coding
ENSG00000048649.13	4660.504666	0.041797368	0.047443698	0.880988835	0.378323863	0.604071255	RSF1	protein_coding
ENSG00000048707.15	5371.505791	0.017060203	0.060299881	0.282922664	0.777236121	0.887057211	VPS13D	protein_coding
ENSG00000048740.18	2525.534197	-0.268652996	0.127695211	-2.103861173	0.035390559	0.141760675	CELF2	protein_coding
ENSG00000048828.17	23277.08937	-0.016148662	0.051317919	-0.314678807	0.753005537	0.873100132	FAM120A	protein_coding
ENSG00000048991.16	2989.691083	-0.048622242	0.045649763	-1.065114877	0.286823948	0.516441913	R3HDM1	protein_coding
ENSG00000049089.15	956.1213973	-0.005902977	0.157776406	-0.037413557	0.970155263	0.986049032	COL9A2	protein_coding
ENSG00000049130.16	4010.427287	-0.305124277	0.101321571	-3.011444393	0.00260008	0.023118485	KITLG	protein_coding
ENSG00000049167.15	646.4303584	-0.042516175	0.050459963	-0.842572455	0.399467602	0.622272159	ERCC8	protein_coding
ENSG00000049192.15	274.8519402	0.281461903	0.108138418	2.602792863	0.00924678	0.057217636	ADAMTS6	protein_coding
ENSG00000049239.13	8910.075132	-0.008447085	0.070108115	-0.120486549	0.904097734	0.954197655	H6PD	protein_coding
ENSG00000049245.13	6663.970764	-0.001689938	0.048547595	-0.034809932	0.972231301	0.986952813	VAMP3	protein_coding
ENSG00000049246.14	1460.850463	-0.255173762	0.107898951	-2.364932745	0.018033345	0.090576198	PER3	protein_coding
ENSG00000049247.14	54.15355521	-0.636849288	0.209955259	-3.033261895	0.002419255	0.021902202	UTS2	protein_coding
ENSG00000049249.9	351.3381973	-0.118573833	0.118521657	-1.000440217	0.317097515	0.546532976	TNFRSF9	protein_coding
ENSG00000049283.18	2379.073896	-0.109357193	0.139371183	-0.784647091	0.432660512	0.650722963	EPN3	protein_coding
ENSG00000049323.16	6496.664659	0.152501246	0.121323826	1.256976895	0.208762002	0.428714971	LTBP1	protein_coding
ENSG00000049449.10	3422.540992	-0.276580413	0.086079286	-3.213089085	0.001313156	0.014089872	RCN1	protein_coding
ENSG00000049540.18	5099.4192	0.43676291	0.148608241	2.939022148	0.003292495	0.027544073	ELN	protein_coding
ENSG00000049541.11	1803.813076	-0.12932848	0.066018922	-1.95896079	0.050117379	0.178350763	RFC2	protein_coding
ENSG00000049618.24	7512.461372	0.065585369	0.046168039	1.420579486	0.155439046	0.359961312	ARID1B	protein_coding
ENSG00000049656.14	7749.181784	0.006391472	0.065642465	0.097367953	0.922434194	0.963331054	CLPTM1L	protein_coding
ENSG00000049759.19	5348.909077	-0.251869731	0.073328952	-3.434792449	0.000593008	0.007732346	NEDD4L	protein_coding
ENSG00000049768.16	433.2714202	-0.13341386	0.107397105	-1.242248204	0.214144999	0.435039367	FOXP3	protein_coding
ENSG00000049769.14	125.1920603	0.128582737	0.091787508	1.400874038	0.161251743	0.367667812	PPP1R3F	protein_coding

ENSG00000049860.14	7286.768109	-0.039211516	0.060138917	-0.652015675	0.514391061	0.716305471	HEXB	protein_coding
ENSG00000049883.15	678.7763801	-0.087414374	0.048983142	-1.78458077	0.074329343	0.228714149	PTCD2	protein_coding
ENSG00000050030.16	82.81045384	-0.229931409	0.205268758	-1.120148098	0.262650657	0.490249682	NEXMIF	protein_coding
ENSG00000050130.18	2047.371205	0.065852704	0.052458595	1.255327237	0.209359991	0.429532973	JKAMP	protein_coding
ENSG00000050165.18	5188.208138	0.110942868	0.099323298	1.116987361	0.263999729	0.491763537	DKK3	protein_coding
ENSG00000050327.15	1275.774663	0.052816821	0.075888988	0.695974779	0.486444625	0.694056818	ARHGEF5	protein_coding
ENSG00000050344.9	3074.953187	-0.069528272	0.089725802	-0.774897185	0.438400416	0.65521744	NFE2L3	protein_coding
ENSG00000050393.11	3431.336438	-0.137062558	0.074284702	-1.845098032	0.06502328	0.209968096	MCUR1	protein_coding
ENSG00000050405.13	11635.54752	-0.121478676	0.081924237	-1.482817292	0.138122961	0.334685512	LIMA1	protein_coding
ENSG00000050426.16	2732.336206	-0.256688618	0.048995393	-5.239035783	1.61418E-07	1.19752E-05	LETMD1	protein_coding
ENSG00000050438.17	336.6141527	-0.32493055	0.133820968	-2.42809893	0.015178202	0.080650739	SLC4A8	protein_coding
ENSG00000050555.19	452.2300789	0.376286431	0.158312027	2.376865729	0.017460443	0.088654248	LAMC3	protein_coding
ENSG00000050628.20	570.6601107	0.23423086	0.158872913	1.474328482	0.14039317	0.33809965	PTGER3	protein_coding
ENSG00000050730.16	104.0913259	-0.240195096	0.129831699	-1.850049702	0.064306386	0.208627114	TNIP3	protein_coding
ENSG00000050748.18	3060.608279	0.007962408	0.050881765	0.156488445	0.875648026	0.93989045	MAPK9	protein_coding
ENSG00000050767.17	144.2593511	-0.316838574	0.124188159	-2.551278444	0.010732854	0.063443513	COL23A1	protein_coding
ENSG00000050820.17	7715.644452	0.231708046	0.06576058	3.523509745	0.000425871	0.006031264	BCAR1	protein_coding
ENSG00000051009.11	3453.749	-0.023318356	0.060952978	-0.382563038	0.70204378	0.843362145	FAM160A2	protein_coding
ENSG00000051108.15	8207.467935	-0.119443355	0.064049138	-1.864870622	0.062199541	0.204532856	HERPUD1	protein_coding
ENSG00000051128.19	423.7669494	0.31477365	0.099019977	3.178890346	0.0014784	0.015372045	HOMER3	protein_coding
ENSG00000051180.17	742.928214	-0.061409258	0.077673567	-0.790606909	0.429173414	0.647377544	RAD51	protein_coding
ENSG00000051341.14	1002.789222	-0.097472566	0.099452147	-0.980095142	0.327039157	0.556725964	POLQ	protein_coding
ENSG00000051382.9	3885.723331	0.051335556	0.059088196	0.868795451	0.384959022	0.609769158	PIK3CB	protein_coding
ENSG00000051523.11	16025.96889	-0.154871033	0.089997972	-1.72082803	0.085282034	0.249871258	CYBA	protein_coding
ENSG00000051596.10	816.784569	-0.018901769	0.071924733	-0.2627993	0.792705275	0.896131185	THOC3	protein_coding
ENSG00000051620.11	5471.198966	0.038604559	0.060013171	0.643268101	0.520050151	0.720281773	HEBP2	protein_coding
ENSG00000051825.15	1334.658008	-0.116045158	0.061385427	-1.890434956	0.058699812	0.197084653	MPHOSPH9	protein_coding
ENSG00000052126.14	3318.799338	-0.047533333	0.069061616	-0.68827426	0.491280088	0.698414915	PLEKHA5	protein_coding
ENSG00000052344.16	9215.926243	-0.03251715	0.119085913	-0.273056229	0.784809992	0.890941099	PRSS8	protein_coding
ENSG00000052723.12	2509.070941	0.052513567	0.042697341	1.229902516	0.218733612	0.440325376	SIKE1	protein_coding
ENSG00000052749.14	2687.112974	0.000475496	0.062530535	0.007604216	0.993932772	0.997296993	RRP12	protein_coding
ENSG00000052795.13	3446.090192	0.02643235	0.059044488	0.447668368	0.654392554	0.814221063	FNIP2	protein_coding
ENSG00000052802.13	3549.24302	0.293414745	0.094453168	3.106457432	0.001893436	0.018381765	MSMO1	protein_coding
ENSG00000052841.15	4425.532218	0.064773821	0.044608131	1.452063086	0.146484065	0.347090212	TTC17	protein_coding
ENSG00000052850.8	14.80500934	1.052682307	0.178873487	5.885066169	3.97895E-09	5.77969E-07	ALX4	protein_coding
ENSG00000053108.17	100.7252227	1.036448788	0.239281634	4.331501637	1.48096E-05	0.000442328	FSTL4	protein_coding
ENSG00000053254.16	5254.183338	-0.13252465	0.069784537	-1.899054652	0.057557291	0.194621351	FOXN3	protein_coding
ENSG00000053328.8	124.6047901	-0.528025963	0.197966413	-2.667250241	0.007647471	0.050120895	METTL24	protein_coding
ENSG00000053371.12	3258.690931	-0.091965814	0.068938465	-1.334027574	0.18219482	0.395673303	AKR7A2	protein_coding
ENSG00000053372.5	2592.220289	-0.135511962	0.061973711	-2.186603966	0.028771448	0.12403773	MRTO4	protein_coding
ENSG00000053438.11	157.2068271	0.06010395	0.129492829	0.464148866	0.642541093	0.806683592	NNAT	protein_coding
ENSG00000053501.13	885.2633589	-0.193098014	0.065340113	-2.955275198	0.003123903	0.026475399	USE1	protein_coding

ENSG00000053524.12	100.6737919	-0.006638246	0.093426577	-0.071053084	0.943355507	0.973330246	MCF2L2	protein_coding
ENSG00000053702.15	282.2784759	-0.090693424	0.085456384	-1.061283187	0.288561224	0.518327801	NRIP2	protein_coding
ENSG00000053747.17	9763.29345	0.274447687	0.145859617	1.881587873	0.059891994	0.19971704	LAMA3	protein_coding
ENSG00000053770.12	2880.656091	0.026089446	0.050447646	0.517158833	0.605045287	0.78230598	AP5M1	protein_coding
ENSG00000053900.11	1343.106222	-0.068262382	0.044352493	-1.539087811	0.12378286	0.313245395	ANAPC4	protein_coding
ENSG00000053918.18	4446.814615	-0.461269068	0.136111951	-3.388894704	0.00070175	0.008762217	KCNQ1	protein_coding
ENSG00000054116.12	2525.065107	-0.052205792	0.041127768	-1.269356329	0.204314004	0.423769223	TRAPPC3	protein_coding
ENSG00000054118.15	8553.916464	-0.009144551	0.035373664	-0.258512971	0.79601104	0.897879624	THRAP3	protein_coding
ENSG00000054148.18	2937.88521	-0.168560716	0.06869909	-2.453609176	0.01414306	0.076942837	PHPT1	protein_coding
ENSG00000054179.12	1986.353937	0.207843226	0.153219771	1.356503955	0.174938878	0.386288076	ENTPD2	protein_coding
ENSG00000054219.11	1644.143526	-0.016799456	0.089352758	-0.188012734	0.850866669	0.926965412	LY75	protein_coding
ENSG00000054267.22	4004.94168	0.047444204	0.052080135	0.910984664	0.362303454	0.589834409	ARID4B	protein_coding
ENSG00000054277.14	127.5725669	0.301884266	0.096095125	3.141514897	0.001680763	0.016887847	OPN3	protein_coding
ENSG00000054282.16	1247.38909	0.011408083	0.048861952	0.233475793	0.815391965	0.908825118	SDCCAG8	protein_coding
ENSG00000054356.14	141.0733526	0.527492295	0.210300411	2.508279902	0.012132052	0.069072177	PTPRN	protein_coding
ENSG00000054392.13	292.5490636	0.297571421	0.097510442	3.051687742	0.002275587	0.020991057	HHAT	protein_coding
ENSG00000054523.18	5041.443955	-0.005762979	0.057317657	-0.100544574	0.919911997	0.962213821	KIF1B	protein_coding
ENSG00000054598.9	2111.222778	0.790642874	0.126606025	6.244907157	4.24052E-10	8.91533E-08	FOXC1	protein_coding
ENSG00000054611.14	2450.347542	-0.089011337	0.045333349	-1.963484696	0.049589881	0.176984295	TBC1D22A	protein_coding
ENSG00000054654.19	12744.561	-0.136146006	0.087286881	-1.559753351	0.118818179	0.305447626	SYNE2	protein_coding
ENSG00000054690.14	2206.870129	-0.101398501	0.088225325	-1.149312858	0.250426998	0.477210831	PLEKHH1	protein_coding
ENSG00000054793.14	10552.66594	-0.006657109	0.081604683	-0.081577537	0.934982665	0.969411588	ATP9A	protein_coding
ENSG00000054796.13	1.510576845	-0.497375619	0.390518825	-1.273627767	0.202795367	0.422001916	SPO11	protein_coding
ENSG00000054803.4	14.95947827	0.118000314	0.211332773	0.55836259	0.576596813	0.761377055	CBLN4	protein_coding
ENSG00000054938.16	2820.776584	-0.13767922	0.213094051	-0.646096027	0.518217168	0.71911254	CHRDL2	protein_coding
ENSG00000054965.11	3988.490525	0.041193228	0.054662162	0.753596755	0.451091393	0.665736771	FAM168A	protein_coding
ENSG00000054967.13	485.0208702	-0.014367956	0.078710364	-0.182542111	0.85515731	0.929329661	RELT	protein_coding
ENSG00000054983.17	4026.417165	-0.132926917	0.09558221	-1.390707712	0.164314078	0.371829183	GALC	protein_coding
ENSG00000055044.11	3771.675827	-0.051889605	0.055608155	-0.933129423	0.350753149	0.579803125	NOP58	protein_coding
ENSG00000055070.17	6882.155483	-0.040568954	0.046756496	-0.867664549	0.385577998	0.610217266	SZRD1	protein_coding
ENSG00000055118.16	1495.436409	-0.221205283	0.183285398	-1.20688983	0.227474575	0.450862347	KCNH2	protein_coding
ENSG00000055130.17	4164.80278	-0.138070961	0.044248466	-3.120355876	0.001806327	0.017764454	CUL1	protein_coding
ENSG00000055147.19	1076.11253	-0.148542938	0.044181719	-3.362090545	0.000773548	0.009462657	FAM114A2	protein_coding
ENSG00000055163.20	2359.447744	-0.433799534	0.11603955	-3.738376549	0.000185212	0.003214484	CYFIP2	protein_coding
ENSG00000055208.19	5727.745241	0.117138792	0.044941887	2.606450222	0.009148612	0.05677429	TAB2	protein_coding
ENSG00000055211.14	1946.216382	0.052350439	0.047971597	1.091279887	0.275149745	0.503933141	GINM1	protein_coding
ENSG00000055332.18	7869.037086	0.102575859	0.057198302	1.793337469	0.07291898	0.225777347	EIF2AK2	protein_coding
ENSG00000055483.19	3557.372829	0.010607362	0.046412686	0.228544464	0.819222993	0.910951552	USP36	protein_coding
ENSG00000055609.19	7016.779754	-0.10362305	0.05672163	-1.826870098	0.067719303	0.215401757	KMT2C	protein_coding
ENSG00000055732.13	302.4689384	0.006489934	0.173352994	0.037437679	0.97013603	0.986049032	MCOLN3	protein_coding
ENSG00000055813.6	41.17074861	0.27381444	0.158608381	1.726355434	0.084283488	0.248034213	CCDC85A	protein_coding
ENSG00000055917.16	9076.880691	-0.013377906	0.03893732	-0.343575413	0.731165618	0.860298462	PUM2	protein_coding

ENSG00000055950.16	2481.806499	-0.127178892	0.055070543	-2.309381468	0.020922422	0.100245894	MRPL43	protein_coding
ENSG00000055955.17	13.47951349	0.014996993	0.143926128	0.104199241	0.917011236	0.960600624	ITIH4	protein_coding
ENSG00000055957.11	19.28416518	-0.760100214	0.211884601	-3.587331069	0.00033408	0.005033958	ITIH1	protein_coding
ENSG00000056050.7	700.3900227	-0.04894558	0.055454784	-0.882621418	0.377440852	0.603254723	HPF1	protein_coding
ENSG00000056097.16	7268.204732	0.055584079	0.041961086	1.324657785	0.185284681	0.399750002	ZFR	protein_coding
ENSG00000056277.16	522.7882328	-0.057206925	0.066326546	-0.862504208	0.388410119	0.612874235	ZNF280C	protein_coding
ENSG00000056291.17	14.40967054	0.149017202	0.225828234	0.659869668	0.509337471	0.712807469	NPFFR2	protein_coding
ENSG00000056487.16	19.81471252	-0.689437735	0.209364373	-3.29300408	0.00099123	0.011421165	PHF21B	protein_coding
ENSG00000056558.11	1025.106875	-0.36901541	0.095443656	-3.866316799	0.000110491	0.002137099	TRAF1	protein_coding
ENSG00000056586.16	4009.636481	0.100467793	0.055104408	1.823226056	0.06826916	0.216534333	RC3H2	protein_coding
ENSG00000056736.10	1505.099233	-0.317978382	0.111240507	-2.858476571	0.004256805	0.033124788	IL17RB	protein_coding
ENSG00000056972.20	2955.041455	-0.234020253	0.063543074	-3.682860135	0.000230632	0.003798757	TRAF3IP2	protein_coding
ENSG00000056998.20	662.9909943	0.129472804	0.131864279	0.981864114	0.326166797	0.555969901	GYG2	protein_coding
ENSG00000057019.16	1797.017951	0.545392827	0.117671443	4.634878396	3.57147E-06	0.000142784	DCBLD2	protein_coding
ENSG00000057149.16	106.0877322	1.325426077	0.338065415	3.920620149	8.83214E-05	0.001801116	SERPINB3	protein_coding
ENSG00000057252.13	2610.139754	-0.034017225	0.081588166	-0.416938226	0.676723593	0.828731564	SOAT1	protein_coding
ENSG00000057294.15	6158.619421	0.084090088	0.092826408	0.905885404	0.36499651	0.592249514	PKP2	protein_coding
ENSG00000057468.7	23.89421897	-0.060805663	0.173153962	-0.351165302	0.72546434	0.856782469	MSH4	protein_coding
ENSG00000057593.14	101.4653157	-0.010250567	0.239625886	-0.04277738	0.965878996	0.984447023	F7	protein_coding
ENSG00000057608.17	17121.71284	-0.09201393	0.052150806	-1.764381758	0.077667727	0.235522009	GDI2	protein_coding
ENSG00000057657.17	3491.523453	-0.036727371	0.094620012	-0.388156478	0.697900239	0.840875629	PRDM1	protein_coding
ENSG00000057663.16	2673.303055	0.137428096	0.064043373	2.145859753	0.03188417	0.132682654	ATG5	protein_coding
ENSG00000057704.13	1647.411888	0.007697829	0.10719709	0.071810058	0.942753069	0.973008159	TMCC3	protein_coding
ENSG00000057757.10	1844.142585	-0.165694321	0.048578895	-3.410829357	0.000647656	0.00824749	PITHD1	protein_coding
ENSG00000057935.14	2483.873291	-0.079620239	0.046432313	-1.714759263	0.086389373	0.251906591	MTA3	protein_coding
ENSG00000058056.9	1147.248801	-0.046788199	0.084279873	-0.5551527	0.578790219	0.763098795	USP13	protein_coding
ENSG00000058063.16	6007.1334	0.004457071	0.068819309	0.064764832	0.948361242	0.975768844	ATP11B	protein_coding
ENSG00000058085.15	14381.14136	0.810075212	0.135036255	5.998946067	1.98602E-09	3.26585E-07	LAMC2	protein_coding
ENSG00000058091.17	1178.482253	0.087479865	0.116903035	0.748311324	0.454272396	0.668529539	CDK14	protein_coding
ENSG00000058262.10	19486.22384	0.161103686	0.053957147	2.98577104	0.002828643	0.02454965	SEC61A1	protein_coding
ENSG00000058272.19	5838.904638	-0.220468782	0.071719359	-3.074048404	0.002111752	0.019897038	PPP1R12A	protein_coding
ENSG00000058335.16	142.6422495	-0.613241624	0.153037674	-4.007128488	6.14614E-05	0.001354732	RASGRF1	protein_coding
ENSG00000058404.20	54.257656	-0.157351075	0.168210962	-0.935438887	0.349562164	0.578734487	CAMK2B	protein_coding
ENSG00000058453.17	1112.559439	0.11088018	0.067157971	1.651035293	0.098731368	0.273412357	CROCC	protein_coding
ENSG00000058600.16	2322.651304	0.012179099	0.043014312	0.283140632	0.777069035	0.886959436	POLR3E	protein_coding
ENSG00000058668.15	12252.42788	-0.210505168	0.121162382	-1.737380563	0.08232002	0.244446985	ATP2B4	protein_coding
ENSG00000058673.17	22.71363404	0.116936062	0.084927553	1.37689192	0.168545666	0.377818934	ZC3H11A	protein_coding
ENSG00000058729.11	1073.107688	-0.046379418	0.051960883	-0.892583332	0.372080349	0.598639297	RIOK2	protein_coding
ENSG00000058799.15	1670.06822	0.034220247	0.045484901	0.752342999	0.451844815	0.666352212	YIPF1	protein_coding
ENSG00000058804.12	3764.951142	-0.041731159	0.070797741	-0.589441965	0.555564832	0.746503306	NDC1	protein_coding
ENSG00000058866.15	327.5525756	-0.204625924	0.14583006	-1.403180688	0.160562965	0.366711337	DGKG	protein_coding
ENSG00000059122.17	1936.884539	0.002381554	0.058456402	0.040740679	0.967502631	0.984813766	FLYWCH1	protein_coding

ENSG00000059145.19	746.6353874	0.10511354	0.064569055	1.6279244	0.103540917	0.281244314	UNKL	protein_coding
ENSG00000059377.18	1199.691033	-0.180998317	0.104617571	-1.730094807	0.083613338	0.246941269	TBXAS1	protein_coding
ENSG00000059378.13	4088.25531	-0.003659043	0.069020364	-0.053013962	0.957720783	0.980306733	PARP12	protein_coding
ENSG00000059573.9	8146.713083	-0.064988724	0.059419338	-1.093730191	0.274073329	0.502598529	ALDH18A1	protein_coding
ENSG00000059588.10	2662.883075	-0.002613468	0.06737415	-0.038790372	0.969057521	0.985682731	TARBP1	protein_coding
ENSG00000059691.12	797.7214947	-0.074151244	0.055697915	-1.331310948	0.183086717	0.396953574	GATB	protein_coding
ENSG00000059728.11	5735.116235	0.396530826	0.102828582	3.856231593	0.000115148	0.002201765	MXD1	protein_coding
ENSG00000059758.8	2029.317706	-0.123825879	0.055166042	-2.244603255	0.024793614	0.11248634	CDK17	protein_coding
ENSG00000059769.20	528.0005025	-0.036503217	0.052284398	-0.698166532	0.485073054	0.692959234	DNAJC25	protein_coding
ENSG00000059804.16	2847.707747	0.261523491	0.116910055	2.23696319	0.025288746	0.113960848	SLC2A3	protein_coding
ENSG00000059915.17	830.5587568	-0.417940626	0.206631711	-2.022635459	0.043110746	0.161770168	PSD	protein_coding
ENSG00000060069.17	1827.540599	0.039999846	0.05265622	0.759641422	0.447468952	0.662772263	CTDP1	protein_coding
ENSG00000060138.13	11758.69977	0.135657843	0.066215378	2.048736199	0.040487917	0.155243465	YBX3	protein_coding
ENSG00000060140.9	536.7806926	0.279782658	0.130112361	2.150315747	0.031530248	0.131800643	STYK1	protein_coding
ENSG00000060237.18	17408.43875	0.126335808	0.067603312	1.86878135	0.061653241	0.203273305	WNK1	protein_coding
ENSG00000060303.5	9.084909879	-0.256744739	0.122150107	-2.101878952	0.035563881	0.142210564	RPS17P5	processed_pseudogene
ENSG00000060339.14	5518.960359	-0.081863028	0.047211376	-1.733968277	0.082923712	0.24562425	CCAR1	protein_coding
ENSG00000060491.16	4573.624377	0.020292986	0.06487537	0.312799548	0.754432959	0.87384931	OGFR	protein_coding
ENSG00000060558.4	680.9542656	0.571012769	0.120285926	4.747128676	2.06325E-06	9.24488E-05	GNA15	protein_coding
ENSG00000060566.14	752.916727	-0.815587773	0.238472511	-3.420049415	0.000626098	0.008043472	CREB3L3	protein_coding
ENSG00000060642.11	1008.30201	-0.108704608	0.051422538	-2.113948698	0.034519649	0.13955433	PIGV	protein_coding
ENSG00000060656.20	3396.521113	0.941211464	0.131864199	7.137733122	9.48826E-13	5.3009E-10	PTPRU	protein_coding
ENSG00000060688.13	1924.23405	-0.088914602	0.04680108	-1.899840829	0.057454011	0.194495839	SNRNP40	protein_coding
ENSG00000060709.15	249.6657079	0.979247224	0.215598941	4.541985315	5.57269E-06	0.000204068	RIMBP2	protein_coding
ENSG00000060718.22	1838.07948	1.140748826	0.209782852	5.437760122	5.39545E-08	4.92909E-06	COL11A1	protein_coding
ENSG00000060749.16	3575.237784	0.042486326	0.068654182	0.618845412	0.536018203	0.732572219	QSER1	protein_coding
ENSG00000060762.19	2177.125917	-0.094889864	0.073399771	-1.292781476	0.196086649	0.413197173	MPC1	protein_coding
ENSG00000060971.19	1915.202031	-0.033223923	0.0614917	-0.540299308	0.588990636	0.770555854	ACAA1	protein_coding
ENSG00000060982.15	1584.137528	0.670910599	0.125236329	5.357156373	8.45419E-08	7.08478E-06	BCAT1	protein_coding
ENSG00000061273.18	4785.907891	0.14158572	0.053775129	2.632921976	0.008465379	0.053751396	HDAC7	protein_coding
ENSG00000061337.15	869.8530101	0.251488141	0.109654248	2.293464647	0.021821268	0.102989864	LZTS1	protein_coding
ENSG00000061455.11	180.2787895	0.500854133	0.136230754	3.676512969	0.000236444	0.003867685	PRDM6	protein_coding
ENSG00000061492.12	2.675524367	-0.020385182	0.375471532	-0.054292219	0.956702349	0.979774246	WNT8A	protein_coding
ENSG00000061656.10	439.5005886	-0.118459824	0.108273956	-1.094074953	0.273922106	0.502462818	SPAG4	protein_coding
ENSG00000061676.15	14503.41801	0.010333279	0.050659312	0.203975911	0.838372323	0.920431088	NCKAP1	protein_coding
ENSG00000061794.13	5563.703014	-0.021574436	0.08718088	-0.247467524	0.804546422	0.902919531	MRPS35	protein_coding
ENSG00000061918.14	1645.41544	-0.077708349	0.11207978	-0.693330671	0.488102059	0.695543885	GUCY1B1	protein_coding
ENSG00000061936.10	2576.877486	-0.057656783	0.039342415	-1.465512062	0.142781264	0.341526724	SFSWAP	protein_coding
ENSG00000061938.20	4307.560501	0.33302104	0.08300737	4.011945471	6.02204E-05	0.001333448	TNK2	protein_coding
ENSG00000061987.16	3089.698943	-0.054263319	0.055855962	-0.971486606	0.33130602	0.560776171	MON2	protein_coding
ENSG00000062038.14	3417.578309	0.721157311	0.143020138	5.042348024	4.59854E-07	2.7564E-05	CDH3	protein_coding
ENSG00000062096.15	5.99020525	-0.292751258	0.223862989	-1.307725141	0.190966553	0.406867441	ARSF	protein_coding

ENSG00000062194.16	4217.128644	-0.013257182	0.04174007	-0.317612841	0.750778632	0.872071097	GPBP1	protein_coding
ENSG00000062282.15	1515.370469	-0.298106795	0.108039409	-2.759241263	0.005793574	0.041320228	DGAT2	protein_coding
ENSG00000062370.17	228.6114463	0.003069096	0.081037727	0.03787243	0.969789395	0.985924016	ZNF112	protein_coding
ENSG00000062485.19	12979.79641	-0.098322897	0.051039213	-1.926418743	0.054052109	0.187057556	CS	protein_coding
ENSG00000062524.16	460.2872183	0.033827834	0.173407709	0.195076875	0.845332766	0.924184398	LTK	protein_coding
ENSG00000062582.14	15.61694759	-0.002692971	0.093457764	-0.028814845	0.977012261	0.989616087	MRPS24	protein_coding
ENSG00000062598.18	2580.322929	0.018769174	0.050287485	0.373237467	0.708971713	0.847688645	ELMO2	protein_coding
ENSG00000062650.19	4490.887682	-0.013865498	0.048871212	-0.283715053	0.776628758	0.886761434	WAPL	protein_coding
ENSG00000062716.13	12759.71664	0.190943979	0.066088756	2.889205225	0.003862169	0.030943818	VMP1	protein_coding
ENSG00000062725.10	2532.636686	0.114996371	0.044077779	2.608942052	0.009082262	0.056450968	APPBP2	protein_coding
ENSG00000062822.15	1982.980929	-0.18224327	0.088145728	-2.067522426	0.038684953	0.150811792	POLD1	protein_coding
ENSG00000063015.20	50.44388605	-0.25288106	0.154699006	-1.634665059	0.102119281	0.278860252	SEZ6	protein_coding
ENSG00000063046.18	16425.57051	-0.075884845	0.052059136	-1.457666239	0.144932562	0.345008535	EIF4B	protein_coding
ENSG00000063127.16	97.03490586	-0.339344692	0.133376483	-2.544261812	0.010950897	0.064361005	SLC6A16	protein_coding
ENSG00000063169.11	2482.80849	0.070438451	0.059385654	1.186118965	0.235575322	0.460467651	BICRA	protein_coding
ENSG00000063176.16	1394.46073	-0.196805218	0.083105483	-2.3681376	0.017877885	0.089992447	SPHK2	protein_coding
ENSG00000063177.13	45214.83025	-0.205262404	0.070812656	-2.898668329	0.003747511	0.030252924	RPL18	protein_coding
ENSG00000063180.9	278.6612304	-0.142674456	0.100574565	-1.418593824	0.156017467	0.360735034	CA11	protein_coding
ENSG00000063241.8	2580.226865	-0.083768063	0.074300314	-1.127425429	0.259562653	0.486786096	ISOC2	protein_coding
ENSG00000063244.13	9558.616002	-0.017515015	0.055962402	-0.312978267	0.754297174	0.87381598	U2AF2	protein_coding
ENSG00000063245.15	16782.98091	-0.1578746	0.067962274	-2.32297406	0.020180546	0.097831533	EPN1	protein_coding
ENSG00000063322.15	3678.483626	0.180242835	0.056394331	3.196116197	0.00139291	0.014790166	MED29	protein_coding
ENSG00000063438.19	14.79297805	0.231537686	0.143652803	1.611786766	0.107008349	0.286624708	AHRR	protein_coding
ENSG00000063515.3	1.784953279	0.318759866	0.289363312	1.101590466	0.270639755	0.498782773	GSC2	protein_coding
ENSG00000063587.15	2181.216281	0.112343913	0.05541206	2.027427115	0.042618751	0.160527087	ZNF275	protein_coding
ENSG00000063601.17	2175.185191	0.144075367	0.053916856	2.672176703	0.007536095	0.049547439	MTMR1	protein_coding
ENSG00000063660.9	5366.110196	0.418335961	0.107931258	3.875948159	0.00010621	0.002075483	GPC1	protein_coding
ENSG00000063761.16	727.9999974	-0.130074845	0.052133606	-2.495028726	0.012594693	0.070909425	ADCK1	protein_coding
ENSG00000063854.13	1499.840997	-0.045614892	0.052514525	-0.868614777	0.385057869	0.609827242	HAGH	protein_coding
ENSG00000063978.16	4562.162492	-0.040779982	0.043461205	-0.938307657	0.348086326	0.577387912	RNF4	protein_coding
ENSG00000064012.22	3059.848441	-0.081678266	0.056751361	-1.439230089	0.150085343	0.352309254	CASP8	protein_coding
ENSG00000064042.18	1184.138464	0.533579735	0.10813208	4.934518385	8.03488E-07	4.39317E-05	LIMCH1	protein_coding
ENSG00000064102.15	1936.276824	0.05752666	0.079404766	0.724473644	0.468775002	0.680156099	INTS13	protein_coding
ENSG00000064115.11	4923.121574	-0.079493918	0.088939876	-0.893793889	0.37143218	0.59799387	TM7SF3	protein_coding
ENSG00000064195.7	95.59782439	1.096112012	0.294030304	3.727887896	0.000193091	0.003304924	DLX3	protein_coding
ENSG00000064199.7	296.715833	-0.042440889	0.072943656	-0.581831114	0.560680444	0.75002369	SPA17	protein_coding
ENSG00000064201.16	84.52926388	-0.681172934	0.152214568	-4.475083724	7.63814E-06	0.000262747	TSPAN32	protein_coding
ENSG00000064205.10	106.4343679	0.525167244	0.186654313	2.813582153	0.004899287	0.036689502	CCN5	protein_coding
ENSG00000064218.5	15.73048075	1.165336278	0.227278497	5.12734946	2.9385E-07	1.93139E-05	DMRT3	protein_coding
ENSG00000064225.12	294.00224	0.199639197	0.098335638	2.030181533	0.04233809	0.159861967	ST3GAL6	protein_coding
ENSG00000064270.13	1744.06954	0.082812856	0.158257528	0.523279094	0.600780047	0.779033272	ATP2C2	protein_coding
ENSG00000064300.9	560.4758311	0.412200783	0.219042287	1.881831997	0.05985883	0.19966842	NGFR	protein_coding

ENSG00000064309.14	616.5602566	-0.083035673	0.144366207	-0.575173892	0.565173698	0.753656494	CDON	protein_coding
ENSG00000064313.12	3350.888237	0.089950898	0.054380149	1.654112749	0.098104606	0.272386392	TAF2	protein_coding
ENSG00000064393.16	9788.647556	-0.285540801	0.087998797	-3.244826193	0.001175223	0.012988259	HIPK2	protein_coding
ENSG00000064419.13	4526.967632	-0.125109169	0.041993654	-2.979239873	0.002889645	0.024930122	TNPO3	protein_coding
ENSG00000064489.23	1.717316837	-0.32766787	0.207986872	-1.575425734	0.115158208	0.299537126	BORCS8-MEF2B	protein_coding
ENSG00000064490.14	1313.736178	-0.164266485	0.064281269	-2.555433144	0.010605573	0.062861679	RFXANK	protein_coding
ENSG00000064545.15	1961.568027	-0.100159694	0.064942516	-1.542282314	0.123005005	0.312128871	TMEM161A	protein_coding
ENSG00000064547.14	1455.779737	-0.122355817	0.076365144	-1.60224692	0.109101018	0.28979002	LPAR2	protein_coding
ENSG00000064601.20	15757.4115	-0.030445722	0.07878477	-0.386441715	0.699169561	0.841761111	CTSA	protein_coding
ENSG00000064607.17	3840.794334	-0.052708593	0.050975619	-1.033996145	0.301137965	0.530764249	SUGP2	protein_coding
ENSG00000064651.14	15466.38456	-0.027854914	0.120989425	-0.230226019	0.817916145	0.910217994	SLC12A2	protein_coding
ENSG00000064652.11	644.7020329	0.162773204	0.07101663	2.29204348	0.021903134	0.103264404	SNX24	protein_coding
ENSG00000064655.19	711.3488424	0.364183458	0.212619391	1.712842163	0.086741581	0.252416553	EYA2	protein_coding
ENSG00000064666.15	8030.409713	0.24599508	0.072709222	3.383272069	0.000716276	0.008900249	CNN2	protein_coding
ENSG00000064687.13	1799.641444	0.325518168	0.086050721	3.782863921	0.000155034	0.002799803	ABCA7	protein_coding
ENSG00000064692.20	529.8838209	0.66385593	0.186716153	3.555428496	0.000377364	0.005507157	SNCAIP	protein_coding
ENSG00000064703.12	1102.0475	-0.096092068	0.039755877	-2.417053131	0.015646733	0.082437152	DDX20	protein_coding
ENSG00000064726.10	4284.942809	0.117572603	0.045523473	2.582680846	0.009803597	0.059633072	BTBD1	protein_coding
ENSG00000064763.11	1570.923572	0.077612837	0.107986537	0.718726977	0.472309158	0.68332723	FAR2	protein_coding
ENSG00000064787.13	4091.449866	0.177899328	0.178204637	0.998286746	0.318140333	0.547578848	BCAS1	protein_coding
ENSG00000064835.11	3.932466758	0.019922932	0.146310885	0.136168488	0.891688086	0.94838162	POU1F1	protein_coding
ENSG00000064886.14	304.9884962	0.30179088	0.175364629	1.720933587	0.085262876	0.249865524	CHI3L2	protein_coding
ENSG00000064932.16	8003.670238	0.247704949	0.066934309	3.700717226	0.000214991	0.003584032	SBNO2	protein_coding
ENSG00000064933.18	1021.585032	-0.11786048	0.05240586	-2.248994286	0.02451286	0.111549382	PMS1	protein_coding
ENSG00000064961.19	3372.057078	0.088065295	0.055475007	1.587476969	0.112404659	0.295342648	HMG20B	protein_coding
ENSG00000064989.13	2218.135698	-0.05432702	0.099812726	-0.544289512	0.586242263	0.768529199	CALCRL	protein_coding
ENSG00000064995.18	1018.226629	0.050885828	0.049337029	1.031392215	0.30235693	0.531909268	TAF11	protein_coding
ENSG00000064999.15	4133.727125	0.114942514	0.059713292	1.924906674	0.054241035	0.187502901	ANKS1A	protein_coding
ENSG00000065000.19	12934.84095	0.106051925	0.043030972	2.464548697	0.013718592	0.07533739	AP3D1	protein_coding
ENSG00000065029.15	1435.427432	0.075995403	0.049754459	1.52740889	0.126659367	0.31757395	ZNF76	protein_coding
ENSG00000065054.14	4539.834802	0.092332247	0.079569516	1.16039725	0.245887106	0.472395991	SLC9A3R2	protein_coding
ENSG00000065057.9	905.3498876	-0.113023077	0.077774432	-1.453216353	0.146163693	0.346631016	NTHL1	protein_coding
ENSG00000065060.17	2254.18935	0.040826071	0.057314211	0.7123202	0.476266518	0.686295627	UHRF1BP1	protein_coding
ENSG00000065135.12	5741.842826	0.033137296	0.042031925	0.788383964	0.430472148	0.648532977	GNAI3	protein_coding
ENSG00000065150.21	11859.31914	0.097729295	0.062299967	1.568689361	0.116720339	0.302038136	IPO5	protein_coding
ENSG00000065154.12	7255.765725	-0.307186945	0.101701725	-3.020469373	0.002523832	0.022594731	OAT	protein_coding
ENSG00000065183.16	2167.700861	0.020672493	0.061366242	0.336870766	0.736214324	0.86320862	WDR3	protein_coding
ENSG00000065243.20	4319.468698	0.007180427	0.05665822	0.126732312	0.89915227	0.951977512	PKN2	protein_coding
ENSG00000065268.11	2574.461355	0.012016338	0.068942051	0.174296208	0.861632679	0.932645303	WDR18	protein_coding
ENSG00000065308.5	3954.702405	0.212923517	0.064170849	3.318072328	0.00090641	0.010640796	TRAM2	protein_coding
ENSG00000065320.9	2003.240245	-0.070949391	0.163252927	-0.43459797	0.663854268	0.820302202	NTN1	protein_coding
ENSG00000065325.13	133.1135437	0.077043873	0.184771411	0.416968581	0.676701389	0.828731564	GLP2R	protein_coding

ENSG00000065328.17	814.2945811	0.011976091	0.100923297	0.118665281	0.905540544	0.955049622	MCM10	protein_coding
ENSG00000065357.20	2295.874633	0.143529538	0.096838561	1.482152733	0.13829966	0.33492457	DGKA	protein_coding
ENSG00000065361.16	20829.88919	0.017382535	0.085134297	0.204177815	0.838214547	0.920420192	ERBB3	protein_coding
ENSG00000065371.17	2.617608722	0.650483426	0.262788468	2.475311912	0.013311986	0.07378484	ROPN1	protein_coding
ENSG00000065413.20	1076.260342	-0.411345155	0.105250362	-3.908254055	9.29655E-05	0.001871205	ANKRD44	protein_coding
ENSG00000065427.15	8264.918207	-0.038834554	0.052541598	-0.739120157	0.459834038	0.673058178	KARS1	protein_coding
ENSG00000065457.10	1449.901473	-0.028958247	0.049999612	-0.579169439	0.56247485	0.751274999	ADAT1	protein_coding
ENSG00000065485.20	2098.136305	-0.005628144	0.066977918	-0.084029846	0.933032702	0.968319466	PDIA5	protein_coding
ENSG00000065491.8	1123.227911	0.102331162	0.054078969	1.892254304	0.058457106	0.196527987	TBC1D22B	protein_coding
ENSG00000065518.8	6582.68576	-0.140511111	0.062887004	-2.234342594	0.02546054	0.114380819	NDUFB4	protein_coding
ENSG00000065526.12	9029.081041	0.060737754	0.045828714	1.32532093	0.18506473	0.399600475	SPEN	protein_coding
ENSG00000065534.19	22833.04777	-0.48455213	0.194590036	-2.49011789	0.012770073	0.071702289	MYLK	protein_coding
ENSG00000065548.18	4042.35927	-0.01380358	0.055783678	-0.247448368	0.804561246	0.902919531	ZC3H15	protein_coding
ENSG00000065559.15	1996.587096	-0.041861994	0.053481778	-0.782733765	0.43378347	0.651579824	MAP2K4	protein_coding
ENSG00000065600.13	556.3126472	0.033663242	0.057739022	0.58302411	0.55987707	0.749432133	PACC1	protein_coding
ENSG00000065609.14	47.11963174	-0.564485026	0.227549603	-2.480711977	0.013112027	0.073038837	SNAP91	protein_coding
ENSG00000065613.15	7968.85698	0.045879564	0.070129344	0.654213512	0.512974257	0.715524836	SLK	protein_coding
ENSG00000065615.14	1443.181314	0.11683	0.053193244	2.196331566	0.02806822	0.12188628	CYB5R4	protein_coding
ENSG00000065618.21	4449.640945	0.000782162	0.207476799	0.003769876	0.996992081	0.998504884	COL17A1	protein_coding
ENSG00000065621.15	1717.52238	-0.220788663	0.100447778	-2.198044276	0.027945951	0.121512742	GSTO2	protein_coding
ENSG00000065665.21	460.8446105	-0.067748576	0.06833384	-0.991435219	0.32147311	0.551246752	SEC61A2	protein_coding
ENSG00000065675.15	271.2244374	-0.52538053	0.134075504	-3.918542285	8.90861E-05	0.001814068	PRKCQ	protein_coding
ENSG00000065717.15	1543.558915	-0.081921126	0.110998045	-0.738041167	0.460489433	0.67353902	TLE2	protein_coding
ENSG00000065802.12	1203.95875	0.047208264	0.054287769	0.869593009	0.38452286	0.609379117	ASB1	protein_coding
ENSG00000065809.13	7246.720672	-0.017975237	0.111058849	-0.161853264	0.871421407	0.937947251	FAM107B	protein_coding
ENSG00000065833.9	1744.358866	0.051720253	0.11043923	0.468314141	0.639559957	0.804889513	ME1	protein_coding
ENSG00000065882.16	4679.314828	-0.125795236	0.068471273	-1.837197265	0.066180779	0.212165072	TBC1D1	protein_coding
ENSG00000065883.16	5045.187812	-0.045746257	0.038512565	-1.187826783	0.23490166	0.459625057	CDK13	protein_coding
ENSG00000065911.13	3731.68517	-0.010653459	0.07198718	-0.147991059	0.882349825	0.943462992	MTHFD2	protein_coding
ENSG00000065923.10	2249.229697	-0.33115308	0.098450311	-3.363657009	0.000769171	0.009431106	SLC9A7	protein_coding
ENSG00000065970.9	2334.012896	0.006477892	0.053560149	0.120946114	0.903733717	0.954142806	FOXJ2	protein_coding
ENSG00000065978.19	33232.08596	-0.09836354	0.051942754	-1.893691269	0.058266001	0.196206004	YBX1	protein_coding
ENSG00000065989.16	1870.472416	0.483164185	0.076377464	6.326004535	2.5159E-10	5.83168E-08	PDE4A	protein_coding
ENSG00000066027.12	4928.55843	0.007349798	0.047148221	0.155887072	0.876122036	0.940138031	PPP2R5A	protein_coding
ENSG00000066032.18	26.21653027	-0.927642154	0.237742674	-3.901874829	9.54505E-05	0.001906793	CTNNA2	protein_coding
ENSG00000066044.15	5211.549543	-0.043777416	0.038301178	-1.142978328	0.253047589	0.479875312	ELAVL1	protein_coding
ENSG00000066056.14	1598.57897	0.152691383	0.096848482	1.576600675	0.114887437	0.299178311	TIE1	protein_coding
ENSG00000066084.13	4864.130662	0.071341577	0.058490771	1.219706553	0.222576137	0.445060354	DIP2B	protein_coding
ENSG00000066117.15	4671.40972	-0.060775295	0.048206468	-1.260729057	0.207406481	0.427295397	SMARCD1	protein_coding
ENSG00000066135.13	995.9871802	-0.086569663	0.047324479	-1.82927876	0.067357859	0.214690858	KDM4A	protein_coding
ENSG00000066136.20	3043.060438	-0.019398826	0.037976723	-0.510808309	0.60948529	0.785158025	NFYC	protein_coding
ENSG00000066185.13	151.2977998	-0.20171326	0.098117598	-2.055831622	0.039798749	0.153563972	ZMYND12	protein_coding

ENSG00000066230.12	1698.249218	-0.544550668	0.227221118	-2.396567149	0.016549461	0.085731794	SLC9A3	protein_coding
ENSG00000066248.15	768.7998929	0.077114994	0.114682067	0.672424166	0.501313702	0.706510558	NGEF	protein_coding
ENSG00000066279.18	2782.382413	0.093161764	0.094540606	0.985415347	0.324420094	0.554249988	ASPM	protein_coding
ENSG00000066294.15	1492.809308	-0.323933334	0.1212877	-2.670784699	0.007567417	0.049699167	CD84	protein_coding
ENSG00000066322.15	6950.164878	-0.014811109	0.062158778	-0.238278634	0.811664992	0.90706071	ELOVL1	protein_coding
ENSG00000066336.12	2140.383996	-0.104392618	0.110616408	-0.943735374	0.345304892	0.574787093	SPI1	protein_coding
ENSG00000066379.15	785.7639442	-0.193542735	0.068959721	-2.806605546	0.005006651	0.037237553	POLR1H	protein_coding
ENSG00000066382.16	93.12084583	-0.099916045	0.135533643	-0.737204744	0.460997849	0.673893371	MPPED2	protein_coding
ENSG00000066405.13	45268.05002	-0.566204801	0.2303428	-2.45809637	0.013967569	0.076285084	CLDN18	protein_coding
ENSG00000066422.5	1927.291426	0.034864775	0.043349152	0.804278125	0.421236368	0.640839869	ZBTB11	protein_coding
ENSG00000066427.24	1391.039653	0.004058242	0.044266781	0.091676927	0.926954729	0.965460605	ATXN3	protein_coding
ENSG00000066455.13	3343.241097	0.134417266	0.040645539	3.30706077	0.000942804	0.010967167	GOLGA5	protein_coding
ENSG00000066468.23	3590.237478	-0.830301572	0.144489989	-5.746429746	9.11475E-09	1.10948E-06	FGFR2	protein_coding
ENSG00000066557.6	1050.933579	-0.046192056	0.045659693	-1.011659364	0.311700951	0.541262895	LRRC40	protein_coding
ENSG00000066583.12	1862.765563	0.007462202	0.059138152	0.126182528	0.899587441	0.952113582	ISOC1	protein_coding
ENSG00000066629.18	1523.797233	-0.081172618	0.132592876	-0.612194412	0.540409138	0.735919031	EML1	protein_coding
ENSG00000066651.20	981.7679508	0.047793157	0.063939648	0.747472939	0.454778132	0.668889788	TRMT11	protein_coding
ENSG00000066654.14	3192.972366	-0.085460135	0.043162225	-1.979975215	0.047706314	0.172737685	THUMPD1	protein_coding
ENSG00000066697.15	919.9339536	0.060881514	0.050407801	1.207779608	0.227132047	0.450547295	MSANTD3	protein_coding
ENSG00000066735.14	1361.580887	0.349485123	0.170606065	2.04849179	0.040511836	0.155292486	KIF26A	protein_coding
ENSG00000066739.12	2729.231479	0.036432829	0.056411199	0.645843895	0.518380458	0.719180681	ATG2B	protein_coding
ENSG00000066777.9	6086.584612	0.069606394	0.063635751	1.093825306	0.274031603	0.502587338	ARFGEF1	protein_coding
ENSG00000066813.14	9.642269432	-0.474577728	0.241616312	-1.964179175	0.049509317	0.176744697	ACSM2B	protein_coding
ENSG00000066827.16	704.816201	0.102545652	0.057280839	1.790226093	0.073417572	0.226724716	ZFAT	protein_coding
ENSG00000066855.16	2276.426528	-0.069285767	0.064373085	-1.076315778	0.281786055	0.51074748	MTFR1	protein_coding
ENSG00000066923.17	277.8402416	0.061158249	0.125378385	0.487789415	0.62569901	0.795834498	STAG3	protein_coding
ENSG00000066926.12	1233.311428	0.031586948	0.064560225	0.489263281	0.624655313	0.795177508	FECH	protein_coding
ENSG00000066933.16	2148.723925	0.017734267	0.066684396	0.265943274	0.790282897	0.894729214	MYO9A	protein_coding
ENSG00000067048.17	1216.459672	0.025420997	0.264150575	0.096236765	0.923332532	0.963825309	DDX3Y	protein_coding
ENSG00000067057.18	7334.732463	0.219153962	0.088704575	2.470604954	0.013488473	0.074497744	PFKP	protein_coding
ENSG00000067064.11	3309.39426	0.093576064	0.080003156	1.169654654	0.242139973	0.46801351	IDI1	protein_coding
ENSG00000067066.17	5958.804676	-0.07627347	0.058999732	-1.29277655	0.196088353	0.413197173	SP100	protein_coding
ENSG00000067082.15	13633.79182	0.187684189	0.071245256	2.634339464	0.008430116	0.053589957	KLF6	protein_coding
ENSG00000067113.17	1961.17243	-0.185669644	0.088145622	-2.106396658	0.035169911	0.141266405	PLPP1	protein_coding
ENSG00000067141.17	6153.275211	0.172487684	0.08775975	1.965453235	0.049361803	0.17650119	NEO1	protein_coding
ENSG00000067167.8	17894.3698	-0.134114038	0.06712208	-1.998061402	0.045710004	0.167965666	TRAM1	protein_coding
ENSG00000067177.15	956.4542827	0.294804155	0.111723286	2.638699286	0.008322477	0.053146039	PHKA1	protein_coding
ENSG00000067182.8	8093.212892	0.080438937	0.056811709	1.415886591	0.156808711	0.361739186	TNFRSF1A	protein_coding
ENSG00000067191.16	494.8730037	0.458987983	0.105732187	4.341043105	1.41808E-05	0.00042706	CACNB1	protein_coding
ENSG00000067208.14	1962.398926	-0.062573221	0.051213804	-1.221803806	0.221781829	0.444296091	EVI5	protein_coding
ENSG00000067221.14	674.8231604	0.062273703	0.065907575	0.944864132	0.344728245	0.574342812	STOML1	protein_coding
ENSG00000067225.18	68789.80767	0.237769567	0.075853944	3.134570929	0.001721056	0.017181781	PKM	protein_coding

ENSG00000067248.10	2246.800622	-0.058515901	0.046076349	-1.269976942	0.204092844	0.423471757	DHX29	protein_coding
ENSG00000067334.14	3058.499989	-0.019651381	0.040966072	-0.479698932	0.631441487	0.799828573	DNTTIP2	protein_coding
ENSG00000067365.15	716.1121537	0.035479098	0.042826614	0.828435757	0.407423763	0.628463511	METTL22	protein_coding
ENSG00000067369.14	2272.728931	0.082862655	0.056900354	1.456276603	0.145316171	0.345511528	TP53BP1	protein_coding
ENSG00000067445.21	229.7325641	0.345555726	0.115344093	2.995868409	0.002736645	0.02398045	TRO	protein_coding
ENSG00000067533.6	1732.723916	-0.055117194	0.054729668	-1.007080726	0.313895983	0.543148238	RRP15	protein_coding
ENSG00000067560.13	30324.43816	0.035429114	0.044504365	0.796081776	0.425984506	0.644687324	RHOA	protein_coding
ENSG00000067596.12	4051.573137	0.183782789	0.045036813	4.080723712	4.48957E-05	0.001057524	DHX8	protein_coding
ENSG00000067601.8	123.1045805	-0.16792189	0.059160854	-2.838395309	0.004534099	0.034748933	PMS2P4	transcribed_unprocessed_pseudogene
ENSG00000067606.17	1339.253233	-0.078956256	0.073740163	-1.070736111	0.284288101	0.513311224	PRKCZ	protein_coding
ENSG00000067646.12	428.3890409	-0.135266186	0.275757406	-0.490526031	0.623761714	0.794678697	ZFY	protein_coding
ENSG00000067704.10	8350.188342	-0.00641056	0.058851508	-0.108927705	0.913259832	0.959013054	IARS2	protein_coding
ENSG00000067715.14	489.5980868	0.305611597	0.195296998	1.564855584	0.117616775	0.303509526	SYT1	protein_coding
ENSG00000067798.16	218.3282522	0.200023186	0.149326938	1.339498338	0.180408495	0.393397898	NAV3	protein_coding
ENSG00000067829.19	4358.335698	-0.116417851	0.060163175	-1.935035042	0.052985996	0.184484152	IDH3G	protein_coding
ENSG00000067836.13	531.4329367	-0.126866721	0.06742027	-1.881729641	0.059872733	0.199668944	ROGDI	protein_coding
ENSG00000067840.12	251.1741029	-0.279537142	0.181279011	-1.542027068	0.123067016	0.312177156	PDZD4	protein_coding
ENSG00000067842.17	8.032724556	-0.265043358	0.208467486	-1.27138943	0.203590141	0.422960243	ATP2B3	protein_coding
ENSG00000067900.8	6179.860205	-0.040274258	0.056348129	-0.714739927	0.474769762	0.685203746	ROCK1	protein_coding
ENSG00000067955.15	3721.935282	0.036721082	0.057779188	0.635541686	0.525075188	0.724164006	CBFB	protein_coding
ENSG00000067992.16	3124.699438	-0.093598161	0.083002677	-1.127652312	0.259466785	0.486682888	PDK3	protein_coding
ENSG00000068001.14	2268.983214	0.111140771	0.05365155	2.07152954	0.038309337	0.149695657	HYAL2	protein_coding
ENSG00000068024.17	2083.245373	-0.06518056	0.088991141	-0.732438749	0.463900815	0.676100529	HDAC4	protein_coding
ENSG00000068028.17	1388.809178	0.018295875	0.052628237	0.347643701	0.728107783	0.858322589	RASSF1	protein_coding
ENSG00000068078.20	2921.520736	0.111934268	0.158740484	0.705140023	0.480723102	0.689720517	FGFR3	protein_coding
ENSG00000068079.8	2138.747706	0.027165154	0.089448682	0.303695412	0.761359954	0.87790492	IFI35	protein_coding
ENSG00000068097.15	1221.531135	0.085288808	0.049823177	1.71182998	0.086928005	0.252689059	HEATR6	protein_coding
ENSG00000068120.15	4495.875449	-0.01751211	0.059436067	-0.294637773	0.768270604	0.882557287	COASY	protein_coding
ENSG00000068137.15	1522.958212	0.138549579	0.068486037	2.023033966	0.043069646	0.16169051	PLEKHH3	protein_coding
ENSG00000068305.17	3937.965175	0.086394195	0.070210703	1.23049893	0.218510327	0.44012131	MEF2A	protein_coding
ENSG00000068308.14	4264.533819	-0.008781095	0.042529256	-0.20647188	0.836422328	0.919722528	OTUD5	protein_coding
ENSG00000068323.17	4234.451344	0.025784546	0.050974455	0.505832691	0.612974107	0.787464187	TFE3	protein_coding
ENSG00000068354.16	1074.244484	0.032446594	0.047891579	0.67750102	0.498088123	0.703959593	TBC1D25	protein_coding
ENSG00000068366.20	6111.106826	0.109131251	0.07185867	1.51869289	0.128839817	0.320880877	ACSL4	protein_coding
ENSG00000068383.19	1771.084865	-0.062685367	0.068542317	-0.914549871	0.360427985	0.588275603	INPP5A	protein_coding
ENSG00000068394.11	1266.417751	-0.02001653	0.048756053	-0.410544516	0.681406555	0.831335838	GPKOW	protein_coding
ENSG00000068400.13	2524.280559	-0.042072121	0.047090163	-0.893437566	0.371622893	0.598137103	GRIPAP1	protein_coding
ENSG00000068438.15	2864.162179	0.143595775	0.052568512	2.731592936	0.006302896	0.043772463	FTSJ1	protein_coding
ENSG00000068489.13	2522.969054	-0.017446905	0.08147269	-0.214144212	0.830434599	0.916570636	PRR11	protein_coding
ENSG00000068615.19	554.8406893	-0.217418707	0.173877309	-1.250414492	0.211148173	0.431598459	REEP1	protein_coding
ENSG00000068650.18	11140.75769	0.301849753	0.09274717	3.254544093	0.001135745	0.012661134	ATP11A	protein_coding
ENSG00000068654.16	5549.587887	-0.045100854	0.050657987	-0.890300955	0.373304309	0.599413459	POLR1A	protein_coding

ENSG00000068697.7	17494.86516	0.001687457	0.045905085	0.036759699	0.970676608	0.98620164	LAPTM4A	protein_coding
ENSG00000068724.17	3309.124696	-0.004969312	0.055543387	-0.08946722	0.928710604	0.966180963	TTC7A	protein_coding
ENSG00000068745.15	3210.169574	-0.083427023	0.051538425	-1.618734426	0.105504416	0.284310314	IP6K2	protein_coding
ENSG00000068781.21	5.394483551	-0.066634504	0.208388726	-0.319760598	0.749149818	0.871072946	STON1-GTF2A1L	protein_coding
ENSG00000068784.13	1125.70673	-0.009143152	0.044019369	-0.20770748	0.835457377	0.919344369	SRBD1	protein_coding
ENSG00000068796.19	2610.757233	-0.0425875	0.05822198	-0.731467741	0.464493502	0.676489199	KIF2A	protein_coding
ENSG00000068831.19	497.3520224	-0.656973911	0.146199785	-4.493672205	7.00054E-06	0.00024503	RASGRP2	protein_coding
ENSG00000068878.15	5864.104694	0.075866884	0.048290086	1.571065413	0.116167455	0.301190265	PSME4	protein_coding
ENSG00000068885.15	679.2195278	0.078899994	0.066333673	1.189441052	0.234266155	0.458873003	IFT80	protein_coding
ENSG00000068903.20	3005.530398	0.012442104	0.057478781	0.216464306	0.828625845	0.915625119	SIRT2	protein_coding
ENSG00000068912.14	4503.066535	0.131200274	0.047338877	2.771512145	0.005579659	0.040222465	ERLEC1	protein_coding
ENSG00000068971.14	1187.447982	0.22147433	0.056947556	3.889092766	0.00010062	0.001993683	PPP2R5B	protein_coding
ENSG00000068976.14	227.1495786	-0.700157533	0.178670674	-3.918704279	8.90263E-05	0.001813697	PYGM	protein_coding
ENSG00000068985.5	14.40288348	0.430210373	0.330896133	1.300137204	0.193553949	0.410088028	PAGE1	protein_coding
ENSG00000069011.16	4791.42707	0.398482672	0.11605136	3.433675166	0.000595457	0.007752687	PITX1	protein_coding
ENSG00000069018.19	14.01910762	-0.22462208	0.193107746	-1.163195597	0.244750152	0.471027576	TRPC7	protein_coding
ENSG00000069020.18	2894.987386	0.148823497	0.090329802	1.647556992	0.099443612	0.274658374	MAST4	protein_coding
ENSG00000069122.19	2334.315693	0.042993012	0.085787518	0.501156963	0.61626066	0.789591332	ADGRF5	protein_coding
ENSG00000069188.17	563.0020204	0.550966434	0.171267403	3.216995315	0.001295407	0.013958941	SDK2	protein_coding
ENSG00000069206.15	2.711651377	0.016828812	0.370047426	0.045477446	0.963726752	0.983375587	ADAM7	protein_coding
ENSG00000069248.12	2964.971212	0.031896899	0.046401656	0.687408622	0.491825266	0.69887076	NUP133	protein_coding
ENSG00000069275.13	18649.58422	0.002401561	0.055307557	0.043421941	0.965365188	0.984247416	NUCKS1	protein_coding
ENSG00000069329.18	11050.50661	-0.078103043	0.046005326	-1.697695664	0.089565217	0.257452734	VPS35	protein_coding
ENSG00000069345.12	3638.718437	-0.047679473	0.040369206	-1.181085246	0.23756886	0.462766002	DNAJA2	protein_coding
ENSG00000069399.15	9324.051469	0.179697076	0.073673568	2.439098319	0.014723962	0.079086168	BCL3	protein_coding
ENSG00000069424.15	2495.164399	-0.325250344	0.091140748	-3.568660013	0.000358812	0.005321966	KCNAB2	protein_coding
ENSG00000069431.11	1303.140795	0.047386732	0.156993612	0.301838597	0.762775106	0.878851309	ABCC9	protein_coding
ENSG00000069482.7	662.4101781	0.40689186	0.241243824	1.686641565	0.091672295	0.26103003	GAL	protein_coding
ENSG00000069493.15	548.4782507	-0.686295317	0.113832423	-6.028996826	1.64981E-09	2.75454E-07	CLEC2D	protein_coding
ENSG00000069509.6	862.3797693	-0.271374088	0.071410888	-3.800178024	0.000144592	0.002641884	FUNDC1	protein_coding
ENSG00000069535.14	1860.731418	-0.317367768	0.163441471	-1.941782377	0.052163451	0.182683172	MAOB	protein_coding
ENSG00000069667.16	1015.535778	0.054761522	0.09953008	0.550200722	0.582181708	0.765673021	RORA	protein_coding
ENSG00000069696.7	102.8573689	0.018113319	0.111119368	0.163007761	0.870512324	0.937459917	DRD4	protein_coding
ENSG00000069702.11	2488.47352	-0.322996809	0.125600861	-2.57161301	0.010122597	0.060977664	TGFBR3	protein_coding
ENSG00000069764.10	479.7787809	0.055754779	0.157102622	0.354894005	0.722669	0.855233249	PLA2G10	protein_coding
ENSG00000069812.11	348.9493164	0.99848138	0.182875191	5.459906153	4.76386E-08	4.42633E-06	HES2	protein_coding
ENSG00000069849.11	8147.867731	-0.075252554	0.063575219	-1.183677466	0.236540762	0.46156801	ATP1B3	protein_coding
ENSG00000069869.16	1404.537319	0.062903926	0.080294264	0.78341743	0.433382024	0.651336563	NEDD4	protein_coding
ENSG00000069943.10	627.7848287	-0.046435834	0.049492573	-0.938238442	0.348121887	0.577388567	PIGB	protein_coding
ENSG00000069956.12	4811.832503	0.185535363	0.064143584	2.892500729	0.003821883	0.030702853	MAPK6	protein_coding
ENSG00000069966.19	960.8551305	0.142556968	0.068630031	2.077180594	0.037784894	0.148311326	GNB5	protein_coding
ENSG00000069974.16	2804.664019	-0.254030366	0.078855258	-3.22147658	0.001275319	0.013814211	RAB27A	protein_coding

ENSG00000069998.12	1899.843844	-0.083129007	0.064229317	-1.294253337	0.195577929	0.412601637	HDHD5	protein_coding
ENSG00000070010.19	2582.590561	-0.062665201	0.045711802	-1.370875767	0.170413687	0.380421288	UFD1	protein_coding
ENSG00000070018.9	3345.028996	-0.02353147	0.062727573	-0.375137582	0.707558148	0.846857939	LRP6	protein_coding
ENSG00000070019.5	1926.228827	-0.082731941	0.218611895	-0.378442081	0.705102212	0.845451369	GUCY2C	protein_coding
ENSG00000070031.4	27.90984804	0.704809093	0.163324366	4.315394626	1.59318E-05	0.000466588	SCT	protein_coding
ENSG00000070047.13	4255.903014	-0.004502121	0.057518006	-0.078273248	0.937610697	0.970434325	PHRF1	protein_coding
ENSG00000070061.16	2817.370626	-0.034490609	0.04837515	-0.712981957	0.475856922	0.686039211	ELP1	protein_coding
ENSG00000070081.17	3645.909776	0.016268134	0.072643243	0.223945584	0.822799645	0.912575695	NUCB2	protein_coding
ENSG00000070087.14	3056.41441	0.459113796	0.137200148	3.346306864	0.000818957	0.009847597	PFN2	protein_coding
ENSG00000070159.14	2626.024579	0.099536055	0.086567938	1.149802773	0.250225113	0.477034725	PTPN3	protein_coding
ENSG00000070182.21	148.3750499	0.804499043	0.121829024	6.603508913	4.01538E-11	1.26796E-08	SPTB	protein_coding
ENSG00000070190.13	657.1889048	-0.103524818	0.119083973	-0.869342997	0.384659552	0.609516355	DAPP1	protein_coding
ENSG00000070193.5	356.3024289	-0.335802189	0.196792615	-1.706375972	0.087938101	0.254711095	FGF10	protein_coding
ENSG00000070214.16	11375.98543	0.077278411	0.057240723	1.350060014	0.176996733	0.38922783	SLC44A1	protein_coding
ENSG00000070269.14	1058.143791	-0.041815757	0.054964292	-0.760780432	0.446788224	0.662301951	TMEM260	protein_coding
ENSG00000070366.14	1557.346014	0.056071993	0.056839677	0.986493877	0.323890818	0.553606205	SMG6	protein_coding
ENSG00000070367.16	3647.105358	0.092279221	0.046881414	1.968354054	0.049027314	0.175710444	EXOC5	protein_coding
ENSG00000070371.16	303.0603381	0.417356205	0.106916695	3.903564392	9.47863E-05	0.001896466	CLTCL1	protein_coding
ENSG00000070388.11	12.46438304	0.350259315	0.113511816	3.085663928	0.002030982	0.019349392	FGF22	protein_coding
ENSG00000070404.10	2497.612177	0.816885146	0.128995226	6.332677364	2.40943E-10	5.61475E-08	FSTL3	protein_coding
ENSG00000070413.20	7666.16989	0.095507516	0.055656253	1.716024901	0.086157485	0.251388331	DGCR2	protein_coding
ENSG00000070423.18	3814.969859	-0.004304637	0.062246938	-0.069154195	0.944866883	0.97410169	RNF126	protein_coding
ENSG00000070444.15	2006.511357	0.203920527	0.047178442	4.32232434	1.54394E-05	0.000456757	MNT	protein_coding
ENSG00000070476.15	2124.631166	0.130518468	0.050020148	2.609317921	0.009072291	0.056405084	ZXDC	protein_coding
ENSG00000070495.15	1530.695444	0.0609007	0.04620441	1.318071169	0.187479825	0.402551668	JMJD6	protein_coding
ENSG00000070501.12	925.1362672	-0.033038968	0.066321746	-0.498161918	0.618369922	0.79101468	POLB	protein_coding
ENSG00000070526.15	3780.731593	0.032789992	0.153565908	0.213523901	0.830918349	0.916725567	ST6GALNAC1	protein_coding
ENSG00000070540.13	1165.26858	0.354721679	0.075998121	4.667505892	3.04878E-06	0.000125811	WIPI1	protein_coding
ENSG00000070601.10	57.02609837	0.212680659	0.191800848	1.108861928	0.26748975	0.495447819	FRMPD1	protein_coding
ENSG00000070610.14	3728.480681	0.003462155	0.063571061	0.054461181	0.956567736	0.979770735	GBA2	protein_coding
ENSG00000070614.15	6729.661107	0.109313865	0.070012029	1.561358334	0.118439227	0.304856834	NDST1	protein_coding
ENSG00000070669.17	68.32662422	-0.034124082	0.096247543	-0.354544967	0.722930511	0.855366355	ASNS	protein_coding
ENSG00000070718.12	1159.002821	0.024806094	0.066817953	0.371248946	0.71045212	0.848559431	AP3M2	protein_coding
ENSG00000070729.14	30.10018988	-0.248951899	0.180173141	-1.381737025	0.167052462	0.375820605	CNGB1	protein_coding
ENSG00000070731.11	183.4709235	0.421402068	0.138128973	3.050786937	0.002282425	0.021032189	ST6GALNAC2	protein_coding
ENSG00000070748.19	9.072153606	-0.381765745	0.267449145	-1.427433037	0.153455114	0.357084077	CHAT	protein_coding
ENSG00000070756.17	137497.5686	0.000625596	0.06856204	0.009124531	0.992719778	0.996561768	PABPC1	protein_coding
ENSG00000070759.17	342.3969915	-0.10377386	0.064041034	-1.620427623	0.10514045	0.283827381	TESK2	protein_coding
ENSG00000070761.8	1189.93595	-0.007498719	0.052981433	-0.141534839	0.88744744	0.94618631	CFAP20	protein_coding
ENSG00000070770.10	2136.531701	0.013408784	0.043885558	0.305539784	0.759955074	0.877090887	CSNK2A2	protein_coding
ENSG00000070778.13	1448.649584	0.036619222	0.066173508	0.553381908	0.580001925	0.763979805	PTPN21	protein_coding
ENSG00000070785.17	902.6930717	-0.09402789	0.043538609	-2.159643854	0.030800251	0.129779784	EIF2B3	protein_coding

ENSG00000070808.16	47.04055364	-0.287540134	0.158657428	-1.812333259	0.069934719	0.219706238	CAMK2A	protein_coding
ENSG00000070814.22	5651.876691	-0.04911442	0.063378559	-0.77493747	0.43837661	0.655204332	TCOF1	protein_coding
ENSG00000070831.17	15874.38016	-0.117536811	0.045764929	-2.568272562	0.010220676	0.061347987	CDC42	protein_coding
ENSG00000070882.13	3897.955023	-0.131938194	0.0696252	-1.894977594	0.05809537	0.195809571	OSBPL3	protein_coding
ENSG00000070886.12	18.42733219	1.018660648	0.261361215	3.897520322	9.71827E-05	0.001935525	EPHA8	protein_coding
ENSG00000070915.10	24.00202062	-0.366194215	0.162961115	-2.247126343	0.024631954	0.111939373	SLC12A3	protein_coding
ENSG00000070950.10	1237.148411	-0.002593396	0.062750011	-0.04132902	0.967033599	0.984565164	RAD18	protein_coding
ENSG00000070961.16	4809.688269	-0.096004088	0.067292974	-1.426658423	0.153678375	0.357374597	ATP2B1	protein_coding
ENSG00000070985.13	78.8262802	0.064227759	0.198333105	0.323837816	0.746060825	0.869303793	TRPM5	protein_coding
ENSG00000071051.14	5357.365596	-0.09293846	0.060647055	-1.532448023	0.125411915	0.315696831	NCK2	protein_coding
ENSG00000071054.16	9394.758483	0.075163987	0.064007366	1.174302144	0.240274025	0.465650586	MAP4K4	protein_coding
ENSG00000071073.13	3834.446954	-0.052774717	0.093260063	-0.56588764	0.571470165	0.757664005	MGAT4A	protein_coding
ENSG00000071082.11	28989.74919	-0.254184178	0.068978443	-3.684979928	0.000228721	0.003772506	RPL31	protein_coding
ENSG00000071127.17	20598.45651	0.047478889	0.051208526	0.927167661	0.353839491	0.58214201	WDR1	protein_coding
ENSG00000071189.21	3342.549159	0.023701149	0.051953999	0.456194903	0.64824983	0.810176306	SNX13	protein_coding
ENSG00000071203.9	4.31082666	-0.124183102	0.30020856	-0.4136561	0.679125982	0.830014248	MS4A12	protein_coding
ENSG00000071205.12	1133.48562	-0.076674206	0.101103345	-0.758374569	0.448226778	0.663306221	ARHGAP10	protein_coding
ENSG00000071242.12	2672.62872	0.263697663	0.078073888	3.377539781	0.000731374	0.00905943	RPS6KA2	protein_coding
ENSG00000071243.16	647.9166636	-0.140326507	0.048774895	-2.877023226	0.00401446	0.031853267	ING3	protein_coding
ENSG00000071246.11	1230.982763	0.139846863	0.085779444	1.630307404	0.103036544	0.280472674	VASH1	protein_coding
ENSG00000071282.12	1552.515523	0.224280563	0.085429946	2.625315513	0.008656871	0.054629597	LMCD1	protein_coding
ENSG00000071462.12	4771.763005	-0.143280637	0.056219801	-2.548579581	0.010816261	0.063841282	BUD23	protein_coding
ENSG00000071537.14	8692.688584	-0.002433215	0.056107042	-0.04336738	0.96540868	0.98426873	SEL1L	protein_coding
ENSG00000071539.14	1515.329851	0.106193048	0.102803998	1.032966136	0.301619747	0.531251109	TRIP13	protein_coding
ENSG00000071553.18	10516.60901	0.086584516	0.058107719	1.490069099	0.136206068	0.332061717	ATP6AP1	protein_coding
ENSG00000071564.17	6986.604802	0.08237937	0.065933024	1.249440193	0.211504115	0.43189854	TCF3	protein_coding
ENSG00000071575.11	3206.148739	0.410539924	0.099920693	4.108657669	3.97965E-05	0.000957072	TRIB2	protein_coding
ENSG00000071626.17	7624.847098	-0.050535234	0.045142885	-1.119450691	0.262947916	0.490601187	DAZAP1	protein_coding
ENSG00000071655.18	2010.013904	-0.015453768	0.06488609	-0.238167662	0.811751058	0.907099176	MBD3	protein_coding
ENSG00000071677.1	0.985509851	0.657197258	0.34017743	1.931924932	0.053368772	0.185355143	PRLH	protein_coding
ENSG00000071794.16	1354.913891	0.17450639	0.112311071	1.553777272	0.120237554	0.307921478	HLTF	protein_coding
ENSG00000071859.15	3621.643973	-0.007262979	0.057615142	-0.126060247	0.899684235	0.95213662	FAM50A	protein_coding
ENSG00000071889.17	2521.046343	-0.066980759	0.071624066	-0.935171141	0.349700108	0.578837382	FAM3A	protein_coding
ENSG00000071894.17	8241.368371	-0.070558831	0.059847709	-1.178972964	0.238408942	0.463801181	CPSF1	protein_coding
ENSG00000071909.19	54.06821045	-0.226567384	0.150077166	-1.509672587	0.13112699	0.32435371	MYO3B	protein_coding
ENSG00000071967.12	6458.627484	0.256963444	0.130077127	1.975469855	0.048214852	0.173928694	CYBRD1	protein_coding
ENSG00000071991.9	171.3445964	-0.079938842	0.240618021	-0.332223003	0.739720875	0.865503614	CDH19	protein_coding
ENSG00000071994.11	2966.39159	-0.052791843	0.051979831	-1.015621659	0.309809587	0.539267919	PDCD2	protein_coding
ENSG00000072041.17	21.36373837	0.263552628	0.255479226	1.031601012	0.302259066	0.531903211	SLC6A15	protein_coding
ENSG00000072042.13	3724.207788	-0.02235878	0.058268522	-0.3837197	0.70118621	0.842866365	RDH11	protein_coding
ENSG00000072062.15	4622.009907	-0.023852427	0.048216513	-0.494694158	0.620816026	0.792552276	PRKACA	protein_coding
ENSG00000072071.16	3683.588425	0.110217899	0.09032303	1.220263536	0.222364988	0.444897563	ADGRL1	protein_coding

ENSG00000072080.11	1.422631852	0.049123265	0.390429192	0.125818628	0.899875496	0.952138522	SPP2	protein_coding
ENSG00000072110.15	26284.08169	0.001473796	0.096124148	0.015332218	0.987767139	0.994154991	ACTN1	protein_coding
ENSG00000072121.17	2717.828181	0.026403196	0.052726434	0.50075824	0.616541279	0.789770128	ZFYVE26	protein_coding
ENSG00000072133.11	139.7357634	-0.354054554	0.202162064	-1.751340223	0.079887323	0.239937272	RPS6KA6	protein_coding
ENSG00000072134.15	1195.745013	0.198921858	0.066159029	3.006722773	0.002640805	0.023380404	EPN2	protein_coding
ENSG00000072135.13	6067.853076	-0.046028	0.060495635	-0.760848294	0.446747685	0.662286915	PTPN18	protein_coding
ENSG00000072163.20	2343.471354	-0.294526741	0.156261616	-1.884831015	0.059452656	0.198688765	LIMS2	protein_coding
ENSG00000072182.13	9.962992338	-0.057014607	0.146010084	-0.390484035	0.696178658	0.839696017	ASIC4	protein_coding
ENSG00000072195.15	1695.977468	-0.313578133	0.201853285	-1.55349532	0.120304847	0.308021404	SPEG	protein_coding
ENSG00000072201.14	705.001715	-0.032885904	0.091910512	-0.357803511	0.720490363	0.853921658	LNX1	protein_coding
ENSG00000072210.19	5362.872066	-0.235651633	0.072416971	-3.254094025	0.001137546	0.012671486	ALDH3A2	protein_coding
ENSG00000072274.13	14960.66389	0.222163005	0.101284744	2.193449823	0.028274986	0.122395834	TFRC	protein_coding
ENSG00000072310.17	9329.160402	0.480830217	0.089565029	5.368504003	7.93924E-08	6.74402E-06	SREBF1	protein_coding
ENSG00000072315.3	4.047948899	-0.281653518	0.222631863	-1.265108751	0.205832346	0.425581522	TRPC5	protein_coding
ENSG00000072364.13	8101.021521	0.151279944	0.061590041	2.456240354	0.014039922	0.07653461	AFF4	protein_coding
ENSG00000072401.15	2074.854068	-0.022103493	0.049478232	-0.446731678	0.655068807	0.814554302	UBE2D1	protein_coding
ENSG00000072415.10	2130.143945	0.048027918	0.04735648	1.014178386	0.31049764	0.539978869	MPP5	protein_coding
ENSG00000072422.17	1458.805852	0.230855551	0.094209126	2.450458472	0.014267442	0.07739282	RHOBTB1	protein_coding
ENSG00000072501.19	9168.549015	-0.074459113	0.058785047	-1.266633548	0.205286351	0.42485578	SMC1A	protein_coding
ENSG00000072506.13	3063.851997	-0.133797895	0.060391844	-2.215496107	0.026726031	0.118129655	HSD17B10	protein_coding
ENSG00000072518.21	5896.43558	0.050277731	0.051197378	0.982037222	0.326081511	0.555889758	MARK2	protein_coding
ENSG00000072571.20	1491.42149	-0.124033292	0.094540261	-1.311962665	0.189532732	0.405039166	HMMR	protein_coding
ENSG00000072609.18	1377.139361	-0.067054702	0.091044755	-0.736502629	0.461424868	0.674092711	CHFR	protein_coding
ENSG00000072657.9	196.766924	-0.664893201	0.190871293	-3.483463599	0.00049497	0.006748851	TRHDE	protein_coding
ENSG00000072682.19	2587.42486	0.257082293	0.06580305	3.906844665	9.35092E-05	0.001878677	P4HA2	protein_coding
ENSG00000072694.21	359.3324404	-0.068891485	0.127494644	-0.540348077	0.588957009	0.770555854	FCGR2B	protein_coding
ENSG00000072736.19	1686.609146	0.011481498	0.061629553	0.186298581	0.852210621	0.927765651	NFATC3	protein_coding
ENSG00000072756.17	876.8900026	0.056306266	0.045882134	1.227193709	0.219749793	0.441646093	TRNT1	protein_coding
ENSG00000072778.20	10235.80482	-0.043784023	0.056017444	-0.781614072	0.434441414	0.652163475	ACADVL	protein_coding
ENSG00000072786.13	2944.203199	-0.005321256	0.066403349	-0.080135351	0.936129607	0.969767071	STK10	protein_coding
ENSG00000072803.17	2901.962973	0.057263592	0.05547663	1.03221107	0.30197325	0.531658855	FBXW11	protein_coding
ENSG00000072818.12	790.5110605	-0.521746368	0.108701405	-4.799812574	1.58814E-06	7.4737E-05	ACAP1	protein_coding
ENSG00000072832.15	697.9907507	0.273991737	0.11991855	2.284815296	0.02232366	0.104609289	CRMP1	protein_coding
ENSG00000072840.13	1232.311719	0.297042546	0.126202664	2.353694729	0.018587869	0.092408006	EVC	protein_coding
ENSG00000072849.11	2017.824718	-0.024751116	0.042206857	-0.586424037	0.557590595	0.747932091	DERL2	protein_coding
ENSG00000072858.11	716.9946702	-0.203462244	0.13472273	-1.510229522	0.130984868	0.324219607	SIDT1	protein_coding
ENSG00000072864.16	2249.734144	-0.02044119	0.057833696	-0.353447754	0.723752791	0.855775133	NDE1	protein_coding
ENSG00000072952.19	3989.194362	-0.078586122	0.149257306	-0.526514406	0.598530845	0.777604676	IRAG1	protein_coding
ENSG00000072954.7	776.8822328	-0.201232793	0.121866704	-1.651253263	0.098686871	0.273315646	TMEM38A	protein_coding
ENSG00000072958.9	2808.208936	-0.022634548	0.05402766	-0.418943709	0.675257272	0.827563803	AP1M1	protein_coding
ENSG00000073008.15	4686.449781	0.0236671	0.084531939	0.279978203	0.779494228	0.888424438	PVR	protein_coding
ENSG00000073050.12	1622.115136	-0.137652183	0.053649018	-2.565791293	0.010294075	0.061682309	XRCC1	protein_coding

ENSG00000073060.16	4037.344171	0.028809492	0.093065208	0.309562426	0.756893734	0.875256295	SCARB1	protein_coding
ENSG00000073067.14	2262.94722	-0.575294184	0.257469887	-2.23441347	0.02545588	0.114380819	CYP2W1	protein_coding
ENSG00000073111.14	2653.600805	0.050326164	0.096042484	0.523998984	0.600279246	0.778679842	MCM2	protein_coding
ENSG00000073146.16	52.55897834	0.349912699	0.135141061	2.589240421	0.009618791	0.058750695	MOV10L1	protein_coding
ENSG00000073150.14	163.1725439	1.016638917	0.18340541	5.543123938	2.97122E-08	2.96966E-06	PANX2	protein_coding
ENSG00000073169.14	1315.709055	-0.127369934	0.053757882	-2.369325747	0.01782055	0.08985568	SELENOO	protein_coding
ENSG00000073282.14	118.9049851	0.240575183	0.182503105	1.318197757	0.187437456	0.402481622	TP63	protein_coding
ENSG00000073331.18	1337.190413	-0.046979661	0.071344107	-0.658493934	0.510220794	0.713493727	ALPK1	protein_coding
ENSG00000073350.14	13137.21899	0.135547843	0.079523696	1.704496266	0.088288413	0.255230475	LLGL2	protein_coding
ENSG00000073417.15	3112.564072	0.076380884	0.056731854	1.346349162	0.178189938	0.390811194	PDE8A	protein_coding
ENSG00000073464.13	1059.887211	0.115766954	0.132239475	0.87543416	0.38133772	0.606694747	CLCN4	protein_coding
ENSG00000073536.18	1364.324393	0.001401007	0.064492871	0.021723434	0.982668571	0.992397797	NLE1	protein_coding
ENSG00000073578.17	840.2178525	-0.191299869	0.078298993	-2.443197054	0.014557786	0.078425596	SDHA	protein_coding
ENSG00000073584.20	615.0581449	0.013981146	0.090176555	0.155041914	0.876788274	0.94050689	SMARCE1	protein_coding
ENSG00000073598.6	4.763419559	0.044767319	0.135120728	0.331313481	0.740407709	0.86582082	FNDC8	protein_coding
ENSG00000073605.18	4790.232812	-0.348704041	0.138820068	-2.511913766	0.012007843	0.068526797	GSDMB	protein_coding
ENSG00000073614.13	4468.425492	0.013371688	0.047737488	0.280108744	0.779394076	0.888395435	KDM5A	protein_coding
ENSG00000073670.14	49.33963101	0.058598936	0.130829632	0.447902626	0.654223473	0.81408042	ADAM11	protein_coding
ENSG00000073711.11	949.4527492	0.061447193	0.115722268	0.530988492	0.595426754	0.775189053	PPP2R3A	protein_coding
ENSG00000073712.15	3919.388828	-0.108305925	0.134522464	-0.805114042	0.420753874	0.640485959	FERMT2	protein_coding
ENSG00000073734.10	42.75438513	0.333563999	0.187755234	1.776589615	0.075635786	0.231459951	ABCB11	protein_coding
ENSG00000073737.17	637.9665788	1.331821078	0.215477706	6.180783631	6.37842E-10	1.22446E-07	DHRS9	protein_coding
ENSG00000073754.6	5.07715164	-0.626897291	0.291532033	-2.15035475	0.031527165	0.131800643	CD5L	protein_coding
ENSG00000073756.12	2031.527965	0.745178718	0.168409928	4.424790899	9.65357E-06	0.000315348	PTGS2	protein_coding
ENSG00000073792.16	4334.989076	0.079952599	0.108086275	0.739710931	0.459475414	0.672801079	IGF2BP2	protein_coding
ENSG00000073803.14	3660.481927	-0.008410532	0.063566948	-0.132309824	0.894739237	0.949980551	MAP3K13	protein_coding
ENSG00000073849.16	7848.096053	-0.144958518	0.110549567	-1.311253594	0.189772101	0.405297924	ST6GAL1	protein_coding
ENSG00000073861.3	89.0120409	-0.562702344	0.12682562	-4.436819188	9.12979E-06	0.000301858	TBX21	protein_coding
ENSG00000073905.8	58.80766396	-0.12186057	0.086133789	-1.414782414	0.157132302	0.362159746	VDAC1P1	processed_pseudogene
ENSG00000073910.22	1618.236741	0.274887364	0.099767809	2.755271125	0.005864352	0.041647793	FRY	protein_coding
ENSG00000073921.18	12825.56584	0.126242542	0.045668319	2.764335221	0.005703891	0.04086788	PICALM	protein_coding
ENSG00000073969.18	3722.372278	0.059036287	0.059731564	0.988359972	0.322976384	0.552867261	NSF	protein_coding
ENSG00000074047.22	910.0474622	0.677656849	0.121137726	5.594102447	2.21766E-08	2.30642E-06	GLI2	protein_coding
ENSG00000074054.20	3853.060492	0.009513299	0.043938735	0.216512815	0.828588037	0.915617622	CLASP1	protein_coding
ENSG00000074071.15	3025.449653	-0.212602345	0.066835511	-3.180978838	0.001467784	0.015306159	MRPS34	protein_coding
ENSG00000074181.9	14818.72324	0.445627675	0.090532605	4.922289333	8.55376E-07	4.60751E-05	NOTCH3	protein_coding
ENSG00000074201.9	4946.29256	-0.114153165	0.058775729	-1.94218203	0.052115068	0.182616461	CLNS1A	protein_coding
ENSG00000074211.14	396.3280429	0.913622437	0.245582464	3.720226696	0.000199044	0.003377625	PPP2R2C	protein_coding
ENSG00000074219.14	1241.442763	0.17104334	0.104393999	1.638440356	0.101329867	0.277644089	TEAD2	protein_coding
ENSG00000074266.22	1229.857202	-0.049311472	0.051645708	-0.954802906	0.339677378	0.569565607	EED	protein_coding
ENSG00000074276.10	3315.06362	0.15608474	0.19385101	0.805178887	0.420716458	0.640485959	CDHR2	protein_coding
ENSG00000074317.11	8.505881723	0.596708606	0.201040664	2.968099054	0.002996477	0.025638622	SNCB	protein_coding

ENSG00000074319.13	3869.301405	0.00457792	0.040162048	0.113986216	0.90924872	0.957061147	TSG101	protein_coding
ENSG00000074356.17	3057.899088	0.03873597	0.045455714	0.852169433	0.39412006	0.617656644	NCBP3	protein_coding
ENSG00000074370.18	11032.7035	-0.013551476	0.124923557	-0.108478148	0.913616414	0.959160308	ATP2A3	protein_coding
ENSG00000074410.14	3420.515517	0.28073695	0.169673424	1.654572316	0.098011283	0.272186375	CA12	protein_coding
ENSG00000074416.14	8657.331963	0.329175429	0.101463699	3.244267962	0.001177529	0.013000554	MGLL	protein_coding
ENSG00000074527.13	1927.027598	0.239972636	0.105671381	2.270933098	0.023151028	0.10736281	NTN4	protein_coding
ENSG00000074582.15	1138.481549	-0.162299675	0.053828139	-3.015145561	0.00256856	0.022917715	BCS1L	protein_coding
ENSG00000074590.13	1215.501021	0.501752306	0.082857847	6.055579788	1.39913E-09	2.37237E-07	NUAK1	protein_coding
ENSG00000074603.19	3308.622782	0.000390767	0.040558302	0.00963469	0.992312748	0.996451577	DPP8	protein_coding
ENSG00000074621.14	889.071138	0.033712049	0.061206193	0.55079473	0.581774397	0.76534288	SLC24A1	protein_coding
ENSG00000074657.14	3355.380959	0.138204589	0.083400291	1.657123579	0.097494493	0.27131474	ZNF532	protein_coding
ENSG00000074660.16	646.6492441	-0.021139092	0.074392804	-0.284155059	0.776291554	0.886582898	SCARF1	protein_coding
ENSG00000074695.6	9303.436798	0.096590094	0.070726612	1.36568248	0.172038646	0.382632985	LMAN1	protein_coding
ENSG00000074696.13	9225.295497	-0.086629389	0.065652629	-1.319511349	0.186998219	0.401962874	HACD3	protein_coding
ENSG00000074706.13	196.4613839	-0.570892202	0.11633238	-4.907423025	9.22809E-07	4.87433E-05	IPCEF1	protein_coding
ENSG00000074755.15	5478.110359	-0.019912253	0.061601471	-0.323243144	0.746511109	0.86953754	ZZEF1	protein_coding
ENSG00000074771.4	3.031502965	-0.173755545	0.346327097	-0.501709355	0.615871983	0.7893022	NOX3	protein_coding
ENSG00000074800.16	48858.65576	0.01685099	0.077399025	0.217715797	0.827650554	0.915154502	ENO1	protein_coding
ENSG00000074803.20	9.242108226	-0.201556524	0.216030592	-0.932999915	0.350820012	0.579836592	SLC12A1	protein_coding
ENSG00000074842.8	6323.348342	0.068279753	0.066945026	1.019937661	0.307758027	0.537155908	MYDGF	protein_coding
ENSG00000074855.11	986.9070169	0.185929886	0.066727888	2.786389487	0.005329879	0.038898029	ANO8	protein_coding
ENSG00000074935.14	861.2740661	-0.108945134	0.063417618	-1.717900139	0.085814831	0.25079334	TUBE1	protein_coding
ENSG00000074964.17	4432.081746	-0.021204469	0.088288911	-0.240171378	0.810197401	0.905978244	ARHGEF10L	protein_coding
ENSG00000074966.11	95.70251991	-0.382985042	0.111892091	-3.422807083	0.00061978	0.007985859	TXK	protein_coding
ENSG00000075035.10	262.7488149	-0.515601532	0.232390246	-2.218688351	0.026507934	0.117427696	WSCD2	protein_coding
ENSG00000075043.19	85.99419511	1.020877864	0.228321916	4.471221512	7.77741E-06	0.000266444	KCNQ2	protein_coding
ENSG00000075073.15	1149.02081	-0.729590148	0.245218345	-2.9752674	0.002927333	0.025185463	TACR2	protein_coding
ENSG00000075089.9	961.6273107	-0.153308977	0.055042284	-2.785294635	0.00534791	0.038957856	ACTR6	protein_coding
ENSG00000075131.10	490.8975473	-0.145804369	0.063570783	-2.293575157	0.021814914	0.102982179	TIPIN	protein_coding
ENSG00000075142.14	5343.357115	0.017123023	0.078767991	0.217385554	0.827907888	0.915254375	SRI	protein_coding
ENSG00000075151.21	5737.923939	0.07644663	0.052338155	1.460629047	0.14411727	0.3435745	EIF4G3	protein_coding
ENSG00000075188.9	1139.653717	-0.183856657	0.061231017	-3.002671919	0.002676208	0.02361229	NUP37	protein_coding
ENSG00000075213.11	1020.809909	0.29738948	0.144764761	2.054294685	0.039947179	0.153833352	SEMA3A	protein_coding
ENSG00000075218.19	1343.497169	0.025434793	0.092300849	0.275564016	0.782882951	0.890005488	GTSE1	protein_coding
ENSG00000075223.14	4943.068434	0.239866763	0.124577013	1.925449621	0.054173133	0.187358602	SEMA3C	protein_coding
ENSG00000075234.17	3212.18876	-0.215045633	0.074873647	-2.872113759	0.004077362	0.032217441	TTC38	protein_coding
ENSG00000075239.14	1871.297599	-0.234851514	0.069579941	-3.375276126	0.000737417	0.009121318	ACAT1	protein_coding
ENSG00000075240.17	3013.156161	0.042731931	0.071753628	0.595536874	0.551484659	0.743701843	GRAMD4	protein_coding
ENSG00000075275.18	3833.768627	0.732730652	0.114043474	6.425011673	1.31859E-10	3.42026E-08	CELSR1	protein_coding
ENSG00000075290.8	21.24893087	0.504796505	0.15641873	3.22721266	0.001250025	0.013607881	WNT8B	protein_coding
ENSG00000075292.19	4675.252039	0.049120043	0.043232754	1.13617659	0.255882666	0.482890948	ZNF638	protein_coding
ENSG00000075303.13	1827.694694	-0.201640258	0.065564445	-3.075451283	0.002101843	0.019846586	SLC25A40	protein_coding

ENSG00000075336.12	980.2522163	-0.173849712	0.062888769	-2.764399999	0.005702758	0.0408674	TIMM21	protein_coding
ENSG00000075340.23	179.5803189	0.556662632	0.175974085	3.163321646	0.001559799	0.015966962	ADD2	protein_coding
ENSG00000075388.4	4.570582376	-0.493521952	0.219423663	-2.249173793	0.024501441	0.111509065	FGF4	protein_coding
ENSG00000075391.17	3531.680749	0.171876884	0.061529997	2.793383578	0.00521598	0.038336442	RASAL2	protein_coding
ENSG00000075399.14	666.7451811	0.218457206	0.069150741	3.159144842	0.001582328	0.016136934	VPS9D1	protein_coding
ENSG00000075407.18	1695.121237	-0.074961602	0.057778035	-1.297406576	0.194491331	0.411344823	ZNF37A	protein_coding
ENSG00000075413.19	3962.803215	0.044531076	0.039404301	1.130106986	0.258431146	0.485600627	MARK3	protein_coding
ENSG00000075415.13	23127.5297	-0.169700998	0.050669871	-3.349149974	0.000810599	0.009779478	SLC25A3	protein_coding
ENSG00000075420.13	8879.941422	0.270954665	0.062115098	4.362138539	1.28797E-05	0.00039637	FNDC3B	protein_coding
ENSG00000075426.12	16292.87574	0.239227754	0.069529064	3.440687105	0.000580239	0.007615988	FOSL2	protein_coding
ENSG00000075429.9	11.99067431	-0.319812875	0.201136749	-1.59002707	0.111828703	0.294308455	CACNG5	protein_coding
ENSG00000075461.6	345.4380746	0.065701909	0.246421351	0.266624254	0.78975848	0.894391883	CACNG4	protein_coding
ENSG00000075539.15	4913.33592	-0.017513469	0.063423355	-0.276135954	0.782443647	0.889877513	FRYL	protein_coding
ENSG00000075568.17	7413.286961	-0.09460596	0.046632538	-2.028754259	0.042483326	0.160205902	TMEM131	protein_coding
ENSG00000075618.18	6838.397871	0.749468966	0.122388973	6.123664127	9.14476E-10	1.67924E-07	FSCN1	protein_coding
ENSG00000075624.17	442036.4144	-0.0451128	0.057599326	-0.7832175	0.4334994	0.651355194	ACTB	protein_coding
ENSG00000075643.6	1446.450191	0.029757899	0.097938679	0.303842155	0.761248149	0.877822456	MOCOS	protein_coding
ENSG00000075651.16	2166.313674	0.179669305	0.093778257	1.915895116	0.055378447	0.189943062	PLD1	protein_coding
ENSG00000075673.12	25.06078407	1.242214289	0.271340171	4.578069963	4.69286E-06	0.00017706	ATP12A	protein_coding
ENSG00000075702.18	1223.942765	-0.002564436	0.096833197	-0.026483025	0.978872073	0.990569863	WDR62	protein_coding
ENSG00000075711.21	5287.725256	0.018452077	0.053307301	0.34614541	0.729233438	0.859022127	DLG1	protein_coding
ENSG00000075785.14	19937.96548	0.038975536	0.040918506	0.952516108	0.340835301	0.570831283	RAB7A	protein_coding
ENSG00000075790.12	47.72737571	0.030637402	0.06351489	0.482365662	0.62954621	0.798653701	BCAP29	protein_coding
ENSG00000075826.17	273.8401312	-0.060450377	0.105894959	-0.570852266	0.56809979	0.755438092	SEC31B	protein_coding
ENSG00000075856.12	2902.23872	-0.061018762	0.034756074	-1.755628738	0.079151822	0.238580618	SART3	protein_coding
ENSG00000075884.14	479.3706152	-0.502996199	0.113191166	-4.4437761	8.83936E-06	0.00029404	ARHGAP15	protein_coding
ENSG00000075886.11	45.25117547	-0.074425529	0.087341891	-0.85211722	0.394149036	0.617656644	TUBA3D	protein_coding
ENSG00000075891.22	21.26173117	1.008175886	0.261918883	3.849191298	0.000118508	0.002259073	PAX2	protein_coding
ENSG00000075914.13	897.0195213	-0.038155753	0.05060113	-0.75404942	0.450819547	0.665538431	EXOSC7	protein_coding
ENSG00000075945.13	1474.951457	0.189105069	0.067584722	2.798044626	0.005141301	0.037939661	KIFAP3	protein_coding
ENSG00000075975.17	1640.350244	0.095585891	0.041910621	2.280708065	0.022565728	0.105475353	MKRN2	protein_coding
ENSG00000076003.5	3578.532526	-0.010320981	0.06531762	-0.158012202	0.874447184	0.939295544	MCM6	protein_coding
ENSG00000076043.11	1653.163317	0.096048304	0.065620352	1.463696884	0.143276787	0.342282362	REXO2	protein_coding
ENSG00000076053.12	1454.456411	0.057101891	0.055248291	1.033550371	0.301346411	0.530962823	RBM7	protein_coding
ENSG00000076067.13	4052.865404	0.211512295	0.065194823	3.244311197	0.00117735	0.013000554	RBMS2	protein_coding
ENSG00000076108.12	9213.921763	0.119623643	0.045329951	2.638953728	0.008316233	0.053121737	BAZ2A	protein_coding
ENSG00000076201.15	6623.062486	0.179929794	0.046895476	3.836826288	0.000124635	0.002340085	PTPN23	protein_coding
ENSG00000076242.16	1539.897018	-0.152561795	0.081740344	-1.866419769	0.061982659	0.204051005	MLH1	protein_coding
ENSG00000076248.11	2643.925022	-0.046226679	0.065798931	-0.702544532	0.48233964	0.690800778	UNG	protein_coding
ENSG00000076258.10	263.7438863	-0.155582368	0.08545446	-1.820646548	0.068660602	0.217222525	FMO4	protein_coding
ENSG00000076321.11	1148.089379	0.093776871	0.037553975	2.497122351	0.012520575	0.070629008	KLHL20	protein_coding
ENSG00000076344.16	130.9612334	0.291306622	0.158214057	1.841218338	0.065589569	0.211077221	RGS11	protein_coding

ENSG00000076351.13	388.0273526	0.212627987	0.100600492	2.113587937	0.034550477	0.139601867	SLC46A1	protein_coding
ENSG00000076356.7	4952.740674	0.16727486	0.079742834	2.097678909	0.035933519	0.143290169	PLXNA2	protein_coding
ENSG00000076382.17	2042.203161	0.075968583	0.089395384	0.849804313	0.395433892	0.618759119	SPAG5	protein_coding
ENSG00000076513.17	3366.377526	-0.165609364	0.050469191	-3.281395236	0.001032949	0.011761906	ANKRD13A	protein_coding
ENSG00000076554.15	9961.146935	-0.110824361	0.093108184	-1.190275183	0.233938252	0.458521108	TPD52	protein_coding
ENSG00000076555.15	1925.45821	-0.166361134	0.108693227	-1.530556582	0.125879019	0.316345446	ACACB	protein_coding
ENSG00000076604.15	3924.635769	0.166693222	0.069448517	2.400241629	0.016384253	0.085271298	TRAF4	protein_coding
ENSG00000076641.4	2896.631872	-0.106331677	0.086883757	-1.223838378	0.221013204	0.443298001	PAG1	protein_coding
ENSG00000076650.7	854.0921016	-0.104348937	0.054810929	-1.903797993	0.056936498	0.193474873	GPATCH1	protein_coding
ENSG00000076662.10	106.1985056	-0.554126198	0.155657774	-3.559900572	0.000370995	0.005443668	ICAM3	protein_coding
ENSG00000076685.19	2257.307647	0.12254299	0.061414548	1.995341415	0.046005656	0.168538293	NT5C2	protein_coding
ENSG00000076706.17	9374.253627	-0.077277529	0.109943079	-0.702886715	0.482126351	0.690754779	MCAM	protein_coding
ENSG00000076716.9	3053.735459	-0.217828342	0.107381049	-2.028554801	0.042503656	0.160249327	GPC4	protein_coding
ENSG00000076770.14	3907.715201	-0.27298316	0.09506248	-2.871618317	0.004083759	0.032251501	MBNL3	protein_coding
ENSG00000076826.10	4163.966392	0.268717711	0.095280073	2.820292856	0.004797984	0.036179572	CAMSAP3	protein_coding
ENSG00000076864.19	3000.660463	0.234948035	0.155862603	1.507404792	0.131706931	0.325211507	RAP1GAP	protein_coding
ENSG00000076924.12	3441.385336	0.070778577	0.043403177	1.630723424	0.102948692	0.280369673	XAB2	protein_coding
ENSG00000076928.18	6742.330299	0.058307311	0.059081628	0.986894115	0.32369455	0.553444399	ARHGEF1	protein_coding
ENSG00000076944.16	2460.858123	0.052061087	0.068443515	0.760643092	0.446870274	0.662355984	STXBP2	protein_coding
ENSG00000076984.18	2164.449946	0.051934938	0.050619885	1.025978968	0.304901498	0.534369124	MAP2K7	protein_coding
ENSG00000077009.13	6.252104315	-0.543609591	0.240731838	-2.258154114	0.023936053	0.109636105	NMRK2	protein_coding
ENSG00000077044.11	3529.606546	-0.021864097	0.073444603	-0.297695083	0.765935897	0.881013292	DGKD	protein_coding
ENSG00000077063.11	332.1842623	-0.255181951	0.157940021	-1.615688978	0.106161575	0.285268728	CTTNBP2	protein_coding
ENSG00000077080.10	10.95834851	-0.361669094	0.202525389	-1.785796323	0.074132242	0.228268847	ACTL6B	protein_coding
ENSG00000077092.19	389.1295243	0.231703275	0.098436815	2.353827417	0.018581236	0.092408006	RARB	protein_coding
ENSG00000077097.15	7592.421495	0.061356585	0.054841798	1.118792369	0.263228728	0.490893922	TOP2B	protein_coding
ENSG00000077147.16	22303.93879	-0.044840771	0.059392276	-0.75499331	0.450252997	0.665107621	TM9SF3	protein_coding
ENSG00000077150.20	5986.360866	-0.02754643	0.069446274	-0.39665814	0.691619572	0.837449625	NFKB2	protein_coding
ENSG00000077152.11	1470.932924	-0.033382384	0.088374647	-0.377737115	0.705625893	0.845706965	UBE2T	protein_coding
ENSG00000077157.22	15858.03554	-0.550445673	0.180634309	-3.047293036	0.002309125	0.021190688	PPP1R12B	protein_coding
ENSG00000077232.18	9549.351594	0.175894703	0.056563718	3.109673642	0.001872942	0.01824663	DNAJC10	protein_coding
ENSG00000077235.18	6686.812535	0.124575415	0.053787776	2.316054416	0.020555298	0.098988597	GTF3C1	protein_coding
ENSG00000077238.14	4985.649405	0.121872609	0.062487093	1.950364521	0.051132687	0.180480246	IL4R	protein_coding
ENSG00000077254.14	3263.245922	0.034704551	0.043537951	0.797110349	0.425386946	0.644242501	USP33	protein_coding
ENSG00000077264.15	197.3139375	0.11171153	0.167945983	0.665163457	0.505945951	0.710067849	PAK3	protein_coding
ENSG00000077274.9	2086.596188	-0.157760787	0.219853102	-0.717573623	0.473020226	0.683856493	CAPN6	protein_coding
ENSG00000077279.19	64.63585994	0.040828325	0.236673671	0.172508946	0.863037429	0.933238593	DCX	protein_coding
ENSG00000077312.9	3737.822862	-0.127862473	0.053785283	-2.377276177	0.017441024	0.08859162	SNRPA	protein_coding
ENSG00000077327.16	11.0212927	-0.761229014	0.20152777	-3.777290912	0.000158544	0.002847836	SPAG6	protein_coding
ENSG00000077348.9	1535.766386	-0.088156818	0.078192083	-1.127439184	0.25955684	0.486786096	EXOSC5	protein_coding
ENSG00000077380.16	6255.968397	-0.019378522	0.046850654	-0.413623293	0.679150011	0.830014248	DYNC1I2	protein_coding
ENSG00000077420.16	1743.000522	-0.462614925	0.128910834	-3.588642721	0.000332404	0.005016245	APBB1IP	protein_coding

ENSG00000077454.16	981.6289335	0.155322121	0.069685013	2.228917148	0.025819418	0.115445596	LRCH4	protein_coding
ENSG00000077458.13	1102.850931	-0.080384511	0.052046888	-1.544463357	0.122476122	0.311185467	FAM76B	protein_coding
ENSG00000077463.15	1364.765553	-0.015420485	0.064125602	-0.240473142	0.809963482	0.905832892	SIRT6	protein_coding
ENSG00000077498.9	1.447853227	0.116447679	0.332643236	0.35006778	0.726287831	0.8572439	TYR	protein_coding
ENSG00000077514.9	2635.004075	-0.143989515	0.085342175	-1.687202303	0.091564459	0.260859337	POLD3	protein_coding
ENSG00000077522.14	99.56712829	-0.60925366	0.225562379	-2.701042885	0.006912243	0.046591615	ACTN2	protein_coding
ENSG00000077549.19	18183.73063	-0.029085512	0.046543485	-0.624910477	0.532029816	0.729546659	CAPZB	protein_coding
ENSG00000077585.14	1614.478261	0.085958341	0.075956889	1.131672743	0.257772045	0.484817913	GPR137B	protein_coding
ENSG00000077616.11	104.9410742	0.016759234	0.119105368	0.140709312	0.888099588	0.946540401	NAALAD2	protein_coding
ENSG00000077684.16	2308.550364	-0.160912458	0.072675583	-2.214119939	0.026820529	0.118385805	JADE1	protein_coding
ENSG00000077713.19	2052.924188	-0.006587227	0.063409312	-0.103884218	0.917261231	0.960760808	SLC25A43	protein_coding
ENSG00000077721.16	4043.341562	-0.023617205	0.047837833	-0.493693037	0.621522983	0.793022781	UBE2A	protein_coding
ENSG00000077782.21	3891.370136	0.143604776	0.132677382	1.082360638	0.279092311	0.507871785	FGFR1	protein_coding
ENSG00000077800.13	7.986507478	-0.133066221	0.223482023	-0.595422484	0.5515611	0.743701843	FKBP6	protein_coding
ENSG00000077935.17	87.53050404	-0.116028656	0.184440399	-0.629084823	0.529293526	0.727465589	SMC1B	protein_coding
ENSG00000077942.19	12784.58087	0.210247244	0.163686946	1.284447231	0.198985516	0.417004512	FBLN1	protein_coding
ENSG00000077943.8	1189.104171	-0.298341706	0.160102113	-1.863446403	0.062399487	0.204912014	ITGA8	protein_coding
ENSG00000077984.6	724.9750996	-0.33310374	0.136002204	-2.449252505	0.014315305	0.077570015	CST7	protein_coding
ENSG00000078018.20	631.8092147	0.276449704	0.141401373	1.955070868	0.050574706	0.179222084	MAP2	protein_coding
ENSG00000078043.16	1463.96312	-0.075051835	0.058150199	-1.290654835	0.196823393	0.414071085	PIAS2	protein_coding
ENSG00000078053.17	172.5210791	0.002758506	0.128857914	0.021407346	0.982920714	0.992450592	AMPH	protein_coding
ENSG00000078061.13	5193.496016	-0.069996674	0.054962119	-1.273543949	0.202825087	0.422023602	ARAF	protein_coding
ENSG00000078070.13	1514.962666	-0.0517938	0.064365211	-0.804686247	0.421000758	0.640705107	MCCC1	protein_coding
ENSG00000078081.8	1353.178196	-0.181321659	0.12229803	-1.482621264	0.138175064	0.334774534	LAMP3	protein_coding
ENSG00000078098.14	910.9943501	0.778744987	0.134334862	5.797043104	6.74943E-09	8.65059E-07	FAP	protein_coding
ENSG00000078114.19	2168.993118	0.339738349	0.183552526	1.850905328	0.064183174	0.208398046	NEBL	protein_coding
ENSG00000078124.12	3195.438865	-0.050399895	0.059103563	-0.852738691	0.39380423	0.617539563	ACER3	protein_coding
ENSG00000078140.14	5871.893732	0.061259168	0.041889209	1.462409297	0.143629083	0.342884342	UBE2K	protein_coding
ENSG00000078142.13	1802.056766	0.041213597	0.053866828	0.76510162	0.444211035	0.660188407	PIK3C3	protein_coding
ENSG00000078177.14	1118.342063	0.002489782	0.068740873	0.036219824	0.971107079	0.98634098	N4BP2	protein_coding
ENSG00000078237.7	637.8375345	-0.001602603	0.076640748	-0.020910581	0.983316986	0.992657225	TIGAR	protein_coding
ENSG00000078246.17	1977.062391	0.132330068	0.05617419	2.355709383	0.018487375	0.092123995	TULP3	protein_coding
ENSG00000078269.15	3515.914417	0.087860797	0.058767171	1.495065955	0.13489723	0.330155383	SYNJ2	protein_coding
ENSG00000078295.17	259.4578696	-0.216219104	0.178873973	-1.208779006	0.226747759	0.450076848	ADCY2	protein_coding
ENSG00000078304.19	7070.071488	0.007795496	0.043749363	0.178185354	0.858577412	0.931142278	PPP2R5C	protein_coding
ENSG00000078319.10	666.2936915	-0.013941945	0.062041595	-0.224719323	0.822197631	0.912411743	PMS2P1	transcribed_unprocessed_pseudogene
ENSG00000078328.22	27.87433536	-0.146067923	0.209160043	-0.698354815	0.484955327	0.692859194	RBFOX1	protein_coding
ENSG00000078369.18	23750.55755	0.036397972	0.040174215	0.906003297	0.364934108	0.592192359	GNB1	protein_coding
ENSG00000078399.19	210.947926	0.990558511	0.212676492	4.657583458	3.19943E-06	0.000130179	HOXA9	protein_coding
ENSG00000078401.7	1225.325769	0.001260633	0.122284259	0.010309035	0.991774726	0.996273665	EDN1	protein_coding
ENSG00000078403.17	2171.485866	-0.166783703	0.052438673	-3.18054773	0.001469969	0.015317276	MLLT10	protein_coding
ENSG00000078487.17	166.816136	-0.045758575	0.087576183	-0.522500221	0.601322093	0.779481002	ZCWPW1	protein_coding

ENSG00000078549.15	62.70799933	-0.262621152	0.214598458	-1.223779308	0.221035492	0.443322301	ADCYAP1R1	protein_coding
ENSG00000078579.9	36.14494728	-0.237445369	0.29282127	-0.810888395	0.417429766	0.637675696	FGF20	protein_coding
ENSG00000078589.13	411.535748	-0.737250257	0.142268232	-5.182114416	2.19385E-07	1.51989E-05	P2RY10	protein_coding
ENSG00000078596.11	1176.78819	-0.278770497	0.129858267	-2.146728916	0.031814869	0.132563014	ITM2A	protein_coding
ENSG00000078618.22	6200.406559	-0.007390479	0.034923498	-0.211619086	0.832404218	0.917453796	NRDC	protein_coding
ENSG00000078668.14	5226.926525	-0.109309449	0.070603878	-1.54820744	0.121572361	0.309937329	VDAC3	protein_coding
ENSG00000078674.18	7270.0749	-0.0672921	0.070260094	-0.957757045	0.338185284	0.567969327	PCM1	protein_coding
ENSG00000078687.17	1867.088007	0.130985678	0.082731576	1.583261002	0.113361988	0.296928321	TNRC6C	protein_coding
ENSG00000078699.22	3348.491127	0.055311726	0.06863665	0.805862843	0.420321938	0.640141512	CBFA2T2	protein_coding
ENSG00000078725.13	27.55835202	-0.284631987	0.210772327	-1.350423897	0.17688005	0.389150398	BRINP1	protein_coding
ENSG00000078747.16	4846.660337	-0.001513898	0.046525614	-0.032539018	0.974042201	0.988009338	ITCH	protein_coding
ENSG00000078795.17	21.29188605	-0.336364959	0.120358304	-2.79469673	0.005194842	0.038213309	PKD2L2	protein_coding
ENSG00000078804.13	5479.555645	0.201856166	0.092396295	2.184678136	0.028912454	0.124472157	TP53INP2	protein_coding
ENSG00000078808.19	10297.32291	0.037490718	0.055205176	0.679116003	0.497064363	0.703311052	SDF4	protein_coding
ENSG00000078814.17	250.6373284	0.281737477	0.130201248	2.163861564	0.030474976	0.128845252	MYH7B	protein_coding
ENSG00000078898.7	35.42066007	0.695007843	0.399438145	1.739963625	0.081865405	0.24369484	BPIFB2	protein_coding
ENSG00000078900.15	361.3594872	0.282465088	0.143145403	1.973273896	0.048464365	0.174403069	TP73	protein_coding
ENSG00000078902.16	4120.042134	-0.070079539	0.050323032	-1.392593728	0.163742681	0.370960704	TOLLIP	protein_coding
ENSG00000078967.13	912.7093508	-0.181195858	0.053357206	-3.395902287	0.000684028	0.00860008	UBE2D4	protein_coding
ENSG00000079101.16	17.40788576	-0.003854693	0.166921272	-0.023092882	0.981576183	0.991956247	CLUL1	protein_coding
ENSG00000079102.16	650.7513472	-0.055489136	0.151428463	-0.366437952	0.714038305	0.850557998	RUNX1T1	protein_coding
ENSG00000079112.10	13725.51099	-0.189429144	0.211462067	-0.895806735	0.370355992	0.596936056	CDH17	protein_coding
ENSG00000079134.12	1278.385574	-0.137636407	0.054817954	-2.510790639	0.012046112	0.068690579	THOC1	protein_coding
ENSG00000079150.18	737.5042876	0.279569594	0.079356023	3.522978873	0.000426725	0.006039432	FKBP7	protein_coding
ENSG00000079156.17	215.5451806	0.265248318	0.134299128	1.975056142	0.048261777	0.17404034	OSBPL6	protein_coding
ENSG00000079215.14	756.28647	0.042859364	0.133697612	0.320569409	0.748536723	0.87074517	SLC1A3	protein_coding
ENSG00000079246.16	17182.14394	0.018625041	0.039271956	0.474258043	0.635315907	0.802189421	XRCC5	protein_coding
ENSG00000079257.8	1065.096065	0.465503068	0.098712909	4.715726381	2.4085E-06	0.000104746	LXN	protein_coding
ENSG00000079263.19	441.5817101	-0.498796451	0.126375683	-3.946933779	7.91584E-05	0.001653637	SP140	protein_coding
ENSG00000079277.22	1611.354361	-0.061856883	0.034831863	-1.775870647	0.07575424	0.231704097	MKNK1	protein_coding
ENSG00000079308.19	25087.97178	-0.116840948	0.163289251	-0.715545863	0.474271812	0.684847994	TNS1	protein_coding
ENSG00000079313.15	2689.791731	0.029357159	0.055502473	0.528934257	0.596851057	0.776312994	REXO1	protein_coding
ENSG00000079332.15	4712.070543	0.049769843	0.04185054	1.189228207	0.234349878	0.458875067	SAR1A	protein_coding
ENSG00000079335.20	530.2876975	-0.160001122	0.060662051	-2.637581828	0.008349948	0.053276179	CDC14A	protein_coding
ENSG00000079337.16	631.1774427	0.258642115	0.092916523	2.783596574	0.005375986	0.03911007	RAPGEF3	protein_coding
ENSG00000079385.23	5522.295867	0.300437235	0.129170822	2.32589086	0.020024374	0.097334285	CEACAM1	protein_coding
ENSG00000079387.14	1744.783527	-0.0093675	0.047158176	-0.198639992	0.842544367	0.922547764	SENP1	protein_coding
ENSG00000079393.20	6.654500433	0.439239716	0.276169341	1.590472408	0.11172836	0.294115425	DUSP13	protein_coding
ENSG00000079432.7	8018.553341	0.174186786	0.057405715	3.034310873	0.002410859	0.021854163	CIC	protein_coding
ENSG00000079435.10	587.7522845	0.318328714	0.107462932	2.96221878	0.003054307	0.026046482	LIPE	protein_coding
ENSG00000079459.13	10231.06938	0.088776308	0.101673149	0.873153909	0.382579194	0.607792985	FDFT1	protein_coding
ENSG00000079462.8	1603.783778	-0.18825901	0.086497081	-2.176478183	0.029519523	0.126201537	PAFAH1B3	protein_coding

ENSG00000079482.13	652.3021099	0.142361127	0.075994571	1.873306555	0.061026065	0.201923527	OPHN1	protein_coding
ENSG00000079557.5	4.019831389	-1.791427044	0.375903389	-4.765658143	1.88238E-06	8.55355E-05	AFM	protein_coding
ENSG00000079616.13	2764.803429	0.017091344	0.070623438	0.242006684	0.808774984	0.905270407	KIF22	protein_coding
ENSG00000079689.14	117.6469117	-1.440133916	0.285434453	-5.045410253	4.52549E-07	2.73519E-05	SCGN	protein_coding
ENSG00000079691.18	3844.728941	0.040259574	0.067634934	0.595248212	0.551677568	0.743742173	CARMIL1	protein_coding
ENSG00000079739.17	3164.674132	0.137021291	0.072389756	1.892827084	0.058380869	0.196376929	PGM1	protein_coding
ENSG00000079785.16	5862.277013	-0.020184893	0.040280263	-0.501111248	0.616292831	0.789609335	DDX1	protein_coding
ENSG00000079805.18	16976.51469	0.111964275	0.061579862	1.818196285	0.069034138	0.217866499	DNM2	protein_coding
ENSG00000079819.19	5506.318988	0.140245429	0.088644005	1.582119725	0.113622241	0.297299415	EPB41L2	protein_coding
ENSG00000079841.18	123.753157	-0.160170743	0.225145804	-0.711408966	0.476830844	0.686719282	RIMS1	protein_coding
ENSG00000079931.15	1167.164374	0.192026519	0.144561078	1.328341775	0.184065225	0.398149962	MOXD1	protein_coding
ENSG00000079950.14	4438.802767	0.078361065	0.04845132	1.617315393	0.105810218	0.284816349	STX7	protein_coding
ENSG00000079974.18	754.6698935	-0.01622132	0.050055003	-0.324069906	0.74588511	0.86921526	RABL2B	protein_coding
ENSG00000079999.14	4012.024449	0.09566452	0.056094517	1.705416582	0.088116758	0.254971046	KEAP1	protein_coding
ENSG00000080007.8	103.6948063	-0.070994838	0.171301536	-0.414443674	0.67854921	0.829615031	DDX43	protein_coding
ENSG00000080031.10	3384.682256	0.091906766	0.11926726	0.7705951	0.440946966	0.657490024	PTPRH	protein_coding
ENSG00000080166.16	11.94344582	-0.078065818	0.186450245	-0.418695173	0.675438924	0.827668471	DCT	protein_coding
ENSG00000080189.15	3708.410243	-0.060097589	0.058982684	-1.018902242	0.308249379	0.537694772	SLC35C2	protein_coding
ENSG00000080200.10	1383.072092	0.276625404	0.095523766	2.89588043	0.003780964	0.030457757	CRYBG3	protein_coding
ENSG00000080224.17	28.87444797	-0.37926588	0.240251794	-1.578618308	0.114423633	0.298457406	EPHA6	protein_coding
ENSG00000080293.10	193.8465294	-0.528354708	0.229298249	-2.304224789	0.021210028	0.101091022	SCTR	protein_coding
ENSG00000080298.15	786.7445942	0.047260038	0.086446779	0.54669519	0.584588159	0.767353402	RFX3	protein_coding
ENSG00000080345.18	4907.580542	-0.006052271	0.051446898	-0.117641119	0.906352023	0.955512328	RIF1	protein_coding
ENSG00000080371.6	4821.187986	-0.088340099	0.044714916	-1.975629304	0.048196777	0.17389228	RAB21	protein_coding
ENSG00000080493.18	2930.304251	-0.084037557	0.17331533	-0.484882423	0.627759752	0.79725048	SLC4A4	protein_coding
ENSG00000080503.24	5040.107281	0.117385177	0.076770659	1.529036999	0.126255272	0.31688734	SMARCA2	protein_coding
ENSG00000080511.5	39.51721572	0.810554533	0.253378093	3.198992167	0.001379089	0.014682768	RDH8	protein_coding
ENSG00000080546.13	2115.16779	-0.45008898	0.10237414	-4.396510467	1.10005E-05	0.00035073	SESN1	protein_coding
ENSG00000080561.14	1041.910453	0.202387161	0.115420098	1.753482831	0.079519164	0.2392768	MID2	protein_coding
ENSG00000080572.13	1.356465262	-0.458915008	0.311876254	-1.471465052	0.141165392	0.339078618	DNAAF6	protein_coding
ENSG00000080573.7	1960.401138	0.359443327	0.106955132	3.360692647	0.000777473	0.009487521	COL5A3	protein_coding
ENSG00000080603.17	490.6551728	0.140047532	0.060611228	2.310587258	0.020855664	0.099980997	SRCAP	protein_coding
ENSG00000080608.10	1940.675714	0.004402782	0.077237663	0.057003046	0.954542769	0.978763107	PUM3	protein_coding
ENSG00000080618.17	15.29279982	0.016454603	0.187592063	0.087714812	0.930103347	0.966811279	CPB2	protein_coding
ENSG00000080644.16	342.9882688	-0.297661492	0.230680696	-1.290361513	0.196925168	0.414160621	CHRNA3	protein_coding
ENSG00000080709.16	68.41923873	-0.163570036	0.130355209	-1.254802449	0.209550483	0.429681037	KCNN2	protein_coding
ENSG00000080802.19	1775.04975	-0.138707009	0.031773167	-4.365539237	1.2681E-05	0.000391913	CNOT4	protein_coding
ENSG00000080815.19	5751.378952	0.107431278	0.041642286	2.579860252	0.00988403	0.059915127	PSEN1	protein_coding
ENSG00000080819.9	1426.498923	0.101690224	0.060351729	1.684959578	0.091996371	0.261608421	CPOX	protein_coding
ENSG00000080822.17	2909.490649	0.068825445	0.04789792	1.436919272	0.150740933	0.353087133	CLDND1	protein_coding
ENSG00000080823.23	856.1863504	-0.016410039	0.086973309	-0.188679019	0.850344396	0.926660973	MOK	protein_coding
ENSG00000080824.19	74954.73359	0.061649505	0.066899744	0.921520786	0.356778594	0.584686377	HSP90AA1	protein_coding

ENSG00000080839.12	1392.223841	-0.080211999	0.070095018	-1.14433237	0.252485824	0.479268839	RBL1	protein_coding
ENSG00000080845.18	6092.705897	0.064856161	0.055830714	1.161657377	0.245374666	0.471791908	DLGAP4	protein_coding
ENSG00000080854.16	1148.428838	-0.201257275	0.159850739	-1.259032497	0.208018594	0.427982261	IGSF9B	protein_coding
ENSG00000080910.13	1.897730179	-1.11670703	0.405909522	-2.751123018	0.005939134	0.042028194	CFHR2	protein_coding
ENSG00000080947.15	239.3064654	0.022928465	0.095799067	0.239339126	0.810842629	0.906374134	CROCCP3	transcribed_unprocessed_pseudogene
ENSG00000080986.13	1082.357162	-0.064741849	0.088454705	-0.731920911	0.464216842	0.676372007	NDC80	protein_coding
ENSG00000081014.11	916.6447264	0.024913911	0.0518958	0.48007566	0.631173595	0.799715078	AP4E1	protein_coding
ENSG00000081019.13	1812.950206	-0.032511665	0.04579072	-0.710005545	0.477700698	0.687376205	RSBN1	protein_coding
ENSG00000081026.19	1937.664107	-0.032181444	0.060235523	-0.534260217	0.593161512	0.773248577	MAGI3	protein_coding
ENSG00000081041.9	1995.200753	0.017008686	0.155790007	0.109177003	0.9130621	0.95894649	CXCL2	protein_coding
ENSG00000081051.8	147.67066	-3.649880516	0.345057751	-10.5775932	3.78558E-26	4.1241E-22	AFP	protein_coding
ENSG00000081052.13	713.4587519	0.524319555	0.171968178	3.048933585	0.002296552	0.021117718	COL4A4	protein_coding
ENSG00000081059.20	2787.267004	-0.516387276	0.106918052	-4.829748266	1.36706E-06	6.66433E-05	TCF7	protein_coding
ENSG00000081087.15	2211.162644	0.019989694	0.064503239	0.309902172	0.756635351	0.875189751	OSTM1	protein_coding
ENSG00000081138.14	13.01153057	-0.569873751	0.260609665	-2.186694614	0.028764825	0.124032202	CDH7	protein_coding
ENSG00000081148.12	28.7992208	-0.094528378	0.119706951	-0.789664908	0.429723491	0.648031472	IMPG2	protein_coding
ENSG00000081154.12	5974.930805	-0.014329368	0.036151641	-0.396368406	0.69183327	0.837532562	PCNP	protein_coding
ENSG00000081177.18	1721.241589	0.03434946	0.044280657	0.775721571	0.4379134	0.654803975	EXD2	protein_coding
ENSG00000081181.8	184.9396909	0.355788287	0.104296528	3.411314787	0.000646504	0.008239622	ARG2	protein_coding
ENSG00000081189.16	1955.690249	-0.367915582	0.107451452	-3.424016854	0.000617028	0.007955097	MEF2C	protein_coding
ENSG00000081237.20	4683.771333	-0.657379496	0.133689329	-4.917217403	8.7783E-07	4.7095E-05	PTPRC	protein_coding
ENSG00000081248.11	9.476387367	-0.324557009	0.213546816	-1.519840078	0.128551174	0.320400051	CACNA1S	protein_coding
ENSG00000081277.13	621.7539438	1.111744938	0.26458516	4.201841619	2.64752E-05	0.000691673	PKP1	protein_coding
ENSG00000081307.14	2058.265049	-0.024647679	0.044077147	-0.559194061	0.576029286	0.760904108	UBA5	protein_coding
ENSG00000081320.11	3249.625304	-0.213371037	0.077849533	-2.740813335	0.006128731	0.042923776	STK17B	protein_coding
ENSG00000081377.17	1878.710572	-0.043373464	0.062472863	-0.694276865	0.487508597	0.695003177	CDC14B	protein_coding
ENSG00000081386.12	833.9930759	-0.058855968	0.052449967	-1.122135453	0.261804852	0.489348462	ZNF510	protein_coding
ENSG00000081479.15	76.41534553	-0.237058256	0.241739401	-0.980635573	0.326772486	0.556523415	LRP2	protein_coding
ENSG00000081665.14	1689.50785	-0.097865344	0.068921867	-1.419946199	0.155623345	0.360244275	ZNF506	protein_coding
ENSG00000081692.12	288.8458025	-0.011858585	0.066082282	-0.17945181	0.857582953	0.930480601	JMJD4	protein_coding
ENSG00000081721.12	879.9909909	0.03259771	0.048067181	0.678169786	0.497664047	0.703679749	DUSP12	protein_coding
ENSG00000081760.17	2495.25557	-0.044745842	0.070946312	-0.630700049	0.528236667	0.726497918	AACS	protein_coding
ENSG00000081791.9	2891.692318	-0.079501609	0.043066763	-1.846008485	0.064890973	0.209725294	DELE1	protein_coding
ENSG00000081800.9	5.362659724	-1.193270298	0.334216977	-3.570346151	0.00035651	0.005291426	SLC13A1	protein_coding
ENSG00000081803.16	2279.694331	-0.02667188	0.077763402	-0.34298757	0.731607811	0.860571448	CADPS2	protein_coding
ENSG00000081818.4	227.1673245	0.146714626	0.127112179	1.154213759	0.248412543	0.475020367	PCDHB4	protein_coding
ENSG00000081842.18	11.19178887	0.791397801	0.189613081	4.173751087	2.99625E-05	0.000762216	PCDHA6	protein_coding
ENSG00000081853.15	98.09111121	0.318073985	0.119841236	2.654128044	0.007951365	0.051538989	PCDHGA2	protein_coding
ENSG00000081870.12	972.7754198	-0.065904002	0.058021503	-1.135854802	0.256017337	0.48300578	HSPB11	protein_coding
ENSG00000081913.14	2224.075117	-0.093905079	0.075163711	-1.24934064	0.211540509	0.431941425	PHLPP1	protein_coding
ENSG00000081923.15	8531.526535	-0.138812898	0.093129612	-1.490534493	0.136083754	0.331866458	ATP8B1	protein_coding
ENSG00000081985.13	212.2950791	-0.211259342	0.121650123	-1.73661429	0.082455276	0.244658368	IL12RB2	protein_coding

ENSG00000082014.17	604.8435475	-0.02986152	0.108359423	-0.275578436	0.782871874	0.890005488	SMARCD3	protein_coding
ENSG00000082068.9	1352.622932	-0.046484505	0.051668689	-0.899664874	0.368298622	0.595324949	WDR70	protein_coding
ENSG00000082074.19	2804.676548	-0.522612491	0.111538044	-4.685508824	2.79265E-06	0.000117474	FYB1	protein_coding
ENSG00000082126.18	14.30439744	-0.042094491	0.160998317	-0.261459201	0.793738409	0.896539764	MPP4	protein_coding
ENSG00000082146.13	962.2487382	-0.304693396	0.067876835	-4.488915784	7.15866E-06	0.000249762	STRADB	protein_coding
ENSG00000082153.18	6543.192149	0.155357799	0.048148608	3.226631137	0.001252568	0.013617977	BZW1	protein_coding
ENSG00000082175.15	227.7514104	-0.218060569	0.165546389	-1.317217312	0.187765792	0.402888864	PGR	protein_coding
ENSG00000082196.21	338.6848481	0.337656623	0.139449966	2.421346039	0.01546315	0.081707876	C1QTNF3	protein_coding
ENSG00000082212.13	4297.15446	-0.307743336	0.064828567	-4.747032871	2.06422E-06	9.24488E-05	ME2	protein_coding
ENSG00000082213.18	2077.368092	0.066229452	0.059420982	1.11458024	0.265030342	0.492868773	C5orf22	protein_coding
ENSG00000082258.13	2402.708965	-0.049345184	0.052279423	-0.943873909	0.345234087	0.574740107	CCNT2	protein_coding
ENSG00000082269.17	3251.874961	0.025908968	0.083148939	0.311597101	0.755346737	0.874488437	FAM135A	protein_coding
ENSG00000082293.13	89.90494075	-0.645328808	0.203921853	-3.164588779	0.001553023	0.015908809	COL19A1	protein_coding
ENSG00000082397.17	1233.906601	0.035029198	0.116838172	0.299809532	0.764322444	0.879900646	EPB41L3	protein_coding
ENSG00000082438.17	3384.401727	-0.171908449	0.080886859	-2.125295144	0.033561993	0.137043757	COBLL1	protein_coding
ENSG00000082458.12	3165.022914	0.050938556	0.070888943	0.718568419	0.472406878	0.683382508	DLG3	protein_coding
ENSG00000082482.14	101.7720132	0.187427936	0.228279258	0.821046721	0.411619656	0.632458297	KCNK2	protein_coding
ENSG00000082497.12	767.9728816	0.50316622	0.148451595	3.389429535	0.000700382	0.008755178	SERTAD4	protein_coding
ENSG00000082512.15	1964.592069	-0.330826607	0.081981057	-4.035403046	5.45086E-05	0.001230737	TRAF5	protein_coding
ENSG00000082515.18	1573.119848	-0.156236883	0.057990232	-2.694193098	0.007055927	0.047253134	MRPL22	protein_coding
ENSG00000082516.9	1814.279796	-0.032134299	0.052642765	-0.610421945	0.541582335	0.736687895	GEMIN5	protein_coding
ENSG00000082556.14	15.45553948	0.353618071	0.26075983	1.356106386	0.175065321	0.386504281	OPRK1	protein_coding
ENSG00000082641.16	12322.34747	0.091191663	0.052603203	1.733576238	0.0829933	0.245742951	NFE2L1	protein_coding
ENSG00000082684.16	218.4603429	0.101349991	0.112119742	0.903944205	0.366024988	0.593233566	SEMA5B	protein_coding
ENSG00000082701.17	5365.689283	0.076136464	0.040731483	1.869228876	0.061590979	0.203138233	GSK3B	protein_coding
ENSG00000082781.12	7721.074406	0.306360759	0.067779917	4.519934085	6.18589E-06	0.000223015	ITGB5	protein_coding
ENSG00000082805.20	4334.416396	0.180761734	0.075950551	2.3799924	0.017312995	0.088177699	ERC1	protein_coding
ENSG00000082898.18	11208.41602	0.028727268	0.04628827	0.620616578	0.53485193	0.731829391	XPO1	protein_coding
ENSG00000082929.8	3.733724904	0.589581957	0.21787987	2.705995539	0.006809996	0.046152286	LINC01587	lncRNA
ENSG00000082996.20	3283.125539	0.061523913	0.049538738	1.241935416	0.214260392	0.435202064	RNF13	protein_coding
ENSG00000083067.23	33.30994341	-0.208642586	0.173500447	-1.202547829	0.229151347	0.452600655	TRPM3	protein_coding
ENSG00000083093.10	1146.750909	-0.011002733	0.051712022	-0.212769345	0.831506875	0.916936385	PALB2	protein_coding
ENSG00000083097.14	1431.931331	0.009379271	0.071202786	0.131726187	0.895200872	0.950264011	DOP1A	protein_coding
ENSG00000083099.11	1667.26192	0.135611282	0.048562182	2.792528596	0.005229784	0.038403941	LYRM2	protein_coding
ENSG00000083123.15	1392.609228	0.054965593	0.070176234	0.783250829	0.433479832	0.651348251	BCKDHB	protein_coding
ENSG00000083168.11	6288.757991	0.022089892	0.05169777	0.427289071	0.669168792	0.823622891	KAT6A	protein_coding
ENSG00000083223.18	2579.991728	0.026741016	0.057058252	0.468661674	0.639311486	0.804693008	TUT7	protein_coding
ENSG00000083290.20	946.9718234	0.153646979	0.08908419	1.724739023	0.084574515	0.248583815	ULK2	protein_coding
ENSG00000083307.12	2562.525286	0.082143111	0.089312768	0.919724168	0.35771692	0.585412503	GRHL2	protein_coding
ENSG00000083312.17	7855.553364	0.007952683	0.046439556	0.171248038	0.864028736	0.933873814	TNPO1	protein_coding
ENSG00000083444.17	9287.156272	0.249524498	0.062581184	3.987212834	6.6854E-05	0.001450124	PLOD1	protein_coding
ENSG00000083454.22	150.5422336	-0.891324044	0.158004983	-5.641113524	1.68954E-08	1.87341E-06	P2RX5	protein_coding

ENSG00000083457.12	1108.640366	-0.273888789	0.071473694	-3.832022279	0.000127094	0.00237597	ITGAE	protein_coding
ENSG00000083520.15	4016.623634	0.156149045	0.053918925	2.895996988	0.00377956	0.030457757	DIS3	protein_coding
ENSG00000083535.16	1149.554323	0.094602435	0.055334477	1.709647238	0.087331125	0.25348766	PIBF1	protein_coding
ENSG00000083544.16	1186.823992	-0.032731038	0.05340685	-0.612862181	0.539967472	0.735616423	TDRD3	protein_coding
ENSG00000083622.8	9.205210753	-1.552406167	0.239007727	-6.495213308	8.29158E-11	2.34625E-08	AC000061.1	lncRNA
ENSG00000083635.8	653.656642	-0.091805361	0.065139586	-1.40936359	0.158727684	0.3642672	NUFIP1	protein_coding
ENSG00000083642.19	3604.057982	0.005471216	0.060238105	0.090826492	0.927630459	0.965725847	PDS5B	protein_coding
ENSG00000083720.13	1977.466492	0.180818686	0.127424981	1.419020707	0.155892979	0.360504583	OXCT1	protein_coding
ENSG00000083750.13	532.6707387	-0.024392604	0.063477019	-0.384274574	0.700774951	0.842529209	RRAGB	protein_coding
ENSG00000083782.8	73.52688138	1.736569984	0.275678539	6.299257067	2.99076E-10	6.75276E-08	EPYC	protein_coding
ENSG00000083799.18	2402.030416	-0.13913296	0.064810969	-2.14675018	0.031813176	0.132563014	CYLD	protein_coding
ENSG00000083807.10	341.3734716	-0.140299833	0.101554879	-1.381517413	0.167119928	0.375914163	SLC27A5	protein_coding
ENSG00000083812.12	578.3967599	0.05218483	0.052629805	0.991545203	0.321419431	0.551219778	ZNF324	protein_coding
ENSG00000083814.13	262.9451582	-0.137169461	0.09082118	-1.51032458	0.130960623	0.32418036	ZNF671	protein_coding
ENSG00000083817.9	238.9185757	0.204426739	0.075428531	2.710204441	0.006724175	0.045734254	ZNF416	protein_coding
ENSG00000083828.16	762.386909	-0.016605778	0.059254413	-0.280245418	0.779289223	0.888374224	ZNF586	protein_coding
ENSG00000083838.16	291.4659781	0.046324248	0.058216562	0.795722844	0.426193146	0.644858666	ZNF446	protein_coding
ENSG00000083844.10	2935.848353	0.198012759	0.068943093	2.872118888	0.004077295	0.032217441	ZNF264	protein_coding
ENSG00000083845.9	42813.89609	-0.129368756	0.07321957	-1.766860368	0.077251616	0.234771857	RPS5	protein_coding
ENSG00000083857.14	22631.06161	0.182542801	0.090667984	2.013310464	0.044081978	0.164142559	FAT1	protein_coding
ENSG00000083896.13	4125.439886	-0.016806732	0.028205626	-0.595864522	0.551265736	0.743546706	YTHDC1	protein_coding
ENSG00000083937.10	4205.764294	0.095834335	0.057753024	1.659382126	0.097038816	0.27043377	CHMP2B	protein_coding
ENSG00000084070.12	3681.616069	-0.293896316	0.076381973	-3.847718294	0.000119223	0.002269717	SMAP2	protein_coding
ENSG00000084072.17	1735.816506	-0.031820973	0.047666867	-0.667570051	0.50440808	0.708594162	PPIE	protein_coding
ENSG00000084073.10	4493.883387	0.025056033	0.047458935	0.527951863	0.597532747	0.776833573	ZMPSTE24	protein_coding
ENSG00000084090.13	7650.397659	-0.07209259	0.045746884	-1.575901646	0.115048472	0.299381606	STARD7	protein_coding
ENSG00000084092.7	2384.384332	0.010931181	0.0508268	0.215067261	0.829714878	0.916289069	NOA1	protein_coding
ENSG00000084093.19	3685.040327	0.05863333	0.043073309	1.361245084	0.173436251	0.384465943	REST	protein_coding
ENSG00000084110.11	95.45748389	0.591754172	0.164078091	3.606539842	0.000310307	0.004768074	HAL	protein_coding
ENSG00000084112.15	4016.610446	0.056525818	0.054511125	1.0369593	0.299754826	0.529481071	SSH1	protein_coding
ENSG00000084207.18	32953.36085	0.037586933	0.088990625	0.422369585	0.672755275	0.825971693	GSTP1	protein_coding
ENSG00000084234.18	29866.44649	0.172562318	0.05760393	2.995669207	0.002738433	0.023991296	APLP2	protein_coding
ENSG00000084444.14	318.4392378	0.007490247	0.105308329	0.07112682	0.943296823	0.973315787	FAM234B	protein_coding
ENSG00000084453.16	53.61658394	1.150158455	0.23900434	4.812291091	1.4921E-06	7.1217E-05	SLCO1A2	protein_coding
ENSG00000084463.8	6012.610322	-0.063069605	0.045198593	-1.395388688	0.16289866	0.36993251	WBP11	protein_coding
ENSG00000084623.12	10262.35974	-0.102610805	0.049456384	-2.074773703	0.038007513	0.148876709	EIF3I	protein_coding
ENSG00000084628.10	29.14806569	-0.067845932	0.198245568	-0.34223177	0.732176478	0.860958534	NKAIN1	protein_coding
ENSG00000084636.18	3312.914101	0.56764547	0.109556461	5.181305296	2.20339E-07	1.52222E-05	COL16A1	protein_coding
ENSG00000084652.16	4301.940742	-0.007513891	0.043131696	-0.174208099	0.861701921	0.932697084	TXLNA	protein_coding
ENSG00000084674.15	1427.669077	-0.753284402	0.298401336	-2.524400231	0.01158959	0.067016925	APOB	protein_coding
ENSG00000084676.15	5261.733047	-0.089118205	0.045574021	-1.955460678	0.05052872	0.179102818	NCOA1	protein_coding
ENSG00000084693.16	1472.252374	-0.065738102	0.058564684	-1.122487095	0.261655391	0.489090078	AGBL5	protein_coding

ENSG00000084710.14	263.2594482	0.001681152	0.138144001	0.012169563	0.990290334	0.995572148	EFR3B	protein_coding
ENSG00000084731.15	755.518192	0.341574302	0.104176929	3.278790273	0.001042531	0.011843161	KIF3C	protein_coding
ENSG00000084733.11	11543.41795	0.024430527	0.045603922	0.535711101	0.59215823	0.772589197	RAB10	protein_coding
ENSG00000084734.9	147.8931186	-0.065198027	0.188689263	-0.3455312	0.729695059	0.859310388	GCKR	protein_coding
ENSG00000084754.12	11270.95092	-0.136865665	0.048240408	-2.837158124	0.004551706	0.034825011	HADHA	protein_coding
ENSG00000084764.12	723.7448909	0.227810126	0.104793651	2.173892449	0.029713213	0.126780838	MAPRE3	protein_coding
ENSG00000084774.14	6086.429775	-0.004304942	0.066221025	-0.065008682	0.948167087	0.975615149	CAD	protein_coding
ENSG00000085063.17	9757.360172	0.164322392	0.079328117	2.071426858	0.038318923	0.149719691	CD59	protein_coding
ENSG00000085117.12	5964.063067	-0.134194979	0.085479301	-1.569911994	0.116435587	0.301605752	CD82	protein_coding
ENSG00000085185.15	1866.81609	0.305158824	0.073513303	4.151069428	3.30925E-05	0.000825457	BCORL1	protein_coding
ENSG00000085224.23	9227.569795	-0.062540553	0.056394445	-1.108984277	0.267436965	0.495413221	ATRX	protein_coding
ENSG00000085231.14	760.3662992	-0.127829279	0.061172181	-2.089663589	0.03664803	0.145148669	AK6	protein_coding
ENSG00000085265.11	289.2590708	0.398719958	0.15426655	2.584617061	0.009748721	0.059384247	FCN1	protein_coding
ENSG00000085274.16	927.3320368	-0.041612074	0.04839875	-0.859775802	0.38991264	0.614423632	MYNN	protein_coding
ENSG00000085276.19	7877.667763	-0.044586288	0.107264162	-0.415668078	0.677652903	0.829263066	MECOM	protein_coding
ENSG00000085365.18	3474.603902	0.067252433	0.054701804	1.229437205	0.218907928	0.440542941	SCAMP1	protein_coding
ENSG00000085377.15	4028.04373	-0.031708613	0.070788412	-0.44793508	0.65420005	0.814074519	PREP	protein_coding
ENSG00000085382.12	771.0624529	0.029199725	0.060807283	0.480201109	0.631084398	0.799673301	HACE1	protein_coding
ENSG00000085415.16	2987.320916	-0.00618405	0.063704958	-0.09707329	0.922668192	0.963413806	SEH1L	protein_coding
ENSG00000085433.16	844.2916961	0.124078594	0.05260276	2.358784844	0.018334883	0.091594541	WDR47	protein_coding
ENSG00000085449.15	3341.922927	-0.051605941	0.053086675	-0.972107247	0.330997197	0.5604929	WDFY1	protein_coding
ENSG00000085465.13	160.1914011	-0.136191125	0.11382836	-1.196460394	0.231516946	0.455572858	OVGP1	protein_coding
ENSG00000085491.17	4712.741459	0.062067678	0.065601005	0.94613913	0.344077628	0.57377433	SLC25A24	protein_coding
ENSG00000085511.20	1776.107359	0.170105113	0.04940309	3.443208003	0.000574857	0.007565854	MAP3K4	protein_coding
ENSG00000085514.16	516.4134884	0.11647255	0.116702694	0.998027942	0.318265809	0.547677889	PILRA	protein_coding
ENSG00000085552.17	1674.624699	-0.163993295	0.131663883	-1.245545031	0.212931473	0.433547787	IGSF9	protein_coding
ENSG00000085563.15	619.7551439	-0.284803035	0.143108411	-1.990120871	0.046577622	0.170005182	ABCB1	protein_coding
ENSG00000085644.14	1945.478339	-0.020032969	0.055769795	-0.359208219	0.719439329	0.853251855	ZNF213	protein_coding
ENSG00000085662.14	2777.385725	0.03922052	0.097949278	0.400416629	0.688849678	0.835661616	AKR1B1	protein_coding
ENSG00000085719.13	8297.764838	0.02851474	0.068553227	0.415950363	0.677446326	0.829240085	CPNE3	protein_coding
ENSG00000085721.13	2630.282424	-0.022124651	0.048639023	-0.454874503	0.649199527	0.810652982	RRN3	protein_coding
ENSG00000085733.16	11627.33327	0.295698038	0.070518685	4.19318705	2.75062E-05	0.000713125	CTTN	protein_coding
ENSG00000085741.13	590.2809311	1.388078738	0.238300985	5.824897187	5.71478E-09	7.63904E-07	WNT11	protein_coding
ENSG00000085760.15	2363.712971	-0.152163966	0.052164402	-2.91700778	0.00353407	0.029008128	MTIF2	protein_coding
ENSG00000085788.14	2163.702084	-0.067565826	0.062928859	-1.073685866	0.282963498	0.511859708	DDHD2	protein_coding
ENSG00000085831.15	2156.386837	0.002687117	0.122634898	0.02191152	0.982518535	0.992392332	TTC39A	protein_coding
ENSG00000085832.17	4635.480533	-0.101424888	0.043916543	-2.309491593	0.020916318	0.100227664	EPS15	protein_coding
ENSG00000085840.13	719.0229128	-0.072257889	0.094535961	-0.764342885	0.444662935	0.660634712	ORC1	protein_coding
ENSG00000085871.9	3443.170231	-0.225231292	0.081331289	-2.769306796	0.005617571	0.040415535	MGST2	protein_coding
ENSG00000085872.15	4019.811972	0.01776888	0.059339026	0.299446768	0.764599182	0.880102979	CHERP	protein_coding
ENSG00000085978.22	1968.596536	0.020443457	0.044962491	0.454678041	0.649340881	0.810736564	ATG16L1	protein_coding
ENSG00000085982.14	2348.001326	-0.014355813	0.048262373	-0.297453524	0.766120285	0.881150355	USP40	protein_coding

ENSG00000085998.14	3455.233959	0.052551016	0.048803326	1.076791694	0.281573337	0.510482981	POMGNT1	protein_coding
ENSG00000085999.13	466.7300463	-0.005356709	0.091416446	-0.058596774	0.95327328	0.978052776	RAD54L	protein_coding
ENSG00000086015.22	5656.613701	-0.022241669	0.056543727	-0.39335343	0.694058461	0.838508055	MAST2	protein_coding
ENSG00000086061.16	10123.82969	0.011442812	0.063199817	0.181057682	0.8563223	0.929946342	DNAJA1	protein_coding
ENSG00000086062.13	12157.23573	0.187036996	0.072772706	2.570153101	0.010165358	0.061136531	B4GALT1	protein_coding
ENSG00000086065.14	4241.573526	0.025585672	0.063375342	0.403716506	0.6864212	0.834486725	CHMP5	protein_coding
ENSG00000086102.19	3426.173514	-0.00709123	0.052600465	-0.134813074	0.892759669	0.948844414	NFX1	protein_coding
ENSG00000086159.13	26.3206357	0.499107767	0.174005704	2.868341405	0.0041263	0.032421886	AQP6	protein_coding
ENSG00000086189.10	696.9800057	-0.054014392	0.053670024	-1.006416394	0.314215311	0.543527464	DIMT1	protein_coding
ENSG00000086200.17	1409.438285	-0.020662388	0.048089416	-0.42966603	0.667438598	0.822422502	IPO11	protein_coding
ENSG00000086205.18	284.1915119	-0.057170958	0.165392972	-0.345667397	0.729592689	0.859269487	FOLH1	protein_coding
ENSG00000086232.13	13854.25125	0.019738391	0.048463414	0.407284361	0.683799148	0.832972227	EIF2AK1	protein_coding
ENSG00000086288.12	18.77301995	-0.094131706	0.137162769	-0.686277383	0.492538209	0.699529508	NME8	protein_coding
ENSG00000086289.12	861.819302	0.021515864	0.128459164	0.167491861	0.866983051	0.935531904	EPDR1	protein_coding
ENSG00000086300.16	1318.757451	-0.221390002	0.095705464	-2.313243089	0.020709278	0.099509119	SNX10	protein_coding
ENSG00000086475.15	3282.148351	-0.147500099	0.051405456	-2.869347147	0.004113201	0.032371492	SEPHS1	protein_coding
ENSG00000086504.17	2463.545107	-0.151114488	0.065070886	-2.322305693	0.020216481	0.097962149	MRPL28	protein_coding
ENSG00000086506.3	6.702940002	-0.20198613	0.236794844	-0.85300054	0.393659005	0.617539563	HBQ1	protein_coding
ENSG00000086544.3	2736.110782	0.231481679	0.082463723	2.807072846	0.004999393	0.037209419	ITPKC	protein_coding
ENSG00000086548.9	40806.83442	0.783848297	0.206839553	3.789644124	0.000150863	0.002740379	CEACAM6	protein_coding
ENSG00000086570.12	80.86379256	0.111390044	0.158190593	0.704150874	0.481338821	0.69028227	FAT2	protein_coding
ENSG00000086589.12	3624.174721	-0.027981894	0.042975171	-0.651117691	0.514970517	0.716695215	RBM22	protein_coding
ENSG00000086598.11	16593.82494	-0.000845608	0.050849133	-0.016629735	0.986732003	0.993775242	TMED2	protein_coding
ENSG00000086619.14	860.7851053	0.024471652	0.10854724	0.225447024	0.821631533	0.912302842	ERO1B	protein_coding
ENSG00000086666.19	3174.006066	0.02209201	0.046388061	0.476243453	0.633900946	0.801374495	ZFAND6	protein_coding
ENSG00000086696.11	2049.81796	-0.005697875	0.16611441	-0.034300906	0.972637202	0.987180887	HSD17B2	protein_coding
ENSG00000086712.13	2117.636908	-0.100310108	0.063372713	-1.582859628	0.113453462	0.297083751	TXLNG	protein_coding
ENSG00000086717.18	35.9174948	0.06246105	0.125102821	0.499277707	0.617583758	0.790380247	PPEF1	protein_coding
ENSG00000086730.17	698.4013391	-0.18815823	0.097968279	-1.920603598	0.054781701	0.188661918	LAT2	protein_coding
ENSG00000086758.16	24866.54647	0.094707819	0.052374926	1.808266391	0.07056505	0.220908497	HUWE1	protein_coding
ENSG00000086827.9	1349.939536	0.00459033	0.054389954	0.084396652	0.932741069	0.968238421	ZW10	protein_coding
ENSG00000086848.15	414.4961311	-0.159322026	0.05336921	-2.985279827	0.00283319	0.024567997	ALG9	protein_coding
ENSG00000086967.10	40.81552577	-0.506040065	0.177298338	-2.854172633	0.004314908	0.033481256	MYBPC2	protein_coding
ENSG00000086991.13	237.1207155	0.576415424	0.114843543	5.01913653	5.19043E-07	3.04829E-05	NOX4	protein_coding
ENSG00000087008.16	1142.728656	-0.139494355	0.05882753	-2.371242776	0.017728382	0.089612539	ACOX3	protein_coding
ENSG00000087053.20	3398.378296	-0.023602478	0.054942953	-0.429581543	0.667500065	0.822444589	MTMR2	protein_coding
ENSG00000087074.8	4948.982161	0.115028094	0.078088076	1.473055806	0.140735989	0.338588427	PPP1R15A	protein_coding
ENSG00000087076.9	400.182226	-0.392827653	0.132932012	-2.955101981	0.003125657	0.026475399	HSD17B14	protein_coding
ENSG00000087077.14	2985.245423	-0.004403631	0.102481547	-0.04296999	0.965725456	0.984384417	TRIP6	protein_coding
ENSG00000087085.16	1677.523707	0.631161827	0.183977343	3.43064975	0.000602138	0.007827039	ACHE	protein_coding
ENSG00000087086.15	169327.8579	-0.299472069	0.079704478	-3.757280358	0.00017177	0.003019452	FTL	protein_coding
ENSG00000087087.20	6061.514194	-0.016892	0.04667063	-0.361940689	0.717396353	0.852280973	SRRT	protein_coding

ENSG00000087088.20	3168.741745	-0.27717444	0.071793158	-3.860736114	0.000113046	0.002173469	BAX		protein_coding
ENSG00000087095.13	1415.42737	0.024564348	0.050316461	0.488197053	0.625410272	0.795606581	NLK		protein_coding
ENSG00000087111.21	3785.918972	0.214409452	0.057141652	3.752244549	0.000175258	0.003073334	PIGS		protein_coding
ENSG00000087116.16	4941.266195	0.685144907	0.124549942	5.500965277	3.77718E-08	3.63351E-06	ADAMTS2		protein_coding
ENSG00000087128.10	53.62688796	1.346053065	0.347460784	3.873971183	0.000107076	0.00208883	TMPRSS11E		protein_coding
ENSG00000087152.15	4348.004339	0.157915546	0.049845532	3.168098337	0.001534396	0.015769901	ATXN7L3		protein_coding
ENSG00000087157.19	1222.03154	0.056119895	0.040716016	1.378324815	0.168103025	0.377249228	PGS1		protein_coding
ENSG00000087191.13	4502.265314	-0.009880099	0.046188325	-0.213909003	0.830618019	0.916682182	PSMC5		protein_coding
ENSG00000087206.17	1113.021461	-0.079997954	0.040474298	-1.976512435	0.048096769	0.173603321	UIMC1		protein_coding
ENSG00000087237.12	155.2715063	-0.522357209	0.111153256	-4.699432378	2.60886E-06	0.000111676	CETP		protein_coding
ENSG00000087245.13	20878.99246	0.733604612	0.126352333	5.806023464	6.3974E-09	8.34669E-07	MMP2		protein_coding
ENSG00000087250.9	9.58804518	-0.113385423	0.235069191	-0.482349142	0.629557943	0.798653701	MT3		protein_coding
ENSG00000087253.13	2304.50375	0.245159308	0.093561241	2.620308425	0.008785027	0.055138289	LPCAT2		protein_coding
ENSG00000087258.16	1248.411188	-0.196618005	0.192752289	-1.020055355	0.307702209	0.53714454	GNAO1		protein_coding
ENSG00000087263.17	1862.247431	-0.047373445	0.041445126	-1.143040183	0.253021908	0.47985043	OGFOD1		protein_coding
ENSG00000087266.17	3805.719814	0.100978952	0.052548741	1.921624553	0.054653017	0.188388576	SH3BP2		protein_coding
ENSG00000087269.16	2035.646451	-0.067382732	0.057663422	-1.168552431	0.242584003	0.468534842	NOP14		protein_coding
ENSG00000087274.17	12460.43175	-0.031697969	0.048502223	-0.653536426	0.513410514	0.715649325	ADD1		protein_coding
ENSG00000087299.12	908.4348723	-0.020299557	0.061079033	-0.332349019	0.73962573	0.865438763	L2HGDH		protein_coding
ENSG00000087301.9	878.6440677	0.079867859	0.070938071	1.125881455	0.2602157	0.487422536	TXNDC16		protein_coding
ENSG00000087302.9	6244.613844	-0.082095442	0.051372983	-1.598027551	0.110036846	0.291202842	RTRAF		protein_coding
ENSG00000087303.18	2209.024316	0.435003508	0.10197561	4.26576031	1.99223E-05	0.000555085	NID2		protein_coding
ENSG00000087338.5	2643.699534	-0.019560128	0.051325963	-0.381096179	0.703131882	0.844110803	GMCL1		protein_coding
ENSG00000087365.16	11276.67241	0.016043007	0.038651787	0.415065088	0.678094256	0.829354821	SF3B2		protein_coding
ENSG00000087448.11	2296.026012	-0.029993507	0.086020123	-0.348680127	0.727329466	0.857753136	KLHL42		protein_coding
ENSG00000087460.27	70081.39672	0.051211252	0.070963085	0.721660441	0.470503269	0.681761147	GNAS		protein_coding
ENSG00000087470.18	4850.937417	0.003565337	0.0544816	0.065441116	0.947822789	0.975463848	DNM1L		protein_coding
ENSG00000087494.16	146.3314772	0.473077032	0.141911143	3.333614407	0.000857254	0.010190006	PTHLH		protein_coding
ENSG00000087495.17	197.6303863	1.389900427	0.21471671	6.473182383	9.596E-11	2.66347E-08	PHACTR3		protein_coding
ENSG00000087502.18	2431.069883	0.11366967	0.065095479	1.746199155	0.080776352	0.241459121	ERGIC2		protein_coding
ENSG00000087510.7	410.699856	0.654165776	0.195473205	3.346575178	0.000818165	0.009840787	TFAP2C		protein_coding
ENSG00000087586.18	2761.208452	0.080014017	0.104567167	0.765192544	0.444156899	0.660152978	AURKA		protein_coding
ENSG00000087589.16	175.0490146	-0.212302621	0.11199824	-1.89558891	0.058014425	0.195688102	CASS4		protein_coding
ENSG00000087842.11	702.624918	-0.26320352	0.092813418	-2.835834796	0.004570608	0.034924319	PIR		protein_coding
ENSG00000087884.14	256.5937907	0.049126372	0.077406001	0.634658444	0.525651202	0.724493229	AAMDC		protein_coding
ENSG00000087903.13	983.3743884	-0.067682571	0.093994342	-0.720070689	0.471481474	0.682651034	RFX2		protein_coding
ENSG00000087995.16	1281.64463	0.019900247	0.048450637	0.410732416	0.681268755	0.831190988	METTL2A		protein_coding
ENSG00000088002.12	727.8678374	0.081262347	0.187220177	0.434046951	0.664254347	0.82051739	SULT2B1		protein_coding
ENSG00000088035.18	1113.957382	-0.101691169	0.053277048	-1.90872379	0.056297733	0.191962933	ALG6		protein_coding
ENSG00000088038.19	3650.49756	0.114613437	0.056205722	2.03917736	0.04143233	0.157506467	CNOT3		protein_coding
ENSG00000088053.11	35.94743196	0.208765466	0.136438497	1.530106754	0.125990307	0.316501134	GP6		protein_coding
ENSG00000088179.9	1681.598059	-0.090541894	0.049487043	-1.82960806	0.067308568	0.214596536	PTPN4		protein_coding

ENSG00000088205.13	5760.156126	-0.101560945	0.047044972	-2.158805515	0.030865258	0.129953176	DDX18	protein_coding
ENSG00000088247.17	14084.38861	-0.077286411	0.055168256	-1.400921771	0.161237468	0.367655732	KHSRP	protein_coding
ENSG00000088256.9	6543.521493	-0.009331993	0.059866434	-0.155880217	0.876127439	0.940138031	GNA11	protein_coding
ENSG00000088280.19	970.0102664	0.273389288	0.101394515	2.696292678	0.007011603	0.047087639	ASAP3	protein_coding
ENSG00000088298.13	2500.794749	-0.067259476	0.057422319	-1.171312441	0.241473212	0.467259244	EDEM2	protein_coding
ENSG00000088305.18	684.6194839	0.055977465	0.135301614	0.413723559	0.679076571	0.830014248	DNMT3B	protein_coding
ENSG00000088320.4	120.5209021	-0.149785802	0.120668198	-1.241303045	0.21449382	0.43549351	REM1	protein_coding
ENSG00000088325.16	7038.955961	-0.023521572	0.096408611	-0.243977916	0.807247923	0.904162389	TPX2	protein_coding
ENSG00000088340.17	1896.853857	0.015621677	0.175673025	0.088924735	0.929141726	0.966398763	FER1L4	transcribed_unitary_pseudogene
ENSG00000088356.6	1113.452551	-0.144662282	0.068206266	-2.120952984	0.033925759	0.138011838	PDRG1	protein_coding
ENSG00000088367.23	8861.879758	-0.156082524	0.083967487	-1.858844765	0.063049145	0.206134097	EPB41L1	protein_coding
ENSG00000088386.17	540.143154	0.329688066	0.249294523	1.322484196	0.186006968	0.40084993	SLC15A1	protein_coding
ENSG00000088387.19	4381.878997	0.17105007	0.064533436	2.650565061	0.008035725	0.051939007	DOCK9	protein_coding
ENSG00000088448.14	5117.442942	0.2544549	0.06484026	3.924335004	8.69696E-05	0.001780957	ANKRD10	protein_coding
ENSG00000088451.11	1133.957052	0.053917949	0.058394074	0.923346242	0.355826797	0.5837477	TGDS	protein_coding
ENSG00000088538.13	206.1572071	0.154236779	0.151530315	1.017860875	0.308744077	0.538165626	DOCK3	protein_coding
ENSG00000088543.15	206.3459731	0.017474142	0.105071582	0.166307024	0.867915339	0.93597418	C3orf18	protein_coding
ENSG00000088682.14	2832.859079	-0.086855523	0.055697783	-1.559407175	0.118900039	0.305555641	COQ9	protein_coding
ENSG00000088726.16	44.44694222	0.183750704	0.232763795	0.789429919	0.429860775	0.6481003	TMEM40	protein_coding
ENSG00000088727.13	534.9635435	-0.052120268	0.067153548	-0.776135731	0.437668848	0.654553712	KIF9	protein_coding
ENSG00000088756.13	305.527005	0.339642937	0.127501561	2.663833555	0.007725578	0.050506528	ARHGAP28	protein_coding
ENSG00000088766.12	603.1107336	-0.323483057	0.069163702	-4.677063959	2.91012E-06	0.000121121	CRLS1	protein_coding
ENSG00000088782.5	0.943701927	-1.072558154	0.543178569	-1.974595862	0.048314029		DEFB127	protein_coding
ENSG00000088808.18	2726.235873	0.023710062	0.056322082	0.420972747	0.673774983	0.826677153	PPP1R13B	protein_coding
ENSG00000088812.18	6798.841078	-0.099069943	0.078085881	-1.268730549	0.204537183	0.423989954	ATRN	protein_coding
ENSG00000088826.18	1536.078415	0.055131194	0.101342477	0.544008744	0.586435456	0.768636257	SMOX	protein_coding
ENSG00000088827.12	1338.486693	-0.021010276	0.140307657	-0.149744327	0.880966334	0.942776767	SIGLEC1	protein_coding
ENSG00000088832.18	16618.9465	-0.146153314	0.053448836	-2.734452702	0.006248407	0.0434963	FKBP1A	protein_coding
ENSG00000088833.17	4996.317521	-0.169585941	0.049224689	-3.445139915	0.000570764	0.007532458	NSFL1C	protein_coding
ENSG00000088836.14	1417.683561	0.513261746	0.143436021	3.578332298	0.000345794	0.005171744	SLC4A11	protein_coding
ENSG00000088854.12	1362.472494	-0.136694095	0.107506383	-1.271497473	0.203551726	0.422912823	C20orf194	protein_coding
ENSG00000088876.13	661.8328613	-0.16137898	0.054203231	-2.977294456	0.002908046	0.025058015	ZNF343	protein_coding
ENSG00000088881.21	834.697483	-0.044509351	0.130158424	-0.341962888	0.732378822	0.860958534	EBF4	protein_coding
ENSG00000088882.8	1181.938677	0.682786179	0.134710284	5.068552754	4.00852E-07	2.49542E-05	CPXM1	protein_coding
ENSG00000088888.18	12800.12365	-0.13154484	0.061416561	-2.141846391	0.032205845	0.133558632	MAVS	protein_coding
ENSG00000088899.15	3329.838585	0.007615378	0.12674828	0.060082693	0.952089774	0.977481826	LZTS3	protein_coding
ENSG00000088926.14	25.12940025	-0.938680511	0.260327014	-3.605774508	0.000311223	0.00477878	F11	protein_coding
ENSG00000088930.8	7327.786263	-0.059476534	0.054587687	-1.089559512	0.275907226	0.504667949	XRN2	protein_coding
ENSG00000088970.16	1224.657637	-0.313863559	0.091308957	-3.43737976	0.000587371	0.007674928	KIZ	protein_coding
ENSG00000088986.11	11805.22733	-0.044568947	0.05437883	-0.819601064	0.412443567	0.633121506	DYNLL1	protein_coding
ENSG00000088992.18	4265.248576	0.046043005	0.164248432	0.280325385	0.779227877	0.888374224	TESC	protein_coding
ENSG00000089006.17	6586.983418	-0.213942096	0.057413577	-3.726332807	0.000194286	0.003320155	SNX5	protein_coding

ENSG00000089009.15	39089.86988	-0.260501061	0.059566368	-4.373290983	1.22387E-05	0.000380948	RPL6	protein_coding
ENSG00000089012.14	310.8681277	-0.594017377	0.139467379	-4.259185056	2.05174E-05	0.000569118	SIRPG	protein_coding
ENSG00000089022.14	2329.825731	-0.142983178	0.041375009	-3.455785988	0.000548691	0.007303084	MAPKAPK5	protein_coding
ENSG00000089041.17	314.4868103	-0.261297047	0.106650531	-2.450030444	0.014284414	0.077450777	P2RX7	protein_coding
ENSG00000089048.14	2289.661723	-0.270900403	0.077088839	-3.514132592	0.000441193	0.006197887	ESF1	protein_coding
ENSG00000089050.16	2285.011016	-0.294196755	0.058220297	-5.05316479	4.34549E-07	2.66641E-05	RBBP9	protein_coding
ENSG00000089053.13	6249.199064	-0.147621826	0.041788933	-3.532557939	0.00041156	0.00587451	ANAPC5	protein_coding
ENSG00000089057.15	2356.118141	0.028035946	0.07669401	0.365555872	0.714696509	0.850914722	SLC23A2	protein_coding
ENSG00000089060.11	2759.093477	0.121290121	0.056687485	2.139627838	0.032384855	0.133994951	SLC8B1	protein_coding
ENSG00000089063.15	7350.517656	-0.207835436	0.05943197	-3.497030921	0.000470467	0.00650843	TMEM230	protein_coding
ENSG00000089091.16	193.5275928	-0.006946201	0.080898712	-0.08586294	0.931575372	0.967567452	DZANK1	protein_coding
ENSG00000089094.19	2465.883187	-0.105411724	0.051295852	-2.05497559	0.039881363	0.153715738	KDM2B	protein_coding
ENSG00000089101.19	25.83315808	0.209651501	0.135641195	1.545632961	0.122193236	0.310938082	CFAP61	protein_coding
ENSG00000089116.4	12.10282569	0.288911146	0.266379493	1.084584787	0.278105596	0.507007804	LHX5	protein_coding
ENSG00000089123.16	757.8807693	-0.089235749	0.054594023	-1.634533325	0.102146915	0.278912244	TASP1	protein_coding
ENSG00000089127.14	5944.848994	0.060917446	0.104402261	0.583487808	0.559564962	0.749175444	OAS1	protein_coding
ENSG00000089154.11	11261.16201	0.084839202	0.05844523	1.451601811	0.146612355	0.347280893	GCN1	protein_coding
ENSG00000089157.16	96689.42431	-0.200137836	0.064585268	-3.098815588	0.001942959	0.018723647	RPLP0	protein_coding
ENSG00000089159.16	4284.955331	0.234204503	0.064499697	3.631094635	0.000282222	0.004441449	PXN	protein_coding
ENSG00000089163.4	43.23717013	-0.173605978	0.092454964	-1.877735608	0.060417343	0.200701826	SIRT4	protein_coding
ENSG00000089169.15	19.5165727	-0.252892854	0.168254347	-1.50303905	0.132828975	0.326966515	RPH3A	protein_coding
ENSG00000089177.19	2399.234927	-0.099104359	0.066148468	-1.498210946	0.134078453	0.328908845	KIF16B	protein_coding
ENSG00000089195.15	1516.868123	-0.286280536	0.068458516	-4.181810431	2.89197E-05	0.000742625	TRMT6	protein_coding
ENSG00000089199.10	258.9922987	-0.876053067	0.243616675	-3.596030804	0.00032311	0.004912088	CHGB	protein_coding
ENSG00000089220.5	11883.75553	-0.343073867	0.058409818	-5.873565087	4.26521E-09	6.13417E-07	PEBP1	protein_coding
ENSG00000089225.20	37.91790843	1.396552822	0.264944154	5.271121489	1.35593E-07	1.03299E-05	TBX5	protein_coding
ENSG00000089234.16	1665.345244	-0.032129278	0.038162448	-0.841908202	0.399839339	0.622678825	BRAP	protein_coding
ENSG00000089248.7	7402.327943	-0.248390262	0.05592202	-4.441725526	8.92403E-06	0.000296179	ERP29	protein_coding
ENSG00000089250.19	93.56891766	-0.346923749	0.224170616	-1.547587969	0.12172153	0.310154316	NOS1	protein_coding
ENSG00000089280.19	11005.19223	-0.043126252	0.044793274	-0.96278411	0.335655876	0.565180685	FUS	protein_coding
ENSG00000089289.16	3108.471636	-0.111352991	0.05320716	-2.092819655	0.036365257	0.144386	IGBP1	protein_coding
ENSG00000089327.15	11541.98994	-0.039909081	0.09984515	-0.399709758	0.689370304	0.835873022	FXYD5	protein_coding
ENSG00000089335.21	1213.423154	-0.086185338	0.0745516	-1.15604948	0.247660919	0.474220927	ZNF302	protein_coding
ENSG00000089351.14	5206.609701	0.04035765	0.077615103	0.519971606	0.603083366	0.780881409	GRAMD1A	protein_coding
ENSG00000089356.18	196.640279	0.331083886	0.121221298	2.731235275	0.006309741	0.043804289	FXYD3	protein_coding
ENSG00000089472.16	5533.289875	-0.059470802	0.137686147	-0.431930175	0.665792163	0.821294163	HEPH	protein_coding
ENSG00000089486.17	1493.637328	-0.045939835	0.098981465	-0.464125635	0.642557736	0.806683592	CDIP1	protein_coding
ENSG00000089505.18	109.9966915	0.184903794	0.076082418	2.4303091	0.015085951	0.080366807	CMTM1	protein_coding
ENSG00000089558.9	61.49863384	-0.252438176	0.123035618	-2.051748753	0.040194086	0.154429912	KCNH4	protein_coding
ENSG00000089597.18	27718.03796	0.110021151	0.047812556	2.301093292	0.021386358	0.101675032	GANAB	protein_coding
ENSG00000089639.11	2721.662032	-0.147973216	0.067606692	-2.188736235	0.028616017	0.123550992	GMIP	protein_coding
ENSG00000089682.16	1146.458778	-0.067692788	0.06093547	-1.110893016	0.266614402	0.494646449	RBM41	protein_coding

ENSG00000089685.15	2301.618421	0.01956676	0.098454161	0.198739797	0.842466291	0.922508634	BIRC5	protein_coding
ENSG00000089692.9	634.6980055	-0.491793142	0.144194252	-3.410629302	0.000648131	0.008251132	LAG3	protein_coding
ENSG00000089693.11	12306.42881	-0.082089867	0.056568637	-1.451155102	0.146736676	0.347480932	MLF2	protein_coding
ENSG00000089723.10	351.3252411	0.353639186	0.116539982	3.034488075	0.002409443	0.021851465	OTUB2	protein_coding
ENSG00000089737.17	7349.194302	-0.002010501	0.03694281	-0.054421987	0.956598962	0.979770735	DDX24	protein_coding
ENSG00000089775.12	1074.96666	-0.145859012	0.054432965	-2.679608062	0.007370841	0.048821877	ZBTB25	protein_coding
ENSG00000089818.18	1567.208389	-0.039871873	0.049969405	-0.797925697	0.42491361	0.643900327	NECAP1	protein_coding
ENSG00000089820.16	2262.393964	-0.161722683	0.109559638	-1.476115522	0.139912883	0.337166603	ARHGAP4	protein_coding
ENSG00000089847.12	89.87673389	-0.121512566	0.099828179	-1.217217095	0.223521628	0.446213842	ANKRD24	protein_coding
ENSG00000089876.12	1895.92017	0.180866411	0.06907622	2.618359979	0.008835354	0.055342567	DHX32	protein_coding
ENSG00000089902.10	3889.660381	0.122115578	0.067052933	1.821181756	0.068579233	0.217028121	RCOR1	protein_coding
ENSG00000089916.18	2090.058604	0.027907638	0.051584623	0.541006927	0.588502808	0.770310791	GPATCH2L	protein_coding
ENSG00000090006.18	14398.59072	0.235401893	0.096246235	2.445829619	0.014451928	0.078077319	LTBP4	protein_coding
ENSG00000090013.11	4473.798543	-0.275045953	0.090796484	-3.029257756	0.002451554	0.022104568	BLVRB	protein_coding
ENSG00000090020.11	9537.895736	0.012967753	0.098825624	0.131218532	0.895602436	0.950498453	SLC9A1	protein_coding
ENSG00000090054.15	4974.881563	0.029550453	0.045575463	0.648385132	0.516735884	0.718067414	SPTLC1	protein_coding
ENSG00000090060.18	14791.97688	-0.035413276	0.040055642	-0.884102075	0.376641113	0.602462643	PAPOLA	protein_coding
ENSG00000090061.17	894.1833228	0.037314444	0.051069767	0.730656229	0.464989158	0.676916968	CCNK	protein_coding
ENSG00000090097.22	1739.267052	0.046370457	0.069049815	0.671550777	0.501869723	0.706996989	PCBP4	protein_coding
ENSG00000090104.12	4742.569213	-0.184785462	0.127804895	-1.44584025	0.148222014	0.349724982	RGS1	protein_coding
ENSG00000090238.12	2933.218441	0.105939063	0.075872091	1.396285014	0.162628685	0.369651724	YPEL3	protein_coding
ENSG00000090263.16	1562.814444	-0.317126606	0.066106468	-4.79720994	1.60891E-06	7.53887E-05	MRPS33	protein_coding
ENSG00000090266.13	2219.706341	-0.219037553	0.063481724	-3.450403342	0.00055975	0.007405042	NDUFB2	protein_coding
ENSG00000090273.14	6090.250362	-0.098878477	0.055817843	-1.771449278	0.07648602	0.233209579	NUDC	protein_coding
ENSG00000090316.16	4629.738831	0.11656136	0.051151323	2.278755504	0.022681603	0.105892032	MAEA	protein_coding
ENSG00000090339.9	4440.597207	0.037828938	0.111526492	0.339192396	0.734464798	0.862178021	ICAM1	protein_coding
ENSG00000090372.15	6793.779071	0.054620799	0.051073516	1.069454445	0.284864946	0.514146775	STRN4	protein_coding
ENSG00000090376.11	1415.364281	0.066304772	0.137882419	0.480879091	0.630602434	0.799321749	IRAK3	protein_coding
ENSG00000090382.7	73810.89078	-0.363707445	0.183057305	-1.986850213	0.046938998	0.170782393	LYZ	protein_coding
ENSG00000090402.8	2772.202304	-0.499777697	0.293680048	-1.701776137	0.088797341	0.256225377	SI	protein_coding
ENSG00000090432.7	1922.076418	0.030876403	0.046918962	0.658079419	0.5104871	0.713705809	MUL1	protein_coding
ENSG00000090447.12	1351.080967	-0.154278336	0.065969916	-2.338616539	0.019355288	0.094790446	TFAP4	protein_coding
ENSG00000090470.15	1203.599116	-0.007423359	0.043909426	-0.169060723	0.865748879	0.934889428	PDCD7	protein_coding
ENSG00000090487.11	5008.012545	-0.033792374	0.041680207	-0.81075349	0.417507248	0.637749347	SPG21	protein_coding
ENSG00000090512.12	4.107933208	0.303885814	0.333973743	0.909909298	0.362870346	0.590228102	FETUB	protein_coding
ENSG00000090520.11	2605.516043	-9.33093E-06	0.063860954	-0.000146113	0.999883419	0.999975208	DNAJB11	protein_coding
ENSG00000090530.10	1147.996086	-0.32445911	0.15046276	-2.156408065	0.031051815	0.130574637	P3H2	protein_coding
ENSG00000090534.20	45.81530528	0.85116429	0.190202214	4.475049331	7.63937E-06	0.000262747	THPO	protein_coding
ENSG00000090539.15	447.9887129	0.387762118	0.119320305	3.249758016	0.001155032	0.012810601	CHRD	protein_coding
ENSG00000090554.13	18.41159303	-0.428079472	0.116309394	-3.680523611	0.000232756	0.003820259	FLT3LG	protein_coding
ENSG00000090565.17	2694.034681	-0.165684779	0.078097138	-2.121521765	0.033877918	0.137891014	RAB11FIP3	protein_coding
ENSG00000090581.10	2601.933787	0.016094291	0.055308998	0.290988655	0.771059996	0.88416054	GNPTG	protein_coding

ENSG00000090612.22	1083.16893	0.005468631	0.081618284	0.067002519	0.946579698	0.974948675	ZNF268	protein_coding
ENSG00000090615.15	7298.571309	0.093693312	0.056155244	1.668469523	0.095222557	0.267073011	GOLGA3	protein_coding
ENSG00000090621.15	10323.91784	-0.18296768	0.066112357	-2.767526209	0.00564835	0.040603458	PABPC4	protein_coding
ENSG00000090659.18	478.1729031	-0.090041202	0.128854534	-0.698781788	0.484688412	0.692591386	CD209	protein_coding
ENSG00000090661.12	1002.66533	-0.248118665	0.159685673	-1.55379415	0.120233526	0.307921478	CERS4	protein_coding
ENSG00000090674.16	1241.219485	0.201986254	0.056286769	3.588521	0.000332559	0.005016245	MCOLN1	protein_coding
ENSG00000090686.16	3702.097012	-0.105996862	0.050290069	-2.107709613	0.035056115	0.140919459	USP48	protein_coding
ENSG00000090776.6	5666.458313	0.156303181	0.091923842	1.700355188	0.089064136	0.256570889	EFNB1	protein_coding
ENSG00000090857.14	2416.052672	0.03759346	0.065620161	0.572894968	0.566715812	0.754645529	PDPR	protein_coding
ENSG00000090861.17	8279.400399	-0.005194004	0.064827104	-0.080120875	0.93614112	0.969767071	AARS1	protein_coding
ENSG00000090863.12	14283.10178	0.221790405	0.04614074	4.806823702	1.53347E-06	7.27139E-05	GLG1	protein_coding
ENSG00000090889.12	1606.850003	0.096372867	0.092752756	1.039029682	0.298790932	0.528640375	KIF4A	protein_coding
ENSG00000090905.19	4213.578069	-0.064363271	0.04578192	-1.405866581	0.159763747	0.365591264	TNRC6A	protein_coding
ENSG00000090924.15	3226.393623	0.26529111	0.076167699	3.482987058	0.000495852	0.006756647	PLEKHG2	protein_coding
ENSG00000090932.11	45.22899516	0.658339982	0.221418608	2.973282093	0.002946335	0.025294021	DLL3	protein_coding
ENSG00000090971.5	480.7748724	0.165865947	0.1033557	1.60480697	0.108536292	0.288924006	NAT14	protein_coding
ENSG00000090975.13	1491.956754	0.084073235	0.077420704	1.085927021	0.277511281	0.506411604	PITPNM2	protein_coding
ENSG00000090989.18	1967.167631	0.0448303	0.047294333	0.947900049	0.34318034	0.572920242	EXOC1	protein_coding
ENSG00000091009.8	1273.655058	-0.108654071	0.044451044	-2.444353613	0.014511196	0.078289071	RBM27	protein_coding
ENSG00000091010.6	6.315972775	0.416850646	0.131386452	3.172706466	0.001510251	0.01559901	POU4F3	protein_coding
ENSG00000091039.17	4742.983026	0.038847777	0.059037778	0.658015577	0.510528122	0.713707643	OSBPL8	protein_coding
ENSG00000091073.19	3206.596992	-0.098572192	0.085298847	-1.155609902	0.247840756	0.474336011	DTX2	protein_coding
ENSG00000091106.19	129.3664547	-0.114957919	0.08762418	-1.311942887	0.189539406	0.405039166	NLRC4	protein_coding
ENSG00000091127.14	1749.759865	0.024412014	0.084282743	0.28964427	0.772088396	0.884611033	PUS7	protein_coding
ENSG00000091128.13	22.60242374	-0.037602879	0.153994407	-0.244183406	0.807088778	0.904156503	LAMB4	protein_coding
ENSG00000091129.21	1072.260658	-0.297269902	0.183107975	-1.623467801	0.104489441	0.282780796	NRCAM	protein_coding
ENSG00000091136.15	11161.46002	0.226135649	0.07613189	2.97031439	0.002974951	0.02548446	LAMB1	protein_coding
ENSG00000091137.14	37.81882873	-0.108608049	0.111858794	-0.970938847	0.331578732	0.56113423	SLC26A4	protein_coding
ENSG00000091138.13	748.8084388	-1.211912778	0.286629986	-4.228143722	2.35627E-05	0.000633234	SLC26A3	protein_coding
ENSG00000091140.14	4943.451061	-0.145144574	0.061083234	-2.376176958	0.017493071	0.088762875	DLD	protein_coding
ENSG00000091157.14	1351.5587	-0.046973498	0.062224627	-0.754902046	0.450307758	0.66514342	WDR7	protein_coding
ENSG00000091164.13	3857.204001	-0.15407636	0.052930272	-2.9109308	0.003603538	0.029445225	TXNL1	protein_coding
ENSG00000091181.20	24.76873779	-0.369197485	0.147691581	-2.499786931	0.012426802	0.070236415	IL5RA	protein_coding
ENSG00000091262.16	645.6399859	-0.13971449	0.131834984	-1.059767946	0.289250183	0.518987739	ABCC6	protein_coding
ENSG00000091317.8	5786.768937	0.078305614	0.055981802	1.398769086	0.16188224	0.368661738	CMTM6	protein_coding
ENSG00000091409.15	17908.71509	0.075006879	0.089701901	0.836179374	0.403054	0.624846292	ITGA6	protein_coding
ENSG00000091428.18	441.0604166	-0.055910096	0.101224644	-0.552336802	0.580717621	0.764460374	RAPGEF4	protein_coding
ENSG00000091436.17	7414.140724	-0.201087176	0.112114567	-1.793586516	0.07287919	0.225672069	MAP3K20	protein_coding
ENSG00000091482.8	109.8041996	-1.289713893	0.308484841	-4.180801527	2.90483E-05	0.000745488	SMPX	protein_coding
ENSG00000091483.7	3885.695127	0.004822853	0.059164473	0.081516028	0.935031579	0.969411588	FH	protein_coding
ENSG00000091490.11	12543.9324	-0.072339258	0.07400243	-0.977525444	0.328309088	0.557791145	SEL1L3	protein_coding
ENSG00000091513.16	245.2050719	0.594693542	0.231921231	2.564204832	0.01034125	0.061816273	TF	protein_coding

ENSG00000091527.16	9502.20899	-0.026641595	0.043008177	-0.619454174	0.535617202	0.732276803	CDV3	protein_coding
ENSG00000091536.19	72.09077556	0.189422417	0.109658457	1.727385396	0.084098472	0.247752778	MYO15A	protein_coding
ENSG00000091542.9	4453.729132	-0.008131565	0.04570236	-0.177924389	0.858782357	0.931240938	ALKBH5	protein_coding
ENSG00000091583.11	102.0984005	-1.990073752	0.258672052	-7.693423924	1.43249E-14	1.20045E-11	APOH	protein_coding
ENSG00000091592.16	1159.046265	-0.21734578	0.112910441	-1.924939607	0.054236914	0.187502901	NLRP1	protein_coding
ENSG00000091622.16	1246.019572	0.012309804	0.144424576	0.085233441	0.932075805	0.967902663	PITPNM3	protein_coding
ENSG00000091640.8	1483.038168	-0.077558502	0.049842246	-1.556079579	0.11968917	0.307040383	SPAG7	protein_coding
ENSG00000091651.9	634.9221289	-0.099082744	0.092888256	-1.06668753	0.28611296	0.515545173	ORC6	protein_coding
ENSG00000091656.19	693.691333	0.3173129	0.162913531	1.947738152	0.051446303	0.181199249	ZFHX4	protein_coding
ENSG00000091664.9	1.746574731	0.163854309	0.447261968	0.366349748	0.714104112	0.850557998	SLC17A6	protein_coding
ENSG00000091704.10	42.55245923	-2.947856604	0.311200896	-9.472519647	2.73173E-21	1.70058E-17	CPA1	protein_coding
ENSG00000091732.17	691.0561845	-0.144768917	0.047368243	-3.056244168	0.002241287	0.020767286	ZC3HC1	protein_coding
ENSG00000091831.24	135.0743858	-0.256548705	0.122714305	-2.090617768	0.036562342	0.144975174	ESR1	protein_coding
ENSG00000091844.8	141.423902	0.190980157	0.101329998	1.884734629	0.059465675	0.198688765	RGS17	protein_coding
ENSG00000091879.14	1021.222534	0.114959074	0.098547818	1.166530882	0.24339987	0.469426355	ANGPT2	protein_coding
ENSG00000091947.10	1185.170659	-0.050170461	0.054543484	-0.91982502	0.357664206	0.585412503	TMEM101	protein_coding
ENSG00000091972.18	654.8834877	0.034811289	0.096813888	0.35956917	0.719169342	0.853092826	CD200	protein_coding
ENSG00000091986.16	10135.37979	0.216913645	0.16990594	1.276668991	0.201719133	0.420646295	CCDC80	protein_coding
ENSG00000092009.10	20.52104467	0.376504675	0.239533633	1.571823842	0.115991411	0.300974021	CMA1	protein_coding
ENSG00000092010.15	10159.64288	-0.094650723	0.069526702	-1.36135786	0.173400626	0.384445642	PSME1	protein_coding
ENSG00000092020.10	745.0940901	-0.142203371	0.040542354	-3.507526278	0.000452294	0.006311112	PPP2R3C	protein_coding
ENSG00000092036.18	475.4710607	-0.1255674	0.061326197	-2.047532809	0.040605799	0.155397701	HAUS4	protein_coding
ENSG00000092051.17	42.82875573	0.09435237	0.133320476	0.707711021	0.479124732	0.688547256	JPH4	protein_coding
ENSG00000092054.13	12.17264569	-0.531198721	0.200748307	-2.646093156	0.008142739	0.052382069	MYH7	protein_coding
ENSG00000092067.6	10.2722781	0.112602855	0.166638832	0.67572998	0.499212098	0.704690853	CEBPE	protein_coding
ENSG00000092068.20	3203.546031	-0.301736102	0.117729403	-2.56296298	0.010378311	0.061893482	SLC7A8	protein_coding
ENSG00000092094.11	1349.810345	-0.048532125	0.047993314	-1.011226792	0.311907896	0.541428074	OSGEP	protein_coding
ENSG00000092096.18	695.8405717	-0.002278564	0.139207705	-0.01636809	0.986940737	0.993805167	SLC22A17	protein_coding
ENSG00000092098.17	48.61456984	0.035140612	0.076032675	0.462177773	0.643953841	0.807642354	RNF31	protein_coding
ENSG00000092108.22	3002.779025	0.054737725	0.04661129	1.174344791	0.240256949	0.465650586	SCFD1	protein_coding
ENSG00000092140.16	1531.872101	0.025383428	0.058033452	0.437393032	0.661826336	0.81886446	G2E3	protein_coding
ENSG00000092148.13	8408.939169	0.032846833	0.05173012	0.634965324	0.525451031	0.724377716	HECTD1	protein_coding
ENSG00000092199.18	18087.85713	-0.057839422	0.039218946	-1.474782678	0.14027098	0.337861402	HNRNPC	protein_coding
ENSG00000092200.12	44.44089346	-0.147996452	0.102909087	-1.438128122	0.150397705	0.352643571	RPGRIP1	protein_coding
ENSG00000092201.10	7552.191071	-0.029205665	0.05125942	-0.569761911	0.568839193	0.75581015	SUPT16H	protein_coding
ENSG00000092203.15	3727.236672	-0.021745819	0.038062924	-0.571312361	0.567787924	0.755288451	TOX4	protein_coding
ENSG00000092208.17	400.4032483	-0.116582498	0.052848753	-2.205964979	0.027386452	0.120043635	GEMIN2	protein_coding
ENSG00000092295.12	183.3008312	0.093130333	0.19939594	0.467062335	0.640455281	0.805404331	TGM1	protein_coding
ENSG00000092330.18	2252.137753	-0.012905916	0.044912396	-0.287357558	0.773838555	0.885581931	TINF2	protein_coding
ENSG00000092345.14	34.71701431	-0.182817181	0.173238226	-1.055293539	0.291291095	0.521300605	DAZL	protein_coding
ENSG00000092377.15	5.623621151	-0.695020222	0.331933575	-2.093853334	0.036273047	0.144234929	TBL1Y	protein_coding
ENSG00000092421.17	2278.171783	-0.209739373	0.145809719	-1.438445765	0.150307615	0.352530215	SEMA6A	protein_coding

ENSG00000092439.16	3423.460739	-0.09085061	0.05518027	-1.646432853	0.099674674	0.274889138	TRPM7	protein_coding
ENSG00000092445.12	1270.080956	-0.031517854	0.089492917	-0.352182669	0.724701274	0.856391892	TYRO3	protein_coding
ENSG00000092470.12	866.5439123	-0.156668045	0.076536558	-2.046970096	0.040661021	0.155537685	WDR76	protein_coding
ENSG00000092529.25	149.2428522	-0.085749832	0.112960345	-0.759114467	0.447784086	0.6629663	CAPN3	protein_coding
ENSG00000092531.10	867.1261275	-0.06628435	0.042175978	-1.571613833	0.116040136	0.301010835	SNAP23	protein_coding
ENSG00000092607.15	79.88133181	0.736345995	0.151711897	4.85358109	1.21252E-06	6.06635E-05	TBX15	protein_coding
ENSG00000092621.13	2380.944941	0.249594112	0.150497553	1.658459601	0.097224735	0.270806155	PHGDH	protein_coding
ENSG00000092758.18	783.0765936	-0.250545213	0.220153021	-1.138050306	0.255099477	0.482052969	COL9A3	protein_coding
ENSG00000092820.18	31104.02344	0.178222688	0.083095597	2.144791009	0.03196956	0.132900895	EZR	protein_coding
ENSG00000092841.19	20462.36892	-0.098070524	0.068325462	-1.435343738	0.15118917	0.353566457	MYL6	protein_coding
ENSG00000092847.13	4140.433328	-0.055694696	0.052457757	-1.06170564	0.288369338	0.51819487	AGO1	protein_coding
ENSG00000092850.12	24.17499187	0.121955645	0.186791058	0.652898735	0.513821564	0.715921798	TEKT2	protein_coding
ENSG00000092853.14	1287.944788	-0.023479899	0.085617365	-0.274242249	0.783898462	0.890346998	CLSPN	protein_coding
ENSG00000092871.17	1535.465099	-0.034695038	0.055469285	-0.625481984	0.531654769	0.72921161	RFFL	protein_coding
ENSG00000092929.12	3758.880556	0.590509221	0.117362332	5.031505513	4.86643E-07	2.88916E-05	UNC13D	protein_coding
ENSG00000092931.11	1376.020421	-0.005847534	0.047002221	-0.124409735	0.900990866	0.952857152	MFSD11	protein_coding
ENSG00000092964.18	7051.269276	0.008500569	0.082431426	0.103122915	0.917865419	0.961139525	DPYSL2	protein_coding
ENSG00000092969.12	615.4626066	0.234164142	0.128390117	1.823848644	0.068174958	0.216330017	TGFB2	protein_coding
ENSG00000092978.11	1115.465021	0.122325366	0.043484526	2.813078026	0.004906974	0.03672183	GPATCH2	protein_coding
ENSG00000093000.19	4891.296227	-0.010638062	0.051833423	-0.205235577	0.837388074	0.920004227	NUP50	protein_coding
ENSG00000093009.11	890.0143575	-0.041264144	0.093294478	-0.442299958	0.658272161	0.816640908	CDC45	protein_coding
ENSG00000093010.15	5228.070567	-0.156215535	0.06561826	-2.380671709	0.017281105	0.088107958	COMT	protein_coding
ENSG00000093072.18	4390.55268	-0.348583662	0.117735409	-2.960737695	0.003069032	0.026131146	ADA2	protein_coding
ENSG00000093100.13	12.33163202	-0.088993709	0.125819348	-0.707313383	0.47937175	0.688697836	AC016026.1	lncRNA
ENSG00000093134.15	133.1214621	1.029874095	0.201940101	5.099898887	3.39835E-07	2.15874E-05	VNN3	protein_coding
ENSG00000093144.19	2926.224124	0.003311193	0.053641672	0.061727997	0.950779444	0.977034284	ECHDC1	protein_coding
ENSG00000093167.18	2546.087418	-0.064622354	0.043014185	-1.502349837	0.133006784	0.327323945	LRRFIP2	protein_coding
ENSG00000093183.14	1726.831956	-0.059037157	0.04570716	-1.29163914	0.196482143	0.413707294	SEC22C	protein_coding
ENSG00000093217.11	869.2103293	-0.092229025	0.074031075	-1.245815025	0.212832312	0.433493511	XYLB	protein_coding
ENSG00000094631.21	2654.075039	0.048887541	0.053162695	0.919583562	0.35779042	0.585461797	HDAC6	protein_coding
ENSG00000094661.3	8.687215742	-0.296345791	0.189166534	-1.566586781	0.11721131	0.302912895	OR1I1	protein_coding
ENSG00000094755.17	6759.614059	0.427832074	0.251247655	1.70283012	0.088599864	0.255859264	GABRP	protein_coding
ENSG00000094796.5	3.284645735	0.683449481	0.434327384	1.573581374	0.115584262	0.300382597	KRT31	protein_coding
ENSG00000094804.12	2399.745844	-0.214248348	0.124624843	-1.719146379	0.085587722	0.250462204	CDC6	protein_coding
ENSG00000094841.14	624.9852782	-0.191734377	0.057371104	-3.342002596	0.000831763	0.009977668	UPRT	protein_coding
ENSG00000094880.11	2049.763186	-5.95923E-05	0.044928791	-0.001326372	0.998941709	0.999515128	CDC23	protein_coding
ENSG00000094914.14	2600.70517	-0.104645916	0.049268581	-2.12398885	0.033671076	0.137334751	AAAS	protein_coding
ENSG00000094916.16	8292.65599	0.155449763	0.071557652	2.172370931	0.029827696	0.127095091	CBX5	protein_coding
ENSG00000094963.14	823.4655927	0.341441508	0.187002464	1.825866356	0.067870395	0.215787845	FMO2	protein_coding
ENSG00000094975.14	3241.198831	0.122973061	0.062033226	1.98237411	0.047437385	0.172066139	SUCO	protein_coding
ENSG00000095002.15	2078.327714	-0.078766424	0.064161172	-1.227633806	0.219584465	0.441427884	MSH2	protein_coding
ENSG00000095015.6	2725.883811	-0.212225767	0.065965427	-3.217227189	0.001294361	0.013958064	MAP3K1	protein_coding

ENSG00000095059.16	76.04317409	0.034344902	0.083188301	0.412857354	0.679711127	0.830358098	DHPS	protein_coding
ENSG00000095066.12	2182.25849	0.029760833	0.059157524	0.503077722	0.614909629	0.788602281	HOOK2	protein_coding
ENSG00000095110.8	23.73646689	1.575595377	0.271894402	5.794879785	6.83701E-09	8.73714E-07	NXPE1	protein_coding
ENSG00000095139.14	14486.34692	0.100055583	0.046586669	2.147729933	0.031735216	0.132367066	ARCN1	protein_coding
ENSG00000095203.14	2796.962057	-0.11561907	0.107066063	-1.079885328	0.280193248	0.509003717	EPB41L4B	protein_coding
ENSG00000095209.12	1418.878239	-0.189748222	0.08354895	-2.271102424	0.023140778	0.107357149	TMEM38B	protein_coding
ENSG00000095261.14	3454.333805	0.065272015	0.050321671	1.297095524	0.194598322	0.411467107	PSMD5	protein_coding
ENSG00000095303.17	1838.131844	0.003802439	0.115936146	0.032797701	0.973835912	0.987868326	PTGS1	protein_coding
ENSG00000095319.14	7078.081051	0.07173369	0.05884602	1.219006656	0.222841666	0.445390566	NUP188	protein_coding
ENSG00000095321.17	4004.649134	-0.011255598	0.100374644	-0.11213587	0.910715677	0.957795512	CRAT	protein_coding
ENSG00000095370.20	1059.571385	-0.137103715	0.089866765	-1.525633139	0.127101254	0.318327043	SH2D3C	protein_coding
ENSG00000095380.11	2838.744463	-0.121361939	0.074853028	-1.621336389	0.104945515	0.283557211	NANS	protein_coding
ENSG00000095383.20	1515.351657	0.487646552	0.085048267	5.733762365	9.82269E-09	1.17918E-06	TBC1D2	protein_coding
ENSG00000095397.16	637.2504049	0.234997077	0.112706362	2.085038258	0.037065828	0.146310556	WHRN	protein_coding
ENSG00000095464.10	25.05350817	-0.246369501	0.115844444	-2.12672695	0.033442777	0.136710684	PDE6C	protein_coding
ENSG00000095485.18	1076.002669	-0.093699452	0.041624939	-2.251041187	0.024382929	0.111076099	CWF19L1	protein_coding
ENSG00000095539.15	3227.333873	-0.070047408	0.106092909	-0.660245899	0.509096042	0.712606907	SEMA4G	protein_coding
ENSG00000095564.14	2931.183529	-0.037818698	0.063551195	-0.595090275	0.551783129	0.743784132	BTAF1	protein_coding
ENSG00000095574.12	1350.819694	-0.037187399	0.049068712	-0.75786377	0.448532541	0.663603078	IKZF5	protein_coding
ENSG00000095585.17	1465.2884	-0.464451401	0.10769845	-4.312517038	1.61406E-05	0.00047079	BLNK	protein_coding
ENSG00000095587.9	163.1617435	0.373120783	0.14273996	2.613989696	0.008949174	0.055854789	TLL2	protein_coding
ENSG00000095596.12	5.232758364	0.472119591	0.221300746	2.133384542	0.032893194	0.135301748	CYP26A1	protein_coding
ENSG00000095627.9	34.32620901	0.527914669	0.210854614	2.503690376	0.012290554	0.069683251	TDRD1	protein_coding
ENSG00000095637.22	10380.59933	-0.450282979	0.18146116	-2.481428963	0.013085679	0.072929356	SORBS1	protein_coding
ENSG00000095713.14	92.88297426	0.549628697	0.218610258	2.514194453	0.011930463	0.068285431	CRTAC1	protein_coding
ENSG00000095739.11	1611.920706	-0.552617914	0.183526134	-3.011112913	0.00260292	0.02312958	BAMBI	protein_coding
ENSG00000095752.7	401.9102535	0.780651837	0.197427462	3.954119803	7.68169E-05	0.001616345	IL11	protein_coding
ENSG00000095777.17	45.13287806	0.008972562	0.221116295	0.040578474	0.967631945	0.984830373	MYO3A	protein_coding
ENSG00000095787.23	9323.3731	-0.068288275	0.037585165	-1.8168944	0.069233284	0.218294886	WAC	protein_coding
ENSG00000095794.19	1089.4943	-0.063260787	0.062503705	-1.012112584	0.311484226	0.541057511	CREM	protein_coding
ENSG00000095906.17	2291.10886	-0.061188442	0.058478114	-1.046347731	0.295400516	0.525263561	NUBP2	protein_coding
ENSG00000095917.14	168.007734	0.056938696	0.229496913	0.248102231	0.80405531	0.902607441	TPSD1	protein_coding
ENSG00000095932.7	4942.082058	-0.554565583	0.177734063	-3.120198645	0.001807291	0.017768293	SMIM24	protein_coding
ENSG00000095951.17	2520.073082	0.012032161	0.054247841	0.22179981	0.824469727	0.91339275	HIVEP1	protein_coding
ENSG00000095970.17	629.2960127	0.15521937	0.136593579	1.1363592	0.255806264	0.482890948	TREM2	protein_coding
ENSG00000095981.11	5.095954948	-0.976872932	0.348306647	-2.804634767	0.005037362	0.037402137	KCNK16	protein_coding
ENSG00000096006.12	174.2340117	1.092109316	0.346594383	3.15097235	0.001627279	0.016487313	CRISP3	protein_coding
ENSG00000096060.15	4025.854505	-0.213510232	0.105897913	-2.016189228	0.043780192	0.163285433	FKBP5	protein_coding
ENSG00000096063.16	4095.112757	0.005680349	0.057500496	0.098787826	0.921306734	0.962915133	SRPK1	protein_coding
ENSG00000096070.19	4464.259531	0.133035819	0.056491605	2.354966159	0.018524393	0.09222409	BRPF3	protein_coding
ENSG00000096080.12	1208.095987	0.049316293	0.076679532	0.643148069	0.520128026	0.720362995	MRPS18A	protein_coding
ENSG00000096088.16	82835.1023	-0.405776404	0.306946119	-1.321979262	0.186175057	0.400995969	PGC	protein_coding

ENSG00000096092.6	1352.30558	0.005736436	0.071784171	0.079912269	0.936307032	0.969825353	TMEM14A	protein_coding
ENSG00000096093.16	926.6530167	0.112608669	0.078443741	1.435534159	0.151134942	0.353478282	EFHC1	protein_coding
ENSG00000096264.14	4.420942872	-0.268370048	0.24779389	-1.083037391	0.278791827	0.507630739	NCR2	protein_coding
ENSG00000096384.20	88712.83894	0.178492705	0.067631109	2.639210078	0.008309947	0.053097148	HSP90AB1	protein_coding
ENSG00000096395.11	11.11828252	-1.114271441	0.372467196	-2.991596183	0.002775231	0.024216308	MLN	protein_coding
ENSG00000096401.8	3933.235054	0.261929666	0.057561831	4.550405378	5.35427E-06	0.000197731	CDC5L	protein_coding
ENSG00000096433.11	18491.16202	0.242947305	0.082807453	2.933882109	0.003347513	0.027874286	ITPR3	protein_coding
ENSG00000096654.15	535.4997519	-0.00589858	0.053628847	-0.109988931	0.912418153	0.958582856	ZNF184	protein_coding
ENSG00000096696.14	30641.18947	0.254497859	0.103548654	2.457761165	0.013980612	0.076322637	DSP	protein_coding
ENSG00000096717.12	1693.534725	-0.02257627	0.050155136	-0.450128784	0.652617583	0.813167789	SIRT1	protein_coding
ENSG00000096746.17	5433.642106	-0.071132311	0.037147558	-1.914858318	0.055510575	0.190141826	HNRNPH3	protein_coding
ENSG00000096872.17	655.6872717	-0.232779149	0.08045497	-2.893284895	0.003812353	0.030640154	IFT74	protein_coding
ENSG00000096968.14	2711.856579	-0.330866125	0.10525034	-3.143610984	0.001668771	0.016794467	JAK2	protein_coding
ENSG00000096996.16	422.2747009	-0.336738506	0.112000522	-3.006579788	0.002642047	0.023386652	IL12RB1	protein_coding
ENSG00000097007.19	8369.245291	0.058548787	0.06217888	0.941618551	0.346387969	0.575820116	ABL1	protein_coding
ENSG00000097021.20	3954.814332	-0.227996317	0.077076017	-2.958070804	0.00309571	0.026306895	ACOT7	protein_coding
ENSG00000097033.14	6731.906217	0.05413826	0.042976112	1.259729106	0.207767102	0.427654316	SH3GLB1	protein_coding
ENSG00000097046.13	735.1134896	-0.185604092	0.086074371	-2.156322371	0.031058502	0.130577552	CDC7	protein_coding
ENSG00000097096.9	524.9428648	-0.29397388	0.104244965	-2.820029528	0.004801923	0.036196748	SYDE2	protein_coding
ENSG00000099139.14	2728.388311	0.055957266	0.114702765	0.487845832	0.625659045	0.795806894	PCSK5	protein_coding
ENSG00000099194.6	18742.88432	0.132545453	0.132727692	0.998626966	0.317975432	0.547359882	SCD	protein_coding
ENSG00000099203.7	283.4203841	0.17155621	0.06680449	2.56803414	0.010227708	0.061370013	TMED1	protein_coding
ENSG00000099204.20	13918.88251	0.06905063	0.09028123	0.764839272	0.444367259	0.660330505	ABLIM1	protein_coding
ENSG00000099219.14	4026.785547	0.147755321	0.09080391	1.627191178	0.103696501	0.281544076	ERMP1	protein_coding
ENSG00000099246.17	4891.583784	-0.078194084	0.049406166	-1.582678642	0.113494729	0.297108251	RAB18	protein_coding
ENSG00000099250.18	4931.047383	0.178036372	0.082743065	2.151677274	0.031422781	0.131579367	NRP1	protein_coding
ENSG00000099251.14	92.53456242	-0.137665537	0.105837653	-1.300723639	0.193353068	0.409861076	HSD17B7P2	transcribed_unprocessed_pseudogene
ENSG00000099256.19	827.8442864	-0.328813096	0.096030576	-3.424045847	0.000616962	0.007955097	PRTFDC1	protein_coding
ENSG00000099260.11	697.4993008	0.076178954	0.118238181	0.644283883	0.519391365	0.71990095	PALMD	protein_coding
ENSG00000099282.10	6002.681408	-0.065619047	0.078442156	-0.83652784	0.402858021	0.624700875	TSPAN15	protein_coding
ENSG00000099284.15	871.1302473	0.106697905	0.134580429	0.792818882	0.427883354	0.646245361	MACROH2A2	protein_coding
ENSG00000099290.17	2530.783976	-0.186924293	0.048649392	-3.842273998	0.0001219	0.002304564	WASHC2A	protein_coding
ENSG00000099308.10	1972.173717	-0.094585776	0.079319869	-1.192460051	0.233080906	0.45754163	MAST3	protein_coding
ENSG00000099326.9	1323.34021	0.114587296	0.062444551	1.835024751	0.066502022	0.212866066	MZF1	protein_coding
ENSG00000099330.9	847.7632065	-0.119306613	0.069461388	-1.717596155	0.085870302	0.250852729	OCEL1	protein_coding
ENSG00000099331.13	7688.031303	0.020326769	0.057364783	0.354342299	0.723082371	0.855433359	MYO9B	protein_coding
ENSG00000099337.5	3334.982326	0.340178292	0.088794994	3.831052604	0.000127596	0.002382288	KCNK6	protein_coding
ENSG00000099338.23	156.2815923	0.038451068	0.094498162	0.40689752	0.684083257	0.833201859	CATSPERG	protein_coding
ENSG00000099341.11	9379.683673	0.017145372	0.061707305	0.277849956	0.781127541	0.889146485	PSMD8	protein_coding
ENSG00000099364.18	2102.499146	0.085153752	0.072481642	1.174832004	0.240061934	0.465490745	FBXL19	protein_coding
ENSG00000099365.11	99.59068729	-0.10907456	0.107229456	-1.017207069	0.309054934	0.538384444	STX1B	protein_coding
ENSG00000099377.14	1633.570729	-0.34658304	0.098428244	-3.521174655	0.00042964	0.006074757	HSD3B7	protein_coding

ENSG00000099381.18	3938.498107	0.063018742	0.048315017	1.30433031	0.192120985	0.408313313	SETD1A	protein_coding
ENSG00000099385.12	1490.457775	-0.052739219	0.071356875	-0.739090932	0.459851783	0.673058178	BCL7C	protein_coding
ENSG00000099399.6	35.33046346	2.319901414	0.393594649	5.894138596	3.76641E-09	5.54915E-07	MAGEB2	protein_coding
ENSG00000099617.4	878.889856	0.192725509	0.171334893	1.124846816	0.260653952	0.487824998	EFNA2	protein_coding
ENSG00000099622.14	5579.617851	-0.162222587	0.054368771	-2.983745689	0.002847433	0.024648907	CIRBP	protein_coding
ENSG00000099624.8	7528.54365	-0.195568251	0.079335479	-2.46507935	0.013698291	0.075312028	ATP5F1D	protein_coding
ENSG00000099625.13	237.947631	0.416416647	0.130471936	3.191618524	0.001414781	0.014933273	CBARP	protein_coding
ENSG00000099715.14	6.999855798	-0.443497669	0.275619495	-1.609093976	0.107595794	0.287509316	PCDH11Y	protein_coding
ENSG00000099721.14	0.482982549	0.533059835	0.502762638	1.060261434	0.289025679		AMELY	protein_coding
ENSG00000099725.14	337.8716906	-0.335321079	0.24517179	-1.367698458	0.171406483	0.381889682	PRKY	protein_coding
ENSG00000099769.6	412.3951163	1.348690775	0.25093543	5.374652664	7.67304E-08	6.56912E-06	IGFALS	protein_coding
ENSG00000099783.12	9370.713335	-0.019709093	0.037398796	-0.526998071	0.598194928	0.777300226	HNRNPM	protein_coding
ENSG00000099785.10	1506.981184	0.007647101	0.069806998	0.109546333	0.912769174	0.958775064	MARCHF2	protein_coding
ENSG00000099795.7	3832.523281	-0.182762441	0.070364264	-2.597375847	0.009393907	0.057820382	NDUFB7	protein_coding
ENSG00000099797.15	8878.85239	-0.039247307	0.070515184	-0.55657951	0.577814756	0.762343202	TECR	protein_coding
ENSG00000099800.8	2940.431868	-0.106103635	0.068024318	-1.559789757	0.118809573	0.305447626	TIMM13	protein_coding
ENSG00000099804.9	2569.274419	0.005774312	0.062807663	0.091936421	0.926748554	0.965338156	CDC34	protein_coding
ENSG00000099810.21	1852.60986	-0.004626074	0.090848686	-0.050920651	0.959388749	0.981161313	MTAP	protein_coding
ENSG00000099812.9	15596.01176	0.162270005	0.107794754	1.505360869	0.132231324	0.325918801	MISP	protein_coding
ENSG00000099814.16	9235.312086	0.231041924	0.070254632	3.288636176	0.001006741	0.011551163	CEP170B	protein_coding
ENSG00000099817.12	6938.114497	-0.105787963	0.05612233	-1.884953162	0.059436162	0.198688765	POLR2E	protein_coding
ENSG00000099821.14	4539.42767	0.000537553	0.057141445	0.009407408	0.992494085	0.996518853	POLRMT	protein_coding
ENSG00000099822.3	143.7353437	0.555311666	0.144568489	3.841166698	0.000122451	0.002309689	HCN2	protein_coding
ENSG00000099834.19	6536.66557	-0.216798726	0.154697909	-1.401432814	0.161084685	0.367472261	CDHR5	protein_coding
ENSG00000099840.14	69.65076127	-0.144051244	0.097196822	-1.482057147	0.13832509	0.33492457	IZUMO4	protein_coding
ENSG00000099849.15	3211.236114	0.062407021	0.086553505	0.721022459	0.470895697	0.682045504	RASSF7	protein_coding
ENSG00000099860.9	2083.250479	0.074705313	0.103688157	0.720480677	0.471229093	0.682353631	GADD45B	protein_coding
ENSG00000099864.18	1211.762202	0.319509421	0.14268873	2.239205718	0.025142534	0.113557371	PALM	protein_coding
ENSG00000099866.15	227.8447925	-0.718361403	0.185771129	-3.866916271	0.00011022	0.002133748	MADCAM1	protein_coding
ENSG00000099869.8	7.382561454	1.634814934	0.251329181	6.504676161	7.78609E-11	2.2322E-08	IGF2-AS	lncRNA
ENSG00000099875.15	10805.77562	-0.095864067	0.069502794	-1.379283648	0.167807316	0.376887314	MKNK2	protein_coding
ENSG00000099889.14	795.7102205	0.117896788	0.0871778	1.352371679	0.176256445	0.38834277	ARVCF	protein_coding
ENSG00000099899.15	1099.926255	-0.061014808	0.055086918	-1.107609746	0.268030392	0.495964349	TRMT2A	protein_coding
ENSG00000099901.17	6925.442601	-0.171531796	0.067667473	-2.534922449	0.011247222	0.065576693	RANBP1	protein_coding
ENSG00000099904.16	3061.210751	0.246731141	0.061146391	4.035089206	5.45816E-05	0.001231745	ZDHHC8	protein_coding
ENSG00000099910.17	942.1633384	0.040499576	0.062462483	0.64838242	0.516737638	0.718067414	KLHL22	protein_coding
ENSG00000099917.17	7409.836993	0.179348263	0.057932512	3.095813669	0.001962737	0.018855858	MED15	protein_coding
ENSG00000099937.11	55.09770729	-0.660197862	0.176452494	-3.741504841	0.000182922	0.003188471	SERPIND1	protein_coding
ENSG00000099940.12	2322.14339	-0.06057723	0.042707752	-1.41841299	0.156070225	0.360799	SNAP29	protein_coding
ENSG00000099942.13	5647.252757	0.050071547	0.048498105	1.03244338	0.301864459	0.531531741	CRKL	protein_coding
ENSG00000099949.21	644.6527999	0.054697335	0.062086577	0.880984862	0.378326014	0.604071255	LZTR1	protein_coding
ENSG00000099953.10	8427.698574	0.931439113	0.168903541	5.51462157	3.49531E-08	3.38478E-06	MMP11	protein_coding

ENSG00000099954.18	90.60853598	0.531209484	0.193585966	2.74404956	0.006068638	0.042654814	CECR2	protein_coding
ENSG00000099956.20	4583.051494	-0.095871931	0.047912043	-2.000998605	0.04539254	0.167165615	SMARCB1	protein_coding
ENSG00000099957.16	26.12256979	-0.073361021	0.170780359	-0.429563568	0.667513143	0.822444589	P2RX6	protein_coding
ENSG00000099958.15	412.2305867	-0.689614481	0.162427169	-4.245684295	2.17927E-05	0.00059856	DERL3	protein_coding
ENSG00000099960.13	179.4633815	0.701804514	0.205506411	3.415000591	0.000637819	0.008162769	SLC7A4	protein_coding
ENSG00000099968.18	3095.32875	0.036311478	0.042737437	0.849640992	0.395524714	0.618879011	BCL2L13	protein_coding
ENSG00000099974.8	78.7817759	-0.064596419	0.079261742	-0.814976024	0.415086034	0.635479257	DDTL	protein_coding
ENSG00000099977.14	0.968615541	0.194027465	0.184089945	1.053981874	0.291891216		DDT	protein_coding
ENSG00000099984.11	5.517184946	0.310859957	0.197817004	1.571452157	0.116077659	0.301072326	GSTT2	polymorphic_pseudogene
ENSG00000099985.4	280.7111231	0.380398338	0.172145935	2.209743363	0.027122978	0.119303324	OSM	protein_coding
ENSG00000099991.18	8282.920767	-0.212920758	0.065845522	-3.233640659	0.001222231	0.013378843	CABIN1	protein_coding
ENSG00000099992.16	602.9520025	0.022923965	0.06119732	0.374590994	0.707964672	0.847033508	TBC1D10A	protein_coding
ENSG00000099994.11	1206.651817	0.461934873	0.124547061	3.708918289	0.000208147	0.003496686	SUSD2	protein_coding
ENSG00000099995.19	8162.458942	0.005482891	0.039921656	0.137341274	0.890761048	0.947917932	SF3A1	protein_coding
ENSG00000099998.18	2888.765011	0.350594275	0.115972811	3.023073022	0.002502219	0.022454526	GGT5	protein_coding
ENSG00000099999.15	407.1706526	0.131784939	0.05802997	2.270980648	0.023148149	0.10736281	RNF215	protein_coding
ENSG00000100003.18	675.9367612	0.034691624	0.093052555	0.372817529	0.709284255	0.847922748	SEC14L2	protein_coding
ENSG00000100012.12	6.611138231	-0.199736934	0.202178217	-0.987925098	0.323189333	0.552971124	SEC14L3	protein_coding
ENSG00000100014.20	1447.825032	-0.053669907	0.049006045	-1.095169129	0.273442548	0.501992919	SPECC1L	protein_coding
ENSG00000100023.19	3001.105228	-0.021963592	0.044403261	-0.494639167	0.620854849	0.792564793	PPIL2	protein_coding
ENSG00000100024.15	43.02551165	0.000717238	0.165433739	0.004335501	0.996540781	0.99835631	UPB1	protein_coding
ENSG00000100027.17	133.1155478	-0.048364	0.111700603	-0.43297886	0.665030128	0.820754422	YPEL1	protein_coding
ENSG00000100028.12	3073.348241	-0.087161625	0.048317133	-1.803948607	0.071239364	0.222187072	SNRPD3	protein_coding
ENSG00000100029.18	3894.612274	0.00021986	0.05564404	0.003951195	0.996847411	0.998504884	PES1	protein_coding
ENSG00000100030.15	10197.07438	0.01942198	0.044066697	0.440740554	0.659400834	0.817447856	MAPK1	protein_coding
ENSG00000100031.19	60.47274068	-0.231083404	0.155505966	-1.486009898	0.137276503	0.333650354	GGT1	protein_coding
ENSG00000100033.16	416.0886949	0.41072631	0.206586304	1.988158473	0.046794166	0.170411949	PRODH	protein_coding
ENSG00000100034.14	1630.713714	0.042068112	0.059915236	0.702127114	0.482599895	0.690922399	PPM1F	protein_coding
ENSG00000100036.13	650.3742484	0.088391401	0.100215224	0.882015704	0.377768315	0.603600992	SLC35E4	protein_coding
ENSG00000100038.20	16.04114339	0.033876266	0.097901338	0.346024547	0.729324267	0.859059454	TOP3B	protein_coding
ENSG00000100053.10	20.25135099	0.076336703	0.108628779	0.702730013	0.48222402	0.690768749	CRYBB3	protein_coding
ENSG00000100055.21	924.889453	-0.252871874	0.106433593	-2.375865235	0.017507856	0.088827551	CYTH4	protein_coding
ENSG00000100056.12	1023.957402	-0.003034875	0.051235342	-0.059234016	0.952765716	0.977784174	ESS2	protein_coding
ENSG00000100058.12	593.2748134	-0.011045964	0.05158976	-0.21411155	0.830460069	0.916570636	CRYBB2P1	transcribed_unprocessed_pseudogene
ENSG00000100060.18	788.4964544	-0.361325057	0.103716214	-3.483785633	0.000494375	0.006742846	MFNG	protein_coding
ENSG00000100065.15	3445.843448	0.067336274	0.081983597	0.821338369	0.411453558	0.632380282	CARD10	protein_coding
ENSG00000100068.13	322.3933807	0.010482571	0.073261403	0.143084493	0.886223452	0.945569741	LRP5L	protein_coding
ENSG00000100075.10	5885.503654	-0.030681623	0.070188564	-0.43713137	0.662016079	0.818926777	SLC25A1	protein_coding
ENSG00000100077.15	1123.228269	-0.134917331	0.092884667	-1.45252532	0.146355594	0.346842383	GRK3	protein_coding
ENSG00000100078.4	70.16635345	0.435137536	0.251482298	1.730290918	0.083578311	0.246904757	PLA2G3	protein_coding
ENSG00000100079.7	1812.042555	-1.181954685	0.19741882	-5.987041573	2.13692E-09	3.4217E-07	LGALS2	protein_coding
ENSG00000100083.19	3457.302296	-0.002872734	0.042439399	-0.067690273	0.946032193	0.974726911	GGA1	protein_coding

ENSG00000100084.14	774.4396482	-0.189790252	0.057071629	-3.325474615	0.000882681	0.010424013	HIRA	protein_coding
ENSG00000100092.24	690.5292956	-0.341604533	0.09564681	-3.571520388	0.000354915	0.005274941	SH3BP1	protein_coding
ENSG00000100095.19	43.64425668	-0.348292942	0.190631794	-1.827045396	0.067692944	0.215356194	SEZ6L	protein_coding
ENSG00000100097.12	11731.62774	0.139292264	0.106777541	1.304509005	0.192060091	0.408277016	LGALS1	protein_coding
ENSG00000100099.21	2352.228735	-0.095557517	0.052623959	-1.815855702	0.06939251	0.218585905	HPS4	protein_coding
ENSG00000100100.13	2093.786261	-0.078493452	0.084981878	-0.923649298	0.355668938	0.583590079	PIK3IP1	protein_coding
ENSG00000100101.15	0						Z83844.1	protein_coding
ENSG00000100104.13	767.1452405	-0.001221299	0.043067524	-0.028357771	0.977376805	0.989893297	SRRD	protein_coding
ENSG00000100105.18	2922.928264	-0.139261493	0.062976252	-2.211333449	0.027012755	0.118998667	PATZ1	protein_coding
ENSG00000100106.22	6274.031655	0.063680924	0.057422509	1.108988883	0.267434978	0.495413221	TRIOBP	protein_coding
ENSG00000100109.17	1502.067776	0.041296227	0.047821795	0.863544064	0.387838404	0.612231382	TFIP11	protein_coding
ENSG00000100116.17	720.6505569	-0.17011855	0.091147032	-1.866418981	0.06198277	0.204051005	GCAT	protein_coding
ENSG00000100121.13	3.549426239	0.065173461	0.196388981	0.331859052	0.739995692	0.865643175	GGTLC2	protein_coding
ENSG00000100122.7	20.48213403	-0.049368651	0.122068259	-0.404434792	0.68589302	0.834197579	CRYBB1	protein_coding
ENSG00000100124.15	1206.333458	-0.069336131	0.054129228	-1.280936994	0.200215798	0.418642691	ANKRD54	protein_coding
ENSG00000100129.18	7361.069723	-0.018424325	0.078821902	-0.233746268	0.815181966	0.908781681	EIF3L	protein_coding
ENSG00000100138.15	5685.611429	-0.126582717	0.052637876	-2.404783894	0.016182032	0.084435295	SNU13	protein_coding
ENSG00000100139.13	3629.589632	-0.057260729	0.080091199	-0.714944084	0.474643596	0.68511242	MICALL1	protein_coding
ENSG00000100142.16	1974.93535	-0.203279148	0.063633923	-3.194509116	0.001400689	0.014847436	POLR2F	protein_coding
ENSG00000100146.18	128.0123406	-0.156434368	0.201065956	-0.778025141	0.436554192	0.654006613	SOX10	protein_coding
ENSG00000100147.14	1157.103566	-0.091410027	0.070075143	-1.304457239	0.19207773	0.408277016	CCDC134	protein_coding
ENSG00000100150.20	862.7322923	-0.082650975	0.045792493	-1.804902275	0.071089976	0.221933198	DEPDC5	protein_coding
ENSG00000100151.16	1259.068212	-0.00876925	0.055301827	-0.15857071	0.874007107	0.939114606	PICK1	protein_coding
ENSG00000100154.14	1715.191917	0.064656715	0.105771468	0.61128692	0.541009649	0.736276623	TTC28	protein_coding
ENSG00000100156.10	76.06223137	0.169967892	0.121291643	1.401315769	0.161119668	0.36750127	SLC16A8	protein_coding
ENSG00000100162.15	804.4832817	-0.269452042	0.094876527	-2.840028522	0.00451095	0.034625772	CENPM	protein_coding
ENSG00000100167.20	322.9759738	0.233026073	0.166440706	1.400054583	0.161496974	0.368034391	SEPTIN3	protein_coding
ENSG00000100170.10	3251.55619	-0.345613162	0.168028775	-2.056868897	0.039698839	0.153358899	SLC5A1	protein_coding
ENSG00000100181.22	164.2779731	0.156043407	0.154760687	1.008288408	0.313316026	0.542640446	TPTEP1	transcribed_unprocessed_pseudogene
ENSG00000100191.6	33.98116348	-0.130452087	0.130411144	-1.000313959	0.317158594	0.54656372	SLC5A4	protein_coding
ENSG00000100196.11	4343.116025	0.331503137	0.094892381	3.493464193	0.000476797	0.006564733	KDELR3	protein_coding
ENSG00000100197.22	152.4290587	-0.221173753	0.131921433	-1.676556629	0.093629214	0.264471134	CYP2D6	protein_coding
ENSG00000100201.23	28950.53592	0.023090122	0.043514536	0.53063009	0.59567514	0.775202376	DDX17	protein_coding
ENSG00000100206.10	63.00845587	-0.249509599	0.101554253	-2.4569094	0.014013802	0.076440038	DMC1	protein_coding
ENSG00000100207.20	5459.034275	-0.054432573	0.059845278	-0.909555023	0.363057228	0.59035579	TCF20	protein_coding
ENSG00000100209.11	380.6710685	-0.141514684	0.053503552	-2.644958686	0.008170089	0.052516282	HSCB	protein_coding
ENSG00000100211.10	584.7370611	-0.110770538	0.056101462	-1.974467946	0.048328559	0.174131747	CBY1	protein_coding
ENSG00000100216.6	2904.521268	-0.075209039	0.058020125	-1.296257792	0.194886685	0.411861157	TOMM22	protein_coding
ENSG00000100218.12	45.83912568	-0.615141035	0.153191152	-4.015512819	5.93167E-05	0.001319003	RSPH14	protein_coding
ENSG00000100219.16	18000.95231	-0.1620786	0.091577817	-1.769845633	0.07675286	0.233826857	XBP1	protein_coding
ENSG00000100220.12	4203.653236	-0.050103881	0.046064369	-1.087692777	0.276730757	0.505575803	RTCB	protein_coding
ENSG00000100221.10	4546.34354	0.140196038	0.048751467	2.875729603	0.004030949	0.031955002	JOSD1	protein_coding

ENSG00000100225.18	5267.983797	-0.009344006	0.038504255	-0.242674634	0.808257459	0.904946823	FBXO7	protein_coding
ENSG00000100226.16	4971.957825	0.082951163	0.046940806	1.767144	0.077204115	0.234725719	GTPBP1	protein_coding
ENSG00000100227.18	4698.875868	0.008977573	0.042438625	0.211542494	0.832463977	0.917453796	POLDIP3	protein_coding
ENSG00000100228.12	596.1432938	0.29805547	0.11742814	2.538194585	0.011142601	0.065167243	RAB36	protein_coding
ENSG00000100234.12	17801.99377	0.225065836	0.120150669	1.87319669	0.061041229	0.201958367	TIMP3	protein_coding
ENSG00000100239.16	3467.12797	-0.122997905	0.047873258	-2.569240347	0.010192174	0.061227511	PPP6R2	protein_coding
ENSG00000100241.21	9201.381047	0.040471133	0.065455557	0.618299431	0.536377977	0.732811108	SBF1	protein_coding
ENSG00000100242.15	4135.382579	0.041975078	0.069106287	0.607398826	0.543586268	0.738284519	SUN2	protein_coding
ENSG00000100243.21	17522.75516	0.107469711	0.057413056	1.871868843	0.061224751	0.202365821	CYB5R3	protein_coding
ENSG00000100246.13	672.3295692	-0.078089756	0.067517063	-1.156592913	0.247438719	0.474057651	DNAL4	protein_coding
ENSG00000100249.4	6.929563856	-0.545384594	0.194289698	-2.807069034	0.004999453	0.037209419	C22orf31	protein_coding
ENSG00000100253.13	66.54367957	0.743393736	0.217812792	3.412993924	0.000642534	0.008203836	MIOX	protein_coding
ENSG00000100258.18	5571.780373	0.081852628	0.062732195	1.304794587	0.191962803	0.408216038	LMF2	protein_coding
ENSG00000100263.14	1169.661181	-0.097963273	0.065763154	-1.489637692	0.136319526	0.332254313	RHBDD3	protein_coding
ENSG00000100266.19	5466.610476	-0.073755513	0.054250559	-1.359534619	0.17397724	0.385057961	PACSIN2	protein_coding
ENSG00000100271.17	228.169476	-0.081974068	0.072735189	-1.127020759	0.259733704	0.486939237	TTLL1	protein_coding
ENSG00000100276.10	40.26834175	0.081337335	0.106170225	0.766103063	0.443614981	0.659797605	RASL10A	protein_coding
ENSG00000100280.16	9838.360458	-0.051953871	0.058229925	-0.892219429	0.37227533	0.598798246	AP1B1	protein_coding
ENSG00000100281.14	2270.51979	-0.000255376	0.041994894	-0.006081124	0.995147995	0.997712278	HMGXB4	protein_coding
ENSG00000100284.21	2725.146968	0.1327144	0.062400062	2.126831234	0.033434108	0.136688069	TOM1	protein_coding
ENSG00000100285.10	67.3990755	-0.329274388	0.137759939	-2.390204225	0.016839006	0.086685573	NEFH	protein_coding
ENSG00000100288.20	23.93624088	-0.074139005	0.092401337	-0.802358568	0.422345572	0.641631328	CHKB	protein_coding
ENSG00000100290.3	1154.421198	-0.101147892	0.114178479	-0.885875276	0.37568474	0.601549515	BIK	protein_coding
ENSG00000100292.18	2204.530194	-0.291240548	0.100966341	-2.884531087	0.003919971	0.031337471	HMOX1	protein_coding
ENSG00000100294.13	1122.866023	-0.127527987	0.062527363	-2.03955486	0.041394682	0.157377077	MCAT	protein_coding
ENSG00000100296.14	1870.719739	-0.039796877	0.042825506	-0.929279787	0.352744108	0.581179391	THOC5	protein_coding
ENSG00000100297.16	5095.391553	-0.146068653	0.07243374	-2.016583061	0.043739042	0.163172352	MCM5	protein_coding
ENSG00000100298.16	61.68119848	-0.561825729	0.134046235	-4.191283181	2.77381E-05	0.000717355	APOBEC3H	protein_coding
ENSG00000100299.18	2875.913547	-0.039774502	0.070623074	-0.563194151	0.573302685	0.758907015	ARSA	protein_coding
ENSG00000100300.18	7920.89066	0.024989908	0.090942387	0.27478834	0.783478857	0.890154816	TSPO	protein_coding
ENSG00000100302.7	515.1339208	0.02779254	0.123436992	0.225155681	0.821858166	0.912391394	RASD2	protein_coding
ENSG00000100304.13	6758.713537	0.174727727	0.079115379	2.208517858	0.027208194	0.119548928	TTLL12	protein_coding
ENSG00000100307.13	1473.196355	-0.238373824	0.100469373	-2.372601882	0.017663291	0.089387206	CBX7	protein_coding
ENSG00000100311.17	1812.891302	0.101440165	0.085124353	1.191670319	0.233390541	0.457901922	PDGFB	protein_coding
ENSG00000100312.11	11.93315497	0.155176074	0.142834972	1.086401122	0.277301565	0.506207372	ACR	protein_coding
ENSG00000100314.4	14.4870452	0.300739844	0.151958278	1.979094843	0.047805329	0.172938139	CABP7	protein_coding
ENSG00000100316.16	83185.36868	-0.144088657	0.061473973	-2.343896949	0.019083443	0.093987252	RPL3	protein_coding
ENSG00000100319.13	350.4803961	-0.156693383	0.071374956	-2.195355226	0.028138126	0.122079981	ZMAT5	protein_coding
ENSG00000100320.23	5387.278876	0.063207185	0.058729234	1.076247391	0.28181663	0.510770287	RBFOX2	protein_coding
ENSG00000100321.15	759.9965996	-0.335510362	0.154772046	-2.167771063	0.03017611	0.128103685	SYNGR1	protein_coding
ENSG00000100324.14	1669.949977	-0.067984197	0.047526256	-1.430455555	0.152586313	0.355755586	TAB1	protein_coding
ENSG00000100325.15	3856.295978	-0.023895232	0.052006072	-0.459470036	0.645896664	0.808900541	ASCC2	protein_coding

ENSG00000100330.16	367.2498054	0.031123251	0.050056402	0.621763645	0.534097295	0.731185254	MTMR3	protein_coding
ENSG00000100335.15	2904.565682	0.00921367	0.045928504	0.20060898	0.841004335	0.921927096	MIEF1	protein_coding
ENSG00000100336.17	943.2596956	0.182624137	0.117867925	1.549396376	0.121286464	0.309552551	APOL4	protein_coding
ENSG00000100341.12	2.752365756	0.13818021	0.318477281	0.433877763	0.664377208	0.820529577	PNPLA5	protein_coding
ENSG00000100342.21	11600.96718	0.397405266	0.121235135	3.27797108	0.001045561	0.011868303	APOL1	protein_coding
ENSG00000100344.11	108.5247241	0.486572474	0.189933208	2.561808334	0.010412877	0.06202767	PNPLA3	protein_coding
ENSG00000100345.22	84130.74273	0.117955534	0.053449739	2.206849581	0.02732457	0.119859829	MYH9	protein_coding
ENSG00000100346.18	87.04371372	0.380081764	0.184189309	2.063538676	0.039061477	0.151682502	CACNA1I	protein_coding
ENSG00000100347.15	2456.362802	-0.127773844	0.049563252	-2.57799557	0.009937527	0.060162215	SAMM50	protein_coding
ENSG00000100348.10	3883.925991	-0.192777384	0.056460139	-3.414397959	0.000639231	0.008178445	TXN2	protein_coding
ENSG00000100350.15	3960.163229	-0.267077284	0.107958188	-2.473895585	0.013364875	0.073983888	FOXRED2	protein_coding
ENSG00000100351.17	286.8175947	-0.585779565	0.11763045	-4.97982933	6.36404E-07	3.61572E-05	GRAP2	protein_coding
ENSG00000100353.18	12264.77833	-0.027332483	0.050266245	-0.543754226	0.586610612	0.768757687	EIF3D	protein_coding
ENSG00000100354.21	5119.375423	-0.011482871	0.053496273	-0.214648049	0.830041729	0.916390345	TNRC6B	protein_coding
ENSG00000100359.21	49.16971881	0.115346626	0.091857404	1.255713974	0.209219691	0.429305763	SGSM3	protein_coding
ENSG00000100360.15	712.3759442	-0.017801775	0.062864265	-0.283177963	0.77704042	0.886950006	IFT27	protein_coding
ENSG00000100362.13	10.36468956	-0.376852306	0.177544091	-2.122584335	0.033788699	0.137634149	PVALB	protein_coding
ENSG00000100364.19	5342.911099	0.097489357	0.057457591	1.6967185	0.089749893	0.257796527	KIAA0930	protein_coding
ENSG00000100365.16	595.5403266	-0.281385568	0.113167049	-2.486462012	0.012902034	0.072170712	NCF4	protein_coding
ENSG00000100368.14	1836.898495	-0.481912502	0.13093191	-3.680634481	0.000232654	0.003820037	CSF2RB	protein_coding
ENSG00000100372.15	1235.346664	-0.093187537	0.048067849	-1.938666687	0.052541938	0.183492548	SLC25A17	protein_coding
ENSG00000100373.10	124.8308343	-1.190527224	0.253058948	-4.704545053	2.54433E-06	0.000109668	UPK3A	protein_coding
ENSG00000100376.12	1483.463307	0.047305728	0.070061619	0.675201745	0.499547598	0.704947723	FAM118A	protein_coding
ENSG00000100379.17	895.9331353	-0.114005246	0.087644225	-1.300773054	0.193336148	0.409861076	KCTD17	protein_coding
ENSG00000100380.14	12855.7329	-0.156908745	0.046759413	-3.355661159	0.000791755	0.009607996	ST13	protein_coding
ENSG00000100385.14	2071.748897	-0.304192238	0.11997857	-2.535388093	0.011232281	0.065542195	IL2RB	protein_coding
ENSG00000100387.9	2611.144537	-0.139326758	0.053537206	-2.602428629	0.009256608	0.057248823	RBX1	protein_coding
ENSG00000100393.14	10556.5461	0.043727261	0.039268751	1.113538369	0.265477279	0.493371216	EP300	protein_coding
ENSG00000100395.15	1802.297134	-0.062253419	0.042826709	-1.453612022	0.146053902	0.346504294	L3MBTL2	protein_coding
ENSG00000100399.16	137.9451952	0.180113574	0.123429472	1.459242843	0.144498281	0.344256825	CHADL	protein_coding
ENSG00000100401.20	6727.101265	0.050348406	0.055031194	0.914906662	0.360240632	0.588113121	RANGAP1	protein_coding
ENSG00000100403.12	7396.876933	-0.046263321	0.053006873	-0.87277966	0.382783188	0.607891509	ZC3H7B	protein_coding
ENSG00000100410.8	1388.444383	-0.148989426	0.056125081	-2.654596213	0.007940339	0.051489948	PHF5A	protein_coding
ENSG00000100412.17	6347.292495	-0.138141488	0.06166735	-2.240107426	0.02508395	0.113413911	ACO2	protein_coding
ENSG00000100413.17	1076.495013	-0.000746921	0.052723644	-0.014166713	0.988696977	0.994792153	POLR3H	protein_coding
ENSG00000100416.15	1354.017816	-0.037533065	0.057941053	-0.647780177	0.517127138	0.718379588	TRMU	protein_coding
ENSG00000100417.12	739.5112503	-0.19081706	0.075526628	-2.526487214	0.011520958	0.066686711	PMM1	protein_coding
ENSG00000100418.8	3407.3075	-0.039518824	0.053316298	-0.741214699	0.458563272	0.671871821	DESI1	protein_coding
ENSG00000100422.14	3686.566713	0.009541313	0.075235842	0.126818716	0.899083882	0.951977512	CERK	protein_coding
ENSG00000100425.18	3082.062727	0.019832687	0.048196261	0.41149846	0.68070707	0.830924452	BRD1	protein_coding
ENSG00000100426.7	2718.253224	0.12957275	0.05547432	2.335724896	0.019505584	0.095343158	ZBED4	protein_coding
ENSG00000100427.16	40.77181773	0.298690241	0.152041455	1.964531583	0.049468477	0.176681242	MLC1	protein_coding

ENSG00000100429.18	72.19433582	-0.223355266	0.089151767	-2.505337512	0.012233459	0.069413732	HDAC10	protein_coding
ENSG00000100433.16	217.7968321	-0.364846489	0.172169918	-2.11910706	0.03408142	0.138348025	KCNK10	protein_coding
ENSG00000100439.10	2979.251007	0.025719441	0.067744604	0.37965298	0.704203028	0.84481487	ABHD4	protein_coding
ENSG00000100441.10	3442.222616	-0.018873797	0.052148484	-0.361924184	0.717408687	0.852280973	KHNYN	protein_coding
ENSG00000100442.11	1777.17043	-0.085426719	0.060267321	-1.417463348	0.156347503	0.361185016	FKBP3	protein_coding
ENSG00000100445.17	162.4681277	-0.055042088	0.05500431	-1.000686823	0.31697824	0.546398764	SDR39U1	protein_coding
ENSG00000100448.4	66.85845619	0.287675761	0.223483187	1.287236706	0.198011797	0.415443432	CTSG	protein_coding
ENSG00000100450.13	260.54357	-0.629803936	0.157391046	-4.001523288	6.2936E-05	0.001380948	GZMH	protein_coding
ENSG00000100453.13	808.2037301	-0.454698787	0.16263605	-2.795805648	0.005177052	0.038121056	GZMB	protein_coding
ENSG00000100461.18	3833.418816	0.011313302	0.034477818	0.328132782	0.742811268	0.867489056	RBM23	protein_coding
ENSG00000100462.16	1524.971135	0.10324805	0.065650955	1.57268161	0.11579256	0.300726646	PRMT5	protein_coding
ENSG00000100473.18	319.4404043	0.463075542	0.175507939	2.638487729	0.008327671	0.053171419	COCH	protein_coding
ENSG00000100478.15	494.6899596	-0.144485665	0.067215615	-2.149584812	0.031588071	0.131954271	AP4S1	protein_coding
ENSG00000100479.13	550.7548204	-0.083100801	0.076799992	-1.082041796	0.279233957	0.507909577	POLE2	protein_coding
ENSG00000100483.14	447.253599	0.027779233	0.054311164	0.511482929	0.609012939	0.784738767	VCPKMT	protein_coding
ENSG00000100485.12	2761.511132	0.099153405	0.051124716	1.939441683	0.052447579	0.183280525	SOS2	protein_coding
ENSG00000100490.9	381.3440725	0.010392542	0.073771895	0.140873996	0.887969486	0.946482555	CDKL1	protein_coding
ENSG00000100503.26	2352.039753	0.1384632	0.085424276	1.620888202	0.105041618	0.283728914	NIN	protein_coding
ENSG00000100504.17	1300.022401	0.844904054	0.125236841	6.746449756	1.51506E-11	5.59508E-09	PYGL	protein_coding
ENSG00000100505.13	101.7977748	0.310516984	0.148353098	2.093094029	0.036340762	0.144384236	TRIM9	protein_coding
ENSG00000100519.12	3671.502766	0.026980756	0.047550908	0.567407797	0.570437154	0.756920768	PSMC6	protein_coding
ENSG00000100522.10	2252.475487	0.076356032	0.075664492	1.009139556	0.31290771	0.542408803	GNPNAT1	protein_coding
ENSG00000100523.16	1356.652279	0.052687891	0.057558586	0.91537848	0.359992972	0.587976698	DDHD1	protein_coding
ENSG00000100526.20	1090.677571	-0.05322816	0.09722922	-0.547450236	0.58406945	0.766972863	CDKN3	protein_coding
ENSG00000100528.12	6155.113187	-0.005246009	0.05254461	-0.099839138	0.920472035	0.962550631	CNIH1	protein_coding
ENSG00000100532.12	461.6333199	0.035589788	0.052242354	0.68124395	0.495717143	0.702134367	CGRRF1	protein_coding
ENSG00000100554.12	2676.252815	0.019306185	0.052238912	0.369574798	0.711699332	0.849244023	ATP6V1D	protein_coding
ENSG00000100557.10	232.0879611	-0.434151018	0.257023454	-1.689149419	0.091190799	0.260259359	CCDC198	protein_coding
ENSG00000100558.9	2521.882489	0.045526231	0.110339417	0.412601706	0.679898451	0.830460015	PLEK2	protein_coding
ENSG00000100564.9	714.0427656	-0.041854852	0.052931946	-0.790729502	0.429101857	0.647314419	PIGH	protein_coding
ENSG00000100565.15	10.70917727	-0.425491124	0.155829241	-2.730496027	0.00632391	0.043867718	LRRC74A	protein_coding
ENSG00000100567.13	4819.630309	-0.138595685	0.054533734	-2.541466996	0.011038837	0.064711868	PSMA3	protein_coding
ENSG00000100568.11	2282.807835	0.023417213	0.039493953	0.59293161	0.553226922	0.744693714	VTI1B	protein_coding
ENSG00000100575.14	1156.906016	-0.168465591	0.0603855	-2.789835152	0.005273488	0.03861583	TIMM9	protein_coding
ENSG00000100577.19	880.0535518	-0.18777585	0.078362171	-2.396256341	0.016563502	0.085763751	GSTZ1	protein_coding
ENSG00000100578.18	1290.122854	-0.036008452	0.050444922	-0.713817168	0.475340243	0.685537486	KIAA0586	protein_coding
ENSG00000100580.8	2121.68088	-0.090726983	0.051827757	-1.750548126	0.080023777	0.240094849	TMED8	protein_coding
ENSG00000100583.4	65.71441282	0.264715464	0.097536404	2.71401705	0.006647274	0.045381213	SAMD15	protein_coding
ENSG00000100591.8	5276.819746	0.015762858	0.056223043	0.280362956	0.779199055	0.888374224	AHSA1	protein_coding
ENSG00000100592.16	2296.708854	-0.146728876	0.061629043	-2.380839768	0.017273224	0.088085055	DAAM1	protein_coding
ENSG00000100593.18	33.83317958	0.179821654	0.259352421	0.69334866	0.488090773	0.695543885	ISM2	protein_coding
ENSG00000100596.7	6737.679529	0.019947891	0.059199336	0.336961403	0.736145997	0.863152072	SPTLC2	protein_coding

ENSG00000100599.16	2840.22059	0.129940352	0.08597235	1.511420256	0.130681412	0.323760815	RIN3	protein_coding
ENSG00000100600.15	9985.899761	-0.034889897	0.07633028	-0.457091166	0.64760552	0.809771778	LGMN	protein_coding
ENSG00000100601.10	751.9502718	-0.102233826	0.041004663	-2.493224415	0.012658881	0.071166408	ALKBH1	protein_coding
ENSG00000100603.14	2562.846518	0.000581102	0.035010447	0.016597963	0.986757349	0.993775242	SNW1	protein_coding
ENSG00000100604.13	1418.535261	-0.903052598	0.285295755	-3.165320837	0.00154912	0.015880379	CHGA	protein_coding
ENSG00000100605.17	8419.788914	0.000431262	0.067436038	0.00639513	0.994897459	0.99764472	ITPK1	protein_coding
ENSG00000100612.14	2288.353296	0.032193971	0.076314518	0.421859061	0.673127894	0.82616235	DHRS7	protein_coding
ENSG00000100614.18	3504.073902	0.030151068	0.04666647	0.646097043	0.51821651	0.71911254	PPM1A	protein_coding
ENSG00000100625.9	442.8735287	0.739632833	0.127194936	5.814955032	6.06502E-09	7.98475E-07	SIX4	protein_coding
ENSG00000100626.17	324.1505162	-0.051153594	0.175001056	-0.292304486	0.770053829	0.883604174	GALNT16	protein_coding
ENSG00000100628.12	831.3137433	-0.562050152	0.184090657	-3.053116111	0.002264783	0.020940472	ASB2	protein_coding
ENSG00000100629.17	367.7654476	-0.076890262	0.060852477	-1.263551893	0.20639091	0.426166435	CEP128	protein_coding
ENSG00000100632.11	3189.396254	-0.109697626	0.062677365	-1.750195248	0.080084628	0.240158185	ERH	protein_coding
ENSG00000100644.17	12817.08634	0.226446824	0.070773879	3.199581931	0.001376271	0.014663507	HIF1A	protein_coding
ENSG00000100647.8	4627.439649	0.093259566	0.059158333	1.576440058	0.114924423	0.299215861	SUSD6	protein_coding
ENSG00000100650.16	6630.586552	-0.068861223	0.042812877	-1.608423183	0.107742528	0.287669908	SRSF5	protein_coding
ENSG00000100652.5	13.001549	-0.146308887	0.147169216	-0.994154151	0.320147826	0.549839664	SLC10A1	protein_coding
ENSG00000100664.11	13215.61545	-0.106645807	0.047151442	-2.261771896	0.0237115	0.108983867	EIF5	protein_coding
ENSG00000100665.12	960.1315975	-0.168190465	0.225841716	-0.744727184	0.456436662	0.670378848	SERPINA4	protein_coding
ENSG00000100678.19	26.60374733	-0.168362794	0.160034775	-1.05203881	0.292781744	0.522677184	SLC8A3	protein_coding
ENSG00000100697.15	4590.542435	0.10766902	0.053746975	2.003257286	0.04514968	0.166637384	DICER1	protein_coding
ENSG00000100711.14	1117.25034	0.129115526	0.050636987	2.549826417	0.010777657	0.063647912	ZFYVE21	protein_coding
ENSG00000100714.17	4201.931406	-0.059300277	0.062753121	-0.944977329	0.34467045	0.574342812	MTHFD1	protein_coding
ENSG00000100721.11	133.0932707	-1.273982676	0.255772582	-4.980919635	6.32828E-07	3.60629E-05	TCL1A	protein_coding
ENSG00000100722.20	2874.015559	0.014753352	0.041980445	0.351433918	0.725262841	0.856707023	ZC3H14	protein_coding
ENSG00000100726.15	2214.562143	-0.047682684	0.061847746	-0.770968826	0.44072541	0.65722713	TELO2	protein_coding
ENSG00000100731.16	4032.110407	0.009063251	0.062157078	0.145812056	0.884069759	0.944428022	PCNX1	protein_coding
ENSG00000100739.11	98.32615379	0.408620782	0.119890192	3.408292012	0.000653709	0.008309096	BDKRB1	protein_coding
ENSG00000100744.15	3763.027105	0.082003527	0.091727684	0.893988852	0.371327856	0.597848	GSKIP	protein_coding
ENSG00000100749.8	946.0793786	-0.129710598	0.070591108	-1.83749202	0.066137293	0.21210329	VRK1	protein_coding
ENSG00000100764.14	1675.754591	-0.007246506	0.049372493	-0.146772131	0.883311883	0.943960371	PSMC1	protein_coding
ENSG00000100767.17	1051.791793	0.019595785	0.125541338	0.156090299	0.875961845	0.94008479	PAPLN	protein_coding
ENSG00000100784.12	1132.673127	-0.248719332	0.077134177	-3.224502297	0.001261918	0.013690659	RPS6KA5	protein_coding
ENSG00000100796.17	4542.141014	0.026007624	0.039924837	0.651414661	0.514778849	0.716510729	PPP4R3A	protein_coding
ENSG00000100802.15	478.3449582	-0.089931764	0.046084438	-1.951456257	0.051002795	0.180205127	C14orf93	protein_coding
ENSG00000100804.19	4817.303787	-0.03452776	0.056236626	-0.61397282	0.539233288	0.734966429	PSMB5	protein_coding
ENSG00000100811.14	8521.377748	0.029042113	0.03113591	0.932752983	0.350947522	0.579946731	YY1	protein_coding
ENSG00000100813.14	8210.001472	0.004606825	0.033521852	0.137427528	0.890692873	0.947901515	ACIN1	protein_coding
ENSG00000100814.18	1877.777019	-0.031178703	0.067369959	-0.462798309	0.643508943	0.807317957	CCNB1IP1	protein_coding
ENSG00000100815.12	3298.492167	0.036011838	0.046368899	0.776637774	0.437372509	0.654380242	TRIP11	protein_coding
ENSG00000100823.12	8193.539631	-0.097394159	0.059520982	-1.636299594	0.101776901	0.278407771	APEX1	protein_coding
ENSG00000100836.10	767.0702079	-0.096510586	0.057168592	-1.688174974	0.091377646	0.26051447	PABPN1	protein_coding

ENSG00000100842.13	359.9762248	0.364645067	0.135353815	2.694013966	0.007059721	0.047256751	EFS	protein_coding
ENSG00000100852.13	3707.250689	0.097349394	0.068882222	1.413273131	0.157575431	0.362644022	ARHGAP5	protein_coding
ENSG00000100865.15	1325.045165	-0.071013678	0.048463412	-1.465304975	0.14283773	0.341608021	CINP	protein_coding
ENSG00000100867.15	368.9141639	-0.026813836	0.229845691	-0.116660164	0.907129359	0.956031153	DHRS2	protein_coding
ENSG00000100883.13	3133.534046	0.015690968	0.046125633	0.340178911	0.733721799	0.861793343	SRP54	protein_coding
ENSG00000100884.9	50.32374087	-0.363386831	0.21009437	-1.729636212	0.083695292	0.247049362	CPNE6	protein_coding
ENSG00000100888.15	5755.720498	0.041775117	0.045176696	0.924704994	0.355119381	0.583088069	CHD8	protein_coding
ENSG00000100889.12	2500.377466	-0.205234442	0.084890126	-2.41764798	0.015621181	0.082342349	PCK2	protein_coding
ENSG00000100890.16	242.477401	-0.107977639	0.046128553	-2.340798296	0.01924256	0.09447195	PRORP	protein_coding
ENSG00000100897.18	3617.912685	-0.038475722	0.04693995	-0.819679654	0.412398751	0.6330947	DCAF11	protein_coding
ENSG00000100902.11	360.0147261	-0.03229952	0.061886817	-0.521912768	0.601731068	0.779848761	PSMA6	protein_coding
ENSG00000100906.11	7837.776008	0.024788542	0.067105735	0.369395286	0.711833111	0.849293755	NFKBIA	protein_coding
ENSG00000100908.14	383.0258469	-0.133550255	0.069116321	-1.932253538	0.05332822	0.185317691	EMC9	protein_coding
ENSG00000100911.16	7817.01212	-0.231977246	0.085334306	-2.718452364	0.00655881	0.044992428	PSME2	protein_coding
ENSG00000100916.14	423.7695476	0.024188612	0.054353629	0.44502294	0.656303176	0.815319427	BRMS1L	protein_coding
ENSG00000100918.13	1972.670824	-0.661442595	0.169130117	-3.910850463	9.19717E-05	0.001855876	REC8	protein_coding
ENSG00000100926.15	261.9508456	0.049330438	0.048840409	1.010033269	0.312479351	0.542009819	TM9SF1	protein_coding
ENSG00000100934.15	5103.392107	0.060723606	0.060609861	1.00187666	0.316403166	0.545601709	SEC23A	protein_coding
ENSG00000100938.18	2022.501344	-0.02656111	0.043607761	-0.609091338	0.542463899	0.737495689	GMPR2	protein_coding
ENSG00000100941.9	5123.750039	0.040514646	0.051812363	0.781949403	0.43424431	0.651979888	PNN	protein_coding
ENSG00000100949.14	1781.030425	0.042227997	0.052488862	0.804513484	0.421100485	0.640742566	RABGGTA	protein_coding
ENSG00000100968.14	929.5598214	0.348755615	0.110101445	3.167584347	0.001537111	0.01578873	NFATC4	protein_coding
ENSG00000100979.15	6798.551291	0.165107518	0.11891236	1.388480708	0.164990715	0.372798942	PLTP	protein_coding
ENSG00000100982.12	4365.929912	-0.004594924	0.052967039	-0.086750625	0.930869735	0.967241624	PCIF1	protein_coding
ENSG00000100983.12	3573.203882	-0.20999454	0.065862308	-3.188387218	0.001430688	0.015030158	GSS	protein_coding
ENSG00000100985.7	2657.865728	0.03000572	0.14941395	0.200822748	0.840837175	0.921927096	MMP9	protein_coding
ENSG00000100987.15	13.81326238	-1.098636733	0.244624433	-4.491116118	7.08509E-06	0.000247592	VSX1	protein_coding
ENSG00000100991.12	6435.20123	-0.00314384	0.04788766	-0.065650308	0.947656236	0.975449811	TRPC4AP	protein_coding
ENSG00000100994.12	20328.43097	0.186498942	0.097105998	1.920570775	0.054785842	0.188661918	PYGB	protein_coding
ENSG00000100997.20	5753.306879	-0.210945025	0.065136825	-3.23849108	0.001201638	0.013215767	ABHD12	protein_coding
ENSG00000101000.6	1473.197892	0.046632766	0.113441715	0.411072468	0.681019398	0.831013445	PROCR	protein_coding
ENSG00000101003.10	1367.715422	-0.188182596	0.091082721	-2.066062516	0.038822577	0.151145487	GINS1	protein_coding
ENSG00000101004.15	951.8939836	0.292098089	0.131913154	2.214321161	0.026806694	0.118378121	NINL	protein_coding
ENSG00000101017.14	1252.322875	-0.446934077	0.119550373	-3.73845823	0.000185152	0.003214484	CD40	protein_coding
ENSG00000101019.22	2175.036604	-0.144150664	0.062087789	-2.321723245	0.020247842	0.098070494	UQCC1	protein_coding
ENSG00000101040.19	7177.415449	-0.231885927	0.079488235	-2.917235841	0.003531486	0.02900161	ZMYND8	protein_coding
ENSG00000101049.17	922.1791768	-0.295878536	0.152497696	-1.940216429	0.052353393	0.183024773	SGK2	protein_coding
ENSG00000101052.13	1526.429247	-0.108051192	0.059805306	-1.806715817	0.0708066	0.221407041	IFT52	protein_coding
ENSG00000101057.16	6652.516401	-0.138427357	0.110520617	-1.252502574	0.210386791	0.430682094	MYBL2	protein_coding
ENSG00000101074.5	25.50814047	0.082925855	0.196265154	0.422519502	0.672645869	0.825928283	R3HDML	protein_coding
ENSG00000101076.17	9903.817994	-0.047395495	0.115721125	-0.40956649	0.682123983	0.831931621	HNF4A	protein_coding
ENSG00000101079.21	3745.995012	-0.142176742	0.052703787	-2.697657056	0.006982934	0.046995413	NDRG3	protein_coding

ENSG00000101082.14	279.1750953	-0.476637616	0.121476222	-3.923711232	8.71952E-05	0.0017839	SLA2	protein_coding
ENSG00000101084.18	730.3304883	-0.074383903	0.103974715	-0.715403767	0.474359585	0.684883959	RAB5IF	protein_coding
ENSG00000101096.20	3187.612739	-0.120278865	0.081948352	-1.467739887	0.142174892	0.340714653	NFATC2	protein_coding
ENSG00000101098.13	94.25229503	-0.574429588	0.284348477	-2.020160593	0.043366732	0.162358148	RIMS4	protein_coding
ENSG00000101104.13	1509.142135	-0.044938113	0.125596356	-0.357797901	0.720494561	0.853921658	PABPC1L	protein_coding
ENSG00000101109.12	5218.770267	-0.131004844	0.052044287	-2.517180082	0.011829835	0.067883686	STK4	protein_coding
ENSG00000101115.13	697.9810173	0.317912504	0.19299548	1.647253622	0.099505926	0.274684515	SALL4	protein_coding
ENSG00000101126.18	8513.001649	-0.039884695	0.05092751	-0.783166013	0.43352963	0.651355699	ADNP	protein_coding
ENSG00000101132.10	1506.617227	-0.178413228	0.084651637	-2.107616994	0.035064132	0.140919459	PFDN4	protein_coding
ENSG00000101134.12	143.4713658	0.300785655	0.124619039	2.413641256	0.015794004	0.082966237	DOK5	protein_coding
ENSG00000101138.12	2641.858928	-0.047513343	0.052593277	-0.903410964	0.366307823	0.59341956	CSTF1	protein_coding
ENSG00000101144.13	1333.249048	0.186232879	0.20793575	0.895627035	0.370451992	0.597011333	BMP7	protein_coding
ENSG00000101146.13	3260.978949	-0.036956211	0.066211716	-0.558152141	0.576740498	0.761437117	RAE1	protein_coding
ENSG00000101150.18	8094.485658	0.007418617	0.051390177	0.144358654	0.885217264	0.945074952	TPD52L2	protein_coding
ENSG00000101152.11	6824.562635	-0.049986909	0.062240998	-0.803118685	0.421906139	0.641210046	DNAJC5	protein_coding
ENSG00000101158.15	6773.807714	0.010104857	0.075131435	0.134495728	0.893010589	0.948975124	NELFCD	protein_coding
ENSG00000101160.14	12298.03791	0.026827426	0.080992932	0.331231692	0.740469483	0.865821581	CTSZ	protein_coding
ENSG00000101161.8	7494.270392	0.025901065	0.057346101	0.451662178	0.651512369	0.812446831	PRPF6	protein_coding
ENSG00000101162.4	32.70994277	0.263797452	0.117351298	2.247929568	0.024580681	0.111729671	TUBB1	protein_coding
ENSG00000101166.16	8701.947116	0.026684009	0.078230369	0.34109527	0.733031862	0.861215105	PRELID3B	protein_coding
ENSG00000101180.16	3.333265588	0.139229167	0.256993926	0.541760534	0.587983482	0.76990854	HRH3	protein_coding
ENSG00000101181.17	2092.615929	-0.095816571	0.068593148	-1.396882547	0.162448894	0.369373099	MTG2	protein_coding
ENSG00000101182.15	10651.80621	-0.073398151	0.064399199	-1.139737024	0.254395877	0.481196681	PSMA7	protein_coding
ENSG00000101187.16	2672.755528	0.084843649	0.128315948	0.661208917	0.508478344	0.712033606	SLCO4A1	protein_coding
ENSG00000101188.5	389.2688975	1.107773599	0.235229068	4.709339736	2.48521E-06	0.000107438	NTSR1	protein_coding
ENSG00000101189.7	1555.544977	-0.018764609	0.076399029	-0.245613192	0.805981676	0.903536312	MRGBP	protein_coding
ENSG00000101190.13	1122.107177	0.078871687	0.072805117	1.083326145	0.278663684	0.507579308	TCFL5	protein_coding
ENSG00000101191.17	7183.319323	0.110966848	0.051844491	2.140378756	0.03232417	0.133807386	DIDO1	protein_coding
ENSG00000101193.8	5269.132475	-0.005168417	0.058688623	-0.088065053	0.929824972	0.96676374	GID8	protein_coding
ENSG00000101194.18	3402.815716	0.282676316	0.133829821	2.112207236	0.034668678	0.139936735	SLC17A9	protein_coding
ENSG00000101197.13	73.79547735	0.690586748	0.19796699	3.488393435	0.000485932	0.00665059	BIRC7	protein_coding
ENSG00000101198.15	61.10934411	0.805760598	0.197437641	4.081089065	4.48252E-05	0.001056508	NKAIN4	protein_coding
ENSG00000101199.13	4967.220213	0.152171996	0.058286058	2.610778642	0.009033635	0.056234336	ARFGAP1	protein_coding
ENSG00000101200.5	2.088933228	0.593197601	0.342484464	1.732042363	0.08326602	0.246266008	AVP	protein_coding
ENSG00000101203.16	11.41727859	0.448286699	0.197861606	2.265657843	0.023472341	0.108284373	COL20A1	protein_coding
ENSG00000101204.18	8.98184311	0.596968917	0.226636029	2.634042432	0.008437494	0.053629039	CHRNA4	protein_coding
ENSG00000101210.13	1180.955605	0.95918767	0.270472111	3.546345933	0.000390613	0.005640075	EEF1A2	protein_coding
ENSG00000101213.7	2866.987069	0.182493129	0.119742395	1.52404776	0.127496781	0.318929895	PTK6	protein_coding
ENSG00000101216.11	1853.048111	0.019847922	0.056139892	0.353543991	0.723680655	0.855742753	GMEB2	protein_coding
ENSG00000101220.18	3091.964191	-0.302995036	0.080994979	-3.740911325	0.000183354	0.00319218	C20orf27	protein_coding
ENSG00000101222.12	25.37793277	0.13869034	0.159359774	0.870297042	0.384138095	0.609066576	SPEF1	protein_coding
ENSG00000101224.17	8421.653309	-0.13511406	0.09711863	-1.391227002	0.164156602	0.371569304	CDC25B	protein_coding

ENSG00000101230.6	383.8056054	0.392770791	0.17880972	2.19658524	0.028050081	0.121819653	ISM1	protein_coding
ENSG00000101236.17	3008.803254	0.111248542	0.072976654	1.52444017	0.127398792	0.318804247	RNF24	protein_coding
ENSG00000101246.20	2171.392424	0.007333367	0.067736072	0.108263836	0.913786409	0.959259687	ARFRP1	protein_coding
ENSG00000101247.18	700.5023962	-0.172320309	0.05489236	-3.139240312	0.001693865	0.016972533	NDUFAF5	protein_coding
ENSG00000101251.13	8.810944736	-0.131048394	0.221628756	-0.591296888	0.554321514	0.745458233	SEL1L2	protein_coding
ENSG00000101255.11	2139.683448	0.098783484	0.147245727	0.670875048	0.502300133	0.707137459	TRIB3	protein_coding
ENSG00000101265.16	2073.87041	-0.265131503	0.120624879	-2.197983575	0.027950276	0.121517732	RASSF2	protein_coding
ENSG00000101266.19	9618.695713	-0.092980998	0.050862197	-1.828096372	0.067535089	0.215051267	CSNK2A1	protein_coding
ENSG00000101276.18	1930.869458	-0.121272614	0.097907137	-1.238649379	0.215475373	0.436611658	SLC52A3	protein_coding
ENSG00000101278.6	22.43448885	-0.258683538	0.084610733	-3.057337162	0.002233129	0.020709339	RPS10P5	processed_pseudogene
ENSG00000101280.8	17.38410196	0.133777006	0.155630356	0.859581701	0.390019666	0.614501662	ANGPT4	protein_coding
ENSG00000101282.9	120.4341451	1.145945522	0.265012783	4.32411413	1.53146E-05	0.000453681	RSPO4	protein_coding
ENSG00000101290.14	3681.456979	-0.120559318	0.05401575	-2.231928983	0.025619657	0.114822611	CDS2	protein_coding
ENSG00000101292.8	3.482290325	0.420641897	0.232780183	1.807034825	0.070756849	0.221315764	PROKR2	protein_coding
ENSG00000101294.18	12161.5686	-0.020869151	0.064788457	-0.322112179	0.747367712	0.870012363	HM13	protein_coding
ENSG00000101298.15	691.2916348	-0.136740176	0.148438725	-0.921189374	0.356951565	0.584703919	SNPH	protein_coding
ENSG00000101306.11	20.7502411	0.232078871	0.110037615	2.10908671	0.034937098	0.140577462	MYLK2	protein_coding
ENSG00000101307.16	301.3171905	-0.189652356	0.148248504	-1.279286811	0.200796075	0.41938701	SIRPB1	protein_coding
ENSG00000101310.17	6442.503559	-0.110825966	0.065598338	-1.689463019	0.091130733	0.260202068	SEC23B	protein_coding
ENSG00000101311.16	8064.079984	-0.28357566	0.110613497	-2.563662386	0.010357424	0.061853564	FERMT1	protein_coding
ENSG00000101323.5	3.366825857	-1.845152936	0.353784878	-5.215465809	1.83356E-07	1.33391E-05	HAO1	protein_coding
ENSG00000101327.9	2.449537655	-0.44002371	0.331375128	-1.327871867	0.18422044	0.39833339	PDYN	protein_coding
ENSG00000101331.17	143.3146453	-0.23563341	0.108275836	-2.17623265	0.029537869	0.126252232	CCM2L	protein_coding
ENSG00000101333.17	2146.380967	-0.427822165	0.136226577	-3.14051908	0.001686487	0.016929752	PLCB4	protein_coding
ENSG00000101335.10	35606.8402	-0.307115476	0.184487826	-1.664692365	0.095974147	0.268334749	MYL9	protein_coding
ENSG00000101336.17	1136.3138	0.01733387	0.108367612	0.159954342	0.872917041	0.938607117	HCK	protein_coding
ENSG00000101337.16	9321.171147	-0.045657816	0.053926605	-0.846665869	0.397181388	0.620401941	TM9SF4	protein_coding
ENSG00000101342.10	254.5628111	-0.154398222	0.13734707	-1.124146456	0.2609509	0.488150134	TLDC2	protein_coding
ENSG00000101343.15	2510.620217	-0.042615659	0.052252249	-0.815575604	0.414742907	0.635085097	CRNKL1	protein_coding
ENSG00000101346.14	7246.614281	-0.086479101	0.082667171	-1.046111784	0.295509425	0.525290601	POFUT1	protein_coding
ENSG00000101347.10	8692.486973	-0.362125408	0.080514239	-4.497656719	6.87065E-06	0.000242039	SAMHD1	protein_coding
ENSG00000101349.17	22.10727116	-0.502670173	0.260368327	-1.930611832	0.053531074	0.185701883	PAK5	protein_coding
ENSG00000101350.8	6585.940959	-0.060921956	0.060486573	-1.007198003	0.313839632	0.543115431	KIF3B	protein_coding
ENSG00000101353.15	93.78435619	-0.192585693	0.081511676	-2.362676141	0.018143517	0.090940884	MROH8	protein_coding
ENSG00000101361.17	7909.483124	-0.191665068	0.067396837	-2.843828839	0.004457499	0.034330933	NOP56	protein_coding
ENSG00000101363.12	3319.376508	-0.109481547	0.053841241	-2.033414268	0.042010687	0.159011527	MANBAL	protein_coding
ENSG00000101365.21	4779.820391	-0.194857891	0.058162895	-3.350209607	0.000807504	0.009752942	IDH3B	protein_coding
ENSG00000101367.9	8613.805112	-0.096478106	0.048787225	-1.977528054	0.047981973	0.173346909	MAPRE1	protein_coding
ENSG00000101384.12	8758.939801	0.020478827	0.08313368	0.246336106	0.805422062	0.903363893	JAG1	protein_coding
ENSG00000101391.21	1499.645552	-0.078483534	0.051560798	-1.522155148	0.127970211	0.319481974	CDK5RAP1	protein_coding
ENSG00000101400.6	1025.844152	-0.25780313	0.115525561	-2.231567879	0.025643537	0.114883341	SNTA1	protein_coding
ENSG00000101405.3	36.93007495	0.147351239	0.183844241	0.801500434	0.422841993	0.642072114	OXT	protein_coding

ENSG00000101407.13	2632.774911	-0.053692908	0.060426566	-0.888564614	0.374237116	0.600027623	TTI1	protein_coding
ENSG00000101412.13	1671.626395	-0.081850722	0.092098921	-0.888726177	0.374150259	0.600020087	E2F1	protein_coding
ENSG00000101413.12	3562.689303	-0.064222475	0.052621644	-1.220457412	0.222291525	0.444813231	RPRD1B	protein_coding
ENSG00000101417.12	1444.651878	-0.070682322	0.067420542	-1.04837962	0.294463739	0.524519554	PXMP4	protein_coding
ENSG00000101421.4	10143.00837	-0.111022757	0.056985651	-1.948258099	0.051384088	0.181076392	CHMP4B	protein_coding
ENSG00000101425.14	16.51836801	0.467469703	0.170737061	2.737950977	0.006182329	0.043174256	BPI	protein_coding
ENSG00000101435.5	1.473856737	0.167686728	0.413254657	0.405770934	0.684910917	0.83372058	CST9L	protein_coding
ENSG00000101438.4	1.391587028	0.020421735	0.275243815	0.074195073	0.940855166	0.972043625	SLC32A1	protein_coding
ENSG00000101439.9	39495.98585	-0.079849511	0.078663755	-1.015073733	0.310070681	0.539605614	CST3	protein_coding
ENSG00000101440.9	25.10089285	0.033886058	0.11308618	0.299648094	0.764445595	0.87997757	ASIP	protein_coding
ENSG00000101441.4	49.58206538	0.519679225	0.219419498	2.368427741	0.01786387	0.089972648	CST4	protein_coding
ENSG00000101442.10	844.764786	-0.163435986	0.061440879	-2.660052845	0.007812839	0.050928959	ACTR5	protein_coding
ENSG00000101443.18	3331.545164	1.374569983	0.221451282	6.207098795	5.39717E-10	1.06906E-07	WFDC2	protein_coding
ENSG00000101444.13	12067.50009	-0.23559481	0.070881577	-3.323780586	0.00088806	0.010476179	AHCY	protein_coding
ENSG00000101445.10	1363.314302	-0.517512306	0.128262651	-4.034785675	5.46522E-05	0.0012327	PPP1R16B	protein_coding
ENSG00000101446.8	0.712109964	-0.217583076	0.455059084	-0.478142474	0.632548803		SPINT3	protein_coding
ENSG00000101447.15	3645.167952	-0.193866642	0.114948049	-1.686558781	0.091688223	0.261058328	FAM83D	protein_coding
ENSG00000101448.14	2.631263007	0.04903039	0.292510842	0.167619052	0.866882982	0.935493418	EPPIN	protein_coding
ENSG00000101452.15	1218.49312	-0.130954792	0.05586447	-2.344151713	0.019070412	0.093950574	DHX35	protein_coding
ENSG00000101457.13	3053.468184	0.097639333	0.068403895	1.427394356	0.153466257	0.357090937	DNTTIP1	protein_coding
ENSG00000101460.13	1437.782646	-0.011048783	0.095540565	-0.11564494	0.907933945	0.956371124	MAP1LC3A	protein_coding
ENSG00000101463.6	307.7074843	0.309484262	0.14041363	2.204089885	0.027518023	0.120421059	SYNDIG1	protein_coding
ENSG00000101464.11	2944.105477	-0.206142467	0.064560867	-3.192994072	0.001408058	0.014889337	PIGU	protein_coding
ENSG00000101470.10	129.0611103	0.178417376	0.19278502	0.925473235	0.3547198	0.582885683	TNNC2	protein_coding
ENSG00000101473.17	1372.462563	-0.128002548	0.064609508	-1.981172004	0.047571988	0.172408891	ACOT8	protein_coding
ENSG00000101474.12	5674.370292	-0.08384048	0.06115862	-1.370869383	0.170415677	0.380421288	APMAP	protein_coding
ENSG00000101489.20	75.72500452	-0.412531503	0.204182007	-2.020410659	0.043340809	0.162311999	CELF4	protein_coding
ENSG00000101493.11	1336.892066	0.148135655	0.083276085	1.778849899	0.075264374	0.230792739	ZNF516	protein_coding
ENSG00000101542.10	7.275007439	-0.14893611	0.165034312	-0.902455423	0.366814991	0.593851353	CDH20	protein_coding
ENSG00000101544.9	2162.41752	0.09685639	0.055477518	1.745867378	0.080834	0.241598299	ADNP2	protein_coding
ENSG00000101546.13	428.0428499	-0.124796386	0.056669878	-2.202164365	0.0276537	0.120669151	RBFA	protein_coding
ENSG00000101557.15	5425.447939	-0.024328215	0.05059127	-0.480877739	0.630603395	0.799321749	USP14	protein_coding
ENSG00000101558.14	10023.22323	-0.010188553	0.046472791	-0.219236943	0.826465479	0.914501198	VAPA	protein_coding
ENSG00000101574.15	805.7194465	-0.018538466	0.051516081	-0.359857846	0.718953442	0.853050793	METTL4	protein_coding
ENSG00000101577.10	5989.18894	0.089070638	0.084216814	1.057634851	0.290221947	0.520034654	LPIN2	protein_coding
ENSG00000101596.16	5511.532076	-0.039453618	0.060548732	-0.651601068	0.514658558	0.716480608	SMCHD1	protein_coding
ENSG00000101605.13	460.313103	-0.249088801	0.160139961	-1.555444368	0.119840274	0.307292091	MYOM1	protein_coding
ENSG00000101608.13	15297.46244	-0.009004344	0.064122296	-0.140424536	0.888324573	0.946675794	MYL12A	protein_coding
ENSG00000101624.11	598.0919843	0.02081283	0.064532618	0.322516439	0.747061486	0.869795297	CEP76	protein_coding
ENSG00000101638.14	19.51593178	0.008047659	0.168807986	0.047673449	0.961976495	0.982355347	ST8SIA5	protein_coding
ENSG00000101639.18	2193.339399	-0.03761291	0.064274265	-0.585193937	0.558417321	0.748506334	CEP192	protein_coding
ENSG00000101654.18	2808.061892	0.080836829	0.054002704	1.496903352	0.134418408	0.329448294	RNMT	protein_coding

ENSG00000101665.9	1602.426731	0.03638527	0.083962474	0.433351568	0.66475938	0.820687414	SMAD7	protein_coding
ENSG00000101670.12	2172.408757	0.049741478	0.126957297	0.391796917	0.695208273	0.83926147	LIPG	protein_coding
ENSG00000101680.15	178.5631845	-0.093141261	0.161872852	-0.575397668	0.565022381	0.753541861	LAMA1	protein_coding
ENSG00000101695.9	914.8282907	-0.104168816	0.093871757	-1.109692828	0.267131415	0.495172533	RNF125	protein_coding
ENSG00000101745.17	4403.741473	-0.166763662	0.056081837	-2.973577029	0.002943505	0.025292202	ANKRD12	protein_coding
ENSG00000101746.15	27.41481234	-0.671007878	0.231484325	-2.898718423	0.003746912	0.030252924	NOL4	protein_coding
ENSG00000101751.10	707.0365637	-0.263561308	0.065002264	-4.054648115	5.02098E-05	0.001154005	POLI	protein_coding
ENSG00000101752.12	4197.735956	0.213232472	0.069056068	3.08781657	0.002016329	0.019251876	MIB1	protein_coding
ENSG00000101773.19	2472.252946	-0.140985359	0.075127849	-1.876605832	0.060572138	0.200940319	RBBP8	protein_coding
ENSG00000101782.15	6079.788086	0.173658862	0.076717347	2.26361922	0.023597545	0.108682981	RIOK3	protein_coding
ENSG00000101811.14	1508.352605	-0.043494553	0.064074293	-0.678814406	0.497255465	0.703375901	CSTF2	protein_coding
ENSG00000101812.14	1.381628236	1.508553561	0.453650095	3.325368113	0.000883019	0.01042517	H2BW2	protein_coding
ENSG00000101825.8	18351.9001	0.679209315	0.118193587	5.746583462	9.10647E-09	1.10948E-06	MXRA5	protein_coding
ENSG00000101842.14	7863.135597	0.079638455	0.261463124	0.304587713	0.760680179	0.877515822	VSIG1	protein_coding
ENSG00000101843.19	2358.007939	0.00046766	0.047858733	0.009771672	0.992203458	0.996444867	PSMD10	protein_coding
ENSG00000101844.18	909.4955323	-0.006301663	0.061978955	-0.10167423	0.919015258	0.961746298	ATG4A	protein_coding
ENSG00000101846.8	2667.472362	0.052756378	0.112687293	0.468166162	0.639665769	0.804951509	STS	protein_coding
ENSG00000101849.17	2509.114484	0.202627416	0.091837578	2.206367165	0.027358302	0.119938907	TBL1X	protein_coding
ENSG00000101850.13	140.9470739	0.268032302	0.211027756	1.27012819	0.204038972	0.423405744	GPR143	protein_coding
ENSG00000101856.10	7843.96093	-0.004200518	0.053743722	-0.078158307	0.937702127	0.970438302	PGRMC1	protein_coding
ENSG00000101868.13	1838.977965	-0.10975512	0.062627449	-1.752508224	0.079686457	0.239600662	POLA1	protein_coding
ENSG00000101871.16	2409.606664	-0.13468325	0.086183593	-1.562748146	0.118111845	0.304355737	MID1	protein_coding
ENSG00000101882.10	776.1774745	-0.108740975	0.049386971	-2.201815013	0.027678378	0.1207258	NKAP	protein_coding
ENSG00000101883.5	4.431049117	-0.344376585	0.174626195	-1.972078614	0.048600633	0.174784994	RHOXF1	protein_coding
ENSG00000101888.12	1224.50314	-0.071458667	0.076671354	-0.932012588	0.35133002	0.580228465	NXT2	protein_coding
ENSG00000101890.5	2.614352596	0.104243305	0.255884156	0.407384757	0.683725421	0.832941534	GUCY2F	protein_coding
ENSG00000101892.12	1.278475001	-0.121866385	0.248493535	-0.490420747	0.623836199	0.794695574	ATP1B4	protein_coding
ENSG00000101898.6	0						MCTS2P	protein_coding
ENSG00000101901.12	2037.231109	-0.127143962	0.048347124	-2.6298144	0.00854315	0.054079725	ALG13	protein_coding
ENSG00000101911.13	3771.79233	-0.303120979	0.071556926	-4.236081611	2.27454E-05	0.000615638	PRPS2	protein_coding
ENSG00000101916.12	295.1149031	-0.27422219	0.155961096	-1.758273032	0.078701064	0.237783836	TLR8	protein_coding
ENSG00000101928.13	821.4805187	0.063058927	0.070126439	0.899217594	0.36853677	0.595439645	MOSPD1	protein_coding
ENSG00000101935.10	1809.383761	0.077353032	0.071117989	1.087671814	0.276740015	0.505575803	AMMECR1	protein_coding
ENSG00000101938.15	849.4890877	0.238449208	0.234026794	1.018897042	0.308251848	0.537694772	CHRDL1	protein_coding
ENSG00000101940.18	3522.715853	0.040743479	0.053240146	0.765277363	0.444106401	0.660113928	WDR13	protein_coding
ENSG00000101945.17	789.2400088	-0.05843473	0.074467136	-0.784704944	0.432626584	0.650694393	SUV39H1	protein_coding
ENSG00000101951.17	3.44060069	0.16360212	0.377529206	0.433349573	0.664760829	0.820687414	PAGE4	protein_coding
ENSG00000101955.15	688.2660335	0.191489832	0.157131445	1.218660162	0.222973204	0.445575288	SRPX	protein_coding
ENSG00000101958.14	10.84188405	-0.235805872	0.232083316	-1.016039737	0.309610464	0.539007439	GLRA2	protein_coding
ENSG00000101966.13	5363.852911	-0.006957162	0.056110069	-0.123991336	0.901322135	0.953022461	XIAP	protein_coding
ENSG00000101972.19	8025.635333	-0.02279685	0.049294215	-0.46246501	0.643747889	0.807455004	STAG2	protein_coding
ENSG00000101974.14	1530.280465	-0.060372707	0.068557158	-0.880618577	0.3785243	0.604232881	ATP11C	protein_coding

ENSG00000101977.21	9.938575533	-0.089541383	0.151369623	-0.5915413	0.554157791	0.745394144	MCF2		protein_coding
ENSG00000101981.12	0.812212211	0.084054459	0.452498829	0.185756192	0.85263596		F9		protein_coding
ENSG00000101986.12	3157.545122	0.096830437	0.092030697	1.052153678	0.292729048	0.522643612	ABCD1		protein_coding
ENSG00000101997.13	1134.066616	-0.039632267	0.05523468	-0.717525052	0.473050184	0.683877115	CCDC22		protein_coding
ENSG00000102001.13	72.6430637	0.222153418	0.131525786	1.689048399	0.091210155	0.260275355	CACNA1F		protein_coding
ENSG00000102003.11	224.6575089	-0.436238548	0.145047713	-3.007552063	0.00263361	0.023345164	SYP		protein_coding
ENSG00000102007.11	11277.13328	-0.021529202	0.087418891	-0.246276315	0.805468343	0.903363893	PLP2		protein_coding
ENSG00000102010.15	98.89019647	0.034199036	0.154906892	0.220771561	0.825270308	0.913781996	BMX		protein_coding
ENSG00000102021.11	1.033020242	0.301643	0.468524803	0.643814367	0.519695816	0.720056919	LUZP4		protein_coding
ENSG00000102024.18	7576.872664	0.212201809	0.067842701	3.127850226	0.001760899	0.017463516	PLS3		protein_coding
ENSG00000102030.16	1169.594375	-0.140791795	0.071146251	-1.978906727	0.047826509	0.172986037	NAA10		protein_coding
ENSG00000102032.13	615.392056	0.003258317	0.11816671	0.027573897	0.978002001	0.990221656	RENBP		protein_coding
ENSG00000102034.17	5333.116372	0.113425392	0.065503422	1.731594919	0.083345711	0.246451521	ELF4		protein_coding
ENSG00000102038.16	1641.226659	0.348644573	0.112011758	3.112571218	0.001854653	0.018117058	SMARCA1		protein_coding
ENSG00000102043.16	87.32343923	0.043369285	0.121012784	0.358385982	0.72005448	0.853585802	MTMR8		protein_coding
ENSG00000102048.16	185.2863641	0.070659212	0.115993457	0.609165497	0.542414748	0.737451874	ASB9		protein_coding
ENSG00000102053.12	305.2267161	0.027205317	0.108172609	0.251499126	0.801428238	0.90116732	ZC3H12B		protein_coding
ENSG00000102054.18	7923.117923	-0.154696929	0.052712322	-2.934739425	0.003338279	0.02782559	RBBP7		protein_coding
ENSG00000102055.7	0.596833655	0.157729418	0.456372546	0.345615483	0.729631709		PPP1R2C		protein_coding
ENSG00000102057.10	293.3456007	0.25481321	0.098321632	2.591629179	0.009552267	0.058488007	KCND1		protein_coding
ENSG00000102076.10	0.574267123	-0.403031238	0.443887782	-0.907957493	0.36390068		OPN1LW		protein_coding
ENSG00000102078.16	395.0865532	-0.03943499	0.055943396	-0.704908763	0.480867017	0.689826567	SLC25A14		protein_coding
ENSG00000102081.15	3201.687944	0.094952873	0.05815038	1.632884821	0.102493223	0.279566048	FMR1		protein_coding
ENSG00000102096.9	2821.578641	-0.503974566	0.102167043	-4.932848688	8.10389E-07	4.42536E-05	PIM2		protein_coding
ENSG00000102098.19	189.9022282	0.018607624	0.149374812	0.124570025	0.90086396	0.952769187	SCML2		protein_coding
ENSG00000102100.16	3183.642036	0.0566672	0.0661733	0.856345383	0.391806766	0.615832137	SLC35A2		protein_coding
ENSG00000102103.17	2214.677361	-0.131907344	0.059666361	-2.210748926	0.027053228	0.119116756	PQBP1		protein_coding
ENSG00000102104.9	2.217807715	-0.061059883	0.253113205	-0.241235469	0.809372622	0.905541771	RS1		protein_coding
ENSG00000102109.9	1919.1571	-0.177684391	0.260736815	-0.68147028	0.495573966	0.702005411	PCSK1N		protein_coding
ENSG00000102119.11	3728.578326	-0.119807711	0.061097494	-1.960926744	0.049887569	0.177809489	EMD		protein_coding
ENSG00000102125.16	1313.718913	-0.101053147	0.057415988	-1.76001755	0.078404831	0.237151893	TAZ		protein_coding
ENSG00000102128.8	1.211437445	0.149602677	0.192224835	0.778269245	0.436410303	0.653979492	RAB40AL		protein_coding
ENSG00000102144.15	22581.15783	0.108653919	0.067718109	1.604503157	0.108603189	0.289031463	PGK1		protein_coding
ENSG00000102145.15	9.28732674	-0.201618349	0.144526569	-1.395026195	0.163007941	0.370091197	GATA1		protein_coding
ENSG00000102158.20	6191.590988	0.078894329	0.051734232	1.524992761	0.127260903	0.318550657	MAGT1		protein_coding
ENSG00000102172.16	5632.44783	-0.005030681	0.063079732	-0.07975114	0.936435185	0.969859815	SMS		protein_coding
ENSG00000102174.9	50.5012094	0.010950978	0.116622913	0.093900743	0.925188004	0.96471831	PHEX		protein_coding
ENSG00000102178.13	3203.017403	0.03581414	0.055786071	0.641990722	0.520879207	0.720948838	UBL4A		protein_coding
ENSG00000102181.21	2264.260603	0.148644483	0.08910014	1.668285635	0.095259038	0.267158135	CD99L2		protein_coding
ENSG00000102189.17	3198.128638	-0.114185863	0.052847537	-2.160665732	0.03072117	0.129571856	EEA1		protein_coding
ENSG00000102195.10	3.146390361	1.101652444	0.374448449	2.942067055	0.003260293	0.027332397	GPR50		protein_coding
ENSG00000102218.6	1950.834331	-0.013541114	0.059434691	-0.22783183	0.819776981	0.911242035	RP2		protein_coding

ENSG00000102221.14	1171.882635	-0.063901204	0.061822707	-1.033620291	0.30131371	0.530926672	JADE3	protein_coding
ENSG00000102225.16	8020.401589	0.060965164	0.05831089	1.045519352	0.295783001	0.525494775	CDK16	protein_coding
ENSG00000102226.10	4546.746682	0.063418454	0.060104796	1.055131343	0.291365259	0.521359612	USP11	protein_coding
ENSG00000102230.14	53.53526248	0.025101742	0.188689138	0.13303226	0.894167868	0.949630416	PCYT1B	protein_coding
ENSG00000102239.5	3.876688949	-0.577201297	0.267676243	-2.156341145	0.031057037	0.130577552	BRS3	protein_coding
ENSG00000102241.12	3866.741654	0.015217521	0.048673339	0.312645931	0.754549678	0.873900397	HTATSF1	protein_coding
ENSG00000102243.13	238.8328107	0.034808246	0.305365546	0.11398878	0.909246688	0.957061147	VGLL1	protein_coding
ENSG00000102245.8	130.8228487	-0.730789318	0.139140315	-5.252175233	1.50313E-07	1.12614E-05	CD40LG	protein_coding
ENSG00000102265.12	39320.69061	0.394149323	0.105223304	3.745836793	0.000179793	0.003142743	TIMP1	protein_coding
ENSG00000102271.14	83.42452431	0.030400368	0.16693014	0.182114315	0.855493015	0.929424191	KLHL4	protein_coding
ENSG00000102287.19	805.2768734	0.077788194	0.198431467	0.392015416	0.695046823	0.839167024	GABRE	protein_coding
ENSG00000102290.22	12.51235856	-0.846209831	0.233476179	-3.624394727	0.000289639	0.00452874	PCDH11X	protein_coding
ENSG00000102302.8	428.25843	0.589262239	0.119645188	4.925080972	8.43254E-07	4.57046E-05	FGD1	protein_coding
ENSG00000102309.15	1084.175958	-0.149439207	0.060970144	-2.451022706	0.014245097	0.077314862	PIN4	protein_coding
ENSG00000102312.23	775.8447639	0.266497249	0.072693123	3.666058623	0.000246317	0.003988769	PORCN	protein_coding
ENSG00000102313.9	25.14597867	0.8103591	0.230427682	3.516761068	0.000436847	0.006148732	ITIH6	protein_coding
ENSG00000102316.17	5864.048615	0.229126468	0.065300857	3.50878196	0.000450164	0.006287432	MAGED2	protein_coding
ENSG00000102317.18	8621.69108	-0.083132988	0.055829598	-1.489048662	0.136474555	0.332440317	RBM3	protein_coding
ENSG00000102349.18	341.4158038	0.098177628	0.126468895	0.77629862	0.437572688	0.654473677	KLF8	protein_coding
ENSG00000102359.8	951.4296125	0.700351888	0.107793305	6.497174297	8.18426E-11	2.33102E-08	SRPX2	protein_coding
ENSG00000102362.15	378.448008	0.463644022	0.091789859	5.051146495	4.39166E-07	2.68786E-05	SYTL4	protein_coding
ENSG00000102383.14	82.74708665	0.322400964	0.150113352	2.147716769	0.031736262	0.132367066	ZDHHC15	protein_coding
ENSG00000102384.13	448.7116949	0.018394906	0.083143919	0.221241753	0.824904201	0.913501271	CENPI	protein_coding
ENSG00000102385.12	74.67477577	0.730535185	0.1447126	5.048179532	4.4604E-07	2.71155E-05	DRP2	protein_coding
ENSG00000102387.16	17.44728545	0.352022841	0.18395564	1.913628964	0.055667582	0.190529862	TAF7L	protein_coding
ENSG00000102390.11	1642.456616	-0.041974188	0.072002306	-0.582956158	0.559922814	0.749447323	PBDC1	protein_coding
ENSG00000102393.14	1758.046692	-0.088445748	0.066363343	-1.332750036	0.182613847	0.396283234	GLA	protein_coding
ENSG00000102401.20	2380.902642	0.367825422	0.083464176	4.4069856	1.04819E-05	0.000338537	ARMCX3	protein_coding
ENSG00000102409.10	732.058087	-0.014668893	0.128456622	-0.11419336	0.909084516	0.957009712	BEX4	protein_coding
ENSG00000102445.20	497.8640757	-0.339173371	0.149123928	-2.274439624	0.022939567	0.106684901	RUBCNL	protein_coding
ENSG00000102452.18	145.5364912	0.41141133	0.157058193	2.619483408	0.008806306	0.055200285	NALCN	protein_coding
ENSG00000102466.16	101.3333067	-0.138684594	0.176686445	-0.784919263	0.432500907	0.650550275	FGF14	protein_coding
ENSG00000102468.11	119.5476548	0.022970687	0.163924907	0.140129329	0.888557808	0.946808578	HTR2A	protein_coding
ENSG00000102471.14	4817.995255	0.061306887	0.071919586	0.852436594	0.393971817	0.617656644	NDFIP2	protein_coding
ENSG00000102524.11	1147.038901	-0.242112693	0.111740764	-2.166735603	0.03025502	0.128260567	TNFSF13B	protein_coding
ENSG00000102531.16	6752.953602	0.13227848	0.062084232	2.13062924	0.033119699	0.135912714	FNDC3A	protein_coding
ENSG00000102539.5	20.84729746	-0.221981057	0.247624179	-0.896443383	0.370016006	0.596705924	MLNR	protein_coding
ENSG00000102543.14	447.0245099	-0.056521448	0.053192175	-1.062589526	0.287968139	0.517689257	CDADC1	protein_coding
ENSG00000102547.19	973.6743436	-0.382801466	0.112565077	-3.40071251	0.000672105	0.008472175	CAB39L	protein_coding
ENSG00000102554.14	24329.23367	0.29176757	0.093243007	3.129109387	0.00175337	0.017408661	KLF5	protein_coding
ENSG00000102572.15	7036.49165	0.189360263	0.062655212	3.022258739	0.00250896	0.022501121	STK24	protein_coding
ENSG00000102575.13	3366.86507	-0.0852247	0.106107313	-0.803193455	0.421862928	0.641210046	ACP5	protein_coding

ENSG00000102580.15	6617.382159	0.186381707	0.057349051	3.249952784	0.001154242	0.012810601	DNAJC3	protein_coding
ENSG00000102595.20	1354.668962	0.22668185	0.055624854	4.075190047	4.59768E-05	0.001073128	UGGT2	protein_coding
ENSG00000102606.18	4295.781286	0.167193818	0.050947892	3.281663124	0.001031968	0.011755816	ARHGEF7	protein_coding
ENSG00000102678.7	130.04606	-0.449507811	0.198033519	-2.269857206	0.023216248	0.10753555	FGF9	protein_coding
ENSG00000102683.8	15.22614628	0.35942017	0.208330252	1.725242332	0.08448381	0.248384225	SGCG	protein_coding
ENSG00000102699.6	10111.56163	0.137761949	0.066676557	2.066122714	0.038816894	0.151145487	PARP4	protein_coding
ENSG00000102710.20	2934.732769	0.141165462	0.057760526	2.443978132	0.014526307	0.078343178	SUPT20H	protein_coding
ENSG00000102738.8	1097.610659	-0.173730725	0.060585615	-2.867524341	0.00413697	0.032482294	MRPS31	protein_coding
ENSG00000102743.15	1083.590692	-0.082645085	0.072522576	-1.139577359	0.254462422	0.481247139	SLC25A15	protein_coding
ENSG00000102753.10	4436.48506	-0.010685976	0.051078146	-0.209208369	0.83428558	0.918745109	KPNA3	protein_coding
ENSG00000102755.12	3238.335736	0.082312131	0.079188184	1.039449662	0.298595658	0.52842361	FLT1	protein_coding
ENSG00000102760.13	1371.484067	0.056507949	0.085486608	0.661015229	0.508602547	0.712167771	RGCC	protein_coding
ENSG00000102763.18	2477.108118	-0.081891295	0.061331485	-1.335224397	0.181802914	0.39519176	VWA8	protein_coding
ENSG00000102780.17	2771.930018	0.164247452	0.08020282	2.047901215	0.04056968	0.155327866	DGKH	protein_coding
ENSG00000102781.14	812.8985319	-0.026178607	0.09823097	-0.266500541	0.789853743	0.894430033	KATNAL1	protein_coding
ENSG00000102786.15	2803.014783	0.033591756	0.049134604	0.68366799	0.494184841	0.700995031	INTS6	protein_coding
ENSG00000102794.10	9.036128341	-0.325011041	0.21903756	-1.483814198	0.13785822	0.33424119	ACOD1	protein_coding
ENSG00000102796.11	205.2625362	-0.166374276	0.073499486	-2.263611415	0.023598025	0.108682981	DHRS12	protein_coding
ENSG00000102802.10	644.4078161	0.727483333	0.150048658	4.848316151	1.24514E-06	6.19226E-05	MEDAG	protein_coding
ENSG00000102804.15	11819.0654	-0.041855702	0.070425647	-0.594324716	0.552294951	0.744146866	TSC22D1	protein_coding
ENSG00000102805.16	541.4459038	0.019642664	0.055789728	0.35208389	0.72477535	0.85645022	CLN5	protein_coding
ENSG00000102837.7	146484.1306	-0.757255473	0.284592654	-2.660839843	0.007794602	0.050825286	OLFM4	protein_coding
ENSG00000102854.16	8320.804478	1.027136041	0.211704517	4.851743616	1.22381E-06	6.11581E-05	MSLN	protein_coding
ENSG00000102858.13	7792.815776	-0.014296859	0.049605899	-0.288208849	0.773186876	0.885287698	MGRN1	protein_coding
ENSG00000102870.6	2495.236062	0.135287548	0.05562484	2.432142695	0.015009793	0.080151681	ZNF629	protein_coding
ENSG00000102871.16	1651.180607	0.021132628	0.0642801	0.328758488	0.742338242	0.867136562	TRADD	protein_coding
ENSG00000102878.17	348.4906215	-0.177115859	0.094210947	-1.879992343	0.060109121	0.200059206	HSF4	protein_coding
ENSG00000102879.16	3199.501826	-0.571395428	0.123968379	-4.609203046	4.04215E-06	0.000157552	CORO1A	protein_coding
ENSG00000102882.12	8495.131502	-0.016378114	0.073688797	-0.222260571	0.824111045	0.913264171	MAPK3	protein_coding
ENSG00000102886.15	349.1265787	-0.128108495	0.142067028	-0.90174685	0.367191359	0.594306116	GDPD3	protein_coding
ENSG00000102890.16	2616.170496	0.05103599	0.086187497	0.59215074	0.553749652	0.745098147	ELMO3	protein_coding
ENSG00000102891.4	1.164048007	0.837409491	0.516713502	1.620645652	0.105093656	0.283794931	MT4	protein_coding
ENSG00000102893.16	4521.474155	-0.025763911	0.052984982	-0.486249323	0.626790405	0.796687827	PHKB	protein_coding
ENSG00000102897.10	1664.552228	-0.347314628	0.066837177	-5.196428753	2.03153E-07	1.43715E-05	LYRM1	protein_coding
ENSG00000102898.12	7008.137978	-0.0758735	0.06359864	-1.193005066	0.232867389	0.457266682	NUTF2	protein_coding
ENSG00000102900.13	2853.139625	-0.024960122	0.049587108	-0.503359112	0.614711813	0.788602281	NUP93	protein_coding
ENSG00000102901.13	1014.643892	-0.021266832	0.060829115	-0.349615995	0.726626906	0.857361493	CENPT	protein_coding
ENSG00000102904.15	35.32326157	0.012960302	0.099518052	0.130230666	0.896383933	0.950870411	TSNAXIP1	protein_coding
ENSG00000102908.22	5046.698221	0.082043241	0.072903771	1.125363473	0.260435043	0.487666632	NFAT5	protein_coding
ENSG00000102910.14	5208.610329	-0.096231329	0.043311385	-2.221848354	0.026293556	0.116774794	LONP2	protein_coding
ENSG00000102921.8	3813.125291	0.034380589	0.044536401	0.77196603	0.440134547	0.656602402	N4BP1	protein_coding
ENSG00000102924.12	46.79408381	-0.965033017	0.242850889	-3.973767689	7.07446E-05	0.001520136	CBLN1	protein_coding

ENSG00000102931.8	77.44868837	0.050851674	0.083126164	0.611740899	0.540709197	0.736125061	ARL2BP	protein_coding
ENSG00000102934.10	3135.572637	-0.132501027	0.11279464	-1.174710315	0.240110631	0.465490745	PLLP	protein_coding
ENSG00000102935.12	776.6762855	0.155686793	0.134764605	1.155249876	0.247988115	0.474553281	ZNF423	protein_coding
ENSG00000102962.5	549.0555968	-0.320375315	0.141612899	-2.262331454	0.023676932	0.108883725	CCL22	protein_coding
ENSG00000102967.12	683.9914182	-0.143593465	0.053510003	-2.683488251	0.007285854	0.048363954	DHODH	protein_coding
ENSG00000102970.11	39.55090324	-0.301112656	0.158324484	-1.901870443	0.057188094	0.194042325	CCL17	protein_coding
ENSG00000102974.16	4490.000661	-0.019166673	0.033034889	-0.580194855	0.561783223	0.750899562	CTCF	protein_coding
ENSG00000102977.17	1379.435926	-0.015172782	0.063433656	-0.239191348	0.810957211	0.906432462	ACD	protein_coding
ENSG00000102978.13	3600.217025	-0.023880431	0.050615261	-0.471802991	0.637067416	0.803458486	POLR2C	protein_coding
ENSG00000102981.10	340.6728527	-0.098490523	0.094192487	-1.045630349	0.295731732	0.525455993	PARD6A	protein_coding
ENSG00000102984.15	120.0336335	-0.086820851	0.062328905	-1.392946834	0.163635868	0.370795083	ZNF821	protein_coding
ENSG00000102996.5	9328.80014	-0.101451901	0.093681476	-1.08294516	0.278832765	0.507640352	MMP15	protein_coding
ENSG00000103005.12	3717.41782	0.046018725	0.045055436	1.021380093	0.307074393	0.536585966	USB1	protein_coding
ENSG00000103018.17	7093.336154	-0.152226255	0.077959861	-1.952623493	0.050864225	0.179896952	CYB5B	protein_coding
ENSG00000103021.9	368.5219173	0.220834137	0.094888504	2.327301295	0.019949235	0.097088207	CCDC113	protein_coding
ENSG00000103023.12	1.852438376	-0.022410097	0.299115138	-0.074921305	0.940277325	0.9717879	PRSS54	protein_coding
ENSG00000103024.7	1333.177646	-0.241810942	0.071934013	-3.361566134	0.000775018	0.009471656	NME3	protein_coding
ENSG00000103034.14	343.1506799	0.320027557	0.132137437	2.4219295	0.015438345	0.081639586	NDRG4	protein_coding
ENSG00000103035.11	2117.925512	-0.011221674	0.048860454	-0.229667809	0.818349911	0.910367786	PSMD7	protein_coding
ENSG00000103037.12	669.9656722	0.009848015	0.069154551	0.142405879	0.886759419	0.945880572	SETD6	protein_coding
ENSG00000103042.9	2151.764298	0.094630097	0.052011192	1.819417969	0.068847687	0.217570881	SLC38A7	protein_coding
ENSG00000103043.15	4311.459126	-0.048586479	0.046442721	-1.046159175	0.295487548	0.525290601	VAC14	protein_coding
ENSG00000103044.11	809.9358004	-0.244654361	0.176347149	-1.387345145	0.165336543	0.373309355	HAS3	protein_coding
ENSG00000103047.8	699.2262393	-0.100742148	0.051798934	-1.94486913	0.051790731	0.182021509	TANGO6	protein_coding
ENSG00000103051.20	3144.614448	0.013328068	0.048975905	0.272135207	0.785518059	0.891443018	COG4	protein_coding
ENSG00000103056.12	3235.464849	0.056661199	0.144713829	0.391539629	0.695398402	0.839335702	SMPD3	protein_coding
ENSG00000103061.13	300.3538194	-0.009599947	0.053909295	-0.178075917	0.858663355	0.931189136	SLC7A6OS	protein_coding
ENSG00000103064.15	749.1722123	0.058343886	0.079678817	0.732238354	0.464023099	0.676210901	SLC7A6	protein_coding
ENSG00000103066.13	1176.670551	0.135295608	0.059691174	2.266593189	0.023415089	0.108111815	PLA2G15	protein_coding
ENSG00000103067.14	4118.897275	0.050121544	0.089125295	0.562371709	0.57386279	0.759293346	ESRP2	protein_coding
ENSG00000103089.9	4162.350771	0.165606688	0.125800167	1.316426622	0.188030892	0.403278973	FA2H	protein_coding
ENSG00000103091.15	2048.053926	-0.053014286	0.04614211	-1.148935028	0.250582773	0.477350038	WDR59	protein_coding
ENSG00000103111.15	4696.632433	0.037008855	0.043322512	0.854263827	0.392958825	0.617009917	MON1B	protein_coding
ENSG00000103121.9	855.5308302	-0.134799384	0.058254372	-2.31397884	0.020668883	0.099358843	CMC2	protein_coding
ENSG00000103126.15	3217.461068	-0.030534938	0.066477474	-0.459327595	0.645998934	0.808900541	AXIN1	protein_coding
ENSG00000103145.11	2131.212077	-0.033036719	0.082165349	-0.402076054	0.687628055	0.835065542	HCFC1R1	protein_coding
ENSG00000103148.17	3437.265772	-0.114366981	0.050147819	-2.280597297	0.022572287	0.105476161	NPRL3	protein_coding
ENSG00000103150.7	881.923446	0.032344639	0.062802265	0.515023451	0.606536632	0.78306238	MLYCD	protein_coding
ENSG00000103152.12	1119.91874	0.057218788	0.071937671	0.795393943	0.426384382	0.645023684	MPG	protein_coding
ENSG00000103154.9	15.95405455	0.44747655	0.169486719	2.640186511	0.008286041	0.053006579	NECAB2	protein_coding
ENSG00000103160.12	823.1794571	0.085665697	0.058067993	1.475265331	0.140141223	0.337623512	HSDL1	protein_coding
ENSG00000103168.17	1919.019538	0.046761607	0.050245827	0.930656519	0.352031268	0.58066038	TAF1C	protein_coding

ENSG00000103174.13	1202.637167	-0.076786137	0.059459983	-1.291391845	0.196567837	0.413788543	NAGPA	protein_coding
ENSG00000103175.11	880.0400271	0.042142041	0.133900167	0.314727326	0.752968694	0.873100132	WFDC1	protein_coding
ENSG00000103184.12	106.517708	0.1026869	0.188201107	0.54562325	0.585324939	0.76783482	SEC14L5	protein_coding
ENSG00000103187.8	14052.51739	-0.173024131	0.087304782	-1.98184024	0.047497124	0.172237708	COTL1	protein_coding
ENSG00000103194.16	6065.776243	-0.015987714	0.041089517	-0.389094719	0.69720608	0.840446731	USP10	protein_coding
ENSG00000103196.12	5554.150462	0.212604048	0.111192091	1.912042895	0.055870692	0.191000013	CRISPLD2	protein_coding
ENSG00000103197.18	6054.720348	0.059527513	0.048757418	1.22089142	0.222127133	0.444693072	TSC2	protein_coding
ENSG00000103199.14	845.0822884	-0.027095567	0.048163424	-0.562575599	0.573723911	0.759175479	ZNF500	protein_coding
ENSG00000103200.6	2.776054539	-0.222009168	0.232171603	-0.956228777	0.338956664	0.568759281	AC069335.1	processed_pseudogene
ENSG00000103202.13	3483.29524	-0.089859748	0.102264152	-0.878702326	0.379562697	0.605146392	NME4	protein_coding
ENSG00000103222.20	6368.8573	-0.018727774	0.067050435	-0.279308757	0.780007887	0.888584375	ABCC1	protein_coding
ENSG00000103226.19	483.9595679	0.167003396	0.105448892	1.5837378	0.113253399	0.296785337	NOMO3	protein_coding
ENSG00000103227.19	770.105844	-0.125880379	0.084012518	-1.498352658	0.13404165	0.328908845	LMF1	protein_coding
ENSG00000103241.7	2124.735927	0.006549624	0.131896755	0.049657203	0.960395562	0.981568999	FOXF1	protein_coding
ENSG00000103245.14	1047.631528	-0.081379622	0.055343523	-1.470445272	0.141441198	0.339506138	CIAO3	protein_coding
ENSG00000103248.19	952.8243516	-0.009660789	0.044906931	-0.215129125	0.829666647	0.916289069	MTHFSD	protein_coding
ENSG00000103249.18	3326.350127	-0.008163208	0.063531905	-0.1284899	0.897761291	0.951450551	CLCN7	protein_coding
ENSG00000103253.19	475.295033	0.059187312	0.122000108	0.485141477	0.627575992	0.797081872	HAGHL	protein_coding
ENSG00000103254.10	909.3499529	-0.156062741	0.080794387	-1.931603749	0.053408433	0.185463326	ANTKMT	protein_coding
ENSG00000103257.9	10964.45652	0.212193849	0.131180072	1.617576863	0.105753819	0.28473202	SLC7A5	protein_coding
ENSG00000103260.9	1369.343299	0.271675498	0.113283391	2.398193548	0.016476157	0.085548496	METRN	protein_coding
ENSG00000103264.18	2051.869011	-0.06167101	0.057735047	-1.068172859	0.285442545	0.514775924	FBXO31	protein_coding
ENSG00000103266.11	1487.868554	-0.010415275	0.069406045	-0.150062939	0.880714959	0.942630462	STUB1	protein_coding
ENSG00000103269.14	157.1263303	-0.269702193	0.121444398	-2.22078743	0.026365362	0.116974485	RHBDL1	protein_coding
ENSG00000103274.11	600.3991455	-0.089241745	0.058452444	-1.526741028	0.126825421	0.317860946	NUBP1	protein_coding
ENSG00000103275.21	4611.120364	-0.096664567	0.048459512	-1.994749081	0.046070253	0.168691995	UBE2I	protein_coding
ENSG00000103310.11	4.213469442	-0.249501918	0.269011617	-0.927476373	0.353679253	0.581968841	ZP2	protein_coding
ENSG00000103313.13	120.157079	0.174377443	0.134669616	1.294853642	0.195370723	0.412451145	MEFV	protein_coding
ENSG00000103316.12	141.1266451	-0.45493707	0.156838255	-2.900676696	0.003723578	0.030115511	CRYM	protein_coding
ENSG00000103319.12	3224.227439	-0.032853632	0.054048218	-0.607857817	0.543281779	0.737992004	EEF2K	protein_coding
ENSG00000103326.12	6190.220728	-0.064743494	0.072196605	-0.896766463	0.369843547	0.596516109	CAPN15	protein_coding
ENSG00000103335.22	8907.396819	0.236723144	0.067316501	3.516569329	0.000437162	0.006151188	PIEZO1	protein_coding
ENSG00000103342.13	10928.37918	0.030132315	0.04522293	0.666306107	0.505215463	0.709248228	GSPT1	protein_coding
ENSG00000103343.13	515.8636712	-0.075496799	0.046337496	-1.62928094	0.103253559	0.280813852	ZNF174	protein_coding
ENSG00000103351.13	845.5115006	-0.171251397	0.057342477	-2.986466699	0.002822216	0.024503989	CLUAP1	protein_coding
ENSG00000103353.16	3566.418121	0.158338698	0.048798996	3.244712208	0.001175694	0.012990164	UBFD1	protein_coding
ENSG00000103355.13	96.60589838	0.019695682	0.218037976	0.09033143	0.928023845	0.966019996	PRSS33	protein_coding
ENSG00000103356.18	1825.714744	-0.009773246	0.055414538	-0.176366102	0.860006334	0.931893212	EARS2	protein_coding
ENSG00000103363.15	10046.45866	-0.106749142	0.071438653	-1.494277075	0.135103214	0.330455365	ELOB	protein_coding
ENSG00000103365.15	3898.91463	-0.19108843	0.049701916	-3.844689372	0.000120705	0.002288935	GGA2	protein_coding
ENSG00000103375.11	8.286934581	-0.634384467	0.242949536	-2.611177938	0.009023093	0.056187387	AQP8	protein_coding
ENSG00000103381.12	2072.601516	-0.192518447	0.073515195	-2.618757202	0.008825074	0.055294066	CPPED1	protein_coding

ENSG00000103404.14	1301.694949	0.058002416	0.055542696	1.044285204	0.296353456	0.526167235	USP31	protein_coding
ENSG00000103415.12	2620.702645	-0.140815646	0.063011972	-2.234744303	0.02543414	0.114325094	HMOX2	protein_coding
ENSG00000103423.14	3740.289593	-0.117726007	0.05589719	-2.106116716	0.035194215	0.141299488	DNAJA3	protein_coding
ENSG00000103426.12	0						CORO7-PAM16	protein_coding
ENSG00000103429.11	3959.28051	-0.011840874	0.049641149	-0.238529412	0.811470506	0.906889881	BFAR	protein_coding
ENSG00000103449.12	177.9539899	0.142548013	0.172072394	0.828418841	0.40743334	0.628463511	SALL1	protein_coding
ENSG00000103460.17	2871.98478	-0.056200827	0.124164967	-0.452630308	0.650814971	0.811858685	TOX3	protein_coding
ENSG00000103472.10	16.83031655	-0.256231813	0.121435664	-2.110021086	0.03485654	0.140379558	RRN3P2	lncRNA
ENSG00000103479.16	3736.711773	0.030051737	0.057784501	0.52006571	0.603017777	0.780842886	RBL2	protein_coding
ENSG00000103485.19	1943.90279	-0.103075054	0.189500301	-0.543930822	0.586489078	0.768667766	QPRT	protein_coding
ENSG00000103489.12	1978.712365	0.166192822	0.092086429	1.80474825	0.071114086	0.221942173	XYLT1	protein_coding
ENSG00000103490.14	1744.314174	-0.018613837	0.094229453	-0.19753736	0.843407048	0.923169068	PYCARD	protein_coding
ENSG00000103494.15	600.6905124	0.040686967	0.058008489	0.701396778	0.483055432	0.691193639	RPGRIP1L	protein_coding
ENSG00000103495.14	3185.417029	-0.011119176	0.086885022	-0.127975747	0.898168166	0.951592637	MAZ	protein_coding
ENSG00000103496.15	2183.263011	-0.051974878	0.047914007	-1.084753307	0.278030931	0.506919864	STX4	protein_coding
ENSG00000103502.14	4246.352008	0.023090085	0.055973906	0.412515156	0.679961875	0.830460015	CDIPT	protein_coding
ENSG00000103507.14	3568.667083	0.038583573	0.059018551	0.653753317	0.513270747	0.715588822	BCKDK	protein_coding
ENSG00000103510.20	2247.830844	-0.004893782	0.045115036	-0.108473414	0.913620168	0.959160308	KAT8	protein_coding
ENSG00000103512.15	3890.823611	0.061117161	0.05754631	1.062051786	0.288212176	0.518032592	NOMO1	protein_coding
ENSG00000103522.16	286.0160236	-0.580252627	0.136962667	-4.236575119	2.26955E-05	0.000615638	IL21R	protein_coding
ENSG00000103528.17	357.870268	0.61622328	0.122067482	5.048218174	4.4595E-07	2.71155E-05	SYT17	protein_coding
ENSG00000103534.17	10975.8486	0.021196468	0.122339679	0.17325914	0.862447739	0.932948383	TMC5	protein_coding
ENSG00000103540.16	1507.31883	0.019406198	0.060371217	0.321447856	0.747871022	0.870296033	CCP110	protein_coding
ENSG00000103544.15	1891.870488	-0.062526287	0.089074617	-0.701954035	0.482707829	0.690962095	VPS35L	protein_coding
ENSG00000103546.18	16.7548785	-0.582660822	0.266769666	-2.184134472	0.028952368	0.124595828	SLC6A2	protein_coding
ENSG00000103549.22	3958.10516	0.134487514	0.061083927	2.20168416	0.027687626	0.12075097	RNF40	protein_coding
ENSG00000103550.14	1391.146309	-0.121922312	0.058627892	-2.079595694	0.037562632	0.147705002	KNOP1	protein_coding
ENSG00000103569.10	463.1443897	0.383881245	0.174244713	2.203115603	0.027586602	0.120563769	AQP9	protein_coding
ENSG00000103591.13	2956.617553	0.109922464	0.051868388	2.119257363	0.034068723	0.138322252	AAGAB	protein_coding
ENSG00000103599.20	141.0864938	-0.02721667	0.111411524	-0.244289543	0.807006582	0.904104582	IQCH	protein_coding
ENSG00000103642.12	1250.270046	-0.022035444	0.051756924	-0.42574872	0.670290957	0.824212563	LACTB	protein_coding
ENSG00000103647.13	164.3860552	0.33394698	0.1373805	2.430817915	0.015064783	0.080322778	CORO2B	protein_coding
ENSG00000103653.16	6450.358065	-0.159298789	0.064360105	-2.475116986	0.013319254	0.073796965	CSK	protein_coding
ENSG00000103657.14	3865.125173	-0.041496458	0.059617306	-0.696047188	0.486399279	0.694056818	HERC1	protein_coding
ENSG00000103671.10	1358.629184	0.074130642	0.040073516	1.849866191	0.064332838	0.208685538	TRIP4	protein_coding
ENSG00000103707.10	693.2696867	-0.052571094	0.046269206	-1.136200467	0.255872675	0.482890948	MTFMT	protein_coding
ENSG00000103710.11	718.2845991	0.215802206	0.109209471	1.976039299	0.048150326	0.173753459	RASL12	protein_coding
ENSG00000103723.16	124.7101225	0.195870671	0.205486539	0.953204388	0.340486523	0.570463433	AP3B2	protein_coding
ENSG00000103740.10	31.86790992	-0.132726898	0.106923557	-1.241325136	0.214485663	0.43549351	ACSBG1	protein_coding
ENSG00000103742.12	483.189377	0.488488723	0.116165968	4.205093204	2.60975E-05	0.000682622	IGDCC4	protein_coding
ENSG00000103769.10	11686.67243	-0.003241256	0.05283946	-0.061341578	0.951087179	0.977130929	RAB11A	protein_coding
ENSG00000103811.18	13828.59752	-0.450141947	0.104004146	-4.328115397	1.50391E-05	0.000447343	CTSH	protein_coding

ENSG00000103832.10	8.505569482	0.13405219	0.164256943	0.816112779	0.414435636	0.634851604	GOLGA8UP	unprocessed_pseudogene
ENSG00000103852.13	841.2535255	-0.038956864	0.079506761	-0.489981779	0.624146793	0.794924329	TTC23	protein_coding
ENSG00000103855.18	5955.350853	0.507678126	0.066329448	7.653887453	1.94993E-14	1.57356E-11	CD276	protein_coding
ENSG00000103876.13	798.8823646	0.050668924	0.089625166	0.56534259	0.571840765	0.757880323	FAH	protein_coding
ENSG00000103888.17	4742.952478	0.624152894	0.171385109	3.641815201	0.000270722	0.004302432	CEMIP	protein_coding
ENSG00000103932.12	2163.580127	-0.010047244	0.049174221	-0.204319341	0.838103957	0.920345139	RPAP1	protein_coding
ENSG00000103942.13	1000.508394	0.58967608	0.181581994	3.247436967	0.001164495	0.01289913	HOMER2	protein_coding
ENSG00000103966.11	3759.474365	0.065724181	0.058542948	1.122666064	0.261579346	0.488989883	EHD4	protein_coding
ENSG00000103978.16	2817.729345	0.084545269	0.047869522	1.76616071	0.077368891	0.234964398	TMEM87A	protein_coding
ENSG00000103994.18	5009.179743	-0.029793369	0.061638624	-0.483355516	0.628843328	0.798109967	ZNF106	protein_coding
ENSG00000103995.14	539.3776763	-0.081204869	0.068557999	-1.184469639	0.236227208	0.461203989	CEP152	protein_coding
ENSG00000104043.15	228.7478355	-0.038142743	0.101612087	-0.375376039	0.707380822	0.846762281	ATP8B4	protein_coding
ENSG00000104044.16	87.76522783	-0.038678473	0.250899689	-0.154159112	0.877484281	0.940945236	OCA2	protein_coding
ENSG00000104047.15	1111.443247	0.023335162	0.057017803	0.409260976	0.682348151	0.832088579	DTWD1	protein_coding
ENSG00000104055.16	8.049344011	0.215297868	0.246656723	0.872864378	0.382737004	0.607881645	TGM5	protein_coding
ENSG00000104059.4	127.6706336	-0.097842374	0.242992045	-0.402656695	0.687200795	0.834909815	FAM189A1	protein_coding
ENSG00000104064.18	1188.639014	-0.095292616	0.040798528	-2.335687604	0.019507529	0.095343158	GABPB1	protein_coding
ENSG00000104067.17	8834.530362	0.234785445	0.062895954	3.732918067	0.000189274	0.003268201	TJP1	protein_coding
ENSG00000104081.14	1484.388502	-0.005898453	0.094204288	-0.06261342	0.950074343	0.976701258	BMF	protein_coding
ENSG00000104093.14	2127.580474	-0.064673162	0.069087907	-0.936099596	0.349221911	0.578368928	DMXL2	protein_coding
ENSG00000104112.9	83.62237948	-1.052603876	0.217927428	-4.830066064	1.36488E-06	6.66433E-05	SCG3	protein_coding
ENSG00000104129.10	504.3296802	-0.101722221	0.046398264	-2.192371256	0.02835271	0.122681567	DNAJC17	protein_coding
ENSG00000104131.13	4798.8752	-0.063577316	0.049067173	-1.295719965	0.19507198	0.412152809	EIF3J	protein_coding
ENSG00000104133.15	4743.876537	-0.035387823	0.054135865	-0.653685384	0.513314522	0.715592531	SPG11	protein_coding
ENSG00000104140.7	532.9071664	1.292822542	0.177038645	7.302487797	2.82495E-13	1.89389E-10	RHOV	protein_coding
ENSG00000104142.11	2072.862462	0.150345348	0.053116838	2.830464948	0.00464804	0.035336297	VPS18	protein_coding
ENSG00000104147.9	113.7614971	-0.083383499	0.085016669	-0.980790006	0.326696308	0.55643717	OIP5	protein_coding
ENSG00000104154.7	719.4523661	0.120042858	0.080409614	1.492891852	0.135465498	0.33096995	SLC30A4	protein_coding
ENSG00000104164.12	2828.386744	-0.011557885	0.042848266	-0.269739845	0.787360405	0.892696874	BLOC1S6	protein_coding
ENSG00000104177.18	468.7017173	-0.134118178	0.176986911	-0.75778586	0.448579189	0.663603078	MYEF2	protein_coding
ENSG00000104205.16	567.8340594	-0.19033794	0.065579506	-2.90239971	0.003703157	0.030011619	SGK3	protein_coding
ENSG00000104213.13	302.5327205	0.663985313	0.153664508	4.321006325	1.55319E-05	0.00045825	PDGFRL	protein_coding
ENSG00000104218.16	1862.230044	-0.189389653	0.06736649	-2.811333251	0.004933666	0.03685828	CSPP1	protein_coding
ENSG00000104219.13	2105.295939	0.364790337	0.124565245	2.928508156	0.003405929	0.028232864	ZDHHC2	protein_coding
ENSG00000104221.13	306.6474585	0.111493803	0.073229708	1.522521468	0.12787847	0.319307822	BRF2	protein_coding
ENSG00000104228.13	1351.358253	0.003924204	0.052922889	0.074149475	0.940891448	0.972043625	TRIM35	protein_coding
ENSG00000104231.11	2689.292876	-0.135801569	0.061858812	-2.19534719	0.028138702	0.122079981	ZFAND1	protein_coding
ENSG00000104237.11	56.91281748	0.030147029	0.19445496	0.155033478	0.876794924	0.94050689	RP1	protein_coding
ENSG00000104267.10	11925.0813	-0.532286109	0.196147588	-2.713702041	0.006653598	0.045415701	CA2	protein_coding
ENSG00000104290.11	1775.971955	-0.112733323	0.098559718	-1.143807273	0.252703573	0.479515071	FZD3	protein_coding
ENSG00000104299.15	1363.483301	-0.068125176	0.052197726	-1.305136859	0.19184625	0.408067752	INTS9	protein_coding
ENSG00000104312.8	2007.049058	0.021232765	0.072773937	0.291763307	0.770467602	0.883801472	RIPK2	protein_coding

ENSG00000104313.20	41.50787277	-0.01088143	0.172191445	-0.06319379	0.94961219	0.976387944	EYA1	protein_coding
ENSG00000104320.14	4542.235729	-0.093017126	0.055930112	-1.663095656	0.096293289	0.26893518	NBN	protein_coding
ENSG00000104321.11	696.0596602	0.06597635	0.189424714	0.34829853	0.727615998	0.857928522	TRPA1	protein_coding
ENSG00000104324.16	1597.87226	-0.097468146	0.090485065	-1.077173856	0.281402603	0.510349643	CPQ	protein_coding
ENSG00000104325.7	3596.850885	-0.248544994	0.074736467	-3.325618713	0.000882225	0.010423406	DECR1	protein_coding
ENSG00000104327.7	224.2335508	1.491628719	0.253099453	5.893448996	3.78217E-09	5.54935E-07	CALB1	protein_coding
ENSG00000104331.9	9269.131259	0.069969025	0.04829992	1.448636467	0.147439131	0.348609236	BPNT2	protein_coding
ENSG00000104332.12	1300.321853	0.10280245	0.227959627	0.450967795	0.65201276	0.812745217	SFRP1	protein_coding
ENSG00000104341.17	13756.59673	0.001234764	0.098410645	0.012547053	0.989989163	0.995414303	LAPTM4B	protein_coding
ENSG00000104343.21	2053.369696	-0.030573646	0.049992557	-0.611563959	0.54082629	0.73617492	UBE2W	protein_coding
ENSG00000104356.11	697.5230618	0.035342036	0.073083853	0.483582005	0.628682548	0.79795239	POP1	protein_coding
ENSG00000104361.10	2433.678172	0.115533379	0.079567936	1.452009236	0.146499037	0.347096648	NIPAL2	protein_coding
ENSG00000104365.16	2355.368633	-0.004817229	0.050665635	-0.095078816	0.924252225	0.964159838	IKBKB	protein_coding
ENSG00000104368.19	4442.043867	0.439527512	0.08749496	5.023460909	5.07486E-07	2.98444E-05	PLAT	protein_coding
ENSG00000104369.5	909.876501	0.360702138	0.154416178	2.335908982	0.019495986	0.095318812	JPH1	protein_coding
ENSG00000104371.5	37.1758972	-0.267333477	0.240968247	-1.109413711	0.26725175	0.495238541	DKK4	protein_coding
ENSG00000104375.17	1885.684893	-0.000748794	0.059021142	-0.012686882	0.989877604	0.995404463	STK3	protein_coding
ENSG00000104381.14	631.9913585	-0.082820373	0.110461057	-0.749769879	0.453393313	0.667876029	GDAP1	protein_coding
ENSG00000104388.15	9690.793627	-0.025556243	0.051084522	-0.500273709	0.616882364	0.789907295	RAB2A	protein_coding
ENSG00000104408.11	19093.98981	-0.101920201	0.063192241	-1.612859409	0.106775056	0.286228875	EIF3E	protein_coding
ENSG00000104412.8	2280.725023	0.024367997	0.054995355	0.443091904	0.657699261	0.81623788	EMC2	protein_coding
ENSG00000104413.18	9070.219538	-0.075131694	0.083168387	-0.903368421	0.366330394	0.59341956	ESRP1	protein_coding
ENSG00000104415.14	1146.572653	0.649712916	0.120802939	5.378287336	7.51977E-08	6.4633E-06	CCN4	protein_coding
ENSG00000104419.17	20728.00828	0.150589164	0.093257251	1.61477164	0.106360157	0.285661421	NDRG1	protein_coding
ENSG00000104427.12	560.2587179	0.261645857	0.085210921	3.070567193	0.002136526	0.020065387	ZC2HC1A	protein_coding
ENSG00000104432.14	355.6028502	-0.091353099	0.10016533	-0.912023144	0.361756534	0.589472553	IL7	protein_coding
ENSG00000104435.14	144.6995676	0.04884841	0.220355785	0.221679726	0.824563213	0.913399045	STMN2	protein_coding
ENSG00000104442.10	2609.225061	-0.130153288	0.048155016	-2.702798126	0.006875849	0.046418107	ARMC1	protein_coding
ENSG00000104447.13	1274.317302	0.332721946	0.123507854	2.693933504	0.007061425	0.0472609	TRPS1	protein_coding
ENSG00000104450.13	1835.331321	0.264829336	0.094137191	2.813227502	0.004904694	0.036717375	SPAG1	protein_coding
ENSG00000104472.10	3992.631046	-0.138055516	0.05453746	-2.53138882	0.011361183	0.066042063	CHRAC1	protein_coding
ENSG00000104490.18	731.9788502	-0.030990288	0.110135311	-0.281383764	0.77841607	0.887892291	NCALD	protein_coding
ENSG00000104497.14	783.5593118	-0.183683738	0.059822263	-3.070491324	0.002137069	0.020066161	SNX16	protein_coding
ENSG00000104499.7	0.78995482	-0.276256836	0.428937229	-0.644049565	0.519543294		GML	protein_coding
ENSG00000104517.13	7128.962099	0.103643273	0.061291907	1.690978126	0.09084098	0.25964695	UBR5	protein_coding
ENSG00000104518.11	3476.654458	-0.152592218	0.066331343	-2.300454219	0.0214225	0.101780232	GSDMD	protein_coding
ENSG00000104522.16	10666.92644	0.058176846	0.094908341	0.612979278	0.539890042	0.735556928	GFUS	protein_coding
ENSG00000104524.14	1303.68079	0.044710337	0.086601624	0.51627596	0.605661684	0.782589152	PYCR3	protein_coding
ENSG00000104529.17	3931.161629	-0.117094607	0.056649146	-2.0670145	0.038732788	0.150944258	EEF1D	protein_coding
ENSG00000104537.17	1775.820195	-0.342809867	0.214303159	-1.599649156	0.109676437	0.290574544	ANXA13	protein_coding
ENSG00000104549.12	3826.31136	0.271193243	0.112014191	2.421061485	0.01547526	0.081751168	SQLE	protein_coding
ENSG00000104611.12	2708.483545	-0.014608491	0.091022646	-0.160492925	0.872492794	0.938340494	SH2D4A	protein_coding

ENSG00000104613.12	3429.568478	-0.039445672	0.065164681	-0.605322875	0.544964485	0.739349856	INTS10	protein_coding
ENSG00000104626.15	1626.932698	-0.138829901	0.076365201	-1.817973353	0.069068206	0.217926667	ERI1	protein_coding
ENSG00000104635.15	15902.78363	-0.272434439	0.08649444	-3.149733554	0.001634194	0.016538152	SLC39A14	protein_coding
ENSG00000104643.10	1109.715523	0.005255656	0.082491194	0.063711717	0.949199775	0.976210607	MTMR9	protein_coding
ENSG00000104660.19	3373.574085	-0.042344816	0.055577369	-0.761907534	0.446115193	0.661709445	LEPROTL1	protein_coding
ENSG00000104671.8	1247.109786	0.013445094	0.06491017	0.207133864	0.835905316	0.91948702	DCTN6	protein_coding
ENSG00000104679.12	1450.962253	-0.063176384	0.062977422	-1.003159254	0.315784029	0.544922213	R3HCC1	protein_coding
ENSG00000104687.14	5774.547801	-0.130580833	0.073901797	-1.766950716	0.077236482	0.234759191	GSR	protein_coding
ENSG00000104689.10	1081.043081	0.064449449	0.08022564	0.803352251	0.421771165	0.641183397	TNFRSF10A	protein_coding
ENSG00000104691.16	516.3275074	-0.025431855	0.079654175	-0.319278375	0.749515429	0.871302189	UBXN8	protein_coding
ENSG00000104695.13	5287.274285	0.049428306	0.05690976	0.868538309	0.38509971	0.609827242	PPP2CB	protein_coding
ENSG00000104714.14	1376.382857	-0.131699651	0.05918828	-2.225096761	0.026074743	0.116110551	ERICH1	protein_coding
ENSG00000104722.14	27.95406526	-0.571539552	0.191718103	-2.981145458	0.002871724	0.024829583	NEFM	protein_coding
ENSG00000104723.21	828.2011863	0.398074978	0.131977022	3.016244584	0.002559267	0.022858207	TUSC3	protein_coding
ENSG00000104728.16	71.64755459	0.02581205	0.136155956	0.189577093	0.849640539	0.92625018	ARHGEF10	protein_coding
ENSG00000104731.14	1534.568357	-0.070308368	0.050021183	-1.405571857	0.159851298	0.365641699	KLHDC4	protein_coding
ENSG00000104738.18	7785.929848	-0.089399328	0.084731736	-1.055086709	0.29138567	0.521359612	MCM4	protein_coding
ENSG00000104755.15	4.943779301	-0.512938397	0.346795839	-1.479078865	0.139119238	0.336186523	ADAM2	protein_coding
ENSG00000104756.16	1976.415429	0.112669924	0.065636477	1.716574819	0.086056887	0.251195723	KCTD9	protein_coding
ENSG00000104760.17	55.9090614	0.315634841	0.282775073	1.116204612	0.264334561	0.492113776	FGL1	protein_coding
ENSG00000104763.20	10480.57161	0.053302067	0.063875624	0.83446648	0.404018169	0.625631632	ASAH1	protein_coding
ENSG00000104765.16	4726.035708	0.230447718	0.062124285	3.709462673	0.0002077	0.003491869	BNIP3L	protein_coding
ENSG00000104774.13	4922.877197	0.04211229	0.065176056	0.646131312	0.518194318	0.71911254	MAN2B1	protein_coding
ENSG00000104783.14	2416.638557	0.32806911	0.137433292	2.387115275	0.016981166	0.087133816	KCNN4	protein_coding
ENSG00000104804.8	18.55610411	-0.22343902	0.162829785	-1.372224494	0.169993562	0.379832404	TULP2	protein_coding
ENSG00000104805.16	15287.37707	-0.028615236	0.061584938	-0.464646658	0.642184514	0.806403842	NUCB1	protein_coding
ENSG00000104808.8	97.07165907	-0.331649039	0.139881768	-2.370923982	0.01774368	0.089632659	DHDH	protein_coding
ENSG00000104812.15	4650.826317	-0.084096525	0.049708238	-1.691802582	0.090683621	0.259350276	GYS1	protein_coding
ENSG00000104814.13	564.6205108	-0.65998679	0.126516814	-5.216593521	1.82244E-07	1.32803E-05	MAP4K1	protein_coding
ENSG00000104818.15	2.175597202	1.391258335	0.443329483	3.138203947	0.001699866	0.01702092	CGB2	protein_coding
ENSG00000104823.9	5246.711332	-0.134237201	0.084844292	-1.582159482	0.113613167	0.297299045	ECH1	protein_coding
ENSG00000104824.17	10946.48301	0.019518911	0.041535499	0.46993322	0.63840273	0.804292571	HNRNPL	protein_coding
ENSG00000104825.17	2260.147526	-0.084693329	0.073259658	-1.156070493	0.247652324	0.474220927	NFKBIB	protein_coding
ENSG00000104826.15	13.66577123	0.51053303	0.136989932	3.726792349	0.000193932	0.00331541	LHB	protein_coding
ENSG00000104827.12	2.39577387	0.808398083	0.344575139	2.346071994	0.018972442	0.093631044	CGB3	protein_coding
ENSG00000104833.12	75.72113637	0.105987255	0.185694932	0.570760082	0.568162286	0.75546358	TUBB4A	protein_coding
ENSG00000104835.15	160.8351013	-0.124542459	0.073826642	-1.686958189	0.091611392	0.260951695	SARS2	protein_coding
ENSG00000104848.1	10.82523443	0.828614073	0.171734097	4.824982843	1.40015E-06	6.74939E-05	KCNA7	protein_coding
ENSG00000104852.15	7663.727556	-0.084475164	0.04344309	-1.94450175	0.051834975	0.182116937	SNRNP70	protein_coding
ENSG00000104853.16	12252.97376	0.054101733	0.056129529	0.963872933	0.335109636	0.564631814	CLPTM1	protein_coding
ENSG00000104856.14	2329.691471	-0.018310401	0.072341524	-0.253110524	0.800182807	0.900334118	RELB	protein_coding
ENSG00000104859.15	2014.089041	0.000737289	0.051458833	0.014327754	0.988568498	0.994685849	CLASRP	protein_coding

ENSG00000104863.12	161.2320155	-0.186607883	0.067495367	-2.764750991	0.005696626	0.040835974	LIN7B	protein_coding
ENSG00000104866.11	2953.079892	0.061062201	0.060722313	1.005597421	0.314609265	0.543873651	PPP1R37	protein_coding
ENSG00000104870.13	11101.85204	-0.319922514	0.086077904	-3.716662433	0.000201872	0.003417627	FCGRT	protein_coding
ENSG00000104872.11	2441.386949	0.009297152	0.053277773	0.174503389	0.861469868	0.932525361	PIH1D1	protein_coding
ENSG00000104879.5	29.23354454	0.229049916	0.1707629	1.341333022	0.179812353	0.392832485	CKM	protein_coding
ENSG00000104880.18	2078.90035	0.038839305	0.056067372	0.692725611	0.488481763	0.695935195	ARHGEF18	protein_coding
ENSG00000104881.16	3787.981137	0.442339953	0.096597288	4.579217079	4.6672E-06	0.000176574	PPP1R13L	protein_coding
ENSG00000104883.8	183.196847	-0.034591551	0.070391802	-0.49141449	0.623133318	0.794333205	PEX11G	protein_coding
ENSG00000104884.16	1438.497322	0.019989003	0.053799965	0.371543058	0.710233092	0.84845083	ERCC2	protein_coding
ENSG00000104885.18	4354.577923	0.103754018	0.063907621	1.623499927	0.104482579	0.282779787	DOT1L	protein_coding
ENSG00000104886.12	2242.681163	-0.135622251	0.066569875	-2.037291658	0.041620823	0.158002493	PLEKHJ1	protein_coding
ENSG00000104888.10	69.60513779	0.322259096	0.156030403	2.065360913	0.038888864	0.151254911	SLC17A7	protein_coding
ENSG00000104889.7	10.90805133	-0.073245948	0.107176462	-0.683414498	0.494344962	0.701111574	RNASEH2A	protein_coding
ENSG00000104892.17	116.8918414	0.862195409	0.197552365	4.364389203	1.27478E-05	0.000393257	KLC3	protein_coding
ENSG00000104894.12	2219.461898	-0.632204969	0.145132192	-4.356062967	1.32423E-05	0.00040382	CD37	protein_coding
ENSG00000104897.10	7577.833868	-0.048393991	0.068489627	-0.706588611	0.479822169	0.689059178	SF3A2	protein_coding
ENSG00000104899.8	255.9808922	0.240947444	0.186330377	1.293119498	0.195969732	0.413040626	AMH	protein_coding
ENSG00000104901.7	22.70651654	-0.050470782	0.147586666	-0.341973859	0.732370565	0.860958534	DKKL1	protein_coding
ENSG00000104903.5	391.4853235	-0.253344407	0.111154594	-2.279207711	0.022654721	0.105777861	LYL1	protein_coding
ENSG00000104904.12	19153.83229	-0.092205297	0.056673085	-1.626968019	0.10374389	0.28160256	OAZ1	protein_coding
ENSG00000104907.12	1658.239513	-0.053707372	0.059295561	-0.905757045	0.365064461	0.592291572	TRMT1	protein_coding
ENSG00000104915.15	2041.921444	0.065296504	0.064210589	1.01691178	0.309195399	0.538584874	STX10	protein_coding
ENSG00000104918.8	20.20816493	1.486870385	0.206438285	7.202493412	5.91213E-13	3.48153E-10	RETN	protein_coding
ENSG00000104921.15	136.6030737	-1.122584424	0.232241072	-4.833703253	1.34016E-06	6.58402E-05	FCER2	protein_coding
ENSG00000104936.19	1832.524723	-0.186590426	0.128932804	-1.447191252	0.147843366	0.349266527	DMPK	protein_coding
ENSG00000104938.18	4.491417742	0.081199736	0.230328768	0.352538402	0.724434524	0.856215982	CLEC4M	protein_coding
ENSG00000104941.8	7.634439895	-0.040230081	0.228812157	-0.175821432	0.860434232	0.932158475	RSPH6A	protein_coding
ENSG00000104946.13	2621.424859	-0.031458485	0.052436804	-0.59993139	0.548551962	0.74166095	TBC1D17	protein_coding
ENSG00000104951.16	764.9844083	-0.055211029	0.124946356	-0.441877864	0.65857759	0.816810011	IL4I1	protein_coding
ENSG00000104953.20	118.7220598	-0.176237255	0.119286621	-1.477426834	0.139561258	0.336810345	TLE6	protein_coding
ENSG00000104957.14	1439.813862	-0.069170456	0.044580878	-1.551572307	0.120764594	0.308672573	CCDC130	protein_coding
ENSG00000104960.15	2198.957472	0.07863576	0.059546316	1.320581453	0.18664096	0.40154735	PTOV1	protein_coding
ENSG00000104964.15	10819.66799	-0.044289468	0.058371389	-0.758753022	0.448000313	0.663061157	TLE5	protein_coding
ENSG00000104967.8	405.6339367	0.087873317	0.088930689	0.988110158	0.323098701	0.552939851	NOVA2	protein_coding
ENSG00000104969.11	4995.792023	0.005577755	0.049025086	0.113773492	0.909417353	0.957123052	SGTA	protein_coding
ENSG00000104970.11	23.74610255	-0.491089707	0.158015539	-3.10785705	0.001884492	0.018322291	KIR3DX1	transcribed_unprocessed_pseudogene
ENSG00000104972.15	617.7086935	-0.300064403	0.10878516	-2.758321111	0.005809909	0.041392836	LILRB1	protein_coding
ENSG00000104973.19	6028.154142	0.013615113	0.063461668	0.214540735	0.830125405	0.916410163	MED25	protein_coding
ENSG00000104974.12	90.46071759	0.051483296	0.120663235	0.42666928	0.669620229	0.823891356	LILRA1	protein_coding
ENSG00000104976.12	1044.575984	0.03787092	0.065302661	0.579929195	0.561962367	0.750933347	SNAPC2	protein_coding
ENSG00000104979.9	3845.960847	-0.111162798	0.070728331	-1.571686998	0.116023159	0.301010835	C19orf53	protein_coding
ENSG00000104980.8	1766.396618	-0.041931542	0.054913359	-0.763594569	0.445108887	0.661019817	TIMM44	protein_coding

ENSG00000104983.8	674.6886095	-0.145750999	0.068227623	-2.136246178	0.032659353	0.134785173	CCDC61	protein_coding
ENSG00000104998.4	1195.547786	-0.523161219	0.092868018	-5.633384152	1.76707E-08	1.92509E-06	IL27RA	protein_coding
ENSG00000105011.9	2054.740888	-0.071436789	0.087302284	-0.818269419	0.413203364	0.633818966	ASF1B	protein_coding
ENSG00000105048.17	279.9262523	0.882958931	0.224228179	3.93776971	8.22425E-05	0.001704986	TNNT1	protein_coding
ENSG00000105053.11	2261.437548	-0.050033431	0.051986033	-0.962439855	0.335828702	0.565323537	VRK3	protein_coding
ENSG00000105058.12	3774.482386	-0.080791134	0.044303615	-1.8235788	0.068215774	0.216418225	FAM32A	protein_coding
ENSG00000105063.19	9217.526975	0.100642949	0.066431071	1.514998136	0.129772876	0.322357215	PPP6R1	protein_coding
ENSG00000105072.9	288.9501881	-0.04614027	0.053533108	-0.861901565	0.388741688	0.613219784	C19orf44	protein_coding
ENSG00000105085.11	399.5276236	-0.048732282	0.049376179	-0.986959374	0.323662555	0.553431739	MED26	protein_coding
ENSG00000105088.8	887.4679445	0.089404492	0.14278157	0.626162691	0.531208236	0.728903046	OLFM2	protein_coding
ENSG00000105122.13	972.0996243	-0.558280097	0.115832074	-4.819736706	1.43748E-06	6.91402E-05	RASAL3	protein_coding
ENSG00000105127.9	2086.095409	0.002356886	0.036433367	0.064690303	0.948420583	0.97580686	AKAP8	protein_coding
ENSG00000105131.8	532.9362981	0.931726168	0.208859517	4.461018488	8.15711E-06	0.000275339	EPHX3	protein_coding
ENSG00000105135.16	3078.707849	0.007559637	0.065637225	0.115173009	0.908307992	0.956556188	ILVBL	protein_coding
ENSG00000105136.21	291.1800969	0.091802991	0.082858089	1.107954486	0.267881473	0.495789754	ZNF419	protein_coding
ENSG00000105137.13	1356.313987	0.315445342	0.099014582	3.185847346	0.001443307	0.015126263	SYDE1	protein_coding
ENSG00000105141.6	9.13334755	0.186465289	0.290194357	0.64255312	0.52051411	0.720644227	CASP14	protein_coding
ENSG00000105143.12	10.32892024	-0.002004768	0.228583488	-0.008770395	0.993002327	0.996776522	SLC1A6	protein_coding
ENSG00000105146.13	63.21269283	0.330135941	0.117576108	2.80784886	0.004987363	0.037157515	AURKC	protein_coding
ENSG00000105171.10	2531.01796	0.242933652	0.100495878	2.417349419	0.015634001	0.082389995	POP4	protein_coding
ENSG00000105173.14	1351.721442	0.598394341	0.162980869	3.671561851	0.000241073	0.003924253	CCNE1	protein_coding
ENSG00000105176.18	6418.614731	0.253208192	0.098595279	2.568157362	0.010224073	0.061359928	URI1	protein_coding
ENSG00000105185.12	2937.982784	-0.172853717	0.074367101	-2.324330451	0.02010779	0.097576522	PDCD5	protein_coding
ENSG00000105186.16	2895.178998	0.025179907	0.058688007	0.429046888	0.667889103	0.822713321	ANKRD27	protein_coding
ENSG00000105193.9	44906.99651	-0.145429251	0.071408241	-2.036589184	0.041691228	0.158156892	RPS16	protein_coding
ENSG00000105197.11	3565.494154	-0.010923519	0.064052747	-0.170539424	0.864585933	0.934078621	TIMM50	protein_coding
ENSG00000105198.11	0.720602573	-0.11868719	0.490922913	-0.241763394	0.808963505		LGALS13	protein_coding
ENSG00000105202.9	8168.817143	-0.158136573	0.072513971	-2.180773864	0.029200147	0.125241613	FBL	protein_coding
ENSG00000105204.14	1809.720488	0.007374994	0.083615704	0.088201068	0.929716868	0.966720083	DYRK1B	protein_coding
ENSG00000105205.7	60.10391214	-0.165964323	0.278050637	-0.596885246	0.550583998	0.743041155	CLC	protein_coding
ENSG00000105219.10	167.0602202	0.403568399	0.187560823	2.151666812	0.031423606	0.131579367	CCNP	protein_coding
ENSG00000105220.17	21910.79843	-0.08817894	0.067448322	-1.307355574	0.191091978	0.406920422	GPI	protein_coding
ENSG00000105221.18	6556.00077	0.041113693	0.055880839	0.735738646	0.461889766	0.6744744	AKT2	protein_coding
ENSG00000105223.20	13631.08193	0.057559318	0.074401832	0.773627706	0.439150983	0.655715532	PLD3	protein_coding
ENSG00000105227.16	279.8088378	-0.043492198	0.087178583	-0.498886268	0.617859509	0.790621064	PRX	protein_coding
ENSG00000105229.7	1880.184965	-0.058886016	0.053202971	-1.106818179	0.268372547	0.496424917	PIAS4	protein_coding
ENSG00000105245.9	1678.322807	0.337642673	0.073050698	4.622032101	3.79999E-06	0.000149993	NUMBL	protein_coding
ENSG00000105246.6	205.1472212	-0.650790855	0.137737164	-4.7248748	2.30257E-06	0.000100704	EBI3	protein_coding
ENSG00000105248.16	955.1894816	-0.151634427	0.059325448	-2.5559761	0.010589039	0.062797844	YJU2	protein_coding
ENSG00000105251.11	125.7841019	-0.518840249	0.175575631	-2.955081218	0.003125868	0.026475399	SHD	protein_coding
ENSG00000105254.12	3168.995028	-0.072883828	0.064226333	-1.13479666	0.256460526	0.483444233	TBCB	protein_coding
ENSG00000105255.11	52.45177741	0.192358653	0.133960035	1.435940605	0.151019243	0.353370901	FSD1	protein_coding

ENSG00000105258.9	624.7520933	-0.198229606	0.08415808	-2.355443545	0.018500608	0.092166842	POLR2I	protein_coding
ENSG00000105261.7	9.236193195	-0.022778909	0.115701414	-0.196876672	0.843924049	0.92345516	OVOL3	protein_coding
ENSG00000105270.15	1663.446627	-0.192735103	0.139193859	-1.384652338	0.1661588	0.374486786	CLIP3	protein_coding
ENSG00000105278.12	21.05956254	0.359107245	0.205143079	1.750520887	0.080028473	0.240094849	ZFR2	protein_coding
ENSG00000105281.12	8860.791291	0.070004287	0.103454164	0.676669593	0.498615613	0.704271119	SLC1A5	protein_coding
ENSG00000105287.13	4546.33062	0.003598379	0.051560835	0.069788988	0.944361612	0.973930183	PRKD2	protein_coding
ENSG00000105289.15	5275.100065	0.035961952	0.101327669	0.354907527	0.72265887	0.855233249	TJP3	protein_coding
ENSG00000105290.13	739.8298415	-0.24871741	0.189258891	-1.314164998	0.188790685	0.404184355	APLP1	protein_coding
ENSG00000105298.14	986.2215056	0.030587886	0.048591948	0.629484656	0.529031811	0.727186805	CACTIN	protein_coding
ENSG00000105321.14	1592.740146	-0.01476152	0.057388768	-0.257219674	0.797009197	0.898440873	CCDC9	protein_coding
ENSG00000105323.17	17406.91594	0.020041651	0.045281303	0.442603233	0.658052746	0.81642081	HNRNPUL1	protein_coding
ENSG00000105325.15	3638.895891	0.082312834	0.049960896	1.647545199	0.099446034	0.274658374	FZR1	protein_coding
ENSG00000105327.18	1207.267568	-0.06888417	0.094540048	-0.72862424	0.466231546	0.677797234	BBC3	protein_coding
ENSG00000105329.11	6621.740737	0.176932157	0.086937156	2.035173051	0.041833464	0.158588677	TGFB1	protein_coding
ENSG00000105339.11	1829.29599	0.136871438	0.070397746	1.944258798	0.05186425	0.182147682	DENND3	protein_coding
ENSG00000105341.19	2967.439047	-0.079299267	0.05227718	-1.516900231	0.129291875	0.321639096	DMAC2	protein_coding
ENSG00000105352.10	28.01667077	0.173416255	0.13926507	1.245224339	0.213049298	0.433651702	CEACAM4	protein_coding
ENSG00000105355.9	3715.292907	0.314038523	0.071492391	4.392614609	1.11996E-05	0.000355716	PLIN3	protein_coding
ENSG00000105357.19	27859.53924	-0.002708044	0.096018136	-0.028203459	0.977499878	0.989940278	MYH14	protein_coding
ENSG00000105364.14	2901.301314	-0.050069684	0.072171419	-0.693760554	0.487832384	0.695328289	MRPL4	protein_coding
ENSG00000105366.15	154.7738228	-0.462160935	0.167065058	-2.766353066	0.005668712	0.040689422	SIGLEC8	protein_coding
ENSG00000105369.10	2551.952469	-1.387617196	0.198081286	-7.005291727	2.46472E-12	1.27863E-09	CD79A	protein_coding
ENSG00000105370.8	2.16608487	-0.557801348	0.303478076	-1.838028484	0.066058208	0.211928038	LIM2	protein_coding
ENSG00000105371.10	0						ICAM4	protein_coding
ENSG00000105372.8	34853.11444	-0.203056373	0.075616957	-2.685328524	0.007245855	0.048184435	RPS19	protein_coding
ENSG00000105373.19	13953.08209	-0.182134004	0.072953878	-2.496563722	0.012540314	0.070710919	NOP53	protein_coding
ENSG00000105374.10	943.8290657	-0.429613657	0.15017973	-2.860663394	0.004227556	0.032950139	NKG7	protein_coding
ENSG00000105376.5	69.90807632	0.397155198	0.142816758	2.780872528	0.005421302	0.039387142	ICAM5	protein_coding
ENSG00000105379.10	1268.736696	-0.356414755	0.081248367	-4.386731296	1.15067E-05	0.000363353	ETFB	protein_coding
ENSG00000105383.15	160.3306772	-0.093163455	0.110606475	-0.84229657	0.39962197	0.622407041	CD33	protein_coding
ENSG00000105388.16	65288.20758	0.692913932	0.212435174	3.261766493	0.001107203	0.012422397	CEACAM5	protein_coding
ENSG00000105392.16	21.154002	-0.150575042	0.212533849	-0.708475581	0.478649971	0.688205418	CRX	protein_coding
ENSG00000105393.16	752.590806	-0.104003289	0.052259014	-1.990150245	0.046574387	0.170005182	BABAM1	protein_coding
ENSG00000105397.14	4727.818128	0.073239091	0.046248231	1.583608496	0.113282839	0.296826786	TYK2	protein_coding
ENSG00000105398.4	60.50240937	-1.214729448	0.294571161	-4.123721559	3.728E-05	0.000910111	SULT2A1	protein_coding
ENSG00000105401.9	8161.483218	0.019303156	0.051996557	0.371239123	0.710459436	0.848559431	CDC37	protein_coding
ENSG00000105402.8	3352.988379	-0.056112735	0.055806011	-1.005496255	0.314657952	0.543874466	NAPA	protein_coding
ENSG00000105404.11	707.9045091	0.001647873	0.089995483	0.018310624	0.985391052	0.993295118	RABAC1	protein_coding
ENSG00000105409.19	56.87936095	-0.079274067	0.180637135	-0.438858087	0.660764368	0.818177731	ATP1A3	protein_coding
ENSG00000105419.18	500.769859	0.686797477	0.10224091	6.717442912	1.84941E-11	6.55218E-09	MEIS3	protein_coding
ENSG00000105426.17	2558.718938	0.44008435	0.136954682	3.213357472	0.001311929	0.014085932	PTPRS	protein_coding
ENSG00000105427.10	355.4067971	0.222973193	0.190996798	1.167418484	0.24304141	0.469060318	CNFN	protein_coding

ENSG00000105428.6	1.487425516	-0.161346159	0.374402957	-0.430942534	0.666510156	0.821789691	ZNRF4	protein_coding
ENSG00000105429.13	3553.663546	0.134567142	0.076506142	1.758906395	0.078593409	0.237548008	MEGF8	protein_coding
ENSG00000105438.9	19048.39642	-0.008022573	0.058208626	-0.137824471	0.890379144	0.947775953	KDELR1	protein_coding
ENSG00000105443.15	2830.12499	0.144132526	0.049233783	2.927512742	0.003416851	0.028301863	CYTH2	protein_coding
ENSG00000105447.13	2825.903798	0.011832388	0.05742951	0.206033241	0.836764946	0.919831527	GRWD1	protein_coding
ENSG00000105464.3	3423.747861	0.285036693	0.136539811	2.087572049	0.036836455	0.145631556	GRIN2D	protein_coding
ENSG00000105467.9	24.6622566	0.145113535	0.143095342	1.014103838	0.310533207	0.539978869	SYNGR4	protein_coding
ENSG00000105472.13	818.3503027	0.527150565	0.114372772	4.60905646	4.045E-06	0.000157552	CLEC11A	protein_coding
ENSG00000105479.16	42.67867032	0.677662436	0.146122643	4.637627818	3.52431E-06	0.000141426	CCDC114	protein_coding
ENSG00000105483.18	1540.67826	-0.210346054	0.060444708	-3.479974699	0.000501461	0.006803291	CARD8	protein_coding
ENSG00000105486.14	2130.540034	-0.109774102	0.072238599	-1.51960453	0.128610399	0.320511	LIG1	protein_coding
ENSG00000105492.16	122.7388389	-0.265525966	0.159136388	-1.668543378	0.095207908	0.267066303	SIGLEC6	protein_coding
ENSG00000105497.8	486.7518429	-0.028776375	0.081247936	-0.354179765	0.723204167	0.855433359	ZNF175	protein_coding
ENSG00000105499.14	454.9229971	-0.312690411	0.122781306	-2.546726535	0.010873861	0.064033815	PLA2G4C	protein_coding
ENSG00000105501.12	9.201250886	-0.100638686	0.140088995	-0.71839109	0.472516179	0.683422534	SIGLEC5	protein_coding
ENSG00000105507.3	4.362629467	-0.006471413	0.338982365	-0.019090708	0.984768744	0.9931098	CABP5	protein_coding
ENSG00000105509.11	77.45707676	1.012651747	0.200501045	5.050605828	4.40411E-07	2.6917E-05	HAS1	protein_coding
ENSG00000105514.8	2428.657813	0.110846273	0.089364551	1.240383027	0.214833756	0.435899361	RAB3D	protein_coding
ENSG00000105516.11	992.7580737	-0.176826967	0.083950009	-2.106336479	0.035175135	0.141274364	DBP	protein_coding
ENSG00000105518.14	1096.437503	-0.063557917	0.080192837	-0.792563517	0.428032171	0.646258596	TMEM205	protein_coding
ENSG00000105519.18	790.4272557	0.545512474	0.159515227	3.419814416	0.000626639	0.00804805	CAPS	protein_coding
ENSG00000105520.10	2058.697623	0.226582994	0.080575916	2.812043646	0.004922782	0.036798426	PLPPR2	protein_coding
ENSG00000105523.3	2622.714416	0.059446597	0.125032832	0.475447894	0.634467764	0.801699242	FAM83E	protein_coding
ENSG00000105538.10	874.6780675	0.006509168	0.094486006	0.068890291	0.945076947	0.974137666	RASIP1	protein_coding
ENSG00000105549.11	5.405252298	0.894562975	0.279931103	3.195654095	0.001395143	0.014803051	THEG	protein_coding
ENSG00000105550.10	2.136808523	-0.143866968	0.285390992	-0.504104798	0.614187736	0.788319265	FGF21	protein_coding
ENSG00000105552.15	2299.022948	-0.222192691	0.079612121	-2.790940491	0.005255513	0.038500353	BCAT2	protein_coding
ENSG00000105556.11	1252.177879	0.142396929	0.055148196	2.582077728	0.009820746	0.059670756	MIER2	protein_coding
ENSG00000105559.12	2524.333013	0.07204812	0.109209838	0.659721881	0.509432322	0.712875856	PLEKHA4	protein_coding
ENSG00000105568.18	15709.86378	0.014593873	0.051033824	0.285964719	0.774905144	0.886049147	PPP2R1A	protein_coding
ENSG00000105576.15	5273.070907	-0.022256107	0.047700776	-0.466577456	0.640802219	0.805545602	TNPO2	protein_coding
ENSG00000105583.11	0.021509542	-0.13844802	2.312445681	-0.05987082	0.95225852		WDR83OS	protein_coding
ENSG00000105605.7	6.823761744	0.115741395	0.209289661	0.553020127	0.580249628	0.764190584	CACNG7	protein_coding
ENSG00000105607.13	997.4732125	-0.030855353	0.050601819	-0.609767662	0.542015726	0.737185371	GCDH	protein_coding
ENSG00000105609.17	299.2428209	-0.118934838	0.133395789	-0.891593646	0.372610777	0.599094559	LILRB5	protein_coding
ENSG00000105610.6	8.017928905	-0.242554807	0.149648218	-1.620833247	0.105053406	0.283743168	KLF1	protein_coding
ENSG00000105612.9	3102.521095	-0.019247886	0.057268693	-0.336097872	0.736797063	0.863566585	DNASE2	protein_coding
ENSG00000105613.10	133.4002197	0.213066098	0.129339633	1.647338042	0.099488583	0.274667363	MAST1	protein_coding
ENSG00000105617.4	442.7370268	-0.05105417	0.061263973	-0.833347359	0.404648854	0.626296683	LENG1	protein_coding
ENSG00000105618.14	1710.985185	-0.026967748	0.054037399	-0.499057107	0.617739154	0.790555905	PRPF31	protein_coding
ENSG00000105619.13	550.0473484	0.102947724	0.071746167	1.434888132	0.151318978	0.353756819	TFPT	protein_coding
ENSG00000105639.20	1904.361489	-0.2724632	0.108046822	-2.521714157	0.011678456	0.067289712	JAK3	protein_coding

ENSG00000105640.13	50376.11468	-0.193595596	0.083502554	-2.318439208	0.020425463	0.098592449	RPL18A	protein_coding
ENSG00000105641.4	512.7052336	-0.584631439	0.261365608	-2.236833846	0.025297202	0.113976029	SLC5A5	protein_coding
ENSG00000105642.15	39.12322782	0.841882532	0.152197288	5.531521247	3.17465E-08	3.12539E-06	KCNN1	protein_coding
ENSG00000105643.10	1647.748988	0.182675824	0.061702259	2.960601866	0.003070386	0.026137565	ARRDC2	protein_coding
ENSG00000105647.19	10.71754059	-0.206502629	0.109020641	-1.89416084	0.058203664	0.196083577	PIK3R2	protein_coding
ENSG00000105649.10	235.0392147	-0.033026113	0.091701919	-0.360146371	0.718737677	0.853045859	RAB3A	protein_coding
ENSG00000105650.22	13.00492515	0.154713527	0.164973262	0.937809713	0.348342209	0.5775046	PDE4C	protein_coding
ENSG00000105655.19	2188.508732	0.182545997	0.129834917	1.405985397	0.159728461	0.365572333	ISYNA1	protein_coding
ENSG00000105656.13	1496.837005	0.042898217	0.049710332	0.862963805	0.388157368	0.612608511	ELL	protein_coding
ENSG00000105662.16	1452.389515	0.010474399	0.061739835	0.169653827	0.865282388	0.934622512	CRTC1	protein_coding
ENSG00000105664.11	1491.656572	0.872245736	0.230462987	3.784754095	0.000153861	0.002782068	COMP	protein_coding
ENSG00000105668.7	25.04560425	0.567111665	0.229790655	2.46794921	0.01358896	0.074900848	UPK1A	protein_coding
ENSG00000105669.14	6369.387777	-0.11211139	0.06748245	-1.661341442	0.096644891	0.269534142	COPE	protein_coding
ENSG00000105671.12	2242.816567	-0.029288246	0.055014841	-0.532369924	0.594469812	0.77430688	DDX49	protein_coding
ENSG00000105672.14	95.9215497	-0.132518834	0.094000156	-1.409772494	0.158606871	0.364135057	ETV2	protein_coding
ENSG00000105675.9	350.5635862	-2.245267567	0.342301886	-6.559319891	5.40537E-11	1.62448E-08	ATP4A	protein_coding
ENSG00000105676.14	1715.050349	-0.054581942	0.056648259	-0.963523738	0.335284758	0.564769658	ARMC6	protein_coding
ENSG00000105677.12	3400.983301	-0.067660992	0.070286786	-0.962641716	0.335727355	0.565213682	TMEM147	protein_coding
ENSG00000105679.9	1.89348595	0.320509824	0.194643354	1.64665177	0.099629643	0.274847366	GAPDHS	protein_coding
ENSG00000105694.3	2.826441324	-0.383344004	0.21810519	-1.757610649	0.07881378	0.237992384	ELOCP28	processed_pseudogene
ENSG00000105695.15	9.680977774	0.195397342	0.21975915	0.889143148	0.373926152	0.599897302	MAG	protein_coding
ENSG00000105696.9	75.86180365	0.166464484	0.190322374	0.874644849	0.381767177	0.607074452	TMEM59L	protein_coding
ENSG00000105697.9	67.6331431	0.37167711	0.160517261	2.315496214	0.020585792	0.099079637	HAMP	protein_coding
ENSG00000105698.16	5790.735202	-0.004508225	0.051728779	-0.087151201	0.930551328	0.967010903	USF2	protein_coding
ENSG00000105699.16	22359.35325	0.086586237	0.08910442	0.971738968	0.331180425	0.560728095	LSR	protein_coding
ENSG00000105700.11	2231.88312	-0.075684982	0.051387432	-1.472830611	0.140796716	0.338651251	KXD1	protein_coding
ENSG00000105701.16	12043.2281	-0.030773926	0.067097365	-0.458645816	0.64648853	0.809238012	FKBP8	protein_coding
ENSG00000105705.16	1241.606461	-0.042683923	0.038140379	-1.119126873	0.263086017	0.490711782	SUGP1	protein_coding
ENSG00000105707.14	530.3082797	0.195265381	0.252243691	0.774114035	0.438863358	0.655504664	HPN	protein_coding
ENSG00000105708.9	417.9456667	-0.133786821	0.069928093	-1.913205637	0.055721732	0.190655302	ZNF14	protein_coding
ENSG00000105711.13	344.1056324	0.458107881	0.110187586	4.157527174	3.21711E-05	0.000807093	SCN1B	protein_coding
ENSG00000105717.14	210.44611	-0.547129047	0.089621832	-6.104863454	1.02889E-09	1.86041E-07	PBX4	protein_coding
ENSG00000105722.10	4363.402368	0.013547124	0.068179134	0.198698977	0.842498224	0.922520419	ERF	protein_coding
ENSG00000105723.13	4020.029184	-0.001352868	0.0534991	-0.025287678	0.979825502	0.991136584	GSK3A	protein_coding
ENSG00000105726.17	7216.789591	0.009624186	0.052869641	0.182036142	0.855554362	0.929459824	ATP13A1	protein_coding
ENSG00000105732.13	2477.778854	0.028925936	0.0528097	0.547739072	0.583871079	0.766903278	ZNF574	protein_coding
ENSG00000105737.9	273.0087076	-0.332222929	0.182771654	-1.817693949	0.069110923	0.218023891	GRIK5	protein_coding
ENSG00000105738.11	9526.567583	0.158494066	0.076274115	2.077953535	0.037713639	0.148125033	SIPA1L3	protein_coding
ENSG00000105750.15	222.7012174	-0.078339031	0.110711137	-0.707598472	0.479194642	0.688589112	ZNF85	protein_coding
ENSG00000105755.8	2716.612482	-0.074145219	0.102642623	-0.722362859	0.470071415	0.681401839	ETHE1	protein_coding
ENSG00000105767.3	749.0484344	0.090801553	0.109359305	0.830304773	0.40636649	0.627705363	CADM4	protein_coding
ENSG00000105771.14	2043.325169	-0.010813594	0.048828083	-0.221462595	0.824732258	0.913441546	SMG9	protein_coding

ENSG00000105778.19	5094.891308	-0.060579771	0.05289506	-1.145282209	0.252092273	0.478824958	AVL9	protein_coding
ENSG00000105784.15	131.1960632	-0.440035822	0.134412581	-3.273769609	0.001061231	0.012007828	RUNDC3B	protein_coding
ENSG00000105792.19	311.7929146	-0.046839786	0.103619357	-0.452037022	0.651242312	0.81217979	CFAP69	protein_coding
ENSG00000105793.16	1605.021093	0.045267878	0.066320789	0.682559406	0.494885293	0.7014966	GTPBP10	protein_coding
ENSG00000105808.18	46.8576467	-0.184208631	0.111943145	-1.645555259	0.099855357	0.275161053	RASA4	protein_coding
ENSG00000105810.10	7866.457133	0.046233042	0.124300584	0.371945495	0.709933431	0.848349717	CDK6	protein_coding
ENSG00000105819.14	3780.793372	-0.138800413	0.053602694	-2.589429788	0.009613503	0.058730913	PMPCB	protein_coding
ENSG00000105821.15	2217.42409	-0.083370351	0.067339352	-1.238062873	0.215692749	0.436899801	DNAJC2	protein_coding
ENSG00000105825.14	265.5876702	0.533872818	0.189819944	2.812522264	0.004915462	0.036760098	TFPI2	protein_coding
ENSG00000105829.13	1483.075571	0.036959734	0.067055857	0.551178304	0.581511452	0.76519309	BET1	protein_coding
ENSG00000105835.12	9844.298764	0.107975323	0.087276099	1.237169446	0.216024182	0.437256063	NAMPT	protein_coding
ENSG00000105849.6	2212.275428	-0.134709553	0.058608272	-2.298473399	0.02153486	0.102158132	POLR1F	protein_coding
ENSG00000105851.11	981.114185	-0.607541797	0.125781464	-4.830137737	1.36439E-06	6.66433E-05	PIK3CG	protein_coding
ENSG00000105852.11	386.2501394	0.71721986	0.226475389	3.166877697	0.001540851	0.015817589	PON3	protein_coding
ENSG00000105854.13	5204.989825	0.378501644	0.102167893	3.704702467	0.000211639	0.003537026	PON2	protein_coding
ENSG00000105855.10	4041.731298	0.205063406	0.112829687	1.817459665	0.069146759	0.218095564	ITGB8	protein_coding
ENSG00000105856.14	2023.331288	0.156492849	0.052956856	2.955100813	0.003125669	0.026475399	HBP1	protein_coding
ENSG00000105865.11	260.5463511	-0.208218002	0.068297323	-3.048699311	0.002298344	0.021121739	DUS4L	protein_coding
ENSG00000105866.15	906.9403237	0.017742453	0.072173979	0.24582894	0.805814654	0.903488529	SP4	protein_coding
ENSG00000105875.14	580.6158865	-0.125565301	0.067113727	-1.870933211	0.061354339	0.202686531	WDR91	protein_coding
ENSG00000105877.18	78.09546582	0.535508892	0.148046417	3.617168886	0.000297843	0.004621143	DNAH11	protein_coding
ENSG00000105879.12	2246.966813	-0.10040143	0.046414491	-2.163148376	0.030529769	0.129039356	CBLL1	protein_coding
ENSG00000105880.7	63.71743882	0.818924493	0.185629443	4.411608846	1.02605E-05	0.000332669	DLX5	protein_coding
ENSG00000105887.11	8994.841128	0.022346583	0.046339416	0.482237029	0.629637575	0.798699287	MTPN	protein_coding
ENSG00000105889.16	44.23376715	0.019753095	0.134477704	0.146887511	0.883220809	0.943960371	STEAP1B	protein_coding
ENSG00000105894.12	541.8686739	-0.173934046	0.145004265	-1.199509859	0.230329754	0.454174281	PTN	protein_coding
ENSG00000105926.16	792.1382413	0.083988015	0.119689235	0.701717371	0.482855438	0.691082583	MPP6	protein_coding
ENSG00000105928.16	550.5968995	0.329106069	0.127166433	2.58799482	0.009653643	0.058918321	GSDME	protein_coding
ENSG00000105929.16	65.89460044	0.611367323	0.26996453	2.264620918	0.023535952	0.108497428	ATP6V0A4	protein_coding
ENSG00000105939.13	5412.576333	-0.091692387	0.038449761	-2.38473231	0.017091553	0.087479282	ZC3HAV1	protein_coding
ENSG00000105948.13	539.4394091	-0.124250523	0.06844116	-1.815435683	0.069456982	0.218690936	TTC26	protein_coding
ENSG00000105953.15	11629.73886	0.004327784	0.054179748	0.079878255	0.936334084	0.969828637	OGDH	protein_coding
ENSG00000105954.2	0.532085207	-0.309642499	0.625725898	-0.494853258	0.620703707		NPVF	protein_coding
ENSG00000105963.15	1958.899403	0.002263268	0.099464899	0.022754438	0.981846152	0.992068024	ADAP1	protein_coding
ENSG00000105967.16	505.7677037	-0.46462064	0.124383989	-3.735373355	0.000187437	0.003245108	TFEC	protein_coding
ENSG00000105968.19	11367.25114	-0.084052504	0.040240388	-2.08875977	0.036729354	0.145273694	H2AZ2	protein_coding
ENSG00000105971.15	3896.254968	0.422325954	0.12789449	3.302143474	0.00095949	0.011102413	CAV2	protein_coding
ENSG00000105974.13	5601.684823	0.02106566	0.132030741	0.159551177	0.873234641	0.938725432	CAV1	protein_coding
ENSG00000105976.16	9146.594365	0.432519068	0.113064773	3.8254096	0.000130555	0.002428164	MET	protein_coding
ENSG00000105982.17	140.2130526	-0.473493017	0.092894975	-5.09707888	3.44935E-07	2.18656E-05	RNF32	protein_coding
ENSG00000105983.22	3446.616209	-0.098915712	0.056607301	-1.747402019	0.080567628	0.241066706	LMBR1	protein_coding
ENSG00000105988.6	26.74050436	-0.141555895	0.078250451	-1.809010607	0.070449355	0.220686619	NHP2P1	processed_pseudogene

ENSG00000105989.10	478.1369603	0.712604591	0.161893106	4.401698189	1.07407E-05	0.000343737	WNT2	protein_coding
ENSG00000105991.10	213.1383107	0.216276461	0.125475611	1.723653372	0.084770437	0.248958171	HOXA1	protein_coding
ENSG00000105993.15	4445.409349	-0.032941304	0.042553523	-0.774114598	0.438863025	0.655504664	DNAJB6	protein_coding
ENSG00000105996.7	152.3077577	0.088837283	0.138709924	0.640453695	0.521877684	0.721621061	HOXA2	protein_coding
ENSG00000105997.22	742.9474668	-0.046495542	0.114243179	-0.406987467	0.684017193	0.833144681	HOXA3	protein_coding
ENSG00000106003.13	4254.340012	0.169080823	0.104926931	1.611414932	0.107089315	0.286753783	LFNG	protein_coding
ENSG00000106004.5	510.1244196	0.176657628	0.116749633	1.51313219	0.130246085	0.323017109	HOXA5	protein_coding
ENSG00000106006.6	53.28507273	0.430943562	0.150996116	2.854004291	0.004317195	0.033487077	HOXA6	protein_coding
ENSG00000106009.16	4428.608342	-0.017017987	0.061938797	-0.274754889	0.783504558	0.890154816	BRAT1	protein_coding
ENSG00000106012.18	4189.306589	-0.086641358	0.074216517	-1.167413417	0.243043455	0.469060318	IQCE	protein_coding
ENSG00000106013.15	8.016120505	-0.195764896	0.219659269	-0.89122074	0.37281076	0.599195017	ANKRD7	protein_coding
ENSG00000106018.14	155.4094454	-0.160868778	0.198152485	-0.81184335	0.416881524	0.637240289	VIPR2	protein_coding
ENSG00000106025.9	1389.375127	-0.159345306	0.104698764	-1.521940666	0.128023949	0.319524605	TSPAN12	protein_coding
ENSG00000106028.11	3938.455104	-0.303203275	0.058766888	-5.159423707	2.47711E-07	1.68664E-05	SSBP1	protein_coding
ENSG00000106031.9	1463.155382	0.176623336	0.198783324	0.888521894	0.374260084	0.600041637	HOXA13	protein_coding
ENSG00000106034.18	1938.786445	-0.390451449	0.16371017	-2.385016454	0.017078357	0.087450596	CPED1	protein_coding
ENSG00000106038.13	91.58403455	0.037930961	0.268748484	0.141139258	0.887759931	0.946351782	EVX1	protein_coding
ENSG00000106049.9	2940.832563	-0.252973213	0.05535141	-4.57031202	4.86999E-06	0.000182319	HIBADH	protein_coding
ENSG00000106052.13	9159.852667	-0.0064941	0.048971656	-0.132609355	0.894502334	0.949798445	TAX1BP1	protein_coding
ENSG00000106066.15	2370.810424	-0.290200276	0.130394343	-2.225558792	0.026043749	0.116009424	CPVL	protein_coding
ENSG00000106069.23	80.31579944	-0.358437054	0.12826747	-2.79445018	0.005198805	0.038229552	CHN2	protein_coding
ENSG00000106070.20	3160.238401	0.146241257	0.084177979	1.737286393	0.082336632	0.244479655	GRB10	protein_coding
ENSG00000106077.18	3234.395956	-0.014586015	0.091076731	-0.160150846	0.872762249	0.938514473	ABHD11	protein_coding
ENSG00000106078.19	3387.179587	0.147226448	0.097358168	1.512214653	0.130479265	0.323466544	COBL	protein_coding
ENSG00000106080.11	1070.656442	0.262868043	0.057239574	4.592417901	4.3814E-06	0.000168535	FKBP14	protein_coding
ENSG00000106086.20	1163.873573	-0.025148352	0.054706874	-0.459692732	0.645736787	0.808785697	PLEKHA8	protein_coding
ENSG00000106089.12	623.1720847	0.462746995	0.106181462	4.358077072	1.3121E-05	0.000400964	STX1A	protein_coding
ENSG00000106100.11	846.595928	0.098011031	0.063677296	1.539183309	0.123759551	0.313218979	NOD1	protein_coding
ENSG00000106105.15	9382.680383	-0.059199501	0.058932632	-1.004528363	0.315124007	0.544322136	GARS1	protein_coding
ENSG00000106113.19	24.09283482	-0.015059278	0.151553925	-0.099365809	0.920847829	0.96273785	CRHR2	protein_coding
ENSG00000106123.12	623.8158254	0.578866283	0.167496641	3.455987406	0.000548281	0.007299859	EPHB6	protein_coding
ENSG00000106125.14	61.7800191	-0.126727579	0.089132021	-1.421796318	0.155085389	0.359379046	MINDY4	protein_coding
ENSG00000106128.19	17.18540009	-0.218398901	0.205601137	-1.062245591	0.288124208	0.51790573	GHRHR	protein_coding
ENSG00000106133.18	20.926494	-0.082519799	0.123279795	-0.669370022	0.50325947	0.707847715	NSUN5P2	transcribed_unprocessed_pseudogene
ENSG00000106144.20	3575.737982	-0.144713177	0.054721075	-2.644560199	0.008179716	0.052557869	CASP2	protein_coding
ENSG00000106153.13	18415.17097	-0.227060121	0.069406883	-3.271435215	0.001070031	0.012092516	CHCHD2	protein_coding
ENSG00000106178.7	538.0677662	-0.110146658	0.1813835	-0.607258419	0.543679428	0.73835352	CCL24	protein_coding
ENSG00000106211.10	42252.53463	-0.161454408	0.130177581	-1.240262774	0.214878217	0.435948975	HSPB1	protein_coding
ENSG00000106236.4	165.0054847	0.685541729	0.155649524	4.404393359	1.0608E-05	0.000341278	NPTX2	protein_coding
ENSG00000106244.13	5981.387379	-0.001424738	0.069094774	-0.020620057	0.983548741	0.99267258	PDAP1	protein_coding
ENSG00000106245.11	2127.168459	-0.028727432	0.075240935	-0.38180589	0.70260535	0.843687884	BUD31	protein_coding
ENSG00000106246.18	217.7679538	-0.036505936	0.078889698	-0.462746551	0.643546047	0.807341262	PTCD1	protein_coding

ENSG00000106258.15	5099.874635	-0.227778526	0.137411863	-1.657633634	0.097391437	0.271166483	CYP3A5	protein_coding
ENSG00000106261.17	11214.20828	0.158152108	0.081455408	1.941578975	0.05218809	0.182695654	ZKSCAN1	protein_coding
ENSG00000106263.18	13250.67963	-0.021990668	0.056069955	-0.39220057	0.694910023	0.839118102	EIF3B	protein_coding
ENSG00000106266.11	2551.745152	-0.037843388	0.067975336	-0.556722338	0.577717153	0.762306748	SNX8	protein_coding
ENSG00000106268.15	807.1977618	-0.199348657	0.075032173	-2.656842389	0.007887631	0.051240205	NUDT1	protein_coding
ENSG00000106278.12	522.9114068	-0.621396993	0.212250191	-2.927662825	0.003415202	0.028293584	PTPRZ1	protein_coding
ENSG00000106290.15	2190.348305	0.212667004	0.070282174	3.025902473	0.002478922	0.022307815	TAF6	protein_coding
ENSG00000106299.8	6438.452409	0.070883031	0.054030786	1.31190079	0.189553611	0.405049659	WASL	protein_coding
ENSG00000106302.10	14.43452757	-0.196301065	0.21228709	-0.924696198	0.355123958	0.583088069	HYAL4	protein_coding
ENSG00000106304.15	2.599511365	-0.570138935	0.303125734	-1.880866161	0.059990127	0.199922743	SPAM1	protein_coding
ENSG00000106305.10	1286.845457	-0.063937997	0.066154073	-0.96650128	0.333793407	0.563310506	AIMP2	protein_coding
ENSG00000106327.13	608.6694231	-0.157596025	0.158794354	-0.992453581	0.320976313	0.55071993	TFR2	protein_coding
ENSG00000106328.10	5.533530952	-0.137987072	0.143074003	-0.964445454	0.334822643	0.564366469	FSCN3	protein_coding
ENSG00000106330.12	1566.078268	0.021621719	0.077863558	0.27768727	0.781252433	0.889172516	MOSPD3	protein_coding
ENSG00000106331.17	22.98876391	-0.082843613	0.330840119	-0.250403769	0.802275115	0.901631017	PAX4	protein_coding
ENSG00000106333.13	2755.993522	0.415893983	0.108627174	3.828636671	0.000128855	0.002402703	PCOLCE	protein_coding
ENSG00000106336.13	21.22097349	0.033502653	0.090959076	0.368326659	0.712629676	0.849754095	FBXO24	protein_coding
ENSG00000106341.11	2.92904031	-0.320438819	0.238150722	-1.345529485	0.178454306	0.391109921	PPP1R17	protein_coding
ENSG00000106344.8	2206.364943	-0.140432769	0.049494103	-2.837363665	0.004548777	0.034824675	RBM28	protein_coding
ENSG00000106346.11	2065.830124	0.009542077	0.041385628	0.230564978	0.817652778	0.910099918	USP42	protein_coding
ENSG00000106348.18	4266.615134	0.044924529	0.082453496	0.544846875	0.585858836	0.76814209	IMPDH1	protein_coding
ENSG00000106351.13	2707.733753	-0.143754063	0.096661339	-1.487192969	0.136963853	0.333099331	AGFG2	protein_coding
ENSG00000106355.10	2490.764409	-0.265009965	0.065166298	-4.066672119	4.76893E-05	0.001105106	LSM5	protein_coding
ENSG00000106366.9	7374.679962	0.854513434	0.145960016	5.854435038	4.78634E-09	6.70657E-07	SERPINE1	protein_coding
ENSG00000106367.15	3164.749653	-0.236627142	0.085103835	-2.780452167	0.005428326	0.039425024	AP1S1	protein_coding
ENSG00000106384.12	471.5494441	-0.586196339	0.198709395	-2.950018237	0.003177552	0.026845322	MOGAT3	protein_coding
ENSG00000106392.11	5049.997318	-0.017853545	0.074025536	-0.241180895	0.809414918	0.905541771	C1GALT1	protein_coding
ENSG00000106397.12	9574.511377	-0.086880412	0.083798962	-1.036771935	0.299842158	0.529532795	PLOD3	protein_coding
ENSG00000106399.11	1810.261742	-0.249331762	0.070014585	-3.561140321	0.000369248	0.005434976	RPA3	protein_coding
ENSG00000106400.12	5131.799754	-0.227071477	0.074758612	-3.037395574	0.002386321	0.021700479	ZNHIT1	protein_coding
ENSG00000106404.14	2339.240645	-0.461042387	0.170578902	-2.702810147	0.006875601	0.046418107	CLDN15	protein_coding
ENSG00000106410.15	2.556007243	0.054614115	0.365128701	0.149574971	0.881099956	0.9428503	NOBOX	protein_coding
ENSG00000106415.13	1003.623757	-0.400427033	0.081591219	-4.907722143	9.21403E-07	4.87281E-05	GLCCI1	protein_coding
ENSG00000106436.7	3.10373778	-0.144172144	0.238971892	-0.60330168	0.546308016	0.740241981	MYL10	protein_coding
ENSG00000106443.17	5088.899898	-0.220231465	0.068971489	-3.193079772	0.001407641	0.014889337	PHF14	protein_coding
ENSG00000106459.15	886.2911087	-0.067054003	0.02836329	-2.364112299	0.018073333	0.090688613	NRF1	protein_coding
ENSG00000106460.19	6687.80161	0.104405517	0.061742291	1.690988711	0.090838959	0.25964695	TMEM106B	protein_coding
ENSG00000106462.11	1734.807516	-0.184300567	0.078113684	-2.359389014	0.018305055	0.091518977	EZH2	protein_coding
ENSG00000106477.20	895.152031	-0.133441696	0.079595679	-1.676494226	0.093641426	0.264476793	CEP41	protein_coding
ENSG00000106479.11	625.3637944	-0.289325593	0.078875988	-3.66810739	0.000244353	0.00396284	ZNF862	protein_coding
ENSG00000106483.12	6086.565914	0.787247123	0.196521405	4.00591032	6.17791E-05	0.001358984	SFRP4	protein_coding
ENSG00000106484.16	3000.751859	-0.021650259	0.106979321	-0.202377981	0.839621241	0.921243725	MEST	protein_coding

ENSG00000106511.6	203.4596937	0.313066087	0.192536388	1.626009969	0.103947535	0.281849314	MEOX2	protein_coding
ENSG00000106524.9	1572.211054	-0.335850399	0.059604615	-5.634637486	1.75427E-08	1.92088E-06	ANKMY2	protein_coding
ENSG00000106526.10	275.9087436	-0.274289478	0.072245114	-3.796650917	0.000146664	0.002673583	ACTR3C	protein_coding
ENSG00000106536.21	171.2085289	0.674394117	0.274083093	2.460546213	0.01387257	0.075955067	POU6F2	protein_coding
ENSG00000106537.8	8435.97234	-0.018938447	0.094992656	-0.199367483	0.841975294	0.922310075	TSPAN13	protein_coding
ENSG00000106538.10	2922.576703	0.209499051	0.112647476	1.859775811	0.062917251	0.205883085	RARRES2	protein_coding
ENSG00000106540.4	0						AC004837.1	processed_pseudogene
ENSG00000106541.12	88445.72931	0.056720738	0.162156886	0.349789263	0.726496857	0.857351285	AGR2	protein_coding
ENSG00000106546.14	6663.468196	0.055284665	0.069540901	0.79499494	0.426616445	0.645217951	AHR	protein_coding
ENSG00000106554.13	3088.216322	-0.173131356	0.057822017	-2.99421166	0.00275155	0.02409168	CHCHD3	protein_coding
ENSG00000106560.11	546.5546422	-0.290122579	0.089059044	-3.257643093	0.001123416	0.01255762	GIMAP2	protein_coding
ENSG00000106565.18	11413.41192	-0.086046129	0.107776824	-0.798373209	0.424653945	0.64359006	TMEM176B	protein_coding
ENSG00000106571.15	796.5899632	0.180365691	0.131603606	1.370522406	0.170523886	0.380584859	GLI3	protein_coding
ENSG00000106588.12	70.07108897	-0.107024179	0.092413297	-1.158103674	0.246821731	0.473281124	PSMA2	protein_coding
ENSG00000106591.4	2010.505404	-0.151472109	0.050616408	-2.99254953	0.002766578	0.024179534	MRPL32	protein_coding
ENSG00000106603.19	2141.126323	-0.156641797	0.038643322	-4.053528277	5.04509E-05	0.001158935	COA1	protein_coding
ENSG00000106605.11	1323.60835	-0.082739958	0.085174517	-0.971416817	0.331340757	0.560796838	BLVRA	protein_coding
ENSG00000106608.17	2623.997213	-0.107549229	0.045209686	-2.378897955	0.017364482	0.088398603	URGCP	protein_coding
ENSG00000106609.16	11287.36554	-0.066399326	0.047986163	-1.383718187	0.166444763	0.374859859	TMEM248	protein_coding
ENSG00000106610.15	514.7872157	-0.121682843	0.057907267	-2.101339758	0.035611152	0.142303284	STAG3L4	transcribed_unprocessed_pseudogene
ENSG00000106615.10	5196.712461	-0.106281324	0.049967825	-2.126995182	0.033420483	0.136645188	RHEB	protein_coding
ENSG00000106617.15	1567.226956	-0.126256183	0.068680555	-1.838310469	0.066016669	0.211825963	PRKAG2	protein_coding
ENSG00000106624.11	20920.20419	0.592771359	0.124358731	4.76662438	1.87338E-06	8.53936E-05	AEBP1	protein_coding
ENSG00000106628.11	5709.211593	-0.083416995	0.068814395	-1.212202703	0.225434797	0.448492572	POLD2	protein_coding
ENSG00000106631.8	1.285024247	-0.031297214	0.321038458	-0.09748743	0.922339316	0.963291021	MYL7	protein_coding
ENSG00000106633.17	22.90758251	0.057856925	0.146566861	0.39474766	0.693029125	0.83789169	GCK	protein_coding
ENSG00000106635.8	3630.570261	-0.104135384	0.051774438	-2.011328139	0.044290808	0.164598367	BCL7B	protein_coding
ENSG00000106636.9	5642.330544	-0.00196918	0.045669346	-0.043118198	0.965607313	0.984349774	YKT6	protein_coding
ENSG00000106638.17	3166.191847	-0.000399728	0.05807713	-0.006882714	0.994508432	0.997553032	TBL2	protein_coding
ENSG00000106648.14	7.525323291	-0.094647139	0.310897857	-0.304431623	0.760799078	0.877538394	GALNTL5	protein_coding
ENSG00000106665.16	5914.859311	-0.078771702	0.079943939	-0.985336769	0.324458678	0.554272445	CLIP2	protein_coding
ENSG00000106682.15	11160.68993	-0.021975134	0.046947065	-0.468083228	0.639725073	0.804960183	EIF4H	protein_coding
ENSG00000106683.15	3670.511711	0.004166366	0.06665558	0.062505888	0.950159973	0.976743204	LIMK1	protein_coding
ENSG00000106686.16	263.8423937	0.022977845	0.125061775	0.183731956	0.854223738	0.928835187	SPATA6L	protein_coding
ENSG00000106688.12	1576.152634	0.308249345	0.138522697	2.225262371	0.02606363	0.116073051	SLC1A1	protein_coding
ENSG00000106689.11	29.2484244	-0.012060068	0.155405722	-0.077603759	0.938143252	0.970696818	LHX2	protein_coding
ENSG00000106692.15	1936.534181	-0.037239221	0.055528156	-0.670636734	0.502451976	0.707211421	FKTN	protein_coding
ENSG00000106701.13	553.8373167	-0.38013302	0.10246165	-3.710002903	0.000207257	0.003485771	FSD1L	protein_coding
ENSG00000106714.17	81.6831785	0.21471027	0.162591826	1.320547749	0.186652205	0.40154735	CNTNAP3	protein_coding
ENSG00000106723.17	5189.842371	0.098238025	0.047720361	2.058618648	0.039530785	0.152919043	SPIN1	protein_coding
ENSG00000106733.21	883.918413	-0.176440707	0.067203364	-2.625474309	0.008652834	0.054615374	NMRK1	protein_coding
ENSG00000106771.13	7089.123628	-0.09991241	0.076617053	-1.304049245	0.192216792	0.408397424	TMEM245	protein_coding

ENSG00000106772.18	5391.726139	-0.505464302	0.218240047	-2.316093261	0.020553177	0.098988597	PRUNE2	protein_coding
ENSG00000106780.9	1879.842467	0.022391429	0.063911814	0.350348829	0.726076924	0.857111042	MEGF9	protein_coding
ENSG00000106785.15	4687.572179	-0.021826515	0.063826445	-0.341966636	0.732376001	0.860958534	TRIM14	protein_coding
ENSG00000106789.13	4788.350883	-0.018330882	0.098451369	-0.186192256	0.852293997	0.927768173	CORO2A	protein_coding
ENSG00000106799.13	4459.855935	0.127018569	0.079270796	1.602337508	0.109080996	0.28979002	TGFBR1	protein_coding
ENSG00000106803.10	4257.882836	-0.138371498	0.064515682	-2.144773065	0.031970996	0.132900895	SEC61B	protein_coding
ENSG00000106804.8	438.5888978	-0.239654705	0.127741601	-1.876089726	0.06064296	0.201068118	C5	protein_coding
ENSG00000106809.11	2562.242862	-0.261741016	0.234746181	-1.11499584	0.264852204	0.49267756	OGN	protein_coding
ENSG00000106819.13	4195.431187	0.186077154	0.155248134	1.198579005	0.230691686	0.45465673	ASPN	protein_coding
ENSG00000106823.12	743.9931384	0.173599149	0.106019166	1.637431759	0.101540287	0.277977821	ECM2	protein_coding
ENSG00000106829.20	1531.077355	0.044074826	0.085888319	0.51316438	0.60783634	0.783984023	TLE4	protein_coding
ENSG00000106852.15	308.8419835	0.123864077	0.109730537	1.12880225	0.258981263	0.486174641	LHX6	protein_coding
ENSG00000106853.20	2175.430261	-0.223528124	0.096130557	-2.325255699	0.020058292	0.097423113	PTGR1	protein_coding
ENSG00000106868.17	1294.592209	0.05564726	0.067549973	0.823793958	0.410056641	0.630942264	SUSD1	protein_coding
ENSG00000106927.12	144.8319163	-0.567423704	0.198191036	-2.863013961	0.004196321	0.032778911	AMBP	protein_coding
ENSG00000106948.17	4435.43729	-0.33472356	0.087369289	-3.831135227	0.000127553	0.002382288	AKNA	protein_coding
ENSG00000106952.7	182.4540844	-0.429643036	0.1331865	-3.225875275	0.001255881	0.013640956	TNFSF8	protein_coding
ENSG00000106976.21	1916.442784	0.454059735	0.129770666	3.498939698	0.000467112	0.006474348	DNM1	protein_coding
ENSG00000106991.14	8350.183292	0.131820739	0.083025835	1.587707479	0.112352501	0.295247186	ENG	protein_coding
ENSG00000106992.19	359.79382	0.11309201	0.095432055	1.185052647	0.235996632	0.460898366	AK1	protein_coding
ENSG00000106993.12	794.8263825	0.093241147	0.060476287	1.541780272	0.123126998	0.312211455	CDC37L1	protein_coding
ENSG00000107014.9	27.75955114	0.282066162	0.177611975	1.588103292	0.112262984	0.295094948	RLN2	protein_coding
ENSG00000107018.8	9.979365189	0.184040015	0.161099783	1.142397658	0.253288765	0.480129221	RLN1	protein_coding
ENSG00000107020.10	1013.643343	-0.063641006	0.081835932	-0.777665804	0.436766057	0.654050148	PLGRKT	protein_coding
ENSG00000107021.16	2139.227724	0.016642559	0.049390162	0.336961017	0.736146287	0.863152072	TBC1D13	protein_coding
ENSG00000107036.12	1720.203326	0.055299719	0.067759783	0.816114168	0.414434841	0.634851604	RIC1	protein_coding
ENSG00000107077.19	1849.741892	-0.005807484	0.05934838	-0.09785413	0.922048124	0.963181779	KDM4C	protein_coding
ENSG00000107099.16	2417.596718	-0.334730075	0.107639636	-3.109728781	0.001872592	0.01824663	DOCK8	protein_coding
ENSG00000107104.20	2702.763695	-0.068321246	0.085505937	-0.799023417	0.424276834	0.643242236	KANK1	protein_coding
ENSG00000107105.15	14.84626388	-0.005034175	0.189631232	-0.026547184	0.9788209	0.990569863	ELAVL2	protein_coding
ENSG00000107130.10	2476.694772	0.120565711	0.142571422	0.845651318	0.397747288	0.620863043	NCS1	protein_coding
ENSG00000107140.16	3712.403635	0.098360706	0.058548929	1.679974466	0.092962284	0.263395152	TESK1	protein_coding
ENSG00000107147.14	68.37749343	0.804129205	0.168200269	4.78078429	1.74613E-06	8.01801E-05	KCNT1	protein_coding
ENSG00000107159.13	1974.988129	0.590888948	0.227530795	2.596962527	0.009405218	0.057842433	CA9	protein_coding
ENSG00000107164.16	4145.137862	0.110372947	0.037062105	2.978053932	0.002900849	0.025015593	FUBP3	protein_coding
ENSG00000107165.13	240.0348982	-0.092423034	0.194612201	-0.474908733	0.634852027	0.802004777	TYRP1	protein_coding
ENSG00000107175.12	2552.073867	0.077142886	0.067672051	1.139951945	0.254306322	0.481078399	CREB3	protein_coding
ENSG00000107185.10	2563.954146	0.059492583	0.062350411	0.95416505	0.340000103	0.569853249	RGP1	protein_coding
ENSG00000107186.17	1117.395181	0.057005382	0.130373756	0.437245835	0.661933072	0.8189188	MPDZ	protein_coding
ENSG00000107187.17	4.133823753	0.214408095	0.273302597	0.784508075	0.432742046	0.650800667	LHX3	protein_coding
ENSG00000107201.10	1927.329544	0.095344236	0.084075814	1.134026916	0.256783258	0.483885147	DDX58	protein_coding
ENSG00000107223.13	9564.898266	-0.139434051	0.058727878	-2.374239545	0.017585138	0.089105529	EDF1	protein_coding

ENSG00000107242.20	2868.199651	-0.202305746	0.116729023	-1.73312293	0.083073822	0.245874846	PIP5K1B	protein_coding
ENSG00000107249.23	1181.431163	0.39616824	0.128699548	3.078241106	0.002082264	0.0197387	GLIS3	protein_coding
ENSG00000107262.26	4654.018184	0.111761722	0.07384385	1.513487196	0.130155951	0.322898123	BAG1	protein_coding
ENSG00000107263.18	7429.855501	-0.038947133	0.059011667	-0.659990386	0.509259999	0.712744828	RAPGEF1	protein_coding
ENSG00000107281.10	3856.431811	0.135444607	0.107356754	1.261630988	0.2070816	0.427023056	NPDC1	protein_coding
ENSG00000107282.8	365.8790288	0.040615578	0.099443794	0.408427475	0.682959866	0.832461808	APBA1	protein_coding
ENSG00000107290.14	6728.516805	0.003804434	0.057177555	0.066537199	0.946950143	0.975122793	SETX	protein_coding
ENSG00000107295.10	19.12694538	0.491115117	0.220295045	2.229351625	0.025790518	0.115363725	SH3GL2	protein_coding
ENSG00000107317.13	163.3287634	-0.605509118	0.183143596	-3.306198687	0.00094571	0.010990006	PTGDS	protein_coding
ENSG00000107331.17	5251.968637	0.224387335	0.08436068	2.659856878	0.007817386	0.050943359	ABCA2	protein_coding
ENSG00000107338.10	1665.634301	0.110317463	0.07592265	1.453024412	0.146216976	0.346664699	SHB	protein_coding
ENSG00000107341.5	6599.206892	-0.01317688	0.044991452	-0.292875181	0.76961756	0.883358818	UBE2R2	protein_coding
ENSG00000107362.14	1172.618255	0.032989994	0.060181403	0.548175891	0.583571134	0.766757503	ABHD17B	protein_coding
ENSG00000107371.14	835.8021969	-0.025752196	0.053657928	-0.479932737	0.631275222	0.799743071	EXOSC3	protein_coding
ENSG00000107372.13	9595.202252	-0.025092846	0.046672179	-0.537640338	0.590825376	0.77170231	ZFAND5	protein_coding
ENSG00000107404.20	4478.446069	0.107952638	0.071896533	1.501499898	0.133226313	0.327490995	DVL1	protein_coding
ENSG00000107438.9	16085.87839	-0.138653392	0.072402147	-1.915045312	0.055486725	0.190141826	PDLIM1	protein_coding
ENSG00000107443.16	749.1347601	-0.077970983	0.063185222	-1.234006624	0.217200436	0.438564976	CCNJ	protein_coding
ENSG00000107447.8	1.921981066	-0.547148872	0.302719094	-1.807447509	0.070692532	0.221170952	DNTT	protein_coding
ENSG00000107485.18	401.3189508	0.016739459	0.122333708	0.136834398	0.891161693	0.948099441	GATA3	protein_coding
ENSG00000107518.18	74.28824255	-0.196073542	0.167025047	-1.173917001	0.240428272	0.465816415	ATRNL1	protein_coding
ENSG00000107521.19	5196.475929	-0.018224501	0.053041639	-0.343588579	0.731155715	0.860298462	HPS1	protein_coding
ENSG00000107537.14	2126.10437	-0.35791928	0.084071635	-4.257313187	2.06898E-05	0.000572582	PHYH	protein_coding
ENSG00000107551.21	1755.497247	-0.116112342	0.087775574	-1.322832044	0.185891238	0.400681728	RASSF4	protein_coding
ENSG00000107554.17	4511.634649	-0.094828743	0.066402318	-1.428093878	0.153264839	0.356793734	DNMBP	protein_coding
ENSG00000107560.12	1550.968958	-0.327205935	0.073078303	-4.477470365	7.55327E-06	0.000260609	RAB11FIP2	protein_coding
ENSG00000107562.16	2886.874651	-0.104428842	0.142563524	-0.73250744	0.463858903	0.676067277	CXCL12	protein_coding
ENSG00000107566.14	3203.2913	-0.058335431	0.060267949	-0.967934569	0.333077048	0.562674334	ERLIN1	protein_coding
ENSG00000107581.13	18026.03036	-0.056307157	0.043465221	-1.295453135	0.195163958	0.412245481	EIF3A	protein_coding
ENSG00000107593.17	39.49289916	-0.105456226	0.135666713	-0.777318349	0.436970972	0.654090549	PKD2L1	protein_coding
ENSG00000107611.16	124.4959307	-0.017434903	0.110936946	-0.157160474	0.875118378	0.93966865	CUBN	protein_coding
ENSG00000107614.22	475.047405	-0.147084882	0.068407902	-2.15011537	0.03154609	0.131838877	TRDMT1	protein_coding
ENSG00000107625.13	1697.306915	-0.012351151	0.0416566	-0.296499264	0.766848829	0.881703796	DDX50	protein_coding
ENSG00000107643.16	2153.774748	0.092999491	0.046748261	1.989367905	0.04666061	0.170203044	MAPK8	protein_coding
ENSG00000107651.13	4599.751814	0.023504683	0.048995979	0.479726785	0.631421679	0.799828573	SEC23IP	protein_coding
ENSG00000107669.18	3182.964282	-0.105830203	0.067375435	-1.570753534	0.116239909	0.30128728	ATE1	protein_coding
ENSG00000107672.15	2057.840003	-0.24816463	0.053688487	-4.622306296	3.79497E-06	0.000149931	NSMCE4A	protein_coding
ENSG00000107679.14	4953.88206	-0.064048897	0.065174697	-0.982726424	0.325742102	0.555451608	PLEKHA1	protein_coding
ENSG00000107719.9	1302.827996	-0.18077192	0.115354587	-1.567097803	0.117091833	0.302747763	PALD1	protein_coding
ENSG00000107731.12	3018.503619	0.492927308	0.08991668	5.482045215	4.20437E-08	4.0003E-06	UNC5B	protein_coding
ENSG00000107736.21	269.4018596	0.249718831	0.134234165	1.860322449	0.062839919	0.205720007	CDH23	protein_coding
ENSG00000107738.20	4391.430415	-0.046554451	0.087781787	-0.530342944	0.595874177	0.77539444	VSIR	protein_coding

ENSG00000107742.13	4248.462977	0.289646022	0.148339581	1.952587566	0.050868486	0.179897419	SPOCK2	protein_coding
ENSG00000107745.19	4435.403009	-0.11803226	0.057190794	-2.063833228	0.039033531	0.151628115	MICU1	protein_coding
ENSG00000107758.16	2808.525171	-0.098364673	0.054734977	-1.79710816	0.07231845	0.224428224	PPP3CB	protein_coding
ENSG00000107771.17	3435.68416	-0.119490396	0.069171085	-1.727461635	0.08408479	0.247729219	CCSER2	protein_coding
ENSG00000107779.14	2517.639821	-0.050938927	0.068193168	-0.746979929	0.455075676	0.669162579	BMPR1A	protein_coding
ENSG00000107789.16	1354.789705	-0.007216656	0.063671731	-0.113341604	0.909759734	0.957316312	MINPP1	protein_coding
ENSG00000107796.13	16027.43695	-0.116055019	0.162160237	-0.715681111	0.474188277	0.684762589	ACTA2	protein_coding
ENSG00000107798.18	7138.026851	-0.191666249	0.089524067	-2.140946616	0.032278343	0.133732632	LIPA	protein_coding
ENSG00000107807.13	212.330556	-0.070947789	0.199842126	-0.355019187	0.722575217	0.855233249	TLX1	protein_coding
ENSG00000107815.10	1046.292994	-0.002717738	0.06966433	-0.039011898	0.968880903	0.98561881	TWNK	protein_coding
ENSG00000107816.17	3698.820557	0.165764766	0.07185856	2.306820043	0.021064854	0.10062136	LZTS2	protein_coding
ENSG00000107819.13	3229.088684	0.194452164	0.066271686	2.934166538	0.003344447	0.027870595	SFXN3	protein_coding
ENSG00000107821.15	698.0149045	-0.275633039	0.100597365	-2.739962823	0.006144613	0.042972849	KAZALD1	protein_coding
ENSG00000107829.15	2300.569689	-0.168975376	0.059702508	-2.830289404	0.004650592	0.035345232	FBXW4	protein_coding
ENSG00000107831.13	8.498999356	-0.214927087	0.17422873	-1.233591541	0.217355147	0.438808684	FGF8	protein_coding
ENSG00000107833.10	783.9174367	-0.159700547	0.097756035	-1.633664319	0.102329354	0.279266825	NPM3	protein_coding
ENSG00000107854.6	4477.076999	0.001305287	0.046594228	0.02801393	0.977651041	0.989997464	TNKS2	protein_coding
ENSG00000107859.10	4.716333725	0.385403377	0.179805493	2.143446066	0.032077297	0.133203011	PITX3	protein_coding
ENSG00000107862.6	8724.3375	0.050890284	0.04422607	1.150685203	0.249861766	0.476612857	GBF1	protein_coding
ENSG00000107863.18	7108.152465	0.033380991	0.063537493	0.52537469	0.599322745	0.778092872	ARHGAP21	protein_coding
ENSG00000107864.15	609.694763	-0.040453759	0.063847855	-0.633596203	0.52634438	0.725167735	CPEB3	protein_coding
ENSG00000107872.13	1145.812658	-0.187216337	0.066918292	-2.797685528	0.00514702	0.037951215	FBXL15	protein_coding
ENSG00000107874.11	1901.234646	-0.109861424	0.057129823	-1.923013546	0.054478349	0.187950519	CUEDC2	protein_coding
ENSG00000107882.12	1443.003046	0.058206219	0.05328439	1.09236905	0.274670921	0.503344778	SUFU	protein_coding
ENSG00000107890.17	996.2684517	-0.216695203	0.067363506	-3.216804107	0.001296271	0.013964794	ANKRD26	protein_coding
ENSG00000107897.20	3756.777321	0.021461976	0.070455912	0.304615684	0.760658873	0.877514473	ACBD5	protein_coding
ENSG00000107902.14	1441.27353	-0.011751132	0.082959556	-0.141648922	0.887357322	0.94618631	LHPP	protein_coding
ENSG00000107929.15	6317.868587	0.007240936	0.053069277	0.136443086	0.891471015	0.948289616	LARP4B	protein_coding
ENSG00000107937.19	3731.597223	0.016185955	0.060057984	0.269505462	0.78754074	0.892878104	GTPBP4	protein_coding
ENSG00000107938.18	783.7810182	-0.019316171	0.058017661	-0.332936043	0.739182562	0.865113901	EDRF1	protein_coding
ENSG00000107949.17	2644.305131	-0.077370364	0.057935639	-1.335453724	0.181727891	0.395168301	BCCIP	protein_coding
ENSG00000107951.15	1541.218252	-0.122991674	0.053350257	-2.305362336	0.021146289	0.100874859	MTPAP	protein_coding
ENSG00000107954.10	469.6646931	0.549694449	0.193892476	2.835047863	0.004581882	0.035003783	NEURL1	protein_coding
ENSG00000107957.16	6980.94299	0.362197862	0.076461974	4.736967214	2.1694E-06	9.56843E-05	SH3PXD2A	protein_coding
ENSG00000107959.17	4465.171839	0.073557425	0.053860943	1.365691379	0.172035851	0.382632985	PITRM1	protein_coding
ENSG00000107960.11	1508.701942	-0.022558983	0.062372427	-0.361681988	0.717589689	0.852340945	STN1	protein_coding
ENSG00000107968.10	1187.983152	-0.038814016	0.09333428	-0.415860237	0.677512277	0.829240085	MAP3K8	protein_coding
ENSG00000107984.10	1390.191494	0.52159624	0.277916675	1.876808002	0.060544413	0.200894228	DKK1	protein_coding
ENSG00000108001.15	186.5224212	0.06540123	0.128433095	0.509224126	0.610595134	0.786006858	EBF3	protein_coding
ENSG00000108010.12	3334.536413	0.012566173	0.053502948	0.234868789	0.814310575	0.908348275	GLRX3	protein_coding
ENSG00000108018.15	86.63998545	-0.546278744	0.213945948	-2.553349339	0.010669243	0.063187494	SORCS1	protein_coding
ENSG00000108021.20	5112.761289	-0.03658611	0.051720893	-0.707375834	0.479332951	0.688687504	TASOR2	protein_coding

ENSG00000108039.18	5015.151036	-0.043549713	0.055598954	-0.783282954	0.433460971	0.651348251	XPNPEP1		protein_coding
ENSG00000108055.10	5557.258681	-0.036625117	0.054919357	-0.666889032	0.504843018	0.708900714	SMC3		protein_coding
ENSG00000108061.12	3216.443138	-0.023973057	0.043759821	-0.547832616	0.583806841	0.766903278	SHOC2		protein_coding
ENSG00000108064.11	2788.965082	-0.109366497	0.052322077	-2.090255262	0.036594876	0.14503819	TFAM		protein_coding
ENSG00000108091.11	7982.59995	-0.011071929	0.063271759	-0.17499006	0.861087444	0.932477049	CCDC6		protein_coding
ENSG00000108094.16	2502.857067	-0.090322387	0.043830894	-2.060701484	0.039331528	0.152359879	CUL2		protein_coding
ENSG00000108100.18	4582.539393	-0.085845841	0.041258671	-2.080673942	0.037463761	0.147409328	CCNY		protein_coding
ENSG00000108106.14	3258.227939	0.151550524	0.100457261	1.50860697	0.131399252	0.324736825	UBE2S		protein_coding
ENSG00000108107.15	90746.70596	-0.221482213	0.082156069	-2.695871605	0.007020472	0.047122244	RPL28		protein_coding
ENSG00000108175.17	11351.44187	0.092904858	0.064669255	1.436615558	0.15082726	0.35315671	ZMIZ1		protein_coding
ENSG00000108176.15	412.4822029	0.544093857	0.184699269	2.945836543	0.003220826	0.02709535	DNAJC12		protein_coding
ENSG00000108179.14	6697.530164	-0.150091734	0.075427825	-1.989872211	0.046605014	0.170049963	PPIF		protein_coding
ENSG00000108187.16	2017.726357	-0.59083247	0.127400079	-4.63761461	3.52453E-06	0.000141426	PBLD		protein_coding
ENSG00000108219.15	7832.148269	0.1722114	0.054405858	3.165309903	0.001549179	0.015880379	TSPAN14		protein_coding
ENSG00000108231.14	31.97096164	-0.171251321	0.211657762	-0.809095394	0.418460277	0.638570011	LGI1		protein_coding
ENSG00000108239.9	1143.356796	-0.115689087	0.053018508	-2.182050996	0.029105769	0.125017847	TBC1D12		protein_coding
ENSG00000108242.13	714.4296643	-0.2189699	0.17938451	-1.220673398	0.222209704	0.444795262	CYP2C18		protein_coding
ENSG00000108244.17	1245.935613	-0.168640196	0.264983745	-0.636417137	0.524504574	0.723663817	KRT23		protein_coding
ENSG00000108255.8	3.987079431	-0.150516569	0.159659918	-0.94273234	0.345817827	0.575289309	CRYBA1		protein_coding
ENSG00000108256.9	8098.984147	0.194254739	0.045557053	4.263988211	2.0081E-05	0.00055915	NUFIP2		protein_coding
ENSG00000108262.16	6094.294694	0.087978175	0.059769549	1.471956486	0.141032628	0.338923245	GIT1		protein_coding
ENSG00000108298.12	63948.53577	-0.074800307	0.075570643	-0.989806409	0.322268752	0.5521331	RPL19		protein_coding
ENSG00000108306.13	2364.909512	0.175404822	0.064879106	2.703564121	0.006860021	0.046361529	FBXL20		protein_coding
ENSG00000108309.14	83.51232548	0.226847404	0.17985015	1.261313401	0.207195955	0.427043377	RUNDC3A		protein_coding
ENSG00000108312.15	6222.062985	-0.032154564	0.034369608	-0.935552242	0.349503773	0.5786598	UBTF		protein_coding
ENSG00000108342.13	407.6905867	0.492863936	0.236176068	2.086849617	0.03690173	0.145816712	CSF3		protein_coding
ENSG00000108344.15	10970.4496	-0.132008563	0.096384647	-1.369601569	0.170811309	0.380917116	PSMD3		protein_coding
ENSG00000108349.17	7567.652	-0.147044893	0.082764195	-1.776672798	0.07562209	0.231459951	CASC3		protein_coding
ENSG00000108352.13	4694.496568	0.004452775	0.143583354	0.031011775	0.975260149	0.988550686	RAPGEFL1		protein_coding
ENSG00000108370.17	364.2161079	-0.275012962	0.100165971	-2.745572761	0.006040537	0.042531667	RGS9		protein_coding
ENSG00000108375.13	1226.747412	-0.397581414	0.140261426	-2.834574157	0.004588681	0.035023679	RNF43		protein_coding
ENSG00000108379.10	189.90736	0.083430524	0.124123456	0.672157603	0.501483368	0.70664966	WNT3		protein_coding
ENSG00000108381.11	122.9387306	-0.308170452	0.154335561	-1.996755974	0.045851698	0.168330199	ASPA		protein_coding
ENSG00000108384.15	707.8303897	0.117461729	0.058414978	2.010815273	0.044344972	0.164727718	RAD51C		protein_coding
ENSG00000108387.15	379.061558	0.066706863	0.087811859	0.759656656	0.447459844	0.662772263	SEPTIN4		protein_coding
ENSG00000108389.9	2824.737133	0.010154769	0.055217381	0.183905294	0.854087751	0.928792722	MTMR4		protein_coding
ENSG00000108395.14	1905.072449	0.079392624	0.048622199	1.632847269	0.102501123	0.279570096	TRIM37		protein_coding
ENSG00000108405.4	245.0645994	0.163547039	0.135441844	1.207507479	0.227236767	0.4506539	P2RX1		protein_coding
ENSG00000108406.10	2688.814631	0.13177087	0.056281067	2.341300141	0.019216712	0.094398223	DHX40		protein_coding
ENSG00000108417.4	1.114689006	-0.106988826	0.461949241	-0.23160299	0.816846385	0.909701626	KRT37		protein_coding
ENSG00000108423.15	520.3183508	0.050988471	0.055026834	0.926611011	0.354128536	0.582374871	TUBD1		protein_coding
ENSG00000108424.11	15823.36058	0.016206536	0.038457524	0.421413921	0.673452856	0.826421534	KPNB1		protein_coding

ENSG00000108433.17	2377.89761	-0.017520317	0.03663585	-0.478228757	0.632487397	0.800479595	GOSR2	protein_coding
ENSG00000108439.11	1467.60793	-0.018011788	0.058040884	-0.310329313	0.756310543	0.8749761	PNPO	protein_coding
ENSG00000108442.2	0.900597519	-0.365228123	0.312506189	-1.168706847	0.242521762		TVP23BP2	processed_pseudogene
ENSG00000108443.14	2140.980954	0.14206608	0.047488047	2.991617674	0.002775036	0.024216308	RPS6KB1	protein_coding
ENSG00000108448.21	466.8672489	-0.3252208	0.096661715	-3.364525452	0.000766754	0.009409418	TRIM16L	protein_coding
ENSG00000108452.1	3.621555125	-0.253987466	0.143935346	-1.764594121	0.077632004	0.235449503	ZNF29P	unprocessed_pseudogene
ENSG00000108465.15	388.5373599	0.123823577	0.095365192	1.298414797	0.194144837	0.410831329	CDK5RAP3	protein_coding
ENSG00000108468.15	2730.966383	0.064160097	0.053699191	1.194805645	0.232162974	0.456421099	CBX1	protein_coding
ENSG00000108469.15	1527.632826	0.079745262	0.055008786	1.449682282	0.147147139	0.348233277	RECQL5	protein_coding
ENSG00000108474.17	617.9214338	-0.181203126	0.063807501	-2.839840491	0.00451361	0.034634543	PIGL	protein_coding
ENSG00000108479.12	985.8158437	-0.036220056	0.071798941	-0.504465047	0.613934618	0.788186927	GALK1	protein_coding
ENSG00000108506.13	1397.259507	-0.087439394	0.057026236	-1.533318714	0.125197346	0.315420691	INTS2	protein_coding
ENSG00000108509.21	2576.531922	-0.004064449	0.052590038	-0.077285523	0.938396407	0.970823446	CAMTA2	protein_coding
ENSG00000108510.10	7030.165657	0.135400465	0.060913951	2.222815336	0.026228255	0.116591722	MED13	protein_coding
ENSG00000108511.10	553.3607096	0.42070867	0.139640064	3.012807762	0.002588428	0.023038386	HOXB6	protein_coding
ENSG00000108515.18	234.0113505	-0.238463065	0.086337687	-2.761981164	0.005745179	0.041075291	ENO3	protein_coding
ENSG00000108516.5	0.274678185	-0.141911315	0.772755728	-0.183643174	0.854293391		AC003958.1	unprocessed_pseudogene
ENSG00000108518.8	37658.3531	-0.133222901	0.062079704	-2.145997686	0.031873163	0.132682654	PFN1	protein_coding
ENSG00000108523.16	5490.042617	-0.129702681	0.054390631	-2.384651169	0.017095323	0.087481259	RNF167	protein_coding
ENSG00000108528.14	2875.63814	-0.098480978	0.053415545	-1.843676369	0.065230319	0.210511857	SLC25A11	protein_coding
ENSG00000108551.5	741.3281672	1.314018132	0.169255249	7.76352957	8.25979E-15	7.34565E-12	RASD1	protein_coding
ENSG00000108556.10	44.69036129	0.188058625	0.11887789	1.581947876	0.11366147	0.297354018	CHRNE	protein_coding
ENSG00000108557.19	4587.395875	0.254770737	0.063566126	4.007963889	6.12445E-05	0.001352001	RAI1	protein_coding
ENSG00000108559.12	2388.456518	-0.033310314	0.05137856	-0.648331014	0.516770879	0.718090707	NUP88	protein_coding
ENSG00000108561.8	6709.406456	-0.120335844	0.074742085	-1.610014539	0.107394682	0.287236118	C1QBP	protein_coding
ENSG00000108576.10	63.29673014	0.034401319	0.175380418	0.196152563	0.844490756	0.923733673	SLC6A4	protein_coding
ENSG00000108578.15	1937.665191	0.02916895	0.066657617	0.437593651	0.661680874	0.818800723	BLMH	protein_coding
ENSG00000108582.12	12959.20572	0.210745002	0.08507724	2.477101999	0.013245404	0.073528023	CPD	protein_coding
ENSG00000108587.16	4008.256141	0.090357105	0.03695735	2.444902153	0.014489145	0.078200824	GOSR1	protein_coding
ENSG00000108588.14	6528.671551	-0.025550375	0.056360256	-0.453340292	0.650303724	0.811608567	CCDC47	protein_coding
ENSG00000108590.11	179.9560708	-0.009604169	0.057032663	-0.168397692	0.866270424	0.935133429	MED31	protein_coding
ENSG00000108591.10	1262.738162	-0.041205	0.045438495	-0.906830209	0.364496592	0.59176095	DRG2	protein_coding
ENSG00000108592.17	2959.421189	0.089847705	0.046888918	1.916182083	0.055341923	0.189892518	FTSJ3	protein_coding
ENSG00000108599.15	1552.459371	-0.006806509	0.042498084	-0.160160376	0.872754741	0.938514473	AKAP10	protein_coding
ENSG00000108602.18	2554.72535	-0.563758021	0.203378141	-2.771969586	0.005571824	0.040179279	ALDH3A1	protein_coding
ENSG00000108604.16	6732.262411	-0.036408119	0.053516998	-0.680309446	0.496308544	0.702572897	SMARCD2	protein_coding
ENSG00000108622.11	307.3379562	0.032551228	0.101153464	0.32180043	0.747603889	0.870154515	ICAM2	protein_coding
ENSG00000108639.8	18457.22933	0.148640182	0.077410085	1.920165591	0.054836986	0.188769362	SYNGR2	protein_coding
ENSG00000108641.19	490.8396067	-0.033257589	0.067302542	-0.494150568	0.621199847	0.792772967	B9D1	protein_coding
ENSG00000108651.10	2402.365238	0.01238627	0.048124064	0.257382048	0.79688386	0.898385532	UTP6	protein_coding
ENSG00000108654.16	24627.73516	0.0310956	0.039844344	0.78042695	0.43513961	0.65282154	DDX5	protein_coding
ENSG00000108666.10	819.5386814	-0.052004666	0.052310296	-0.994157363	0.320146262	0.549839664	C17orf75	protein_coding

ENSG00000108669.16	3591.721741	-0.037504201	0.055230415	-0.679049777	0.497106323	0.703311503	CYTH1	protein_coding
ENSG00000108671.11	8385.735045	0.013280644	0.05677089	0.233934049	0.815036179	0.908711589	PSMD11	protein_coding
ENSG00000108679.13	61979.33705	0.149450107	0.074017434	2.019120349	0.043474712	0.162532193	LGALS3BP	protein_coding
ENSG00000108684.15	22.2728237	-0.136931768	0.210440356	-0.650691582	0.515245598	0.716870509	ASIC2	protein_coding
ENSG00000108688.11	41.35667056	0.727321647	0.223516006	3.254002524	0.001137913	0.012672328	CCL7	protein_coding
ENSG00000108691.9	2277.753357	-0.00456272	0.129470469	-0.035241399	0.971887251	0.986737869	CCL2	protein_coding
ENSG00000108700.5	194.3782135	0.039609838	0.164142072	0.241314352	0.809311488	0.905498786	CCL8	protein_coding
ENSG00000108702.4	3.758941244	-0.263956911	0.253217598	-1.042411401	0.297220983	0.527103971	CCL1	protein_coding
ENSG00000108733.11	376.0361006	-0.125068894	0.048545002	-2.576349537	0.009984965	0.060390677	PEX12	protein_coding
ENSG00000108759.4	3.365253187	0.495961156	0.344408364	1.44003807	0.149856629	0.352018883	KRT32	protein_coding
ENSG00000108771.13	995.2914867	0.043038381	0.081305509	0.529341509	0.596568566	0.776066284	DHX58	protein_coding
ENSG00000108773.11	3456.17816	-0.115895585	0.064099881	-1.808046811	0.070599215	0.220971692	KAT2A	protein_coding
ENSG00000108774.15	3622.690385	0.024786972	0.054309284	0.456403956	0.648099521	0.810124776	RAB5C	protein_coding
ENSG00000108784.10	1854.623127	-0.00383989	0.059995087	-0.064003411	0.948967511	0.976116068	NAGLU	protein_coding
ENSG00000108785.7	60.21244787	-0.012353496	0.097676872	-0.126473087	0.899357451	0.952071747	HSD17B1P1	unprocessed_pseudogene
ENSG00000108786.11	65.55928659	0.342096243	0.14088724	2.42815633	0.0151758	0.080650739	HSD17B1	protein_coding
ENSG00000108788.12	3356.884855	0.012489186	0.05825304	0.214395439	0.830238698	0.916442367	MLX	protein_coding
ENSG00000108797.12	1161.870051	0.110060055	0.123658527	0.890032071	0.373448666	0.599512709	CNTNAP1	protein_coding
ENSG00000108798.9	1100.938001	-0.291950116	0.099333503	-2.939090104	0.003291774	0.027543322	ABI3	protein_coding
ENSG00000108799.13	1782.85539	-0.060304028	0.060330991	-0.99955308	0.317526839	0.546782071	EZH1	protein_coding
ENSG00000108813.11	108.4250648	0.321561754	0.172882459	1.860002202	0.062885214	0.20584008	DLX4	protein_coding
ENSG00000108819.11	5003.998931	-0.019898512	0.05437664	-0.365938605	0.714410889	0.850760618	PPP1R9B	protein_coding
ENSG00000108821.14	266441.133	0.891447227	0.130164037	6.848644565	7.4553E-12	3.15417E-09	COL1A1	protein_coding
ENSG00000108823.16	339.6749763	-0.078622175	0.192907562	-0.407563985	0.68359381	0.832931648	SGCA	protein_coding
ENSG00000108825.18	0.077452856	-0.108568527	0.948177219	-0.114502358	0.908839578		PTGES3L-AARSD1	protein_coding
ENSG00000108826.16	2122.365731	-0.148584642	0.057810716	-2.570192026	0.010164216	0.061136531	MRPL27	protein_coding
ENSG00000108828.16	8293.199278	0.23754191	0.079787377	2.977186602	0.002909069	0.025058015	VAT1	protein_coding
ENSG00000108829.10	11189.75838	0.064107251	0.06018163	1.065229547	0.286772066	0.51639117	LRRC59	protein_coding
ENSG00000108830.10	119.0739657	-0.307800092	0.174217698	-1.766755588	0.077269169	0.234803831	RND2	protein_coding
ENSG00000108839.12	127.4171105	0.24339746	0.096471602	2.522995924	0.011635975	0.067171258	ALOX12	protein_coding
ENSG00000108840.15	3179.512602	0.176082143	0.073435761	2.397771064	0.016495171	0.085572628	HDAC5	protein_coding
ENSG00000108846.16	11603.32701	0.031806631	0.111104134	0.286277656	0.77466547	0.886001165	ABCC3	protein_coding
ENSG00000108848.16	8213.351872	-0.129287692	0.076831866	-1.682735299	0.092426345	0.262473955	LUC7L3	protein_coding
ENSG00000108849.8	3.879920398	-0.558114414	0.264728528	-2.108251869	0.035009209	0.140776626	PPY	protein_coding
ENSG00000108852.15	286.7424174	0.088401312	0.140237399	0.630369023	0.528453173	0.726723916	MPP2	protein_coding
ENSG00000108854.16	2100.388096	0.142292783	0.048054896	2.961046523	0.003065956	0.026120524	SMURF2	protein_coding
ENSG00000108861.9	6740.090378	-0.053558841	0.07096486	-0.754723413	0.450414956	0.66523016	DUSP3	protein_coding
ENSG00000108878.5	4.908360611	-0.041737152	0.201841813	-0.206781494	0.836180509	0.919595616	CACNG1	protein_coding
ENSG00000108883.13	6638.773408	0.053064527	0.0491731	1.079137312	0.28052652	0.50939679	EFTUD2	protein_coding
ENSG00000108924.14	456.2738555	-0.780626559	0.171845165	-4.542615781	5.55605E-06	0.00020363	HLF	protein_coding
ENSG00000108932.12	176.0169603	-0.053567247	0.110561404	-0.484502231	0.628029482	0.797471902	SLC16A6	protein_coding
ENSG00000108946.15	8557.513397	0.063060665	0.047952716	1.315059297	0.188489975	0.403806481	PRKAR1A	protein_coding

ENSG00000108947.5	129.2756532	0.906765817	0.150601451	6.020963344	1.73382E-09	2.8728E-07	EFNB3	protein_coding
ENSG00000108950.12	600.691609	0.091511221	0.120601578	0.758789581	0.447978439	0.663052373	FAM20A	protein_coding
ENSG00000108953.17	23227.98095	0.041885095	0.057810308	0.72452641	0.468742619	0.680147533	YWHAE	protein_coding
ENSG00000108958.4	23.91089018	0.083479853	0.074692751	1.117643303	0.263719367	0.491430354	AC130689.1	processed_pseudogene
ENSG00000108960.9	1139.378388	-0.070883478	0.081992485	-0.864511882	0.387306756	0.611904694	MMD	protein_coding
ENSG00000108961.14	30.84925952	-0.226241282	0.088698409	-2.550680272	0.010751291	0.063543875	RANGRF	protein_coding
ENSG00000108963.19	921.0288665	-0.093413855	0.055379567	-1.686792804	0.0916432	0.260981291	DPH1	protein_coding
ENSG00000108984.15	1374.1604	0.106119622	0.11320563	0.93740587	0.348549823	0.57769495	MAP2K6	protein_coding
ENSG00000109016.17	1232.11481	-0.126704631	0.058699522	-2.158529176	0.030886712	0.130012522	DHRS7B	protein_coding
ENSG00000109046.15	5238.438422	0.033845622	0.073204975	0.462340459	0.64383719	0.807543771	WSB1	protein_coding
ENSG00000109047.8	8.47590022	-0.040545985	0.154435903	-0.262542482	0.792903238	0.896174888	RCVRN	protein_coding
ENSG00000109061.10	9.819632049	-0.377449663	0.252572852	-1.494418974	0.135066145	0.330438885	MYH1	protein_coding
ENSG00000109062.12	9854.031263	0.035738537	0.089399492	0.399762189	0.689331682	0.835873022	SLC9A3R1	protein_coding
ENSG00000109063.15	104.7485036	-0.077075258	0.105661105	-0.729457247	0.465722011	0.677508531	MYH3	protein_coding
ENSG00000109065.12	1145.512368	0.057536758	0.055833745	1.030501505	0.302774646	0.532365858	NAT9	protein_coding
ENSG00000109066.14	1941.972378	0.238767428	0.057977699	4.118263295	3.81738E-05	0.000928355	TMEM104	protein_coding
ENSG00000109072.14	324.3435081	-0.865053423	0.221397201	-3.907246424	9.33539E-05	0.001876423	VTN	protein_coding
ENSG00000109079.10	4584.48041	0.211377313	0.051477113	4.106238687	4.02154E-05	0.000965546	TNFAIP1	protein_coding
ENSG00000109083.14	754.6501617	0.067130156	0.050193232	1.337434404	0.181080881	0.394283458	IFT20	protein_coding
ENSG00000109084.14	4872.30535	-0.272937031	0.094431201	-2.890326815	0.003848415	0.030861683	TMEM97	protein_coding
ENSG00000109089.7	5186.559057	0.11331042	0.102786332	1.102388018	0.270293018	0.498436751	CDR2L	protein_coding
ENSG00000109099.16	3922.512478	0.291735453	0.096093488	3.035954466	0.002397756	0.021763591	PMP22	protein_coding
ENSG00000109101.8	35.00718092	0.27026668	0.217857649	1.240565486	0.214766309	0.435863983	FOXN1	protein_coding
ENSG00000109103.12	1407.746967	0.018927589	0.058297287	0.324673587	0.745428127	0.868990605	UNC119	protein_coding
ENSG00000109107.14	834.1077298	0.071781369	0.108864198	0.659366167	0.509660663	0.71296211	ALDOC	protein_coding
ENSG00000109111.15	9784.54366	0.064741852	0.042513154	1.522866358	0.127792144	0.319255205	SUPT6H	protein_coding
ENSG00000109113.20	2507.472783	0.33160156	0.118764061	2.792103577	0.005236659	0.038430093	RAB34	protein_coding
ENSG00000109118.14	4138.100524	0.091495317	0.038930026	2.350250606	0.018760775	0.092912637	PHF12	protein_coding
ENSG00000109132.7	7.853932587	-0.879242871	0.315718181	-2.784897809	0.00535446	0.038992527	PHOX2B	protein_coding
ENSG00000109133.13	6161.695044	0.041977142	0.056660647	0.740851792	0.458783308	0.672037457	TMEM33	protein_coding
ENSG00000109158.11	34.36615651	-1.800364984	0.248472876	-7.245720382	4.30147E-13	2.69984E-10	GABRA4	protein_coding
ENSG00000109163.7	15.5361454	0.337125722	0.127161891	2.651153722	0.008021732	0.05188316	GNRHR	protein_coding
ENSG00000109171.15	4214.383559	0.030402019	0.04395305	0.691693042	0.489130117	0.696494564	SLAIN2	protein_coding
ENSG00000109180.15	6021.307136	-0.073974192	0.049645246	-1.490055887	0.136209542	0.332061717	OCIAD1	protein_coding
ENSG00000109181.12	5.446228809	0.32042897	0.411794516	0.778128309	0.436493375	0.653995488	UGT2B10	protein_coding
ENSG00000109182.12	23.15458514	-0.108610407	0.371318521	-0.292499299	0.769904896	0.88353314	CWH43	protein_coding
ENSG00000109184.15	1600.314013	-0.019508049	0.05117424	-0.38120837	0.703048639	0.844057375	DCUN1D4	protein_coding
ENSG00000109189.13	1034.617011	0.056507546	0.057169415	0.988422674	0.322945688	0.552836433	USP46	protein_coding
ENSG00000109193.12	292.2742886	-0.803283132	0.239370599	-3.355813683	0.000791319	0.009607291	SULT1E1	protein_coding
ENSG00000109205.16	415.3005455	-0.532877427	0.223645349	-2.38268951	0.017186683	0.08776003	ODAM	protein_coding
ENSG00000109208.5	0.623793569	0.238830038	0.647829371	0.368661949	0.712379714		SMR3A	protein_coding
ENSG00000109220.11	736.8319862	0.183265582	0.047239236	3.879520461	0.000104663	0.002052602	CHIC2	protein_coding

ENSG00000109255.11	563.1018806	0.473272689	0.177670216	2.663770557	0.007727025	0.050506528	NMU	protein_coding
ENSG00000109265.14	1562.341962	-0.052645121	0.10927176	-0.481781576	0.629961119	0.798932328	CRACD	protein_coding
ENSG00000109270.13	2720.132412	-0.024537758	0.048257887	-0.508471446	0.611122759	0.786337629	LAMTOR3	protein_coding
ENSG00000109272.4	14.59558218	0.651144377	0.207964113	3.131042029	0.001741872	0.017342054	PF4V1	protein_coding
ENSG00000109320.13	4654.413203	-0.040652541	0.049251341	-0.825409821	0.409138961	0.630001714	NFKB1	protein_coding
ENSG00000109321.11	2120.394228	0.532170621	0.166906508	3.188435416	0.00143045	0.015030158	AREG	protein_coding
ENSG00000109323.11	1988.61044	0.10361933	0.061779352	1.677248586	0.093493882	0.264299896	MANBA	protein_coding
ENSG00000109332.20	12595.53454	-0.018859766	0.041698488	-0.452288959	0.651060829	0.811999935	UBE2D3	protein_coding
ENSG00000109339.24	443.2353049	-0.127227817	0.135896378	-0.936211979	0.349164057	0.578361035	MAPK10	protein_coding
ENSG00000109381.20	2130.13329	0.064732161	0.039595799	1.634823977	0.102085953	0.278804185	ELF2	protein_coding
ENSG00000109390.13	2513.166312	-0.126006635	0.072976726	-1.72666878	0.084227166	0.247930775	NDUFC1	protein_coding
ENSG00000109424.4	2.748665454	0.134623509	0.223316255	0.602837932	0.54661651	0.740415519	UCP1	protein_coding
ENSG00000109436.8	1932.087387	0.061946763	0.084665834	0.73166188	0.464374969	0.676384385	TBC1D9	protein_coding
ENSG00000109445.11	1459.918369	-0.012709377	0.051116752	-0.24863429	0.803643681	0.902413155	ZNF330	protein_coding
ENSG00000109452.12	2136.201054	0.108499411	0.098628975	1.100076432	0.271298822	0.499530517	INPP4B	protein_coding
ENSG00000109458.8	1994.254377	-0.02138332	0.076548124	-0.279344793	0.779980234	0.888584375	GAB1	protein_coding
ENSG00000109466.14	955.2057741	0.219602602	0.05655676	3.882870957	0.00010323	0.002030912	KLHL2	protein_coding
ENSG00000109471.5	4.25953376	-0.802389998	0.188602836	-4.254389886	2.0962E-05	0.000578872	IL2	protein_coding
ENSG00000109472.14	2063.529053	0.563345985	0.151615905	3.715612722	0.000202712	0.003426523	CPE	protein_coding
ENSG00000109475.16	22299.34152	-0.239898347	0.070038292	-3.425245528	0.000614244	0.00793564	RPL34	protein_coding
ENSG00000109501.14	4061.273012	-0.119495319	0.105724584	-1.130251025	0.258370465	0.485532354	WFS1	protein_coding
ENSG00000109511.12	5145.315687	-0.027634202	0.246401074	-0.112151303	0.91070344	0.957795512	ANXA10	protein_coding
ENSG00000109519.13	1952.487584	-0.04273448	0.063689627	-0.670980217	0.502233132	0.707104807	GRPEL1	protein_coding
ENSG00000109534.17	1315.713777	-0.130502415	0.061538775	-2.120653461	0.033950976	0.138062865	GAR1	protein_coding
ENSG00000109536.12	1160.77476	-0.15860998	0.057890669	-2.739819451	0.006147294	0.042984699	FRG1	protein_coding
ENSG00000109572.14	5915.853916	0.065354831	0.078464558	0.832921682	0.4048889	0.626479739	CLCN3	protein_coding
ENSG00000109576.14	332.6858828	0.102555082	0.11714816	0.875430582	0.381339666	0.606694747	AADAT	protein_coding
ENSG00000109586.12	5181.135888	0.231425393	0.093696962	2.469934871	0.013513765	0.074590165	GALNT7	protein_coding
ENSG00000109606.13	8561.228756	0.066670896	0.051035358	1.306366789	0.191427854	0.40745624	DHX15	protein_coding
ENSG00000109610.6	6069.350076	0.163394809	0.140933235	1.15937741	0.24630238	0.472824618	SOD3	protein_coding
ENSG00000109618.12	1087.193874	-0.065248965	0.066181739	-0.985905876	0.324179303	0.553973424	SEPSECS	protein_coding
ENSG00000109625.19	21.59205597	0.887001406	0.153596556	5.774878219	7.70088E-09	9.6155E-07	CPZ	protein_coding
ENSG00000109654.16	5884.476463	0.154230166	0.093172508	1.655318386	0.097859931	0.271932293	TRIM2	protein_coding
ENSG00000109667.12	277.1535735	0.077339472	0.082179922	0.941099349	0.346653949	0.575960416	SLC2A9	protein_coding
ENSG00000109670.16	1319.28876	-0.136868067	0.045245067	-3.025038416	0.002486015	0.022350546	FBXW7	protein_coding
ENSG00000109674.4	232.2734233	-0.005996307	0.095013895	-0.063109789	0.949679079	0.976395197	NEIL3	protein_coding
ENSG00000109680.11	285.4941645	0.113795211	0.071166023	1.599010389	0.109818294	0.290826607	TBC1D19	protein_coding
ENSG00000109684.15	69.08298887	-0.491005127	0.130302106	-3.768205612	0.000164425	0.002916223	CLNK	protein_coding
ENSG00000109685.19	6394.205951	0.055298294	0.064885978	0.852237965	0.394082029	0.617656644	NSD2	protein_coding
ENSG00000109686.19	7097.193299	-0.04125327	0.075230029	-0.548361745	0.583443539	0.766705439	SH3D19	protein_coding
ENSG00000109689.18	2337.65596	0.056888988	0.059562728	0.955110526	0.339521806	0.569402007	STIM2	protein_coding
ENSG00000109705.8	174.5043532	0.091442406	0.216524735	0.422318524	0.672792539	0.825971693	NKX3-2	protein_coding

ENSG00000109736.15	4676.568628	0.230045076	0.068187981	3.373689517	0.00074168	0.009158452	MFSD10	protein_coding
ENSG00000109738.11	137.5081038	0.19222462	0.148633366	1.293280404	0.195914096	0.413010911	GLRB	protein_coding
ENSG00000109743.11	344.6358542	0.091769906	0.12069114	0.760369869	0.447033529	0.662482757	BST1	protein_coding
ENSG00000109756.9	2918.283547	0.03635683	0.054415901	0.668128789	0.504051386	0.708275741	RAPGEF2	protein_coding
ENSG00000109758.9	12.35593795	0.327947351	0.186444302	1.758956143	0.078584958	0.237548008	HGFAC	protein_coding
ENSG00000109762.16	824.1479612	0.082647199	0.06835216	1.209138083	0.2266098	0.449884978	SNX25	protein_coding
ENSG00000109771.15	130.0051149	-0.082989329	0.085458867	-0.97110262	0.331497179	0.561039792	LRP2BP	protein_coding
ENSG00000109775.11	828.779022	0.005405939	0.060868507	0.088813397	0.929230211	0.966467728	UFSP2	protein_coding
ENSG00000109787.13	9471.598121	0.136284831	0.061117045	2.229898892	0.025754156	0.11523656	KLF3	protein_coding
ENSG00000109790.16	2118.027657	-0.05342858	0.09266151	-0.576599496	0.56421004	0.753034637	KLHL5	protein_coding
ENSG00000109794.13	553.7091146	0.117341379	0.12780702	0.918113719	0.358559333	0.586235698	FAM149A	protein_coding
ENSG00000109805.10	1357.629275	-0.013607406	0.094706641	-0.143679533	0.885753534	0.945376628	NCAPG	protein_coding
ENSG00000109814.12	4178.447094	-0.107040223	0.066917955	-1.599574024	0.109693115	0.290583396	UGDH	protein_coding
ENSG00000109819.9	1038.4386	-0.014588337	0.175320417	-0.083209573	0.933684901	0.968719481	PPARGC1A	protein_coding
ENSG00000109832.14	15.70067269	-0.995262867	0.19739383	-5.042016099	4.60653E-07	2.7574E-05	DDX25	protein_coding
ENSG00000109846.9	1560.102366	-0.059878082	0.171120843	-0.349916941	0.726401032	0.857331071	CRYAB	protein_coding
ENSG00000109851.7	2.025233152	1.064574261	0.359680577	2.959776894	0.003078619	0.026191468	DBX1	protein_coding
ENSG00000109854.14	3263.014172	-0.203949531	0.06606775	-3.08697559	0.002022042	0.019285298	HTATIP2	protein_coding
ENSG00000109861.17	12310.56124	-0.266057915	0.085033721	-3.128851835	0.001754908	0.017419958	CTSC	protein_coding
ENSG00000109881.17	1430.94807	-0.485131248	0.088574741	-5.477083447	4.32393E-08	4.06086E-06	CCDC34	protein_coding
ENSG00000109906.14	824.8905083	-0.320475112	0.196256657	-1.632938815	0.102481866	0.279566048	ZBTB16	protein_coding
ENSG00000109911.19	919.5145546	-0.103311945	0.048643238	-2.123870633	0.033680963	0.137336512	ELP4	protein_coding
ENSG00000109917.11	2572.427238	-0.049309409	0.053271922	-0.925617231	0.354644935	0.582853568	ZPR1	protein_coding
ENSG00000109919.10	5600.821037	-0.154066853	0.052767916	-2.919707001	0.003503606	0.028850463	MTCH2	protein_coding
ENSG00000109920.13	4225.879889	-0.098783354	0.056735863	-1.741109559	0.081664376	0.243253201	FNBP4	protein_coding
ENSG00000109927.11	40.32047811	0.174343676	0.123113066	1.416126515	0.156738466	0.36165372	TECTA	protein_coding
ENSG00000109929.10	3465.90286	0.102373686	0.074991861	1.365130625	0.172211998	0.382790428	SC5D	protein_coding
ENSG00000109943.9	90.11971465	-0.490145598	0.153593029	-3.191196911	0.001416847	0.014939681	CRTAM	protein_coding
ENSG00000109944.11	251.5296524	0.372348372	0.110089173	3.382243341	0.000718964	0.008928557	JHY	protein_coding
ENSG00000109956.13	115.3374136	-0.218544051	0.163222686	-1.338931839	0.180592863	0.393583156	B3GAT1	protein_coding
ENSG00000109971.14	72751.71013	0.112962677	0.070659876	1.598682065	0.109891265	0.290949125	HSPA8	protein_coding
ENSG00000109991.9	80.25449722	-0.679640711	0.207078665	-3.2820412	0.001030585	0.0117442	P2RX3	protein_coding
ENSG00000110002.16	1968.370426	0.142232931	0.099911532	1.423588729	0.15456556	0.358672103	VWA5A	protein_coding
ENSG00000110011.13	1550.027956	-0.07379002	0.062811007	-1.174794404	0.240076979	0.465490745	DNAJC4	protein_coding
ENSG00000110013.13	3413.36283	0.037321665	0.09092291	0.410475913	0.681456869	0.831373946	SIAE	protein_coding
ENSG00000110025.13	33.44053491	0.017282259	0.08741697	0.197699136	0.843280466	0.92307543	SNX15	protein_coding
ENSG00000110031.13	1112.284014	-0.404814002	0.088639047	-4.566994058	4.94768E-06	0.00018491	LPXN	protein_coding
ENSG00000110042.8	4661.614849	-0.10540124	0.079125994	-1.332068442	0.182837699	0.396670239	DTX4	protein_coding
ENSG00000110046.13	4025.414298	0.127215645	0.055787421	2.280364321	0.02258609	0.105525253	ATG2A	protein_coding
ENSG00000110047.18	6798.951163	0.001564108	0.065211733	0.023985067	0.98086452	0.991614871	EHD1	protein_coding
ENSG00000110048.12	5485.874281	0.061346857	0.041389683	1.482177502	0.138293071	0.33492457	OSBP	protein_coding
ENSG00000110057.8	6444.257743	0.120274201	0.09002027	1.336079099	0.181523421	0.394868874	UNC93B1	protein_coding

ENSG00000110060.9	762.5532179	-0.168785298	0.054320276	-3.107224595	0.001888529	0.018353348	PUS3	protein_coding
ENSG00000110063.10	1592.526454	-0.064460238	0.058305853	-1.105553465	0.268919841	0.496956021	DCPS	protein_coding
ENSG00000110066.15	3079.857002	0.064510066	0.047443215	1.35973216	0.173914697	0.385049801	KMT5B	protein_coding
ENSG00000110074.11	1497.874515	-0.162703122	0.065131804	-2.498059497	0.012487523	0.070524728	FOXRED1	protein_coding
ENSG00000110075.15	7173.600049	0.027852584	0.046645687	0.59710954	0.550434249	0.742906376	PPP6R3	protein_coding
ENSG00000110076.19	435.5198829	0.392230215	0.176501168	2.222252807	0.026266226	0.116689093	NRXN2	protein_coding
ENSG00000110077.14	2132.282712	-0.135888851	0.119603716	-1.136159105	0.255889982	0.482890948	MS4A6A	protein_coding
ENSG00000110079.18	796.0365068	0.047862345	0.128808183	0.371578449	0.710206737	0.84845083	MS4A4A	protein_coding
ENSG00000110080.20	1294.852599	0.939529112	0.114227621	8.225060665	1.95092E-16	3.14871E-13	ST3GAL4	protein_coding
ENSG00000110090.13	9868.415778	-0.247754884	0.07260224	-3.412496412	0.000643708	0.008211608	CPT1A	protein_coding
ENSG00000110092.4	14735.02599	0.167723305	0.102527074	1.635892829	0.101862019	0.278524264	CCND1	protein_coding
ENSG00000110104.13	1507.393187	-0.02329968	0.070875145	-0.328742611	0.742350244	0.867136562	CCDC86	protein_coding
ENSG00000110107.9	7605.778875	-0.014111322	0.055020928	-0.256471908	0.797586469	0.89874911	PRPF19	protein_coding
ENSG00000110108.10	6173.045721	-0.026336416	0.055975816	-0.470496336	0.638000451	0.803971709	TMEM109	protein_coding
ENSG00000110148.10	76.34794157	-0.432600366	0.269736191	-1.603790594	0.108760219	0.289235202	CCKBR	protein_coding
ENSG00000110169.11	34.71416016	-0.086088462	0.128752157	-0.668637047	0.503727034	0.708093967	HPX	protein_coding
ENSG00000110171.20	807.0777789	-0.010918186	0.057932742	-0.188463138	0.850513609	0.926779314	TRIM3	protein_coding
ENSG00000110172.12	2095.069374	-0.028024495	0.066060249	-0.424226297	0.671400784	0.825050725	CHORDC1	protein_coding
ENSG00000110195.14	707.7709773	1.182348565	0.25188446	4.694011553	2.67899E-06	0.000114006	FOLR1	protein_coding
ENSG00000110200.8	247.948751	-0.043586812	0.062697965	-0.695187025	0.486938103	0.69444456	ANAPC15	protein_coding
ENSG00000110203.9	12.37264397	0.740663619	0.243398675	3.043005956	0.002342277	0.021407174	FOLR3	protein_coding
ENSG00000110218.9	1384.082477	0.196982875	0.053203003	3.702476604	0.000213505	0.003561987	PANX1	protein_coding
ENSG00000110237.5	3598.379624	0.251615566	0.093218117	2.699213154	0.006950364	0.046797903	ARHGEF17	protein_coding
ENSG00000110243.12	16.49807097	-1.129164082	0.279330903	-4.04238869	5.29094E-05	0.001203987	APOA5	protein_coding
ENSG00000110244.7	130.4403772	-1.322750103	0.379358289	-3.486809548	0.000488819	0.006683803	APOA4	protein_coding
ENSG00000110245.12	101.5075403	-1.6108703	0.336564428	-4.786216736	1.69955E-06	7.82887E-05	APOC3	protein_coding
ENSG00000110274.16	1663.509604	0.107678656	0.057238259	1.881235687	0.059939865	0.199800974	CEP164	protein_coding
ENSG00000110315.7	2977.779563	0.055065343	0.054598869	1.008543661	0.313193538	0.542579105	RNF141	protein_coding
ENSG00000110318.15	396.8740804	0.250915949	0.116856085	2.147221926	0.031775618	0.132455147	CEP126	protein_coding
ENSG00000110321.19	56456.30617	0.048289225	0.040743201	1.185209382	0.235934672	0.46082711	EIF4G2	protein_coding
ENSG00000110324.11	1896.898172	-0.451633261	0.125120307	-3.60959201	0.000306679	0.004725654	IL10RA	protein_coding
ENSG00000110328.6	637.1372022	0.120793429	0.092956887	1.299456468	0.193787324	0.410353768	GALNT18	protein_coding
ENSG00000110330.8	3763.095572	0.153826548	0.045346132	3.392274944	0.000693148	0.008682188	BIRC2	protein_coding
ENSG00000110344.10	3394.002825	0.06753872	0.064692473	1.043996575	0.296486974	0.526330237	UBE4A	protein_coding
ENSG00000110367.13	8778.645027	-0.079915167	0.045983621	-1.737905021	0.082227551	0.244338901	DDX6	protein_coding
ENSG00000110375.3	35.3337056	0.991746158	0.214128441	4.631548027	3.62942E-06	0.00014457	UPK2	protein_coding
ENSG00000110395.7	3269.28679	-0.009242985	0.062382771	-0.148165663	0.88221203	0.943441809	CBL	protein_coding
ENSG00000110400.11	6044.913186	-0.222729689	0.110783439	-2.010496242	0.044378694	0.164762438	NECTIN1	protein_coding
ENSG00000110422.12	7567.53929	-0.050013253	0.060496797	-0.826709105	0.408401959	0.629422889	HIPK3	protein_coding
ENSG00000110427.17	494.0344319	0.568149292	0.183565145	3.095082622	0.001967581	0.018885742	KIAA1549L	protein_coding
ENSG00000110429.14	2145.90542	0.064051995	0.044065249	1.453571608	0.146065113	0.346512028	FBXO3	protein_coding
ENSG00000110435.12	2110.156728	-0.115104012	0.060376094	-1.906450121	0.056591831	0.192589006	PDHX	protein_coding

ENSG00000110436.13	149.1322276	-0.542326529	0.159122068	-3.408242084	0.000653829	0.008309096	SLC1A2	protein_coding
ENSG00000110442.12	2133.872246	0.018226873	0.058416156	0.312017669	0.755027095	0.874234509	COMMD9	protein_coding
ENSG00000110446.11	2459.50656	0.018846082	0.10205864	0.184659347	0.853496234	0.928404318	SLC15A3	protein_coding
ENSG00000110448.11	765.9059796	-0.597255523	0.134715687	-4.43345193	9.27362E-06	0.000305224	CD5	protein_coding
ENSG00000110455.14	574.2931149	-0.162592134	0.081359734	-1.998434913	0.04566953	0.167887726	ACCS	protein_coding
ENSG00000110484.7	4.72845373	-0.49388409	0.305028221	-1.619142283	0.105416653	0.284233332	SCGB2A2	protein_coding
ENSG00000110492.15	19830.98837	-0.369479988	0.116163592	-3.180686674	0.001469264	0.015313594	MDK	protein_coding
ENSG00000110497.14	2625.848356	0.008937453	0.050236872	0.177906249	0.858796603	0.931240938	AMBRA1	protein_coding
ENSG00000110514.19	4237.463379	0.108495337	0.049462394	2.193491437	0.028271991	0.122395834	MADD	protein_coding
ENSG00000110536.14	180.8272555	-0.0702451	0.061273764	-1.146413984	0.251623899	0.478314575	PTPMT1	protein_coding
ENSG00000110583.13	1712.516514	0.039984254	0.052167627	0.766457218	0.443404298	0.6595743	NAA40	protein_coding
ENSG00000110619.18	2518.784398	0.050504762	0.046029337	1.097229829	0.272540937	0.500907482	CARS1	protein_coding
ENSG00000110628.16	2777.98618	-0.111785736	0.117739942	-0.949429177	0.342402377	0.572283953	SLC22A18	protein_coding
ENSG00000110651.12	19111.15354	-0.05001417	0.064165417	-0.77945679	0.435710682	0.653281186	CD81	protein_coding
ENSG00000110660.15	1718.446007	0.120908101	0.09279816	1.302914852	0.192603833	0.408860932	SLC35F2	protein_coding
ENSG00000110665.12	78.90159388	-0.707342156	0.150134687	-4.71138397	2.4604E-06	0.000106577	C11orf21	protein_coding
ENSG00000110675.13	11.89963413	-0.089788898	0.170971689	-0.525168221	0.599466254	0.778186451	ELMOD1	protein_coding
ENSG00000110680.13	33.6266361	3.091036978	0.345973611	8.934314281	4.09711E-19	9.91888E-16	CALCA	protein_coding
ENSG00000110693.18	482.147043	-0.111200335	0.128485236	-0.865471695	0.386779945	0.611320216	SOX6	protein_coding
ENSG00000110696.10	6702.998903	-0.078170547	0.035614809	-2.194888827	0.028171573	0.122152503	C11orf58	protein_coding
ENSG00000110697.13	5235.304332	0.183154489	0.075572251	2.423568	0.015368876	0.081375726	PITPNM1	protein_coding
ENSG00000110700.7	22749.6307	-0.253809549	0.06453918	-3.932642898	8.4017E-05	0.001732707	RPS13	protein_coding
ENSG00000110711.10	2422.803532	-0.018095831	0.060529676	-0.298957995	0.764972096	0.880369445	AIP	protein_coding
ENSG00000110713.17	8743.358414	0.005365982	0.05028765	0.106705755	0.915022417	0.95976336	NUP98	protein_coding
ENSG00000110717.13	7158.67945	-0.138820251	0.078155475	-1.776206342	0.075698913	0.231603703	NDUFS8	protein_coding
ENSG00000110719.10	6516.152149	0.107229563	0.06346091	1.689694704	0.091086377	0.260126551	TCIRG1	protein_coding
ENSG00000110721.12	1692.704744	-0.25104681	0.090638556	-2.76975737	0.005609806	0.040366337	CHKA	protein_coding
ENSG00000110723.12	1386.839572	-0.020505874	0.104385663	-0.196443396	0.844263133	0.923608042	EXPH5	protein_coding
ENSG00000110756.18	1370.260664	-0.109324257	0.053412364	-2.046796827	0.040678037	0.155589119	HPS5	protein_coding
ENSG00000110768.12	2448.426838	-0.070558563	0.037228993	-1.895258426	0.058058173	0.195757075	GTF2H1	protein_coding
ENSG00000110777.12	1629.755249	-0.836264015	0.160054191	-5.224880474	1.74268E-07	1.27846E-05	POU2AF1	protein_coding
ENSG00000110786.18	62.8520622	-0.153980676	0.165442226	-0.93072174	0.351997521	0.580641264	PTPN5	protein_coding
ENSG00000110799.14	11890.19802	0.134446615	0.106409451	1.263483771	0.206415375	0.426176776	VWF	protein_coding
ENSG00000110801.14	308.5279584	-0.08935178	0.046205923	-1.933773305	0.053141006	0.184876347	PSMD9	protein_coding
ENSG00000110811.20	1031.829397	0.471710166	0.105956274	4.451932373	8.5101E-06	0.000284172	P3H3	protein_coding
ENSG00000110841.14	3999.449297	0.210562981	0.076526675	2.75149783	0.005932341	0.041993768	PPFIBP1	protein_coding
ENSG00000110844.14	397.3953343	0.197534528	0.073505787	2.687333	0.007202511	0.047991409	PRPF40B	protein_coding
ENSG00000110848.8	430.9399573	-0.338301956	0.134575009	-2.513854244	0.011941978	0.068338223	CD69	protein_coding
ENSG00000110851.12	2201.203686	0.016050217	0.045208697	0.355024975	0.722570881	0.855233249	PRDM4	protein_coding
ENSG00000110852.4	599.0815502	-0.095849558	0.100827529	-0.950628853	0.341792816	0.571604772	CLEC2B	protein_coding
ENSG00000110871.15	1040.082188	-0.068401099	0.051001597	-1.341156005	0.179869807	0.392832485	COQ5	protein_coding
ENSG00000110876.10	1564.444766	0.020017715	0.106124687	0.188624489	0.850387138	0.926664674	SELPLG	protein_coding

ENSG00000110880.11	7708.490032	0.003234894	0.058552508	0.055247752	0.955941086	0.97954346	CORO1C	protein_coding
ENSG00000110881.12	609.2490764	-0.028810149	0.138633962	-0.207814513	0.8353738	0.919344369	ASIC1	protein_coding
ENSG00000110887.8	11.0460558	0.441675826	0.243651551	1.812735537	0.069872621	0.219526978	DAO	protein_coding
ENSG00000110888.17	1310.321214	0.300422865	0.098902089	3.037578556	0.002384872	0.021696364	CAPRIN2	protein_coding
ENSG00000110900.16	805.0523642	0.111287805	0.134781729	0.825689106	0.408980474	0.629868956	TSPAN11	protein_coding
ENSG00000110906.13	3976.902772	0.09092576	0.059358853	1.531797803	0.125572338	0.315902655	KCTD10	protein_coding
ENSG00000110911.16	4366.992966	-0.163294199	0.070455146	-2.317704373	0.020465393	0.098674534	SLC11A2	protein_coding
ENSG00000110917.8	19658.82255	-0.182184799	0.072727839	-2.505021481	0.012244396	0.069448657	MLEC	protein_coding
ENSG00000110921.14	1046.245086	-0.11002012	0.073889856	-1.488974615	0.136494053	0.332457736	MVK	protein_coding
ENSG00000110925.7	2047.424474	0.067662248	0.042905504	1.577006248	0.114794088	0.299031501	CSRNP2	protein_coding
ENSG00000110931.19	3615.072539	0.066139345	0.038078998	1.736898239	0.082405134	0.244567651	CAMKK2	protein_coding
ENSG00000110934.13	744.8770916	-0.425252727	0.101983117	-4.169834558	3.04821E-05	0.000772193	BIN2	protein_coding
ENSG00000110944.9	161.9973161	0.257123822	0.138599207	1.855160841	0.063573262	0.207208029	IL23A	protein_coding
ENSG00000110955.9	39035.41107	-0.084045694	0.065714686	-1.278948424	0.200915219	0.419567638	ATP5F1B	protein_coding
ENSG00000110958.16	17625.52477	-0.070772464	0.053464	-1.323740534	0.185589232	0.400208924	PTGES3	protein_coding
ENSG00000110975.8	8.545860186	-0.252921683	0.257780719	-0.981150506	0.326518527	0.556264821	SYT10	protein_coding
ENSG00000110987.9	1579.826671	-0.083642097	0.067945747	-1.231012979	0.21831801	0.439902053	BCL7A	protein_coding
ENSG00000111011.18	3393.12529	-0.014861131	0.040597892	-0.366056718	0.714322754	0.850725407	RSRC2	protein_coding
ENSG00000111012.10	290.3710912	0.178075599	0.120260686	1.480746572	0.138674118	0.335517298	CYP27B1	protein_coding
ENSG00000111046.4	1.171012485	0.46123292	0.310227624	1.486756445	0.137079149	0.333249543	MYF6	protein_coding
ENSG00000111049.4	0.339476952	0.028789509	0.667729875	0.043115502	0.965609462		MYF5	protein_coding
ENSG00000111052.7	247.5089755	0.139128819	0.135932751	1.023512122	0.306065772	0.535618173	LIN7A	protein_coding
ENSG00000111057.11	46850.5904	-0.083878485	0.103385844	-0.811314988	0.417184805	0.6375051	KRT18	protein_coding
ENSG00000111058.8	296.1067432	0.14986954	0.149847097	1.000149777	0.31723803	0.546608739	ACSS3	protein_coding
ENSG00000111077.18	4503.17263	0.135419324	0.09979254	1.357008482	0.174778516	0.386154624	TNS2	protein_coding
ENSG00000111087.10	358.9406574	0.393620689	0.137801435	2.856433881	0.004284292	0.033291121	GLI1	protein_coding
ENSG00000111110.12	2513.648528	-0.040734761	0.103140036	-0.394946155	0.692882627	0.837765949	PPM1H	protein_coding
ENSG00000111142.14	5946.469874	-0.12470374	0.048586896	-2.566612594	0.010269728	0.061557624	METAP2	protein_coding
ENSG00000111144.10	5398.571718	-0.236303573	0.056260925	-4.200136643	2.66754E-05	0.000696068	LTA4H	protein_coding
ENSG00000111145.8	4135.101923	0.09384135	0.071347185	1.31527754	0.188416644	0.403769946	ELK3	protein_coding
ENSG00000111181.12	158.0573769	-0.06548462	0.150200557	-0.435981202	0.662850368	0.819503234	SLC6A12	protein_coding
ENSG00000111186.13	485.5106819	0.244221398	0.126303889	1.933601569	0.053162134	0.184917491	WNT5B	protein_coding
ENSG00000111196.10	1162.663337	-0.150237717	0.064466431	-2.330479808	0.019780806	0.096440835	MAGOHB	protein_coding
ENSG00000111199.12	333.3509009	0.806076738	0.126171166	6.388755575	1.67241E-10	4.21264E-08	TRPV4	protein_coding
ENSG00000111203.13	1287.133904	-0.073063001	0.058135374	-1.25677356	0.208835643	0.428772904	ITFG2	protein_coding
ENSG00000111206.13	4335.777228	0.085924845	0.098620053	0.871271531	0.383605911	0.608532755	FOXM1	protein_coding
ENSG00000111215.12	21.16378482	0.056110036	0.129982397	0.431674111	0.665978286	0.821389555	PRR4	protein_coding
ENSG00000111218.12	6.196084057	0.063998667	0.248452928	0.2575887	0.796724351	0.898288669	PRMT8	protein_coding
ENSG00000111224.14	656.5305581	-0.009479102	0.072514177	-0.130720673	0.895996278	0.950666646	PARP11	protein_coding
ENSG00000111229.16	9355.888986	-0.154550883	0.055371211	-2.791177557	0.005251665	0.038482763	ARPC3	protein_coding
ENSG00000111231.9	940.6132596	-0.100809624	0.051558141	-1.955261025	0.050552269	0.179157143	GPN3	protein_coding
ENSG00000111237.19	3955.720475	-0.190019018	0.053630132	-3.543139114	0.000395394	0.005689375	VPS29	protein_coding

ENSG00000111241.2	1.316505562	0.129185874	0.369851245	0.34929144	0.726870524	0.857420736	FGF6	protein_coding
ENSG00000111245.17	3.536577441	-0.246336881	0.192421183	-1.280196274	0.200476116	0.419008932	MYL2	protein_coding
ENSG00000111247.15	948.9428374	-0.106008937	0.087918171	-1.205768214	0.227906875	0.451308637	RAD51AP1	protein_coding
ENSG00000111249.14	39.62904197	-0.938227492	0.190532967	-4.924226529	8.46947E-07	4.58477E-05	CUX2	protein_coding
ENSG00000111252.11	3534.739428	-0.0414847	0.067058286	-0.618636449	0.536155885	0.732668475	SH2B3	protein_coding
ENSG00000111254.8	68.387747	-0.03269156	0.120863888	-0.270482447	0.786789119	0.892258092	AKAP3	protein_coding
ENSG00000111261.14	4974.689581	-0.066170223	0.085042105	-0.778087786	0.436517263	0.653995488	MANSC1	protein_coding
ENSG00000111262.6	38.27246378	-0.385703324	0.291653992	-1.322468867	0.18601207	0.40084993	KCNA1	protein_coding
ENSG00000111266.9	3947.562094	-0.178196318	0.077467667	-2.300266999	0.021433098	0.101819483	DUSP16	protein_coding
ENSG00000111269.3	3097.850042	-0.079673942	0.056343137	-1.414084231	0.157337172	0.362396466	CREBL2	protein_coding
ENSG00000111271.15	1672.796203	-0.166293903	0.046860296	-3.548716429	0.000387114	0.005604404	ACAD10	protein_coding
ENSG00000111275.13	8980.207647	-0.306611882	0.082891754	-3.698943122	0.000216499	0.003599535	ALDH2	protein_coding
ENSG00000111276.11	3174.920987	-0.074734892	0.06099591	-1.225244318	0.220483181	0.442520062	CDKN1B	protein_coding
ENSG00000111291.8	82.8197769	0.38121804	0.108963181	3.498595005	0.000467716	0.006480661	GPRC5D	protein_coding
ENSG00000111300.10	2378.469512	-0.09514806	0.052524632	-1.8114941	0.070064402	0.219863907	NAA25	protein_coding
ENSG00000111305.19	6.879243543	-0.59019404	0.216895632	-2.721096935	0.006506568	0.044707775	GSG1	protein_coding
ENSG00000111319.13	8846.301391	0.523078822	0.15555995	3.362554586	0.000772249	0.009460859	SCNN1A	protein_coding
ENSG00000111321.11	9429.721699	0.006103843	0.060624247	0.100683202	0.919801946	0.962150061	LTBR	protein_coding
ENSG00000111325.16	31.6913548	0.056714985	0.076712064	0.739322892	0.459710952	0.673010285	OGFOD2	protein_coding
ENSG00000111328.7	7459.750467	0.072981092	0.057510146	1.269012453	0.204436622	0.423862151	CDK2AP1	protein_coding
ENSG00000111331.13	11992.88459	0.100512712	0.097725943	1.028516159	0.303707094	0.533203498	OAS3	protein_coding
ENSG00000111335.13	7910.186303	0.035766685	0.117020366	0.305644958	0.759874986	0.877051311	OAS2	protein_coding
ENSG00000111339.12	32.66303114	-0.290332453	0.170513102	-1.70269879	0.088624451	0.255879396	ART4	protein_coding
ENSG00000111341.10	11561.00619	0.175933648	0.171288722	1.027117526	0.304365127	0.533828001	MGP	protein_coding
ENSG00000111344.11	1378.288978	0.029267892	0.152845666	0.191486567	0.848144404	0.925563174	RASAL1	protein_coding
ENSG00000111348.9	6494.475981	-0.3352704	0.099273022	-3.377255928	0.000732129	0.009066207	ARHGDIB	protein_coding
ENSG00000111358.14	1699.146026	-0.076987489	0.050318678	-1.529998239	0.126017165	0.316528329	GTF2H3	protein_coding
ENSG00000111361.13	2860.753343	-0.06394671	0.037598759	-1.700766517	0.088986839	0.256489617	EIF2B1	protein_coding
ENSG00000111364.16	1176.886252	-0.066370922	0.053006134	-1.252136622	0.210520085	0.430812789	DDX55	protein_coding
ENSG00000111371.16	11325.98687	0.250128199	0.090073928	2.776921185	0.005487648	0.039690499	SLC38A1	protein_coding
ENSG00000111404.7	62.47946188	-0.439611355	0.187674091	-2.342418989	0.019159193	0.094211255	RERGL	protein_coding
ENSG00000111405.9	12.67508384	0.056812999	0.168565747	0.337037623	0.736088538	0.863130808	ENDOU	protein_coding
ENSG00000111412.6	4125.344803	-0.040996092	0.066274716	-0.618578168	0.536194288	0.732675002	SPRING1	protein_coding
ENSG00000111424.12	4056.233614	-0.00106973	0.105048817	-0.010183169	0.991875147	0.996287647	VDR	protein_coding
ENSG00000111432.4	243.9073841	0.973809938	0.190840171	5.10275135	3.34751E-07	2.13892E-05	FZD10	protein_coding
ENSG00000111445.14	1156.631429	-0.064283305	0.059287188	-1.084269758	0.27824521	0.50713022	RFC5	protein_coding
ENSG00000111450.14	897.432788	0.036030703	0.077537136	0.464689623	0.642153741	0.806403842	STX2	protein_coding
ENSG00000111452.13	615.8261357	0.209883549	0.164305531	1.27739795	0.201461788	0.420368255	ADGRD1	protein_coding
ENSG00000111481.10	10331.27971	-0.1047677	0.050409563	-2.078329879	0.037678986	0.148028957	COPZ1	protein_coding
ENSG00000111490.15	349.7340767	0.176977688	0.100677233	1.757871984	0.078769294	0.237874533	TBC1D30	protein_coding
ENSG00000111530.13	8529.656594	0.080533772	0.062196431	1.294829468	0.195379064	0.412451145	CAND1	protein_coding
ENSG00000111536.5	12.77341493	-0.735909369	0.165236112	-4.453683634	8.44094E-06	0.000282512	IL26	protein_coding

ENSG00000111537.5	55.27115689	-0.846646978	0.187898836	-4.505865997	6.61028E-06	0.000235148	IFNG	protein_coding
ENSG00000111540.16	10663.9924	0.01630085	0.045974723	0.354561141	0.722918392	0.855366355	RAB5B	protein_coding
ENSG00000111554.14	709.9735826	-0.041650468	0.076246428	-0.54626123	0.584886382	0.767490556	MDM1	protein_coding
ENSG00000111581.10	2572.123577	0.104128278	0.074612788	1.395582197	0.162840347	0.369891524	NUP107	protein_coding
ENSG00000111596.14	4720.390053	-0.001694587	0.054797975	-0.03092426	0.975329943	0.988550686	CNOT2	protein_coding
ENSG00000111602.12	2834.822558	0.021558127	0.075308692	0.286263463	0.77467634	0.886001165	TIMELESS	protein_coding
ENSG00000111605.18	5021.696452	0.079042322	0.050255291	1.572815906	0.115761452	0.300683992	CPSF6	protein_coding
ENSG00000111615.14	2016.229534	0.024671329	0.05437853	0.453696136	0.650047549	0.811432885	KRR1	protein_coding
ENSG00000111639.8	3419.594662	-0.186093933	0.064080946	-2.90404473	0.003683755	0.029896363	MRPL51	protein_coding
ENSG00000111640.15	105011.0776	0.010092911	0.079641971	0.126728543	0.899155254	0.951977512	GAPDH	protein_coding
ENSG00000111641.12	35.45214818	0.003609326	0.066098214	0.054605502	0.956452756	0.979676141	NOP2	protein_coding
ENSG00000111642.16	1696.961703	-0.15283002	0.068462075	-2.232331124	0.025593086	0.114739704	CHD4	protein_coding
ENSG00000111644.8	323.530364	-0.109668383	0.123120533	-0.890739996	0.373068672	0.59941207	ACRBP	protein_coding
ENSG00000111647.13	1641.103067	-0.053325848	0.061046098	-0.873534097	0.382372029	0.60763715	UHRF1BP1L	protein_coding
ENSG00000111652.10	3464.161949	0.06347294	0.049719275	1.276626437	0.201734164	0.420646295	COPS7A	protein_coding
ENSG00000111653.20	871.492683	-0.039342576	0.056013021	-0.702382684	0.482440541	0.690818371	ING4	protein_coding
ENSG00000111664.11	59.96924711	-0.066948193	0.116271903	-0.575789944	0.564757171	0.753368337	GNB3	protein_coding
ENSG00000111665.12	1434.870373	-0.236828282	0.094309708	-2.511176084	0.012032966	0.068642565	CDCA3	protein_coding
ENSG00000111666.11	3371.238579	-0.447245213	0.071500581	-6.255127002	3.97193E-10	8.44315E-08	CHPT1	protein_coding
ENSG00000111667.14	5084.094618	-0.021005171	0.049613539	-0.423375789	0.672021107	0.825522819	USP5	protein_coding
ENSG00000111669.15	26178.67864	-0.037471685	0.070867976	-0.528753425	0.596976511	0.77634204	TPI1	protein_coding
ENSG00000111670.16	3822.652643	0.048825184	0.051621561	0.945829272	0.344235673	0.573950028	GNPTAB	protein_coding
ENSG00000111671.10	574.0768463	-0.068795338	0.084896698	-0.810341745	0.417743789	0.63795413	SPSB2	protein_coding
ENSG00000111674.9	1663.514281	0.118385735	0.110585217	1.07053853	0.284376976	0.513436173	ENO2	protein_coding
ENSG00000111676.15	12616.23538	0.061645002	0.053788693	1.146058755	0.251770842	0.478411807	ATN1	protein_coding
ENSG00000111678.11	1349.11411	0.015144828	0.093807217	0.161446294	0.871741908	0.938065417	C12orf57	protein_coding
ENSG00000111679.17	3250.044908	-0.272438613	0.071563109	-3.806970081	0.00014068	0.002584486	PTPN6	protein_coding
ENSG00000111684.11	2016.251144	-0.336211557	0.083208664	-4.040583542	5.33184E-05	0.001211797	LPCAT3	protein_coding
ENSG00000111696.12	1335.405603	0.021991324	0.10300389	0.213499942	0.830937035	0.916725567	NT5DC3	protein_coding
ENSG00000111700.13	130.9053044	1.168418245	0.315866714	3.699086328	0.000216377	0.003598878	SLCO1B3	protein_coding
ENSG00000111701.7	201.8782885	-0.433451273	0.21890966	-1.980046345	0.047698321	0.172723097	APOBEC1	protein_coding
ENSG00000111704.11	12.12729394	-0.109449371	0.18990479	-0.57633813	0.564386655	0.753155022	NANOG	protein_coding
ENSG00000111707.12	3371.539356	-0.085332643	0.043999577	-1.939396908	0.052453027	0.183284865	SUDS3	protein_coding
ENSG00000111711.10	2868.591148	0.12655637	0.070125605	1.804709848	0.071120098	0.221942173	GOLT1B	protein_coding
ENSG00000111713.3	0.535514186	0.429414798	0.598345496	0.71767031	0.472960594		GYS2	protein_coding
ENSG00000111716.14	838.4203814	-0.270028257	0.13747715	-1.964168285	0.049510579	0.176744697	LDHB	protein_coding
ENSG00000111725.11	3033.26546	0.008466544	0.062032587	0.136485425	0.891437546	0.948277162	PRKAB1	protein_coding
ENSG00000111726.13	2393.612782	0.097291134	0.0887915	1.095725754	0.273198809	0.501672264	CMAS	protein_coding
ENSG00000111727.12	974.6873073	-0.15148733	0.070173506	-2.15875391	0.030869264	0.129957484	HCFC2	protein_coding
ENSG00000111728.10	223.844657	-0.312842665	0.121584336	-2.57305074	0.010080643	0.060825833	ST8SIA1	protein_coding
ENSG00000111729.14	10.31057624	-0.04133152	0.14651887	-0.28209008	0.777874439	0.887483557	CLEC4A	protein_coding
ENSG00000111731.12	3591.278857	0.039428267	0.080023428	0.492709043	0.622218187	0.793538059	C2CD5	protein_coding

ENSG00000111732.11	25.89473185	-1.215643041	0.210505108	-5.774886199	7.70051E-09	9.6155E-07	AICDA	protein_coding
ENSG00000111737.12	3521.087629	0.068210333	0.048233816	1.41416001	0.157314926	0.362396466	RAB35	protein_coding
ENSG00000111752.11	620.6337713	0.034180956	0.090009134	0.379749861	0.704131104	0.844786286	PHC1	protein_coding
ENSG00000111775.3	3552.945757	-0.167217521	0.079151485	-2.112626452	0.034632753	0.139817628	COX6A1	protein_coding
ENSG00000111780.8	0						AL021546.1	protein_coding
ENSG00000111783.13	10.06083714	0.29755857	0.209675526	1.419138303	0.155858699	0.360463567	RFX4	protein_coding
ENSG00000111785.21	993.0001415	-0.062617717	0.051546474	-1.214781758	0.22444934	0.447348559	RIC8B	protein_coding
ENSG00000111786.9	2671.598139	-0.056232571	0.056124542	-1.001924815	0.316379907	0.545583189	SRSF9	protein_coding
ENSG00000111788.10	277.1189402	0.071424896	0.088064593	0.811051224	0.417336255	0.637633792	AC009533.1	unprocessed_pseudogene
ENSG00000111790.14	1960.13445	0.076252647	0.060763498	1.254908764	0.209511882	0.429642319	FGFR1OP2	protein_coding
ENSG00000111796.4	188.448722	-0.687414371	0.124302446	-5.530175723	3.1991E-08	3.1398E-06	KLRB1	protein_coding
ENSG00000111799.21	19674.69642	0.80694405	0.118466968	6.81155317	9.65506E-12	3.8249E-09	COL12A1	protein_coding
ENSG00000111801.16	2191.778567	-0.173627156	0.085233091	-2.037086215	0.041641403	0.158040182	BTN3A3	protein_coding
ENSG00000111802.14	2635.627319	-0.052861171	0.053977685	-0.979315267	0.327424229	0.557038255	TDP2	protein_coding
ENSG00000111816.8	3556.1112	0.182057384	0.090956998	2.001576434	0.045330306	0.167035238	FRK	protein_coding
ENSG00000111817.19	1404.465413	0.282970496	0.088898427	3.183076539	0.001457191	0.015224169	DSE	protein_coding
ENSG00000111832.13	2287.685523	-0.063686012	0.053540813	-1.189485337	0.234248738	0.458873003	RWDD1	protein_coding
ENSG00000111834.13	41.13838502	-0.003583048	0.109927076	-0.032594771	0.97399774	0.988009338	RSPH4A	protein_coding
ENSG00000111837.12	127.6917145	-0.206863175	0.103683634	-1.995138158	0.046027814	0.168587379	MAK	protein_coding
ENSG00000111843.14	4556.909557	-0.105818218	0.052805333	-2.003930507	0.045077505	0.166483807	TMEM14C	protein_coding
ENSG00000111845.5	1294.903506	-0.142353394	0.064488561	-2.207420859	0.02728467	0.119753738	PAK1IP1	protein_coding
ENSG00000111846.19	653.5350632	-0.09965079	0.122086349	-0.816232041	0.414367435	0.634851604	GCNT2	protein_coding
ENSG00000111850.11	350.6757271	-0.050683789	0.0612645	-0.827294578	0.408070116	0.629089449	SMIM8	protein_coding
ENSG00000111859.17	5066.153505	0.113738133	0.086753723	1.31104613	0.189842179	0.405332128	NEDD9	protein_coding
ENSG00000111860.14	432.200526	-0.176666915	0.093817623	-1.883088788	0.059688336	0.199206451	CEP85L	protein_coding
ENSG00000111863.13	890.8014069	-0.72154684	0.190513555	-3.787377959	0.000152245	0.002759733	ADTRP	protein_coding
ENSG00000111875.8	1041.044676	-0.123997388	0.0541829	-2.288496708	0.022108612	0.103883971	ASF1A	protein_coding
ENSG00000111877.17	887.3102492	0.059195939	0.050123654	1.180998089	0.237603482	0.462812755	MCM9	protein_coding
ENSG00000111879.20	164.5528054	0.081365844	0.149826284	0.543067888	0.587083062	0.769145529	FAM184A	protein_coding
ENSG00000111880.16	1350.312237	0.067889596	0.052858118	1.284374077	0.199011099	0.417038069	RNGTT	protein_coding
ENSG00000111885.7	6387.044853	-0.144189279	0.09277649	-1.554157504	0.120146851	0.307852023	MAN1A1	protein_coding
ENSG00000111886.11	62.47101615	0.359994716	0.122310916	2.943275437	0.003247593	0.027262642	GABRR2	protein_coding
ENSG00000111897.7	9339.683233	0.132619239	0.05152492	2.573885397	0.010056358	0.06073817	SERINC1	protein_coding
ENSG00000111906.18	2099.682198	0.052612356	0.065065274	0.808608846	0.418740175	0.638804152	HDDC2	protein_coding
ENSG00000111907.21	713.7832011	0.428039542	0.157486078	2.717951617	0.006568744	0.045035584	TPD52L1	protein_coding
ENSG00000111911.7	1970.514303	0.065018762	0.054188025	1.199873264	0.230188565	0.453990816	HINT3	protein_coding
ENSG00000111912.20	9321.979579	0.214610056	0.084935933	2.526728657	0.011513042	0.066653887	NCOA7	protein_coding
ENSG00000111913.20	766.711737	-0.570925051	0.137081874	-4.164847136	3.11561E-05	0.000785246	RIPOR2	protein_coding
ENSG00000111961.18	2393.108818	0.188773802	0.080526407	2.344247179	0.019065531	0.09394562	SASH1	protein_coding
ENSG00000111962.8	682.415342	0.382481223	0.144976013	2.638237969	0.008333808	0.05318722	UST	protein_coding
ENSG00000111981.5	79.00889477	0.685118297	0.184753949	3.708274163	0.000208677	0.00350424	ULBP1	protein_coding
ENSG00000112029.10	814.198259	0.000212373	0.071865988	0.002955122	0.997642157	0.998864405	FBXO5	protein_coding

ENSG00000112031.16	546.9938021	0.000251592	0.049768602	0.005055245	0.995966515	0.99811956	MTRF1L	protein_coding
ENSG00000112033.14	4148.32319	0.301920761	0.068881253	4.383206589	1.16945E-05	0.000367951	PPARD	protein_coding
ENSG00000112038.18	18.73005881	-0.358594098	0.226691262	-1.581861144	0.113681273	0.297386034	OPRM1	protein_coding
ENSG00000112039.5	532.9899391	0.098038232	0.066602789	1.471983886	0.141025229	0.338923245	FANCE	protein_coding
ENSG00000112041.13	17.27355734	0.239624009	0.140884945	1.700848938	0.088971357	0.256489617	TULP1	protein_coding
ENSG00000112053.14	60.73835019	0.006900625	0.11962451	0.057685715	0.953998972	0.978519789	SLC26A8	protein_coding
ENSG00000112062.11	3842.601204	0.127327578	0.048210556	2.641072568	0.008264402	0.052922532	MAPK14	protein_coding
ENSG00000112077.17	5.998802533	0.212015569	0.275944636	0.768326474	0.44229325	0.658641048	RHAG	protein_coding
ENSG00000112078.14	4588.432265	0.15499908	0.051504789	3.009411007	0.002617548	0.023231134	KCTD20	protein_coding
ENSG00000112079.9	4968.327554	0.026778011	0.048106899	0.556635559	0.577776454	0.762319231	STK38	protein_coding
ENSG00000112081.17	13067.80273	-0.047089935	0.037785305	-1.246249952	0.212672646	0.433282341	SRSF3	protein_coding
ENSG00000112096.19	22947.19892	0.154015989	0.08788421	1.752487602	0.07969	0.239600662	SOD2	protein_coding
ENSG00000112110.10	3234.040912	-0.051817038	0.060293883	-0.859407877	0.390115525	0.614593883	MRPL18	protein_coding
ENSG00000112115.7	25.30214682	-1.255242208	0.245345686	-5.116218778	3.11721E-07	2.02745E-05	IL17A	protein_coding
ENSG00000112116.10	9.861873692	-1.208406603	0.261060366	-4.628839757	3.6772E-06	0.000145939	IL17F	protein_coding
ENSG00000112118.20	7883.582972	0.056548638	0.068869652	0.821096615	0.411591238	0.632436932	MCM3	protein_coding
ENSG00000112130.17	933.7170624	0.135129234	0.04825576	2.800271607	0.005105963	0.037750683	RNF8	protein_coding
ENSG00000112137.18	300.4444395	-0.408521526	0.11359741	-3.596222197	0.000322872	0.004912088	PHACTR1	protein_coding
ENSG00000112139.16	395.786762	0.096209759	0.122812677	0.783386217	0.433400347	0.651336563	MDGA1	protein_coding
ENSG00000112144.16	3137.655164	-0.041577896	0.069627625	-0.597146549	0.550409542	0.742906376	CILK1	protein_coding
ENSG00000112146.17	2558.508526	-0.010372467	0.050691214	-0.204620612	0.83786855	0.920202347	FBXO9	protein_coding
ENSG00000112149.10	801.2665502	-0.248669074	0.09323942	-2.666995072	0.007653279	0.050143882	CD83	protein_coding
ENSG00000112159.12	4199.101295	0.035266537	0.061875433	0.569960253	0.568704657	0.755755325	MDN1	protein_coding
ENSG00000112164.6	80.81996955	-0.216535982	0.226872833	-0.954437683	0.339862139	0.569709665	GLP1R	protein_coding
ENSG00000112167.10	946.7195887	0.02040306	0.046434991	0.439389769	0.660379138	0.817999121	SAYSD1	protein_coding
ENSG00000112175.8	58.91426892	-0.057176809	0.201771017	-0.283374736	0.776889593	0.886870767	BMP5	protein_coding
ENSG00000112182.15	298.3664078	-0.341648426	0.144567253	-2.363249066	0.01811549	0.09081085	BACH2	protein_coding
ENSG00000112183.15	121.0822072	-0.509364367	0.182102171	-2.797135061	0.005155798	0.038003077	RBM24	protein_coding
ENSG00000112186.12	905.7269576	-0.413426139	0.147987767	-2.793650764	0.005211673	0.038311247	CAP2	protein_coding
ENSG00000112195.9	154.521351	0.596821467	0.19229177	3.103728608	0.001910985	0.018505558	TREML2	protein_coding
ENSG00000112200.17	2313.380306	0.129897803	0.052683196	2.465640133	0.013676867	0.075252533	ZNF451	protein_coding
ENSG00000112208.11	1754.711374	-0.129788364	0.136523526	-0.950666658	0.341773619	0.571594604	BAG2	protein_coding
ENSG00000112210.12	1126.379461	-0.166369137	0.134286586	-1.238911066	0.215378435	0.436496422	RAB23	protein_coding
ENSG00000112212.12	34.8969325	-0.541112819	0.142011079	-3.81035637	0.000138767	0.002556885	TSPO2	protein_coding
ENSG00000112214.10	148.9498495	0.027561606	0.124426433	0.221509247	0.824695937	0.913424534	FHL5	protein_coding
ENSG00000112218.9	192.9823025	0.341751759	0.100097794	3.414178736	0.000639746	0.008179318	GPR63	protein_coding
ENSG00000112232.10	11.81852583	-0.448051787	0.210009217	-2.133486296	0.032884855	0.135292986	KHDRBS2	protein_coding
ENSG00000112234.9	1761.4403	0.14292024	0.055608006	2.570137849	0.010165805	0.061136531	FBXL4	protein_coding
ENSG00000112237.13	2776.535389	-0.006314863	0.056811262	-0.111155119	0.911493341	0.958265302	CCNC	protein_coding
ENSG00000112238.12	7.553605953	0.964385395	0.380604507	2.533825471	0.011282491	0.06571199	PRDM13	protein_coding
ENSG00000112242.15	1611.71593	0.062297437	0.059306526	1.050431406	0.293519812	0.523501738	E2F3	protein_coding
ENSG00000112245.12	61.3529549	0.438738794	0.126859159	3.458471576	0.00054325	0.007250593	PTP4A1	protein_coding

ENSG00000112246.10	3.245301365	0.159730957	0.279777333	0.570921721	0.568052706	0.75543313	SIM1	protein_coding
ENSG00000112249.14	4493.238369	0.152794727	0.063995589	2.387582148	0.016959612	0.087113802	ASCC3	protein_coding
ENSG00000112273.7	1.681660508	-1.443757027	0.497947394	-2.899416776	0.003738576	0.030208774	HDGFL1	protein_coding
ENSG00000112276.14	615.3274457	-0.393031348	0.171286023	-2.294590898	0.02175658	0.102795891	BVES	protein_coding
ENSG00000112280.17	368.0139929	0.4810869	0.181620413	2.648859192	0.008076397	0.052116862	COL9A1	protein_coding
ENSG00000112282.18	2516.684953	0.068708802	0.046640243	1.4731656	0.140706388	0.338535903	MED23	protein_coding
ENSG00000112290.13	635.4249936	0.24844118	0.120488258	2.061953451	0.039212168	0.152078021	WASF1	protein_coding
ENSG00000112293.15	139.8918341	-0.367658063	0.107582759	-3.417444069	0.000632121	0.00811311	GPLD1	protein_coding
ENSG00000112294.14	2150.212973	-0.01664194	0.104380553	-0.159435257	0.873325963	0.938755898	ALDH5A1	protein_coding
ENSG00000112297.18	5680.808864	-0.00216488	0.120409602	-0.017979297	0.985655369	0.993327193	CRYBG1	protein_coding
ENSG00000112299.8	3775.353433	0.356373729	0.207924434	1.71395791	0.086536456	0.252055287	VNN1	protein_coding
ENSG00000112303.14	424.646521	0.226879711	0.150894567	1.503564481	0.132693542	0.326743883	VNN2	protein_coding
ENSG00000112304.11	958.7151066	-0.118853977	0.053845673	-2.207307848	0.027292559	0.119753738	ACOT13	protein_coding
ENSG00000112305.15	1287.658839	-0.05743748	0.073497387	-0.781490095	0.4345143	0.652250419	SMAP1	protein_coding
ENSG00000112306.8	38744.72774	-0.052357155	0.065434847	-0.800141791	0.42362865	0.642751495	RPS12	protein_coding
ENSG00000112308.13	5232.530816	0.031517157	0.046179732	0.682488949	0.494929828	0.701520854	C6orf62	protein_coding
ENSG00000112309.11	38.36885537	0.029686014	0.092627148	0.320489345	0.748597406	0.870746027	B3GAT2	protein_coding
ENSG00000112312.10	1310.780536	-0.048602714	0.082411274	-0.589758071	0.555352857	0.746356559	GMNN	protein_coding
ENSG00000112319.19	132.5858267	1.150152743	0.166983167	6.887836449	5.66473E-12	2.62504E-09	EYA4	protein_coding
ENSG00000112320.12	842.9253609	-0.042996057	0.13662741	-0.314695688	0.752992719	0.873100132	SOBP	protein_coding
ENSG00000112333.12	15.72683237	1.530644081	0.263086999	5.818014896	5.95506E-09	7.86375E-07	NR2E1	protein_coding
ENSG00000112335.15	8861.559147	0.065662239	0.055761465	1.177555855	0.238973718	0.464381614	SNX3	protein_coding
ENSG00000112337.10	3.681500403	-1.016438355	0.368486687	-2.758412694	0.005808282	0.041392836	SLC17A2	protein_coding
ENSG00000112339.15	2540.663577	0.042121214	0.046996649	0.896259958	0.37011394	0.5967795	HBS1L	protein_coding
ENSG00000112343.11	2807.632045	0.00583934	0.062534126	0.09337845	0.92560291	0.964905938	TRIM38	protein_coding
ENSG00000112357.14	572.3762865	-0.050578794	0.065147542	-0.776373019	0.437528771	0.654430948	PEX7	protein_coding
ENSG00000112365.5	1213.471465	-0.068884785	0.051337939	-1.34179101	0.179663768	0.392638316	ZBTB24	protein_coding
ENSG00000112367.12	1182.977702	0.0251025	0.047479966	0.52869667	0.597015887	0.776370078	FIG4	protein_coding
ENSG00000112378.12	20305.5258	0.438704896	0.103548374	4.236714479	2.26814E-05	0.000615638	PERP	protein_coding
ENSG00000112379.9	5785.615252	0.367785892	0.095825081	3.838096322	0.000123992	0.002331978	ARFGEF3	protein_coding
ENSG00000112394.17	435.7759696	0.201172186	0.137192612	1.466348535	0.142553359	0.341246305	SLC16A10	protein_coding
ENSG00000112406.5	2546.665853	0.091262405	0.051003556	1.78933418	0.073561013	0.227087578	HECA	protein_coding
ENSG00000112414.15	3660.086773	0.266627825	0.120569511	2.21140339	0.027007915	0.118989377	ADGRG6	protein_coding
ENSG00000112419.14	3802.70787	-0.026050483	0.057846319	-0.450339506	0.652465657	0.813077181	PHACTR2	protein_coding
ENSG00000112425.16	374.6466458	-0.177562874	0.099280775	-1.788492033	0.07369666	0.227377467	EPM2A	protein_coding
ENSG00000112462.8	1.55880126	-0.387554559	0.41469833	-0.934545743	0.350022452	0.579131376	OR12D3	protein_coding
ENSG00000112473.18	7899.192814	0.137166344	0.064582086	2.12390699	0.033677922	0.137336512	SLC39A7	protein_coding
ENSG00000112486.17	13.83001192	-0.47759348	0.153598633	-3.109360231	0.00187493	0.018253756	CCR6	protein_coding
ENSG00000112494.10	271.9766557	-0.570234675	0.235107934	-2.425416555	0.015290831	0.081121076	UNC93A	protein_coding
ENSG00000112499.13	13.47144972	-0.320095847	0.212365427	-1.50728794	0.131736868	0.325251982	SLC22A2	protein_coding
ENSG00000112511.18	2196.187896	0.077986901	0.060175969	1.295980798	0.1949821	0.41200286	PHF1	protein_coding
ENSG00000112514.18	5551.941338	-0.024505849	0.063955638	-0.383169479	0.701594106	0.843070023	CUTA	protein_coding

ENSG00000112530.11	43.39579444	-0.269527948	0.164262779	-1.640833975	0.100831883	0.276825424	PACRG	protein_coding
ENSG00000112531.17	4217.412009	0.122970825	0.083713739	1.468944361	0.141847881	0.340174185	QKI	protein_coding
ENSG00000112539.15	3.84013293	0.422914606	0.283591744	1.491279681	0.135888081	0.331611968	C6orf118	protein_coding
ENSG00000112541.17	720.576026	0.87465869	0.142843349	6.123202056	9.17133E-10	1.67924E-07	PDE10A	protein_coding
ENSG00000112559.15	1712.456839	0.611325538	0.113530623	5.384675256	7.25756E-08	6.28753E-06	MDFI	protein_coding
ENSG00000112561.19	1395.573549	-0.083879634	0.084782634	-0.989349227	0.322492307	0.552351546	TFEB	protein_coding
ENSG00000112562.19	5080.497954	-0.258930774	0.17199271	-1.505475278	0.132201928	0.325910224	SMOC2	protein_coding
ENSG00000112576.13	5114.535342	0.031679896	0.080198351	0.39501929	0.692828652	0.83776553	CCND3	protein_coding
ENSG00000112578.10	1282.891818	0.071941027	0.075936185	0.947387955	0.343441127	0.573041849	BYSL	protein_coding
ENSG00000112584.14	2259.85212	0.051222425	0.047528251	1.077725844	0.281156122	0.510072453	FAM120B	protein_coding
ENSG00000112592.14	917.1688688	-0.013055645	0.040094999	-0.325617801	0.744713543	0.868624887	TBP	protein_coding
ENSG00000112599.9	52.36996815	-0.025508031	0.103231459	-0.247095515	0.804834304	0.902972222	GUCA1B	protein_coding
ENSG00000112619.8	74.11040772	0.217244152	0.119198626	1.822539066	0.068373232	0.216646672	PRPH2	protein_coding
ENSG00000112624.12	2621.129755	0.079847015	0.064393919	1.239977568	0.214983691	0.436081754	BICRAL	protein_coding
ENSG00000112640.16	2090.135353	0.084029289	0.071572255	1.174048367	0.240375653	0.465776594	PPP2R5D	protein_coding
ENSG00000112651.12	393.1456128	-0.218547413	0.108777134	-2.009130086	0.04452334	0.165150968	MRPL2	protein_coding
ENSG00000112655.16	5326.966694	0.284769473	0.125312973	2.272466023	0.023058378	0.107066056	PTK7	protein_coding
ENSG00000112658.8	6220.98076	0.026496173	0.069925492	0.378920079	0.704747213	0.84521176	SRF	protein_coding
ENSG00000112659.14	2322.531279	0.211883949	0.071780301	2.951839787	0.003158868	0.026708185	CUL9	protein_coding
ENSG00000112667.13	2838.552844	-0.189013569	0.08250926	-2.29081645	0.021974032	0.103441977	DNPH1	protein_coding
ENSG00000112679.14	1766.388871	-0.088091846	0.062247615	-1.415184285	0.157014471	0.362041356	DUSP22	protein_coding
ENSG00000112685.14	2110.098424	-0.009261117	0.04458493	-0.207718557	0.835448727	0.919344369	EXOC2	protein_coding
ENSG00000112695.11	8435.234404	-0.006462281	0.065225251	-0.099076374	0.921077631	0.962865497	COX7A2	protein_coding
ENSG00000112697.17	9216.850138	0.133260038	0.049890967	2.671025343	0.007561994	0.049680235	TMEM30A	protein_coding
ENSG00000112699.11	7988.140205	-0.284985525	0.104663746	-2.722867619	0.006471799	0.044560209	GMDS	protein_coding
ENSG00000112701.18	4534.920445	-0.005785238	0.045366888	-0.12752116	0.898527927	0.951764556	SENP6	protein_coding
ENSG00000112706.12	35.07561416	0.171631829	0.110971871	1.546624631	0.121953787	0.310528232	IMPG1	protein_coding
ENSG00000112715.25	11265.20214	0.190017562	0.102545672	1.85300421	0.063881755	0.207853002	VEGFA	protein_coding
ENSG00000112739.17	5434.756216	-0.045895375	0.047849748	-0.95915604	0.337480142	0.567222284	PRPF4B	protein_coding
ENSG00000112742.10	1356.870401	0.107173971	0.099457357	1.077587167	0.281218032	0.510142293	TTK	protein_coding
ENSG00000112759.19	5378.267548	0.121490906	0.081301242	1.494330249	0.135089323	0.330455365	SLC29A1	protein_coding
ENSG00000112761.21	190.7536874	0.975169849	0.239799178	4.066610472	4.77019E-05	0.001105106	CCN6	protein_coding
ENSG00000112763.17	1549.218964	0.02473439	0.048183925	0.513332818	0.607718531	0.783878476	BTN2A1	protein_coding
ENSG00000112769.20	6742.486672	0.130447258	0.095624963	1.364154858	0.172518832	0.383181732	LAMA4	protein_coding
ENSG00000112773.16	4352.149339	0.214755169	0.089260534	2.405936421	0.016131072	0.084285817	TENT5A	protein_coding
ENSG00000112782.19	1514.21682	0.762260983	0.144767938	5.265399178	1.39885E-07	1.06013E-05	CLIC5	protein_coding
ENSG00000112787.13	8381.602915	0.020727272	0.061749029	0.335669593	0.737120038	0.86382896	FBRSL1	protein_coding
ENSG00000112796.10	414.4000625	0.501866038	0.171075453	2.933594666	0.003350615	0.027877637	ENPP5	protein_coding
ENSG00000112799.9	397.4056659	-0.354311593	0.129041529	-2.745717567	0.006037872	0.04251977	LY86	protein_coding
ENSG00000112812.16	1124.685327	-0.014877859	0.105336517	-0.141241225	0.88767938	0.946335364	PRSS16	protein_coding
ENSG00000112818.10	1421.288461	-0.143503578	0.266406326	-0.538664301	0.59011851	0.771221039	MEP1A	protein_coding
ENSG00000112837.17	139.010299	0.709986243	0.184220983	3.853992263	0.000116207	0.002219944	TBX18	protein_coding

ENSG00000112851.15	5766.591858	0.017325093	0.070516702	0.245687788	0.805923926	0.903511934	ERBIN	protein_coding
ENSG00000112852.7	114.5108358	0.712343559	0.151030818	4.716544392	2.39884E-06	0.00010443	PCDHB2	protein_coding
ENSG00000112855.17	1282.158861	-0.021338818	0.043539985	-0.490097032	0.624065238	0.79490281	HARS2	protein_coding
ENSG00000112874.10	1068.41312	-0.225490858	0.100531668	-2.242983343	0.024897889	0.112785474	NUDT12	protein_coding
ENSG00000112877.8	669.5843184	0.003031303	0.103445548	0.029303373	0.976622637	0.989428421	CEP72	protein_coding
ENSG00000112893.9	4915.880648	-0.027825605	0.06325435	-0.439900267	0.660009343	0.817755568	MAN2A1	protein_coding
ENSG00000112902.12	1840.040984	-0.113003595	0.114638225	-0.985740967	0.324260241	0.554063778	SEMA5A	protein_coding
ENSG00000112936.19	3948.65603	0.046105151	0.242880709	0.18982632	0.849445231	0.92625018	C7	protein_coding
ENSG00000112941.14	2311.531408	0.019884563	0.054842078	0.362578575	0.716919717	0.852021945	TENT4A	protein_coding
ENSG00000112964.14	300.7717906	0.361032115	0.154349752	2.339052132	0.019332736	0.094722579	GHR	protein_coding
ENSG00000112972.15	4026.436275	0.265299852	0.098413747	2.695760105	0.007022822	0.047122244	HMGCS1	protein_coding
ENSG00000112977.16	13465.63046	0.095182043	0.063083255	1.508832136	0.131341686	0.324644166	DAP	protein_coding
ENSG00000112981.5	50.1949674	-0.103320865	0.146998991	-0.702867853	0.482138107	0.690754779	NME5	protein_coding
ENSG00000112983.18	2499.650238	0.113738201	0.045992959	2.472948128	0.013400359	0.074114412	BRD8	protein_coding
ENSG00000112984.12	1810.388112	0.128959922	0.090731194	1.421340515	0.15521779	0.359630245	KIF20A	protein_coding
ENSG00000112992.18	4558.659973	0.026579132	0.082436602	0.322419059	0.747135248	0.869834697	NNT	protein_coding
ENSG00000112996.11	3143.593991	-0.097639711	0.059685808	-1.635894942	0.101861577	0.278524264	MRPS30	protein_coding
ENSG00000113013.16	14589.85617	-0.061969134	0.057496316	-1.077793126	0.281126088	0.510039199	HSPA9	protein_coding
ENSG00000113048.17	3315.176428	-0.081498533	0.056030196	-1.454546629	0.145794816	0.346191188	MRPS27	protein_coding
ENSG00000113068.10	2294.01456	-0.043199026	0.047264766	-0.913979487	0.360727625	0.588456716	PFDN1	protein_coding
ENSG00000113070.8	1798.743367	0.208528225	0.112889446	1.847189731	0.064719647	0.209375506	HBEGF	protein_coding
ENSG00000113073.15	22.13220142	-0.006470345	0.154852775	-0.041783847	0.966671012	0.984499865	SLC4A9	protein_coding
ENSG00000113083.15	2661.465958	0.768760124	0.112376426	6.840937634	7.86765E-12	3.26522E-09	LOX	protein_coding
ENSG00000113088.6	119.1704172	-0.379070683	0.172801607	-2.193675679	0.028258734	0.1223843	GZMK	protein_coding
ENSG00000113100.10	5.56816887	-0.501610259	0.42514619	-1.17985359	0.238058451	0.463471078	CDH9	protein_coding
ENSG00000113108.19	483.7673383	0.029126839	0.052347671	0.556411369	0.577929669	0.762358646	APBB3	protein_coding
ENSG00000113119.13	635.7145761	-0.042915627	0.053592031	-0.800783737	0.423256855	0.642366314	TMCO6	protein_coding
ENSG00000113140.11	62258.22963	0.590623053	0.104629366	5.644907119	1.6527E-08	1.84194E-06	SPARC	protein_coding
ENSG00000113141.18	4764.24847	-0.034711729	0.034841035	-0.996288662	0.319109911	0.548522881	IK	protein_coding
ENSG00000113161.16	3471.238494	0.102527368	0.077764878	1.31842768	0.18736052	0.40235607	HMGCR	protein_coding
ENSG00000113163.18	2434.076104	-0.001227844	0.046569721	-0.026365717	0.978965638	0.990604566	CERT1	protein_coding
ENSG00000113194.13	4864.039088	0.102744734	0.042510715	2.416913819	0.015652723	0.082458579	FAF2	protein_coding
ENSG00000113196.3	3.72221378	1.279947395	0.373223799	3.4294367	0.000604835	0.007851331	HAND1	protein_coding
ENSG00000113205.5	112.5792093	0.664557578	0.150554191	4.414075576	1.01443E-05	0.000329647	PCDHB3	protein_coding
ENSG00000113209.9	193.3737956	0.4574924	0.186678832	2.45069243	0.014258173	0.077356623	PCDHB5	protein_coding
ENSG00000113211.5	103.1285663	0.876023508	0.186800789	4.689613527	2.73722E-06	0.000116257	PCDHB6	protein_coding
ENSG00000113212.7	95.79570507	0.530446687	0.125695115	4.220105811	2.44188E-05	0.000645298	PCDHB7	protein_coding
ENSG00000113231.14	118.7366907	-0.116705393	0.103373645	-1.128966603	0.258911922	0.486127318	PDE8B	protein_coding
ENSG00000113240.13	995.6917184	-0.134446869	0.058818246	-2.285802073	0.02226584	0.104415617	CLK4	protein_coding
ENSG00000113248.6	178.4615717	0.31818642	0.120044679	2.650566626	0.008035688	0.051939007	PCDHB15	protein_coding
ENSG00000113249.13	149.8640329	0.196797741	0.253428776	0.776540629	0.437429841	0.654382804	HAVCR1	protein_coding
ENSG00000113262.16	22.35947872	-0.032484081	0.147655736	-0.219998774	0.825872109	0.914076727	GRM6	protein_coding

ENSG00000113263.13	565.3180304	-0.773546806	0.137671167	-5.618800376	1.92288E-08	2.06347E-06	ITK	protein_coding
ENSG00000113269.14	3223.395963	-0.035553663	0.072214783	-0.492332198	0.622484522	0.79383126	RNF130	protein_coding
ENSG00000113272.14	813.2686223	-0.109975491	0.057153717	-1.9242054	0.054328843	0.187644969	THG1L	protein_coding
ENSG00000113273.17	1333.872372	0.061051907	0.077482006	0.787949497	0.430726247	0.648782159	ARSB	protein_coding
ENSG00000113282.14	9392.995668	-0.010755347	0.067064526	-0.160373111	0.87258717	0.938372516	CLINT1	protein_coding
ENSG00000113296.14	2778.42864	0.346336138	0.240144218	1.442200608	0.149245789	0.351227724	THBS4	protein_coding
ENSG00000113300.13	3081.580496	0.039509271	0.046885597	0.842673951	0.399410819	0.622233851	CNOT6	protein_coding
ENSG00000113302.5	11.88405582	-0.204407831	0.169244649	-1.207765395	0.227137516	0.450547295	IL12B	protein_coding
ENSG00000113303.12	974.0358483	-0.832805962	0.193293399	-4.308506996	1.6436E-05	0.00047717	BTNL8	protein_coding
ENSG00000113312.11	2464.515816	-0.04404248	0.044617846	-0.987104589	0.323591369	0.553354827	TTC1	protein_coding
ENSG00000113318.11	1209.939202	0.022035855	0.05310443	0.414953231	0.678176141	0.829400196	MSH3	protein_coding
ENSG00000113319.13	627.2098256	0.140851627	0.08726962	1.613982356	0.106531253	0.285909493	RASGRF2	protein_coding
ENSG00000113327.17	35.81658784	0.434809146	0.276754531	1.571100368	0.116159337	0.301190265	GABRG2	protein_coding
ENSG00000113328.19	5792.649933	-0.237427172	0.071706304	-3.311105964	0.00092928	0.010847908	CCNG1	protein_coding
ENSG00000113356.13	340.5316221	-0.02733573	0.113155932	-0.241575756	0.809108909	0.905452101	POLR3G	protein_coding
ENSG00000113360.16	3964.889224	0.039169562	0.052326497	0.748560746	0.454121999	0.66841844	DROSHA	protein_coding
ENSG00000113361.13	689.5878452	0.183192066	0.098665375	1.85670066	0.063353754	0.206721566	CDH6	protein_coding
ENSG00000113368.12	4026.805019	-0.08384708	0.085553186	-0.98005795	0.327057514	0.556725964	LMNB1	protein_coding
ENSG00000113369.9	4216.267247	0.265331127	0.072899142	3.639701648	0.000272954	0.004328429	ARRDC3	protein_coding
ENSG00000113384.14	9468.014261	0.009428689	0.050912637	0.185193489	0.853077276	0.928249811	GOLPH3	protein_coding
ENSG00000113387.12	9931.872215	-0.201269784	0.068071245	-2.956751912	0.003108982	0.026393939	SUB1	protein_coding
ENSG00000113389.16	286.6373079	0.848198021	0.175465372	4.833990962	1.33823E-06	6.58193E-05	NPR3	protein_coding
ENSG00000113391.19	1298.496762	-0.009949518	0.06246579	-0.159279464	0.873448699	0.938755898	FAM172A	protein_coding
ENSG00000113396.13	28.23268374	-0.206736485	0.220005363	-0.939688386	0.347377424	0.57656317	SLC27A6	protein_coding
ENSG00000113407.14	6283.453543	0.131471054	0.067671227	1.94279105	0.05204141	0.182490425	TARS1	protein_coding
ENSG00000113430.10	16.25783332	1.974055357	0.291545467	6.77100342	1.27892E-11	4.92204E-09	IRX4	protein_coding
ENSG00000113441.16	4884.972467	-0.136089303	0.064444259	-2.111736658	0.034709043	0.140021845	LNPEP	protein_coding
ENSG00000113448.19	2043.430814	0.013370585	0.103161188	0.129608679	0.896876036	0.951047386	PDE4D	protein_coding
ENSG00000113456.19	1554.711639	0.082478059	0.056453903	1.460980645	0.144020753	0.343390788	RAD1	protein_coding
ENSG00000113460.13	2158.743844	-0.02643192	0.074926586	-0.352770917	0.724260188	0.856074577	BRIX1	protein_coding
ENSG00000113492.14	10.63842508	-0.772934812	0.288779911	-2.676553256	0.007438374	0.049075252	AGXT2	protein_coding
ENSG00000113494.17	1321.542007	-0.197093433	0.181333279	-1.086912639	0.27707542	0.505976431	PRLR	protein_coding
ENSG00000113504.21	11218.52094	0.080123193	0.084536933	0.947789217	0.343236771	0.572920242	SLC12A7	protein_coding
ENSG00000113520.11	3.243351983	-0.672980672	0.203823719	-3.301777999	0.000960741	0.011113937	IL4	protein_coding
ENSG00000113522.14	283.6627791	0.015040862	0.057875296	0.259883978	0.79495327	0.89728564	RAD50	protein_coding
ENSG00000113525.10	7.118091804	-0.10518977	0.146228187	-0.719353578	0.471923092	0.682949421	IL5	protein_coding
ENSG00000113532.13	849.8049731	-0.287314946	0.094365454	-3.044704739	0.002329088	0.021308983	ST8SIA4	protein_coding
ENSG00000113552.16	3061.145638	-0.212355175	0.061196016	-3.470081675	0.0005203	0.007008692	GNPDA1	protein_coding
ENSG00000113555.6	906.975119	0.095261874	0.086376804	1.102864069	0.2700862	0.498202943	PCDH12	protein_coding
ENSG00000113558.19	7559.108424	-0.0751461	0.048496539	-1.54951472	0.121258036	0.309498121	SKP1	protein_coding
ENSG00000113569.16	4050.276353	0.106674864	0.072001169	1.481571272	0.138454408	0.335115959	NUP155	protein_coding
ENSG00000113575.10	6466.41013	-0.026558993	0.047404638	-0.560261479	0.575301098	0.760223669	PPP2CA	protein_coding

ENSG00000113578.18	125.7467147	0.256157599	0.104323322	2.455420264	0.014071997	0.07669027	FGF1	protein_coding
ENSG00000113580.15	2906.412146	-0.017473338	0.091225411	-0.191540251	0.848102348	0.925563174	NR3C1	protein_coding
ENSG00000113583.8	4469.070507	0.123994299	0.064010289	1.937099511	0.05273318	0.183911927	C5orf15	protein_coding
ENSG00000113593.12	1105.758778	-0.071381667	0.046810617	-1.524903342	0.127283208	0.318588189	PPWD1	protein_coding
ENSG00000113594.10	1400.809065	-0.083781473	0.142965481	-0.586025884	0.557858121	0.748144357	LIFR	protein_coding
ENSG00000113595.15	473.7096261	0.056138509	0.055877256	1.004675473	0.315053142	0.544251809	TRIM23	protein_coding
ENSG00000113597.18	854.6204974	-0.008150465	0.048439947	-0.168259173	0.866379391	0.935187824	TRAPPC13	protein_coding
ENSG00000113600.11	8.670247479	-0.159972038	0.213980492	-0.74760104	0.454700837	0.668821251	C9	protein_coding
ENSG00000113615.13	4229.072588	0.021980562	0.063256064	0.347485449	0.728226648	0.85839305	SEC24A	protein_coding
ENSG00000113621.15	2266.5742	-0.031100144	0.051725165	-0.601257516	0.547668481	0.741100464	TXNDC15	protein_coding
ENSG00000113638.14	1204.92693	-0.014346813	0.059373955	-0.241634788	0.809063164	0.905452101	TTC33	protein_coding
ENSG00000113643.9	4102.967072	-0.012800663	0.048780086	-0.262415747	0.793000933	0.896213178	RARS1	protein_coding
ENSG00000113645.15	3924.695958	0.146870846	0.081968398	1.791798421	0.073165262	0.226202695	WWC1	protein_coding
ENSG00000113648.16	11571.16519	-0.062461929	0.049935139	-1.250861233	0.21098511	0.431390226	MACROH2A1	protein_coding
ENSG00000113649.11	4004.610591	-0.057121331	0.043921439	-1.300534148	0.19341796	0.40997006	TCERG1	protein_coding
ENSG00000113657.13	11977.56494	-0.01809349	0.154848012	-0.116846773	0.906981478	0.955912919	DPYSL3	protein_coding
ENSG00000113658.18	4004.833651	0.080124076	0.050215222	1.595613289	0.110575159	0.292244887	SMAD5	protein_coding
ENSG00000113712.19	5242.964351	0.068139281	0.053818034	1.266104989	0.205475497	0.425085725	CSNK1A1	protein_coding
ENSG00000113716.13	3466.942298	0.098654415	0.045138459	2.185595561	0.028845208	0.124306528	HMGXB3	protein_coding
ENSG00000113719.16	13194.66659	0.202530346	0.062236789	3.254190176	0.001137161	0.012670436	ERGIC1	protein_coding
ENSG00000113721.14	12139.75048	0.386981325	0.100880276	3.836045466	0.000125031	0.002345453	PDGFRB	protein_coding
ENSG00000113722.17	3184.925597	-0.048818151	0.257916228	-0.1892791	0.849874075	0.926337233	CDX1	protein_coding
ENSG00000113732.9	8854.4565	-0.016740905	0.055044905	-0.304131786	0.761027491	0.877637679	ATP6V0E1	protein_coding
ENSG00000113734.18	451.2716592	-0.06166852	0.053435911	-1.154065111	0.248473476	0.475108761	BNIP1	protein_coding
ENSG00000113739.10	653.8546149	0.456399941	0.125687977	3.631213992	0.000282091	0.004440999	STC2	protein_coding
ENSG00000113742.14	2797.575728	0.06291876	0.066952834	0.939747524	0.347347082	0.57656317	CPEB4	protein_coding
ENSG00000113749.7	393.4131348	0.047487517	0.120904635	0.392768375	0.694490564	0.838820791	HRH2	protein_coding
ENSG00000113758.13	3857.995231	0.58885087	0.09419288	6.251543319	4.06416E-10	8.59728E-08	DBN1	protein_coding
ENSG00000113761.12	757.2304695	-0.055031819	0.042642548	-1.290537791	0.196863999	0.414071942	ZNF346	protein_coding
ENSG00000113763.12	331.6062716	0.956776	0.218613341	4.376567298	1.20563E-05	0.000376707	UNC5A	protein_coding
ENSG00000113790.11	1089.137357	-0.061935544	0.077677493	-0.797342216	0.425252309	0.644138477	EHHADH	protein_coding
ENSG00000113805.8	45.83566408	0.543459732	0.204095472	2.662772117	0.007749988	0.050603831	CNTN3	protein_coding
ENSG00000113810.16	5228.397547	-0.057791894	0.075677039	-0.763664844	0.445066996	0.660987134	SMC4	protein_coding
ENSG00000113811.11	2469.708799	-0.01370631	0.053618372	-0.255627127	0.79823877	0.899075217	SELENOK	protein_coding
ENSG00000113812.14	1119.510351	0.102716328	0.04448845	2.308831361	0.020952941	0.10031491	ACTR8	protein_coding
ENSG00000113838.13	846.738016	0.073436225	0.05053061	1.453301756	0.14613999	0.346614181	TBCCD1	protein_coding
ENSG00000113845.10	3775.513899	-0.106177084	0.048263326	-2.199953748	0.027810177	0.121103635	TIMMDC1	protein_coding
ENSG00000113851.14	1454.223736	-0.057506482	0.045719866	-1.257800758	0.208463821	0.428460897	CRBN	protein_coding
ENSG00000113889.14	19.90355356	-0.074763869	0.213373239	-0.350390093	0.726045961	0.857103819	KNG1	protein_coding
ENSG00000113905.5	5.426587635	0.137753956	0.222317188	0.619628008	0.535502723	0.732147425	HRG	protein_coding
ENSG00000113916.18	2785.081342	0.099493641	0.082026092	1.212951131	0.225148505	0.448151537	BCL6	protein_coding
ENSG00000113924.12	1228.202356	-0.105090716	0.163415146	-0.643090429	0.520165425	0.720367105	HGD	protein_coding

ENSG00000113946.4	68.79934065	-0.498347982	0.154533562	-3.22485275	0.001260375	0.013684432	CLDN16	protein_coding
ENSG00000113966.10	321.258141	0.086782023	0.064237546	1.35095484	0.176709899	0.388874219	ARL6	protein_coding
ENSG00000113971.20	353.705342	-0.020027351	0.081030729	-0.247157479	0.804786351	0.902962868	NPHP3	protein_coding
ENSG00000114013.16	777.7921548	-0.125454683	0.113150804	-1.108738769	0.267542892	0.495471796	CD86	protein_coding
ENSG00000114019.15	3301.449054	0.234120458	0.078564339	2.979983791	0.002882636	0.024889369	AMOTL2	protein_coding
ENSG00000114021.12	2275.887577	-0.134251959	0.046595416	-2.881226754	0.003961305	0.031566402	NIT2	protein_coding
ENSG00000114023.15	3115.156907	-0.255767484	0.06890831	-3.71170742	0.000205866	0.003471675	FAM162A	protein_coding
ENSG00000114026.21	1025.685545	-0.163865769	0.051712298	-3.16879687	0.001530713	0.015739475	OGG1	protein_coding
ENSG00000114030.13	2668.761632	0.078341826	0.039014633	2.008011343	0.044642086	0.165407978	KPNA1	protein_coding
ENSG00000114054.14	2472.228396	-0.038748315	0.072573189	-0.533920526	0.59339652	0.773397244	PCCB	protein_coding
ENSG00000114062.22	6036.875794	-0.053908809	0.050009342	-1.077974772	0.281045015	0.50997704	UBE3A	protein_coding
ENSG00000114098.18	2274.348123	-0.023351363	0.046364321	-0.503649404	0.614507769	0.788527828	ARMC8	protein_coding
ENSG00000114107.9	1052.609418	0.074790383	0.067161127	1.113596309	0.265452411	0.493351802	CEP70	protein_coding
ENSG00000114113.6	459.6480511	-1.061040308	0.28575192	-3.713151985	0.000204694	0.003453328	RBP2	protein_coding
ENSG00000114115.10	929.1255784	0.210942547	0.16204627	1.301742687	0.193004362	0.409404599	RBP1	protein_coding
ENSG00000114120.14	4193.389214	0.020868833	0.058001122	0.359800514	0.718996318	0.853050793	SLC25A36	protein_coding
ENSG00000114124.2	6.791724703	-0.1732288	0.190399761	-0.909816268	0.362919414	0.590237734	GRK7	protein_coding
ENSG00000114125.14	3037.528034	-0.009162028	0.058226887	-0.157350465	0.874968651	0.939611841	RNF7	protein_coding
ENSG00000114126.18	4047.818776	-0.127914289	0.074221313	-1.723417233	0.084813101	0.249016338	TFDP2	protein_coding
ENSG00000114127.11	3731.489035	-0.028578018	0.064523175	-0.442910912	0.657830174	0.816307358	XRN1	protein_coding
ENSG00000114166.8	1588.206144	-0.155185742	0.082677228	-1.877007086	0.060517123	0.200849555	KAT2B	protein_coding
ENSG00000114200.10	178.613673	-0.032933871	0.216523775	-0.152102792	0.879105862	0.941618232	BCHE	protein_coding
ENSG00000114204.15	4.851435622	-0.513444214	0.249828953	-2.055182992	0.039861333	0.153669602	SERPINI2	protein_coding
ENSG00000114209.15	2868.96269	0.037300938	0.052238948	0.714044585	0.475199611	0.685446393	PDCD10	protein_coding
ENSG00000114248.10	495.0739875	0.290409136	0.179697445	1.616100526	0.10607258	0.28513962	LRRC31	protein_coding
ENSG00000114251.15	2422.457192	0.628801549	0.114737545	5.480346877	4.24493E-08	4.02133E-06	WNT5A	protein_coding
ENSG00000114268.12	596.1971586	0.256450639	0.12068843	2.124898294	0.0335951	0.137076188	PFKFB4	protein_coding
ENSG00000114270.17	2896.662982	1.087058074	0.14955348	7.268691289	3.62987E-13	2.39665E-10	COL7A1	protein_coding
ENSG00000114279.13	127.196599	0.144502807	0.144936855	0.997005262	0.318761956	0.548274181	FGF12	protein_coding
ENSG00000114302.16	6111.089895	-0.049293544	0.048652554	-1.013174835	0.310976658	0.540471882	PRKAR2A	protein_coding
ENSG00000114315.4	6775.082825	-0.012257702	0.083331979	-0.14709481	0.883057187	0.943927791	HES1	protein_coding
ENSG00000114316.13	3084.597361	0.050977415	0.0363114	1.403895607	0.160349938	0.366359968	USP4	protein_coding
ENSG00000114331.15	4816.32087	-0.019739274	0.048905507	-0.40362068	0.686491676	0.83454912	ACAP2	protein_coding
ENSG00000114346.14	6866.212485	0.156252149	0.092081748	1.696885132	0.089718379	0.257756976	ECT2	protein_coding
ENSG00000114349.10	5.446223302	0.197975308	0.237964296	0.831953834	0.405435004	0.6269345	GNAT1	protein_coding
ENSG00000114353.17	20608.10624	0.12277556	0.058558134	2.096643994	0.036025102	0.143548987	GNAI2	protein_coding
ENSG00000114354.15	10852.35584	0.034213105	0.049033807	0.697745233	0.485336535	0.693153847	TFG	protein_coding
ENSG00000114374.13	737.5677825	-0.116461433	0.258082296	-0.451256962	0.65180436	0.812560808	USP9Y	protein_coding
ENSG00000114378.17	973.4184004	0.342889973	0.160742502	2.133163096	0.032911349	0.135338102	HYAL1	protein_coding
ENSG00000114383.10	1511.32164	-0.095572752	0.04985317	-1.917084738	0.055227167	0.189663036	TUSC2	protein_coding
ENSG00000114388.14	1174.767833	-0.008295976	0.048629677	-0.170594916	0.864542296	0.934077793	NPRL2	protein_coding
ENSG00000114391.13	26651.49577	-0.169605412	0.063639997	-2.665075742	0.007697098	0.050362832	RPL24	protein_coding

ENSG00000114395.11	795.5390373	0.098212471	0.07024731	1.398095829	0.162084292	0.368948349	CYB561D2	protein_coding
ENSG00000114405.11	340.6237036	-0.28826127	0.147069302	-1.96003697	0.049991469	0.178052984	C3orf14	protein_coding
ENSG00000114416.18	5172.455723	-0.006317737	0.05359484	-0.117879567	0.906163083	0.955388696	FXR1	protein_coding
ENSG00000114423.23	2733.001612	0.091506472	0.054757994	1.671107081	0.094700529	0.266183026	CBLB	protein_coding
ENSG00000114439.19	4412.786404	0.224065583	0.057718326	3.882052706	0.000103578	0.00203684	BBX	protein_coding
ENSG00000114446.5	1959.497434	-0.108366203	0.061763552	-1.754533188	0.079339188	0.238933228	IFT57	protein_coding
ENSG00000114450.11	2072.201467	0.087628809	0.100103179	0.875384871	0.381364529	0.606699842	GNB4	protein_coding
ENSG00000114455.13	1483.385387	0.263079553	0.196179525	1.341014323	0.179915802	0.392869461	HHLA2	protein_coding
ENSG00000114473.14	466.8615139	-0.063225424	0.068254137	-0.926323681	0.354277793	0.582513805	IQCG	protein_coding
ENSG00000114480.13	1273.189662	0.223890992	0.06468925	3.461023168	0.000538126	0.007186619	GBE1	protein_coding
ENSG00000114487.10	5.13505098	-0.434275931	0.295700188	-1.468635966	0.141931554	0.340291799	MORC1	protein_coding
ENSG00000114491.14	1980.03399	-0.008165647	0.053538093	-0.152520319	0.878776565	0.941471389	UMPS	protein_coding
ENSG00000114503.11	3607.836277	-0.053898741	0.05163004	-1.043941487	0.296512462	0.526330237	NCBP2	protein_coding
ENSG00000114520.11	2443.543854	-0.069215861	0.04749006	-1.457480998	0.144983653	0.345054759	SNX4	protein_coding
ENSG00000114529.13	839.1061227	0.190881765	0.096924833	1.969379352	0.048909545	0.175447089	C3orf52	protein_coding
ENSG00000114541.15	2005.401552	0.144993737	0.080625836	1.798353284	0.072121041	0.223976842	FRMD4B	protein_coding
ENSG00000114544.17	2390.186518	0.164303066	0.049013057	3.352230565	0.000801632	0.009698148	SLC41A3	protein_coding
ENSG00000114547.10	12.60013911	0.863883581	0.195523429	4.418312358	9.94746E-06	0.000323976	ROPN1B	protein_coding
ENSG00000114554.11	7290.724639	0.313228064	0.072294949	4.332641057	1.47331E-05	0.000440479	PLXNA1	protein_coding
ENSG00000114573.10	4508.650011	0.074697949	0.05153146	1.449560095	0.147181231	0.348268706	ATP6V1A	protein_coding
ENSG00000114626.18	1944.109988	0.325878213	0.072995259	4.464375078	8.03028E-06	0.0002719	ABTB1	protein_coding
ENSG00000114631.11	3149.948264	0.259463794	0.142114158	1.825742052	0.067889126	0.215815905	PODXL2	protein_coding
ENSG00000114638.8	490.6597147	0.74492653	0.294790864	2.526966136	0.01150526	0.066617685	UPK1B	protein_coding
ENSG00000114646.10	79.03356719	0.538984942	0.142749478	3.775740197	0.000159533	0.002858341	CSPG5	protein_coding
ENSG00000114648.12	1338.049129	0.128596825	0.045987613	2.796336173	0.005168561	0.038071395	KLHL18	protein_coding
ENSG00000114650.20	6317.53162	0.083347091	0.052428938	1.589715408	0.111898969	0.294475594	SCAP	protein_coding
ENSG00000114654.7	302.5504109	0.282039499	0.105369706	2.676665899	0.007435874	0.049067482	EFCC1	protein_coding
ENSG00000114656.12	273.5546047	0.321423627	0.125293041	2.565374942	0.010306437	0.061709754	CFAP92	protein_coding
ENSG00000114670.14	388.6924202	0.025934659	0.08288069	0.31291558	0.7543448	0.87381598	NEK11	protein_coding
ENSG00000114686.9	6247.137196	-0.04055205	0.054554795	-0.743326962	0.457283752	0.670998953	MRPL3	protein_coding
ENSG00000114698.15	1342.040967	0.379994234	0.109137573	3.481791123	0.000498072	0.006776299	PLSCR4	protein_coding
ENSG00000114735.10	888.4693384	-0.012212114	0.050454361	-0.242042773	0.80874702	0.905262359	HEMK1	protein_coding
ENSG00000114737.16	995.0762419	-0.195522529	0.08061339	-2.4254349	0.015290058	0.081121076	CISH	protein_coding
ENSG00000114738.11	2936.610681	-0.008707419	0.055466156	-0.156986155	0.875255759	0.939688591	MAPKAPK3	protein_coding
ENSG00000114739.14	954.5658835	0.106949061	0.091528457	1.16847879	0.24261369	0.468571412	ACVR2B	protein_coding
ENSG00000114742.14	2302.610823	0.097773203	0.048512015	2.015443023	0.04385825	0.163477117	WDR48	protein_coding
ENSG00000114744.9	1972.267134	0.060249341	0.043650778	1.380258118	0.167507185	0.376488476	COMMD2	protein_coding
ENSG00000114745.13	2727.41938	0.016243725	0.040191495	0.404158267	0.68609634	0.834347832	GORASP1	protein_coding
ENSG00000114757.19	26.35610235	0.096656527	0.143902357	0.671681334	0.501786587	0.706985486	PEX5L	protein_coding
ENSG00000114767.7	1332.791603	-0.000586705	0.071853474	-0.008165291	0.993485113	0.997034047	RRP9	protein_coding
ENSG00000114770.17	2420.069621	-0.11572705	0.082862259	-1.396619538	0.162528012	0.369533714	ABCC5	protein_coding
ENSG00000114771.14	713.8933837	-0.994647179	0.21958496	-4.529668966	5.90762E-06	0.00021453	AADAC	protein_coding

ENSG00000114779.19	4455.103832	-0.055760489	0.069348873	-0.804057606	0.421363706	0.640921716	ABHD14B	protein_coding
ENSG00000114784.4	2074.977574	-0.067069954	0.047930807	-1.399307828	0.161720693	0.368370845	EIF1B	protein_coding
ENSG00000114786.16	3.278257332	0.243827085	0.150072796	1.624725406	0.104221081	0.282335078	ABHD14A-ACY1	protein_coding
ENSG00000114790.13	751.5852896	-0.379551044	0.163008955	-2.328406101	0.01989055	0.096867577	ARHGEF26	protein_coding
ENSG00000114796.16	4257.556395	0.134428905	0.064355623	2.088844762	0.0367217	0.14526339	KLHL24	protein_coding
ENSG00000114805.18	921.7097752	-0.150826943	0.102659753	-1.469192531	0.141780576	0.34010636	PLCH1	protein_coding
ENSG00000114812.13	948.4535102	-0.264381171	0.140822252	-1.877410478	0.060461857	0.200773865	VIPR1	protein_coding
ENSG00000114841.17	966.9062702	-0.087691307	0.095186541	-0.921257421	0.356916046	0.584701784	DNAH1	protein_coding
ENSG00000114850.7	11347.29685	0.033161975	0.055441729	0.598141077	0.549745804	0.742530853	SSR3	protein_coding
ENSG00000114853.14	1183.269926	0.221930989	0.093412776	2.375809795	0.017510486	0.088830555	ZBTB47	protein_coding
ENSG00000114854.8	225.9463875	-0.026311862	0.187742452	-0.140148709	0.888542496	0.946808578	TNNC1	protein_coding
ENSG00000114857.18	3837.050016	-0.042873385	0.069412117	-0.617664272	0.536796668	0.733080572	NKTR	protein_coding
ENSG00000114859.16	904.7529549	0.022891275	0.09403719	0.243427895	0.807673936	0.904534978	CLCN2	protein_coding
ENSG00000114861.23	3987.49185	0.024721582	0.067446263	0.36653746	0.713964066	0.850557998	FOXP1	protein_coding
ENSG00000114867.22	27964.69507	0.154417738	0.056838263	2.716791997	0.006591802	0.04514395	EIF4G1	protein_coding
ENSG00000114902.14	6256.993946	-0.050067757	0.060299893	-0.830312528	0.406362107	0.627705363	SPCS1	protein_coding
ENSG00000114904.13	1495.875637	0.069075399	0.057121228	1.20927721	0.226556363	0.449840372	NEK4	protein_coding
ENSG00000114923.17	362.5397435	0.366830643	0.169738011	2.161157899	0.030683144	0.129511757	SLC4A3	protein_coding
ENSG00000114933.16	2517.758212	0.03914045	0.050981197	0.767742857	0.442639969	0.658999724	INO80D	protein_coding
ENSG00000114942.14	17118.22056	-0.15082636	0.067038908	-2.24983319	0.024459536	0.11135324	EEF1B2	protein_coding
ENSG00000114948.13	306.5224202	0.217082665	0.149718889	1.44993505	0.147076632	0.348134623	ADAM23	protein_coding
ENSG00000114956.20	1637.21782	-0.078562169	0.058874993	-1.334389443	0.182076258	0.395552799	DGUOK	protein_coding
ENSG00000114978.18	12826.66296	-0.049489194	0.036278816	-1.364134752	0.172525159	0.383181732	MOB1A	protein_coding
ENSG00000114982.19	3714.01949	0.065827324	0.038406356	1.713969556	0.086534317	0.252055287	KANSL3	protein_coding
ENSG00000114988.12	1238.919393	-0.003276989	0.050767231	-0.064549299	0.948532854	0.975852427	LMAN2L	protein_coding
ENSG00000114993.17	3787.728946	0.023911833	0.078279807	0.305466177	0.760011127	0.877115519	RTKN	protein_coding
ENSG00000114999.8	2389.263132	-0.007772171	0.056217839	-0.138250974	0.890042071	0.947602329	TTL	protein_coding
ENSG00000115008.6	158.8207998	0.191618314	0.193695375	0.989276662	0.322527799	0.552359753	IL1A	protein_coding
ENSG00000115009.13	4330.133601	-0.762545375	0.201858636	-3.777620759	0.000158334	0.002845241	CCL20	protein_coding
ENSG00000115020.17	2408.667503	-0.008807453	0.05586526	-0.157655284	0.874728438	0.939469056	PIKFYVE	protein_coding
ENSG00000115041.13	262.6229157	0.151042013	0.153097152	0.986576237	0.323850425	0.553602305	KCNIP3	protein_coding
ENSG00000115042.10	720.7447703	-0.185920579	0.052198841	-3.561776007	0.000368355	0.005428403	FAHD2A	protein_coding
ENSG00000115053.17	28744.23872	-0.008650807	0.049984873	-0.173068499	0.862597585	0.932971332	NCL	protein_coding
ENSG00000115073.8	3491.372763	-0.037936264	0.047026229	-0.806704363	0.41983683	0.639760448	ACTR1B	protein_coding
ENSG00000115084.15	5807.143844	0.024073017	0.051776492	0.46494106	0.641973666	0.806344315	SLC35F5	protein_coding
ENSG00000115085.14	526.1329643	-0.698869766	0.132269918	-5.283663714	1.26626E-07	9.76631E-06	ZAP70	protein_coding
ENSG00000115091.12	15815.15373	-0.01718463	0.042607717	-0.403322006	0.686711355	0.834629908	ACTR3	protein_coding
ENSG00000115107.20	3012.09486	0.170069574	0.086252426	1.9717657	0.04863636	0.174841769	STEAP3	protein_coding
ENSG00000115109.14	1893.854349	0.016042726	0.057918921	0.276985924	0.781790915	0.889599506	EPB41L5	protein_coding
ENSG00000115112.8	2130.796897	0.349177338	0.15846995	2.203429335	0.027564502	0.120508605	TFCP2L1	protein_coding
ENSG00000115128.7	4093.732762	-0.105775174	0.055194252	-1.916416485	0.055312104	0.189805147	SF3B6	protein_coding
ENSG00000115129.14	1022.110757	-0.009122058	0.081330313	-0.112160615	0.910696057	0.957795512	TP53I3	protein_coding

ENSG00000115137.12	380.3811273	-0.166508074	0.052287705	-3.184459386	0.001450247	0.015177088	DNAJC27	protein_coding
ENSG00000115138.11	57.53335498	0.088440392	0.148593964	0.595181591	0.551722094	0.743749188	POMC	protein_coding
ENSG00000115145.10	2865.419223	-0.037906832	0.039252445	-0.965718991	0.334184814	0.563657324	STAM2	protein_coding
ENSG00000115155.19	68.39143758	0.079039767	0.169322977	0.466798828	0.640643815	0.805462652	OTOF	protein_coding
ENSG00000115159.16	4147.130333	0.126723059	0.074006326	1.712327394	0.086836351	0.252574267	GPD2	protein_coding
ENSG00000115163.15	621.0978716	-0.049521347	0.097932192	-0.505669749	0.613088508	0.787564718	CENPA	protein_coding
ENSG00000115165.10	1093.806296	-0.588499233	0.118337114	-4.973074092	6.58995E-07	3.72718E-05	CYTIP	protein_coding
ENSG00000115170.15	2559.256233	0.039942267	0.051229769	0.779669081	0.435585683	0.653206142	ACVR1	protein_coding
ENSG00000115183.15	3534.046863	0.026995311	0.059525615	0.453507464	0.65018337	0.811532687	TANC1	protein_coding
ENSG00000115194.11	39.13619185	-0.011204433	0.20329688	-0.05511365	0.956047921	0.979560797	SLC30A3	protein_coding
ENSG00000115204.15	3090.495761	-0.103917068	0.057088651	-1.820275416	0.068717073	0.217338068	MPV17	protein_coding
ENSG00000115207.13	3495.517152	-0.025728279	0.039287388	-0.654873744	0.512549046	0.715120219	GTF3C2	protein_coding
ENSG00000115211.16	1631.526067	-0.09927128	0.040770525	-2.43487865	0.014896785	0.07972945	EIF2B4	protein_coding
ENSG00000115216.14	6058.706178	0.02541023	0.047268563	0.537571439	0.590872952	0.771720984	NRBP1	protein_coding
ENSG00000115221.12	2721.600277	0.579802675	0.159352114	3.638500042	0.000274231	0.004339196	ITGB6	protein_coding
ENSG00000115226.10	349.644662	0.450945213	0.116441275	3.872726511	0.000107625	0.002094666	FNDC4	protein_coding
ENSG00000115232.14	1532.580566	-0.25121449	0.105147552	-2.389161574	0.016886874	0.086870417	ITGA4	protein_coding
ENSG00000115233.12	3503.114678	-0.039936187	0.051701536	-0.772437163	0.43985555	0.656424155	PSMD14	protein_coding
ENSG00000115234.11	5201.573016	0.000503515	0.041915785	0.012012551	0.990415602	0.995670766	SNX17	protein_coding
ENSG00000115239.24	838.3676815	0.010903046	0.039125021	0.278671953	0.780496588	0.88874039	ASB3	protein_coding
ENSG00000115241.11	7784.69226	-0.026829082	0.051955508	-0.516385714	0.605585042	0.782539494	PPM1G	protein_coding
ENSG00000115252.18	496.1352654	0.061810151	0.125003873	0.494465885	0.620977194	0.792604879	PDE1A	protein_coding
ENSG00000115255.12	2539.6226	-0.485571286	0.155935875	-3.113916458	0.001846218	0.018054897	REEP6	protein_coding
ENSG00000115257.15	207.5716835	0.079701223	0.125171888	0.636734206	0.524297989	0.723497687	PCSK4	protein_coding
ENSG00000115263.15	19.68585539	0.091953877	0.388026398	0.236978404	0.812673544	0.907481163	GCG	protein_coding
ENSG00000115266.12	350.8177855	0.061584286	0.09619189	0.640223264	0.52202746	0.721712901	APC2	protein_coding
ENSG00000115267.8	2281.984726	-0.048187247	0.084045313	-0.573348416	0.56640881	0.754512787	IFIH1	protein_coding
ENSG00000115268.10	39242.5925	-0.204186059	0.082308436	-2.480742783	0.013110894	0.073038837	RPS15	protein_coding
ENSG00000115271.11	1690.044254	-0.20464488	0.059787938	-3.422845595	0.000619693	0.007985859	GCA	protein_coding
ENSG00000115274.15	11.59143772	-0.103256553	0.08174876	-1.263096261	0.206554588	0.426326399	INO80B	protein_coding
ENSG00000115275.14	3892.878279	0.072300943	0.054206215	1.3338128	0.182265215	0.395782902	MOGS	protein_coding
ENSG00000115282.21	1706.665215	-0.117806683	0.04027822	-2.924823444	0.003446517	0.028498837	TTC31	protein_coding
ENSG00000115286.20	629.8360869	-0.16125205	0.08377744	-1.924766989	0.054258516	0.187534899	NDUFS7	protein_coding
ENSG00000115289.14	881.6772556	-0.037171275	0.054405594	-0.683225242	0.494464525	0.70117023	PCGF1	protein_coding
ENSG00000115290.10	293.6206862	-0.282941527	0.157021211	-1.801931889	0.071556122	0.222903792	GRB14	protein_coding
ENSG00000115295.20	928.7355786	-0.000463553	0.120224563	-0.003855727	0.996923583	0.998504884	CLIP4	protein_coding
ENSG00000115297.11	48.63139393	0.304713128	0.154329583	1.9744311	0.048332745	0.174131747	TLX2	protein_coding
ENSG00000115306.16	45595.36397	0.261043431	0.072219458	3.614585859	0.000300828	0.004651947	SPTBN1	protein_coding
ENSG00000115307.17	8311.6208	-0.027077471	0.055446255	-0.488355272	0.625298218	0.795556931	AUP1	protein_coding
ENSG00000115310.18	14211.11923	0.037418531	0.060322804	0.620304889	0.535057077	0.731912241	RTN4	protein_coding
ENSG00000115317.12	851.9051731	0.062629132	0.063962709	0.979150701	0.327505523	0.557038255	HTRA2	protein_coding
ENSG00000115318.12	315.931725	0.300900076	0.077694045	3.872884675	0.000107555	0.002094241	LOXL3	protein_coding

ENSG00000115325.13	692.7622908	0.019707532	0.065401431	0.301331807	0.763161489	0.879069956	DOK1	protein_coding
ENSG00000115339.14	6530.222041	0.294657432	0.100103205	2.943536448	0.003244856	0.027250163	GALNT3	protein_coding
ENSG00000115350.12	1027.078353	-0.104569465	0.075863733	-1.378385431	0.168084319	0.377249228	POLE4	protein_coding
ENSG00000115353.11	75.31763602	0.182185799	0.171641722	1.061430737	0.288494194	0.51832542	TACR1	protein_coding
ENSG00000115355.17	2508.443226	-0.032210071	0.092236316	-0.349212463	0.726929811	0.85744425	CCDC88A	protein_coding
ENSG00000115361.8	31.92974489	0.419657429	0.188213752	2.22968526	0.025768345	0.115276376	ACADL	protein_coding
ENSG00000115363.14	259.4150146	0.230280129	0.151208795	1.522928136	0.127776686	0.319255205	EVA1A	protein_coding
ENSG00000115364.14	2925.123068	-0.048075113	0.045011161	-1.06807094	0.285488513	0.514782168	MRPL19	protein_coding
ENSG00000115365.12	4418.8988	-0.184847097	0.082663572	-2.236137312	0.025342778	0.114067108	LANCL1	protein_coding
ENSG00000115368.10	2907.941127	-0.028428725	0.049385801	-0.575645715	0.564854674	0.753433491	WDR75	protein_coding
ENSG00000115380.20	4942.407159	0.324257221	0.15272163	2.123191202	0.033737833	0.137516934	EFEMP1	protein_coding
ENSG00000115386.6	45995.0399	-0.190612431	0.294901565	-0.646359511	0.518046555	0.71905591	REG1A	protein_coding
ENSG00000115392.12	747.036194	-0.078705785	0.052285071	-1.505320441	0.132241712	0.325919737	FANCL	protein_coding
ENSG00000115414.21	104206.6585	0.567564167	0.138905895	4.085961701	4.38946E-05	0.001040128	FN1	protein_coding
ENSG00000115415.20	23226.31787	-0.112363349	0.092084686	-1.220217538	0.22238242	0.444897563	STAT1	protein_coding
ENSG00000115419.13	5835.646008	-0.169664125	0.08744365	-1.940268098	0.052347116	0.183024773	GLS	protein_coding
ENSG00000115421.13	1507.954458	0.006933411	0.040354587	0.171812226	0.863585153	0.933614957	PAPOLG	protein_coding
ENSG00000115423.19	198.9113829	-0.126437774	0.132954265	-0.950986977	0.341610987	0.571454203	DNAH6	protein_coding
ENSG00000115425.14	586.3286441	-0.069133028	0.082704524	-0.835903831	0.403209007	0.624990558	PECR	protein_coding
ENSG00000115446.12	1736.94439	-0.046850787	0.039694879	-1.180272819	0.237891725	0.46318058	UNC50	protein_coding
ENSG00000115457.10	12154.2427	-0.362984318	0.16853314	-2.153786008	0.031256957	0.131087186	IGFBP2	protein_coding
ENSG00000115459.18	862.705076	-0.036835408	0.039892614	-0.923364117	0.355817485	0.5837477	ELMOD3	protein_coding
ENSG00000115461.5	35522.02416	0.29448445	0.12703936	2.318056779	0.020446235	0.098632528	IGFBP5	protein_coding
ENSG00000115464.15	5522.594021	0.0870462	0.063974456	1.360639951	0.1736275	0.384740807	USP34	protein_coding
ENSG00000115468.12	500.1731425	0.352870547	0.123891261	2.848227902	0.004396343	0.033931887	EFHD1	protein_coding
ENSG00000115474.7	35.14116852	-0.523324574	0.149945634	-3.490095442	0.000482848	0.006625022	KCNJ13	protein_coding
ENSG00000115484.15	6553.260739	-0.001946775	0.050928477	-0.038225667	0.969507756	0.985841547	CCT4	protein_coding
ENSG00000115486.12	2493.738049	0.105628253	0.052930523	1.995601906	0.045977272	0.168534062	GGCX	protein_coding
ENSG00000115488.3	1.501776791	0.256510855	0.346052982	0.741247348	0.45854348	0.671871821	NEU2	protein_coding
ENSG00000115504.15	2676.683317	0.033100779	0.061852663	0.535155277	0.592542489	0.772789754	EHBP1	protein_coding
ENSG00000115507.10	533.3919024	-0.066184874	0.107205646	-0.617363696	0.536994862	0.733171197	OTX1	protein_coding
ENSG00000115514.12	1449.41848	-0.01849882	0.048665667	-0.380120536	0.703855942	0.84460581	TXNDC9	protein_coding
ENSG00000115520.8	1585.083699	0.07370622	0.048585176	1.51705162	0.129253652	0.321568852	COQ10B	protein_coding
ENSG00000115523.17	698.7507654	-0.250865324	0.158377107	-1.583974665	0.113199485	0.296715425	GNLY	protein_coding
ENSG00000115524.17	18097.77164	0.07495893	0.046797607	1.601768463	0.109206819	0.289876687	SF3B1	protein_coding
ENSG00000115525.18	619.7108089	-0.181564682	0.091290772	-1.988861282	0.046716516	0.170271465	ST3GAL5	protein_coding
ENSG00000115526.11	322.3691134	0.185888011	0.111534665	1.666638903	0.095586228	0.267696727	CHST10	protein_coding
ENSG00000115539.14	1146.739227	-0.081572496	0.048162911	-1.693678695	0.090326355	0.258753061	PDCL3	protein_coding
ENSG00000115540.15	1850.918838	-0.073783521	0.048143565	-1.532572861	0.125381133	0.315679762	MOB4	protein_coding
ENSG00000115541.11	3248.496811	-0.165055402	0.089127397	-1.851904209	0.064039579	0.208055823	HSPE1	protein_coding
ENSG00000115548.17	3233.39042	0.10427344	0.05464127	1.90832753	0.056348897	0.192077275	KDM3A	protein_coding
ENSG00000115556.14	58.03934823	-0.505556503	0.195037527	-2.592098609	0.009539242	0.058449321	PLCD4	protein_coding

ENSG00000115561.16	6133.081787	-0.108755188	0.049207976	-2.210113021	0.027097319	0.119250645	CHMP3	protein_coding
ENSG00000115568.16	2615.570423	0.021926167	0.042702901	0.513458492	0.607630639	0.783811507	ZNF142	protein_coding
ENSG00000115590.14	1360.3688	0.261327959	0.183994384	1.420304002	0.155519197	0.3600797	IL1R2	protein_coding
ENSG00000115592.11	3.716482273	0.12193034	0.241657626	0.504558214	0.613869165	0.788126115	PRKAG3	protein_coding
ENSG00000115593.15	259.2814179	-0.820521916	0.325911931	-2.517618525	0.011815121	0.067827781	SMYD1	protein_coding
ENSG00000115594.12	5342.846505	0.245197835	0.099695852	2.459458741	0.013914669	0.076108891	IL1R1	protein_coding
ENSG00000115596.4	44.65212385	0.214462843	0.162191955	1.322277935	0.186075617	0.400861043	WNT6	protein_coding
ENSG00000115598.10	155.9772014	0.55194812	0.150111535	3.676920107	0.000236067	0.003864421	IL1RL2	protein_coding
ENSG00000115602.17	211.0678574	-0.383504455	0.17147133	-2.236551473	0.02531567	0.11402157	IL1RL1	protein_coding
ENSG00000115604.11	399.1385657	-0.071489057	0.100321548	-0.712599223	0.476093792	0.686143291	IL18R1	protein_coding
ENSG00000115607.9	101.9271325	-0.283542295	0.1250336	-2.267728803	0.023345741	0.107861573	IL18RAP	protein_coding
ENSG00000115616.3	1585.896265	-0.167207927	0.17613622	-0.949310295	0.342462819	0.572329904	SLC9A2	protein_coding
ENSG00000115641.19	7557.044017	0.131261379	0.101465492	1.29365538	0.195784484	0.412817558	FHL2	protein_coding
ENSG00000115648.14	5810.845635	0.227628425	0.143928182	1.581541718	0.113754228	0.297435977	MLPH	protein_coding
ENSG00000115649.16	3556.776537	-0.076781212	0.054951974	-1.397242105	0.162340779	0.369262142	CNPPD1	protein_coding
ENSG00000115652.15	2368.54286	-0.13976259	0.055583377	-2.514467411	0.011921232	0.068272137	UXS1	protein_coding
ENSG00000115657.14	47.65299732	-0.11424304	0.103569471	-1.103057092	0.270002373	0.498132659	ABCB6	protein_coding
ENSG00000115661.14	1395.82618	-0.052336166	0.050474866	-1.036875776	0.299793755	0.529481071	STK16	protein_coding
ENSG00000115665.9	23.54222526	-0.489008315	0.261556864	-1.869606125	0.061538534	0.203064115	SLC5A7	protein_coding
ENSG00000115677.17	37658.41687	0.067518098	0.052964049	1.274791105	0.20238319	0.42138909	HDLBP	protein_coding
ENSG00000115685.15	3228.258834	-0.096119972	0.044968384	-2.137501127	0.032557254	0.134529439	PPP1R7	protein_coding
ENSG00000115687.14	1025.718004	-0.212925423	0.066126234	-3.219984107	0.001281977	0.013865969	PASK	protein_coding
ENSG00000115694.15	5031.158528	-0.037416733	0.04866344	-0.768887958	0.441959827	0.658347008	STK25	protein_coding
ENSG00000115705.22	87.66871262	0.359327112	0.197818732	1.816446337	0.069301933	0.218395308	TPO	protein_coding
ENSG00000115718.18	293.1613767	-0.362451925	0.179781237	-2.016072041	0.043792443	0.16331548	PROC	protein_coding
ENSG00000115738.10	4504.429924	-0.242390839	0.080740897	-3.002082564	0.002681394	0.023638908	ID2	protein_coding
ENSG00000115750.17	829.4531285	-0.028004802	0.048744101	-0.57452699	0.565611242	0.753958003	TAF1B	protein_coding
ENSG00000115756.13	3516.615995	0.036118332	0.070138206	0.514959444	0.60658136	0.78306238	HPCAL1	protein_coding
ENSG00000115758.13	9715.204897	0.057195969	0.087484316	0.653785414	0.513250065	0.715588822	ODC1	protein_coding
ENSG00000115760.14	8079.640989	0.004647349	0.053281315	0.087222862	0.930494367	0.967010903	BIRC6	protein_coding
ENSG00000115761.16	1904.528538	-0.014810056	0.043039562	-0.344103311	0.730768593	0.860016825	NOL10	protein_coding
ENSG00000115762.16	8770.05663	-0.038600661	0.05616604	-0.687259788	0.491919034	0.698935629	PLEKHB2	protein_coding
ENSG00000115806.13	9288.295649	0.050245483	0.043747648	1.148529942	0.25074986	0.477429398	GORASP2	protein_coding
ENSG00000115808.12	4105.148513	0.065802573	0.048252328	1.363718095	0.172656307	0.383361046	STRN	protein_coding
ENSG00000115816.15	2151.565127	0.039105782	0.051304865	0.762223658	0.445926528	0.661556969	CEBPZ	protein_coding
ENSG00000115825.11	2286.639257	-0.004373736	0.076235461	-0.057371409	0.954249338	0.978600343	PRKD3	protein_coding
ENSG00000115827.14	1502.973202	-0.119042917	0.043388603	-2.743644846	0.006076124	0.042692557	DCAF17	protein_coding
ENSG00000115828.17	798.0682039	0.493358242	0.157832748	3.125829395	0.001773044	0.017540052	QPCT	protein_coding
ENSG00000115839.18	3682.77411	-0.003064717	0.038106877	-0.08042426	0.935899833	0.969767071	RAB3GAP1	protein_coding
ENSG00000115840.14	1260.245816	-0.013571552	0.074206538	-0.182888902	0.854885191	0.929168335	SLC25A12	protein_coding
ENSG00000115841.21	264.2324097	-0.140452863	0.096560751	-1.454554383	0.145792668	0.346191188	RMDN2	protein_coding
ENSG00000115844.11	17.60599366	0.03405192	0.207913662	0.163779138	0.869905015	0.937083797	DLX2	protein_coding

ENSG00000115850.10	30.74096631	-0.293567393	0.233724332	-1.25604121	0.209101029	0.429128232	LCT	protein_coding
ENSG00000115866.11	6211.687187	-0.122197578	0.051861815	-2.356214812	0.018462238	0.092082974	DARS1	protein_coding
ENSG00000115875.19	6505.74102	-0.08727389	0.040094028	-2.176730395	0.029500689	0.126142227	SRSF7	protein_coding
ENSG00000115884.11	17183.81373	0.099561954	0.104152438	0.955925331	0.33910996	0.568880475	SDC1	protein_coding
ENSG00000115896.16	334.2922837	-0.025757988	0.106582016	-0.241672931	0.809033606	0.905452101	PLCL1	protein_coding
ENSG00000115902.11	3242.476184	-0.048833234	0.076345658	-0.63963341	0.522410956	0.722014026	SLC1A4	protein_coding
ENSG00000115904.14	5023.09801	-0.040731017	0.045222192	-0.900686492	0.367755033	0.594887006	SOS1	protein_coding
ENSG00000115919.15	1044.263356	0.148680776	0.141302437	1.052216644	0.292700165	0.522617188	KYNU	protein_coding
ENSG00000115934.11	0.744226172	0.657621782	0.414707386	1.585748901	0.112796281		AC087235.1	lncRNA
ENSG00000115935.18	4540.357585	-0.259849029	0.097828106	-2.656179693	0.007903149	0.051318063	WIPF1	protein_coding
ENSG00000115942.9	1895.110407	-0.118035768	0.047191056	-2.501231761	0.012376216	0.070050442	ORC2	protein_coding
ENSG00000115944.15	3877.663357	-0.124171691	0.043827165	-2.833212912	0.004608268	0.035131998	COX7A2L	protein_coding
ENSG00000115946.8	1425.157723	-0.051969936	0.048894798	-1.062892934	0.287830508	0.517527233	PNO1	protein_coding
ENSG00000115947.14	1772.666975	0.007638587	0.043475425	0.175698956	0.860530456	0.932201115	ORC4	protein_coding
ENSG00000115956.10	1850.327489	-0.441903608	0.134534562	-3.284684632	0.001020966	0.011674266	PLEK	protein_coding
ENSG00000115963.13	2209.124344	0.355909335	0.082751859	4.300922518	1.70089E-05	0.00049036	RND3	protein_coding
ENSG00000115966.17	3097.441531	0.019867408	0.041713273	0.476285055	0.633871311	0.801374495	ATF2	protein_coding
ENSG00000115970.18	2596.156774	-0.052529126	0.04312702	-1.218009655	0.223220304	0.445836978	THADA	protein_coding
ENSG00000115977.20	4251.918838	0.068999172	0.053555435	1.288369191	0.197617478	0.414955758	AAK1	protein_coding
ENSG00000115993.13	2991.710966	-0.006767289	0.05350513	-0.126479258	0.899352567	0.952071747	TRAK2	protein_coding
ENSG00000115998.8	656.6475669	-0.059722533	0.035421437	-1.686056174	0.09178498	0.26124847	C2orf42	protein_coding
ENSG00000116001.17	4058.554077	-0.114427281	0.060958495	-1.877134299	0.060499689	0.200849555	TIA1	protein_coding
ENSG00000116005.12	5971.928915	0.096175243	0.053587916	1.794718842	0.072698507	0.225350892	PCYOX1	protein_coding
ENSG00000116014.10	43.4744489	0.262396508	0.211092109	1.243042716	0.213852093	0.434779325	KISS1R	protein_coding
ENSG00000116016.14	14857.1331	0.128796168	0.068550429	1.878852851	0.060264588	0.200386643	EPAS1	protein_coding
ENSG00000116017.11	2450.657367	0.182942328	0.14561349	1.256355629	0.208987061	0.429019785	ARID3A	protein_coding
ENSG00000116030.17	4690.924762	-0.063725146	0.046329368	-1.375480593	0.168982498	0.378287801	SUMO1	protein_coding
ENSG00000116031.9	68.43380491	-0.176377958	0.178552631	-0.987820553	0.32324054	0.553015312	CD207	protein_coding
ENSG00000116032.5	43.01547487	1.000495535	0.146212899	6.842730999	7.76975E-12	3.2556E-09	GRIN3B	protein_coding
ENSG00000116035.4	43.71263219	0.510653072	0.184991279	2.760417003	0.005772762	0.04123253	VAX2	protein_coding
ENSG00000116039.13	7.35970094	0.651621095	0.183357589	3.553826692	0.000379669	0.00553497	ATP6V1B1	protein_coding
ENSG00000116044.16	9076.891668	0.003408307	0.04922064	0.06924548	0.944794222	0.974076651	NFE2L2	protein_coding
ENSG00000116062.17	2971.285896	0.007900315	0.057657193	0.137022191	0.891013255	0.948032607	MSH6	protein_coding
ENSG00000116095.11	1915.651819	0.002961426	0.042108805	0.070327961	0.943932628	0.973649067	PLEKHA3	protein_coding
ENSG00000116096.6	2535.607167	-0.170990546	0.072561415	-2.356494099	0.018448361	0.092030299	SPR	protein_coding
ENSG00000116106.12	643.2133234	0.085823049	0.138112316	0.621400409	0.534336203	0.731368913	EPHA4	protein_coding
ENSG00000116117.18	1595.664617	-0.014838613	0.078487398	-0.189057267	0.850047933	0.926434043	PARD3B	protein_coding
ENSG00000116120.11	3634.875716	-0.133150588	0.060114025	-2.214967126	0.026762321	0.118254072	FARSB	protein_coding
ENSG00000116127.19	2260.974532	0.070299098	0.059559254	1.180321991	0.237872175	0.46318058	ALMS1	protein_coding
ENSG00000116128.11	2740.079703	0.204280141	0.056227776	3.633082354	0.000280056	0.004415334	BCL9	protein_coding
ENSG00000116132.12	2214.413037	0.488799326	0.135686637	3.602413138	0.000315277	0.004824022	PRRX1	protein_coding
ENSG00000116133.13	13816.69465	0.027912188	0.106794104	0.261364507	0.793811425	0.896539764	DHCR24	protein_coding

ENSG00000116138.13	1925.192275	-0.056338785	0.047609685	-1.183347146	0.236671595	0.461657928	DNAJC16	protein_coding
ENSG00000116141.17	527.6244865	0.018644481	0.148216203	0.12579246	0.899896211	0.952138522	MARK1	protein_coding
ENSG00000116147.17	52.38009224	-0.520412365	0.182089424	-2.858004347	0.004263146	0.033156605	TNR	protein_coding
ENSG00000116151.14	373.9280627	0.113779079	0.060652227	1.87592583	0.060665465	0.201096834	MORN1	protein_coding
ENSG00000116157.6	588.1092558	0.215246065	0.113807923	1.89131002	0.058582972	0.196783332	GPX7	protein_coding
ENSG00000116161.18	4692.260278	-0.067940127	0.063523446	-1.069528358	0.284831658	0.514107987	CACYBP	protein_coding
ENSG00000116171.19	8900.975957	-0.16515414	0.05340032	-3.092755641	0.001983074	0.019000966	SCP2	protein_coding
ENSG00000116176.6	50.50185425	0.396690736	0.187718697	2.113219102	0.034582019	0.139703406	TPSG1	protein_coding
ENSG00000116183.11	65.45094258	-0.096233139	0.198729841	-0.48424101	0.628214835	0.797620366	PAPPA2	protein_coding
ENSG00000116191.17	2711.792584	-0.154073941	0.076219584	-2.021448192	0.04323339	0.162076862	RALGPS2	protein_coding
ENSG00000116194.13	571.5587867	-0.601365307	0.218769238	-2.748856797	0.005980351	0.042223873	ANGPTL1	protein_coding
ENSG00000116198.14	2413.048691	-0.012797315	0.042626278	-0.300221269	0.764008383	0.879678529	CEP104	protein_coding
ENSG00000116199.12	4296.844502	0.03064962	0.051912815	0.590405672	0.554918709	0.745911119	FAM20B	protein_coding
ENSG00000116205.14	862.8429402	-0.094883482	0.044574817	-2.128634253	0.033284532	0.136306744	TCEANC2	protein_coding
ENSG00000116209.12	16945.96523	0.0594078	0.050612273	1.173782507	0.240482152	0.465879379	TMEM59	protein_coding
ENSG00000116212.15	1493.235978	0.043320531	0.053949548	0.802982285	0.421984974	0.641262352	LRRC42	protein_coding
ENSG00000116213.16	1182.855705	0.0463779	0.048749379	0.951353664	0.341424873	0.571384143	WRAP73	protein_coding
ENSG00000116218.13	5.13603579	-0.332957427	0.2487609	-1.338463672	0.180745335	0.393836665	NPHS2	protein_coding
ENSG00000116221.15	5348.008615	-0.121168362	0.056114856	-2.15929202	0.030827519	0.12984446	MRPL37	protein_coding
ENSG00000116237.16	6478.881892	0.09035778	0.047176805	1.915300946	0.055454135	0.190112779	ICMT	protein_coding
ENSG00000116251.11	5073.493579	-0.265277316	0.060724233	-4.368557715	1.2507E-05	0.000387361	RPL22	protein_coding
ENSG00000116254.18	99.44461004	0.544337756	0.178414417	3.050974042	0.002281003	0.021026109	CHD5	protein_coding
ENSG00000116260.17	20567.53335	0.283850967	0.096239064	2.949436067	0.003183544	0.026866054	QSOX1	protein_coding
ENSG00000116266.11	2926.04558	0.013047582	0.04570858	0.285451475	0.775298276	0.886191716	STXBP3	protein_coding
ENSG00000116273.6	1173.868277	0.151762039	0.05204191	2.916150428	0.003543796	0.029055126	PHF13	protein_coding
ENSG00000116285.13	5197.247336	0.388545999	0.094432004	4.114558424	3.87921E-05	0.000940181	ERRFI1	protein_coding
ENSG00000116288.13	6981.335829	-0.109406401	0.051959322	-2.105616411	0.035237686	0.141421315	PARK7	protein_coding
ENSG00000116299.17	12074.76718	-0.104529517	0.189989635	-0.550185365	0.582192241	0.765673021	ELAPOR1	protein_coding
ENSG00000116329.11	59.88355946	0.579203664	0.188623341	3.070689242	0.002135653	0.020061511	OPRD1	protein_coding
ENSG00000116337.19	2640.385324	0.049163673	0.050333041	0.976767388	0.328684325	0.558143435	AMPD2	protein_coding
ENSG00000116350.17	5151.395355	-0.046539769	0.033804073	-1.376750363	0.168589442	0.377818934	SRSF4	protein_coding
ENSG00000116353.16	901.1649766	-0.058505733	0.051213125	-1.142397237	0.253288939	0.480129221	MECR	protein_coding
ENSG00000116396.15	1597.334841	0.297986334	0.086137302	3.459434251	0.000541311	0.007226939	KCNC4	protein_coding
ENSG00000116406.19	6991.222019	0.291223112	0.084638011	3.44080761	0.000579981	0.007615988	EDEM3	protein_coding
ENSG00000116455.14	1686.627504	-0.219194079	0.062441896	-3.510368744	0.000447486	0.006260059	WDR77	protein_coding
ENSG00000116459.11	9221.524365	-0.131851483	0.052340386	-2.519115579	0.011765003	0.067631745	ATP5PB	protein_coding
ENSG00000116473.14	4973.676713	-0.240409543	0.058341566	-4.12072488	3.77682E-05	0.000920484	RAP1A	protein_coding
ENSG00000116478.12	10068.49347	-0.116329806	0.054483419	-2.13514144	0.032749459	0.135029157	HDAC1	protein_coding
ENSG00000116489.13	9560.924625	0.060488257	0.046751287	1.293830847	0.195723855	0.412755646	CAPZA1	protein_coding
ENSG00000116497.18	1331.670757	-0.037238504	0.046030009	-0.809004923	0.418512315	0.638590677	S100PBP	protein_coding
ENSG00000116514.17	3315.424193	0.144547568	0.070930389	2.037879254	0.04156201	0.157875497	RNF19B	protein_coding
ENSG00000116521.11	5428.899711	0.002887894	0.051513803	0.056060587	0.955293541	0.979225316	SCAMP3	protein_coding

ENSG00000116525.14	724.8064113	0.249479012	0.059465858	4.195331906	2.72472E-05	0.000708022	TRIM62	protein_coding
ENSG00000116539.14	7091.906621	0.11573513	0.054003115	2.143119531	0.032103502	0.133235647	ASH1L	protein_coding
ENSG00000116544.12	102.8854294	0.226442946	0.149183258	1.517884441	0.129043533	0.321314784	DLGAP3	protein_coding
ENSG00000116560.11	15180.38937	-0.021433902	0.035821281	-0.598356642	0.549601989	0.742438822	SFPQ	protein_coding
ENSG00000116574.6	2944.057998	-0.421746402	0.099885298	-4.222307097	2.41814E-05	0.000641772	RHOU	protein_coding
ENSG00000116580.18	2783.631325	0.112019527	0.044962778	2.491383595	0.012724666	0.071493392	GON4L	protein_coding
ENSG00000116584.20	4961.940357	0.159841557	0.052367978	3.052276627	0.002271127	0.020976876	ARHGEF2	protein_coding
ENSG00000116586.12	2033.612224	-0.029542129	0.058218351	-0.507436713	0.611848432	0.786899599	LAMTOR2	protein_coding
ENSG00000116604.18	6238.656378	0.209897743	0.057514165	3.649496498	0.000262755	0.004197239	MEF2D	protein_coding
ENSG00000116641.18	2934.558581	-0.109194723	0.053357406	-2.046477348	0.040709429	0.155681859	DOCK7	protein_coding
ENSG00000116649.10	7736.648239	-0.044531802	0.068300323	-0.651999875	0.514401253	0.716305471	SRM	protein_coding
ENSG00000116652.6	20.58775753	-0.126190091	0.098913558	-1.275761315	0.202039906	0.421030665	DLEU2L	lncRNA
ENSG00000116661.11	950.6854138	-0.070568315	0.192533683	-0.366524519	0.71397372	0.850557998	FBXO2	protein_coding
ENSG00000116663.11	1229.91779	-0.04906861	0.089538175	-0.548018875	0.583678942	0.766846567	FBXO6	protein_coding
ENSG00000116667.15	3075.249696	0.018562367	0.092946712	0.19970978	0.841707564	0.922196835	C1orf21	protein_coding
ENSG00000116668.13	475.9301232	-0.036321696	0.050573581	-0.718195051	0.472637029	0.683551951	SWT1	protein_coding
ENSG00000116670.15	1657.698765	0.006258326	0.075613551	0.082767259	0.934036603	0.96886345	MAD2L2	protein_coding
ENSG00000116675.16	273.9132135	0.52420582	0.152665289	3.433693558	0.000595417	0.007752687	DNAJC6	protein_coding
ENSG00000116678.20	951.3002888	0.067940273	0.12966953	0.523949408	0.600313729	0.778679842	LEPR	protein_coding
ENSG00000116679.16	9807.088046	0.051239476	0.068439439	0.748683459	0.454048015	0.668402485	IVNS1ABP	protein_coding
ENSG00000116685.16	6925.425179	-0.043640469	0.057001089	-0.76560764	0.443909797	0.659969884	KIAA2013	protein_coding
ENSG00000116688.18	6783.466517	-0.0408511	0.0570651	-0.715868361	0.474072636	0.684677153	MFN2	protein_coding
ENSG00000116690.13	153.1078016	0.876245663	0.184730527	4.743372285	2.10189E-06	9.36864E-05	PRG4	protein_coding
ENSG00000116691.11	1398.342845	-0.07812607	0.058760568	-1.329566277	0.183661215	0.397588479	MIIP	protein_coding
ENSG00000116698.21	7983.719035	0.097670945	0.044266911	2.206409774	0.027355321	0.119937905	SMG7	protein_coding
ENSG00000116701.15	876.9914333	0.17194881	0.112713287	1.52554162	0.127124061	0.318352105	NCF2	protein_coding
ENSG00000116703.14	11.09572865	0.049746351	0.146948515	0.338529118	0.734964488	0.862464081	PDC	protein_coding
ENSG00000116704.8	2700.807603	-0.101922694	0.071788647	-1.419760628	0.155677381	0.360290417	SLC35D1	protein_coding
ENSG00000116711.10	1435.692428	0.135179642	0.140964679	0.958961086	0.337578349	0.567343582	PLA2G4A	protein_coding
ENSG00000116717.13	1754.002821	-0.153784966	0.065993911	-2.330290233	0.019790817	0.096478847	GADD45A	protein_coding
ENSG00000116721.9	0.636102579	-0.261066943	0.775619882	-0.33659135	0.736424978		PRAMEF1	protein_coding
ENSG00000116726.5	1.223679056	-0.612845955	0.515215226	-1.189495039	0.234244922	0.458873003	PRAMEF12	protein_coding
ENSG00000116729.14	4280.926422	0.057144001	0.094855355	0.602433054	0.546885913	0.740619287	WLS	protein_coding
ENSG00000116731.22	2983.116101	-0.031029581	0.051423992	-0.603406681	0.546238179	0.740210179	PRDM2	protein_coding
ENSG00000116741.8	2775.793402	0.046785127	0.121381684	0.385438112	0.699912851	0.842079025	RGS2	protein_coding
ENSG00000116745.7	12.70768351	1.31276236	0.243239848	5.396987267	6.77692E-08	5.97923E-06	RPE65	protein_coding
ENSG00000116747.13	2330.188255	0.124032844	0.04840171	2.562571497	0.010390019	0.061929404	RO60	protein_coding
ENSG00000116748.21	51.3287871	-1.012858391	0.188768672	-5.365606369	8.06777E-08	6.82659E-06	AMPD1	protein_coding
ENSG00000116750.14	2161.148789	0.06028756	0.054175596	1.11281766	0.265786749	0.493678409	UCHL5	protein_coding
ENSG00000116752.6	1854.494711	-0.029928101	0.048163845	-0.621381057	0.534348933	0.731368913	BCAS2	protein_coding
ENSG00000116754.13	8272.880375	-0.051596335	0.046930625	-1.099417173	0.271586144	0.499806131	SRSF11	protein_coding
ENSG00000116761.12	415.2190316	0.211334836	0.109321126	1.933156406	0.053216933	0.185049018	CTH	protein_coding

ENSG00000116771.6	1750.743862	-0.49735877	0.110238722	-4.511652157	6.43246E-06	0.000230327	AGMAT	protein_coding
ENSG00000116774.12	1991.838648	0.207239689	0.123701003	1.675327471	0.093869998	0.264762002	OLFML3	protein_coding
ENSG00000116783.15	2.106658061	-0.011738853	0.2504785	-0.04686571	0.962620257	0.982718436	TNNI3K	protein_coding
ENSG00000116785.14	15.3992785	0.898235576	0.220203439	4.079116925	4.52071E-05	0.001060842	CFHR3	protein_coding
ENSG00000116786.13	4575.266608	0.061879183	0.046825915	1.321473016	0.186343694	0.401200551	PLEKHM2	protein_coding
ENSG00000116791.14	2719.955828	-0.029333898	0.080193453	-0.365789183	0.714522392	0.850823873	CRYZ	protein_coding
ENSG00000116793.16	852.5513296	0.05702044	0.059521404	0.957982105	0.338071782	0.567913383	PHTF1	protein_coding
ENSG00000116809.12	1367.903054	-0.017011501	0.047769517	-0.356116253	0.721753506	0.854925454	ZBTB17	protein_coding
ENSG00000116815.16	1159.336386	0.020506084	0.060225465	0.340488596	0.73348861	0.861612345	CD58	protein_coding
ENSG00000116819.8	89.79458826	0.403161647	0.125085039	3.223100467	0.001268111	0.013749803	TFAP2E	protein_coding
ENSG00000116824.5	1172.214087	-0.562337899	0.125993817	-4.463218198	8.07378E-06	0.000272949	CD2	protein_coding
ENSG00000116830.12	1952.864302	0.005429617	0.062218368	0.08726711	0.930459197	0.967010903	TTF2	protein_coding
ENSG00000116833.14	1024.284053	-0.243802093	0.136429532	-1.787018466	0.073934504	0.227837061	NR5A2	protein_coding
ENSG00000116852.15	1573.263367	-0.118454004	0.100264501	-1.181415186	0.237437828	0.462676217	KIF21B	protein_coding
ENSG00000116857.17	3337.299115	0.056100823	0.074475779	0.753276076	0.451284032	0.665975965	TMEM9	protein_coding
ENSG00000116863.11	1365.964285	-0.020241464	0.052016052	-0.389138798	0.697173474	0.840446731	ADPRS	protein_coding
ENSG00000116871.15	7211.071066	0.102110044	0.074503295	1.370544009	0.170517147	0.380584859	MAP7D1	protein_coding
ENSG00000116874.12	1042.431472	-0.045008854	0.055860526	-0.805736303	0.420394913	0.640207902	WARS2	protein_coding
ENSG00000116882.15	5.56246228	-0.776916004	0.212565293	-3.654952285	0.00025723	0.004133224	HAO2	protein_coding
ENSG00000116885.18	266.5098247	-0.034169148	0.06525366	-0.523635736	0.600531921	0.778827401	OSCP1	protein_coding
ENSG00000116898.12	3607.626118	-0.096208021	0.061056974	-1.575708953	0.115092893	0.299414761	MRPS15	protein_coding
ENSG00000116903.7	1112.046368	0.019884791	0.040166797	0.495055425	0.620560997	0.792421807	EXOC8	protein_coding
ENSG00000116906.13	4118.267441	-0.257279699	0.048970685	-5.253749247	1.49034E-07	1.11973E-05	GNPAT	protein_coding
ENSG00000116918.15	1793.801538	0.020651749	0.048688686	0.424159109	0.67144978	0.825050725	TSNAX	protein_coding
ENSG00000116922.14	1040.071515	-0.039750765	0.049710616	-0.799643381	0.423917446	0.642965805	C1orf109	protein_coding
ENSG00000116954.8	1010.130219	-0.025577882	0.045704545	-0.559635403	0.575728152	0.760626536	RRAGC	protein_coding
ENSG00000116962.15	11431.80292	0.051558574	0.091918137	0.560918398	0.574853167	0.759947965	NID1	protein_coding
ENSG00000116977.19	3557.612332	0.076071816	0.046689043	1.629329074	0.103243374	0.280811191	LGALS8	protein_coding
ENSG00000116981.3	4.553003165	-0.121787032	0.216480532	-0.562577295	0.573722757	0.759175479	NT5C1A	protein_coding
ENSG00000116983.13	27.32780063	0.209741809	0.185541858	1.130428521	0.258295702	0.485433726	HPCAL4	protein_coding
ENSG00000116984.14	2967.728665	0.014865493	0.056495362	0.263127666	0.792452181	0.895951177	MTR	protein_coding
ENSG00000116985.12	604.212878	0.286150356	0.104883659	2.728264424	0.006366855	0.044060415	BMP8B	protein_coding
ENSG00000116990.11	517.193936	0.083694926	0.108108127	0.774177929	0.438825578	0.65549317	MYCL	protein_coding
ENSG00000116991.11	2623.184755	0.145483114	0.099180295	1.466855017	0.1424155	0.341084926	SIPA1L2	protein_coding
ENSG00000116996.9	1.612390453	0.088826722	0.379554243	0.234029058	0.81496242	0.908674333	ZP4	protein_coding
ENSG00000117000.9	1691.519868	0.118859462	0.051708098	2.298662433	0.021524115	0.102151642	RLF	protein_coding
ENSG00000117009.12	98.87818559	-0.404678711	0.111009733	-3.64543449	0.00026694	0.004248525	KMO	protein_coding
ENSG00000117010.17	193.4473821	-0.177615946	0.054921669	-3.233986694	0.001220751	0.01336936	ZNF684	protein_coding
ENSG00000117013.17	318.6923726	0.111720087	0.143596078	0.778016287	0.436559412	0.654006613	KCNQ4	protein_coding
ENSG00000117016.10	339.8019953	-0.253663427	0.13872663	-1.828512856	0.067472618	0.21489945	RIMS3	protein_coding
ENSG00000117020.18	1930.478494	0.058979464	0.11734187	0.502629313	0.615224916	0.788867329	AKT3	protein_coding
ENSG00000117036.12	2200.693153	0.188964443	0.048443029	3.900756129	9.58927E-05	0.001913332	ETV3	protein_coding

ENSG00000117054.14	2502.932298	-0.071111264	0.068320507	-1.040848017	0.297946091	0.52785286	ACADM	protein_coding
ENSG00000117069.15	470.3041857	0.232340571	0.13572957	1.711790367	0.086935308	0.252693431	ST6GALNAC5	protein_coding
ENSG00000117090.15	236.9182001	-0.615901325	0.118379552	-5.20276784	1.96342E-07	1.40262E-05	SLAMF1	protein_coding
ENSG00000117091.10	534.2316522	-0.580706152	0.137415116	-4.225926301	2.3796E-05	0.000638521	CD48	protein_coding
ENSG00000117114.20	2474.491752	0.385230268	0.103167673	3.734021086	0.000188447	0.003257415	ADGRL2	protein_coding
ENSG00000117115.13	1577.313989	0.325835708	0.166196539	1.960544485	0.049932184	0.17789999	PADI2	protein_coding
ENSG00000117118.10	4980.479053	-0.154220447	0.060319984	-2.556705686	0.010566858	0.062694235	SDHB	protein_coding
ENSG00000117122.14	1086.571291	0.701443055	0.133951225	5.236555742	1.63601E-07	1.20834E-05	MFAP2	protein_coding
ENSG00000117133.11	2010.178433	-0.004572991	0.046705621	-0.097910933	0.922003018	0.963172363	RPF1	protein_coding
ENSG00000117139.18	5100.701313	0.32025067	0.060507299	5.292760945	1.20483E-07	9.34218E-06	KDM5B	protein_coding
ENSG00000117143.13	3154.208489	0.157441565	0.059455592	2.648053116	0.00809568	0.052171764	UAP1	protein_coding
ENSG00000117148.8	114.9717793	1.514628661	0.290606635	5.211954849	1.86861E-07	1.35488E-05	ACTL8	protein_coding
ENSG00000117151.13	408.1167194	-0.032765374	0.055795745	-0.587237858	0.557043969	0.747591779	CTBS	protein_coding
ENSG00000117152.14	538.8599186	0.46012891	0.157752534	2.916776654	0.003536689	0.029024163	RGS4	protein_coding
ENSG00000117153.16	2329.25704	-0.037107331	0.046065096	-0.805541174	0.420507456	0.640320203	KLHL12	protein_coding
ENSG00000117154.12	97.11255196	0.16786854	0.143524483	1.169616058	0.242155512	0.468018395	IGSF21	protein_coding
ENSG00000117155.17	1980.312631	-0.223734381	0.070076202	-3.192729839	0.001409347	0.014895739	SSX2IP	protein_coding
ENSG00000117174.11	1325.834551	0.001795514	0.042000059	0.042750266	0.96590061	0.984447023	ZNHIT6	protein_coding
ENSG00000117215.15	447.9964968	-0.732549333	0.209142116	-3.502639004	0.000460673	0.006409567	PLA2G2D	protein_coding
ENSG00000117222.14	1753.097566	0.069244474	0.045236946	1.530706208	0.125842019	0.316307184	RBBP5	protein_coding
ENSG00000117226.12	4259.752989	-0.077183161	0.119621822	-0.64522643	0.518780461	0.719506561	GBP3	protein_coding
ENSG00000117228.10	5626.075805	-0.146253224	0.122462581	-1.194268665	0.232372891	0.456686667	GBP1	protein_coding
ENSG00000117242.7	252.430545	-0.020785746	0.065446138	-0.317600798	0.750787768	0.872071097	PINK1-AS	lncRNA
ENSG00000117245.13	105.4670282	-0.125280592	0.114865172	-1.090675175	0.275415838	0.50422972	KIF17	protein_coding
ENSG00000117262.19	348.0712901	-0.071727465	0.063165659	-1.135545254	0.256146932	0.483082959	GPR89A	protein_coding
ENSG00000117266.16	2443.116316	0.049025282	0.079940735	0.613270345	0.539697598	0.735369781	CDK18	protein_coding
ENSG00000117280.13	1680.913668	-0.11968981	0.067252436	-1.779709661	0.075123488	0.230555409	RAB29	protein_coding
ENSG00000117281.16	67.50266964	-0.417045001	0.119473804	-3.490681518	0.00048179	0.006616758	CD160	protein_coding
ENSG00000117298.16	10534.92936	0.13591759	0.064869835	2.095235624	0.036150052	0.143885973	ECE1	protein_coding
ENSG00000117305.15	1978.617162	-0.174624415	0.055783799	-3.130378657	0.001745811	0.017366492	HMGCL	protein_coding
ENSG00000117308.15	4186.369677	-0.10531284	0.092903787	-1.133568865	0.256975439	0.484009694	GALE	protein_coding
ENSG00000117318.9	2613.220217	0.12958464	0.087082774	1.488062854	0.136734319	0.332773488	ID3	protein_coding
ENSG00000117322.18	1648.88622	-1.196156806	0.254275634	-4.704173917	2.54896E-06	0.000109759	CR2	protein_coding
ENSG00000117335.20	18147.98284	0.144099298	0.067722571	2.127788356	0.033354635	0.136478398	CD46	protein_coding
ENSG00000117360.13	2297.71873	0.148639319	0.049457925	3.00536908	0.002652588	0.023451372	PRPF3	protein_coding
ENSG00000117362.13	7945.437309	0.022580369	0.058430663	0.386447253	0.69916546	0.841761111	APH1A	protein_coding
ENSG00000117385.16	2327.519978	0.421774522	0.061892087	6.814676086	9.44764E-12	3.77706E-09	P3H1	protein_coding
ENSG00000117394.24	10568.97761	0.780291817	0.130392879	5.984159721	2.1751E-09	3.4467E-07	SLC2A1	protein_coding
ENSG00000117395.13	3950.046852	0.011919511	0.054617673	0.218235432	0.827245679	0.914923098	EBNA1BP2	protein_coding
ENSG00000117399.14	3618.293821	0.031888721	0.1004526	0.317450425	0.75090185	0.872122866	CDC20	protein_coding
ENSG00000117400.18	82.05919869	0.030279288	0.092534407	0.327221935	0.743500031	0.868090185	MPL	protein_coding
ENSG00000117407.17	100.9403975	0.468493471	0.151080329	3.100956123	0.001928969	0.01864242	ARTN	protein_coding

ENSG00000117408.11	2441.782008	0.081955462	0.046340869	1.768535281	0.076971459	0.234230816	IPO13	protein_coding
ENSG00000117410.14	7230.52549	-0.012567241	0.062358117	-0.201533362	0.840281545	0.921621628	ATP6V0B	protein_coding
ENSG00000117411.17	3490.924604	0.080424605	0.054604084	1.472867945	0.140786647	0.338651251	B4GALT2	protein_coding
ENSG00000117419.16	2697.961034	0.012875702	0.046801755	0.275111526	0.783230556	0.89011674	ERI3	protein_coding
ENSG00000117425.14	407.6335679	0.113059185	0.16488261	0.685695021	0.492905447	0.699926378	PTCH2	protein_coding
ENSG00000117448.14	6191.233035	-0.099003598	0.059001575	-1.677982286	0.093350557	0.26410032	AKR1A1	protein_coding
ENSG00000117450.15	13376.52716	-0.119510624	0.064299764	-1.858647953	0.063077055	0.206134097	PRDX1	protein_coding
ENSG00000117461.15	1470.754658	-0.119336204	0.072089114	-1.655398398	0.097843711	0.271904558	PIK3R3	protein_coding
ENSG00000117472.10	11179.87435	0.327604094	0.161124125	2.033240488	0.042028232	0.15906412	TSPAN1	protein_coding
ENSG00000117475.14	1895.788399	0.119955453	0.051735021	2.318650902	0.020413973	0.098589101	BLZF1	protein_coding
ENSG00000117477.12	73.32922425	-0.066903542	0.132987133	-0.503082817	0.614906047	0.788602281	CCDC181	protein_coding
ENSG00000117479.15	1356.496915	0.239267042	0.070568453	3.390566612	0.000697483	0.008726727	SLC19A2	protein_coding
ENSG00000117480.16	1212.15678	-0.072858614	0.083262387	-0.875048344	0.381547602	0.60688031	FAAH	protein_coding
ENSG00000117481.10	1480.887861	0.078561387	0.03669044	2.141195039	0.032258313	0.133732632	NSUN4	protein_coding
ENSG00000117500.13	5124.836788	-0.12374107	0.05287609	-2.340208415	0.019272981	0.094557387	TMED5	protein_coding
ENSG00000117501.14	10.37279407	-0.642987876	0.199566468	-3.221923416	0.001273332	0.013802978	MROH9	protein_coding
ENSG00000117505.13	4334.471434	-0.044688361	0.036867072	-1.21214837	0.225455591	0.448492572	DR1	protein_coding
ENSG00000117507.6	10.83517573	-0.510891152	0.214793794	-2.378519148	0.017382334	0.08840686	FMO6P	transcribed_unitary_pseudogene
ENSG00000117519.16	6044.828038	0.01417097	0.081640203	0.173578329	0.862196865	0.932883995	CNN3	protein_coding
ENSG00000117523.16	22548.48147	0.076171482	0.04387505	1.736100179	0.082546123	0.244851432	PRRC2C	protein_coding
ENSG00000117525.14	3933.49334	0.795643054	0.122279611	6.506751572	7.67933E-11	2.21617E-08	F3	protein_coding
ENSG00000117528.14	4204.976477	-0.189088625	0.061472025	-3.076011013	0.002097901	0.019830853	ABCD3	protein_coding
ENSG00000117533.15	1160.062806	0.09425934	0.051716414	1.822619392	0.068361056	0.216646672	VAMP4	protein_coding
ENSG00000117543.21	946.9852672	-0.22404347	0.051349656	-4.363095821	1.28235E-05	0.000394918	DPH5	protein_coding
ENSG00000117560.8	92.1250352	-0.676436272	0.145118864	-4.66125667	3.14284E-06	0.000128356	FASLG	protein_coding
ENSG00000117569.19	1160.923271	-0.270886882	0.076177292	-3.556005664	0.000376536	0.00549692	PTBP2	protein_coding
ENSG00000117586.11	308.8498123	0.418732968	0.115877918	3.613569996	0.00030201	0.004666908	TNFSF4	protein_coding
ENSG00000117592.9	9943.105462	0.010058983	0.052339729	0.192186386	0.84759621	0.925345272	PRDX6	protein_coding
ENSG00000117593.12	3160.206658	0.01211717	0.077124718	0.157111371	0.875157076	0.93966865	DARS2	protein_coding
ENSG00000117594.10	240.4000809	0.182789589	0.141068079	1.295754435	0.195060101	0.412147693	HSD11B1	protein_coding
ENSG00000117595.12	4965.088906	0.033637264	0.084574395	0.397723972	0.690833665	0.837004621	IRF6	protein_coding
ENSG00000117597.18	2403.999052	0.054032561	0.052535975	1.028486883	0.303720858	0.533206181	UTP25	protein_coding
ENSG00000117598.13	6.133110468	0.142702755	0.251926646	0.566445656	0.571090865	0.757370177	PLPPR5	protein_coding
ENSG00000117600.12	354.1833629	0.338291254	0.142209289	2.378826696	0.017367839	0.088398677	PLPPR4	protein_coding
ENSG00000117601.14	8.560060519	0.090606741	0.168498841	0.537729161	0.590764044	0.771671255	SERPINC1	protein_coding
ENSG00000117602.13	2027.586903	-0.167046522	0.064200982	-2.601930943	0.009270052	0.057315698	RCAN3	protein_coding
ENSG00000117614.10	3229.740976	-0.091832617	0.047855974	-1.918937373	0.05499226	0.189151614	SYF2	protein_coding
ENSG00000117616.18	2248.642492	-0.027147115	0.072526497	-0.374306174	0.708176539	0.847156272	RSRP1	protein_coding
ENSG00000117620.15	3909.059971	-0.202205196	0.080109887	-2.524097873	0.011599563	0.067024541	SLC35A3	protein_coding
ENSG00000117625.14	2387.317291	-0.018034391	0.048894844	-0.368840352	0.712246726	0.849622939	RCOR3	protein_coding
ENSG00000117632.23	7290.175533	-0.158823442	0.080081476	-1.983273167	0.047336925	0.171788283	STMN1	protein_coding
ENSG00000117640.18	1295.849707	-0.119438301	0.049807627	-2.397992178	0.016485217	0.085561733	MTFR1L	protein_coding

ENSG00000117643.14	1215.994579	0.098713949	0.112730132	0.875666051	0.381211606	0.60659465	MAN1C1	protein_coding
ENSG00000117650.13	1462.77969	0.016639363	0.099473526	0.167274283	0.867154238	0.935670288	NEK2	protein_coding
ENSG00000117676.14	5250.466751	-0.056780519	0.075403901	-0.753018324	0.451438903	0.666136837	RPS6KA1	protein_coding
ENSG00000117682.17	2111.341145	0.001594158	0.044839886	0.035552223	0.971639405	0.986604771	DHDDS	protein_coding
ENSG00000117691.10	2778.929827	-0.088010362	0.074750923	-1.177381616	0.239043224	0.464427861	NENF	protein_coding
ENSG00000117697.15	1613.899133	-0.102626372	0.046599371	-2.202312363	0.027643251	0.120654043	NSL1	protein_coding
ENSG00000117707.16	1638.312854	0.247190004	0.210820807	1.172512372	0.240991408	0.466596285	PROX1	protein_coding
ENSG00000117713.20	11069.90014	0.015031541	0.050885764	0.295397763	0.767690042	0.882241701	ARID1A	protein_coding
ENSG00000117724.13	6046.890897	0.162401326	0.099236618	1.63650605	0.101733721	0.278330634	CENPF	protein_coding
ENSG00000117748.10	1955.418962	-0.103840579	0.047150179	-2.202336893	0.02764152	0.120654043	RPA2	protein_coding
ENSG00000117751.18	2071.268745	-0.090442635	0.036251196	-2.494886948	0.012599727	0.070928599	PPP1R8	protein_coding
ENSG00000117758.14	3267.335406	-0.091345798	0.049081922	-1.861088443	0.062731688	0.205476456	STX12	protein_coding
ENSG00000117791.16	994.3976599	-0.134741147	0.082895292	-1.625437872	0.104069292	0.282046616	MTARC2	protein_coding
ENSG00000117834.13	215.9506713	-0.678445219	0.184724601	-3.672738855	0.000239965	0.003909137	SLC5A9	protein_coding
ENSG00000117859.19	4347.317037	0.051782049	0.046170336	1.121543703	0.2620565	0.489506192	OSBPL9	protein_coding
ENSG00000117862.13	357.1068502	0.087068987	0.071999741	1.209295841	0.226549208	0.449840372	TXNDC12	protein_coding
ENSG00000117868.17	8844.075691	0.08814408	0.061772121	1.426923339	0.153601993	0.357330453	ESYT2	protein_coding
ENSG00000117877.11	325.0519711	-0.0611338	0.0854529	-0.715409303	0.474356166	0.684883959	POLR1G	protein_coding
ENSG00000117899.11	3729.961429	0.097331284	0.047919868	2.031125879	0.042242227	0.15965217	MESD	protein_coding
ENSG00000117906.14	4372.209286	0.042166671	0.055926904	0.753960399	0.450873001	0.665565613	RCN2	protein_coding
ENSG00000117971.12	63.93646196	-0.019115958	0.158361324	-0.120711027	0.903919924	0.954171989	CHRNB4	protein_coding
ENSG00000117983.17	18528.90254	1.807424372	0.256170514	7.055551961	1.71917E-12	9.13613E-10	MUC5B	protein_coding
ENSG00000117984.15	20719.81895	0.068682983	0.075183465	0.913538412	0.360959441	0.588660442	CTSD	protein_coding
ENSG00000118004.18	89.26168656	-0.069251656	0.142769219	-0.485060133	0.627633691	0.797131921	COLEC11	protein_coding
ENSG00000118007.13	2233.824851	0.137940061	0.05981202	2.306226421	0.021097984	0.100725631	STAG1	protein_coding
ENSG00000118017.4	403.542573	-0.859134317	0.283370333	-3.03184284	0.002430657	0.021957037	A4GNT	protein_coding
ENSG00000118046.16	3577.533749	0.006240194	0.056091123	0.111251011	0.911417302	0.958232768	STK11	protein_coding
ENSG00000118058.23	7432.016857	-0.052840736	0.052563708	-1.005270326	0.3147667	0.54400684	KMT2A	protein_coding
ENSG00000118094.12	100.5836862	-0.544192892	0.156944468	-3.467423215	0.000525474	0.00706528	TREH	protein_coding
ENSG00000118096.10	899.6412521	0.021675245	0.061960296	0.349824743	0.726470228	0.857343078	IFT46	protein_coding
ENSG00000118113.12	30.85625949	0.99595811	0.225627195	4.41417583	1.01396E-05	0.000329647	MMP8	protein_coding
ENSG00000118137.10	708.22509	-1.201454742	0.281851772	-4.262718431	2.01955E-05	0.00056162	APOA1	protein_coding
ENSG00000118156.12	50.88607547	0.133188241	0.188253112	0.707495561	0.47925857	0.688626045	ZNF541	protein_coding
ENSG00000118160.14	171.2575315	-0.376169263	0.231500706	-1.62491627	0.104180401	0.282277519	SLC8A2	protein_coding
ENSG00000118162.14	285.689659	-0.165991102	0.080078905	-2.072844307	0.038186772	0.149352213	KPTN	protein_coding
ENSG00000118181.11	22265.038	-0.24063761	0.06913268	-3.480808372	0.000499903	0.006789679	RPS25	protein_coding
ENSG00000118193.12	1018.790991	0.152580715	0.093220492	1.636772262	0.101678064	0.278230768	KIF14	protein_coding
ENSG00000118194.20	34.79112883	0.985541053	0.169337505	5.819980933	5.88543E-09	7.81919E-07	TNNT2	protein_coding
ENSG00000118197.14	856.3214904	-0.004244132	0.041471913	-0.102337513	0.91848878	0.96147949	DDX59	protein_coding
ENSG00000118200.14	2901.576706	0.219626531	0.055720058	3.941606308	8.09377E-05	0.001683544	CAMSAP2	protein_coding
ENSG00000118217.6	5279.748369	0.125679418	0.047292671	2.65748193	0.007872681	0.051188872	ATF6	protein_coding
ENSG00000118231.5	1.853454887	0.43409107	0.412369983	1.052673784	0.292490528	0.522328486	CRYGD	protein_coding

ENSG00000118242.16	594.7810141	0.004906349	0.073380652	0.066861621	0.946691866	0.974995071	MREG	protein_coding
ENSG00000118245.2	0.160908876	-0.356179421	1.024620666	-0.347620767	0.728125008		TNP1	protein_coding
ENSG00000118246.14	2711.918939	-0.062064623	0.046098569	-1.346345981	0.178190963	0.390811194	FASTKD2	protein_coding
ENSG00000118257.17	4223.908268	0.112099709	0.123350455	0.908790398	0.363460776	0.590784485	NRP2	protein_coding
ENSG00000118260.15	3145.237654	0.011932029	0.037915727	0.314698665	0.752990458	0.873100132	CREB1	protein_coding
ENSG00000118263.15	2150.551174	0.087981139	0.092431641	0.951850887	0.34117261	0.571203275	KLF7	protein_coding
ENSG00000118271.12	373.6009459	-1.54726027	0.275391177	-5.618409014	1.92724E-08	2.06347E-06	TTR	protein_coding
ENSG00000118276.11	566.7483023	-0.308336223	0.112660582	-2.736859852	0.006202871	0.043269173	B4GALT6	protein_coding
ENSG00000118292.9	357.989997	-0.076969176	0.083074122	-0.926512064	0.354179931	0.582374871	C1orf54	protein_coding
ENSG00000118298.12	97.58452566	0.053771688	0.115218962	0.46669131	0.640720748	0.805484971	CA14	protein_coding
ENSG00000118307.20	35.48515239	-0.077911543	0.164810416	-0.472734336	0.636402727	0.802933543	CFAP94	protein_coding
ENSG00000118308.15	397.7765289	-0.431828147	0.150735714	-2.864803138	0.004172686	0.032668545	IRAG2	protein_coding
ENSG00000118322.14	6102.711724	-0.182692921	0.177493039	-1.029296254	0.303340483	0.532764354	ATP10B	protein_coding
ENSG00000118363.12	3906.535715	-0.013096421	0.055812679	-0.23464957	0.814480732	0.908390327	SPCS2	protein_coding
ENSG00000118369.13	554.2379596	0.339604014	0.065845689	5.157574003	2.5017E-07	1.69544E-05	USP35	protein_coding
ENSG00000118402.6	103.0275037	0.524387533	0.142063095	3.691229836	0.000223172	0.003694978	ELOVL4	protein_coding
ENSG00000118407.15	449.9277489	-0.510505304	0.164269498	-3.107730355	0.0018853	0.018326058	FILIP1	protein_coding
ENSG00000118412.13	1569.175882	0.157450694	0.058676511	2.683368378	0.007288466	0.048371836	CASP8AP2	protein_coding
ENSG00000118418.14	2281.94328	-0.059211442	0.086456593	-0.684869018	0.493426578	0.700438139	HMGN3	protein_coding
ENSG00000118420.17	291.037733	-0.047535023	0.065085363	-0.730348905	0.465176942	0.677032016	UBE3D	protein_coding
ENSG00000118432.12	302.6611522	-0.724362764	0.214938475	-3.370093536	0.000751427	0.009257824	CNR1	protein_coding
ENSG00000118434.9	0.826349845	0.123220542	0.439789845	0.280180507	0.779339021		SPACA1	protein_coding
ENSG00000118454.13	1678.359354	0.073153298	0.039671059	1.843996593	0.065183637	0.210392366	ANKRD13C	protein_coding
ENSG00000118473.22	467.8177758	0.176285676	0.102092747	1.72672086	0.084217807	0.247930775	SGIP1	protein_coding
ENSG00000118482.12	5601.28426	0.148985837	0.055065701	2.705601373	0.006818083	0.046185273	PHF3	protein_coding
ENSG00000118491.10	0.780449942	-0.327902218	0.395339691	-0.829418917	0.406867401		ZC2HC1B	protein_coding
ENSG00000118492.17	8.789834992	-0.177202093	0.269756923	-0.65689544	0.511248151	0.714228033	ADGB	protein_coding
ENSG00000118495.20	1344.577505	0.240818445	0.108041761	2.22893854	0.025817994	0.115445596	PLAGL1	protein_coding
ENSG00000118496.5	1488.797289	-0.048822165	0.070669216	-0.69085478	0.489656806	0.697016779	FBXO30	protein_coding
ENSG00000118503.15	5610.885458	-0.043092374	0.087612207	-0.491853539	0.622822885	0.794123324	TNFAIP3	protein_coding
ENSG00000118507.17	526.9215505	-0.151498971	0.113246418	-1.337781574	0.18096765	0.394124008	AKAP7	protein_coding
ENSG00000118508.5	1130.039916	0.289146014	0.092686054	3.119628064	0.001810795	0.017788329	RAB32	protein_coding
ENSG00000118513.19	1745.218582	-0.005189449	0.136024487	-0.038150847	0.969567411	0.985856198	MYB	protein_coding
ENSG00000118514.14	26.39352345	0.141037701	0.138592469	1.017643326	0.30884749	0.538212042	ALDH8A1	protein_coding
ENSG00000118515.11	6039.310365	0.143503993	0.101378365	1.415528771	0.156913519	0.36190433	SGK1	protein_coding
ENSG00000118518.16	1248.892348	0.042470853	0.045672525	0.929899377	0.352423186	0.580944627	RNF146	protein_coding
ENSG00000118520.15	16.93045325	0.117400407	0.129718237	0.905041649	0.365443323	0.592665563	ARG1	protein_coding
ENSG00000118523.6	12791.27919	0.285577397	0.108954158	2.621078472	0.008765208	0.055067561	CCN2	protein_coding
ENSG00000118526.7	301.9617407	-0.019913041	0.12560284	-0.158539739	0.874031509	0.939114606	TCF21	protein_coding
ENSG00000118557.16	127.1860006	-0.32191049	0.140590411	-2.289704457	0.022038455	0.103666855	PMFBP1	protein_coding
ENSG00000118564.15	4605.721902	-0.057298844	0.050740878	-1.129244236	0.258794817	0.485973531	FBXL5	protein_coding
ENSG00000118579.13	2867.651654	-0.074906412	0.041509717	-1.804551249	0.071144933	0.222003776	MED28	protein_coding

ENSG00000118596.12	902.2055228	0.41115121	0.138423415	2.970243223	0.002975641	0.025485355	SLC16A7	protein_coding
ENSG00000118600.12	591.0274687	0.123235098	0.058784566	2.096385255	0.036048029	0.143575996	RXYLT1	protein_coding
ENSG00000118620.13	726.2452116	0.019431244	0.063342343	0.306765474	0.75902189	0.876647148	ZNF430	protein_coding
ENSG00000118640.11	5179.434965	-0.09433194	0.071040076	-1.327869344	0.184221274	0.39833339	VAMP8	protein_coding
ENSG00000118655.7	858.096111	-0.075840402	0.05527426	-1.37207449	0.17004025	0.379875114	DCLRE1B	protein_coding
ENSG00000118680.14	26482.91071	-0.097300186	0.063364236	-1.535569468	0.124644007	0.314511402	MYL12B	protein_coding
ENSG00000118689.15	5660.625663	0.19745276	0.062950444	3.136638075	0.001708969	0.01710029	FOXO3	protein_coding
ENSG00000118690.13	237.7830248	-0.220772051	0.083877735	-2.632069765	0.008486643	0.053870715	ARMC2	protein_coding
ENSG00000118702.9	3.433747206	-0.486441769	0.278250923	-1.748212597	0.080427221	0.240894701	GHRH	protein_coding
ENSG00000118705.17	27871.60166	0.028931955	0.064343498	0.449648456	0.652963943	0.813393934	RPN2	protein_coding
ENSG00000118707.11	1792.442087	-0.048991744	0.067743705	-0.723192566	0.469561586	0.680979939	TGIF2	protein_coding
ENSG00000118729.12	306.9189163	-0.764098785	0.223105528	-3.424831247	0.000615181	0.007941676	CASQ2	protein_coding
ENSG00000118733.17	5.150139275	-0.428048217	0.308777379	-1.386268055	0.165665068	0.373741284	OLFM3	protein_coding
ENSG00000118762.8	2099.409726	0.138538358	0.092384776	1.499579951	0.133723244	0.328407226	PKD2	protein_coding
ENSG00000118777.12	445.1995385	-0.508229073	0.143067315	-3.552377246	0.000381767	0.005554679	ABCG2	protein_coding
ENSG00000118785.14	10772.85205	0.41135737	0.200005436	2.056730951	0.039712113	0.153361818	SPP1	protein_coding
ENSG00000118804.8	135.2171836	0.1560065	0.078873709	1.977927766	0.047936856	0.173262872	STBD1	protein_coding
ENSG00000118816.11	22213.87882	0.039732736	0.045499052	0.873265134	0.38251858	0.607771138	CCNI	protein_coding
ENSG00000118849.10	2345.690243	1.147607791	0.158382604	7.245794446	4.29912E-13	2.69984E-10	RARRES1	protein_coding
ENSG00000118855.21	3848.665263	0.068222164	0.05577788	1.223104295	0.221290307	0.44371084	MFSD1	protein_coding
ENSG00000118873.16	3803.153096	-0.049440056	0.050379985	-0.98134322	0.326423516	0.556255184	RAB3GAP2	protein_coding
ENSG00000118894.15	438.3891738	0.011346283	0.06445357	0.17603808	0.860264026	0.932043596	EEF2KMT	protein_coding
ENSG00000118898.16	5185.593587	0.652043044	0.132115006	4.935420018	7.99785E-07	4.37842E-05	PPL	protein_coding
ENSG00000118900.15	4951.206194	-0.026962644	0.050989266	-0.528790597	0.596950721	0.776331669	UBN1	protein_coding
ENSG00000118903.6	1.948609416	-0.085393488	0.196012139	-0.43565408	0.663087727	0.819677064	BTF3P11	processed_pseudogene
ENSG00000118922.17	1180.848318	0.053431853	0.12179932	0.438687617	0.660887901	0.818265758	KLF12	protein_coding
ENSG00000118939.18	289.9448239	-0.283423668	0.096391696	-2.940332836	0.003278599	0.027470004	UCHL3	protein_coding
ENSG00000118946.12	1374.05407	0.020045077	0.093989504	0.213269312	0.831116909	0.916779941	PCDH17	protein_coding
ENSG00000118960.13	2408.74563	-0.113290451	0.059971338	-1.88907661	0.058881564	0.197459885	HS1BP3	protein_coding
ENSG00000118961.15	1252.817141	-0.050324879	0.074793477	-0.672851179	0.501041975	0.706324603	LDAH	protein_coding
ENSG00000118965.16	1021.519327	-0.041310436	0.085310847	-0.484234274	0.628219616	0.797620366	WDR35	protein_coding
ENSG00000118971.9	7449.17525	0.027936466	0.14279245	0.195643856	0.844888931	0.923911835	CCND2	protein_coding
ENSG00000118972.3	21.31821337	-0.520641983	0.216611067	-2.403579795	0.016235423	0.084658495	FGF23	protein_coding
ENSG00000118976.5	18.87251849	0.131940919	0.098760764	1.335964951	0.181560729	0.394902516	OTUD4P1	processed_pseudogene
ENSG00000118985.16	2937.502073	-0.100704139	0.090725186	-1.109990989	0.26700291	0.495093221	ELL2	protein_coding
ENSG00000118990.5	1.934529594	0.250002366	0.198523027	1.259311675	0.207917778	0.427919165	GLRXP3	processed_pseudogene
ENSG00000118997.14	107.1945546	0.18364113	0.125698907	1.460960432	0.144026301	0.343390788	DNAH7	protein_coding
ENSG00000119004.16	2828.564068	-0.173452869	0.05945956	-2.917156941	0.00353238	0.02900161	CYP20A1	protein_coding
ENSG00000119013.9	2520.092815	-0.16404868	0.061763792	-2.656065552	0.007905824	0.051327788	NDUFB3	protein_coding
ENSG00000119041.11	2202.182102	-0.062645149	0.049938807	-1.254438228	0.209682765	0.429891595	GTF3C3	protein_coding
ENSG00000119042.17	801.7752728	0.401843535	0.123585689	3.251537773	0.001147825	0.012756639	SATB2	protein_coding
ENSG00000119048.8	2882.942599	-0.019582007	0.053347111	-0.367067814	0.713568435	0.850431345	UBE2B	protein_coding

ENSG00000119121.22	177.8225465	0.235465098	0.195577879	1.203945455	0.228610661	0.452217702	TRPM6	protein_coding
ENSG00000119125.17	2538.922469	-0.02983602	0.165365438	-0.180424763	0.856819115	0.930161346	GDA	protein_coding
ENSG00000119138.4	4051.775732	0.010469034	0.110077964	0.09510563	0.924230926	0.964159838	KLF9	protein_coding
ENSG00000119139.21	419.3968613	0.04332189	0.073926682	0.586011562	0.557867745	0.748144357	TJP2	protein_coding
ENSG00000119147.10	541.6972063	-0.653613549	0.255010123	-2.563088643	0.010374556	0.061888024	ECRG4	protein_coding
ENSG00000119185.13	2071.669292	-0.081123797	0.044493868	-1.823257909	0.068264338	0.216534333	ITGB1BP1	protein_coding
ENSG00000119203.14	2503.592365	-0.094093483	0.045877904	-2.050954287	0.040271399	0.154603713	CPSF3	protein_coding
ENSG00000119227.8	511.4025253	0.099792758	0.111886347	0.891911841	0.372440188	0.59890867	PIGZ	protein_coding
ENSG00000119231.11	2134.276051	0.054014859	0.046922385	1.15115331	0.24966917	0.476413518	SENP5	protein_coding
ENSG00000119242.9	2718.004152	-0.157803015	0.063681023	-2.478022605	0.013211277	0.073406201	CCDC92	protein_coding
ENSG00000119280.17	4916.288748	0.075309665	0.058472253	1.287955594	0.197761421	0.415157985	C1orf198	protein_coding
ENSG00000119283.15	29.44316117	-0.30723062	0.137542837	-2.233708621	0.025502251	0.114462005	TRIM67	protein_coding
ENSG00000119285.11	3875.938344	0.092317914	0.068219754	1.35324315	0.175977964	0.387995609	HEATR1	protein_coding
ENSG00000119314.16	14334.33458	0.065232938	0.058790015	1.109592125	0.267174826	0.495191203	PTBP3	protein_coding
ENSG00000119318.13	12216.37224	0.007285305	0.048146664	0.151314847	0.879727358	0.942052368	RAD23B	protein_coding
ENSG00000119321.9	2847.173006	0.071949818	0.044761955	1.607387741	0.107969338	0.28797771	FKBP15	protein_coding
ENSG00000119326.15	1690.318233	-0.126070164	0.076915462	-1.639074396	0.101197767	0.277460178	CTNNAL1	protein_coding
ENSG00000119328.12	1460.139611	-0.117155891	0.064306976	-1.821822432	0.068481933	0.216861942	ABITRAM	protein_coding
ENSG00000119333.11	4499.017676	0.007522576	0.076120582	0.098824469	0.92127764	0.962915133	DYNC2I2	protein_coding
ENSG00000119335.17	18556.04264	0.022891626	0.050323781	0.454886853	0.649190641	0.810652982	SET	protein_coding
ENSG00000119383.20	6149.943901	-0.06942831	0.061365866	-1.131383191	0.257893843	0.48492645	PTPA	protein_coding
ENSG00000119392.15	2461.63499	0.000583204	0.053532118	0.010894469	0.991307643	0.996016075	GLE1	protein_coding
ENSG00000119396.11	6605.042118	0.044270997	0.045937142	0.96372989	0.335181365	0.564665339	RAB14	protein_coding
ENSG00000119397.17	1547.34085	-0.202484654	0.067919241	-2.981256155	0.002870686	0.024825534	CNTRL	protein_coding
ENSG00000119401.11	1073.679929	0.143521765	0.050584572	2.837263574	0.004550203	0.034825011	TRIM32	protein_coding
ENSG00000119402.17	4240.019865	0.002129516	0.042639334	0.049942522	0.960168192	0.981470642	FBXW2	protein_coding
ENSG00000119403.15	2421.663257	-0.085415339	0.062454718	-1.367636285	0.171425953	0.381913535	PHF19	protein_coding
ENSG00000119408.17	5184.035769	-0.000846153	0.068914552	-0.012278294	0.990203585	0.995567528	NEK6	protein_coding
ENSG00000119411.11	1280.664049	-0.136165627	0.092806344	-1.467201714	0.142321191	0.340915267	BSPRY	protein_coding
ENSG00000119414.12	4336.539619	-0.038184892	0.039334566	-0.970771901	0.331661878	0.561183174	PPP6C	protein_coding
ENSG00000119421.7	2134.999829	-0.153294581	0.057482869	-2.66678723	0.007658014	0.050159817	NDUFA8	protein_coding
ENSG00000119431.9	3710.386449	-0.210281644	0.076331353	-2.754852841	0.005871854	0.041673388	HDHD3	protein_coding
ENSG00000119440.8	4.046268022	0.272600912	0.197381813	1.381084243	0.16725306	0.376097145	LCN1P1	unprocessed_pseudogene
ENSG00000119446.14	2113.647039	0.072443375	0.045951569	1.576515814	0.114906977	0.299211314	RBM18	protein_coding
ENSG00000119457.8	13.72803117	0.441495811	0.151269665	2.918601107	0.003516058	0.028909297	SLC46A2	protein_coding
ENSG00000119471.15	3774.927572	-0.256972068	0.060814914	-4.225477778	2.38434E-05	0.000639007	HSDL2	protein_coding
ENSG00000119487.17	3924.789455	0.027885622	0.042174892	0.661190127	0.508490392	0.712033606	MAPKAP1	protein_coding
ENSG00000119508.18	1100.68333	0.348378199	0.14740822	2.363356658	0.018110231	0.09081085	NR4A3	protein_coding
ENSG00000119509.13	1068.476284	0.034369668	0.04682571	0.733991398	0.46295398	0.675262606	INVS	protein_coding
ENSG00000119514.7	1949.089232	-0.025420844	0.095196971	-0.267034173	0.78944285	0.894190524	GALNT12	protein_coding
ENSG00000119522.16	2798.219788	0.01227896	0.056707572	0.216531215	0.828573696	0.915617622	DENND1A	protein_coding
ENSG00000119523.10	2393.250725	0.002729093	0.049862365	0.05473252	0.956351561	0.979676141	ALG2	protein_coding

ENSG00000119535.18	1220.79806	0.301201849	0.13558672	2.221470136	0.026319135	0.116840766	CSF3R	protein_coding
ENSG00000119537.18	4412.924151	0.058780615	0.056484068	1.040658326	0.298034151	0.527883203	KDSR	protein_coding
ENSG00000119541.10	3988.254386	-0.025267689	0.059326355	-0.425910011	0.67017342	0.824091289	VPS4B	protein_coding
ENSG00000119547.6	5537.194398	0.083375181	0.162574591	0.51284263	0.608061407	0.784088653	ONECUT2	protein_coding
ENSG00000119559.17	1340.623682	0.014222622	0.061043863	0.23299021	0.815769008	0.909044813	C19orf25	protein_coding
ENSG00000119574.13	1558.940722	0.09053984	0.055955392	1.618071756	0.105647135	0.284570725	ZBTB45	protein_coding
ENSG00000119596.18	4601.030202	0.010783936	0.04111271	0.262301754	0.79308881	0.896262994	YLPM1	protein_coding
ENSG00000119599.17	806.1319998	-0.060239048	0.059403798	-1.01406055	0.310553861	0.539981071	DCAF4	protein_coding
ENSG00000119608.14	34.14968899	-0.234470607	0.118805756	-1.973562695	0.048431489	0.174320558	PROX2	protein_coding
ENSG00000119614.3	3.016197162	0.089468725	0.235729498	0.379539795	0.704287059	0.844845904	VSX2	protein_coding
ENSG00000119616.11	1993.588084	-0.001573825	0.037569392	-0.041891152	0.96658547	0.984499865	FCF1	protein_coding
ENSG00000119630.14	739.7831011	0.152929674	0.095866857	1.595229868	0.110660842	0.29235175	PGF	protein_coding
ENSG00000119632.4	1120.744034	0.020125399	0.093627801	0.2149511	0.829805444	0.916289069	IFI27L2	protein_coding
ENSG00000119636.16	214.835257	0.071551162	0.074242777	0.963745759	0.335173407	0.564665339	BBOF1	protein_coding
ENSG00000119638.14	3326.535978	-0.008866632	0.050032675	-0.177216825	0.859338081	0.931526755	NEK9	protein_coding
ENSG00000119640.9	525.5871585	-0.125622602	0.080075797	-1.568796152	0.116695445	0.302009586	ACYP1	protein_coding
ENSG00000119650.13	819.256058	-0.070112478	0.047396162	-1.479285966	0.139063902	0.336157304	IFT43	protein_coding
ENSG00000119655.11	6484.67651	-0.125825965	0.06602099	-1.905847883	0.056669944	0.192809664	NPC2	protein_coding
ENSG00000119660.4	0.623083924	0.700002155	0.365699756	1.914144439	0.055601703		DPPA5P4	processed_pseudogene
ENSG00000119661.15	766.8304901	0.059829976	0.045005234	1.329400389	0.183715909	0.39764507	DNAL1	protein_coding
ENSG00000119669.5	3550.699988	0.022099968	0.06683958	0.330641926	0.740914973	0.866079013	IRF2BPL	protein_coding
ENSG00000119673.14	1097.720345	-0.068770016	0.069004513	-0.99660171	0.318957875	0.548434373	ACOT2	protein_coding
ENSG00000119681.12	8159.2167	0.598677457	0.113035368	5.296372881	1.18126E-07	9.19206E-06	LTBP2	protein_coding
ENSG00000119682.17	2880.025423	0.074747066	0.049991523	1.495194793	0.134863612	0.330110185	AREL1	protein_coding
ENSG00000119684.16	1238.536595	-0.113867993	0.050921709	-2.236138481	0.025342701	0.114067108	MLH3	protein_coding
ENSG00000119685.20	1892.716101	0.033593342	0.04032044	0.83315912	0.404754995	0.626416465	TTLL5	protein_coding
ENSG00000119686.10	352.8401674	0.023103063	0.09418816	0.245286279	0.806234775	0.90368056	FLVCR2	protein_coding
ENSG00000119688.21	1704.239273	0.025791707	0.042357367	0.608907242	0.542585924	0.737554618	ABCD4	protein_coding
ENSG00000119689.15	5097.614467	0.050218715	0.050468193	0.995056736	0.319708671	0.549301927	DLST	protein_coding
ENSG00000119698.12	61.37075761	-0.952698207	0.18473941	-5.156984142	2.50959E-07	1.69814E-05	PPP4R4	protein_coding
ENSG00000119699.8	958.0141977	0.425748377	0.123921878	3.435619144	0.000591201	0.007715719	TGFB3	protein_coding
ENSG00000119703.15	213.446501	-0.127680069	0.067087285	-1.903193275	0.05701533	0.193691861	ZC2HC1C	protein_coding
ENSG00000119705.10	2678.780584	-0.253672198	0.080247501	-3.161122712	0.001571623	0.016047147	SLIRP	protein_coding
ENSG00000119707.14	9369.704394	-0.113119691	0.059054411	-1.915516357	0.055426685	0.190063634	RBM25	protein_coding
ENSG00000119711.13	1946.34226	-0.074146516	0.071258958	-1.040522049	0.298097426	0.527949259	ALDH6A1	protein_coding
ENSG00000119714.11	627.3634777	0.267436707	0.101792271	2.627279107	0.00860707	0.054397434	GPR68	protein_coding
ENSG00000119715.15	29.48341283	-0.035254659	0.153087233	-0.230291308	0.817865415	0.910208427	ESRRB	protein_coding
ENSG00000119718.11	1470.295474	-0.019048255	0.053557781	-0.355658026	0.722096682	0.85507596	EIF2B2	protein_coding
ENSG00000119720.18	1014.517075	-0.030441882	0.040718307	-0.747621494	0.454688497	0.668821251	NRDE2	protein_coding
ENSG00000119723.17	331.6046324	-0.091532319	0.045343754	-2.018631275	0.043525558	0.16259131	COQ6	protein_coding
ENSG00000119725.20	48.92815488	0.008936986	0.079778641	0.112022285	0.910805737	0.957841438	ZNF410	protein_coding
ENSG00000119729.12	2441.669582	0.012462142	0.063754291	0.195471428	0.845023903	0.923981276	RHOQ	protein_coding

ENSG00000119737.6	102.6017685	-0.153523944	0.080249463	-1.913083751	0.055737332	0.190690173	GPR75	protein_coding
ENSG00000119760.16	2451.111394	-0.020552434	0.035626231	-0.57689049	0.564013436	0.752841409	SUPT7L	protein_coding
ENSG00000119771.15	754.4055131	0.271065899	0.104365803	2.597267403	0.009396874	0.057820894	KLHL29	protein_coding
ENSG00000119772.17	2202.387183	0.013344682	0.071519135	0.186588968	0.851982917	0.927661276	DNMT3A	protein_coding
ENSG00000119777.20	10610.42521	0.112548155	0.046225546	2.434760957	0.01490163	0.079741476	TMEM214	protein_coding
ENSG00000119778.15	1237.037542	-0.046622293	0.049473899	-0.942361407	0.346007639	0.575414494	ATAD2B	protein_coding
ENSG00000119782.14	212.3029795	-0.172705253	0.130079576	-1.327689241	0.184280789	0.398384398	FKBP1B	protein_coding
ENSG00000119787.14	3996.772438	-0.052987871	0.056101077	-0.944507197	0.344910526	0.574508081	ATL2	protein_coding
ENSG00000119801.13	3559.467789	-0.027913519	0.05151275	-0.541875911	0.587903993	0.769850721	YPEL5	protein_coding
ENSG00000119812.19	2968.863668	-0.031991957	0.040188759	-0.7960424	0.426007391	0.644691913	FAM98A	protein_coding
ENSG00000119820.11	4774.440823	-0.070372813	0.048996162	-1.436292354	0.150919169	0.353258023	YIPF4	protein_coding
ENSG00000119844.15	4547.207191	-0.033675962	0.039625907	-0.849847113	0.395410092	0.618757402	AFTPH	protein_coding
ENSG00000119862.13	1429.553186	0.006427213	0.068808431	0.09340735	0.925579952	0.964905088	LGALSL	protein_coding
ENSG00000119865.9	546.4528272	0.021593338	0.103679743	0.208269592	0.835018473	0.919166016	CNRIP1	protein_coding
ENSG00000119866.22	1360.05021	-0.561522224	0.148843176	-3.772576219	0.000161571	0.002880835	BCL11A	protein_coding
ENSG00000119878.6	1666.867244	0.012362077	0.043600601	0.283529971	0.77677061	0.886799025	CRIPT	protein_coding
ENSG00000119888.11	34387.72247	-0.10548383	0.090893691	-1.160518732	0.245837672	0.472369515	EPCAM	protein_coding
ENSG00000119899.13	2222.46753	0.088244451	0.073803888	1.195661285	0.231828765	0.455985181	SLC17A5	protein_coding
ENSG00000119900.9	1659.409838	0.090077759	0.093082493	0.967719664	0.333184394	0.562758774	OGFRL1	protein_coding
ENSG00000119906.13	1895.410222	-0.082167103	0.053478	-1.536465519	0.124424248	0.314175192	SLF2	protein_coding
ENSG00000119912.18	4657.428891	-0.044358785	0.051799752	-0.856351307	0.39180349	0.615832137	IDE	protein_coding
ENSG00000119913.6	7.40432359	-0.46476559	0.216444829	-2.147270471	0.031771755	0.132455147	TECTB	protein_coding
ENSG00000119915.5	31.44096301	0.1359032	0.17024911	0.798260854	0.42471913	0.643666465	ELOVL3	protein_coding
ENSG00000119917.14	3060.086668	0.041973464	0.11967595	0.350725974	0.725793938	0.857032366	IFIT3	protein_coding
ENSG00000119919.11	97.97416004	-0.293924723	0.152121234	-1.932174191	0.05333801	0.185329999	NKX2-3	protein_coding
ENSG00000119922.10	1050.40528	0.164950652	0.12094671	1.36382918	0.172621334	0.383322456	IFIT2	protein_coding
ENSG00000119927.14	1194.770864	-0.266733924	0.089184473	-2.990811229	0.002782374	0.024239809	GPAM	protein_coding
ENSG00000119929.13	529.6509777	-0.210114722	0.045027298	-4.666385291	3.06545E-06	0.000126232	CUTC	protein_coding
ENSG00000119938.9	494.4380194	-0.242660374	0.18114117	-1.339620219	0.180368847	0.393390052	PPP1R3C	protein_coding
ENSG00000119943.13	292.8730707	0.291855349	0.094731728	3.08086166	0.002064025	0.019600773	PYROXD2	protein_coding
ENSG00000119946.11	514.044657	-0.151679087	0.185123994	-0.819337808	0.412593707	0.633240446	CNNM1	protein_coding
ENSG00000119950.21	2069.237141	0.017216899	0.073707145	0.233585211	0.81530701	0.908800163	MXI1	protein_coding
ENSG00000119953.14	1880.179186	-0.000208885	0.04492318	-0.00464982	0.996289994	0.998266626	SMNDC1	protein_coding
ENSG00000119965.13	517.5843896	-0.072420109	0.055286489	-1.309906084	0.190227609	0.405813311	C10orf88	protein_coding
ENSG00000119969.15	2068.647763	-0.185623191	0.08204968	-2.262326812	0.023677219	0.108883725	HELLS	protein_coding
ENSG00000119973.6	12.52052764	-0.100949127	0.278165018	-0.362910935	0.716671418	0.851968579	PRLHR	protein_coding
ENSG00000119977.21	2821.481674	0.018098989	0.049739107	0.363878455	0.715948774	0.851614568	TCTN3	protein_coding
ENSG00000119979.18	986.3176421	-0.03090557	0.049123406	-0.629141432	0.529256468	0.7274376	DENND10	protein_coding
ENSG00000119986.7	2113.102842	0.122608724	0.093792572	1.30723277	0.191133669	0.406989099	AVPI1	protein_coding
ENSG00000120008.16	3398.269234	-0.029747808	0.050219041	-0.592361137	0.553608784	0.745000617	WDR11	protein_coding
ENSG00000120029.13	2313.663031	-0.125246091	0.040254011	-3.1113941	0.001862062	0.018159691	ARMH3	protein_coding
ENSG00000120049.20	73.27025158	0.028517704	0.104855239	0.271972147	0.785643436	0.89151565	KCNIP2	protein_coding

ENSG00000120051.15	18.36013019	-0.106197587	0.142361953	-0.745968878	0.455686215	0.669660345	CFAP58	protein_coding
ENSG00000120053.12	3634.437471	-0.201744154	0.071678165	-2.814583151	0.004884054	0.036606884	GOT1	protein_coding
ENSG00000120054.12	24.23479701	-0.046853928	0.347398352	-0.134870898	0.892713949	0.948824287	CPN1	protein_coding
ENSG00000120055.7	3.826559052	0.182562538	0.161292516	1.13187234	0.257688109	0.484772696	C10orf95	protein_coding
ENSG00000120057.5	265.4609907	-0.802271294	0.24763652	-3.239713161	0.0011965	0.013169962	SFRP5	protein_coding
ENSG00000120063.10	7390.291678	-0.032871051	0.050517319	-0.650688743	0.515247431	0.716870509	GNA13	protein_coding
ENSG00000120068.7	362.1120285	0.514184966	0.216122146	2.379140569	0.017353057	0.088350762	HOXB8	protein_coding
ENSG00000120071.15	4783.145721	0.038186898	0.04736814	0.806172631	0.420143318	0.639989273	KANSL1	protein_coding
ENSG00000120075.5	597.1765393	0.26662514	0.123110751	2.165733999	0.030331519	0.128475566	HOXB5	protein_coding
ENSG00000120088.15	0.831557897	0.036661143	0.391511215	0.093640083	0.925395068		CRHR1	protein_coding
ENSG00000120093.11	1342.8937	0.328943819	0.114198043	2.880468093	0.003970851	0.031599305	HOXB3	protein_coding
ENSG00000120094.9	8.653231464	0.693124952	0.234295237	2.958339915	0.003093008	0.026289064	HOXB1	protein_coding
ENSG00000120129.6	13270.35966	0.409152696	0.119591801	3.421243701	0.000623354	0.00801532	DUSP1	protein_coding
ENSG00000120137.7	4991.577843	0.142483216	0.062990947	2.261963402	0.023699665	0.108940958	PANK3	protein_coding
ENSG00000120149.9	1069.334215	-0.243126113	0.145491193	-1.671070996	0.094707655	0.266183026	MSX2	protein_coding
ENSG00000120156.22	730.2177623	-0.07286583	0.101603648	-0.717157615	0.473276849	0.684159405	TEK	protein_coding
ENSG00000120158.12	1042.934733	-0.123737392	0.070492757	-1.755320648	0.079204477	0.238659485	RCL1	protein_coding
ENSG00000120159.13	1421.808585	-0.229624495	0.071906071	-3.193395082	0.001406104	0.014887199	CAAP1	protein_coding
ENSG00000120160.11	5.826456777	-0.168189013	0.15874529	-1.05948978	0.289376781	0.519093771	EQTN	protein_coding
ENSG00000120162.10	2447.584944	-0.297874333	0.098857322	-3.013174203	0.002585305	0.023024693	MOB3B	protein_coding
ENSG00000120210.8	18.17655269	0.374534774	0.162587015	2.303595858	0.021245341	0.101197428	INSL6	protein_coding
ENSG00000120211.4	19.78978472	1.134991144	0.290124061	3.912088986	9.15012E-05	0.001852856	INSL4	protein_coding
ENSG00000120215.10	70.62922734	0.000161324	0.092457735	0.001744841	0.998607819	0.999341668	MLANA	protein_coding
ENSG00000120217.14	475.2611384	-0.012907085	0.134259728	-0.096135198	0.923413197	0.963825309	CD274	protein_coding
ENSG00000120235.4	0.546613779	-0.52125446	0.569677467	-0.914999259	0.360192019		IFNA6	protein_coding
ENSG00000120242.4	0.500234481	0.428812426	0.432983735	0.99036613	0.321995194		IFNA8	protein_coding
ENSG00000120251.21	15.33501356	0.694994537	0.247478514	2.808302529	0.004980342	0.037117897	GRIA2	protein_coding
ENSG00000120253.14	2341.925866	0.064656691	0.049328711	1.310731409	0.189948523	0.405515716	NUP43	protein_coding
ENSG00000120254.16	2019.3661	0.108165761	0.068658964	1.575406259	0.115162701	0.299537126	MTHFD1L	protein_coding
ENSG00000120256.11	118.8821265	0.213501284	0.073779854	2.893761259	0.003806575	0.030607259	LRP11	protein_coding
ENSG00000120262.10	339.3834438	-0.614227488	0.128086876	-4.795397517	1.62353E-06	7.59103E-05	CCDC170	protein_coding
ENSG00000120265.18	2590.717808	0.044913885	0.047424739	0.947056035	0.343610226	0.573192315	PCMT1	protein_coding
ENSG00000120278.16	2373.862836	0.206542572	0.073381124	2.81465532	0.004882957	0.036604961	PLEKHG1	protein_coding
ENSG00000120279.6	462.8088779	-0.111691039	0.091101762	-1.226003058	0.220197524	0.442150378	MYCT1	protein_coding
ENSG00000120280.6	135.9610592	-0.336253534	0.109788561	-3.062737416	0.002193224	0.020447971	CXorf21	protein_coding
ENSG00000120289.12	0.48731512	-0.299041214	0.461762467	-0.647608317	0.517238317		MAGEB4	protein_coding
ENSG00000120306.11	19855.84204	-0.385899645	0.123126002	-3.134184801	0.001723323	0.017200467	CYSTM1	protein_coding
ENSG00000120314.18	1807.492039	0.006036928	0.050914256	0.118570492	0.905615644	0.955105712	WDR55	protein_coding
ENSG00000120318.16	1686.004405	0.107325356	0.08452683	1.269719405	0.204184598	0.4235613	ARAP3	protein_coding
ENSG00000120322.4	159.6951158	0.952896544	0.18615285	5.118893117	3.07334E-07	2.0079E-05	PCDHB8	protein_coding
ENSG00000120324.9	196.3452872	0.48402087	0.100942704	4.79500598	1.6267E-06	7.59631E-05	PCDHB10	protein_coding
ENSG00000120327.6	478.9705756	0.506910943	0.096528756	5.251398299	1.50949E-07	1.12828E-05	PCDHB14	protein_coding

ENSG00000120328.6	117.2604886	0.559996719	0.10611575	5.27722529	1.31155E-07	1.00799E-05	PCDHB12	protein_coding
ENSG00000120329.7	5.301282481	0.110976286	0.167365846	0.663076064	0.50728184	0.711209727	SLC25A2	protein_coding
ENSG00000120332.16	199.326071	0.204748966	0.19426122	1.053987856	0.291888477	0.521681513	TNN	protein_coding
ENSG00000120333.4	1507.073407	-0.025181289	0.044391073	-0.567260195	0.570537416	0.756958593	MRPS14	protein_coding
ENSG00000120334.15	706.7255969	0.139082431	0.062407344	2.228622806	0.025839012	0.115497655	CENPL	protein_coding
ENSG00000120337.8	60.04505134	0.091462645	0.171201484	0.534239788	0.593175645	0.773248577	TNFSF18	protein_coding
ENSG00000120341.18	33.17046419	-0.41520169	0.100651164	-4.125155375	3.70485E-05	0.000904967	SEC16B	protein_coding
ENSG00000120370.13	629.3527171	0.117822078	0.050359423	2.339623286	0.019303199	0.094626207	GORAB	protein_coding
ENSG00000120436.4	9.717918686	-0.590838451	0.168286147	-3.510915554	0.000446566	0.00625121	GPR31	protein_coding
ENSG00000120437.9	993.0793264	0.129105571	0.08674382	1.488354686	0.13665738	0.332669608	ACAT2	protein_coding
ENSG00000120438.12	9348.868195	0.046782979	0.060146465	0.777817606	0.436676547	0.654050148	TCP1	protein_coding
ENSG00000120440.15	47.64142575	-0.060048605	0.178031061	-0.337292857	0.735896144	0.863067776	TTLL2	protein_coding
ENSG00000120451.11	5552.918476	0.093404017	0.072508773	1.2881754	0.197684913	0.415077357	SNX19	protein_coding
ENSG00000120457.12	233.5861216	-0.335482718	0.153272618	-2.188797475	0.028611563	0.123546919	KCNJ5	protein_coding
ENSG00000120458.13	727.2195934	-0.096903177	0.058995037	-1.642564899	0.100472988	0.276303888	MSANTD2	protein_coding
ENSG00000120471.15	13.46467175	0.476502641	0.192622993	2.473757855	0.013370028	0.073993614	TP53AIP1	protein_coding
ENSG00000120498.14	39.56297085	-0.097767494	0.143418541	-0.681693544	0.49543275	0.701936201	TEX11	protein_coding
ENSG00000120500.18	23.2345119	-0.005294753	0.092372943	-0.05731931	0.954290838	0.978606228	ARR3	protein_coding
ENSG00000120509.11	2860.709535	-0.105062596	0.060461561	-1.737675866	0.082267944	0.244385316	PDZD11	protein_coding
ENSG00000120519.15	978.0092653	0.09262756	0.059446366	1.558170266	0.119192891	0.306187482	SLC10A7	protein_coding
ENSG00000120526.12	1718.535953	0.09546646	0.06930248	1.377533103	0.168347488	0.377606541	NUDCD1	protein_coding
ENSG00000120533.13	3215.663875	-0.005927125	0.056569733	-0.104775544	0.916553916	0.960402627	ENY2	protein_coding
ENSG00000120539.15	1130.260383	-0.0397858	0.064692045	-0.615002964	0.538552763	0.734446647	MASTL	protein_coding
ENSG00000120549.18	7834.75823	-0.094853126	0.070170375	-1.351754579	0.176453839	0.388605091	KIAA1217	protein_coding
ENSG00000120555.13	14.73266258	-0.091668615	0.111759189	-0.820233361	0.412083087	0.632813359	SEPTIN7P9	transcribed_unprocessed_pseudogene
ENSG00000120563.10	1.707960113	-0.253886454	0.399721776	-0.635157924	0.525325422	0.724349165	LYZL1	protein_coding
ENSG00000120594.17	5016.458565	0.397989232	0.120510122	3.302537804	0.000958142	0.011098602	PLXDC2	protein_coding
ENSG00000120616.16	1683.998942	-0.067939262	0.044360132	-1.53153875	0.125636297	0.315992031	EPC1	protein_coding
ENSG00000120645.12	129.8455458	0.518948239	0.141742118	3.661214095	0.000251023	0.00405442	IQSEC3	protein_coding
ENSG00000120647.10	445.0234927	0.019346637	0.066556641	0.290679289	0.771296613	0.884321105	CCDC77	protein_coding
ENSG00000120656.12	1036.7825	-0.149153546	0.051994727	-2.868628295	0.00412256	0.032412934	TAF12	protein_coding
ENSG00000120658.13	203.111391	0.04867775	0.126588197	0.384536244	0.700581038	0.84251076	ENOX1	protein_coding
ENSG00000120659.15	176.5522639	0.088255154	0.130407923	0.676762212	0.498556837	0.704248939	TNFSF11	protein_coding
ENSG00000120662.16	699.0563759	-0.119304435	0.072485344	-1.645911146	0.099782054	0.275028625	MTRF1	protein_coding
ENSG00000120664.11	9.625378903	0.050535385	0.158799884	0.318233133	0.750308102	0.871828285	SPART-AS1	lncRNA
ENSG00000120669.16	16.75049399	0.488638159	0.29602391	1.650671254	0.098805721	0.273566103	SOHLH2	protein_coding
ENSG00000120675.6	5448.101039	-0.056724985	0.081865642	-0.692903443	0.488370149	0.695844428	DNAJC15	protein_coding
ENSG00000120685.20	6325.546341	0.133254001	0.06800335	1.959521131	0.050051788	0.178177598	PROSER1	protein_coding
ENSG00000120686.12	4419.43919	0.085926063	0.052603993	1.633451348	0.102374105	0.279363541	UFM1	protein_coding
ENSG00000120688.9	1517.408247	-0.118356477	0.065704956	-1.801332586	0.071650475	0.223133905	WBP4	protein_coding
ENSG00000120690.16	6716.080707	0.020596496	0.056749612	0.362936329	0.716652448	0.851968579	ELF1	protein_coding
ENSG00000120693.14	959.4996956	0.3887227	0.160851044	2.41666258	0.015663529	0.082485755	SMAD9	protein_coding

ENSG00000120694.20	10652.58077	0.027032194	0.090471591	0.298792073	0.7650987	0.880468641	HSPH1	protein_coding
ENSG00000120696.9	951.9523614	0.015277589	0.070818466	0.215728885	0.829199084	0.91603733	KBTBD7	protein_coding
ENSG00000120697.9	2265.987409	0.063539239	0.064892716	0.979142853	0.3275094	0.557038255	ALG5	protein_coding
ENSG00000120699.13	1584.020459	-0.167531858	0.066370957	-2.524174196	0.011597045	0.067024541	EXOSC8	protein_coding
ENSG00000120705.13	6710.994042	0.003753058	0.044576589	0.084193476	0.932902606	0.968256984	ETF1	protein_coding
ENSG00000120708.17	14702.70452	0.371088239	0.120736508	3.073537937	0.002115368	0.019909587	TGFBI	protein_coding
ENSG00000120709.11	1851.094859	0.041202186	0.047159075	0.873685196	0.382289715	0.607575576	FAM53C	protein_coding
ENSG00000120725.13	1679.483482	-0.058639407	0.063162161	-0.928394578	0.35320293	0.581580354	SIL1	protein_coding
ENSG00000120727.13	1825.593558	-0.016601371	0.052257126	-0.317686263	0.750722932	0.872071097	PAIP2	protein_coding
ENSG00000120729.10	9.12596904	-0.517130195	0.152778981	-3.384825528	0.000712235	0.008867734	MYOT	protein_coding
ENSG00000120733.14	6776.499331	0.020750755	0.044243342	0.469014193	0.639059491	0.804515252	KDM3B	protein_coding
ENSG00000120738.8	14154.71958	0.245626099	0.127051154	1.93328507	0.05320109	0.18500869	EGR1	protein_coding
ENSG00000120742.11	13565.37749	-0.007945079	0.051036528	-0.15567436	0.876289709	0.940196396	SERP1	protein_coding
ENSG00000120756.13	9602.460374	-0.048216084	0.101350746	-0.475734874	0.634263272	0.801533817	PLS1	protein_coding
ENSG00000120784.16	463.1183146	-0.207526947	0.107920986	-1.922952653	0.054485996	0.187962022	ZFP30	protein_coding
ENSG00000120798.17	1597.874832	-0.144190835	0.053494795	-2.695418027	0.007030037	0.047138865	NR2C1	protein_coding
ENSG00000120800.5	2406.269005	-0.050883498	0.057696214	-0.881920927	0.37781957	0.603660753	UTP20	protein_coding
ENSG00000120802.14	7751.747416	-0.222803049	0.062082778	-3.588806015	0.000332196	0.005015974	TMPO	protein_coding
ENSG00000120805.14	5547.677756	-0.098730133	0.04658658	-2.119282719	0.034066581	0.138322252	ARL1	protein_coding
ENSG00000120820.12	695.1728962	0.430801145	0.110343052	3.904198214	9.45383E-05	0.001894112	GLT8D2	protein_coding
ENSG00000120832.10	339.2562959	-0.35364897	0.061685532	-5.733094277	9.86148E-09	1.18059E-06	MTERF2	protein_coding
ENSG00000120833.14	967.2105322	0.036291792	0.092751114	0.391281464	0.695589198	0.839430883	SOCS2	protein_coding
ENSG00000120837.8	1779.602121	-0.066485567	0.042996259	-1.546310527	0.122029591	0.310678198	NFYB	protein_coding
ENSG00000120860.11	1408.614645	-0.227891075	0.059313226	-3.842162913	0.000121955	0.002304608	WASHC3	protein_coding
ENSG00000120868.14	1931.539207	-0.023154647	0.064955855	-0.356467427	0.721490541	0.8546372	APAF1	protein_coding
ENSG00000120875.9	6937.347077	0.40433313	0.127747685	3.16509163	0.001550341	0.015885074	DUSP4	protein_coding
ENSG00000120885.22	21918.95174	-0.691284312	0.160374237	-4.31044489	1.62926E-05	0.000474272	CLU	protein_coding
ENSG00000120889.13	4652.277858	0.166155028	0.075422623	2.202986618	0.027595692	0.120591402	TNFRSF10B	protein_coding
ENSG00000120896.13	3935.390817	0.124197879	0.080206141	1.54848341	0.121505953	0.309856524	SORBS3	protein_coding
ENSG00000120899.18	3843.450249	-0.28147737	0.071410398	-3.941686029	8.09108E-05	0.001683544	PTK2B	protein_coding
ENSG00000120903.13	3.591424113	-0.129164552	0.230524549	-0.560307147	0.575269953	0.760217436	CHRNA2	protein_coding
ENSG00000120907.18	24.96228307	-0.024593607	0.155180839	-0.158483529	0.874075799	0.939139038	ADRA1A	protein_coding
ENSG00000120910.15	689.1912816	-0.051753157	0.071879387	-0.719999984	0.471525005	0.68269138	PPP3CC	protein_coding
ENSG00000120913.23	1156.113324	-0.006716409	0.087482703	-0.076774138	0.938803225	0.970978286	PDLIM2	protein_coding
ENSG00000120915.14	1839.560011	-0.67178759	0.108832271	-6.172687397	6.71389E-10	1.2776E-07	EPHX2	protein_coding
ENSG00000120925.16	1486.733558	-0.066532319	0.054477427	-1.221282339	0.221979138	0.444498893	RNF170	protein_coding
ENSG00000120937.9	3.434735048	-0.123185724	0.34601153	-0.35601624	0.721828403	0.854967691	NPPB	protein_coding
ENSG00000120942.14	1808.809686	-0.087601394	0.049507989	-1.769439582	0.076820545	0.233934933	UBIAD1	protein_coding
ENSG00000120948.18	3966.564419	-0.021223475	0.037656256	-0.563610874	0.573018985	0.758763265	TARDBP	protein_coding
ENSG00000120949.15	118.1412848	-0.165177664	0.124240721	-1.329496986	0.183684059	0.39761563	TNFRSF8	protein_coding
ENSG00000120952.4	0.709898762	-0.90356798	0.56539647	-1.59811394	0.110017622		PRAMEF2	protein_coding
ENSG00000120963.12	5588.411353	-0.073808581	0.060951556	-1.210938425	0.225919005	0.449043598	ZNF706	protein_coding

ENSG00000120992.18	7090.080005	-0.033400668	0.063267908	-0.527924327	0.597551859	0.776835243	LYPLA1	protein_coding
ENSG00000121005.9	547.4133638	0.321150061	0.143295995	2.241165638	0.025015348	0.113225068	CRISPLD1	protein_coding
ENSG00000121022.14	2258.41419	-0.106825112	0.051125911	-2.089451517	0.036667098	0.145192379	COPS5	protein_coding
ENSG00000121039.10	4045.669558	0.147596203	0.078736264	1.874564459	0.060852667	0.201545078	RDH10	protein_coding
ENSG00000121053.6	8.808367875	-0.066960154	0.127622954	-0.524671717	0.599811422	0.77839928	EPX	protein_coding
ENSG00000121057.13	8015.770408	-0.000399052	0.053719432	-0.00742845	0.994073009	0.997322885	AKAP1	protein_coding
ENSG00000121058.5	1163.434537	-0.06328033	0.044844936	-1.411091966	0.158217499	0.363574662	COIL	protein_coding
ENSG00000121060.18	4040.223634	-0.009422282	0.060640833	-0.155378512	0.876522924	0.940326024	TRIM25	protein_coding
ENSG00000121064.13	3057.910776	-0.206423353	0.095975943	-2.150782236	0.031493392	0.131719698	SCPEP1	protein_coding
ENSG00000121067.19	3589.026102	-0.166230014	0.050875907	-3.267362187	0.001085547	0.012236131	SPOP	protein_coding
ENSG00000121068.14	1326.791445	0.247179125	0.096551604	2.560072685	0.010465027	0.062240275	TBX2	protein_coding
ENSG00000121073.15	3120.220139	-0.073534679	0.060954979	-1.206376896	0.2276722	0.451069401	SLC35B1	protein_coding
ENSG00000121075.11	48.8472314	-0.124504183	0.241266109	-0.516045057	0.60582294	0.78269353	TBX4	protein_coding
ENSG00000121089.4	123.1563923	-0.218118291	0.089100465	-2.448003975	0.014365007	0.077723356	NACA3P	processed_pseudogene
ENSG00000121101.16	34.92402827	-0.052983019	0.131043211	-0.404317163	0.685979506	0.834229033	TEX14	protein_coding
ENSG00000121104.8	1049.486714	-0.334566683	0.095132867	-3.516835896	0.000436724	0.006148732	FAM117A	protein_coding
ENSG00000121152.10	1523.341525	-0.035745906	0.089439572	-0.399665442	0.689402948	0.835873022	NCAPH	protein_coding
ENSG00000121207.12	77.64941303	0.486630184	0.23055349	2.110704048	0.034797758	0.140223959	LRAT	protein_coding
ENSG00000121210.16	1151.49448	-0.364047536	0.085000209	-4.282901673	1.84472E-05	0.000521656	TMEM131L	protein_coding
ENSG00000121211.8	8.078282565	0.004114661	0.119381985	0.034466351	0.972505274	0.987102547	MND1	protein_coding
ENSG00000121236.21	94.99483866	0.258565895	0.122300493	2.114185216	0.034499451	0.139518586	TRIM6	protein_coding
ENSG00000121270.16	33.29878893	-0.315860884	0.130810704	-2.414640964	0.015750727	0.082814844	ABCC11	protein_coding
ENSG00000121274.14	1519.367088	-0.177172781	0.04593779	-3.85679814	0.000114882	0.002199688	TENT4B	protein_coding
ENSG00000121281.13	1071.854284	0.29900363	0.094643308	3.159268584	0.001581657	0.016133859	ADCY7	protein_coding
ENSG00000121289.18	1259.700133	0.092198516	0.050331953	1.831808848	0.066979905	0.213892958	CEP89	protein_coding
ENSG00000121297.8	910.3530839	0.348028218	0.123468811	2.8187541	0.004821044	0.03628439	TSHZ3	protein_coding
ENSG00000121310.17	2155.321692	-0.054866815	0.084472407	-0.649523517	0.516000051	0.717408488	ECHDC2	protein_coding
ENSG00000121314.3	0.764515537	-0.401803353	0.388506874	-1.034224565	0.301031193		TAS2R8	protein_coding
ENSG00000121316.11	6024.241721	-0.109136383	0.109860539	-0.993408406	0.320510967	0.550203128	PLBD1	protein_coding
ENSG00000121318.2	6.098589189	0.073108235	0.166980271	0.437825589	0.66151272	0.818669971	TAS2R10	protein_coding
ENSG00000121335.12	1.854242407	0.535090494	0.27248733	1.963726148	0.049561859	0.176898772	PRB2	protein_coding
ENSG00000121350.16	829.670996	-0.025566336	0.083061857	-0.307798746	0.758235475	0.876273035	PYROXD1	protein_coding
ENSG00000121351.8	2.809473772	0.027687116	0.271550332	0.101959426	0.918788881	0.961667461	IAPP	protein_coding
ENSG00000121361.5	471.9602488	0.120972984	0.113262925	1.068072218	0.285487937	0.514782168	KCNJ8	protein_coding
ENSG00000121377.2	0.214147125	-0.063720747	0.670427444	-0.095044956	0.924279119		TAS2R7	protein_coding
ENSG00000121380.12	1057.669206	-0.381533991	0.11729272	-3.252836077	0.001142594	0.012711467	BCL2L14	protein_coding
ENSG00000121381.4	0.824253328	0.158897347	0.341962547	0.46466301	0.642172802		TAS2R9	protein_coding
ENSG00000121388.6	3.235872191	-0.466565422	0.331644039	-1.406825895	0.159479022	0.365307893	AL139327.1	transcribed_unprocessed_pseudogene
ENSG00000121390.19	2273.57746	-0.059920185	0.047289287	-1.267098509	0.205120068	0.424677857	PSPC1	protein_coding
ENSG00000121406.9	277.1477864	0.091680304	0.11805157	0.776612322	0.43738753	0.654380242	ZNF549	protein_coding
ENSG00000121410.12	10.11020948	-0.255439693	0.137407073	-1.858999589	0.063027197	0.206103568	A1BG	protein_coding
ENSG00000121413.13	565.3777261	-0.275239285	0.13856434	-1.986364486	0.046992867	0.170921306	ZSCAN18	protein_coding

ENSG00000121417.14	605.4854139	0.060276888	0.078390212	0.768933856	0.441932578	0.658340792	ZNF211	protein_coding
ENSG00000121440.15	1846.189141	-0.116643958	0.148814461	-0.783821393	0.433144919	0.651139426	PDZRN3	protein_coding
ENSG00000121446.20	8.966298869	0.108510458	0.24262616	0.447233135	0.654706739	0.814449203	RGSL1	protein_coding
ENSG00000121454.6	103.1259458	0.003623157	0.085063478	0.042593566	0.966025525	0.98448128	LHX4	protein_coding
ENSG00000121481.11	1486.581237	0.189655072	0.05722516	3.314190317	0.000919089	0.010766439	RNF2	protein_coding
ENSG00000121486.12	1464.359885	-0.032679937	0.04573459	-0.714556239	0.474883294	0.685276816	TRMT1L	protein_coding
ENSG00000121542.12	607.4174736	0.020301232	0.041112465	0.493797483	0.621449211	0.792978102	SEC22A	protein_coding
ENSG00000121552.4	383.7433707	-0.077643211	0.159249588	-0.487556747	0.625863839	0.795974657	CSTA	protein_coding
ENSG00000121570.12	19.48687636	-0.309898239	0.195724622	-1.583338039	0.113344437	0.296916774	DPPA4	protein_coding
ENSG00000121577.14	261.4959309	-0.427724676	0.168057538	-2.545108551	0.010924377	0.06424534	POPDC2	protein_coding
ENSG00000121578.13	2294.596832	0.115033082	0.077859931	1.477436232	0.13955874	0.336810345	B4GALT4	protein_coding
ENSG00000121579.13	8907.514354	-0.006564458	0.054021874	-0.121514806	0.903283288	0.954054385	NAA50	protein_coding
ENSG00000121594.12	109.6368756	-0.264563177	0.12206493	-2.167397116	0.030204587	0.128162151	CD80	protein_coding
ENSG00000121621.7	839.687276	0.050897932	0.0934966	0.544382705	0.586178145	0.768491381	KIF18A	protein_coding
ENSG00000121634.6	1.535418384	0.016089547	0.323589594	0.049722078	0.960343863	0.981539181	GJA8	protein_coding
ENSG00000121644.19	3670.282689	0.138711016	0.04692542	2.955988775	0.003116684	0.026443877	DESI2	protein_coding
ENSG00000121653.11	612.0608352	0.293325753	0.146484322	2.002437858	0.04523766	0.16681547	MAPK8IP1	protein_coding
ENSG00000121671.12	1861.499838	-0.028984126	0.066102186	-0.438474545	0.661042319	0.818428876	CRY2	protein_coding
ENSG00000121680.16	1491.037098	-0.070836455	0.061237691	-1.156746017	0.247376143	0.473967208	PEX16	protein_coding
ENSG00000121690.11	272.0203081	-0.320996497	0.156725803	-2.0481407	0.040546215	0.155308249	DEPDC7	protein_coding
ENSG00000121691.7	6127.467148	-0.151138613	0.070513561	-2.143397815	0.032081168	0.133206391	CAT	protein_coding
ENSG00000121716.20	304.7112604	0.177320147	0.116151099	1.526633401	0.126852197	0.317860946	PILRB	protein_coding
ENSG00000121741.16	5089.332515	0.160067203	0.063577915	2.517654155	0.011813926	0.067827781	ZMYM2	protein_coding
ENSG00000121742.19	118.1649069	1.193540835	0.258746884	4.612773756	3.97331E-06	0.000155566	GJB6	protein_coding
ENSG00000121743.4	133.2421671	0.317766083	0.210953566	1.506331883	0.131981997	0.3256358	GJA3	protein_coding
ENSG00000121749.15	2750.979594	-0.074744242	0.047884607	-1.560924205	0.118541635	0.304976316	TBC1D15	protein_coding
ENSG00000121753.12	430.9334797	0.456854957	0.139097387	3.28442516	0.001021906	0.011678891	ADGRB2	protein_coding
ENSG00000121764.11	9.395418133	0.090935689	0.143553713	0.633461072	0.526432594	0.725167735	HCRTR1	protein_coding
ENSG00000121766.16	1208.170129	-0.079406196	0.044716984	-1.775750245	0.075774092	0.231736094	ZCCHC17	protein_coding
ENSG00000121769.8	527.6224156	-0.078459377	0.135030589	-0.581048918	0.561207485	0.750475299	FABP3	protein_coding
ENSG00000121774.18	9463.177201	-0.081254402	0.033330542	-2.43783623	0.014775466	0.07927487	KHDRBS1	protein_coding
ENSG00000121775.18	1005.022804	-0.020139612	0.050914795	-0.395555191	0.692433199	0.837682505	TMEM39B	protein_coding
ENSG00000121797.10	658.7027091	-0.140723912	0.098308418	-1.431453325	0.152300335	0.355326678	CCRL2	protein_coding
ENSG00000121807.6	185.3756962	-0.404282366	0.14894317	-2.714339744	0.006640802	0.04536249	CCR2	protein_coding
ENSG00000121851.13	695.6821708	-0.164494545	0.060219563	-2.731579863	0.006303146	0.043772463	POLR3GL	protein_coding
ENSG00000121853.4	3.028998981	-0.419125861	0.23746408	-1.765007416	0.077562519	0.235367593	GHSR	protein_coding
ENSG00000121858.11	5421.556918	-0.088637262	0.097901127	-0.905375299	0.365266597	0.59250473	TNFSF10	protein_coding
ENSG00000121864.10	2128.529813	0.072590058	0.052864846	1.37312532	0.169713391	0.379388887	ZNF639	protein_coding
ENSG00000121871.4	31.69186929	0.322448481	0.255479077	1.262132637	0.206901063	0.426778738	SLITRK3	protein_coding
ENSG00000121879.6	2002.243073	0.156626789	0.056367952	2.778649634	0.005458537	0.03954878	PIK3CA	protein_coding
ENSG00000121892.15	9175.572714	-0.008557218	0.047826018	-0.178923897	0.857997459	0.930744947	PDS5A	protein_coding
ENSG00000121895.8	148.0352364	-0.896886009	0.130677759	-6.863340911	6.72684E-12	2.93188E-09	TMEM156	protein_coding

ENSG00000121897.15	590.7645342	-0.260602548	0.055601734	-4.68695005	2.77307E-06	0.000117095	LIAS	protein_coding
ENSG00000121898.13	2057.769414	-0.185830418	0.179346154	-1.036155026	0.300129825	0.529740264	CPXM2	protein_coding
ENSG00000121900.19	4005.559951	-0.140319402	0.099312204	-1.412911973	0.157681608	0.362837096	TMEM54	protein_coding
ENSG00000121903.14	237.6944872	0.067889963	0.061444685	1.104895615	0.269204823	0.497250702	ZSCAN20	protein_coding
ENSG00000121904.17	266.0520415	0.401290941	0.132253203	3.034262552	0.002411245	0.021854163	CSMD2	protein_coding
ENSG00000121905.10	30.47312011	0.290465981	0.125919077	2.306767075	0.021067809	0.100621646	HPCA	protein_coding
ENSG00000121931.16	697.6677041	-0.128926408	0.097102866	-1.327730203	0.184267252	0.398383312	LRIF1	protein_coding
ENSG00000121933.19	77.55880302	0.097647026	0.124048679	0.787166999	0.431184113	0.64918005	TMIGD3	protein_coding
ENSG00000121940.16	1622.091651	-0.0639622	0.038417816	-1.664909849	0.095930743	0.268302386	CLCC1	protein_coding
ENSG00000121957.15	1889.945322	-0.004664897	0.067426408	-0.069185009	0.944842356	0.97410169	GPSM2	protein_coding
ENSG00000121964.14	955.2099219	0.0599136	0.044383106	1.349919046	0.177041951	0.389231478	GTDC1	protein_coding
ENSG00000121966.7	5039.004509	-0.162494947	0.119522142	-1.359538443	0.173976029	0.385057961	CXCR4	protein_coding
ENSG00000121988.18	376.4690288	-0.088697139	0.06260315	-1.416815918	0.156536755	0.361402921	ZRANB3	protein_coding
ENSG00000121989.15	835.494532	0.04024707	0.059615297	0.675113141	0.499603885	0.704981494	ACVR2A	protein_coding
ENSG00000122008.15	1041.072943	0.043077757	0.059691987	0.721667329	0.470499033	0.681761147	POLK	protein_coding
ENSG00000122012.14	55.58816458	-0.62728847	0.184067192	-3.407931975	0.000654572	0.008311273	SV2C	protein_coding
ENSG00000122025.15	63.94546492	-0.556029049	0.149120715	-3.728717686	0.000192457	0.003296652	FLT3	protein_coding
ENSG00000122026.10	31345.19493	-0.168949162	0.064540372	-2.61772836	0.008851724	0.055413241	RPL21	protein_coding
ENSG00000122033.14	1917.696634	-0.146157965	0.050037759	-2.920953446	0.00348962	0.028767907	MTIF3	protein_coding
ENSG00000122034.17	6628.822972	-0.143674275	0.068128733	-2.108864616	0.034956269	0.140641616	GTF3A	protein_coding
ENSG00000122035.7	759.6558126	-0.253663531	0.142648353	-1.778243678	0.075363842	0.230935247	RASL11A	protein_coding
ENSG00000122042.10	4428.219736	-0.021126217	0.064149861	-0.329326006	0.74190929	0.866756782	UBL3	protein_coding
ENSG00000122043.11	2.554744903	-0.685038364	0.245937674	-2.785414499	0.005345934	0.038950453	LINC00544	lncRNA
ENSG00000122068.13	4186.244071	0.139667891	0.051675259	2.702800029	0.00687581	0.046418107	FYTTD1	protein_coding
ENSG00000122085.17	997.9577805	-0.120613131	0.040203732	-3.000048105	0.00269937	0.023711033	MTERF4	protein_coding
ENSG00000122121.11	827.6000302	-0.267815548	0.230939486	-1.159678465	0.246179741	0.472664511	XPNPEP2	protein_coding
ENSG00000122122.10	1198.634959	-0.556869442	0.13234555	-4.207692978	2.57991E-05	0.000676036	SASH3	protein_coding
ENSG00000122126.17	3226.526887	0.034498667	0.057726217	0.597625641	0.550089752	0.742874375	OCRL	protein_coding
ENSG00000122133.17	79.34332397	1.535925974	0.25243149	6.084526032	1.16836E-09	2.03833E-07	PAEP	protein_coding
ENSG00000122136.14	3.815919019	-0.012661663	0.242818351	-0.052144587	0.958413486	0.980686155	OBP2A	protein_coding
ENSG00000122140.11	1898.093366	-0.05973128	0.070286001	-0.849831821	0.395418595	0.618757402	MRPS2	protein_coding
ENSG00000122145.15	2.254119153	-0.303659427	0.337155695	-0.900650445	0.367774204	0.594895936	TBX22	protein_coding
ENSG00000122176.12	7726.566285	0.024869966	0.133390605	0.18644466	0.852096073	0.927714942	FMOD	protein_coding
ENSG00000122180.5	3.500180533	-0.356340131	0.269074104	-1.324319682	0.185396897	0.399893113	MYOG	protein_coding
ENSG00000122188.13	353.5812467	-0.749584349	0.138800845	-5.40043072	6.64811E-08	5.92443E-06	LAX1	protein_coding
ENSG00000122194.19	6.625043606	0.037274367	0.22259214	0.1674559	0.867011344	0.935539268	PLG	protein_coding
ENSG00000122203.15	5003.746017	0.039972443	0.043202086	0.925243341	0.354839343	0.582887292	KIAA1191	protein_coding
ENSG00000122218.16	19376.16438	0.219313661	0.046773316	4.688862818	2.74727E-06	0.000116543	COPA	protein_coding
ENSG00000122223.13	93.19806552	-0.590324743	0.127728929	-4.621699634	3.80609E-06	0.000150098	CD244	protein_coding
ENSG00000122224.18	302.2972835	-0.75966293	0.152526594	-4.980527721	6.34111E-07	3.6074E-05	LY9	protein_coding
ENSG00000122254.7	203.5490395	0.242418554	0.170437698	1.422329431	0.154930639	0.359232332	HS3ST2	protein_coding
ENSG00000122257.20	4593.604021	-0.07432249	0.040257969	-1.84615599	0.064869559	0.20968925	RBBP6	protein_coding

ENSG00000122299.12	2628.46011	-0.008494012	0.042075897	-0.201873576	0.84001556	0.921538645	ZC3H7A	protein_coding
ENSG00000122304.11	0.956689251	-0.182153587	0.372906	-0.488470517	0.625216604		PRM2	protein_coding
ENSG00000122335.17	913.705788	0.057814447	0.061967037	0.932987119	0.350826619	0.579836592	SERAC1	protein_coding
ENSG00000122359.18	25161.04096	0.15395075	0.066856779	2.302694693	0.021296027	0.101355859	ANXA11	protein_coding
ENSG00000122367.20	510.0812181	-0.747726598	0.210343202	-3.554793265	0.000378276	0.005518631	LDB3	protein_coding
ENSG00000122375.12	5.487365343	-0.047052007	0.190337982	-0.247202407	0.804751583	0.902952021	OPN4	protein_coding
ENSG00000122376.12	1740.834925	0.069382478	0.054637449	1.269870377	0.204130807	0.423490034	SHLD2	protein_coding
ENSG00000122378.14	3289.953455	0.023415407	0.100202451	0.233680979	0.815232656	0.908791466	PRXL2A	protein_coding
ENSG00000122386.11	667.533159	0.01742242	0.068600375	0.253969754	0.799518925	0.900042268	ZNF205	protein_coding
ENSG00000122390.19	4269.694724	-0.062722912	0.05016611	-1.250304483	0.211188341	0.43162576	NAA60	protein_coding
ENSG00000122406.14	41593.45064	-0.166385664	0.05810315	-2.863625547	0.004188228	0.032746409	RPL5	protein_coding
ENSG00000122417.15	1117.569403	0.273840546	0.070602106	3.878645588	0.00010504	0.00205907	ODF2L	protein_coding
ENSG00000122420.10	238.8368794	0.08704177	0.15373067	0.566196518	0.571260196	0.757476823	PTGFR	protein_coding
ENSG00000122432.18	37.72436726	-0.237391105	0.087779099	-2.704414927	0.006842479	0.04630761	SPATA1	protein_coding
ENSG00000122435.10	692.5559921	-0.140711426	0.048869988	-2.879301392	0.003985572	0.031681738	TRMT13	protein_coding
ENSG00000122477.13	40.34862587	-0.14100174	0.096681309	-1.458417773	0.144725425	0.344628406	LRRC39	protein_coding
ENSG00000122481.17	103.0244424	-0.143132134	0.058173318	-2.460443017	0.013876561	0.075967196	RWDD3	protein_coding
ENSG00000122482.21	2938.829473	0.032574889	0.041154981	0.791517527	0.428642052	0.646844711	ZNF644	protein_coding
ENSG00000122483.17	395.4580404	-0.214954603	0.070580471	-3.045525221	0.002322743	0.02126879	CCDC18	protein_coding
ENSG00000122484.9	1509.465931	0.024240136	0.04864037	0.498354272	0.618234361	0.790890851	RPAP2	protein_coding
ENSG00000122490.19	2292.285626	-0.01291889	0.064205293	-0.201212231	0.840532628	0.921711426	SLC66A2	protein_coding
ENSG00000122507.21	675.4616549	0.086621884	0.053450764	1.620592061	0.105105156	0.283794931	BBS9	protein_coding
ENSG00000122512.16	827.0542709	-0.077006777	0.046122593	-1.669610759	0.0949964	0.266581749	PMS2	protein_coding
ENSG00000122515.15	9823.159155	-0.000950127	0.054607723	-0.017399131	0.986118203	0.993549548	ZMIZ2	protein_coding
ENSG00000122543.11	7.714836075	-0.44064295	0.1705947	-2.582981482	0.009795058	0.059597772	OCM	protein_coding
ENSG00000122545.20	8732.989547	-0.054183672	0.040173247	-1.348750127	0.177417239	0.389690284	SEPTIN7	protein_coding
ENSG00000122547.11	1662.645289	-0.130098562	0.095301815	-1.365121563	0.172214846	0.382790428	EEPD1	protein_coding
ENSG00000122548.5	4.514242082	-0.099851251	0.241014679	-0.41429531	0.678657848	0.829701297	KIAA0087	lncRNA
ENSG00000122550.18	1454.25752	-0.079992908	0.051543518	-1.551948936	0.120674442	0.308551019	KLHL7	protein_coding
ENSG00000122557.11	2283.077019	-0.029509963	0.037833998	-0.779985309	0.435399523	0.65303934	HERPUD2	protein_coding
ENSG00000122565.19	9914.274057	-0.108692707	0.056382864	-1.927761359	0.053884816	0.186621387	CBX3	protein_coding
ENSG00000122566.22	40865.47645	-0.055523605	0.043065999	-1.289267793	0.197305004	0.414616818	HNRNPA2B1	protein_coding
ENSG00000122574.11	722.7494072	0.215834824	0.155665271	1.386531645	0.165584624	0.373598487	WIPF3	protein_coding
ENSG00000122584.13	8.332612418	0.248068539	0.272244638	0.911197155	0.362191503	0.589731649	NXPH1	protein_coding
ENSG00000122585.8	32.87186342	-0.188157625	0.271176738	-0.693856066	0.487772478	0.695311131	NPY	protein_coding
ENSG00000122591.12	1378.5432	0.025898904	0.093665208	0.276505064	0.78216017	0.889807228	FAM126A	protein_coding
ENSG00000122592.8	70.53528163	0.634404807	0.181529309	3.49477894	0.000474455	0.006542818	HOXA7	protein_coding
ENSG00000122641.11	4309.244554	0.609900985	0.127969412	4.765990367	1.87928E-06	8.54838E-05	INHBA	protein_coding
ENSG00000122642.11	7274.502295	0.270187564	0.057515675	4.697633553	2.63193E-06	0.000112113	FKBP9	protein_coding
ENSG00000122643.22	2992.237872	-0.201415983	0.070668528	-2.850151083	0.004369847	0.033783564	NT5C3A	protein_coding
ENSG00000122644.13	1361.594255	-0.06878287	0.073287832	-0.938530558	0.347971821	0.577255722	ARL4A	protein_coding
ENSG00000122674.12	446.1026518	0.061772737	0.06282442	0.983259962	0.32547951	0.555145822	CCZ1	protein_coding

ENSG00000122678.17	1665.094948	0.009950755	0.057694838	0.172472193	0.86306632	0.933239609	POLM	protein_coding
ENSG00000122679.8	728.9694071	-0.155478527	0.100454029	-1.547757997	0.121680573	0.310086217	RAMP3	protein_coding
ENSG00000122687.19	11.81291444	0.023882887	0.096553658	0.247353514	0.804634647	0.902924708	MRM2	protein_coding
ENSG00000122691.13	267.2696596	0.506479294	0.145566257	3.479372923	0.000502589	0.006814345	TWIST1	protein_coding
ENSG00000122692.9	3643.69751	0.002115239	0.046780354	0.045216389	0.963934831	0.983433338	SMU1	protein_coding
ENSG00000122694.16	1171.202613	-0.145499193	0.101002842	-1.440545533	0.149713117	0.351795552	GLIPR2	protein_coding
ENSG00000122696.14	590.9669718	0.017259134	0.053003247	0.325624081	0.74470879	0.868624887	SLC25A51	protein_coding
ENSG00000122705.17	9532.642738	-0.010314478	0.065827031	-0.156690616	0.875488683	0.939862002	CLTA	protein_coding
ENSG00000122707.12	674.9164422	0.141241658	0.107967968	1.308181122	0.190811884	0.406621813	RECK	protein_coding
ENSG00000122711.9	1544.173911	1.885643411	0.278528432	6.770021279	1.28763E-11	4.92204E-09	SPINK4	protein_coding
ENSG00000122728.6	11.24005092	0.018164162	0.118608772	0.153143496	0.878285116	0.941316258	TAF1L	protein_coding
ENSG00000122729.19	4201.015022	-0.02064674	0.070115771	-0.294466416	0.768401523	0.882593801	ACO1	protein_coding
ENSG00000122733.12	42.83106313	-0.35641657	0.173753962	-2.051271609	0.040240504	0.154537634	PHF24	protein_coding
ENSG00000122735.16	11.52207598	0.216120371	0.20319688	1.063600833	0.287509565	0.517184904	DNAI1	protein_coding
ENSG00000122741.16	3029.095808	0.038165727	0.049349407	0.773377613	0.439298935	0.655840546	DCAF10	protein_coding
ENSG00000122756.15	313.2118332	0.74024966	0.248368397	2.980450294	0.002878249	0.024871203	CNTFR	protein_coding
ENSG00000122778.10	1506.135854	0.459917758	0.10551073	4.358966682	1.30678E-05	0.00040018	KIAA1549	protein_coding
ENSG00000122779.18	4370.092941	-0.424582375	0.080474538	-5.275984002	1.32046E-07	1.01127E-05	TRIM24	protein_coding
ENSG00000122783.17	819.8875174	-0.01709499	0.053310753	-0.320666823	0.748462892	0.87070577	CYREN	protein_coding
ENSG00000122786.20	41189.41036	-0.223528511	0.149103574	-1.49914925	0.133834917	0.328570377	CALD1	protein_coding
ENSG00000122787.15	10.5160831	-0.688462123	0.224865142	-3.061666724	0.002201084	0.02048089	AKR1D1	protein_coding
ENSG00000122824.11	31.94620865	0.359133384	0.183354373	1.958684582	0.050149737	0.178427098	NUDT10	protein_coding
ENSG00000122852.15	4.194374776	-0.51412183	0.280284269	-1.834287141	0.066611382	0.213059615	SFTPA1	protein_coding
ENSG00000122859.5	83.21365425	-0.056850704	0.284123867	-0.200091264	0.841409205	0.922023008	NEUROG3	protein_coding
ENSG00000122861.16	3675.227876	0.786325092	0.117261597	6.705734133	2.00396E-11	6.98614E-09	PLAU	protein_coding
ENSG00000122862.5	7336.159895	0.01581449	0.117334551	0.134781185	0.892784882	0.948844414	SRGN	protein_coding
ENSG00000122863.6	1525.734585	0.262413115	0.126797365	2.06954707	0.038494779	0.150218231	CHST3	protein_coding
ENSG00000122870.12	1235.450893	0.490688074	0.124515514	3.94077862	8.12176E-05	0.001687753	BICC1	protein_coding
ENSG00000122872.8	1.781018388	-0.191636747	0.185340057	-1.033973716	0.301148451	0.530764249	ARL4AP1	processed_pseudogene
ENSG00000122873.12	1684.259	-0.142088867	0.062019671	-2.291029023	0.021961735	0.103428783	CISD1	protein_coding
ENSG00000122877.16	739.8068417	0.303463975	0.1297829	2.338243137	0.019374639	0.094863892	EGR2	protein_coding
ENSG00000122882.11	2444.846787	-0.067963825	0.046698904	-1.455362311	0.145568985	0.345918837	ECD	protein_coding
ENSG00000122884.12	2964.967613	0.268919003	0.085347582	3.150868431	0.001627858	0.016489347	P4HA1	protein_coding
ENSG00000122912.15	1264.401312	-0.14122151	0.060076492	-2.350695021	0.018738385	0.092856133	SLC25A16	protein_coding
ENSG00000122952.17	2583.822567	-0.11047047	0.084801832	-1.302689667	0.192680731	0.408945173	ZWINT	protein_coding
ENSG00000122958.15	4594.829587	-0.010531211	0.045575221	-0.231073181	0.81725795	0.909943735	VPS26A	protein_coding
ENSG00000122965.11	1753.880325	-0.066240225	0.043746468	-1.514184529	0.129979045	0.322667017	RBM19	protein_coding
ENSG00000122966.17	2114.768397	0.060308959	0.075672848	0.796969591	0.425468692	0.644279198	CIT	protein_coding
ENSG00000122970.16	670.2960906	0.053898836	0.059640442	0.903729643	0.366138777	0.593307655	IFT81	protein_coding
ENSG00000122971.9	2191.457322	-0.380239903	0.089851655	-4.231863116	2.31763E-05	0.000625359	ACADS	protein_coding
ENSG00000122986.14	334.6075154	-0.333540029	0.115798247	-2.880354729	0.00397228	0.031604899	HVCN1	protein_coding
ENSG00000123009.4	146.5901072	-0.209925115	0.11039762	-1.901536595	0.057231764	0.194108297	NME2P1	processed_pseudogene

ENSG00000123064.13	4525.785875	-0.038457365	0.060376208	-0.63696225	0.524149433	0.723384318	DDX54	protein_coding
ENSG00000123066.9	5841.181082	0.013626461	0.05202821	0.261905236	0.793394503	0.896411041	MED13L	protein_coding
ENSG00000123080.12	928.8199817	0.10690671	0.084502365	1.265132765	0.205823739	0.425581522	CDKN2C	protein_coding
ENSG00000123091.5	5049.686242	-0.026580386	0.047774699	-0.556369512	0.577958276	0.762358646	RNF11	protein_coding
ENSG00000123094.15	1136.474296	0.28959877	0.136614712	2.119821255	0.034021122	0.13824036	RASSF8	protein_coding
ENSG00000123095.6	2547.173489	0.172994976	0.133950238	1.291486888	0.196534899	0.413759181	BHLHE41	protein_coding
ENSG00000123096.12	1494.758722	0.101515677	0.12636091	0.803378804	0.421755822	0.641183397	SSPN	protein_coding
ENSG00000123104.12	2763.322254	0.065294343	0.097266546	0.671292912	0.502033949	0.707082528	ITPR2	protein_coding
ENSG00000123106.10	1425.752344	0.049987311	0.076014276	0.657604255	0.510792459	0.713903685	CCDC91	protein_coding
ENSG00000123119.12	167.5342447	-0.54930915	0.175922609	-3.122447729	0.00179354	0.017686597	NECAB1	protein_coding
ENSG00000123124.14	4761.756418	-0.141108848	0.061156362	-2.307345345	0.021035576	0.100568193	WWP1	protein_coding
ENSG00000123130.17	2215.500356	-0.070832645	0.057298532	-1.236203476	0.216382939	0.437596034	ACOT9	protein_coding
ENSG00000123131.13	5457.201262	-0.071131767	0.073206542	-0.971658615	0.331220412	0.560728095	PRDX4	protein_coding
ENSG00000123136.15	4591.523397	-0.112396697	0.070169996	-1.601777163	0.109204895	0.289876687	DDX39A	protein_coding
ENSG00000123143.13	6668.796102	0.133592786	0.074438862	1.794664527	0.072707166	0.225361701	PKN1	protein_coding
ENSG00000123144.11	7202.668817	-0.078131239	0.06401011	-1.220607788	0.222234556	0.444805694	TRIR	protein_coding
ENSG00000123146.20	6746.647337	0.091302033	0.069619609	1.311441337	0.189708701	0.405182378	ADGRE5	protein_coding
ENSG00000123154.12	121.6121457	0.021509003	0.057482242	0.374185179	0.70826655	0.847157517	WDR83	protein_coding
ENSG00000123159.16	8152.924492	0.090742769	0.075596381	1.20035864	0.230000085	0.453721761	GIPC1	protein_coding
ENSG00000123165.8	0.415829262	-0.053747945	0.576079872	-0.093299466	0.925665656		ACTRT1	protein_coding
ENSG00000123171.7	2.131474431	0.494378096	0.234963326	2.104064942	0.035372783	0.141728553	CCDC70	protein_coding
ENSG00000123178.15	1063.12051	-0.131234359	0.055631952	-2.358974561	0.018325512	0.091568723	SPRYD7	protein_coding
ENSG00000123179.14	1955.85827	-0.203926146	0.075603256	-2.697319619	0.006990014	0.04702128	EBPL	protein_coding
ENSG00000123191.16	2773.573601	0.035886676	0.126014449	0.284782232	0.775810985	0.886356674	ATP7B	protein_coding
ENSG00000123200.17	9129.559175	0.048739287	0.064487579	0.755793409	0.449773069	0.664623975	ZC3H13	protein_coding
ENSG00000123201.14	74.54996467	0.158916432	0.164851709	0.963996266	0.335047798	0.564571281	GUCY1B2	transcribed_unitary_pseudogene
ENSG00000123213.23	2150.663691	0.085497148	0.064657602	1.322306205	0.186066207	0.400860588	NLN	protein_coding
ENSG00000123219.13	660.9027011	-0.110517878	0.082397696	-1.341273885	0.179831545	0.392832485	CENPK	protein_coding
ENSG00000123240.17	5588.699238	0.01801293	0.062072711	0.290190802	0.771670273	0.884525122	OPTN	protein_coding
ENSG00000123243.15	1969.386023	-0.110070137	0.144096043	-0.763866476	0.444946817	0.660898747	ITIH5	protein_coding
ENSG00000123268.9	1670.58843	-0.004712616	0.041448555	-0.113697946	0.90947724	0.957162964	ATF1	protein_coding
ENSG00000123297.20	33.60546898	-0.093124584	0.072488696	-1.284677322	0.198905066	0.416896064	TSFM	protein_coding
ENSG00000123307.4	1.256061409	0.687068476	0.52988513	1.296636642	0.194756238	0.411645371	NEUROD4	protein_coding
ENSG00000123329.20	684.2422796	-0.452174993	0.11308247	-3.998630313	6.37101E-05	0.001393722	ARHGAP9	protein_coding
ENSG00000123338.13	2118.980415	-0.482671428	0.119733653	-4.031209421	5.54906E-05	0.001245166	NCKAP1L	protein_coding
ENSG00000123342.16	932.9546712	0.477413955	0.108875706	4.384944738	1.16015E-05	0.000365817	MMP19	protein_coding
ENSG00000123349.15	75.61057418	-0.073587601	0.072435754	-1.015901648	0.309676224	0.539100384	PFDN5	protein_coding
ENSG00000123352.18	1689.753577	-0.119881472	0.049480536	-2.422800589	0.015401378	0.081528895	SPATS2	protein_coding
ENSG00000123353.10	775.956762	-0.042057927	0.070405092	-0.597370537	0.55026002	0.742906376	ORMDL2	protein_coding
ENSG00000123358.20	7302.663109	0.37064934	0.134437596	2.757036356	0.005832787	0.041491708	NR4A1	protein_coding
ENSG00000123360.12	270.3112066	0.296317073	0.13251531	2.23609689	0.025345425	0.114067108	PDE1B	protein_coding
ENSG00000123364.5	161.5705085	0.493022355	0.292156084	1.68753068	0.091501356	0.260747749	HOXC13	protein_coding

ENSG00000123374.11	1460.78739	-0.005979024	0.060679613	-0.098534308	0.921508031	0.962963849	CDK2	protein_coding
ENSG00000123384.14	30725.85492	0.33639705	0.092801237	3.624919885	0.000289051	0.004523773	LRP1	protein_coding
ENSG00000123388.4	467.22166	0.128910946	0.178138505	0.72365571	0.469277131	0.680671774	HOXC11	protein_coding
ENSG00000123395.14	845.5525673	0.11083639	0.06614929	1.675549195	0.093826527	0.264742203	ATG101	protein_coding
ENSG00000123405.14	102.0172142	-0.282117327	0.191560961	-1.472728714	0.140824201	0.338656201	NFE2	protein_coding
ENSG00000123407.4	388.7469802	0.660895745	0.376444874	1.755624239	0.079152591	0.238580618	HOXC12	protein_coding
ENSG00000123411.15	910.584224	0.01939143	0.061226205	0.316717813	0.751457731	0.872365666	IKZF4	protein_coding
ENSG00000123415.15	1509.775632	-0.056468721	0.05738316	-0.984064333	0.325083883	0.554840214	SMUG1	protein_coding
ENSG00000123416.15	14960.02908	0.017459317	0.073879515	0.236321484	0.813183218	0.907775348	TUBA1B	protein_coding
ENSG00000123427.17	617.6832086	0.034415642	0.060871545	0.565381447	0.571814341	0.757880323	EEF1AKMT3	protein_coding
ENSG00000123444.14	314.2419601	0.059609423	0.050912643	1.170817691	0.241672065	0.467498716	KBTBD4	protein_coding
ENSG00000123447.6	0.23727089	-0.140658937	0.694361396	-0.202573095	0.839468721		TYRL	unprocessed_pseudogene
ENSG00000123453.18	224.1530078	-0.232431836	0.118250521	-1.965588252	0.049346192	0.176461433	SARDH	protein_coding
ENSG00000123454.12	32.69687715	-0.338627911	0.147623394	-2.293863473	0.021798342	0.102926249	DBH	protein_coding
ENSG00000123472.12	2792.009178	-0.117574565	0.04363901	-2.694253716	0.007054644	0.047253134	ATPAF1	protein_coding
ENSG00000123473.16	2009.45085	0.003724546	0.088316627	0.042172652	0.966361063	0.984499865	STIL	protein_coding
ENSG00000123485.12	1301.078175	0.025725006	0.093722505	0.274480566	0.783715337	0.89030144	HJURP	protein_coding
ENSG00000123496.8	92.88830741	0.246167462	0.192727847	1.277280194	0.201503343	0.420380658	IL13RA2	protein_coding
ENSG00000123500.10	2938.425089	1.123436995	0.192363558	5.840175789	5.21458E-09	7.07899E-07	COL10A1	protein_coding
ENSG00000123505.19	6964.681055	-0.066877126	0.055094157	-1.213869666	0.224797498	0.447697404	AMD1	protein_coding
ENSG00000123545.6	1312.554129	-0.137688654	0.067115208	-2.051526893	0.040215663	0.154475616	NDUFAF4	protein_coding
ENSG00000123552.18	863.1094642	-0.054150392	0.059378223	-0.911957095	0.361791304	0.589497945	USP45	protein_coding
ENSG00000123560.14	126.2667003	-0.521966864	0.251190286	-2.077973928	0.037711761	0.148125033	PLP1	protein_coding
ENSG00000123561.15	9.516304936	-2.012540902	0.526121451	-3.825240154	0.000130645	0.002428798	SERPINA7	protein_coding
ENSG00000123562.18	11561.60907	0.177760049	0.048691488	3.650741797	0.000261484	0.004184608	MORF4L2	protein_coding
ENSG00000123569.8	3.087574935	0.733579314	0.432501631	1.696130746	0.089861122	0.257976215	H2BW1	protein_coding
ENSG00000123570.4	151.318457	-0.356274904	0.157217795	-2.266123273	0.023443837	0.108187236	RAB9B	protein_coding
ENSG00000123572.17	225.7316471	0.375446175	0.214368552	1.751405098	0.079876155	0.239937272	NRK	protein_coding
ENSG00000123575.9	3675.939127	0.101615728	0.055146175	1.842661385	0.065378465	0.210723131	FAM199X	protein_coding
ENSG00000123576.5	2.661709269	1.858994536	0.415160581	4.477772263	7.5426E-06	0.000260503	ESX1	protein_coding
ENSG00000123584.7	4.24457473	0.541296515	0.52219064	1.036587931	0.299927941	0.529586719	MAGEA9	protein_coding
ENSG00000123594.5	0.20186896	-0.140495185	0.975601206	-0.144008827	0.885493499		ATXN3L	protein_coding
ENSG00000123595.8	1475.562886	-0.081313484	0.05329883	-1.525614796	0.127105825	0.318327043	RAB9A	protein_coding
ENSG00000123600.20	1367.704456	-0.218649975	0.055552768	-3.935897056	8.28865E-05	0.001714297	METTL8	protein_coding
ENSG00000123607.15	867.7440159	-0.044861997	0.052348779	-0.856982681	0.391454456	0.615600549	TTC21B	protein_coding
ENSG00000123609.11	2553.504284	-0.040274123	0.069446842	-0.579927352	0.56196361	0.750933347	NMI	protein_coding
ENSG00000123610.5	467.0283662	0.515504937	0.142542967	3.616488056	0.000298627	0.004627764	TNFAIP6	protein_coding
ENSG00000123612.16	288.4367262	0.167410347	0.129414898	1.293594085	0.195805667	0.412842246	ACVR1C	protein_coding
ENSG00000123636.18	2674.604749	-0.007986354	0.064385003	-0.124040596	0.901283132	0.953004344	BAZ2B	protein_coding
ENSG00000123643.13	1771.454558	0.013625567	0.06813874	0.199967989	0.841505617	0.922035912	SLC36A1	protein_coding
ENSG00000123684.13	5426.024221	0.167516129	0.065247698	2.567387568	0.010246801	0.061445557	LPGAT1	protein_coding
ENSG00000123685.9	120.6520449	0.145071591	0.092228332	1.572961233	0.115727795	0.300665959	BATF3	protein_coding

ENSG00000123689.6	1049.752734	0.423884576	0.147612272	2.871607965	0.004083893	0.032251501	G0S2	protein_coding
ENSG00000123700.5	469.5454428	-0.221068316	0.128337574	-1.722553335	0.08496933	0.249357431	KCNJ2	protein_coding
ENSG00000123728.10	2108.23836	-0.044861896	0.052755181	-0.850378968	0.395114425	0.618592416	RAP2C	protein_coding
ENSG00000123737.13	1146.45703	-0.053727583	0.055189105	-0.973517933	0.330295952	0.559845465	EXOSC9	protein_coding
ENSG00000123739.11	1441.456358	-0.151485975	0.0492175	-3.077888442	0.002084729	0.01975348	PLA2G12A	protein_coding
ENSG00000123810.9	249.3670976	-0.045600171	0.061203616	-0.745056813	0.456237376	0.670266878	B9D2	protein_coding
ENSG00000123815.13	1825.483513	0.012774528	0.055193358	0.23145045	0.816964875	0.909787084	COQ8B	protein_coding
ENSG00000123836.15	3847.688133	-0.079964317	0.104136797	-0.767877628	0.44255989	0.658903014	PFKFB2	protein_coding
ENSG00000123838.11	463.9896488	-0.219110377	0.234095684	-0.935986404	0.349280188	0.578421477	C4BPA	protein_coding
ENSG00000123843.13	805.5670602	0.645572406	0.170023052	3.796969877	0.000146476	0.002672934	C4BPB	protein_coding
ENSG00000123870.10	156.012309	0.032721719	0.116696496	0.28040018	0.779170499	0.888374224	ZNF137P	transcribed_unprocessed_pseudogene
ENSG00000123892.12	223.3798552	0.255783975	0.125133883	2.044082462	0.040945401	0.15619583	RAB38	protein_coding
ENSG00000123901.9	29.76418576	0.30850644	0.149993625	2.056797021	0.039705755	0.153358899	GPR83	protein_coding
ENSG00000123908.12	7024.659479	0.095951863	0.063796381	1.504033012	0.132572867	0.326520535	AGO2	protein_coding
ENSG00000123933.17	4360.84561	-0.07764299	0.06407883	-1.211679267	0.225635179	0.448726916	MXD4	protein_coding
ENSG00000123965.13	54.39067314	-0.026547162	0.102776517	-0.258299883	0.796175476	0.897971859	PMS2P5	unprocessed_pseudogene
ENSG00000123975.5	3190.43771	-0.222607003	0.093627445	-2.377582795	0.01742653	0.088548963	CKS2	protein_coding
ENSG00000123977.10	16.4192984	-0.453962443	0.216417561	-2.097622952	0.035938466	0.143296782	DAW1	protein_coding
ENSG00000123983.14	7186.778874	0.150136982	0.060413048	2.485174781	0.012948784	0.072376906	ACSL3	protein_coding
ENSG00000123989.14	12972.77034	0.423557921	0.07943122	5.332385922	9.69307E-08	7.86582E-06	CHPF	protein_coding
ENSG00000123992.20	4978.50121	-0.052433834	0.046408865	-1.129823666	0.258550534	0.485769571	DNPEP	protein_coding
ENSG00000123999.5	47.16411232	0.423158559	0.238679614	1.772914544	0.076242868	0.232728739	INHA	protein_coding
ENSG00000124003.13	11.0859702	0.736939171	0.223839873	3.292260497	0.000993855	0.011442331	MOGAT1	protein_coding
ENSG00000124006.15	2134.993409	0.508270372	0.143201185	3.549344733	0.000386191	0.005592905	OBSL1	protein_coding
ENSG00000124019.10	117.6786471	0.082890648	0.122304222	0.677741515	0.497935599	0.703881	FAM124B	protein_coding
ENSG00000124067.17	2497.401663	0.195963329	0.07185283	2.727287551	0.006385736	0.044146264	SLC12A4	protein_coding
ENSG00000124074.12	622.1252393	-0.010302409	0.070384255	-0.146373781	0.883626324	0.944139474	ENKD1	protein_coding
ENSG00000124089.4	1.044648791	1.189935369	0.388810924	3.06044737	0.002210066	0.020530819	MC3R	protein_coding
ENSG00000124091.9	29.18320806	0.022139936	0.101729216	0.21763596	0.827712764	0.915160965	GCNT7	protein_coding
ENSG00000124092.12	37.15443452	0.824544626	0.270757299	3.045327419	0.002324271	0.021278313	CTCFL	protein_coding
ENSG00000124097.7	33.7509722	-0.024115904	0.095355804	-0.252904418	0.800342075	0.900379135	HMGB1P1	processed_pseudogene
ENSG00000124098.10	6058.108643	0.056833594	0.09134508	0.622185606	0.53381983	0.730981916	FAM210B	protein_coding
ENSG00000124102.5	11299.00378	0.60604334	0.24343394	2.489559752	0.012790142	0.071777979	PI3	protein_coding
ENSG00000124103.10	3.544118293	-0.050386609	0.147472984	-0.341666709	0.732601728	0.861006273	FAM209A	protein_coding
ENSG00000124104.19	832.1576142	0.060526584	0.080851814	0.74861133	0.454091501	0.66841844	SNX21	protein_coding
ENSG00000124107.5	8303.570228	0.438842349	0.169543324	2.58837882	0.009642887	0.058860916	SLPI	protein_coding
ENSG00000124116.19	60.17258454	0.337164061	0.143294605	2.352943161	0.018625481	0.092515967	WFDC3	protein_coding
ENSG00000124120.11	1702.911063	0.022504218	0.064480719	0.349006935	0.727084103	0.857562514	TTPAL	protein_coding
ENSG00000124126.14	3099.502624	-0.05735037	0.09211369	-0.622604197	0.533544652	0.730830448	PREX1	protein_coding
ENSG00000124134.9	85.70189697	0.37057774	0.189657305	1.953933388	0.050709095	0.179505634	KCNS1	protein_coding
ENSG00000124140.14	57.32767102	0.059246044	0.136760834	0.433209146	0.664862835	0.820687414	SLC12A5	protein_coding
ENSG00000124143.10	109.4827287	0.914077353	0.268309774	3.406798569	0.000657296	0.00833645	ARHGAP40	protein_coding

ENSG00000124145.6	21236.40121	-0.129792377	0.095483674	-1.359314856	0.174046838	0.385192436	SDC4	protein_coding
ENSG00000124151.19	6004.990147	-0.098927826	0.063254637	-1.563961636	0.117826578	0.303890199	NCOA3	protein_coding
ENSG00000124155.18	17152.52054	0.018824871	0.077117049	0.244107774	0.807147352	0.904156503	PIGT	protein_coding
ENSG00000124157.7	18.9707278	1.584159115	0.327139104	4.842463335	1.28239E-06	6.34311E-05	SEMG2	protein_coding
ENSG00000124159.15	24.49903192	0.324904239	0.147773908	2.198657685	0.027902272	0.121383379	MATN4	protein_coding
ENSG00000124160.12	2734.186633	-0.066744691	0.050517824	-1.321210744	0.186431105	0.401311381	NCOA5	protein_coding
ENSG00000124164.16	5791.881203	-0.020575276	0.063283316	-0.325129542	0.745083031	0.868801806	VAPB	protein_coding
ENSG00000124171.9	1929.381615	-0.021573493	0.094531894	-0.22821391	0.819479948	0.911051297	PARD6B	protein_coding
ENSG00000124172.10	13281.47998	-0.0594167	0.079057846	-0.751559808	0.452315821	0.666837058	ATP5F1E	protein_coding
ENSG00000124177.15	4205.89781	0.00084219	0.065723543	0.012814124	0.989776088	0.995404463	CHD6	protein_coding
ENSG00000124181.14	4512.434661	-0.123131336	0.068153191	-1.806684822	0.070811436	0.221407041	PLCG1	protein_coding
ENSG00000124191.18	410.5102625	-0.049189125	0.103229636	-0.476501976	0.6337168	0.801354487	TOX2	protein_coding
ENSG00000124193.16	6049.16395	-0.106794259	0.050494104	-2.114984737	0.034431248	0.139352698	SRSF6	protein_coding
ENSG00000124194.17	25.77037446	-0.538146088	0.168977999	-3.18471097	0.001448986	0.015167544	GDAP1L1	protein_coding
ENSG00000124196.6	3.643543023	-0.835274642	0.205302671	-4.068503539	4.7316E-05	0.001097919	GTSF1L	protein_coding
ENSG00000124198.9	7758.419812	0.030208019	0.067965953	0.444458111	0.656711408	0.815522869	ARFGEF2	protein_coding
ENSG00000124201.15	9569.799955	0.037173974	0.061294357	0.60648281	0.544194194	0.738648695	ZNFX1	protein_coding
ENSG00000124203.6	225.4822079	-0.695871947	0.15023106	-4.632011162	3.62131E-06	0.000144378	ZNF831	protein_coding
ENSG00000124205.18	383.6497215	0.077704427	0.212950677	0.364894014	0.715190523	0.8511153	EDN3	protein_coding
ENSG00000124207.17	10190.71584	-0.16052336	0.069240927	-2.318330613	0.02043136	0.098592449	CSE1L	protein_coding
ENSG00000124208.16	0						PEDS1-UBE2V1	protein_coding
ENSG00000124209.4	4764.052884	0.049802785	0.051141037	0.973832138	0.330139893	0.559741952	RAB22A	protein_coding
ENSG00000124212.6	2844.996504	0.49403693	0.188047826	2.627187667	0.008609384	0.054404164	PTGIS	protein_coding
ENSG00000124214.20	12244.74955	-0.036395207	0.05074398	-0.717232014	0.473230949	0.684115746	STAU1	protein_coding
ENSG00000124215.17	65.74160574	0.860926095	0.161985176	5.314844953	1.06748E-07	8.45776E-06	CDH26	protein_coding
ENSG00000124216.4	538.8462988	0.458736799	0.093723368	4.894582953	9.85144E-07	5.13512E-05	SNAI1	protein_coding
ENSG00000124217.5	918.568668	0.032585964	0.068996636	0.472283377	0.636724534	0.803153531	MOCS3	protein_coding
ENSG00000124222.22	3195.891146	0.043180676	0.071750834	0.601814268	0.547297777	0.740848452	STX16	protein_coding
ENSG00000124224.17	658.8665489	0.11065064	0.083996898	1.317318172	0.187731996	0.402888864	PPP4R1L	transcribed_unprocessed_pseudogene
ENSG00000124225.16	13489.68095	0.607639605	0.134962706	4.502277862	6.7229E-06	0.000238376	PMEPA1	protein_coding
ENSG00000124226.11	5859.122688	-0.020182504	0.052909905	-0.381450381	0.702869083	0.843911557	RNF114	protein_coding
ENSG00000124227.5	0.470168584	0.035158418	0.371856119	0.094548446	0.924673501		ANKRD60	protein_coding
ENSG00000124228.14	4329.618193	-0.059369501	0.06715025	-0.884129262	0.376626438	0.602461284	DDX27	protein_coding
ENSG00000124232.11	16.08183222	0.007583028	0.210788067	0.035974656	0.971302567	0.986458933	RBPJL	protein_coding
ENSG00000124233.12	90.13734071	0.375869427	0.272590454	1.378879638	0.167931867	0.377077904	SEMG1	protein_coding
ENSG00000124237.6	4.163079253	0.467926828	0.283322327	1.651570609	0.098622116	0.273200124	C20orf85	protein_coding
ENSG00000124243.17	769.4761568	-0.46438237	0.117939086	-3.937476406	8.2343E-05	0.001706259	BCAS4	protein_coding
ENSG00000124249.7	507.4378992	0.261625977	0.207044947	1.263619231	0.206366728	0.426166435	KCNK15	protein_coding
ENSG00000124251.11	75.62128805	-0.108557819	0.089955056	-1.206800642	0.227508929	0.450889421	TP53TG5	protein_coding
ENSG00000124253.11	1722.996066	-0.273344578	0.245877082	-1.111712306	0.266261867	0.494244904	PCK1	protein_coding
ENSG00000124256.15	400.9263715	-0.417365437	0.120161532	-3.473369811	0.000513967	0.00693839	ZBP1	protein_coding
ENSG00000124257.6	27.35577907	0.034935494	0.098787603	0.35364249	0.723606827	0.855701892	NEURL2	protein_coding

ENSG00000124260.12	140.6799789	0.896817096	0.481163278	1.863851911	0.062342503	0.204740318	MAGEA10	protein_coding
ENSG00000124275.15	2286.786233	0.035397218	0.054901368	0.644742011	0.519094387	0.71975869	MTRR	protein_coding
ENSG00000124279.12	539.5497256	-0.048956113	0.058168416	-0.841627057	0.399996739	0.622728091	FASTKD3	protein_coding
ENSG00000124299.15	4605.345923	-0.094615272	0.068677718	-1.377670588	0.168305016	0.377585858	PEPD	protein_coding
ENSG00000124302.13	24.0537302	0.231043614	0.196032383	1.178599221	0.238557802	0.463982351	CHST8	protein_coding
ENSG00000124313.18	1437.626182	0.084574417	0.06970594	1.213302874	0.225014045	0.448002788	IQSEC2	protein_coding
ENSG00000124333.16	3004.636219	0.053809623	0.050212177	1.071644889	0.283879563	0.512819356	VAMP7	protein_coding
ENSG00000124333.16_PAR_Y	0						VAMP7	protein_coding
ENSG00000124334.17	28.2484918	-0.279170985	0.132760056	-2.102823644	0.035481188	0.141967106	IL9R	protein_coding
ENSG00000124334.17_PAR_Y	0						IL9R	protein_coding
ENSG00000124343.14	174.5133362	-0.277175474	0.217867766	-1.272218825	0.203295381	0.422541391	XG	protein_coding
ENSG00000124356.16	3006.43779	0.059578378	0.035469072	1.679727564	0.093010334	0.263497031	STAMBP	protein_coding
ENSG00000124357.13	3005.398051	0.002087055	0.053695598	0.038868267	0.968995417	0.98565019	NAGK	protein_coding
ENSG00000124370.11	417.245286	-0.057529455	0.056907235	-1.010933948	0.312048044	0.541541921	MCEE	protein_coding
ENSG00000124374.9	400.267122	-0.128309776	0.135194025	-0.949078747	0.342580563	0.572373023	PAIP2B	protein_coding
ENSG00000124380.11	1414.869081	-0.033446403	0.047201355	-0.708589889	0.478579013	0.688126098	SNRNP27	protein_coding
ENSG00000124383.9	2088.142159	-0.07458964	0.043054841	-1.732433293	0.083196444	0.246143761	MPHOSPH10	protein_coding
ENSG00000124391.5	75.34883673	0.522480115	0.221296531	2.360995506	0.018225951	0.091260522	IL17C	protein_coding
ENSG00000124399.4	22.71051121	-0.176470729	0.098833806	-1.785530038	0.074175383	0.228369414	NDUFB4P12	processed_pseudogene
ENSG00000124406.16	2137.689207	0.073648608	0.12356648	0.596024168	0.551159081	0.743471886	ATP8A1	protein_coding
ENSG00000124422.12	8167.453001	0.105627479	0.062690581	1.684901919	0.092007496	0.261608421	USP22	protein_coding
ENSG00000124429.18	6631.141823	-0.012084591	0.119700396	-0.100956986	0.919584606	0.962061277	POF1B	protein_coding
ENSG00000124440.16	1174.076955	-0.306043964	0.215559315	-1.41976682	0.155675578	0.360290417	HIF3A	protein_coding
ENSG00000124444.16	503.9182633	-0.080490693	0.054275397	-1.483005152	0.138073042	0.334620374	ZNF576	protein_coding
ENSG00000124449.7	2.643456695	0.352240625	0.234293192	1.503418095	0.132731263	0.326818299	IRGC	protein_coding
ENSG00000124459.12	689.837469	0.012755175	0.053708602	0.237488489	0.812277848	0.907271413	ZNF45	protein_coding
ENSG00000124466.9	738.8330115	1.125866064	0.184061768	6.116783926	9.54827E-10	1.73369E-07	LYPD3	protein_coding
ENSG00000124467.19	2.185691769	0.384141452	0.376495988	1.020306893	0.307582935	0.537038728	PSG8	protein_coding
ENSG00000124469.12	18.51459768	0.334505222	0.167019548	2.002790847	0.045199743	0.166739059	CEACAM8	protein_coding
ENSG00000124479.11	26.97687659	0.160902756	0.18757922	0.857785612	0.391010856	0.615334117	NDP	protein_coding
ENSG00000124486.13	12360.73426	0.009539116	0.057198036	0.166773487	0.867548281	0.935822781	USP9X	protein_coding
ENSG00000124490.14	13.60254485	0.120028619	0.377016931	0.318364003	0.75020884	0.871782121	CRISP2	protein_coding
ENSG00000124491.16	1532.026309	0.215054237	0.184989195	1.162523236	0.245022992	0.471323692	F13A1	protein_coding
ENSG00000124493.14	29.01814402	0.411502871	0.184785785	2.226918431	0.025952726	0.115744748	GRM4	protein_coding
ENSG00000124496.12	1714.926818	0.153830821	0.085839759	1.792069581	0.073121821	0.226132254	TRERF1	protein_coding
ENSG00000124507.11	141.0242938	-0.067588255	0.171049804	-0.395137868	0.692741143	0.8377527	PACSIN1	protein_coding
ENSG00000124508.17	695.8236652	-0.279727131	0.078487968	-3.563949201	0.000365317	0.005390926	BTN2A2	protein_coding
ENSG00000124523.16	828.7070634	-0.117667305	0.048995988	-2.401570196	0.016324877	0.085074043	SIRT5	protein_coding
ENSG00000124532.15	1824.799025	-0.086081215	0.050953559	-1.689405331	0.09114178	0.260216559	MRS2	protein_coding
ENSG00000124535.16	3998.810777	-0.007970552	0.05458512	-0.146020596	0.88390513	0.944321602	WRNIP1	protein_coding
ENSG00000124541.7	2766.955217	0.079677427	0.056373986	1.413372241	0.157546303	0.362615288	RRP36	protein_coding
ENSG00000124549.14	220.6391599	0.034822984	0.075329839	0.462273436	0.643885247	0.807580803	BTN2A3P	transcribed_unprocessed_pseudogene

ENSG00000124557.12	46.30511913	-0.369148535	0.194711622	-1.895873143	0.057976821	0.195667281	BTN1A1	protein_coding
ENSG00000124562.10	4305.253076	-0.115672252	0.059393279	-1.947564689	0.051467072	0.181220364	SNRPC	protein_coding
ENSG00000124564.17	4.183595033	-0.401442085	0.255989382	-1.568198186	0.116834888	0.302262758	SLC17A3	protein_coding
ENSG00000124568.12	5.742454473	-0.841314236	0.271052713	-3.103876832	0.001910028	0.018500401	SLC17A1	protein_coding
ENSG00000124570.21	10036.79782	-0.097823198	0.076416439	-1.280132917	0.200498394	0.419008932	SERPINB6	protein_coding
ENSG00000124571.18	4556.795331	0.219533506	0.077612816	2.828572853	0.004675606	0.035496319	XPO5	protein_coding
ENSG00000124574.15	2307.143366	0.278474629	0.067364162	4.133869116	3.56707E-05	0.000875562	ABCC10	protein_coding
ENSG00000124575.7	37.55238929	0.115232681	0.139859514	0.823917359	0.409986516	0.630883238	H1-3	protein_coding
ENSG00000124587.14	1766.128403	0.143287117	0.106534457	1.344983784	0.178630471	0.391263134	PEX6	protein_coding
ENSG00000124588.21	1672.731834	-0.012334774	0.071057489	-0.173588658	0.862188746	0.932883995	NQO2	protein_coding
ENSG00000124593.16	35.94523082	0.031924681	0.111359211	0.286681999	0.774355822	0.885856378	AL365205.1	protein_coding
ENSG00000124596.17	1652.100144	-0.267643306	0.063548866	-4.2116142	2.53552E-05	0.000664804	OARD1	protein_coding
ENSG00000124602.10	1738.003184	-0.649983839	0.147724696	-4.39996734	1.08267E-05	0.000345891	UNC5CL	protein_coding
ENSG00000124608.5	1642.059189	0.241277368	0.074331936	3.245944899	0.001170615	0.012953755	AARS2	protein_coding
ENSG00000124610.5	3.144649506	0.980877569	0.256119168	3.82977025	0.000128263	0.002392686	H1-1	protein_coding
ENSG00000124613.8	312.0693391	0.144431382	0.107374795	1.345114388	0.178588297	0.391190421	ZNF391	protein_coding
ENSG00000124614.16	212.4122788	-0.182060364	0.08740106	-2.083045259	0.037247098	0.146795406	RPS10	protein_coding
ENSG00000124615.20	707.4669334	0.095836782	0.10509525	0.911904034	0.361819238	0.589497945	MOCS1	protein_coding
ENSG00000124635.9	211.4239284	-0.181010742	0.146507565	-1.235504407	0.216642837	0.437938717	H2BC11	protein_coding
ENSG00000124641.16	1367.433966	0.116509754	0.063292322	1.840819693	0.065647986	0.211185035	MED20	protein_coding
ENSG00000124657.1	17.15367866	-0.38533355	0.200785938	-1.919126181	0.054968367	0.189117048	OR2B6	protein_coding
ENSG00000124659.7	1284.585013	0.049194703	0.053755451	0.915157482	0.360108962	0.588019194	TBCC	protein_coding
ENSG00000124664.11	1825.644778	0.307363725	0.192563049	1.596171883	0.110450423	0.292095217	SPDEF	protein_coding
ENSG00000124678.20	20.79510321	0.920207372	0.237600956	3.872911074	0.000107543	0.002094241	TCP11	protein_coding
ENSG00000124688.14	1410.857639	0.121235237	0.069253713	1.750595485	0.080015613	0.240094849	MAD2L1BP	protein_coding
ENSG00000124701.5	85.24987188	-0.977711858	0.174722122	-5.595810342	2.19594E-08	2.29478E-06	APOBEC2	protein_coding
ENSG00000124702.18	4820.35088	0.126242378	0.062465947	2.020979197	0.043281919	0.162206755	KLHDC3	protein_coding
ENSG00000124713.6	26.84851857	-0.330659372	0.118009103	-2.801981903	0.005078972	0.037614951	GNMT	protein_coding
ENSG00000124721.18	55.90989458	-0.175074035	0.185011102	-0.946289345	0.344001026	0.573690498	DNAH8	protein_coding
ENSG00000124731.13	430.9733463	0.650976772	0.157694295	4.128093355	3.65784E-05	0.000893985	TREM1	protein_coding
ENSG00000124733.5	2358.213548	0.053443613	0.066833789	0.799649608	0.423913837	0.642965805	MEA1	protein_coding
ENSG00000124743.6	72.96706655	0.031957831	0.115270736	0.277241494	0.78159468	0.889469115	KLHL31	protein_coding
ENSG00000124749.17	160.8711568	0.069470811	0.158128064	0.439332587	0.660420565	0.817999121	COL21A1	protein_coding
ENSG00000124762.14	7449.238287	0.337126485	0.09250733	3.644321872	0.000268098	0.00426383	CDKN1A	protein_coding
ENSG00000124766.7	12130.46947	0.093818781	0.094131775	0.996674938	0.318922318	0.548404935	SOX4	protein_coding
ENSG00000124767.7	8742.221945	0.070019881	0.060997488	1.147914169	0.251003998	0.477680224	GLO1	protein_coding
ENSG00000124772.12	731.6618288	-0.572631677	0.111215387	-5.148852963	2.62084E-07	1.76248E-05	CPNE5	protein_coding
ENSG00000124780.14	36.89173983	0.216680647	0.156802728	1.381867845	0.167012283	0.37579175	KCNK17	protein_coding
ENSG00000124782.20	7051.920751	0.101735769	0.051040564	1.993233637	0.046235869	0.169167093	RREB1	protein_coding
ENSG00000124783.14	13840.84886	0.076650616	0.050411732	1.52049161	0.128387466	0.320083573	SSR1	protein_coding
ENSG00000124784.9	1314.600264	0.013800345	0.053225631	0.259280057	0.795419166	0.897629962	RIOK1	protein_coding
ENSG00000124785.9	368.5282555	0.2905307	0.152469968	1.905494592	0.05671581	0.192920525	NRN1	protein_coding

ENSG00000124786.12	1751.565758	-0.019293478	0.05585985	-0.345390793	0.729800598	0.85931649	SLC35B3	protein_coding
ENSG00000124787.14	376.2483503	-0.060947228	0.082292302	-0.740618822	0.458924593	0.672175214	RPP40	protein_coding
ENSG00000124788.19	2970.525725	0.045349412	0.072156712	0.628485022	0.529686256	0.727752246	ATXN1	protein_coding
ENSG00000124789.12	4881.533511	-0.010786393	0.052931132	-0.203781647	0.838524136	0.920544428	NUP153	protein_coding
ENSG00000124795.17	11163.49775	-0.033534238	0.056326509	-0.595354444	0.55160657	0.743701843	DEK	protein_coding
ENSG00000124802.12	663.8104566	-0.225344767	0.083537479	-2.69752893	0.006985621	0.047006168	EEF1E1	protein_coding
ENSG00000124812.15	2.185009649	0.044607739	0.381373928	0.116965884	0.906887088	0.955912919	CRISP1	protein_coding
ENSG00000124813.23	961.5261908	0.309319243	0.097313953	3.17857033	0.001480033	0.015379607	RUNX2	protein_coding
ENSG00000124818.15	1.985137399	0.574036109	0.297444892	1.929890625	0.053620391	0.185962259	OPN5	protein_coding
ENSG00000124827.7	3.032743829	-0.186280512	0.247944693	-0.751298644	0.452472945	0.666939979	GCM2	protein_coding
ENSG00000124831.19	10984.6804	0.1363654	0.051284096	2.659019271	0.007836848	0.051010764	LRRFIP1	protein_coding
ENSG00000124835.2	2.34394478	-0.186409357	0.213565924	-0.87284223	0.382749078	0.607881645	AC105760.1	lncRNA
ENSG00000124839.13	1147.149101	-0.115105253	0.092055876	-1.250384625	0.211159078	0.431598459	RAB17	protein_coding
ENSG00000124875.10	571.1438849	1.024516327	0.214321899	4.78026899	1.75061E-06	8.03013E-05	CXCL6	protein_coding
ENSG00000124882.4	591.7982398	1.032873177	0.225827729	4.573721674	4.79136E-06	0.000179684	EREG	protein_coding
ENSG00000124900.12	0.516509126	-0.341866223	0.670449457	-0.509906034	0.610117295		TRIM51	protein_coding
ENSG00000124915.10	31.61167385	0.027159203	0.142885065	0.190077272	0.84924858	0.926152884	MYRF-AS1	lncRNA
ENSG00000124920.14	10763.0052	0.26650534	0.110375568	2.414531989	0.015755439	0.082829628	MYRF	protein_coding
ENSG00000124935.4	1.20154975	1.085737559	0.458675938	2.367112525	0.017927481	0.090162634	SCGB1D2	protein_coding
ENSG00000124939.6	80.62732313	0.624481299	0.280869159	2.223388643	0.026189605	0.116479323	SCGB2A1	protein_coding
ENSG00000124942.14	51225.81479	0.145172431	0.095750179	1.516158324	0.129479323	0.321903267	AHNAK	protein_coding
ENSG00000125037.12	2812.03083	0.013352449	0.051283886	0.260363446	0.794583436	0.897089848	EMC3	protein_coding
ENSG00000125046.15	127.6049197	0.129332849	0.164267306	0.787331645	0.43108775	0.649119242	SSUH2	protein_coding
ENSG00000125084.12	18.18218573	-0.067314105	0.153371681	-0.438895267	0.660737427	0.818167619	WNT1	protein_coding
ENSG00000125089.17	1492.093779	0.203330363	0.086335345	2.355123077	0.018516572	0.092210046	SH3TC1	protein_coding
ENSG00000125107.18	14038.24968	0.002421885	0.066376035	0.036487334	0.970893778	0.98634098	CNOT1	protein_coding
ENSG00000125122.15	122.1028799	-0.024541714	0.081349647	-0.301681877	0.762894585	0.878860531	LRRC29	protein_coding
ENSG00000125124.13	1288.346355	-0.057602507	0.058706392	-0.981196502	0.326495849	0.556264821	BBS2	protein_coding
ENSG00000125144.14	2725.91201	-0.841174736	0.194700145	-4.320360085	1.55775E-05	0.000459283	MT1G	protein_coding
ENSG00000125148.7	6366.200752	-0.055695364	0.127092042	-0.438228571	0.661220599	0.818510072	MT2A	protein_coding
ENSG00000125149.12	1267.405341	0.069625125	0.048113424	1.447103928	0.147867818	0.349286422	C16orf70	protein_coding
ENSG00000125166.13	8085.76222	-0.086983903	0.055068807	-1.57954945	0.114210084	0.298162761	GOT2	protein_coding
ENSG00000125170.11	4115.538118	0.072180862	0.081498839	0.885667366	0.375796799	0.601642128	DOK4	protein_coding
ENSG00000125207.7	379.4728513	0.983112399	0.26913741	3.652827006	0.000259369	0.004155539	PIWIL1	protein_coding
ENSG00000125245.13	93.78696257	-0.730445423	0.122026241	-5.985970029	2.15104E-09	3.4217E-07	GPR18	protein_coding
ENSG00000125246.15	397.8823521	-0.073992871	0.085673544	-0.863660678	0.387774321	0.612231382	CLYBL	protein_coding
ENSG00000125247.16	2027.423785	0.086020707	0.073226536	1.174720426	0.240106585	0.465490745	TMTC4	protein_coding
ENSG00000125249.7	2708.239194	0.172262285	0.063500371	2.712776037	0.006672218	0.0454801	RAP2A	protein_coding
ENSG00000125255.7	32.62780538	-0.346800777	0.320337907	-1.082609235	0.278981906	0.507798109	SLC10A2	protein_coding
ENSG00000125257.16	1900.111073	-0.192037488	0.104734467	-1.833565329	0.066718543	0.213308433	ABCC4	protein_coding
ENSG00000125266.8	3364.122534	0.248854794	0.079829467	3.117330038	0.001824971	0.017887265	EFNB2	protein_coding
ENSG00000125285.6	542.8402039	0.108298242	0.280426319	0.386191433	0.699354898	0.841799058	SOX21	protein_coding

ENSG00000125304.10	17903.1767	0.159332124	0.058722783	2.71329314	0.006661815	0.045435374	TM9SF2	protein_coding
ENSG00000125319.15	464.1833616	0.153903931	0.08376367	1.837358975	0.066156919	0.212127072	HROB	protein_coding
ENSG00000125337.19	15.62219594	0.219524442	0.171578365	1.279441279	0.200741706	0.419333747	KIF25	protein_coding
ENSG00000125347.14	4718.99883	-0.149158733	0.096072501	-1.552564276	0.120527266	0.308375171	IRF1	protein_coding
ENSG00000125351.12	1468.894127	-0.112974837	0.07855485	-1.438165006	0.150387242	0.352638013	UPF3B	protein_coding
ENSG00000125352.6	828.9859012	-0.132545274	0.053488565	-2.478011399	0.013211692	0.073406201	RNF113A	protein_coding
ENSG00000125354.24	2985.889405	-0.351573459	0.095414373	-3.684701264	0.000228971	0.003775207	SEPTIN6	protein_coding
ENSG00000125355.16	191.1863919	-0.007000153	0.14143181	-0.049494898	0.960524903	0.981655145	TMEM255A	protein_coding
ENSG00000125356.7	4646.819112	-0.283622851	0.072325851	-3.9214589	8.80145E-05	0.001798128	NDUFA1	protein_coding
ENSG00000125363.14	7.910277582	-0.482652526	0.214313629	-2.252085076	0.024316896	0.11088922	AMELX	protein_coding
ENSG00000125375.16	709.6945363	-0.035600589	0.048304599	-0.737002064	0.461121094	0.673893371	DMAC2L	protein_coding
ENSG00000125378.16	2600.040132	0.140255135	0.13634771	1.028657795	0.303640509	0.533172521	BMP4	protein_coding
ENSG00000125384.7	782.7058338	0.260029149	0.135202012	1.923263902	0.054446916	0.187886699	PTGER2	protein_coding
ENSG00000125385.9	5.673721117	-0.382149672	0.133765856	-2.856855121	0.004278611	0.033252903	AL161781.1	processed_pseudogene
ENSG00000125386.16	2181.539946	-0.02516003	0.040736024	-0.617635895	0.536815378	0.733080572	FAM193A	protein_coding
ENSG00000125388.20	235.1162482	0.014849621	0.05969152	0.248772713	0.8035366	0.902413155	GRK4	protein_coding
ENSG00000125398.8	16483.0543	0.124305297	0.084165171	1.476920868	0.13969685	0.336947205	SOX9	protein_coding
ENSG00000125409.13	15.50539376	0.050958964	0.130259711	0.391210481	0.695641662	0.839430883	TEKT3	protein_coding
ENSG00000125414.19	4.687255296	-0.133309019	0.247472354	-0.53868247	0.590105972	0.771221039	MYH2	protein_coding
ENSG00000125430.9	317.4556338	0.351606207	0.106320561	3.307038668	0.000942879	0.010967167	HS3ST3B1	protein_coding
ENSG00000125434.11	612.4420248	-0.200500907	0.084175444	-2.381940587	0.017221676	0.087876928	SLC25A35	protein_coding
ENSG00000125445.11	2331.50659	0.022271071	0.061186253	0.363988143	0.715866863	0.851561582	MRPS7	protein_coding
ENSG00000125447.18	3049.345855	0.028712987	0.042489259	0.675770474	0.499186384	0.704690853	GGA3	protein_coding
ENSG00000125449.7	1252.547893	0.022795709	0.066932954	0.340575276	0.733423345	0.861582129	ARMC7	protein_coding
ENSG00000125450.11	2483.436676	-0.029053228	0.047103426	-0.616796502	0.53736896	0.733544064	NUP85	protein_coding
ENSG00000125454.12	433.9426336	-0.018603138	0.06912095	-0.269138926	0.787822776	0.893081687	SLC25A19	protein_coding
ENSG00000125457.15	1397.008208	0.021958336	0.062107804	0.353551969	0.723674675	0.855742753	MIF4GD	protein_coding
ENSG00000125458.7	783.6658764	-0.037298383	0.063828805	-0.584350332	0.558984636	0.748866368	NT5C	protein_coding
ENSG00000125459.17	578.1652053	0.156507039	0.060129836	2.602818345	0.009246093	0.057217636	MSTO1	protein_coding
ENSG00000125462.19	75.68168357	-0.052908228	0.184941021	-0.286081626	0.774815604	0.886035101	MIR9-1HG	lncRNA
ENSG00000125482.13	900.3995119	-0.006171031	0.045557518	-0.135455827	0.892251487	0.948587256	TTF1	protein_coding
ENSG00000125484.12	3260.244065	0.104592953	0.060753941	1.72158301	0.085145083	0.249671441	GTF3C4	protein_coding
ENSG00000125485.17	1081.446914	-0.001540734	0.055067718	-0.027978903	0.977678978	0.989997464	DDX31	protein_coding
ENSG00000125492.10	2.439495757	0.355488618	0.213349247	1.666228608	0.09566789	0.267814413	BARHL1	protein_coding
ENSG00000125498.20	10.03560193	-0.203664904	0.1946006	-1.046579015	0.295293785	0.525146329	KIR2DL1	protein_coding
ENSG00000125503.13	4658.020648	-0.06861419	0.082156678	-0.835162669	0.403626126	0.625280136	PPP1R12C	protein_coding
ENSG00000125505.17	7584.901159	0.191980248	0.0641349	2.993381903	0.002759043	0.024133039	MBOAT7	protein_coding
ENSG00000125508.3	331.7634453	0.384350718	0.154344009	2.490221162	0.012766363	0.071690694	SRMS	protein_coding
ENSG00000125510.18	13.21142106	0.081283206	0.142034595	0.572277519	0.567133978	0.754787202	OPRL1	protein_coding
ENSG00000125514.9	2.547150706	-0.063487697	0.320946461	-0.197813979	0.843190609	0.923000255	LINC00029	lncRNA
ENSG00000125520.14	4386.824135	-0.16762505	0.092670802	-1.808822692	0.070478554	0.220746348	SLC2A4RG	protein_coding
ENSG00000125522.3	0.738362311	0.177614084	0.377116745	0.470979043	0.637655701		NPBWR2	protein_coding

ENSG00000125531.7	78.62084744	0.51834521	0.141230094	3.670217843	0.000242344	0.003933584	FNDC11	protein_coding
ENSG00000125533.6	0.753129774	-0.251652848	0.375226641	-0.670668926	0.502431462		BHLHE23	protein_coding
ENSG00000125534.10	42603.19775	-0.068149593	0.086932631	-0.783935698	0.433077842	0.651103437	PPDPF	protein_coding
ENSG00000125538.12	2172.30734	0.002236036	0.192470758	0.011617538	0.990730754	0.995826777	IL1B	protein_coding
ENSG00000125551.19	7.9837861	0.153198792	0.133330675	1.14901385	0.25055027	0.477350038	PLGLB2	protein_coding
ENSG00000125571.9	11.80235332	-0.073209393	0.187413364	-0.390630591	0.69607031	0.839658295	IL37	protein_coding
ENSG00000125611.15	1215.29555	-0.167671692	0.068691609	-2.440934099	0.014649328	0.078801846	CHCHD5	protein_coding
ENSG00000125618.17	532.8394605	0.268306831	0.12195072	2.200125031	0.027798025	0.121081765	PAX8	protein_coding
ENSG00000125629.15	1417.40287	0.008124833	0.05709481	0.142304227	0.886839708	0.945880572	INSIG2	protein_coding
ENSG00000125630.16	2685.906569	-0.074991655	0.052896601	-1.417702719	0.156277576	0.361068232	POLR1B	protein_coding
ENSG00000125631.7	6.405855074	-0.086055877	0.120093969	-0.716571177	0.473638734	0.684364705	HTR5BP	transcribed_unprocessed_pseudogene
ENSG00000125633.11	2255.766322	-0.013438357	0.049125835	-0.273549681	0.784430708	0.890649739	CCDC93	protein_coding
ENSG00000125637.16	5386.860533	-0.114202004	0.072620383	-1.572588851	0.115814051	0.300764535	PSD4	protein_coding
ENSG00000125648.15	6422.417591	0.014138896	0.083596475	0.169132686	0.865692275	0.934856697	SLC25A23	protein_coding
ENSG00000125650.4	192.7102388	-0.025222382	0.063447016	-0.397534572	0.690973297	0.837108268	PSPN	protein_coding
ENSG00000125651.14	4539.361464	0.014400972	0.044572084	0.323093982	0.746624067	0.869598361	GTF2F1	protein_coding
ENSG00000125652.8	2429.328315	-0.18217434	0.079594939	-2.288767879	0.022092843	0.103831553	ALKBH7	protein_coding
ENSG00000125656.11	2798.454516	-0.058443555	0.061449324	-0.951085394	0.341561029	0.571448406	CLPP	protein_coding
ENSG00000125657.5	572.949678	0.941708558	0.180925511	5.204951764	1.94047E-07	1.39079E-05	TNFSF9	protein_coding
ENSG00000125675.18	32.64099828	0.027075185	0.130526915	0.207429897	0.835674136	0.919467379	GRIA3	protein_coding
ENSG00000125676.20	6423.707765	-0.14563197	0.060852355	-2.393201858	0.016702049	0.086286331	THOC2	protein_coding
ENSG00000125686.12	4447.256831	0.084263089	0.077297495	1.090114094	0.275662888	0.504404858	MED1	protein_coding
ENSG00000125691.14	38386.33973	-0.190197134	0.065920361	-2.885256251	0.003910952	0.031271423	RPL23	protein_coding
ENSG00000125695.12	0						AC046185.1	protein_coding
ENSG00000125703.15	720.6677172	-0.152075825	0.04107707	-3.702207224	0.000213732	0.003564407	ATG4C	protein_coding
ENSG00000125726.11	145.1306194	0.530314442	0.166566881	3.183792838	0.00145359	0.015201126	CD70	protein_coding
ENSG00000125730.17	47903.96647	0.254890819	0.154005838	1.65507245	0.097909803	0.272024255	C3	protein_coding
ENSG00000125731.13	1766.040757	0.199834566	0.076665125	2.60659021	0.009144873	0.056759171	SH2D3A	protein_coding
ENSG00000125733.18	3476.221356	0.246418158	0.070636265	3.48855022	0.000485648	0.006648779	TRIP10	protein_coding
ENSG00000125734.15	3659.370346	0.108778094	0.053658923	2.027213501	0.042640583	0.160573227	GPR108	protein_coding
ENSG00000125735.11	266.3480966	-0.272827736	0.113239486	-2.4092986	0.015983215	0.083703949	TNFSF14	protein_coding
ENSG00000125740.14	4800.046187	0.46365481	0.180716501	2.565647339	0.010298347	0.061695227	FOSB	protein_coding
ENSG00000125741.5	1951.677066	0.007554245	0.046273844	0.163250869	0.870320916	0.93732328	OPA3	protein_coding
ENSG00000125743.10	7205.449738	-0.126767905	0.064012255	-1.980369298	0.047662047	0.172634779	SNRPD2	protein_coding
ENSG00000125744.12	539.8356851	0.108056292	0.096836779	1.115860036	0.26448205	0.492273262	RTN2	protein_coding
ENSG00000125746.18	2416.683865	0.15971309	0.065125015	2.452407737	0.014190376	0.077096027	EML2	protein_coding
ENSG00000125753.14	10962.27938	0.084978935	0.063913727	1.329588173	0.183653997	0.397588479	VASP	protein_coding
ENSG00000125755.19	6367.29582	0.039214743	0.05515569	0.71098272	0.477094944	0.686876119	SYMPK	protein_coding
ENSG00000125772.14	3070.302985	-0.122162787	0.084510726	-1.445529967	0.148309082	0.349873585	GPCPD1	protein_coding
ENSG00000125775.15	3450.260719	-0.062056369	0.126636122	-0.49003687	0.624107809	0.794924327	SDCBP2	protein_coding
ENSG00000125779.22	1468.542817	-0.096771824	0.046273833	-2.091286122	0.036502423	0.144790287	PANK2	protein_coding
ENSG00000125780.12	1.295537006	-0.025603847	0.361494704	-0.070827724	0.943534867	0.973443884	TGM3	protein_coding

ENSG00000125787.11	9.910278953	0.004937149	0.1340013	0.036844038	0.970609361	0.986192758	GNRH2	protein_coding
ENSG00000125788.6	6.645711568	-0.072406293	0.439171085	-0.164870355	0.869046026	0.936510232	DEFB126	protein_coding
ENSG00000125798.15	2543.417044	-0.130378673	0.152070287	-0.857357968	0.391247081	0.61552309	FOXA2	protein_coding
ENSG00000125804.14	10.55180887	0.120538938	0.190393345	0.633104788	0.526665216	0.725363152	FAM182A	lncRNA
ENSG00000125810.10	5665.706504	0.1109164	0.096551865	1.148775327	0.250648636	0.47737364	CD93	protein_coding
ENSG00000125812.16	1315.878399	-0.069160571	0.054346225	-1.272592009	0.203162856	0.422427129	GZF1	protein_coding
ENSG00000125813.13	11.1731033	0.157254768	0.281433465	0.558763572	0.576323087	0.761135627	PAX1	protein_coding
ENSG00000125814.17	772.5942723	-0.021467443	0.075926143	-0.282741123	0.777375291	0.887099878	NAPB	protein_coding
ENSG00000125815.9	2.368545729	0.31308194	0.355779074	0.879989754	0.37886486	0.604587839	CST8	protein_coding
ENSG00000125816.5	0.799394885	0.784541599	0.315311014	2.488151586	0.012840899		NKX2-4	protein_coding
ENSG00000125817.8	8370.650313	-0.096847969	0.072160001	-1.342128159	0.179554445	0.392486609	CENPB	protein_coding
ENSG00000125818.18	10879.73202	-0.204695773	0.05132799	-3.987995114	6.6634E-05	0.001446071	PSMF1	protein_coding
ENSG00000125820.6	10.66205247	0.315408359	0.334430528	0.943120716	0.345619161	0.575156426	NKX2-2	protein_coding
ENSG00000125821.13	601.5905987	0.083219535	0.078701661	1.057405066	0.290326761	0.520148388	DTD1	protein_coding
ENSG00000125823.12	12.02090823	-0.105595619	0.216673576	-0.487348856	0.626011131	0.796092073	CSTL1	protein_coding
ENSG00000125826.21	7479.884426	-0.121924553	0.062192279	-1.96044518	0.049943779	0.17791489	RBCK1	protein_coding
ENSG00000125827.9	4399.773407	-0.224317753	0.069341358	-3.234977779	0.001216522	0.013333093	TMX4	protein_coding
ENSG00000125831.10	1.316256434	-0.024724143	0.300288947	-0.08233451	0.934380713	0.969071546	CST11	protein_coding
ENSG00000125834.13	4364.947111	0.049269075	0.05972395	0.824946699	0.409401852	0.63029516	STK35	protein_coding
ENSG00000125835.19	11099.57566	-0.178260769	0.075213334	-2.370068711	0.01778478	0.089741471	SNRPB	protein_coding
ENSG00000125841.13	1814.194064	0.142916099	0.111261526	1.284506014	0.198964961	0.416981489	NRSN2	protein_coding
ENSG00000125843.11	1066.313687	-0.189443339	0.0618438	-3.063255168	0.002189433	0.0204258	AP5S1	protein_coding
ENSG00000125844.16	38963.22731	-0.089693971	0.083419319	-1.075218222	0.282277039	0.511044423	RRBP1	protein_coding
ENSG00000125845.7	2446.836504	-0.356058248	0.119572319	-2.977764845	0.002903587	0.025030583	BMP2	protein_coding
ENSG00000125846.15	731.636634	-0.052553305	0.06010249	-0.874394814	0.381903281	0.607135794	ZNF133	protein_coding
ENSG00000125848.10	1594.569395	-0.260951522	0.155902523	-1.673812047	0.094167544	0.265144484	FLRT3	protein_coding
ENSG00000125850.11	941.459335	-0.182607299	0.094611606	-1.930072917	0.053597804	0.185898718	OVOL2	protein_coding
ENSG00000125851.10	54.2092087	-0.989901707	0.239228038	-4.137900026	3.50499E-05	0.000864169	PCSK2	protein_coding
ENSG00000125861.16	1.667315783	0.0651478	0.299715015	0.217365819	0.827923267	0.915254375	GFRA4	protein_coding
ENSG00000125863.20	1463.341588	-0.259501091	0.060404862	-4.296029851	1.73884E-05	0.000497169	MKKS	protein_coding
ENSG00000125864.14	125.3245981	-0.114483764	0.104537968	-1.095140516	0.273455081	0.501994779	BFSP1	protein_coding
ENSG00000125868.16	26448.75432	-0.176245464	0.081599029	-2.159896585	0.030780676	0.129734938	DSTN	protein_coding
ENSG00000125869.10	205.9105179	0.598745819	0.168855078	3.545915388	0.000391252	0.005647426	LAMP5	protein_coding
ENSG00000125870.11	4569.123168	-0.333599913	0.075143854	-4.439483689	9.01749E-06	0.000298825	SNRPB2	protein_coding
ENSG00000125871.14	1646.666231	-0.232194073	0.065454964	-3.547386793	0.000389073	0.005621562	MGME1	protein_coding
ENSG00000125872.8	122.8106239	-0.365313107	0.187710217	-1.946154625	0.051636169	0.181654099	LRRN4	protein_coding
ENSG00000125875.14	3791.666313	-0.093393978	0.042980845	-2.172920936	0.029786268	0.127017929	TBC1D20	protein_coding
ENSG00000125877.13	2650.970408	-0.122311203	0.067519812	-1.81148614	0.070065633	0.219863907	ITPA	protein_coding
ENSG00000125878.6	36.82955362	0.375274805	0.144368226	2.59942797	0.009337927	0.057595158	TCF15	protein_coding
ENSG00000125879.5	1.133150468	0.306049577	0.339933169	0.900322782	0.367948501	0.595002138	OTOR	protein_coding
ENSG00000125885.13	902.9769389	-0.239311573	0.077775707	-3.076944998	0.002091339	0.019790287	MCM8	protein_coding
ENSG00000125888.15	9.493774789	-1.41330648	0.287891658	-4.909160938	9.14669E-07	4.84897E-05	BANF2	protein_coding

ENSG00000125895.5	135.9903808	-0.455161525	0.153951413	-2.956527095	0.003111249	0.02640478	TMEM74B	protein_coding
ENSG00000125898.13	1398.791706	-0.214150226	0.083725287	-2.557772366	0.010534503	0.062542509	FAM110A	protein_coding
ENSG00000125899.8	4.596558592	-0.427613541	0.174307148	-2.453218618	0.014158426	0.076979113	LINC02871	lncRNA
ENSG00000125900.13	4.545888244	-0.436355269	0.187524866	-2.326919512	0.01996955	0.097165371	SIRPD	protein_coding
ENSG00000125901.6	2348.310906	-0.227566936	0.06993948	-3.253769369	0.001138847	0.012679493	MRPS26	protein_coding
ENSG00000125903.4	0.402207915	-0.380778736	0.664879852	-0.572703076	0.566845754		DEFB129	protein_coding
ENSG00000125910.6	255.5045963	-0.261019672	0.132855395	-1.964690044	0.049450123	0.176663091	S1PR4	protein_coding
ENSG00000125912.11	8411.078918	0.073490705	0.063407304	1.159025858	0.246445645	0.47293297	NCLN	protein_coding
ENSG00000125931.11	6.376585707	-0.200110236	0.216069887	-0.926136626	0.354374982	0.58259449	CITED1	protein_coding
ENSG00000125944.21	9251.809789	-0.034800589	0.040207714	-0.865520192	0.386753338	0.611300334	HNRNPR	protein_coding
ENSG00000125945.15	1163.710415	0.034023187	0.059463174	0.572172405	0.567205181	0.754835858	ZNF436	protein_coding
ENSG00000125952.20	2599.181578	-0.006293429	0.043956979	-0.143172454	0.886153984	0.945550136	MAX	protein_coding
ENSG00000125954.12	0						CHURC1-FNTB	protein_coding
ENSG00000125962.14	736.5767127	-0.007832213	0.057624766	-0.135917484	0.891886514	0.948476917	ARMCX5	protein_coding
ENSG00000125965.9	9.748015669	-0.134530307	0.185513874	-0.725176528	0.468343741	0.679824629	GDF5	protein_coding
ENSG00000125966.10	281.0258867	0.13791876	0.141084629	0.977560497	0.328291743	0.557791145	MMP24	protein_coding
ENSG00000125967.17	1612.059555	-0.267542226	0.074515095	-3.590443328	0.000330116	0.004994953	NECAB3	protein_coding
ENSG00000125968.9	6169.312608	0.097633016	0.122047714	0.799957759	0.423735271	0.642846118	ID1	protein_coding
ENSG00000125970.12	11294.58744	-0.127543969	0.060909692	-2.093984807	0.036261334	0.14421467	RALY	protein_coding
ENSG00000125971.17	1172.216312	-0.245208441	0.088777108	-2.762068357	0.005743645	0.041075291	DYNLRB1	protein_coding
ENSG00000125975.14	2.988779471	-0.192055125	0.24802267	-0.774345042	0.438726774	0.655412981	C20orf173	protein_coding
ENSG00000125977.7	8426.103836	-0.155756063	0.060379124	-2.579634363	0.009890497	0.059944118	EIF2S2	protein_coding
ENSG00000125991.20	16466.52097	-0.063179202	0.063711288	-0.99164847	0.321369036	0.551176735	ERGIC3	protein_coding
ENSG00000125995.16	3366.79126	-0.199049839	0.073529692	-2.707067516	0.006788045	0.046046488	ROMO1	protein_coding
ENSG00000125997.5	11.68387218	-0.27310502	0.143138049	-1.907983388	0.056393363	0.19219878	BPIFB9P	transcribed_unitary_pseudogene
ENSG00000125998.8	19.37108105	1.000395481	0.26570216	3.765101045	0.000166482	0.002944308	FAM83C	protein_coding
ENSG00000125999.11	13158.84746	-0.2267545	0.296177649	-0.765603012	0.443912552	0.659969884	BPIFB1	protein_coding
ENSG00000126001.16	3102.114576	-0.115685203	0.066678405	-1.734972562	0.082745665	0.245276366	CEP250	protein_coding
ENSG00000126003.7	5274.011578	-0.113084289	0.098915581	-1.143240408	0.25293879	0.479794264	PLAGL2	protein_coding
ENSG00000126005.17	2859.853565	-0.142148173	0.08334434	-1.705552801	0.088091373	0.254931451	MMP24OS	protein_coding
ENSG00000126010.6	54.32077031	-0.754662223	0.218258028	-3.457660782	0.000544887	0.007267995	GRPR	protein_coding
ENSG00000126012.12	7683.076996	0.063646237	0.055987362	1.136796496	0.255623368	0.482638627	KDM5C	protein_coding
ENSG00000126016.16	1938.746191	-0.235455501	0.123233314	-1.910648132	0.056049815	0.191376116	AMOT	protein_coding
ENSG00000126062.4	1744.686868	0.079734908	0.051833796	1.538280307	0.123980093	0.313442736	TMEM115	protein_coding
ENSG00000126067.12	11290.37178	-0.073943552	0.051517252	-1.435316308	0.151196983	0.353566457	PSMB2	protein_coding
ENSG00000126070.20	2130.988111	0.061807267	0.05524869	1.11871008	0.263263843	0.490938399	AGO3	protein_coding
ENSG00000126088.14	1720.99183	0.020192516	0.049568932	0.407362345	0.683741879	0.832941534	UROD	protein_coding
ENSG00000126091.21	149.2112857	-0.141329932	0.089258534	-1.583377253	0.113335504	0.296916774	ST3GAL3	protein_coding
ENSG00000126106.14	771.2757421	0.004224461	0.0694231	0.060850938	0.951477923	0.977271197	TMEM53	protein_coding
ENSG00000126107.15	5745.508799	-0.021840405	0.05376378	-0.406228977	0.684574364	0.833520455	HECTD3	protein_coding
ENSG00000126214.21	1276.501279	0.100416748	0.045442711	2.209743779	0.02712295	0.119303324	KLC1	protein_coding
ENSG00000126215.14	970.1552302	0.029238047	0.062829585	0.465354775	0.641677415	0.806134192	XRCC3	protein_coding

ENSG00000126216.15	2103.868404	0.094632347	0.054724988	1.729234674	0.083767103	0.247127415	TUBGCP3	protein_coding
ENSG00000126217.21	3170.596744	0.357493407	0.094992505	3.763385397	0.000167629	0.002962186	MCF2L	protein_coding
ENSG00000126218.12	425.5690148	0.159691921	0.192278327	0.83052481	0.406242127	0.627692556	F10	protein_coding
ENSG00000126226.22	2220.841533	0.039487422	0.052928728	0.746048945	0.455637849	0.669660345	PCID2	protein_coding
ENSG00000126231.15	20.23518286	0.04555204	0.155784567	0.292404063	0.769977702	0.883572892	PROZ	protein_coding
ENSG00000126233.2	20.15889835	0.334686433	0.376283602	0.889452612	0.37375988	0.59974172	SLURP1	protein_coding
ENSG00000126243.9	1781.268195	0.07559419	0.064048243	1.180269534	0.237893031	0.46318058	LRFN3	protein_coding
ENSG00000126246.10	54.51045974	-0.316249298	0.098022837	-3.22628182	0.001254098	0.013628387	IGFLR1	protein_coding
ENSG00000126247.11	21681.3249	-0.064838336	0.059006578	-1.098832338	0.271841204	0.500043231	CAPNS1	protein_coding
ENSG00000126249.8	345.9940937	-0.229709248	0.078263492	-2.935075373	0.003334667	0.027816764	PDCD2L	protein_coding
ENSG00000126251.6	1.729598012	-0.088787116	0.22057658	-0.402522862	0.687299267	0.834971847	GPR42	protein_coding
ENSG00000126254.12	4384.607884	0.026784428	0.068704343	0.389850584	0.696647037	0.840074935	RBM42	protein_coding
ENSG00000126259.20	60.86047436	-0.70964024	0.212734025	-3.335809777	0.000850513	0.010134761	KIRREL2	protein_coding
ENSG00000126261.13	6485.703997	-0.074577741	0.056062948	-1.330250082	0.183435888	0.39735036	UBA2	protein_coding
ENSG00000126262.5	140.6581551	0.194998229	0.168032855	1.160476795	0.245854736	0.472381474	FFAR2	protein_coding
ENSG00000126264.10	174.5121948	-0.373012866	0.133778511	-2.788286866	0.00529876	0.038774822	HCST	protein_coding
ENSG00000126266.3	3.293710738	-1.210048364	0.262449895	-4.610588103	4.01531E-06	0.000156788	FFAR1	protein_coding
ENSG00000126267.11	12079.81048	-0.236556912	0.074499263	-3.175291971	0.001496858	0.015501089	COX6B1	protein_coding
ENSG00000126337.13	8.445899659	-0.033345464	0.170900855	-0.195115843	0.84530226	0.924184398	KRT36	protein_coding
ENSG00000126351.13	5791.25156	-0.451678835	0.102042326	-4.426387091	9.58245E-06	0.000313259	THRA	protein_coding
ENSG00000126353.3	539.8186871	-0.734323178	0.168985376	-4.34548358	1.38969E-05	0.000419963	CCR7	protein_coding
ENSG00000126368.6	1811.009945	0.075850173	0.0907433	0.835876293	0.403224501	0.624990558	NR1D1	protein_coding
ENSG00000126391.14	3508.292316	0.1661185	0.059619085	2.786330925	0.005330842	0.038898543	FRMD8	protein_coding
ENSG00000126432.14	6303.621519	-0.026465959	0.069044831	-0.383315567	0.701485798	0.843017199	PRDX5	protein_coding
ENSG00000126453.9	1310.939263	-0.115074796	0.081504815	-1.411877262	0.157986104	0.36324076	BCL2L12	protein_coding
ENSG00000126456.16	2752.022753	-0.047776061	0.059053348	-0.80903222	0.418496613	0.638589079	IRF3	protein_coding
ENSG00000126457.22	6487.54826	-0.078971548	0.06394293	-1.23503174	0.216818692	0.438149836	PRMT1	protein_coding
ENSG00000126458.4	4594.255645	0.034295872	0.095185072	0.36030726	0.718617371	0.852939586	RRAS	protein_coding
ENSG00000126460.11	586.6187987	-0.020355948	0.080766371	-0.252034946	0.801014053	0.900817811	PRRG2	protein_coding
ENSG00000126461.15	7564.036666	0.133163326	0.061535515	2.164007657	0.030463762	0.128822838	SCAF1	protein_coding
ENSG00000126464.14	5377.334123	0.088965944	0.06225351	1.42909121	0.15297802	0.356373525	PRR12	protein_coding
ENSG00000126467.11	16.10725148	0.005956489	0.175028353	0.034031566	0.972851979	0.987237919	TSKS	protein_coding
ENSG00000126500.3	59.78800269	-0.154060047	0.152620946	-1.009429252	0.312768816	0.542334406	FLRT1	protein_coding
ENSG00000126522.17	2305.056629	-0.134106841	0.085072177	-1.576388971	0.114936189	0.299215861	ASL	protein_coding
ENSG00000126524.10	5378.439997	-0.214452264	0.068814844	-3.116366346	0.001830947	0.017935419	SBDS	protein_coding
ENSG00000126545.14	1.732556085	-0.909958372	0.474537964	-1.917567067	0.055165929	0.189504782	CSN1S1	protein_coding
ENSG00000126549.11	2.176739375	0.409263215	0.405172547	1.010096113	0.312449244	0.542000745	STATH	protein_coding
ENSG00000126550.10	3.482649795	-0.387000906	0.374331108	-1.033846499	0.30120793	0.530804673	HTN1	protein_coding
ENSG00000126561.17	2593.58721	-0.037306941	0.076340231	-0.488693049	0.625059025	0.795345494	STAT5A	protein_coding
ENSG00000126562.17	608.9365336	0.169594605	0.176158736	0.962737406	0.33567932	0.565182479	WNK4	protein_coding
ENSG00000126581.13	2781.324014	0.048670646	0.046573821	1.045021549	0.29601301	0.52575337	BECN1	protein_coding
ENSG00000126583.11	361.5792575	-0.511186583	0.199776718	-2.558789574	0.01050373	0.062393817	PRKCG	protein_coding

ENSG00000126602.11	3658.059191	-0.07427215	0.07306323	-1.016546212	0.309369353	0.538738343	TRAP1	protein_coding
ENSG00000126603.9	1641.738918	0.433574961	0.102220334	4.241572552	2.21959E-05	0.000605276	GLIS2	protein_coding
ENSG00000126653.18	2769.796372	-0.012113749	0.05657257	-0.214127604	0.83044755	0.916570636	NSRP1	protein_coding
ENSG00000126698.11	4201.036526	-0.091739581	0.042418459	-2.162727791	0.030562122	0.129126004	DNAJC8	protein_coding
ENSG00000126705.15	3117.918205	0.26384596	0.073498414	3.589818428	0.000330908	0.005001732	AHDC1	protein_coding
ENSG00000126709.15	20264.97717	0.141417994	0.146030452	0.968414411	0.332837445	0.562434363	IFI6	protein_coding
ENSG00000126733.21	17.63767248	-0.213989182	0.210995531	-1.014188218	0.310492949	0.539978869	DACH2	protein_coding
ENSG00000126746.17	2114.65209	0.054816102	0.048631333	1.127176626	0.259667811	0.486836648	ZNF384	protein_coding
ENSG00000126749.16	1874.387421	-0.157071216	0.059885187	-2.622872609	0.008719187	0.054891072	EMG1	protein_coding
ENSG00000126752.8	16.52230792	0.543193041	0.419512395	1.294820004	0.19538233	0.412451145	SSX1	protein_coding
ENSG00000126756.12	3146.976381	-0.278394671	0.071587957	-3.888847813	0.000100721	0.001994153	UXT	protein_coding
ENSG00000126759.14	27.36395764	-0.296208826	0.11266648	-2.629076783	0.008561703	0.054175153	CFP	protein_coding
ENSG00000126767.18	2837.855846	0.118115249	0.05608887	2.10585897	0.035216605	0.141362747	ELK1	protein_coding
ENSG00000126768.12	2716.536846	-0.10241816	0.0759402	-1.348668545	0.177443453	0.389701308	TIMM17B	protein_coding
ENSG00000126773.13	2432.853339	0.060918444	0.042236001	1.442334547	0.149208019	0.351214705	PCNX4	protein_coding
ENSG00000126775.9	1208.689846	0.024340764	0.048956772	0.497188906	0.619055844	0.791381029	ATG14	protein_coding
ENSG00000126777.18	11235.87218	0.108657007	0.052442603	2.071922463	0.038272673	0.149606051	KTN1	protein_coding
ENSG00000126778.12	256.4001703	0.301061873	0.173328906	1.736939786	0.0823978	0.244567651	SIX1	protein_coding
ENSG00000126785.13	1032.497791	-0.023354503	0.105315079	-0.221758396	0.824501968	0.91339275	RHOJ	protein_coding
ENSG00000126787.13	1542.636014	0.053142177	0.091135183	0.583113734	0.559816739	0.749420436	DLGAP5	protein_coding
ENSG00000126790.12	310.6890649	-0.115434639	0.066578454	-1.733813747	0.082951136	0.245668183	L3HYPDH	protein_coding
ENSG00000126803.9	1069.929501	-0.026764508	0.134305209	-0.199281235	0.842042757	0.922326142	HSPA2	protein_coding
ENSG00000126804.13	1847.999882	-0.015222411	0.044039179	-0.34565611	0.729601173	0.859269487	ZBTB1	protein_coding
ENSG00000126814.7	1053.677584	-0.029397618	0.046994697	-0.625551817	0.531608951	0.729199007	TRMT5	protein_coding
ENSG00000126821.8	1415.892712	0.007218118	0.079150638	0.091194686	0.927337897	0.965674498	SGPP1	protein_coding
ENSG00000126822.17	2692.407919	0.387163387	0.080121966	4.832175304	1.35049E-06	6.6273E-05	PLEKHG3	protein_coding
ENSG00000126838.10	26.79216386	-0.176539379	0.131162615	-1.345958056	0.178316044	0.390947788	PZP	protein_coding
ENSG00000126856.15	4.146237795	-0.233303002	0.211834804	-1.101344055	0.270746944	0.498936	PRDM7	protein_coding
ENSG00000126858.18	2155.806609	0.006200718	0.048471714	0.12792446	0.898208754	0.95160077	RHOT1	protein_coding
ENSG00000126860.12	374.2977905	-0.264254907	0.103029796	-2.564839661	0.010322349	0.061779565	EVI2A	protein_coding
ENSG00000126861.5	60.15562813	-0.031796141	0.093904835	-0.338599619	0.734911369	0.862446685	OMG	protein_coding
ENSG00000126870.16	1428.276467	-0.126371543	0.059526248	-2.122954954	0.033757627	0.137559015	DYNC2I1	protein_coding
ENSG00000126878.13	788.5025701	0.304087452	0.14789204	2.056144817	0.039768559	0.153525382	AIF1L	protein_coding
ENSG00000126882.13	677.6628879	-0.308495695	0.101598168	-3.036429714	0.002393979	0.021738368	FAM78A	protein_coding
ENSG00000126883.17	5393.433943	0.052004795	0.042199594	1.232352949	0.217817269	0.439293892	NUP214	protein_coding
ENSG00000126890.13	110.4272915	0.34133583	0.568843934	0.600051806	0.54847171	0.741637499	CTAG2	protein_coding
ENSG00000126895.15	59.86924992	0.228514915	0.142835585	1.599845831	0.109632789	0.29051189	AVPR2	protein_coding
ENSG00000126903.16	2452.623041	0.122721283	0.06223889	1.971778152	0.048634937	0.174841769	SLC10A3	protein_coding
ENSG00000126934.14	7373.350864	-0.023723674	0.056925395	-0.416750275	0.676861077	0.828783477	MAP2K2	protein_coding
ENSG00000126945.9	4040.302277	0.033703981	0.043800303	0.769491955	0.441601318	0.658086558	HNRNPH2	protein_coding
ENSG00000126947.13	437.2417985	0.092065537	0.119936142	0.767621298	0.442712206	0.659062236	ARMCX1	protein_coding
ENSG00000126950.8	132.6144688	-0.397557464	0.215735606	-1.842799487	0.065358292	0.210723131	TMEM35A	protein_coding

ENSG00000126952.17	1.404863415	0.193450919	0.301544355	0.641533878	0.521175878	0.721061727	NXF5	protein_coding
ENSG00000126953.8	556.6317696	-0.11174494	0.06213094	-1.798539338	0.072091581	0.223976842	TIMM8A	protein_coding
ENSG00000126970.16	598.3491978	-0.104573779	0.089261479	-1.171544324	0.241380052	0.467141206	ZC4H2	protein_coding
ENSG00000127022.15	41574.3732	0.042790018	0.053645115	0.797649858	0.425073709	0.644014722	CANX	protein_coding
ENSG00000127054.21	4481.234201	-0.051630122	0.046743922	-1.104531254	0.269362754	0.497445675	INTS11	protein_coding
ENSG00000127074.15	78.43580359	-0.99151285	0.172490885	-5.74820432	9.01962E-09	1.10407E-06	RGS13	protein_coding
ENSG00000127080.10	317.6597753	0.123887058	0.068531602	1.807736216	0.070647565	0.221069148	IPPK	protein_coding
ENSG00000127081.14	535.7289817	-0.003717218	0.056775734	-0.065471945	0.947798243	0.975463848	ZNF484	protein_coding
ENSG00000127083.7	420.8022234	0.173009931	0.200409356	0.863282708	0.387982049	0.612420537	OMD	protein_coding
ENSG00000127084.19	776.5210586	-0.263718834	0.097825626	-2.695805223	0.007021871	0.047122244	FGD3	protein_coding
ENSG00000127124.16	1095.833482	0.164058898	0.079038745	2.075676919	0.037923842	0.148693181	HIVEP3	protein_coding
ENSG00000127125.9	2687.54166	-0.064870243	0.04764423	-1.361555084	0.173338338	0.384346653	PPCS	protein_coding
ENSG00000127129.10	75.82397534	-0.060928706	0.21695471	-0.280836062	0.778836143	0.888185455	EDN2	protein_coding
ENSG00000127152.18	1321.728952	-0.326350472	0.087633226	-3.724049502	0.000196052	0.003335954	BCL11B	protein_coding
ENSG00000127184.13	11681.18483	-0.182897223	0.067580123	-2.706375999	0.006802198	0.046113781	COX7C	protein_coding
ENSG00000127191.18	1670.895692	-0.000437629	0.057359601	-0.007629575	0.993912539	0.997296993	TRAF2	protein_coding
ENSG00000127220.6	566.8800524	0.156835985	0.092288776	1.699404751	0.089242947	0.256944273	ABHD8	protein_coding
ENSG00000127241.18	156.0487019	-0.053724172	0.175104068	-0.306812816	0.758985853	0.876647148	MASP1	protein_coding
ENSG00000127249.15	493.2053884	-0.060079981	0.217171714	-0.276647358	0.782050897	0.889755938	ATP13A4	protein_coding
ENSG00000127252.7	39.50257943	-0.04379265	0.21723816	-0.201588202	0.840238669	0.921597797	PLAAT1	protein_coding
ENSG00000127311.10	381.5458409	0.073316892	0.080495494	0.910819836	0.362390309	0.589888793	HELB	protein_coding
ENSG00000127314.18	4876.806528	0.143218856	0.067503405	2.121653805	0.033866821	0.137875041	RAP1B	protein_coding
ENSG00000127318.11	4.327882943	-0.576834972	0.274138553	-2.104173114	0.035363349	0.141716816	IL22	protein_coding
ENSG00000127324.9	31239.10207	0.014771942	0.146916476	0.100546533	0.919910442	0.962213821	TSPAN8	protein_coding
ENSG00000127325.19	21.13277611	0.571412605	0.219119688	2.607764787	0.009113555	0.056613172	BEST3	protein_coding
ENSG00000127328.22	2441.734927	0.155422027	0.087316613	1.779982318	0.075078854	0.230434656	RAB3IP	protein_coding
ENSG00000127329.16	2196.492867	-0.05132263	0.082632114	-0.621097865	0.534535234	0.731531998	PTPRB	protein_coding
ENSG00000127334.11	4765.709727	0.058529098	0.063526521	0.921333272	0.356876455	0.584696094	DYRK2	protein_coding
ENSG00000127337.7	1370.994228	0.003636808	0.089528145	0.040621948	0.967597287	0.984830373	YEATS4	protein_coding
ENSG00000127362.2	3.663174335	-0.047244114	0.153067811	-0.308648263	0.757589104	0.875809113	TAS2R3	protein_coding
ENSG00000127364.4	49.59873138	-0.231837007	0.107779935	-2.151021965	0.031474466	0.131682575	TAS2R4	protein_coding
ENSG00000127366.5	25.44153969	-0.141644095	0.106786261	-1.326426206	0.184698563	0.399038636	TAS2R5	protein_coding
ENSG00000127377.10	11.19632512	0.665238598	0.142989797	4.652350122	3.28173E-06	0.000133031	CRYGN	protein_coding
ENSG00000127399.15	883.3784313	-0.189593816	0.084596572	-2.241152474	0.0250162	0.113225068	LRRC61	protein_coding
ENSG00000127412.7	7.937284671	-0.41841184	0.187238109	-2.234651072	0.025440265	0.114325094	TRPV5	protein_coding
ENSG00000127415.13	1216.139961	0.215954381	0.079291851	2.723538137	0.006458676	0.044488682	IDUA	protein_coding
ENSG00000127418.15	4345.629019	0.515139117	0.119637643	4.30582802	1.66362E-05	0.000481379	FGFRL1	protein_coding
ENSG00000127419.17	1442.61574	-0.012869273	0.060328347	-0.213320499	0.831076985	0.916763917	TMEM175	protein_coding
ENSG00000127423.11	285.8031265	0.002865894	0.094889828	0.030202327	0.975905693	0.989023986	AUNIP	protein_coding
ENSG00000127445.14	2361.763235	-0.039885712	0.056209337	-0.709592283	0.477957006	0.687617447	PIN1	protein_coding
ENSG00000127452.9	1223.360796	-0.002561065	0.042308723	-0.06053279	0.951731301	0.977282689	FBXL12	protein_coding
ENSG00000127463.15	4016.118189	0.081361159	0.049460092	1.644986004	0.099972697	0.275397322	EMC1	protein_coding

ENSG00000127472.11	88.17412298	0.184712688	0.161275545	1.145323601	0.252075133	0.478822984	PLA2G5	protein_coding
ENSG00000127481.15	13640.73633	0.086992972	0.052860072	1.645721774	0.099821054	0.275083923	UBR4	protein_coding
ENSG00000127483.19	10741.81568	-0.048640006	0.042553004	-1.143045174	0.253019836	0.47985043	HP1BP3	protein_coding
ENSG00000127507.18	664.8020167	0.111497855	0.116380605	0.958044984	0.338040076	0.567900551	ADGRE2	protein_coding
ENSG00000127511.10	3332.646914	0.110165865	0.04413906	2.495881544	0.012564455	0.070794684	SIN3B	protein_coding
ENSG00000127515.3	0.818183785	-0.230543078	0.490288962	-0.470218782	0.638198717		OR7A10	protein_coding
ENSG00000127526.15	4360.665428	0.019654934	0.040883549	0.480754111	0.630691268	0.799321749	SLC35E1	protein_coding
ENSG00000127527.14	2420.57644	-0.086771781	0.036647255	-2.36775661	0.017896305	0.090053957	EPS15L1	protein_coding
ENSG00000127528.6	5845.63966	0.071464314	0.120976805	0.590727403	0.554703084	0.745759303	KLF2	protein_coding
ENSG00000127529.7	1.031340972	0.112910574	0.423931017	0.266341856	0.78997594	0.89452192	OR7C2	protein_coding
ENSG00000127530.4	22.59823597	-0.177095677	0.14668781	-1.207296479	0.227317986	0.450693111	OR7C1	protein_coding
ENSG00000127533.4	802.9002718	-0.068405833	0.123138639	-0.555518829	0.578539835	0.762837919	F2RL3	protein_coding
ENSG00000127540.12	1722.934265	-0.125289848	0.067407382	-1.85869626	0.063070204	0.206134097	UQCR11	protein_coding
ENSG00000127554.13	568.8265668	-0.12826338	0.070898398	-1.809115333	0.070433087	0.220667385	GFER	protein_coding
ENSG00000127561.15	288.4697967	0.173946786	0.151282954	1.149810872	0.250221776	0.477034725	SYNGR3	protein_coding
ENSG00000127564.17	1499.245014	-0.034559679	0.102923913	-0.335778912	0.737037594	0.863755573	PKMYT1	protein_coding
ENSG00000127578.7	88.25729717	0.126975811	0.132594272	0.957626667	0.338251047	0.568035989	WFIKKN1	protein_coding
ENSG00000127580.17	865.7893757	-0.012530006	0.0582818	-0.214990031	0.829775091	0.916289069	WDR24	protein_coding
ENSG00000127585.12	586.70084	0.439618702	0.160348263	2.741649288	0.006113157	0.04286982	FBXL16	protein_coding
ENSG00000127586.17	1253.030199	-0.044586915	0.085563789	-0.521095618	0.602300163	0.780354232	CHTF18	protein_coding
ENSG00000127588.5	8.126164991	0.072567707	0.191848911	0.378254461	0.705241571	0.845502144	GNG13	protein_coding
ENSG00000127589.4	166.2362874	0.141676076	0.076783054	1.845147702	0.065016056	0.209968096	TUBBP1	transcribed_processed_pseudogene
ENSG00000127603.30	16051.97711	0.067591891	0.081505096	0.8292965	0.406936651	0.628135196	MACF1	protein_coding
ENSG00000127616.19	10379.32071	0.108741186	0.057691867	1.884861632	0.059448521	0.198688765	SMARCA4	protein_coding
ENSG00000127663.15	2696.145395	0.120571729	0.053394389	2.258134821	0.023937255	0.109636105	KDM4B	protein_coding
ENSG00000127666.10	3034.087046	0.209641836	0.072942834	2.874056621	0.004052363	0.032083907	TICAM1	protein_coding
ENSG00000127720.8	214.1721159	-0.172174045	0.050279846	-3.424315274	0.000616351	0.007953425	METTL25	protein_coding
ENSG00000127743.6	18.74859364	-0.087828267	0.124606621	-0.704844309	0.480907131	0.689857464	IL17B	protein_coding
ENSG00000127774.7	42.34589775	0.045023053	0.105121598	0.428294985	0.668436369	0.822977105	EMC6	protein_coding
ENSG00000127780.3	1.124483863	-0.282807061	0.433276588	-0.652717154	0.513938641	0.716004482	OR1E2	protein_coding
ENSG00000127804.13	2093.569686	-0.03580162	0.041230498	-0.868328586	0.385214477	0.609827242	METTL16	protein_coding
ENSG00000127824.14	5023.102463	0.081216762	0.090519697	0.8972275	0.369597534	0.596273805	TUBA4A	protein_coding
ENSG00000127831.11	9691.587681	-0.271205893	0.14903431	-1.819754754	0.068796361	0.217494125	VIL1	protein_coding
ENSG00000127837.10	5649.905517	-0.087815234	0.056216641	-1.562086114	0.118267704	0.304559256	AAMP	protein_coding
ENSG00000127838.14	7768.18815	-0.216317481	0.080878803	-2.674588082	0.007482112	0.049311085	PNKD	protein_coding
ENSG00000127863.16	1299.286736	-0.187144795	0.183644488	-1.019060231	0.308174373	0.537625675	TNFRSF19	protein_coding
ENSG00000127870.17	3414.651249	0.059706588	0.057482686	1.038688195	0.298949773	0.528832923	RNF6	protein_coding
ENSG00000127884.5	7167.901879	-0.188445428	0.066831627	-2.819704326	0.004806792	0.036212229	ECHS1	protein_coding
ENSG00000127903.14	60.93116086	-0.164272568	0.154900371	-1.060504675	0.288915064	0.518638572	ZNF835	protein_coding
ENSG00000127914.18	8807.004409	0.061694683	0.08313044	0.742143114	0.458000631	0.671476415	AKAP9	protein_coding
ENSG00000127920.6	1290.622111	0.00836005	0.09805522	0.085258591	0.932055811	0.967902663	GNG11	protein_coding
ENSG00000127922.9	6342.157443	-0.074073706	0.086712787	-0.854242016	0.392970907	0.617009917	SEM1	protein_coding

ENSG00000127928.13	80.53793566	0.89926878	0.293901319	3.059764357	0.002215112	0.020572876	GNGT1	protein_coding
ENSG00000127946.17	3300.231363	0.179478019	0.075158486	2.387994059	0.016940616	0.087086549	HIP1	protein_coding
ENSG00000127947.16	6257.018626	0.058963796	0.059006767	0.999271758	0.317663063	0.546908588	PTPN12	protein_coding
ENSG00000127948.16	7447.632489	-0.125466902	0.080810537	-1.552605718	0.120517359	0.308375171	POR	protein_coding
ENSG00000127951.8	3722.147471	-0.39549497	0.145660836	-2.715177133	0.006624033	0.045286396	FGL2	protein_coding
ENSG00000127952.17	1467.524883	-0.123530077	0.073569447	-1.679094807	0.093133568	0.263751748	STYXL1	protein_coding
ENSG00000127954.13	1042.053498	0.693137502	0.151962166	4.561250477	5.08499E-06	0.000188747	STEAP4	protein_coding
ENSG00000127955.17	1174.333119	0.332503926	0.108396126	3.067489028	0.002158654	0.020220907	GNAI1	protein_coding
ENSG00000127957.18	224.5273053	-0.037724021	0.063972244	-0.589693571	0.555396106	0.746368647	PMS2P3	transcribed_unprocessed_pseudogene
ENSG00000127980.16	1494.711958	0.102885233	0.080629825	1.276019552	0.201948608	0.420926698	PEX1	protein_coding
ENSG00000127989.14	678.9851487	-0.200439654	0.112796226	-1.777006746	0.07556713	0.231346693	MTERF1	protein_coding
ENSG00000127990.19	1128.3338	0.403964365	0.122489505	3.297950836	0.000973932	0.011236703	SGCE	protein_coding
ENSG00000127993.16	805.6054949	0.204145244	0.074254586	2.749261076	0.005972979	0.042186251	RBM48	protein_coding
ENSG00000127995.17	1136.914346	0.059336497	0.065105168	0.911394566	0.362087516	0.589659094	CASD1	protein_coding
ENSG00000128000.17	980.3104148	-0.011296889	0.061116686	-0.184841313	0.853353503	0.928371361	ZNF780B	protein_coding
ENSG00000128011.5	394.7059882	-0.031359135	0.156210491	-0.200749225	0.840894667	0.921927096	LRFN1	protein_coding
ENSG00000128016.7	21481.3133	0.208483309	0.102151975	2.04091315	0.041259461	0.157054814	ZFP36	protein_coding
ENSG00000128039.12	2613.183784	0.443199847	0.103166958	4.295947602	1.73949E-05	0.000497169	SRD5A3	protein_coding
ENSG00000128040.11	12.23608113	-0.570898222	0.209252895	-2.728269174	0.006366763	0.044060415	SPINK2	protein_coding
ENSG00000128045.7	14.91281443	-0.261957067	0.174717413	-1.4993186	0.133790999	0.328536593	RASL11B	protein_coding
ENSG00000128050.9	8687.677769	0.016355355	0.071256223	0.229528795	0.818457943	0.910426082	PAICS	protein_coding
ENSG00000128052.10	2063.043132	-0.029532202	0.08918699	-0.331126795	0.740548712	0.865890988	KDR	protein_coding
ENSG00000128059.8	1263.27724	0.041133426	0.074292695	0.553667164	0.579806651	0.763768763	PPAT	protein_coding
ENSG00000128159.12	2521.631276	-0.064459936	0.056674187	-1.137377346	0.255380573	0.482383767	TUBGCP6	protein_coding
ENSG00000128165.9	1036.528046	0.087680754	0.124124872	0.706391498	0.479944707	0.689157457	ADM2	protein_coding
ENSG00000128185.10	1606.136825	-0.10685036	0.067326334	-1.587051523	0.112500976	0.295524445	DGCR6L	protein_coding
ENSG00000128191.16	1600.813826	-0.018920029	0.041623438	-0.454552293	0.649431363	0.810826304	DGCR8	protein_coding
ENSG00000128203.7	635.8688987	0.022811077	0.116613876	0.195612031	0.844913843	0.923911835	ASPHD2	protein_coding
ENSG00000128218.8	187.1855699	-1.304916141	0.209800543	-6.219793926	4.97808E-10	9.99677E-08	VPREB3	protein_coding
ENSG00000128228.5	2055.63773	-0.180979666	0.080597965	-2.245461976	0.024738491	0.112328947	SDF2L1	protein_coding
ENSG00000128242.13	754.4034039	-0.18219961	0.13522518	-1.34737931	0.177858101	0.390336546	GAL3ST1	protein_coding
ENSG00000128245.15	9924.701226	-0.11513667	0.052223647	-2.204684614	0.027476234	0.120274418	YWHAH	protein_coding
ENSG00000128250.5	0.552129176	-0.630276576	0.455740643	-1.382972062	0.166673432		RFPL1	protein_coding
ENSG00000128253.15	9.186038011	0.171975568	0.183772345	0.935807661	0.349372226	0.578503007	RFPL2	protein_coding
ENSG00000128254.16	39.45940739	-0.105964004	0.102056095	-1.038291781	0.299134234	0.528990037	C22orf24	lncRNA
ENSG00000128262.8	43.01378125	0.077254559	0.128000839	0.60354729	0.546144666	0.74019053	POM121L9P	transcribed_unprocessed_pseudogene
ENSG00000128266.9	551.957173	0.069502215	0.142656629	0.487199337	0.626117075	0.796133868	GNAZ	protein_coding
ENSG00000128268.12	1712.54663	-0.045349388	0.123868424	-0.366109352	0.714283479	0.850722814	MGAT3	protein_coding
ENSG00000128271.22	18.62311197	-0.439673881	0.136670814	-3.217028339	0.001295258	0.013958941	ADORA2A	protein_coding
ENSG00000128272.15	13861.8264	-0.098630257	0.049293926	-2.000860253	0.045407452	0.167178146	ATF4	protein_coding
ENSG00000128274.17	1029.197343	0.410324101	0.10786252	3.804139776	0.000142298	0.002607616	A4GALT	protein_coding
ENSG00000128276.10	2.138031347	-0.086040845	0.194055475	-0.443382722	0.657488931	0.81609306	RFPL3	protein_coding

ENSG00000128283.7	19752.02766	-0.03757777	0.099263518	-0.378565771	0.705010344	0.845451369	CDC42EP1	protein_coding
ENSG00000128284.19	1952.362155	-0.260627143	0.105717024	-2.465328036	0.013688786	0.075289861	APOL3	protein_coding
ENSG00000128285.4	18.05313895	-0.161805148	0.154947717	-1.044256418	0.296366771	0.526167235	MCHR1	protein_coding
ENSG00000128294.16	1894.296357	0.041498311	0.059517639	0.697243916	0.485650159	0.693424502	TPST2	protein_coding
ENSG00000128298.17	3949.616956	-0.031228434	0.109411199	-0.285422652	0.775320355	0.886193708	BAIAP2L2	protein_coding
ENSG00000128309.16	5536.428681	-0.181189205	0.079953795	-2.26617393	0.023440736	0.108187236	MPST	protein_coding
ENSG00000128310.3	7.194010414	0.536078036	0.194794569	2.752017366	0.005922938	0.041934016	GALR3	protein_coding
ENSG00000128311.14	4453.448465	-0.151521897	0.098240696	-1.54235366	0.122987676	0.312103072	TST	protein_coding
ENSG00000128313.2	3.377255221	-0.03110731	0.230562808	-0.134919027	0.892675895	0.948806983	APOL5	protein_coding
ENSG00000128322.7	37.1097248	-1.151510841	0.21326635	-5.399402391	6.68633E-08	5.93422E-06	IGLL1	protein_coding
ENSG00000128335.14	3543.982038	0.145428449	0.088355753	1.645942056	0.099775689	0.275028625	APOL2	protein_coding
ENSG00000128340.15	4168.547035	-0.392476177	0.106662388	-3.679611767	0.000233589	0.003831058	RAC2	protein_coding
ENSG00000128342.5	1703.788194	0.900310578	0.114821151	7.840982015	4.47036E-15	4.53035E-12	LIF	protein_coding
ENSG00000128346.11	56.52456472	-0.124974188	0.125877414	-0.992824558	0.32079546	0.550539688	C22orf23	protein_coding
ENSG00000128383.13	108.6415926	0.577416204	0.161957721	3.565228006	0.00036354	0.00537415	APOBEC3A	protein_coding
ENSG00000128394.17	626.818212	-0.22602269	0.093027747	-2.429626609	0.015114385	0.080459266	APOBEC3F	protein_coding
ENSG00000128408.9	143.9479715	0.106441758	0.142120748	0.748952982	0.453885545	0.668321352	RIBC2	protein_coding
ENSG00000128422.17	8120.413151	1.92781777	0.237554125	8.115278034	4.84674E-16	6.81311E-13	KRT17	protein_coding
ENSG00000128438.10	52.24887507	-1.064295591	0.21705157	-4.903422677	9.4181E-07	4.9507E-05	TBC1D27P	transcribed_unprocessed_pseudogene
ENSG00000128463.13	1715.651575	0.011037046	0.048763015	0.226340522	0.820936584	0.911931925	EMC4	protein_coding
ENSG00000128482.16	30.7932005	-0.08278402	0.128420561	-0.644632128	0.519165609	0.719811637	RNF112	protein_coding
ENSG00000128487.17	2013.400527	0.321526924	0.074238439	4.331003286	1.48431E-05	0.000443027	SPECC1	protein_coding
ENSG00000128510.12	38.9910912	0.660712023	0.21172998	3.120540714	0.001805193	0.017757317	CPA4	protein_coding
ENSG00000128512.23	1092.254247	0.00769471	0.082967931	0.09274318	0.926107592	0.965062665	DOCK4	protein_coding
ENSG00000128513.16	1401.309762	-0.086948272	0.06014275	-1.445698319	0.148261836	0.349800001	POT1	protein_coding
ENSG00000128519.3	0.575593818	0.333224857	0.580243359	0.574284655	0.565775191		TAS2R16	protein_coding
ENSG00000128524.5	3492.696915	-0.156017456	0.072355266	-2.156269548	0.031062624	0.130581849	ATP6V1F	protein_coding
ENSG00000128534.8	2737.979662	-0.23406061	0.066438094	-3.522988021	0.000426711	0.006039432	LSM8	protein_coding
ENSG00000128536.16	328.0053093	-0.359251389	0.099108869	-3.624815729	0.000289168	0.004523773	CDHR3	protein_coding
ENSG00000128563.13	1057.270255	-0.151222578	0.05083881	-2.974549888	0.002934187	0.025234475	PRKRIP1	protein_coding
ENSG00000128564.7	227.3804033	0.078335434	0.18815736	0.416329365	0.677169008	0.828988116	VGF	protein_coding
ENSG00000128567.17	7294.392601	0.353277655	0.078640099	4.492334794	7.04466E-06	0.000246376	PODXL	protein_coding
ENSG00000128573.26	692.2545528	-0.226510067	0.190821879	-1.187023564	0.235218326	0.459944987	FOXP2	protein_coding
ENSG00000128578.10	640.3316423	0.527754859	0.12354317	4.271825441	1.93879E-05	0.000543323	STRIP2	protein_coding
ENSG00000128581.17	1020.508106	-0.081515083	0.069943971	-1.165434011	0.243843357	0.469863452	IFT22	protein_coding
ENSG00000128585.18	2939.931603	-0.061593304	0.053090633	-1.160153889	0.245986157	0.472467329	MKLN1	protein_coding
ENSG00000128590.5	1762.864374	0.00728533	0.065719988	0.110854094	0.911732049	0.958412902	DNAJB9	protein_coding
ENSG00000128591.16	13224.31399	-0.908238417	0.219405398	-4.139544541	3.47996E-05	0.000859673	FLNC	protein_coding
ENSG00000128594.8	183.831028	-0.013424574	0.123278196	-0.10889658	0.913284519	0.959015868	LRRC4	protein_coding
ENSG00000128595.17	12920.01734	0.259303132	0.063347898	4.093318643	4.25243E-05	0.001013258	CALU	protein_coding
ENSG00000128596.17	403.1833931	-0.243410293	0.17613828	-1.381927275	0.166994032	0.375786158	CCDC136	protein_coding
ENSG00000128602.11	917.154077	0.266685957	0.129192331	2.064255331	0.038993514	0.151532051	SMO	protein_coding

ENSG00000128604.20	859.3623337	0.093757607	0.086623259	1.082360657	0.279092303	0.507871785	IRF5	protein_coding
ENSG00000128606.13	182.5840527	0.578923619	0.131683814	4.396315693	1.10104E-05	0.00035073	LRRC17	protein_coding
ENSG00000128607.14	2969.953259	0.002904688	0.050244263	0.057811327	0.953898916	0.978464545	KLHDC10	protein_coding
ENSG00000128609.16	4298.808778	-0.165145919	0.056188257	-2.939153651	0.003291099	0.027542963	NDUFA5	protein_coding
ENSG00000128610.12	176.4238549	0.060110037	0.221400284	0.271499368	0.786006986	0.891719206	FEZF1	protein_coding
ENSG00000128617.2	6.678602484	-0.361896498	0.192506461	-1.879918708	0.060119158	0.20006205	OPN1SW	protein_coding
ENSG00000128626.12	2141.842974	-0.05679875	0.077327652	-0.734520559	0.462631535	0.67504083	MRPS12	protein_coding
ENSG00000128641.19	5269.769918	0.213955922	0.078125517	2.738617698	0.006169807	0.043100623	MYO1B	protein_coding
ENSG00000128645.15	21.55509728	0.301503225	0.168031304	1.794327709	0.072760878	0.225451509	HOXD1	protein_coding
ENSG00000128652.11	48.06876646	0.08131562	0.135726815	0.599112416	0.549097923	0.74205694	HOXD3	protein_coding
ENSG00000128654.14	1597.715358	-0.078602964	0.052921343	-1.485279074	0.137469913	0.333893336	MTX2	protein_coding
ENSG00000128655.18	171.6708955	0.504453761	0.157602714	3.20079361	0.001370497	0.014616283	PDE11A	protein_coding
ENSG00000128656.15	824.5423769	0.192712589	0.091729937	2.100869087	0.03565246	0.142403545	CHN1	protein_coding
ENSG00000128683.14	332.3885102	-0.326837502	0.195743898	-1.669720003	0.094974774	0.266549606	GAD1	protein_coding
ENSG00000128692.8	120.2158889	-0.170312468	0.077556917	-2.195967468	0.028094272	0.121950801	EIF2S2P4	processed_pseudogene
ENSG00000128694.12	341.230516	-0.20093071	0.054633642	-3.677783569	0.000235269	0.003856631	OSGEPL1	protein_coding
ENSG00000128699.14	1805.779345	-0.082880551	0.045639843	-1.815969245	0.06937509	0.21854683	ORMDL1	protein_coding
ENSG00000128708.13	2696.30013	-0.054651213	0.047278912	-1.155932131	0.247708919	0.474229093	HAT1	protein_coding
ENSG00000128709.13	128.9964218	0.328576103	0.12337471	2.663237081	0.007739287	0.050562952	HOXD9	protein_coding
ENSG00000128710.6	79.10605761	0.849954137	0.289082628	2.940177149	0.003280246	0.027471866	HOXD10	protein_coding
ENSG00000128713.14	14.11209911	0.930414154	0.401992724	2.314504959	0.02064004	0.099253035	HOXD11	protein_coding
ENSG00000128714.6	73.43052431	0.469571405	0.44049381	1.066011359	0.286418507	0.515903744	HOXD13	protein_coding
ENSG00000128731.18	5076.233077	0.08465953	0.057641713	1.468719886	0.141908781	0.340282781	HERC2	protein_coding
ENSG00000128739.23	101.0840342	-0.319225603	0.119754072	-2.665676398	0.007683361	0.050303159	SNRPN	protein_coding
ENSG00000128789.21	3944.865814	-0.072069933	0.055860015	-1.290188209	0.196985319	0.414222627	PSMG2	protein_coding
ENSG00000128791.12	4025.972404	0.236077464	0.071475462	3.302916219	0.00095685	0.011086583	TWSG1	protein_coding
ENSG00000128805.15	197.7539061	0.320989238	0.103479928	3.101946848	0.001922525	0.018588387	ARHGAP22	protein_coding
ENSG00000128815.19	1263.9007	-0.664839072	0.153540802	-4.330048195	1.49077E-05	0.000444344	WDFY4	protein_coding
ENSG00000128829.12	3307.882026	-0.023263741	0.044830129	-0.518930944	0.603808898	0.781217314	EIF2AK4	protein_coding
ENSG00000128833.13	3901.643334	0.126743124	0.083341699	1.520764816	0.128318867	0.320022392	MYO5C	protein_coding
ENSG00000128849.11	902.9201866	-0.005773313	0.147407078	-0.03916578	0.968758216	0.985539321	CGNL1	protein_coding
ENSG00000128872.10	893.1681162	0.153851082	0.09763089	1.575844314	0.115061687	0.299381606	TMOD2	protein_coding
ENSG00000128881.17	779.8244634	-0.041909982	0.070846386	-0.591561326	0.554144378	0.745394144	TTBK2	protein_coding
ENSG00000128886.12	27.85741499	-0.260815619	0.131064963	-1.989972088	0.04659401	0.170024048	ELL3	protein_coding
ENSG00000128891.16	564.0512183	-0.108182139	0.050605902	-2.137737608	0.032538045	0.134483826	CCDC32	protein_coding
ENSG00000128908.17	2884.468426	-0.025065501	0.038445852	-0.651968927	0.514421218	0.716310382	INO80	protein_coding
ENSG00000128915.12	2438.275468	0.045255138	0.046635502	0.970401013	0.331846644	0.561369612	ICE2	protein_coding
ENSG00000128917.8	1206.705857	0.170459936	0.083443914	2.042808501	0.041071399	0.156525758	DLL4	protein_coding
ENSG00000128918.15	110.4973826	1.451425291	0.173414588	8.369683921	5.7773E-17	1.04899E-13	ALDH1A2	protein_coding
ENSG00000128923.11	2244.533777	0.220133765	0.063703142	3.455618659	0.000549031	0.007305388	MINDY2	protein_coding
ENSG00000128928.10	4017.837444	-0.087032695	0.056647629	-1.53638726	0.124443429	0.314190041	IVD	protein_coding
ENSG00000128944.13	1258.388726	-0.005962285	0.073949381	-0.080626568	0.935738937	0.969720852	KNSTRN	protein_coding

ENSG00000128951.14	2424.572753	-0.186215753	0.063386472	-2.937783847	0.003305674	0.027633102	DUT	protein_coding
ENSG00000128965.13	644.4832982	0.124418817	0.125313481	0.992860592	0.320777897	0.550539525	CHAC1	protein_coding
ENSG00000128973.13	1898.815788	-0.10529775	0.06579956	-1.600280457	0.10953638	0.290327039	CLN6	protein_coding
ENSG00000128989.11	11346.68899	0.032959538	0.047538991	0.693315891	0.488111333	0.695543885	ARPP19	protein_coding
ENSG00000129003.19	6239.741634	-0.15150126	0.061564164	-2.460867655	0.013860148	0.075915495	VPS13C	protein_coding
ENSG00000129007.14	22.56795483	-0.091292607	0.105275394	-0.867178959	0.385843963	0.610438657	CALML4	protein_coding
ENSG00000129009.13	5818.658975	0.699801373	0.142832037	4.899470638	9.60952E-07	5.04523E-05	ISLR	protein_coding
ENSG00000129028.9	264.9137876	0.192397568	0.076152951	2.52646241	0.011521772	0.066686711	THAP10	protein_coding
ENSG00000129038.16	2359.305636	0.402915683	0.101265375	3.978809973	6.92611E-05	0.001494893	LOXL1	protein_coding
ENSG00000129048.7	381.8833638	-0.377334829	0.153811936	-2.453222025	0.014158292	0.076979113	ACKR4	protein_coding
ENSG00000129055.13	3544.292545	-0.018905415	0.056487555	-0.334682834	0.737864352	0.864236389	ANAPC13	protein_coding
ENSG00000129071.10	2055.47503	-0.13971892	0.048254518	-2.895457787	0.003786059	0.030480946	MBD4	protein_coding
ENSG00000129083.13	8381.631224	0.081815999	0.048078809	1.701706011	0.088810493	0.256225377	COPB1	protein_coding
ENSG00000129084.18	1570.589951	-0.232145129	0.071289107	-3.256389908	0.001128387	0.012601669	PSMA1	protein_coding
ENSG00000129103.19	7131.729632	-0.153083351	0.068854435	-2.223289609	0.026196278	0.116493323	SUMF2	protein_coding
ENSG00000129116.19	15505.97844	-0.13559638	0.127000797	-1.067681327	0.285664284	0.514992243	PALLD	protein_coding
ENSG00000129128.13	7783.497475	0.022590494	0.052139625	0.433269211	0.664819203	0.820687414	SPCS3	protein_coding
ENSG00000129151.9	43.32825767	0.032584803	0.185751578	0.175421405	0.860748523	0.93235989	BBOX1	protein_coding
ENSG00000129152.4	1.127193166	0.071938743	0.307396296	0.234026056	0.814964751	0.908674333	MYOD1	protein_coding
ENSG00000129158.11	442.6407847	-0.082982967	0.055256592	-1.501774988	0.13315523	0.327418207	SERGEF	protein_coding
ENSG00000129159.9	35.8662995	0.118834287	0.13493753	0.880661496	0.378501063	0.604217921	KCNC1	protein_coding
ENSG00000129167.9	117.3797001	-0.819529115	0.186583409	-4.392293616	1.12161E-05	0.000355983	TPH1	protein_coding
ENSG00000129170.10	3.5808618	0.081647924	0.207998819	0.392540325	0.694659023	0.838954499	CSRP3	protein_coding
ENSG00000129173.13	1204.076613	-0.127042603	0.093946134	-1.352291975	0.176281931	0.388344254	E2F8	protein_coding
ENSG00000129187.14	3791.217901	-0.041819478	0.051569446	-0.81093518	0.417402896	0.637675696	DCTD	protein_coding
ENSG00000129194.8	160.9722448	0.678494631	0.162645281	4.171621991	3.02439E-05	0.000768477	SOX15	protein_coding
ENSG00000129195.16	479.70719	0.239063903	0.104234248	2.293525476	0.02181777	0.102984508	PIMREG	protein_coding
ENSG00000129197.14	881.6144038	-0.045687683	0.047603118	-0.959762416	0.337174802	0.56690587	RPAIN	protein_coding
ENSG00000129204.17	56.16322936	0.112703812	0.118819543	0.948529247	0.342860091	0.572686349	USP6	protein_coding
ENSG00000129214.15	27.97243914	-0.04119814	0.147683054	-0.27896322	0.780273051	0.88874039	SHBG	protein_coding
ENSG00000129219.14	1789.84214	0.209704229	0.062608151	3.349471674	0.000809658	0.009776249	PLD2	protein_coding
ENSG00000129221.16	5.761809172	-0.490831383	0.233580912	-2.101333451	0.035611705	0.142303284	AIPL1	protein_coding
ENSG00000129226.14	8.814105383	-0.088913493	0.126419244	-0.703322457	0.481854818	0.690629668	CD68	protein_coding
ENSG00000129235.11	2618.494891	-0.072538324	0.076419743	-0.949209217	0.342514214	0.572343007	TXNDC17	protein_coding
ENSG00000129244.9	258.8602116	-0.076126539	0.143739381	-0.529615044	0.596378862	0.775888988	ATP1B2	protein_coding
ENSG00000129245.12	1808.421318	0.019463867	0.048010006	0.405412719	0.685174163	0.833791757	FXR2	protein_coding
ENSG00000129250.12	12181.39163	-0.043330696	0.060467596	-0.716593664	0.473624855	0.684364705	KIF1C	protein_coding
ENSG00000129255.16	2951.739315	-0.12205016	0.067125312	-1.818243474	0.069026928	0.217864172	MPDU1	protein_coding
ENSG00000129292.21	3755.928601	-0.005609397	0.051768987	-0.10835439	0.913714579	0.959213207	PHF20L1	protein_coding
ENSG00000129295.9	243.3270104	0.028493667	0.138999974	0.204990451	0.837579586	0.92008445	LRRC6	protein_coding
ENSG00000129315.11	2225.771716	0.084161886	0.051821369	1.624076853	0.104359407	0.282551718	CCNT1	protein_coding
ENSG00000129317.15	2161.495162	-0.081918936	0.055423238	-1.478061156	0.13939141	0.336561363	PUS7L	protein_coding

ENSG00000129347.21	1594.294601	0.074844397	0.046634155	1.604926631	0.108509952	0.288889185	KRI1	protein_coding
ENSG00000129351.17	15292.21074	0.03066708	0.042967213	0.713732118	0.475392842	0.685580762	ILF3	protein_coding
ENSG00000129353.15	11810.81527	0.13442929	0.066714848	2.014983072	0.043906423	0.163628683	SLC44A2	protein_coding
ENSG00000129354.12	5612.104729	0.038051117	0.083835205	0.453879932	0.649915248	0.811337462	AP1M2	protein_coding
ENSG00000129355.7	535.6706552	0.138517087	0.067065193	2.06540952	0.038884269	0.151254911	CDKN2D	protein_coding
ENSG00000129422.14	8844.738365	0.187662882	0.098784621	1.899717599	0.05747019	0.194495839	MTUS1	protein_coding
ENSG00000129437.11	37.53674842	0.468008216	0.165962783	2.819958834	0.004802981	0.036198462	KLK14	protein_coding
ENSG00000129450.8	183.2574443	0.082360274	0.114733016	0.71784284	0.472854195	0.68373277	SIGLEC9	protein_coding
ENSG00000129451.12	2997.143152	1.518546786	0.236520904	6.420349151	1.35962E-10	3.50581E-08	KLK10	protein_coding
ENSG00000129455.15	105.8347854	1.519860383	0.268880689	5.6525457	1.58089E-08	1.78472E-06	KLK8	protein_coding
ENSG00000129460.16	1214.383901	-0.078280879	0.050318256	-1.555715251	0.119775818	0.307208407	NGDN	protein_coding
ENSG00000129465.16	760.9867509	0.065320269	0.080869713	0.807722281	0.419250474	0.639269321	RIPK3	protein_coding
ENSG00000129467.14	645.3578404	0.095077355	0.088376528	1.075821353	0.282007161	0.510850767	ADCY4	protein_coding
ENSG00000129472.15	1369.447013	-0.075230254	0.044778061	-1.680069511	0.092943792	0.263371758	RAB2B	protein_coding
ENSG00000129473.11	2014.136913	-0.084524548	0.055571717	-1.520999381	0.128259993	0.319930492	BCL2L2	protein_coding
ENSG00000129474.16	1770.397494	0.002094006	0.096297593	0.021745153	0.982651245	0.992397797	AJUBA	protein_coding
ENSG00000129480.13	218.7995622	-0.055692564	0.064788902	-0.859600364	0.390009374	0.614501662	DTD2	protein_coding
ENSG00000129484.14	956.7661722	-0.001439461	0.0543841	-0.026468421	0.978883721	0.990569863	PARP2	protein_coding
ENSG00000129493.15	1419.695873	0.066253883	0.051954264	1.275234746	0.202226167	0.421168033	HEATR5A	protein_coding
ENSG00000129514.8	4083.730805	0.184042305	0.134932541	1.363957894	0.172580818	0.383252016	FOXA1	protein_coding
ENSG00000129515.20	4184.682694	0.022070082	0.046346342	0.476199009	0.633932606	0.801374495	SNX6	protein_coding
ENSG00000129518.9	1974.909975	-0.062877655	0.04899646	-1.283310166	0.199383433	0.417637562	EAPP	protein_coding
ENSG00000129521.15	1835.650436	0.019567373	0.109761356	0.178271968	0.858509392	0.93113803	EGLN3	protein_coding
ENSG00000129534.14	1889.192228	-0.074098007	0.054429376	-1.361360581	0.173399767	0.384445642	MIS18BP1	protein_coding
ENSG00000129535.13	92.21973741	-0.066709024	0.06033481	-1.10564737	0.268879178	0.496929936	NRL	protein_coding
ENSG00000129538.14	12412.96448	-0.255833555	0.132078402	-1.936982516	0.052747481	0.183945019	RNASE1	protein_coding
ENSG00000129559.13	1718.03997	-0.116960117	0.062291155	-1.877636031	0.060430973	0.200731803	NEDD8	protein_coding
ENSG00000129562.11	6914.129305	-0.102055661	0.06099956	-1.673055689	0.094316334	0.265453909	DAD1	protein_coding
ENSG00000129566.13	3972.177628	0.018795857	0.060113401	0.312673333	0.754528858	0.873900397	TEP1	protein_coding
ENSG00000129595.14	316.4815674	0.214770143	0.1084842	1.979736614	0.047733132	0.17278852	EPB41L4A	protein_coding
ENSG00000129596.5	97.00906809	0.238998791	0.177546185	1.346121802	0.178263239	0.390910686	CDO1	protein_coding
ENSG00000129625.13	8057.049152	0.027099469	0.05180179	0.523137687	0.600878441	0.779114492	REEP5	protein_coding
ENSG00000129636.13	3201.979992	0.042169515	0.047022604	0.896792427	0.36982969	0.596516109	ITFG1	protein_coding
ENSG00000129646.14	168.9185943	0.21720053	0.09396346	2.311542494	0.020802909	0.099815939	QRICH2	protein_coding
ENSG00000129654.8	797.157357	0.509277122	0.238962924	2.131197233	0.033072897	0.135784589	FOXJ1	protein_coding
ENSG00000129657.16	4409.501444	0.037297806	0.06078806	0.613571259	0.539498681	0.735182906	SEC14L1	protein_coding
ENSG00000129667.13	3744.468869	0.192092884	0.079018905	2.43097375	0.015058305	0.080312294	RHBDF2	protein_coding
ENSG00000129673.10	17.91712475	0.164896089	0.135316362	1.21859682	0.222997257	0.445575288	AANAT	protein_coding
ENSG00000129675.16	1305.72425	-0.283534492	0.103903243	-2.72883198	0.006355908	0.044012617	ARHGEF6	protein_coding
ENSG00000129680.16	766.2075057	0.078859027	0.101848	0.77428155	0.438764312	0.655446588	MAP7D3	protein_coding
ENSG00000129682.16	257.5845401	-0.132897578	0.160798777	-0.826483767	0.408529723	0.629536023	FGF13	protein_coding
ENSG00000129691.16	2905.822393	-0.046502073	0.058150608	-0.799683339	0.423894288	0.642965805	ASH2L	protein_coding

ENSG00000129696.13	568.787794	0.035828221	0.05901063	0.607148593	0.543752305	0.73835352	TTI2	protein_coding
ENSG00000129744.3	4.682796969	0.191413762	0.184511425	1.037408726	0.299545413	0.529434741	ART1	protein_coding
ENSG00000129749.3	52.66024008	-0.161526362	0.080012188	-2.018771968	0.043510926	0.162582933	CHRNA10	protein_coding
ENSG00000129757.14	1436.516307	0.483009401	0.138111249	3.497248809	0.000470083	0.006505181	CDKN1C	protein_coding
ENSG00000129810.15	531.5295583	-0.024738973	0.090050031	-0.274724759	0.783527708	0.890157909	SGO1	protein_coding
ENSG00000129816.5	0.027562383	-0.215350511	2.251021024	-0.095667925	0.923784316		TTTY1B	lncRNA
ENSG00000129824.16	3497.499111	-0.187309079	0.301345766	-0.621575281	0.534221179	0.731285931	RPS4Y1	protein_coding
ENSG00000129845.5	0.035237247	-0.092402045	1.662317891	-0.055586267	0.955671405		TTTY1	lncRNA
ENSG00000129862.7	0.154783822	-0.278953923	1.138563213	-0.245005213	0.806452395		VCY1B	protein_coding
ENSG00000129864.6	0.160420669	-0.309974617	1.099880119	-0.281825821	0.778077071		VCY	protein_coding
ENSG00000129873.7	0.095712266	-0.278132947	0.915128853	-0.303927634	0.761183024		CDY2B	protein_coding
ENSG00000129910.8	28.29600129	0.307057386	0.159389413	1.926460359	0.054046917	0.187054444	CDH15	protein_coding
ENSG00000129911.9	2730.358943	0.124212744	0.076205508	1.629970688	0.103107692	0.280574741	KLF16	protein_coding
ENSG00000129925.11	7014.969583	0.025092158	0.068672185	0.36539041	0.714819998	0.850968749	PGAP6	protein_coding
ENSG00000129932.10	1036.686833	-0.07569343	0.068864134	-1.099170574	0.271693671	0.499940677	DOHH	protein_coding
ENSG00000129933.21	4465.099818	0.064219909	0.043815051	1.465704323	0.142728856	0.341441335	MAU2	protein_coding
ENSG00000129946.10	1033.149816	0.165446399	0.120614986	1.371690231	0.170159891	0.380044978	SHC2	protein_coding
ENSG00000129951.19	22.5588851	1.059756222	0.194970421	5.435471777	5.46516E-08	4.98233E-06	PLPPR3	protein_coding
ENSG00000129965.15	0						INS-IGF2	protein_coding
ENSG00000129968.17	3151.769576	-0.013071136	0.05639758	-0.231767681	0.81671846	0.90963045	ABHD17A	protein_coding
ENSG00000129988.6	78.29096628	0.212909198	0.230962968	0.921832624	0.356615888	0.584549578	LBP	protein_coding
ENSG00000129990.15	111.0336886	0.681050899	0.200926569	3.389551229	0.000700071	0.008753804	SYT5	protein_coding
ENSG00000129991.13	48.04465718	0.389166963	0.218694147	1.779503335	0.075157279	0.230626627	TNNI3	protein_coding
ENSG00000129993.15	414.9341833	-0.237264623	0.14508272	-1.635374788	0.101970504	0.278628584	CBFA2T3	protein_coding
ENSG00000130005.13	528.5011989	-0.8125705	0.16921887	-4.801890594	1.57175E-06	7.42058E-05	GAMT	protein_coding
ENSG00000130021.15	1385.874761	-0.218041524	0.090384922	-2.412366142	0.015849356	0.08316873	PUDP	protein_coding
ENSG00000130023.16	746.3540197	-0.075326532	0.061731695	-1.220224589	0.222379748	0.444897563	ERMARD	protein_coding
ENSG00000130024.15	3498.524702	0.046779131	0.054574383	0.857162787	0.391354925	0.615600549	PHF10	protein_coding
ENSG00000130032.17	212.410363	-0.624111827	0.14614157	-4.270597516	1.9495E-05	0.000545973	PRRG3	protein_coding
ENSG00000130035.9	48.25010448	-0.151862761	0.21462809	-0.707562376	0.479217064	0.688589112	GALNT8	protein_coding
ENSG00000130037.5	77.20257417	-0.368719807	0.170040103	-2.168428505	0.030126099	0.127991129	KCNA5	protein_coding
ENSG00000130038.10	843.531184	0.210577135	0.131324429	1.603487911	0.108826976	0.289327665	CRACR2A	protein_coding
ENSG00000130045.16	17.7165735	0.363209198	0.139120863	2.610745724	0.009034504	0.056234336	NXNL2	protein_coding
ENSG00000130052.13	569.4901023	0.092998176	0.091008498	1.021862551	0.306845959	0.536338943	STARD8	protein_coding
ENSG00000130054.5	242.9969893	0.6331872	0.24029473	2.635044055	0.008412636	0.053502255	FAM155B	protein_coding
ENSG00000130055.14	151.1783237	-0.100279506	0.180007269	-0.557085868	0.577468762	0.762112445	GDPD2	protein_coding
ENSG00000130066.16	20387.4931	-0.019311097	0.08824393	-0.218837684	0.82677649	0.914652592	SAT1	protein_coding
ENSG00000130119.17	5960.30678	-0.067795397	0.052397552	-1.293865721	0.195711807	0.412755646	GNL3L	protein_coding
ENSG00000130147.16	8697.871389	0.070578938	0.077776776	0.907455178	0.364166142	0.591386598	SH3BP4	protein_coding
ENSG00000130150.12	1439.773356	-0.032423374	0.064399677	-0.503471054	0.614633126	0.788582476	MOSPD2	protein_coding
ENSG00000130158.14	4928.709271	0.089988988	0.056975747	1.579426194	0.114238333	0.298218645	DOCK6	protein_coding
ENSG00000130159.14	2240.166187	-0.061594325	0.065035826	-0.947082998	0.343596488	0.573191338	ECSIT	protein_coding

ENSG00000130164.14	9765.83636	0.418269322	0.096153229	4.350028854	1.3612E-05	0.000412495	LDLR	protein_coding
ENSG00000130165.10	2022.367631	-0.016541479	0.065344568	-0.253142378	0.800158192	0.900334118	ELOF1	protein_coding
ENSG00000130167.14	8.20130382	0.124770379	0.155807712	0.800797198	0.423249062	0.642366314	TSPAN16	protein_coding
ENSG00000130173.13	16.74442651	0.032652275	0.105802863	0.308614281	0.757614956	0.875809113	ANGPTL8	protein_coding
ENSG00000130175.10	19964.57552	0.077324241	0.059316306	1.303591652	0.192372848	0.408616239	PRKCSH	protein_coding
ENSG00000130176.8	15314.12088	-0.577426529	0.240812636	-2.397824881	0.016492748	0.085570455	CNN1	protein_coding
ENSG00000130177.16	4036.695071	0.056059249	0.050618712	1.107480745	0.268086132	0.496025365	CDC16	protein_coding
ENSG00000130182.8	11.93965542	0.339014874	0.24555252	1.380620629	0.167395637	0.3763418	ZSCAN10	protein_coding
ENSG00000130193.8	4704.658188	-0.090985535	0.090412059	-1.006342911	0.314250645	0.543529933	THEM6	protein_coding
ENSG00000130202.10	13227.35709	0.237505068	0.083033934	2.860337427	0.004231905	0.032972235	NECTIN2	protein_coding
ENSG00000130203.10	16974.63498	-0.297069776	0.148481783	-2.000715312	0.045423078	0.16722155	APOE	protein_coding
ENSG00000130204.13	5798.335047	-0.067853723	0.082095139	-0.826525462	0.40850608	0.629536023	TOMM40	protein_coding
ENSG00000130208.9	1936.646631	-0.543051779	0.153983802	-3.526681205	0.000420803	0.00597191	APOC1	protein_coding
ENSG00000130222.11	413.4936228	-0.403066962	0.12312218	-3.273715276	0.001061435	0.012007828	GADD45G	protein_coding
ENSG00000130224.15	209.9645698	0.014849912	0.123556807	0.120186916	0.904335082	0.954382061	LRCH2	protein_coding
ENSG00000130226.17	127.0683885	-0.375766714	0.24655716	-1.524055171	0.12749493	0.318929895	DPP6	protein_coding
ENSG00000130227.17	6143.099979	0.010826604	0.051133316	0.211732874	0.83231544	0.917433347	XPO7	protein_coding
ENSG00000130234.12	293.8782767	-0.973374675	0.201696343	-4.825941113	1.39344E-06	6.73713E-05	ACE2	protein_coding
ENSG00000130244.12	1077.506864	-0.104151988	0.065397233	-1.592605428	0.111248735	0.293321768	FAM98C	protein_coding
ENSG00000130254.12	3023.120822	0.032070987	0.037949461	0.845097291	0.398056521	0.62109728	SAFB2	protein_coding
ENSG00000130255.13	22686.38889	-0.187733369	0.080265809	-2.338895868	0.019340823	0.094740902	RPL36	protein_coding
ENSG00000130270.16	149.6764838	0.682115064	0.126701041	5.383657918	7.29872E-08	6.31064E-06	ATP8B3	protein_coding
ENSG00000130283.9	0.010548927	-0.168982477	2.893054209	-0.058409717	0.953422275		GDF1	protein_coding
ENSG00000130287.14	10.12740941	0.252151461	0.201108384	1.253808797	0.209911511	0.430184258	NCAN	protein_coding
ENSG00000130294.17	425.3225839	1.3709018	0.259897466	5.274779397	1.32916E-07	1.01473E-05	KIF1A	protein_coding
ENSG00000130299.17	923.925856	0.006491258	0.05999579	0.108195223	0.913840834	0.95927642	GTPBP3	protein_coding
ENSG00000130300.9	10622.05473	-0.132510711	0.086121842	-1.538642323	0.12389164	0.313356648	PLVAP	protein_coding
ENSG00000130303.13	7826.235357	-0.113256631	0.12120088	-0.934453865	0.350069823	0.579178886	BST2	protein_coding
ENSG00000130304.17	1719.045202	0.134174768	0.083786841	1.601382345	0.109292261	0.28999247	SLC27A1	protein_coding
ENSG00000130305.17	1973.221294	-0.013336601	0.068562127	-0.19451848	0.845769927	0.924384042	NSUN5	protein_coding
ENSG00000130307.12	117.4529143	-0.086241078	0.088517222	-0.974285864	0.329914621	0.559469567	USHBP1	protein_coding
ENSG00000130309.11	7052.261953	0.13747226	0.054290944	2.532139803	0.011336878	0.065958229	COLGALT1	protein_coding
ENSG00000130311.11	3173.764816	0.0536482	0.051990969	1.031875365	0.302130508	0.531819805	DDA1	protein_coding
ENSG00000130312.7	2709.480946	-0.247780533	0.065864311	-3.761984762	0.00016857	0.002974004	MRPL34	protein_coding
ENSG00000130313.7	2690.447061	-0.086046354	0.071311958	-1.206618867	0.227578957	0.450946172	PGLS	protein_coding
ENSG00000130332.15	2742.775517	-0.124248495	0.064948757	-1.913023441	0.055745052	0.190690173	LSM7	protein_coding
ENSG00000130338.13	3513.357447	0.115287373	0.067171489	1.7163141	0.086104569	0.251281595	TULP4	protein_coding
ENSG00000130340.16	5708.06664	0.200512654	0.054215471	3.698439799	0.000216929	0.003605303	SNX9	protein_coding
ENSG00000130347.13	517.8424381	-0.128863533	0.083429962	-1.544571396	0.122449969	0.311185467	RTN4IP1	protein_coding
ENSG00000130348.11	1642.271645	-0.045257686	0.065123546	-0.6949512	0.487085885	0.694572752	QRSL1	protein_coding
ENSG00000130349.10	725.2310511	0.028223882	0.070805771	0.398609914	0.690180655	0.836490124	MTRES1	protein_coding
ENSG00000130363.12	1276.457592	0.222989361	0.054224326	4.112349165	3.91653E-05	0.000946593	RSPH3	protein_coding

ENSG00000130368.7	9.161486559	-0.407382324	0.194168355	-2.098088143	0.03589736	0.143211503	MAS1	protein_coding
ENSG00000130377.13	17.60331987	-0.094567117	0.134202103	-0.704661961	0.481020629	0.68997485	ACSBG2	protein_coding
ENSG00000130382.9	5980.263007	0.055916512	0.047962741	1.165832287	0.24368226	0.469688665	MLLT1	protein_coding
ENSG00000130383.7	0.449409553	0.077895729	0.395086244	0.19716133	0.843701291		FUT5	protein_coding
ENSG00000130385.6	0.339325691	0.30439935	0.432895026	0.703171282	0.481949014		BMP15	protein_coding
ENSG00000130396.20	9266.856088	0.167087212	0.069851072	2.392049347	0.01675459	0.086445033	AFDN	protein_coding
ENSG00000130402.13	71905.60341	0.23317566	0.065327835	3.569315622	0.000357915	0.005310474	ACTN4	protein_coding
ENSG00000130413.15	240.8123276	-0.342759336	0.139834002	-2.451187346	0.014238582	0.077298455	STK33	protein_coding
ENSG00000130414.13	5621.736564	-0.203569109	0.047743134	-4.263840497	2.00943E-05	0.000559163	NDUFA10	protein_coding
ENSG00000130427.3	6.400375121	0.718846431	0.202607439	3.5479765	0.000388203	0.005612713	EPO	protein_coding
ENSG00000130429.15	170.3983532	0.129787099	0.091586265	1.417102211	0.156453046	0.361331726	ARPC1B	protein_coding
ENSG00000130433.7	31.63191036	1.480377821	0.277242787	5.339644125	9.31292E-08	7.65377E-06	CACNG6	protein_coding
ENSG00000130449.6	1633.221506	0.114341	0.056320344	2.030190033	0.042337227	0.159861967	ZSWIM6	protein_coding
ENSG00000130475.14	1168.576172	0.019709574	0.105216244	0.187324436	0.851406265	0.927213779	FCHO1	protein_coding
ENSG00000130477.15	240.9100038	0.3677914	0.170766047	2.153773573	0.031257933	0.131087186	UNC13A	protein_coding
ENSG00000130479.11	3856.085982	0.169982951	0.059959965	2.834940817	0.004583418	0.035003783	MAP1S	protein_coding
ENSG00000130487.9	384.4941464	0.235791484	0.147943084	1.593798626	0.110981145	0.29294478	KLHDC7B	protein_coding
ENSG00000130508.11	6217.527222	0.43925509	0.096635304	4.545492937	5.48068E-06	0.000201353	PXDN	protein_coding
ENSG00000130511.16	2989.428781	-0.040323291	0.062806048	-0.64202879	0.52085449	0.720948766	SSBP4	protein_coding
ENSG00000130513.6	5710.955602	0.136229781	0.138275302	0.985206899	0.324522453	0.554294474	GDF15	protein_coding
ENSG00000130517.14	2758.355962	-0.100966904	0.06881217	-1.46728267	0.142299176	0.340902524	PGPEP1	protein_coding
ENSG00000130518.17	321.0621321	-0.039676973	0.124596512	-0.318443687	0.750148404	0.8717357	IQCN	protein_coding
ENSG00000130520.11	5349.993791	-0.162916899	0.067971158	-2.396853379	0.016536539	0.085675041	LSM4	protein_coding
ENSG00000130522.6	18460.16238	0.021828347	0.075558625	0.288892857	0.77266337	0.884990582	JUND	protein_coding
ENSG00000130528.12	47.7814808	-0.289470396	0.106906143	-2.707705923	0.006775003	0.04600212	HRC	protein_coding
ENSG00000130529.16	4978.873598	-0.028984237	0.088841329	-0.326247218	0.744237319	0.868456235	TRPM4	protein_coding
ENSG00000130538.6	0.207632087	-0.063703767	0.697572121	-0.091322123	0.92723664		OR11H1	protein_coding
ENSG00000130540.14	118.8080967	-0.265717426	0.260947661	-1.018278627	0.308545563	0.537948707	SULT4A1	protein_coding
ENSG00000130544.12	633.4227479	0.000605341	0.05909082	0.010244249	0.991826415	0.996273665	ZNF557	protein_coding
ENSG00000130545.16	1615.684601	0.031917776	0.081944805	0.389503355	0.696903829	0.840338088	CRB3	protein_coding
ENSG00000130558.20	663.8539078	0.446835228	0.150179911	2.975332879	0.002926708	0.02518506	OLFM1	protein_coding
ENSG00000130559.19	2615.489759	0.198522092	0.059695724	3.325566384	0.000882391	0.010423406	CAMSAP1	protein_coding
ENSG00000130560.9	2213.347128	0.021395895	0.048404421	0.442023562	0.658472156	0.81673188	UBAC1	protein_coding
ENSG00000130561.17	15.24990833	-0.41728054	0.182495461	-2.286525578	0.02222353	0.104300996	SAG	protein_coding
ENSG00000130584.12	483.9334731	0.231360785	0.092243528	2.508151965	0.012136446	0.069079141	ZBTB46	protein_coding
ENSG00000130589.16	10253.55796	0.17742989	0.089066674	1.992101893	0.046359879	0.169481918	HELZ2	protein_coding
ENSG00000130590.14	1189.693132	-0.203628255	0.086366942	-2.357710613	0.018388021	0.091817899	SAMD10	protein_coding
ENSG00000130592.17	2839.43149	-0.209874979	0.110458341	-1.900037408	0.057428211	0.194492003	LSP1	protein_coding
ENSG00000130595.19	17.12945986	0.076679685	0.196128641	0.390966281	0.69582216	0.839498388	TNNT3	protein_coding
ENSG00000130598.16	96.10163894	0.12941248	0.174498242	0.741626269	0.458313803	0.671754754	TNNI2	protein_coding
ENSG00000130600.19	5135.34926	0.952462533	0.234433781	4.0628212	4.84832E-05	0.001120823	H19	lncRNA
ENSG00000130612.16	10.89957223	-0.019289284	0.172988539	-0.111506139	0.911214998	0.958066302	CYP2G1P	transcribed_unprocessed_pseudogene

ENSG00000130635.16	25440.51921	0.779833686	0.112528927	6.930073087	4.20623E-12	1.99234E-09	COL5A1		protein_coding
ENSG00000130638.17	3823.80792	-0.078382144	0.049347691	-1.588364967	0.112203835	0.29499285	ATXN10		protein_coding
ENSG00000130640.13	4472.339765	0.056907215	0.043200553	1.317279762	0.187744866	0.402888864	TUBGCP2		protein_coding
ENSG00000130643.9	31.69825806	0.024513349	0.207798291	0.117967039	0.906093774	0.955375517	CALY		protein_coding
ENSG00000130649.10	261.6320997	-0.098561144	0.189774178	-0.51936014	0.603509622	0.781133006	CYP2E1		protein_coding
ENSG00000130653.16	491.9319018	0.351299855	0.116908464	3.004913786	0.002656561	0.023472217	PNPLA7		protein_coding
ENSG00000130656.6	1.462659094	0.069916783	0.282838604	0.247196747	0.804755963	0.902952021	HBZ		protein_coding
ENSG00000130669.17	5774.01178	0.137806042	0.072676596	1.896154341	0.057939639	0.195587237	PAK4		protein_coding
ENSG00000130675.15	593.5379653	-0.200928455	0.103339876	-1.944345818	0.051853763	0.182147682	MNX1		protein_coding
ENSG00000130684.14	152.0597068	-0.122284893	0.07818908	-1.563963831	0.117826063	0.303890199	ZNF337		protein_coding
ENSG00000130695.16	1041.009787	-0.019115348	0.06948737	-0.275090963	0.783246354	0.89011674	CEP85		protein_coding
ENSG00000130699.18	2928.600102	-0.027234146	0.062371783	-0.436642101	0.662370925	0.819166131	TAF4		protein_coding
ENSG00000130700.7	663.8073126	-0.329015897	0.270874911	-1.214641461	0.224502868	0.447425063	GATA5		protein_coding
ENSG00000130701.4	493.695708	-0.130507654	0.150304023	-0.868291157	0.385234962	0.609827242	RBBP8NL		protein_coding
ENSG00000130702.15	17989.27873	0.519302014	0.095845901	5.418093072	6.02381E-08	5.43567E-06	LAMA5		protein_coding
ENSG00000130703.17	4968.48673	0.146151688	0.064538584	2.264562983	0.023539511	0.108502354	OSBPL2		protein_coding
ENSG00000130706.13	4296.34058	-0.06184367	0.072097362	-0.857779921	0.391013999	0.615334117	ADRM1		protein_coding
ENSG00000130707.18	14384.74447	0.216201977	0.138939794	1.556083902	0.119688142	0.307040383	ASS1		protein_coding
ENSG00000130711.5	14.87490153	0.160240131	0.128579607	1.246232858	0.212678919	0.433282341	PRDM12		protein_coding
ENSG00000130713.16	1530.00296	-0.035032027	0.052074361	-0.672730807	0.501118565	0.70638687	EXOSC2		protein_coding
ENSG00000130714.18	1341.525526	0.044490241	0.056269989	0.790656641	0.429144385	0.647356163	POMT1		protein_coding
ENSG00000130717.13	1146.158919	0.068188101	0.051978409	1.311854326	0.189569291	0.405063302	UCK1		protein_coding
ENSG00000130720.13	489.0967666	0.488872972	0.251739954	1.941976091	0.052139995	0.1826715	FIBCD1		protein_coding
ENSG00000130723.21	13199.20423	0.174246534	0.06822647	2.553943271	0.010651061	0.063105544	PRRC2B		protein_coding
ENSG00000130724.9	5096.641005	-0.086833154	0.059312041	-1.46400549	0.143192448	0.34215591	CHMP2A		protein_coding
ENSG00000130725.8	4513.79262	0.035699766	0.063258732	0.564345271	0.572519178	0.758555277	UBE2M		protein_coding
ENSG00000130726.12	22747.45494	0.039281013	0.068000929	0.577654059	0.563497701	0.75229127	TRIM28		protein_coding
ENSG00000130731.16	4001.404368	-0.151728295	0.072222918	-2.100833064	0.035655623	0.142403545	METTL26		protein_coding
ENSG00000130733.11	2484.880954	0.134998421	0.067379971	2.00353932	0.045119432	0.166568068	YIPF2		protein_coding
ENSG00000130734.10	1164.931452	-0.017349316	0.069743975	-0.248757197	0.803548602	0.902413155	ATG4D		protein_coding
ENSG00000130741.11	14217.56228	-0.116444627	0.059784827	-1.947728766	0.051447426	0.181199249	EIF2S3		protein_coding
ENSG00000130748.7	960.7748185	-0.120845616	0.075732601	-1.595688181	0.110558429	0.292244151	TMEM160		protein_coding
ENSG00000130749.10	4371.46523	-0.013883688	0.045006345	-0.308482897	0.757714913	0.875857257	ZC3H4		protein_coding
ENSG00000130751.10	89.0494074	-0.170457618	0.117046005	-1.456330074	0.145301396	0.345511528	NPAS1		protein_coding
ENSG00000130755.13	687.9582466	-0.339921104	0.094131301	-3.611137858	0.000304857	0.004700897	GMFG		protein_coding
ENSG00000130758.8	1173.883369	0.152761835	0.067581669	2.260403409	0.023796225	0.109189018	MAP3K10		protein_coding
ENSG00000130762.15	3081.298036	0.109715865	0.094356303	1.162782567	0.244917732	0.471183612	ARHGEF16		protein_coding
ENSG00000130764.10	3151.013934	-0.014285984	0.042278738	-0.337899964	0.735438574	0.862802672	LRRC47		protein_coding
ENSG00000130766.5	1741.01307	-0.208988764	0.084099092	-2.48502998	0.012954052	0.072390195	SESN2		protein_coding
ENSG00000130768.15	946.5550314	0.011525103	0.117665101	0.097948355	0.921973303	0.963172363	SMPDL3B		protein_coding
ENSG00000130770.18	6361.565891	-0.258706987	0.065333079	-3.959816234	7.50075E-05	0.001589013	ATP5IF1		protein_coding
ENSG00000130772.14	792.9965781	-0.139996486	0.05514742	-2.538586306	0.011130135	0.065111811	MED18		protein_coding

ENSG00000130775.16	1563.672555	-0.019919947	0.096789053	-0.20580785	0.836941009	0.919949006	THEMIS2	protein_coding
ENSG00000130779.21	6146.492555	0.002833564	0.065476417	0.043276096	0.965481446	0.984285086	CLIP1	protein_coding
ENSG00000130783.14	27.63327561	-0.26245547	0.12116037	-2.166182469	0.030297246	0.128380309	CCDC62	protein_coding
ENSG00000130787.14	5259.652007	-0.324767303	0.084115993	-3.860945931	0.000112949	0.002173057	HIP1R	protein_coding
ENSG00000130803.15	2461.223902	-0.038362335	0.039723444	-0.965735379	0.334176611	0.563657324	ZNF317	protein_coding
ENSG00000130810.20	243.9653368	-0.045792772	0.070621557	-0.648424843	0.516710207	0.718067414	PPAN	protein_coding
ENSG00000130811.12	6285.397265	-0.084162152	0.054469732	-1.545117797	0.122317773	0.311082328	EIF3G	protein_coding
ENSG00000130812.10	50.72971496	-0.824783229	0.121007288	-6.815979776	9.36235E-12	3.77706E-09	ANGPTL6	protein_coding
ENSG00000130813.18	1295.094006	-0.036357556	0.069607591	-0.522321704	0.60144636	0.779572517	SHFL	protein_coding
ENSG00000130816.17	6618.587506	0.00277123	0.065129088	0.042549809	0.966060406	0.98448128	DNMT1	protein_coding
ENSG00000130818.12	1023.468201	0.209797788	0.077484943	2.707594278	0.006777282	0.04600212	ZNF426	protein_coding
ENSG00000130821.17	5649.926622	0.2609718	0.130460656	2.000386997	0.045458491	0.167309517	SLC6A8	protein_coding
ENSG00000130822.16	317.093465	-0.501404805	0.233473312	-2.147589378	0.03174639	0.132396635	PNCK	protein_coding
ENSG00000130826.18	5173.042971	-0.087994345	0.064506576	-1.36411434	0.172531582	0.383181732	DKC1	protein_coding
ENSG00000130827.6	4503.483635	0.368415407	0.078635556	4.685099551	2.79824E-06	0.000117588	PLXNA3	protein_coding
ENSG00000130829.18	103.6345618	0.556556798	0.262955389	2.116544558	0.03429852	0.138944557	DUSP9	protein_coding
ENSG00000130830.15	1223.178646	-0.106364088	0.103430346	-1.02836442	0.303778438	0.533216344	MPP1	protein_coding
ENSG00000130844.19	1006.466476	0.001516591	0.11945738	0.012695667	0.989870595	0.995404463	ZNF331	protein_coding
ENSG00000130856.17	1204.295729	-0.086421128	0.050881147	-1.698490181	0.089415284	0.257208387	ZNF236	protein_coding
ENSG00000130876.11	39.65174336	1.046436251	0.297964202	3.511952926	0.000444827	0.006232867	SLC7A10	protein_coding
ENSG00000130881.14	2421.501201	0.029131333	0.119409112	0.243962392	0.807259947	0.904162389	LRP3	protein_coding
ENSG00000130921.8	966.2869002	-0.10348529	0.045376691	-2.280582533	0.022573162	0.105476161	C12orf65	protein_coding
ENSG00000130935.10	2934.776974	-0.024339687	0.049647241	-0.490252562	0.623955191	0.794799631	NOL11	protein_coding
ENSG00000130939.20	4783.463775	-0.001251304	0.05075063	-0.024655921	0.980329415	0.991327585	UBE4B	protein_coding
ENSG00000130940.15	2512.837422	0.077876406	0.081908822	0.95076945	0.341721424	0.571529246	CASZ1	protein_coding
ENSG00000130943.7	68.93011537	-0.162262599	0.112242312	-1.44564556	0.148276641	0.349815992	PKDREJ	protein_coding
ENSG00000130948.10	1.097952038	0.082305548	0.232297018	0.354311687	0.72310531	0.855433359	HSD17B3	protein_coding
ENSG00000130950.14	4.757551796	-0.260912955	0.186943433	-1.395678629	0.162811293	0.369869028	NUTM2F	protein_coding
ENSG00000130956.14	624.2435606	-0.240852748	0.081708724	-2.947699287	0.003201483	0.026974291	HABP4	protein_coding
ENSG00000130957.5	66.66207234	-0.988985807	0.185683858	-5.326180826	1.00299E-07	8.07901E-06	FBP2	protein_coding
ENSG00000130958.13	735.2169659	-0.381310692	0.063679277	-5.987987137	2.12454E-09	3.41627E-07	SLC35D2	protein_coding
ENSG00000130962.18	849.1321472	0.416717951	0.096602389	4.313743765	1.60513E-05	0.000468877	PRRG1	protein_coding
ENSG00000130985.17	27967.11334	-0.019922998	0.04941231	-0.403199091	0.686801768	0.834653468	UBA1	protein_coding
ENSG00000130988.13	305.2500081	-0.937076491	0.191300044	-4.89846458	9.65884E-07	5.05286E-05	RGN	protein_coding
ENSG00000130997.16	29.71092758	0.074947329	0.124251959	0.603188311	0.546383423	0.740299891	POLN	protein_coding
ENSG00000131002.12	781.2167022	-0.235706484	0.285814879	-0.824682341	0.40955196	0.630503983	TXLNGY	transcribed_unprocessed_pseudogene
ENSG00000131007.9	0.190325415	-0.248723555	0.514275207	-0.483639016	0.62864208		TTTY9B	lncRNA
ENSG00000131013.4	1827.393637	0.045658509	0.043638287	1.046294708	0.295424988	0.525263561	PPIL4	protein_coding
ENSG00000131015.5	229.614402	1.293390391	0.167482463	7.722542203	1.14032E-14	9.74349E-12	ULBP2	protein_coding
ENSG00000131016.17	4242.224588	0.409404194	0.156451493	2.616812318	0.008875513	0.055538925	AKAP12	protein_coding
ENSG00000131018.24	3491.629398	-0.166402267	0.126459175	-1.315857605	0.18822184	0.403589192	SYNE1	protein_coding
ENSG00000131019.11	399.5108027	0.451000432	0.121960655	3.697917422	0.000217376	0.00361088	ULBP3	protein_coding

ENSG00000131023.13	3385.416886	0.063401874	0.047633657	1.331030992	0.183178813	0.396969725	LATS1	protein_coding
ENSG00000131037.15	3379.001882	0.266632211	0.130662254	2.04062155	0.041288459	0.1571103	EPS8L1	protein_coding
ENSG00000131042.15	663.9179106	0.018132976	0.117947164	0.153738127	0.877816221	0.941115915	LILRB2	protein_coding
ENSG00000131043.12	2992.326615	-0.023902179	0.046787498	-0.510866786	0.60944434	0.785128481	AAR2	protein_coding
ENSG00000131044.17	31.73093682	-0.062027114	0.127735902	-0.485588728	0.62725879	0.796963837	TTLL9	protein_coding
ENSG00000131050.11	28.07345122	-0.285116553	0.280547426	-1.016286469	0.30949299	0.538904593	BPIFA2	protein_coding
ENSG00000131051.24	10746.83663	-0.03064776	0.051319463	-0.597195654	0.55037676	0.742906376	RBM39	protein_coding
ENSG00000131055.5	98.94693181	-0.106458414	0.09648334	-1.103386489	0.269859361	0.497999017	COX4I2	protein_coding
ENSG00000131059.12	1.830372121	-0.018267265	0.395463354	-0.046192054	0.963157176	0.983047049	BPIFA3	protein_coding
ENSG00000131061.14	525.3236231	-0.005760275	0.0439328	-0.131115585	0.895683873	0.950498453	ZNF341	protein_coding
ENSG00000131067.17	1144.67869	0.178292724	0.117236794	1.520791538	0.128312159	0.320022392	GGT7	protein_coding
ENSG00000131068.4	5.324719005	-0.31155302	0.281470452	-1.106876468	0.268347341	0.496403281	DEFB118	protein_coding
ENSG00000131069.20	5393.616095	-0.285938393	0.087624694	-3.263217042	0.001101551	0.012374916	ACSS2	protein_coding
ENSG00000131080.15	112.0647153	0.174106093	0.150600009	1.156082883	0.247647257	0.474220927	EDA2R	protein_coding
ENSG00000131089.17	1347.220514	-0.114975481	0.06405934	-1.794827738	0.07268115	0.225313116	ARHGEF9	protein_coding
ENSG00000131094.4	149.9366211	0.155051623	0.153546075	1.009805193	0.312588632	0.542134634	C1QL1	protein_coding
ENSG00000131095.13	24.26351559	0.265057496	0.151788149	1.74623314	0.080770448	0.241459121	GFAP	protein_coding
ENSG00000131096.10	21.12126614	-0.252368555	0.209173286	-1.206504708	0.227622945	0.450992319	PYY	protein_coding
ENSG00000131097.7	42.7484126	0.068825537	0.078551438	0.876184303	0.380929848	0.606495433	HIGD1B	protein_coding
ENSG00000131100.13	5067.059479	-0.002612269	0.04667225	-0.055970497	0.95536531	0.979241325	ATP6V1E1	protein_coding
ENSG00000131115.16	488.0076686	-0.000481586	0.052475755	-0.009177308	0.99267767	0.996542454	ZNF227	protein_coding
ENSG00000131116.12	610.2078873	-0.086054551	0.071866199	-1.19742733	0.231140037	0.455125813	ZNF428	protein_coding
ENSG00000131126.19	18.30887464	-0.453606939	0.212487206	-2.134749416	0.032781484	0.135067166	TEX101	protein_coding
ENSG00000131127.14	848.1741189	-0.053821691	0.114660535	-0.469400312	0.638783525	0.804400222	ZNF141	protein_coding
ENSG00000131142.13	1610.94794	-0.622586004	0.333870018	-1.864755655	0.062215662	0.204554994	CCL25	protein_coding
ENSG00000131143.10	17323.72309	-0.157961444	0.062045159	-2.545910842	0.010899302	0.064140296	COX4I1	protein_coding
ENSG00000131148.9	1692.767887	0.010900467	0.054463014	0.200144396	0.841367653	0.92200346	EMC8	protein_coding
ENSG00000131149.19	6081.76504	0.155155912	0.058687733	2.643753705	0.008199229	0.052652198	GSE1	protein_coding
ENSG00000131152.4	0						AC010531.1	protein_coding
ENSG00000131153.9	1070.665126	-0.004442212	0.10867956	-0.040874397	0.967396028	0.984728258	GINS2	protein_coding
ENSG00000131165.16	5775.782011	-0.006463323	0.05696759	-0.113456141	0.909668933	0.957272231	CHMP1A	protein_coding
ENSG00000131171.13	7954.987401	-0.204999602	0.09250628	-2.216061459	0.026687293	0.11800631	SH3BGRL	protein_coding
ENSG00000131174.7	8333.726094	-0.234943513	0.070637361	-3.326051695	0.000880856	0.010413743	COX7B	protein_coding
ENSG00000131183.11	6.387468165	-0.113250202	0.208183813	-0.54399139	0.586447398	0.768636257	SLC34A1	protein_coding
ENSG00000131187.10	449.9139573	-0.064544724	0.141070849	-0.457534098	0.647287199	0.809673823	F12	protein_coding
ENSG00000131188.12	1025.697956	-0.095560212	0.104684498	-0.912840141	0.361326623	0.589146301	PRR7	protein_coding
ENSG00000131196.18	1406.726962	0.159372578	0.102650086	1.552581049	0.120523256	0.308375171	NFATC1	protein_coding
ENSG00000131203.13	2377.015983	0.113843346	0.215748805	0.527666172	0.597731056	0.776909714	IDO1	protein_coding
ENSG00000131233.10	2.047485726	-0.218879871	0.18229573	-1.200685671	0.229873154	0.453553493	GJA9	protein_coding
ENSG00000131236.18	22001.01846	0.081680257	0.043163559	1.892342972	0.058445299	0.196517871	CAP1	protein_coding
ENSG00000131238.18	7846.030782	0.047307937	0.055797058	0.84785719	0.396517508	0.61976483	PPT1	protein_coding
ENSG00000131242.18	3415.44563	-0.155741544	0.073828479	-2.109504964	0.034901018	0.140458225	RAB11FIP4	protein_coding

ENSG00000131263.13	4388.149763	0.090236836	0.05453541	1.654646675	0.09799619	0.272186375	RLIM	protein_coding
ENSG00000131264.3	0.594824242	-0.218350796	0.449468187	-0.485798111	0.627110314		CDX4	protein_coding
ENSG00000131269.19	1522.172784	-0.028045332	0.053030666	-0.528851205	0.596908673	0.776323323	ABCB7	protein_coding
ENSG00000131323.16	2685.4727	-0.0049179	0.052297184	-0.094037572	0.925079311	0.96471831	TRAF3	protein_coding
ENSG00000131351.15	532.3865091	-0.142771998	0.056097766	-2.545056749	0.010925998	0.06424534	HAUS8	protein_coding
ENSG00000131355.15	61.55958807	0.169750301	0.164997267	1.028806744	0.303570497	0.533092547	ADGRE3	protein_coding
ENSG00000131368.8	1681.871444	-0.114827388	0.054204155	-2.118424089	0.034139168	0.138543723	MRPS25	protein_coding
ENSG00000131370.16	372.5462578	0.076547463	0.099717787	0.767641014	0.442700489	0.659062236	SH3BP5	protein_coding
ENSG00000131373.15	1009.541939	-0.140840717	0.053044362	-2.655149614	0.007927324	0.051424885	HACL1	protein_coding
ENSG00000131374.14	4407.311793	-0.013544344	0.055700873	-0.243162143	0.807879792	0.904640192	TBC1D5	protein_coding
ENSG00000131375.10	2153.094675	0.085264509	0.048523928	1.757164175	0.07888983	0.238102163	CAPN7	protein_coding
ENSG00000131378.14	2121.584724	-0.13426787	0.100819641	-1.331763026	0.182938071	0.396808976	RFTN1	protein_coding
ENSG00000131379.10	14.96930437	-0.289972646	0.148059716	-1.958484407	0.050173199	0.178481428	C3orf20	protein_coding
ENSG00000131381.12	1973.704902	0.037076924	0.052335394	0.708448365	0.478666867	0.688207003	RBSN	protein_coding
ENSG00000131386.19	343.0332566	0.988305687	0.148861048	6.639115491	3.15571E-11	1.01115E-08	GALNT15	protein_coding
ENSG00000131389.17	4969.523921	0.110822764	0.070683294	1.567877744	0.116909667	0.302434047	SLC6A6	protein_coding
ENSG00000131398.15	459.0633577	0.348705466	0.120604889	2.891304568	0.003836461	0.030786543	KCNC3	protein_coding
ENSG00000131400.8	28.20144909	-0.119268886	0.124359723	-0.959063619	0.337526697	0.567278651	NAPSA	protein_coding
ENSG00000131401.11	421.4866373	-0.421640591	0.145537391	-2.897128975	0.003765949	0.030362397	NAPSB	transcribed_unprocessed_pseudogene
ENSG00000131408.15	4565.561771	0.090649772	0.059493802	1.523684311	0.127587591	0.319031063	NR1H2	protein_coding
ENSG00000131409.13	237.0882908	0.020515589	0.131111863	0.156473936	0.875659462	0.93989045	LRRC4B	protein_coding
ENSG00000131435.13	1308.757975	0.293172241	0.126785027	2.312356969	0.02075802	0.099644439	PDLIM4	protein_coding
ENSG00000131437.16	974.7684919	-0.026288515	0.061288139	-0.428933159	0.667971868	0.822713321	KIF3A	protein_coding
ENSG00000131446.17	8319.873111	0.136008444	0.053758955	2.529968149	0.011407288	0.066199946	MGAT1	protein_coding
ENSG00000131459.13	743.8131509	0.741340919	0.13660237	5.426998939	5.73095E-08	5.19821E-06	GFPT2	protein_coding
ENSG00000131462.8	2836.074721	0.032722534	0.062842194	0.520709599	0.602569086	0.780493805	TUBG1	protein_coding
ENSG00000131467.11	10565.84756	0.043949048	0.051329935	0.856206972	0.391883307	0.615865069	PSME3	protein_coding
ENSG00000131469.15	40508.86498	-0.144555606	0.068947288	-2.096610473	0.036028071	0.143548987	RPL27	protein_coding
ENSG00000131470.15	188.0292481	0.003621709	0.06778625	0.053428374	0.957390598	0.980129918	PSMC3IP	protein_coding
ENSG00000131471.7	3642.762206	-0.120293609	0.164391753	-0.731749658	0.464321381	0.676384385	AOC3	protein_coding
ENSG00000131473.17	9993.615207	0.081455579	0.06549098	1.24376791	0.213584994	0.434336334	ACLY	protein_coding
ENSG00000131475.7	3549.10837	0.049919876	0.059621069	0.837285819	0.402431928	0.624328959	VPS25	protein_coding
ENSG00000131477.11	1708.316691	-0.104415163	0.099141114	-1.053197394	0.292250533	0.521985506	RAMP2	protein_coding
ENSG00000131480.9	53.83924924	0.309263693	0.098236404	3.148157708	0.00164303	0.016600586	AOC2	protein_coding
ENSG00000131482.10	9.649444691	-0.480948872	0.254392295	-1.890579556	0.058680491	0.197050149	G6PC	protein_coding
ENSG00000131484.4	4.457475545	-0.530380014	0.166090678	-3.193315959	0.00140649	0.014887199	AC091132.1	lncRNA
ENSG00000131495.9	1371.410192	-0.163991902	0.066893783	-2.451526784	0.01422516	0.077244834	NDUFA2	protein_coding
ENSG00000131503.21	207.5973307	-0.209927461	0.072758768	-2.885253093	0.003910991	0.031271423	ANKHD1	protein_coding
ENSG00000131504.17	12609.20207	-0.010421872	0.064702186	-0.161074498	0.872034726	0.938222603	DIAPH1	protein_coding
ENSG00000131507.11	7938.529307	-0.006764691	0.049209991	-0.137465794	0.890662628	0.947901515	NDFIP1	protein_coding
ENSG00000131508.16	5029.971972	-0.073159467	0.040173845	-1.821072043	0.068595906	0.217065123	UBE2D2	protein_coding
ENSG00000131538.7	0.003245054	-0.122775387	2.893054209	-0.042437984	0.966149549		TTTY6	lncRNA

ENSG00000131548.7	0.002056852	-0.122775387	2.893054209	-0.042437984	0.966149549		TTTY6B	lncRNA
ENSG00000131558.15	4124.253137	-0.068357877	0.044842938	-1.524384442	0.127412704	0.318820753	EXOC4	protein_coding
ENSG00000131584.19	2554.672061	0.10071472	0.066891078	1.505652521	0.132156398	0.325910224	ACAP3	protein_coding
ENSG00000131591.18	1094.39152	0.070149083	0.064199735	1.09266937	0.274538993	0.503217028	C1orf159	protein_coding
ENSG00000131620.17	6957.213828	0.325585495	0.135835067	2.396917831	0.016533631	0.085675041	ANO1	protein_coding
ENSG00000131626.19	4446.358389	0.244742339	0.063447591	3.857393696	0.000114602	0.002196145	PPFIA1	protein_coding
ENSG00000131634.14	1061.716394	0.135249031	0.086644101	1.560972172	0.118530317	0.304976316	TMEM204	protein_coding
ENSG00000131650.14	117.8546715	0.30736254	0.150041966	2.048510489	0.040510005	0.155292486	KREMEN2	protein_coding
ENSG00000131652.14	1265.072213	-0.164583594	0.064180739	-2.564376742	0.010336128	0.061816273	THOC6	protein_coding
ENSG00000131653.13	7776.976698	0.055095476	0.06035711	0.912824962	0.361334608	0.589146301	TRAF7	protein_coding
ENSG00000131668.14	2107.024064	-0.128432877	0.197096724	-0.651623599	0.51464402	0.716480608	BARX1	protein_coding
ENSG00000131669.10	3399.885544	-0.087974766	0.068747194	-1.279685197	0.200655873	0.419214737	NINJ1	protein_coding
ENSG00000131686.15	12.9856025	-0.095170557	0.268892948	-0.353934745	0.723387787	0.855512514	CA6	protein_coding
ENSG00000131697.18	1147.893137	0.259037958	0.080420365	3.22104927	0.001277222	0.013824515	NPHP4	protein_coding
ENSG00000131711.15	5221.157699	-0.092048922	0.142685369	-0.645118153	0.518850621	0.719558064	MAP1B	protein_coding
ENSG00000131721.6	5.947529406	-0.791940826	0.209816252	-3.774449403	0.000160361	0.002867489	RHOXF2	protein_coding
ENSG00000131724.11	9830.18228	0.167830838	0.065455845	2.564031339	0.01034642	0.061829135	IL13RA1	protein_coding
ENSG00000131725.14	1600.476494	0.102713684	0.057870657	1.77488366	0.075917099	0.232030545	WDR44	protein_coding
ENSG00000131730.16	105.5622003	0.032930575	0.151025488	0.21804647	0.827392904	0.914993036	CKMT2	protein_coding
ENSG00000131732.12	1122.822194	-0.047231989	0.04942478	-0.955633763	0.339257297	0.569089816	ZCCHC9	protein_coding
ENSG00000131737.5	3.819213711	0.392308543	0.332932951	1.178340989	0.238660693	0.46406305	KRT34	protein_coding
ENSG00000131738.11	1.738228058	-0.160338066	0.363101398	-0.441579314	0.658793656	0.816944627	KRT33B	protein_coding
ENSG00000131746.13	9941.203061	0.018449788	0.225368635	0.081864933	0.93475412	0.969251899	TNS4	protein_coding
ENSG00000131747.15	13905.16346	0.065569983	0.119613713	0.548181153	0.583567522	0.766757503	TOP2A	protein_coding
ENSG00000131748.16	3530.672177	-0.157588475	0.106678563	-1.477227205	0.139614744	0.336810345	STARD3	protein_coding
ENSG00000131759.18	3437.982182	-0.022622911	0.083996401	-0.269331904	0.787674283	0.892983044	RARA	protein_coding
ENSG00000131771.14	11659.15047	-0.470743093	0.210698678	-2.234200503	0.025469883	0.114399207	PPP1R1B	protein_coding
ENSG00000131773.14	598.5371232	0.234471688	0.13018191	1.801108057	0.07168585	0.223179854	KHDRBS3	protein_coding
ENSG00000131778.19	33.80912434	0.011244003	0.084639074	0.132846478	0.894314796	0.949696826	CHD1L	protein_coding
ENSG00000131779.11	1455.480995	-0.055058935	0.048201239	-1.142272193	0.253340896	0.480129221	PEX11B	protein_coding
ENSG00000131781.13	1388.664055	-0.156549709	0.136700621	-1.145201156	0.25212584	0.478856682	FMO5	protein_coding
ENSG00000131788.16	2217.530141	0.283401188	0.061932625	4.575959555	4.74042E-06	0.000178458	PIAS3	protein_coding
ENSG00000131791.8	2142.360182	-0.083618904	0.080295944	-1.041383913	0.297697406	0.527648249	PRKAB2	protein_coding
ENSG00000131797.12	745.9273645	-0.118156469	0.071053476	-1.662923137	0.096327822	0.268978439	CLUHP3	transcribed_unprocessed_pseudogene
ENSG00000131808.11	0.782245874	-0.413326929	0.58662243	-0.704587665	0.481066877		FSHB	protein_coding
ENSG00000131828.14	4592.616005	-0.222261368	0.062030099	-3.583121269	0.000339513	0.005098192	PDHA1	protein_coding
ENSG00000131831.18	1214.11622	-0.090204345	0.131613124	-0.685375002	0.493107314	0.700079209	RAI2	protein_coding
ENSG00000131844.16	6982.648777	-0.187914364	0.061814423	-3.039976036	0.00236597	0.021567333	MCCC2	protein_coding
ENSG00000131845.15	619.2009136	0.01294268	0.084150266	0.153804381	0.87776398	0.941083059	ZNF304	protein_coding
ENSG00000131848.9	363.6780797	-0.139146817	0.063948884	-2.17590689	0.029562223	0.126309738	ZSCAN5A	protein_coding
ENSG00000131849.12	179.0922711	-0.168251117	0.085186195	-1.975098408	0.048256981	0.174037448	ZNF132	protein_coding
ENSG00000131864.10	3.819770956	-0.035342509	0.356521523	-0.099131488	0.921033872	0.962860882	USP29	protein_coding

ENSG00000131871.15	3665.959244	0.199206024	0.060680813	3.282850289	0.001027632	0.011722809	SELENOS	protein_coding
ENSG00000131873.7	2935.932297	0.236901358	0.060968727	3.885620863	0.000102069	0.00201625	CHSY1	protein_coding
ENSG00000131876.17	2061.139408	0.041172162	0.06069311	0.678366325	0.497539455	0.703597839	SNRPA1	protein_coding
ENSG00000131885.17	6.747853624	1.177398824	0.245976908	4.786623401	1.69611E-06	7.82559E-05	KRT17P1	transcribed_unprocessed_pseudogene
ENSG00000131899.11	2777.805921	0.223770012	0.062842556	3.560803797	0.000369721	0.005437511	LLGL1	protein_coding
ENSG00000131910.5	858.510682	-0.144920299	0.192643367	-0.752272457	0.451887228	0.666392207	NR0B2	protein_coding
ENSG00000131914.11	8.857115025	-0.089573698	0.196283178	-0.456349336	0.648138792	0.810140956	LIN28A	protein_coding
ENSG00000131931.8	542.7265038	-0.0610222	0.047261336	-1.291165368	0.196646341	0.413873828	THAP1	protein_coding
ENSG00000131941.8	6834.456785	0.130908576	0.099363	1.317478103	0.187678416	0.402860073	RHPN2	protein_coding
ENSG00000131943.18	1849.69463	0.262156208	0.097612112	2.685693431	0.007237947	0.048166827	C19orf12	protein_coding
ENSG00000131944.10	314.0713577	-0.219043161	0.076746803	-2.854101423	0.004315875	0.0334828	FAAP24	protein_coding
ENSG00000131951.11	12.65944473	0.016638446	0.186461441	0.089232635	0.92889703	0.966282635	LRRC9	protein_coding
ENSG00000131966.14	2374.2512	0.018873582	0.044094079	0.428029852	0.668629387	0.823168233	ACTR10	protein_coding
ENSG00000131969.15	57.34865477	1.574448204	0.196549775	8.010429932	1.14308E-15	1.4232E-12	ABHD12B	protein_coding
ENSG00000131979.20	1179.336419	0.070836378	0.076373916	0.927494386	0.353669905	0.581968841	GCH1	protein_coding
ENSG00000131981.16	26740.33364	0.062036607	0.095880533	0.647019832	0.517619108	0.718788052	LGALS3	protein_coding
ENSG00000131982.5	1.588586259	-0.085173757	0.199703671	-0.426500709	0.669743032	0.823906178	UBE2L2	processed_pseudogene
ENSG00000132000.13	494.7909082	0.797585503	0.123881274	6.438305627	1.20815E-10	3.19075E-08	PODNL1	protein_coding
ENSG00000132002.9	10869.42759	0.178031386	0.096310626	1.848512389	0.064528254	0.209020123	DNAJB1	protein_coding
ENSG00000132003.9	798.6228087	0.460825573	0.074636911	6.174231615	6.6486E-10	1.27073E-07	ZSWIM4	protein_coding
ENSG00000132004.13	411.1494019	0.135742051	0.065486024	2.072840015	0.038187171	0.149352213	FBXW9	protein_coding
ENSG00000132005.9	1901.632894	0.084154449	0.04839951	1.738745885	0.082079471	0.24406531	RFX1	protein_coding
ENSG00000132010.16	27.3111565	-0.133814326	0.092686191	-1.443735306	0.148813446	0.350551032	ZNF20	protein_coding
ENSG00000132016.11	237.2612986	-0.428189102	0.128378902	-3.335354142	0.000851908	0.010148608	C19orf57	protein_coding
ENSG00000132017.10	1891.938008	0.012172803	0.055350716	0.21992132	0.825932432	0.914099596	DCAF15	protein_coding
ENSG00000132024.17	3289.540462	-0.098185352	0.065148237	-1.507106806	0.131783282	0.325311284	CC2D1A	protein_coding
ENSG00000132026.14	50.57867795	-0.376769359	0.21130167	-1.783087463	0.074572067	0.229298205	RTBDN	protein_coding
ENSG00000132031.13	375.880086	0.872781001	0.163170802	5.348879746	8.85003E-08	7.34586E-06	MATN3	protein_coding
ENSG00000132109.10	1595.615445	-0.060470907	0.065792259	-0.919118864	0.358033403	0.585661472	TRIM21	protein_coding
ENSG00000132122.12	719.2381835	-0.183636588	0.083559855	-2.197665215	0.027972972	0.121581708	SPATA6	protein_coding
ENSG00000132128.16	4921.239168	0.040069401	0.039916912	1.003820163	0.315465304	0.544631019	LRRC41	protein_coding
ENSG00000132141.14	86.3260004	-0.154726029	0.09286105	-1.666210197	0.095671556	0.267814413	CCT6B	protein_coding
ENSG00000132153.15	5239.447119	0.080753631	0.043283549	1.865688758	0.062084923	0.204263851	DHX30	protein_coding
ENSG00000132155.13	2848.15879	0.095905688	0.049810652	1.925405208	0.054178685	0.187359849	RAF1	protein_coding
ENSG00000132164.10	33.65840823	1.418563074	0.264690106	5.359335462	8.35286E-08	7.02689E-06	SLC6A11	protein_coding
ENSG00000132170.23	2355.085398	0.127217697	0.111691163	1.139013095	0.254697693	0.481516761	PPARG	protein_coding
ENSG00000132182.12	5140.083151	0.048740562	0.135107739	0.360753297	0.718283879	0.852756705	NUP210	protein_coding
ENSG00000132185.18	275.2814248	-1.170807964	0.214392476	-5.461049682	4.73328E-08	4.40731E-06	FCRLA	protein_coding
ENSG00000132196.15	599.3640342	0.097874836	0.062007374	1.578438651	0.114464872	0.298488164	HSD17B7	protein_coding
ENSG00000132199.20	2224.753132	-0.272365947	0.065163605	-4.179724984	2.91862E-05	0.000747705	ENOSF1	protein_coding
ENSG00000132204.14	54.30012616	0.0064556	0.29302853	0.022030618	0.982423532	0.99233132	LINC00470	lncRNA
ENSG00000132205.11	1483.029957	0.202879636	0.087960032	2.30649797	0.021082823	0.100682323	EMILIN2	protein_coding

ENSG00000132207.18	2.232706099	-0.116617454	0.157970781	-0.738221673	0.460379755	0.673446411	SLX1A	protein_coding
ENSG00000132254.13	3227.145275	-0.044349097	0.053186075	-0.83384791	0.404366694	0.626060094	ARFIP2	protein_coding
ENSG00000132256.19	1182.958977	-0.089837096	0.059142576	-1.518991944	0.128764524	0.320785024	TRIM5	protein_coding
ENSG00000132259.13	11.6791212	-0.155014799	0.140857959	-1.10050437	0.271112427	0.499349026	CNGA4	protein_coding
ENSG00000132274.16	3672.050379	-0.268726343	0.11229521	-2.39303478	0.016709657	0.086298029	TRIM22	protein_coding
ENSG00000132275.11	965.9078521	-0.085033853	0.036998342	-2.29831526	0.021543853	0.102172748	RRP8	protein_coding
ENSG00000132286.12	2055.699694	-0.03880003	0.043139104	-0.899416682	0.368430757	0.595345229	TIMM10B	protein_coding
ENSG00000132294.15	6609.594087	-0.012867286	0.049422237	-0.260354177	0.794590586	0.897089848	EFR3A	protein_coding
ENSG00000132297.12	2.391297732	0.471361167	0.30449983	1.54798499	0.12162591	0.310037572	HHLA1	protein_coding
ENSG00000132300.19	4142.318488	-0.163796089	0.05302593	-3.088980973	0.002008443	0.019201826	PTCD3	protein_coding
ENSG00000132305.21	6369.887943	-0.091512742	0.040325582	-2.269347118	0.023247225	0.107650746	IMMT	protein_coding
ENSG00000132313.15	2359.47567	-0.175590246	0.046969875	-3.738358847	0.000185225	0.003214484	MRPL35	protein_coding
ENSG00000132321.17	70.27580916	0.5827676	0.13209581	4.411703893	1.0256E-05	0.000332669	IQCA1	protein_coding
ENSG00000132323.9	1144.4001	-0.079413367	0.040294248	-1.970836297	0.048742603	0.175049978	ILKAP	protein_coding
ENSG00000132326.12	2495.906196	0.163038818	0.070554034	2.310836237	0.020841902	0.099956091	PER2	protein_coding
ENSG00000132329.11	1649.153844	-0.010620818	0.151249864	-0.070220347	0.94401828	0.97369132	RAMP1	protein_coding
ENSG00000132330.18	211.7071588	-0.187075514	0.07100355	-2.634734664	0.008420308	0.053543229	SCLY	protein_coding
ENSG00000132334.17	2740.613901	0.040026793	0.078097471	0.512523553	0.608284641	0.78419087	PTPRE	protein_coding
ENSG00000132341.12	16071.11966	-0.065966771	0.057228302	-1.15269488	0.249035644	0.475703601	RAN	protein_coding
ENSG00000132356.11	7939.167086	0.039955758	0.062326014	0.64107674	0.521472826	0.721289361	PRKAA1	protein_coding
ENSG00000132357.14	1238.444784	0.200706609	0.099476828	2.017621716	0.043630673	0.162851722	CARD6	protein_coding
ENSG00000132359.15	2913.708273	0.236412395	0.080919068	2.921590679	0.003482489	0.028725424	RAP1GAP2	protein_coding
ENSG00000132361.18	10279.57255	-0.023777693	0.067779132	-0.350811407	0.725729839	0.857003121	CLUH	protein_coding
ENSG00000132376.20	1877.270857	0.146537265	0.048988373	2.991266224	0.002778232	0.024221064	INPP5K	protein_coding
ENSG00000132382.15	4342.777061	0.062385851	0.068228452	0.914367096	0.360523985	0.588343915	MYBBP1A	protein_coding
ENSG00000132383.12	4661.094261	0.040710641	0.045815062	0.888586397	0.374225405	0.600027623	RPA1	protein_coding
ENSG00000132386.11	5698.234426	0.365386622	0.12954551	2.820527112	0.004794482	0.036165683	SERPINF1	protein_coding
ENSG00000132388.12	3821.5133	0.007514481	0.042771337	0.17568965	0.860537767	0.932201115	UBE2G1	protein_coding
ENSG00000132394.11	1443.030045	0.026972073	0.051394761	0.524801998	0.599720843	0.778369816	EEFSEC	protein_coding
ENSG00000132405.19	1694.170441	0.027141694	0.070570517	0.384603871	0.700530926	0.842473745	TBC1D14	protein_coding
ENSG00000132406.12	925.1481195	-0.084149316	0.052289572	-1.609294407	0.107551981	0.287477301	TMEM128	protein_coding
ENSG00000132423.12	477.9088794	-0.067311409	0.068209255	-0.986836892	0.323722606	0.553470653	COQ3	protein_coding
ENSG00000132424.16	5558.004343	-0.065468094	0.067693724	-0.967122059	0.333483019	0.563044925	PNISR	protein_coding
ENSG00000132429.10	56.13075087	0.517068845	0.21676266	2.385414741	0.017059876	0.087389001	POPDC3	protein_coding
ENSG00000132432.14	3973.132455	0.046310975	0.077284958	0.599223664	0.549023744	0.74205694	SEC61G	protein_coding
ENSG00000132434.9	1795.304521	-0.095818475	0.080541502	-1.189678274	0.234172868	0.458796469	LANCL2	protein_coding
ENSG00000132436.11	1163.724598	-0.166333121	0.069956688	-2.377658605	0.017422948	0.088548963	FIGNL1	protein_coding
ENSG00000132437.18	1875.02245	-0.807209579	0.171037021	-4.719502108	2.36423E-06	0.000103232	DDC	protein_coding
ENSG00000132446.7	0.993246443	-0.636642925	0.58336972	-1.091319797	0.27513219	0.503933141	FTHL17	protein_coding
ENSG00000132463.14	5990.627256	0.038981315	0.048990346	0.795693809	0.426210026	0.644858666	GRSF1	protein_coding
ENSG00000132464.13	46.74892168	-0.042183983	0.209010597	-0.201827004	0.84005197	0.921546068	ENAM	protein_coding
ENSG00000132465.12	21459.02956	-1.213362372	0.201664815	-6.016728177	1.77978E-09	2.93778E-07	JCHAIN	protein_coding

ENSG00000132466.19	8697.178021	0.065002624	0.043859354	1.482069782	0.138321728	0.33492457	ANKRD17	protein_coding
ENSG00000132467.4	1440.974141	0.028328418	0.043128465	0.656838082	0.511285035	0.714228033	UTP3	protein_coding
ENSG00000132470.14	36701.31812	0.2990007	0.112868666	2.649102815	0.008070577	0.052087019	ITGB4	protein_coding
ENSG00000132471.12	7578.624808	0.063077766	0.054187817	1.164058085	0.244400473	0.470624809	WBP2	protein_coding
ENSG00000132475.10	25246.73183	0.041948882	0.045853176	0.914852253	0.360269199	0.588118993	H3-3B	protein_coding
ENSG00000132478.10	2030.427874	0.01805946	0.044084437	0.409656123	0.682058221	0.831931621	UNK	protein_coding
ENSG00000132481.7	2572.520781	0.123975183	0.083632544	1.48237967	0.1382393	0.334892928	TRIM47	protein_coding
ENSG00000132485.14	3666.960962	-0.046136492	0.052450477	-0.879620061	0.379065167	0.60474182	ZRANB2	protein_coding
ENSG00000132507.18	17369.53038	-0.051093692	0.081624712	-0.625958621	0.531342083	0.728964147	EIF5A	protein_coding
ENSG00000132510.10	4992.657697	0.152374012	0.058283332	2.614366847	0.0089393	0.055847611	KDM6B	protein_coding
ENSG00000132514.14	269.2056971	-0.32206218	0.160890554	-2.001746975	0.045311951	0.166995848	CLEC10A	protein_coding
ENSG00000132517.15	107.9872657	-0.260001832	0.166517143	-1.561411806	0.118426618	0.304842385	SLC52A1	protein_coding
ENSG00000132518.7	14.55650429	0.31526888	0.165461654	1.905389388	0.056729474	0.192936882	GUCY2D	protein_coding
ENSG00000132522.16	67.11279574	-0.031759928	0.069614777	-0.456223942	0.64822895	0.810176306	GPS2	protein_coding
ENSG00000132530.17	1131.499636	0.147698521	0.107252745	1.377107148	0.168479123	0.377818934	XAF1	protein_coding
ENSG00000132535.21	602.0676048	0.184242432	0.090465907	2.036595201	0.041690624	0.158156892	DLG4	protein_coding
ENSG00000132541.11	1189.639128	-0.403052466	0.07954653	-5.06687678	4.04396E-07	2.51389E-05	RIDA	protein_coding
ENSG00000132549.18	3408.071415	0.010828246	0.057284506	0.189025734	0.850072647	0.926437808	VPS13B	protein_coding
ENSG00000132554.20	31.38239062	0.031779363	0.172635863	0.184083208	0.853948179	0.928733648	RGS22	protein_coding
ENSG00000132561.14	2888.35804	0.041424566	0.144188646	0.28729423	0.773887041	0.885600724	MATN2	protein_coding
ENSG00000132563.16	283.0092385	-0.122979024	0.170888437	-0.71964509	0.471743542	0.682888808	REEP2	protein_coding
ENSG00000132570.15	879.0625968	-0.079200023	0.051866019	-1.527011789	0.12675808	0.317730046	PCBD2	protein_coding
ENSG00000132581.10	2149.336893	0.060226946	0.051606198	1.167048682	0.24319071	0.469249096	SDF2	protein_coding
ENSG00000132589.16	6062.609316	0.058575339	0.053730026	1.090178854	0.275634366	0.504404858	FLOT2	protein_coding
ENSG00000132591.12	3440.287637	-0.048411928	0.057598998	-0.840499478	0.400628394	0.62316677	ERAL1	protein_coding
ENSG00000132600.17	1626.716577	-0.056635133	0.048994568	-1.155947181	0.247702762	0.474229093	PRMT7	protein_coding
ENSG00000132603.15	1884.113361	-0.05366712	0.056643739	-0.947450158	0.343409442	0.573015567	NIP7	protein_coding
ENSG00000132604.11	2230.166112	-0.06904473	0.049137103	-1.405144501	0.159978313	0.365855417	TERF2	protein_coding
ENSG00000132612.16	2386.997612	-0.089608524	0.049234412	-1.820038467	0.068753147	0.217420602	VPS4A	protein_coding
ENSG00000132613.15	1621.957997	0.017064708	0.105622765	0.161562785	0.871650166	0.938046208	MTSS2	protein_coding
ENSG00000132622.11	519.4797363	0.027579148	0.09895111	0.278714895	0.780463631	0.88874039	HSPA12B	protein_coding
ENSG00000132623.16	1252.459453	-0.254029451	0.077424672	-3.280988401	0.00103444	0.011769658	ANKEF1	protein_coding
ENSG00000132631.6	0.374791332	0.017265225	0.515541973	0.033489466	0.973284266		SCP2D1	protein_coding
ENSG00000132635.17	1332.358078	-0.131482515	0.070598449	-1.862399488	0.062546801	0.205163866	PCED1A	protein_coding
ENSG00000132639.13	180.8322756	-0.612054258	0.203376315	-3.009466758	0.002617067	0.023231134	SNAP25	protein_coding
ENSG00000132640.15	3540.05651	-0.101259279	0.076438312	-1.324718933	0.185264391	0.39974581	BTBD3	protein_coding
ENSG00000132646.11	9422.436028	-0.327607682	0.079692545	-4.11089495	3.94128E-05	0.000951491	PCNA	protein_coding
ENSG00000132661.4	1430.264626	-0.185369202	0.06285733	-2.94904669	0.003187558	0.026882948	NXT1	protein_coding
ENSG00000132664.12	737.8089716	-0.10390084	0.056638976	-1.834440654	0.06658861	0.213029735	POLR3F	protein_coding
ENSG00000132669.13	4139.818204	0.188857604	0.06083638	3.104353072	0.001906956	0.018482969	RIN2	protein_coding
ENSG00000132670.20	5775.585625	-0.06374479	0.044199335	-1.442211495	0.149242719	0.351227724	PTPRA	protein_coding
ENSG00000132671.6	2.170065664	-0.362272798	0.308325522	-1.174968568	0.240007291	0.465476778	SSTR4	protein_coding

ENSG00000132676.16	5331.53753	-0.04575654	0.045976634	-0.995212929	0.319632715	0.549204106	DAP3	protein_coding
ENSG00000132677.13	47.81477465	-0.628213124	0.227139192	-2.765762785	0.005678983	0.040743009	RHBG	protein_coding
ENSG00000132680.11	2734.562287	0.16727657	0.058826641	2.843551268	0.004461383	0.034342641	KHDC4	protein_coding
ENSG00000132681.17	16.26065507	-0.743829985	0.189129721	-3.932909016	8.3924E-05	0.001732113	ATP1A4	protein_coding
ENSG00000132688.11	2360.351794	0.214459615	0.103536169	2.071349732	0.038326125	0.149720981	NES	protein_coding
ENSG00000132692.19	181.9878818	0.100083349	0.120003852	0.834001139	0.404280342	0.625948639	BCAN	protein_coding
ENSG00000132693.12	10.14903279	-2.441028664	0.308670246	-7.90820851	2.61119E-15	2.8447E-12	CRP	protein_coding
ENSG00000132694.19	6276.192536	0.090257213	0.046113132	1.957299562	0.050312259	0.178775153	ARHGEF11	protein_coding
ENSG00000132698.15	8290.321057	0.027344553	0.089135797	0.306774089	0.759015332	0.876647148	RAB25	protein_coding
ENSG00000132702.13	13.93497165	0.270429414	0.179713083	1.504784237	0.132379558	0.326173469	HAPLN2	protein_coding
ENSG00000132703.4	0.588710728	-0.438367629	0.636264174	-0.688971102	0.490841453		APCS	protein_coding
ENSG00000132704.16	149.2596738	-1.145508604	0.197348364	-5.804500126	6.45583E-09	8.39778E-07	FCRL2	protein_coding
ENSG00000132716.19	1934.64957	-0.068219568	0.045783223	-1.490056018	0.136209507	0.332061717	DCAF8	protein_coding
ENSG00000132718.9	1531.139343	-0.041054104	0.116557871	-0.352220777	0.724672696	0.856381345	SYT11	protein_coding
ENSG00000132740.10	1413.395864	-0.047800402	0.053685429	-0.89037944	0.373262178	0.59941207	IGHMBP2	protein_coding
ENSG00000132744.8	1016.288588	-0.137623524	0.13649382	-1.008276596	0.313321695	0.542640446	ACY3	protein_coding
ENSG00000132746.15	589.6191884	0.727433365	0.234326725	3.104355102	0.001906943	0.018482969	ALDH3B2	protein_coding
ENSG00000132749.11	555.8915263	0.01069909	0.111596246	0.095873207	0.923621274	0.963937521	TESMIN	protein_coding
ENSG00000132763.15	761.0047709	-0.113724145	0.062792954	-1.811097219	0.070125804	0.219946299	MMACHC	protein_coding
ENSG00000132768.14	1684.553007	-0.024408786	0.053334032	-0.457658743	0.647197633	0.809673823	DPH2	protein_coding
ENSG00000132773.12	823.0085669	-0.08649175	0.050710623	-1.705594315	0.088083639	0.254925996	TOE1	protein_coding
ENSG00000132780.17	4893.723592	-0.095089106	0.054926798	-1.731196952	0.083416643	0.246594332	NASP	protein_coding
ENSG00000132781.19	62.9063417	-0.145018786	0.065563722	-2.211875426	0.026975274	0.118881624	MUTYH	protein_coding
ENSG00000132792.19	2652.260689	-0.193153549	0.055429985	-3.484640115	0.000492799	0.006727669	CTNNBL1	protein_coding
ENSG00000132793.12	1596.082971	-0.131608092	0.082837649	-1.588747306	0.112117454	0.294801321	LPIN3	protein_coding
ENSG00000132801.7	327.1670109	-0.110536967	0.072652379	-1.521450074	0.128146931	0.319739984	ZSWIM3	protein_coding
ENSG00000132819.17	3424.175182	-0.033730199	0.086768701	-0.388736936	0.697470756	0.840533273	RBM38	protein_coding
ENSG00000132821.12	467.4199912	0.973776341	0.216676118	4.494156311	6.98463E-06	0.00024467	VSTM2L	protein_coding
ENSG00000132823.11	2265.799254	-0.088918942	0.05438225	-1.635072871	0.102033772	0.278731551	OSER1	protein_coding
ENSG00000132824.14	11624.65359	0.097607998	0.048374681	2.017749685	0.043617337	0.162829839	SERINC3	protein_coding
ENSG00000132825.7	904.1864456	0.124473627	0.065803889	1.89158467	0.05854634	0.196705772	PPP1R3D	protein_coding
ENSG00000132832.10	4.565173991	-0.354920104	0.240339481	-1.476744906	0.13974403	0.336978813	AL139352.1	lncRNA
ENSG00000132837.15	45.60520735	-0.08649739	0.123650501	-0.699531251	0.48422009	0.692149146	DMGDH	protein_coding
ENSG00000132840.10	194.9271473	-0.135768804	0.156132459	-0.86957449	0.384532984	0.609379117	BHMT2	protein_coding
ENSG00000132842.14	4001.40659	0.03073482	0.047927708	0.641274571	0.521344308	0.721180271	AP3B1	protein_coding
ENSG00000132846.6	1663.533508	-0.159997694	0.068816665	-2.324984712	0.020072778	0.097471744	ZBED3	protein_coding
ENSG00000132849.21	4525.395008	-0.011169046	0.064222405	-0.173911992	0.861934628	0.932805719	PATJ	protein_coding
ENSG00000132854.19	196.9105097	0.104724017	0.192203474	0.54486017	0.585849692	0.76814209	KANK4	protein_coding
ENSG00000132855.5	38.31022531	-0.705152546	0.179658968	-3.924950437	8.67476E-05	0.001778918	ANGPTL3	protein_coding
ENSG00000132872.12	26.19676708	0.092360282	0.275185902	0.335628683	0.737150891	0.863841887	SYT4	protein_coding
ENSG00000132874.15	44.31195895	-0.273404152	0.217194232	-1.258800245	0.208102492	0.428101888	SLC14A2	protein_coding
ENSG00000132879.14	807.7123722	-0.038752455	0.096205506	-0.402809116	0.687088654	0.83485051	FBXO44	protein_coding

ENSG00000132881.12	236.4517893	-0.012036799	0.071052316	-0.169407558	0.865476079	0.934739048	CPLANE2	protein_coding
ENSG00000132906.18	901.866679	0.034902896	0.055956038	0.623755673	0.532788055	0.730195781	CASP9	protein_coding
ENSG00000132911.5	359.456373	-0.510867137	0.280213094	-1.823137994	0.068282493	0.216537054	NMUR2	protein_coding
ENSG00000132912.13	4294.352683	0.023325341	0.048955264	0.476462364	0.633745014	0.801364608	DCTN4	protein_coding
ENSG00000132915.11	48.78236598	-0.136535058	0.141500677	-0.964907454	0.334591167	0.564041753	PDE6A	protein_coding
ENSG00000132932.17	78.32039721	0.050382392	0.115249006	0.437161187	0.661994456	0.818926777	ATP8A2	protein_coding
ENSG00000132938.21	46.29221499	-0.092189156	0.174697073	-0.527708645	0.597701572	0.776909714	MTUS2	protein_coding
ENSG00000132950.19	800.5504497	0.061460583	0.06212292	0.989338288	0.322497657	0.552351546	ZMYM5	protein_coding
ENSG00000132952.12	1674.660664	0.038879915	0.054462766	0.713880666	0.475300974	0.685516335	USPL1	protein_coding
ENSG00000132953.16	3256.58608	0.026332832	0.065315657	0.403162623	0.686828594	0.834656004	XPO4	protein_coding
ENSG00000132958.17	14.52278136	-0.299309027	0.174175967	-1.71842897	0.0857184	0.250670796	TPTE2	protein_coding
ENSG00000132963.8	7499.193696	-0.056084741	0.066567222	-0.842527902	0.399492529	0.622272159	POMP	protein_coding
ENSG00000132964.12	1579.072166	-0.001439181	0.064539376	-0.022299274	0.982209228	0.992260457	CDK8	protein_coding
ENSG00000132965.10	1568.473617	0.063556769	0.123160471	0.516048438	0.605820579	0.78269353	ALOX5AP	protein_coding
ENSG00000132967.9	588.4627963	-0.084617638	0.086770764	-0.975186042	0.329467985	0.558951427	HMGB1P5	transcribed_processed_pseudogene
ENSG00000132970.14	369.9525556	-0.093178081	0.162425045	-0.573668185	0.566192362	0.75438649	WASF3	protein_coding
ENSG00000132972.19	9.3517782	-0.684503286	0.269185314	-2.542870098	0.01099461	0.064492139	RNF17	protein_coding
ENSG00000132975.8	3.793133219	0.154457961	0.259896947	0.59430464	0.552308376	0.744146866	GPR12	protein_coding
ENSG00000133019.12	1208.84728	-0.096647345	0.162947874	-0.593118172	0.55310207	0.744623763	CHRM3	protein_coding
ENSG00000133020.4	5.624770984	-0.542354998	0.240595628	-2.254218006	0.024182457	0.110495849	MYH8	protein_coding
ENSG00000133026.13	3450.434216	0.424486214	0.112297511	3.780014443	0.000156819	0.002825796	MYH10	protein_coding
ENSG00000133027.18	838.9295284	-0.021410887	0.072222275	-0.296458216	0.766880172	0.881703796	PEMT	protein_coding
ENSG00000133028.12	2362.085776	-0.121859248	0.050124651	-2.431124111	0.015052058	0.080312294	SCO1	protein_coding
ENSG00000133030.22	10672.00476	0.205981823	0.053721212	3.834273535	0.000125936	0.002356338	MPRIP	protein_coding
ENSG00000133048.13	2927.9144	-0.111609548	0.166622079	-0.669836488	0.502962031	0.707647512	CHI3L1	protein_coding
ENSG00000133055.9	5.492014943	-0.318263278	0.173401318	-1.835414418	0.066444309	0.212821288	MYBPH	protein_coding
ENSG00000133056.13	5327.286185	-0.048596332	0.060453238	-0.803866494	0.421474082	0.641000108	PIK3C2B	protein_coding
ENSG00000133059.17	1345.108171	0.081108667	0.050973285	1.591199541	0.111564676	0.293915355	DSTYK	protein_coding
ENSG00000133063.16	573.1660925	-1.526990331	0.236077728	-6.468167696	9.91984E-11	2.73593E-08	CHIT1	protein_coding
ENSG00000133065.11	2399.859214	0.030251024	0.060249592	0.502095087	0.615600636	0.789115485	SLC41A1	protein_coding
ENSG00000133067.17	976.0235604	0.249114554	0.216081723	1.152871933	0.248962954	0.475627296	LGR6	protein_coding
ENSG00000133069.17	149.4439552	0.171733589	0.10449645	1.643439462	0.100292041	0.276001082	TMCC2	protein_coding
ENSG00000133083.15	496.8585601	-0.007510467	0.170307039	-0.044099567	0.964825038	0.984062284	DCLK1	protein_coding
ENSG00000133101.10	11.14242268	0.415411409	0.185659953	2.237485261	0.025254642	0.113866685	CCNA1	protein_coding
ENSG00000133103.17	2176.079032	0.195765098	0.058103471	3.369249635	0.000753731	0.009273108	COG6	protein_coding
ENSG00000133104.14	1450.037547	0.117025045	0.115902795	1.009682683	0.312647342	0.542171723	SPART	protein_coding
ENSG00000133105.8	4.24891188	0.127636951	0.36390604	0.350741503	0.725782287	0.857032366	RXFP2	protein_coding
ENSG00000133106.15	3246.374368	-0.088853194	0.110503202	-0.804077999	0.42135193	0.640921716	EPSTI1	protein_coding
ENSG00000133107.15	180.1562237	-0.36219253	0.151694013	-2.387652102	0.016956385	0.087113802	TRPC4	protein_coding
ENSG00000133110.15	17028.3729	0.574377347	0.129098747	4.449131846	8.62181E-06	0.000287462	POSTN	protein_coding
ENSG00000133111.4	424.5727747	0.070979499	0.069324495	1.023873284	0.305895131	0.535448568	RFXAP	protein_coding
ENSG00000133112.17	76439.48683	-0.134214742	0.071382392	-1.880221969	0.060077833	0.19998622	TPT1	protein_coding

ENSG00000133114.18	1489.813602	0.003086702	0.057262932	0.053904015	0.957011637	0.979926127	GPALPP1	protein_coding
ENSG00000133115.12	14.17651373	-0.281962031	0.246379859	-1.144419971	0.25244951	0.479220783	STOML3	protein_coding
ENSG00000133116.8	182.6083258	-0.011486701	0.144226774	-0.079643333	0.93652093	0.969859815	KL	protein_coding
ENSG00000133119.13	1996.895859	0.012707647	0.086605761	0.146729814	0.883345285	0.943960371	RFC3	protein_coding
ENSG00000133121.21	1771.329929	-0.326678316	0.09135913	-3.575759945	0.000349212	0.005209726	STARD13	protein_coding
ENSG00000133124.11	19.2994006	-0.111393783	0.270903301	-0.411193892	0.680930367	0.83095636	IRS4	protein_coding
ENSG00000133131.15	2276.35133	-0.115816771	0.089607167	-1.292494508	0.196185947	0.413324067	MORC4	protein_coding
ENSG00000133134.12	209.4489874	0.283028673	0.22650567	1.249543434	0.211466378	0.431875851	BEX2	protein_coding
ENSG00000133135.14	4248.735916	-0.230943509	0.120399149	-1.91814902	0.055092118	0.189333536	RNF128	protein_coding
ENSG00000133136.4	27.69184789	-0.233089413	0.123603154	-1.885788557	0.059323453	0.198475097	GNG5P2	processed_pseudogene
ENSG00000133138.20	1275.663771	0.170998323	0.080993038	2.111271878	0.03474895	0.140090362	TBC1D8B	protein_coding
ENSG00000133142.18	2499.164892	0.105847323	0.081298559	1.301958165	0.192930687	0.409295582	TCEAL4	protein_coding
ENSG00000133169.6	187.0778183	-1.157080831	0.293177766	-3.946686834	7.92401E-05	0.001654037	BEX1	protein_coding
ENSG00000133193.12	1202.870797	0.01738673	0.045371075	0.383211777	0.701562746	0.843063172	FAM104A	protein_coding
ENSG00000133195.11	2546.539086	0.130078518	0.082401497	1.578594117	0.114429185	0.298457406	SLC39A11	protein_coding
ENSG00000133216.17	6391.231787	-0.292393912	0.144533293	-2.023021169	0.043070965	0.16169051	EPHB2	protein_coding
ENSG00000133226.17	8854.341023	-0.096182165	0.05088669	-1.890124213	0.05874135	0.197178539	SRRM1	protein_coding
ENSG00000133243.10	5888.282459	-0.040200541	0.054593879	-0.736356193	0.461513957	0.674112935	BTBD2	protein_coding
ENSG00000133246.12	169.0893457	0.076062889	0.112035988	0.678914784	0.497191858	0.703311503	PRAM1	protein_coding
ENSG00000133247.14	1026.263889	0.121024965	0.073099213	1.65562611	0.097797559	0.271817843	KMT5C	protein_coding
ENSG00000133250.14	1050.458916	-0.061816751	0.057158396	-1.081499045	0.279475185	0.50810199	ZNF414	protein_coding
ENSG00000133256.13	119.7424169	0.070603989	0.143930158	0.490543397	0.623749429	0.794678697	PDE6B	protein_coding
ENSG00000133265.11	3417.779489	0.003369114	0.073115717	0.046079207	0.963247119	0.983076952	HSPBP1	protein_coding
ENSG00000133275.16	4633.136821	0.074966465	0.049231149	1.522744567	0.127822623	0.319296444	CSNK1G2	protein_coding
ENSG00000133302.13	491.3383722	-0.099010361	0.054583037	-1.813940138	0.069686942	0.219125042	SLF1	protein_coding
ENSG00000133313.15	9276.974202	-0.067876846	0.070313958	-0.965339577	0.334374755	0.563735477	CNDP2	protein_coding
ENSG00000133315.12	1457.27419	-0.113779824	0.096498041	-1.179089464	0.238362553	0.463801181	MACROD1	protein_coding
ENSG00000133316.15	1233.981296	0.006533346	0.058334678	0.111997629	0.910825287	0.957841438	WDR74	protein_coding
ENSG00000133317.16	26.12496089	0.270196752	0.18123162	1.490891884	0.135989882	0.331711789	LGALS12	protein_coding
ENSG00000133318.14	9500.684885	0.101169734	0.048990613	2.06508407	0.038915046	0.151326298	RTN3	protein_coding
ENSG00000133321.11	5596.975438	0.130311097	0.160991241	0.809429736	0.418268004	0.638486174	PLAAT4	protein_coding
ENSG00000133328.4	272.8892607	-0.944094509	0.192862384	-4.895171833	9.82199E-07	5.1259E-05	PLAAT2	protein_coding
ENSG00000133392.18	97119.46834	-0.6721419	0.25107221	-2.677086002	0.007426557	0.049034405	MYH11	protein_coding
ENSG00000133393.13	2087.540646	-0.106371177	0.053204994	-1.999270555	0.045579088	0.167654252	CEP20	protein_coding
ENSG00000133398.4	1357.386814	-0.069848347	0.05942621	-1.17537947	0.239842933	0.465304843	MED10	protein_coding
ENSG00000133401.16	930.6208807	0.316453723	0.1124671	2.813744831	0.004896808	0.036677244	PDZD2	protein_coding
ENSG00000133422.14	2646.963546	0.191401135	0.047001556	4.072229745	4.65652E-05	0.001085119	MORC2	protein_coding
ENSG00000133424.22	1923.200353	-0.138649865	0.076159502	-1.820519578	0.068679918	0.21726786	LARGE1	protein_coding
ENSG00000133433.11	61.07922164	0.437398811	0.15290592	2.860574733	0.004228739	0.03295346	GSTT2B	protein_coding
ENSG00000133454.16	114.5885609	0.930258065	0.244474404	3.805134813	0.000141727	0.002600174	MYO18B	protein_coding
ENSG00000133460.19	336.2510553	-0.27668531	0.068922688	-4.014430042	5.95896E-05	0.001321494	SLC2A11	protein_coding
ENSG00000133466.14	1139.567884	0.561788945	0.093733147	5.993492847	2.05381E-09	3.34195E-07	C1QTNF6	protein_coding

ENSG00000133475.17	6.206788515	-0.313383281	0.207617871	-1.509423439	0.131190607	0.324466474	GGT2	protein_coding
ENSG00000133477.17	1580.411814	0.143094305	0.119764045	1.194801878	0.232164446	0.456421099	FAM83F	protein_coding
ENSG00000133488.15	129.4500108	-0.438444226	0.230567599	-1.901586471	0.057225238	0.194107901	SEC14L4	protein_coding
ENSG00000133519.12	108.8740912	-0.080273652	0.207245079	-0.387336828	0.698506865	0.841437886	ZDHHC8P1	transcribed_unprocessed_pseudogene
ENSG00000133561.15	1226.996493	-0.257923689	0.101289576	-2.546399135	0.010884066	0.064076594	GIMAP6	protein_coding
ENSG00000133574.10	1631.370805	-0.33572226	0.097635532	-3.438525436	0.000584892	0.007651702	GIMAP4	protein_coding
ENSG00000133597.11	1524.235038	-0.23571783	0.065808553	-3.581872243	0.000341141	0.005115088	ADCK2	protein_coding
ENSG00000133606.11	5487.01987	-0.079472564	0.045301691	-1.754295745	0.079379844	0.239006114	MKRN1	protein_coding
ENSG00000133612.19	4037.345827	-0.067655557	0.053633473	-1.261442777	0.207149364	0.42702814	AGAP3	protein_coding
ENSG00000133619.17	533.107013	0.013871538	0.101309144	0.136922859	0.89109177	0.948048196	KRBA1	protein_coding
ENSG00000133624.13	480.4088609	-0.19185751	0.065818256	-2.914958901	0.003557354	0.029144352	ZNF767P	transcribed_unprocessed_pseudogene
ENSG00000133627.18	448.4444422	-0.251440443	0.065686695	-3.827874776	0.000129254	0.00240912	ACTR3B	protein_coding
ENSG00000133636.11	170.3134234	-1.023222077	0.26676133	-3.835721151	0.000125196	0.00234653	NTS	protein_coding
ENSG00000133639.6	7526.977349	-0.279078142	0.065787058	-4.242143542	2.21395E-05	0.000604494	BTG1	protein_coding
ENSG00000133640.20	55.26820282	0.657303959	0.21220708	3.097464793	0.001951836	0.018780117	LRRIQ1	protein_coding
ENSG00000133641.18	975.1400317	-0.04245275	0.055084291	-0.770687057	0.440892446	0.657453722	C12orf29	protein_coding
ENSG00000133657.16	10648.25786	0.031019891	0.065188539	0.475848839	0.634182073	0.801477689	ATP13A3	protein_coding
ENSG00000133661.17	22.93947232	0.2016967	0.174970829	1.152744724	0.249015179	0.475700754	SFTPD	protein_coding
ENSG00000133665.13	16.14471426	-0.196621822	0.243140702	-0.808675059	0.418702078	0.638789476	DYDC2	protein_coding
ENSG00000133678.14	1306.087565	-0.030176769	0.067286064	-0.448484687	0.653803435	0.813908893	TMEM254	protein_coding
ENSG00000133687.16	1492.512805	0.095052416	0.137591848	0.690828832	0.489673114	0.697017224	TMTC1	protein_coding
ENSG00000133703.13	5698.141157	0.560392005	0.110814791	5.057014497	4.25871E-07	2.61751E-05	KRAS	protein_coding
ENSG00000133704.10	3996.313388	0.122078064	0.057335037	2.129205272	0.033237281	0.136222882	IPO8	protein_coding
ENSG00000133706.19	6176.381429	-0.125015638	0.044838159	-2.788152767	0.005300954	0.038783573	LARS1	protein_coding
ENSG00000133710.16	942.3191478	-0.413268556	0.198776062	-2.079066015	0.037611283	0.147869622	SPINK5	protein_coding
ENSG00000133731.10	2055.05414	-0.084120253	0.073675223	-1.14177127	0.253549107	0.480366482	IMPA1	protein_coding
ENSG00000133739.16	1718.00976	-0.18564773	0.072432635	-2.563039835	0.010376014	0.061888253	LRRCC1	protein_coding
ENSG00000133740.11	705.6530293	-0.13943112	0.076915402	-1.812785443	0.06986492	0.219518611	E2F5	protein_coding
ENSG00000133742.14	124.1233618	-0.708563881	0.231273197	-3.063752698	0.002185795	0.020403054	CA1	protein_coding
ENSG00000133773.12	1393.848925	-0.169391224	0.055824712	-3.034341212	0.002410616	0.021854163	CCDC59	protein_coding
ENSG00000133789.15	3353.133849	-0.126482181	0.068071127	-1.858088541	0.063156442	0.206247622	SWAP70	protein_coding
ENSG00000133794.20	751.1157619	0.018337799	0.060689315	0.302158602	0.762531161	0.878716408	ARNTL	protein_coding
ENSG00000133800.9	331.4858171	0.142854568	0.149120904	0.957978146	0.338073779	0.567913383	LYVE1	protein_coding
ENSG00000133805.15	1000.606209	0.193320227	0.078186916	2.47253934	0.013415695	0.074161579	AMPD3	protein_coding
ENSG00000133812.16	2420.722888	0.036927844	0.058365591	0.632698881	0.526930301	0.72551987	SBF2	protein_coding
ENSG00000133816.17	9022.826644	0.153352517	0.084129188	1.822821783	0.068330387	0.216602407	MICAL2	protein_coding
ENSG00000133818.14	1295.516559	0.032541833	0.085049687	0.382621436	0.702000474	0.84333337	RRAS2	protein_coding
ENSG00000133835.17	5405.543683	-0.121672348	0.058844419	-2.067695622	0.038668654	0.150761737	HSD17B4	protein_coding
ENSG00000133858.17	2996.431402	-0.054137842	0.057084621	-0.948378745	0.342936676	0.572770362	ZFC3H1	protein_coding
ENSG00000133863.9	81.39347345	0.588476476	0.276140251	2.131078225	0.033082698	0.135812034	TEX15	protein_coding
ENSG00000133872.14	12251.00271	-0.074567432	0.054592324	-1.365895914	0.171971636	0.382581579	SARAF	protein_coding
ENSG00000133874.2	523.7459978	-0.016953067	0.084774831	-0.199977596	0.841498103	0.922035912	RNF122	protein_coding

ENSG00000133878.9	56.98066971	-0.007519817	0.182137195	-0.041286552	0.967067454	0.984565164	DUSP26	protein_coding
ENSG00000133884.10	3551.198088	-0.002615195	0.041287231	-0.063341498	0.949494571	0.976381644	DPF2	protein_coding
ENSG00000133895.17	2949.925271	0.037051122	0.05919798	0.625884908	0.531390435	0.728991058	MEN1	protein_coding
ENSG00000133935.7	3125.83602	0.016608183	0.071112137	0.2335492	0.815334969	0.908808082	ERG28	protein_coding
ENSG00000133937.5	53.21110099	0.833598959	0.189740537	4.393362498	1.11611E-05	0.000354753	GSC	protein_coding
ENSG00000133943.21	2140.034566	-0.123982746	0.063586536	-1.949827019	0.05119674	0.180618551	DGLUCY	protein_coding
ENSG00000133958.13	57.47456814	-0.126521001	0.109281373	-1.157754489	0.246964241	0.473448626	UNC79	protein_coding
ENSG00000133961.20	5363.212296	0.043518822	0.038999121	1.115892374	0.264468207	0.492273262	NUMB	protein_coding
ENSG00000133962.8	338.7084273	0.345102698	0.158714763	2.174357898	0.029678267	0.126681346	CATSPERB	protein_coding
ENSG00000133980.5	84.56867859	-0.91606058	0.227220363	-4.031595445	5.53995E-05	0.001244404	VRTN	protein_coding
ENSG00000133983.16	286.3144301	-0.026466757	0.076647528	-0.345304769	0.729865262	0.85931649	COX16	protein_coding
ENSG00000133985.3	1068.838802	0.326469172	0.147885693	2.207577799	0.027273718	0.119753738	TTC9	protein_coding
ENSG00000133997.12	836.632852	-0.012618984	0.040755773	-0.30962445	0.756846561	0.875253294	MED6	protein_coding
ENSG00000134001.15	4243.859911	0.026240803	0.049405517	0.531131034	0.59532798	0.775169791	EIF2S1	protein_coding
ENSG00000134007.5	6.321584313	-0.294240354	0.163369168	-1.801076412	0.071690837	0.223179854	ADAM20	protein_coding
ENSG00000134013.16	4225.656397	0.361831716	0.093940163	3.851725464	0.000117288	0.002237776	LOXL2	protein_coding
ENSG00000134014.17	1691.564331	-0.051181551	0.05400627	-0.947696457	0.343284005	0.572955191	ELP3	protein_coding
ENSG00000134020.8	13.70019334	-0.248255688	0.190531279	-1.302965524	0.192586532	0.408844122	PEBP4	protein_coding
ENSG00000134028.14	952.8169888	-0.484189271	0.14945101	-3.239785866	0.001196195	0.013169962	ADAMDEC1	protein_coding
ENSG00000134030.14	2083.885235	-0.058987827	0.07980584	-0.739141732	0.459820938	0.673058178	CTIF	protein_coding
ENSG00000134042.14	72.76881963	-0.329135079	0.127460861	-2.582244271	0.009816008	0.059665873	MRO	protein_coding
ENSG00000134046.12	4642.613699	-0.153590453	0.050342056	-3.050937251	0.002281282	0.021026109	MBD2	protein_coding
ENSG00000134049.6	1902.695028	-0.084665477	0.062598613	-1.352513624	0.176211063	0.388334948	IER3IP1	protein_coding
ENSG00000134056.12	1373.191973	-0.150429252	0.061378539	-2.450844445	0.014252153	0.077343222	MRPS36	protein_coding
ENSG00000134057.15	3283.421135	-0.032774778	0.090227929	-0.363244267	0.716422423	0.851883151	CCNB1	protein_coding
ENSG00000134058.12	1294.854408	-0.048283479	0.056899707	-0.84857167	0.396119677	0.619431842	CDK7	protein_coding
ENSG00000134061.5	570.616803	-0.756368283	0.144407893	-5.237721192	1.62571E-07	1.20278E-05	CD180	protein_coding
ENSG00000134070.5	1404.677648	0.131184178	0.106961422	1.226462541	0.220024663	0.441925459	IRAK2	protein_coding
ENSG00000134072.11	114.3138285	-0.1248461	0.078405301	-1.592317092	0.111313474	0.293439745	CAMK1	protein_coding
ENSG00000134077.16	1643.233738	-0.009120391	0.04545287	-0.200655987	0.840967577	0.921927096	THUMPD3	protein_coding
ENSG00000134086.8	2832.316422	-0.006673274	0.053366547	-0.125046011	0.900487125	0.952509344	VHL	protein_coding
ENSG00000134107.5	11098.46606	0.589714573	0.085756946	6.876580849	6.13061E-12	2.75416E-09	BHLHE40	protein_coding
ENSG00000134108.14	5277.653864	0.074642361	0.042906952	1.739633261	0.081923435	0.243800965	ARL8B	protein_coding
ENSG00000134109.11	3512.180391	0.063306834	0.064715955	0.978226055	0.327962532	0.557482574	EDEM1	protein_coding
ENSG00000134115.13	7.566397447	0.086143626	0.227622794	0.37844903	0.705097051	0.845451369	CNTN6	protein_coding
ENSG00000134121.10	455.5834982	0.240026461	0.159954614	1.500591044	0.133461369	0.327930425	CHL1	protein_coding
ENSG00000134138.20	1311.674928	-0.099680925	0.107731606	-0.925270945	0.354824988	0.582885683	MEIS2	protein_coding
ENSG00000134146.12	546.6088188	-0.170203136	0.056977385	-2.987205133	0.002815407	0.02446401	DPH6	protein_coding
ENSG00000134152.11	1287.864476	-0.019673399	0.046909099	-0.419394089	0.674928143	0.827435353	KATNBL1	protein_coding
ENSG00000134153.10	2924.725598	-0.00654559	0.049964704	-0.131004287	0.895771916	0.950498453	EMC7	protein_coding
ENSG00000134160.14	7.361532726	-0.138773191	0.22803275	-0.60856693	0.54281153	0.737738142	TRPM1	protein_coding
ENSG00000134183.12	45.24931417	0.099614382	0.099755226	0.998588104	0.317994265	0.547370678	GNAT2	protein_coding

ENSG00000134184.13	594.3762273	-0.513660071	0.331403442	-1.549953941	0.121152571	0.309340811	GSTM1	protein_coding
ENSG00000134186.12	4666.432887	-0.101029686	0.04871675	-2.073818271	0.038096192	0.14915703	PRPF38B	protein_coding
ENSG00000134193.15	38821.92472	0.31734459	0.264737501	1.198714154	0.230639112	0.45459137	REG4	protein_coding
ENSG00000134198.10	985.3155289	-0.420486051	0.152975157	-2.748721165	0.005982826	0.042234505	TSPAN2	protein_coding
ENSG00000134200.4	2.240506291	-0.814831167	0.257276462	-3.167142309	0.00153945	0.015806927	TSHB	protein_coding
ENSG00000134201.12	202.6200181	-0.068992174	0.156108907	-0.441948988	0.658526121	0.816775566	GSTM5	protein_coding
ENSG00000134202.11	2241.466009	-0.658910633	0.125457451	-5.252064556	1.50404E-07	1.12614E-05	GSTM3	protein_coding
ENSG00000134207.17	15.79074889	-0.174824067	0.171678015	-1.018325306	0.308523386	0.537948707	SYT6	protein_coding
ENSG00000134215.16	1367.90491	-0.274639467	0.161409124	-1.701511412	0.088846997	0.256283724	VAV3	protein_coding
ENSG00000134216.19	14.98468277	-0.803827457	0.435226334	-1.846918244	0.06475899	0.209456136	CHIA	protein_coding
ENSG00000134222.16	557.4961749	-0.120701807	0.094207234	-1.281237139	0.200110385	0.418536609	PSRC1	protein_coding
ENSG00000134240.12	7488.806719	-0.46325737	0.238578991	-1.941735805	0.052169092	0.182688244	HMGCS2	protein_coding
ENSG00000134242.16	608.5971798	-0.404117113	0.110394789	-3.660653883	0.000251572	0.00406179	PTPN22	protein_coding
ENSG00000134243.12	9242.321497	0.070744448	0.069082201	1.024061866	0.305806055	0.535384265	SORT1	protein_coding
ENSG00000134245.18	790.2419691	0.004091324	0.132274691	0.030930516	0.975324953	0.988550686	WNT2B	protein_coding
ENSG00000134247.10	7456.569432	0.150045832	0.070604132	2.125170682	0.033572373	0.137048877	PTGFRN	protein_coding
ENSG00000134248.14	2936.930657	-0.057001004	0.058199802	-0.979402033	0.327381373	0.557016168	LAMTOR5	protein_coding
ENSG00000134249.7	3.086697553	0.109312529	0.344916796	0.316924344	0.75130101	0.872360729	ADAM30	protein_coding
ENSG00000134250.20	9127.843569	0.292240028	0.073249467	3.989653977	6.61698E-05	0.00143958	NOTCH2	protein_coding
ENSG00000134253.10	227.2303233	0.075643219	0.074432375	1.016267707	0.309501922	0.538904593	TRIM45	protein_coding
ENSG00000134255.14	1381.875551	-0.084913737	0.04916321	-1.727180476	0.084135256	0.247802401	CEPT1	protein_coding
ENSG00000134256.12	176.2139373	-0.342139836	0.095285672	-3.590674534	0.000329823	0.004993624	CD101	protein_coding
ENSG00000134258.17	240.7504401	0.82650342	0.232133939	3.56045921	0.000370207	0.005439144	VTCN1	protein_coding
ENSG00000134259.5	29.99308744	0.231089504	0.155318882	1.487839089	0.136793334	0.3328704	NGF	protein_coding
ENSG00000134262.13	872.3222855	-0.240577943	0.05634508	-4.26972402	1.95715E-05	0.000547763	AP4B1	protein_coding
ENSG00000134265.13	2403.397047	0.174880886	0.064200194	2.72399311	0.006449785	0.044464846	NAPG	protein_coding
ENSG00000134278.15	995.1845543	0.422086986	0.10972746	3.846685108	0.000119727	0.00227831	SPIRE1	protein_coding
ENSG00000134283.18	2546.962714	-0.031335327	0.041063842	-0.763088051	0.445410886	0.661203064	PPHLN1	protein_coding
ENSG00000134285.11	1806.088855	-0.141245766	0.081542462	-1.732174413	0.083242513	0.246213195	FKBP11	protein_coding
ENSG00000134287.10	8827.026582	-0.018287071	0.047255211	-0.386985288	0.698767099	0.84153879	ARF3	protein_coding
ENSG00000134291.12	5352.403475	-0.216431089	0.071522928	-3.026037864	0.002477813	0.022304409	TMEM106C	protein_coding
ENSG00000134294.14	10220.34394	0.165481565	0.060057502	2.755385432	0.005862303	0.04164003	SLC38A2	protein_coding
ENSG00000134297.6	159.0000235	-0.070640536	0.061809084	-1.142882741	0.253087279	0.47990881	PLEKHA8P1	transcribed_processed_pseudogene
ENSG00000134308.14	17619.2108	0.019426978	0.042246684	0.459846215	0.645626608	0.808749846	YWHAQ	protein_coding
ENSG00000134313.16	5024.832646	0.033561513	0.045290439	0.741028664	0.458676061	0.671969296	KIDINS220	protein_coding
ENSG00000134317.18	734.062747	0.606985446	0.123147349	4.928936356	8.26785E-07	4.49798E-05	GRHL1	protein_coding
ENSG00000134318.14	6120.189049	-0.01617862	0.05679314	-0.284869271	0.7757443	0.886356674	ROCK2	protein_coding
ENSG00000134321.12	1402.520434	0.198684156	0.142459687	1.394669328	0.163115578	0.370155056	RSAD2	protein_coding
ENSG00000134323.12	374.3487991	0.072611811	0.182467844	0.397943055	0.690672162	0.836922886	MYCN	protein_coding
ENSG00000134324.12	3172.259256	-0.039898163	0.075281372	-0.529987191	0.596120812	0.77561207	LPIN1	protein_coding
ENSG00000134326.11	1499.603357	0.162180309	0.110392624	1.469122683	0.141799516	0.340133072	CMPK2	protein_coding
ENSG00000134330.19	1416.637523	-0.039843006	0.052808274	-0.754484159	0.450558554	0.665353128	IAH1	protein_coding

ENSG00000134333.14	54321.47715	0.029011316	0.076665832	0.378412587	0.705124119	0.845454373	LDHA	protein_coding
ENSG00000134339.9	342.3798669	0.040100017	0.244466182	0.164030939	0.869706788	0.936939746	SAA2	protein_coding
ENSG00000134343.14	61.31521889	1.275065639	0.228563088	5.578615729	2.4244E-08	2.48847E-06	ANO3	protein_coding
ENSG00000134352.20	10260.12218	-0.101462019	0.081241016	-1.248901408	0.211701136	0.432179351	IL6ST	protein_coding
ENSG00000134363.12	417.8910102	0.463952957	0.170030473	2.728645921	0.006359495	0.044030458	FST	protein_coding
ENSG00000134365.13	8.242065337	0.236487069	0.325073704	0.72748754	0.466927345	0.678446632	CFHR4	protein_coding
ENSG00000134369.15	3698.881163	0.466394095	0.08129706	5.736912195	9.64182E-09	1.16067E-06	NAV1	protein_coding
ENSG00000134371.14	3374.546084	0.096121382	0.040542788	2.37086267	0.017746624	0.089632659	CDC73	protein_coding
ENSG00000134375.11	3473.955976	0.069843892	0.055462875	1.259290867	0.207925291	0.427919165	TIMM17A	protein_coding
ENSG00000134376.16	15.04241304	-0.094122276	0.198392383	-0.474424848	0.635196977	0.802108694	CRB1	protein_coding
ENSG00000134389.10	2.630643016	-0.090547332	0.405384623	-0.223361536	0.823254138	0.912941408	CFHR5	protein_coding
ENSG00000134398.15	4972.67813	0.407916691	0.157863881	2.583977348	0.009766821	0.059450867	ERN2	protein_coding
ENSG00000134419.15	6370.039682	-0.242331192	0.071189584	-3.404025952	0.000664004	0.008396785	RPS15A	protein_coding
ENSG00000134438.10	2.58548512	-0.252257603	0.269918762	-0.934568613	0.350010661	0.579131376	RAX	protein_coding
ENSG00000134440.12	10720.46151	-0.135323681	0.061255904	-2.20915326	0.027163983	0.119447515	NARS1	protein_coding
ENSG00000134443.10	48.2760874	0.169447504	0.269469975	0.628817755	0.529468375	0.727614109	GRP	protein_coding
ENSG00000134444.15	2419.02376	-0.089315466	0.062981428	-1.418123872	0.156154603	0.360898919	RELCH	protein_coding
ENSG00000134452.20	3311.448293	0.032662453	0.046391901	0.70405507	0.481398479	0.690289619	FBH1	protein_coding
ENSG00000134453.16	4596.493353	-0.127170198	0.048422421	-2.626266822	0.008632712	0.054513825	RBM17	protein_coding
ENSG00000134460.18	482.7937347	-0.157264336	0.124029431	-1.267959822	0.204812299	0.424348299	IL2RA	protein_coding
ENSG00000134461.16	452.2856787	-0.066268234	0.063652206	-1.041098782	0.297829705	0.52779687	ANKRD16	protein_coding
ENSG00000134463.15	563.9546837	-0.484678549	0.183892386	-2.635664042	0.008397283	0.053452253	ECHDC3	protein_coding
ENSG00000134470.21	1920.046054	0.011513953	0.077992678	0.14762864	0.882635851	0.943682209	IL15RA	protein_coding
ENSG00000134480.16	1207.673247	-0.065638206	0.048930476	-1.341458564	0.179771614	0.392812763	CCNH	protein_coding
ENSG00000134489.7	7.585698849	-0.2377885	0.157950344	-1.505463645	0.132204917	0.325910224	HRH4	protein_coding
ENSG00000134490.14	744.6791585	0.018677003	0.076188506	0.245142005	0.80634648	0.903759273	TMEM241	protein_coding
ENSG00000134504.14	1462.527084	0.152991569	0.099864787	1.531987132	0.12552561	0.315813211	KCTD1	protein_coding
ENSG00000134508.12	2145.241227	-0.293695881	0.095316441	-3.08127201	0.002061183	0.019581459	CABLES1	protein_coding
ENSG00000134516.18	1607.684147	-0.513141265	0.12578782	-4.07941935	4.51483E-05	0.001060842	DOCK2	protein_coding
ENSG00000134531.10	8833.566453	0.297736593	0.119494345	2.491637513	0.012715573	0.071451521	EMP1	protein_coding
ENSG00000134532.19	233.5526277	0.232689342	0.124799404	1.864506837	0.062250562	0.20459256	SOX5	protein_coding
ENSG00000134533.6	663.0769118	-0.23761396	0.15915829	-1.492941147	0.135452593	0.33096995	RERG	protein_coding
ENSG00000134538.3	17.1352908	0.805150643	0.294369672	2.735168456	0.006234836	0.043415699	SLCO1B1	protein_coding
ENSG00000134539.17	495.3843495	-0.24730729	0.130270918	-1.898407517	0.05764242	0.194825389	KLRD1	protein_coding
ENSG00000134545.14	40.65947388	-0.336699708	0.143793803	-2.341545325	0.019204094	0.094378798	KLRC1	protein_coding
ENSG00000134548.11	24.64251609	-0.119953359	0.153938122	-0.779231017	0.435843642	0.653390614	SPX	protein_coding
ENSG00000134551.13	17.56359301	-0.113996738	0.108617664	-1.049523012	0.29393747	0.523967648	PRH2	protein_coding
ENSG00000134569.10	1515.542081	0.160574922	0.16124954	0.995816311	0.319339404	0.548783136	LRP4	protein_coding
ENSG00000134571.11	39.331351	0.290445874	0.15269498	1.902131123	0.057154014	0.193966534	MYBPC3	protein_coding
ENSG00000134574.12	1560.61117	-0.228155429	0.071464082	-3.192588827	0.001410036	0.0148994	DDB2	protein_coding
ENSG00000134575.13	3545.140929	0.049295141	0.05005356	0.984847857	0.324698809	0.554508759	ACP2	protein_coding
ENSG00000134588.12	4.244460988	-0.140005161	0.289202628	-0.484107499	0.62830958	0.797664867	USP26	protein_coding

ENSG00000134590.14	4606.163979	0.367768454	0.095191648	3.863452948	0.000111795	0.002158489	RTL8C	protein_coding
ENSG00000134594.5	46.80252906	-0.434761045	0.103020515	-4.220140485	2.4415E-05	0.000645298	RAB33A	protein_coding
ENSG00000134595.9	1.033675534	-0.043996643	0.393523569	-0.111801798	0.910980562	0.957935376	SOX3	protein_coding
ENSG00000134597.16	1787.296266	-0.169780057	0.063092239	-2.69098163	0.007124212	0.047593404	RBMX2	protein_coding
ENSG00000134602.16	2248.52723	0.006820656	0.07099941	0.096066382	0.923467851	0.96385616	STK26	protein_coding
ENSG00000134612.12	5.675128668	-0.160870878	0.2247152	-0.715887836	0.47406061	0.684677153	FOLH1B	transcribed_unprocessed_pseudogene
ENSG00000134627.12	666.9176422	-0.151277347	0.103942659	-1.455392318	0.145560682	0.345917971	PIWIL4	protein_coding
ENSG00000134640.3	1.078431284	0.06580637	0.321223202	0.20486182	0.837680086	0.920088347	MTNR1B	protein_coding
ENSG00000134644.16	9426.477964	0.025468089	0.033099835	0.769432506	0.441636598	0.658112985	PUM1	protein_coding
ENSG00000134668.12	208.9213234	0.424395252	0.116702534	3.636555591	0.000276308	0.004365727	SPOCD1	protein_coding
ENSG00000134684.11	5566.992854	-0.056364821	0.057694054	-0.976960646	0.328588636	0.558046182	YARS1	protein_coding
ENSG00000134686.18	7659.120296	0.154313703	0.046970713	3.285317439	0.001018675	0.011657252	PHC2	protein_coding
ENSG00000134690.11	2054.243369	-0.086913265	0.084730416	-1.025762273	0.305003653	0.534446266	CDCA8	protein_coding
ENSG00000134697.13	2982.58868	0.001436311	0.04474535	0.032099661	0.974392573	0.988081138	GNL2	protein_coding
ENSG00000134698.11	1655.130758	0.050582311	0.061494618	0.822548592	0.410764743	0.631590107	AGO4	protein_coding
ENSG00000134709.12	2808.725334	-0.078776441	0.092328051	-0.853223268	0.393535502	0.617539563	HOOK1	protein_coding
ENSG00000134716.11	722.8409371	-0.199042478	0.131320679	-1.515697902	0.129595759	0.322100859	CYP2J2	protein_coding
ENSG00000134717.18	2494.548564	-0.061667754	0.044783393	-1.377022833	0.168505189	0.377818934	BTF3L4	protein_coding
ENSG00000134744.14	2138.300776	-0.038832643	0.07825745	-0.496216566	0.619741624	0.79179315	TUT4	protein_coding
ENSG00000134748.13	2761.759469	-0.05189476	0.036732921	-1.412758892	0.157726629	0.362843708	PRPF38A	protein_coding
ENSG00000134755.17	5931.052261	0.363722043	0.147861761	2.459879014	0.013898386	0.076038916	DSC2	protein_coding
ENSG00000134757.5	1168.363249	0.652255807	0.27723327	2.352732795	0.01863602	0.092526132	DSG3	protein_coding
ENSG00000134758.14	1911.532411	-0.153896804	0.055127657	-2.791644187	0.005244099	0.038452312	RNF138	protein_coding
ENSG00000134759.14	2628.386905	-0.036721826	0.052461929	-0.699970934	0.483945456	0.691972815	ELP2	protein_coding
ENSG00000134760.6	48.11724501	-0.278239488	0.246984425	-1.126546696	0.259934187	0.487120768	DSG1	protein_coding
ENSG00000134762.17	252.1783746	0.384362527	0.22508818	1.707608667	0.087708979	0.254263849	DSC3	protein_coding
ENSG00000134765.10	7.127959976	-0.164701584	0.234761712	-0.701569193	0.48294787	0.691151432	DSC1	protein_coding
ENSG00000134769.21	557.7551682	-0.162250894	0.17520897	-0.926042164	0.354424069	0.582600439	DTNA	protein_coding
ENSG00000134775.15	291.3762884	0.24933131	0.147629799	1.688895547	0.091239449	0.260298797	FHOD3	protein_coding
ENSG00000134779.15	4000.222987	-0.069461298	0.063139159	-1.100130229	0.271275385	0.499520117	TPGS2	protein_coding
ENSG00000134780.10	1335.155087	0.196405096	0.096919374	2.026479215	0.042715701	0.160814003	DAGLA	protein_coding
ENSG00000134802.18	1444.421319	0.122050802	0.100772842	1.211147752	0.225838783	0.448988348	SLC43A3	protein_coding
ENSG00000134809.9	1113.953543	-0.172684046	0.067303072	-2.565767675	0.010294776	0.061682309	TIMM10	protein_coding
ENSG00000134812.8	709.2640174	-1.971298361	0.315905519	-6.240151691	4.37147E-10	9.11461E-08	CBLIF	protein_coding
ENSG00000134815.19	2576.315212	0.057235641	0.064569283	0.886422138	0.375390094	0.601211883	DHX34	protein_coding
ENSG00000134817.11	2749.963515	0.024977689	0.120615075	0.207085962	0.835942725	0.91948702	APLNR	protein_coding
ENSG00000134824.14	3066.928525	0.754028553	0.12806138	5.88802458	3.90839E-09	5.69618E-07	FADS2	protein_coding
ENSG00000134825.16	2596.522048	-0.075998782	0.066898368	-1.136033418	0.255942578	0.482948374	TMEM258	protein_coding
ENSG00000134827.8	2751.672112	0.548960979	0.236667143	2.319548765	0.0203653	0.098426832	TCN1	protein_coding
ENSG00000134830.6	113.5762254	0.21307236	0.097970288	2.174867128	0.029640075	0.126555511	C5AR2	protein_coding
ENSG00000134851.13	5382.983705	0.188428134	0.050170095	3.755785889	0.000172798	0.003032636	TMEM165	protein_coding
ENSG00000134852.15	3546.65848	0.085090538	0.054761583	1.553836335	0.120223461	0.307921478	CLOCK	protein_coding

ENSG00000134853.12	4020.710923	0.262592794	0.117684578	2.231327155	0.025659466	0.114930797	PDGFRA	protein_coding
ENSG00000134864.10	378.6424877	0.149986801	0.088164241	1.701220352	0.088901618	0.256339958	GGACT	protein_coding
ENSG00000134871.19	36905.48602	0.155020371	0.096854076	1.600555985	0.109475298	0.290235751	COL4A2	protein_coding
ENSG00000134873.10	66.79991484	1.317053592	0.268004151	4.914302961	8.90989E-07	4.75262E-05	CLDN10	protein_coding
ENSG00000134874.18	586.7948035	0.197373316	0.120489047	1.63810173	0.101400474	0.277802619	DZIP1	protein_coding
ENSG00000134882.16	4188.993859	0.1398844	0.053924702	2.594069041	0.009484744	0.058197224	UBAC2	protein_coding
ENSG00000134884.15	5497.134548	0.019474837	0.067865492	0.286962297	0.774141189	0.885705487	ARGLU1	protein_coding
ENSG00000134897.14	800.4322785	-0.110144016	0.06348575	-1.734940779	0.082751295	0.24527637	BIVM	protein_coding
ENSG00000134899.23	182.4440278	0.09611104	0.05932883	1.619971946	0.105238303	0.283960962	ERCC5	protein_coding
ENSG00000134900.12	3788.412838	0.093686805	0.052542801	1.783056939	0.074577035	0.229298205	TPP2	protein_coding
ENSG00000134901.13	429.1963484	0.294107553	0.076314303	3.853898174	0.000116252	0.002219944	POGLUT2	protein_coding
ENSG00000134905.17	2846.329554	-0.011603911	0.052076721	-0.222823387	0.823672966	0.913038715	CARS2	protein_coding
ENSG00000134909.18	6527.994646	0.194422884	0.083471251	2.329219728	0.019847428	0.096689925	ARHGAP32	protein_coding
ENSG00000134910.14	10008.11812	0.004324674	0.052170498	0.082895008	0.933935024	0.968817523	STT3A	protein_coding
ENSG00000134917.10	457.0334279	-0.034404685	0.201388978	-0.17083698	0.86435195	0.934008735	ADAMTS8	protein_coding
ENSG00000134940.14	27.6943891	-0.262060494	0.113060459	-2.31787928	0.020455883	0.09865051	ACRV1	protein_coding
ENSG00000134954.14	6768.070729	-0.134949318	0.08327182	-1.620588066	0.105106013	0.283794931	ETS1	protein_coding
ENSG00000134955.12	843.4757005	0.139664038	0.116807012	1.195681969	0.23182069	0.455985181	SLC37A2	protein_coding
ENSG00000134962.7	43.82392587	-0.065649072	0.128793865	-0.509722044	0.610246207	0.785860128	KLB	protein_coding
ENSG00000134970.14	5862.120665	0.076135741	0.057464064	1.324927891	0.185195069	0.399714984	TMED7	protein_coding
ENSG00000134982.17	2610.054909	0.015155448	0.064856718	0.233675843	0.815236643	0.908791466	APC	protein_coding
ENSG00000134986.14	375.4164654	0.130358441	0.095482957	1.365253499	0.172173389	0.38275685	NREP	protein_coding
ENSG00000134987.11	2913.597116	0.03497809	0.05551593	0.630055003	0.528658598	0.726936851	WDR36	protein_coding
ENSG00000134996.12	2012.714091	-0.004468059	0.055659564	-0.08027477	0.936018724	0.969767071	OSTF1	protein_coding
ENSG00000135002.12	3583.444564	0.039282583	0.068797787	0.570986146	0.568009034	0.755414295	RFK	protein_coding
ENSG00000135018.14	7965.925261	-0.01482399	0.047772447	-0.310304177	0.756329655	0.8749761	UBQLN1	protein_coding
ENSG00000135040.16	1746.911166	0.048179335	0.051368593	0.937914245	0.348288482	0.5775046	NAA35	protein_coding
ENSG00000135045.7	500.1757662	-0.166986313	0.086431052	-1.932017594	0.053357334	0.185330189	C9orf40	protein_coding
ENSG00000135046.14	12430.45031	0.680921011	0.148685138	4.579617155	4.65828E-06	0.000176516	ANXA1	protein_coding
ENSG00000135047.16	4922.303359	0.229287233	0.088449117	2.592306627	0.009533476	0.058438638	CTSL	protein_coding
ENSG00000135048.14	8667.796377	0.165785581	0.08281066	2.001983582	0.045286497	0.166930274	CEMIP2	protein_coding
ENSG00000135049.16	1175.674981	0.040839829	0.060338319	0.676847309	0.498502838	0.704225959	AGTPBP1	protein_coding
ENSG00000135052.16	21666.22122	-0.015513594	0.110820369	-0.13998865	0.888668958	0.946834406	GOLM1	protein_coding
ENSG00000135063.19	628.209051	-0.419800185	0.177900538	-2.359746571	0.018287423	0.091483302	FAM189A2	protein_coding
ENSG00000135069.14	2840.604669	-0.31325616	0.126671903	-2.472972713	0.013399437	0.074114412	PSAT1	protein_coding
ENSG00000135070.15	1685.997025	-0.020008874	0.04981676	-0.401649447	0.687942034	0.835193905	ISCA1	protein_coding
ENSG00000135074.16	2572.894224	0.07237991	0.095061198	0.761403296	0.446416219	0.662065806	ADAM19	protein_coding
ENSG00000135077.9	888.4359508	-0.052035177	0.111917973	-0.464940315	0.641974199	0.806344315	HAVCR2	protein_coding
ENSG00000135083.16	221.4936183	0.363439252	0.118105545	3.077241226	0.002089262	0.019783518	CCNJL	protein_coding
ENSG00000135090.14	5506.099832	-0.18670749	0.053913065	-3.463121406	0.000533947	0.007143944	TAOK3	protein_coding
ENSG00000135093.13	957.7200344	-0.128167516	0.049371855	-2.595963142	0.009432618	0.05793449	USP30	protein_coding
ENSG00000135094.11	888.04711	0.164375393	0.126631107	1.298064879	0.194265041	0.411005861	SDS	protein_coding

ENSG00000135097.7	561.964615	-0.362021114	0.199298937	-1.816472878	0.069297865	0.218395308	MSI1	protein_coding
ENSG00000135100.18	1303.957077	-0.14790999	0.105024977	-1.408331562	0.159032914	0.364765371	HNF1A	protein_coding
ENSG00000135108.15	3319.188877	0.029362263	0.043937624	0.668271535	0.50396028	0.708250639	FBXO21	protein_coding
ENSG00000135111.16	1819.561493	0.046263778	0.126582529	0.36548312	0.714750805	0.850956118	TBX3	protein_coding
ENSG00000135114.12	1629.372227	-0.118533453	0.137988629	-0.859008841	0.390335638	0.614777219	OASL	protein_coding
ENSG00000135116.9	39.50604798	0.606046573	0.192179646	3.153541931	0.001613021	0.016388574	HRK	protein_coding
ENSG00000135119.15	650.7689327	-0.112868573	0.108608629	-1.039222888	0.298701088	0.528545796	RNFT2	protein_coding
ENSG00000135124.15	2077.294956	-0.191747381	0.073171888	-2.620506139	0.008779935	0.055122205	P2RX4	protein_coding
ENSG00000135127.11	1416.840806	-0.148989233	0.091769033	-1.623524057	0.104477425	0.282779787	BICDL1	protein_coding
ENSG00000135144.7	711.3304834	-0.326648328	0.138654429	-2.35584488	0.018480633	0.092111466	DTX1	protein_coding
ENSG00000135148.12	2277.125865	-0.060314798	0.057422374	-1.050371035	0.293547557	0.523529586	TRAFD1	protein_coding
ENSG00000135164.19	2462.714261	-0.136057093	0.066869671	-2.034660738	0.041885021	0.158711708	DMTF1	protein_coding
ENSG00000135175.5	2.362801371	0.136399844	0.371885702	0.366778942	0.713783916	0.850477973	OCM2	protein_coding
ENSG00000135185.12	1026.629785	-0.22187416	0.061342917	-3.616948323	0.000298097	0.004622834	TMEM243	protein_coding
ENSG00000135205.15	402.6258167	-0.271627384	0.102788961	-2.642573492	0.008227861	0.052773842	CCDC146	protein_coding
ENSG00000135211.6	886.7665116	-0.217098792	0.059358756	-3.657401321	0.000254785	0.004104539	TMEM60	protein_coding
ENSG00000135218.19	1119.283632	0.123631085	0.143653191	0.860621918	0.38944631	0.613953471	CD36	protein_coding
ENSG00000135220.11	1110.571234	-1.568782196	0.27100619	-5.788731964	7.09198E-09	8.932E-07	UGT2A3	protein_coding
ENSG00000135222.6	1.740162817	-1.185614997	0.512508327	-2.313357529	0.02070299	0.099489877	CSN2	protein_coding
ENSG00000135226.18	2.995825718	-1.353143859	0.342024382	-3.956278942	7.61263E-05	0.00160498	UGT2B28	protein_coding
ENSG00000135241.17	2001.135387	0.132111038	0.047960072	2.754604649	0.005876309	0.041691947	PNPLA8	protein_coding
ENSG00000135245.10	639.5317552	0.261818219	0.114248806	2.291649491	0.021925877	0.103315738	HILPDA	protein_coding
ENSG00000135248.15	5.95191951	0.217483048	0.228530612	0.951658272	0.341270318	0.571239279	FAM71F1	protein_coding
ENSG00000135249.8	1031.993304	-0.020610243	0.061560431	-0.334796931	0.737778276	0.864222476	RINT1	protein_coding
ENSG00000135250.17	2305.247946	0.042551232	0.055966374	0.760299966	0.447075302	0.66250214	SRPK2	protein_coding
ENSG00000135253.15	442.7131475	0.52337764	0.12764805	4.100161659	4.12862E-05	0.000989075	KCP	protein_coding
ENSG00000135269.18	5883.695316	0.086434951	0.07690336	1.123942454	0.261037439	0.488179936	TES	protein_coding
ENSG00000135272.12	1692.426067	0.006796699	0.096784654	0.070224962	0.944014607	0.97369132	MDFIC	protein_coding
ENSG00000135297.16	1219.805424	-0.081930717	0.041191256	-1.989031767	0.046697697	0.170229226	MTO1	protein_coding
ENSG00000135298.14	55.50358072	-0.148636119	0.194850106	-0.762822881	0.445569033	0.661315452	ADGRB3	protein_coding
ENSG00000135299.17	275.6206947	0.292492634	0.114400058	2.556752506	0.010565436	0.062694235	ANKRD6	protein_coding
ENSG00000135312.7	98.64756108	0.751302973	0.177920622	4.222686313	2.41408E-05	0.000641772	HTR1B	protein_coding
ENSG00000135314.12	121.2496384	0.289716025	0.12483028	2.3208794	0.020293354	0.098214513	KHDC1	protein_coding
ENSG00000135315.12	551.4928073	0.044953784	0.061365157	0.73256203	0.463825597	0.676062151	CEP162	protein_coding
ENSG00000135316.19	10110.63861	0.056879391	0.049516984	1.148684478	0.250686109	0.47739145	SYNCRIP	protein_coding
ENSG00000135317.13	2996.829794	0.09078653	0.051236694	1.771904516	0.076410409	0.23312584	SNX14	protein_coding
ENSG00000135318.12	3965.993645	0.558323372	0.128514269	4.34444654	1.39627E-05	0.000421497	NT5E	protein_coding
ENSG00000135324.6	354.4565205	0.179579636	0.164408131	1.092279526	0.274710257	0.503344778	MRAP2	protein_coding
ENSG00000135333.14	449.8938787	-0.30204857	0.223493201	-1.351488852	0.176538889	0.388654903	EPHA7	protein_coding
ENSG00000135334.9	2814.620572	0.079057935	0.049940018	1.583057797	0.113408292	0.296994779	AKIRIN2	protein_coding
ENSG00000135336.15	1602.256335	0.079707263	0.054076873	1.473962121	0.14049179	0.338243688	ORC3	protein_coding
ENSG00000135338.14	369.2100709	0.075066867	0.080782264	0.929249358	0.352759873	0.581179391	LCA5	protein_coding

ENSG00000135341.18	2512.552254	0.16719396	0.041020223	4.075891102	4.58385E-05	0.001071374	MAP3K7	protein_coding
ENSG00000135346.9	26.49480111	0.791413016	0.267277151	2.961020095	0.003066219	0.026120524	CGA	protein_coding
ENSG00000135355.4	3.062981213	0.677985379	0.375608049	1.805034213	0.071069328	0.221926774	GJA10	protein_coding
ENSG00000135362.14	854.1328413	0.298689195	0.104687259	2.853157088	0.004328722	0.033552598	PRR5L	protein_coding
ENSG00000135363.12	665.1207955	0.09758198	0.087557421	1.114491254	0.265068495	0.492869508	LMO2	protein_coding
ENSG00000135365.16	2214.807192	-0.086286975	0.058844406	-1.466358159	0.142550739	0.341246305	PHF21A	protein_coding
ENSG00000135372.9	4385.327701	-0.036673925	0.055275455	-0.66347576	0.507025899	0.711011025	NAT10	protein_coding
ENSG00000135373.13	14819.83216	0.056411681	0.104456436	0.540049837	0.589162664	0.770642095	EHF	protein_coding
ENSG00000135374.11	117.1302973	-0.793826371	0.276581084	-2.870139777	0.004102904	0.032331328	ELF5	protein_coding
ENSG00000135378.4	2841.081635	-0.046575577	0.095900316	-0.485666567	0.627203592	0.796957309	PRRG4	protein_coding
ENSG00000135387.21	19211.3962	0.02354307	0.050960727	0.461984569	0.644092386	0.807762325	CAPRIN1	protein_coding
ENSG00000135390.21	11785.28669	-0.263954274	0.065098952	-4.054662432	5.02068E-05	0.001154005	ATP5MC2	protein_coding
ENSG00000135392.18	902.9884797	0.019306776	0.056564159	0.341325258	0.732858735	0.861151007	DNAJC14	protein_coding
ENSG00000135404.12	26927.55597	0.029022595	0.058105596	0.499480201	0.617441132	0.790290554	CD63	protein_coding
ENSG00000135406.14	30.16074205	-0.131305826	0.222473273	-0.59020944	0.555050244	0.746041901	PRPH	protein_coding
ENSG00000135407.10	329.138954	0.034024452	0.11771407	0.289043206	0.772548314	0.884928578	AVIL	protein_coding
ENSG00000135409.11	16.36127848	-0.78968302	0.201510643	-3.918815454	8.89852E-05	0.001813697	AMHR2	protein_coding
ENSG00000135413.9	0.546457229	-0.146325688	0.512026926	-0.28577733	0.775048673		LACRT	protein_coding
ENSG00000135414.10	834.7221412	-0.003863682	0.088234479	-0.0437888	0.965072755	0.984139479	GDF11	protein_coding
ENSG00000135423.13	21.87281119	-0.27056616	0.112965803	-2.395115615	0.016615125	0.085969759	GLS2	protein_coding
ENSG00000135424.18	2052.436237	-0.254227371	0.148929796	-1.707028257	0.0878168	0.254508693	ITGA7	protein_coding
ENSG00000135426.16	234.931953	-0.663911323	0.133673661	-4.966657722	6.81167E-07	3.83009E-05	TESPA1	protein_coding
ENSG00000135436.8	47.82030112	-0.104776165	0.09067503	-1.155512885	0.247880459	0.474391163	FAM186B	protein_coding
ENSG00000135437.10	98.30428807	-0.420163357	0.096381705	-4.359368356	1.30438E-05	0.000399727	RDH5	protein_coding
ENSG00000135439.11	455.6016873	-0.07505914	0.113301267	-0.662473968	0.507667513	0.711532314	AGAP2	protein_coding
ENSG00000135441.7	693.2484058	-0.299228691	0.094355334	-3.17129597	0.001517604	0.01566011	BLOC1S1	protein_coding
ENSG00000135443.8	1.925823203	0.316404083	0.271291908	1.166286475	0.24349864	0.469526406	KRT85	protein_coding
ENSG00000135446.17	3136.634489	-0.036504468	0.080982822	-0.450768038	0.65215674	0.812854935	CDK4	protein_coding
ENSG00000135447.17	233.4667415	-0.232695111	0.232731439	-0.999843907	0.317386054	0.54671247	PPP1R1A	protein_coding
ENSG00000135451.13	1542.35546	-0.064538905	0.096463891	-0.669047288	0.503465313	0.708000128	TROAP	protein_coding
ENSG00000135452.10	1551.222594	0.068097234	0.070787644	0.961993219	0.33605301	0.565587132	TSPAN31	protein_coding
ENSG00000135454.14	172.5053441	0.751820213	0.134124129	5.605406126	2.07767E-08	2.18693E-06	B4GALNT1	protein_coding
ENSG00000135457.10	2617.339328	-0.053972613	0.037366004	-1.444430971	0.148617784	0.350292268	TFCP2	protein_coding
ENSG00000135469.14	259.0521914	-0.029083411	0.06127693	-0.474622522	0.63505605	0.802070472	COQ10A	protein_coding
ENSG00000135472.9	333.3683778	0.4283778	0.160330117	2.671848617	0.007543467	0.049579108	FAIM2	protein_coding
ENSG00000135473.15	1607.270058	-0.016978081	0.064529213	-0.263106902	0.792468184	0.895951177	PAN2	protein_coding
ENSG00000135476.12	2115.86017	-0.038400482	0.098656176	-0.389235459	0.697101975	0.840437482	ESPL1	protein_coding
ENSG00000135477.11	28.03024144	1.139793848	0.166070442	6.863315573	6.72804E-12	2.93188E-09	KRT87P	transcribed_unprocessed_pseudogene
ENSG00000135480.16	8926.780617	1.38318693	0.210836475	6.560472654	5.36375E-11	1.62317E-08	KRT7	protein_coding
ENSG00000135482.7	296.0257675	0.020409927	0.054171568	0.376764571	0.706348572	0.84613095	ZC3H10	protein_coding
ENSG00000135486.19	34095.30712	-0.090159424	0.052328614	-1.722946916	0.084898125	0.24923239	HNRNPA1	protein_coding
ENSG00000135502.19	1.959390953	-0.316611122	0.2252278	-1.405737307	0.159802145	0.365591264	SLC26A10	transcribed_unitary_pseudogene

ENSG00000135503.13	5143.289805	0.013068401	0.073914463	0.176804383	0.859662046	0.931738869	ACVR1B	protein_coding
ENSG00000135506.16	9241.576122	0.109428498	0.059157194	1.84979189	0.06434355	0.208685538	OS9	protein_coding
ENSG00000135517.9	3.411616254	-0.162128836	0.177064862	-0.915646581	0.359852291	0.587901804	MIP	protein_coding
ENSG00000135519.8	107.301914	-0.174657981	0.17866152	-0.977591483	0.328276412	0.557791145	KCNH3	protein_coding
ENSG00000135521.9	802.9973725	-0.109209179	0.061513642	-1.775365207	0.075837605	0.231863533	LTV1	protein_coding
ENSG00000135525.18	4221.26172	0.071500023	0.082315436	0.86861014	0.385060406	0.609827242	MAP7	protein_coding
ENSG00000135535.17	29545.19524	0.019234112	0.063414565	0.303307482	0.761655545	0.878107556	CD164	protein_coding
ENSG00000135537.17	290.598317	-0.016419079	0.057461738	-0.285739334	0.775077776	0.886111972	AFG1L	protein_coding
ENSG00000135540.11	3388.032422	-0.061999781	0.095072529	-0.652131396	0.514316412	0.716278884	NHSL1	protein_coding
ENSG00000135541.21	1283.882433	0.009275569	0.060471171	0.153388274	0.878092091	0.941250336	AHI1	protein_coding
ENSG00000135547.9	235.4258756	0.040973209	0.091882056	0.445932654	0.655645894	0.814873114	HEY2	protein_coding
ENSG00000135549.15	414.883721	0.359384515	0.152071438	2.363261109	0.018114901	0.09081085	PKIB	protein_coding
ENSG00000135569.4	2.152764775	-0.53867369	0.416623409	-1.292951089	0.196027976	0.41311109	TAAR5	protein_coding
ENSG00000135577.4	3.186387954	-0.187714602	0.217085683	-0.86470282	0.387201921	0.611831827	NMBR	protein_coding
ENSG00000135587.9	824.796833	-0.080456348	0.072228427	-1.113915273	0.265315537	0.493181553	SMPD2	protein_coding
ENSG00000135596.18	3013.405754	-0.032241191	0.06737493	-0.478533943	0.632270221	0.800335565	MICAL1	protein_coding
ENSG00000135597.19	2608.776798	0.105571547	0.047633888	2.216311758	0.026670158	0.117958779	REPS1	protein_coding
ENSG00000135604.10	498.219858	-0.076633268	0.102070202	-0.750789814	0.452779161	0.667233353	STX11	protein_coding
ENSG00000135605.13	239.0188216	-0.048153854	0.070461748	-0.683404187	0.494351475	0.701111574	TEC	protein_coding
ENSG00000135617.4	738.0162267	-0.057116361	0.074972301	-0.761832842	0.446159777	0.661753049	PRADC1	protein_coding
ENSG00000135622.13	491.0923349	0.17082511	0.077672926	2.199287708	0.027857471	0.12124109	SEMA4F	protein_coding
ENSG00000135624.16	12385.1857	-0.037885748	0.053953801	-0.702188673	0.482561509	0.690922399	CCT7	protein_coding
ENSG00000135625.8	81.77691137	0.434509431	0.19913498	2.181984459	0.02911068	0.125017847	EGR4	protein_coding
ENSG00000135631.17	2482.682689	0.354348273	0.067679704	5.235665207	1.64392E-07	1.21213E-05	RAB11FIP5	protein_coding
ENSG00000135632.12	2061.467208	-0.068354554	0.050039934	-1.366000073	0.171938941	0.382554399	SMYD5	protein_coding
ENSG00000135636.14	2318.018799	-0.038028055	0.096084833	-0.395775834	0.692270406	0.837663468	DYSF	protein_coding
ENSG00000135637.14	645.4924982	-0.010640054	0.061437463	-0.17318512	0.86250592	0.932948383	CCDC142	protein_coding
ENSG00000135638.14	254.7608766	0.396124466	0.225759305	1.754631843	0.079322301	0.23893137	EMX1	protein_coding
ENSG00000135643.5	303.2839511	0.003128357	0.146343918	0.021376749	0.982945121	0.992450592	KCNMB4	protein_coding
ENSG00000135655.16	3270.589735	-0.01763041	0.049408508	-0.35682944	0.721219495	0.854385808	USP15	protein_coding
ENSG00000135677.11	13175.9507	0.114432357	0.07091777	1.613592146	0.106615922	0.28600995	GNS	protein_coding
ENSG00000135678.12	2755.009398	0.313831107	0.131660801	2.383633584	0.017142662	0.087658503	CPM	protein_coding
ENSG00000135679.25	5931.261094	0.019227411	0.104025706	0.184833266	0.853359815	0.928371361	MDM2	protein_coding
ENSG00000135686.13	2835.106786	0.011577577	0.052231577	0.221658575	0.82457968	0.913399045	KLHL36	protein_coding
ENSG00000135697.10	278.0856771	-0.304697958	0.15104201	-2.017306034	0.043663586	0.162946656	BCO1	protein_coding
ENSG00000135698.10	1167.819946	-0.111962138	0.063150908	-1.772929966	0.076240312	0.232728739	MPHOSPH6	protein_coding
ENSG00000135702.14	163.0451793	-0.389389282	0.237214521	-1.64150694	0.100692228	0.276702417	CHST5	protein_coding
ENSG00000135709.12	1640.574665	-0.113409218	0.09854805	-1.150801244	0.249814013	0.476563728	KIAA0513	protein_coding
ENSG00000135720.13	5095.042563	0.112482002	0.052776878	2.131274262	0.033066554	0.13577134	DYNC1LI2	protein_coding
ENSG00000135722.9	374.2718074	0.110766059	0.070883362	1.562652458	0.118134363	0.304367277	FBXL8	protein_coding
ENSG00000135723.14	1352.552705	-0.02104119	0.055429289	-0.37960418	0.704239257	0.844835076	FHOD1	protein_coding
ENSG00000135736.6	529.8573991	0.096038154	0.082671021	1.161690678	0.245361134	0.471786706	CCDC102A	protein_coding

ENSG00000135740.17	107.9775509	-0.005545376	0.08889192	-0.062383356	0.950257549	0.976776854	SLC9A5	protein_coding
ENSG00000135744.8	3152.707446	-0.031506555	0.16493222	-0.191027287	0.848504215	0.925700828	AGT	protein_coding
ENSG00000135747.11	18.1345463	0.122277042	0.135810722	0.900348956	0.367934576	0.595002138	ZNF670-ZNF695	protein_coding
ENSG00000135749.19	1271.24113	0.123287834	0.079331824	1.554077886	0.120165839	0.307882572	PCNX2	protein_coding
ENSG00000135750.15	3295.72384	0.400405422	0.111583053	3.588407131	0.000332704	0.0050167	KCNK1	protein_coding
ENSG00000135763.10	1435.399174	0.137042882	0.066320792	2.066363774	0.038794144	0.151102289	URB2	protein_coding
ENSG00000135766.9	1312.778305	0.134643758	0.075638447	1.780096811	0.075060118	0.230393378	EGLN1	protein_coding
ENSG00000135773.13	515.9718448	-0.013863446	0.227051859	-0.061058501	0.951312619	0.977190439	CAPN9	protein_coding
ENSG00000135775.14	1897.390475	0.032631787	0.052651609	0.619768079	0.535410488	0.732135432	COG2	protein_coding
ENSG00000135776.5	1640.836331	-0.046088265	0.056439809	-0.816591444	0.414161946	0.634696798	ABCB10	protein_coding
ENSG00000135778.12	1672.664894	-0.066931219	0.047306801	-1.414832932	0.157117486	0.362159746	NTPCR	protein_coding
ENSG00000135801.10	2035.532121	0.068071284	0.041038692	1.658709907	0.097174262	0.27071748	TAF5L	protein_coding
ENSG00000135821.19	21319.95821	0.027901906	0.08093982	0.344724092	0.730301804	0.859606767	GLUL	protein_coding
ENSG00000135823.14	2398.813744	-0.01478455	0.040719914	-0.363079104	0.716545793	0.851891146	STX6	protein_coding
ENSG00000135824.12	8.052523073	-0.093827284	0.188452245	-0.497883607	0.618566082	0.791075417	RGS8	protein_coding
ENSG00000135828.11	890.9018286	0.249259352	0.067570072	3.6889017	0.000225224	0.003724704	RNASEL	protein_coding
ENSG00000135829.17	11556.94599	0.03545796	0.042056854	0.843095875	0.399174826	0.622037489	DHX9	protein_coding
ENSG00000135835.12	244.0788317	0.141001861	0.102418889	1.376717355	0.168599651	0.377818934	KIAA1614	protein_coding
ENSG00000135837.16	4577.720515	0.102094796	0.052585235	1.941510701	0.052196363	0.182695654	CEP350	protein_coding
ENSG00000135838.13	1043.395692	-0.070762032	0.083234336	-0.850154342	0.395239282	0.61869898	NPL	protein_coding
ENSG00000135842.17	5489.897473	-0.306274262	0.157059111	-1.950057284	0.051169292	0.180536331	NIBAN1	protein_coding
ENSG00000135845.10	1454.493662	0.043803025	0.049861813	0.878488421	0.379678719	0.605211044	PIGC	protein_coding
ENSG00000135862.6	17328.38084	0.230004225	0.088597744	2.596050583	0.009430218	0.05793449	LAMC1	protein_coding
ENSG00000135870.12	3297.254169	0.0196774	0.04327288	0.454728227	0.649304771	0.810714707	RC3H1	protein_coding
ENSG00000135898.10	33.63565038	-0.33782304	0.146615692	-2.304139727	0.021214801	0.101091022	GPR55	protein_coding
ENSG00000135899.19	2359.053432	-0.129096776	0.069936024	-1.845926719	0.064902846	0.209725294	SP110	protein_coding
ENSG00000135900.4	1397.405942	-0.07654175	0.052842706	-1.448482795	0.147482074	0.348658729	MRPL44	protein_coding
ENSG00000135902.10	6.836264473	0.014728159	0.229514106	0.064171041	0.948834036	0.976047892	CHRND	protein_coding
ENSG00000135903.20	9.186901008	0.530074826	0.307685838	1.722779409	0.084928424	0.249287749	PAX3	protein_coding
ENSG00000135905.20	1167.921657	-0.354434334	0.111131208	-3.189332145	0.001426019	0.014999189	DOCK10	protein_coding
ENSG00000135912.11	2722.576293	0.016899959	0.067435896	0.250607758	0.802117383	0.901546754	TTLL4	protein_coding
ENSG00000135913.11	834.8865853	0.108203379	0.064887183	1.667561663	0.095402774	0.267392487	USP37	protein_coding
ENSG00000135914.6	99.60294413	0.16719989	0.162730891	1.027462512	0.304202729	0.533689255	HTR2B	protein_coding
ENSG00000135916.16	7635.775311	0.331720201	0.101449653	3.269801239	0.001076231	0.012153125	ITM2C	protein_coding
ENSG00000135917.16	1096.729322	-0.873876438	0.207154868	-4.218469242	2.45967E-05	0.000649212	SLC19A3	protein_coding
ENSG00000135919.14	2716.032354	0.11232162	0.133102149	0.843875326	0.398739078	0.621651171	SERPINE2	protein_coding
ENSG00000135924.16	2674.234035	0.018098092	0.059700053	0.30315035	0.761775284	0.878159776	DNAJB2	protein_coding
ENSG00000135925.9	752.9255738	0.748881222	0.196812163	3.805055591	0.000141772	0.002600174	WNT10A	protein_coding
ENSG00000135926.15	19172.15772	0.115371914	0.07482069	1.54197874	0.12307876	0.312188773	TMBIM1	protein_coding
ENSG00000135929.9	2004.777863	-0.149924996	0.126587784	-1.184355953	0.236272189	0.461250478	CYP27A1	protein_coding
ENSG00000135930.14	3525.336634	-0.019349476	0.043153962	-0.448382362	0.653877269	0.813951774	EIF4E2	protein_coding
ENSG00000135931.18	1209.672518	0.136814923	0.076960697	1.777724577	0.075449101	0.231117512	ARMC9	protein_coding

ENSG00000135932.11	6930.228002	-0.015046636	0.042332609	-0.355438438	0.722261157	0.855166936	CAB39	protein_coding
ENSG00000135940.7	9391.703824	-0.179702114	0.069099159	-2.600641122	0.009304974	0.05746639	COX5B	protein_coding
ENSG00000135945.10	1513.935808	0.020728794	0.040885508	0.506996123	0.612157539	0.7871113	REV1	protein_coding
ENSG00000135951.16	295.1637897	-0.025729596	0.070587006	-0.364508963	0.715477982	0.851319119	TSGA10	protein_coding
ENSG00000135953.11	1498.671341	0.026676715	0.053774924	0.496080932	0.619837312	0.791845755	MFSD9	protein_coding
ENSG00000135956.9	9106.571646	0.075244651	0.037829599	1.989041715	0.046696599	0.170229226	TMEM127	protein_coding
ENSG00000135960.10	737.3128071	-0.163227983	0.197614288	-0.825992822	0.408808164	0.629737119	EDAR	protein_coding
ENSG00000135966.13	3121.059108	-0.016327123	0.040876695	-0.399423756	0.689580993	0.836030135	TGFBRAP1	protein_coding
ENSG00000135968.21	5506.25757	-0.008610372	0.064691811	-0.133098326	0.894115619	0.949630416	GCC2	protein_coding
ENSG00000135972.9	757.1214286	-0.096374714	0.05026871	-1.917190907	0.055213683	0.189646619	MRPS9	protein_coding
ENSG00000135973.3	3.932470644	0.067614836	0.175913973	0.38436308	0.700709361	0.842527437	GPR45	protein_coding
ENSG00000135974.10	1382.410244	-0.010463441	0.038014059	-0.275251877	0.783122733	0.890081799	C2orf49	protein_coding
ENSG00000135976.20	465.8348263	-0.200714199	0.081258349	-2.470074787	0.013508481	0.074570442	ANKRD36	protein_coding
ENSG00000135999.12	1645.924408	0.035219311	0.040979556	0.859436136	0.39009994	0.614593883	EPC2	protein_coding
ENSG00000136002.20	750.2532127	1.042315062	0.171381232	6.081850662	1.18803E-09	2.06059E-07	ARHGEF4	protein_coding
ENSG00000136003.16	4564.523051	-0.160310345	0.050397847	-3.180896665	0.0014682	0.015306159	ISCU	protein_coding
ENSG00000136010.14	1002.481111	0.388754933	0.112694791	3.449626468	0.000561363	0.007424106	ALDH1L2	protein_coding
ENSG00000136011.15	44.23995591	-0.018534074	0.130007561	-0.142561508	0.886636499	0.945848337	STAB2	protein_coding
ENSG00000136014.12	45.21800248	-0.352211563	0.142692003	-2.468334277	0.013574349	0.074858189	USP44	protein_coding
ENSG00000136021.19	4596.374717	-0.027361758	0.047529092	-0.575684434	0.564828499	0.753421638	SCYL2	protein_coding
ENSG00000136026.14	9312.598029	0.000457708	0.070886727	0.006456896	0.994848178	0.997626702	CKAP4	protein_coding
ENSG00000136040.9	1693.18641	0.03580222	0.103223908	0.34684038	0.728711239	0.85864134	PLXNC1	protein_coding
ENSG00000136044.12	3652.34414	-0.089437942	0.05954713	-1.501968971	0.133105123	0.327404204	APPL2	protein_coding
ENSG00000136045.12	2764.020577	-0.062439495	0.041350746	-1.5099968	0.131044241	0.324258411	PWP1	protein_coding
ENSG00000136048.14	2137.004555	-0.015838717	0.089989767	-0.176005751	0.860289425	0.93204794	DRAM1	protein_coding
ENSG00000136051.15	3806.674464	-0.055414636	0.047763086	-1.160197999	0.245968202	0.472467329	WASHC4	protein_coding
ENSG00000136052.9	2453.530906	-0.095926759	0.096182237	-0.997343807	0.318597657	0.548084564	SLC41A2	protein_coding
ENSG00000136059.14	3999.645318	0.061495354	0.132946606	0.462556783	0.643682092	0.807395715	VILL	protein_coding
ENSG00000136068.15	23786.35914	0.33852304	0.089765876	3.771177374	0.000162479	0.002894666	FLNB	protein_coding
ENSG00000136098.17	934.5614467	-0.16097287	0.100538171	-1.601111975	0.10935212	0.290042017	NEK3	protein_coding
ENSG00000136099.13	31.04952184	0.940122987	0.262728885	3.578300834	0.000345835	0.005171744	PCDH8	protein_coding
ENSG00000136100.14	3864.58659	-0.01088914	0.053818562	-0.202330572	0.839658301	0.921243725	VPS36	protein_coding
ENSG00000136104.21	1922.458962	-0.14973297	0.055232807	-2.710942607	0.006709224	0.045653942	RNASEH2B	protein_coding
ENSG00000136108.15	3330.41098	0.070372679	0.086392328	0.814570935	0.415317953	0.635719921	CKAP2	protein_coding
ENSG00000136110.13	12.12306283	0.110606542	0.244803252	0.451818108	0.651400024	0.81232998	CNMD	protein_coding
ENSG00000136111.14	2657.562577	0.112266727	0.103884578	1.080687127	0.279836311	0.508591915	TBC1D4	protein_coding
ENSG00000136114.17	247.0374938	0.068129339	0.087027382	0.782849463	0.433715518	0.651522669	THSD1	protein_coding
ENSG00000136122.18	565.4365709	0.109473869	0.080359428	1.362302741	0.173102363	0.38403837	BORA	protein_coding
ENSG00000136141.15	2772.042049	-0.004820482	0.059765303	-0.080656866	0.935714841	0.969720852	LRCH1	protein_coding
ENSG00000136143.16	2568.518042	0.054843383	0.058430004	0.938616788	0.34792753	0.577234371	SUCLA2	protein_coding
ENSG00000136144.12	1811.99533	0.036422861	0.065722885	0.554188408	0.579449911	0.763576769	RCBTB1	protein_coding
ENSG00000136146.15	1741.261771	-0.052619011	0.05150345	-1.021659935	0.30694188	0.53646197	MED4	protein_coding

ENSG00000136147.18	2005.7088	-0.158954302	0.055857718	-2.8456999	0.004431394	0.034160063	PHF11	protein_coding
ENSG00000136149.6	1181.997201	-0.1869871	0.089292068	-2.094106495	0.036250495	0.144191371	RPL13AP25	processed_pseudogene
ENSG00000136152.15	2665.626417	0.100682136	0.060696688	1.658774778	0.097161185	0.270698353	COG3	protein_coding
ENSG00000136153.20	9863.562919	0.4103212	0.101679187	4.035449271	5.44979E-05	0.001230737	LMO7	protein_coding
ENSG00000136155.17	436.9883374	1.376309699	0.268191274	5.131821331	2.86952E-07	1.89462E-05	SCEL	protein_coding
ENSG00000136156.15	45423.43705	0.010002198	0.057260404	0.17467913	0.861331768	0.932477049	ITM2B	protein_coding
ENSG00000136158.12	2298.717467	0.191763959	0.093226156	2.056975926	0.039688542	0.15335233	SPRY2	protein_coding
ENSG00000136159.4	1551.161406	0.024522184	0.070074756	0.349943192	0.726381331	0.857331039	NUDT15	protein_coding
ENSG00000136160.17	1124.332179	-0.042820488	0.108526844	-0.394561258	0.69316671	0.837965038	EDNRB	protein_coding
ENSG00000136161.13	683.1687951	0.028929567	0.074394634	0.388866306	0.697375048	0.840491603	RCBTB2	protein_coding
ENSG00000136167.15	8143.930106	-0.511856216	0.117557342	-4.354098253	1.33616E-05	0.000406604	LCP1	protein_coding
ENSG00000136169.16	1086.263592	-0.177341413	0.052745074	-3.362236513	0.000773139	0.009462657	SETDB2	protein_coding
ENSG00000136193.17	4977.58123	0.406972623	0.105216049	3.867970988	0.000109745	0.002126432	SCRN1	protein_coding
ENSG00000136197.13	28.14524596	-0.061195121	0.073478254	-0.832833082	0.404938874	0.626479739	C7orf25	protein_coding
ENSG00000136205.17	10367.74633	-0.034217763	0.066201996	-0.516869051	0.605247574	0.782340546	TNS3	protein_coding
ENSG00000136206.4	95.37579018	0.006264737	0.095655333	0.065492818	0.947781625	0.975463848	SPDYE1	protein_coding
ENSG00000136213.10	1780.742795	0.006560515	0.062078487	0.105680969	0.91583548	0.960083828	CHST12	protein_coding
ENSG00000136231.14	906.0266062	-0.022356089	0.186059822	-0.12015538	0.904360063	0.954382061	IGF2BP3	protein_coding
ENSG00000136235.17	10024.62097	-0.024697884	0.133627005	-0.184827044	0.853364696	0.928371361	GPNMB	protein_coding
ENSG00000136237.19	2291.836172	-0.086910314	0.065980078	-1.317220545	0.187764709	0.402888864	RAPGEF5	protein_coding
ENSG00000136238.18	30819.24605	-0.033152733	0.051893543	-0.638860467	0.522913708	0.722356642	RAC1	protein_coding
ENSG00000136240.10	23546.54646	0.04217997	0.060186956	0.700815802	0.483417975	0.691514041	KDELR2	protein_coding
ENSG00000136243.17	910.3983959	-0.262896188	0.05183302	-5.071982856	3.93692E-07	2.45787E-05	NUP42	protein_coding
ENSG00000136244.12	441.4663171	0.427491463	0.192435265	2.221481923	0.026318338	0.116840766	IL6	protein_coding
ENSG00000136247.15	1542.45664	-0.022214791	0.055498726	-0.400275687	0.688953474	0.835661616	ZDHHC4	protein_coding
ENSG00000136250.12	664.4922146	-0.267906219	0.128639971	-2.082604776	0.037287264	0.146930832	AOAH	protein_coding
ENSG00000136261.15	5368.653699	-0.163472169	0.069153608	-2.363899357	0.018083724	0.090714224	BZW2	protein_coding
ENSG00000136267.14	46.58260098	0.105560546	0.185743505	0.568313522	0.569822099	0.756572668	DGKB	protein_coding
ENSG00000136270.14	4414.499406	-0.051014858	0.059941118	-0.851082857	0.394723328	0.618224435	TBRG4	protein_coding
ENSG00000136271.10	4614.370533	-0.144045726	0.052474147	-2.745079916	0.006049616	0.042581834	DDX56	protein_coding
ENSG00000136273.13	1362.379293	-0.005880699	0.045114951	-0.130349231	0.896290132	0.950870411	HUS1	protein_coding
ENSG00000136274.9	248.8619552	0.17646908	0.148039819	1.192037936	0.233246371	0.457695065	NACAD	protein_coding
ENSG00000136275.10	5.045705004	-0.343596234	0.194481106	-1.766733243	0.077272913	0.234803831	C7orf69	lncRNA
ENSG00000136279.21	7141.689928	-0.022228857	0.051116483	-0.434866724	0.663659169	0.820107634	DBNL	protein_coding
ENSG00000136280.17	2191.589299	-0.029445874	0.050920411	-0.578272516	0.563080147	0.751895065	CCM2	protein_coding
ENSG00000136286.16	952.2077604	-0.500834127	0.118419962	-4.229304928	2.34414E-05	0.00063095	MYO1G	protein_coding
ENSG00000136295.15	15865.81491	0.215686102	0.089905394	2.399034061	0.016438386	0.085440783	TTYH3	protein_coding
ENSG00000136297.15	3.609226014	0.046361081	0.301205925	0.153918225	0.877674214	0.94102964	MMD2	protein_coding
ENSG00000136305.11	0						CIDEB	protein_coding
ENSG00000136315.4	2.894389148	-0.032043044	0.199254951	-0.160814291	0.872239669	0.938226491	AL355922.1	lncRNA
ENSG00000136319.12	833.7319507	-0.026668134	0.043172873	-0.617705784	0.536769299	0.733080572	TTC5	protein_coding
ENSG00000136327.7	25.61076492	0.975913717	0.365408707	2.670745661	0.007568297	0.049699167	NKX2-8	protein_coding

ENSG00000136352.19	11.06530153	0.330268681	0.417405097	0.791242568	0.428802455	0.646997147	NKX2-1	protein_coding
ENSG00000136367.14	353.4750519	0.129887507	0.094140534	1.379719247	0.167673104	0.376769498	ZFHX2	protein_coding
ENSG00000136371.11	112.2501817	-0.192192017	0.088791348	-2.164535417	0.030423281	0.128733721	MTHFS	protein_coding
ENSG00000136378.15	1006.630192	0.450597761	0.095768022	4.705096251	2.53746E-06	0.00010948	ADAMTS7	protein_coding
ENSG00000136379.12	6445.502465	0.184714612	0.088793408	2.080273927	0.037500415	0.147500278	ABHD17C	protein_coding
ENSG00000136381.13	3995.110661	0.139102851	0.050858402	2.735100702	0.006236119	0.043417699	IREB2	protein_coding
ENSG00000136383.7	1427.831558	0.342646903	0.146466957	2.339414359	0.019313999	0.094641419	ALPK3	protein_coding
ENSG00000136404.16	80.23496014	0.023477169	0.110204645	0.213032483	0.831301626	0.916918876	TM6SF1	protein_coding
ENSG00000136425.14	551.6209039	-0.101870935	0.145610502	-0.699612556	0.484169299	0.692149146	CIB2	protein_coding
ENSG00000136436.15	7370.057491	-0.139784886	0.049197766	-2.841285236	0.00449321	0.034520563	CALCOCO2	protein_coding
ENSG00000136444.10	1358.173155	-0.092097413	0.045334792	-2.031495203	0.042204786	0.159579865	RSAD1	protein_coding
ENSG00000136448.13	7339.24123	0.051393738	0.036283328	1.416456	0.156642038	0.361526936	NMT1	protein_coding
ENSG00000136449.15	31.2343928	0.079470974	0.126767	0.626905851	0.530720952	0.728669048	MYCBPAP	protein_coding
ENSG00000136450.13	3767.187052	-0.027255384	0.045755033	-0.595680575	0.551388637	0.743659228	SRSF1	protein_coding
ENSG00000136451.9	3957.086675	0.040967683	0.048430552	0.84590576	0.397605319	0.620774855	VEZF1	protein_coding
ENSG00000136457.10	58.59701115	-0.134722135	0.163435605	-0.824313253	0.40976159	0.630737577	CHAD	protein_coding
ENSG00000136463.8	1072.753652	-0.039210942	0.054379071	-0.721066788	0.470868424	0.682035343	TACO1	protein_coding
ENSG00000136478.8	3032.450245	-0.009142092	0.05135001	-0.178034859	0.8586956	0.931200929	TEX2	protein_coding
ENSG00000136485.15	9277.222762	0.020740566	0.041432419	0.50058787	0.616661201	0.78984887	DCAF7	protein_coding
ENSG00000136487.18	1.02265744	-0.093135562	0.537057613	-0.173418196	0.862322723	0.932938848	GH2	protein_coding
ENSG00000136488.15	0.266637746	0.121476397	0.64727814	0.187672639	0.85113328		CSH1	protein_coding
ENSG00000136490.9	1619.071054	-0.441291721	0.118465614	-3.7250617	0.000195267	0.003326493	LIMD2	protein_coding
ENSG00000136492.9	738.3153119	-0.122959093	0.084629693	-1.452907224	0.146249515	0.346704119	BRIP1	protein_coding
ENSG00000136504.13	3178.442594	0.004398674	0.038653192	0.113798473	0.909397549	0.957123052	KAT7	protein_coding
ENSG00000136514.3	754.7479898	0.07292392	0.100701472	0.724159426	0.468967865	0.680345318	RTP4	protein_coding
ENSG00000136518.17	2834.686883	0.162770141	0.063053786	2.581449137	0.009838649	0.059737885	ACTL6A	protein_coding
ENSG00000136521.13	4701.071333	-0.109663479	0.057601658	-1.903825031	0.056932975	0.193474873	NDUFB5	protein_coding
ENSG00000136522.14	2447.807743	0.013819016	0.067870326	0.203609107	0.838658976	0.920636147	MRPL47	protein_coding
ENSG00000136527.18	7862.021225	-0.016646685	0.036980935	-0.450142364	0.652607792	0.813167789	TRA2B	protein_coding
ENSG00000136531.18	197.169172	0.657207791	0.230375091	2.852772785	0.00433396	0.033587225	SCN2A	protein_coding
ENSG00000136535.15	7.544755501	-0.196411683	0.233008893	-0.842936427	0.399264	0.622116327	TBR1	protein_coding
ENSG00000136536.15	4380.303399	0.005153525	0.043101275	0.119567801	0.904825528	0.954665198	MARCHF7	protein_coding
ENSG00000136541.15	71.70517183	-0.371052093	0.138508743	-2.678907381	0.007386282	0.048894428	ERMN	protein_coding
ENSG00000136542.9	3333.747975	0.589217304	0.156312301	3.769487753	0.000163583	0.002905264	GALNT5	protein_coding
ENSG00000136546.16	273.5155342	-0.381036451	0.228682789	-1.666222685	0.095669069	0.267814413	SCN7A	protein_coding
ENSG00000136560.14	2933.049118	-0.08743663	0.044931786	-1.945986079	0.051656412	0.181710644	TANK	protein_coding
ENSG00000136573.14	253.5210406	-1.131075754	0.216936359	-5.213859776	1.84951E-07	1.34327E-05	BLK	protein_coding
ENSG00000136574.18	5931.719838	0.015805507	0.1840071	0.085896178	0.931548949	0.967563069	GATA4	protein_coding
ENSG00000136603.14	6459.417712	0.127253702	0.078591234	1.619184419	0.105407589	0.284233332	SKIL	protein_coding
ENSG00000136628.18	8666.145137	0.030035862	0.057285413	0.524319548	0.600056303	0.778534954	EPRS1	protein_coding
ENSG00000136630.13	190.494237	0.145090986	0.083821725	1.730947262	0.083461171	0.246675763	HLX	protein_coding
ENSG00000136631.15	2059.331811	0.07948787	0.042905422	1.852629972	0.063935413	0.207918919	VPS45	protein_coding

ENSG00000136634.7	45.57872675	0.170413178	0.120042434	1.419607826	0.155721886	0.36031926	IL10	protein_coding
ENSG00000136636.13	4483.845285	0.145831127	0.065473514	2.22733009	0.025925221	0.115684579	KCTD3	protein_coding
ENSG00000136643.12	1408.729315	0.041766598	0.049715261	0.840116235	0.400843218	0.623280215	RPS6KC1	protein_coding
ENSG00000136682.15	711.904256	-0.098369829	0.045202503	-2.176203158	0.029540073	0.126252232	CBWD2	protein_coding
ENSG00000136688.11	7.878565927	0.1361848	0.241586917	0.563709333	0.572951964	0.758746606	IL36G	protein_coding
ENSG00000136689.19	4815.393241	0.311145257	0.162261532	1.917554045	0.055167582	0.189504782	IL1RN	protein_coding
ENSG00000136694.9	9.593661083	0.617227294	0.461905952	1.336261834	0.181463707	0.394841798	IL36A	protein_coding
ENSG00000136695.15	17.44200463	1.264373444	0.3344423	3.780542848	0.000156487	0.002822527	IL36RN	protein_coding
ENSG00000136696.11	5.318462407	1.009457606	0.284074529	3.553495654	0.000380147	0.005537338	IL36B	protein_coding
ENSG00000136697.13	1.000799642	-0.168934962	0.399637422	-0.422720579	0.672499139	0.825855855	IL1F10	protein_coding
ENSG00000136698.10	3.527944159	-0.713760927	0.371067998	-1.923531352	0.054413353	0.187830377	CFC1	protein_coding
ENSG00000136699.19	5490.111991	-0.010546101	0.048150181	-0.219025169	0.826630442	0.914567618	SMPD4	protein_coding
ENSG00000136709.12	3824.177486	-0.031986611	0.028409916	-1.125896023	0.260209533	0.487422536	WDR33	protein_coding
ENSG00000136710.10	1731.264165	-0.168432015	0.056602744	-2.97568638	0.002923337	0.025162363	CCDC115	protein_coding
ENSG00000136715.19	3150.086831	-0.018782981	0.040070534	-0.468747961	0.639249801	0.804638607	SAP130	protein_coding
ENSG00000136717.15	3840.817922	-0.181858229	0.081707042	-2.225735076	0.026031932	0.115990147	BIN1	protein_coding
ENSG00000136718.10	4061.777273	-0.217485493	0.060258613	-3.609201765	0.000307141	0.004731095	IMP4	protein_coding
ENSG00000136720.7	2293.624941	0.176997099	0.074043888	2.39043498	0.016828429	0.086661833	HS6ST1	protein_coding
ENSG00000136731.13	8970.640054	0.054893197	0.044174697	1.242638908	0.214000926	0.434877744	UGGT1	protein_coding
ENSG00000136732.16	1429.468315	-0.143460344	0.121424316	-1.181479533	0.237412279	0.46264712	GYPC	protein_coding
ENSG00000136738.15	1850.250908	-0.020480022	0.047655436	-0.429752069	0.667376002	0.822410826	STAM	protein_coding
ENSG00000136750.13	5.525919321	0.06563109	0.287757974	0.228077399	0.81958607	0.911122996	GAD2	protein_coding
ENSG00000136754.18	5171.423788	-0.063231685	0.043279756	-1.460999085	0.144015693	0.343390788	ABI1	protein_coding
ENSG00000136758.19	8915.493372	-0.011421582	0.043357981	-0.263425139	0.792222917	0.895784787	YME1L1	protein_coding
ENSG00000136770.11	2416.492786	0.042909211	0.060255592	0.712119987	0.476390479	0.686425944	DNAJC1	protein_coding
ENSG00000136783.10	1225.739538	0.05524159	0.059093889	0.93481054	0.349885947	0.579072537	NIPSNAP3A	protein_coding
ENSG00000136802.12	6734.600478	0.402152488	0.077547585	5.185880235	2.14997E-07	1.49876E-05	LRRC8A	protein_coding
ENSG00000136807.14	2908.85928	0.040795413	0.042819264	0.952735019	0.340724345	0.570693962	CDK9	protein_coding
ENSG00000136810.13	16253.53318	-0.232206571	0.084187417	-2.758209946	0.005811886	0.041392836	TXN	protein_coding
ENSG00000136811.16	2364.273601	0.08603169	0.053960088	1.59435785	0.110855907	0.292720589	ODF2	protein_coding
ENSG00000136813.14	8053.663619	-0.013736993	0.049888729	-0.275352635	0.783045329	0.890081799	ECPAS	protein_coding
ENSG00000136816.16	2468.221402	0.090315875	0.058970765	1.531536426	0.125636871	0.315992031	TOR1B	protein_coding
ENSG00000136819.15	3151.510485	-0.092068104	0.048612321	-1.8939253	0.058234926	0.196154738	C9orf78	protein_coding
ENSG00000136824.19	3107.45711	0.001571256	0.071897214	0.021854197	0.982564262	0.992397797	SMC2	protein_coding
ENSG00000136826.15	6232.267642	0.200316828	0.110428096	1.814002374	0.06967736	0.219118881	KLF4	protein_coding
ENSG00000136827.12	2404.81253	0.104233475	0.043336585	2.405207417	0.016163289	0.08437322	TOR1A	protein_coding
ENSG00000136828.19	2058.649001	-0.151326364	0.071857497	-2.105923113	0.035211032	0.141353397	RALGPS1	protein_coding
ENSG00000136830.12	17628.61586	0.325329005	0.080642069	4.034234335	5.47806E-05	0.001234959	NIBAN2	protein_coding
ENSG00000136834.3	2.57462927	0.02378552	0.260772183	0.091211875	0.92732424	0.965674498	OR1J1	protein_coding
ENSG00000136839.1	0.211127613	0.059765169	0.828150215	0.072167064	0.942468956		OR13C9	protein_coding
ENSG00000136840.19	2278.106942	-0.004692703	0.085661507	-0.054781929	0.956312198	0.979676141	ST6GALNAC4	protein_coding
ENSG00000136842.14	427.5105122	-0.084110011	0.176335312	-0.476989038	0.633369927	0.801101829	TMOD1	protein_coding

ENSG00000136848.17	8863.162024	-0.166715787	0.075260543	-2.21518183	0.026747586	0.118200951	DAB2IP	protein_coding
ENSG00000136854.24	2979.933658	0.133326816	0.124972047	1.066853105	0.286038174	0.515495678	STXBP1	protein_coding
ENSG00000136856.18	1319.329842	-0.129677411	0.071563921	-1.812050112	0.069978454	0.219770127	SLC2A8	protein_coding
ENSG00000136859.10	5512.390469	0.28969323	0.122232869	2.37001089	0.017787561	0.089745115	ANGPTL2	protein_coding
ENSG00000136861.18	3260.881162	-0.039689577	0.048879087	-0.811995052	0.416794471	0.637196627	CDK5RAP2	protein_coding
ENSG00000136866.14	146.1053315	0.113253266	0.10925776	1.036569534	0.299936518	0.529586719	ZFP37	protein_coding
ENSG00000136867.11	27.106414	0.073780761	0.09844737	0.749443698	0.453589821	0.667979493	SLC31A2	protein_coding
ENSG00000136868.11	4824.94578	0.017213488	0.069221142	0.248673852	0.803613077	0.902413155	SLC31A1	protein_coding
ENSG00000136869.16	1156.178216	0.100406443	0.108054009	0.929224597	0.352772703	0.581179391	TLR4	protein_coding
ENSG00000136870.10	1389.552587	-0.138435996	0.062683086	-2.208506394	0.027208993	0.119548928	ZNF189	protein_coding
ENSG00000136872.20	3876.774848	-0.816183892	0.248675971	-3.282118046	0.001030305	0.0117442	ALDOB	protein_coding
ENSG00000136874.11	2463.810533	-0.043668366	0.041377292	-1.055370328	0.291255988	0.521277402	STX17	protein_coding
ENSG00000136875.13	2606.638536	0.066648479	0.053758293	1.239780431	0.215056618	0.436128176	PRPF4	protein_coding
ENSG00000136877.15	4100.773854	0.039901115	0.056886303	0.701418665	0.483041777	0.691193639	FPGS	protein_coding
ENSG00000136878.14	1913.271864	0.011136671	0.057188136	0.19473743	0.845598508	0.924295023	USP20	protein_coding
ENSG00000136881.12	379.3908359	-0.784950469	0.238685981	-3.288632481	0.001006754	0.011551163	BAAT	protein_coding
ENSG00000136883.14	1431.034791	-0.174145774	0.133592561	-1.303558915	0.192384016	0.408616239	KIF12	protein_coding
ENSG00000136888.8	5857.25203	-0.080264109	0.056142003	-1.429662369	0.152813946	0.356099216	ATP6V1G1	protein_coding
ENSG00000136891.14	1727.907839	-0.05149891	0.050756097	-1.014634953	0.310279871	0.539724179	TEX10	protein_coding
ENSG00000136895.19	269.8796296	0.010455931	0.09047989	0.115560829	0.908000608	0.956371124	GARNL3	protein_coding
ENSG00000136897.8	2156.813641	-0.222671193	0.06057035	-3.676240787	0.000236696	0.003870359	MRPL50	protein_coding
ENSG00000136908.18	3540.628657	-0.129169943	0.065223133	-1.980431428	0.047655071	0.172623861	DPM2	protein_coding
ENSG00000136918.8	8.808215277	0.301059234	0.162395657	1.85386259	0.063758821	0.207600845	WDR38	protein_coding
ENSG00000136925.15	698.2560012	0.036065643	0.063564737	0.567384445	0.570453015	0.756920768	TSTD2	protein_coding
ENSG00000136928.7	54.30388147	0.164311106	0.196341951	0.836861943	0.402670175	0.624542931	GABBR2	protein_coding
ENSG00000136929.13	4.283250486	-0.54702845	0.215773849	-2.535193449	0.011238524	0.065565258	HEMGN	protein_coding
ENSG00000136930.13	6267.022014	-0.0341435	0.05212196	-0.655069373	0.51242309	0.714990266	PSMB7	protein_coding
ENSG00000136931.10	7.019699095	0.849275391	0.21536015	3.943512257	8.02969E-05	0.001674209	NR5A1	protein_coding
ENSG00000136932.14	568.2535698	-0.079337668	0.045980704	-1.725455699	0.084445381	0.248299483	TRMO	protein_coding
ENSG00000136933.17	1228.8652	-0.091775989	0.050056924	-1.833432454	0.066738285	0.213355898	RABEPK	protein_coding
ENSG00000136935.14	2049.888888	-0.020494369	0.044980328	-0.4556296	0.648656354	0.810328266	GOLGA1	protein_coding
ENSG00000136936.11	776.7141458	-0.130538681	0.05137165	-2.541064597	0.01105155	0.064765114	XPA	protein_coding
ENSG00000136937.13	3550.849948	0.024575691	0.054379066	0.451932939	0.651317294	0.812250056	NCBP1	protein_coding
ENSG00000136938.9	10484.89887	-0.155530672	0.055010482	-2.827291552	0.004694357	0.035594774	ANP32B	protein_coding
ENSG00000136939.1	0.696038439	-0.109529912	0.370383911	-0.295719951	0.76744396		OR1L4	protein_coding
ENSG00000136940.14	1312.853931	0.041003357	0.040401947	1.014885661	0.310160334	0.539653457	PDCL	protein_coding
ENSG00000136942.15	29353.09255	-0.208783429	0.077059344	-2.709384966	0.006740808	0.045840227	RPL35	protein_coding
ENSG00000136943.11	1058.43783	-0.576429462	0.149237709	-3.862492034	0.000112236	0.002164123	CTSV	protein_coding
ENSG00000136944.19	174.8607372	0.891033538	0.231747108	3.844852889	0.000120625	0.002288935	LMX1B	protein_coding
ENSG00000136950.13	3009.167087	-0.081750491	0.056056166	-1.458367506	0.144739272	0.344642548	ARPC5L	protein_coding
ENSG00000136960.13	2190.135903	-0.373251832	0.120627555	-3.094250174	0.00197311	0.018930477	ENPP2	protein_coding
ENSG00000136982.6	816.2930117	-0.095784594	0.089247601	-1.073245595	0.283160938	0.512031548	DSCC1	protein_coding

ENSG00000136986.10	5152.844591	-0.008783002	0.047289523	-0.185728293	0.85265784	0.927953301	DERL1	protein_coding
ENSG00000136997.21	7350.495083	-0.038990906	0.098856924	-0.394417556	0.693272784	0.838038536	MYC	protein_coding
ENSG00000136999.5	470.6706225	0.76051482	0.146348486	5.196601882	2.02964E-07	1.43715E-05	CCN3	protein_coding
ENSG00000137033.11	1653.794185	0.010907435	0.168374871	0.064780659	0.948348641	0.975768844	IL33	protein_coding
ENSG00000137038.8	2074.7439	-0.167009138	0.073459497	-2.273486013	0.022996908	0.106905938	DMAC1	protein_coding
ENSG00000137040.10	1171.591265	-0.016593851	0.075131865	-0.220863027	0.825199087	0.913749571	RANBP6	protein_coding
ENSG00000137054.16	1222.702163	-0.034811007	0.058179316	-0.598339909	0.549613152	0.742438822	POLR1E	protein_coding
ENSG00000137055.15	2171.256339	-0.063989303	0.064102453	-0.998234853	0.318165489	0.547600519	PLAA	protein_coding
ENSG00000137070.18	202.0120467	-0.06706883	0.085774203	-0.781923096	0.434259771	0.651980639	IL11RA	protein_coding
ENSG00000137073.23	5531.458353	-0.033852214	0.056050216	-0.603962238	0.545868749	0.74002372	UBAP2	protein_coding
ENSG00000137074.20	1278.384238	-0.07436277	0.050497087	-1.472615048	0.140854865	0.338706128	APTX	protein_coding
ENSG00000137075.18	3393.993237	-0.031470172	0.062816306	-0.500987314	0.616380051	0.789640647	RNF38	protein_coding
ENSG00000137076.21	37019.49424	-0.021298435	0.086893557	-0.245109488	0.806371657	0.903764247	TLN1	protein_coding
ENSG00000137077.8	2098.696156	-0.26769221	0.184122697	-1.453879476	0.145979724	0.346422567	CCL21	protein_coding
ENSG00000137078.9	295.3617499	-0.710268203	0.146757345	-4.839745529	1.30005E-06	6.41591E-05	SIT1	protein_coding
ENSG00000137080.4	1.217743157	-0.944017073	0.363644735	-2.595987185	0.009431958	0.05793449	IFNA21	protein_coding
ENSG00000137090.12	6.345238022	0.374575838	0.261332332	1.433331402	0.151763152	0.354433939	DMRT1	protein_coding
ENSG00000137094.15	1207.397273	-0.390547374	0.158407352	-2.465462428	0.013683653	0.075279955	DNAJB5	protein_coding
ENSG00000137098.14	39.91110006	-0.100102556	0.096340098	-1.039053918	0.298779661	0.528640375	SPAG8	protein_coding
ENSG00000137100.16	2013.018057	-0.036090781	0.063512031	-0.568251089	0.569864486	0.756572668	DCTN3	protein_coding
ENSG00000137101.13	288.1457758	-0.366507087	0.12202788	-3.003470085	0.002669198	0.023569531	CD72	protein_coding
ENSG00000137103.20	1230.659677	-0.052514822	0.084988826	-0.617902662	0.536639504	0.733023792	TMEM8B	protein_coding
ENSG00000137106.18	3743.746103	-0.005134422	0.059980706	-0.085601224	0.931783425	0.967743741	GRHPR	protein_coding
ENSG00000137124.8	5573.328813	-0.229292874	0.115440271	-1.986246837	0.047005922	0.170954522	ALDH1B1	protein_coding
ENSG00000137133.11	8.831364235	-0.044061124	0.114417939	-0.385089302	0.700171254	0.842157404	HINT2	protein_coding
ENSG00000137135.18	843.2013324	0.026703174	0.099622829	0.268042723	0.788666434	0.893642862	ARHGEF39	protein_coding
ENSG00000137142.5	533.2630851	-0.051016305	0.21862272	-0.23335317	0.815487174	0.90890799	IGFBPL1	protein_coding
ENSG00000137145.20	4191.565916	-0.116521592	0.063632096	-1.831176398	0.067074218	0.214035131	DENND4C	protein_coding
ENSG00000137154.13	67239.65777	-0.195947181	0.071585172	-2.737259345	0.006195343	0.043230499	RPS6	protein_coding
ENSG00000137161.17	4378.79413	0.084854933	0.067216015	1.262421367	0.206797205	0.426698944	CNPY3	protein_coding
ENSG00000137166.16	14789.75139	0.117829794	0.084740006	1.390486031	0.164381338	0.371962072	FOXP4	protein_coding
ENSG00000137168.8	2325.211193	-0.003752513	0.062452584	-0.060085792	0.952087306	0.977481826	PPIL1	protein_coding
ENSG00000137171.15	2003.520443	0.284694915	0.09165003	3.106326495	0.001894274	0.018384587	KLC4	protein_coding
ENSG00000137177.20	3962.207628	-0.078371981	0.063799023	-1.228419772	0.219289429	0.441017882	KIF13A	protein_coding
ENSG00000137185.13	458.7855382	0.114502401	0.059059224	1.938772528	0.052529043	0.183462218	ZSCAN9	protein_coding
ENSG00000137193.14	3189.2291	0.287495946	0.087704987	3.277988563	0.001045496	0.011868303	PIM1	protein_coding
ENSG00000137198.10	416.8739947	-0.013922758	0.131756332	-0.105670501	0.915843786	0.960083828	GMPR	protein_coding
ENSG00000137200.13	3253.395478	-0.003731103	0.051541078	-0.072390868	0.942290852	0.972807906	CMTR1	protein_coding
ENSG00000137203.15	1405.531961	0.543673417	0.141249269	3.849035258	0.000118584	0.002259524	TFAP2A	protein_coding
ENSG00000137204.15	9.14316735	-0.054989165	0.223718623	-0.245796098	0.805840078	0.903489903	SLC22A7	protein_coding
ENSG00000137207.12	7954.289381	0.209651858	0.065608574	3.195494825	0.001395913	0.014807618	YIPF3	protein_coding
ENSG00000137210.14	2501.422148	-0.168017835	0.063556067	-2.643615975	0.008202566	0.052658105	TMEM14B	protein_coding

ENSG00000137216.19	5803.113071	0.304424706	0.081423645	3.738775201	0.000184919	0.003213004	TMEM63B	protein_coding
ENSG00000137218.10	349.9078059	-0.032991553	0.070553931	-0.467607585	0.64006524	0.80515942	FRS3	protein_coding
ENSG00000137221.14	2157.740609	0.160206262	0.061312687	2.6129382	0.008976753	0.055970808	TJAP1	protein_coding
ENSG00000137225.13	41.014276	-0.060641785	0.120038579	-0.505185793	0.613428348	0.787745833	CAPN11	protein_coding
ENSG00000137251.16	143.6477235	-0.650560188	0.242652529	-2.681036093	0.00733946	0.048650996	TINAG	protein_coding
ENSG00000137252.9	2.715842538	0.086657461	0.287112098	0.301824484	0.762785865	0.878851309	HCRTR2	protein_coding
ENSG00000137261.14	81.96106535	-0.147704915	0.163489363	-0.903452756	0.366285651	0.59341956	KIAA0319	protein_coding
ENSG00000137265.15	607.3870683	-0.818269173	0.147305009	-5.554931093	2.77721E-08	2.81448E-06	IRF4	protein_coding
ENSG00000137266.15	2906.708414	-0.128058489	0.089100028	-1.437244095	0.150648648	0.353004039	SLC22A23	protein_coding
ENSG00000137267.6	1516.523755	0.004187338	0.098670329	0.042437657	0.96614981	0.984485957	TUBB2A	protein_coding
ENSG00000137269.15	2388.830427	0.138172174	0.066912927	2.064954854	0.038927272	0.151350262	LRRC1	protein_coding
ENSG00000137270.11	14.87372026	-0.685214651	0.206333378	-3.320910358	0.000897244	0.010558785	GCM1	protein_coding
ENSG00000137273.6	1071.300439	0.086825154	0.13510558	0.64264669	0.52045338	0.720588324	FOXF2	protein_coding
ENSG00000137274.13	599.5487347	-0.171465425	0.067975955	-2.522442321	0.011654306	0.067212771	BPHL	protein_coding
ENSG00000137275.15	2965.60896	0.008348621	0.039199944	0.212975338	0.831346199	0.91692428	RIPK1	protein_coding
ENSG00000137285.10	295.4766611	0.782011761	0.173392857	4.510057526	6.481E-06	0.000231115	TUBB2B	protein_coding
ENSG00000137288.10	1373.918979	-0.134208151	0.070287539	-1.909415994	0.056208449	0.19173351	UQCC2	protein_coding
ENSG00000137309.20	34718.41421	0.132929392	0.095634224	1.38997721	0.164535798	0.372195622	HMGA1	protein_coding
ENSG00000137310.12	1853.234478	0.043461253	0.076860991	0.565452675	0.571765905	0.757873307	TCF19	protein_coding
ENSG00000137312.15	8142.04963	0.148858263	0.057872041	2.572196526	0.010105551	0.060933941	FLOT1	protein_coding
ENSG00000137331.12	4384.74513	0.342060574	0.12522808	2.731500593	0.006304663	0.043776018	IER3	protein_coding
ENSG00000137337.15	2620.407796	0.127395979	0.060043463	2.121729374	0.03386047	0.137865262	MDC1	protein_coding
ENSG00000137338.5	222.5934178	0.239464598	0.090350852	2.650385614	0.008039995	0.051958898	PGBD1	protein_coding
ENSG00000137343.18	1099.709379	-0.011671342	0.066824207	-0.174657396	0.861348846	0.932477049	ATAT1	protein_coding
ENSG00000137364.5	3207.887216	-0.131989777	0.080189064	-1.645982271	0.099767409	0.275028625	TPMT	protein_coding
ENSG00000137392.10	11.18840083	-0.950411487	0.284840604	-3.336643279	0.000847967	0.010112715	CLPS	protein_coding
ENSG00000137393.10	1158.168892	-0.114635968	0.106103931	-1.080412072	0.279958722	0.508704914	RNF144B	protein_coding
ENSG00000137404.14	1578.646232	-0.01704508	0.074504039	-0.228780622	0.819039429	0.910863648	NRM	protein_coding
ENSG00000137409.19	8576.606762	0.093388515	0.044356752	2.105395712	0.035256877	0.14145925	MTCH1	protein_coding
ENSG00000137411.19	28.05740818	0.140038952	0.10486572	1.335412099	0.181741507	0.395177369	VARS2	protein_coding
ENSG00000137413.16	2094.605972	0.072882959	0.05616842	1.297578952	0.194432059	0.411239422	TAF8	protein_coding
ENSG00000137414.6	3351.157179	-0.05902303	0.057832771	-1.020581055	0.307452969	0.536980818	FAM8A1	protein_coding
ENSG00000137434.11	46.16888518	-0.047455454	0.110542959	-0.429294231	0.667709114	0.822569759	C6orf52	protein_coding
ENSG00000137440.5	654.6729133	1.889220669	0.261044787	7.23715148	4.58206E-13	2.81229E-10	FGFBP1	protein_coding
ENSG00000137441.8	25.48838393	0.311390002	0.139600265	2.230583177	0.025708752	0.11509249	FGFBP2	protein_coding
ENSG00000137449.17	1574.391851	0.053479577	0.068746091	0.777928995	0.436610874	0.654023308	CPEB2	protein_coding
ENSG00000137460.9	1266.356199	-0.269695318	0.092385037	-2.919253229	0.003508711	0.028877791	FHDC1	protein_coding
ENSG00000137462.9	838.0944447	0.242890722	0.103054038	2.3569258	0.018426928	0.091980557	TLR2	protein_coding
ENSG00000137463.5	8.600989915	0.163937438	0.161821384	1.013076479	0.311023632	0.54051752	MGARP	protein_coding
ENSG00000137473.18	4.958847107	-0.329400131	0.315734246	-1.043282873	0.296817301	0.526623815	TTC29	protein_coding
ENSG00000137474.22	982.9720542	0.071043114	0.10383063	0.684221159	0.493835523	0.700790367	MYO7A	protein_coding
ENSG00000137478.15	1881.096798	-0.071732532	0.062210688	-1.153058005	0.248886577	0.475585633	FCHSD2	protein_coding

ENSG00000137486.17	2920.075871	-0.059302805	0.079460439	-0.746318615	0.455474969	0.669558345	ARRB1	protein_coding
ENSG00000137491.15	5110.439782	-0.135016468	0.106095038	-1.272599268	0.203160279	0.422427129	SLCO2B1	protein_coding
ENSG00000137492.8	3229.59285	0.01292841	0.057666976	0.224190878	0.822608781	0.912563783	THAP12	protein_coding
ENSG00000137494.14	719.9089381	0.03368186	0.046603698	0.722729332	0.469846191	0.681211321	ANKRD42	protein_coding
ENSG00000137496.18	1689.838887	-0.087589688	0.106968738	-0.818834445	0.412880876	0.633480386	IL18BP	protein_coding
ENSG00000137497.18	11547.88363	0.086285162	0.052417498	1.646113703	0.099740352	0.275006364	NUMA1	protein_coding
ENSG00000137500.9	1825.837598	0.014160086	0.044416029	0.318805769	0.749873804	0.871581088	CCDC90B	protein_coding
ENSG00000137501.17	4188.056328	-0.15517782	0.112123138	-1.383994625	0.1663601	0.374801862	SYTL2	protein_coding
ENSG00000137502.10	1272.52667	-0.245877445	0.086064494	-2.856897573	0.004278039	0.033252903	RAB30	protein_coding
ENSG00000137504.14	3445.809694	0.093163993	0.065229165	1.428256713	0.153217982	0.356760887	CREBZF	protein_coding
ENSG00000137507.11	3690.870182	0.43286652	0.109383494	3.957329442	7.57924E-05	0.001600196	LRRC32	protein_coding
ENSG00000137509.11	4410.977215	0.031833132	0.064009284	0.497320545	0.618963026	0.791285586	PRCP	protein_coding
ENSG00000137513.10	993.145273	-0.084070707	0.060956444	-1.3791931	0.167835225	0.376919996	NARS2	protein_coding
ENSG00000137522.18	1069.558911	0.082771957	0.048058072	1.722332042	0.085009386	0.249407799	RNF121	protein_coding
ENSG00000137547.9	2793.62337	-0.206485096	0.067370669	-3.064910872	0.002177349	0.020356646	MRPL15	protein_coding
ENSG00000137558.9	737.8197927	0.72999026	0.194444264	3.7542391	0.000173869	0.003050194	PI15	protein_coding
ENSG00000137561.5	110.5160946	0.364794664	0.221392534	1.647727942	0.099408512	0.274606954	TTPA	protein_coding
ENSG00000137563.13	3062.656843	0.140230707	0.139369675	1.006178046	0.314329931	0.543553786	GGH	protein_coding
ENSG00000137571.11	179.6394511	-0.190281118	0.129933636	-1.464448505	0.143071443	0.341941769	SLCO5A1	protein_coding
ENSG00000137573.14	15529.55004	0.782523415	0.134832844	5.803655778	6.48844E-09	8.41508E-07	SULF1	protein_coding
ENSG00000137574.11	2143.043698	-0.109845891	0.058095062	-1.890795658	0.058651627	0.196968399	TGS1	protein_coding
ENSG00000137575.12	12841.95626	0.111609186	0.060381814	1.848390733	0.064545838	0.209061543	SDCBP	protein_coding
ENSG00000137601.17	798.5540105	-0.062071689	0.054408565	-1.140844078	0.253934814	0.480679267	NEK1	protein_coding
ENSG00000137628.17	3692.624732	-0.058233207	0.11187812	-0.520505773	0.602711105	0.780608151	DDX60	protein_coding
ENSG00000137634.10	8.257341382	0.640434494	0.24823837	2.579917413	0.009882395	0.059915127	NXPE4	protein_coding
ENSG00000137642.13	6096.839304	0.074948722	0.101184144	0.740716075	0.458865611	0.672134007	SORL1	protein_coding
ENSG00000137648.19	9562.3356	0.203032559	0.120635798	1.683020816	0.092371062	0.262368254	TMPRSS4	protein_coding
ENSG00000137656.12	1174.126974	-0.082919699	0.043982876	-1.885272343	0.059393078	0.198616542	BUD13	protein_coding
ENSG00000137672.13	244.0289933	0.077294307	0.090606954	0.853072561	0.393619066	0.617539563	TRPC6	protein_coding
ENSG00000137673.9	8107.188775	0.622367288	0.236592994	2.63053981	0.008524938	0.05400367	MMP7	protein_coding
ENSG00000137674.4	8.179809079	-0.201779243	0.239679931	-0.84186958	0.399860959	0.622690242	MMP20	protein_coding
ENSG00000137675.5	3.534015567	-0.308584695	0.219694273	-1.404609646	0.160137387	0.366046313	MMP27	protein_coding
ENSG00000137691.13	102.5413269	0.318974165	0.140392543	2.272016435	0.023085518	0.107146406	CFAP300	protein_coding
ENSG00000137692.12	1403.557129	0.015912964	0.05468739	0.290980508	0.771066227	0.88416054	DCUN1D5	protein_coding
ENSG00000137693.14	7742.24681	-0.002496591	0.068005049	-0.036711853	0.970714757	0.986217406	YAP1	protein_coding
ENSG00000137699.17	7033.013814	0.95901403	0.211310934	4.538402301	5.6682E-06	0.000207218	TRIM29	protein_coding
ENSG00000137700.18	1594.839348	-0.299070511	0.078983999	-3.786469617	0.000152803	0.002768685	SLC37A4	protein_coding
ENSG00000137707.13	1.724713876	-0.453623905	0.344344273	-1.317355742	0.187719409	0.402888864	BTG4	protein_coding
ENSG00000137709.10	281.198285	-0.515586349	0.162671616	-3.169491782	0.001527058	0.015720434	POU2F3	protein_coding
ENSG00000137710.17	2621.571737	0.219972955	0.094932897	2.317141495	0.020496025	0.098800364	RDX	protein_coding
ENSG00000137713.16	2695.215947	0.018578636	0.0618825	0.300224395	0.764005999	0.879678529	PPP2R1B	protein_coding
ENSG00000137714.3	1858.416346	-0.193166022	0.066091038	-2.922726411	0.003469813	0.028656557	FDX1	protein_coding

ENSG00000137720.8	1116.972308	-0.310368429	0.080182564	-3.870772054	0.000108491	0.002107767	C11orf1	protein_coding
ENSG00000137726.17	441.5949292	0.110817191	0.140226641	0.790272024	0.429368921	0.647605201	FXYD6	protein_coding
ENSG00000137727.12	186.0037038	0.017015341	0.136628779	0.12453702	0.900890091	0.952773699	ARHGAP20	protein_coding
ENSG00000137731.14	15.4584261	0.185058759	0.192141687	0.963136951	0.335478801	0.564948011	FXYD2	protein_coding
ENSG00000137745.12	114.6137598	0.944145308	0.20718356	4.557047431	5.18777E-06	0.000192234	MMP13	protein_coding
ENSG00000137747.16	275.3242859	0.507016588	0.222739244	2.276278655	0.022829337	0.106394469	TMPRSS13	protein_coding
ENSG00000137752.24	2341.920656	-0.53860705	0.126881302	-4.244967878	2.18625E-05	0.000599183	CASP1	protein_coding
ENSG00000137757.11	138.7708155	-0.69569691	0.194679835	-3.573543753	0.000352182	0.005248649	CASP5	protein_coding
ENSG00000137760.15	480.5574258	-0.059710227	0.047111254	-1.267430227	0.205001497	0.424567274	ALKBH8	protein_coding
ENSG00000137764.20	933.5807886	-0.058113699	0.044050843	-1.319241489	0.187088392	0.402029235	MAP2K5	protein_coding
ENSG00000137766.17	22.65293018	-0.33776875	0.230853848	-1.463128089	0.143432333	0.34254317	UNC13C	protein_coding
ENSG00000137767.14	2064.847057	-0.012758322	0.086723262	-0.147115342	0.88304098	0.943927791	SQOR	protein_coding
ENSG00000137770.14	2590.17194	0.016429704	0.045056034	0.364650478	0.71537233	0.851254199	CTDSPL2	protein_coding
ENSG00000137776.17	6032.579921	-0.017591572	0.046420725	-0.37895944	0.704717984	0.84521176	SLTM	protein_coding
ENSG00000137801.11	26089.93094	0.554476094	0.12542478	4.420785854	9.83426E-06	0.000321009	THBS1	protein_coding
ENSG00000137802.14	1405.181518	-0.022664051	0.060210611	-0.376412913	0.706609948	0.846312246	MAPKBP1	protein_coding
ENSG00000137804.14	19.0103964	0.033381254	0.087538939	0.381330353	0.702958134	0.843995222	NUSAP1	protein_coding
ENSG00000137806.10	594.0154787	-0.086241405	0.046828373	-1.841648555	0.065526574	0.21099898	NDUFAF1	protein_coding
ENSG00000137807.16	1939.719949	0.064226019	0.090029979	0.713384801	0.475607676	0.685822491	KIF23	protein_coding
ENSG00000137808.12	0.002997893	-0.122775387	2.893054209	-0.042437984	0.966149549		AC027088.1	lncRNA
ENSG00000137809.17	3062.124271	0.842823502	0.127744981	6.597703472	4.17575E-11	1.29976E-08	ITGA11	protein_coding
ENSG00000137812.20	1948.090871	-0.023725264	0.09039415	-0.26246459	0.792963282	0.896204604	KNL1	protein_coding
ENSG00000137814.12	1335.422282	-0.028320556	0.049548217	-0.571575678	0.567609477	0.755189393	HAUS2	protein_coding
ENSG00000137815.14	5855.007585	-0.097102156	0.04849762	-2.002204564	0.045262735	0.166870914	RTF1	protein_coding
ENSG00000137817.17	1522.0544	-0.014325564	0.107686816	-0.133029879	0.89416975	0.949630416	PARP6	protein_coding
ENSG00000137818.12	70249.32797	-0.231093884	0.068075969	-3.394646996	0.000687171	0.008634622	RPLP1	protein_coding
ENSG00000137819.14	492.7760904	0.222843742	0.140488881	1.586201986	0.112693497	0.295905311	PAQR5	protein_coding
ENSG00000137821.12	277.9829423	0.093522509	0.090694007	1.031187305	0.302452993	0.531987652	LRRC49	protein_coding
ENSG00000137822.13	1239.325671	-0.01538829	0.047480516	-0.324096953	0.745864634	0.86921526	TUBGCP4	protein_coding
ENSG00000137824.16	2148.053251	-0.047257856	0.048606553	-0.972252765	0.330924817	0.560481436	RMDN3	protein_coding
ENSG00000137825.11	903.0247551	-0.435289298	0.131626769	-3.306996761	0.00094302	0.010967167	ITPKA	protein_coding
ENSG00000137831.15	4188.682836	0.161200797	0.057353756	2.810640642	0.004944297	0.036906072	UACA	protein_coding
ENSG00000137834.15	2178.003943	-0.191493958	0.08618927	-2.221784207	0.026297893	0.116782152	SMAD6	protein_coding
ENSG00000137841.12	991.0901626	-0.152538274	0.096761982	-1.576427753	0.114927257	0.299215861	PLCB2	protein_coding
ENSG00000137842.7	1585.926245	0.014640807	0.067079954	0.218259053	0.827227276	0.914923098	TMEM62	protein_coding
ENSG00000137843.12	833.9729487	0.389464507	0.122326555	3.183809984	0.001453504	0.015201126	PAK6	protein_coding
ENSG00000137845.15	8638.652082	0.139417735	0.059128087	2.357893566	0.018378962	0.091783178	ADAM10	protein_coding
ENSG00000137857.17	1324.233465	0.970355744	0.170585726	5.688375968	1.28253E-08	1.47854E-06	DUOX1	protein_coding
ENSG00000137860.12	1086.674348	-1.511124031	0.275792307	-5.479210236	4.27228E-08	4.02973E-06	SLC28A2	protein_coding
ENSG00000137868.19	842.4497401	0.515921314	0.187273514	2.75490807	0.005870863	0.041673388	STRA6	protein_coding
ENSG00000137869.15	59.01780579	0.652809955	0.155184389	4.206672836	2.59158E-05	0.000678685	CYP19A1	protein_coding
ENSG00000137871.20	959.3176616	-0.090878487	0.06126434	-1.483383107	0.137972653	0.334432879	ZNF280D	protein_coding

ENSG00000137872.17	558.4547854	-0.189693047	0.14098899	-1.345445814	0.178481309	0.391109921	SEMA6D	protein_coding
ENSG00000137875.4	87.56973258	0.145696848	0.197812953	0.736538462	0.461403069	0.674092711	BCL2L10	protein_coding
ENSG00000137876.11	4372.473053	-0.121442313	0.050625413	-2.398840911	0.016447059	0.085465475	RSL24D1	protein_coding
ENSG00000137877.10	354.5376131	0.113547928	0.132484841	0.857063551	0.391409764	0.615600549	SPTBN5	protein_coding
ENSG00000137878.17	404.7890405	0.191889067	0.138688003	1.383602492	0.166480205	0.374897666	GCOM1	protein_coding
ENSG00000137880.6	35.32102128	-0.215096143	0.113118151	-1.901517487	0.057234264	0.194108297	GCHFR	protein_coding
ENSG00000137936.18	1192.489786	0.204463415	0.080091403	2.552875928	0.010683755	0.063234671	BCAR3	protein_coding
ENSG00000137941.17	741.1977991	0.044752591	0.165865437	0.269812638	0.7873044	0.892667419	TTLL7	protein_coding
ENSG00000137942.17	4271.783074	0.045250759	0.066448682	0.680988052	0.49587905	0.702272388	FNBP1L	protein_coding
ENSG00000137944.18	1399.253311	-0.0731074	0.063552144	-1.150352999	0.24999851	0.476769588	KYAT3	protein_coding
ENSG00000137947.12	1642.050115	-0.015107757	0.049479378	-0.305334412	0.76011147	0.877126552	GTF2B	protein_coding
ENSG00000137948.18	18.61892162	0.248512214	0.228984695	1.085278709	0.277798233	0.506701837	BRDT	protein_coding
ENSG00000137955.16	2467.869669	-0.083197204	0.057721453	-1.441356722	0.14948393	0.351560323	RABGGTB	protein_coding
ENSG00000137959.16	2981.794417	-0.263425126	0.155527706	-1.693750479	0.090312708	0.258753061	IFI44L	protein_coding
ENSG00000137960.6	1428.358843	-0.205511624	0.124695713	-1.648104971	0.099331132	0.274515015	GIPC2	protein_coding
ENSG00000137962.13	1958.51751	0.106630559	0.089148608	1.196098977	0.231657937	0.455754307	ARHGAP29	protein_coding
ENSG00000137965.11	1838.508981	0.02432747	0.120879683	0.201253588	0.840500291	0.921699159	IFI44	protein_coding
ENSG00000137968.16	471.5346679	0.475134051	0.200209747	2.373181421	0.017635599	0.089288546	SLC44A5	protein_coding
ENSG00000137970.7	1357.378589	-0.409064902	0.098877335	-4.137094715	3.51731E-05	0.000865954	RPL7P9	processed_pseudogene
ENSG00000137975.8	72.29773068	-0.205747132	0.286630086	-0.717814151	0.472871887	0.68373277	CLCA2	protein_coding
ENSG00000137976.7	20.69811457	-0.5230956	0.188363113	-2.777059655	0.005485311	0.039680179	DNASE2B	protein_coding
ENSG00000137992.14	1720.206763	0.035785284	0.050902515	0.703016035	0.482045756	0.690737602	DBT	protein_coding
ENSG00000137996.13	2073.326994	-0.07194639	0.04301159	-1.672721006	0.094382232	0.265551053	RTCA	protein_coding
ENSG00000138002.16	1918.629556	-0.020260355	0.055145173	-0.367400336	0.713320421	0.850275288	IFT172	protein_coding
ENSG00000138018.18	4142.977489	-0.003156841	0.069936155	-0.045138901	0.963996595	0.983471548	SELENOI	protein_coding
ENSG00000138028.16	844.6470572	0.341841762	0.146399537	2.33499209	0.019543834	0.095477764	CGREF1	protein_coding
ENSG00000138029.14	5256.67652	-0.125350844	0.043803526	-2.861661041	0.004214273	0.032870126	HADHB	protein_coding
ENSG00000138030.13	370.0313984	-0.290083269	0.113150869	-2.563685726	0.010356727	0.061853564	KHK	protein_coding
ENSG00000138031.14	2316.051362	0.038230626	0.069007148	0.554009643	0.579572246	0.763658618	ADCY3	protein_coding
ENSG00000138032.21	1501.827322	-0.112137167	0.052236857	-2.146705869	0.031816705	0.132563014	PPM1B	protein_coding
ENSG00000138035.15	2442.719466	-0.003246518	0.057826942	-0.056141966	0.955228713	0.979204931	PNPT1	protein_coding
ENSG00000138036.18	902.3570146	-0.153543778	0.054481165	-2.818290994	0.004828003	0.036324222	DYNC2LI1	protein_coding
ENSG00000138039.15	6.634163169	-0.441913684	0.233298826	-1.894195923	0.058199009	0.196083054	LHCGR	protein_coding
ENSG00000138050.15	663.9434268	-0.068968231	0.051264084	-1.345351873	0.178511629	0.391109921	THUMPD2	protein_coding
ENSG00000138061.12	1847.875637	0.274265024	0.180130384	1.522591675	0.127860894	0.319302727	CYP1B1	protein_coding
ENSG00000138068.10	2.799829432	-0.413243917	0.320431054	-1.289650025	0.197172198	0.414420146	SULT6B1	protein_coding
ENSG00000138069.18	10263.44673	0.076206952	0.044063908	1.729464219	0.083726045	0.247058741	RAB1A	protein_coding
ENSG00000138071.14	16738.36743	-0.011602671	0.039728592	-0.292048383	0.770249631	0.883759036	ACTR2	protein_coding
ENSG00000138073.14	3072.142845	0.015278051	0.056287846	0.27142717	0.786062508	0.891735764	PREB	protein_coding
ENSG00000138074.15	3671.253397	-0.286198896	0.082063441	-3.487532236	0.0004875	0.006669951	SLC5A6	protein_coding
ENSG00000138075.14	112.7868221	-0.456162041	0.176655477	-2.582212838	0.009816902	0.059665873	ABCG5	protein_coding
ENSG00000138078.16	3283.183783	0.046341885	0.048997473	0.94580153	0.344249825	0.573951663	PREPL	protein_coding

ENSG00000138079.14	39.45167859	-0.800072555	0.153122442	-5.225050921	1.74107E-07	1.27846E-05	SLC3A1	protein_coding
ENSG00000138080.14	8163.055102	0.254437598	0.122750644	2.072800516	0.038190848	0.14935319	EMILIN1	protein_coding
ENSG00000138081.21	2160.78074	0.094226079	0.045245206	2.082564917	0.0372909	0.146930832	FBXO11	protein_coding
ENSG00000138083.5	88.79350168	1.361827933	0.259174672	5.254479229	1.48444E-07	1.11723E-05	SIX3	protein_coding
ENSG00000138085.17	5245.702866	-0.13138785	0.052537	-2.500863188	0.012389103	0.070105173	ATRAID	protein_coding
ENSG00000138092.11	748.4761643	9.53166E-05	0.066803446	0.001426822	0.998861561	0.999503783	CENPO	protein_coding
ENSG00000138095.19	12641.4888	-0.098256836	0.058607072	-1.676535475	0.093633354	0.264471134	LRPPRC	protein_coding
ENSG00000138100.14	272.0623936	-0.12456809	0.241731742	-0.515315402	0.606332638	0.782948332	TRIM54	protein_coding
ENSG00000138101.19	755.0868319	-0.116067947	0.085276079	-1.361084482	0.173486993	0.384558693	DTNB	protein_coding
ENSG00000138107.13	5769.684737	-0.031058461	0.044786882	-0.693472289	0.48801321	0.695495035	ACTR1A	protein_coding
ENSG00000138109.11	309.0596283	-0.224630615	0.189045412	-1.18823627	0.234740338	0.459442434	CYP2C9	protein_coding
ENSG00000138111.14	772.8510886	-0.185813	0.071771016	-2.588969905	0.009626351	0.058784681	MFSD13A	protein_coding
ENSG00000138115.14	38.59195025	-0.564752315	0.155155413	-3.639913712	0.000272729	0.00432644	CYP2C8	protein_coding
ENSG00000138119.17	12015.23366	0.251219996	0.099984217	2.512596528	0.011984631	0.068447481	MYOF	protein_coding
ENSG00000138131.4	338.008994	0.836148432	0.164124169	5.094608771	3.49462E-07	2.20384E-05	LOXL4	protein_coding
ENSG00000138134.12	872.2212347	-0.111510539	0.085185929	-1.309025328	0.190525771	0.406236292	STAMBPL1	protein_coding
ENSG00000138135.7	287.4413646	0.347452612	0.144366387	2.406741748	0.016095547	0.084140555	CH25H	protein_coding
ENSG00000138136.6	3.812920298	2.461714419	0.322353598	7.636689762	2.22878E-14	1.76588E-11	LBX1	protein_coding
ENSG00000138138.13	3161.374414	0.02378976	0.061132975	0.389147753	0.69716685	0.840446731	ATAD1	protein_coding
ENSG00000138152.10	136.7082631	0.269743698	0.172158961	1.566829259	0.117154606	0.302838194	BTBD16	protein_coding
ENSG00000138160.7	3169.911132	-0.066014438	0.078883134	-0.836863784	0.40266914	0.624542931	KIF11	protein_coding
ENSG00000138161.14	2.659061033	0.047957698	0.184739962	0.259595691	0.79517566	0.897430698	CUZD1	protein_coding
ENSG00000138162.19	6594.518031	-0.071471576	0.085048872	-0.840358897	0.400707188	0.623184052	TACC2	protein_coding
ENSG00000138166.6	1578.329427	0.355444434	0.12227345	2.90696332	0.00364956	0.029671058	DUSP5	protein_coding
ENSG00000138172.11	867.1793119	0.312366454	0.089426245	3.493006505	0.000477615	0.006573918	CALHM2	protein_coding
ENSG00000138175.9	1121.409428	-0.069240347	0.053080702	-1.30443541	0.192085168	0.408277016	ARL3	protein_coding
ENSG00000138180.16	2367.989873	0.010398973	0.089824152	0.115770341	0.907834557	0.956333677	CEP55	protein_coding
ENSG00000138182.15	2217.406378	-0.04844532	0.07198878	-0.672956534	0.500974944	0.706298655	KIF20B	protein_coding
ENSG00000138185.20	2731.153244	-0.054824221	0.072819925	-0.752873902	0.451525692	0.66618439	ENTPD1	protein_coding
ENSG00000138190.17	1421.280024	-0.153834302	0.058635788	-2.62355649	0.008701701	0.054804746	EXOC6	protein_coding
ENSG00000138193.16	1947.69882	-0.134113733	0.078955843	-1.698591611	0.089396158	0.257187323	PLCE1	protein_coding
ENSG00000138207.14	1663.758016	0.845011025	0.237445132	3.558763321	0.000372605	0.005457819	RBP4	protein_coding
ENSG00000138231.13	1670.53809	-0.337988854	0.083804505	-4.033063061	5.50545E-05	0.001240491	DBR1	protein_coding
ENSG00000138246.17	4468.865598	0.031846784	0.050589827	0.629509631	0.529015466	0.727186805	DNAJC13	protein_coding
ENSG00000138271.6	5.296970489	0.887825658	0.309807366	2.865734505	0.00416043	0.032601881	GPR87	protein_coding
ENSG00000138279.16	5018.389056	0.011481779	0.050891827	0.225611456	0.82150363	0.912230556	ANXA7	protein_coding
ENSG00000138286.15	1022.773236	-0.012606343	0.052611146	-0.23961354	0.810629867	0.90627579	FAM149B1	protein_coding
ENSG00000138303.18	1718.613392	-0.109038791	0.054637845	-1.995664182	0.045970488	0.168524941	ASCC1	protein_coding
ENSG00000138308.6	97.33696637	-0.425108033	0.290972228	-1.460991779	0.144017698	0.343390788	PLA2G12B	protein_coding
ENSG00000138311.18	57.78114938	0.660091249	0.168279489	3.92258885	8.76026E-05	0.001791392	ZNF365	protein_coding
ENSG00000138315.13	86.98377309	-0.372583381	0.148590266	-2.507454829	0.012160412	0.06916096	OIT3	protein_coding
ENSG00000138316.11	869.5088347	0.500432805	0.121658946	4.113407373	3.89862E-05	0.000943309	ADAMTS14	protein_coding

ENSG00000138326.21	47076.27505	-0.186275323	0.072781936	-2.559362023	0.010486447	0.062336664	RPS24	protein_coding
ENSG00000138336.9	248.0314076	0.000673404	0.109032881	0.006176159	0.995072169	0.997700212	TET1	protein_coding
ENSG00000138346.15	738.9360244	-0.187297659	0.08854959	-2.115172524	0.034415246	0.13931381	DNA2	protein_coding
ENSG00000138347.16	37.29363539	-0.073069975	0.18483086	-0.395334281	0.692596203	0.837699816	MYPN	protein_coding
ENSG00000138356.14	419.2804216	0.48434189	0.182994501	2.646756532	0.008126784	0.05233351	AOX1	protein_coding
ENSG00000138363.15	5642.663265	-0.126532209	0.056902087	-2.223683087	0.026169774	0.116418209	ATIC	protein_coding
ENSG00000138375.13	1344.66367	0.014531887	0.037175056	0.390904228	0.695868029	0.839507242	SMARCAL1	protein_coding
ENSG00000138376.11	1043.390549	0.048846687	0.06041277	0.808549032	0.418774592	0.638834293	BARD1	protein_coding
ENSG00000138378.19	229.0104455	-0.488348332	0.104158001	-4.688534048	2.75169E-06	0.000116543	STAT4	protein_coding
ENSG00000138379.5	14.44691301	-0.123676732	0.163306194	-0.757330316	0.448851993	0.663779822	MSTN	protein_coding
ENSG00000138380.18	463.1893707	-0.23543005	0.073749769	-3.192281882	0.001411535	0.014908291	CARF	protein_coding
ENSG00000138381.10	816.5061297	-0.037170599	0.043041809	-0.86359285	0.387811593	0.612231382	ASNSD1	protein_coding
ENSG00000138382.15	1664.959515	-0.168045469	0.058579676	-2.868665058	0.004122081	0.032412934	METTL5	protein_coding
ENSG00000138385.16	3789.854489	-0.030783584	0.055515506	-0.554504246	0.579233801	0.763396643	SSB	protein_coding
ENSG00000138386.17	3278.670172	0.247668179	0.07281093	3.401524726	0.000670111	0.008456824	NAB1	protein_coding
ENSG00000138395.17	38.01554137	-0.133766133	0.139362592	-0.959842458	0.33713451	0.566859997	CDK15	protein_coding
ENSG00000138398.17	6098.518874	-0.128552229	0.053856008	-2.386961709	0.016988261	0.087145077	PPIG	protein_coding
ENSG00000138399.18	1437.914643	-0.189732568	0.063809495	-2.973422162	0.002944991	0.025292202	FASTKD1	protein_coding
ENSG00000138400.13	39.44144289	-0.019495635	0.129692247	-0.150322283	0.880510353	0.94254341	MDH1B	protein_coding
ENSG00000138411.13	967.2156955	-0.002245886	0.089588887	-0.025068797	0.980000089	0.991148319	HECW2	protein_coding
ENSG00000138413.14	9230.396504	0.000479571	0.079245176	0.006051732	0.995171446	0.997712835	IDH1	protein_coding
ENSG00000138430.16	6505.920757	-0.11210043	0.061300759	-1.828695617	0.067445219	0.214859297	OLA1	protein_coding
ENSG00000138433.16	2316.675859	-0.117548883	0.057762801	-2.035027406	0.041848115	0.158630422	CIR1	protein_coding
ENSG00000138434.17	11537.40733	-0.03513391	0.078692642	-0.446470077	0.655257723	0.814693077	ITPRID2	protein_coding
ENSG00000138435.16	78.37271172	0.185242479	0.141137269	1.312498679	0.189351932	0.404862397	CHRNA1	protein_coding
ENSG00000138439.12	1738.480352	-0.048784007	0.064347204	-0.758137163	0.448368874	0.663448911	FAM117B	protein_coding
ENSG00000138442.10	1968.312041	-0.074373348	0.058277677	-1.276189299	0.20188861	0.420853617	WDR12	protein_coding
ENSG00000138443.17	2658.538998	0.089472475	0.05435077	1.646204346	0.099721695	0.274984009	ABI2	protein_coding
ENSG00000138448.12	10159.90429	0.202409333	0.073862185	2.740364826	0.006137102	0.042962886	ITGAV	protein_coding
ENSG00000138449.11	15246.30046	-0.177944121	0.109411718	-1.626371696	0.103870608	0.281773528	SLC40A1	protein_coding
ENSG00000138459.9	1791.367887	0.050005417	0.055387539	0.902827916	0.366617233	0.593729714	SLC35A5	protein_coding
ENSG00000138463.9	1187.891908	0.126544847	0.056283256	2.248356909	0.024553441	0.111675742	SLC49A4	protein_coding
ENSG00000138468.16	872.5063168	0.005283406	0.07868248	0.067148445	0.946463528	0.974890132	SENP7	protein_coding
ENSG00000138472.11	1.940921664	-0.362547575	0.398180463	-0.910510707	0.362553238	0.590034623	GUCA1C	protein_coding
ENSG00000138483.3	1.18387889	0.638171624	0.254524089	2.507313259	0.012165284	0.069161983	CCDC54	protein_coding
ENSG00000138495.7	1820.709535	-0.078212567	0.073951292	-1.057622725	0.290227478	0.520034654	COX17	protein_coding
ENSG00000138496.16	6230.247936	0.06581254	0.069907518	0.941422923	0.346488171	0.575898811	PARP9	protein_coding
ENSG00000138587.6	302.1426321	-0.250850194	0.113472954	-2.210660652	0.027059345	0.119131652	MNS1	protein_coding
ENSG00000138592.14	3276.847814	-0.033374886	0.036576399	-0.91247053	0.361521076	0.589309241	USP8	protein_coding
ENSG00000138593.9	3154.224266	-0.000291355	0.057380675	-0.005077584	0.995948691	0.99811956	SECISBP2L	protein_coding
ENSG00000138594.14	5826.110908	0.088026023	0.047400232	1.857079992	0.063299774	0.206591841	TMOD3	protein_coding
ENSG00000138600.10	5108.845947	0.060564734	0.046635084	1.298694654	0.194048739	0.410687805	SPPL2A	protein_coding

ENSG00000138604.10	2564.233049	-0.060335487	0.077880388	-0.77471991	0.438505183	0.655284125	GLCE	protein_coding
ENSG00000138606.19	290.3744956	0.206598689	0.132026211	1.564830857	0.117622574	0.303509526	SHF	protein_coding
ENSG00000138613.14	673.2117804	0.10517772	0.080545632	1.305815322	0.191615368	0.407735871	APH1B	protein_coding
ENSG00000138614.15	2528.430391	-0.019530405	0.044477147	-0.439111011	0.660581101	0.818067029	INTS14	protein_coding
ENSG00000138615.6	1058.884085	0.325963176	0.219883546	1.482435512	0.138224451	0.334875571	CILP	protein_coding
ENSG00000138617.16	856.1613773	-0.152391677	0.051697274	-2.947770085	0.00320075	0.02697333	PARP16	protein_coding
ENSG00000138621.12	539.0210015	-0.073405833	0.057343764	-1.280101405	0.200509475	0.419008932	PPCDC	protein_coding
ENSG00000138622.4	66.21414634	-0.400265767	0.236272394	-1.694086053	0.090248932	0.258650294	HCN4	protein_coding
ENSG00000138623.10	1110.937712	0.413923168	0.12289678	3.368055443	0.000757004	0.009308361	SEMA7A	protein_coding
ENSG00000138629.16	2755.145069	-0.071961534	0.052640362	-1.367041024	0.171612447	0.382139764	UBL7	protein_coding
ENSG00000138639.18	704.6435851	-0.184961754	0.104026685	-1.778022178	0.075400213	0.231014207	ARHGAP24	protein_coding
ENSG00000138640.15	1500.608621	0.152426583	0.097113161	1.569576983	0.116513557	0.301700116	FAM13A	protein_coding
ENSG00000138641.18	22.47640643	-0.002056213	0.081341371	-0.025278813	0.979832573	0.991136584	HERC3	protein_coding
ENSG00000138642.15	1964.361236	0.132826412	0.104482495	1.271279099	0.203629375	0.422993483	HERC6	protein_coding
ENSG00000138646.9	678.9505492	0.091428663	0.109368044	0.83597237	0.403170447	0.624962954	HERC5	protein_coding
ENSG00000138650.9	113.2613574	-0.060654369	0.245114801	-0.247452901	0.804557738	0.902919531	PCDH10	protein_coding
ENSG00000138653.10	13.3903386	-0.60220611	0.232025343	-2.595432474	0.009447196	0.058007676	NDST4	protein_coding
ENSG00000138658.16	445.0801985	-0.097864867	0.077634275	-1.260588415	0.207457175	0.42737963	ZGRF1	protein_coding
ENSG00000138660.12	1836.535863	-0.007720121	0.05348906	-0.144330851	0.885239218	0.945074952	AP1AR	protein_coding
ENSG00000138663.9	1857.998776	-0.017347978	0.043500395	-0.398800474	0.690040227	0.836404672	COPS4	protein_coding
ENSG00000138668.19	13576.76695	-0.08977587	0.047359492	-1.895625708	0.058009555	0.195687609	HNRNPD	protein_coding
ENSG00000138669.9	111.9010442	0.148735794	0.152238822	0.976989917	0.328574144	0.558043319	PRKG2	protein_coding
ENSG00000138670.18	1247.20077	-0.299123049	0.076678049	-3.901025835	9.57859E-05	0.001912077	RASGEF1B	protein_coding
ENSG00000138674.17	14164.19566	0.093025795	0.050159255	1.854608801	0.06365211	0.20736902	SEC31A	protein_coding
ENSG00000138675.16	30.8315336	0.316720787	0.222150786	1.425701849	0.153954423	0.357711111	FGF5	protein_coding
ENSG00000138678.11	534.5947011	0.143740752	0.127416684	1.128115621	0.259271093	0.486448653	GPAT3	protein_coding
ENSG00000138684.9	22.55784672	-0.953484997	0.186582998	-5.110245881	3.2174E-07	2.0771E-05	IL21	protein_coding
ENSG00000138685.17	462.6542662	0.039290953	0.156424769	0.251181147	0.80167406	0.901370097	FGF2	protein_coding
ENSG00000138686.10	736.7339977	0.018769002	0.045608512	0.411524103	0.680688271	0.830924452	BBS7	protein_coding
ENSG00000138688.15	4593.063588	-0.022430113	0.062101155	-0.361186725	0.717959865	0.852565403	KIAA1109	protein_coding
ENSG00000138696.11	262.8971369	0.509806536	0.185395399	2.749833815	0.00596255	0.042147307	BMPR1B	protein_coding
ENSG00000138698.15	1781.161281	0.051014366	0.050555026	1.009085938	0.312933422	0.542408803	RAP1GDS1	protein_coding
ENSG00000138709.19	1990.017999	-0.033236924	0.055036566	-0.603906213	0.545905998	0.740051195	LARP1B	protein_coding
ENSG00000138722.10	511.0826654	-0.037664697	0.170249935	-0.221231786	0.824911961	0.913501271	MMRN1	protein_coding
ENSG00000138735.16	2354.76879	-0.010041978	0.110077613	-0.091226343	0.927312743	0.965674498	PDE5A	protein_coding
ENSG00000138738.11	228.6111329	0.332947968	0.130448932	2.552324219	0.010700689	0.063302671	PRDM5	protein_coding
ENSG00000138741.11	57.66858808	0.129480331	0.116160592	1.114666592	0.264993322	0.492868773	TRPC3	protein_coding
ENSG00000138744.16	1246.65991	0.11832993	0.075608957	1.565025296	0.117576978	0.303461974	NAAA	protein_coding
ENSG00000138750.15	2253.910211	0.027575365	0.048537188	0.568128611	0.569947642	0.756598932	NUP54	protein_coding
ENSG00000138755.6	7426.278994	-0.729313374	0.18649096	-3.910717031	9.20225E-05	0.001855876	CXCL9	protein_coding
ENSG00000138756.18	919.8028146	0.038585372	0.065058587	0.593086529	0.553123245	0.744623763	BMP2K	protein_coding
ENSG00000138757.15	9080.941819	0.150930442	0.050269218	3.002442591	0.002678225	0.023625304	G3BP2	protein_coding

ENSG00000138758.12	8690.505179	0.230200636	0.060819395	3.78498727	0.000153716	0.002780615	SEPTIN11	protein_coding
ENSG00000138759.19	2183.594865	0.00708772	0.168335416	0.042104744	0.966415198	0.984499865	FRAS1	protein_coding
ENSG00000138760.10	14260.98527	0.055257122	0.048827482	1.13168075	0.257768678	0.484817913	SCARB2	protein_coding
ENSG00000138764.15	2498.854638	0.264871225	0.063298587	4.184472926	2.85829E-05	0.000735274	CCNG2	protein_coding
ENSG00000138767.13	3547.232957	-0.083592155	0.055452347	-1.507459286	0.131692972	0.325198881	CNOT6L	protein_coding
ENSG00000138768.15	6274.492352	0.110507813	0.05199588	2.125318653	0.033560033	0.137043757	USO1	protein_coding
ENSG00000138769.11	100.9124103	-0.160982875	0.159155105	-1.011484205	0.311784737	0.541278972	CDKL2	protein_coding
ENSG00000138771.16	11277.80362	0.094230465	0.097575628	0.965717222	0.334185699	0.563657324	SHROOM3	protein_coding
ENSG00000138772.13	4786.667483	0.122897606	0.09831173	1.250080789	0.211270037	0.431712096	ANXA3	protein_coding
ENSG00000138777.20	3114.901913	-0.0926206	0.059789913	-1.54910077	0.121357498	0.309679434	PPA2	protein_coding
ENSG00000138778.13	1940.52641	-0.05641402	0.097716005	-0.577326301	0.56371905	0.752540668	CENPE	protein_coding
ENSG00000138780.15	936.1323792	-0.015679347	0.063896263	-0.24538754	0.806156376	0.903629249	GSTCD	protein_coding
ENSG00000138785.15	721.3087206	-0.092515608	0.044396779	-2.083836061	0.037175082	0.146657482	INTS12	protein_coding
ENSG00000138792.10	641.9172089	-0.162109492	0.116328059	-1.39355451	0.163452173	0.370639969	ENPEP	protein_coding
ENSG00000138794.10	1418.666425	-0.179626875	0.07388891	-2.431039723	0.015055564	0.080312294	CASP6	protein_coding
ENSG00000138795.10	1132.637358	0.156171461	0.104605396	1.492957982	0.135448186	0.33096995	LEF1	protein_coding
ENSG00000138796.17	3763.26872	-0.215661036	0.06695159	-3.221148834	0.001276778	0.013824515	HADH	protein_coding
ENSG00000138798.13	52.14562705	0.470079658	0.244994748	1.918733612	0.055018055	0.189176492	EGF	protein_coding
ENSG00000138801.9	3185.385322	-0.052389267	0.071344595	-0.734313042	0.462757971	0.675120272	PAPSS1	protein_coding
ENSG00000138802.11	3594.758573	0.08732209	0.043286159	2.017321271	0.043661997	0.162946656	SEC24B	protein_coding
ENSG00000138813.10	2.575323871	-0.224110401	0.36934436	-0.606778997	0.543997588	0.738485875	C4orf17	protein_coding
ENSG00000138814.17	4500.133965	0.355801581	0.064257128	5.537153498	3.07427E-08	3.05862E-06	PPP3CA	protein_coding
ENSG00000138821.13	1943.450367	0.280924854	0.112137877	2.505173648	0.012239129	0.069427821	SLC39A8	protein_coding
ENSG00000138823.13	606.8860229	-0.827738472	0.271358957	-3.050345121	0.002285785	0.021044931	MTTP	protein_coding
ENSG00000138829.12	454.8261256	0.559723227	0.131147103	4.267903871	1.97318E-05	0.000551188	FBN2	protein_coding
ENSG00000138834.14	3132.803198	0.000693871	0.073537441	0.00943562	0.992471576	0.996518853	MAPK8IP3	protein_coding
ENSG00000138835.22	3414.709977	0.074488021	0.056297213	1.323120937	0.185795163	0.400534076	RGS3	protein_coding
ENSG00000138867.17	8013.243791	-0.140325217	0.055105562	-2.546480081	0.010881542	0.064070391	GUCD1	protein_coding
ENSG00000138892.11	3.194471584	0.143107476	0.242916841	0.589121261	0.555779932	0.746663956	TTLL8	protein_coding
ENSG00000138942.16	2153.342454	-0.068705952	0.0383165	-1.793116574	0.072954286	0.225797577	RNF185	protein_coding
ENSG00000138944.8	595.0802824	-0.335499942	0.198440593	-1.69068202	0.09089755	0.259774563	SHISAL1	protein_coding
ENSG00000138964.17	559.6070559	-0.344993689	0.10479947	-3.291941162	0.000994984	0.011446259	PARVG	protein_coding
ENSG00000139044.12	4060.010193	0.048190345	0.117189516	0.411217204	0.680913274	0.83095636	B4GALNT3	protein_coding
ENSG00000139053.3	1.426726842	-0.495459621	0.314000478	-1.577894481	0.114589853	0.298706827	PDE6H	protein_coding
ENSG00000139055.7	156.7335568	-0.387248383	0.156754576	-2.470411983	0.013495753	0.074519058	ERP27	protein_coding
ENSG00000139083.11	5154.561987	-0.028601037	0.04763593	-0.60040892	0.548233745	0.741384741	ETV6	protein_coding
ENSG00000139112.11	2064.794425	0.259195933	0.091544839	2.831354943	0.004635125	0.035268876	GABARAPL1	protein_coding
ENSG00000139116.19	2506.623149	0.016746531	0.071151482	0.235364468	0.813925862	0.908086436	KIF21A	protein_coding
ENSG00000139117.14	520.7878855	0.427697895	0.103855458	4.11820335	3.81838E-05	0.000928355	CPNE8	protein_coding
ENSG00000139131.13	929.5129174	0.016413219	0.063741975	0.257494678	0.796796923	0.898347249	YARS2	protein_coding
ENSG00000139132.14	3164.352805	0.150472314	0.080024422	1.880329903	0.06006313	0.199982505	FGD4	protein_coding
ENSG00000139133.7	285.8917181	0.00093127	0.066058985	0.014097548	0.988752156	0.994824703	ALG10	protein_coding

ENSG00000139144.11	978.7970552	-0.528995395	0.24785824	-2.134265922	0.032821019	0.135157962	PIK3C2G	protein_coding
ENSG00000139146.14	4474.188598	0.204785121	0.081129633	2.524171663	0.011597128	0.067024541	SINHCAF	protein_coding
ENSG00000139151.16	17.83098287	-0.355745051	0.250156079	-1.422092371	0.154999437	0.359315378	PLCZ1	protein_coding
ENSG00000139154.16	2570.522414	0.057491806	0.056780628	1.012525016	0.311287092	0.540810008	AEBP2	protein_coding
ENSG00000139155.9	32.98453704	0.151941643	0.124284445	1.222531455	0.221506716	0.443940495	SLCO1C1	protein_coding
ENSG00000139160.13	208.6512493	-0.079679003	0.068980159	-1.155100314	0.24804935	0.474584911	ETFBKMT	protein_coding
ENSG00000139163.16	7052.205339	-0.012087378	0.089450208	-0.13512968	0.892509344	0.948722519	ETNK1	protein_coding
ENSG00000139168.9	3783.749865	-0.152482438	0.060992669	-2.500012533	0.012418891	0.070209915	ZCRB1	protein_coding
ENSG00000139173.10	715.5060411	-0.00457226	0.072447376	-0.063111458	0.94967775	0.976395197	TMEM117	protein_coding
ENSG00000139174.12	269.866576	0.403585389	0.126927833	3.17964452	0.001474558	0.015346745	PRICKLE1	protein_coding
ENSG00000139178.11	2492.798906	0.018335158	0.064068198	0.286181889	0.774738814	0.886001165	C1RL	protein_coding
ENSG00000139180.11	853.6759893	-0.167903767	0.067220225	-2.497816185	0.012496097	0.070554862	NDUFA9	protein_coding
ENSG00000139182.15	3250.34028	0.023394451	0.085356545	0.274079169	0.784023782	0.890419711	CLSTN3	protein_coding
ENSG00000139187.10	100.127307	-0.304400802	0.109273696	-2.785673157	0.00534167	0.038931922	KLRG1	protein_coding
ENSG00000139190.17	369.967687	-0.363739303	0.089050144	-4.084657091	4.4142E-05	0.001043719	VAMP1	protein_coding
ENSG00000139192.12	2421.953074	-0.169391487	0.080889561	-2.094108127	0.036250349	0.144191371	TAPBPL	protein_coding
ENSG00000139193.4	294.782942	-0.635876512	0.147350968	-4.315387396	1.59323E-05	0.000466588	CD27	protein_coding
ENSG00000139194.8	227.2421679	-0.311679349	0.111947418	-2.784158445	0.005366681	0.039064489	RBP5	protein_coding
ENSG00000139197.11	2177.340899	-0.131686622	0.05731442	-2.297617637	0.021583561	0.10227782	PEX5	protein_coding
ENSG00000139200.14	81.00799195	0.415011562	0.155473181	2.669345012	0.007599934	0.049884368	PIANP	protein_coding
ENSG00000139209.16	127.8656196	-0.624169399	0.205805823	-3.032807285	0.002422903	0.021908022	SLC38A4	protein_coding
ENSG00000139211.7	1291.766964	0.896565559	0.130863484	6.851151527	7.32579E-12	3.12976E-09	AMIGO2	protein_coding
ENSG00000139218.18	7947.996853	-0.032720543	0.042242871	-0.774581423	0.438587038	0.655312165	SCAF11	protein_coding
ENSG00000139219.19	147.6762534	-0.888685076	0.305157684	-2.91221595	0.003588744	0.029368397	COL2A1	protein_coding
ENSG00000139220.17	53.57234257	0.307003904	0.141849313	2.164296019	0.030441638	0.128778112	PPFIA2	protein_coding
ENSG00000139223.4	2.129556883	0.006557008	0.137062579	0.047839522	0.961844139	0.982355347	ANP32D	protein_coding
ENSG00000139233.7	1101.881053	0.05934527	0.065343981	0.908197965	0.363773639	0.590991458	LLPH	protein_coding
ENSG00000139239.7	740.8529607	-0.052220663	0.080673774	-0.647306566	0.517433553	0.71870509	RPL14P1	processed_pseudogene
ENSG00000139263.12	3161.808814	-0.224908597	0.080586015	-2.79091351	0.005255952	0.038500353	LRIG3	protein_coding
ENSG00000139266.6	1514.411849	-0.010332174	0.075652472	-0.136574176	0.891367389	0.948248828	MARCHF9	protein_coding
ENSG00000139269.3	11.32545803	0.231711645	0.164449217	1.40901641	0.158830316	0.364396582	INHBE	protein_coding
ENSG00000139278.10	1594.994029	0.061531662	0.121361805	0.507010105	0.612147728	0.7871113	GLIPR1	protein_coding
ENSG00000139287.13	6.828694677	-0.319111906	0.269140646	-1.185669691	0.235752768	0.460669853	TPH2	protein_coding
ENSG00000139289.13	5195.218698	0.184738365	0.11206751	1.648456044	0.099259124	0.274420431	PHLDA1	protein_coding
ENSG00000139291.14	1726.395336	-0.277469375	0.056061991	-4.949331463	7.44688E-07	4.11818E-05	TMEM19	protein_coding
ENSG00000139292.13	1805.191698	-0.345063581	0.203417317	-1.696333363	0.089822766	0.257920839	LGR5	protein_coding
ENSG00000139304.16	15.27018409	0.155958948	0.212722389	0.733157186	0.463462565	0.67575562	PTPRQ	protein_coding
ENSG00000139318.8	3177.8908	0.31018341	0.086995238	3.565521728	0.000363133	0.00537326	DUSP6	protein_coding
ENSG00000139323.14	2295.025525	-0.080220123	0.061480436	-1.304807312	0.191958469	0.408216038	POC1B	protein_coding
ENSG00000139324.12	2149.380521	0.168602119	0.049148125	3.430489331	0.000602494	0.007827927	TMTC3	protein_coding
ENSG00000139329.5	30549.15517	0.554844039	0.120516899	4.603869193	4.14713E-06	0.000160782	LUM	protein_coding
ENSG00000139330.6	29.14563918	0.216543182	0.231191371	0.936640418	0.348943554	0.578105663	KERA	protein_coding

ENSG00000139343.10	4025.426951	-0.322117884	0.082408003	-3.908818	9.27488E-05	0.001867705	SNRPF	protein_coding
ENSG00000139344.8	64.18717401	-0.000621065	0.162479123	-0.003822429	0.99695015	0.998504884	AMDHD1	protein_coding
ENSG00000139350.12	1597.188492	-0.145350456	0.056575661	-2.569134013	0.010195302	0.061229421	NEDD1	protein_coding
ENSG00000139351.15	80.79262887	-0.594019201	0.114096509	-5.206287237	1.92656E-07	1.38767E-05	SYCP3	protein_coding
ENSG00000139352.4	29.0997502	0.177921313	0.239326946	0.743423654	0.457225228	0.670998953	ASCL1	protein_coding
ENSG00000139354.11	980.903936	0.111834905	0.090075886	1.241563195	0.214397768	0.435363076	GAS2L3	protein_coding
ENSG00000139364.10	125.026446	0.588721848	0.181299246	3.247238254	0.001165308	0.01290486	TMEM132B	protein_coding
ENSG00000139370.12	1579.077715	0.056137307	0.045947691	1.221765561	0.221796295	0.444296091	SLC15A4	protein_coding
ENSG00000139372.15	1848.744189	0.087814267	0.054812237	1.602092367	0.109135186	0.289807319	TDG	protein_coding
ENSG00000139405.16	1366.945017	-0.075496334	0.061929202	-1.219074872	0.222815776	0.445376042	RITA1	protein_coding
ENSG00000139410.15	651.1604734	-0.049862807	0.078107786	-0.63838459	0.52322336	0.722609715	SDSL	protein_coding
ENSG00000139428.12	1022.50698	-0.112957339	0.060712687	-1.860522809	0.062811595	0.205660895	MMAB	protein_coding
ENSG00000139433.11	2701.907768	0.029684277	0.073295207	0.404996155	0.685480337	0.833924529	GLTP	protein_coding
ENSG00000139436.21	1359.288952	-0.098029582	0.055567613	-1.764149606	0.077706795	0.235580144	GIT2	protein_coding
ENSG00000139437.18	1197.114171	-0.087873857	0.042552598	-2.065064431	0.038916904	0.151326298	TCHP	protein_coding
ENSG00000139438.6	873.8865027	-0.030739053	0.137510338	-0.223539944	0.823115299	0.912810672	FAM222A	protein_coding
ENSG00000139445.18	10.10355217	0.341950129	0.274348134	1.246409531	0.212614084	0.43323127	FOXN4	protein_coding
ENSG00000139496.16	3882.406098	0.110801706	0.061092734	1.813664238	0.069729434	0.219205733	NUP58	protein_coding
ENSG00000139505.11	2590.327488	0.086373458	0.054121719	1.595911198	0.110508621	0.292175354	MTMR6	protein_coding
ENSG00000139508.15	1812.245436	0.130729114	0.103439146	1.263826305	0.206292377	0.42608792	SLC46A3	protein_coding
ENSG00000139514.13	10044.29075	0.166015031	0.080635282	2.058838603	0.039509702	0.152865655	SLC7A1	protein_coding
ENSG00000139515.6	1170.071464	-0.136960998	0.138913851	-0.985941982	0.324161584	0.553973424	PDX1	protein_coding
ENSG00000139517.9	2671.567548	0.079452838	0.065455465	1.21384574	0.224806636	0.447697404	LNX2	protein_coding
ENSG00000139531.13	1197.350855	-0.085712493	0.061943588	-1.383718584	0.166444641	0.374859859	SUOX	protein_coding
ENSG00000139537.11	116.8929533	-0.001462652	0.073026252	-0.020029121	0.984020142	0.992793967	CCDC65	protein_coding
ENSG00000139540.12	1883.74855	-0.647069435	0.195877926	-3.303432134	0.000955091	0.011069147	SLC39A5	protein_coding
ENSG00000139546.11	1126.324542	-0.090916348	0.058243101	-1.560980557	0.118528339	0.304976316	TARBP2	protein_coding
ENSG00000139547.7	62.10516183	-0.237319564	0.16286644	-1.457142211	0.145077128	0.345213483	RDH16	protein_coding
ENSG00000139549.4	58.18693937	0.016871631	0.124221432	0.135819	0.891964371	0.948494893	DHH	protein_coding
ENSG00000139567.13	1726.763996	0.137749635	0.084913514	1.622234533	0.10475314	0.283293123	ACVRL1	protein_coding
ENSG00000139572.4	109.5555944	0.192659338	0.142825541	1.348913765	0.177364666	0.389665746	GPR84	protein_coding
ENSG00000139574.8	0						NPFF	protein_coding
ENSG00000139579.13	2859.405935	-0.231471376	0.060218379	-3.843865968	0.000121111	0.002293638	NABP2	protein_coding
ENSG00000139597.18	719.2601814	-0.037660395	0.085473537	-0.440608827	0.659496211	0.817466902	N4BP2L1	protein_coding
ENSG00000139610.2	4.058710112	-0.405792296	0.24570177	-1.651564398	0.098623383	0.273200124	CELA1	protein_coding
ENSG00000139613.12	5993.361467	0.000391663	0.048488329	0.008077469	0.993555182	0.997055871	SMARCC2	protein_coding
ENSG00000139618.16	1436.840332	-0.11056244	0.092344425	-1.197283324	0.231196142	0.455171518	BRCA2	protein_coding
ENSG00000139620.13	1266.117157	0.026252381	0.042333206	0.620136846	0.535167696	0.731983763	KANSL2	protein_coding
ENSG00000139624.14	1699.972867	0.037811661	0.03588932	1.053563048	0.292083014	0.521886101	CERS5	protein_coding
ENSG00000139625.13	641.744083	0.138570854	0.074932899	1.849265875	0.064419431	0.208822847	MAP3K12	protein_coding
ENSG00000139626.16	341.5682607	-0.309408798	0.125196196	-2.471391365	0.013458844	0.074352945	ITGB7	protein_coding
ENSG00000139629.16	4132.124207	-0.048476551	0.114697489	-0.422647017	0.672552818	0.825875226	GALNT6	protein_coding

ENSG00000139631.19	603.3746535	-0.057884364	0.087542184	-0.661216809	0.508473283	0.712033606	CSAD	protein_coding
ENSG00000139636.15	1161.744573	0.043537433	0.04518162	0.963609373	0.335241806	0.564723498	LMBR1L	protein_coding
ENSG00000139637.14	7.893671188	-0.25169386	0.117081785	-2.149726871	0.031576826	0.131942021	MYG1	protein_coding
ENSG00000139641.13	6797.883991	0.087140973	0.054115638	1.610273403	0.107338183	0.287165005	ESYT1	protein_coding
ENSG00000139644.13	70121.75116	-0.086811802	0.049271932	-1.76189158	0.078087617	0.236471446	TMBIM6	protein_coding
ENSG00000139645.10	6221.97537	0.119917505	0.06082902	1.971386425	0.048679693	0.174945092	ANKRD52	protein_coding
ENSG00000139648.7	1.730092402	-0.029866423	0.284850481	-0.104849474	0.916495252	0.960394325	KRT71	protein_coding
ENSG00000139651.11	1405.583679	-0.052284919	0.048311583	-1.082243954	0.279144142	0.507898522	ZNF740	protein_coding
ENSG00000139656.7	1.905220733	-0.177233766	0.239031848	-0.741465071	0.458411503	0.671807569	SMIM2	protein_coding
ENSG00000139668.9	877.6387548	-0.005713378	0.063499486	-0.089975192	0.928306929	0.966035235	WDFY2	protein_coding
ENSG00000139675.12	163.7425583	-0.021429656	0.059347869	-0.361085522	0.718035516	0.852607256	HNRNPA1L2	protein_coding
ENSG00000139679.15	1164.950717	-0.102013397	0.090384212	-1.128663903	0.259039642	0.486202803	LPAR6	protein_coding
ENSG00000139684.15	5071.589581	-0.099525013	0.069170226	-1.438841804	0.150195349	0.352405906	ESD	protein_coding
ENSG00000139687.16	6232.188908	-0.039914319	0.063576924	-0.627811426	0.53012748	0.728097949	RB1	protein_coding
ENSG00000139697.14	4135.569391	0.023345931	0.050978407	0.457957247	0.646983157	0.809560375	SBNO1	protein_coding
ENSG00000139714.12	113.3920229	0.088999541	0.153366566	0.580306014	0.561708273	0.750845442	MORN3	protein_coding
ENSG00000139718.11	4391.652971	0.057497879	0.049113478	1.17071486	0.24171341	0.467516435	SETD1B	protein_coding
ENSG00000139719.11	928.8161173	-0.154006333	0.044740813	-3.442188998	0.000577027	0.007582814	VPS33A	protein_coding
ENSG00000139722.7	3517.900639	0.114997954	0.071569774	1.606794988	0.108099348	0.288200996	VPS37B	protein_coding
ENSG00000139725.8	252.9292115	0.349268809	0.112543932	3.103399739	0.00191311	0.018514135	RHOF	protein_coding
ENSG00000139726.11	4276.823893	-0.048779465	0.046549341	-1.047908818	0.294680619	0.524734282	DENR	protein_coding
ENSG00000139734.19	973.3066675	0.066583347	0.089411704	0.744682677	0.456463574	0.670395779	DIAPH3	protein_coding
ENSG00000139737.22	516.9229677	0.038434579	0.137327334	0.279875667	0.779572897	0.888490862	SLAIN1	protein_coding
ENSG00000139746.16	5787.282	-0.110075833	0.061341815	-1.794466532	0.072738736	0.225427487	RBM26	protein_coding
ENSG00000139767.10	12.31685924	-0.082939208	0.208852063	-0.397119408	0.691279409	0.837246536	SRRM4	protein_coding
ENSG00000139780.7	1.144109982	0.376930189	0.253702017	1.485720111	0.137353169	0.333757006	METTL21C	protein_coding
ENSG00000139793.18	7068.252065	0.032101896	0.073000524	0.439748844	0.660119022	0.817796919	MBNL2	protein_coding
ENSG00000139797.8	9.049579332	0.219997779	0.156318534	1.407368492	0.159318149	0.365180334	RNF113B	protein_coding
ENSG00000139800.8	258.2120178	0.482820762	0.258411846	1.868415747	0.061704144	0.203410354	ZIC5	protein_coding
ENSG00000139826.6	1680.672736	0.10579408	0.04980585	2.124129597	0.033659308	0.137299605	ABHD13	protein_coding
ENSG00000139832.5	1926.425936	-0.101242395	0.07215447	-1.40313406	0.160576866	0.366723867	RAB20	protein_coding
ENSG00000139835.14	1269.027189	0.057290719	0.085333398	0.671375101	0.501981603	0.707051083	GRTP1	protein_coding
ENSG00000139842.15	6197.660343	0.132828215	0.052759199	2.51763137	0.01181469	0.067827781	CUL4A	protein_coding
ENSG00000139865.16	120.1590633	-0.119315616	0.14072305	-0.847875431	0.396507349	0.61976483	TTC6	protein_coding
ENSG00000139874.6	1255.302886	-0.114717613	0.178806212	-0.641575098	0.521149106	0.721061727	SSTR1	protein_coding
ENSG00000139880.19	785.9024162	0.124233848	0.077079757	1.611757125	0.107014801	0.286624708	CDH24	protein_coding
ENSG00000139890.10	49.21151047	0.211186346	0.097726883	2.160985197	0.030696483	0.129531245	REM2	protein_coding
ENSG00000139899.11	149.4741433	-0.14248044	0.081790731	-1.742012047	0.081506336	0.242924671	CBLN3	protein_coding
ENSG00000139908.15	104.5767344	-0.050441009	0.079133444	-0.637417085	0.523853203	0.723112896	TSSK4	protein_coding
ENSG00000139910.20	297.0019781	-0.188468905	0.17590388	-1.071431202	0.283975589	0.512942582	NOVA1	protein_coding
ENSG00000139914.6	23.8460828	-0.471815922	0.109765641	-4.298393552	1.7204E-05	0.000493494	FITM1	protein_coding
ENSG00000139915.21	14.46858837	0.434632026	0.274017903	1.586144633	0.112706504	0.295921632	MDGA2	protein_coding

ENSG00000139921.13	3655.68975	0.081821629	0.052892184	1.546951226	0.121875007	0.310418329	TMX1	protein_coding
ENSG00000139926.16	1635.487913	0.409945749	0.119970985	3.417040792	0.000633058	0.00811853	FRMD6	protein_coding
ENSG00000139946.10	1534.20217	-0.007435972	0.111178654	-0.066883089	0.946674776	0.974995071	PELI2	protein_coding
ENSG00000139970.17	336.8286855	-0.173088127	0.139076106	-1.244556894	0.213294674	0.433964771	RTN1	protein_coding
ENSG00000139971.15	250.7105985	0.445133757	0.100728688	4.419135845	9.90963E-06	0.000322986	ARMH4	protein_coding
ENSG00000139973.16	46.49202606	0.592615452	0.21402526	2.768904251	0.005624516	0.04045569	SYT16	protein_coding
ENSG00000139974.16	438.0909452	-0.251143624	0.064127951	-3.916289558	8.99222E-05	0.001825975	SLC38A6	protein_coding
ENSG00000139977.14	1754.397846	0.035934699	0.044412977	0.809103583	0.418455567	0.638570011	NAA30	protein_coding
ENSG00000139985.7	11.52390845	0.556367844	0.168392001	3.304004	0.000953145	0.011052471	ADAM21	protein_coding
ENSG00000139988.10	86.76845653	-0.549922516	0.187538542	-2.932317332	0.003364428	0.027968651	RDH12	protein_coding
ENSG00000139990.18	3724.701717	0.083904087	0.039855162	2.105225125	0.035271716	0.141505761	DCAF5	protein_coding
ENSG00000139998.16	2748.538573	-0.10822842	0.107753357	-1.004408797	0.315181611	0.544378481	RAB15	protein_coding
ENSG00000140006.11	613.8422558	-0.121643672	0.045399105	-2.679428835	0.007374788	0.048837782	WDR89	protein_coding
ENSG00000140009.18	125.12956	-0.404178584	0.105326555	-3.837385396	0.000124351	0.00233672	ESR2	protein_coding
ENSG00000140015.20	22.61943634	0.113671491	0.281745047	0.403455152	0.686613421	0.834570051	KCNH5	protein_coding
ENSG00000140022.13	643.9027815	0.207560482	0.10413395	1.993206641	0.046238824	0.169167093	STON2	protein_coding
ENSG00000140025.16	406.5854697	-0.074201635	0.050940642	-1.456629364	0.145218718	0.345387843	EFCAB11	protein_coding
ENSG00000140030.6	242.3903171	-0.368906833	0.110626193	-3.334715078	0.000853868	0.010163623	GPR65	protein_coding
ENSG00000140043.11	100.5185243	-0.123153804	0.070641083	-1.743373679	0.081268363	0.242513967	PTGR2	protein_coding
ENSG00000140044.13	1302.733892	0.03198777	0.07442921	0.429774418	0.667359744	0.822410826	JDP2	protein_coding
ENSG00000140057.9	277.4843021	0.03104928	0.116248994	0.26709289	0.789397641	0.894190524	AK7	protein_coding
ENSG00000140067.6	1.505480938	0.245722628	0.308492814	0.796526263	0.425726217	0.644513563	FAM181A	protein_coding
ENSG00000140090.18	49.18197103	-0.207868417	0.11832383	-1.756775604	0.078956065	0.23822011	SLC24A4	protein_coding
ENSG00000140092.14	3178.286296	0.149966354	0.12580937	1.192012595	0.233256307	0.457695065	FBLN5	protein_coding
ENSG00000140093.10	79.27681123	-1.351473037	0.281082734	-4.808096952	1.52374E-06	7.241E-05	SERPINA10	protein_coding
ENSG00000140104.14	538.7568948	0.185617662	0.059182069	3.136383451	0.001710454	0.017107289	CLBA1	protein_coding
ENSG00000140105.18	19433.60994	-0.316021342	0.141806661	-2.228536662	0.025844749	0.115511451	WARS1	protein_coding
ENSG00000140107.12	9.434168252	-0.135219416	0.168119851	-0.804303686	0.421221609	0.640839789	SLC25A47	protein_coding
ENSG00000140153.18	1169.914794	-0.024070989	0.034244596	-0.702913502	0.482109657	0.690754779	WDR20	protein_coding
ENSG00000140157.15	2767.873135	0.091236104	0.054358946	1.678400904	0.093268861	0.263937734	NIPA2	protein_coding
ENSG00000140199.13	2428.433187	0.116732944	0.060316625	1.935336137	0.052949061	0.184397124	SLC12A6	protein_coding
ENSG00000140254.12	255.3279574	1.215968999	0.207595816	5.857386827	4.70207E-09	6.60975E-07	DUOXA1	protein_coding
ENSG00000140259.7	1739.345787	0.010644484	0.038040129	0.279822504	0.779613685	0.888514111	MFAP1	protein_coding
ENSG00000140262.18	7313.128195	0.026079457	0.066982393	0.389347948	0.697018772	0.840406923	TCF12	protein_coding
ENSG00000140263.15	2558.277251	-0.244619221	0.097346532	-2.512870428	0.011975331	0.068428969	SORD	protein_coding
ENSG00000140264.21	10610.10843	-0.101990941	0.073512627	-1.387393501	0.165321806	0.3732989	SERF2	protein_coding
ENSG00000140265.12	1467.054192	0.007072118	0.044308813	0.159609739	0.873188506	0.938718035	ZSCAN29	protein_coding
ENSG00000140274.14	1610.662163	0.105014738	0.261569025	0.401480022	0.688066744	0.835275497	DUOXA2	protein_coding
ENSG00000140279.13	11938.54581	0.105314907	0.243835303	0.431910003	0.665806824	0.821294163	DUOX2	protein_coding
ENSG00000140280.14	976.9418182	-0.170665303	0.066398845	-2.57030531	0.010160892	0.061132293	LYSMD2	protein_coding
ENSG00000140284.11	907.4967998	-0.114701587	0.123984205	-0.925130645	0.354897954	0.582961595	SLC27A2	protein_coding
ENSG00000140285.12	1397.302619	0.179872431	0.160019631	1.124064775	0.260985548	0.488174683	FGF7	protein_coding

ENSG00000140287.11	111.5423043	-0.340432419	0.153073657	-2.223977835	0.026149936	0.116362273	HDC	protein_coding
ENSG00000140297.13	8132.269534	0.457347681	0.166831579	2.741373565	0.00611829	0.042885111	GCNT3	protein_coding
ENSG00000140299.13	3522.768897	0.103694709	0.058430088	1.774680021	0.075950736	0.232097141	BNIP2	protein_coding
ENSG00000140307.11	3079.09617	0.070729649	0.072578593	0.974524936	0.329795964	0.559333647	GTF2A2	protein_coding
ENSG00000140319.11	12620.24768	-0.081959037	0.047236705	-1.735070992	0.082728232	0.245241371	SRP14	protein_coding
ENSG00000140320.12	2253.678116	0.054051567	0.055292116	0.977563722	0.328290147	0.557791145	BAHD1	protein_coding
ENSG00000140323.6	500.9013025	-0.032368723	0.143204428	-0.226031579	0.821176859	0.912088605	DISP2	protein_coding
ENSG00000140326.13	842.0717156	-0.087930756	0.052995375	-1.659215676	0.09707234	0.270468117	CDAN1	protein_coding
ENSG00000140332.17	5457.64989	0.009139349	0.059516146	0.153560828	0.877956025	0.941196332	TLE3	protein_coding
ENSG00000140350.15	5571.968967	-0.126119478	0.047760681	-2.640654927	0.008274595	0.052969258	ANP32A	protein_coding
ENSG00000140365.16	2654.965232	0.008755946	0.055765051	0.157014942	0.875233072	0.939687385	COMMD4	protein_coding
ENSG00000140367.12	2907.522737	0.331937159	0.069385554	4.783952016	1.71882E-06	7.90928E-05	UBE2Q2	protein_coding
ENSG00000140368.13	338.9299004	-0.23340639	0.097865342	-2.384974966	0.017080284	0.087450596	PSTPIP1	protein_coding
ENSG00000140374.16	4252.753791	-0.058721708	0.056763308	-1.034501154	0.300901938	0.530531876	ETFA	protein_coding
ENSG00000140379.9	531.8587842	0.007806773	0.133422369	0.058511725	0.953341023	0.978075145	BCL2A1	protein_coding
ENSG00000140382.15	1336.405517	0.029277012	0.052746241	0.555053997	0.578857728	0.763118536	HMG20A	protein_coding
ENSG00000140386.13	1100.892518	-0.095823427	0.052006377	-1.842532248	0.065397334	0.210723131	SCAPER	protein_coding
ENSG00000140391.15	27120.31422	0.072327057	0.077114887	0.937913033	0.348289105	0.5775046	TSPAN3	protein_coding
ENSG00000140395.9	1742.712588	-0.012056652	0.042997934	-0.280400725	0.779170081	0.888374224	WDR61	protein_coding
ENSG00000140396.13	5044.840387	-0.110275904	0.059912117	-1.840627734	0.065676131	0.211199819	NCOA2	protein_coding
ENSG00000140398.14	502.0769679	0.170619742	0.097818414	1.744249737	0.081115552	0.242240433	NEIL1	protein_coding
ENSG00000140400.17	1952.024789	0.05721483	0.053099524	1.077501751	0.281256169	0.510169001	MAN2C1	protein_coding
ENSG00000140403.12	2672.533913	0.231103014	0.091696089	2.520314845	0.011724991	0.06745972	DNAJA4	protein_coding
ENSG00000140406.4	3800.662083	0.180133489	0.063427788	2.839977465	0.004511672	0.034625772	TLNRD1	protein_coding
ENSG00000140416.23	16594.93965	-0.114812108	0.12213411	-0.940049488	0.347192177	0.576409383	TPM1	protein_coding
ENSG00000140443.15	4551.133858	0.510980092	0.104569451	4.886514044	1.02637E-06	5.2993E-05	IGF1R	protein_coding
ENSG00000140450.9	2028.175325	0.133376089	0.095974904	1.389697554	0.164620738	0.372329781	ARRDC4	protein_coding
ENSG00000140451.13	441.0390343	0.016051577	0.086371385	0.185843692	0.85256734	0.927949821	PIF1	protein_coding
ENSG00000140455.17	1627.220959	-0.01462302	0.045159122	-0.323810994	0.746081133	0.869304212	USP3	protein_coding
ENSG00000140459.18	35.03259529	0.130841364	0.17845375	0.733194813	0.463439619	0.675744772	CYP11A1	protein_coding
ENSG00000140463.14	824.1528234	-0.068378217	0.051994069	-1.315115697	0.188471022	0.403797673	BBS4	protein_coding
ENSG00000140464.20	8830.215399	0.164773731	0.069298829	2.377727474	0.017419695	0.088545206	PML	protein_coding
ENSG00000140465.14	20.71819047	0.302584404	0.286489258	1.056180628	0.290885699	0.520892793	CYP1A1	protein_coding
ENSG00000140470.15	300.4416739	0.298350611	0.145313966	2.053144776	0.040058538	0.15411238	ADAMTS17	protein_coding
ENSG00000140471.17	951.184852	-0.012017913	0.054556087	-0.220285461	0.825648842	0.91392541	LINS1	protein_coding
ENSG00000140474.14	2188.050548	-0.054661848	0.057388024	-0.952495732	0.340845629	0.570831283	ULK3	protein_coding
ENSG00000140478.15	0.551747738	0.177608754	0.410252518	0.432925445	0.665068934		GOLGA6D	protein_coding
ENSG00000140479.18	1516.582872	0.39338944	0.098671627	3.986854715	6.6955E-05	0.001451591	PCSK6	protein_coding
ENSG00000140481.15	36.31178716	0.656052009	0.187279607	3.503061643	0.000459943	0.006403494	CCDC33	protein_coding
ENSG00000140488.16	1.414886822	0.113442203	0.227489189	0.498670745	0.618011358	0.790741703	CELF6	protein_coding
ENSG00000140497.16	10599.37157	0.061487977	0.055955726	1.098868356	0.271825491	0.500035435	SCAMP2	protein_coding
ENSG00000140505.7	5.760560328	0.046063635	0.238394765	0.193224188	0.846783396	0.925031066	CYP1A2	protein_coding

ENSG00000140506.17	1.729018212	-0.630919141	0.30350136	-2.078801691	0.037635581	0.147938457	LMAN1L	protein_coding
ENSG00000140511.12	1168.559964	0.112807827	0.117239942	0.9621962	0.335951057	0.565481063	HAPLN3	protein_coding
ENSG00000140519.14	529.1561474	-0.063891533	0.301919349	-0.211617882	0.832405157	0.917453796	RHCG	protein_coding
ENSG00000140521.17	3475.955707	0.12133826	0.063777416	1.902527065	0.057102284	0.193841726	POLG	protein_coding
ENSG00000140522.12	11.39145353	0.052165055	0.202839092	0.257174563	0.79704402	0.898440873	RLBP1	protein_coding
ENSG00000140525.19	2674.738453	0.086674545	0.083905083	1.033007074	0.301600588	0.531238837	FANCI	protein_coding
ENSG00000140526.18	24491.07542	0.21840031	0.105535624	2.069446338	0.038504222	0.150241626	ABHD2	protein_coding
ENSG00000140527.15	42.58088612	-0.166912219	0.140011496	-1.19213224	0.233209397	0.45767016	WDR93	protein_coding
ENSG00000140534.14	1119.739489	0.057525986	0.10105583	0.569249556	0.569186795	0.75606453	TICRR	protein_coding
ENSG00000140538.16	105.0326456	0.208297819	0.180884951	1.15154864	0.249506597	0.476253386	NTRK3	protein_coding
ENSG00000140543.15	66.97478387	-0.098947416	0.097111144	-1.018908971	0.308246184	0.537694772	DET1	protein_coding
ENSG00000140545.15	7152.82045	0.284030812	0.087514227	3.245538736	0.001172286	0.012965663	MFGE8	protein_coding
ENSG00000140548.10	2290.291355	0.070329422	0.065642036	1.071408303	0.283985881	0.512942582	ZNF710	protein_coding
ENSG00000140553.18	3714.094187	0.126828086	0.054194006	2.340260383	0.0192703	0.094554875	UNC45A	protein_coding
ENSG00000140557.12	48.12204306	0.451243001	0.170840743	2.641307888	0.008258663	0.052909108	ST8SIA2	protein_coding
ENSG00000140563.15	1676.812444	0.205391876	0.086730483	2.368162487	0.017876683	0.089992447	MCTP2	protein_coding
ENSG00000140564.12	12528.90833	0.395913706	0.084444267	4.688461635	2.75267E-06	0.000116543	FURIN	protein_coding
ENSG00000140575.13	27642.64653	0.06063304	0.061454166	0.986638393	0.323819942	0.553571911	IQGAP1	protein_coding
ENSG00000140577.16	3883.190298	0.111384267	0.065545368	1.699346123	0.089253987	0.256944273	CRTC3	protein_coding
ENSG00000140598.15	1503.341995	0.010072179	0.05592333	0.180106916	0.857068633	0.930223657	EFL1	protein_coding
ENSG00000140600.17	13.078274	0.552301747	0.261113908	2.115175522	0.03441499	0.13931381	SH3GL3	protein_coding
ENSG00000140612.14	5154.46036	0.073308232	0.049892227	1.469331734	0.141742833	0.340046735	SEC11A	protein_coding
ENSG00000140623.14	4.048040292	0.020232038	0.20813125	0.097208075	0.922561155	0.963373667	SEPTIN12	protein_coding
ENSG00000140632.17	7036.770242	-0.108922099	0.048833679	-2.230470872	0.025716199	0.115102179	GLYR1	protein_coding
ENSG00000140650.12	1072.817994	0.068282101	0.064202874	1.063536516	0.287538715	0.51721599	PMM2	protein_coding
ENSG00000140675.13	54.32560661	0.137286173	0.118503927	1.158494715	0.246662207	0.473161025	SLC5A2	protein_coding
ENSG00000140678.17	1740.483067	0.006698915	0.109462413	0.061198313	0.951201273	0.977149514	ITGAX	protein_coding
ENSG00000140682.19	4107.945351	0.012415983	0.126988912	0.097772183	0.922113196	0.963204297	TGFB1I1	protein_coding
ENSG00000140688.17	2517.577618	0.154169713	0.062549834	2.464750147	0.013710882	0.07533739	RUSF1	protein_coding
ENSG00000140691.18	773.187895	0.058544485	0.060472353	0.968119852	0.332984516	0.562573809	ARMC5	protein_coding
ENSG00000140694.17	2928.979685	-0.047542556	0.042715207	-1.113012435	0.265703089	0.49361275	PARN	protein_coding
ENSG00000140718.21	2846.405361	0.017618795	0.051612939	0.34136392	0.732829633	0.861151007	FTO	protein_coding
ENSG00000140740.11	10441.56165	-0.116531367	0.056235541	-2.072201397	0.038246663	0.149531208	UQCRC2	protein_coding
ENSG00000140743.8	2062.775074	0.089815417	0.051522009	1.743243677	0.081291059	0.242565084	CDR2	protein_coding
ENSG00000140749.9	973.3702907	-0.368353091	0.106387861	-3.462360166	0.00053546	0.007155395	IGSF6	protein_coding
ENSG00000140750.17	4237.184397	0.012402963	0.045233275	0.274199987	0.783930938	0.890360678	ARHGAP17	protein_coding
ENSG00000140795.13	30.31798726	-0.113426168	0.125222957	-0.905793722	0.365045045	0.592284158	MYLK3	protein_coding
ENSG00000140798.16	8.07177264	-0.213019942	0.306068516	-0.695987764	0.486436492	0.694056818	ABCC12	protein_coding
ENSG00000140807.7	1516.475933	-0.758904454	0.191688506	-3.959050388	7.52484E-05	0.001592091	NKD1	protein_coding
ENSG00000140829.12	2968.039971	0.033885415	0.049909185	0.678941476	0.497174944	0.703311503	DHX38	protein_coding
ENSG00000140830.9	749.9732856	-0.098042777	0.053036314	-1.848597132	0.064516007	0.209011527	TXNL4B	protein_coding
ENSG00000140832.10	1333.249094	-0.069682942	0.093085806	-0.748588274	0.454105402	0.66841844	MARVELD3	protein_coding

ENSG00000140835.10	266.9614031	0.54272241	0.236382312	2.295951863	0.021678634	0.102594353	CHST4	protein_coding
ENSG00000140836.17	5103.799385	-0.056372012	0.076351775	-0.73831959	0.460320265	0.673427206	ZFHX3	protein_coding
ENSG00000140839.11	18.47807716	0.566105004	0.152298035	3.717086731	0.000201533	0.00341322	CLEC18B	protein_coding
ENSG00000140848.17	1980.992017	0.177454507	0.071080527	2.496527739	0.012541586	0.070710919	CPNE2	protein_coding
ENSG00000140853.16	5071.004628	-0.089469172	0.09379125	-0.95391811	0.340125095	0.569953135	NLRC5	protein_coding
ENSG00000140854.13	1409.451016	0.035023216	0.052198478	0.670962395	0.502244486	0.707104807	KATNB1	protein_coding
ENSG00000140859.16	1729.076547	0.274148091	0.088446958	3.099576253	0.001937977	0.018696307	KIFC3	protein_coding
ENSG00000140873.16	67.91994969	0.036281546	0.149393851	0.242858363	0.808115121	0.904833954	ADAMTS18	protein_coding
ENSG00000140876.11	63.21721545	-0.361390776	0.092989157	-3.886375437	0.000101752	0.002010908	NUDT7	protein_coding
ENSG00000140905.11	59.28629047	-0.228906778	0.090868724	-2.519093111	0.011765754	0.067631745	GCSH	protein_coding
ENSG00000140931.20	1752.322779	0.397983935	0.095644852	4.161059665	3.16774E-05	0.000796797	CMTM3	protein_coding
ENSG00000140932.10	41.14918653	0.309771873	0.12451348	2.487858128	0.012851499	0.07200205	CMTM2	protein_coding
ENSG00000140937.14	5230.645253	0.518009788	0.112804427	4.592105153	4.38797E-06	0.00016862	CDH11	protein_coding
ENSG00000140939.14	831.0612159	0.13353101	0.082457524	1.619391463	0.105363063	0.284209606	NOL3	protein_coding
ENSG00000140941.14	6403.210884	0.102785573	0.051190449	2.007905265	0.04465336	0.165407978	MAP1LC3B	protein_coding
ENSG00000140943.17	7130.278436	0.080815153	0.037124779	2.176852128	0.029491602	0.126131667	MBTPS1	protein_coding
ENSG00000140945.17	695.0759147	0.173855149	0.100087888	1.73702486	0.082382783	0.244550036	CDH13	protein_coding
ENSG00000140948.13	4158.078969	0.141402485	0.054240257	2.606965587	0.009134854	0.056713141	ZCCHC14	protein_coding
ENSG00000140950.16	928.8611182	0.223279707	0.06560756	3.403261889	0.000665864	0.008412979	MEAK7	protein_coding
ENSG00000140955.11	6.216533518	-0.265144627	0.202603427	-1.308687771	0.190640136	0.406394932	ADAD2	protein_coding
ENSG00000140961.14	471.4788899	-0.293494315	0.106249043	-2.762324307	0.005739144	0.04106645	OSGIN1	protein_coding
ENSG00000140968.11	3370.528187	-0.175719707	0.104565895	-1.680468634	0.092866172	0.263276897	IRF8	protein_coding
ENSG00000140983.14	3508.385305	-0.073628727	0.05120667	-1.43787376	0.150469876	0.352774832	RHOT2	protein_coding
ENSG00000140986.8	24.18998161	-0.272656801	0.146141019	-1.865710272	0.062081912	0.204263851	RPL3L	protein_coding
ENSG00000140987.20	852.5964236	-0.150920248	0.038591739	-3.910687953	9.20336E-05	0.001855876	ZSCAN32	protein_coding
ENSG00000140988.16	84029.87051	-0.184829947	0.077378175	-2.38865735	0.016910065	0.086969187	RPS2	protein_coding
ENSG00000140990.15	3988.861874	-0.112907485	0.062057796	-1.819392433	0.06885158	0.217570881	NDUFB10	protein_coding
ENSG00000140992.19	2272.957023	-0.08835995	0.049097248	-1.79969251	0.071909204	0.223607932	PDPK1	protein_coding
ENSG00000140993.11	65.1895974	0.085520401	0.086356244	0.990320991	0.32201725	0.551832384	TIGD7	protein_coding
ENSG00000140995.17	1936.60628	-0.01310946	0.053226481	-0.246295823	0.805453243	0.903363893	DEF8	protein_coding
ENSG00000141002.20	5032.041928	-0.021097952	0.044776803	-0.471180395	0.637511918	0.803727957	TCF25	protein_coding
ENSG00000141012.13	1941.433242	0.146125334	0.065892781	2.217622806	0.026580562	0.117653746	GALNS	protein_coding
ENSG00000141013.17	564.6611084	0.032451209	0.060736036	0.53429909	0.593134622	0.773248577	GAS8	protein_coding
ENSG00000141026.6	722.2779707	-0.011567343	0.050916378	-0.227183147	0.820281332	0.911546886	MED9	protein_coding
ENSG00000141027.21	15294.40196	-0.177817528	0.059492092	-2.988927131	0.002799589	0.024350835	NCOR1	protein_coding
ENSG00000141028.6	19.68543664	0.139580322	0.123637918	1.128944289	0.258921336	0.486127318	CDRT15P1	unprocessed_pseudogene
ENSG00000141030.13	2689.569874	-0.062754431	0.049751374	-1.26136078	0.207178892	0.427043377	COPS3	protein_coding
ENSG00000141034.10	870.2531357	-0.024313724	0.060714605	-0.400459237	0.688818301	0.835652425	GID4	protein_coding
ENSG00000141040.15	205.8835027	-0.061873015	0.097664559	-0.633525768	0.526390359	0.725167735	ZNF287	protein_coding
ENSG00000141052.18	1227.448421	-0.587654172	0.201448971	-2.917136628	0.00353261	0.02900161	MYOCD	protein_coding
ENSG00000141068.14	1359.988842	0.210607399	0.079715544	2.641986594	0.008242132	0.05284982	KSR1	protein_coding
ENSG00000141076.18	3482.122657	-0.059344702	0.056928557	-1.042441709	0.297206937	0.527103971	UTP4	protein_coding

ENSG00000141084.11	1810.818283	-0.019449911	0.044533156	-0.436751237	0.662291766	0.819107943	RANBP10	protein_coding
ENSG00000141086.18	0.230012229	0.103410214	0.429047424	0.241022807	0.80953744		CTRL	protein_coding
ENSG00000141096.6	13.07408528	-0.495455197	0.179557539	-2.759311577	0.005792328	0.0413181	DPEP3	protein_coding
ENSG00000141098.13	1410.986223	0.052448836	0.049431531	1.061040104	0.288671676	0.51844072	GFOD2	protein_coding
ENSG00000141101.13	2897.947093	-0.071364007	0.062465984	-1.142445899	0.253268722	0.480129221	NOB1	protein_coding
ENSG00000141127.15	1294.128304	-0.149162467	0.050587516	-2.948602322	0.003192144	0.026906012	PRPSAP2	protein_coding
ENSG00000141161.12	26.47833198	-0.351587271	0.195244303	-1.800755598	0.07174141	0.223298785	UNC45B	protein_coding
ENSG00000141179.14	1405.522029	-0.218734985	0.081232044	-2.692717978	0.007087219	0.047376166	PCTP	protein_coding
ENSG00000141194.6	5.843612293	-0.491582776	0.190885104	-2.575280969	0.010015869	0.060543975	OR4D1	protein_coding
ENSG00000141198.16	2304.021521	-0.061983875	0.078180674	-0.79282861	0.427877685	0.646245361	TOM1L1	protein_coding
ENSG00000141200.8	2.047876433	0.03905831	0.484908569	0.080547782	0.935801595	0.969736361	KIF2B	protein_coding
ENSG00000141219.15	924.5192852	0.026387069	0.050165962	0.525995461	0.598891361	0.777782856	C17orf80	protein_coding
ENSG00000141232.5	6874.872025	0.193865693	0.079360603	2.442845508	0.014571974	0.078472924	TOB1	protein_coding
ENSG00000141252.20	3546.969668	0.036378569	0.047509793	0.765706754	0.443850808	0.659945634	VPS53	protein_coding
ENSG00000141255.13	3.663563893	-0.429278369	0.263390527	-1.629817042	0.103140171	0.280628073	SPATA22	protein_coding
ENSG00000141258.13	2242.67501	-0.113182386	0.068543995	-1.651237084	0.098690174	0.273315646	SGSM2	protein_coding
ENSG00000141279.17	4670.382511	0.076638847	0.040318809	1.900821209	0.057325435	0.194281419	NPEPPS	protein_coding
ENSG00000141293.16	543.271154	-0.031878435	0.10822266	-0.294563402	0.768327424	0.882569021	SKAP1	protein_coding
ENSG00000141294.10	80.49143227	0.066457624	0.097423995	0.682148415	0.495145109	0.701705176	LRRC46	protein_coding
ENSG00000141295.14	1027.845833	-0.045928576	0.063555142	-0.722657126	0.469890563	0.681230333	SCRN2	protein_coding
ENSG00000141298.19	2301.422652	-0.067446631	0.055896818	-1.206627366	0.227575683	0.450946172	SSH2	protein_coding
ENSG00000141314.13	48.47026792	0.580459424	0.165145828	3.514829476	0.000440036	0.006187631	RHBDL3	protein_coding
ENSG00000141316.13	6.221873496	-0.351754819	0.229291312	-1.534095712	0.125006108	0.315096667	SPACA3	protein_coding
ENSG00000141337.12	258.741172	0.066892952	0.077308806	0.865269498	0.386890888	0.611473388	ARSG	protein_coding
ENSG00000141338.14	712.1836892	-0.102274437	0.193938814	-0.527354142	0.597947682	0.777094812	ABCA8	protein_coding
ENSG00000141349.8	4906.526387	0.018776315	0.063202802	0.297080428	0.766405106	0.881343625	G6PC3	protein_coding
ENSG00000141367.12	30138.85304	0.161498109	0.051300804	3.148061978	0.001643568	0.016602175	CLTC	protein_coding
ENSG00000141371.13	5.663722097	0.528152387	0.188776965	2.797758648	0.005145855	0.037949046	C17orf64	protein_coding
ENSG00000141376.23	1418.239678	0.054678435	0.057907884	0.944231266	0.345051481	0.574560871	BCAS3	protein_coding
ENSG00000141378.15	1356.179421	-0.001788066	0.058700123	-0.030461032	0.975699371	0.988837887	PTRH2	protein_coding
ENSG00000141380.14	3980.246028	-0.002968956	0.051629564	-0.057504955	0.954142959	0.978552312	SS18	protein_coding
ENSG00000141384.13	767.486251	-0.153717448	0.083475595	-1.841465737	0.065553337	0.211020845	TAF4B	protein_coding
ENSG00000141385.10	2903.516232	-0.046251506	0.06379571	-0.724993977	0.468455725	0.679873947	AFG3L2	protein_coding
ENSG00000141391.14	146.1354249	-0.004591658	0.095578367	-0.04804077	0.96168375	0.982281748	PRELID3A	protein_coding
ENSG00000141401.12	3496.281949	-0.177063981	0.093703721	-1.889615271	0.058809433	0.197300691	IMPA2	protein_coding
ENSG00000141404.17	449.995667	0.016062753	0.130199259	0.123370538	0.901813686	0.953357148	GNAL	protein_coding
ENSG00000141424.13	3352.684155	0.077365948	0.068299524	1.132745058	0.25732133	0.484375447	SLC39A6	protein_coding
ENSG00000141425.18	3552.543026	0.057216312	0.066323232	0.862688839	0.388308571	0.612802759	RPRD1A	protein_coding
ENSG00000141428.17	524.6310822	-0.206254442	0.063713792	-3.23720241	0.001207078	0.013256255	C18orf21	protein_coding
ENSG00000141429.13	6405.830388	0.060914655	0.068515901	0.889058652	0.373971559	0.599897302	GALNT1	protein_coding
ENSG00000141431.12	84.8889405	-0.077768488	0.160785539	-0.483678378	0.628614141	0.797888804	ASXL3	protein_coding
ENSG00000141433.13	85.66592134	-0.22320427	0.213205503	-1.046897319	0.295146938	0.525045757	ADCYAP1	protein_coding

ENSG00000141434.12	81.63503684	-1.281409514	0.228122978	-5.617187394	1.94091E-08	2.07301E-06	MEP1B	protein_coding
ENSG00000141437.10	3.521628261	-0.294251347	0.175687229	-1.674859063	0.093961887	0.264879841	SLC25A52	protein_coding
ENSG00000141441.16	1824.335238	0.017120667	0.082934949	0.206434895	0.836451216	0.919722528	GAREM1	protein_coding
ENSG00000141446.11	1626.737192	0.073525966	0.064422902	1.141301676	0.253744405	0.48056934	ESCO1	protein_coding
ENSG00000141447.18	1953.708823	-0.027746438	0.095261883	-0.291264853	0.770848766	0.884074025	OSBPL1A	protein_coding
ENSG00000141448.11	5693.535543	-0.326457803	0.108340482	-3.013257806	0.002584593	0.023024122	GATA6	protein_coding
ENSG00000141449.15	235.1182689	0.22202182	0.197360768	1.124954176	0.260608453	0.487802696	GREB1L	protein_coding
ENSG00000141452.10	1400.135613	-0.084749169	0.058699518	-1.443779628	0.148800974	0.350540602	RMC1	protein_coding
ENSG00000141456.16	4639.683417	0.03099539	0.058070417	0.533755261	0.593510871	0.77349712	PELP1	protein_coding
ENSG00000141458.13	4085.172996	0.129982497	0.076245193	1.704795961	0.088232485	0.255119567	NPC1	protein_coding
ENSG00000141469.18	141.0568136	-0.112704749	0.145090149	-0.776791191	0.437281975	0.654286383	SLC14A1	protein_coding
ENSG00000141480.18	2979.776608	-0.062419041	0.061098028	-1.0216212	0.30696022	0.536472507	ARRB2	protein_coding
ENSG00000141485.17	59.07495303	-0.590078894	0.2207779	-2.672726272	0.007523761	0.049488748	SLC13A5	protein_coding
ENSG00000141497.14	459.2733544	-0.046141041	0.099298334	-0.464670849	0.642167187	0.806403842	ZMYND15	protein_coding
ENSG00000141499.17	727.9209143	0.010218004	0.056465193	0.180961104	0.856398107	0.929985884	WRAP53	protein_coding
ENSG00000141503.17	8714.282541	0.07600115	0.060121169	1.264132938	0.206182316	0.426022133	MINK1	protein_coding
ENSG00000141504.12	1372.296441	-0.193876813	0.065492802	-2.960276654	0.003073629	0.026160064	SAT2	protein_coding
ENSG00000141505.12	180.7651979	-0.428059052	0.162651098	-2.631762453	0.008494323	0.053883193	ASGR1	protein_coding
ENSG00000141506.14	710.0989789	-0.163664425	0.112320143	-1.457124442	0.145082033	0.345213483	PIK3R5	protein_coding
ENSG00000141510.18	5286.775892	-0.081883286	0.094056426	-0.8705762	0.383985596	0.608913404	TP53	protein_coding
ENSG00000141519.15	307.9015615	0.011272866	0.102753801	0.10970753	0.912641328	0.958725835	CCDC40	protein_coding
ENSG00000141522.12	12720.13748	0.081203145	0.058816514	1.380618135	0.167396404	0.3763418	ARHGDIA	protein_coding
ENSG00000141524.17	5059.113014	0.188235396	0.074392645	2.5302958	0.01139664	0.066164587	TMC6	protein_coding
ENSG00000141526.17	4379.962335	0.820377002	0.101300879	8.0984194	5.56778E-16	7.5821E-13	SLC16A3	protein_coding
ENSG00000141527.18	469.0022793	0.447940596	0.146308491	3.061617214	0.002201448	0.02048089	CARD14	protein_coding
ENSG00000141540.11	540.9254583	0.117023269	0.119143417	0.982205077	0.325998827	0.555770544	TTYH2	protein_coding
ENSG00000141542.11	2088.641519	0.033307717	0.09171985	0.363146228	0.716495653	0.851891146	RAB40B	protein_coding
ENSG00000141543.12	5017.727896	0.097870327	0.053794397	1.819340538	0.068859492	0.217570881	EIF4A3	protein_coding
ENSG00000141551.14	6693.421361	0.087568324	0.039420725	2.221377806	0.026325383	0.116856597	CSNK1D	protein_coding
ENSG00000141552.18	3180.668349	0.039586564	0.066584594	0.594530387	0.552157424	0.744081519	ANAPC11	protein_coding
ENSG00000141556.21	4360.246893	0.072477086	0.051788441	1.399483828	0.161667944	0.368355628	TBCD	protein_coding
ENSG00000141560.15	1552.088375	0.038645897	0.054676738	0.706806937	0.479686463	0.688909419	FN3KRP	protein_coding
ENSG00000141562.18	2037.648023	0.079843434	0.04840006	1.649655675	0.099013381	0.273931881	NARF	protein_coding
ENSG00000141564.15	3153.06337	0.048816869	0.055522279	0.879230284	0.379276427	0.604928628	RPTOR	protein_coding
ENSG00000141568.21	4501.971504	0.159042416	0.048326684	3.290985522	0.000998371	0.011479154	FOXK2	protein_coding
ENSG00000141569.12	1547.746149	0.119116349	0.050644001	2.352032768	0.01867113	0.092658221	TRIM65	protein_coding
ENSG00000141570.11	520.8785489	0.03722002	0.082980783	0.448537818	0.653765099	0.813908893	CBX8	protein_coding
ENSG00000141574.8	3408.08392	0.00566981	0.125759742	0.045084457	0.96403999	0.983471548	SECTM1	protein_coding
ENSG00000141576.16	541.440782	-0.042529433	0.136335988	-0.31194576	0.755081745	0.874274556	RNF157	protein_coding
ENSG00000141577.14	1105.79982	0.054379775	0.070902665	0.766963766	0.443103059	0.659261225	CEP131	protein_coding
ENSG00000141579.7	121.5543153	1.162056119	0.214492775	5.417693533	6.03728E-08	5.43567E-06	ZNF750	protein_coding
ENSG00000141580.16	6668.139753	0.049627772	0.060762199	0.816754049	0.414068997	0.634640068	WDR45B	protein_coding

ENSG00000141582.15	3431.575189	0.023761091	0.076353435	0.311198713	0.755649561	0.87467636	CBX4	protein_coding
ENSG00000141622.14	68.93212314	-0.045078285	0.160814195	-0.280312848	0.779237494	0.888374224	RNF165	protein_coding
ENSG00000141627.14	3019.130524	-0.069947816	0.049589244	-1.410544112	0.158379081	0.363840223	DYM	protein_coding
ENSG00000141639.12	215.9966403	-0.144650661	0.24832945	-0.582494992	0.560233314	0.749655688	MAPK4	protein_coding
ENSG00000141642.9	252.2896556	-0.250214924	0.060202879	-4.156195305	3.23591E-05	0.00081041	ELAC1	protein_coding
ENSG00000141644.17	3193.037845	0.00481996	0.043797672	0.110050597	0.912369248	0.958582856	MBD1	protein_coding
ENSG00000141646.14	3014.828192	-0.08688858	0.061858082	-1.404643956	0.160127179	0.366046313	SMAD4	protein_coding
ENSG00000141655.17	2823.767118	-0.235871342	0.134100047	-1.758920656	0.078590986	0.237548008	TNFRSF11A	protein_coding
ENSG00000141664.10	1658.350293	-0.034800417	0.056560675	-0.615275841	0.538372569	0.734356602	ZCCHC2	protein_coding
ENSG00000141665.13	20.98899232	0.179333163	0.143523567	1.249503248	0.211481066	0.431875851	FBXO15	protein_coding
ENSG00000141668.10	12.76007721	-0.096336799	0.215657735	-0.44671154	0.655083348	0.814554302	CBLN2	protein_coding
ENSG00000141682.12	769.2445266	0.145618513	0.114645253	1.270166096	0.204025472	0.423405744	PMAIP1	protein_coding
ENSG00000141696.13	2889.161602	0.007715014	0.121002235	0.063759271	0.949161909	0.976210607	P3H4	protein_coding
ENSG00000141698.17	1795.189982	-0.091095399	0.11140826	-0.817671864	0.413544581	0.63409684	NT5C3B	protein_coding
ENSG00000141699.11	3631.359698	0.062174257	0.040473028	1.53618992	0.124491807	0.314218331	RETREG3	protein_coding
ENSG00000141736.14	21460.17001	-0.124117253	0.134845808	-0.920438354	0.357343734	0.585104377	ERBB2	protein_coding
ENSG00000141738.14	5405.949532	0.014634192	0.157847135	0.092711169	0.926133023	0.965066089	GRB7	protein_coding
ENSG00000141741.12	3463.318575	-0.45306372	0.121134591	-3.740167985	0.000183897	0.003199079	MIEN1	protein_coding
ENSG00000141744.4	46.91933818	0.19816154	0.229891976	0.861976757	0.388700308	0.613176706	PNMT	protein_coding
ENSG00000141748.13	3.388146419	-0.19280907	0.243327327	-0.792385598	0.428135874	0.646327063	ARL5C	protein_coding
ENSG00000141750.7	17.18541971	0.44540753	0.168081516	2.649949504	0.00805038	0.052002879	STAC2	protein_coding
ENSG00000141753.7	30945.14582	0.157362371	0.125891885	1.24998026	0.211306759	0.431718065	IGFBP4	protein_coding
ENSG00000141756.19	7346.779196	0.129807429	0.146328767	0.88709439	0.375028084	0.600808751	FKBP10	protein_coding
ENSG00000141759.15	2963.664469	-0.095927723	0.060984336	-1.572989559	0.115721236	0.300665959	TXNL4A	protein_coding
ENSG00000141837.21	426.4187105	-0.082457973	0.143644077	-0.574043665	0.565938253	0.754211588	CACNA1A	protein_coding
ENSG00000141854.9	1294.826451	-0.250186973	0.110074977	-2.272877817	0.023033544	0.107019165	MISP3	protein_coding
ENSG00000141858.12	4147.937289	-0.15986636	0.064269197	-2.487449151	0.012866285	0.072038299	SAMD1	protein_coding
ENSG00000141867.18	10036.11017	0.057771788	0.040704977	1.419280698	0.155817197	0.360405839	BRD4	protein_coding
ENSG00000141873.11	1019.815846	0.009515028	0.060008446	0.158561481	0.874014378	0.939114606	SLC39A3	protein_coding
ENSG00000141905.19	15650.9286	0.013980177	0.082613651	0.169223565	0.865620795	0.934842448	NFIC	protein_coding
ENSG00000141933.9	426.4409104	0.056501342	0.076037733	0.743069788	0.45743943	0.670998953	TPGS1	protein_coding
ENSG00000141934.10	5716.326474	0.064458676	0.099815108	0.645780758	0.518421351	0.719205043	PLPP2	protein_coding
ENSG00000141946.1	3.954709325	0.049062685	0.330316354	0.148532411	0.881922609	0.94338312	ZIM3	protein_coding
ENSG00000141956.14	701.2772101	-0.029291452	0.047676244	-0.614382533	0.538962575	0.734768781	PRDM15	protein_coding
ENSG00000141959.17	11838.80316	0.000443976	0.064263984	0.006908629	0.994487756	0.997553032	PFKL	protein_coding
ENSG00000141965.5	168.2696028	-0.051807704	0.069730853	-0.742966731	0.457501823	0.671002953	FEM1A	protein_coding
ENSG00000141968.8	780.8561891	-0.353374752	0.114031216	-3.098929956	0.001942209	0.018722724	VAV1	protein_coding
ENSG00000141971.13	1891.911116	-0.080336232	0.064498067	-1.245560313	0.21292586	0.433547787	MVB12A	protein_coding
ENSG00000141977.9	2.862722405	-0.141986132	0.211050228	-0.672759909	0.501100047	0.706383617	CIB3	protein_coding
ENSG00000141979.4	0.68654822	-0.040442041	0.304083939	-0.132996308	0.894196301		AC008764.1	protein_coding
ENSG00000141985.9	5495.395065	0.116072175	0.057487388	2.019089381	0.04347793	0.162532193	SH3GL1	protein_coding
ENSG00000141994.16	1207.142033	0.026370675	0.063444513	0.415649416	0.677666561	0.829263066	DUS3L	protein_coding

ENSG00000142002.17	6254.915438	0.020359339	0.043254482	0.470687386	0.637863994	0.803945443	DPP9	protein_coding
ENSG00000142025.16	4.974217833	0.396769073	0.282251108	1.405730791	0.15980408	0.365591264	DMRTC2	protein_coding
ENSG00000142039.4	2689.490372	0.023277092	0.05310095	0.438355472	0.66112862	0.818452557	CCDC97	protein_coding
ENSG00000142046.15	110.5718708	-0.023841054	0.080556088	-0.295955956	0.767263717	0.881912086	TMEM91	protein_coding
ENSG00000142065.14	533.5895876	-0.216358479	0.068007475	-3.181392628	0.001465688	0.015294613	ZFP14	protein_coding
ENSG00000142082.15	883.0824751	-0.08877275	0.043929544	-2.02079838	0.043300641	0.16223128	SIRT3	protein_coding
ENSG00000142089.16	42070.43854	-0.035351681	0.094090256	-0.375720958	0.707124356	0.846666043	IFITM3	protein_coding
ENSG00000142102.16	2735.267274	-0.1483237	0.123453244	-1.201456475	0.22957418	0.453148257	PGGHG	protein_coding
ENSG00000142149.9	677.231318	-0.368557903	0.171465176	-2.149462129	0.031597785	0.131972858	HUNK	protein_coding
ENSG00000142156.15	31385.06063	0.45532126	0.10265046	4.435647528	9.17959E-06	0.000303045	COL6A1	protein_coding
ENSG00000142163.8	0.399782392	0.231936133	0.483976449	0.479230205	0.631774868		OR1E3	unprocessed_pseudogene
ENSG00000142166.13	4957.583981	0.033201706	0.041668932	0.796797608	0.425568584	0.644360722	IFNAR1	protein_coding
ENSG00000142168.15	8901.980243	-0.013893383	0.07338877	-0.189312117	0.849848199	0.926337233	SOD1	protein_coding
ENSG00000142173.16	39333.53742	0.295625999	0.112330688	2.631747414	0.008494699	0.053883193	COL6A2	protein_coding
ENSG00000142178.9	196.1006599	0.520537398	0.166635357	3.123811222	0.00178525	0.017644777	SIK1	protein_coding
ENSG00000142182.9	3.353726586	0.174020789	0.314872056	0.552671428	0.580488422	0.764320279	DNMT3L	protein_coding
ENSG00000142185.16	1044.867759	0.109403355	0.096522627	1.133447752	0.257026271	0.484050036	TRPM2	protein_coding
ENSG00000142186.17	5310.864927	0.173934607	0.044455566	3.912549633	9.13268E-05	0.001850184	SCYL1	protein_coding
ENSG00000142188.17	2872.845663	-0.039189575	0.053003499	-0.739377131	0.459678025	0.673010285	TMEM50B	protein_coding
ENSG00000142192.21	46221.96422	0.114374536	0.059436386	1.92431849	0.054314674	0.187638383	APP	protein_coding
ENSG00000142197.12	3657.42488	0.022743714	0.072592298	0.313307538	0.754047024	0.873703293	DOP1B	protein_coding
ENSG00000142207.7	3128.931127	0.104024935	0.067318452	1.545266286	0.122281867	0.311072791	URB1	protein_coding
ENSG00000142208.17	6828.763095	0.086971407	0.05303806	1.639792388	0.101048341	0.277193415	AKT1	protein_coding
ENSG00000142224.17	44.94189268	-0.102052001	0.204812712	-0.498269858	0.61829385	0.790940587	IL19	protein_coding
ENSG00000142227.11	1981.246941	-0.049887936	0.10689121	-0.46671692	0.640702423	0.805484971	EMP3	protein_coding
ENSG00000142230.13	4633.467869	-0.046667169	0.056623833	-0.82416125	0.409847941	0.63082593	SAE1	protein_coding
ENSG00000142233.14	25.30481513	-0.045060387	0.115960472	-0.388584028	0.697583884	0.840646357	NTN5	protein_coding
ENSG00000142235.12	761.4690949	0.024372391	0.114291337	0.213247926	0.831133588	0.916779941	LMTK3	protein_coding
ENSG00000142252.11	35.32032974	-0.144563235	0.079137634	-1.826731836	0.067740099	0.215440062	GEMIN7	protein_coding
ENSG00000142273.13	3780.883717	-0.01175481	0.109951078	-0.106909456	0.914860811	0.959722907	CBLC	protein_coding
ENSG00000142279.13	634.2525658	0.262654891	0.107733912	2.437996417	0.01476892	0.079256455	WTIP	protein_coding
ENSG00000142303.14	470.1963123	0.247352637	0.110487515	2.238738354	0.025172945	0.11358449	ADAMTS10	protein_coding
ENSG00000142319.18	16.04061181	-0.080449428	0.198096539	-0.406112235	0.684660136	0.83357695	SLC6A3	protein_coding
ENSG00000142327.13	9984.828401	-0.005364887	0.061120984	-0.087774882	0.930055602	0.966811279	RNPEPL1	protein_coding
ENSG00000142330.20	1081.475003	-0.005093322	0.056640373	-0.08992388	0.928347704	0.966035235	CAPN10	protein_coding
ENSG00000142347.19	1582.167709	-0.148343516	0.097811613	-1.516624781	0.129361445	0.321720334	MYO1F	protein_coding
ENSG00000142396.10	294.1702626	-0.095841809	0.064579111	-1.484099218	0.137782601	0.334193951	ERVK3-1	protein_coding
ENSG00000142405.22	45.80164001	0.219974234	0.141020801	1.559870824	0.11879041	0.305418002	NLRP12	protein_coding
ENSG00000142408.6	112.4444231	-0.160191814	0.168322428	-0.951696194	0.34125108	0.571239279	CACNG8	protein_coding
ENSG00000142409.6	2791.356325	-0.039105277	0.075649525	-0.516926942	0.60520716	0.782340546	ZNF787	protein_coding
ENSG00000142444.7	810.3678335	-0.058261973	0.048547574	-1.200100622	0.230100264	0.453839173	TIMM29	protein_coding
ENSG00000142449.13	59.73333079	0.039694749	0.235498744	0.168556096	0.866145817	0.935082793	FBN3	protein_coding

ENSG00000142453.12	4870.742191	0.058068622	0.056770048	1.022874267	0.306367298	0.535845612	CARM1	protein_coding
ENSG00000142459.8	1246.285397	0.099532542	0.068288664	1.457526574	0.144971081	0.345043684	EVI5L	protein_coding
ENSG00000142484.7	1375.479552	-0.219544634	0.161837516	-1.356574416	0.174916475	0.386273833	TM4SF5	protein_coding
ENSG00000142494.13	164.3526546	-0.479655024	0.128945143	-3.719837852	0.000199351	0.003380197	SLC47A1	protein_coding
ENSG00000142507.10	4234.639173	-0.063799729	0.057821984	-1.103381869	0.269861367	0.497999017	PSMB6	protein_coding
ENSG00000142511.4	2.477829232	-0.019237092	0.346336255	-0.055544553	0.955704637	0.979439358	GPR32	protein_coding
ENSG00000142512.15	570.9329986	-0.385953538	0.129285217	-2.985287463	0.002833119	0.024567997	SIGLEC10	protein_coding
ENSG00000142513.6	14.63632798	0.056083429	0.129709804	0.432376179	0.665468029	0.821132073	ACP4	protein_coding
ENSG00000142515.15	16.25076423	0.480505387	0.23495861	2.045063971	0.040848552	0.156008531	KLK3	protein_coding
ENSG00000142528.16	837.6927842	-0.000148881	0.060428694	-0.002463755	0.99803421	0.998928218	ZNF473	protein_coding
ENSG00000142530.11	159.4877475	0.249811057	0.143332804	1.742874272	0.081355579	0.242691133	FAM71E1	protein_coding
ENSG00000142534.7	57311.87701	-0.216949266	0.067599926	-3.209312195	0.001330529	0.014243873	RPS11	protein_coding
ENSG00000142538.2	0.794327021	0.593303374	0.378128953	1.569050371	0.116636203		PTH2	protein_coding
ENSG00000142539.9	0						AC020909.1	protein_coding
ENSG00000142541.18	78380.04214	-0.200621955	0.067757679	-2.960874079	0.003067673	0.026124683	RPL13A	protein_coding
ENSG00000142544.7	760.6972915	-0.040996238	0.078986052	-0.519031363	0.60373887	0.781217314	CTU1	protein_coding
ENSG00000142546.14	2412.648798	-0.127567066	0.059324904	-2.150312226	0.031530526	0.131800643	NOSIP	protein_coding
ENSG00000142549.9	45.59897425	0.641824047	0.149973927	4.279570842	1.87254E-05	0.000527471	IGLON5	protein_coding
ENSG00000142552.8	2636.597132	0.498438418	0.107899566	4.619466398	3.84728E-06	0.000151448	RCN3	protein_coding
ENSG00000142556.19	658.1543429	0.11730903	0.099585416	1.177973994	0.238806974	0.464243911	ZNF614	protein_coding
ENSG00000142583.18	392.9670444	-0.254104957	0.128815079	-1.972633628	0.048537319	0.174629355	SLC2A5	protein_coding
ENSG00000142599.19	11091.46302	-0.123026104	0.069406055	-1.772555789	0.076302343	0.232877658	RERE	protein_coding
ENSG00000142606.16	95.7111843	0.322103592	0.149231304	2.158418396	0.030895317	0.130029478	MMEL1	protein_coding
ENSG00000142609.18	66.18506052	0.870126218	0.19143261	4.545339563	5.48468E-06	0.000201353	CFAP74	protein_coding
ENSG00000142611.17	1099.511052	-0.376248733	0.132661669	-2.836152557	0.004566063	0.034905587	PRDM16	protein_coding
ENSG00000142615.8	6.147934858	-0.906058218	0.230204041	-3.935891887	8.28883E-05	0.001714297	CELA2A	protein_coding
ENSG00000142619.4	62.36404062	0.598373111	0.246814341	2.424385508	0.015334318	0.081254205	PADI3	protein_coding
ENSG00000142621.19	223.8751335	0.117512269	0.114547711	1.025880552	0.304947891	0.534370043	FHAD1	protein_coding
ENSG00000142623.11	128.4438171	1.784783268	0.255636757	6.981716116	2.91596E-12	1.47754E-09	PADI1	protein_coding
ENSG00000142627.13	9436.722655	0.542408109	0.110265062	4.919129407	8.693E-07	4.67096E-05	EPHA2	protein_coding
ENSG00000142632.17	781.5383667	-0.030576295	0.115034841	-0.265800298	0.790393014	0.894783026	ARHGEF19	protein_coding
ENSG00000142634.13	14240.68311	-0.022362391	0.077438678	-0.288775476	0.7727532	0.885000426	EFHD2	protein_coding
ENSG00000142655.13	1356.505674	-0.014800827	0.057099211	-0.259212457	0.79547132	0.897642326	PEX14	protein_coding
ENSG00000142657.21	8130.407183	-0.059322015	0.08058193	-0.736170188	0.461627133	0.674207379	PGD	protein_coding
ENSG00000142661.19	581.6809325	-0.267517143	0.192619294	-1.388838772	0.164881782	0.372713413	MYOM3	protein_coding
ENSG00000142669.15	18778.15927	0.006825663	0.08094162	0.084328224	0.932795473	0.968238421	SH3BGRL3	protein_coding
ENSG00000142675.18	1868.803992	0.057911264	0.082061855	0.705702599	0.480373104	0.689386424	CNKSR1	protein_coding
ENSG00000142676.14	41154.1302	-0.273511211	0.06322173	-4.326221525	1.51689E-05	0.000450282	RPL11	protein_coding
ENSG00000142677.4	1986.280279	-0.209606021	0.135344051	-1.548690317	0.121456183	0.309856524	IL22RA1	protein_coding
ENSG00000142684.9	0						ZNF593	protein_coding
ENSG00000142686.8	615.9985295	0.107488911	0.060548433	1.775255036	0.075855786	0.231872025	C1orf216	protein_coding
ENSG00000142687.18	6181.249273	-0.037573881	0.053627212	-0.700649547	0.48352175	0.691559252	KIAA0319L	protein_coding

ENSG00000142694.6	0						EVA1B	protein_coding
ENSG00000142698.15	3.132841406	0.244819896	0.235447826	1.039805291	0.298430371	0.528251334	C1orf94	protein_coding
ENSG00000142700.12	58.32701401	0.90497195	0.303034276	2.986368281	0.002823124	0.024506631	DMRTA2	protein_coding
ENSG00000142731.11	947.9758536	-0.025699714	0.083080474	-0.309335187	0.757066567	0.875393631	PLK4	protein_coding
ENSG00000142733.17	2215.213523	0.234687975	0.082211286	2.8546929	0.004307846	0.033432416	MAP3K6	protein_coding
ENSG00000142748.13	67.23333858	-0.185525302	0.098375202	-1.885894997	0.059309106	0.198457568	FCN3	protein_coding
ENSG00000142751.15	1680.610886	-0.07430208	0.040298211	-1.843805933	0.065211428	0.210466478	GPN2	protein_coding
ENSG00000142765.18	1662.739453	0.022218901	0.107820122	0.20607379	0.836733272	0.919831527	SYTL1	protein_coding
ENSG00000142784.16	5811.285202	-0.036482111	0.057601654	-0.633351788	0.526503941	0.725209794	WDTC1	protein_coding
ENSG00000142789.20	4.012098558	-0.950524731	0.283893047	-3.348178981	0.000813445	0.009800242	CELA3A	protein_coding
ENSG00000142794.19	438.8259415	-0.070388696	0.093539924	-0.752498966	0.451751052	0.666347262	NBPF3	protein_coding
ENSG00000142798.20	47174.70632	0.159205774	0.104927242	1.51729685	0.129191753	0.321500144	HSPG2	protein_coding
ENSG00000142856.17	877.2728641	-0.161863291	0.057498023	-2.815110545	0.004876046	0.036578318	ITGB3BP	protein_coding
ENSG00000142864.15	20081.0765	-0.056570893	0.041650888	-1.358215775	0.174395227	0.385728392	SERBP1	protein_coding
ENSG00000142867.14	1948.720287	0.102020153	0.067245065	1.517139618	0.129231438	0.321562174	BCL10	protein_coding
ENSG00000142871.18	7021.465586	0.407691768	0.129501607	3.148159919	0.001643018	0.016600586	CCN1	protein_coding
ENSG00000142875.19	2738.290025	-0.181266957	0.100059895	-1.81158451	0.07005042	0.219847833	PRKACB	protein_coding
ENSG00000142892.15	2257.129844	-0.058785057	0.047472828	-1.238288485	0.215609112	0.436842025	PIGK	protein_coding
ENSG00000142910.16	7314.314899	0.201073004	0.081827777	2.45727077	0.013999712	0.076377467	TINAGL1	protein_coding
ENSG00000142920.17	173.1578252	0.305084078	0.090745917	3.36195928	0.000773915	0.009462657	AZIN2	protein_coding
ENSG00000142937.12	50351.38359	-0.163577712	0.063604786	-2.571783071	0.010117627	0.060966406	RPS8	protein_coding
ENSG00000142945.13	1888.35694	0.050569637	0.089564526	0.564616812	0.572334428	0.758350078	KIF2C	protein_coding
ENSG00000142949.17	27473.23004	0.094510795	0.079167428	1.193809079	0.232552659	0.456944023	PTPRF	protein_coding
ENSG00000142959.5	50.3485948	0.382608867	0.133043994	2.875807135	0.004029959	0.031952967	BEST4	protein_coding
ENSG00000142961.15	1093.746981	-0.049532192	0.050528597	-0.980280383	0.326947735	0.556723797	MOB3C	protein_coding
ENSG00000142973.14	116.5322719	0.178282887	0.221162465	0.806117289	0.420175224	0.639993309	CYP4B1	protein_coding
ENSG00000143001.5	40.54981435	0.65647001	0.179421746	3.658809621	0.000253389	0.004086585	TMEM61	protein_coding
ENSG00000143006.7	2.760158578	-0.352815688	0.35902803	-0.982696777	0.325756698	0.555451608	DMRTB1	protein_coding
ENSG00000143013.13	7298.254121	-0.466530734	0.105882348	-4.406123798	1.05237E-05	0.000339194	LMO4	protein_coding
ENSG00000143028.9	129.0395255	-0.043709657	0.15526754	-0.281511881	0.778317817	0.887826697	SYPL2	protein_coding
ENSG00000143032.8	3.026623392	0.873143142	0.25782596	3.386560228	0.000707747	0.008826991	BARHL2	protein_coding
ENSG00000143033.18	1468.596768	-0.168805724	0.049076343	-3.439655689	0.000582455	0.007631277	MTF2	protein_coding
ENSG00000143036.17	1764.927331	-0.022361496	0.087069713	-0.256822894	0.797315496	0.898572557	SLC44A3	protein_coding
ENSG00000143061.18	2097.51652	0.190429438	0.099727014	1.909507063	0.056196711	0.191723487	IGSF3	protein_coding
ENSG00000143067.5	649.6977842	0.202248688	0.085099302	2.376619824	0.017472086	0.088677041	ZNF697	protein_coding
ENSG00000143079.15	2936.538306	0.217650269	0.052468798	4.148184775	3.35122E-05	0.000832589	CTTNBP2NL	protein_coding
ENSG00000143093.15	1843.593891	-0.017000384	0.040252727	-0.42234116	0.672776019	0.825971693	STRIP1	protein_coding
ENSG00000143105.7	7.076102955	-0.047717335	0.244379222	-0.19525938	0.845189895	0.92412094	KCNA10	protein_coding
ENSG00000143106.13	5899.792812	-0.092848454	0.054407556	-1.70653603	0.087908324	0.25467232	PSMA5	protein_coding
ENSG00000143107.10	4.227235332	-0.137438593	0.233398647	-0.588857711	0.555956728	0.74675779	FNDC7	protein_coding
ENSG00000143110.12	1073.708128	-0.141813863	0.115389252	-1.229004087	0.219070274	0.440719511	C1orf162	protein_coding
ENSG00000143119.14	2965.761878	-0.400954042	0.121163812	-3.309189716	0.000935664	0.010901988	CD53	protein_coding

ENSG00000143125.6	3.100471943	0.834163437	0.244828862	3.407128685	0.000656502	0.008330918	PROK1	protein_coding
ENSG00000143126.8	2929.723948	0.257035721	0.11573997	2.220803412	0.026364279	0.116974485	CELSR2	protein_coding
ENSG00000143127.13	129.7542487	0.164574466	0.108372247	1.51860343	0.128862347	0.320900309	ITGA10	protein_coding
ENSG00000143147.14	1019.250176	0.045253282	0.109755463	0.412310065	0.680112173	0.830523978	GPR161	protein_coding
ENSG00000143149.12	3768.705304	-0.009035423	0.055450728	-0.16294507	0.870561684	0.937489906	ALDH9A1	protein_coding
ENSG00000143153.13	32361.38407	0.231578813	0.103403823	2.239557555	0.025119661	0.113528258	ATP1B1	protein_coding
ENSG00000143155.13	2741.303414	0.047308749	0.040316304	1.173439645	0.240619547	0.466062672	TIPRL	protein_coding
ENSG00000143156.14	460.7923747	0.11273983	0.061636392	1.829111439	0.067382916	0.21473931	NME7	protein_coding
ENSG00000143157.12	5223.98566	0.205883638	0.044531573	4.623318317	3.7765E-06	0.000149441	POGK	protein_coding
ENSG00000143158.11	3114.237531	-0.305017037	0.071842675	-4.245624724	2.17985E-05	0.00059856	MPC2	protein_coding
ENSG00000143162.9	9880.868906	-0.076837595	0.052173772	-1.472724559	0.140825322	0.338656201	CREG1	protein_coding
ENSG00000143164.15	3034.88609	-0.052561876	0.043867085	-1.198207646	0.23083619	0.454803808	DCAF6	protein_coding
ENSG00000143167.12	2768.912724	-0.126908379	0.224341468	-0.565692916	0.571602551	0.757748977	GPA33	protein_coding
ENSG00000143171.13	22.23386701	0.019901531	0.196061557	0.101506545	0.919148363	0.961830145	RXRG	protein_coding
ENSG00000143178.13	208.0393054	0.17844056	0.068879622	2.590614673	0.00958047	0.058627739	TBX19	protein_coding
ENSG00000143179.16	2172.179294	0.008821449	0.063199923	0.139580061	0.888991795	0.946992776	UCK2	protein_coding
ENSG00000143183.18	6621.763844	-0.014494777	0.070107142	-0.206751793	0.836203706	0.919595616	TMCO1	protein_coding
ENSG00000143184.5	65.47997615	0.124730915	0.158681231	0.786047061	0.431839917	0.649802764	XCL1	protein_coding
ENSG00000143185.4	41.53447511	-0.474009949	0.149572476	-3.16909877	0.001529124	0.015733328	XCL2	protein_coding
ENSG00000143190.23	4056.607239	0.029715036	0.053754106	0.552795663	0.580403339	0.76427753	POU2F1	protein_coding
ENSG00000143194.13	23.70794787	0.367661836	0.191845492	1.916447611	0.055308146	0.189805147	MAEL	protein_coding
ENSG00000143195.13	251.5455296	0.599529011	0.154720458	3.874917499	0.000106661	0.002083352	ILDR2	protein_coding
ENSG00000143196.5	160.942549	-0.530219137	0.19259241	-2.753063518	0.005904044	0.041841035	DPT	protein_coding
ENSG00000143198.13	6232.050828	-0.114281425	0.069398213	-1.646748822	0.099609685	0.274847366	MGST3	protein_coding
ENSG00000143199.18	28.42120618	-0.15245848	0.14844725	-1.027021249	0.304410459	0.533836152	ADCY10	protein_coding
ENSG00000143207.21	3041.991289	0.022909616	0.042865601	0.534452232	0.59302869	0.773194983	COP1	protein_coding
ENSG00000143217.9	1189.368263	0.552414724	0.137112394	4.028918969	5.60339E-05	0.001255419	NECTIN4	protein_coding
ENSG00000143222.12	4027.966109	-0.069857884	0.055725563	-1.253605717	0.209985352	0.430309508	UFC1	protein_coding
ENSG00000143224.18	592.0709444	-0.035737501	0.054804645	-0.652088906	0.514343821	0.716294164	PPOX	protein_coding
ENSG00000143226.15	2706.978962	0.178684019	0.108735042	1.643297472	0.100321401	0.276049708	FCGR2A	protein_coding
ENSG00000143228.13	1022.578506	0.029285717	0.095983637	0.30511156	0.760281187	0.877287787	NUF2	protein_coding
ENSG00000143248.13	18838.61685	-0.413020406	0.112778456	-3.662227878	0.000250031	0.004039901	RGS5	protein_coding
ENSG00000143252.16	3562.458538	-0.005102122	0.055439086	-0.092031141	0.926673298	0.965318453	SDHC	protein_coding
ENSG00000143256.5	2252.596771	0.030851037	0.065404964	0.471692587	0.637146229	0.803499384	PFDN2	protein_coding
ENSG00000143257.12	28.83559857	-0.130231591	0.110200351	-1.181771105	0.237296536	0.462462931	NR1I3	protein_coding
ENSG00000143258.16	2075.941101	0.039450607	0.045484632	0.867339256	0.385756153	0.610344053	USP21	protein_coding
ENSG00000143278.5	3.975104281	-0.77318025	0.280388802	-2.757528992	0.005824005	0.041455841	F13B	protein_coding
ENSG00000143294.15	3576.553294	0.066926701	0.045790385	1.461588519	0.143854002	0.343190948	PRCC	protein_coding
ENSG00000143297.19	525.0723505	-0.780199387	0.167266886	-4.66439834	3.09521E-06	0.000127006	FCRL5	protein_coding
ENSG00000143303.12	1146.404743	0.100047	0.055483939	1.803170463	0.071361448	0.222456385	RRNAD1	protein_coding
ENSG00000143314.12	3183.889491	-0.087591477	0.065494175	-1.337393387	0.181094262	0.394283458	MRPL24	protein_coding
ENSG00000143315.8	2073.805381	0.130010944	0.060358086	2.153993798	0.031240658	0.131039962	PIGM	protein_coding

ENSG00000143318.13	37.24245904	-0.435884887	0.206387846	-2.111969742	0.034689045	0.139967083	CASQ1	protein_coding
ENSG00000143319.17	3464.433677	0.124394897	0.046417126	2.679935361	0.007363638	0.048781582	ISG20L2	protein_coding
ENSG00000143320.9	1381.079276	1.253414851	0.195016926	6.427210599	1.29967E-10	3.39135E-08	CRABP2	protein_coding
ENSG00000143321.19	25138.54277	0.034446295	0.064693746	0.532451698	0.594413187	0.774302558	HDGF	protein_coding
ENSG00000143322.21	2718.069609	0.184198588	0.050704854	3.632760471	0.000280405	0.004417651	ABL2	protein_coding
ENSG00000143324.14	3903.407896	0.067376694	0.060640249	1.111088665	0.266530186	0.494554786	XPR1	protein_coding
ENSG00000143333.7	1074.071412	0.149150082	0.114578756	1.301725441	0.193010259	0.409404599	RGS16	protein_coding
ENSG00000143337.19	4322.973833	0.031430113	0.056074949	0.56050186	0.575137171	0.760135843	TOR1AIP1	protein_coding
ENSG00000143340.6	54.46188148	0.143892216	0.178888975	0.804366037	0.42118561	0.640829766	FAM163A	protein_coding
ENSG00000143341.12	1444.664637	0.547302372	0.145541059	3.760467162	0.000169596	0.002989684	HMCN1	protein_coding
ENSG00000143344.16	1383.985686	-0.16660868	0.097404143	-1.71048865	0.08717555	0.253138466	RGL1	protein_coding
ENSG00000143353.12	827.4193352	-0.25868078	0.062502079	-4.138754803	3.49196E-05	0.000861848	LYPLAL1	protein_coding
ENSG00000143355.16	15.97138457	1.798623173	0.261185317	6.886387013	5.72272E-12	2.62504E-09	LHX9	protein_coding
ENSG00000143363.17	1956.165247	0.045801816	0.049764362	0.920373825	0.357377442	0.5851156	PRUNE1	protein_coding
ENSG00000143365.19	1013.555658	-0.094187635	0.157608076	-0.597606657	0.550102421	0.742874375	RORC	protein_coding
ENSG00000143367.16	1626.029003	0.253630911	0.076124508	3.331790474	0.000862892	0.010245843	TUFT1	protein_coding
ENSG00000143368.10	7931.107725	0.083509068	0.050623046	1.649625524	0.099019551	0.273931881	SF3B4	protein_coding
ENSG00000143369.15	4121.770157	1.166904874	0.139027121	8.393361412	4.72436E-17	8.95103E-14	ECM1	protein_coding
ENSG00000143373.18	3718.174691	0.154606223	0.056899434	2.717183837	0.006584003	0.045111805	ZNF687	protein_coding
ENSG00000143374.17	1971.116308	-0.045785164	0.05386419	-0.850011179	0.395318871	0.618757402	TARS2	protein_coding
ENSG00000143375.15	7644.985338	0.201274655	0.104178199	1.932022797	0.053356692	0.185330189	CGN	protein_coding
ENSG00000143376.14	3485.849684	0.028472851	0.047074814	0.604842557	0.545283614	0.739608228	SNX27	protein_coding
ENSG00000143379.12	1896.400574	0.106672452	0.048868912	2.182828467	0.029048444	0.124851798	SETDB1	protein_coding
ENSG00000143382.15	1213.898293	0.452107338	0.123390112	3.664048351	0.00024826	0.004015747	ADAMTSL4	protein_coding
ENSG00000143384.14	24464.5351	0.193894329	0.053280127	3.639149125	0.00027354	0.004331502	MCL1	protein_coding
ENSG00000143387.14	4851.335245	0.468873452	0.109150625	4.295655206	1.74178E-05	0.000497389	CTSK	protein_coding
ENSG00000143390.18	3035.149647	-0.12485653	0.060682386	-2.057541545	0.039634163	0.153182964	RFX5	protein_coding
ENSG00000143393.16	4453.600537	0.120741116	0.042060992	2.870619794	0.004096679	0.0322998	PI4KB	protein_coding
ENSG00000143398.20	4243.209374	0.160860254	0.049416382	3.255200989	0.001133121	0.012644823	PIP5K1A	protein_coding
ENSG00000143401.15	4585.11071	0.114626065	0.070477394	1.626423136	0.103859672	0.281773528	ANP32E	protein_coding
ENSG00000143409.15	1756.768837	-0.101520226	0.073700808	-1.377464213	0.168368772	0.377631683	MINDY1	protein_coding
ENSG00000143412.10	701.9555784	-0.124087261	0.12340114	-1.005560088	0.314627231	0.543873651	ANXA9	protein_coding
ENSG00000143416.21	4289.702121	-0.217677471	0.113710511	-1.914312671	0.055580216	0.190275677	SELENBP1	protein_coding
ENSG00000143418.20	6128.849373	0.105906586	0.067243088	1.574981	0.11526083	0.299704233	CERS2	protein_coding
ENSG00000143420.19	8023.368788	-0.002400207	0.047288658	-0.050756501	0.959519553	0.981202975	ENSA	protein_coding
ENSG00000143429.10	87.04366674	0.164674786	0.13098991	1.257156258	0.208697059	0.428657228	LSP1P4	transcribed_unprocessed_pseudogene
ENSG00000143434.16	599.1617681	0.041754329	0.104407444	0.399917163	0.689217531	0.835787737	SEMA6C	protein_coding
ENSG00000143436.11	1294.501442	-0.006503538	0.056780047	-0.114539151	0.908810412	0.956900267	MRPL9	protein_coding
ENSG00000143437.21	3423.495271	0.112912301	0.041428622	2.725465989	0.006421079	0.044316023	ARNT	protein_coding
ENSG00000143442.22	5504.700002	0.061710205	0.049819087	1.238685993	0.215461808	0.436611658	POGZ	protein_coding
ENSG00000143443.10	458.1936157	0.04153219	0.085938583	0.483277575	0.62889866	0.798110459	C1orf56	protein_coding
ENSG00000143450.18	43.51680588	0.097619549	0.069260357	1.409457776	0.15869985	0.3642672	OAZ3	protein_coding

ENSG00000143452.16	137.9315064	0.923990007	0.24151012	3.825885261	0.000130303	0.002424514	HORMAD1	protein_coding
ENSG00000143457.11	3188.615795	0.1768709	0.059134366	2.991000191	0.002780653	0.024234505	GOLPH3L	protein_coding
ENSG00000143458.12	1593.657132	-0.019506298	0.068926404	-0.283001828	0.777175436	0.887011182	GABPB2	protein_coding
ENSG00000143469.20	50.25412384	-0.121307763	0.202689613	-0.598490279	0.549512843	0.742400284	SYT14	protein_coding
ENSG00000143473.13	12.06335283	0.296322561	0.1295511	2.287302541	0.02217817	0.104127444	KCNH1	protein_coding
ENSG00000143476.18	1603.169362	0.039872594	0.088093529	0.452616609	0.650824837	0.811858685	DTL	protein_coding
ENSG00000143479.17	847.0494812	0.313240947	0.077436272	4.04514497	5.22908E-05	0.001193648	DYRK3	protein_coding
ENSG00000143486.16	2221.652742	-0.028347383	0.051077126	-0.554991742	0.578900309	0.763151584	EIF2D	protein_coding
ENSG00000143493.13	2142.777627	0.063478587	0.054811912	1.158116641	0.24681644	0.473281124	INTS7	protein_coding
ENSG00000143494.15	247.8679499	0.035217795	0.105355758	0.334274987	0.738172063	0.864434161	VASH2	protein_coding
ENSG00000143498.18	385.803317	0.130119502	0.063970817	2.034044714	0.041947087	0.158853587	TAF1A	protein_coding
ENSG00000143499.14	1844.803192	0.123280067	0.057984172	2.12609858	0.033495052	0.136867004	SMYD2	protein_coding
ENSG00000143502.15	488.5560603	-0.383100651	0.200821568	-1.907666865	0.056434287	0.192278102	SUSD4	protein_coding
ENSG00000143507.18	969.8522721	0.199957582	0.095666213	2.09015885	0.036603533	0.145048832	DUSP10	protein_coding
ENSG00000143512.13	20.00971394	0.332859369	0.181027957	1.838718039	0.065956669	0.211695791	HHIPL2	protein_coding
ENSG00000143514.17	3585.839053	0.169530138	0.046983899	3.608260319	0.000308257	0.004744938	TP53BP2	protein_coding
ENSG00000143515.19	2031.671046	0.041489342	0.113240372	0.366382951	0.71407934	0.850557998	ATP8B2	protein_coding
ENSG00000143520.6	4.632048652	0.443509266	0.266891122	1.661761032	0.096560698	0.269352551	FLG2	protein_coding
ENSG00000143536.7	179.5271694	-0.014930847	0.649462861	-0.022989531	0.981658624	0.99199355	CRNN	protein_coding
ENSG00000143537.14	11513.21218	0.19165074	0.073155185	2.619783429	0.008798562	0.055161997	ADAM15	protein_coding
ENSG00000143543.15	1078.291721	-0.059143327	0.082215774	-0.719367148	0.471914733	0.682949421	JTB	protein_coding
ENSG00000143545.10	1801.354873	0.023550914	0.092909968	0.253481021	0.799896526	0.900234786	RAB13	protein_coding
ENSG00000143546.10	1454.246509	0.726140435	0.215704223	3.366370967	0.000761642	0.009357225	S100A8	protein_coding
ENSG00000143549.21	26378.25354	0.059336663	0.050587503	1.172951007	0.240815453	0.466317766	TPM3	protein_coding
ENSG00000143552.9	33.49739334	0.049024222	0.16326962	0.300265427	0.763974702	0.879678529	NUP210L	protein_coding
ENSG00000143553.11	1350.069611	0.092028104	0.051213554	1.796948213	0.072343842	0.224475049	SNAPIN	protein_coding
ENSG00000143554.14	1557.618281	-0.048679568	0.077432743	-0.628669035	0.529565754	0.727679087	SLC27A3	protein_coding
ENSG00000143556.9	850.2868102	2.442878609	0.392290201	6.227223115	4.74775E-10	9.66788E-08	S100A7	protein_coding
ENSG00000143569.19	12297.60595	0.158358819	0.038005102	4.166777937	3.08935E-05	0.000779981	UBAP2L	protein_coding
ENSG00000143570.19	61.20313928	0.129896631	0.089265069	1.455178741	0.145619785	0.345982954	SLC39A1	protein_coding
ENSG00000143575.15	4463.623089	0.003218952	0.058098585	0.055404991	0.95581582	0.979484197	HAX1	protein_coding
ENSG00000143578.16	682.090807	0.23846854	0.09718591	2.453735732	0.014138084	0.076925369	CREB3L4	protein_coding
ENSG00000143590.14	554.3910459	0.314590678	0.149247851	2.107840582	0.035044781	0.140893665	EFNA3	protein_coding
ENSG00000143595.13	20.9913524	-0.340824211	0.238484436	-1.429125595	0.152968138	0.356369557	AQP10	protein_coding
ENSG00000143603.19	762.4714923	-0.409099436	0.112918643	-3.622957418	0.000291254	0.004547462	KCNN3	protein_coding
ENSG00000143612.21	10736.98586	0.126555952	0.051380089	2.463132233	0.013772911	0.075533125	C1orf43	protein_coding
ENSG00000143614.10	3760.517712	0.135595171	0.042364588	3.2006725	0.001371073	0.014618849	GATAD2B	protein_coding
ENSG00000143621.17	10413.90548	0.034446531	0.056420461	0.610532608	0.54150905	0.736657193	ILF2	protein_coding
ENSG00000143622.11	1223.840085	0.145941186	0.051168815	2.852151009	0.004342447	0.033635051	RIT1	protein_coding
ENSG00000143624.14	4835.262215	0.070370592	0.046489214	1.513697166	0.130102664	0.322868726	INTS3	protein_coding
ENSG00000143627.19	46.65150889	-0.626634714	0.251296946	-2.49360258	0.012645404	0.071121421	PKLR	protein_coding
ENSG00000143630.10	427.97878	0.259148591	0.088791626	2.918615208	0.003515899	0.028909297	HCN3	protein_coding

ENSG00000143631.11	38.98664607	0.738178921	0.194429118	3.796647998	0.000146666	0.002673583	FLG	protein_coding
ENSG00000143632.14	39.75039578	-0.214446603	0.134780799	-1.591076798	0.111592293	0.293960053	ACTA1	protein_coding
ENSG00000143633.13	1310.84743	-0.219390617	0.058382942	-3.757786273	0.000171423	0.003015789	C1orf131	protein_coding
ENSG00000143641.10	5904.662481	0.142520113	0.065851628	2.16426106	0.030444319	0.128778112	GALNT2	protein_coding
ENSG00000143643.13	1510.449118	-0.068790909	0.059073911	-1.164488823	0.24422597	0.47047589	TTC13	protein_coding
ENSG00000143653.10	2792.059612	-0.019835923	0.070716084	-0.280500862	0.779093264	0.888362435	SCCPDH	protein_coding
ENSG00000143669.14	2117.988929	0.030157707	0.069073667	0.436602081	0.662399953	0.819178782	LYST	protein_coding
ENSG00000143674.11	1960.606449	-0.013171599	0.087104822	-0.151215495	0.879805728	0.942112069	MAP3K21	protein_coding
ENSG00000143702.16	1602.878029	0.049903823	0.075912166	0.657388994	0.510930827	0.713982575	CEP170	protein_coding
ENSG00000143727.16	4436.742825	-0.052329847	0.050483763	-1.036567863	0.299937297	0.529586719	ACP1	protein_coding
ENSG00000143740.14	1461.518057	0.041629407	0.044163305	0.942624351	0.34587308	0.575315337	SNAP47	protein_coding
ENSG00000143742.14	9854.588853	0.042640525	0.054703011	0.779491364	0.435690323	0.653273138	SRP9	protein_coding
ENSG00000143748.18	1050.542761	-0.013701088	0.044398933	-0.308590476	0.757633066	0.875809113	NVL	protein_coding
ENSG00000143751.10	1931.687595	0.050562146	0.046584503	1.085385524	0.277750941	0.506669363	SDE2	protein_coding
ENSG00000143753.13	3578.964898	-0.10592017	0.062327819	-1.699404407	0.089243012	0.256944273	DEGS1	protein_coding
ENSG00000143756.12	3258.112066	0.130343783	0.042349578	3.077805915	0.002085307	0.019754655	FBXO28	protein_coding
ENSG00000143761.16	35272.07254	0.060305195	0.047054778	1.281595573	0.199984554	0.418385703	ARF1	protein_coding
ENSG00000143768.13	28.72607841	0.261150978	0.205940047	1.268092253	0.204765008	0.424280573	LEFTY2	protein_coding
ENSG00000143771.12	2314.187333	0.051341185	0.052978994	0.969085698	0.332502433	0.562042611	CNIH4	protein_coding
ENSG00000143772.9	3574.148219	-0.354704157	0.117595559	-3.016305722	0.002558751	0.022858207	ITPKB	protein_coding
ENSG00000143774.17	10039.31352	0.072571712	0.058063243	1.249873544	0.211345745	0.431777475	GUK1	protein_coding
ENSG00000143776.18	6626.069572	0.152140425	0.076557115	1.987280014	0.046891375	0.170672013	CDC42BPA	protein_coding
ENSG00000143786.8	143.4355279	0.343928857	0.102860145	3.343655178	0.000826824	0.009925765	CNIH3	protein_coding
ENSG00000143793.13	1123.362681	-0.043149827	0.064249991	-0.671592738	0.501843003	0.706996989	C1orf35	protein_coding
ENSG00000143797.12	2877.243421	0.632887286	0.110652965	5.719569181	1.06794E-08	1.26462E-06	MBOAT2	protein_coding
ENSG00000143799.14	10885.20279	-0.022364052	0.057976014	-0.385746627	0.699684327	0.841966806	PARP1	protein_coding
ENSG00000143801.18	84.23817225	-0.045759548	0.069833639	-0.655265125	0.51229707	0.714860208	PSEN2	protein_coding
ENSG00000143811.19	1525.131581	0.056044808	0.05536788	1.01222599	0.311430012	0.540986432	PYCR2	protein_coding
ENSG00000143815.15	7145.475049	-0.105910724	0.068778376	-1.539884051	0.123588619	0.312935575	LBR	protein_coding
ENSG00000143816.8	350.1548286	0.097819111	0.171723135	0.569632688	0.568926853	0.755903575	WNT9A	protein_coding
ENSG00000143819.12	5107.191675	-0.092307073	0.08212928	-1.123924044	0.26104525	0.488179936	EPHX1	protein_coding
ENSG00000143839.15	12.78989828	0.186858525	0.215701375	0.866283423	0.386334754	0.610926792	REN	protein_coding
ENSG00000143842.15	2804.252179	0.184798521	0.09185155	2.011925997	0.044227738	0.164460462	SOX13	protein_coding
ENSG00000143845.15	246.3228703	0.428376532	0.161136614	2.658468001	0.00784968	0.051069801	ETNK2	protein_coding
ENSG00000143847.15	81.89318501	0.245215878	0.142341456	1.722729871	0.084937386	0.249296215	PPFIA4	protein_coding
ENSG00000143850.16	7728.318073	0.143188515	0.085502526	1.674669989	0.093998999	0.264912105	PLEKHA6	protein_coding
ENSG00000143851.15	989.0130671	-0.47063304	0.125799778	-3.741127752	0.000183196	0.003191027	PTPN7	protein_coding
ENSG00000143858.12	63.098816	0.067766261	0.123508871	0.548675249	0.583228335	0.766491982	SYT2	protein_coding
ENSG00000143862.8	3257.339191	0.124438246	0.054484459	2.283921844	0.022376125	0.104802706	ARL8A	protein_coding
ENSG00000143867.7	236.8990988	-0.038940668	0.150378638	-0.258950796	0.795673205	0.897777148	OSR1	protein_coding
ENSG00000143869.7	102.1687916	-0.175064423	0.165326024	-1.058904209	0.289643407	0.519458011	GDF7	protein_coding
ENSG00000143870.13	10315.94512	0.112907028	0.069392345	1.627081907	0.103719703	0.281571985	PDIA6	protein_coding

ENSG00000143878.10	20088.36574	0.034190563	0.107548417	0.31790857	0.750554292	0.871951596	RHOB	protein_coding
ENSG00000143882.12	328.8236761	0.424845947	0.162161199	2.6198989	0.008795584	0.05515688	ATP6V1C2	protein_coding
ENSG00000143889.16	2645.824269	-0.093693387	0.033511373	-2.795868363	0.005176048	0.038120101	HNRNPLL	protein_coding
ENSG00000143891.17	3182.717064	-0.317128163	0.079677979	-3.980123055	6.88796E-05	0.001487397	GALM	protein_coding
ENSG00000143919.15	521.9651702	-0.169749664	0.059236984	-2.865602732	0.004162162	0.032609589	CAMKMT	protein_coding
ENSG00000143921.9	192.7977371	-0.406944908	0.205869725	-1.976710792	0.04807433	0.17355108	ABCG8	protein_coding
ENSG00000143924.19	8028.256686	-0.086403521	0.055272572	-1.563226006	0.117999445	0.304150364	EML4	protein_coding
ENSG00000143933.19	23268.11059	-0.112410581	0.051435661	-2.185460021	0.028855134	0.124324716	CALM2	protein_coding
ENSG00000143942.5	322.3143331	-0.183001809	0.092537747	-1.977590933	0.047974873	0.173346909	CHAC2	protein_coding
ENSG00000143947.14	30931.55327	-0.196164968	0.058403897	-3.358765023	0.000782916	0.009529926	RPS27A	protein_coding
ENSG00000143951.16	364.1316486	-0.099818656	0.053716039	-1.858265407	0.063131334	0.206205936	WDPCP	protein_coding
ENSG00000143952.20	2613.919844	-0.02953576	0.04475941	-0.659878223	0.50933198	0.712807469	VPS54	protein_coding
ENSG00000143954.13	62.93818755	-2.971895324	0.368111557	-8.073355122	6.83923E-16	9.03131E-13	REG3G	protein_coding
ENSG00000143970.17	4385.314329	0.037247683	0.049570944	0.751401533	0.45241104	0.666904436	ASXL2	protein_coding
ENSG00000143971.9	911.50188	0.005965063	0.051030685	0.116891691	0.906945883	0.955912919	ETAA1	protein_coding
ENSG00000143977.14	2818.340569	-0.101044429	0.064733228	-1.560936041	0.118538843	0.304976316	SNRPG	protein_coding
ENSG00000143994.14	44.84721753	-0.283708035	0.133073382	-2.131966818	0.033009574	0.13561405	ABHD1	protein_coding
ENSG00000143995.20	1310.652097	-0.113024583	0.121748914	-0.928341611	0.353230396	0.581603603	MEIS1	protein_coding
ENSG00000144010.10	1.100990214	-0.932407868	0.417160907	-2.235127626	0.025408971	0.114255158	TRIM43B	protein_coding
ENSG00000144015.5	0.882173346	0.060541664	0.396680168	0.152620848	0.878697283		TRIM43	protein_coding
ENSG00000144021.3	4703.56132	0.002823935	0.030077338	0.093889127	0.925197231	0.96471831	CIAO1	protein_coding
ENSG00000144026.12	854.9496902	0.000122528	0.069600856	0.001760444	0.99859537	0.999341668	ZNF514	protein_coding
ENSG00000144028.15	18324.35499	0.150219075	0.050473447	2.97620007	0.002918444	0.025128835	SNRNP200	protein_coding
ENSG00000144029.12	3070.605132	-0.122048267	0.053447941	-2.283498002	0.02240105	0.104896902	MRPS5	protein_coding
ENSG00000144031.12	57.34884989	0.075358918	0.11328342	0.665224607	0.505906844	0.710036733	ANKRD53	protein_coding
ENSG00000144034.16	1328.542705	-0.158375449	0.061250245	-2.585711278	0.009717829	0.059243684	TPRKB	protein_coding
ENSG00000144035.4	14.04967879	-0.663064526	0.258460355	-2.565439973	0.010304505	0.061706666	NAT8	protein_coding
ENSG00000144036.16	1696.659882	0.051008916	0.057287366	0.890404274	0.373248848	0.59941207	EXOC6B	protein_coding
ENSG00000144040.13	917.2688572	-0.188855375	0.068174629	-2.770170913	0.005602688	0.040341764	SFXN5	protein_coding
ENSG00000144043.12	6387.276569	-0.0373027	0.04231595	-0.881528115	0.378032044	0.603778858	TEX261	protein_coding
ENSG00000144045.14	583.5914923	-0.122378836	0.139098286	-0.879801182	0.378967024	0.604665756	DQX1	protein_coding
ENSG00000144048.10	1470.092358	0.006528561	0.040425453	0.161496293	0.871702531	0.938046208	DUSP11	protein_coding
ENSG00000144057.15	119.1096841	0.916883802	0.17989934	5.096649065	3.45718E-07	2.18656E-05	ST6GAL2	protein_coding
ENSG00000144061.14	267.7897674	-0.068621771	0.097690692	-0.702439189	0.482405313	0.690818371	NPHP1	protein_coding
ENSG00000144063.4	762.1035134	0.204157268	0.145141451	1.406608974	0.159543371	0.365321113	MALL	protein_coding
ENSG00000144115.17	753.9187997	0.270486322	0.156440758	1.729001603	0.083808808	0.247173747	THNSL2	protein_coding
ENSG00000144118.14	4461.748793	0.001272852	0.05108811	0.024914833	0.980122896	0.991223176	RALB	protein_coding
ENSG00000144119.4	4.017301175	-0.069337101	0.330234962	-0.209962933	0.833696603	0.918349023	C1QL2	protein_coding
ENSG00000144120.13	771.6602136	-0.083413335	0.063824659	-1.306913921	0.191241948	0.407159974	TMEM177	protein_coding
ENSG00000144130.11	28.76853039	-0.09690858	0.16926477	-0.572526577	0.566965286	0.754685221	NT5DC4	protein_coding
ENSG00000144134.18	317.3087673	0.057082693	0.06260741	0.911756175	0.361897085	0.589497945	RABL2A	protein_coding
ENSG00000144136.11	3555.546335	0.125355581	0.068935772	1.818440223	0.068996875	0.217834165	SLC20A1	protein_coding

ENSG00000144152.13	160.5910442	0.258943517	0.10963194	2.361935014	0.018179829	0.091080984	FBLN7	protein_coding
ENSG00000144158.4	19.35348099	0.053714106	0.089035908	0.60328588	0.546318524	0.740241981	SNRPA1P1	processed_pseudogene
ENSG00000144161.13	715.1715949	-0.195211914	0.059167851	-3.299290256	0.000969296	0.011186183	ZC3H8	protein_coding
ENSG00000144182.17	251.8927998	-0.159348899	0.051831581	-3.074359203	0.002109553	0.019885078	LIPT1	protein_coding
ENSG00000144188.9	0.424433215	-0.141724208	0.551304508	-0.25707065	0.797124235		TRIM43CP	unprocessed_pseudogene
ENSG00000144191.12	33.52931767	-0.5124208	0.20277168	-2.527082676	0.011501442	0.066604433	CNGA3	protein_coding
ENSG00000144199.12	283.582547	-0.150978148	0.091809006	-1.64448081	0.100076925	0.275632168	FAHD2B	protein_coding
ENSG00000144214.10	54.20174473	0.067359803	0.117554208	0.573010563	0.566637542	0.754587421	LYG1	protein_coding
ENSG00000144218.19	399.5766959	-0.667854643	0.172845671	-3.863878331	0.000111601	0.002155687	AFF3	protein_coding
ENSG00000144224.17	11077.42225	-0.032558072	0.043036512	-0.756522088	0.449336234	0.664156818	UBXN4	protein_coding
ENSG00000144227.5	4.336396216	0.022790077	0.30272978	0.075281912	0.939990413	0.971622282	NXPH2	protein_coding
ENSG00000144228.9	1804.225019	-0.035216764	0.049516464	-0.711213214	0.476952122	0.686806642	SPOPL	protein_coding
ENSG00000144229.12	99.81609401	0.263176869	0.203671858	1.292161183	0.196301331	0.413487196	THSD7B	protein_coding
ENSG00000144230.16	52.14765714	-0.385117629	0.172857974	-2.227942513	0.025884349	0.115593591	GPR17	protein_coding
ENSG00000144231.11	1822.387435	-0.028917281	0.043348708	-0.667085178	0.504717727	0.708777816	POLR2D	protein_coding
ENSG00000144233.10	1711.411791	0.127619025	0.036572097	3.489518913	0.000483891	0.006630978	AMMECR1L	protein_coding
ENSG00000144278.15	74.21222741	1.442761454	0.221510348	6.513291457	7.35217E-11	2.1359E-08	GALNT13	protein_coding
ENSG00000144283.22	6067.415818	-0.113130728	0.054146674	-2.089338442	0.036677268	0.145206261	PKP4	protein_coding
ENSG00000144285.22	14.72443569	-0.357149432	0.242377121	-1.473527827	0.140608766	0.338391177	SCN1A	protein_coding
ENSG00000144290.17	25.37276327	-0.415744299	0.148185702	-2.80556284	0.005022879	0.037320031	SLC4A10	protein_coding
ENSG00000144306.15	1192.321758	0.040066571	0.061279868	0.653829263	0.513221811	0.715588822	SCRN3	protein_coding
ENSG00000144320.14	2891.91952	0.003358472	0.044261781	0.075877476	0.939516577	0.971324173	LNPK	protein_coding
ENSG00000144331.20	150.2317261	-0.34393516	0.180642188	-1.903958117	0.056915639	0.193449248	ZNF385B	protein_coding
ENSG00000144339.12	22.898428	-0.059781947	0.209683545	-0.285105571	0.775563262	0.886285377	TMEFF2	protein_coding
ENSG00000144354.14	4483.038699	-0.309062348	0.113102804	-2.732579004	0.00628406	0.043681684	CDCA7	protein_coding
ENSG00000144355.15	15.23057936	0.146248043	0.205986114	0.709989815	0.477710452	0.687376205	DLX1	protein_coding
ENSG00000144357.18	3392.725657	-0.002214028	0.046639402	-0.047471202	0.962137682	0.98245088	UBR3	protein_coding
ENSG00000144362.12	136.710246	-0.137889671	0.070676729	-1.950991122	0.051058102	0.180319224	PHOSPHO2	protein_coding
ENSG00000144366.16	1720.861309	0.360020112	0.115331122	3.121621518	0.00179858	0.017718143	GULP1	protein_coding
ENSG00000144369.13	533.0277971	0.310831621	0.105602017	2.943425032	0.003246024	0.027254721	FAM171B	protein_coding
ENSG00000144381.18	23934.33898	-0.108148052	0.075518383	-1.432075841	0.152122118	0.3549679	HSPD1	protein_coding
ENSG00000144395.18	185.1809409	0.115890738	0.110326829	1.050431151	0.29351993	0.523501738	CCDC150	protein_coding
ENSG00000144401.14	995.7746229	-0.115649901	0.068491339	-1.688533212	0.091308919	0.260403715	METTL21A	protein_coding
ENSG00000144406.19	33.85610981	-0.420196609	0.204800734	-2.051733904	0.04019553	0.154429912	UNC80	protein_coding
ENSG00000144407.9	81.80817319	-0.286753499	0.273450864	-1.048647258	0.294340497	0.524385766	PTH2R	protein_coding
ENSG00000144410.5	9.520390729	-0.095905761	0.161334494	-0.59445292	0.552209223	0.744119968	CPO	protein_coding
ENSG00000144426.18	1344.539642	0.188305753	0.079093127	2.380810589	0.017274592	0.088085055	NBEAL1	protein_coding
ENSG00000144445.17	1156.096155	0.02569368	0.061905574	0.415046315	0.678107999	0.829354821	KANSL1L	protein_coding
ENSG00000144451.19	540.3040118	0.014923044	0.125569688	0.118842724	0.90539996	0.955016921	SPAG16	protein_coding
ENSG00000144452.15	516.3126352	1.02294168	0.215697911	4.742473755	2.11124E-06	9.381E-05	ABCA12	protein_coding
ENSG00000144455.14	1855.947279	0.116351149	0.062806506	1.852533372	0.06394927	0.207932946	SUMF1	protein_coding
ENSG00000144460.12	21.87677582	1.106377	0.204944862	5.398412958	6.7233E-08	5.9549E-06	NYAP2	protein_coding

ENSG00000144468.17	2180.639278	-0.123745268	0.039951704	-3.097371449	0.001952451	0.018781885	RHBDD1	protein_coding
ENSG00000144476.6	1280.48915	0.501285448	0.094512836	5.303887495	1.13362E-07	8.90087E-06	ACKR3	protein_coding
ENSG00000144481.17	21.1493768	0.33204633	0.190125183	1.746461591	0.080730776	0.241409635	TRPM8	protein_coding
ENSG00000144485.11	1078.280923	0.345276506	0.129692128	2.662278062	0.007761373	0.050661679	HES6	protein_coding
ENSG00000144488.15	137.0792145	0.073864613	0.194808057	0.379166107	0.704564518	0.845108946	ESPNL	protein_coding
ENSG00000144504.15	710.4056937	-0.007891037	0.053040854	-0.148772818	0.8817329	0.94326201	ANKMY1	protein_coding
ENSG00000144524.18	1851.200436	0.005663925	0.042786135	0.132377586	0.894685643	0.949946792	COPS7B	protein_coding
ENSG00000144535.20	1462.605043	-0.032009044	0.03629635	-0.881880516	0.377841424	0.603673538	DIS3L2	protein_coding
ENSG00000144550.13	25.43441911	-0.135684381	0.125197223	-1.083765106	0.27846896	0.507309443	CPNE9	protein_coding
ENSG00000144554.12	1204.318744	0.053585211	0.079677944	0.672522513	0.501251113	0.706464073	FANCD2	protein_coding
ENSG00000144559.10	526.792134	-0.00712919	0.047820512	-0.149082263	0.881488722	0.943104199	TAMM41	protein_coding
ENSG00000144560.15	3199.190253	0.314303807	0.065103936	4.827723568	1.38103E-06	6.70167E-05	VGLL4	protein_coding
ENSG00000144566.11	3730.105449	0.159383969	0.043022699	3.704648323	0.000211684	0.003537026	RAB5A	protein_coding
ENSG00000144567.11	5798.253671	-0.047163206	0.049625163	-0.950388945	0.341914659	0.571720774	RETREG2	protein_coding
ENSG00000144579.8	7857.041632	0.006063384	0.047876224	0.126647078	0.899219734	0.951977512	CTDSP1	protein_coding
ENSG00000144580.14	3449.742353	-0.084555126	0.043942498	-1.924222127	0.054326747	0.187644969	CNOT9	protein_coding
ENSG00000144583.5	41.6137025	0.259251695	0.154192062	1.681355655	0.092693854	0.262925214	MARCHF4	protein_coding
ENSG00000144589.22	1238.467269	0.023338367	0.051090698	0.456802666	0.64781289	0.809948926	STK11IP	protein_coding
ENSG00000144591.19	3608.533015	-0.013725434	0.057982939	-0.23671505	0.812877859	0.907531729	GMPPA	protein_coding
ENSG00000144596.13	141.6518628	0.122376057	0.131460222	0.930897998	0.35190633	0.580628609	GRIP2	protein_coding
ENSG00000144597.14	1656.698664	0.169160351	0.04686271	3.609700556	0.000306551	0.004725348	EAF1	protein_coding
ENSG00000144619.15	328.5461964	0.088084305	0.127555043	0.690559173	0.489842612	0.697132632	CNTN4	protein_coding
ENSG00000144635.9	1999.300741	0.018801699	0.042804796	0.439242823	0.660485599	0.818041751	DYNC1LI1	protein_coding
ENSG00000144642.22	908.341739	0.044217612	0.125833834	0.351396841	0.725290652	0.856716653	RBMS3	protein_coding
ENSG00000144644.14	10.03325953	-0.065829409	0.226162672	-0.291071059	0.770996973	0.884150924	GADL1	protein_coding
ENSG00000144645.14	2575.659081	0.148618444	0.075020222	1.981045106	0.047586215	0.172431774	OSBPL10	protein_coding
ENSG00000144647.6	1383.249769	0.110964715	0.064705741	1.71491298	0.086361183	0.251864627	POMGNT2	protein_coding
ENSG00000144648.16	123.5611041	0.132021943	0.119562639	1.104207333	0.269503209	0.497548778	ACKR2	protein_coding
ENSG00000144649.9	169.3234455	0.167465806	0.166230891	1.007428915	0.313728702	0.5429967	GASK1A	protein_coding
ENSG00000144655.15	2636.242634	0.194706315	0.085391079	2.280171619	0.022597512	0.105562472	CSRNP1	protein_coding
ENSG00000144659.13	1916.193823	-0.140236158	0.048681428	-2.880691137	0.003968043	0.031587368	SLC25A38	protein_coding
ENSG00000144668.12	1638.757352	-0.27494099	0.134519487	-2.043874794	0.040965918	0.156251361	ITGA9	protein_coding
ENSG00000144671.10	11.45617414	0.204377354	0.157887507	1.294449182	0.195510312	0.412559342	SLC22A14	protein_coding
ENSG00000144674.16	9847.965109	0.046076615	0.065261627	0.706029215	0.48016997	0.689289731	GOLGA4	protein_coding
ENSG00000144677.15	5685.023686	0.096186774	0.060793925	1.582177401	0.113609078	0.297299045	CTDSPL	protein_coding
ENSG00000144681.10	124.7544412	0.016145953	0.174416546	0.092571221	0.926244208	0.965089636	STAC	protein_coding
ENSG00000144711.16	5223.634875	0.0392191	0.06920965	0.566670967	0.570937749	0.757370027	IQSEC1	protein_coding
ENSG00000144712.12	315.2335421	-0.078530421	0.160382567	-0.48964437	0.624385574	0.794996066	CAND2	protein_coding
ENSG00000144713.13	44922.4956	-0.19445645	0.074961443	-2.594086261	0.009484269	0.058197224	RPL32	protein_coding
ENSG00000144724.20	3460.843223	0.273222893	0.0772164	3.538404984	0.000402552	0.005775857	PTPRG	protein_coding
ENSG00000144730.19	707.4525191	0.545855255	0.134985418	4.043809067	5.25898E-05	0.001197963	IL17RD	protein_coding
ENSG00000144736.14	1156.745131	-0.032862505	0.043379617	-0.757556375	0.448716605	0.663727259	SHQ1	protein_coding

ENSG00000144741.18	776.071498	-0.031123313	0.042489074	-0.732501569	0.463862485	0.676067277	SLC25A26	protein_coding
ENSG00000144744.17	2603.192126	-0.019406165	0.048268716	-0.402044363	0.687651378	0.835065542	UBA3	protein_coding
ENSG00000144746.7	7819.188381	-0.032529002	0.062073136	-0.524043149	0.600248528	0.778679842	ARL6IP5	protein_coding
ENSG00000144747.17	3178.421162	0.083963816	0.05477452	1.532899161	0.125300703	0.315590174	TMF1	protein_coding
ENSG00000144749.14	5253.795323	-0.186245465	0.098838066	-1.884349544	0.05951771	0.19880448	LRIG1	protein_coding
ENSG00000144771.8	3.125894076	-0.415881277	0.23212384	-1.791635354	0.073191396	0.226239021	LRTM1	protein_coding
ENSG00000144785.8	0						AC073896.1	protein_coding
ENSG00000144791.10	1683.634685	-0.024519947	0.055079879	-0.445170679	0.656196414	0.815319427	LIMD1	protein_coding
ENSG00000144792.9	124.7157269	0.050584698	0.117632405	0.430023499	0.667178547	0.822276634	ZNF660	protein_coding
ENSG00000144802.11	3155.896689	0.001907266	0.104279711	0.0182899	0.985407585	0.993295118	NFKBIZ	protein_coding
ENSG00000144810.16	5017.343632	0.642982946	0.147431585	4.361229297	1.29334E-05	0.00039746	COL8A1	protein_coding
ENSG00000144815.16	1029.494228	-0.166717575	0.085455825	-1.950921127	0.051066429	0.180334018	NXPE3	protein_coding
ENSG00000144820.8	872.8052193	-0.776398055	0.215140969	-3.608787565	0.000307631	0.00473698	ADGRG7	protein_coding
ENSG00000144821.10	56.72031829	-0.022720655	0.13564848	-0.167496569	0.866979347	0.935531904	MYH15	protein_coding
ENSG00000144824.21	2000.073949	0.030877091	0.134289337	0.229929582	0.818146489	0.910265518	PHLDB2	protein_coding
ENSG00000144827.9	1822.424422	-0.043049565	0.05385513	-0.799358672	0.424082467	0.64316442	ABHD10	protein_coding
ENSG00000144834.14	17.72662173	0.118823146	0.248591415	0.477985718	0.63266037	0.800592108	TAGLN3	protein_coding
ENSG00000144837.9	567.6802355	-0.348396568	0.14525769	-2.398472443	0.016463615	0.085520916	PLA1A	protein_coding
ENSG00000144840.10	1627.520111	0.02501449	0.046799095	0.534507987	0.592990125	0.773188435	RABL3	protein_coding
ENSG00000144843.11	600.0308111	0.008027383	0.080788298	0.099363194	0.920849905	0.96273785	ADPRH	protein_coding
ENSG00000144847.13	59.01220768	-0.401601214	0.184976446	-2.171093789	0.029924083	0.127406134	IGSF11	protein_coding
ENSG00000144848.11	3478.108948	-0.038497295	0.050802393	-0.757785072	0.44857966	0.663603078	ATG3	protein_coding
ENSG00000144852.19	825.4705763	-0.391488168	0.203726351	-1.921637363	0.054651404	0.188388576	NR1I2	protein_coding
ENSG00000144857.14	1460.651916	0.21793354	0.170658614	1.277014586	0.201597098	0.420485836	BOC	protein_coding
ENSG00000144867.13	3002.66639	0.04464671	0.061217388	0.729314188	0.465809495	0.677590559	SRPRB	protein_coding
ENSG00000144868.14	121.4746811	0.035052478	0.118941281	0.294704059	0.768219962	0.882522376	TMEM108	protein_coding
ENSG00000144891.18	158.1178534	-0.593567641	0.243270595	-2.439948157	0.01468937	0.07895876	AGTR1	protein_coding
ENSG00000144893.12	104.2869545	-0.049885984	0.16706143	-0.298608628	0.765238682	0.880571972	MED12L	protein_coding
ENSG00000144895.12	6865.445973	-0.007173423	0.05255413	-0.136495885	0.891429277	0.948277162	EIF2A	protein_coding
ENSG00000144908.14	248.0055358	0.981087922	0.216153053	4.538857568	5.65598E-06	0.000206944	ALDH1L1	protein_coding
ENSG00000144909.8	1292.160787	0.031477516	0.044388137	0.709142544	0.478236024	0.687798736	OSBPL11	protein_coding
ENSG00000144935.15	238.8076021	0.065364897	0.122573206	0.533272315	0.593845089	0.773755595	TRPC1	protein_coding
ENSG00000144959.11	4473.855085	0.097927997	0.096309739	1.016802643	0.309247324	0.538599902	NCEH1	protein_coding
ENSG00000144962.7	2.5839095	-0.606393457	0.323729825	-1.873146711	0.061048129	0.201965859	SPATA16	protein_coding
ENSG00000145002.12	15.54468721	0.3910852	0.156395988	2.500608901	0.012398001	0.070128204	FAM86B2	protein_coding
ENSG00000145012.13	31636.2303	-0.227743818	0.11636654	-1.957124594	0.050332822	0.178775153	LPP	protein_coding
ENSG00000145014.18	872.8176627	0.182977079	0.072318649	2.530150704	0.011401354	0.066183138	TMEM44	protein_coding
ENSG00000145016.16	1112.846794	0.065853628	0.06768924	0.972881781	0.330612061	0.560145325	RUBCN	protein_coding
ENSG00000145020.16	17.81718591	-0.082195015	0.094900217	-0.866120405	0.386424136	0.611023788	AMT	protein_coding
ENSG00000145022.4	2084.127689	-0.027912012	0.062276842	-0.448192466	0.6540143	0.814017055	TCTA	protein_coding
ENSG00000145029.14	0.057802159	-0.231515383	1.108722175	-0.208812801	0.834594378		NICN1	protein_coding
ENSG00000145040.4	33.29378479	1.200934838	0.180699566	6.646030558	3.01103E-11	9.79191E-09	UCN2	protein_coding

ENSG00000145041.15	3052.197834	0.073405921	0.049758756	1.475236263	0.140149035	0.337623667	DCAF1	protein_coding
ENSG00000145050.19	3928.092816	-0.124360732	0.068974913	-1.802984973	0.071390575	0.222515347	MANF	protein_coding
ENSG00000145063.15	10.08050205	0.067199714	0.147314934	0.456163624	0.648272321	0.810176306	AC062028.1	lncRNA
ENSG00000145075.13	0.092495958	0.091281058	1.014948281	0.089936659	0.928337549		CCDC39	lncRNA
ENSG00000145087.13	31.42281267	0.085217441	0.190617238	0.447060517	0.654831365	0.814534479	STXBP5L	protein_coding
ENSG00000145088.9	235.9672094	-0.422031738	0.079046555	-5.33902762	9.34464E-08	7.65435E-06	EAF2	protein_coding
ENSG00000145103.15	744.6427875	0.314274724	0.10263052	3.062195589	0.002197198	0.02047195	ILDR1	protein_coding
ENSG00000145107.16	12.07762022	-0.097456009	0.161686295	-0.60274749	0.546676683	0.740474009	TM4SF19	protein_coding
ENSG00000145113.22	7019.242987	1.643431483	0.221020712	7.43564468	1.04059E-13	7.31385E-11	MUC4	protein_coding
ENSG00000145147.20	1036.437211	0.292934871	0.174410862	1.679567824	0.093041432	0.263567997	SLIT2	protein_coding
ENSG00000145191.15	3085.425586	-0.030924002	0.044196043	-0.699700694	0.484114243	0.692134067	EIF2B5	protein_coding
ENSG00000145192.13	23.29619091	-0.717007635	0.297402056	-2.410903422	0.015913062	0.083387763	AHSG	protein_coding
ENSG00000145194.19	1.912790735	-0.177229353	0.281202589	-0.630255054	0.528527724	0.726779825	ECE2	protein_coding
ENSG00000145198.14	100.6651177	-0.161102365	0.182536096	-0.882578123	0.377464253	0.603269998	VWA5B2	protein_coding
ENSG00000145214.14	2128.514903	0.154872693	0.07459234	2.076254653	0.037870405	0.148499832	DGKQ	protein_coding
ENSG00000145216.16	611.8041018	0.020115408	0.044851284	0.44849124	0.653798707	0.813908893	FIP1L1	protein_coding
ENSG00000145217.14	340.6794683	0.084826409	0.097694621	0.868281259	0.385240379	0.609827242	SLC26A1	protein_coding
ENSG00000145220.14	1753.702645	-0.179652932	0.077689665	-2.312443113	0.020753277	0.099644439	LYAR	protein_coding
ENSG00000145241.11	1076.478383	-0.060674578	0.047826006	-1.268652416	0.204565061	0.424027573	CENPC	protein_coding
ENSG00000145242.13	17.10809504	-0.136717513	0.18779289	-0.728022841	0.466599605	0.678196557	EPHA5	protein_coding
ENSG00000145244.12	237.5190388	0.616458896	0.140235713	4.39587666	1.10327E-05	0.000351183	CORIN	protein_coding
ENSG00000145246.14	1210.328973	0.087679047	0.076612776	1.144444204	0.252439465	0.479220783	ATP10D	protein_coding
ENSG00000145247.12	3961.656402	0.258561411	0.084723291	3.051833904	0.002274479	0.020990042	OCIAD2	protein_coding
ENSG00000145248.7	25.57925801	-0.206582939	0.184226432	-1.121353418	0.262137456	0.489592145	SLC10A4	protein_coding
ENSG00000145283.8	37.04259295	0.305728235	0.141569676	2.159560186	0.030806733	0.129794549	SLC10A6	protein_coding
ENSG00000145284.12	1018.861733	-0.030071638	0.119932519	-0.250737981	0.802016694	0.901546754	SCD5	protein_coding
ENSG00000145287.11	4298.083665	0.351852216	0.168152187	2.092462926	0.036397125	0.14447782	PLAC8	protein_coding
ENSG00000145293.16	2717.129195	-0.01102424	0.056093387	-0.196533693	0.844192463	0.923608042	ENOPH1	protein_coding
ENSG00000145309.6	1.89282217	-0.032727603	0.429048327	-0.076279526	0.939196714	0.97113172	CABS1	protein_coding
ENSG00000145321.13	408.2733674	-1.927971272	0.285081667	-6.76287358	1.35281E-11	5.08203E-09	GC	protein_coding
ENSG00000145331.14	381.181899	-0.099912745	0.062287385	-1.604060665	0.108700681	0.289182643	TRMT10A	protein_coding
ENSG00000145332.14	1267.014443	-0.070310621	0.05587589	-1.258335586	0.208270417	0.428204763	KLHL8	protein_coding
ENSG00000145335.17	126.0039387	0.388224094	0.142586352	2.72272969	0.006474501	0.044571773	SNCA	protein_coding
ENSG00000145337.5	0						PYURF	protein_coding
ENSG00000145348.17	1402.842602	-0.000945413	0.048290585	-0.019577575	0.984380353	0.992970894	TBCK	protein_coding
ENSG00000145349.17	3733.743681	-0.115334813	0.061952638	-1.861661062	0.06265088	0.205358721	CAMK2D	protein_coding
ENSG00000145354.12	1542.559337	-0.055519615	0.053631235	-1.035210449	0.30057064	0.530196194	CISD2	protein_coding
ENSG00000145358.6	55.63643923	0.401949261	0.172120841	2.335273624	0.019529131	0.095438034	DDIT4L	protein_coding
ENSG00000145362.20	1124.734972	-0.417627161	0.171047376	-2.441587648	0.014622838	0.078698212	ANK2	protein_coding
ENSG00000145365.11	607.8071745	-0.113489805	0.068313306	-1.66131331	0.096650538	0.269534142	TIFA	protein_coding
ENSG00000145375.9	1056.281809	0.016272101	0.073341561	0.221867398	0.824417111	0.91339275	SPATA5	protein_coding
ENSG00000145384.4	420.6046272	-0.8369801	0.292344158	-2.862995807	0.004196561	0.032778911	FABP2	protein_coding

ENSG00000145386.11	2570.504836	0.001149452	0.093788351	0.012255809	0.990221524	0.995567528	CCNA2	protein_coding
ENSG00000145388.15	1141.488978	-0.092849005	0.039588615	-2.345346158	0.019009422	0.093749838	METTL14	protein_coding
ENSG00000145390.11	2580.382769	0.19457033	0.065200236	2.984196711	0.002843239	0.024622383	USP53	protein_coding
ENSG00000145391.13	6064.783898	0.274379512	0.08033486	3.415447693	0.000636773	0.008155499	SETD7	protein_coding
ENSG00000145414.9	1001.493483	-0.029143138	0.047386467	-0.615009726	0.538548297	0.734446647	NAF1	protein_coding
ENSG00000145416.13	848.1575429	-0.321592497	0.095826537	-3.355985804	0.000790826	0.009607291	MARCHF1	protein_coding
ENSG00000145423.5	10326.95164	0.823629165	0.215987297	3.813322249	0.000137111	0.002530662	SFRP2	protein_coding
ENSG00000145425.10	21293.90444	-0.177503011	0.065723915	-2.700737036	0.006918602	0.046620056	RPS3A	protein_coding
ENSG00000145428.15	38.65708789	0.017674306	0.116748467	0.151387906	0.879669729	0.942030963	RNF175	protein_coding
ENSG00000145431.11	1193.899135	0.569486903	0.093001866	6.12339224	9.16038E-10	1.67924E-07	PDGFC	protein_coding
ENSG00000145439.12	1127.036679	-0.148482515	0.060228378	-2.465324844	0.013688908	0.075289861	CBR4	protein_coding
ENSG00000145451.13	9.946821284	-0.126879724	0.205460843	-0.617537247	0.536880421	0.733083854	GLRA3	protein_coding
ENSG00000145476.16	1908.681651	-0.130417114	0.073338239	-1.77829624	0.075355214	0.230925045	CYP4V2	protein_coding
ENSG00000145491.12	10.97115444	0.178022738	0.136136715	1.307676175	0.190983168	0.406867441	ROPN1L	protein_coding
ENSG00000145494.12	3576.575607	-0.143832693	0.072055269	-1.996144	0.045918251	0.168432628	NDUFS6	protein_coding
ENSG00000145495.16	8329.459055	0.011782814	0.052431777	0.224726574	0.82219199	0.912411743	MARCHF6	protein_coding
ENSG00000145506.14	698.4860242	0.331196749	0.196545008	1.685093673	0.0919705	0.261608421	NKD2	protein_coding
ENSG00000145526.12	11.68305476	-0.074742414	0.257540531	-0.290216122	0.771650903	0.884525122	CDH18	protein_coding
ENSG00000145536.15	140.3255624	1.551380355	0.165795348	9.357200725	8.18771E-21	3.98336E-17	ADAMTS16	protein_coding
ENSG00000145545.12	1584.253214	0.026626448	0.086029958	0.309502041	0.75693966	0.875286177	SRD5A1	protein_coding
ENSG00000145555.15	10552.65275	0.025702438	0.081185319	0.316589729	0.75155493	0.872375074	MYO10	protein_coding
ENSG00000145569.6	1664.962604	0.11264286	0.129249165	0.871517124	0.38347186	0.608364397	OTULINL	protein_coding
ENSG00000145592.14	47873.15064	-0.208643798	0.079815974	-2.614060679	0.008947314	0.055853084	RPL37	protein_coding
ENSG00000145604.17	2555.522638	-0.028607537	0.086705215	-0.329940208	0.741445142	0.866497397	SKP2	protein_coding
ENSG00000145623.13	5935.339179	0.474146756	0.101943401	4.651078455	3.30204E-06	0.000133605	OSMR	protein_coding
ENSG00000145626.12	7.608526676	0.055681322	0.345018518	0.161386474	0.871789019	0.93807366	UGT3A1	protein_coding
ENSG00000145632.15	2715.957837	0.662750919	0.113414102	5.843637671	5.10731E-09	6.95503E-07	PLK2	protein_coding
ENSG00000145642.12	5.005293567	-0.201422075	0.21155687	-0.952094225	0.341049198	0.571040532	SHISAL2B	protein_coding
ENSG00000145649.8	567.4078039	-0.463063452	0.148493234	-3.118414486	0.001818269	0.017841636	GZMA	protein_coding
ENSG00000145675.15	4497.53389	-0.254552317	0.078879344	-3.227109958	0.001250474	0.013609367	PIK3R1	protein_coding
ENSG00000145681.11	182.3013661	-0.121415985	0.177678122	-0.683347975	0.494386986	0.701139118	HAPLN1	protein_coding
ENSG00000145685.14	3876.871032	0.044901581	0.078014231	0.57555629	0.564915133	0.753468007	LHFPL2	protein_coding
ENSG00000145687.16	935.9606482	-0.056442896	0.119405024	-0.472701179	0.636426386	0.802940145	SSBP2	protein_coding
ENSG00000145692.15	29.8811653	-0.0614111	0.218898908	-0.280545485	0.779059034	0.888346648	BHMT	protein_coding
ENSG00000145700.10	27.79348102	-0.330609638	0.134167749	-2.464151333	0.013733811	0.075375099	ANKRD31	protein_coding
ENSG00000145703.16	6384.814549	-0.39630244	0.11825149	-3.351352602	0.000804178	0.009720855	IQGAP2	protein_coding
ENSG00000145708.11	24.11828047	-0.36026299	0.139543524	-2.581724906	0.009830791	0.059715137	CRHBP	protein_coding
ENSG00000145715.15	1807.773236	0.066221056	0.053316347	1.242040389	0.21422166	0.435153279	RASA1	protein_coding
ENSG00000145721.12	30.38848604	-1.127431616	0.229386048	-4.914996472	8.87841E-07	4.74717E-05	LIX1	protein_coding
ENSG00000145723.17	289.8608676	-0.111103234	0.048692839	-2.281716071	0.022506109	0.105287031	GIN1	protein_coding
ENSG00000145725.20	2892.865563	-0.02554486	0.06100265	-0.418750002	0.675398849	0.827668471	PPIP5K2	protein_coding
ENSG00000145730.20	7356.742942	0.168439796	0.083323311	2.021520692	0.043225893	0.162062697	PAM	protein_coding

ENSG00000145734.19	3290.07826	-0.069871666	0.053121524	-1.315317424	0.188403245	0.403769946	BDP1	protein_coding
ENSG00000145736.14	184.5946818	-0.226823314	0.095926256	-2.364559244	0.01805154	0.090625801	GTF2H2	protein_coding
ENSG00000145740.19	2789.460004	0.006093893	0.040929074	0.148889112	0.881641133	0.943220948	SLC30A5	protein_coding
ENSG00000145741.16	20051.88502	-0.122864191	0.059597304	-2.061572971	0.03924841	0.152147963	BTF3	protein_coding
ENSG00000145743.17	1839.469357	0.025713427	0.055378426	0.464322103	0.64241699	0.806621483	FBXL17	protein_coding
ENSG00000145757.16	15.63345358	0.05941869	0.107452765	0.55297498	0.580280543	0.764208203	SPATA9	protein_coding
ENSG00000145777.15	52.40417128	0.155149173	0.150189504	1.033022747	0.301593254	0.531238837	TSLP	protein_coding
ENSG00000145779.8	1552.834368	-0.210153299	0.086471312	-2.430323932	0.015085334	0.080366807	TNFAIP8	protein_coding
ENSG00000145780.8	2606.739124	-0.037471019	0.05768031	-0.649632765	0.515929463	0.717379012	FEM1C	protein_coding
ENSG00000145781.9	904.75816	-0.061105567	0.057482547	-1.063028173	0.287769175	0.517438306	COMMD10	protein_coding
ENSG00000145782.13	2501.533273	0.022851771	0.046522184	0.491201607	0.623283862	0.794501868	ATG12	protein_coding
ENSG00000145794.17	53.17103946	0.725205101	0.171314403	4.233182325	2.30408E-05	0.000622858	MEGF10	protein_coding
ENSG00000145808.10	16.66124327	0.859704745	0.277604299	3.096871149	0.001955749	0.01880116	ADAMTS19	protein_coding
ENSG00000145817.17	2891.338445	0.119398456	0.048812671	2.446054554	0.014442914	0.078051578	YIPF5	protein_coding
ENSG00000145819.18	4515.624777	0.206441422	0.085396845	2.417436166	0.015630275	0.082380322	ARHGAP26	protein_coding
ENSG00000145824.13	6446.811158	0.007199231	0.172446092	0.041747718	0.966699813	0.984499865	CXCL14	protein_coding
ENSG00000145826.9	0.921883532	-0.343957335	0.459199917	-0.749036143	0.453835421		LECT2	protein_coding
ENSG00000145832.14	20.8959367	0.054617364	0.191401008	0.285355674	0.775371664	0.886214564	SLC25A48	protein_coding
ENSG00000145833.16	5565.409816	0.001274938	0.038021653	0.033531886	0.973250439	0.987504293	DDX46	protein_coding
ENSG00000145835.6	0.864338596	-0.286798882	0.28001397	-1.024230617	0.30572636		CDKN2AIPNLP2	processed_pseudogene
ENSG00000145839.2	0.878925833	-0.739764907	0.424045301	-1.744542163	0.081064596		IL9	protein_coding
ENSG00000145850.9	39.62108439	-0.402417132	0.195201796	-2.061544205	0.039251151	0.152147963	TIMD4	protein_coding
ENSG00000145860.12	5583.953294	0.206057816	0.065166434	3.162023817	0.001566767	0.016015723	RNF145	protein_coding
ENSG00000145861.9	112.2671546	0.159896469	0.130909619	1.221426433	0.221924604	0.444447487	C1QTNF2	protein_coding
ENSG00000145863.11	2.672244853	-0.439151428	0.396030252	-1.10888354	0.267480425	0.495447819	GABRA6	protein_coding
ENSG00000145864.14	148.5737118	1.002126847	0.225067025	4.452570728	8.48483E-06	0.000283546	GABRB2	protein_coding
ENSG00000145868.17	2160.898076	-0.012087818	0.043797685	-0.275992178	0.782554074	0.889910698	FBXO38	protein_coding
ENSG00000145879.11	21.61965253	0.333870355	0.295783329	1.128766644	0.258996287	0.486174641	SPINK7	protein_coding
ENSG00000145882.11	621.1188357	-0.08129149	0.065836313	-1.234751563	0.216922979	0.438200105	PCYOX1L	protein_coding
ENSG00000145888.11	4.787362999	-0.221167339	0.210408561	-1.051132798	0.293197603	0.523133844	GLRA1	protein_coding
ENSG00000145901.16	9671.766007	0.042385918	0.060865771	0.696383483	0.486188704	0.693847884	TNIP1	protein_coding
ENSG00000145907.16	11884.17723	-0.041931544	0.04189096	-1.000968808	0.316841889	0.546228547	G3BP1	protein_coding
ENSG00000145908.12	266.8682313	0.01382853	0.137166654	0.100815541	0.91969689	0.962126522	ZNF300	protein_coding
ENSG00000145911.6	568.1581819	0.277414901	0.105334639	2.633653121	0.008447174	0.053667078	N4BP3	protein_coding
ENSG00000145912.9	1753.672319	-0.082354593	0.06762609	-1.217793208	0.223302566	0.445894884	NHP2	protein_coding
ENSG00000145916.19	2085.269709	-0.021420838	0.048800028	-0.438951346	0.660696791	0.818163798	RMND5B	protein_coding
ENSG00000145919.11	1701.410907	-0.062076671	0.046352677	-1.33922514	0.180497391	0.393483218	BOD1	protein_coding
ENSG00000145920.15	77.20666369	-0.778069305	0.231953068	-3.354425583	0.000795299	0.009641597	CPLX2	protein_coding
ENSG00000145934.16	239.2531726	-0.386072811	0.214550216	-1.799451976	0.071947214	0.223706297	TENM2	protein_coding
ENSG00000145936.9	1625.921948	-0.373920797	0.189808831	-1.969986296	0.048839941	0.175255115	KCNMB1	protein_coding
ENSG00000145945.7	614.8473954	0.130159163	0.117207547	1.110501554	0.266782959	0.494853829	FAM50B	protein_coding
ENSG00000145949.11	69.73747444	0.195119646	0.095488898	2.043375209	0.04101531	0.156371316	MYLK4	protein_coding

ENSG00000145975.15	12.04574437	-0.13209835	0.127060114	-1.039652383	0.29850143	0.528299766	FAM217A	protein_coding
ENSG00000145979.18	961.4963246	-0.210538865	0.063805574	-3.299693914	0.000967903	0.011177923	TBC1D7	protein_coding
ENSG00000145982.13	731.8187077	-0.05788471	0.044974781	-1.287048193	0.198077491	0.415520024	FARS2	protein_coding
ENSG00000145990.11	1082.61825	-0.110751534	0.110911581	-0.998556978	0.31800935	0.547375022	GFOD1	protein_coding
ENSG00000145996.11	1095.099436	0.031067313	0.069881836	0.444569213	0.6566311	0.815516853	CDKAL1	protein_coding
ENSG00000146001.5	40.08487479	0.284342744	0.116655603	2.43745468	0.014791068	0.07931848	PCDHB18P	transcribed_unprocessed_pseudogene
ENSG00000146005.4	23.04897444	-0.142036674	0.16186623	-0.877494176	0.380218281	0.605827344	PSD2	protein_coding
ENSG00000146006.8	63.55090828	-0.152055338	0.102240276	-1.487235205	0.136952701	0.333090799	LRRTM2	protein_coding
ENSG00000146007.11	2651.096243	-0.065778257	0.0434398	-1.514239402	0.129965131	0.322667017	ZMAT2	protein_coding
ENSG00000146013.11	203.0196555	0.352541012	0.203943832	1.728618161	0.083877457	0.247302297	GFRA3	protein_coding
ENSG00000146021.15	309.660191	-0.054777733	0.100945678	-0.542645646	0.587373804	0.76938227	KLHL3	protein_coding
ENSG00000146038.12	404.4484987	0.236853667	0.219638074	1.078381645	0.280863475	0.509774986	DCDC2	protein_coding
ENSG00000146039.11	319.9465695	-0.739048024	0.253429955	-2.916182601	0.003543431	0.029055126	SLC17A4	protein_coding
ENSG00000146047.7	0.315547887	-0.142223592	0.629859266	-0.225802174	0.821355286		H2BC1	protein_coding
ENSG00000146049.2	4.031890356	-0.037168169	0.221203507	-0.168027033	0.866562012	0.93530918	KAAG1	protein_coding
ENSG00000146054.18	424.4390355	0.529583979	0.169984859	3.11547736	0.001836474	0.017975752	TRIM7	protein_coding
ENSG00000146063.20	1511.38414	0.001903387	0.048350693	0.039366279	0.968598364	0.985445716	TRIM41	protein_coding
ENSG00000146066.3	3734.183401	-0.209097386	0.074423725	-2.809552802	0.004961038	0.037012011	HIGD2A	protein_coding
ENSG00000146067.16	1508.646956	0.035711796	0.056191765	0.635534343	0.525079976	0.724164006	FAM193B	protein_coding
ENSG00000146070.17	982.8959609	-0.236895468	0.124996508	-1.895216692	0.0580637	0.195757075	PLA2G7	protein_coding
ENSG00000146072.6	13552.1432	0.358341586	0.094281863	3.800747832	0.00014426	0.002638028	TNFRSF21	protein_coding
ENSG00000146083.12	5314.935469	-0.003439224	0.047352622	-0.072630057	0.942100508	0.972703261	RNF44	protein_coding
ENSG00000146085.8	2134.830691	0.11637072	0.053049271	2.193634673	0.028261684	0.122384914	MMUT	protein_coding
ENSG00000146090.16	28.80985559	0.601962436	0.189327273	3.179480842	0.001475391	0.015350873	RASGEF1C	protein_coding
ENSG00000146094.15	917.4888057	-0.183981943	0.096781543	-1.90100238	0.057301701	0.194261414	DOK3	protein_coding
ENSG00000146109.5	1908.575097	-0.044372404	0.050035608	-0.886816512	0.375177696	0.600982152	ABT1	protein_coding
ENSG00000146112.12	4003.890597	0.130619711	0.079768547	1.6374839	0.1015294	0.277977821	PPP1R18	protein_coding
ENSG00000146122.17	1366.262511	-0.007076877	0.139783816	-0.050627299	0.95962251	0.981216155	DAAM2	protein_coding
ENSG00000146143.19	967.9992238	0.012179287	0.0559561	0.217657893	0.827695673	0.915160965	PRIM2	protein_coding
ENSG00000146147.15	19.12773358	0.232270364	0.179496861	1.294007944	0.195662677	0.412680532	MLIP	protein_coding
ENSG00000146151.14	15.39657228	-0.422722754	0.183653495	-2.301740859	0.021349791	0.101556193	HMGCLL1	protein_coding
ENSG00000146166.17	151.3839012	0.202842686	0.315170965	0.643595726	0.519837623	0.720102392	LGSN	protein_coding
ENSG00000146192.15	711.4377892	-0.336995775	0.111066766	-3.034172931	0.002411961	0.021856111	FGD2	protein_coding
ENSG00000146197.9	369.2536504	0.429964224	0.124556338	3.451965847	0.000556518	0.007375728	SCUBE3	protein_coding
ENSG00000146205.14	474.6897894	0.143722704	0.151140516	0.950921089	0.341644435	0.571479947	ANO7	protein_coding
ENSG00000146215.14	33.79218759	-0.182907348	0.174888034	-1.045853988	0.295628451	0.525391967	CRIP3	protein_coding
ENSG00000146216.13	223.9006613	0.272077934	0.159378775	1.707115226	0.087800638	0.254495702	TTBK1	protein_coding
ENSG00000146221.10	10.03609011	0.172962752	0.153935609	1.123604559	0.261180821	0.488370592	TCTE1	protein_coding
ENSG00000146223.16	6782.516165	0.085906478	0.059978513	1.432287568	0.15206154	0.354902561	RPL7L1	protein_coding
ENSG00000146232.17	1612.799461	0.114878255	0.080702073	1.423485797	0.154595376	0.358722189	NFKBIE	protein_coding
ENSG00000146233.8	204.6879932	0.081690465	0.147516125	0.553773122	0.579734125	0.763738377	CYP39A1	protein_coding
ENSG00000146242.9	1779.200976	1.524685444	0.074515573	20.4612993	4.76515E-93	2.07651E-88	TPBG	protein_coding

ENSG00000146243.14	469.7525549	0.051119106	0.076449276	0.668666969	0.503707943	0.708090309	IRAK1BP1	protein_coding
ENSG00000146247.14	6049.23928	0.053380281	0.055451825	0.962642456	0.335726984	0.565213682	PHIP	protein_coding
ENSG00000146250.7	47.44629423	-0.045322978	0.16516521	-0.274409958	0.783769591	0.89030886	PRSS35	protein_coding
ENSG00000146263.11	1233.335842	0.117201533	0.071174527	1.646678084	0.099624231	0.274847366	MMS22L	protein_coding
ENSG00000146267.12	278.0019656	-0.173564474	0.194074076	-0.894320756	0.371150298	0.597652113	FAXC	protein_coding
ENSG00000146276.12	64.69634034	0.897582682	0.269487449	3.330703104	0.000866269	0.010274748	GABRR1	protein_coding
ENSG00000146278.11	8867.619845	-0.046873261	0.059874168	-0.782862834	0.433707665	0.651522669	PNRC1	protein_coding
ENSG00000146281.6	2664.998656	-0.021484584	0.081838122	-0.262525377	0.792916424	0.896174888	PM20D2	protein_coding
ENSG00000146282.18	1765.727422	0.137296397	0.045037893	3.048463999	0.002300145	0.021128833	RARS2	protein_coding
ENSG00000146285.14	264.8797254	-0.797187549	0.139564281	-5.711974047	1.11673E-08	1.31169E-06	SCML4	protein_coding
ENSG00000146350.14	361.2832619	0.017420163	0.076170089	0.228700832	0.819101448	0.91088348	TBC1D32	protein_coding
ENSG00000146352.13	27.83476742	-0.593220049	0.322521487	-1.839319466	0.065868212	0.211505347	CLVS2	protein_coding
ENSG00000146360.8	0.761860735	0.727248486	0.407766835	1.783491014	0.074506409		GPR6	protein_coding
ENSG00000146373.16	684.7626819	0.232622829	0.114136872	2.038104116	0.041539522	0.15780383	RNF217	protein_coding
ENSG00000146374.14	629.9206761	0.164682907	0.171940465	0.957790285	0.338168519	0.567969327	RSPO3	protein_coding
ENSG00000146376.11	2861.615224	0.123284166	0.065514398	1.881787352	0.059864894	0.199668944	ARHGAP18	protein_coding
ENSG00000146378.6	0.505748075	-0.531631235	0.523332227	-1.01585801	0.309697007		TAAR2	protein_coding
ENSG00000146383.7	0.7722599	-0.883439492	0.435093876	-2.030457199	0.042310088		TAAR6	protein_coding
ENSG00000146385.1	0.583603427	0.011318115	0.434916405	0.026023657	0.979238469		TAAR8	protein_coding
ENSG00000146386.8	2168.63953	-0.030410288	0.085409936	-0.356050939	0.721802417	0.854960151	ABRACL	protein_coding
ENSG00000146399.2	18.82306382	1.844418774	0.29322549	6.290103819	3.17254E-10	7.01775E-08	TAAR1	protein_coding
ENSG00000146409.12	1755.183175	0.077181144	0.073153865	1.055052169	0.291401466	0.521366467	SLC18B1	protein_coding
ENSG00000146410.12	454.4476336	-0.031037133	0.096138737	-0.322836916	0.746818754	0.869666255	MTFR2	protein_coding
ENSG00000146411.6	424.5575112	0.284805015	0.156233839	1.822940649	0.06831238	0.216576835	SLC2A12	protein_coding
ENSG00000146414.16	861.9390644	0.124181698	0.073526446	1.688939203	0.091231082	0.260298797	SHPRH	protein_coding
ENSG00000146416.19	2469.728361	0.057942638	0.078537112	0.737773985	0.460651805	0.673731287	AIG1	protein_coding
ENSG00000146425.11	4402.782106	0.073225811	0.058401645	1.253831298	0.209903331	0.430184258	DYNLT1	protein_coding
ENSG00000146426.18	547.6680032	0.010819749	0.0839194	0.12893025	0.89741284	0.951262658	TIAM2	protein_coding
ENSG00000146433.9	5081.037127	0.224626211	0.06611968	3.397267082	0.000680625	0.008561817	TMEM181	protein_coding
ENSG00000146453.13	16.23120664	-0.245948407	0.159761029	-1.539476859	0.123687923	0.313110303	PNLDC1	protein_coding
ENSG00000146457.16	3670.364212	0.052260845	0.036697082	1.424114462	0.154413341	0.358509784	WTAP	protein_coding
ENSG00000146463.12	3456.283845	0.061444908	0.041090365	1.495360474	0.13482039	0.33007151	ZMYM4	protein_coding
ENSG00000146469.13	102.3684737	0.074222371	0.259111996	0.286448995	0.774534254	0.885899346	VIP	protein_coding
ENSG00000146476.11	1555.716471	-0.070746875	0.053652898	-1.318603049	0.187301854	0.402349053	ARMT1	protein_coding
ENSG00000146477.6	941.6688183	0.049659317	0.161708052	0.307092418	0.758773029	0.876568815	SLC22A3	protein_coding
ENSG00000146521.10	80.98869772	-0.064802279	0.166109696	-0.390117377	0.696449754	0.839906762	LINC01558	lncRNA
ENSG00000146530.14	42.70789359	1.423948232	0.243969581	5.836581036	5.32829E-09	7.19611E-07	VWDE	protein_coding
ENSG00000146535.14	5085.656595	0.122185607	0.051539438	2.370720614	0.017753445	0.089635255	GNA12	protein_coding
ENSG00000146540.15	3040.520258	-0.082886914	0.073380164	-1.129554765	0.258663881	0.485826455	C7orf50	protein_coding
ENSG00000146555.19	1152.129951	0.543214402	0.14130737	3.844204316	0.000120944	0.002292395	SDK1	protein_coding
ENSG00000146556.14	226.094521	-0.107814582	0.066504	-1.621174391	0.104980243	0.283633459	WASH2P	transcribed_unprocessed_pseudogene
ENSG00000146574.16	560.3380879	0.024852731	0.059843428	0.415295914	0.677925292	0.829340851	CCZ1B	protein_coding

ENSG00000146576.13	2396.189727	-0.07367226	0.046934934	-1.569667919	0.116492389	0.301663229	C7orf26	protein_coding
ENSG00000146587.18	1450.971846	0.075888129	0.054133716	1.401864404	0.160955742	0.367281163	RBAK	protein_coding
ENSG00000146592.17	589.3798715	0.087019817	0.124076112	0.701342225	0.483089468	0.691213218	CREB5	protein_coding
ENSG00000146618.3	0.016586131	-0.076814322	2.893054209	-0.02655129	0.978817624		FERD3L	protein_coding
ENSG00000146648.19	8154.472404	0.306374077	0.119716035	2.559173274	0.010492143	0.062350487	EGFR	protein_coding
ENSG00000146666.5	18.71161841	-0.394274975	0.152243776	-2.589760872	0.009604262	0.058707383	LINC00525	lncRNA
ENSG00000146670.10	1770.596788	0.050119079	0.094199183	0.532054284	0.594688399	0.774492075	CDCA5	protein_coding
ENSG00000146674.15	15547.77558	0.495357415	0.109813219	4.510908771	6.45505E-06	0.000230756	IGFBP3	protein_coding
ENSG00000146676.10	5129.983055	-0.026190892	0.049012789	-0.534368528	0.593086589	0.773244802	PURB	protein_coding
ENSG00000146677.7	576.2945505	-0.216183822	0.092254431	-2.34334351	0.019111778	0.094041773	AC004453.1	processed_pseudogene
ENSG00000146678.10	102.0750369	0.891784608	0.258760345	3.446372774	0.000568166	0.007502718	IGFBP1	protein_coding
ENSG00000146700.9	75.65411782	0.10096644	0.101246935	0.997229589	0.318653082	0.54811985	SSC4D	protein_coding
ENSG00000146701.12	18126.43803	-0.163785605	0.064499443	-2.539333656	0.011106385	0.065033805	MDH2	protein_coding
ENSG00000146707.14	218.6079173	-0.206792737	0.087993137	-2.350100737	0.018768331	0.09293764	POMZP3	protein_coding
ENSG00000146722.11	2.676909729	-0.18338563	0.175478434	-1.045060788	0.295994875	0.525742589	AC211486.1	transcribed_unprocessed_pseudogene
ENSG00000146729.10	4893.526764	-0.284306145	0.063226762	-4.496610894	6.90452E-06	0.000242771	NIPSNAP2	protein_coding
ENSG00000146731.11	12404.82625	-0.233739054	0.067142106	-3.481258873	0.000499063	0.006785543	CCT6A	protein_coding
ENSG00000146733.14	1154.565123	-0.401959133	0.088653769	-4.534033238	5.78679E-06	0.000211199	PSPH	protein_coding
ENSG00000146755.11	25.62087933	-0.535041528	0.220666555	-2.424660722	0.015322699	0.081242031	TRIM50	protein_coding
ENSG00000146757.14	945.6553351	-0.099398813	0.066950517	-1.484660881	0.137633679	0.334080071	ZNF92	protein_coding
ENSG00000146776.14	969.3072603	-0.026894865	0.054004216	-0.49801417	0.618474054	0.79103771	ATXN7L1	protein_coding
ENSG00000146802.13	2932.42639	-0.121185243	0.067607652	-1.792478214	0.073056396	0.225978035	TMEM168	protein_coding
ENSG00000146809.13	9.028657953	-0.359863996	0.253793038	-1.41794274	0.156207483	0.360982844	ASB15	protein_coding
ENSG00000146826.17	1255.701732	0.048862215	0.076292137	0.640462006	0.521872282	0.721621061	MAP11	protein_coding
ENSG00000146828.18	3650.604847	-0.041710513	0.066863105	-0.62381956	0.532746093	0.730184201	SLC12A9	protein_coding
ENSG00000146830.11	3826.783528	0.052798993	0.066648297	0.792203179	0.428242215	0.646398026	GIGYF1	protein_coding
ENSG00000146833.16	2271.246819	0.044897021	0.057502096	0.780789293	0.434926433	0.652666729	TRIM4	protein_coding
ENSG00000146834.14	3866.210661	0.046327203	0.060014197	0.7719374	0.440151505	0.656602402	MEPCE	protein_coding
ENSG00000146839.19	36.23878443	0.17288097	0.236269808	0.731709954	0.46434562	0.676384385	ZAN	protein_coding
ENSG00000146842.17	1594.474621	-0.132451949	0.055388733	-2.391315713	0.01678811	0.086525778	TMEM209	protein_coding
ENSG00000146856.14	118.6107177	-0.311904921	0.079871005	-3.90510825	9.41832E-05	0.001889605	AGBL3	protein_coding
ENSG00000146857.4	4.441224511	0.506050878	0.33595815	1.506291417	0.131992381	0.325642981	STRA8	protein_coding
ENSG00000146858.8	586.609207	0.186100016	0.080540963	2.310625661	0.020853541	0.099980997	ZC3HAV1L	protein_coding
ENSG00000146859.6	1188.287573	0.017977734	0.067060939	0.268080555	0.788637313	0.893633101	TMEM140	protein_coding
ENSG00000146872.18	2013.210797	0.032989646	0.042059178	0.78436258	0.432827389	0.650817523	TLK2	protein_coding
ENSG00000146904.9	2241.505887	-0.039174906	0.106779281	-0.366877409	0.713710463	0.850437802	EPHA1	protein_coding
ENSG00000146909.8	1866.637738	-0.171948699	0.05081507	-3.383813097	0.000714866	0.008891296	NOM1	protein_coding
ENSG00000146910.12	1.60152381	0.04848273	0.312450666	0.155169233	0.876687903	0.940438391	CNPY1	protein_coding
ENSG00000146918.20	2172.606272	-0.121450001	0.079190679	-1.533640102	0.125118217	0.315288067	NCAPG2	protein_coding
ENSG00000146926.11	1.217387885	0.369683733	0.355694261	1.039330047	0.298651265	0.528500556	ASB10	protein_coding
ENSG00000146938.16	297.1330108	-0.225937499	0.180322971	-1.25296016	0.210220206	0.430588292	NLGN4X	protein_coding
ENSG00000146950.13	684.087602	0.32495981	0.153384173	2.118600663	0.03412423	0.138496	SHROOM2	protein_coding

ENSG00000146955.11	354.5138873	-0.04765257	0.101412935	-0.46988651	0.638436102	0.804311363	RAB19	protein_coding
ENSG00000146963.18	1898.820182	-0.214752979	0.038481541	-5.58067512	2.39587E-08	2.4682E-06	LUC7L2	protein_coding
ENSG00000146966.13	443.8873277	0.135556231	0.09707522	1.396404055	0.162592855	0.369623289	DENND2A	protein_coding
ENSG00000147003.7	41.68973906	-0.395686908	0.137997216	-2.867354276	0.004139194	0.032493901	CLTRN	protein_coding
ENSG00000147010.18	4964.142564	-0.064726987	0.072125672	-0.897419534	0.369495093	0.596255651	SH3KBP1	protein_coding
ENSG00000147027.4	2130.814907	0.062599508	0.12257715	0.510694756	0.609564813	0.785214043	TMEM47	protein_coding
ENSG00000147036.12	102.8270442	0.325043519	0.139494089	2.330159807	0.019797706	0.096493194	LANCL3	protein_coding
ENSG00000147041.12	417.401018	0.095394939	0.201571692	0.473255638	0.636030808	0.802650213	SYTL5	protein_coding
ENSG00000147044.22	6671.47503	-0.125700718	0.067832716	-1.853098694	0.063868214	0.207853002	CASK	protein_coding
ENSG00000147050.16	2720.948245	-0.06415649	0.059448474	-1.079194894	0.280500855	0.509393067	KDM6A	protein_coding
ENSG00000147059.8	15.53187189	0.100618268	0.106608541	0.943810573	0.345266457	0.574745068	SPIN2A	protein_coding
ENSG00000147065.17	20094.61889	-0.075252164	0.08566717	-0.878424773	0.379713246	0.605243941	MSN	protein_coding
ENSG00000147081.15	5.685417577	-0.211717426	0.272438114	-0.777121172	0.437087284	0.654103509	AKAP4	protein_coding
ENSG00000147082.18	83.89520388	0.21324371	0.142949556	1.491740976	0.135767063	0.331450031	CCNB3	protein_coding
ENSG00000147099.21	568.1394079	-0.107629599	0.059604083	-1.805742048	0.070958641	0.221692337	HDAC8	protein_coding
ENSG00000147100.11	1550.813699	-0.17302389	0.138780556	-1.24674446	0.212491211	0.433122667	SLC16A2	protein_coding
ENSG00000147113.17	968.1416923	0.030222944	0.096183902	0.314220396	0.753353653	0.87330756	DIPK2B	protein_coding
ENSG00000147117.8	8.626463358	-0.397970528	0.182694811	-2.178335145	0.029381094	0.125769691	ZNF157	protein_coding
ENSG00000147118.12	459.0869279	-0.069964488	0.066924022	-1.045431612	0.295823533	0.525523933	ZNF182	protein_coding
ENSG00000147119.4	279.264695	0.29036324	0.089347106	3.249833729	0.001154725	0.012810601	CHST7	protein_coding
ENSG00000147121.16	488.1910867	-0.086806782	0.056322965	-1.541232462	0.123260219	0.312358141	KRBOX4	protein_coding
ENSG00000147123.11	5235.320509	-0.195774056	0.068018395	-2.8782516	0.003998861	0.031781571	NDUFB11	protein_coding
ENSG00000147124.12	899.6653526	0.018279726	0.048433335	0.377420335	0.705861257	0.845813279	ZNF41	protein_coding
ENSG00000147127.8	22.79420327	-0.028240161	0.083776474	-0.337089403	0.736049506	0.863108264	RAB41	protein_coding
ENSG00000147130.14	3023.472522	0.033679382	0.058109849	0.579581298	0.562197008	0.751084033	ZMYM3	protein_coding
ENSG00000147133.16	3155.351506	-0.00695145	0.05015825	-0.13859037	0.889773853	0.947363057	TAF1	protein_coding
ENSG00000147138.3	371.4050523	-0.839391698	0.165811609	-5.062321644	4.14181E-07	2.56375E-05	GPR174	protein_coding
ENSG00000147140.17	21058.81078	0.036648617	0.044169728	0.829722491	0.406695702	0.627931636	NONO	protein_coding
ENSG00000147144.13	1945.997356	0.236849895	0.065509402	3.615509981	0.000299757	0.004640322	CCDC120	protein_coding
ENSG00000147145.12	41.45653865	0.015050819	0.124663601	0.120731464	0.903903737	0.954171989	LPAR4	protein_coding
ENSG00000147155.11	1114.442583	-0.197342463	0.092769764	-2.127228266	0.033401122	0.136586849	EBP	protein_coding
ENSG00000147160.9	1.890759309	-0.407662555	0.284536372	-1.432725638	0.15193626	0.354716241	AWAT2	protein_coding
ENSG00000147162.14	9367.544417	0.009283418	0.070783474	0.131152331	0.895654805	0.950498453	OGT	protein_coding
ENSG00000147164.12	3120.226423	-0.033413695	0.047631857	-0.7014989	0.482991721	0.691186832	SNX12	protein_coding
ENSG00000147166.11	73.85650653	-0.297248509	0.126717447	-2.34575835	0.018988414	0.093688972	ITGB1BP2	protein_coding
ENSG00000147168.13	2637.732119	-0.334154496	0.135484906	-2.466359576	0.013649424	0.075158066	IL2RG	protein_coding
ENSG00000147174.11	68.46320738	-0.029419239	0.103668817	-0.283780988	0.776578225	0.886747617	GCNA	protein_coding
ENSG00000147180.17	760.2210996	-0.125535859	0.10226685	-1.227532272	0.2196226	0.441463814	ZNF711	protein_coding
ENSG00000147183.10	0.555673985	0.665487509	0.484344145	1.373997221	0.169442545		CPXCR1	protein_coding
ENSG00000147202.18	2741.160812	-0.029883439	0.062973342	-0.474541106	0.635114093	0.802108694	DIAPH2	protein_coding
ENSG00000147206.17	250.9035633	0.144660415	0.252635235	0.572605857	0.566911593	0.754652569	NXF3	protein_coding
ENSG00000147223.6	4.860553219	-0.160401151	0.183774696	-0.872814124	0.3827644	0.607883824	RIPPLY1	protein_coding

ENSG00000147224.13	2046.223772	-0.013375442	0.061125653	-0.218818798	0.826791203	0.914652592	PRPS1	protein_coding
ENSG00000147231.14	170.418144	-0.166183438	0.132005305	-1.258914847	0.20806109	0.428036925	RADX	protein_coding
ENSG00000147234.10	136.8958023	-0.109422172	0.175098896	-0.624916399	0.532025929	0.729546659	FRMPD3	protein_coding
ENSG00000147246.10	36.10034444	0.836027465	0.368513518	2.268648028	0.023289738	0.107720193	HTR2C	protein_coding
ENSG00000147251.15	1717.769074	-0.16352039	0.108296775	-1.50992852	0.131061664	0.324283111	DOCK11	protein_coding
ENSG00000147255.19	118.1285225	0.437054839	0.194114102	2.251535748	0.024351625	0.111001127	IGSF1	protein_coding
ENSG00000147256.12	4.206962898	-0.086527032	0.278368805	-0.310835949	0.755925339	0.874745775	ARHGAP36	protein_coding
ENSG00000147257.15	1901.582748	-0.667615429	0.208998078	-3.19436158	0.001401405	0.014851414	GPC3	protein_coding
ENSG00000147262.4	1.625773166	-0.253002644	0.359824502	-0.703127894	0.48197605	0.690666738	GPR119	protein_coding
ENSG00000147274.14	10857.14115	-0.084645551	0.03861103	-2.192263469	0.028360488	0.122703036	RBMX	protein_coding
ENSG00000147316.13	1251.488239	0.042690836	0.056586796	0.75443105	0.450590433	0.665357978	MCPH1	protein_coding
ENSG00000147324.11	5071.607151	-0.034479906	0.074154637	-0.464973026	0.641950773	0.806344315	MFHAS1	protein_coding
ENSG00000147364.17	1895.735588	0.010648819	0.067066314	0.158780446	0.873841856	0.939003441	FBXO25	protein_coding
ENSG00000147378.11	10.27887479	-0.385983683	0.153634288	-2.512353774	0.01199288	0.068476639	FATE1	protein_coding
ENSG00000147381.11	363.0671619	1.364277043	0.50356008	2.709263696	0.006743272	0.045849831	MAGEA4	protein_coding
ENSG00000147383.11	1612.042645	0.031140128	0.067515655	0.461228251	0.644634855	0.808125574	NSDHL	protein_coding
ENSG00000147394.18	2552.255143	1.081883025	0.145405187	7.440470621	1.00327E-13	7.16715E-11	ZNF185	protein_coding
ENSG00000147400.10	2392.917727	-0.037413859	0.064022841	-0.584382981	0.558962674	0.748866368	CETN2	protein_coding
ENSG00000147403.18	77075.71764	-0.196750123	0.071101024	-2.767191142	0.005654159	0.040624335	RPL10	protein_coding
ENSG00000147408.14	1280.122946	0.347375605	0.097639049	3.557752855	0.000374041	0.005470871	CSGALNACT1	protein_coding
ENSG00000147416.11	5396.920571	-0.0437361	0.056904413	-0.768588902	0.442137396	0.658521475	ATP6V1B2	protein_coding
ENSG00000147419.19	2373.038957	-0.220418911	0.061470394	-3.585773504	0.000336081	0.005057107	CCDC25	protein_coding
ENSG00000147421.18	1168.54416	-0.065811263	0.059407085	-1.107801571	0.267947522	0.495832059	HMBOX1	protein_coding
ENSG00000147432.7	3.347967961	-0.003062257	0.294574934	-0.010395512	0.99170573	0.996255259	CHRNB3	protein_coding
ENSG00000147434.9	15.09452376	-0.241327515	0.147347008	-1.637817541	0.101459761	0.277842771	CHRNA6	protein_coding
ENSG00000147437.10	68.41872051	-0.121346328	0.099044344	-1.225171711	0.220510531	0.44253419	GNRH1	protein_coding
ENSG00000147439.13	798.1242134	-0.097696402	0.059183803	-1.65072869	0.098793987	0.273550995	BIN3	protein_coding
ENSG00000147443.13	520.4883461	-0.150157777	0.109769787	-1.367933568	0.171332871	0.381765738	DOK2	protein_coding
ENSG00000147454.14	3081.95164	0.065154162	0.073738532	0.883583656	0.376921005	0.602674359	SLC25A37	protein_coding
ENSG00000147457.14	2671.899475	-0.07969355	0.051663026	-1.542564502	0.122936476	0.312064326	CHMP7	protein_coding
ENSG00000147459.18	4703.33869	0.239427716	0.084854109	2.821639635	0.004777883	0.036102968	DOCK5	protein_coding
ENSG00000147465.12	40.20789737	-0.356628532	0.106268496	-3.355919638	0.000791016	0.009607291	STAR	protein_coding
ENSG00000147471.12	4469.388747	-0.118109759	0.067992237	-1.737106543	0.082368367	0.244523899	PLPBP	protein_coding
ENSG00000147475.17	4494.839851	0.04611396	0.071290347	0.646847183	0.517730852	0.718846984	ERLIN2	protein_coding
ENSG00000147481.17	10.46980916	-0.244187669	0.265255638	-0.920574851	0.357272437	0.585024644	SNTG1	protein_coding
ENSG00000147485.13	44.389896	-0.053127515	0.111658211	-0.475804821	0.634213435	0.80149408	PXDNL	protein_coding
ENSG00000147488.11	73.63917628	-0.446594245	0.175165861	-2.549550704	0.010786183	0.063681002	ST18	protein_coding
ENSG00000147509.14	21.96881017	0.795487934	0.16403153	4.849603819	1.23708E-06	6.17507E-05	RGS20	protein_coding
ENSG00000147526.20	10386.34234	-0.15809408	0.095143835	-1.661632407	0.096586501	0.269390039	TACC1	protein_coding
ENSG00000147533.17	1018.963787	0.141836567	0.06027351	2.353215658	0.018611836	0.092469272	GOLGA7	protein_coding
ENSG00000147535.17	3080.358818	-0.097347897	0.071119347	-1.368796257	0.171062971	0.381338812	PLPP5	protein_coding
ENSG00000147536.12	420.8428834	0.008203567	0.089377239	0.091785866	0.926868174	0.965393528	GINS4	protein_coding

ENSG00000147548.17	5223.114935	0.00311563	0.059046405	0.052765792	0.957918518	0.980370963	NSD3	protein_coding
ENSG00000147570.10	76.92435508	-0.282529509	0.141026563	-2.003377965	0.045136735	0.166617831	DNAJC5B	protein_coding
ENSG00000147571.5	2.314356911	0.799378313	0.449510614	1.778330229	0.075349634	0.230924187	CRH	protein_coding
ENSG00000147573.17	11.41282286	-0.750182778	0.189403398	-3.960767263	7.47093E-05	0.001583467	TRIM55	protein_coding
ENSG00000147576.17	40.68748881	-0.557344798	0.147374473	-3.78182726	0.000155681	0.002810326	ADHFE1	protein_coding
ENSG00000147586.10	246.8695737	-0.138588558	0.066429276	-2.08625724	0.036955328	0.145975554	MRPS28	protein_coding
ENSG00000147588.7	19.25046288	-0.273995883	0.243309103	-1.126122614	0.260113623	0.487337003	PMP2	protein_coding
ENSG00000147592.9	2229.687986	-0.403915992	0.102904561	-3.925151516	8.66752E-05	0.001778269	LACTB2	protein_coding
ENSG00000147596.4	5.361339166	0.073975444	0.2725415	0.271428184	0.786061728	0.891735764	PRDM14	protein_coding
ENSG00000147601.15	2023.020439	-0.064053094	0.045159324	-1.418380248	0.156079779	0.360799	TERF1	protein_coding
ENSG00000147604.14	46530.18306	-0.211616602	0.066779059	-3.168906614	0.001530136	0.015737247	RPL7	protein_coding
ENSG00000147606.9	49.38464878	-0.346077514	0.188147832	-1.839391455	0.06585763	0.211493855	SLC26A7	protein_coding
ENSG00000147613.8	1.272953081	-0.255083992	0.378702778	-0.673573068	0.50058278	0.705859948	PSKH2	protein_coding
ENSG00000147614.4	85.72900218	-0.098470941	0.160287569	-0.614339229	0.538991185	0.734768781	ATP6V0D2	protein_coding
ENSG00000147642.17	1553.646125	-0.368648684	0.117639723	-3.133709209	0.001726118	0.017224423	SYBU	protein_coding
ENSG00000147647.13	11.87010694	0.479429824	0.212914324	2.251749981	0.024338076	0.110962581	DPYS	protein_coding
ENSG00000147649.10	17275.50194	-0.010097243	0.050085233	-0.201601204	0.840228504	0.921597797	MTDH	protein_coding
ENSG00000147650.11	596.4577894	0.369087172	0.091537028	4.032107882	5.52788E-05	0.001242835	LRP12	protein_coding
ENSG00000147654.15	1842.990402	-0.174719952	0.056796104	-3.076266488	0.002096104	0.019822599	EBAG9	protein_coding
ENSG00000147655.12	106.6351101	-0.3867697	0.20671141	-1.871061207	0.061336598	0.202643285	RSPO2	protein_coding
ENSG00000147669.11	4254.782429	-0.075895456	0.0662411	-1.145745694	0.251900391	0.478616235	POLR2K	protein_coding
ENSG00000147676.14	19756.67706	0.235817215	0.102798285	2.293980056	0.021791644	0.102905774	MAL2	protein_coding
ENSG00000147677.11	17774.82248	-0.094303469	0.051869895	-1.818077103	0.069052349	0.217892412	EIF3H	protein_coding
ENSG00000147679.12	1384.068378	0.01446044	0.048746094	0.296648178	0.766735125	0.881606811	UTP23	protein_coding
ENSG00000147684.10	6137.5591	-0.185067916	0.075826408	-2.440678914	0.014659682	0.078847813	NDUFB9	protein_coding
ENSG00000147687.19	1023.584756	-0.105095192	0.06289598	-1.67093657	0.094734207	0.266183026	TATDN1	protein_coding
ENSG00000147689.16	1087.289675	1.753967824	0.237640811	7.380751728	1.57398E-13	1.07171E-10	FAM83A	protein_coding
ENSG00000147697.9	58.3886658	0.417282573	0.16649454	2.506283832	0.012200764	0.069300401	GSDMC	protein_coding
ENSG00000147724.12	29.12291221	-0.923908371	0.213934176	-4.318657211	1.56981E-05	0.000460969	FAM135B	protein_coding
ENSG00000147753.9	0.065997867	-0.169878422	1.303956063	-0.130279253	0.896345494		TTTY7	lncRNA
ENSG00000147761.8	0.027187829	-0.107832404	1.808139191	-0.059637225	0.95244457		TTTY7B	lncRNA
ENSG00000147789.15	1209.700513	-0.072651145	0.049766586	-1.459837844	0.144334645	0.34397981	ZNF7	protein_coding
ENSG00000147799.11	1524.643917	0.21782599	0.088225266	2.468975146	0.013550063	0.074752639	ARHGAP39	protein_coding
ENSG00000147804.10	6189.394928	-0.009370354	0.127877555	-0.07327599	0.941586497	0.972345757	SLC39A4	protein_coding
ENSG00000147813.16	3120.059035	-0.007848477	0.100144115	-0.078371819	0.937532289	0.970400812	NAPRT	protein_coding
ENSG00000147852.16	418.7946475	0.504642711	0.139707365	3.612141076	0.000303679	0.004688348	VLDLR	protein_coding
ENSG00000147853.17	5534.814843	-0.004389834	0.065027679	-0.067507157	0.946177965	0.97474225	AK3	protein_coding
ENSG00000147854.17	2112.110171	0.138812686	0.065211028	2.128668884	0.033281665	0.136306744	UHRF2	protein_coding
ENSG00000147862.17	7018.546663	-0.037708338	0.094289867	-0.399919308	0.689215951	0.835787737	NFIB	protein_coding
ENSG00000147869.5	2.176304928	-1.205321441	0.35669228	-3.379163237	0.000727068	0.009013784	CER1	protein_coding
ENSG00000147872.10	3092.448561	-0.008338321	0.10817532	-0.077081544	0.938558675	0.970886407	PLIN2	protein_coding
ENSG00000147873.5	0.555656036	-0.075131518	0.338181309	-0.222163426	0.824186665		IFNA5	protein_coding

ENSG00000147874.11	1567.628946	-0.020610954	0.071461743	-0.28841942	0.773025704	0.885219454	HAUS6	protein_coding
ENSG00000147883.12	1560.436133	0.489612202	0.121630979	4.025390617	5.68808E-05	0.001271126	CDKN2B	protein_coding
ENSG00000147885.4	0.159288813	-0.031993597	0.930094525	-0.034398221	0.972559602		IFNA16	protein_coding
ENSG00000147889.18	1755.934499	0.517815993	0.201218942	2.57339587	0.010070595	0.060784119	CDKN2A	protein_coding
ENSG00000147894.17	921.2069224	-0.015630119	0.068939369	-0.226722689	0.820639383	0.911786963	C9orf72	protein_coding
ENSG00000147896.3	4.436633205	-0.493988348	0.183951216	-2.685431271	0.007243627	0.048176975	IFNK	protein_coding
ENSG00000147905.18	1409.635719	0.048622252	0.054608096	0.890385407	0.373258975	0.59941207	ZCCHC7	protein_coding
ENSG00000147912.13	215.4855841	-0.097706077	0.070602683	-1.383886181	0.166393309	0.374833199	FBXO10	protein_coding
ENSG00000147955.17	9037.691035	-0.071609895	0.086736641	-0.825601427	0.409030225	0.629903211	SIGMAR1	protein_coding
ENSG00000147996.16	418.4535608	-0.040644265	0.073056524	-0.55633998	0.577978461	0.762358646	CBWD5	protein_coding
ENSG00000148019.14	1703.978503	-0.023061382	0.059742351	-0.386013962	0.699486328	0.841821528	CEP78	protein_coding
ENSG00000148053.17	778.8401198	0.085364733	0.194434766	0.439040481	0.660632204	0.818107067	NTRK2	protein_coding
ENSG00000148057.16	297.8614164	-0.353608542	0.075101508	-4.708408031	2.49659E-06	0.000107823	IDNK	protein_coding
ENSG00000148082.10	265.6780994	-0.015956722	0.147654334	-0.108068094	0.913941678	0.959312951	SHC3	protein_coding
ENSG00000148090.12	793.5987927	-0.139200199	0.051808303	-2.686831875	0.007213325	0.048044988	AUH	protein_coding
ENSG00000148110.16	4782.589444	0.039101132	0.045115685	0.866685988	0.386114085	0.610688642	MFSD14B	protein_coding
ENSG00000148120.17	2260.699072	0.199308367	0.078359436	2.54351458	0.010974348	0.06442532	AOPEP	protein_coding
ENSG00000148123.15	50.30980005	0.274167568	0.233236259	1.175492905	0.239797574	0.465304843	PLPPR1	protein_coding
ENSG00000148136.5	0.216287052	-0.140413012	0.907986347	-0.154642207	0.877103394		OR13C4	protein_coding
ENSG00000148143.13	1834.148614	0.067834387	0.096991511	0.699384789	0.484311592	0.69225233	ZNF462	protein_coding
ENSG00000148153.14	1665.858268	-0.040395706	0.041813308	-0.966096863	0.333995714	0.563517548	INIP	protein_coding
ENSG00000148154.10	3805.619637	0.019567972	0.066477987	0.294352659	0.768488438	0.882594308	UGCG	protein_coding
ENSG00000148156.8	1.141911716	0.207827285	0.276383702	0.751952026	0.452079909	0.666608676	ACTL7B	protein_coding
ENSG00000148158.17	1788.797463	0.097791942	0.067037907	1.458755901	0.144632304	0.344481959	SNX30	protein_coding
ENSG00000148175.13	10703.35415	-0.022551179	0.087817614	-0.256795628	0.797336546	0.898572557	STOM	protein_coding
ENSG00000148180.21	26667.37939	0.183932156	0.097154274	1.893196747	0.05833171	0.196308473	GSN	protein_coding
ENSG00000148187.18	2846.685916	-0.074727994	0.058190509	-1.28419557	0.199073535	0.417148847	MRRF	protein_coding
ENSG00000148200.17	460.726675	-0.152787265	0.100408536	-1.521656142	0.128095262	0.319629366	NR6A1	protein_coding
ENSG00000148204.12	77.69887838	-0.104431301	0.159674974	-0.654024222	0.5130962	0.715539211	CRB2	protein_coding
ENSG00000148215.4	2.825376837	0.129141284	0.266390241	0.484782337	0.627830753	0.797266019	OR5C1	protein_coding
ENSG00000148218.16	3345.441986	-0.182574777	0.05324371	-3.429039378	0.000605722	0.007857725	ALAD	protein_coding
ENSG00000148219.17	262.8625878	0.106849014	0.109876741	0.972444338	0.330829543	0.560388673	ASTN2	protein_coding
ENSG00000148225.16	309.4223802	-0.007682057	0.07834513	-0.098054043	0.92188938	0.963172363	WDR31	protein_coding
ENSG00000148229.13	3729.48844	-0.039091067	0.050979632	-0.766797761	0.443201768	0.659365953	POLE3	protein_coding
ENSG00000148248.14	20144.96968	0.044034595	0.051450621	0.855861288	0.39207451	0.61600198	SURF4	protein_coding
ENSG00000148288.13	271.107499	0.044921208	0.113337663	0.396348456	0.691847985	0.837532562	GBGT1	protein_coding
ENSG00000148290.10	1852.211359	-0.166791099	0.054341591	-3.069308335	0.00214555	0.020124116	SURF1	protein_coding
ENSG00000148291.10	742.4670369	-0.179599432	0.071915605	-2.497363835	0.012512051	0.070608344	SURF2	protein_coding
ENSG00000148296.7	2769.788645	-0.114443647	0.062802726	-1.82227197	0.068413729	0.216708953	SURF6	protein_coding
ENSG00000148297.16	2347.085427	-0.000388184	0.054475513	-0.007125848	0.994314444	0.99747325	MED22	protein_coding
ENSG00000148300.12	1544.587354	-0.078311307	0.057178342	-1.369597386	0.170812616	0.380917116	REXO4	protein_coding
ENSG00000148303.17	56743.0554	-0.164872395	0.067064383	-2.458419617	0.013955001	0.076272852	RPL7A	protein_coding

ENSG00000148308.18	3462.098504	-0.002992078	0.062018751	-0.048244732	0.9615212	0.982189624	GTF3C5	protein_coding
ENSG00000148331.12	1525.587305	0.092033421	0.047834122	1.924011909	0.054353091	0.187681825	ASB6	protein_coding
ENSG00000148334.16	4182.526529	-0.109899762	0.067897279	-1.618618046	0.10552947	0.28434494	PTGES2	protein_coding
ENSG00000148335.15	664.269543	0.070927523	0.064808589	1.094415487	0.273772795	0.502345239	NTMT1	protein_coding
ENSG00000148337.21	7220.376069	0.014182756	0.050066868	0.283276277	0.776965061	0.886910451	CIZ1	protein_coding
ENSG00000148339.12	1649.614652	0.277377401	0.079345335	3.495824926	0.000472599	0.006529623	SLC25A25	protein_coding
ENSG00000148341.18	4608.236947	0.021665646	0.063905975	0.339023795	0.734591804	0.862253465	SH3GLB2	protein_coding
ENSG00000148343.18	1651.195495	-0.062928581	0.056574519	-1.112313129	0.266003541	0.493933709	MIGA2	protein_coding
ENSG00000148344.11	764.1479767	0.601367247	0.138071119	4.355489037	1.3277E-05	0.000404596	PTGES	protein_coding
ENSG00000148346.12	95498.23364	-0.314556388	0.221392549	-1.420808377	0.155372475	0.359912095	LCN2	protein_coding
ENSG00000148356.14	1832.975226	-0.060512753	0.049017004	-1.234525724	0.217007067	0.438288699	LRSAM1	protein_coding
ENSG00000148357.16	1039.018462	0.145540574	0.164588615	0.884268783	0.376551136	0.602407168	HMCN2	protein_coding
ENSG00000148358.20	7036.935444	0.108203982	0.053577249	2.019588246	0.043426115	0.162493245	GPR107	protein_coding
ENSG00000148362.11	1740.533838	-0.150689714	0.066695156	-2.259380182	0.023859746	0.109396277	PAXX	protein_coding
ENSG00000148377.6	1.868901785	-0.247290709	0.196766332	-1.256773484	0.20883567	0.428772904	IDI2	protein_coding
ENSG00000148384.14	1200.96993	0.040538552	0.053792082	0.753615598	0.451080075	0.665736771	INPP5E	protein_coding
ENSG00000148386.9	0.897321225	-0.856792021	0.556798081	-1.538784077	0.123857018		LCN9	protein_coding
ENSG00000148396.18	14297.26968	0.18622983	0.06078101	3.063947613	0.002184372	0.020403054	SEC16A	protein_coding
ENSG00000148399.13	1028.013921	0.046308459	0.04834969	0.957781929	0.338172733	0.567969327	DPH7	protein_coding
ENSG00000148400.12	9756.05745	0.198729728	0.086600049	2.294799238	0.021744632	0.102769902	NOTCH1	protein_coding
ENSG00000148408.13	60.24167787	0.450680882	0.236152953	1.908427888	0.056335935	0.192048115	CACNA1B	protein_coding
ENSG00000148411.8	3390.760813	-0.05964857	0.095378991	-0.62538479	0.531718542	0.72921161	NACC2	protein_coding
ENSG00000148426.13	838.0390612	0.174563427	0.114409698	1.525774746	0.127065972	0.318263815	PROSER2	protein_coding
ENSG00000148429.14	2591.562117	-0.059683715	0.058515917	-1.019956928	0.307748889	0.537155908	USP6NL	protein_coding
ENSG00000148444.16	622.3727035	-0.198462008	0.069669303	-2.848629163	0.004390803	0.033902238	COMMD3	protein_coding
ENSG00000148450.13	1774.897091	-0.307750313	0.075323607	-4.085708645	4.39425E-05	0.001040697	MSRB2	protein_coding
ENSG00000148459.16	906.0700309	-0.305424447	0.084292049	-3.623407567	0.000290747	0.004542807	PDSS1	protein_coding
ENSG00000148468.17	3113.40827	-0.092098988	0.089269601	-1.031694863	0.302215085	0.531877476	FAM171A1	protein_coding
ENSG00000148481.14	1109.812166	0.013920835	0.061940661	0.224744703	0.822177886	0.912411743	MINDY3	protein_coding
ENSG00000148482.12	3.374493215	-0.192449868	0.252393589	-0.762499035	0.445762219	0.661439749	SLC39A12	protein_coding
ENSG00000148483.9	108.992882	0.106277149	0.176360475	0.60261319	0.546766043	0.740527864	TMEM236	protein_coding
ENSG00000148484.18	4808.130912	-0.086201343	0.049334052	-1.747299077	0.080585473	0.241103547	RSU1	protein_coding
ENSG00000148488.17	90.63417461	-0.11792453	0.162264003	-0.72674486	0.467382267	0.678865185	ST8SIA6	protein_coding
ENSG00000148498.16	3585.657787	0.033977518	0.060632011	0.56038909	0.575214072	0.760177825	PARD3	protein_coding
ENSG00000148513.18	5.350373082	0.129057901	0.265579683	0.485947942	0.627004076	0.796773746	ANKRD30A	protein_coding
ENSG00000148516.22	3474.267169	-0.126015924	0.120729684	-1.043785752	0.296584525	0.526403708	ZEB1	protein_coding
ENSG00000148541.13	243.8589866	-0.061853064	0.132191701	-0.467904292	0.639853034	0.805025859	FAM13C	protein_coding
ENSG00000148572.16	1403.04511	0.032451778	0.043673577	0.743052897	0.457449655	0.670998953	NRBF2	protein_coding
ENSG00000148584.15	998.2051928	-0.553555151	0.207824418	-2.663571271	0.007731603	0.050523589	A1CF	protein_coding
ENSG00000148600.15	124.2154336	-0.376900558	0.229550111	-1.641909722	0.100608715	0.276554972	CDHR1	protein_coding
ENSG00000148602.6	1.017979431	-0.098572158	0.415047741	-0.237495952	0.812272059	0.907271413	LRIT1	protein_coding
ENSG00000148604.15	7.336557476	-0.707251886	0.27757284	-2.547986629	0.010834662	0.063906617	RGR	protein_coding

ENSG00000148606.13	2141.53919	0.025526749	0.058266115	0.438106248	0.661309265	0.818589369	POLR3A	protein_coding
ENSG00000148634.16	2525.113756	0.005554622	0.050139799	0.1107827	0.911788665	0.958438215	HERC4	protein_coding
ENSG00000148655.15	309.3244159	0.023652214	0.119254314	0.198334239	0.842783565	0.922693752	LRMDA	protein_coding
ENSG00000148660.20	3299.88793	-0.17921159	0.073788078	-2.428733666	0.015151658	0.080578936	CAMK2G	protein_coding
ENSG00000148671.14	0						ADIRF	protein_coding
ENSG00000148672.9	7957.69382	-0.055860072	0.060253306	-0.927087259	0.353881231	0.582147315	GLUD1	protein_coding
ENSG00000148677.7	40.7873988	0.872380685	0.221481131	3.938848792	8.18735E-05	0.001699763	ANKRD1	protein_coding
ENSG00000148680.16	31.17315725	0.009787711	0.148885429	0.065739884	0.947584918	0.975430704	HTR7	protein_coding
ENSG00000148688.14	1379.803151	-0.137527057	0.049668917	-2.768875694	0.005625009	0.04045569	RPP30	protein_coding
ENSG00000148690.12	1088.969265	-0.170185162	0.056764239	-2.998105209	0.002716639	0.023836082	FRA10AC1	protein_coding
ENSG00000148700.15	9534.019564	-0.335127473	0.079802425	-4.199464775	2.67547E-05	0.000697719	ADD3	protein_coding
ENSG00000148702.15	1745.62087	-0.817035332	0.221948357	-3.68119567	0.000232143	0.003815152	HABP2	protein_coding
ENSG00000148704.13	7.521805298	0.556486839	0.30034635	1.852817049	0.063908586	0.207878215	VAX1	protein_coding
ENSG00000148719.15	3367.767465	-0.064348968	0.049306749	-1.305074234	0.191867572	0.408093184	DNAJB12	protein_coding
ENSG00000148730.7	13398.78819	-0.08711453	0.052558436	-1.657479495	0.097422571	0.271218513	EIF4EBP2	protein_coding
ENSG00000148734.8	165.9802078	0.064619218	0.161391686	0.400387528	0.688871109	0.835661616	NPFFR1	protein_coding
ENSG00000148735.15	3120.61699	-0.420512926	0.16269911	-2.584604959	0.009749063	0.059384247	PLEKHS1	protein_coding
ENSG00000148737.17	4331.357293	0.032982508	0.056448186	0.584297036	0.559020486	0.748868034	TCF7L2	protein_coding
ENSG00000148773.14	15702.05614	0.018735331	0.090172727	0.20777159	0.835407317	0.919344369	MKI67	protein_coding
ENSG00000148795.7	31.92407588	0.00973055	0.296141089	0.032857819	0.97378797	0.987842688	CYP17A1	protein_coding
ENSG00000148798.11	44.66423682	-0.405363371	0.182439557	-2.221905041	0.026289724	0.116774794	INA	protein_coding
ENSG00000148803.12	685.1182077	-0.352379609	0.108046476	-3.261370671	0.00110875	0.01243335	FUOM	protein_coding
ENSG00000148814.18	398.8701756	-0.044084038	0.060150585	-0.732894577	0.463622731	0.67589869	LRRC27	protein_coding
ENSG00000148824.19	265.429855	-0.192258068	0.066235899	-2.90262638	0.003700478	0.030001068	MTG1	protein_coding
ENSG00000148826.9	73.78833018	-1.532715527	0.335891324	-4.563129251	5.03968E-06	0.000187224	NKX6-2	protein_coding
ENSG00000148832.16	49.83984632	-0.220843139	0.089203806	-2.475714298	0.013296994	0.073720495	PAOX	protein_coding
ENSG00000148834.13	4416.672143	-0.11126801	0.062108059	-1.791522884	0.073209426	0.226274711	GSTO1	protein_coding
ENSG00000148835.11	458.4577456	-0.166354802	0.054118633	-3.07389144	0.002112863	0.019898905	TAF5	protein_coding
ENSG00000148840.11	4667.054643	0.029825651	0.050426778	0.591464547	0.554209203	0.745440288	PPRC1	protein_coding
ENSG00000148841.17	2089.709945	0.36108305	0.089549475	4.032218507	5.52528E-05	0.001242835	ITPRIP	protein_coding
ENSG00000148842.18	1560.64947	-0.011362374	0.055440431	-0.204947433	0.837613195	0.92008445	CNNM2	protein_coding
ENSG00000148843.15	3885.563842	-0.073722194	0.056427154	-1.30650207	0.191381875	0.407378272	PDCD11	protein_coding
ENSG00000148848.14	1521.473927	0.865548531	0.148314722	5.835890876	5.35039E-09	7.19611E-07	ADAM12	protein_coding
ENSG00000148908.15	1324.459826	-0.105254602	0.078599543	-1.339124858	0.180530029	0.39352494	RGS10	protein_coding
ENSG00000148925.11	1769.517978	0.030121496	0.042804647	0.703696884	0.481621561	0.690541334	BTBD10	protein_coding
ENSG00000148926.10	1055.751119	0.66735418	0.124176541	5.374237136	7.69076E-08	6.57138E-06	ADM	protein_coding
ENSG00000148935.11	146.1637543	-0.498517179	0.132337797	-3.767005264	0.000165218	0.002925375	GAS2	protein_coding
ENSG00000148942.15	130.7440056	0.171508102	0.219232441	0.782311694	0.434031416	0.651772529	SLC5A12	protein_coding
ENSG00000148943.12	3181.378722	-0.022732291	0.055285942	-0.411176709	0.680942965	0.83095636	LIN7C	protein_coding
ENSG00000148948.8	53.13352852	0.004242208	0.168802209	0.025131232	0.979950288	0.991148319	LRRC4C	protein_coding
ENSG00000148950.11	233.7190992	-0.065212994	0.05018244	-1.299518196	0.193766153	0.410353768	IMMP1L	protein_coding
ENSG00000148965.10	8.665822225	0.201190454	0.313046129	0.642686285	0.520427682	0.720588324	SAA4	protein_coding

ENSG00000148985.20	1458.112972	-0.170419787	0.05705478	-2.986950196	0.002817756	0.024479115	PGAP2	protein_coding
ENSG00000149016.16	444.1863481	0.010729821	0.054637385	0.196382399	0.844310871	0.923608042	TUT1	protein_coding
ENSG00000149021.7	10.83525134	-0.774365272	0.33653729	-2.300979103	0.021392812	0.101685297	SCGB1A1	protein_coding
ENSG00000149043.16	1307.120815	0.113185671	0.222938332	0.507699461	0.611664127	0.786755443	SYT8	protein_coding
ENSG00000149050.10	93.45933442	0.011810598	0.104903818	0.112585014	0.910359567	0.957559652	ZNF214	protein_coding
ENSG00000149054.16	161.1421093	0.130913773	0.140909408	0.929063389	0.352856236	0.581231458	ZNF215	protein_coding
ENSG00000149084.13	3388.642285	0.182102217	0.070288107	2.590796996	0.009575396	0.058604919	HSD17B12	protein_coding
ENSG00000149089.13	1118.917235	-0.239538393	0.072330974	-3.311698681	0.000927314	0.010834094	APIP	protein_coding
ENSG00000149090.12	359.5024744	0.335602786	0.117001075	2.86837353	0.004125881	0.032421886	PAMR1	protein_coding
ENSG00000149091.15	7351.437473	-0.088659976	0.057493091	-1.542097922	0.1230498	0.312151655	DGKZ	protein_coding
ENSG00000149100.13	6684.279553	-0.131285296	0.058067387	-2.260912757	0.02376466	0.109124614	EIF3M	protein_coding
ENSG00000149115.14	8348.084162	0.225775874	0.064591664	3.495433629	0.000473292	0.006533882	TNKS1BP1	protein_coding
ENSG00000149124.11	2.734595138	0.146389656	0.268509963	0.545192642	0.585621031	0.767963955	GLYAT	protein_coding
ENSG00000149131.17	19029.50847	0.232839031	0.115077895	2.023316739	0.043040502	0.161645778	SERPING1	protein_coding
ENSG00000149133.1	0.331236701	-0.064634297	0.644547824	-0.100278513	0.920123216		OR5F1	protein_coding
ENSG00000149136.9	8419.665314	-0.003559475	0.050610039	-0.070331394	0.943929896	0.973649067	SSRP1	protein_coding
ENSG00000149150.9	2251.879433	-0.175826572	0.109533626	-1.605229173	0.10844338	0.288789103	SLC43A1	protein_coding
ENSG00000149177.13	4398.134213	0.043916161	0.077197434	0.568881096	0.569436835	0.756283552	PTPRJ	protein_coding
ENSG00000149179.14	2057.077459	-0.004060749	0.053134961	-0.0764233	0.939082333	0.97110562	C11orf49	protein_coding
ENSG00000149182.15	4016.163698	-0.068523801	0.042143214	-1.625974737	0.10395503	0.281849314	ARFGAP2	protein_coding
ENSG00000149187.18	7603.740289	-0.017351337	0.034475298	-0.503297664	0.614755008	0.788602281	CELF1	protein_coding
ENSG00000149196.16	1488.539334	-0.101419857	0.058219087	-1.742037927	0.081501808	0.242924671	HIKESHI	protein_coding
ENSG00000149201.10	31.50446315	-0.059471004	0.094635907	-0.628419018	0.529729482	0.727752246	CCDC81	protein_coding
ENSG00000149212.12	2612.443509	0.019368939	0.124673864	0.155356854	0.876539997	0.940326024	SESN3	protein_coding
ENSG00000149218.5	2883.040977	0.254663366	0.076203118	3.341902167	0.000832064	0.009977668	ENDOD1	protein_coding
ENSG00000149231.15	1619.166678	0.03973295	0.054802309	0.725023281	0.468437748	0.679870499	CCDC82	protein_coding
ENSG00000149243.16	300.0370102	0.188781834	0.147195915	1.282520877	0.199659986	0.41791552	KLHL35	protein_coding
ENSG00000149256.16	655.7452732	0.577601175	0.139849762	4.130154868	3.62519E-05	0.000887001	TENM4	protein_coding
ENSG00000149257.16	14089.43393	0.414689523	0.075488666	5.493401151	3.94266E-08	3.78434E-06	SERPINH1	protein_coding
ENSG00000149260.18	11685.56921	0.119045568	0.106144661	1.121540797	0.262057736	0.489506192	CAPN5	protein_coding
ENSG00000149262.17	1462.826712	0.043394686	0.043420321	0.999409594	0.317596314	0.54683688	INTS4	protein_coding
ENSG00000149269.10	5420.783431	-0.100861676	0.058333881	-1.729041065	0.083801746	0.247173747	PAK1	protein_coding
ENSG00000149273.15	67200.61697	-0.122147343	0.070752032	-1.726414635	0.084272845	0.248031728	RPS3	protein_coding
ENSG00000149289.11	908.9100873	0.081011577	0.109378109	0.740656224	0.458901908	0.672164581	ZC3H12C	protein_coding
ENSG00000149292.17	566.2881804	-0.188171766	0.062048603	-3.032651142	0.002424157	0.021908022	TTC12	protein_coding
ENSG00000149294.17	584.1541965	-0.375455095	0.184637656	-2.033469789	0.042005083	0.159011527	NCAM1	protein_coding
ENSG00000149295.14	51.08826933	-0.063706938	0.235175033	-0.270891586	0.786474416	0.892063709	DRD2	protein_coding
ENSG00000149300.10	1.932769849	0.136488659	0.179727786	0.759418796	0.447602073	0.662854467	C11orf52	protein_coding
ENSG00000149305.7	8.851184616	-0.718384171	0.264257269	-2.71850297	0.006557807	0.044992428	HTR3B	protein_coding
ENSG00000149308.17	1781.629617	-0.019538674	0.051403463	-0.380104235	0.703868042	0.84460581	NPAT	protein_coding
ENSG00000149311.19	3278.7451	-0.142088111	0.070672091	-2.010526472	0.044375497	0.164762438	ATM	protein_coding
ENSG00000149313.11	1835.006714	-0.071178245	0.050800595	-1.401130126	0.161175164	0.367571886	AASDHPPT	protein_coding

ENSG00000149328.15	1579.530914	-0.060854421	0.117703356	-0.51701517	0.605145569	0.782340546	GLB1L2	protein_coding
ENSG00000149346.15	1046.578962	-0.30433973	0.063575736	-4.787042201	1.69257E-06	7.82155E-05	SLX4IP	protein_coding
ENSG00000149357.10	5666.052953	-0.033139057	0.050554214	-0.655515235	0.512136081	0.714767721	LAMTOR1	protein_coding
ENSG00000149380.12	226.4895845	0.694990381	0.117377804	5.920969339	3.2005E-09	4.79271E-07	P4HA3	protein_coding
ENSG00000149403.13	35.29046642	-0.198010843	0.149263016	-1.326590125	0.184644304	0.398941189	GRIK4	protein_coding
ENSG00000149418.11	20868.88706	0.15205837	0.081185057	1.872984721	0.061070495	0.202010942	ST14	protein_coding
ENSG00000149428.19	12305.12064	0.12087763	0.078383476	1.542131539	0.123041632	0.312151655	HYOU1	protein_coding
ENSG00000149435.12	2.519308039	-0.162190817	0.233133389	-0.695699648	0.486616946	0.694140779	GGTLC1	protein_coding
ENSG00000149443.7	1.018387285	-0.196527006	0.307883239	-0.638316676	0.523267559	0.722610203	SCP2D1-AS1	lncRNA
ENSG00000149451.18	589.1516852	-0.01137255	0.184532902	-0.061628846	0.950858404	0.97706123	ADAM33	protein_coding
ENSG00000149452.16	6.137279669	-0.395617146	0.275214725	-1.437485387	0.150580122	0.352919377	SLC22A8	protein_coding
ENSG00000149474.14	1481.25519	-0.218939331	0.061607941	-3.553751813	0.000379777	0.00553497	KAT14	protein_coding
ENSG00000149476.16	2501.120164	-0.007147907	0.068044759	-0.105047133	0.916338408	0.960383969	TKFC	protein_coding
ENSG00000149480.7	6966.208005	0.056407709	0.047890723	1.177842081	0.238859569	0.46428402	MTA2	protein_coding
ENSG00000149483.12	2053.485334	-0.052617565	0.050223157	-1.047675368	0.2947882	0.524845559	TMEM138	protein_coding
ENSG00000149485.19	1665.849034	0.602622363	0.116208595	5.185695281	2.1521E-07	1.49876E-05	FADS1	protein_coding
ENSG00000149488.13	28.14104372	-0.746075573	0.200886241	-3.713920723	0.000204073	0.003444673	TMC2	protein_coding
ENSG00000149489.9	103.8244599	0.054747763	0.080607488	0.679189547	0.497017769	0.703267959	ROM1	protein_coding
ENSG00000149499.12	2685.508262	0.046884986	0.048669822	0.963327679	0.335383107	0.564857097	EML3	protein_coding
ENSG00000149503.13	2602.766265	0.095342738	0.069788412	1.36616862	0.171886044	0.382469268	INCENP	protein_coding
ENSG00000149506.11	10.15951955	-0.302703579	0.141718894	-2.135943703	0.032684003	0.134841648	ZP1	protein_coding
ENSG00000149507.7	1.776539465	-0.205999616	0.438844953	-0.469413206	0.638774311	0.804400222	OOSP2	protein_coding
ENSG00000149516.14	4.255937929	-0.075162942	0.263799816	-0.284924165	0.775702242	0.886351223	MS4A3	protein_coding
ENSG00000149527.18	417.9966285	0.613003047	0.170192184	3.601828425	0.000315987	0.004829411	PLCH2	protein_coding
ENSG00000149531.15	840.2018636	-0.028362678	0.090418655	-0.313681704	0.753762798	0.873513322	FRG1BP	unprocessed_pseudogene
ENSG00000149532.16	5128.748022	0.046625191	0.039729178	1.17357553	0.240565087	0.466019329	CPSF7	protein_coding
ENSG00000149534.9	193.6109637	-0.416749415	0.155606831	-2.678220563	0.007401446	0.048953376	MS4A2	protein_coding
ENSG00000149541.10	1416.168973	0.087528131	0.063572843	1.376816362	0.168569031	0.377818934	B3GAT3	protein_coding
ENSG00000149547.15	9226.318929	-0.11296071	0.051175165	-2.207334541	0.027290696	0.119753738	EI24	protein_coding
ENSG00000149548.15	197.3315779	-0.204822371	0.079562348	-2.574363083	0.010042482	0.060671185	CCDC15	protein_coding
ENSG00000149554.13	1440.217058	-0.124964225	0.079511927	-1.571641265	0.116033771	0.301010835	CHEK1	protein_coding
ENSG00000149557.14	529.2015345	0.036181883	0.112847292	0.320626953	0.748493109	0.870717678	FEZ1	protein_coding
ENSG00000149564.12	2448.376808	0.006292273	0.082560623	0.076213968	0.93924887	0.971139563	ESAM	protein_coding
ENSG00000149571.12	38.42343157	0.378280745	0.155430217	2.433765794	0.01494266	0.079886677	KIRREL3	protein_coding
ENSG00000149573.9	3678.52175	0.137639588	0.10123114	1.359656608	0.173938616	0.385049801	MPZL2	protein_coding
ENSG00000149575.9	49.04540228	0.063542905	0.198727212	0.319749391	0.749158315	0.871072946	SCN2B	protein_coding
ENSG00000149577.16	4406.806466	-0.001786988	0.074177839	-0.024090585	0.980780353	0.991611682	SIDT2	protein_coding
ENSG00000149582.16	721.290889	0.025767262	0.119091119	0.216365941	0.828702513	0.915634984	TMEM25	protein_coding
ENSG00000149591.17	22551.30067	-0.196651762	0.184808454	-1.064084237	0.287290541	0.516982983	TAGLN	protein_coding
ENSG00000149596.7	1719.535508	-0.348726921	0.208041468	-1.676237551	0.093691672	0.264529232	JPH2	protein_coding
ENSG00000149599.15	192.9663691	-0.0403217	0.178252985	-0.226204908	0.821042054	0.912020127	DUSP15	protein_coding
ENSG00000149600.12	1802.941687	-0.213075173	0.065812234	-3.237622533	0.001205302	0.013240088	COMMD7	protein_coding

ENSG00000149609.6	32.77600052	0.163368833	0.096518265	1.692620907	0.090527649	0.259108267	C20orf144	protein_coding
ENSG00000149633.12	616.731533	0.303989415	0.116290592	2.614049943	0.008947596	0.055853084	KIAA1755	protein_coding
ENSG00000149634.4	50.13641758	-0.026242519	0.118458854	-0.221532778	0.824677617	0.913424534	SPATA25	protein_coding
ENSG00000149635.3	2.587690842	0.164982145	0.234113386	0.704710429	0.48099046	0.689954286	OCSTAMP	protein_coding
ENSG00000149636.16	1492.094633	-0.122881535	0.066795066	-1.839679826	0.065815257	0.211413199	DSN1	protein_coding
ENSG00000149639.15	5294.01265	0.181003617	0.102161156	1.771745979	0.076436734	0.233140865	SOGA1	protein_coding
ENSG00000149646.12	28.04652291	0.037471618	0.145764702	0.257069217	0.79712534	0.898485061	CNBD2	protein_coding
ENSG00000149651.4	2.246640023	-0.070067597	0.271903308	-0.257693064	0.796643799	0.89824433	SPINT4	protein_coding
ENSG00000149654.11	31.06049618	-1.013066783	0.236842013	-4.277394759	1.89093E-05	0.000531621	CDH22	protein_coding
ENSG00000149656.8	2.943832674	0.015057667	0.209219085	0.071970809	0.942625138	0.972949834	LINC00266-1	lncRNA
ENSG00000149657.20	2827.488874	-0.053677656	0.074889617	-0.716756976	0.473524063	0.684335161	LSM14B	protein_coding
ENSG00000149658.18	5542.017499	-0.008798861	0.052383061	-0.167971496	0.866605703	0.935310004	YTHDF1	protein_coding
ENSG00000149679.11	1403.251182	0.15468578	0.094784669	1.631970461	0.102685709	0.279933384	CABLES2	protein_coding
ENSG00000149716.13	883.2386321	0.041854405	0.074741105	0.559991787	0.57548504	0.760397562	LTO1	protein_coding
ENSG00000149735.7	6.163781857	0.053997636	0.242411598	0.222751868	0.82372863	0.913038715	GPHA2	protein_coding
ENSG00000149742.10	12.54305053	-0.068234994	0.225120559	-0.303104233	0.761810429	0.878168745	SLC22A9	protein_coding
ENSG00000149743.14	1173.924391	0.005287929	0.055455695	0.095354119	0.924033558	0.964123317	TRPT1	protein_coding
ENSG00000149761.9	939.9122153	0.05843759	0.063180991	0.924923612	0.355005643	0.583053384	NUDT22	protein_coding
ENSG00000149781.13	2138.101219	-0.474359044	0.115152671	-4.119392458	3.79873E-05	0.000924788	FERMT3	protein_coding
ENSG00000149782.13	6791.202431	-0.005088476	0.081792121	-0.062212302	0.950393766	0.976799196	PLCB3	protein_coding
ENSG00000149792.9	3396.918301	-0.010607213	0.054480247	-0.194698327	0.845629122	0.924295023	MRPL49	protein_coding
ENSG00000149798.5	2.955859026	0.38665375	0.155620799	2.484589158	0.012970102	0.072452013	CDC42EP2	protein_coding
ENSG00000149806.11	10274.57359	-0.201231655	0.064299288	-3.129609376	0.001750389	0.017386984	FAU	protein_coding
ENSG00000149809.16	1133.871766	-0.106569959	0.113762325	-0.936777259	0.348873146	0.578039695	TM7SF2	protein_coding
ENSG00000149823.9	4965.230699	-0.000933682	0.061955666	-0.015070161	0.987976206	0.99431948	VPS51	protein_coding
ENSG00000149922.11	226.8075726	0.24192959	0.126356538	1.914658257	0.0555361	0.190214308	TBX6	protein_coding
ENSG00000149923.14	6747.053218	-0.000705165	0.062066565	-0.011361438	0.990935079	0.995928813	PPP4C	protein_coding
ENSG00000149925.22	1697.348366	0.125260169	0.132167778	0.947736059	0.343263839	0.572943478	ALDOA	protein_coding
ENSG00000149926.13	24.72391564	0.279651223	0.143687148	1.946250776	0.051624623	0.181632247	TLCD3B	protein_coding
ENSG00000149927.18	103.2842496	0.691401662	0.185346974	3.730309956	0.000191244	0.003282338	DOC2A	protein_coding
ENSG00000149929.16	962.434796	-0.051940015	0.061833208	-0.84000195	0.400907293	0.623295055	HIRIP3	protein_coding
ENSG00000149930.18	4598.527794	-0.011406318	0.049505604	-0.230404581	0.817777402	0.910133721	TAOK2	protein_coding
ENSG00000149932.17	4556.225851	-0.058091862	0.053522149	-1.085379848	0.277753454	0.506669363	TMEM219	protein_coding
ENSG00000149948.14	1401.686671	0.170714873	0.201825481	0.845853914	0.397634244	0.620797774	HMGA2	protein_coding
ENSG00000149968.12	2404.662736	0.640748864	0.239310857	2.677475109	0.007417936	0.049003848	MMP3	protein_coding
ENSG00000149970.16	139.6983831	-0.659883379	0.208271467	-3.168381104	0.001532904	0.015758286	CNKSR2	protein_coding
ENSG00000149972.11	19.0654789	0.447799708	0.23226466	1.927971774	0.053858637	0.186565805	CNTN5	protein_coding
ENSG00000150045.12	20.98420297	-0.417882413	0.134862894	-3.098572196	0.001944556	0.018730749	KLRF1	protein_coding
ENSG00000150048.10	165.757469	-0.10932605	0.094816295	-1.153030187	0.248897994	0.475586595	CLEC1A	protein_coding
ENSG00000150051.14	206.5916571	-0.085939784	0.180028427	-0.477367858	0.633100198	0.800904614	MKX	protein_coding
ENSG00000150054.18	2027.885621	-0.544424054	0.123623286	-4.403895681	1.06324E-05	0.000341638	MPP7	protein_coding
ENSG00000150093.20	24898.81661	0.190491686	0.070068952	2.718631855	0.006555252	0.044985548	ITGB1	protein_coding

ENSG00000150175.13	5.698375747	-0.483516132	0.354000178	-1.365864096	0.171981624	0.382584268	FRMPD2B	unprocessed_pseudogene
ENSG00000150201.15	87.03338679	1.438663948	0.217923697	6.601686591	4.06506E-11	1.27441E-08	FXYD4	protein_coding
ENSG00000150244.12	0.811395349	0.69197885	0.592728942	1.167445692	0.243030428		TRIM48	protein_coding
ENSG00000150261.4	0.80508509	-0.602511764	0.592910765	-1.016192992	0.309537493		OR8K1	protein_coding
ENSG00000150269.1	0						OR5M9	protein_coding
ENSG00000150275.19	13.22031006	-0.059742299	0.231868076	-0.257656422	0.796672081	0.898252976	PCDH15	protein_coding
ENSG00000150276.8	4.177820215	-0.05511803	0.153255455	-0.359648077	0.719110326	0.853088321	PPIAP26	processed_pseudogene
ENSG00000150281.7	451.1680204	0.291442406	0.153461856	1.899119515	0.057548764	0.194614503	CTF1	protein_coding
ENSG00000150316.12	2388.834005	-0.100677733	0.051449092	-1.956841789	0.050366072	0.178792734	CWC15	protein_coding
ENSG00000150337.13	248.745876	0.010948528	0.126902217	0.086275312	0.931247564	0.967365318	FCGR1A	protein_coding
ENSG00000150347.16	5355.238833	-0.178677043	0.076257817	-2.343065287	0.019126036	0.094090993	ARID5B	protein_coding
ENSG00000150361.12	3.269171336	-0.240668428	0.371392111	-0.648017071	0.516973909	0.718258314	KLHL1	protein_coding
ENSG00000150394.14	25.49090206	0.087624253	0.1650244	0.530977558	0.595434331	0.775189053	CDH8	protein_coding
ENSG00000150401.15	843.3037537	0.128620331	0.059028351	2.178958568	0.029334746	0.125621092	DCUN1D2	protein_coding
ENSG00000150403.18	3192.994607	0.316851149	0.063788557	4.967209841	6.79231E-07	3.82414E-05	TMCO3	protein_coding
ENSG00000150433.9	648.5858748	-0.045823257	0.045211094	-1.0135401	0.310802253	0.540326728	TMEM218	protein_coding
ENSG00000150455.13	510.9957953	-0.033736188	0.055684742	-0.605842593	0.544619283	0.739047345	TIRAP	protein_coding
ENSG00000150456.11	433.625075	-0.158020098	0.075342521	-2.097356131	0.035962061	0.143377745	EEF1AKMT1	protein_coding
ENSG00000150457.9	2546.896895	0.337120153	0.064544118	5.223096447	1.75956E-07	1.28868E-05	LATS2	protein_coding
ENSG00000150459.12	9038.8711	0.010260954	0.060129134	0.170648623	0.864500063	0.934077793	SAP18	protein_coding
ENSG00000150471.16	404.0454446	0.179982024	0.146599844	1.227709521	0.219556031	0.441391085	ADGRL3	protein_coding
ENSG00000150477.15	414.4921494	-0.055874611	0.062801954	-0.889695418	0.373629455	0.599688535	KIAA1328	protein_coding
ENSG00000150510.17	497.7866938	-0.06570027	0.136521666	-0.481244274	0.630342898	0.7991985	FAM124A	protein_coding
ENSG00000150527.17	3917.81693	-0.035185848	0.063309203	-0.55577777	0.578362784	0.762696778	MIA2	protein_coding
ENSG00000150540.14	2301.03021	-0.176937649	0.074444876	-2.376760623	0.017465418	0.088654248	HNMT	protein_coding
ENSG00000150551.11	70.09586369	0.218683324	0.138670934	1.576994676	0.11479675	0.299031501	LYPD1	protein_coding
ENSG00000150556.17	910.1653115	-0.013650388	0.182001573	-0.075001482	0.940213533	0.971745016	LYPD6B	protein_coding
ENSG00000150593.18	3757.526021	-0.299452538	0.08662374	-3.456933826	0.000546359	0.007280944	PDCD4	protein_coding
ENSG00000150594.7	2751.966044	-0.450764692	0.19588596	-2.30115875	0.02138266	0.101668539	ADRA2A	protein_coding
ENSG00000150625.16	80.01343758	-0.115389517	0.197110123	-0.585406346	0.558274523	0.748482567	GPM6A	protein_coding
ENSG00000150627.16	98.98234979	-0.216881509	0.190743206	-1.137033995	0.255524075	0.482513871	WDR17	protein_coding
ENSG00000150628.7	5.694205011	-0.321546023	0.226974314	-1.41666261	0.156581594	0.361425717	SPATA4	protein_coding
ENSG00000150630.4	549.5506171	0.195654461	0.090189574	2.169368944	0.030054685	0.127780292	VEGFC	protein_coding
ENSG00000150636.17	266.8685658	-0.124514389	0.094513554	-1.317423631	0.187696664	0.402879398	CCDC102B	protein_coding
ENSG00000150637.9	281.2948282	-0.768826264	0.117964858	-6.517417805	7.1528E-11	2.09193E-08	CD226	protein_coding
ENSG00000150656.15	69.25842722	-0.253232643	0.204873609	-1.236043255	0.216442486	0.437655524	CNDP1	protein_coding
ENSG00000150667.8	22.06687779	-0.006831499	0.102966291	-0.066346943	0.947101611	0.975196627	FSIP1	protein_coding
ENSG00000150672.17	179.3334831	-0.131114305	0.150183985	-0.873024543	0.382649701	0.607803743	DLG2	protein_coding
ENSG00000150676.13	7.320095906	-0.153999272	0.212012903	-0.726367451	0.467613539	0.679149287	CCDC83	protein_coding
ENSG00000150681.10	134.2390843	-0.294097885	0.121070752	-2.429140652	0.01513466	0.080518015	RGS18	protein_coding
ENSG00000150687.12	6175.257541	-0.023644042	0.103362663	-0.228748384	0.819064487	0.910868269	PRSS23	protein_coding
ENSG00000150712.11	3183.852664	-0.010470931	0.061416778	-0.170489745	0.864624999	0.934097669	MTMR12	protein_coding

ENSG00000150722.10	114.3415794	0.436638674	0.158449851	2.755690024	0.005856847	0.041617093	PPP1R1C	protein_coding
ENSG00000150732.8	0.61432289	0.242677979	0.607909582	0.39920078	0.689745269		AL136372.1	processed_pseudogene
ENSG00000150750.8	180.5513517	-0.208467455	0.186464142	-1.118002917	0.263565747	0.491264663	C11orf53	protein_coding
ENSG00000150753.12	11204.93309	0.003777833	0.066566693	0.056752599	0.954742274	0.97886747	CCT5	protein_coding
ENSG00000150756.14	697.115383	-0.108610095	0.06590542	-1.647969099	0.099359012	0.274557241	ATPSCKMT	protein_coding
ENSG00000150760.13	4222.658749	0.077282152	0.060331741	1.280953453	0.200210016	0.418642691	DOCK1	protein_coding
ENSG00000150764.14	1884.657399	-0.258232372	0.13266559	-1.946490959	0.051595793	0.181570691	DIXDC1	protein_coding
ENSG00000150768.16	2753.024694	0.007275674	0.06277403	0.115902619	0.90772972	0.956333677	DLAT	protein_coding
ENSG00000150773.11	79.25306978	0.053710312	0.075611025	0.710350268	0.477486955	0.687213457	PIH1D2	protein_coding
ENSG00000150776.18	1790.99264	-0.055230715	0.041295087	-1.337464544	0.181071048	0.394283458	NKAPD1	protein_coding
ENSG00000150779.12	1659.650276	-0.097904737	0.065982937	-1.483788712	0.137864983	0.33424119	TIMM8B	protein_coding
ENSG00000150782.12	1866.295399	0.149424975	0.110102064	1.357149626	0.174733673	0.386145151	IL18	protein_coding
ENSG00000150783.10	6.31663932	-0.059220264	0.141750533	-0.417778065	0.67610939	0.828281996	TEX12	protein_coding
ENSG00000150787.8	905.9546459	0.025047738	0.062523955	0.400610259	0.688707091	0.835625243	PTS	protein_coding
ENSG00000150867.14	2683.578137	-0.152813276	0.06434192	-2.375018903	0.017548051	0.088969336	PIP4K2A	protein_coding
ENSG00000150873.12	72.5646756	0.151538966	0.132467268	1.143972907	0.252634873	0.479405585	C2orf50	protein_coding
ENSG00000150893.11	648.9607445	0.544956551	0.223791241	2.43511117	0.014887215	0.079694211	FREM2	protein_coding
ENSG00000150907.10	2228.303295	0.315642992	0.079627118	3.964013751	7.37E-05	0.001567411	FOXO1	protein_coding
ENSG00000150938.10	5740.754464	0.089987732	0.073160948	1.229996797	0.218698304	0.440316762	CRIM1	protein_coding
ENSG00000150961.15	6031.259998	0.330148818	0.068925516	4.789936146	1.66834E-06	7.75068E-05	SEC24D	protein_coding
ENSG00000150967.18	567.7959857	0.222342602	0.089376203	2.487715898	0.012856639	0.072008436	ABCB9	protein_coding
ENSG00000150977.10	1134.805024	-0.076723624	0.064013538	-1.19855309	0.230701768	0.45465673	RILPL2	protein_coding
ENSG00000150990.8	2292.734328	0.005392923	0.055679325	0.09685684	0.922840084	0.9635498	DHX37	protein_coding
ENSG00000150991.15	29138.91305	0.104276576	0.050671413	2.057897534	0.03959997	0.153105129	UBC	protein_coding
ENSG00000150995.20	1808.591572	-0.162843643	0.103977636	-1.566141037	0.117315603	0.303024274	ITPR1	protein_coding
ENSG00000151005.5	3.114503569	0.456743356	0.243283892	1.877408951	0.060462066	0.200773865	TKTL2	protein_coding
ENSG00000151006.7	32.15035251	0.039282732	0.093438	0.420414945	0.674182352	0.826897587	PRSS53	protein_coding
ENSG00000151012.13	1872.215774	0.416726742	0.145548794	2.863141163	0.004194636	0.032775627	SLC7A11	protein_coding
ENSG00000151014.6	476.7408597	0.200585056	0.073946259	2.712578821	0.00667619	0.045485982	NOCT	protein_coding
ENSG00000151023.17	74.69655725	-0.026245652	0.13815134	-0.189977538	0.849326732	0.926203979	ENKUR	protein_coding
ENSG00000151025.11	302.0510708	0.394596191	0.191898394	2.056276675	0.039755855	0.153495244	GPR158	protein_coding
ENSG00000151033.10	1.514152861	-0.151957605	0.38923185	-0.390403829	0.696237957	0.839744297	LYZL2	protein_coding
ENSG00000151062.15	258.8563659	0.118313417	0.093729204	1.262289789	0.20684453	0.426698944	CACNA2D4	protein_coding
ENSG00000151065.14	1378.817617	0.064558292	0.071073565	0.908330575	0.363703593	0.590987825	DCP1B	protein_coding
ENSG00000151067.22	1646.049279	-0.045977714	0.159683625	-0.287930052	0.773400283	0.885415085	CACNA1C	protein_coding
ENSG00000151079.7	28.9657131	0.033611188	0.210338905	0.159795393	0.873042253	0.938607117	KCNA6	protein_coding
ENSG00000151090.20	1280.609252	-0.069398035	0.137040726	-0.506404459	0.612572745	0.787180635	THRB	protein_coding
ENSG00000151092.18	1570.513512	-0.061775932	0.045841383	-1.347601844	0.177786477	0.390238318	NGLY1	protein_coding
ENSG00000151093.8	557.3005382	0.0089305	0.059573825	0.149906436	0.880838434	0.942709346	OXSM	protein_coding
ENSG00000151116.17	2094.814372	0.007261421	0.046092905	0.157538799	0.874820233	0.939513865	UEVLD	protein_coding
ENSG00000151117.9	468.1153257	0.163769109	0.087807302	1.865096702	0.062167851	0.204474936	TMEM86A	protein_coding
ENSG00000151131.11	1042.145085	-0.226299709	0.059567315	-3.799058421	0.000145247	0.002652734	C12orf45	protein_coding

ENSG00000151135.10	3198.052984	0.064496116	0.061136416	1.054954142	0.291446298	0.521415779	TMEM263	protein_coding
ENSG00000151136.15	350.2253524	0.852791635	0.166294093	5.128213638	2.92504E-07	1.92545E-05	BTBD11	protein_coding
ENSG00000151148.14	3287.91343	-0.003182885	0.039315103	-0.080958336	0.935475086	0.969630318	UBE3B	protein_coding
ENSG00000151150.22	1788.382435	-0.145211899	0.077342124	-1.877526663	0.060445947	0.200750935	ANK3	protein_coding
ENSG00000151151.6	1495.267612	0.054789125	0.066917878	0.818751676	0.412928107	0.633530547	IPMK	protein_coding
ENSG00000151164.19	57.94569155	-0.229287026	0.100775175	-2.275233218	0.022891943	0.106565774	RAD9B	protein_coding
ENSG00000151176.8	7345.585872	0.110592152	0.047979274	2.304998434	0.021166661	0.100938891	PLBD2	protein_coding
ENSG00000151208.17	4249.485785	0.153353775	0.079610145	1.926309467	0.054065744	0.187089885	DLG5	protein_coding
ENSG00000151224.13	124.7467184	-0.855830391	0.243173618	-3.519421215	0.000432489	0.006103171	MAT1A	protein_coding
ENSG00000151229.13	1296.312823	0.110084712	0.08579316	1.283140884	0.199442722	0.417669179	SLC2A13	protein_coding
ENSG00000151233.11	1261.665826	0.037487047	0.059938953	0.625420451	0.531695143	0.72921161	GXYLT1	protein_coding
ENSG00000151239.14	7400.525834	-0.025315695	0.055313815	-0.457674	0.64718667	0.809673823	TWF1	protein_coding
ENSG00000151240.17	1884.740675	0.199565057	0.087090753	2.291460935	0.021936769	0.103344711	DIP2C	protein_coding
ENSG00000151247.12	2138.065193	-0.022386992	0.052985723	-0.422509888	0.672652885	0.825928283	EIF4E	protein_coding
ENSG00000151276.23	4088.625654	-0.207871026	0.071904204	-2.890943987	0.003840866	0.030812482	MAGI1	protein_coding
ENSG00000151287.17	295.668049	-0.005410652	0.089162477	-0.060683059	0.951611624	0.977271197	TEX30	protein_coding
ENSG00000151292.17	2037.392366	0.03234991	0.048266194	0.670239502	0.502705126	0.707371485	CSNK1G3	protein_coding
ENSG00000151303.11	4.209050598	-0.024777074	0.191974796	-0.129064202	0.897306848	0.9512493	AL136982.1	lncRNA
ENSG00000151304.6	745.3239011	0.027935255	0.049506672	0.564272515	0.572568684	0.758568209	SRFBP1	protein_coding
ENSG00000151320.11	637.9230973	-0.569843032	0.177528373	-3.209870185	0.001327949	0.014221688	AKAP6	protein_coding
ENSG00000151322.19	97.40181641	0.243684251	0.155482066	1.567282048	0.11704878	0.302690325	NPAS3	protein_coding
ENSG00000151327.13	3359.007332	-0.081236662	0.054354055	-1.494583282	0.135023232	0.330370993	FAM177A1	protein_coding
ENSG00000151332.19	1353.941276	-0.105489637	0.081653373	-1.291920143	0.196384801	0.413603039	MBIP	protein_coding
ENSG00000151338.18	509.6895097	-0.125428454	0.100629653	-1.246436323	0.212604253	0.43323127	MIPOL1	protein_coding
ENSG00000151348.15	4454.274913	0.152998988	0.042416204	3.607088162	0.000309652	0.004759692	EXT2	protein_coding
ENSG00000151353.15	1188.673593	-0.054916766	0.050921794	-1.078453081	0.280831609	0.509738381	TMEM18	protein_coding
ENSG00000151360.10	5.484372572	-0.195991553	0.229802194	-0.852870675	0.393731026	0.617539563	ALLC	protein_coding
ENSG00000151364.17	261.5824101	-0.070216387	0.128232912	-0.547569154	0.583987774	0.766934942	KCTD14	protein_coding
ENSG00000151365.2	8.787658634	0.766292575	0.203206461	3.771004975	0.000162591	0.002895483	THRSP	protein_coding
ENSG00000151366.13	1514.888775	-0.152063441	0.084235008	-1.80522855	0.071038925	0.221879533	NDUFC2	protein_coding
ENSG00000151376.17	700.0347519	-0.153461851	0.076010866	-2.018946227	0.043492809	0.162559922	ME3	protein_coding
ENSG00000151379.4	1.644423916	-0.395124262	0.366902533	-1.076918871	0.281516511	0.510471248	MSGN1	protein_coding
ENSG00000151388.11	2060.365457	0.730182752	0.125219681	5.831213944	5.50256E-09	7.378E-07	ADAMTS12	protein_coding
ENSG00000151413.17	836.9707504	-0.237768985	0.059269366	-4.011667429	6.02914E-05	0.001334341	NUBPL	protein_coding
ENSG00000151414.15	4916.390233	0.128144335	0.053872997	2.378637553	0.017376752	0.08840686	NEK7	protein_coding
ENSG00000151418.12	0.760106648	-0.470720373	0.375732313	-1.252807801	0.210275662		ATP6V1G3	protein_coding
ENSG00000151422.13	1179.152388	0.172563375	0.082404095	2.094111641	0.036250036	0.144191371	FER	protein_coding
ENSG00000151445.16	1391.504664	0.024872522	0.035550429	0.699640543	0.484151816	0.692149146	VIPAS39	protein_coding
ENSG00000151458.12	2206.220473	0.356136509	0.071825552	4.95835396	7.10929E-07	3.96166E-05	ANKRD50	protein_coding
ENSG00000151461.20	4046.564547	-0.158350861	0.052278764	-3.028971001	0.002453882	0.022120983	UPF2	protein_coding
ENSG00000151465.14	2899.395511	-0.065006331	0.055577639	-1.169649013	0.242142245	0.46801351	CDC123	protein_coding
ENSG00000151466.12	576.1374958	-0.034054968	0.047772174	-0.712862022	0.475931142	0.686039211	SCLT1	protein_coding

ENSG00000151468.11	1744.628502	0.061301982	0.095722773	0.640411678	0.521904993	0.721635166	CCDC3	protein_coding
ENSG00000151470.13	824.5196131	-0.120143576	0.07481205	-1.605938834	0.10828735	0.288525702	C4orf33	protein_coding
ENSG00000151474.23	1075.097108	0.068964036	0.080550707	0.856156803	0.391911053	0.615865069	FRMD4A	protein_coding
ENSG00000151475.6	3.354489132	-0.334949919	0.294174849	-1.138608278	0.254866574	0.481752438	SLC25A31	protein_coding
ENSG00000151490.15	428.4227615	-0.684676696	0.156443395	-4.37651391	1.20592E-05	0.000376707	PTPRO	protein_coding
ENSG00000151491.14	7864.103717	0.124920701	0.078363681	1.594114759	0.110910334	0.292828817	EPS8	protein_coding
ENSG00000151498.12	1028.059141	-0.069420029	0.046860143	-1.481430141	0.138491988	0.335188301	ACAD8	protein_coding
ENSG00000151500.15	1559.123735	-0.215846289	0.053286836	-4.050649356	5.10757E-05	0.001169588	THYN1	protein_coding
ENSG00000151502.11	3442.964931	-0.081086452	0.042163653	-1.923136328	0.054462931	0.187912205	VPS26B	protein_coding
ENSG00000151503.13	3011.165846	-0.054445709	0.071896372	-0.757280346	0.448881923	0.663779822	NCAPD3	protein_coding
ENSG00000151532.14	1312.172981	-0.156698048	0.042344072	-3.700589993	0.000215099	0.003584459	VTI1A	protein_coding
ENSG00000151552.12	1422.903706	-0.124277316	0.06028131	-2.061622679	0.039243673	0.152147963	QDPR	protein_coding
ENSG00000151553.15	2073.740401	-0.074641261	0.050621499	-1.474497237	0.140347761	0.338027656	FAM160B1	protein_coding
ENSG00000151572.18	22.53079697	0.448307208	0.168049276	2.667712823	0.00763695	0.050059475	ANO4	protein_coding
ENSG00000151575.14	270.9315622	-0.257285186	0.100526535	-2.559375861	0.01048603	0.062336664	TEX9	protein_coding
ENSG00000151576.10	1595.4353	0.00771058	0.049521842	0.155700589	0.876269033	0.940196396	QTRT2	protein_coding
ENSG00000151577.13	2.400919172	-0.356847821	0.274516238	-1.299915163	0.193630048	0.410160246	DRD3	protein_coding
ENSG00000151611.16	750.0196551	-0.075353341	0.054024069	-1.394810538	0.16307298	0.37013549	MMAA	protein_coding
ENSG00000151612.18	2089.517623	0.102428684	0.08190329	1.250605253	0.211078533	0.431535033	ZNF827	protein_coding
ENSG00000151615.3	1.000602341	1.327943829	0.358026947	3.709061114	0.000208029	0.003496062	POU4F2	protein_coding
ENSG00000151617.17	1494.281238	0.163819528	0.112539312	1.455664919	0.145485272	0.345776477	EDNRA	protein_coding
ENSG00000151623.15	1051.9699	-0.03587163	0.116965051	-0.306686736	0.759081827	0.876683503	NR3C2	protein_coding
ENSG00000151631.8	5.542620794	-0.229117296	0.19284406	-1.188096205	0.234795509	0.459499884	AKR1C6P	unprocessed_pseudogene
ENSG00000151632.17	1002.735697	-0.749115227	0.204804071	-3.657716492	0.000254472	0.004101273	AKR1C2	protein_coding
ENSG00000151640.13	93.22567208	0.427969901	0.149886558	2.855292067	0.004299727	0.033386842	DPYSL4	protein_coding
ENSG00000151650.8	35.3665162	0.194346778	0.130596579	1.488146008	0.136712392	0.332747761	VENTX	protein_coding
ENSG00000151651.16	2405.492684	0.395422434	0.114484517	3.45393808	0.000552464	0.007328686	ADAM8	protein_coding
ENSG00000151655.19	151.7577718	-1.314625903	0.230014453	-5.715405635	1.09443E-08	1.29246E-06	ITIH2	protein_coding
ENSG00000151657.12	766.470908	-0.105941058	0.052404852	-2.021588729	0.043218857	0.162050262	KIN	protein_coding
ENSG00000151665.13	477.0577404	-0.103138869	0.060376923	-1.708249841	0.087589994	0.25402031	PIGF	protein_coding
ENSG00000151687.14	208.8973492	-0.128625013	0.071295958	-1.804099665	0.071215684	0.222160919	ANKAR	protein_coding
ENSG00000151689.13	1980.885133	0.109366274	0.079292962	1.379268375	0.167812023	0.376887314	INPP1	protein_coding
ENSG00000151690.15	5152.253812	0.171017154	0.063007586	2.714231154	0.00664298	0.045366106	MFSD6	protein_coding
ENSG00000151692.15	1405.26402	0.516830162	0.093873708	5.505590146	3.67934E-08	3.54722E-06	RNF144A	protein_coding
ENSG00000151693.11	3159.886279	0.284884644	0.078007957	3.651994684	0.000260211	0.00416577	ASAP2	protein_coding
ENSG00000151694.14	1494.62149	0.180221076	0.046910153	3.841835156	0.000122118	0.00230601	ADAM17	protein_coding
ENSG00000151702.17	1063.819916	-0.298144725	0.099134979	-3.007462418	0.002634387	0.023347301	FLI1	protein_coding
ENSG00000151704.16	5.096238527	-0.168246661	0.169300367	-0.993776116	0.320331877	0.549982356	KCNJ1	protein_coding
ENSG00000151715.8	7511.315199	-0.055344628	0.119870487	-0.461703538	0.644293933	0.807860872	TMEM45B	protein_coding
ENSG00000151718.16	1164.843199	0.204549507	0.089526666	2.284788601	0.022325226	0.104609289	WWC2	protein_coding
ENSG00000151725.12	1147.184158	-0.113615094	0.090651079	-1.253323131	0.210088134	0.430429234	CENPU	protein_coding
ENSG00000151726.15	3435.114809	0.278184411	0.085364257	3.258792607	0.001118874	0.012517891	ACSL1	protein_coding

ENSG00000151729.11	1848.610228	-0.137591825	0.099993468	-1.376008122	0.168819119	0.378058006	SLC25A4	protein_coding
ENSG00000151743.11	191.8726194	-0.015116891	0.07287567	-0.207433991	0.835670939	0.919467379	AMN1	protein_coding
ENSG00000151746.15	1121.073176	0.157626057	0.076318704	2.065366014	0.038888382	0.151254911	BICD1	protein_coding
ENSG00000151748.15	1832.110273	0.358553459	0.062426609	5.74359984	9.26847E-09	1.12505E-06	SAV1	protein_coding
ENSG00000151773.13	283.7998945	0.02097952	0.102060466	0.205559711	0.837134851	0.920004227	CCDC122	protein_coding
ENSG00000151778.11	106.9199141	0.036670535	0.137849247	0.266019115	0.790224488	0.894687118	SERP2	protein_coding
ENSG00000151779.13	2895.721904	0.026647804	0.048071288	0.554339285	0.579346669	0.763509037	NBAS	protein_coding
ENSG00000151789.12	218.9410231	-0.086940906	0.154202149	-0.563811248	0.572882596	0.758741256	ZNF385D	protein_coding
ENSG00000151790.9	231.5619323	-0.292343556	0.135029037	-2.165042141	0.030384458	0.128637281	TDO2	protein_coding
ENSG00000151806.14	2587.421924	-0.030485622	0.056081232	-0.543597586	0.586718422	0.768875851	GUF1	protein_coding
ENSG00000151812.15	7.044189883	0.017629925	0.178380629	0.098833183	0.921270721	0.962915133	SLC35F4	protein_coding
ENSG00000151834.16	242.1599934	0.233308655	0.215385576	1.083213926	0.27871348	0.507627573	GABRA2	protein_coding
ENSG00000151835.16	1732.684203	0.220794391	0.104928951	2.104227575	0.0353586	0.141710818	SACS	protein_coding
ENSG00000151838.12	9.481851275	-0.493271112	0.265949725	-1.854753232	0.063631473	0.207317287	CCDC175	protein_coding
ENSG00000151846.8	383.0670133	0.025651676	0.086145085	0.297772954	0.765876458	0.880968177	PABPC3	protein_coding
ENSG00000151849.15	878.6493206	0.084270263	0.073581259	1.145268022	0.252098148	0.478824958	CENPJ	protein_coding
ENSG00000151876.13	637.8199167	-0.233273667	0.065928741	-3.538269704	0.000402758	0.005775857	FBXO4	protein_coding
ENSG00000151881.15	1147.174428	-0.068966733	0.066711561	-1.033804826	0.301227415	0.530817545	TMEM267	protein_coding
ENSG00000151882.11	2058.673288	-0.060687687	0.164290018	-0.369393635	0.711834341	0.849293755	CCL28	protein_coding
ENSG00000151883.19	2181.466116	0.079372308	0.065984977	1.202884536	0.229021005	0.452550309	PARP8	protein_coding
ENSG00000151892.15	1011.385622	-0.548028551	0.203477443	-2.693313531	0.007074571	0.047312548	GFRA1	protein_coding
ENSG00000151893.15	4065.494391	0.032462351	0.045444602	0.714327993	0.475024387	0.685294145	CACUL1	protein_coding
ENSG00000151914.20	11126.07534	0.367938215	0.108768542	3.382763149	0.000717605	0.008914216	DST	protein_coding
ENSG00000151917.18	115.0211187	0.404153375	0.130187973	3.104383345	0.001906761	0.018482969	BEND6	protein_coding
ENSG00000151923.17	4456.385414	-0.08523662	0.03488267	-2.443523375	0.014544627	0.078386308	TIAL1	protein_coding
ENSG00000151929.10	5311.261627	0.2333753	0.089757894	2.600053201	0.009320931	0.057524176	BAG3	protein_coding
ENSG00000151948.12	53.05200803	0.727568887	0.140348421	5.184019055	2.17155E-07	1.50924E-05	GLT1D1	protein_coding
ENSG00000151952.16	15.27140501	-0.411224297	0.211131284	-1.947718451	0.051448661	0.181199249	TMEM132D	protein_coding
ENSG00000151962.8	6.732361592	-0.139011712	0.273197648	-0.508832024	0.61086997	0.78619479	RBM46	protein_coding
ENSG00000151963.4	2.088764617	-0.552884634	0.223273098	-2.476270715	0.013276287	0.073643171	ZNF37CP	unprocessed_pseudogene
ENSG00000151967.18	50.57537249	0.106330677	0.126779975	0.838702461	0.401636297	0.623695175	SCHIP1	protein_coding
ENSG00000152034.11	6.133495894	0.20467199	0.31608597	0.647520011	0.517295448	0.718566711	MCHR2	protein_coding
ENSG00000152049.6	626.9640056	0.193080466	0.133095443	1.45069178	0.146865705	0.347729793	KCNE4	protein_coding
ENSG00000152056.17	915.4970926	0.002767199	0.09892584	0.027972456	0.97768412	0.989997464	AP1S3	protein_coding
ENSG00000152061.23	4566.979963	-0.191129995	0.069485567	-2.750643108	0.005947841	0.042069314	RABGAP1L	protein_coding
ENSG00000152076.19	65.86954473	0.470377267	0.143597954	3.275654393	0.001054175	0.011938091	CCDC74B	protein_coding
ENSG00000152078.10	1012.405054	0.199432379	0.111640517	1.786379925	0.074037764	0.228090748	TLCD4	protein_coding
ENSG00000152082.15	1343.737811	-0.206118577	0.075302884	-2.737193649	0.00619658	0.043232211	MZT2B	protein_coding
ENSG00000152086.9	7.848527597	0.118352508	0.172683709	0.68537159	0.493109467	0.700079209	TUBA3E	protein_coding
ENSG00000152092.16	54.41118053	0.072941429	0.231183191	0.31551355	0.752371765	0.872903145	ASTN1	protein_coding
ENSG00000152093.8	0.327282702	-0.190926632	0.469504083	-0.406655956	0.684260693		CFC1B	protein_coding
ENSG00000152102.18	5981.417501	0.022484656	0.051751224	0.434475834	0.66394294	0.820365246	FAM168B	protein_coding

ENSG00000152104.12	3281.790457	0.092029624	0.071936484	1.279317787	0.200785171	0.419384337	PTPN14	protein_coding
ENSG00000152117.17	1208.440452	0.029793215	0.060675754	0.491023401	0.623409897	0.794576977	SMPD4BP	transcribed_processed_pseudogene
ENSG00000152127.9	7081.618597	-0.127081288	0.078139155	-1.626345825	0.103876108	0.281773528	MGAT5	protein_coding
ENSG00000152128.13	569.469316	-0.342029616	0.157927463	-2.165738678	0.030331162	0.128475566	TMEM163	protein_coding
ENSG00000152133.16	890.3448567	-0.096392736	0.041557101	-2.31952501	0.020366587	0.098426832	GPATCH11	protein_coding
ENSG00000152137.8	2324.087689	-0.328556575	0.191977655	-1.71143134	0.087001516	0.252818422	HSPB8	protein_coding
ENSG00000152147.11	674.3413544	-0.246811854	0.052303215	-4.718865832	2.37163E-06	0.000103349	GEMIN6	protein_coding
ENSG00000152154.11	75.603627	0.416257862	0.131280612	3.170748936	0.001520465	0.015685914	TMEM178A	protein_coding
ENSG00000152192.8	17.69915646	0.882318093	0.287964606	3.063981043	0.002184127	0.020403054	POU4F1	protein_coding
ENSG00000152193.8	603.0286493	0.003509179	0.063878644	0.054935091	0.956190175	0.979614418	OBI1	protein_coding
ENSG00000152207.7	89.74774616	-0.454065255	0.126067667	-3.601758212	0.000316072	0.004829411	CYSLTR2	protein_coding
ENSG00000152208.13	25.02573638	0.036581584	0.229631486	0.159305611	0.8734281	0.938755898	GRID2	protein_coding
ENSG00000152213.4	194.3907141	0.248239769	0.122027468	2.034294179	0.041921944	0.158799769	ARL11	protein_coding
ENSG00000152214.14	1.398361272	-0.356955733	0.362541914	-0.984591626	0.324824705	0.554615083	RIT2	protein_coding
ENSG00000152217.20	1224.299125	-0.247984637	0.140316526	-1.767323088	0.077174135	0.234667316	SETBP1	protein_coding
ENSG00000152219.5	1062.61904	-0.112725176	0.04576021	-2.463388503	0.013763069	0.075507148	ARL14EP	protein_coding
ENSG00000152223.15	2398.668907	-0.072313151	0.061604379	-1.173831346	0.240462586	0.465862184	EPG5	protein_coding
ENSG00000152229.18	868.0317442	-0.022351039	0.121516925	-0.183933543	0.854065591	0.928791801	PSTPIP2	protein_coding
ENSG00000152234.16	20394.27405	-0.205870882	0.066634668	-3.08954614	0.002004626	0.019172664	ATP5F1A	protein_coding
ENSG00000152240.13	818.058963	-0.197679429	0.06825536	-2.896174427	0.003777424	0.030449278	HAUS1	protein_coding
ENSG00000152242.11	1811.109195	0.009832468	0.060146104	0.163476388	0.870143363	0.93722471	C18orf25	protein_coding
ENSG00000152253.9	616.5602393	-0.088416199	0.085272967	-1.036860834	0.299800719	0.529481071	SPC25	protein_coding
ENSG00000152254.11	3.843902474	-0.150216303	0.227031226	-0.661654812	0.508192471	0.711890939	G6PC2	protein_coding
ENSG00000152256.14	1551.014309	-0.095449807	0.072711519	-1.312719199	0.189277586	0.404831866	PDK1	protein_coding
ENSG00000152266.7	1.493106041	-0.483758361	0.395531154	-1.223060069	0.221307009	0.443723913	PTH	protein_coding
ENSG00000152270.9	924.6658982	0.103686175	0.108279526	0.957578768	0.33827521	0.568054675	PDE3B	protein_coding
ENSG00000152284.5	892.4069716	0.064623077	0.112828594	0.572754425	0.566810981	0.754652569	TCF7L1	protein_coding
ENSG00000152291.14	21415.77505	-0.0227769	0.048280012	-0.471766665	0.637093347	0.803458486	TGOLN2	protein_coding
ENSG00000152292.17	28.85508909	-0.021156243	0.196142239	-0.107861741	0.914105366	0.959368031	SH2D6	protein_coding
ENSG00000152315.5	65.45494301	0.023112051	0.120969421	0.19105697	0.84848096	0.925700828	KCNK13	protein_coding
ENSG00000152332.16	8484.723632	0.161934774	0.05970804	2.712110026	0.00668564	0.045521894	UHMK1	protein_coding
ENSG00000152348.16	327.6753571	-0.148603224	0.054224922	-2.740496793	0.006134638	0.042958239	ATG10	protein_coding
ENSG00000152359.15	593.389757	0.019323335	0.045252834	0.427008274	0.669373301	0.823735007	POC5	protein_coding
ENSG00000152377.14	2450.291574	0.555302874	0.177659298	3.125661757	0.001774055	0.01754607	SPOCK1	protein_coding
ENSG00000152380.10	83.8535533	0.04612295	0.063640889	0.724737666	0.468612983	0.680034232	FAM151B	protein_coding
ENSG00000152382.6	1059.579888	0.016925329	0.042810946	0.395350506	0.692584231	0.837699816	TADA1	protein_coding
ENSG00000152402.11	515.2175546	0.164360184	0.105670663	1.555400329	0.119850756	0.307292091	GUCY1A2	protein_coding
ENSG00000152404.15	1432.905941	-0.204471173	0.065819544	-3.106541927	0.001892895	0.018381765	CWF19L2	protein_coding
ENSG00000152409.9	1851.85507	0.072720275	0.071123766	1.022446917	0.306569425	0.536014445	JMY	protein_coding
ENSG00000152413.15	676.9561274	0.127580398	0.084503709	1.509760919	0.131104441	0.324338931	HOMER1	protein_coding
ENSG00000152422.16	637.0501342	0.076443909	0.084907345	0.900321511	0.367949177	0.595002138	XRCC4	protein_coding
ENSG00000152430.18	18.47675879	0.423281829	0.182842478	2.315008161	0.020612485	0.099186206	BOLL	protein_coding

ENSG00000152433.15	107.7271854	-0.042106942	0.082950958	-0.507612491	0.611725129	0.786810683	ZNF547	protein_coding
ENSG00000152439.13	190.062821	0.100008286	0.091539659	1.092513199	0.274607592	0.503283637	ZNF773	protein_coding
ENSG00000152443.13	848.6861554	0.053171166	0.062349989	0.852785504	0.393778264	0.617539563	ZNF776	protein_coding
ENSG00000152454.4	170.2557242	0.173798306	0.128623114	1.351221414	0.176624517	0.388787943	ZNF256	protein_coding
ENSG00000152455.16	794.4059377	-0.114749425	0.063019225	-1.82086381	0.068627562	0.217149525	SUV39H2	protein_coding
ENSG00000152457.18	2113.779416	-0.171728488	0.059010332	-2.910142734	0.003612637	0.029480879	DCLRE1C	protein_coding
ENSG00000152463.15	15.59411605	-0.266632821	0.185083532	-1.440608025	0.149695452	0.351791981	OLAH	protein_coding
ENSG00000152464.15	611.8973958	-0.19356086	0.057474426	-3.36777369	0.000757778	0.009315001	RPP38	protein_coding
ENSG00000152465.18	1044.035255	-0.153090325	0.058787263	-2.60414104	0.009210485	0.057052635	NMT2	protein_coding
ENSG00000152467.10	8.15639219	-0.042975864	0.181235858	-0.237126717	0.812558486	0.9074309	ZSCAN1	protein_coding
ENSG00000152475.7	181.1321983	-0.065489742	0.082175152	-0.796953093	0.425478274	0.644279198	ZNF837	protein_coding
ENSG00000152484.14	2898.044676	0.028434416	0.068758626	0.413539627	0.679211296	0.8300519	USP12	protein_coding
ENSG00000152492.15	4624.980379	-0.041786645	0.064839987	-0.644457943	0.519278521	0.719836975	CCDC50	protein_coding
ENSG00000152495.11	356.3920227	-0.325045262	0.114781756	-2.831854754	0.004627887	0.035238408	CAMK4	protein_coding
ENSG00000152503.10	463.3319959	-0.201830896	0.13299742	-1.517554969	0.129126626	0.321444339	TRIM36	protein_coding
ENSG00000152518.8	26921.74173	0.12592191	0.074882968	1.681582787	0.092649771	0.262885731	ZFP36L2	protein_coding
ENSG00000152520.14	2717.861068	0.053881197	0.056188845	0.958930488	0.337593765	0.567347608	PAN3	protein_coding
ENSG00000152527.14	857.5672545	0.251529834	0.124001886	2.028435554	0.042515815	0.160255311	PLEKHH2	protein_coding
ENSG00000152556.17	2454.098781	-0.021872737	0.067998959	-0.321662822	0.747708146	0.870179525	PFKM	protein_coding
ENSG00000152558.15	19179.12106	0.024301229	0.057946584	0.419372942	0.674943596	0.827435353	TMEM123	protein_coding
ENSG00000152578.13	29.71273468	-0.581378901	0.176841853	-3.287563956	0.001010582	0.011582889	GRIA4	protein_coding
ENSG00000152580.8	195.0646041	0.353982745	0.182380721	1.940900018	0.052270406	0.182852009	IGSF10	protein_coding
ENSG00000152582.14	336.0501117	0.218510014	0.114360169	1.910717836	0.056040851	0.191371537	SPEF2	protein_coding
ENSG00000152583.12	18017.70278	-0.252284226	0.157924416	-1.597499822	0.110154337	0.291486249	SPARCL1	protein_coding
ENSG00000152591.15	5.865919354	1.10519479	0.264945074	4.171410981	3.02719E-05	0.000768741	DSPP	protein_coding
ENSG00000152592.14	14.08654496	0.485972241	0.221054418	2.198428086	0.027918614	0.121442348	DMP1	protein_coding
ENSG00000152595.16	2.840856121	0.615114979	0.292061434	2.106115042	0.035194361	0.141299488	MEPE	protein_coding
ENSG00000152601.17	11258.65239	-0.157789586	0.057225741	-2.757318355	0.005827758	0.041475783	MBNL1	protein_coding
ENSG00000152611.12	2.141394301	0.858609178	0.300306547	2.859109094	0.004248327	0.033070619	CAPSL	protein_coding
ENSG00000152620.13	1623.454639	-0.006431536	0.068566415	-0.093800094	0.925267957	0.96471831	NADK2	protein_coding
ENSG00000152642.11	2916.527725	-0.159571316	0.08121209	-1.964871423	0.049429121	0.176642021	GPD1L	protein_coding
ENSG00000152661.9	3686.171022	0.266380543	0.11840533	2.249734394	0.024465811	0.11137017	GJA1	protein_coding
ENSG00000152669.9	478.542014	-0.172228968	0.117881103	-1.461039669	0.144004555	0.343390788	CCNO	protein_coding
ENSG00000152670.19	14.33856368	-0.663495305	0.278652368	-2.381086188	0.017261673	0.088050089	DDX4	protein_coding
ENSG00000152672.8	17.05212564	-0.370494921	0.171327632	-2.162493682	0.030580144	0.129189618	CLEC4F	protein_coding
ENSG00000152683.14	2950.953862	0.021076	0.045981766	0.458355596	0.646696987	0.809383498	SLC30A6	protein_coding
ENSG00000152684.11	1331.095528	0.148463135	0.048284716	3.074743871	0.002106834	0.019872187	PELO	protein_coding
ENSG00000152689.18	1004.642413	-0.158820527	0.078149756	-2.032258775	0.042127466	0.15937048	RASGRP3	protein_coding
ENSG00000152700.14	4208.39294	-0.137708081	0.056041576	-2.457248548	0.014000578	0.076377467	SAR1B	protein_coding
ENSG00000152705.8	21.9892997	-0.02001265	0.090861287	-0.220254972	0.825672586	0.91392541	CATSPER3	protein_coding
ENSG00000152749.8	1501.451791	0.318374297	0.066244556	4.806044693	1.53945E-06	7.29183E-05	GPR180	protein_coding
ENSG00000152760.10	35.60491174	0.202515423	0.10788657	1.877114292	0.060502431	0.200849555	TCTEX1D1	protein_coding

ENSG00000152763.17	144.2064948	-0.13308527	0.092313913	-1.441659935	0.149398331	0.351434876	DNAI4	protein_coding
ENSG00000152766.6	2273.054842	-0.194398709	0.122369922	-1.588615122	0.112147312	0.294862038	ANKRD22	protein_coding
ENSG00000152767.17	4893.516803	0.329914958	0.089001883	3.706831211	0.000209869	0.003517735	FARP1	protein_coding
ENSG00000152778.9	1497.224716	0.000651362	0.070541262	0.009233773	0.992632619	0.996542454	IFIT5	protein_coding
ENSG00000152779.14	23.88304925	-0.32855549	0.150696027	-2.180253165	0.029238701	0.125345289	SLC16A12	protein_coding
ENSG00000152782.16	856.5889073	-0.32707728	0.088020739	-3.715911562	0.000202472	0.003424562	PANK1	protein_coding
ENSG00000152784.16	329.8678137	0.323516009	0.138246667	2.340136042	0.019276717	0.094565066	PRDM8	protein_coding
ENSG00000152785.8	295.0228567	-0.332728356	0.270965191	-1.227937635	0.21947038	0.44130033	BMP3	protein_coding
ENSG00000152795.17	10115.24512	0.006752396	0.041593836	0.162341262	0.871037124	0.937720434	HNRNPDL	protein_coding
ENSG00000152804.11	889.2889516	-0.203620788	0.12480418	-1.631522181	0.102780183	0.280103318	HHEX	protein_coding
ENSG00000152818.18	5867.432916	-0.0612161	0.090882147	-0.673576736	0.500580447	0.705859948	UTRN	protein_coding
ENSG00000152822.14	11.25708222	-0.043301861	0.154901508	-0.279544479	0.779827008	0.888584375	GRM1	protein_coding
ENSG00000152894.15	8725.638735	0.250164113	0.071270528	3.510063989	0.000447999	0.006263217	PTPRK	protein_coding
ENSG00000152904.11	952.0322691	-0.048513758	0.054589766	-0.888696935	0.374165979	0.600023215	GGPS1	protein_coding
ENSG00000152910.19	5.960148358	0.275588546	0.382382654	0.720714036	0.471085475	0.682213676	CNTNAP4	protein_coding
ENSG00000152926.15	1476.416297	0.39280193	0.125460142	3.130890212	0.001742773	0.017346921	ZNF117	protein_coding
ENSG00000152931.9	221.500022	-0.561163847	0.214691023	-2.613820734	0.0089536	0.055866414	PART1	lncRNA
ENSG00000152932.8	281.1417417	-0.280387409	0.192476825	-1.45673334	0.145190003	0.345387843	RAB3C	protein_coding
ENSG00000152936.10	7.199452759	-0.069071937	0.299472122	-0.230645631	0.817590115	0.910099918	LMNTD1	protein_coding
ENSG00000152939.17	2306.199872	0.075399048	0.069794123	1.080306538	0.280005699	0.50876906	MARVELD2	protein_coding
ENSG00000152942.19	1383.459106	-0.083843643	0.04071822	-2.059118582	0.03948288	0.152801551	RAD17	protein_coding
ENSG00000152944.9	2105.768121	0.121770779	0.069156383	1.76080317	0.078271723	0.236831473	MED21	protein_coding
ENSG00000152952.12	3672.81215	0.451997836	0.091346203	4.948184186	7.4909E-07	4.12869E-05	PLOD2	protein_coding
ENSG00000152953.13	171.6128671	0.15344714	0.133065077	1.153173651	0.248839116	0.475536647	STK32B	protein_coding
ENSG00000152954.12	12.66186036	-0.279528329	0.257395637	-1.085987053	0.27748472	0.50638434	NRSN1	protein_coding
ENSG00000152969.20	6.291776402	-0.63802211	0.158323468	-4.029864422	5.5809E-05	0.001251668	JAKMIP1	protein_coding
ENSG00000152977.10	27.26716698	0.656119473	0.322496945	2.03449826	0.041901384	0.158749487	ZIC1	protein_coding
ENSG00000152990.14	2218.81614	0.076328896	0.073263804	1.041836387	0.297487541	0.527394035	ADGRA3	protein_coding
ENSG00000153002.12	31.38361197	-1.851304533	0.243784336	-7.594025782	3.10117E-14	2.41321E-11	CPB1	protein_coding
ENSG00000153006.16	3034.880453	-0.074257213	0.062160932	-1.194596189	0.232244839	0.456496768	SREK1IP1	protein_coding
ENSG00000153012.12	766.2709461	-0.047424257	0.154216914	-0.307516574	0.758450209	0.876404985	LGI2	protein_coding
ENSG00000153015.16	1155.623414	-0.029863656	0.044961593	-0.664203687	0.506559954	0.710609166	CWC27	protein_coding
ENSG00000153029.14	1566.602383	-0.022928604	0.079834139	-0.287203002	0.773956888	0.885617772	MR1	protein_coding
ENSG00000153037.15	719.8677115	-0.020120055	0.05074786	-0.396470999	0.691757598	0.837532562	SRP19	protein_coding
ENSG00000153044.10	553.2711846	-0.328230262	0.080113877	-4.097046263	4.18455E-05	0.001000276	CENPH	protein_coding
ENSG00000153046.18	1684.29104	0.202002053	0.041344478	4.885829125	1.02994E-06	5.31147E-05	CDYL	protein_coding
ENSG00000153048.11	2250.316806	0.115903184	0.069504072	1.667574006	0.095400322	0.267392487	CARHSP1	protein_coding
ENSG00000153060.8	19.47408138	-0.519985694	0.17960042	-2.89523651	0.00378873	0.030495284	TEKT5	protein_coding
ENSG00000153064.12	715.8322227	-0.579741361	0.156549099	-3.703255813	0.00021285	0.003552419	BANK1	protein_coding
ENSG00000153066.13	3699.962037	-0.080745305	0.057553868	-1.402951849	0.160631198	0.36679028	TXNDC11	protein_coding
ENSG00000153071.15	4143.228769	0.140090324	0.091386968	1.532935455	0.125291759	0.315590174	DAB2	protein_coding
ENSG00000153086.14	44.40772518	-0.54709808	0.199016091	-2.749014294	0.005977478	0.042210429	ACMSD	protein_coding

ENSG00000153093.20	140.9558116	0.207344368	0.088650421	2.338898858	0.019340669	0.094740902	ACOXL	protein_coding
ENSG00000153094.24	1788.13168	0.036290735	0.064388379	0.563622427	0.573011121	0.758763265	BCL2L11	protein_coding
ENSG00000153107.13	2207.505613	-0.024760598	0.058936916	-0.420120356	0.674397533	0.826998573	ANAPC1	protein_coding
ENSG00000153113.24	11226.73056	0.077358157	0.064278677	1.203480859	0.228790294	0.452297539	CAST	protein_coding
ENSG00000153130.17	3097.005804	0.093779874	0.082259362	1.140051069	0.254265026	0.481078399	SCOC	protein_coding
ENSG00000153132.13	124.4978179	0.305057415	0.190291557	1.603105364	0.108911394	0.289445131	CLGN	protein_coding
ENSG00000153140.9	638.184101	-0.114882467	0.060404872	-1.901874195	0.057187604	0.194042325	CETN3	protein_coding
ENSG00000153147.6	6111.313105	0.092115385	0.04612312	1.997162894	0.045807491	0.168256785	SMARCA5	protein_coding
ENSG00000153157.13	31.33706834	-0.163276381	0.192294063	-0.849097361	0.395827119	0.619093265	SYCP2L	protein_coding
ENSG00000153162.9	263.0050229	0.015312287	0.127810658	0.119804462	0.904638047	0.954520339	BMP6	protein_coding
ENSG00000153165.19	35.90439609	-0.114944256	0.093869034	-1.2245173	0.220757144	0.442906725	RGPD3	protein_coding
ENSG00000153179.13	3737.007732	-0.020548912	0.082263271	-0.249794488	0.802746282	0.902020957	RASSF3	protein_coding
ENSG00000153187.20	25998.62916	0.014901866	0.037252194	0.400026536	0.689136972	0.835774455	HNRNPU	protein_coding
ENSG00000153201.16	8392.380291	0.033047639	0.054267556	0.608975993	0.542540352	0.737541412	RANBP2	protein_coding
ENSG00000153207.16	4155.457807	0.100613025	0.0531833	1.891816123	0.058515484	0.196647587	AHCTF1	protein_coding
ENSG00000153208.17	985.6202143	-0.129297865	0.093460959	-1.383442523	0.16652922	0.374972552	MERTK	protein_coding
ENSG00000153214.11	5319.89693	-0.020148353	0.05706717	-0.35306383	0.724040589	0.855936106	TMEM87B	protein_coding
ENSG00000153230.4	0.328451669	0.010172292	0.523082551	0.019446819	0.984484661		OR14K1	protein_coding
ENSG00000153233.13	515.7081191	0.282856386	0.165938707	1.70458352	0.088272127	0.255200324	PTPRR	protein_coding
ENSG00000153234.15	2251.485501	0.631345782	0.135415754	4.662277211	3.1273E-06	0.000127961	NR4A2	protein_coding
ENSG00000153237.18	50.14976716	-0.23054902	0.12501278	-1.844203607	0.065153473	0.210326164	CCDC148	protein_coding
ENSG00000153246.13	900.8045521	0.391015617	0.114712448	3.408659005	0.00065283	0.008303673	PLA2R1	protein_coding
ENSG00000153250.20	3634.945127	0.220427434	0.087559696	2.517453173	0.011820668	0.067840018	RBMS1	protein_coding
ENSG00000153253.19	108.1943787	-0.197637303	0.145214368	-1.361003776	0.173512496	0.38457627	SCN3A	protein_coding
ENSG00000153266.13	5.397990387	-0.036463193	0.308020103	-0.118379264	0.905767155	0.95514992	FEZF2	protein_coding
ENSG00000153283.13	808.6480324	-0.557888034	0.123188747	-4.528725621	5.93405E-06	0.000215311	CD96	protein_coding
ENSG00000153291.16	199.8140111	0.05929751	0.14757506	0.401812546	0.687821988	0.835117962	SLC25A27	protein_coding
ENSG00000153292.16	733.5589467	1.059188395	0.217949316	4.85979224	1.17509E-06	5.92672E-05	ADGRF1	protein_coding
ENSG00000153294.12	260.1787851	0.499907119	0.204737548	2.441697304	0.014618398	0.078693752	ADGRF4	protein_coding
ENSG00000153303.17	120.3997159	-0.354786161	0.228397223	-1.553373354	0.120333965	0.308077856	FRMD1	protein_coding
ENSG00000153310.19	4817.577871	-0.070354391	0.060611292	-1.16074727	0.245744692	0.47224479	CYRIB	protein_coding
ENSG00000153317.15	3773.916395	0.211265802	0.069031162	3.060441072	0.002210112	0.020530819	ASAP1	protein_coding
ENSG00000153339.15	3063.957687	-0.084862291	0.057142211	-1.485106888	0.137515512	0.33393778	TRAPPC8	protein_coding
ENSG00000153347.10	11.55241274	0.342822809	0.205281504	1.670013136	0.094916765	0.26643828	FAM81B	protein_coding
ENSG00000153363.13	638.8497282	-0.002169737	0.079210557	-0.027392021	0.978147062	0.990259142	LINC00467	lncRNA
ENSG00000153391.16	615.1205091	-0.099468659	0.075800712	-1.312239114	0.189439468	0.404924889	INO80C	protein_coding
ENSG00000153395.10	4641.980658	0.28072745	0.089544725	3.135052908	0.001718231	0.017157507	LPCAT1	protein_coding
ENSG00000153404.14	152.1991295	1.20655085	0.203763053	5.921342627	3.19324E-09	4.79271E-07	PLEKHG4B	protein_coding
ENSG00000153406.14	2106.223666	-0.127498405	0.060390221	-2.111242555	0.034751469	0.140090362	NMRAL1	protein_coding
ENSG00000153443.13	2346.54375	-0.010278666	0.060317368	-0.170409721	0.864687928	0.934142495	UBALD1	protein_coding
ENSG00000153446.16	276.7465179	-0.41754525	0.202507322	-2.0618773	0.039219419	0.152092608	C16orf89	protein_coding
ENSG00000153485.6	459.4894511	-0.069748622	0.051917976	-1.34343877	0.17912994	0.391982796	TMEM251	protein_coding

ENSG00000153487.13	1553.398483	0.145123819	0.05316594	2.729638916	0.006340373	0.043943999	ING1	protein_coding
ENSG00000153495.11	10.52572224	0.188210135	0.124205453	1.515312982	0.129693162	0.32222322	TEX29	protein_coding
ENSG00000153498.12	1.187790598	-0.37438529	0.383753492	-0.975587969	0.329268689	0.558767988	SPACA7	protein_coding
ENSG00000153531.13	451.7728529	0.296623932	0.104363607	2.842216171	0.00448011	0.034456365	ADPRHL1	protein_coding
ENSG00000153551.14	1995.314343	0.109571567	0.098842561	1.10854641	0.267625907	0.495535746	CMTM7	protein_coding
ENSG00000153558.16	332.8313195	0.463588097	0.103375662	4.484499451	7.30853E-06	0.000253855	FBXL2	protein_coding
ENSG00000153560.12	5047.9388	0.064523663	0.04553487	1.417016523	0.156478097	0.361359089	UBP1	protein_coding
ENSG00000153561.13	4865.490955	0.101851943	0.056116399	1.815012093	0.069522051	0.218772561	RMND5A	protein_coding
ENSG00000153563.16	1151.505007	-0.592459457	0.150883923	-3.926591008	8.61583E-05	0.001770148	CD8A	protein_coding
ENSG00000153574.9	1646.294821	-0.210493636	0.070535717	-2.984213452	0.002843084	0.024622383	RPIA	protein_coding
ENSG00000153684.15	1.603009858	-0.350895428	0.245858637	-1.42722433	0.153515244	0.357166779	GOLGA8F	protein_coding
ENSG00000153707.17	290.6487988	0.293223956	0.155573065	1.884798988	0.059456982	0.198688765	PTPRD	protein_coding
ENSG00000153714.6	1468.138206	0.197498537	0.095677815	2.064204093	0.03899837	0.151532051	LURAP1L	protein_coding
ENSG00000153721.19	1186.288809	-0.063339424	0.088485901	-0.715813743	0.474106366	0.684677153	CNKSR3	protein_coding
ENSG00000153767.10	704.6258119	-0.054269853	0.042001192	-1.292102688	0.196321585	0.41350987	GTF2E1	protein_coding
ENSG00000153774.9	1252.062248	-0.159175607	0.061250545	-2.598762315	0.009356053	0.05764297	CFDP1	protein_coding
ENSG00000153779.11	1.681374903	1.874314194	0.483847693	3.873768997	0.000107165	0.002089454	TGIF2LX	protein_coding
ENSG00000153786.13	4605.990569	0.051714934	0.062931093	0.821770791	0.411207361	0.632114546	ZDHHC7	protein_coding
ENSG00000153789.13	16.80792999	-0.274907561	0.180850935	-1.520078185	0.128491327	0.320269206	CIBAR2	protein_coding
ENSG00000153790.12	517.5084448	-0.480943142	0.090698655	-5.302649108	1.14134E-07	8.91331E-06	C7orf31	protein_coding
ENSG00000153802.12	22.58226148	-0.340402151	0.286871523	-1.186601399	0.235384884	0.460198622	TMPRSS11D	protein_coding
ENSG00000153814.13	768.9095287	-0.069641528	0.081666637	-0.85275372	0.393795893	0.617539563	JAZF1	protein_coding
ENSG00000153815.16	12926.15385	0.078792888	0.05711702	1.379499287	0.167740866	0.376859566	CMIP	protein_coding
ENSG00000153820.13	8.817265122	-0.267044552	0.286711276	-0.931405824	0.351643679	0.580502946	SPHKAP	protein_coding
ENSG00000153822.14	126.2383837	-0.726870775	0.247056534	-2.942123262	0.003259701	0.027332397	KCNJ16	protein_coding
ENSG00000153823.19	791.7834597	-0.023933217	0.142810239	-0.167587541	0.866907773	0.935497004	PID1	protein_coding
ENSG00000153827.14	11125.09434	0.09533953	0.043669372	2.183212776	0.029020144	0.124813075	TRIP12	protein_coding
ENSG00000153832.12	246.4069459	-0.083238625	0.080372629	-1.035658854	0.300361324	0.529963131	FBXO36	protein_coding
ENSG00000153879.9	3806.698949	-0.027673816	0.066509769	-0.416086476	0.677346726	0.829145152	CEBPG	protein_coding
ENSG00000153885.14	1309.370591	0.287841322	0.103623059	2.777772875	0.005473286	0.03961292	KCTD15	protein_coding
ENSG00000153896.19	235.1034738	-0.11434798	0.079055387	-1.446428687	0.148057	0.349597808	ZNF599	protein_coding
ENSG00000153898.13	225.6260134	-0.28372656	0.121129147	-2.34234755	0.019162861	0.094218661	MCOLN2	protein_coding
ENSG00000153902.14	227.9289642	-0.088090482	0.130736309	-0.673802735	0.500436736	0.705791042	LGI4	protein_coding
ENSG00000153904.21	9767.611633	-0.228368934	0.077234494	-2.956825654	0.003108238	0.02639277	DDAH1	protein_coding
ENSG00000153914.16	4570.173832	0.005106734	0.054166537	0.094278383	0.924888021	0.964645301	SREK1	protein_coding
ENSG00000153922.11	3292.087656	0.03413313	0.053448555	0.63861651	0.523072438	0.722492873	CHD1	protein_coding
ENSG00000153923.10	10.81589086	0.451812537	0.202814125	2.227717304	0.025899372	0.115636983	CLCA3P	transcribed_unprocessed_pseudogene
ENSG00000153930.12	78.22675289	0.316133114	0.198371218	1.59364406	0.11101578	0.293000705	ANKFN1	protein_coding
ENSG00000153933.10	918.7273856	0.062169332	0.066219482	0.938837494	0.347814185	0.577090266	DGKE	protein_coding
ENSG00000153936.17	2732.455703	-0.112990492	0.057299343	-1.971933476	0.048617201	0.174815725	HS2ST1	protein_coding
ENSG00000153944.12	6180.563926	0.124880839	0.063374774	1.970513351	0.048779566	0.175139422	MSI2	protein_coding
ENSG00000153956.16	1336.369958	-0.156513947	0.144445311	-1.083551598	0.278563661	0.507418328	CACNA2D1	protein_coding

ENSG00000153975.10	535.9482744	-0.033760338	0.054246721	-0.622348004	0.533713062	0.730980546	ZUP1	protein_coding
ENSG00000153976.3	192.2342215	0.502788776	0.125456828	4.007663707	6.13224E-05	0.001352487	HS3ST3A1	protein_coding
ENSG00000153982.11	427.8570853	-0.310704037	0.093164463	-3.335005922	0.000852976	0.010158548	GDPD1	protein_coding
ENSG00000153989.8	2480.319967	0.10259807	0.045215584	2.26908649	0.023263067	0.107695173	NUS1	protein_coding
ENSG00000153993.13	244.6051388	0.875923183	0.182159235	4.808557651	1.52023E-06	7.23222E-05	SEMA3D	protein_coding
ENSG00000154001.14	3808.387271	0.052955182	0.035190697	1.504806307	0.132373882	0.326173469	PPP2R5E	protein_coding
ENSG00000154007.7	1.164388909	-0.224761504	0.44477442	-0.505338198	0.613321318	0.787712488	ASB17	protein_coding
ENSG00000154016.14	49.77330583	-0.44315251	0.130939162	-3.384415342	0.0007133	0.008878457	GRAP	protein_coding
ENSG00000154025.16	17.87023028	-0.398913792	0.135561221	-2.942683685	0.003253807	0.027293772	SLC5A10	protein_coding
ENSG00000154027.19	60.44102116	-0.17637734	0.153148542	-1.151674953	0.249454668	0.476190909	AK5	protein_coding
ENSG00000154040.21	121.9409724	0.025220817	0.134446138	0.187590493	0.851197679	0.927101312	CABYR	protein_coding
ENSG00000154059.11	1823.911419	-0.14541081	0.078423815	-1.854166488	0.063715345	0.207481959	IMPACT	protein_coding
ENSG00000154065.17	531.4205235	-0.356522058	0.143990395	-2.476012775	0.013285882	0.073668265	ANKRD29	protein_coding
ENSG00000154079.6	397.3985061	-0.061228906	0.062472567	-0.980092692	0.327040366	0.556725964	SDHAF4	protein_coding
ENSG00000154080.14	101.4213704	-0.260621749	0.288801946	-0.902423798	0.366831784	0.593856477	CHST9	protein_coding
ENSG00000154096.14	6719.168623	0.357372418	0.111711802	3.199056977	0.001378779	0.014682768	THY1	protein_coding
ENSG00000154099.18	33.1432551	-0.031446804	0.121649042	-0.258504331	0.796017707	0.897879624	DNAAF1	protein_coding
ENSG00000154102.11	100.1262881	0.420447921	0.154300856	2.724857998	0.006432915	0.044383649	C16orf74	protein_coding
ENSG00000154114.12	624.3733687	0.094005447	0.085599354	1.098202754	0.272115964	0.500337442	TBCEL	protein_coding
ENSG00000154118.13	93.77557654	1.282872552	0.191672737	6.69303613	2.18587E-11	7.50029E-09	JPH3	protein_coding
ENSG00000154122.14	5405.627016	0.090627207	0.091923279	0.985900508	0.324181938	0.553973424	ANKH	protein_coding
ENSG00000154124.5	1678.215577	0.055118058	0.055709785	0.989378406	0.322478035	0.552351546	OTULIN	protein_coding
ENSG00000154127.10	910.1351023	0.373873415	0.101373782	3.688068123	0.000225963	0.00373409	UBASH3B	protein_coding
ENSG00000154133.15	1147.743256	0.004513779	0.093292383	0.048383142	0.961410894	0.982122991	ROBO4	protein_coding
ENSG00000154134.15	266.3508632	0.31047824	0.119445867	2.599321747	0.009340818	0.057595158	ROBO3	protein_coding
ENSG00000154143.3	4.953912003	0.028003261	0.340574954	0.082223488	0.934468996	0.969071546	PANX3	protein_coding
ENSG00000154144.13	1566.109919	-0.00045716	0.041639533	-0.010978983	0.991240215	0.995994255	TBRG1	protein_coding
ENSG00000154146.13	360.8664246	-0.0847051	0.094085834	-0.900296004	0.367962747	0.595002138	NRGN	protein_coding
ENSG00000154153.13	2276.778599	-0.176194396	0.139330734	-1.264576676	0.206023119	0.425803553	RETREG1	protein_coding
ENSG00000154162.15	24.1527776	-0.351818738	0.289747909	-1.214223561	0.224662366	0.447609359	CDH12	protein_coding
ENSG00000154165.5	111.9945649	-0.762649367	0.208702332	-3.654244584	0.00025794	0.00414006	GPR15	protein_coding
ENSG00000154174.8	5553.516614	-0.042990507	0.046390661	-0.926706071	0.354079164	0.582374871	TOMM70	protein_coding
ENSG00000154175.18	2763.584571	-0.370450347	0.17746302	-2.087479112	0.036844847	0.145631556	ABI3BP	protein_coding
ENSG00000154188.10	338.7551285	-0.090042962	0.114182577	-0.788587575	0.430353095	0.648438741	ANGPT1	protein_coding
ENSG00000154198.14	3.332164406	-0.145770798	0.270730724	-0.538434632	0.590277022	0.771312537	CYP4Z2P	transcribed_unprocessed_pseudogene
ENSG00000154217.15	1568.859338	0.160537962	0.081194878	1.977193218	0.048019794	0.173408903	PITPNC1	protein_coding
ENSG00000154222.15	2014.298686	0.033324686	0.039545678	0.842688445	0.399402711	0.622233851	CC2D1B	protein_coding
ENSG00000154227.13	25.70252946	0.951194796	0.252047519	3.773870897	0.000160734	0.00286944	CERS3	protein_coding
ENSG00000154229.12	5304.604921	0.077545521	0.085475157	0.907228763	0.364285836	0.591492805	PRKCA	protein_coding
ENSG00000154237.13	3226.591871	0.044508499	0.072073782	0.617540773	0.536878097	0.733083854	LRRK1	protein_coding
ENSG00000154240.17	299.9510671	0.153752395	0.094390614	1.628894966	0.103335256	0.280878271	CEP112	protein_coding
ENSG00000154252.12	165.3737175	0.33854828	0.208762344	1.621692272	0.104869254	0.283439031	GAL3ST2	protein_coding

ENSG00000154258.17	218.5189151	0.268927978	0.161470762	1.665490234	0.09581499	0.268027336	ABCA9	protein_coding
ENSG00000154262.13	323.4629119	0.131686752	0.141663287	0.929575723	0.352590802	0.58101151	ABCA6	protein_coding
ENSG00000154263.17	184.6914969	-0.012143653	0.138809285	-0.087484444	0.93028645	0.966943175	ABCA10	protein_coding
ENSG00000154265.16	1045.141498	0.013460512	0.088211892	0.152592937	0.878719295	0.941471389	ABCA5	protein_coding
ENSG00000154269.15	56.93492546	-0.75630891	0.162081159	-4.666235818	3.06768E-06	0.000126232	ENPP3	protein_coding
ENSG00000154274.15	1666.823927	-0.108512125	0.113878653	-0.95287503	0.340653393	0.570618985	C4orf19	protein_coding
ENSG00000154277.13	678.7034009	0.736171048	0.180831218	4.0710396	4.68038E-05	0.001088932	UCHL1	protein_coding
ENSG00000154305.18	5540.157329	0.155925214	0.060302391	2.58572194	0.009717529	0.059243684	MIA3	protein_coding
ENSG00000154309.9	992.3479722	-0.002794326	0.060947731	-0.045847907	0.963431475	0.98321265	DISP1	protein_coding
ENSG00000154310.17	2736.483739	0.006783487	0.10291487	0.065913578	0.94744663	0.975334431	TNIK	protein_coding
ENSG00000154316.16	8.327527302	0.143503742	0.175234777	0.81892273	0.412830501	0.633425398	TDH	transcribed_unitary_pseudogene
ENSG00000154319.16	506.5797822	1.2478475	0.155274485	8.036397606	9.25182E-16	1.18578E-12	FAM167A	protein_coding
ENSG00000154328.16	1145.731509	0.121150074	0.111338569	1.088123147	0.276540746	0.505360065	NEIL2	protein_coding
ENSG00000154330.13	2482.448045	-0.550017435	0.218180902	-2.520923831	0.011704719	0.067396475	PGM5	protein_coding
ENSG00000154342.6	9.847906685	0.977532889	0.193822472	5.043444558	4.57225E-07	2.75039E-05	WNT3A	protein_coding
ENSG00000154358.22	1386.424711	0.290350273	0.118814312	2.44373147	0.014536241	0.078357966	OBSCN	protein_coding
ENSG00000154359.13	1040.962123	0.109830693	0.080191312	1.369608384	0.170809181	0.380917116	LONRF1	protein_coding
ENSG00000154370.16	1798.150841	0.116121157	0.050577375	2.295911107	0.021680965	0.102594353	TRIM11	protein_coding
ENSG00000154380.17	8271.966309	-0.026663788	0.088358272	-0.301769	0.762828164	0.878853715	ENAH	protein_coding
ENSG00000154415.8	1.759660436	0.012873818	0.444395561	0.028969276	0.976889094	0.989514332	PPP1R3A	protein_coding
ENSG00000154429.11	993.0874265	-0.026721743	0.065802885	-0.406087717	0.684678151	0.83357695	CCSAP	protein_coding
ENSG00000154438.8	2.373620737	0.208463992	0.328341524	0.634899874	0.525493719	0.724388273	ASZ1	protein_coding
ENSG00000154447.15	4043.159842	0.094473143	0.080692245	1.170783427	0.241685841	0.467504612	SH3RF1	protein_coding
ENSG00000154451.14	2444.998351	-0.502331884	0.173877096	-2.889005492	0.003864623	0.030957479	GBP5	protein_coding
ENSG00000154473.18	7147.175014	-0.051682152	0.057061087	-0.905733743	0.365076798	0.592291572	BUB3	protein_coding
ENSG00000154478.4	8.525973655	-0.136448344	0.253698298	-0.537837049	0.590689552	0.77159708	GPR26	protein_coding
ENSG00000154479.13	30.62697583	-0.103254687	0.122827236	-0.840649766	0.40054417	0.62316677	CCDC173	protein_coding
ENSG00000154485.5	29.41665028	-0.109207955	0.101985051	-1.070823171	0.284248946	0.513270654	MMP21	protein_coding
ENSG00000154493.19	5.339097842	-0.085831304	0.243561253	-0.352401309	0.724537321	0.856314253	C10orf90	protein_coding
ENSG00000154511.12	1062.953699	-0.215606306	0.074298937	-2.901876073	0.003709352	0.030050651	DIPK1A	protein_coding
ENSG00000154518.10	10706.39728	-0.171066293	0.067862281	-2.520786077	0.011709301	0.067413955	ATP5MC3	protein_coding
ENSG00000154529.15	73.95702121	-0.013643375	0.176900591	-0.077124532	0.938524477	0.970886407	CNTNAP3B	protein_coding
ENSG00000154545.16	6.86842061	0.689559069	0.155679956	4.429337525	9.4523E-06	0.000310168	MAGED4	protein_coding
ENSG00000154548.9	83.38722847	-0.063496772	0.139734191	-0.454411136	0.649532939	0.81092989	SRSF12	protein_coding
ENSG00000154553.16	4521.623127	-0.152165664	0.160225965	-0.949694162	0.342267677	0.572091541	PDLIM3	protein_coding
ENSG00000154556.18	2378.823618	-0.093261117	0.13976156	-0.667287321	0.504588624	0.708683772	SORBS2	protein_coding
ENSG00000154582.17	3346.814134	-0.157979852	0.060395508	-2.615754992	0.008903042	0.055678508	ELOC	protein_coding
ENSG00000154589.7	461.3238168	-0.170444131	0.109889981	-1.551043414	0.12089128	0.308887677	LY96	protein_coding
ENSG00000154608.14	19.87174336	0.000308605	0.112229952	0.002749752	0.997806018	0.998890638	CEP170P1	transcribed_unprocessed_pseudogene
ENSG00000154611.15	5.878065288	-0.626755783	0.236569316	-2.649353652	0.008064589	0.052073194	PSMA8	protein_coding
ENSG00000154620.6	22.46926857	0.162360732	0.287826893	0.564091598	0.572691796	0.758640216	TMSB4Y	protein_coding
ENSG00000154639.19	5225.967853	0.169316435	0.098801951	1.713695254	0.086584709	0.252115033	CXADR	protein_coding

ENSG00000154640.15	1674.005004	0.206125555	0.069160629	2.980388651	0.002878829	0.024871277	BTG3	protein_coding
ENSG00000154642.11	784.7983478	-0.080679715	0.063430349	-1.27194185	0.203393781	0.422665274	C21orf91	protein_coding
ENSG00000154645.14	35.29442305	-0.863130348	0.182330241	-4.733884761	2.20263E-06	9.69535E-05	CHODL	protein_coding
ENSG00000154646.9	100.9784174	-1.291662579	0.315877739	-4.0891219	4.33009E-05	0.001029419	TMPRSS15	protein_coding
ENSG00000154654.15	140.6253511	0.156291761	0.175937299	0.888337843	0.374359049	0.600089913	NCAM2	protein_coding
ENSG00000154655.16	185.7359858	-0.189053778	0.14842598	-1.273724301	0.202761141	0.421994567	L3MBTL4	protein_coding
ENSG00000154678.18	330.6395528	-0.520932598	0.141868413	-3.67194209	0.000240714	0.003919882	PDE1C	protein_coding
ENSG00000154710.18	216.2528099	-0.01375744	0.069922145	-0.196753685	0.844020297	0.92349091	RABGEF1	protein_coding
ENSG00000154719.14	1048.72748	-0.167627408	0.070779501	-2.368304479	0.017869823	0.089972648	MRPL39	protein_coding
ENSG00000154721.15	954.9241714	-0.202582124	0.136729046	-1.481631954	0.138438252	0.335114082	JAM2	protein_coding
ENSG00000154723.12	4342.271356	-0.265589915	0.068267668	-3.890420232	0.000100071	0.001984881	ATP5PF	protein_coding
ENSG00000154727.11	1972.170399	-0.011522469	0.044340656	-0.2598624	0.794969915	0.89728564	GABPA	protein_coding
ENSG00000154734.16	3361.836514	0.043294494	0.124699675	0.347190109	0.728448501	0.858498575	ADAMTS1	protein_coding
ENSG00000154736.6	854.3871064	0.232449455	0.09128799	2.546331182	0.010886185	0.064080413	ADAMTS5	protein_coding
ENSG00000154743.18	731.4608669	-0.081771286	0.058928804	-1.387628479	0.165250205	0.373191759	TSEN2	protein_coding
ENSG00000154760.14	1629.737874	0.290661161	0.131887701	2.203853421	0.027534654	0.120445455	SLFN13	protein_coding
ENSG00000154764.6	15.0349431	1.467031095	0.246656867	5.94765966	2.72003E-09	4.17362E-07	WNT7A	protein_coding
ENSG00000154767.15	1171.756553	0.011545512	0.052294405	0.2207791	0.825264438	0.913781996	XPC	protein_coding
ENSG00000154781.16	1082.409694	-0.02845226	0.038699449	-0.735210984	0.46221101	0.67472093	CCDC174	protein_coding
ENSG00000154783.12	1579.67517	0.225968766	0.098405317	2.29630647	0.021658364	0.102576519	FGD5	protein_coding
ENSG00000154803.13	1376.967214	0.056842852	0.049794813	1.141541632	0.253644597	0.480442955	FLCN	protein_coding
ENSG00000154813.10	1741.054487	0.014821781	0.047099168	0.314693065	0.75299471	0.873100132	DPH3	protein_coding
ENSG00000154814.14	837.01553	-0.096498354	0.050491687	-1.911173114	0.055982338	0.191231663	OXNAD1	protein_coding
ENSG00000154822.18	730.3011533	-0.019856873	0.109165962	-0.181896194	0.855664191	0.929509622	PLCL2	protein_coding
ENSG00000154832.15	2101.66472	-0.071297777	0.051399767	-1.387122576	0.165404389	0.3734045	CXXC1	protein_coding
ENSG00000154839.10	667.2378382	-0.25052983	0.096727777	-2.590050527	0.009596185	0.058699177	SKA1	protein_coding
ENSG00000154845.16	5466.814491	0.193560419	0.061569718	3.143760031	0.001667922	0.016793673	PPP4R1	protein_coding
ENSG00000154856.13	2191.393562	-0.413810411	0.159372096	-2.596504784	0.009417759	0.05788402	APCDD1	protein_coding
ENSG00000154864.13	581.4967535	0.326136692	0.115491065	2.823912753	0.00474413	0.035885253	PIEZO2	protein_coding
ENSG00000154874.15	106.5406732	-0.136028852	0.131595982	-1.033685452	0.301283236	0.530894443	CCDC144B	transcribed_unprocessed_pseudogene
ENSG00000154889.17	738.5722991	-0.042816062	0.057268106	-0.747642362	0.454675906	0.668821251	MPPE1	protein_coding
ENSG00000154898.15	21.95445807	0.32275073	0.126800661	2.545339491	0.010917154	0.064219334	CCDC144CP	transcribed_processed_pseudogene
ENSG00000154914.17	842.8624751	0.162982898	0.090271792	1.805468725	0.071001365	0.221794014	USP43	protein_coding
ENSG00000154917.11	471.3091208	0.167043229	0.125393907	1.332147895	0.182811594	0.396638853	RAB6B	protein_coding
ENSG00000154920.15	210.4684744	-0.151577086	0.100020979	-1.515452939	0.12965774	0.322218154	EME1	protein_coding
ENSG00000154928.18	411.1801064	-0.289360293	0.164973996	-1.753975176	0.079434761	0.239121896	EPHB1	protein_coding
ENSG00000154930.15	4568.116108	-0.222497027	0.096369828	-2.308783074	0.020955622	0.100316723	ACSS1	protein_coding
ENSG00000154945.7	2785.297713	-0.05863325	0.046752054	-1.25413207	0.209794005	0.430038731	ANKRD40	protein_coding
ENSG00000154957.14	394.0144335	-0.154641542	0.046438996	-3.329993244	0.000868481	0.010298175	ZNF18	protein_coding
ENSG00000154975.14	7.75562092	-0.251198549	0.207330526	-1.211584971	0.22567129	0.44876778	CA10	protein_coding
ENSG00000154978.13	3718.516648	0.001830685	0.072876475	0.025120381	0.979958944	0.991148319	VOPP1	protein_coding
ENSG00000154997.9	9.781437785	0.064656424	0.275108701	0.235021371	0.814192146	0.908293652	SEPTIN14	protein_coding

ENSG00000155008.14	2226.039204	-0.046466928	0.062320812	-0.745608515	0.455903937	0.669912526	APOOL	protein_coding
ENSG00000155011.9	257.6885924	1.046027566	0.15546496	6.728381511	1.71561E-11	6.17859E-09	DKK2	protein_coding
ENSG00000155016.18	571.7235107	0.009232637	0.096679912	0.095496955	0.923920108	0.964101079	CYP2U1	protein_coding
ENSG00000155026.16	6.576211185	-0.251689387	0.155698159	-1.616521281	0.105981656	0.285119313	RSPH10B	protein_coding
ENSG00000155034.19	1290.594135	0.249481759	0.073721006	3.384133935	0.000714032	0.008885025	FBXL18	protein_coding
ENSG00000155052.14	15.74882934	0.477963574	0.27261331	1.753265726	0.079556406	0.239355806	CNTNAP5	protein_coding
ENSG00000155066.16	4874.047885	0.286567939	0.183822285	1.558940144	0.119010548	0.305803564	PROM2	protein_coding
ENSG00000155070.8	9.035136307	0.274126979	0.162188288	1.690177404	0.09099402	0.259915702	UNC93B2	processed_pseudogene
ENSG00000155085.15	429.6479913	0.012417885	0.07350176	0.16894677	0.865838511	0.934945108	AK9	protein_coding
ENSG00000155087.4	1.437868998	0.194195502	0.384371629	0.505228501	0.613398354	0.787737052	ODF1	protein_coding
ENSG00000155090.15	5974.722239	0.055646572	0.068043107	0.817813503	0.413463687	0.634062045	KLF10	protein_coding
ENSG00000155093.19	3095.068212	-0.217311355	0.155510855	-1.397403121	0.162292381	0.36917285	PTPRN2	protein_coding
ENSG00000155096.14	10732.78821	0.007669372	0.05926828	0.129400948	0.897040398	0.951093183	AZIN1	protein_coding
ENSG00000155097.12	4356.245044	0.095333969	0.060915156	1.565028721	0.117576175	0.303461974	ATP6V1C1	protein_coding
ENSG00000155099.8	570.0768118	-0.022703683	0.102391263	-0.221734569	0.824520518	0.91339275	PIP4P2	protein_coding
ENSG00000155100.11	786.2108472	-0.014092323	0.071161433	-0.198033148	0.843019129	0.922882093	OTUD6B	protein_coding
ENSG00000155111.15	2340.961451	0.014375678	0.060180076	0.238877696	0.811200421	0.906634541	CDK19	protein_coding
ENSG00000155115.7	2427.315341	-0.185052689	0.066789826	-2.77067181	0.005594078	0.04028737	GTF3C6	protein_coding
ENSG00000155158.20	2119.454607	0.063893156	0.076180354	0.838709097	0.401632572	0.623695175	TTC39B	protein_coding
ENSG00000155189.12	2961.586166	-0.109979517	0.078607104	-1.399104047	0.161781784	0.36847148	AGPAT5	protein_coding
ENSG00000155229.21	3623.050331	0.020249074	0.040149005	0.504348095	0.614016786	0.788269193	MMS19	protein_coding
ENSG00000155249.5	0.514327278	-0.382385729	0.561849964	-0.680583347	0.496135167		OR4K1	protein_coding
ENSG00000155252.13	1626.544603	0.043511278	0.048005946	0.906372694	0.364738621	0.592007407	PI4K2A	protein_coding
ENSG00000155254.13	3776.449128	0.426462901	0.09314158	4.5786522	4.67982E-06	0.000176871	MARVELD1	protein_coding
ENSG00000155256.17	1802.374372	-0.000272807	0.056340443	-0.004842118	0.996136564	0.998251231	ZFYVE27	protein_coding
ENSG00000155265.11	322.3359455	-0.305135154	0.164997374	-1.84933339	0.064409687	0.20882233	GOLGA7B	protein_coding
ENSG00000155269.12	26.23046311	0.859776647	0.190383555	4.516023699	6.30116E-06	0.000226718	GPR78	protein_coding
ENSG00000155275.19	618.8606016	-0.008941218	0.055091912	-0.162296377	0.871072468	0.937720434	TRMT44	protein_coding
ENSG00000155287.11	1550.671942	-0.124428155	0.05315152	-2.341008419	0.019231733	0.094440078	SLC25A28	protein_coding
ENSG00000155304.6	2012.721849	0.117301196	0.062377214	1.880513569	0.060038119	0.199931933	HSPA13	protein_coding
ENSG00000155307.19	897.9374812	-0.340735633	0.115122468	-2.959766567	0.003078722	0.026191468	SAMSN1	protein_coding
ENSG00000155313.15	2366.184414	-0.054408262	0.050095084	-1.086099819	0.277434833	0.506314503	USP25	protein_coding
ENSG00000155324.10	903.9071759	0.058923411	0.063142106	0.933187289	0.350723276	0.579800803	GRAMD2B	protein_coding
ENSG00000155329.12	1003.295422	-0.015144118	0.047805242	-0.316787805	0.751404618	0.872360729	ZCCHC10	protein_coding
ENSG00000155330.10	596.4304509	-0.02848007	0.054283967	-0.524649752	0.599826694	0.77839928	C16orf87	protein_coding
ENSG00000155363.18	4160.195531	-0.045593815	0.059588641	-0.765142725	0.444186561	0.660174549	MOV10	protein_coding
ENSG00000155366.17	6263.923816	0.102028335	0.074705959	1.365732205	0.172023032	0.382632985	RHOC	protein_coding
ENSG00000155367.15	68.11848081	0.179730216	0.099787372	1.801131869	0.071682098	0.223179854	PPM1J	protein_coding
ENSG00000155368.16	6380.945573	-0.178786379	0.071164322	-2.512303566	0.011994586	0.068477411	DBI	protein_coding
ENSG00000155380.12	5674.409456	-0.122792558	0.089537219	-1.371413574	0.170246068	0.380198479	SLC16A1	protein_coding
ENSG00000155393.13	1253.310286	-0.127137191	0.046070052	-2.759649409	0.005786342	0.04128216	HEATR3	protein_coding
ENSG00000155428.12	8.684822303	-0.297636485	0.131224134	-2.268153552	0.023319849	0.107777831	TRIM74	protein_coding

ENSG00000155438.12	1922.420727	-0.071572237	0.056636649	-1.263708904	0.206334528	0.426139237	NIFK	protein_coding
ENSG00000155463.14	8341.280447	-0.169852529	0.048572231	-3.496906053	0.000470688	0.00650941	OXA1L	protein_coding
ENSG00000155465.20	2409.071657	-0.054723835	0.090477762	-0.604831882	0.545290708	0.739608228	SLC7A7	protein_coding
ENSG00000155495.9	30.44619521	0.944947505	0.467911833	2.019499055	0.043435375	0.162498571	MAGEC1	protein_coding
ENSG00000155506.18	17473.73573	-0.024362449	0.048444257	-0.502896535	0.615037017	0.788719228	LARP1	protein_coding
ENSG00000155508.14	2612.147172	0.02676868	0.037489695	0.714027664	0.475210074	0.685446393	CNOT8	protein_coding
ENSG00000155511.18	28.3988196	-0.265669255	0.199606835	-1.330962717	0.183201279	0.396969725	GRIA1	protein_coding
ENSG00000155530.3	58.84020472	0.040057618	0.123390627	0.324640684	0.745453032	0.868990605	LRGUK	protein_coding
ENSG00000155542.12	337.2582285	-0.139546109	0.086135588	-1.620074961	0.105216175	0.283960962	SETD9	protein_coding
ENSG00000155545.20	1656.110641	-0.007320083	0.056943129	-0.128550772	0.897713121	0.951450551	MIER3	protein_coding
ENSG00000155561.15	5153.915439	-0.10089496	0.059521919	-1.695089176	0.090058505	0.258308398	NUP205	protein_coding
ENSG00000155592.16	410.1579242	0.086818422	0.073204613	1.185969287	0.235634429	0.460553377	ZKSCAN2	protein_coding
ENSG00000155621.15	545.5947251	-0.052265864	0.05716229	-0.914341662	0.360537345	0.588343915	C9orf85	protein_coding
ENSG00000155622.7	5.594827047	-0.275396084	0.422453942	-0.651896116	0.51446819	0.716346106	XAGE2	protein_coding
ENSG00000155629.15	3370.125997	-0.244084111	0.099816999	-2.445316054	0.014472525	0.078159529	PIK3AP1	protein_coding
ENSG00000155636.15	507.9855981	-0.113281367	0.03237468	-3.499072974	0.000466879	0.006473171	RBM45	protein_coding
ENSG00000155657.28	567.4930318	-0.21016159	0.105992562	-1.982795639	0.047390261	0.171936177	TTN	protein_coding
ENSG00000155659.15	1122.910109	0.275764003	0.143522312	1.921401625	0.054681094	0.188411325	VSIG4	protein_coding
ENSG00000155660.11	18446.71152	-0.080694826	0.077708129	-1.038434807	0.299067672	0.528956078	PDIA4	protein_coding
ENSG00000155666.12	215.3444282	-0.065966098	0.049604158	-1.329850157	0.183567646	0.397501855	KDM8	protein_coding
ENSG00000155714.14	8.563471633	-0.309196812	0.127500375	-2.4250659	0.015305608	0.081179709	PDZD9	protein_coding
ENSG00000155719.18	29.696165	-0.260498957	0.129006792	-2.019265441	0.043459638	0.162517428	OTOA	protein_coding
ENSG00000155729.13	931.0714059	-0.079894025	0.040275619	-1.983682123	0.047291288	0.171677145	KCTD18	protein_coding
ENSG00000155744.9	2199.142779	-0.110078945	0.061867498	-1.779269386	0.075195607	0.23071175	FAM126B	protein_coding
ENSG00000155749.13	25.24341686	-0.057686807	0.111798534	-0.515988939	0.605862135	0.78269353	FLACC1	protein_coding
ENSG00000155754.15	34.05502261	0.116447799	0.160530752	0.72539247	0.468211292	0.679768231	C2CD6	protein_coding
ENSG00000155755.19	890.2437207	0.114078556	0.062979457	1.811361368	0.070084932	0.219894104	TMEM237	protein_coding
ENSG00000155760.2	1970.17338	-0.225874368	0.116275189	-1.942584394	0.052066394	0.182546995	FZD7	protein_coding
ENSG00000155761.14	86.40000852	1.091384447	0.206349696	5.289004391	1.22984E-07	9.51913E-06	SPAG17	protein_coding
ENSG00000155792.10	2293.030364	-0.331518898	0.096303968	-3.442422018	0.00057653	0.007581005	DEPTOR	protein_coding
ENSG00000155816.20	86.91073652	-0.104364708	0.208775421	-0.499889823	0.617152659	0.79005158	FMN2	protein_coding
ENSG00000155827.12	3108.309834	-0.069141893	0.038254942	-1.807397668	0.070700297	0.221170952	RNF20	protein_coding
ENSG00000155833.15	2.722905644	-0.609773849	0.361866725	-1.685078532	0.091973421	0.261608421	CYLC2	protein_coding
ENSG00000155846.17	1432.369982	-0.33831534	0.093813928	-3.60623788	0.000310668	0.004771941	PPARGC1B	protein_coding
ENSG00000155849.15	1632.53751	-0.155519909	0.104681733	-1.485645147	0.137373007	0.333759116	ELMO1	protein_coding
ENSG00000155850.8	1630.889532	0.110326975	0.089529856	1.232292553	0.217839821	0.439319042	SLC26A2	protein_coding
ENSG00000155858.6	571.1858284	0.011363003	0.058282753	0.194963382	0.845421614	0.924223933	LSM11	protein_coding
ENSG00000155868.8	574.4785924	-0.016094199	0.042845054	-0.375637272	0.707186578	0.846685251	MED7	protein_coding
ENSG00000155875.15	16.40612683	-0.215732052	0.143298138	-1.50547701	0.132201483	0.325910224	SAXO1	protein_coding
ENSG00000155876.6	3505.141529	0.042096852	0.052787622	0.797475811	0.425174746	0.644065766	RRAGA	protein_coding
ENSG00000155886.11	62.26357878	0.638791417	0.171365211	3.727661018	0.000193265	0.003306601	SLC24A2	protein_coding
ENSG00000155890.4	1.612610774	-0.663935224	0.497990692	-1.33322818	0.182456934	0.396041334	TRIM42	protein_coding

ENSG00000155893.13	1159.656309	0.16482795	0.075539173	2.182019536	0.029108091	0.125017847	PXYLP1	protein_coding
ENSG00000155897.10	3.849391123	-0.047134428	0.301195768	-0.156491003	0.87564601	0.93989045	ADCY8	protein_coding
ENSG00000155903.13	1507.51311	0.012911361	0.053918641	0.239460062	0.810748862	0.906315816	RASA2	protein_coding
ENSG00000155906.19	977.0467364	-0.03833689	0.051915319	-0.738450431	0.460240779	0.673356133	RMND1	protein_coding
ENSG00000155918.8	85.06772597	1.47293635	0.211486827	6.964671842	3.2917E-12	1.61171E-09	RAET1L	protein_coding
ENSG00000155926.14	1323.452074	-0.440953591	0.12260614	-3.596504953	0.000322521	0.004912088	SLA	protein_coding
ENSG00000155957.18	963.1754415	-0.175685914	0.087147307	-2.015964936	0.043803642	0.16331548	TMBIM4	protein_coding
ENSG00000155959.12	2157.725845	0.025697863	0.05618545	0.457375768	0.647400979	0.809706459	VBP1	protein_coding
ENSG00000155961.5	89.87718024	-0.048097666	0.154620097	-0.31106995	0.755747445	0.874676745	RAB39B	protein_coding
ENSG00000155962.13	678.254309	-0.13802861	0.094762422	-1.456575376	0.145233629	0.345404456	CLIC2	protein_coding
ENSG00000155966.14	123.1937286	-0.031912815	0.176863853	-0.18043718	0.856809368	0.930161346	AFF2	protein_coding
ENSG00000155970.12	158.3952343	-0.139507747	0.13154594	-1.06052492	0.288905859	0.518638572	MICU3	protein_coding
ENSG00000155974.12	226.0901056	0.429275045	0.119429216	3.594388878	0.000325154	0.004937014	GRIP1	protein_coding
ENSG00000155975.10	2166.428075	0.034553452	0.060175051	0.574215574	0.565821932	0.754169643	VPS37A	protein_coding
ENSG00000155980.13	134.53186	0.171735595	0.170763023	1.005695444	0.314562095	0.543847349	KIF5A	protein_coding
ENSG00000156006.5	142.7868063	-0.10952227	0.244205143	-0.448484698	0.653803427	0.813908893	NAT2	protein_coding
ENSG00000156009.10	10.57296822	0.795972894	0.25105187	3.170551542	0.001521498	0.01569286	MAGEA8	protein_coding
ENSG00000156011.17	2285.802609	0.366996321	0.095544816	3.841090897	0.000122489	0.002309689	PSD3	protein_coding
ENSG00000156017.13	1577.689841	0.007171404	0.065712703	0.109132686	0.91309725	0.95894649	CARNMT1	protein_coding
ENSG00000156026.14	7493.171892	-0.031484655	0.093572678	-0.336472735	0.736514409	0.863361487	MCU	protein_coding
ENSG00000156030.13	4280.461228	0.08182101	0.05564659	1.470368806	0.141461895	0.339528806	MIDEAS	protein_coding
ENSG00000156042.18	92.84908299	0.069737955	0.123955807	0.562603369	0.573704997	0.759175479	CFAP70	protein_coding
ENSG00000156049.7	403.4959064	0.17090942	0.126698124	1.348949886	0.177353063	0.389659899	GNA14	protein_coding
ENSG00000156050.10	236.7607869	0.095934692	0.077785807	1.233318716	0.217456878	0.438868336	FAM161B	protein_coding
ENSG00000156052.11	7115.306098	0.047837949	0.051502974	0.928838577	0.352972749	0.581333137	GNAQ	protein_coding
ENSG00000156076.10	41.50278413	1.66082222	0.307754159	5.396587402	6.79203E-08	5.97932E-06	WIF1	protein_coding
ENSG00000156096.14	8.13344456	0.774444182	0.308603196	2.509514459	0.012089727	0.068876195	UGT2B4	protein_coding
ENSG00000156097.12	11.98520644	-0.122376712	0.113115543	-1.081873529	0.279308728	0.507943898	GPR61	protein_coding
ENSG00000156103.16	285.6614469	0.331757997	0.129954877	2.552870686	0.010683915	0.063234671	MMP16	protein_coding
ENSG00000156110.15	1947.695082	-0.060935084	0.068419678	-0.890607583	0.37313973	0.59941207	ADK	protein_coding
ENSG00000156113.24	3340.021341	-0.427240684	0.207775371	-2.056262403	0.03975723	0.153495244	KCNMA1	protein_coding
ENSG00000156127.8	592.5686714	-0.287336714	0.112168946	-2.561642268	0.010417856	0.062044407	BATF	protein_coding
ENSG00000156136.10	2110.121104	-0.129611442	0.057274939	-2.26296953	0.023637568	0.108771783	DCK	protein_coding
ENSG00000156140.10	91.39467223	0.304023821	0.110069662	2.762103699	0.005743023	0.041075291	ADAMTS3	protein_coding
ENSG00000156150.9	4.426097587	0.524131729	0.414174667	1.265484758	0.205697609	0.42538367	ALX3	protein_coding
ENSG00000156162.16	2124.504191	0.017706971	0.059353075	0.298332832	0.765449148	0.880709129	DPY19L4	protein_coding
ENSG00000156170.13	846.3841292	-0.288095866	0.059526188	-4.839817171	1.29959E-06	6.41591E-05	NDUFAF6	protein_coding
ENSG00000156171.15	2566.061645	-0.107644162	0.045935676	-2.343367328	0.019110558	0.094041773	DRAM2	protein_coding
ENSG00000156172.6	275.4158229	-0.006249652	0.0715127	-0.087392192	0.930359776	0.966980768	C8orf37	protein_coding
ENSG00000156194.18	8.041373416	-0.384866964	0.227844808	-1.689162752	0.091188245	0.260259359	PPEF2	protein_coding
ENSG00000156206.14	15.95387422	0.01989978	0.129159202	0.154071719	0.877553187	0.940966995	CFAP161	protein_coding
ENSG00000156218.13	497.871512	-0.219412155	0.160464641	-1.367355165	0.171514008	0.38201206	ADAMTSL3	protein_coding

ENSG00000156219.17	40.41588585	-0.622180994	0.177897216	-3.497418379	0.000469784	0.006503112	ART3	protein_coding
ENSG00000156222.12	22.29755087	-0.099624339	0.211299203	-0.471484689	0.637294651	0.803596556	SLC28A1	protein_coding
ENSG00000156232.7	1119.883832	0.082890838	0.046838808	1.769704267	0.076776419	0.233878047	WHAMM	protein_coding
ENSG00000156234.7	1980.548639	-0.899331755	0.192797419	-4.664646239	3.09148E-06	0.000126972	CXCL13	protein_coding
ENSG00000156239.12	468.4954369	-0.02167798	0.053482788	-0.405326285	0.685237688	0.833791757	N6AMT1	protein_coding
ENSG00000156253.7	909.3798028	-0.121460388	0.061643482	-1.970368693	0.048796131	0.175159129	RWDD2B	protein_coding
ENSG00000156256.15	2505.358296	-0.091189532	0.044307915	-2.058086733	0.039581808	0.15308924	USP16	protein_coding
ENSG00000156261.13	7722.60108	-0.061329592	0.057934953	-1.058593963	0.289784738	0.519604555	CCT8	protein_coding
ENSG00000156265.16	276.7359481	-0.034056753	0.070753455	-0.481344036	0.630272004	0.799174254	MAP3K7CL	protein_coding
ENSG00000156269.5	9.201647365	2.478549069	0.389350208	6.365860397	1.94198E-10	4.80828E-08	NAA11	protein_coding
ENSG00000156273.16	2992.488057	0.153702538	0.061069739	2.516836325	0.011841382	0.067941003	BACH1	protein_coding
ENSG00000156282.5	1.837233934	0.701960444	0.656921347	1.068560867	0.28526759	0.514577747	CLDN17	protein_coding
ENSG00000156284.6	6.829386102	-0.347411044	0.426584593	-0.814401293	0.415415098	0.63580162	CLDN8	protein_coding
ENSG00000156298.13	604.0771923	-0.389644406	0.148818898	-2.618245465	0.00883832	0.055353188	TSPAN7	protein_coding
ENSG00000156299.13	613.8002526	0.235400283	0.126830558	1.856021812	0.063450449	0.206909145	TIAM1	protein_coding
ENSG00000156304.15	3339.073791	-0.01516828	0.04280244	-0.354378858	0.723054977	0.855433359	SCAF4	protein_coding
ENSG00000156313.15	839.1429751	-0.23792898	0.060758161	-3.916000389	9.00301E-05	0.00182721	RPGR	protein_coding
ENSG00000156345.18	320.9251369	-0.002076239	0.100115789	-0.020738379	0.983454354	0.992657225	CDK20	protein_coding
ENSG00000156374.16	501.9367552	-0.172555748	0.052760796	-3.270529644	0.001073463	0.012128155	PCGF6	protein_coding
ENSG00000156381.9	2468.555966	0.007497821	0.070246002	0.106736616	0.914997933	0.95976336	ANKRD9	protein_coding
ENSG00000156384.14	595.38232	-0.163143447	0.069836147	-2.336088865	0.019486611	0.095283665	SFR1	protein_coding
ENSG00000156395.13	15.32475547	0.0954117	0.240065438	0.397440383	0.691042741	0.837108268	SORCS3	protein_coding
ENSG00000156398.13	1091.265178	-0.196307192	0.078293172	-2.507334779	0.012164544	0.069161983	SFXN2	protein_coding
ENSG00000156411.10	4279.215138	-0.197750221	0.0705238	-2.804021082	0.00504696	0.03744788	ATP5MPL	protein_coding
ENSG00000156413.14	1224.076098	0.408613311	0.191398285	2.134884915	0.032770412	0.135061823	FUT6	protein_coding
ENSG00000156414.19	108.0048673	1.318036103	0.204040203	6.459688253	1.04919E-10	2.85753E-08	TDRD9	protein_coding
ENSG00000156427.8	199.2472463	0.656340161	0.14694622	4.466533135	7.94974E-06	0.000270645	FGF18	protein_coding
ENSG00000156453.14	10996.45852	0.252425618	0.091718282	2.752184342	0.005919919	0.04191945	PCDH1	protein_coding
ENSG00000156463.18	2738.001787	-0.150444825	0.112212725	-1.34071091	0.180014331	0.392951185	SH3RF2	protein_coding
ENSG00000156466.10	48.61796433	0.431214668	0.20229243	2.131640161	0.03303644	0.135686044	GDF6	protein_coding
ENSG00000156467.10	7339.470422	-0.247961786	0.068951491	-3.596177275	0.000322928	0.004912088	UQCRB	protein_coding
ENSG00000156469.9	1093.004313	-0.206632591	0.061914006	-3.337412737	0.000845623	0.010093043	MTERF3	protein_coding
ENSG00000156471.13	5630.367065	-0.098873213	0.058664977	-1.68538742	0.091913849	0.261484178	PTDSS1	protein_coding
ENSG00000156475.18	116.5926625	-0.366430922	0.139528605	-2.626206443	0.008634243	0.054513825	PPP2R2B	protein_coding
ENSG00000156482.11	12323.56304	-0.201975012	0.061071215	-3.307204754	0.00094232	0.010967167	RPL30	protein_coding
ENSG00000156486.8	28.52395149	-0.027809332	0.167517948	-0.166008076	0.868150594	0.936096364	KCNS2	protein_coding
ENSG00000156500.15	398.9512314	-0.04977312	0.084596606	-0.58835836	0.556291781	0.747042433	FAM122C	protein_coding
ENSG00000156502.14	1755.678869	-0.131970635	0.046289586	-2.850978958	0.004358485	0.033723316	SUPV3L1	protein_coding
ENSG00000156504.16	2523.944429	-0.07580944	0.070836346	-1.070205401	0.284526864	0.513685509	FAM122B	protein_coding
ENSG00000156508.19	228667.1893	0.017245803	0.055929659	0.308348087	0.75781748	0.875911587	EEF1A1	protein_coding
ENSG00000156509.14	38.72958471	0.036224039	0.125992692	0.287509045	0.773722577	0.885581931	FBXO43	protein_coding
ENSG00000156510.13	2349.509505	-0.111296681	0.143263387	-0.776867585	0.437236897	0.65425157	HKDC1	protein_coding

ENSG00000156515.24	6074.500265	0.175581726	0.067782195	2.590381248	0.009586969	0.058659275	HK1	protein_coding
ENSG00000156521.14	1337.437381	-0.136444577	0.070745354	-1.928671902	0.053771607	0.186368035	TYSND1	protein_coding
ENSG00000156531.17	2111.866857	0.0707455	0.068257891	1.036444268	0.299994927	0.529649024	PHF6	protein_coding
ENSG00000156535.15	1632.418578	0.716494313	0.140333314	5.105660882	3.2964E-07	2.10936E-05	CD109	protein_coding
ENSG00000156564.9	10.08235591	1.103116474	0.240041095	4.59553176	4.31647E-06	0.000166459	LRFN2	protein_coding
ENSG00000156574.10	21.73121728	0.1647536	0.162976308	1.010905217	0.312061797	0.54154422	NODAL	protein_coding
ENSG00000156575.2	1.483475079	-0.474664688	0.406006012	-1.169107535	0.242360309	0.468248068	PRG3	protein_coding
ENSG00000156587.16	6007.278711	-0.08100003	0.100195084	-0.808423201	0.418847001	0.638877656	UBE2L6	protein_coding
ENSG00000156599.11	9367.508467	0.085731125	0.042470285	2.018614291	0.043527324	0.16259131	ZDHHC5	protein_coding
ENSG00000156603.19	1138.872759	-0.100980625	0.063327094	-1.59458802	0.110804393	0.292623381	MED19	protein_coding
ENSG00000156639.12	5009.787003	0.125676646	0.05210345	2.412059993	0.015862671	0.083201368	ZFAND3	protein_coding
ENSG00000156642.17	7364.243656	0.086014266	0.046139549	1.864219896	0.06229083	0.204632304	NPTN	protein_coding
ENSG00000156650.14	3302.118218	-0.015029359	0.069944802	-0.214874566	0.829865115	0.91633174	KAT6B	protein_coding
ENSG00000156671.15	1854.036594	0.10971496	0.070559558	1.554926982	0.11996346	0.307472516	SAMD8	protein_coding
ENSG00000156675.16	10782.12224	0.111433228	0.099983488	1.114516311	0.265057751	0.492869508	RAB11FIP1	protein_coding
ENSG00000156687.11	23.16212896	-0.615498638	0.214948766	-2.863466714	0.004190328	0.032753711	UNC5D	protein_coding
ENSG00000156689.7	14.89308513	0.198857536	0.184820596	1.075949003	0.281950065	0.510832278	GLYATL2	protein_coding
ENSG00000156697.13	1836.908315	-0.15463564	0.05885101	-2.627578372	0.008599503	0.054373265	UTP14A	protein_coding
ENSG00000156709.15	3308.537372	-0.200669833	0.062434649	-3.214078035	0.001308641	0.014059825	AIFM1	protein_coding
ENSG00000156711.17	6791.633886	0.037172687	0.069382505	0.535764559	0.592121279	0.772579843	MAPK13	protein_coding
ENSG00000156735.11	1700.145894	0.004004449	0.06547243	0.061162377	0.951229892	0.977151529	BAG4	protein_coding
ENSG00000156738.18	1294.911875	-1.815611182	0.240726698	-7.542209463	4.62075E-14	3.4717E-11	MS4A1	protein_coding
ENSG00000156755.10	1.983761721	-0.795168894	0.238604755	-3.332577736	0.000860454	0.010225257	IGKV1OR-2	IG_V_pseudogene
ENSG00000156787.17	776.0491926	-0.095047089	0.068478772	-1.387978877	0.165143477	0.373086076	TBC1D31	protein_coding
ENSG00000156795.8	725.4546551	0.090239963	0.06050408	1.491469069	0.135838386	0.331546396	NTAQ1	protein_coding
ENSG00000156802.13	4294.230525	0.026072198	0.08683529	0.300248867	0.763987333	0.879678529	ATAD2	protein_coding
ENSG00000156804.7	5308.466552	0.02128618	0.138584977	0.15359659	0.877927826	0.941189256	FBXO32	protein_coding
ENSG00000156831.8	1049.094331	-0.046873515	0.06409287	-0.731337439	0.464573068	0.676537247	NSMCE2	protein_coding
ENSG00000156853.13	919.7863614	-0.068860647	0.056024582	-1.229114872	0.21902874	0.44068489	ZNF689	protein_coding
ENSG00000156858.12	2969.528268	-0.005884041	0.042555463	-0.138267589	0.89002894	0.947602329	PRR14	protein_coding
ENSG00000156860.16	7871.360422	0.113602661	0.053212595	2.134882914	0.032770575	0.135061823	FBRS	protein_coding
ENSG00000156869.14	917.4119121	-0.029921194	0.095442037	-0.31350121	0.753899901	0.873584431	FRRS1	protein_coding
ENSG00000156873.16	1528.521291	-0.087761896	0.05253015	-1.670695718	0.094781795	0.26623312	PHKG2	protein_coding
ENSG00000156875.14	78.11560188	-0.009950073	0.072093969	-0.138015331	0.890228301	0.947707986	MFSD14A	protein_coding
ENSG00000156876.10	657.2845306	-0.039373244	0.065364867	-0.602360954	0.546933895	0.740622214	SASS6	protein_coding
ENSG00000156885.6	2.021109292	-0.43113653	0.348287538	-1.23787527	0.215762313	0.43696087	COX6A2	protein_coding
ENSG00000156886.12	38.84363822	-0.172645112	0.1546114	-1.116638956	0.264148728	0.491919883	ITGAD	protein_coding
ENSG00000156920.11	10.19088563	-0.50296105	0.273370901	-1.839848525	0.065790479	0.211395938	ADGRG4	protein_coding
ENSG00000156925.12	1.729939365	-0.123597015	0.364374691	-0.339203074	0.734456754	0.862178021	ZIC3	protein_coding
ENSG00000156928.5	1194.953341	-0.192616905	0.059943026	-3.213333009	0.001312041	0.014085932	MALSU1	protein_coding
ENSG00000156931.16	2147.269883	-0.01124014	0.045608094	-0.246450558	0.805333473	0.903320895	VPS8	protein_coding
ENSG00000156958.15	1011.489331	0.008574913	0.057173338	0.149980982	0.880779619	0.942669552	GALK2	protein_coding

ENSG00000156959.9	33.81065488	0.010007419	0.209899961	0.047677087	0.961973595	0.982355347	LHFPL4	protein_coding
ENSG00000156966.7	4912.768695	0.291895437	0.15303652	1.907358045	0.056474238	0.192335398	B3GNT7	protein_coding
ENSG00000156968.9	739.4005045	-0.147593112	0.172122787	-0.857487347	0.391175604	0.615499524	MPV17L	protein_coding
ENSG00000156970.13	1469.594868	0.002467379	0.089798767	0.027476753	0.978079481	0.990259142	BUB1B	protein_coding
ENSG00000156973.14	1313.937442	-0.086081578	0.05343117	-1.611074182	0.107163555	0.28686444	PDE6D	protein_coding
ENSG00000156976.17	16662.75885	0.028688874	0.05751406	0.498814965	0.617909744	0.790658119	EIF4A2	protein_coding
ENSG00000156983.16	1858.673988	0.108648753	0.043754657	2.483135763	0.013023144	0.072683089	BRPF1	protein_coding
ENSG00000156990.14	1912.024977	-0.074592504	0.056485695	-1.320555657	0.186649567	0.40154735	RPUSD3	protein_coding
ENSG00000157005.4	221.1461554	-0.108131205	0.298940641	-0.361714634	0.71756529	0.852340945	SST	protein_coding
ENSG00000157014.11	1369.014786	0.025366177	0.041080522	0.617474541	0.536921769	0.73309437	TATDN2	protein_coding
ENSG00000157017.16	136.8006265	-0.464355613	0.183508499	-2.530431096	0.011392246	0.066147888	GHRL	protein_coding
ENSG00000157020.18	5741.91197	0.038054888	0.053576529	0.710290289	0.47752414	0.687244277	SEC13	protein_coding
ENSG00000157021.9	27.52263186	0.217385968	0.117262232	1.853844701	0.063761381	0.207600845	CIBAR1P1	unprocessed_pseudogene
ENSG00000157036.13	473.4195562	-0.058462064	0.05120497	-1.141726366	0.253567778	0.480376761	EXOG	protein_coding
ENSG00000157045.9	905.1838797	-0.031611989	0.067661442	-0.467208324	0.640350839	0.805396384	NTAN1	protein_coding
ENSG00000157060.16	5.945969482	-0.170260886	0.274636488	-0.619949983	0.535290717	0.732106069	SHCBP1L	protein_coding
ENSG00000157064.11	380.5287201	0.603379815	0.17334707	3.480761552	0.00049999	0.006789679	NMNAT2	protein_coding
ENSG00000157077.15	1417.616057	0.003773562	0.059247918	0.063691046	0.949216234	0.976210607	ZFYVE9	protein_coding
ENSG00000157087.20	20.52108218	-0.272607556	0.179031184	-1.522681967	0.127838292	0.319298976	ATP2B2	protein_coding
ENSG00000157093.9	2.967755821	-0.453970753	0.272319466	-1.667052159	0.095504034	0.267604121	LYZL4	protein_coding
ENSG00000157103.12	121.6054778	0.126981445	0.102334753	1.240843808	0.214663454	0.435736414	SLC6A1	protein_coding
ENSG00000157106.17	9374.477046	-0.030213937	0.058732315	-0.514434632	0.606948152	0.783213822	SMG1	protein_coding
ENSG00000157107.14	2893.42485	0.094926133	0.066565675	1.426052293	0.153853248	0.357533222	FCHO2	protein_coding
ENSG00000157110.16	5359.251306	-0.143719689	0.110145338	-1.304818637	0.191954611	0.408216038	RBPMS	protein_coding
ENSG00000157111.13	829.1315509	-0.15417748	0.139580442	-1.104577959	0.269342506	0.497441763	TMEM171	protein_coding
ENSG00000157119.12	1.411036174	-0.144297013	0.287281396	-0.502284571	0.615467361	0.789033759	KLHL40	protein_coding
ENSG00000157131.11	16.89400244	-1.801300691	0.279328351	-6.448685514	1.12824E-10	3.03491E-08	C8A	protein_coding
ENSG00000157150.5	128.3180771	0.843412198	0.159324153	5.293687021	1.19875E-07	9.31154E-06	TIMP4	protein_coding
ENSG00000157152.17	198.0128544	-0.233259057	0.196826807	-1.185098012	0.235978697	0.460883994	SYN2	protein_coding
ENSG00000157168.20	531.5590991	-0.193316061	0.193032579	-1.001468568	0.316600329	0.545876891	NRG1	protein_coding
ENSG00000157181.16	2122.161368	0.100759772	0.055682933	1.809527036	0.070369164	0.220531966	ODR4	protein_coding
ENSG00000157184.7	1086.434577	-0.128553872	0.052311362	-2.457475129	0.01399175	0.076357982	CPT2	protein_coding
ENSG00000157191.20	3390.510183	-0.009344739	0.046328349	-0.201706712	0.840146015	0.921587474	NECAP2	protein_coding
ENSG00000157193.18	1191.19602	0.051729419	0.118227646	0.437540802	0.661719192	0.818815616	LRP8	protein_coding
ENSG00000157211.11	0.667446182	0.060100353	0.310751774	0.193403089	0.846643296		CDCP2	protein_coding
ENSG00000157212.19	1492.550642	-0.154491935	0.052212465	-2.958909046	0.003087302	0.026245681	PAXIP1	protein_coding
ENSG00000157214.14	3956.290094	-0.028659993	0.109146226	-0.26258345	0.792871658	0.896174888	STEAP2	protein_coding
ENSG00000157216.15	6868.799477	-0.187136796	0.060673052	-3.084347808	0.00203999	0.019418223	SSBP3	protein_coding
ENSG00000157219.5	1.554656957	-0.311485795	0.404753163	-0.769569762	0.441555147	0.658086558	HTR5A	protein_coding
ENSG00000157224.16	4563.042332	0.236918709	0.086420958	2.741449696	0.006116873	0.042885111	CLDN12	protein_coding
ENSG00000157227.13	18636.67727	0.52097327	0.087545825	5.950863681	2.66731E-09	4.12175E-07	MMP14	protein_coding
ENSG00000157240.4	2426.155286	0.350243421	0.085788995	4.08261483	4.45318E-05	0.001051225	FZD1	protein_coding

ENSG00000157259.8	3156.052711	0.08000261	0.0762559	1.049133374	0.294116738	0.524148015	GATAD1	protein_coding
ENSG00000157303.11	325.4221531	-0.433214084	0.122273042	-3.543005703	0.000395594	0.005689375	SUSD3	protein_coding
ENSG00000157306.14	33.28614568	-0.199691572	0.094910941	-2.103988956	0.03537941	0.141742077	ZFHX2-AS1	lncRNA
ENSG00000157315.5	61.17843699	-0.581278065	0.110887984	-5.242029328	1.5882E-07	1.18104E-05	TMED6	protein_coding
ENSG00000157322.17	15.62492045	-0.173450914	0.14156907	-1.225203453	0.220498574	0.442530574	CLEC18A	protein_coding
ENSG00000157326.19	531.884304	-0.221252822	0.076349824	-2.897882545	0.003756913	0.030317589	DHRS4	protein_coding
ENSG00000157330.10	6.122148538	0.002215404	0.261567466	0.008469723	0.993242219	0.996902508	C1orf158	protein_coding
ENSG00000157335.21	3.689144713	-0.038833696	0.223147387	-0.174027116	0.861844152	0.932781527	CLEC18C	protein_coding
ENSG00000157343.9	10.85264914	-0.167377094	0.142923929	-1.171092168	0.241561732	0.467333526	ARMC12	protein_coding
ENSG00000157349.17	646.7647452	-0.035196207	0.046306039	-0.760078105	0.447207899	0.662631031	DDX19B	protein_coding
ENSG00000157350.13	2507.594319	-0.047736602	0.083254513	-0.573381546	0.566386382	0.754512787	ST3GAL2	protein_coding
ENSG00000157353.17	1598.753089	-0.076191621	0.066843844	-1.139844992	0.254350885	0.481132462	FCSK	protein_coding
ENSG00000157368.11	338.4840847	-0.127180894	0.122641366	-1.037014654	0.299729028	0.529481071	IL34	protein_coding
ENSG00000157379.14	1064.508847	-0.096465169	0.072840958	-1.324325923	0.185394825	0.399893113	DHRS1	protein_coding
ENSG00000157388.19	827.2088699	-0.07543668	0.1256009	-0.600606204	0.548102305	0.741345029	CACNA1D	protein_coding
ENSG00000157399.16	1560.354703	-0.441312909	0.150281434	-2.936576385	0.003318571	0.027709017	ARSL	protein_coding
ENSG00000157404.16	949.4284442	0.173614309	0.147675587	1.175646645	0.239736108	0.465251091	KIT	protein_coding
ENSG00000157423.18	47.76644413	0.161468218	0.211712805	0.762675729	0.445656809	0.66136721	HYDIN	protein_coding
ENSG00000157426.14	599.3533388	-0.063298547	0.048174383	-1.313946191	0.188864313	0.404230852	AASDH	protein_coding
ENSG00000157429.16	167.1578772	-0.011659635	0.061033637	-0.191036209	0.848497225	0.925700828	ZNF19	protein_coding
ENSG00000157445.15	76.57198514	0.136481831	0.136935359	0.996688012	0.31891597	0.548404935	CACNA2D3	protein_coding
ENSG00000157450.16	2174.071489	0.07450825	0.047128467	1.58096061	0.113887045	0.297622533	RNF111	protein_coding
ENSG00000157456.8	2346.61129	-0.001745355	0.084866914	-0.020565791	0.98359203	0.992677217	CCNB2	protein_coding
ENSG00000157470.12	606.0509698	-0.159105828	0.114448694	-1.390193474	0.164470135	0.372085716	FAM81A	protein_coding
ENSG00000157483.9	7084.317955	0.218363514	0.066718775	3.272894532	0.001064522	0.012039624	MYO1E	protein_coding
ENSG00000157500.12	4005.141567	-0.025069829	0.055078964	-0.455161605	0.648992981	0.810534424	APPL1	protein_coding
ENSG00000157502.13	26.02964398	0.819811828	0.270280633	3.033187463	0.002419852	0.021902202	PWWP3B	protein_coding
ENSG00000157510.14	696.0421786	0.200368269	0.085709232	2.337767628	0.019399306	0.094952662	AFAP1L1	protein_coding
ENSG00000157514.16	5340.62571	-0.031144461	0.113264811	-0.27497032	0.783339041	0.890154816	TSC22D3	protein_coding
ENSG00000157538.14	2665.172251	-0.022268755	0.045893127	-0.485230713	0.627512698	0.797051811	VPS26C	protein_coding
ENSG00000157540.22	4338.22971	0.064362445	0.042801662	1.503737034	0.13264909	0.326652881	DYRK1A	protein_coding
ENSG00000157542.11	83.6379162	0.053623332	0.192554469	0.278483964	0.780640873	0.88874039	KCNJ6	protein_coding
ENSG00000157551.19	646.1444038	-0.46203847	0.193119386	-2.39250175	0.016733948	0.086367781	KCNJ15	protein_coding
ENSG00000157554.19	901.6892935	0.033729022	0.097807293	0.344851811	0.73020578	0.859563394	ERG	protein_coding
ENSG00000157557.13	14194.31776	-0.01251422	0.089647481	-0.13959366	0.88898105	0.946992776	ETS2	protein_coding
ENSG00000157570.12	2565.741946	-0.201730795	0.130751071	-1.542861513	0.122864381	0.311953686	TSPAN18	protein_coding
ENSG00000157578.13	93.5715699	-0.137868449	0.072796057	-1.893899953	0.058238291	0.196154738	LCA5L	protein_coding
ENSG00000157593.19	3515.810193	0.348621412	0.079026389	4.411455674	1.02678E-05	0.000332669	SLC35B2	protein_coding
ENSG00000157600.12	5027.516726	0.064536263	0.07908928	0.815992541	0.414504402	0.634876043	TMEM164	protein_coding
ENSG00000157601.14	6454.571947	0.171622056	0.121932322	1.407518959	0.159273559	0.365132027	MX1	protein_coding
ENSG00000157613.11	20986.64713	0.143822567	0.14096695	1.020257353	0.307606424	0.537038728	CREB3L1	protein_coding
ENSG00000157617.17	2050.306203	0.048683095	0.05708261	0.852853341	0.393740639	0.617539563	C2CD2	protein_coding

ENSG00000157625.15	2390.986624	-0.071694975	0.052093784	-1.376267375	0.16873887	0.377994845	TAB3	protein_coding
ENSG00000157637.13	7564.463119	0.128543645	0.052702864	2.439025793	0.014726917	0.079092293	SLC38A10	protein_coding
ENSG00000157653.12	48.28175561	-0.173567951	0.098260625	-1.766403898	0.077328112	0.234906038	C9orf43	protein_coding
ENSG00000157654.19	5849.463801	0.062597113	0.086200722	0.726178518	0.467729339	0.67922692	PALM2AKAP2	protein_coding
ENSG00000157657.14	2211.462831	0.100329241	0.082241252	1.219938152	0.222488322	0.445010285	ZNF618	protein_coding
ENSG00000157680.15	151.6789613	0.150401092	0.134972107	1.114312394	0.265145192	0.492949017	DGKI	protein_coding
ENSG00000157693.15	1159.697657	-0.147677122	0.052053772	-2.837010987	0.004553804	0.034832568	TMEM268	protein_coding
ENSG00000157703.16	34.31882592	-0.139392551	0.208820293	-0.667523971	0.504437503	0.708599172	SVOPL	protein_coding
ENSG00000157734.14	134.9772559	-0.736670131	0.117743441	-6.2565704	3.93536E-10	8.40642E-08	SNX22	protein_coding
ENSG00000157741.15	2649.43977	-0.172366766	0.05291216	-3.257602158	0.001123578	0.01255762	UBN2	protein_coding
ENSG00000157764.14	2227.772523	-0.067771774	0.044754269	-1.514308605	0.129947587	0.322662451	BRAF	protein_coding
ENSG00000157765.13	1206.202677	0.56195799	0.24959988	2.251435339	0.024357978	0.111018472	SLC34A2	protein_coding
ENSG00000157766.19	864.1454891	0.285961669	0.13392226	2.135281091	0.032738057	0.135003012	ACAN	protein_coding
ENSG00000157778.9	1250.309717	0.018513223	0.069929974	0.264739456	0.79121018	0.895268125	PSMG3	protein_coding
ENSG00000157782.9	80.66520086	-0.459546504	0.129860989	-3.538757156	0.000402016	0.0057703	CABP1	protein_coding
ENSG00000157796.18	878.3464049	0.04794231	0.059888651	0.800524116	0.423407197	0.642460233	WDR19	protein_coding
ENSG00000157800.18	2610.739665	0.076598772	0.050725317	1.510069856	0.1310256	0.324230698	SLC37A3	protein_coding
ENSG00000157823.17	1281.108316	-0.056060796	0.061090173	-0.917672902	0.358790138	0.586525035	AP3S2	protein_coding
ENSG00000157827.20	2903.764886	-0.030470461	0.070751909	-0.430666268	0.666711049	0.822014129	FMNL2	protein_coding
ENSG00000157833.13	569.6606187	0.386161438	0.103555522	3.729027959	0.00019222	0.00329389	GAREM2	protein_coding
ENSG00000157837.16	4708.943401	-0.073328869	0.042667068	-1.718629203	0.085681911	0.25060478	SPPL3	protein_coding
ENSG00000157851.17	40.32022847	0.219074839	0.232403708	0.94264778	0.345861092	0.575315337	DPYSL5	protein_coding
ENSG00000157856.12	19.8329619	0.144106312	0.181921309	0.792135416	0.428281722	0.646428221	DRC1	protein_coding
ENSG00000157869.16	788.7472504	-0.025471418	0.042140867	-0.60443507	0.545554425	0.739781715	RAB28	protein_coding
ENSG00000157870.17	4870.701743	-0.074424682	0.080408826	-0.925578518	0.354665061	0.582857919	PRXL2B	protein_coding
ENSG00000157873.17	3708.782003	-0.169442842	0.072576644	-2.334674527	0.01956043	0.095548129	TNFRSF14	protein_coding
ENSG00000157881.16	1178.245486	0.012602403	0.048115797	0.261918195	0.793384512	0.896411041	PANK4	protein_coding
ENSG00000157884.11	6.965645026	0.398483223	0.204221104	1.951234302	0.05102918	0.18026091	CIB4	protein_coding
ENSG00000157890.17	60.7906949	-0.045238629	0.134477662	-0.336402555	0.736567323	0.863366981	MEGF11	protein_coding
ENSG00000157895.11	894.4025634	-0.05360251	0.042214613	-1.269761965	0.204169433	0.423550003	C12orf43	protein_coding
ENSG00000157911.11	859.6638582	-0.011069724	0.064116146	-0.172651113	0.862925673	0.933140901	PEX10	protein_coding
ENSG00000157916.20	5380.746194	-0.021811968	0.051144387	-0.426478243	0.669759398	0.823906178	RER1	protein_coding
ENSG00000157927.17	100.7213161	0.341806839	0.151764499	2.252218678	0.024308456	0.110878861	RADIL	protein_coding
ENSG00000157933.10	8482.898013	0.06989725	0.056766767	1.231305817	0.218208507	0.439816337	SKI	protein_coding
ENSG00000157954.15	4401.539476	0.065181902	0.03925051	1.660663838	0.09678098	0.269846104	WIPI2	protein_coding
ENSG00000157965.12	2.514715644	-0.195854936	0.331290527	-0.591187856	0.554394559	0.74552852	SSX8P	transcribed_unprocessed_pseudogene
ENSG00000157978.12	2261.381198	0.122205036	0.059391693	2.057611588	0.039627433	0.153182964	LDLRAP1	protein_coding
ENSG00000157985.19	4149.708144	0.012657474	0.069892446	0.181099318	0.85628962	0.929946342	AGAP1	protein_coding
ENSG00000157992.12	2389.820003	-0.201297485	0.101936575	-1.974732672	0.048298493	0.174115109	KRTCAP3	protein_coding
ENSG00000157999.6	47.56760817	0.032530913	0.087848734	0.370305996	0.711154507	0.849008826	ANKRD61	protein_coding
ENSG00000158006.14	2001.537791	-0.132990005	0.060574464	-2.195479667	0.028129208	0.122078129	PAFAH2	protein_coding
ENSG00000158008.10	30.41791031	-0.017825333	0.157302535	-0.113318793	0.909777818	0.957316312	EXTL1	protein_coding

ENSG00000158014.15	81.75652609	-0.265326451	0.224789559	-1.180332627	0.237867946	0.46318058	SLC30A2	protein_coding
ENSG00000158019.21	1783.045559	-0.189859547	0.042459	-4.471597223	7.76375E-06	0.000266185	BABAM2	protein_coding
ENSG00000158022.7	12.00328983	-0.51118235	0.194758092	-2.624704033	0.008672432	0.054667807	TRIM63	protein_coding
ENSG00000158023.10	229.0663514	0.397172556	0.129073084	3.077113715	0.002090155	0.019787683	CFAP251	protein_coding
ENSG00000158042.9	2802.043294	-0.059224732	0.057100248	-1.037206209	0.299639765	0.529463471	MRPL17	protein_coding
ENSG00000158050.5	713.0381638	-0.185926323	0.120118881	-1.547852598	0.12165779	0.310081874	DUSP2	protein_coding
ENSG00000158055.16	285.6046514	0.743292426	0.164214083	4.526362248	6.00077E-06	0.000217369	GRHL3	protein_coding
ENSG00000158062.20	976.9546188	-0.163156994	0.063646705	-2.563479022	0.010362896	0.061860813	UBXN11	protein_coding
ENSG00000158077.5	9.530036077	-0.195028927	0.183840184	-1.060861248	0.288752962	0.518510398	NLRP14	protein_coding
ENSG00000158079.16	675.1763503	0.083716641	0.057430329	1.457707834	0.144921092	0.345000077	PTPDC1	protein_coding
ENSG00000158089.15	286.1646287	0.381169601	0.197435817	1.930600064	0.05353253	0.185701883	GALNT14	protein_coding
ENSG00000158092.7	1741.545118	-0.017488033	0.05148293	-0.339686057	0.734092963	0.861981297	NCK1	protein_coding
ENSG00000158104.11	45.4793653	-0.248004981	0.129699843	-1.912145579	0.055857524	0.190999947	HPD	protein_coding
ENSG00000158106.14	1175.183049	0.182721899	0.104347151	1.751096191	0.079929342	0.240006129	RHPN1	protein_coding
ENSG00000158109.15	4324.811632	-0.061241954	0.050223385	-1.219391193	0.222695751	0.445217816	TPRG1L	protein_coding
ENSG00000158113.13	51.48421319	0.231623115	0.145066316	1.596670551	0.110339163	0.291868989	LRRC43	protein_coding
ENSG00000158122.12	938.4398377	-0.010630179	0.062976201	-0.168796768	0.865956501	0.93500911	PRXL2C	protein_coding
ENSG00000158125.10	1216.299999	0.026645956	0.168366845	0.158261302	0.874250901	0.93925487	XDH	protein_coding
ENSG00000158156.9	674.2864299	-0.049056988	0.052217015	-0.939482813	0.347482912	0.576694324	XKR8	protein_coding
ENSG00000158158.12	3807.33138	0.206685415	0.086961082	2.376757624	0.01746556	0.088654248	CNNM4	protein_coding
ENSG00000158161.16	2061.326992	-0.003843827	0.047971827	-0.080126758	0.936136441	0.969767071	EYA3	protein_coding
ENSG00000158163.15	501.9974051	-0.40018122	0.144836364	-2.762988574	0.005727477	0.041003166	DZIP1L	protein_coding
ENSG00000158164.7	27.62353302	0.694723095	0.143782818	4.831753218	1.35336E-06	6.6339E-05	TMSB15A	protein_coding
ENSG00000158169.13	1057.784303	-0.017496829	0.053665344	-0.326035908	0.744397188	0.868541221	FANCC	protein_coding
ENSG00000158186.13	1147.679087	0.342111881	0.103964112	3.290672842	0.000999481	0.011488888	MRAS	protein_coding
ENSG00000158195.11	13264.72449	0.026134215	0.046400619	0.563229893	0.57327835	0.758907015	WASF2	protein_coding
ENSG00000158201.10	2646.106013	-0.008030693	0.084374004	-0.095179702	0.924172093	0.964159838	ABHD3	protein_coding
ENSG00000158220.14	262.2155489	0.313710742	0.139256227	2.252759166	0.024274338	0.110792759	ESYT3	protein_coding
ENSG00000158234.12	574.7677832	0.169255742	0.067706893	2.499830307	0.012425281	0.070236415	FAIM	protein_coding
ENSG00000158246.8	501.9823647	-0.2595367	0.184006549	-1.410475343	0.158399372	0.36385842	TENT5B	protein_coding
ENSG00000158258.16	437.0437271	0.512824701	0.144140683	3.557806801	0.000373964	0.005470871	CLSTN2	protein_coding
ENSG00000158270.12	761.0689114	0.253328438	0.146291326	1.731670933	0.083332169	0.246428197	COLEC12	protein_coding
ENSG00000158286.13	973.1801089	0.003950028	0.098354532	0.04016112	0.967964674	0.98503098	RNF207	protein_coding
ENSG00000158290.18	4699.039256	-0.005380533	0.050722524	-0.106077784	0.915520638	0.960032363	CUL4B	protein_coding
ENSG00000158292.7	1933.187759	0.557476581	0.120737181	4.617273439	3.88815E-06	0.000152643	GPR153	protein_coding
ENSG00000158296.14	347.8726664	-0.29728011	0.183386543	-1.621057385	0.105005332	0.283683656	SLC13A3	protein_coding
ENSG00000158301.18	277.6033359	0.090925484	0.093508211	0.972379679	0.330861697	0.560417345	GPRASP2	protein_coding
ENSG00000158315.11	555.7285154	-0.011860754	0.140661669	-0.084321155	0.932801093	0.968238421	RHBDL2	protein_coding
ENSG00000158321.18	2379.538304	0.043915775	0.105805947	0.415059611	0.678098265	0.829354821	AUTS2	protein_coding
ENSG00000158352.16	1161.157154	-0.014523903	0.09881457	-0.146981392	0.883146708	0.943951045	SHROOM4	protein_coding
ENSG00000158373.8	886.5792123	-0.054229677	0.118637819	-0.457102781	0.647597171	0.809771778	H2BC5	protein_coding
ENSG00000158402.20	498.6324138	-0.113604001	0.093724005	-1.212112105	0.225469471	0.448499709	CDC25C	protein_coding

ENSG00000158406.5	389.8156608	0.031812628	0.137892777	0.230705542	0.817543568	0.910099918	H4C8	protein_coding
ENSG00000158411.11	959.9979046	-0.207181824	0.054184309	-3.823649829	0.000131491	0.002441401	MITD1	protein_coding
ENSG00000158417.11	14316.85528	-0.169490518	0.066264842	-2.557774431	0.01053444	0.062542509	EIF5B	protein_coding
ENSG00000158423.17	72.49632707	-0.250859247	0.093020047	-2.696829942	0.007000301	0.047075944	RIBC1	protein_coding
ENSG00000158427.15	21.1369574	-0.477073393	0.145788283	-3.272371295	0.001066494	0.0120588	TMSB15B	protein_coding
ENSG00000158428.4	9.730978311	-0.011030442	0.144554095	-0.076306676	0.939175115	0.97113172	CATIP	protein_coding
ENSG00000158435.8	4436.797585	0.020738399	0.04702205	0.441035615	0.659187214	0.817316372	CNOT11	protein_coding
ENSG00000158445.10	228.3482922	-0.389937427	0.210190892	-1.855158537	0.063573591	0.207208029	KCNB1	protein_coding
ENSG00000158457.6	1130.955227	-0.177152945	0.095970288	-1.845914487	0.064904623	0.209725294	TSPAN33	protein_coding
ENSG00000158458.21	65.59324149	0.182079735	0.16850056	1.080588312	0.279880284	0.508647224	NRG2	protein_coding
ENSG00000158467.16	5898.027898	-0.005296024	0.125325815	-0.042258042	0.966292993	0.984499865	AHCYL2	protein_coding
ENSG00000158470.5	10295.83981	0.112419635	0.062945714	1.785977587	0.074102887	0.228194581	B4GALT5	protein_coding
ENSG00000158473.8	158.1542731	-0.31898317	0.115152692	-2.770088685	0.005604103	0.040345284	CD1D	protein_coding
ENSG00000158477.7	59.11278201	-0.300494905	0.163087735	-1.84253527	0.065396892	0.210723131	CD1A	protein_coding
ENSG00000158480.11	2501.31933	-0.096973421	0.067758388	-1.431164807	0.152382987	0.355449091	SPATA2	protein_coding
ENSG00000158481.13	158.0564048	-0.489196769	0.167391894	-2.922463912	0.003472739	0.028663862	CD1C	protein_coding
ENSG00000158482.10	14.40063858	-0.702330582	0.156095517	-4.499364207	6.8157E-06	0.000240492	SNX29P1	unprocessed_pseudogene
ENSG00000158483.17	381.6582944	-0.020924818	0.062621495	-0.334147535	0.738268232	0.864523546	FAM86C1P	transcribed_unprocessed_pseudogene
ENSG00000158485.11	20.29144862	-0.273291019	0.169876595	-1.608762047	0.107668383	0.287568308	CD1B	protein_coding
ENSG00000158486.13	189.6791845	0.005170192	0.127756542	0.040469099	0.967719142	0.984878251	DNAH3	protein_coding
ENSG00000158488.16	80.03025911	-0.527704069	0.168413253	-3.133388018	0.001728008	0.017235391	CD1E	protein_coding
ENSG00000158497.5	1.992574613	-0.12218884	0.229511788	-0.532385899	0.594458749	0.77430688	HMHB1	protein_coding
ENSG00000158516.12	230.3081522	-2.794047687	0.298970792	-9.345554011	9.14097E-21	3.98336E-17	CPA2	protein_coding
ENSG00000158517.15	344.2476629	-0.394319928	0.135501422	-2.910079631	0.003613367	0.029481312	NCF1	protein_coding
ENSG00000158525.16	14.90204828	-0.596106526	0.21155182	-2.817780182	0.00483569	0.036347651	CPA5	protein_coding
ENSG00000158526.8	1908.770584	-0.068061487	0.048387912	-1.406580359	0.159551861	0.365321113	TSR2	protein_coding
ENSG00000158528.12	1297.5869	0.310934317	0.168382853	1.846591329	0.064806392	0.209547239	PPP1R9A	protein_coding
ENSG00000158545.16	3301.925254	-0.043624132	0.050765603	-0.85932461	0.39016145	0.614593883	ZC3H18	protein_coding
ENSG00000158552.13	1265.053729	-0.097581344	0.053452971	-1.825555101	0.067917304	0.215873987	ZFAND2B	protein_coding
ENSG00000158553.4	6.947971612	0.52463865	0.245227669	2.139394185	0.032403758	0.134009542	POM121L2	protein_coding
ENSG00000158555.15	1522.50814	0.168490961	0.125458565	1.343000864	0.179271694	0.392155144	GDPD5	protein_coding
ENSG00000158560.14	219.1773013	-0.132936494	0.160751084	-0.826971057	0.408253466	0.629305316	DYNC1I1	protein_coding
ENSG00000158571.11	20.96188095	0.089642055	0.111830153	0.8015911	0.422789528	0.642014818	PFKFB1	protein_coding
ENSG00000158578.21	15.51957138	-0.003865453	0.20300368	-0.019041297	0.984808161	0.993126568	ALAS2	protein_coding
ENSG00000158604.15	6055.228825	-0.097209399	0.045194392	-2.150917305	0.031482727	0.131700376	TMED4	protein_coding
ENSG00000158615.9	6478.943365	0.122758391	0.047263503	2.597318943	0.009395464	0.057820382	PPP1R15B	protein_coding
ENSG00000158623.14	1303.096919	-0.024603793	0.068644371	-0.358424038	0.720026005	0.853585802	COPG2	protein_coding
ENSG00000158636.16	1827.945259	0.075336955	0.04664587	1.615083076	0.106292706	0.285550658	EMSY	protein_coding
ENSG00000158639.13	8.864336151	0.131307505	0.316033351	0.415486228	0.677785995	0.829300333	PAGE5	protein_coding
ENSG00000158669.11	3114.879493	0.0509513	0.050288981	1.013170269	0.310978839	0.540471882	GPAT4	protein_coding
ENSG00000158683.9	115.4116331	-0.170281056	0.111846765	-1.522449533	0.127896482	0.319334497	PKD1L1	protein_coding
ENSG00000158691.14	357.3643239	-0.043783964	0.071209088	-0.614864838	0.538643986	0.734485596	ZSCAN12	protein_coding

ENSG00000158710.15	41044.22099	0.027561706	0.069851473	0.394575876	0.69315592	0.837965038	TAGLN2	protein_coding
ENSG00000158711.14	4286.786356	0.024800647	0.052065997	0.476330976	0.633838601	0.801374495	ELK4	protein_coding
ENSG00000158714.11	338.5870731	-0.052747408	0.140608701	-0.375136159	0.707559206	0.846857939	SLAMF8	protein_coding
ENSG00000158715.6	1987.214231	0.284065973	0.112791777	2.518498955	0.011785623	0.067737023	SLC45A3	protein_coding
ENSG00000158716.9	1811.262371	0.157471534	0.092077774	1.710201353	0.087228646	0.2532665	DUSP23	protein_coding
ENSG00000158717.11	924.2211976	-0.172976916	0.061628971	-2.806746783	0.005004456	0.037227585	RNF166	protein_coding
ENSG00000158731.2	0.194442528	0.013557551	0.858960889	0.015783665	0.98740698		OR10J6P	unprocessed_pseudogene
ENSG00000158747.15	8755.747665	0.197476903	0.091676285	2.154067457	0.031234882	0.131031439	NBL1	protein_coding
ENSG00000158748.4	22.2608684	-0.03405132	0.124338915	-0.273858917	0.784193045	0.890519109	HTR6	protein_coding
ENSG00000158764.7	22.84776023	-0.225776513	0.268269302	-0.841603982	0.40000966	0.622728091	ITLN2	protein_coding
ENSG00000158769.18	5599.811454	0.158346233	0.069996261	2.262209878	0.023684439	0.108905436	F11R	protein_coding
ENSG00000158773.14	2335.337666	-0.00892729	0.043648524	-0.204526724	0.837941911	0.92021357	USF1	protein_coding
ENSG00000158786.5	146.9847465	0.407787426	0.238884971	1.707045131	0.087813664	0.254508693	PLA2G2F	protein_coding
ENSG00000158792.16	1028.522082	0.096943196	0.074137978	1.307605068	0.191007297	0.406892377	SPATA2L	protein_coding
ENSG00000158793.14	1366.012347	0.011943089	0.050728376	0.235432129	0.813873352	0.908061527	NIT1	protein_coding
ENSG00000158796.17	1533.321181	0.059628987	0.040488996	1.472720817	0.140826331	0.338656201	DEDD	protein_coding
ENSG00000158805.12	936.7678485	0.047457282	0.059133572	0.802543797	0.422238464	0.641535705	ZNF276	protein_coding
ENSG00000158806.14	106.5104162	0.368341614	0.149803678	2.458828916	0.013939103	0.0762234	NPM2	protein_coding
ENSG00000158813.18	259.5402361	0.119050511	0.156217114	0.762083666	0.446010071	0.66162108	EDA	protein_coding
ENSG00000158815.11	13.00716516	0.321013217	0.166501527	1.927989627	0.053856416	0.186565805	FGF17	protein_coding
ENSG00000158816.15	73.66220326	0.957932086	0.267526443	3.580700563	0.000342674	0.005129754	VWA5B1	protein_coding
ENSG00000158825.6	1192.517772	1.013916887	0.184137454	5.506304476	3.66445E-08	3.5407E-06	CDA	protein_coding
ENSG00000158828.8	1064.857926	0.193376775	0.076055531	2.542573463	0.011003947	0.064538221	PINK1	protein_coding
ENSG00000158850.15	2399.419755	0.075194618	0.055824851	1.346973917	0.177988633	0.390471716	B4GALT3	protein_coding
ENSG00000158856.18	3157.748382	0.138636556	0.092443581	1.499688301	0.133695163	0.328356766	DMTN	protein_coding
ENSG00000158859.10	2452.90302	0.26974522	0.107763259	2.503127898	0.012310105	0.069757795	ADAMTS4	protein_coding
ENSG00000158863.22	3679.235677	0.033643498	0.05081791	0.662040181	0.507945471	0.711747662	FAM160B2	protein_coding
ENSG00000158864.13	5670.704705	0.009975272	0.050897858	0.195986092	0.844621052	0.92378154	NDUFS2	protein_coding
ENSG00000158865.12	30.60789852	-0.078670329	0.174926066	-0.449734741	0.652901718	0.813341476	SLC5A11	protein_coding
ENSG00000158869.11	4488.601196	-0.140973862	0.117622114	-1.198531946	0.230709994	0.45465673	FCER1G	protein_coding
ENSG00000158874.11	217.9242717	-3.347604289	0.312980813	-10.69587702	1.06408E-26	1.54565E-22	APOA2	protein_coding
ENSG00000158882.15	563.7836258	-0.021260692	0.06992522	-0.304048987	0.761090571	0.877663971	TOMM40L	protein_coding
ENSG00000158887.19	106.7240361	-0.236779676	0.121540457	-1.948155226	0.051396392	0.181102983	MPZ	protein_coding
ENSG00000158901.12	1.958460704	0.23140013	0.299655809	0.772219738	0.439984293	0.656526351	WFDC8	protein_coding
ENSG00000158941.16	4019.98447	-0.078978765	0.053593445	-1.473664639	0.140571908	0.338361801	CCAR2	protein_coding
ENSG00000158955.11	34.6818859	0.059282771	0.138968109	0.426592631	0.669676066	0.823898191	WNT9B	protein_coding
ENSG00000158966.16	1155.590999	-0.458940808	0.117154746	-3.917389808	8.95129E-05	0.001821058	CACHD1	protein_coding
ENSG00000158985.14	4648.383894	-0.051270083	0.064115773	-0.799648519	0.423914468	0.642965805	CDC42SE2	protein_coding
ENSG00000158987.22	716.5055097	-0.198280883	0.057263728	-3.462591241	0.000535	0.007153641	RAPGEF6	protein_coding
ENSG00000159023.22	4157.967589	-0.185489434	0.066623666	-2.784137317	0.005367031	0.039064489	EPB41	protein_coding
ENSG00000159055.4	905.1360943	-0.245519318	0.084081083	-2.920030382	0.003499973	0.028828943	MIS18A	protein_coding
ENSG00000159063.13	2666.41873	-0.021844152	0.073594333	-0.296818401	0.766605157	0.881550413	ALG8	protein_coding

ENSG00000159069.14	8091.149692	0.005600891	0.052822049	0.106033196	0.915556014	0.960032363	FBXW5	protein_coding
ENSG00000159079.19	517.9687542	-0.148408827	0.072235297	-2.054519502	0.039925438	0.153790401	CFAP298	protein_coding
ENSG00000159082.18	1128.027271	-0.075797443	0.052607116	-1.440821095	0.149635232	0.351745308	SYNJ1	protein_coding
ENSG00000159086.15	1676.158332	-0.095055114	0.059118948	-1.60786206	0.107865393	0.287823784	PAXBP1	protein_coding
ENSG00000159110.20	115.0997406	-0.114900046	0.067340448	-1.706256037	0.087960419	0.254721637	IFNAR2	protein_coding
ENSG00000159111.13	3326.141631	-0.027004226	0.050103435	-0.538969556	0.589907861	0.771130756	MRPL10	protein_coding
ENSG00000159128.15	3702.916295	0.054722752	0.063869717	0.856787136	0.391562536	0.615631588	IFNGR2	protein_coding
ENSG00000159131.17	5004.20646	0.004409058	0.054733681	0.080554754	0.93579605	0.969736361	GART	protein_coding
ENSG00000159140.21	20795.99723	0.031502544	0.040236526	0.782933984	0.43366588	0.651522669	SON	protein_coding
ENSG00000159147.18	868.3401986	0.017078549	0.064838859	0.263399899	0.792242368	0.895784787	DONSON	protein_coding
ENSG00000159164.10	412.6053562	0.375107718	0.129270128	2.901735481	0.003711018	0.030052966	SV2A	protein_coding
ENSG00000159166.15	11211.98784	0.215194533	0.098042008	2.194921726	0.028169213	0.122152503	LAD1	protein_coding
ENSG00000159167.12	2805.917909	0.327588959	0.112754556	2.905327919	0.003668685	0.029793197	STC1	protein_coding
ENSG00000159173.19	339.159584	-0.016501614	0.104349454	-0.158137999	0.874348058	0.93925487	TNNI1	protein_coding
ENSG00000159176.14	25614.69417	-0.312412596	0.138054595	-2.262964133	0.0236379	0.108771783	CSRP1	protein_coding
ENSG00000159182.5	3.005109209	0.994005026	0.517393661	1.921177434	0.054709342	0.188493754	PRAC1	protein_coding
ENSG00000159184.8	687.2680765	0.121836947	0.195179041	0.624231712	0.532475424	0.72998923	HOXB13	protein_coding
ENSG00000159186.4	2.908326819	-0.202947772	0.143566274	-1.413617326	0.157474291	0.362526134	ATP5POP1	processed_pseudogene
ENSG00000159189.12	10723.10308	-0.029582288	0.127273729	-0.23243043	0.816203717	0.909311245	C1QC	protein_coding
ENSG00000159197.4	18.23297345	-0.906846239	0.302216443	-3.000651553	0.002694027	0.023707107	KCNE2	protein_coding
ENSG00000159199.14	4782.672478	-0.197855077	0.077474188	-2.553819308	0.010654853	0.063119432	ATP5MC1	protein_coding
ENSG00000159200.18	2636.498539	0.175538713	0.078346838	2.240533461	0.025056311	0.113335983	RCAN1	protein_coding
ENSG00000159202.18	7215.85979	0.003531345	0.047958073	0.073634004	0.941301613	0.972219864	UBE2Z	protein_coding
ENSG00000159208.16	241.9951714	-0.02674591	0.133474253	-0.200382538	0.841181415	0.921982055	CIART	protein_coding
ENSG00000159210.10	1563.96886	-0.060329028	0.048647678	-1.240121432	0.214930483	0.436014416	SNF8	protein_coding
ENSG00000159212.13	1767.531653	-0.027412514	0.191032022	-0.143496957	0.885897714	0.945453681	CLIC6	protein_coding
ENSG00000159214.13	612.4798257	0.112356189	0.092022459	1.220964862	0.222099324	0.444660469	CCDC24	protein_coding
ENSG00000159216.19	8078.930957	0.281108091	0.068940983	4.07751788	4.5519E-05	0.001065297	RUNX1	protein_coding
ENSG00000159217.10	938.6413529	0.356468062	0.293327395	1.215256633	0.224268227	0.447171654	IGF2BP1	protein_coding
ENSG00000159224.4	14.33791009	-0.641691043	0.28009992	-2.290936187	0.021967105	0.103429894	GIP	protein_coding
ENSG00000159228.13	1950.625456	-0.156455983	0.088203828	-1.773800388	0.076096174	0.23239491	CBR1	protein_coding
ENSG00000159231.6	53.72639547	0.365899705	0.100352743	3.646135571	0.000266214	0.004240054	CBR3	protein_coding
ENSG00000159239.13	75.53206185	0.317649748	0.196565205	1.616001919	0.106093898	0.28513962	AC005041.1	protein_coding
ENSG00000159247.13	155.2097627	0.220191207	0.204787973	1.075215519	0.282278249	0.511044423	TUBBP5	transcribed_unprocessed_pseudogene
ENSG00000159248.5	6.564987239	-0.350344701	0.279683591	-1.252646606	0.210334346	0.430680377	GJD2	protein_coding
ENSG00000159251.8	294.383288	-0.200159434	0.190761642	-1.049264578	0.294056364	0.524093831	ACTC1	protein_coding
ENSG00000159256.13	2156.453383	-0.019578351	0.047898292	-0.408748424	0.682724293	0.832278641	MORC3	protein_coding
ENSG00000159259.8	852.355809	-0.155504307	0.080970077	-1.920515747	0.054792786	0.188661918	CHAF1B	protein_coding
ENSG00000159261.12	39.7945549	0.152251652	0.149111957	1.021055962	0.307227926	0.536768155	CLDN14	protein_coding
ENSG00000159263.16	1592.869452	0.230467754	0.120952099	1.9054465	0.056722056	0.19292671	SIM2	protein_coding
ENSG00000159267.15	1846.062842	0.116087834	0.053128303	2.185046923	0.028885406	0.124393651	HLCS	protein_coding
ENSG00000159289.6	2.397384884	-0.065304828	0.225410032	-0.289715713	0.772033734	0.884611033	GOLGA6A	protein_coding

ENSG00000159307.19	479.9657131	-0.499838959	0.154439904	-3.236462505	0.001210211	0.013273943	SCUBE1	protein_coding
ENSG00000159314.11	4368.808345	0.09734907	0.077684131	1.253139715	0.210154865	0.430516547	ARHGAP27	protein_coding
ENSG00000159322.17	2987.77011	0.001037462	0.044613377	0.023254506	0.981447261	0.991917974	ADPGK	protein_coding
ENSG00000159335.18	13453.63589	-0.004959023	0.088502961	-0.056032279	0.955316093	0.979225399	PTMS	protein_coding
ENSG00000159337.7	43.54931135	0.481107618	0.184918395	2.601729356	0.009275502	0.057324996	PLA2G4D	protein_coding
ENSG00000159339.13	25.91304297	0.331652849	0.174490058	1.90069768	0.057341623	0.194321167	PADI4	protein_coding
ENSG00000159346.13	9056.060754	0.124073149	0.049390952	2.512062293	0.01200279	0.068515271	ADIPOR1	protein_coding
ENSG00000159348.13	1998.555929	0.011662536	0.06458399	0.180579366	0.856697753	0.930099108	CYB5R1	protein_coding
ENSG00000159352.16	6773.732236	0.027365149	0.047204505	0.579714773	0.562106979	0.751036033	PSMD4	protein_coding
ENSG00000159363.19	5613.551793	0.074234184	0.064514499	1.150658933	0.249872578	0.476612857	ATP13A2	protein_coding
ENSG00000159374.17	187.3788624	0.167873914	0.172443115	0.973503141	0.3303033	0.559845465	M1AP	protein_coding
ENSG00000159377.11	9698.830098	-0.00212632	0.057647591	-0.036884803	0.970576857	0.986192266	PSMB4	protein_coding
ENSG00000159387.8	8.495073088	0.29964786	0.27102531	1.105608403	0.268896051	0.496933131	IRX6	protein_coding
ENSG00000159388.6	9699.391361	-0.382434333	0.102413499	-3.734218009	0.000188299	0.003256159	BTG2	protein_coding
ENSG00000159398.16	6.589316401	-0.264703537	0.273879255	-0.966497215	0.33379544	0.563310506	CES5A	protein_coding
ENSG00000159399.10	5377.185171	0.651878648	0.115532767	5.642370254	1.67725E-08	1.86453E-06	HK2	protein_coding
ENSG00000159403.18	11263.12624	0.17867169	0.110481727	1.617205798	0.105833865	0.284842206	C1R	protein_coding
ENSG00000159409.15	83.60531153	-0.233667869	0.189788764	-1.231199703	0.218248182	0.439816337	CELF3	protein_coding
ENSG00000159423.17	1504.594197	-0.345862768	0.093692249	-3.691476853	0.000222956	0.003692794	ALDH4A1	protein_coding
ENSG00000159433.12	809.9637085	-0.227548216	0.123920774	-1.836239462	0.066322249	0.212556051	STARD9	protein_coding
ENSG00000159445.13	1641.756809	-0.067504931	0.072855484	-0.926559368	0.354155359	0.582374871	THEM4	protein_coding
ENSG00000159450.12	46.05900179	0.675100903	0.146141587	4.619498918	3.84668E-06	0.000151448	TCHH	protein_coding
ENSG00000159455.9	0.169785905	0.152343465	1.212543314	0.125639607	0.90001721		LCE2B	protein_coding
ENSG00000159459.12	2662.377754	0.028813181	0.05328438	0.540743476	0.588684407	0.770452806	UBR1	protein_coding
ENSG00000159461.15	4893.160705	-0.086598107	0.050537369	-1.713546002	0.086612138	0.2521409	AMFR	protein_coding
ENSG00000159479.17	1806.721516	0.053996	0.04217996	1.280133959	0.200498027	0.419008932	MED8	protein_coding
ENSG00000159495.8	2.169916799	0.070009432	0.382899999	0.18283999	0.85492357	0.929168335	TGM7	protein_coding
ENSG00000159496.14	55.68435266	-0.317844229	0.096866524	-3.281259779	0.001033445	0.011764482	RGL4	protein_coding
ENSG00000159516.9	8.382940849	1.16714499	0.439238283	2.657202335	0.007879214	0.051216065	SPRR2G	protein_coding
ENSG00000159527.3	29.22926958	0.582513139	0.279231462	2.086130035	0.036966846	0.146007816	PGLYRP3	protein_coding
ENSG00000159556.10	84.09057338	0.055957033	0.125693261	0.445187212	0.656184467	0.815319427	ISL2	protein_coding
ENSG00000159579.14	2462.592585	0.065812364	0.042959269	1.53197122	0.125529537	0.315813211	RSPRY1	protein_coding
ENSG00000159588.15	95.45788612	0.083268895	0.098950597	0.841519889	0.400056748	0.622728091	CCDC17	protein_coding
ENSG00000159592.11	6748.097958	-0.011058076	0.038170649	-0.289701031	0.772044968	0.884611033	GPBP1L1	protein_coding
ENSG00000159593.15	2797.864914	-0.117155956	0.055338971	-2.117060625	0.034254703	0.138818673	NAE1	protein_coding
ENSG00000159596.7	2160.667389	-0.091258287	0.048510621	-1.8812022	0.059944419	0.199800974	TMEM69	protein_coding
ENSG00000159618.16	745.5906588	-0.091250301	0.10045206	-0.90839651	0.363668769	0.590987825	ADGRG5	protein_coding
ENSG00000159625.15	19.68376188	0.219935625	0.205874575	1.068299108	0.285385611	0.514724091	DRC7	protein_coding
ENSG00000159640.17	2435.567976	0.248601367	0.088180439	2.819234836	0.004813829	0.036248869	ACE	protein_coding
ENSG00000159648.11	8.489058181	0.020933136	0.183445	0.11411124	0.909149612	0.957009712	TEPP	protein_coding
ENSG00000159650.9	5.702598442	0.319636391	0.215178126	1.485450206	0.137424605	0.333828641	UROC1	protein_coding
ENSG00000159658.14	12467.91355	-0.042025949	0.048836397	-0.860545644	0.389488334	0.613967671	EFCAB14	protein_coding

ENSG00000159674.12	5005.578444	0.53077892	0.103723459	5.117250498	3.10022E-07	2.02019E-05	SPON2	protein_coding
ENSG00000159685.11	395.9837108	0.013916011	0.058156336	0.239286241	0.810883633	0.906396719	CHCHD6	protein_coding
ENSG00000159692.16	5465.69051	0.022118994	0.053142457	0.416220764	0.677248467	0.829048161	CTBP1	protein_coding
ENSG00000159708.18	77.2328872	-0.723771887	0.148776317	-4.864832672	1.14554E-06	5.7978E-05	LRRC36	protein_coding
ENSG00000159712.10	7.546217216	-0.119164398	0.214878861	-0.554565476	0.579191909	0.763396643	ANKRD18CP	unprocessed_pseudogene
ENSG00000159713.11	2878.658496	-0.574640385	0.167902822	-3.422458168	0.000620576	0.007993751	TPPP3	protein_coding
ENSG00000159714.12	1514.092541	-0.362213904	0.102007094	-3.550869756	0.00038396	0.005571709	ZDHHC1	protein_coding
ENSG00000159720.12	5874.446863	-0.046779714	0.060822776	-0.76911508	0.441824997	0.658277786	ATP6V0D1	protein_coding
ENSG00000159723.5	9.058211424	-0.160223647	0.125643178	-1.275227594	0.202228697	0.421168033	AGRP	protein_coding
ENSG00000159733.14	858.7712762	-0.036599295	0.078124597	-0.468473396	0.639446092	0.804769454	ZFYVE28	protein_coding
ENSG00000159753.14	461.9446229	-0.372050389	0.140855145	-2.641368836	0.008257177	0.052907369	CARMIL2	protein_coding
ENSG00000159761.15	56.38255123	0.045818281	0.087056542	0.526304859	0.598676408	0.777647604	C16orf86	protein_coding
ENSG00000159763.4	6.626779466	0.970147512	0.301664772	3.215978802	0.001300004	0.013994637	PIP	protein_coding
ENSG00000159784.18	263.4618315	-0.062257927	0.188963468	-0.329470706	0.741799932	0.866756782	FAM131B	protein_coding
ENSG00000159788.19	1553.69993	0.144299969	0.068094032	2.119128003	0.034079651	0.138348025	RGS12	protein_coding
ENSG00000159792.10	1909.090837	0.01781988	0.058112442	0.306644838	0.759113721	0.876683503	PSKH1	protein_coding
ENSG00000159840.16	16135.19776	0.030056074	0.076013815	0.39540279	0.69254565	0.837699816	ZYX	protein_coding
ENSG00000159842.15	9223.028707	0.12438881	0.057816888	2.15142692	0.031442518	0.131611649	ABR	protein_coding
ENSG00000159860.7	4.736116502	-0.24683734	0.153363263	-1.609494577	0.10750824	0.287398722	TCAF2P1	transcribed_unprocessed_pseudogene
ENSG00000159871.15	343.6231611	0.340084053	0.107131503	3.174454236	0.001501186	0.015534829	LYPD5	protein_coding
ENSG00000159873.10	2383.219031	-0.085501051	0.053686743	-1.59259152	0.111251857	0.293321768	CCDC117	protein_coding
ENSG00000159882.13	262.2394426	0.063181853	0.064254769	0.983302164	0.325458746	0.555135939	ZNF230	protein_coding
ENSG00000159884.12	298.7981573	-0.113842106	0.101736899	-1.118985412	0.263146363	0.49079468	CCDC107	protein_coding
ENSG00000159885.14	95.50619601	0.136018471	0.076874337	1.769361228	0.076833612	0.233943872	ZNF222	protein_coding
ENSG00000159899.15	545.7438928	0.133911636	0.084774462	1.579622367	0.114193374	0.298154085	NPR2	protein_coding
ENSG00000159904.11	10.16117272	0.059977429	0.169391188	0.354076442	0.723281596	0.855433359	ZNF890P	transcribed_unprocessed_pseudogene
ENSG00000159905.15	98.9955478	0.210525116	0.108378289	1.942502673	0.052076277	0.182546995	ZNF221	protein_coding
ENSG00000159915.12	106.0695719	-0.053596206	0.09362313	-0.572467572	0.567005249	0.754708077	ZNF233	protein_coding
ENSG00000159917.17	209.4430925	-0.028359096	0.072400075	-0.391699822	0.695280021	0.839309053	ZNF235	protein_coding
ENSG00000159921.19	3120.086539	0.724179017	0.123345399	5.871147381	4.32789E-09	6.18349E-07	GNE	protein_coding
ENSG00000159958.7	422.6777654	-0.873608286	0.172021402	-5.078486023	3.80454E-07	2.38548E-05	TNFRSF13C	protein_coding
ENSG00000159961.2	4.589323687	0.010053975	0.2549796	0.039430507	0.968547158	0.985433379	OR3A3	protein_coding
ENSG00000160007.19	7710.595051	0.076364228	0.05423274	1.408083549	0.159106332	0.364876151	ARHGAP35	protein_coding
ENSG00000160013.9	279.2525984	0.055679978	0.098010873	0.568100009	0.569967061	0.756598932	PTGIR	protein_coding
ENSG00000160014.17	18958.06342	-0.031160776	0.060742824	-0.512995177	0.607954694	0.784088653	CALM3	protein_coding
ENSG00000160049.12	3875.923613	-0.028205997	0.052532787	-0.536921769	0.591321653	0.771936841	DFFA	protein_coding
ENSG00000160050.15	222.0809422	-0.138249947	0.076126134	-1.816064196	0.069360525	0.218532543	CCDC28B	protein_coding
ENSG00000160051.12	165.959252	-0.088773507	0.054391409	-1.632123703	0.102653429	0.279874452	IQCC	protein_coding
ENSG00000160055.20	335.867468	0.065976372	0.05247495	1.257292704	0.208647665	0.428656796	TMEM234	protein_coding
ENSG00000160058.19	5259.581678	0.020470897	0.037537107	0.545350953	0.585512166	0.767918608	BSDC1	protein_coding
ENSG00000160062.15	510.0016297	0.09883473	0.064031377	1.543535908	0.122700802	0.311629144	ZBTB8A	protein_coding
ENSG00000160072.20	1516.739587	-0.077604213	0.079945015	-0.970719846	0.331687807	0.561188055	ATAD3B	protein_coding

ENSG00000160075.12	6896.092484	-0.13753308	0.048888981	-2.813171333	0.004905551	0.036717481	SSU72	protein_coding
ENSG00000160087.21	2472.465401	-0.043639592	0.050702535	-0.860698424	0.389404161	0.613909234	UBE2J2	protein_coding
ENSG00000160094.15	2125.44411	0.021884238	0.070488754	0.310464248	0.756207943	0.874974073	ZNF362	protein_coding
ENSG00000160097.20	127.7229979	-0.193375868	0.164851186	-1.173032924	0.240782603	0.466295595	FNDC5	protein_coding
ENSG00000160111.14	264.4796624	0.547733212	0.127662235	4.290487426	1.78281E-05	0.000507776	CPAMD8	protein_coding
ENSG00000160113.5	5105.844147	-0.044603472	0.075629871	-0.589759994	0.555351567	0.746356559	NR2F6	protein_coding
ENSG00000160117.16	53.08972751	-0.052939467	0.142440383	-0.371660522	0.710145621	0.84845083	ANKLE1	protein_coding
ENSG00000160124.9	1070.330461	-0.213034971	0.070089235	-3.039482022	0.002369853	0.021586769	CCDC58	protein_coding
ENSG00000160131.13	3489.453992	0.026958515	0.058955619	0.45726795	0.647478463	0.809749828	VMA21	protein_coding
ENSG00000160145.15	3017.409923	-0.412271027	0.090649303	-4.547977915	5.41638E-06	0.000199687	KALRN	protein_coding
ENSG00000160161.9	362.2188847	0.9587253	0.190091481	5.0434943	4.57106E-07	2.75039E-05	CILP2	protein_coding
ENSG00000160172.10	230.5967438	-0.054654221	0.075117439	-0.727583654	0.46686849	0.678395216	FAM86C2P	transcribed_unprocessed_pseudogene
ENSG00000160179.19	1645.805401	-0.015120994	0.089692581	-0.168586899	0.866121586	0.935082793	ABCG1	protein_coding
ENSG00000160180.15	9567.343029	0.67477633	0.216062205	3.123065093	0.001789782	0.017661529	TFF3	protein_coding
ENSG00000160181.9	13275.65408	0.164389965	0.288425103	0.569957202	0.568706726	0.755755325	TFF2	protein_coding
ENSG00000160182.3	28160.78932	-0.472100989	0.245699534	-1.921456592	0.05467417	0.188402364	TFF1	protein_coding
ENSG00000160183.17	2775.127243	0.241938057	0.148164184	1.632905137	0.10248895	0.279566048	TMPRSS3	protein_coding
ENSG00000160185.15	181.6768287	-0.394718433	0.124408128	-3.172770442	0.001509919	0.01559901	UBASH3A	protein_coding
ENSG00000160188.10	294.5731116	-0.184190797	0.111750976	-1.648225395	0.099306427	0.274493114	RSPH1	protein_coding
ENSG00000160190.14	5291.161746	-0.075970355	0.094590503	-0.803149924	0.421888085	0.641210046	SLC37A1	protein_coding
ENSG00000160191.18	474.8713666	0.187636454	0.146247259	1.283008346	0.199489152	0.417685203	PDE9A	protein_coding
ENSG00000160193.12	844.0982106	-0.119276597	0.065104117	-1.83208993	0.066938023	0.213790549	WDR4	protein_coding
ENSG00000160194.18	2192.066702	-0.111143159	0.055663389	-1.996701281	0.045857643	0.16833784	NDUFV3	protein_coding
ENSG00000160199.15	1353.890623	0.063958117	0.041161561	1.553831204	0.120224685	0.307921478	PKNOX1	protein_coding
ENSG00000160200.18	30.71764942	-0.052966624	0.187870085	-0.281932185	0.77799551	0.887575211	CBS	protein_coding
ENSG00000160201.12	118.6073982	-0.137914738	0.117017838	-1.17857876	0.238565954	0.463982351	U2AF1	protein_coding
ENSG00000160202.7	0.361987857	-0.099587321	0.331013216	-0.300856027	0.763524283		CRYAA	protein_coding
ENSG00000160207.9	39.29019223	0.099604992	0.120457073	0.826892018	0.408298268	0.629329854	HSF2BP	protein_coding
ENSG00000160208.13	3862.900798	0.019944151	0.05461868	0.365152565	0.714997525	0.851074674	RRP1B	protein_coding
ENSG00000160209.19	9298.728025	0.104584702	0.066271095	1.578134505	0.114534713	0.298598814	PDXK	protein_coding
ENSG00000160211.19	5001.365329	0.009366201	0.089269237	0.104920818	0.916438639	0.960383969	G6PD	protein_coding
ENSG00000160213.9	14029.68016	0.470446153	0.112999102	4.16327339	3.13717E-05	0.000789767	CSTB	protein_coding
ENSG00000160214.13	1595.164442	-0.029537451	0.066799678	-0.442179537	0.658359292	0.816656953	RRP1	protein_coding
ENSG00000160216.21	6193.793864	-0.060665372	0.047408702	-1.279625239	0.200676969	0.419238712	AGPAT3	protein_coding
ENSG00000160218.13	2345.374046	0.007208489	0.057384152	0.125618121	0.900034218	0.952238301	TRAPPC10	protein_coding
ENSG00000160219.12	327.7659295	-0.331679349	0.097381633	-3.405974402	0.000659284	0.008356089	GAB3	protein_coding
ENSG00000160221.18	126.4307552	-0.28107759	0.16048006	-1.751479841	0.079863291	0.239937272	GATD3A	protein_coding
ENSG00000160223.17	145.5267815	-0.076487597	0.12249163	-0.624431207	0.532344436	0.729886212	ICOSLG	protein_coding
ENSG00000160224.17	20.52732194	-0.100431694	0.199775311	-0.502723253	0.615158859	0.78882036	AIRE	protein_coding
ENSG00000160226.16	264.7803324	0.064422104	0.078932958	0.81616229	0.414407321	0.634851604	CFAP410	protein_coding
ENSG00000160229.13	187.7046948	-0.183022388	0.127271647	-1.438045255	0.150421214	0.352679718	ZNF66	protein_coding
ENSG00000160233.8	656.3600222	0.13723251	0.107356161	1.278291888	0.201146527	0.419877477	LRRC3	protein_coding

ENSG00000160255.18	4840.912294	-0.132692977	0.127311436	-1.042270677	0.297286203	0.527177421	ITGB2	protein_coding
ENSG00000160256.13	829.1072449	-0.078893426	0.07475354	-1.055380468	0.291251352	0.521277402	FAM207A	protein_coding
ENSG00000160271.16	635.1740701	-0.058179607	0.065810824	-0.884043122	0.376672935	0.602491429	RALGDS	protein_coding
ENSG00000160282.14	41.73984077	0.581545943	0.242941237	2.393772046	0.016676109	0.086187119	FTCD	protein_coding
ENSG00000160284.15	643.718119	-0.084725792	0.129987057	-0.65180176	0.514529066	0.716346106	SPATC1L	protein_coding
ENSG00000160285.15	2767.973458	0.140406458	0.067960762	2.065992991	0.038829141	0.15115689	LSS	protein_coding
ENSG00000160293.17	4676.507228	0.186800504	0.071876466	2.598910535	0.009352014	0.057629264	VAV2	protein_coding
ENSG00000160294.11	3080.052595	0.05084509	0.059198564	0.858890604	0.390400874	0.614794605	MCM3AP	protein_coding
ENSG00000160298.18	406.7535799	0.043248944	0.082164424	0.526370679	0.598630684	0.777647604	C21orf58	protein_coding
ENSG00000160299.17	2804.63791	0.013434789	0.05661759	0.237290024	0.812431801	0.907350288	PCNT	protein_coding
ENSG00000160305.18	2591.730013	-0.006282702	0.04855821	-0.129384952	0.897053054	0.951093183	DIP2A	protein_coding
ENSG00000160307.10	204.0297049	-0.262330569	0.140944129	-1.861238001	0.062710574	0.205422739	S100B	protein_coding
ENSG00000160310.18	3009.046832	0.021721793	0.04934447	0.440207246	0.659787012	0.81762959	PRMT2	protein_coding
ENSG00000160318.6	70.45089411	-0.416161625	0.111402564	-3.735655703	0.000187227	0.003242757	CLDND2	protein_coding
ENSG00000160321.15	72.97718622	0.223555281	0.156574854	1.427785343	0.153353653	0.356924217	ZNF208	protein_coding
ENSG00000160323.19	217.8630912	-0.148781346	0.097066824	-1.532772374	0.12533195	0.315625888	ADAMTS13	protein_coding
ENSG00000160325.15	2188.182886	-0.053094995	0.081954727	-0.647857636	0.517077033	0.718355784	CACFD1	protein_coding
ENSG00000160326.14	619.249378	0.146247773	0.10814438	1.352338164	0.176267161	0.38834277	SLC2A6	protein_coding
ENSG00000160336.16	1488.204847	0.091621372	0.081518011	1.123940227	0.261038384	0.488179936	ZNF761	protein_coding
ENSG00000160339.16	1.665710754	0.163076865	0.353246315	0.461651992	0.644330904	0.807866016	FCN2	protein_coding
ENSG00000160345.13	169.2256908	-0.156957778	0.107921142	-1.454374688	0.145842454	0.346266569	C9orf116	protein_coding
ENSG00000160349.10	3.19401967	0.225154297	0.209727081	1.073558529	0.283020593	0.511920487	LCN1	protein_coding
ENSG00000160352.16	936.9106857	-0.048017923	0.098026369	-0.489846996	0.624242172	0.794924329	ZNF714	protein_coding
ENSG00000160360.13	716.0594869	0.51405159	0.116896689	4.397486303	1.09512E-05	0.000349611	GPSM1	protein_coding
ENSG00000160392.13	1174.068665	0.091551438	0.055026923	1.663757171	0.096160966	0.268684689	C19orf47	protein_coding
ENSG00000160396.9	13.91260252	-0.058503231	0.140223944	-0.41721285	0.676522727	0.828625299	HIPK4	protein_coding
ENSG00000160401.15	185.2482567	0.232123885	0.078187698	2.968803149	0.00298962	0.02559	CFAP157	protein_coding
ENSG00000160404.18	1422.96709	-0.114056502	0.06856823	-1.663401575	0.096232078	0.268848908	TOR2A	protein_coding
ENSG00000160408.15	27.62353933	-0.103026822	0.103688493	-0.993618657	0.320408557	0.550092337	ST6GALNAC6	protein_coding
ENSG00000160410.15	6204.658924	0.086667245	0.062823173	1.379542621	0.167727515	0.376859566	SHKBP1	protein_coding
ENSG00000160439.16	1368.208	0.114880207	0.067518474	1.701463325	0.088856019	0.256283724	RDH13	protein_coding
ENSG00000160445.11	4603.289206	0.030722447	0.053456277	0.574721035	0.565479979	0.753881231	ZER1	protein_coding
ENSG00000160446.19	3988.014942	-0.006613188	0.074858673	-0.088342307	0.929604615	0.96668738	ZDHHC12	protein_coding
ENSG00000160447.7	1363.523638	0.202113793	0.084660038	2.387357712	0.01696997	0.087127646	PKN3	protein_coding
ENSG00000160460.16	222.5852829	-0.348644063	0.162202097	-2.14944239	0.031599348	0.131972858	SPTBN4	protein_coding
ENSG00000160469.17	198.1185843	0.142265382	0.113200462	1.256756196	0.208841932	0.428772904	BRSK1	protein_coding
ENSG00000160471.13	111.2204744	1.046904741	0.184321293	5.679781897	1.34867E-08	1.5466E-06	COX6B2	protein_coding
ENSG00000160472.5	7.389308558	0.323698168	0.149081783	2.171279154	0.029910077	0.127358944	TMEM190	protein_coding
ENSG00000160505.16	18.68270084	-0.004892414	0.270669729	-0.018075217	0.985578849	0.993327193	NLRP4	protein_coding
ENSG00000160539.6	145.9399015	0.020206861	0.12891436	0.156746391	0.875444725	0.939845157	PLPP7	protein_coding
ENSG00000160551.12	6604.89889	0.146896958	0.052502259	2.797916903	0.005143334	0.037943301	TAOK1	protein_coding
ENSG00000160563.14	980.6495701	0.022857808	0.050046603	0.456730465	0.647864791	0.809984335	MED27	protein_coding

ENSG00000160570.14	2059.617015	0.153490493	0.060254335	2.547376759	0.010853618	0.063945728	DEDD2	protein_coding
ENSG00000160584.16	3364.136867	-0.051982017	0.051802544	-1.00346457	0.315636763	0.544754375	SIK3	protein_coding
ENSG00000160588.10	1006.537178	0.27551944	0.08547998	3.223204333	0.001267651	0.013748239	MPZL3	protein_coding
ENSG00000160593.19	660.2051181	-0.346268263	0.113136209	-3.060631651	0.002208706	0.020526508	JAML	protein_coding
ENSG00000160602.14	342.2528449	-0.049154363	0.062255538	-0.789558077	0.4297859	0.648054677	NEK8	protein_coding
ENSG00000160606.11	764.2083329	0.068413521	0.107071592	0.638951188	0.522854686	0.722356642	TLCD1	protein_coding
ENSG00000160613.14	2862.313731	-0.091258911	0.065922742	-1.384331233	0.166257055	0.374630729	PCSK7	protein_coding
ENSG00000160633.13	4216.973506	0.012068395	0.03514376	0.343400794	0.731296962	0.860406278	SAFB	protein_coding
ENSG00000160654.11	492.5646803	-0.646204245	0.124505678	-5.190158844	2.10115E-07	1.4768E-05	CD3G	protein_coding
ENSG00000160678.12	34.24736553	0.179842072	0.118022179	1.523798945	0.127558943	0.318986007	S100A1	protein_coding
ENSG00000160679.13	3637.368593	0.050700882	0.037612846	1.347967176	0.17766894	0.390058915	CHTOP	protein_coding
ENSG00000160683.5	26.64376438	-1.109979156	0.233622374	-4.751168026	2.02245E-06	9.1063E-05	CXCR5	protein_coding
ENSG00000160685.13	6603.317659	0.118854774	0.072181171	1.646617426	0.099636706	0.274847366	ZBTB7B	protein_coding
ENSG00000160688.19	2718.119879	0.115980151	0.053075068	2.185209655	0.028873478	0.124366862	FLAD1	protein_coding
ENSG00000160691.19	5875.915201	0.250880399	0.051990651	4.82549061	1.39659E-06	6.73967E-05	SHC1	protein_coding
ENSG00000160695.15	2725.736997	-0.016927905	0.041753448	-0.40542533	0.685164895	0.833791757	VPS11	protein_coding
ENSG00000160703.16	1453.986592	0.051349136	0.0732819	0.700706935	0.483485927	0.691530714	NLRX1	protein_coding
ENSG00000160710.17	26043.00684	0.157742681	0.049439279	3.190634723	0.001419606	0.014952195	ADAR	protein_coding
ENSG00000160712.13	1478.961593	-0.37311165	0.107148015	-3.482207775	0.000497298	0.006769991	IL6R	protein_coding
ENSG00000160714.10	4496.504209	0.10739807	0.04466099	2.404740022	0.016183975	0.084435295	UBE2Q1	protein_coding
ENSG00000160716.6	45.41468139	-0.320231728	0.181895312	-1.760527664	0.078318381	0.236923298	CHRNB2	protein_coding
ENSG00000160741.17	3134.23903	0.153846342	0.043519369	3.53512346	0.000407585	0.005832899	CRTC2	protein_coding
ENSG00000160746.13	1556.324192	-0.059053846	0.058839885	-1.003636322	0.31555394	0.544719302	ANO10	protein_coding
ENSG00000160752.15	2035.0361	0.21348174	0.061781772	3.455416255	0.000549444	0.007308643	FDPS	protein_coding
ENSG00000160753.16	3591.108217	0.179014872	0.051306056	3.48915679	0.000484547	0.006637881	RUSC1	protein_coding
ENSG00000160766.14	162.9864965	0.094640638	0.070348642	1.345308665	0.178525576	0.391109921	GBAP1	transcribed_unprocessed_pseudogene
ENSG00000160767.21	3606.056112	0.134199982	0.057092777	2.350559727	0.018745199	0.092863329	FAM189B	protein_coding
ENSG00000160781.17	179.217534	0.302610237	0.105745128	2.861694376	0.00421383	0.032870126	PAQR6	protein_coding
ENSG00000160783.19	1142.602116	-0.125685864	0.063251999	-1.987065493	0.04691514	0.170738353	PMF1	protein_coding
ENSG00000160785.13	2974.859167	0.110880863	0.047299464	2.344230838	0.019066367	0.09394562	SLC25A44	protein_coding
ENSG00000160789.22	27626.16538	0.237380103	0.062954797	3.770643628	0.000162827	0.002898495	LMNA	protein_coding
ENSG00000160791.13	528.1002806	-0.476165455	0.128460214	-3.706715423	0.000209965	0.003517735	CCR5	protein_coding
ENSG00000160796.18	10960.57121	0.170171702	0.078452304	2.169110331	0.030074309	0.127827901	NBEAL2	protein_coding
ENSG00000160799.11	1573.671259	-0.080611401	0.052205258	-1.544124189	0.12255825	0.31133968	CCDC12	protein_coding
ENSG00000160801.14	60.05873136	0.425379843	0.140001432	3.038396373	0.002378409	0.021642081	PTH1R	protein_coding
ENSG00000160803.8	4194.165296	0.115604303	0.058573434	1.973664429	0.048419912	0.174293286	UBQLN4	protein_coding
ENSG00000160808.11	28.60458057	0.361295606	0.15741843	2.295129009	0.021725732	0.102738328	MYL3	protein_coding
ENSG00000160813.7	693.7500193	-0.085590649	0.07885265	-1.085450518	0.277722168	0.506669363	PPP1R35	protein_coding
ENSG00000160818.17	3266.998884	-0.00276723	0.059963617	-0.046148486	0.963191901	0.983047049	GPATCH4	protein_coding
ENSG00000160838.14	13.06164181	0.225862673	0.16352084	1.381247021	0.167203022	0.376004029	LRRC71	protein_coding
ENSG00000160856.21	315.0351743	-1.088755015	0.187318243	-5.812327716	6.16101E-09	8.06241E-07	FCRL3	protein_coding
ENSG00000160862.13	3169.198064	-0.228274489	0.199351892	-1.14508313	0.252174723	0.478907779	AZGP1	protein_coding

ENSG00000160867.15	5321.141657	-0.434337902	0.126140743	-3.443280041	0.000574704	0.007565854	FGFR4	protein_coding
ENSG00000160868.15	192.4279562	-1.15174841	0.248454125	-4.635658225	3.55803E-06	0.000142508	CYP3A4	protein_coding
ENSG00000160870.15	10.31352198	0.050859124	0.173664837	0.292857926	0.769630749	0.883358818	CYP3A7	protein_coding
ENSG00000160877.6	6387.082823	0.125126215	0.067400947	1.856445944	0.063390021	0.206766399	NACC1	protein_coding
ENSG00000160882.13	1.623432033	0.705451659	0.291338615	2.421414883	0.015460221	0.081707876	CYP11B1	protein_coding
ENSG00000160883.11	237.8075171	0.077074097	0.125352712	0.614857829	0.538648614	0.734485596	HK3	protein_coding
ENSG00000160886.13	98.90923146	0.616758908	0.281085727	2.194202158	0.02822088	0.122293284	LY6K	protein_coding
ENSG00000160888.7	2602.383056	0.106084344	0.078455724	1.352155572	0.176325553	0.388420716	IER2	protein_coding
ENSG00000160908.15	1649.853698	0.039712953	0.059299258	0.669704045	0.503046473	0.70768518	ZNF394	protein_coding
ENSG00000160917.15	1246.366496	-0.015479285	0.069076634	-0.224088584	0.822688375	0.912575695	CPSF4	protein_coding
ENSG00000160932.11	28302.74721	0.114785044	0.120903501	0.949393879	0.342420322	0.572283953	LY6E	protein_coding
ENSG00000160948.14	5363.517175	-0.071856524	0.057729664	-1.244707135	0.213239422	0.433917082	VPS28	protein_coding
ENSG00000160949.17	2854.774156	-0.06510257	0.089535654	-0.727113354	0.467156519	0.678666477	TONSL	protein_coding
ENSG00000160951.4	195.1806123	-0.081349147	0.168909297	-0.481614385	0.630079906	0.799004541	PTGER1	protein_coding
ENSG00000160953.16	1621.241435	0.045619298	0.044564705	1.02366431	0.305993858	0.535556846	PWWP3A	protein_coding
ENSG00000160957.14	2091.27996	-0.095398199	0.103139946	-0.924939396	0.354997432	0.583053384	RECQL4	protein_coding
ENSG00000160959.8	1472.022388	0.03295526	0.051840392	0.63570622	0.524967921	0.724101133	LRRC14	protein_coding
ENSG00000160961.12	584.9842652	-0.057537101	0.058652675	-0.980980003	0.326602603	0.556354749	ZNF333	protein_coding
ENSG00000160963.14	33.43060994	0.085173845	0.200842777	0.424082192	0.671505872	0.82507786	COL26A1	protein_coding
ENSG00000160972.9	2260.279333	-0.112252239	0.083796961	-1.339574107	0.180383846	0.393397898	PPP1R16A	protein_coding
ENSG00000160973.8	70.79685128	-0.192330547	0.207715743	-0.92593149	0.354481585	0.582651627	FOXH1	protein_coding
ENSG00000160991.16	2033.724602	0.148788892	0.087364192	1.703087837	0.088551632	0.255736925	ORAI2	protein_coding
ENSG00000160993.4	523.3566337	-0.071928002	0.061320885	-1.172977227	0.240804938	0.466317766	ALKBH4	protein_coding
ENSG00000160994.4	2.675853631	-0.420296299	0.326084525	-1.28891826	0.197426505	0.414774603	CCDC105	protein_coding
ENSG00000160999.10	522.5507051	-0.051551704	0.084229766	-0.61203665	0.540513509	0.736038161	SH2B2	protein_coding
ENSG00000161010.15	719.4195787	0.011788755	0.066258175	0.177921523	0.858784608	0.931240938	MRNIP	protein_coding
ENSG00000161011.20	15309.16787	0.070023431	0.077902368	0.898861389	0.368726496	0.595552058	SQSTM1	protein_coding
ENSG00000161013.17	13196.32688	-0.032570239	0.072449556	-0.449557462	0.653029566	0.813407729	MGAT4B	protein_coding
ENSG00000161016.18	80247.00992	-0.191447426	0.07275637	-2.631349333	0.008504657	0.053914377	RPL8	protein_coding
ENSG00000161021.13	4416.260489	0.10165634	0.044297486	2.294855768	0.021741391	0.102768696	MAML1	protein_coding
ENSG00000161031.13	16.72780956	-0.580830478	0.16540436	-3.511579014	0.000445453	0.006239137	PGLYRP2	protein_coding
ENSG00000161036.13	1263.944784	-0.021384215	0.0698477	-0.306154896	0.759486713	0.876835408	LRWD1	protein_coding
ENSG00000161040.16	91.72658155	-0.236808638	0.101531676	-2.33236215	0.019681648	0.0960111	FBXL13	protein_coding
ENSG00000161048.11	1976.239544	-0.170515755	0.064186136	-2.65658235	0.007893717	0.051272097	NAPEPLD	protein_coding
ENSG00000161055.4	140.8525558	2.298250259	0.25463584	9.025635422	1.78656E-19	4.86579E-16	SCGB3A1	protein_coding
ENSG00000161057.13	5328.964969	-0.087523996	0.054592905	-1.603211918	0.108887875	0.289411788	PSMC2	protein_coding
ENSG00000161082.13	50.20079687	-0.131173042	0.15290081	-0.857896319	0.390949717	0.615332095	CELF5	protein_coding
ENSG00000161091.13	4019.815628	0.177308079	0.066838832	2.652770471	0.007983414	0.051692901	MFSD12	protein_coding
ENSG00000161103.11	2.264066295	-0.257776279	0.208091162	-1.238766109	0.215432128	0.436584934	AC008132.1	transcribed_processed_pseudogene
ENSG00000161132.6	14.49210533	0.145586239	0.133686828	1.089009603	0.276149651	0.504878743	AC007663.1	unprocessed_pseudogene
ENSG00000161133.16	13.62003842	0.586997432	0.183608214	3.197010747	0.001388598	0.01475876	USP41	protein_coding
ENSG00000161149.12	7.957583037	-0.30374531	0.147979903	-2.052611896	0.040110233	0.154207916	TUBA3FP	lncRNA

ENSG00000161179.14	2366.242564	-0.067230708	0.080456671	-0.835613845	0.403372177	0.625073847	YDJC	protein_coding
ENSG00000161180.11	21.87740128	-0.095940593	0.124253087	-0.772138503	0.4400324	0.65655317	CCDC116	protein_coding
ENSG00000161202.20	9495.297869	0.10402595	0.048933793	2.12585094	0.033515673	0.136931603	DVL3	protein_coding
ENSG00000161203.13	16325.13726	0.08584899	0.051466253	1.668063747	0.095303073	0.267264432	AP2M1	protein_coding
ENSG00000161204.11	2786.880773	0.041260383	0.046564781	0.886085631	0.375571386	0.60139172	ABCF3	protein_coding
ENSG00000161217.12	3717.939998	-0.04425911	0.047204264	-0.937608303	0.348445743	0.577610321	PCYT1A	protein_coding
ENSG00000161243.9	196.8520433	0.508688397	0.158374325	3.211937272	0.001318432	0.014126701	FBXO27	protein_coding
ENSG00000161249.21	1629.536133	0.274602709	0.193606185	1.41835711	0.156086531	0.360799	DMKN	protein_coding
ENSG00000161265.15	129.5846126	-0.17420105	0.06808207	-2.558692029	0.010506677	0.062402819	U2AF1L4	protein_coding
ENSG00000161267.12	2593.792349	-0.078430792	0.094618068	-0.828919825	0.407149776	0.628270744	BDH1	protein_coding
ENSG00000161270.20	87.70658221	-0.61611475	0.190292883	-3.23771831	0.001204897	0.013240049	NPHS1	protein_coding
ENSG00000161277.11	425.0809217	-0.157030377	0.073357573	-2.140615747	0.032305038	0.133807386	THAP8	protein_coding
ENSG00000161281.11	370.1703503	0.07073791	0.116373742	0.607851126	0.543286217	0.737992004	COX7A1	protein_coding
ENSG00000161298.18	149.4531883	-0.172774577	0.116803613	-1.479188634	0.139089907	0.336157304	ZNF382	protein_coding
ENSG00000161328.11	344.2762291	0.04701793	0.121478448	0.387047505	0.69872104	0.841528018	LRRC56	protein_coding
ENSG00000161381.14	1119.478853	0.203107162	0.084892498	2.392521905	0.016733029	0.086367781	PLXDC1	protein_coding
ENSG00000161395.14	3067.63627	-0.466794522	0.131928367	-3.538242263	0.0004028	0.005775857	PGAP3	protein_coding
ENSG00000161405.17	2848.704249	-1.215064527	0.148805844	-8.16543551	3.20278E-16	4.81267E-13	IKZF3	protein_coding
ENSG00000161509.14	97.73228066	0.525199102	0.132808098	3.954571369	7.6672E-05	0.001614855	GRIN2C	protein_coding
ENSG00000161513.12	1074.437651	-0.12633272	0.097805025	-1.291679236	0.196468251	0.413698815	FDXR	protein_coding
ENSG00000161526.15	2794.991498	0.032433304	0.042613657	0.761101166	0.446596644	0.662228896	SAP30BP	protein_coding
ENSG00000161533.12	5872.953073	0.043457463	0.069231609	0.627711303	0.53019308	0.728104873	ACOX1	protein_coding
ENSG00000161542.17	2348.72661	0.023696596	0.044293974	0.534984635	0.592660482	0.772874247	PRPSAP1	protein_coding
ENSG00000161544.10	1845.263844	0.186260063	0.096101041	1.938169046	0.052602602	0.183636146	CYGB	protein_coding
ENSG00000161547.17	6850.774653	-0.105689818	0.053029336	-1.993044356	0.04625659	0.169203679	SRSF2	protein_coding
ENSG00000161551.14	323.7280902	-0.068508483	0.119763891	-0.572029535	0.567301965	0.754941603	ZNF577	protein_coding
ENSG00000161558.11	566.594923	-0.176888766	0.063550606	-2.783431636	0.00537872	0.0391169	TMEM143	protein_coding
ENSG00000161594.7	9.119656519	-0.546552169	0.184834135	-2.956987187	0.00310661	0.026384088	KLHL10	protein_coding
ENSG00000161609.10	16.43172221	-0.369765888	0.242127	-1.527156777	0.126722031	0.317676233	KASH5	protein_coding
ENSG00000161610.1	6.512957381	0.18267552	0.201411833	0.906975113	0.364419958	0.591681327	HCRT	protein_coding
ENSG00000161618.10	1974.705451	-0.158928175	0.073855705	-2.151874043	0.031407276	0.131548911	ALDH16A1	protein_coding
ENSG00000161634.12	2.842922756	-0.242262531	0.2949621	-0.821334437	0.411455797	0.632380282	DCD	protein_coding
ENSG00000161638.11	9686.789784	0.024556036	0.118822521	0.206661462	0.836274257	0.919627887	ITGA5	protein_coding
ENSG00000161640.15	31.26187357	-0.168309451	0.138654288	-1.213878442	0.224794146	0.447697404	SIGLEC11	protein_coding
ENSG00000161642.18	2717.797983	0.085888949	0.098112113	0.875416356	0.381347404	0.606694747	ZNF385A	protein_coding
ENSG00000161643.13	52.51812227	0.106824478	0.134692767	0.793097356	0.427721103	0.646193403	SIGLEC16	polymorphic_pseudogene
ENSG00000161647.19	293.5745632	0.381191175	0.088450412	4.309659674	1.63506E-05	0.000475324	MPP3	protein_coding
ENSG00000161649.13	18.51854858	0.356187305	0.218484654	1.63026235	0.103046061	0.280472674	CD300LG	protein_coding
ENSG00000161652.12	16.00634917	0.356403338	0.231639253	1.53861374	0.123898622	0.313356648	IZUMO2	protein_coding
ENSG00000161653.11	327.8967165	0.281572394	0.128271269	2.195132205	0.028154116	0.122116505	NAGS	protein_coding
ENSG00000161654.10	1071.869246	0.074973626	0.057018046	1.314910475	0.188539992	0.403855717	LSM12	protein_coding
ENSG00000161664.7	62.48733859	0.029690942	0.09451808	0.314129762	0.753422486	0.873327969	ASB16	protein_coding

ENSG00000161671.17	7194.838174	0.170139457	0.077222167	2.203246349	0.02757739	0.1205356	EMC10	protein_coding
ENSG00000161677.12	1018.775762	0.028987375	0.075715356	0.382846716	0.701833421	0.843248918	JOSD2	protein_coding
ENSG00000161681.16	76.01184403	0.613774749	0.134152913	4.57518763	4.75793E-06	0.000178584	SHANK1	protein_coding
ENSG00000161682.15	209.8226182	0.316497261	0.141017603	2.244381231	0.024807883	0.112515937	FAM171A2	protein_coding
ENSG00000161692.18	752.9763648	0.108417993	0.067394067	1.608717163	0.107678201	0.287570814	DBF4B	protein_coding
ENSG00000161714.12	7235.707028	0.336654104	0.109247533	3.081571678	0.002059109	0.019570292	PLCD3	protein_coding
ENSG00000161791.14	3049.989567	-0.048891972	0.081855311	-0.597297486	0.550308783	0.742906376	FMNL3	protein_coding
ENSG00000161798.7	2241.425122	0.307038071	0.255542798	1.201513304	0.229552149	0.45314582	AQP5	protein_coding
ENSG00000161800.13	3427.915423	-0.010505769	0.076010723	-0.138214305	0.89007105	0.94761003	RACGAP1	protein_coding
ENSG00000161807.3	1.516661654	0.075238493	0.424277269	0.177333312	0.859246586	0.931473916	OR7G1	protein_coding
ENSG00000161813.22	3852.732043	0.111316685	0.056536144	1.968947247	0.048959149	0.17556605	LARP4	protein_coding
ENSG00000161835.11	577.2283754	0.003817006	0.097844267	0.039011038	0.968881588	0.98561881	TAMALIN	protein_coding
ENSG00000161847.14	3623.744366	0.086398555	0.069970581	1.234784016	0.216910898	0.438196012	RAVER1	protein_coding
ENSG00000161849.3	1.388426957	0.405022095	0.272526444	1.486175395	0.137232734	0.333567093	KRT84	protein_coding
ENSG00000161850.3	0.754225375	-0.020078546	0.401256703	-0.050039153	0.960091188		KRT82	protein_coding
ENSG00000161860.8	39.47900949	-0.485362328	0.112786689	-4.303365358	1.68223E-05	0.000486118	SYCE2	protein_coding
ENSG00000161888.11	609.1260883	-0.047107786	0.086408117	-0.545177782	0.58563125	0.767963955	SPC24	protein_coding
ENSG00000161896.12	26.77183506	-0.004248498	0.177576671	-0.023924863	0.980912542	0.991630535	IP6K3	protein_coding
ENSG00000161904.12	3807.0672	0.016614194	0.048005233	0.346091318	0.729274088	0.859023569	LEMD2	protein_coding
ENSG00000161905.13	98.41561354	0.623842146	0.220811438	2.825225684	0.004724732	0.035778392	ALOX15	protein_coding
ENSG00000161911.11	25.83306273	-0.369660775	0.124414615	-2.97120057	0.00296638	0.025426029	TREML1	protein_coding
ENSG00000161912.18	182.5544871	0.069783212	0.115197362	0.605770923	0.544666881	0.739084158	ADCY10P1	transcribed_unprocessed_pseudogene
ENSG00000161914.10	335.3374677	0.116832332	0.059339491	1.968879928	0.048966881	0.17556605	ZNF653	protein_coding
ENSG00000161920.11	499.6191301	-0.109408755	0.062341643	-1.754986707	0.079261582	0.238815041	MED11	protein_coding
ENSG00000161921.17	12043.57548	0.055189024	0.085278489	0.647162308	0.517526903	0.718751628	CXCL16	protein_coding
ENSG00000161929.15	431.8450227	-0.482489208	0.127658	-3.779545421	0.000157115	0.002829172	SCIMP	protein_coding
ENSG00000161939.19	0.80405332	-0.051268947	0.21779132	-0.235403998	0.813895183		RNASEK-C17orf49	protein_coding
ENSG00000161940.11	817.4688597	0.094374597	0.091659261	1.02962424	0.303186432	0.532568331	BCL6B	protein_coding
ENSG00000161944.16	41.55564114	-0.365197852	0.162554398	-2.246619331	0.024664366	0.112051612	ASGR2	protein_coding
ENSG00000161955.16	716.1106603	-0.248896955	0.110483248	-2.252802653	0.024271595	0.110792759	TNFSF13	protein_coding
ENSG00000161956.13	525.4166095	0.001100643	0.049264982	0.022341284	0.982175717	0.992260457	SENP3	protein_coding
ENSG00000161958.11	13.48223145	0.333887966	0.141852492	2.353768777	0.018584167	0.092408006	FGF11	protein_coding
ENSG00000161960.16	197.2899228	0.083662209	0.082209604	1.017669531	0.308835032	0.538212042	EIF4A1	protein_coding
ENSG00000161970.15	4349.083861	-0.214020813	0.085079339	-2.515543918	0.011884887	0.068145752	RPL26	protein_coding
ENSG00000161973.11	3.649471704	0.000377484	0.177002707	0.002132647	0.998298395	0.999100849	CCDC42	protein_coding
ENSG00000161980.6	837.6564155	-0.174235055	0.069916656	-2.492039298	0.012701198	0.071379949	POLR3K	protein_coding
ENSG00000161981.11	1050.228944	-0.163647595	0.068215253	-2.398988317	0.016440439	0.085441267	SNRNP25	protein_coding
ENSG00000161992.6	2.479487013	0.398412072	0.255078998	1.561916409	0.118307682	0.304623977	PRR35	protein_coding
ENSG00000161996.19	2310.619822	-0.023806067	0.081068323	-0.29365437	0.769022025	0.883024764	WDR90	protein_coding
ENSG00000161999.13	1567.390577	-0.046250717	0.066054254	-0.700192849	0.483806877	0.691838828	JMJD8	protein_coding
ENSG00000162004.18	119.8137547	0.084290898	0.126397629	0.666870882	0.504854611	0.708900714	CCDC78	protein_coding
ENSG00000162006.9	56.97518151	1.897651749	0.244278083	7.76840772	7.94789E-15	7.21552E-12	MSLNL	protein_coding

ENSG00000162009.8	70.39326425	1.154548533	0.236456163	4.882717037	1.04634E-06	5.38964E-05	SSTR5	protein_coding
ENSG00000162032.16	16.69416258	0.086160257	0.110543712	0.779422507	0.43573087	0.653286884	SPSB3	protein_coding
ENSG00000162039.17	12.03363966	-0.058596029	0.173565067	-0.337602663	0.735662635	0.862918984	MEIOB	protein_coding
ENSG00000162040.7	15.32138492	1.571785868	0.257565839	6.102462489	1.04447E-09	1.86842E-07	HS3ST6	protein_coding
ENSG00000162062.15	347.6716752	-0.007013083	0.094655137	-0.074090882	0.94093807	0.972044828	TEDC2	protein_coding
ENSG00000162063.13	1465.997612	0.043810286	0.083551781	0.524348927	0.600035872	0.778534954	CCNF	protein_coding
ENSG00000162065.14	1165.402426	0.00847983	0.055991719	0.151447939	0.879622376	0.942030963	TBC1D24	protein_coding
ENSG00000162066.16	463.4905203	-0.075909694	0.067247504	-1.128810574	0.258977751	0.486174641	AMDHD2	protein_coding
ENSG00000162068.2	19.03134751	-0.081149028	0.17433837	-0.46546855	0.641595954	0.806101571	NTN3	protein_coding
ENSG00000162069.16	1413.545658	0.087166758	0.099620184	0.874990937	0.381578837	0.60688677	BICDL2	protein_coding
ENSG00000162073.14	1234.265637	-0.075707147	0.097324303	-0.777885318	0.436636624	0.654039398	PAQR4	protein_coding
ENSG00000162076.13	953.345166	0.04987169	0.075099623	0.664073773	0.506643095	0.710657166	FLYWCH2	protein_coding
ENSG00000162078.11	1845.454417	-0.207920219	0.172487291	-1.205423419	0.228039885	0.451489963	ZG16B	protein_coding
ENSG00000162086.16	712.4087967	-0.021155978	0.084439059	-0.250547303	0.802164128	0.90157604	ZNF75A	protein_coding
ENSG00000162104.10	2903.11023	0.078551846	0.078702585	0.998084693	0.318238292	0.547677889	ADCY9	protein_coding
ENSG00000162105.19	2262.105258	0.142419032	0.114478478	1.244068181	0.213474471	0.434192626	SHANK2	protein_coding
ENSG00000162129.14	2343.257647	0.036317735	0.063361112	0.573186508	0.566518419	0.754587421	CLPB	protein_coding
ENSG00000162139.10	867.0768315	0.016410658	0.060696244	0.270373528	0.786872904	0.89229417	NEU3	protein_coding
ENSG00000162144.10	1634.214815	-0.03164796	0.057672949	-0.548748771	0.583177871	0.766464835	CYB561A3	protein_coding
ENSG00000162148.11	60.39744521	0.154506385	0.08976151	1.721298873	0.085196604	0.249788899	PPP1R32	protein_coding
ENSG00000162174.12	528.7176882	-0.112836007	0.110604679	-1.020173901	0.307645993	0.537067921	ASRGL1	protein_coding
ENSG00000162188.6	11.53712257	0.204236754	0.114724955	1.780229547	0.075038402	0.230345632	GNG3	protein_coding
ENSG00000162191.14	3634.34958	-0.022458454	0.053275856	-0.421550322	0.673353273	0.82634303	UBXN1	protein_coding
ENSG00000162194.12	89.28795865	0.059431785	0.071384595	0.83255756	0.405094303	0.626607782	LBHD1	protein_coding
ENSG00000162222.14	1082.859397	-0.026454545	0.04088958	-0.646975218	0.517647983	0.718805243	TTC9C	protein_coding
ENSG00000162227.8	613.4057039	-0.00026641	0.048004144	-0.005549728	0.995571981	0.997953677	TAF6L	protein_coding
ENSG00000162231.14	4210.576416	-0.014995895	0.034455923	-0.435219658	0.663402995	0.819930578	NXF1	protein_coding
ENSG00000162236.13	2645.472658	0.115859555	0.045184374	2.564150943	0.010342855	0.061817393	STX5	protein_coding
ENSG00000162241.12	522.1394042	-0.083818569	0.080992107	-1.034897984	0.300716556	0.53036771	SLC25A45	protein_coding
ENSG00000162244.12	43342.87125	-0.168765293	0.075451824	-2.236729137	0.025304048	0.113983308	RPL29	protein_coding
ENSG00000162267.12	89.4848566	0.217495687	0.160816714	1.352444541	0.176233149	0.388334948	ITIH3	protein_coding
ENSG00000162298.19	7905.643802	-0.018705683	0.048453576	-0.38605371	0.699456891	0.841821528	SYVN1	protein_coding
ENSG00000162300.13	250.0674924	0.090263276	0.056342976	1.602032444	0.109148435	0.289809978	ZFPL1	protein_coding
ENSG00000162302.13	4667.028212	0.091215252	0.065959657	1.382894576	0.166697194	0.375253828	RPS6KA4	protein_coding
ENSG00000162337.12	13090.39764	0.268227701	0.075503057	3.552540953	0.00038153	0.005553078	LRP5	protein_coding
ENSG00000162341.18	719.4365506	-0.009517197	0.057085747	-0.166717575	0.867592277	0.935822781	TPCN2	protein_coding
ENSG00000162344.4	89.19676351	0.338237402	0.29397108	1.150580531	0.249904846	0.476653542	FGF19	protein_coding
ENSG00000162365.12	4.296248611	0.179921695	0.214423263	0.839095968	0.401415458	0.623695175	CYP4A22	protein_coding
ENSG00000162366.8	7642.730408	-0.191962661	0.168499489	-1.139247734	0.254599841	0.481412775	PDZK1IP1	protein_coding
ENSG00000162367.11	129.7424219	0.079975369	0.108221207	0.738999048	0.459907575	0.673064256	TAL1	protein_coding
ENSG00000162368.13	15797.20889	-0.11409169	0.064704773	-1.763265428	0.077855733	0.235933191	CMPK1	protein_coding
ENSG00000162373.13	96.11686671	-0.325219397	0.158583576	-2.05077604	0.040288762	0.154643123	BEND5	protein_coding

ENSG00000162374.18	48.58201578	-0.383647318	0.154312487	-2.486171564	0.01291257	0.072213944	ELAVL4	protein_coding
ENSG00000162377.6	1586.188258	-0.015691753	0.053152568	-0.29522098	0.767825076	0.882254801	COA7	protein_coding
ENSG00000162378.13	2785.918904	-0.034665958	0.049670342	-0.697920665	0.48522681	0.693065283	ZYG11B	protein_coding
ENSG00000162383.13	195.7873463	0.249733282	0.171005842	1.460378663	0.144186034	0.343665753	SLC1A7	protein_coding
ENSG00000162384.14	1547.091048	-0.137203864	0.049431937	-2.775611748	0.005509796	0.039817643	CZIB	protein_coding
ENSG00000162385.11	1625.126982	-0.201507933	0.057384901	-3.511514843	0.000445561	0.006239137	MAGOH	protein_coding
ENSG00000162390.18	1468.21683	-0.183799211	0.087377866	-2.103498516	0.035422215	0.141852173	ACOT11	protein_coding
ENSG00000162391.12	78.49165117	-0.32144627	0.128850571	-2.494721349	0.012605608	0.070943377	FAM151A	protein_coding
ENSG00000162396.6	491.8757418	0.044329139	0.056298148	0.787399589	0.431047987	0.649104228	PARS2	protein_coding
ENSG00000162398.11	20.27918498	0.379996517	0.148223054	2.563680261	0.01035689	0.061853564	LEXM	protein_coding
ENSG00000162399.9	5.373659542	-0.00299227	0.238756584	-0.012532724	0.990000594	0.995414303	BSND	protein_coding
ENSG00000162402.14	5338.544536	0.014449268	0.048693197	0.296740992	0.766664259	0.88157185	USP24	protein_coding
ENSG00000162407.9	2715.555742	-0.185290405	0.073740839	-2.512724399	0.011980289	0.068434808	PLPP3	protein_coding
ENSG00000162408.11	2673.329024	-0.096830693	0.051799535	-1.869335152	0.0615762	0.203138233	NOL9	protein_coding
ENSG00000162409.11	565.671875	-0.003873268	0.212846137	-0.018197504	0.985481294	0.993310157	PRKAA2	protein_coding
ENSG00000162413.16	3201.13323	0.12093177	0.069404248	1.742426056	0.08143392	0.242814474	KLHL21	protein_coding
ENSG00000162415.7	927.4319706	-0.229247668	0.116536429	-1.967176014	0.049162922	0.176051661	ZSWIM5	protein_coding
ENSG00000162419.13	1181.662389	0.010752922	0.036822588	0.292019718	0.770271548	0.883760913	GMEB1	protein_coding
ENSG00000162426.16	119.2825986	0.190425776	0.115174979	1.653360627	0.098257491	0.27260245	SLC45A1	protein_coding
ENSG00000162430.17	10206.13491	0.019050253	0.059204095	0.321772554	0.747625008	0.870154515	SELENON	protein_coding
ENSG00000162433.15	2454.082441	0.281274365	0.134369992	2.093282593	0.036323936	0.144358244	AK4	protein_coding
ENSG00000162434.13	13118.91199	-0.112132132	0.052236095	-2.14664078	0.031821891	0.132571944	JAK1	protein_coding
ENSG00000162437.14	2556.867518	-0.356105524	0.072775052	-4.89323625	9.91912E-07	5.15191E-05	RAVER2	protein_coding
ENSG00000162438.12	16.21212866	-0.62042303	0.184705525	-3.358984677	0.000782294	0.009529926	CTRC	protein_coding
ENSG00000162441.12	3036.037421	-0.120002785	0.046129731	-2.601419589	0.009283883	0.057368655	LZIC	protein_coding
ENSG00000162444.12	122.8764758	0.311615497	0.129977934	2.397449212	0.01650967	0.085617264	RBP7	protein_coding
ENSG00000162456.10	1.471160766	-0.363433885	0.312117751	-1.164412739	0.244256787	0.470496947	KNCN	protein_coding
ENSG00000162458.13	7320.974287	0.00262864	0.068683394	0.038271839	0.969470944	0.985827118	FBLIM1	protein_coding
ENSG00000162460.7	199.4754438	-0.315651494	0.201124156	-1.569436011	0.116546379	0.301767174	TMEM82	protein_coding
ENSG00000162461.8	112.7897072	-0.412959013	0.123833866	-3.334782541	0.000853661	0.010163623	SLC25A34	protein_coding
ENSG00000162482.5	3296.816919	-0.076518974	0.154961284	-0.49379414	0.621451572	0.792978102	AKR7A3	protein_coding
ENSG00000162490.7	69.57330966	0.666091732	0.143103787	4.654605919	3.24601E-06	0.000131705	DRAXIN	protein_coding
ENSG00000162493.16	2203.450633	0.568802618	0.109443318	5.197234786	2.02275E-07	1.43715E-05	PDPN	protein_coding
ENSG00000162494.6	6.455879498	0.617987819	0.194141253	3.18318652	0.001456637	0.015222036	LRRC38	protein_coding
ENSG00000162496.9	4580.751334	0.018250436	0.088096704	0.207163668	0.83588204	0.91948702	DHRS3	protein_coding
ENSG00000162510.6	10.27405879	0.100429187	0.112195894	0.895123545	0.370721049	0.597289845	MATN1	protein_coding
ENSG00000162511.8	8551.262164	-0.151437152	0.120281459	-1.25902324	0.208021938	0.427982261	LAPTM5	protein_coding
ENSG00000162512.16	7173.325736	-0.080756186	0.092163826	-0.87622432	0.380908098	0.606495433	SDC3	protein_coding
ENSG00000162517.13	3160.278216	-0.051060504	0.045284027	-1.127561036	0.25950535	0.486720523	PEF1	protein_coding
ENSG00000162520.15	331.2488719	-0.01964031	0.178057319	-0.110303302	0.912168838	0.958582856	SYNC	protein_coding
ENSG00000162521.19	6756.978669	-0.020720904	0.046436475	-0.44622044	0.655438019	0.814823911	RBBP4	protein_coding
ENSG00000162522.11	16361.63105	0.13608332	0.082183827	1.655840634	0.097754096	0.271776844	KIAA1522	protein_coding

ENSG00000162526.7	31.80380026	-0.171343866	0.103310399	-1.658534551	0.09720962	0.27079867	TSSK3	protein_coding
ENSG00000162542.13	1885.40477	-0.190796173	0.070765073	-2.696191297	0.007013737	0.047093627	TMCO4	protein_coding
ENSG00000162543.6	841.5367074	0.021402806	0.153889236	0.139079291	0.889387492	0.947183591	UBXN10	protein_coding
ENSG00000162545.6	9078.743845	0.10277749	0.113524697	0.905331554	0.365289765	0.59250473	CAMK2N1	protein_coding
ENSG00000162551.14	680.234427	0.12222315	0.129048018	0.947113732	0.343580828	0.573191338	ALPL	protein_coding
ENSG00000162552.15	425.68937	0.33872696	0.15878925	2.13318572	0.032909494	0.135338102	WNT4	protein_coding
ENSG00000162571.14	44.58247742	0.307115886	0.130733288	2.34917894	0.018814862	0.09307956	TTLL10	protein_coding
ENSG00000162572.21	132.5571005	0.312422	0.113934753	2.742113277	0.006104529	0.042823091	SCNN1D	protein_coding
ENSG00000162576.17	7222.620515	0.672089375	0.126667859	5.305918814	1.12107E-07	8.8182E-06	MXRA8	protein_coding
ENSG00000162585.17	2602.038525	-0.047658397	0.071511884	-0.666440236	0.505129752	0.70915524	FAAP20	protein_coding
ENSG00000162591.16	1910.078832	0.670289967	0.112998892	5.931827758	2.99581E-09	4.53293E-07	MEGF6	protein_coding
ENSG00000162592.10	9.253153426	-0.107228644	0.216300589	-0.495739028	0.620078547	0.792015286	CCDC27	protein_coding
ENSG00000162594.16	38.94207962	-0.431415618	0.122148663	-3.531889817	0.000412601	0.005887336	IL23R	protein_coding
ENSG00000162595.7	137.8612688	0.138999825	0.159396446	0.87203842	0.383187418	0.608134537	DIRAS3	protein_coding
ENSG00000162598.13	4.75405686	0.124016873	0.289095254	0.428982736	0.667935789	0.822713321	C1orf87	protein_coding
ENSG00000162599.17	5928.834075	-0.190051988	0.090502552	-2.099962758	0.035732117	0.142656755	NFIA	protein_coding
ENSG00000162600.12	723.4739018	-0.183241181	0.05232288	-3.502123344	0.000461566	0.006419935	OMA1	protein_coding
ENSG00000162601.11	2343.730331	-0.067320657	0.060401844	-1.114546376	0.265044861	0.492868773	MYSM1	protein_coding
ENSG00000162604.12	1348.967898	-0.063599028	0.046881118	-1.356602198	0.174907643	0.386273833	TM2D1	protein_coding
ENSG00000162607.13	3171.482278	0.010487279	0.053643301	0.195500258	0.845001336	0.923981276	USP1	protein_coding
ENSG00000162613.17	6433.78018	0.046925397	0.040565383	1.156784255	0.247360516	0.473958106	FUBP1	protein_coding
ENSG00000162614.19	2565.708749	-0.328401025	0.16531423	-1.986526053	0.046974943	0.170870375	NEXN	protein_coding
ENSG00000162616.9	1244.151388	0.06745924	0.090311465	0.746962083	0.455086449	0.669162579	DNAJB4	protein_coding
ENSG00000162618.15	1459.107402	-0.065935971	0.089217676	-0.739046046	0.459879037	0.673064256	ADGRL4	protein_coding
ENSG00000162620.16	58.61506531	-0.009276438	0.118048666	-0.078581471	0.937365526	0.970291207	LRRIQ3	protein_coding
ENSG00000162621.7	2.357791815	0.414918483	0.390892457	1.061464543	0.288478838	0.51832542	LRRC53	protein_coding
ENSG00000162623.16	1318.178647	-0.102945051	0.05136363	-2.004240185	0.045044338	0.166414049	TYW3	protein_coding
ENSG00000162624.14	4.051931509	-0.055021975	0.331307459	-0.166075267	0.868097718	0.936096364	LHX8	protein_coding
ENSG00000162627.17	1714.795236	-0.124687494	0.058316916	-2.138101644	0.032508495	0.134388402	SNX7	protein_coding
ENSG00000162630.6	79.71256211	-0.113775058	0.13548682	-0.839750005	0.40104857	0.623414942	B3GALT2	protein_coding
ENSG00000162631.19	35.45474038	0.338814236	0.212711425	1.592835158	0.111197176	0.293274576	NTNG1	protein_coding
ENSG00000162636.16	3615.16394	0.082477279	0.071634326	1.151365327	0.249581972	0.476308732	FAM102B	protein_coding
ENSG00000162639.16	735.1858336	-0.092945726	0.102005718	-0.911181528	0.362199735	0.589731649	HENMT1	protein_coding
ENSG00000162641.20	17.83906262	-0.261519865	0.162059994	-1.613722541	0.106587622	0.285997785	AKNAD1	protein_coding
ENSG00000162642.14	775.1755557	-0.022807398	0.039321383	-0.580025334	0.561897534	0.750933347	C1orf52	protein_coding
ENSG00000162643.13	25.95618929	0.022138707	0.12362465	0.179080041	0.857874854	0.930682334	DNAI3	protein_coding
ENSG00000162645.13	6156.359352	0.044091496	0.098855865	0.446018007	0.655584239	0.814855385	GBP2	protein_coding
ENSG00000162650.16	392.1844977	-0.005773961	0.059866579	-0.096447153	0.923165444	0.963771365	ATXN7L2	protein_coding
ENSG00000162654.9	7490.390468	-0.286832645	0.15446766	-1.856910657	0.063323866	0.206654992	GBP4	protein_coding
ENSG00000162664.17	2422.935697	-0.068729511	0.045364067	-1.515065025	0.129755938	0.322333514	ZNF326	protein_coding
ENSG00000162669.16	28.17800093	-0.183655593	0.171631201	-1.070059475	0.284592538	0.513782793	HFM1	protein_coding
ENSG00000162670.11	30.79959093	0.477699864	0.22102968	2.161247598	0.030676218	0.129501599	BRINP3	protein_coding

ENSG00000162676.12	303.4720137	-0.079413132	0.117202649	-0.677571137	0.498043652	0.70391963	GFI1	protein_coding
ENSG00000162685.6	1.090787439	-0.261059	0.243234207	-1.073282423	0.283144419	0.512031548	LSP1P3	processed_pseudogene
ENSG00000162687.19	97.4934307	0.045375946	0.140206399	0.323636768	0.746213048	0.869341165	KCNT2	protein_coding
ENSG00000162688.17	2294.467149	-0.024818153	0.066793022	-0.371568055	0.710214478	0.84845083	AGL	protein_coding
ENSG00000162692.12	1941.59417	-0.146123826	0.112116581	-1.303320391	0.192465402	0.408647539	VCAM1	protein_coding
ENSG00000162694.14	690.0816557	0.13002918	0.061119159	2.127470049	0.033381047	0.136548005	EXTL2	protein_coding
ENSG00000162695.12	4590.299752	0.04933797	0.052765703	0.935038623	0.349768395	0.578899972	SLC30A7	protein_coding
ENSG00000162699.5	0.553970169	-0.136619962	0.414964325	-0.32923303	0.741979559		DNAJA1P5	processed_pseudogene
ENSG00000162702.8	1708.791623	0.128668515	0.047598937	2.703180405	0.006867946	0.046386311	ZNF281	protein_coding
ENSG00000162704.16	11228.96208	0.062778517	0.045928166	1.366884911	0.171661382	0.38219039	ARPC5	protein_coding
ENSG00000162706.13	125.0700947	-0.210093994	0.225642053	-0.931094145	0.351804867	0.580599608	CADM3	protein_coding
ENSG00000162711.18	214.1176518	0.184040615	0.123050496	1.495651149	0.134744587	0.330004207	NLRP3	protein_coding
ENSG00000162714.12	2247.061619	0.123614592	0.058528332	2.112047072	0.034682412	0.139962966	ZNF496	protein_coding
ENSG00000162722.9	68.27058624	0.374461118	0.172530696	2.170402872	0.029976339	0.127566303	TRIM58	protein_coding
ENSG00000162723.10	28.204828	0.080591071	0.176571219	0.456422464	0.648086215	0.810124776	SLAMF9	protein_coding
ENSG00000162727.3	0.163256024	0.121812657	0.963809812	0.126386612	0.899425899		OR2M5	protein_coding
ENSG00000162728.5	12.88626589	-0.106529548	0.172302619	-0.618270046	0.536397344	0.732814592	KCNJ9	protein_coding
ENSG00000162729.14	3167.272525	0.064857849	0.084285367	0.769503069	0.441594724	0.658086558	IGSF8	protein_coding
ENSG00000162733.19	3950.759562	0.077134451	0.141293132	0.545917906	0.585122369	0.767684635	DDR2	protein_coding
ENSG00000162734.13	10013.12995	0.071875571	0.060397955	1.190033184	0.23403335	0.458613218	PEA15	protein_coding
ENSG00000162735.19	2569.165906	0.039371454	0.041603458	0.946350507	0.34396984	0.573660443	PEX19	protein_coding
ENSG00000162736.17	10027.87733	0.159455278	0.045779511	3.483114467	0.000495616	0.006755545	NCSTN	protein_coding
ENSG00000162738.6	1276.831546	0.166072832	0.162273566	1.023412724	0.306112746	0.535635847	VANGL2	protein_coding
ENSG00000162739.14	544.189759	-0.668590067	0.137144054	-4.875093374	1.08757E-06	5.56256E-05	SLAMF6	protein_coding
ENSG00000162745.10	3016.932424	0.528093716	0.129434376	4.080011305	4.50335E-05	0.001059464	OLFML2B	protein_coding
ENSG00000162746.14	61.66953681	0.177453767	0.105262021	1.685828991	0.091828742	0.261287698	FCRLB	protein_coding
ENSG00000162747.12	364.4311777	0.451408505	0.199891605	2.258266449	0.023929053	0.109636105	FCGR3B	protein_coding
ENSG00000162753.13	11.68692653	-0.255376603	0.230583247	-1.107524531	0.268067212	0.496011418	SLC9C2	protein_coding
ENSG00000162755.14	176.1478688	0.047905578	0.107309472	0.446424505	0.655290635	0.814710413	KLHDC9	protein_coding
ENSG00000162757.4	231.4505881	0.063287135	0.063062902	1.003555691	0.315592821	0.544738712	C1orf74	protein_coding
ENSG00000162761.14	6.798611831	-0.337967932	0.307622705	-1.098644303	0.271923245	0.500109701	LMX1A	protein_coding
ENSG00000162763.4	0.762416307	-0.640302756	0.395756777	-1.61791988	0.105679865		LRRC52	protein_coding
ENSG00000162769.13	2246.199175	-0.168247442	0.078879786	-2.132960166	0.032927994	0.135380998	FLVCR1	protein_coding
ENSG00000162771.8	0.090475713	-0.201065522	0.832531242	-0.241511083	0.809159027		FAM71A	protein_coding
ENSG00000162772.17	2962.680276	0.295698509	0.120577179	2.452358827	0.014192306	0.077096027	ATF3	protein_coding
ENSG00000162775.16	1278.595775	0.018796223	0.047159988	0.398562935	0.690215277	0.836508819	RBM15	protein_coding
ENSG00000162777.17	3291.272244	-0.07172704	0.067157918	-1.068035494	0.285504501	0.514782168	DENND2D	protein_coding
ENSG00000162779.22	126.2227345	-0.396888551	0.200812932	-1.976409323	0.048108436	0.173631053	AXDND1	protein_coding
ENSG00000162782.16	221.2554222	0.381796261	0.19128603	1.995944305	0.045939985	0.16846981	TDRD5	protein_coding
ENSG00000162783.11	2228.036551	0.294043199	0.072220599	4.071458863	4.67196E-05	0.001087585	IER5	protein_coding
ENSG00000162804.14	1120.294795	0.462089782	0.106515843	4.338225827	1.43638E-05	0.000431675	SNED1	protein_coding
ENSG00000162813.18	2321.502949	-0.054687585	0.067586473	-0.809149857	0.418428953	0.638570011	BPNT1	protein_coding

ENSG00000162814.11	87.01728998	0.079134253	0.126801711	0.624078751	0.532575868	0.730021966	SPATA17	protein_coding
ENSG00000162817.7	1560.480225	-0.028943934	0.114669029	-0.252412828	0.800721986	0.90058232	C1orf115	protein_coding
ENSG00000162819.12	4621.25948	0.126703594	0.049493715	2.559993618	0.010467409	0.062245942	BROX	protein_coding
ENSG00000162825.16	168.0950853	0.032978305	0.072930886	0.452185723	0.651135192	0.812069439	NBPF20	protein_coding
ENSG00000162836.12	1556.638088	0.187546521	0.066609093	2.815629405	0.004868179	0.036544466	ACP6	protein_coding
ENSG00000162840.4	21.55431802	-0.140047227	0.142725884	-0.981232152	0.326478272	0.556264821	MT2P1	processed_pseudogene
ENSG00000162843.17	13.58483951	-0.595513637	0.19019677	-3.131039704	0.001741886	0.017342054	WDR64	protein_coding
ENSG00000162849.16	2229.644295	0.623483398	0.120788775	5.161766046	2.44631E-07	1.66828E-05	KIF26B	protein_coding
ENSG00000162851.8	1196.200385	-0.035392382	0.060523659	-0.584769375	0.558702799	0.748803343	TFB2M	protein_coding
ENSG00000162852.14	1723.254502	-0.000813298	0.047949311	-0.016961613	0.98646724	0.993595118	CNST	protein_coding
ENSG00000162869.16	1414.870925	-0.030537166	0.040800541	-0.748450009	0.454188768	0.668474194	PPP1R21	protein_coding
ENSG00000162873.15	105.1892046	0.097526601	0.116168403	0.839527775	0.40117321	0.623530512	KLHDC8A	protein_coding
ENSG00000162877.13	33.48505033	-0.282017033	0.119832613	-2.353424709	0.018601375	0.092438373	PM20D1	protein_coding
ENSG00000162878.13	4974.183598	-0.081564886	0.122654542	-0.664996868	0.506052496	0.710171295	PKDCC	protein_coding
ENSG00000162881.6	206.3130167	-0.434178287	0.144809629	-2.998269452	0.002715175	0.023830651	OXER1	protein_coding
ENSG00000162882.15	904.4452259	-0.581400209	0.137013222	-4.243387606	2.20171E-05	0.000601948	HAAO	protein_coding
ENSG00000162885.14	1533.126848	0.023178557	0.048693059	0.476013568	0.634064712	0.801422338	B3GALNT2	protein_coding
ENSG00000162888.4	23.7269492	-0.123857133	0.104864748	-1.181113151	0.237557776	0.462766002	C1orf147	lncRNA
ENSG00000162889.11	7996.484564	0.071679081	0.04435144	1.616161316	0.10605944	0.28513962	MAPKAPK2	protein_coding
ENSG00000162891.10	6.192188523	0.138873147	0.215196294	0.645332431	0.51871178	0.719458975	IL20	protein_coding
ENSG00000162892.16	130.1203806	0.715285502	0.215339545	3.321663476	0.000894825	0.010538867	IL24	protein_coding
ENSG00000162894.12	942.7101573	-0.654712152	0.149543711	-4.37806544	1.19737E-05	0.000374842	FCMR	protein_coding
ENSG00000162896.6	95082.21739	-0.198744048	0.226524248	-0.877363242	0.380289372	0.605875785	PIGR	protein_coding
ENSG00000162897.16	172.7475978	-0.742211859	0.203573023	-3.645924441	0.000266432	0.004241987	FCAMR	protein_coding
ENSG00000162909.18	17059.4319	0.158606196	0.070456583	2.251119618	0.024377962	0.111074703	CAPN2	protein_coding
ENSG00000162910.19	1551.233594	-0.059339712	0.059930961	-0.990134506	0.322108379	0.551923437	MRPL55	protein_coding
ENSG00000162913.10	23.80776787	0.285506747	0.138491345	2.061549386	0.039250657	0.152147963	OBSCN-AS1	lncRNA
ENSG00000162923.17	7107.906373	0.195440929	0.042768466	4.5697437	4.88321E-06	0.000182657	WDR26	protein_coding
ENSG00000162924.15	1460.593983	0.000475596	0.05974595	0.007960298	0.993648668	0.997126724	REL	protein_coding
ENSG00000162927.14	596.8858315	-0.257190671	0.056513316	-4.550974673	5.3398E-06	0.000197364	PUS10	protein_coding
ENSG00000162928.9	2368.472898	0.044794118	0.041276531	1.085220022	0.277824219	0.506728025	PEX13	protein_coding
ENSG00000162929.14	678.9276868	-0.060328532	0.063522971	-0.949712066	0.342258577	0.572091541	KIAA1841	protein_coding
ENSG00000162931.12	125.3555888	0.340434505	0.118544178	2.871794382	0.004081484	0.032244171	TRIM17	protein_coding
ENSG00000162944.11	283.3130264	0.141323403	0.078966109	1.789671607	0.073506719	0.226968207	RFTN2	protein_coding
ENSG00000162946.23	332.3401274	0.272752617	0.080773614	3.376753924	0.000733466	0.009080189	DISC1	protein_coding
ENSG00000162947.5	8.86546483	0.000499874	0.202418357	0.002469507	0.99802962	0.998928218	LINC01931	lncRNA
ENSG00000162949.16	477.485578	-0.061295468	0.191830091	-0.319529992	0.749324652	0.871196616	CAPN13	protein_coding
ENSG00000162951.11	28.92001461	0.075533174	0.24576889	0.307334154	0.758589042	0.876465006	LRRTM1	protein_coding
ENSG00000162959.13	291.1605844	-0.248462733	0.066274561	-3.748991021	0.000177547	0.003108471	MEMO1	protein_coding
ENSG00000162961.14	2211.139638	-0.145527623	0.056967047	-2.554593063	0.010631201	0.062996441	DPY30	protein_coding
ENSG00000162971.11	735.1067056	-0.010559145	0.04594116	-0.229840628	0.818215613	0.910305965	TYW5	protein_coding
ENSG00000162972.10	879.091901	-0.100844403	0.055752871	-1.808775056	0.070485957	0.220753669	MAIP1	protein_coding

ENSG00000162975.5	72.13532799	0.334375912	0.189813413	1.76160318	0.078136366	0.236520452	KCNF1	protein_coding
ENSG00000162976.13	1986.477112	0.007750358	0.063982273	0.121132898	0.903585773	0.95409637	SLC66A3	protein_coding
ENSG00000162980.17	2058.244371	-0.117900534	0.041656955	-2.83027247	0.004650838	0.035345232	ARL5A	protein_coding
ENSG00000162981.14	4814.870001	0.007021455	0.167189801	0.041996909	0.966501162	0.984499865	LRATD1	protein_coding
ENSG00000162989.5	502.9905105	-0.321585544	0.215692264	-1.490946122	0.135975641	0.331711789	KCNJ3	protein_coding
ENSG00000162992.4	36.31552808	-0.482911625	0.270284703	-1.786677604	0.073989611	0.227974636	NEUROD1	protein_coding
ENSG00000162994.16	195.5545535	-0.147395097	0.082222938	-1.792627469	0.073032511	0.225920191	CLHC1	protein_coding
ENSG00000162997.15	96.26452383	-0.046557092	0.076046468	-0.612218994	0.540392876	0.735919031	PRORSD1P	transcribed_unitary_pseudogene
ENSG00000162998.5	1619.571268	-0.160962878	0.153041901	-1.051756915	0.292911091	0.522801009	FRZB	protein_coding
ENSG00000162999.13	300.6644653	-0.337218099	0.078820716	-4.278292775	1.88332E-05	0.000529823	DUSP19	protein_coding
ENSG00000163001.12	1652.818177	-0.132781399	0.051864983	-2.560135792	0.010463127	0.062240275	CFAP36	protein_coding
ENSG00000163002.13	1021.034616	-0.232851001	0.059824656	-3.892224646	9.93292E-05	0.001971968	NUP35	protein_coding
ENSG00000163006.12	452.8633894	-0.064778191	0.073935058	-0.87614986	0.38094857	0.606503081	CCDC138	protein_coding
ENSG00000163012.4	1.178532448	-0.133268768	0.364781792	-0.365338322	0.714858875	0.850991783	ZSWIM2	protein_coding
ENSG00000163013.12	1236.649454	0.157730754	0.101613756	1.552257877	0.120600532	0.308452248	FBXO41	protein_coding
ENSG00000163016.10	21.07164862	0.03357262	0.123473592	0.271901217	0.785697975	0.891554323	ALMS1P1	transcribed_unprocessed_pseudogene
ENSG00000163017.14	42600.86069	-1.046002407	0.245703245	-4.257177833	2.07024E-05	0.000572582	ACTG2	protein_coding
ENSG00000163026.12	493.5088088	-0.09636729	0.048867739	-1.972002236	0.048609351	0.174801923	WDCP	protein_coding
ENSG00000163029.16	2293.629912	-0.049793335	0.047741714	-1.04297333	0.296960645	0.526813793	SMC6	protein_coding
ENSG00000163032.12	1071.661308	0.245266254	0.203878771	1.203000454	0.228976145	0.452500724	VSNL1	protein_coding
ENSG00000163040.15	300.3406967	0.440020727	0.108946639	4.038864631	5.37105E-05	0.001217132	CCDC74A	protein_coding
ENSG00000163041.13	4218.497605	-0.08022229	0.071525508	-1.121589937	0.262036832	0.489506192	H3-3A	protein_coding
ENSG00000163046.15	8.076944016	-0.440507737	0.202466794	-2.175703626	0.029577429	0.126351561	ANKRD30BL	protein_coding
ENSG00000163050.18	35.72881892	-0.116117171	0.094574047	-1.227791067	0.21952541	0.441384016	COQ8A	protein_coding
ENSG00000163053.11	500.5761766	0.236742321	0.122165267	1.937885675	0.052637172	0.18372207	SLC16A14	protein_coding
ENSG00000163060.8	3.758882216	-0.387398019	0.212539243	-1.822712898	0.068346886	0.216638947	TEKT4	protein_coding
ENSG00000163064.7	15.13837249	1.231512871	0.29310588	4.201597284	2.65038E-05	0.000692006	EN1	protein_coding
ENSG00000163069.13	2972.895678	0.14168824	0.071342182	1.986037386	0.047029173	0.171024382	SGCB	protein_coding
ENSG00000163071.11	564.6474689	0.152444168	0.125672308	1.213029111	0.225118691	0.44812924	SPATA18	protein_coding
ENSG00000163072.16	1207.340363	-0.061265702	0.098032603	-0.624952309	0.532002359	0.729546659	NOSTRIN	protein_coding
ENSG00000163075.13	206.1015365	-0.459241151	0.199038417	-2.307299053	0.021038154	0.100568193	CFAP221	protein_coding
ENSG00000163081.4	0.708595017	0.39257628	0.444949305	0.8822944	0.377617624		CCDC140	lncRNA
ENSG00000163082.10	8645.303108	0.115826845	0.109150483	1.06116658	0.288614204	0.518358866	SGPP2	protein_coding
ENSG00000163083.6	807.0564439	0.177712826	0.156310218	1.136923921	0.255570091	0.482579854	INHBB	protein_coding
ENSG00000163092.21	4.20705305	-0.226634155	0.280113607	-0.809079423	0.418469464	0.638570011	XIRP2	protein_coding
ENSG00000163093.12	309.3993854	0.057367841	0.077312249	0.742027843	0.458070466	0.671533615	BBS5	protein_coding
ENSG00000163098.6	1.107259736	-0.122355596	0.379638419	-0.322295083	0.747229158	0.869897548	BIRC8	transcribed_processed_pseudogene
ENSG00000163104.18	2620.854044	0.013924705	0.055939621	0.248923832	0.8034197	0.902413021	SMARCAD1	protein_coding
ENSG00000163106.11	75.39574928	-0.205322428	0.135713738	-1.512908209	0.130302976	0.323103039	HPGDS	protein_coding
ENSG00000163110.15	10419.92864	0.123466174	0.051606275	2.392464374	0.016735653	0.086367781	PDLIM5	protein_coding
ENSG00000163114.6	0.541203507	-0.230440009	0.346370457	-0.66529926	0.505859104		PDHA2	protein_coding
ENSG00000163116.10	14.01164136	0.069596586	0.175803575	0.395876965	0.692195795	0.837654883	STPG2	protein_coding

ENSG00000163121.10	279.7521195	-1.025346772	0.182806702	-5.608912372	2.03602E-08	2.15136E-06	NEURL3	protein_coding
ENSG00000163125.15	6572.903671	0.067255855	0.045384825	1.481901829	0.138366418	0.334996022	RPRD2	protein_coding
ENSG00000163126.15	28.65494265	-0.123103332	0.085343717	-1.442441643	0.149177823	0.351162597	ANKRD23	protein_coding
ENSG00000163131.11	12706.09816	-0.164143934	0.092406619	-1.77632227	0.075679815	0.231561528	CTSS	protein_coding
ENSG00000163132.8	455.4620791	0.007643699	0.123662582	0.061810932	0.950713398	0.977012493	MSX1	protein_coding
ENSG00000163138.19	594.9628728	0.041263264	0.047911861	0.861232759	0.389109861	0.613600652	PACRGL	protein_coding
ENSG00000163141.20	50.32367743	-0.182034737	0.129563229	-1.404987648	0.160024951	0.365904465	BNIPL	protein_coding
ENSG00000163145.13	281.3453825	-0.104592616	0.180523788	-0.579384118	0.562330018	0.751208783	C1QTNF7	protein_coding
ENSG00000163154.6	208.153721	-0.231773051	0.103756818	-2.233810313	0.025495557	0.114449211	TNFAIP8L2	protein_coding
ENSG00000163155.12	440.6050361	0.160457851	0.057576223	2.786877008	0.005321867	0.038859912	LYSMD1	protein_coding
ENSG00000163156.12	1703.553578	0.030032742	0.055908855	0.53717326	0.59114794	0.771848802	SCNM1	protein_coding
ENSG00000163157.15	33.32724247	-0.313310045	0.09886568	-3.169047597	0.001529393	0.015733328	TMOD4	protein_coding
ENSG00000163159.14	2743.737974	0.084202082	0.054607928	1.541938778	0.123088472	0.31219507	VPS72	protein_coding
ENSG00000163161.14	2214.119647	0.053970704	0.036337684	1.485254384	0.13747645	0.333893336	ERCC3	protein_coding
ENSG00000163162.9	3922.844168	0.072254352	0.047740445	1.513482985	0.13015702	0.322898123	RNF149	protein_coding
ENSG00000163166.15	2772.67525	-0.022401812	0.037248732	-0.601411392	0.547566013	0.741097169	IWS1	protein_coding
ENSG00000163170.12	340.5495859	-0.164287846	0.073382126	-2.238799196	0.025168985	0.11358449	BOLA3	protein_coding
ENSG00000163171.8	2502.237298	-0.079637536	0.117817224	-0.675941371	0.499077869	0.70467279	CDC42EP3	protein_coding
ENSG00000163191.6	48804.41496	0.102706242	0.097282918	1.055747957	0.291083381	0.521139707	S100A11	protein_coding
ENSG00000163202.5	5.61323135	1.635352273	0.500788144	3.265557089	0.00109249	0.012295307	LCE3D	protein_coding
ENSG00000163206.6	0.497580629	-0.08957428	0.501552585	-0.178593995	0.85825651		SMCP	protein_coding
ENSG00000163207.7	46.81762877	1.293924807	0.476704967	2.714309469	0.006641409	0.04536249	IVL	protein_coding
ENSG00000163209.15	1072.381684	0.25096347	0.393793306	0.637297449	0.523931112	0.723157203	SPRR3	protein_coding
ENSG00000163214.21	1393.639159	-0.038454554	0.042474715	-0.905351677	0.365279107	0.59250473	DHX57	protein_coding
ENSG00000163216.7	87.81539052	0.079630704	0.376053358	0.211753738	0.832299162	0.917433347	SPRR2D	protein_coding
ENSG00000163217.2	2.993345264	-0.719664089	0.267851043	-2.686807121	0.00721386	0.048044988	BMP10	protein_coding
ENSG00000163218.15	34.73973913	0.267865011	0.255876036	1.04685462	0.295166635	0.525045757	PGLYRP4	protein_coding
ENSG00000163219.12	849.2256746	-0.466647534	0.109028788	-4.28003965	1.8686E-05	0.000527011	ARHGAP25	protein_coding
ENSG00000163220.11	3294.119383	0.501420816	0.187178241	2.678841364	0.007387739	0.04889664	S100A9	protein_coding
ENSG00000163221.9	94.65575398	1.206482263	0.205956702	5.857941259	4.6864E-09	6.60904E-07	S100A12	protein_coding
ENSG00000163235.16	1607.982034	0.393477648	0.109746381	3.585335989	0.000336645	0.005062097	TGFA	protein_coding
ENSG00000163239.12	32.40037596	-0.152532157	0.12481044	-1.222110562	0.22166582	0.444164258	TDRD10	protein_coding
ENSG00000163249.13	1514.137304	0.022075148	0.063098023	0.34985483	0.726447647	0.857340819	CCNYL1	protein_coding
ENSG00000163251.4	6717.228887	-0.22226934	0.0918885	-2.418902685	0.015567404	0.082158263	FZD5	protein_coding
ENSG00000163254.5	1.141445076	-0.015472757	0.41600989	-0.037193243	0.970330926	0.986080333	CRYGC	protein_coding
ENSG00000163257.11	2489.634244	-0.077505393	0.050355643	-1.539160026	0.123765234	0.313218979	DCAF16	protein_coding
ENSG00000163263.7	4.194227824	-0.239178839	0.146902913	-1.628142246	0.103494727	0.281136376	C1orf189	protein_coding
ENSG00000163273.4	26.01674849	-0.733710567	0.226563322	-3.238434896	0.001201874	0.013215767	NPPC	protein_coding
ENSG00000163281.12	856.3516554	0.101432042	0.065756886	1.542531104	0.122944585	0.312064326	GNPDA2	protein_coding
ENSG00000163283.7	152.0251607	1.596414798	0.260608678	6.125716183	9.02766E-10	1.67404E-07	ALPP	protein_coding
ENSG00000163285.8	2.168007749	0.32938094	0.313484155	1.050710012	0.293391796	0.523380314	GABRG1	protein_coding
ENSG00000163286.9	739.0340565	0.936701977	0.283330474	3.306040347	0.000946245	0.010990006	ALPG	protein_coding

ENSG00000163288.14	33.70581382	-1.012402033	0.244677613	-4.137697853	3.50808E-05	0.000864169	GABRB1	protein_coding
ENSG00000163291.14	1010.847374	0.128115046	0.057900086	2.212691829	0.026918899	0.118705208	PAQR3	protein_coding
ENSG00000163293.12	1219.498737	-0.015034667	0.109482019	-0.137325447	0.890773557	0.947917932	NIPAL1	protein_coding
ENSG00000163295.5	382.5477616	0.483375118	0.263549711	1.83409466	0.066639944	0.213119686	ALPI	protein_coding
ENSG00000163297.17	5047.986679	0.280535753	0.112693422	2.489371137	0.01279693	0.071797582	ANTXR2	protein_coding
ENSG00000163312.11	509.8637007	-0.037587932	0.039382275	-0.954437794	0.339862083	0.569709665	HELQ	protein_coding
ENSG00000163319.11	354.6433895	-0.108688483	0.048245888	-2.252803029	0.024271571	0.110792759	MRPS18C	protein_coding
ENSG00000163320.12	5358.073042	0.055027655	0.040498297	1.358764672	0.174221171	0.385462444	CGGBP1	protein_coding
ENSG00000163322.14	336.1200406	-0.140438641	0.065590872	-2.141130882	0.032263485	0.133732632	ABRAXAS1	protein_coding
ENSG00000163328.14	1426.824963	-0.311595564	0.135985397	-2.29138989	0.021940874	0.103349196	GPR155	protein_coding
ENSG00000163331.12	108.0870544	-0.864857214	0.257625932	-3.357027024	0.000787854	0.009576658	DAPL1	protein_coding
ENSG00000163344.6	1866.804762	-0.028530465	0.063412935	-0.449915542	0.65277134	0.81323989	PMVK	protein_coding
ENSG00000163346.17	6077.411425	0.026807433	0.079497995	0.337208914	0.735959418	0.863108264	PBXIP1	protein_coding
ENSG00000163347.6	6292.753742	-0.040559528	0.137319843	-0.295365381	0.767714776	0.882241701	CLDN1	protein_coding
ENSG00000163348.4	3671.829886	0.102728158	0.045538882	2.25583401	0.024081029	0.110101666	PYGO2	protein_coding
ENSG00000163349.22	6187.543092	0.167532193	0.060010438	2.79171755	0.00524291	0.038450065	HIPK1	protein_coding
ENSG00000163352.5	4.737748271	0.343658448	0.156378165	2.197611463	0.027976806	0.121586243	LENEP	protein_coding
ENSG00000163354.15	62.89589886	0.248881685	0.114717084	2.169525893	0.030042781	0.127749244	DCST2	protein_coding
ENSG00000163357.11	30.24318117	0.175911939	0.100374982	1.752547652	0.079679684	0.239600662	DCST1	protein_coding
ENSG00000163359.16	49902.92771	0.569842542	0.11471571	4.967432447	6.78452E-07	3.82414E-05	COL6A3	protein_coding
ENSG00000163362.11	7188.742024	0.253574573	0.100718378	2.517659416	0.011813749	0.067827781	INAVA	protein_coding
ENSG00000163364.10	90.9070877	0.499677832	0.140929952	3.545575843	0.000391756	0.005651392	LINC01116	lncRNA
ENSG00000163374.19	2969.354411	0.057514589	0.035831245	1.605151864	0.108460388	0.288792516	YY1AP1	protein_coding
ENSG00000163376.11	216.2876776	-0.388158181	0.098452132	-3.942608179	8.06003E-05	0.001678126	KBTBD8	protein_coding
ENSG00000163377.16	45.66824109	-0.637003242	0.30008088	-2.122771843	0.033772975	0.13759632	TAFA4	protein_coding
ENSG00000163378.14	1951.744203	0.054635794	0.071928008	0.759589981	0.44749971	0.662772263	EOGT	protein_coding
ENSG00000163380.16	52.90183326	-0.389216249	0.126157548	-3.085160227	0.002034425	0.019373721	LMOD3	protein_coding
ENSG00000163382.12	5366.152687	-0.034712375	0.069580356	-0.498881821	0.617862642	0.790621064	NAXE	protein_coding
ENSG00000163389.12	1273.596871	-0.051029977	0.046171583	-1.105224781	0.269062201	0.497092617	POGLUT1	protein_coding
ENSG00000163393.13	339.2144115	0.245595246	0.106397131	2.308288237	0.02098311	0.100396777	SLC22A15	protein_coding
ENSG00000163394.6	52.95339755	-0.904423321	0.320018289	-2.826161353	0.004710953	0.03568385	CCKAR	protein_coding
ENSG00000163395.17	64.10464348	-0.210839997	0.231683985	-0.910032675	0.362805278	0.590166322	IGFN1	protein_coding
ENSG00000163399.16	18059.09598	-0.101343902	0.09171164	-1.105027695	0.269147589	0.497208211	ATP1A1	protein_coding
ENSG00000163406.11	245.2073718	0.270070218	0.120553725	2.240247809	0.025074839	0.113392913	SLC15A2	protein_coding
ENSG00000163412.13	1506.154734	0.004518359	0.090312687	0.050030172	0.960098345	0.981470642	EIF4E3	protein_coding
ENSG00000163421.9	57.17551442	0.24338812	0.203216125	1.197681136	0.231041176	0.455013392	PROK2	protein_coding
ENSG00000163424.9	3.768971215	1.356536202	0.332548208	4.079216701	4.51877E-05	0.001060842	TEX55	protein_coding
ENSG00000163428.4	5517.637328	0.069169222	0.048764275	1.418440506	0.156062197	0.360799	LRRC58	protein_coding
ENSG00000163430.12	16717.19756	0.324541019	0.103499594	3.135674328	0.001714595	0.017132975	FSTL1	protein_coding
ENSG00000163431.13	5239.302212	-0.438515186	0.214395984	-2.045351675	0.0408202	0.155913913	LMOD1	protein_coding
ENSG00000163435.16	19616.23508	0.169781236	0.092376085	1.837934951	0.066071991	0.211956652	ELF3	protein_coding
ENSG00000163440.12	4.516254597	0.043111129	0.273904106	0.157394972	0.874933575	0.939597329	PDCL2	protein_coding

ENSG00000163444.12	2599.062654	0.013397423	0.050528788	0.265144365	0.790898252	0.895031374	TMEM183A	protein_coding
ENSG00000163449.11	54.09946368	0.626979274	0.130531486	4.803279974	1.56087E-06	7.38526E-05	TMEM169	protein_coding
ENSG00000163453.11	19099.8444	0.176303366	0.101676474	1.733964201	0.082924435	0.24562425	IGFBP7	protein_coding
ENSG00000163462.18	174.5937742	0.480427126	0.113717332	4.224748475	2.39208E-05	0.000639507	TRIM46	protein_coding
ENSG00000163463.13	158.2384325	0.135909289	0.081059442	1.676662033	0.093608589	0.264468213	KRTCAP2	protein_coding
ENSG00000163464.8	121.436725	0.490989105	0.205472593	2.38956008	0.016868565	0.086796722	CXCR1	protein_coding
ENSG00000163466.16	18914.99939	-0.054608724	0.04794037	-1.139096856	0.254662759	0.481471606	ARPC2	protein_coding
ENSG00000163467.12	55.40412976	-0.001625184	0.094913388	-0.017122807	0.986338644	0.993595118	TSACC	protein_coding
ENSG00000163468.15	15172.15809	0.038281454	0.057786646	0.662461951	0.507675212	0.711532314	CCT3	protein_coding
ENSG00000163472.19	423.4236806	0.354569894	0.086775298	4.086069422	4.38743E-05	0.001040128	TMEM79	protein_coding
ENSG00000163479.14	9734.022597	0.032656218	0.058735419	0.555988505	0.578218713	0.762552942	SSR2	protein_coding
ENSG00000163481.8	1049.076569	0.035641335	0.045905055	0.776414157	0.437504488	0.65441709	RNF25	protein_coding
ENSG00000163482.12	1475.139505	0.019881587	0.062679458	0.317194629	0.751095925	0.872260266	STK36	protein_coding
ENSG00000163485.17	135.879984	0.502593308	0.12598843	3.989202075	6.62959E-05	0.00144097	ADORA1	protein_coding
ENSG00000163491.16	57.73116752	-0.050549848	0.113270823	-0.446274219	0.655399177	0.814798868	NEK10	protein_coding
ENSG00000163492.15	78.37539933	-0.463177841	0.157542784	-2.940013049	0.003281984	0.027471961	CCDC141	protein_coding
ENSG00000163497.3	64.07040865	-0.983167802	0.278014236	-3.536393728	0.00040563	0.005809556	FEV	protein_coding
ENSG00000163499.12	28.89867207	0.2984685	0.228281455	1.307458371	0.191057084	0.406920422	CRYBA2	protein_coding
ENSG00000163501.7	2340.303357	-0.392099046	0.179443012	-2.185089522	0.028882283	0.124392495	IHH	protein_coding
ENSG00000163507.14	891.6294185	0.061676635	0.077000781	0.80098714	0.423139091	0.64225469	CIP2A	protein_coding
ENSG00000163508.13	285.936755	-0.409264129	0.147690944	-2.771084804	0.005586987	0.040255316	EOMES	protein_coding
ENSG00000163510.14	1882.164503	-0.035218378	0.037814579	-0.931343914	0.351675693	0.580510563	CWC22	protein_coding
ENSG00000163512.14	876.3644183	0.145050109	0.042617577	3.403527805	0.000665216	0.008409671	AZI2	protein_coding
ENSG00000163513.19	11482.74179	-0.051949294	0.083483388	-0.622271027	0.533763669	0.730980546	TGFBR2	protein_coding
ENSG00000163515.6	35.67190998	0.149935365	0.253538033	0.591372282	0.554271008	0.745458233	RETNLB	protein_coding
ENSG00000163516.14	1624.925307	-0.012839518	0.048696986	-0.263661451	0.792040802	0.895649835	ANKZF1	protein_coding
ENSG00000163517.15	1107.376805	-0.055062396	0.081021692	-0.679600675	0.496757342	0.70303643	HDAC11	protein_coding
ENSG00000163518.11	33.52730519	-1.142399843	0.199013664	-5.740308595	9.45042E-09	1.14195E-06	FCRL4	protein_coding
ENSG00000163519.14	148.5456151	-0.660163034	0.151014611	-4.371517626	1.23386E-05	0.000383141	TRAT1	protein_coding
ENSG00000163520.14	4050.792559	0.597403177	0.141492232	4.222162379	2.4197E-05	0.000641772	FBLN2	protein_coding
ENSG00000163521.16	481.0297377	0.215954888	0.098033002	2.202879476	0.027603245	0.120612312	GLB1L	protein_coding
ENSG00000163527.10	11445.42665	0.086647674	0.05535069	1.565430785	0.117481935	0.303324462	STT3B	protein_coding
ENSG00000163528.13	867.399529	-0.037507318	0.059292317	-0.632583092	0.527005931	0.725557231	CHCHD4	protein_coding
ENSG00000163530.4	7.224626811	-0.100098978	0.35004612	-0.285959398	0.774909219	0.886049147	DPPA2	protein_coding
ENSG00000163531.15	1986.631173	-0.326663458	0.167466596	-1.95061861	0.051102432	0.180411916	NFASC	protein_coding
ENSG00000163534.15	179.3824368	-1.454287165	0.248449181	-5.85345929	4.81452E-09	6.72444E-07	FCRL1	protein_coding
ENSG00000163535.18	895.5914155	0.069235024	0.082835131	0.835817161	0.403257771	0.624990558	SGO2	protein_coding
ENSG00000163536.13	261.1701814	-0.210670248	0.096766364	-2.177102034	0.029472955	0.126064289	SERPINI1	protein_coding
ENSG00000163539.17	2709.933028	-0.018737633	0.050656325	-0.36989721	0.71145908	0.849116527	CLASP2	protein_coding
ENSG00000163541.12	4811.674645	-0.199611882	0.061187295	-3.262309324	0.001105085	0.012405019	SUCLG1	protein_coding
ENSG00000163545.11	1509.089124	-0.228378318	0.111558184	-2.047167763	0.040641615	0.155490753	NUAK2	protein_coding
ENSG00000163554.15	14.720851	-0.096456755	0.203493749	-0.474003526	0.635497393	0.80230234	SPTA1	protein_coding

ENSG00000163558.13	6921.940083	0.138035029	0.083719903	1.648771965	0.099194361	0.274293775	PRKCI	protein_coding
ENSG00000163563.8	657.0402283	-0.01250682	0.125543473	-0.099621431	0.920644879	0.962643357	MNDA	protein_coding
ENSG00000163564.15	215.6441005	-0.693380635	0.134842985	-5.142133545	2.71636E-07	1.81321E-05	PYHIN1	protein_coding
ENSG00000163565.19	5436.161234	0.135563188	0.097116364	1.395884096	0.162749401	0.369766978	IFI16	protein_coding
ENSG00000163568.15	327.5502977	0.394757572	0.188086773	2.098805604	0.035834041	0.143024362	AIM2	protein_coding
ENSG00000163576.18	28.55843611	-0.243220144	0.137765486	-1.765465006	0.077485647	0.235253398	EFHB	protein_coding
ENSG00000163577.8	879.9721478	0.031129116	0.090526179	0.343868657	0.730945064	0.860178046	EIF5A2	protein_coding
ENSG00000163581.14	29.91423822	-1.303380759	0.265488255	-4.909372582	9.13682E-07	4.84897E-05	SLC2A2	protein_coding
ENSG00000163584.18	3904.025844	-0.142026264	0.133824155	-1.061290199	0.288558038	0.518327801	RPL22L1	protein_coding
ENSG00000163586.10	3357.369825	-0.89494116	0.307568771	-2.909727012	0.003617446	0.029486987	FABP1	protein_coding
ENSG00000163590.14	2111.172381	-0.06710514	0.095388941	-0.70348972	0.481750611	0.69061275	PPM1L	protein_coding
ENSG00000163596.16	342.6840386	-0.287895267	0.104471925	-2.75571899	0.005856329	0.041617093	ICA1L	protein_coding
ENSG00000163597.15	2245.983923	0.084471041	0.062763496	1.34586257	0.178346842	0.390977989	SNHG16	lncRNA
ENSG00000163599.17	238.1742964	-0.635840997	0.127218284	-4.998031546	5.79185E-07	3.33851E-05	CTLA4	protein_coding
ENSG00000163600.13	169.4909153	-0.513734385	0.130838411	-3.926479857	8.61981E-05	0.001770148	ICOS	protein_coding
ENSG00000163602.11	2980.208709	0.099720467	0.04189665	2.380153688	0.017305419	0.088168518	RYBP	protein_coding
ENSG00000163605.15	3353.035427	-0.061366769	0.053598455	-1.144935405	0.252235916	0.478949643	PPP4R2	protein_coding
ENSG00000163606.11	149.797239	-0.31037184	0.111437423	-2.785167069	0.005350015	0.038966673	CD200R1	protein_coding
ENSG00000163607.17	495.1175104	0.031543004	0.049312702	0.639652725	0.522398396	0.722014026	GTPBP8	protein_coding
ENSG00000163608.15	2040.862928	0.020025023	0.044258388	0.452457122	0.650939704	0.811918582	NEPRO	protein_coding
ENSG00000163611.11	183.621891	-0.003397692	0.068146779	-0.04985844	0.960235196	0.981474155	SPICE1	protein_coding
ENSG00000163612.10	3.549288368	-0.083393877	0.176619406	-0.472167123	0.636807504	0.803214281	FAM86KP	unprocessed_pseudogene
ENSG00000163617.11	417.5692809	-0.106750203	0.077389371	-1.379391015	0.167774228	0.376860697	CCDC191	protein_coding
ENSG00000163618.18	554.8707539	-0.625851845	0.218327061	-2.866579344	0.004149341	0.032542562	CADPS	protein_coding
ENSG00000163623.9	75.38811041	1.046155784	0.274491954	3.811243897	0.000138269	0.002549304	NKX6-1	protein_coding
ENSG00000163624.6	2840.857388	-0.083954677	0.094148152	-0.891729417	0.372537982	0.598999618	CDS1	protein_coding
ENSG00000163625.16	2920.196848	0.256397727	0.059075043	4.340203848	1.42351E-05	0.000428399	WDFY3	protein_coding
ENSG00000163626.17	979.0910215	-0.071553692	0.054331062	-1.316994175	0.187840577	0.403007223	COX18	protein_coding
ENSG00000163629.13	787.2134363	0.296894375	0.141860442	2.092862338	0.036361445	0.144386	PTPN13	protein_coding
ENSG00000163630.11	75.96152903	-0.846139417	0.243487144	-3.475088674	0.000510685	0.006902636	SYNPR	protein_coding
ENSG00000163631.17	424.311421	-2.532243762	0.327600306	-7.729674586	1.07822E-14	9.39711E-12	ALB	protein_coding
ENSG00000163632.14	23.08255627	-0.085680686	0.094708751	-0.904675496	0.365637326	0.592847811	C3orf49	protein_coding
ENSG00000163633.12	3.334403585	0.103098259	0.150122307	0.686761753	0.492232876	0.699244752	C4orf36	protein_coding
ENSG00000163634.12	2521.183083	-0.136522652	0.055619597	-2.454578231	0.014104997	0.076812479	THOC7	protein_coding
ENSG00000163635.20	2385.120142	0.003605983	0.046300072	0.077882878	0.93792122	0.970549795	ATXN7	protein_coding
ENSG00000163636.11	3221.754491	-0.063526149	0.040849692	-1.555119402	0.119917635	0.307391222	PSMD6	protein_coding
ENSG00000163637.13	1013.453235	-0.122965088	0.142772471	-0.86126609	0.389091507	0.613600652	PRICKLE2	protein_coding
ENSG00000163638.13	1656.633057	0.196163639	0.100226858	1.957196333	0.05032439	0.178775153	ADAMTS9	protein_coding
ENSG00000163644.15	1177.342921	-0.241361349	0.087477923	-2.759111559	0.005795875	0.041329868	PPM1K	protein_coding
ENSG00000163645.15	7.015618356	-0.038292945	0.170804654	-0.224191462	0.822608327	0.912563783	ERICH6	protein_coding
ENSG00000163646.12	2.449808019	0.146481484	0.34711764	0.421993777	0.67302956	0.826064926	CLRN1	protein_coding
ENSG00000163655.16	5214.662647	0.004778207	0.052760402	0.090564258	0.927838833	0.965873554	GMPS	protein_coding

ENSG00000163659.13	2356.216178	0.286866521	0.069013776	4.156655927	3.2294E-05	0.000809244	TIPARP	protein_coding
ENSG00000163660.12	5388.729928	0.027477503	0.062831858	0.437318	0.661880743	0.818908524	CCNL1	protein_coding
ENSG00000163661.4	67.80956323	0.24394542	0.155679907	1.566967918	0.117122191	0.302790324	PTX3	protein_coding
ENSG00000163666.10	44.9708047	-0.338993868	0.087669938	-3.866705917	0.000110315	0.002134641	HESX1	protein_coding
ENSG00000163673.7	56.35668479	0.022437898	0.115117825	0.194912455	0.845461484	0.924232373	DCLK3	protein_coding
ENSG00000163681.16	7151.432041	-0.129372222	0.08453289	-1.530436521	0.125908715	0.316365339	SLMAP	protein_coding
ENSG00000163682.17	28004.17403	-0.23278362	0.066130664	-3.520055704	0.000431456	0.006095525	RPL9	protein_coding
ENSG00000163683.12	3836.944323	-0.001284628	0.070878541	-0.018124361	0.985539644	0.993312542	SMIM14	protein_coding
ENSG00000163684.12	405.5716526	-0.047340383	0.059441157	-0.796424316	0.425785449	0.644520374	RPP14	protein_coding
ENSG00000163686.15	809.7922091	-0.203202781	0.066423946	-3.059179622	0.00221944	0.020595518	ABHD6	protein_coding
ENSG00000163687.14	200.6405638	-0.662703309	0.170501764	-3.886782711	0.000101582	0.002008449	DNASE1L3	protein_coding
ENSG00000163689.20	217.2065007	0.18114552	0.167860693	1.079141976	0.280524441	0.50939679	CFAP20DC	protein_coding
ENSG00000163694.15	14723.83082	-0.121818864	0.075700683	-1.609217515	0.107568788	0.287488951	RBM47	protein_coding
ENSG00000163697.17	3127.534044	0.032213197	0.070074458	0.459699544	0.645731897	0.808785697	APBB2	protein_coding
ENSG00000163701.19	2299.171254	-0.25093656	0.102459174	-2.449137063	0.014319894	0.077570473	IL17RE	protein_coding
ENSG00000163702.20	1230.113668	0.083377263	0.069012741	1.208143033	0.22699225	0.45045955	IL17RC	protein_coding
ENSG00000163703.19	132.9710892	-0.012490062	0.06328067	-0.197375627	0.843533601	0.923282941	CRELD1	protein_coding
ENSG00000163704.12	249.5149262	0.168812929	0.077700573	2.172608534	0.029809793	0.127054664	PRRT3	protein_coding
ENSG00000163705.13	6.560028275	0.071186799	0.150647089	0.472540154	0.636541288	0.803015363	FANCD2OS	protein_coding
ENSG00000163710.9	122.7402154	0.604572392	0.18712389	3.23086695	0.001234154	0.013478877	PCOLCE2	protein_coding
ENSG00000163714.18	7403.990786	-0.017297201	0.053581422	-0.322820865	0.746830911	0.869666255	U2SURP	protein_coding
ENSG00000163719.20	2656.284759	0.031491841	0.04890416	0.643950141	0.519607766	0.720023432	MTMR14	protein_coding
ENSG00000163728.11	1170.276738	0.085371145	0.09171494	0.930831395	0.351940787	0.580641264	TTC14	protein_coding
ENSG00000163734.4	2490.803551	-0.025837884	0.160586448	-0.160897036	0.872174497	0.938226491	CXCL3	protein_coding
ENSG00000163735.7	9346.294007	0.111689571	0.264297108	0.422590968	0.672593717	0.825902176	CXCL5	protein_coding
ENSG00000163736.4	66.85960941	1.278883133	0.238919455	5.352779391	8.66134E-08	7.23056E-06	PPBP	protein_coding
ENSG00000163737.4	92.34984799	0.764117389	0.220770894	3.461132832	0.000537907	0.007185894	PF4	protein_coding
ENSG00000163738.18	588.3077694	0.040582377	0.074455268	0.545057158	0.585714207	0.768044896	MTHFD2L	protein_coding
ENSG00000163739.5	5175.510835	0.447942054	0.171864823	2.606362646	0.009150952	0.056780724	CXCL1	protein_coding
ENSG00000163743.13	1162.07861	0.102700025	0.05903926	1.739520872	0.081943184	0.243826434	RCHY1	protein_coding
ENSG00000163746.12	22.02543911	0.438902088	0.131626219	3.334457928	0.000854658	0.010164695	PLSCR2	protein_coding
ENSG00000163749.17	32.29217678	0.093483587	0.12380791	0.755069579	0.450207235	0.665062567	CCDC158	protein_coding
ENSG00000163751.4	1099.83068	-0.306345747	0.163723454	-1.871117057	0.061328858	0.202633076	CPA3	protein_coding
ENSG00000163754.18	2981.389735	-0.072150579	0.068012045	-1.060850015	0.288758068	0.518510398	GYG1	protein_coding
ENSG00000163755.9	2160.39429	0.199471804	0.058895488	3.386877493	0.000706929	0.008819314	HPS3	protein_coding
ENSG00000163762.7	667.3003403	-0.139459619	0.09329179	-1.49487559	0.134946914	0.330239887	TM4SF18	protein_coding
ENSG00000163781.14	3814.25113	0.070488883	0.060957256	1.15636574	0.247531589	0.474119118	TOPBP1	protein_coding
ENSG00000163785.13	3113.088612	0.059220536	0.047395474	1.2494977	0.211483094	0.431875851	RYK	protein_coding
ENSG00000163788.14	2214.802663	-0.020493287	0.055047682	-0.372282463	0.709682555	0.848253537	SNRK	protein_coding
ENSG00000163792.6	44.18310227	-0.22125481	0.226249795	-0.977922698	0.328112558	0.557636163	TCF23	protein_coding
ENSG00000163793.13	6.391856227	-0.116210758	0.281490906	-0.412840185	0.679723707	0.830358098	DNAJC5G	protein_coding
ENSG00000163794.6	38.51672393	-0.148564339	0.106641559	-1.3931186	0.163583929	0.370754506	UCN	protein_coding

ENSG00000163795.14	831.9042364	-0.073915606	0.060249902	-1.226817023	0.219891371	0.44180028	ZNF513	protein_coding
ENSG00000163798.13	1582.961292	-0.024705599	0.033445881	-0.738673888	0.460105045	0.673247962	SLC4A1AP	protein_coding
ENSG00000163803.13	308.8043377	-0.031671191	0.113061106	-0.28012455	0.77938195	0.888395435	PLB1	protein_coding
ENSG00000163806.16	41.36606993	-0.107788758	0.09909554	-1.087725623	0.276716253	0.505574782	SPDYA	protein_coding
ENSG00000163807.6	2364.281812	0.047008268	0.047781121	0.983825136	0.325201498	0.554910552	KIAA1143	protein_coding
ENSG00000163808.17	975.0553906	-0.022930898	0.090904776	-0.252251856	0.800846398	0.900652511	KIF15	protein_coding
ENSG00000163810.12	9.589111756	-0.115070686	0.181131294	-0.635288822	0.525240062	0.724316019	TGM4	protein_coding
ENSG00000163811.12	3671.044568	-0.059236197	0.053456865	-1.108112065	0.267813421	0.495749827	WDR43	protein_coding
ENSG00000163812.15	6285.236207	0.021004814	0.055820229	0.376293944	0.706698381	0.846363858	ZDHHC3	protein_coding
ENSG00000163814.8	7460.317442	0.32758478	0.093211163	3.514437212	0.000440687	0.00619278	CDCP1	protein_coding
ENSG00000163815.6	136.9133169	-0.155338548	0.154270703	-1.006921892	0.313972311	0.543237171	CLEC3B	protein_coding
ENSG00000163817.16	2205.286151	0.336832488	0.190837231	1.765025023	0.07755956	0.235367593	SLC6A20	protein_coding
ENSG00000163818.17	1323.928476	0.209425554	0.059284475	3.532553089	0.000411568	0.00587451	LZTFL1	protein_coding
ENSG00000163820.15	4636.077749	-0.071786549	0.088709106	-0.809235406	0.418379753	0.638570011	FYCO1	protein_coding
ENSG00000163823.4	806.8940109	-0.062832608	0.114962475	-0.546548844	0.584688722	0.76743917	CCR1	protein_coding
ENSG00000163825.4	0.980269185	-0.341274466	0.347724392	-0.981451038	0.326370368	0.556186364	RTP3	protein_coding
ENSG00000163827.13	141.292013	-0.263055945	0.154973247	-1.697428104	0.089615753	0.257547034	LRRC2	protein_coding
ENSG00000163832.16	794.9536828	-0.030889525	0.043635392	-0.707900704	0.479006923	0.688514189	ELP6	protein_coding
ENSG00000163833.8	6.773061656	-0.030815918	0.215684368	-0.142875063	0.886388854	0.945676756	FBXO40	protein_coding
ENSG00000163840.10	8065.379494	0.086356183	0.062677462	1.377786857	0.168269105	0.377544166	DTX3L	protein_coding
ENSG00000163848.20	3743.8896	0.036020648	0.044360546	0.811997394	0.416793127	0.637196627	ZNF148	protein_coding
ENSG00000163864.17	420.2757267	-0.210235004	0.115213749	-1.824738844	0.068040448	0.216076273	NMNAT3	protein_coding
ENSG00000163866.9	1921.107882	-0.069203044	0.03839981	-1.802171517	0.071518425	0.222834147	SMIM12	protein_coding
ENSG00000163867.17	688.7158893	-0.11287914	0.046574305	-2.423635507	0.015366019	0.081371373	ZMYM6	protein_coding
ENSG00000163870.16	1851.248883	0.028434615	0.047281332	0.601392007	0.547578921	0.741097169	TPRA1	protein_coding
ENSG00000163872.16	3158.916896	0.124343256	0.049595339	2.507156093	0.012170695	0.069174695	YEATS2	protein_coding
ENSG00000163873.10	101.710727	-0.379202341	0.216912765	-1.748179002	0.080433036	0.240895562	GRIK3	protein_coding
ENSG00000163874.11	4148.464568	0.130704881	0.120794682	1.082041691	0.279234003	0.507909577	ZC3H12A	protein_coding
ENSG00000163875.16	2909.238254	-0.123298104	0.042144907	-2.925575411	0.003438198	0.028446244	MEAF6	protein_coding
ENSG00000163877.11	950.0110771	0.068729661	0.031530699	2.179769655	0.02927454	0.125412568	SNIP1	protein_coding
ENSG00000163879.11	742.940037	0.018663643	0.152548693	0.122345479	0.902625416	0.953683147	DNALI1	protein_coding
ENSG00000163882.9	3090.260828	-0.014001952	0.062531077	-0.223919883	0.822819644	0.912575695	POLR2H	protein_coding
ENSG00000163884.4	272.2210824	-0.105637532	0.181009027	-0.583603668	0.559486992	0.749094068	KLF15	protein_coding
ENSG00000163885.12	9.516425498	0.306381732	0.142959285	2.143139794	0.032101875	0.133235647	CFAP100	protein_coding
ENSG00000163888.4	187.9822161	-0.709023477	0.182498601	-3.885089924	0.000102292	0.002017476	CAMK2N2	protein_coding
ENSG00000163898.10	4658.57991	0.347183767	0.143427433	2.420623158	0.01549393	0.081829958	LIPH	protein_coding
ENSG00000163900.11	2663.814611	-0.139019654	0.066995153	-2.075070318	0.037980018	0.148795761	TMEM41A	protein_coding
ENSG00000163902.12	21877.20062	0.079682533	0.049088721	1.623235074	0.104539163	0.282880222	RPN1	protein_coding
ENSG00000163904.13	2393.317032	-0.030903237	0.051070577	-0.605108429	0.545106954	0.739474076	SENP2	protein_coding
ENSG00000163909.8	2272.694662	0.180226412	0.120320681	1.497883903	0.134163417	0.329069343	HEYL	protein_coding
ENSG00000163913.12	1417.84379	0.073184889	0.05769284	1.268526362	0.204610043	0.424080468	IFT122	protein_coding
ENSG00000163914.5	2.151939034	-0.074535609	0.260966473	-0.285613735	0.775173983	0.886142619	RHO	protein_coding

ENSG00000163915.7	49.71632772	0.078379449	0.144566572	0.542168548	0.5877024	0.769679254	IGF2BP2-AS1	lncRNA
ENSG00000163918.11	1228.470827	-0.125471194	0.067711595	-1.853023759	0.063878953	0.207853002	RFC4	protein_coding
ENSG00000163923.10	486.0695073	0.068742082	0.140509081	0.489235867	0.62467472	0.795177508	RPL39L	protein_coding
ENSG00000163930.10	3753.340066	0.111615496	0.044142126	2.528548252	0.011453534	0.066362273	BAP1	protein_coding
ENSG00000163931.16	16989.99081	0.053640071	0.07200276	0.744972425	0.45628839	0.670286062	TKT	protein_coding
ENSG00000163932.15	5151.358593	-0.013720784	0.063412744	-0.216372659	0.828697277	0.915634984	PRKCD	protein_coding
ENSG00000163933.10	1072.369563	0.005037525	0.047997315	0.104954309	0.916412064	0.960383969	RFT1	protein_coding
ENSG00000163935.14	1537.145004	0.074320069	0.061978927	1.199118371	0.230481922	0.454363751	SFMBT1	protein_coding
ENSG00000163938.17	4577.493057	0.065001972	0.062969132	1.032283124	0.301939505	0.531642401	GNL3	protein_coding
ENSG00000163939.18	4508.73942	0.091573377	0.054283711	1.686940247	0.091614842	0.260951695	PBRM1	protein_coding
ENSG00000163945.19	1832.661615	0.088887234	0.068603754	1.295661379	0.195092173	0.41215283	UVSSA	protein_coding
ENSG00000163946.13	4687.930981	0.036248159	0.052805009	0.686453033	0.492427472	0.699475583	TASOR	protein_coding
ENSG00000163947.12	2077.972178	-0.171482198	0.064375039	-2.663799523	0.007726359	0.050506528	ARHGEF3	protein_coding
ENSG00000163950.13	3820.520319	-0.115260153	0.062017089	-1.858522457	0.063094857	0.206152818	SLBP	protein_coding
ENSG00000163956.13	6339.112543	0.121697961	0.059304782	2.052076697	0.040162209	0.154374931	LRPAP1	protein_coding
ENSG00000163958.14	13.29764134	0.032431276	0.147062867	0.22052661	0.825461051	0.913880144	ZDHHC19	protein_coding
ENSG00000163959.10	172.8594906	0.051418998	0.095093349	0.540721284	0.588699706	0.770452806	SLC51A	protein_coding
ENSG00000163960.12	3275.532148	0.076371771	0.049448173	1.54448114	0.122471817	0.311185467	UBXN7	protein_coding
ENSG00000163961.4	1514.198155	0.04288958	0.050964044	0.841565467	0.400031226	0.622728091	RNF168	protein_coding
ENSG00000163964.17	1606.563111	-0.050295162	0.050767281	-0.990700339	0.321831925	0.551666659	PIGX	protein_coding
ENSG00000163975.12	2432.58775	0.567601944	0.150574356	3.769579092	0.000163523	0.002905264	MELTF	protein_coding
ENSG00000163982.6	2.678241988	0.698549183	0.328736967	2.124948677	0.033590896	0.137071866	OTOP1	protein_coding
ENSG00000163993.7	12455.13401	0.50956542	0.169847429	3.000136201	0.002698589	0.023711033	S100P	protein_coding
ENSG00000163995.21	716.669067	0.265016616	0.122519604	2.163054789	0.030536966	0.12904474	ABLIM2	protein_coding
ENSG00000164002.11	144.7890449	0.086982246	0.076071492	1.14342764	0.252861082	0.479688624	EXO5	protein_coding
ENSG00000164007.11	23.05132292	-0.208597039	0.221197891	-0.943033581	0.345663727	0.575186659	CLDN19	protein_coding
ENSG00000164008.16	150.3144227	-0.118093521	0.058293757	-2.025834777	0.042781719	0.16096216	C1orf50	protein_coding
ENSG00000164010.15	498.7580931	0.024874457	0.057832259	0.430113873	0.667112809	0.822230311	ERMAP	protein_coding
ENSG00000164011.18	492.7724515	-0.060428493	0.04498209	-1.343390064	0.179145703	0.391997604	ZNF691	protein_coding
ENSG00000164022.17	3417.155461	-0.06669726	0.041613936	-1.6027626	0.108987077	0.289593283	AIMP1	protein_coding
ENSG00000164023.14	3134.936094	0.304411914	0.089198588	3.412743643	0.000643124	0.008208969	SGMS2	protein_coding
ENSG00000164024.12	3380.214169	0.026781435	0.055458247	0.482911669	0.629158456	0.79832391	METAP1	protein_coding
ENSG00000164031.17	2398.829469	0.028782189	0.046212401	0.622823921	0.533400237	0.730712834	DNAJB14	protein_coding
ENSG00000164032.12	6644.78926	-0.092057003	0.073512124	-1.252269654	0.210471623	0.43075906	H2AZ1	protein_coding
ENSG00000164035.10	902.5323501	-0.192307152	0.102962521	-1.86773935	0.061798411	0.203644083	EMCN	protein_coding
ENSG00000164037.17	26.45690018	-0.079174723	0.094425106	-0.838492292	0.401754275	0.623766204	SLC9B1	protein_coding
ENSG00000164038.16	1118.395909	0.254771396	0.126389242	2.015768054	0.043824236	0.16337828	SLC9B2	protein_coding
ENSG00000164039.15	753.2733253	-0.17119349	0.07893989	-2.168656305	0.030108787	0.127930052	BDH2	protein_coding
ENSG00000164040.18	4900.383031	-0.172709696	0.049009429	-3.524009553	0.000425069	0.006023814	PGRMC2	protein_coding
ENSG00000164045.12	736.4611737	-0.114460178	0.099796727	-1.146933184	0.251409237	0.478028815	CDC25A	protein_coding
ENSG00000164047.6	11.05024661	0.274010417	0.200512104	1.366553001	0.171765457	0.382298551	CAMP	protein_coding
ENSG00000164048.14	662.7219188	-0.13657141	0.062921712	-2.17049736	0.029969188	0.127548327	ZNF589	protein_coding

ENSG00000164049.14	17.73410373	-0.139042797	0.161263952	-0.862206314	0.388573996	0.613066221	FBXW12	protein_coding
ENSG00000164050.13	7253.161818	0.162571975	0.076030115	2.138257634	0.032495839	0.134352104	PLXNB1	protein_coding
ENSG00000164051.14	861.0987873	-0.029626056	0.064137253	-0.46191651	0.644141194	0.807762325	CCDC51	protein_coding
ENSG00000164053.21	624.4166468	0.006282859	0.045824464	0.1371071	0.89094614	0.948013826	ATRIP	protein_coding
ENSG00000164054.15	11707.26776	0.06083205	0.053966742	1.127213672	0.259652151	0.486828233	SHISA5	protein_coding
ENSG00000164056.11	3711.388266	0.035403975	0.066083369	0.535747123	0.592133331	0.772579843	SPRY1	protein_coding
ENSG00000164061.5	192.6552087	0.024572132	0.152081831	0.161571778	0.871643083	0.938046208	BSN	protein_coding
ENSG00000164062.13	5754.51465	0.029805948	0.059202683	0.50345604	0.61464368	0.788582476	APEH	protein_coding
ENSG00000164066.13	634.645764	0.121450072	0.080958515	1.500151933	0.133575051	0.328154245	INTU	protein_coding
ENSG00000164068.16	1989.733239	0.117255522	0.053340532	2.198244353	0.027931698	0.121475008	RNF123	protein_coding
ENSG00000164070.12	1277.596504	0.065163968	0.136174634	0.478532352	0.632271354	0.800335565	HSPA4L	protein_coding
ENSG00000164073.11	909.131819	-0.065903768	0.048811163	-1.350178182	0.176958835	0.389226982	MFSD8	protein_coding
ENSG00000164074.15	1113.193239	-0.069922297	0.04398687	-1.589617476	0.111921055	0.294495971	ABHD18	protein_coding
ENSG00000164076.17	29.67412502	0.1915334	0.230417615	0.831244608	0.405835459	0.627375636	CAMKV	protein_coding
ENSG00000164077.14	989.2782258	-0.065400704	0.063599653	-1.028318566	0.3038	0.533216344	MON1A	protein_coding
ENSG00000164078.13	4550.984175	0.320369734	0.114096126	2.807893185	0.004986676	0.037157515	MST1R	protein_coding
ENSG00000164080.13	2002.414176	-0.032812379	0.046904568	-0.699556143	0.48420454	0.692149146	RAD54L2	protein_coding
ENSG00000164081.13	2754.343304	-0.057408403	0.061028902	-0.940675651	0.3468711	0.576183653	TEX264	protein_coding
ENSG00000164082.15	56.02508791	0.249145038	0.103934727	2.397129873	0.016524066	0.085651149	GRM2	protein_coding
ENSG00000164086.10	2042.422172	0.425991388	0.071812844	5.931966537	2.99328E-09	4.53293E-07	DUSP7	protein_coding
ENSG00000164087.8	753.6082516	0.003183377	0.084063859	0.037868558	0.969792482	0.985924016	POC1A	protein_coding
ENSG00000164088.18	761.737534	0.039467729	0.071083717	0.555228837	0.578738147	0.763053227	PPM1M	protein_coding
ENSG00000164089.9	18.73733159	-0.221122533	0.274920528	-0.804314377	0.421215437	0.640839789	ETNPPL	protein_coding
ENSG00000164091.12	6730.230282	0.030991258	0.039672106	0.781185094	0.434693639	0.652414939	WDR82	protein_coding
ENSG00000164093.17	135.8146772	0.309648041	0.235853039	1.312885523	0.189221526	0.404796585	PITX2	protein_coding
ENSG00000164096.13	4704.754166	-0.005882811	0.060882902	-0.09662501	0.923024194	0.963693165	C4orf3	protein_coding
ENSG00000164099.3	574.1042969	0.512396768	0.147040968	3.484721124	0.00049265	0.006727669	PRSS12	protein_coding
ENSG00000164100.9	13.61867966	0.50553659	0.152931685	3.305636694	0.000947609	0.010998871	NDST3	protein_coding
ENSG00000164104.12	6295.789212	-0.161410579	0.075685558	-2.132647001	0.032953695	0.135435551	HMGB2	protein_coding
ENSG00000164105.4	808.9210004	-0.085063036	0.071583569	-1.188303927	0.234713691	0.459422293	SAP30	protein_coding
ENSG00000164106.8	356.9624231	-0.230470623	0.237627715	-0.969881072	0.332105776	0.56165535	SCRG1	protein_coding
ENSG00000164107.9	834.2882555	-0.364276215	0.261867702	-1.391069658	0.164204305	0.371638672	HAND2	protein_coding
ENSG00000164109.14	2140.966714	-0.03178189	0.094459268	-0.336461325	0.736523011	0.863361487	MAD2L1	protein_coding
ENSG00000164111.15	18665.72171	0.280674153	0.07511241	3.736721434	0.000186435	0.003232334	ANXA5	protein_coding
ENSG00000164112.13	15.67375615	-0.538027276	0.157871188	-3.408014364	0.000654374	0.008311186	TMEM155	protein_coding
ENSG00000164113.11	4.797800144	-0.540333351	0.356623393	-1.515137148	0.129737676	0.322306522	ADAD1	protein_coding
ENSG00000164114.19	574.8664116	-0.011966815	0.124811569	-0.095879056	0.923616629	0.963937521	MAP9	protein_coding
ENSG00000164116.17	3039.820778	-0.079484254	0.139992355	-0.567775676	0.570187298	0.756701544	GUCY1A1	protein_coding
ENSG00000164117.14	1004.639277	0.028297587	0.049171536	0.575487152	0.564961877	0.753507291	FBXO8	protein_coding
ENSG00000164118.13	722.4268165	-0.018330643	0.057595944	-0.318262731	0.750285652	0.871825446	CEP44	protein_coding
ENSG00000164120.14	3828.592926	-0.199496786	0.166300479	-1.199616428	0.230288343	0.454125945	HPGD	protein_coding
ENSG00000164122.9	156.403679	-0.354069766	0.32738806	-1.081498712	0.279475333	0.50810199	ASB5	protein_coding

ENSG00000164123.7	4.728087792	-0.200646756	0.172768215	-1.161363832	0.245493971	0.471979651	C4orf45	protein_coding
ENSG00000164124.11	1488.585073	-0.261247639	0.094969961	-2.75084498	0.005944177	0.042056519	TMEM144	protein_coding
ENSG00000164125.16	4063.502682	0.013671933	0.099968125	0.13676292	0.891218194	0.948136403	GASK1B	protein_coding
ENSG00000164128.7	83.95215773	0.510033318	0.201623305	2.52963475	0.011418132	0.066254053	NPY1R	protein_coding
ENSG00000164129.12	4.535507599	0.302816032	0.240156351	1.260912029	0.207340544	0.427240348	NPY5R	protein_coding
ENSG00000164134.13	4001.521629	0.079225989	0.05769609	1.373160439	0.169702475	0.379388887	NAA15	protein_coding
ENSG00000164136.17	356.9194868	-0.09243936	0.090322711	-1.0234343	0.306102549	0.535635847	IL15	protein_coding
ENSG00000164142.16	1360.310619	0.153249505	0.098873916	1.549948777	0.121153811	0.309340811	FAM160A1	protein_coding
ENSG00000164144.16	2671.82789	-0.006638866	0.046972899	-0.141333957	0.887606125	0.946280418	ARFIP1	protein_coding
ENSG00000164151.12	3116.035064	0.138210617	0.057672132	2.396488798	0.016552999	0.085739933	ICE1	protein_coding
ENSG00000164161.10	438.2591403	-0.249606118	0.185682784	-1.344260966	0.178864013	0.391676237	HHIP	protein_coding
ENSG00000164162.14	269.5393146	0.001808984	0.062147927	0.029107718	0.97677868	0.989496111	ANAPC10	protein_coding
ENSG00000164163.11	4559.071786	0.051988866	0.061311602	0.847944992	0.396468606	0.619755083	ABCE1	protein_coding
ENSG00000164164.17	4377.858011	0.201719829	0.056790592	3.551993802	0.000382324	0.005559068	OTUD4	protein_coding
ENSG00000164167.12	922.0757313	-0.159168437	0.060252435	-2.641693026	0.008249279	0.052869089	LSM6	protein_coding
ENSG00000164168.8	2438.607203	0.108353507	0.045757534	2.367992721	0.017884888	0.090006901	TMEM184C	protein_coding
ENSG00000164169.13	510.4216138	-0.009469691	0.049301323	-0.192077819	0.847681249	0.925358997	PRMT9	protein_coding
ENSG00000164171.11	6463.702264	0.374539432	0.104691919	3.577539078	0.000346844	0.005183278	ITGA2	protein_coding
ENSG00000164172.20	1838.563793	-0.144591333	0.055955997	-2.584018542	0.009765654	0.059450867	MOCS2	protein_coding
ENSG00000164175.15	13.23142941	-0.346746709	0.204750146	-1.693511408	0.090358165	0.258827172	SLC45A2	protein_coding
ENSG00000164176.13	2292.788002	0.145237084	0.102893519	1.411528008	0.158088983	0.363346182	EDIL3	protein_coding
ENSG00000164180.14	949.8463804	-0.113160768	0.05742233	-1.970675319	0.048761025	0.175101706	TMEM161B	protein_coding
ENSG00000164181.14	1580.921199	-0.174868603	0.113121183	-1.545851971	0.122140322	0.310839737	ELOVL7	protein_coding
ENSG00000164182.12	1023.554426	-0.132176093	0.060814063	-2.173446201	0.029746751	0.126899086	NDUFAF2	protein_coding
ENSG00000164185.6	17.18874649	0.2724361	0.13762748	1.979518197	0.047757693	0.172810768	ZNF474	protein_coding
ENSG00000164187.7	2273.867958	0.059270851	0.069556614	0.852123866	0.394145347	0.617656644	LMBRD2	protein_coding
ENSG00000164188.9	37.6967872	0.073419337	0.164296408	0.446871226	0.654968041	0.814554302	RANBP3L	protein_coding
ENSG00000164190.19	6627.502258	0.082604845	0.054680827	1.510672932	0.130871802	0.324034121	NIPBL	protein_coding
ENSG00000164197.12	241.6553295	-0.105191774	0.129234981	-0.813957434	0.415669337	0.636056699	RNF180	protein_coding
ENSG00000164199.18	161.7138985	0.461635155	0.204672175	2.255485663	0.024102861	0.110178368	ADGRV1	protein_coding
ENSG00000164209.17	2963.209804	-0.073102056	0.064490659	-1.133529378	0.256992011	0.484009694	SLC25A46	protein_coding
ENSG00000164211.13	1257.967596	0.202896721	0.100476267	2.019349717	0.043450884	0.162517428	STARD4	protein_coding
ENSG00000164219.10	1707.148419	-0.031409828	0.053623111	-0.585751687	0.558042395	0.748284847	PGGT1B	protein_coding
ENSG00000164220.7	1301.021487	-0.047355131	0.125653121	-0.376871901	0.706268804	0.846116968	F2RL2	protein_coding
ENSG00000164221.13	718.22721	-0.167560398	0.068666524	-2.440205023	0.014678928	0.078922105	CCDC112	protein_coding
ENSG00000164236.12	308.62738	-0.047082468	0.131818186	-0.35717733	0.720959056	0.854272885	ANKRD33B	protein_coding
ENSG00000164237.9	4055.908779	-0.332940205	0.160836282	-2.070056584	0.038447046	0.15008043	CMBL	protein_coding
ENSG00000164241.13	330.2935178	0.08255159	0.095046761	0.868536589	0.385100651	0.609827242	C5orf63	protein_coding
ENSG00000164244.21	5071.280311	0.168895068	0.052827268	3.197119082	0.001388076	0.014756817	PRRC1	protein_coding
ENSG00000164251.5	4913.847085	0.133057903	0.112985124	1.177658604	0.238932736	0.464322683	F2RL1	protein_coding
ENSG00000164252.13	1006.012035	-0.069454984	0.042672346	-1.627634539	0.103602401	0.281358708	AGGF1	protein_coding
ENSG00000164253.14	1571.712803	0.095662812	0.049067562	1.949614119	0.051222129	0.180678868	WDR41	protein_coding

ENSG00000164256.11	5.528317742	0.135232108	0.328346649	0.411857737	0.6804437	0.830789137	PRDM9	protein_coding
ENSG00000164258.12	1730.820934	-0.039868979	0.059459098	-0.670527813	0.502521383	0.7072433	NDUFS4	protein_coding
ENSG00000164265.9	5.809451717	-0.895422126	0.256600808	-3.489553026	0.000483829	0.006630978	SCGB3A2	protein_coding
ENSG00000164266.11	19747.17286	-0.495482815	0.189420864	-2.615777399	0.008902457	0.055678508	SPINK1	protein_coding
ENSG00000164270.18	19.57158592	-1.449349474	0.237937451	-6.09130452	1.11994E-09	1.96789E-07	HTR4	protein_coding
ENSG00000164283.13	536.9474985	0.082923053	0.141303434	0.586843861	0.557308576	0.747751679	ESM1	protein_coding
ENSG00000164284.15	1211.643039	0.104542103	0.061013035	1.713438824	0.086631839	0.252147719	GRPEL2	protein_coding
ENSG00000164287.13	12.77618663	-0.024935173	0.200042281	-0.124649515	0.900801027	0.952748874	CDC20B	protein_coding
ENSG00000164291.17	350.4787867	-0.041752149	0.053048997	-0.787048785	0.431253308	0.649199513	ARSK	protein_coding
ENSG00000164292.13	2871.659762	0.219778221	0.11684042	1.881011903	0.0599703	0.19987195	RHOBTB3	protein_coding
ENSG00000164294.14	1484.52358	0.771931046	0.098995843	7.797610695	6.30903E-15	5.84954E-12	GPX8	protein_coding
ENSG00000164296.7	228.686036	-0.029893776	0.045847189	-0.652030733	0.514381346	0.716305471	TIGD6	protein_coding
ENSG00000164299.7	2.202987325	-0.291044587	0.244470314	-1.190510954	0.233845627	0.458464521	SPZ1	protein_coding
ENSG00000164300.17	6363.558231	-0.005228167	0.071565985	-0.073053803	0.941763304	0.972447265	SERINC5	protein_coding
ENSG00000164303.11	45.61881962	-0.18660011	0.16782931	-1.111844587	0.266204978	0.494181405	ENPP6	protein_coding
ENSG00000164304.16	10.06676545	0.211399269	0.151907144	1.391634809	0.164033012	0.371366718	CAGE1	protein_coding
ENSG00000164305.19	2981.1419	0.069039865	0.065488573	1.054227662	0.291778698	0.521595604	CASP3	protein_coding
ENSG00000164306.11	315.4185843	-0.050019522	0.049994676	-1.000496971	0.317070063	0.546532976	PRIMPOL	protein_coding
ENSG00000164307.13	5457.797454	-0.104814881	0.074352223	-1.409707428	0.15862609	0.364158105	ERAP1	protein_coding
ENSG00000164308.17	4572.459169	-0.221855347	0.151733975	-1.462133629	0.143704595	0.342965942	ERAP2	protein_coding
ENSG00000164309.15	201.9951519	0.045573168	0.130422373	0.34942753	0.726768369	0.857361493	CMYA5	protein_coding
ENSG00000164318.18	306.7998494	0.187054996	0.105840048	1.767336656	0.077171865	0.234667316	EGFLAM	protein_coding
ENSG00000164323.14	2493.121899	-0.024484666	0.061053403	-0.401036874	0.688392976	0.835445715	CFAP97	protein_coding
ENSG00000164325.8	0.640864782	-0.296942692	0.419052492	-0.708605002	0.478569631		TMEM174	protein_coding
ENSG00000164326.5	56.60780003	-0.414280639	0.340106347	-1.218091465	0.223189217	0.44579532	CARTPT	protein_coding
ENSG00000164327.13	3043.438785	0.169776808	0.06947091	2.443854676	0.014531279	0.078349073	RICTOR	protein_coding
ENSG00000164329.13	2719.077462	-0.148837902	0.04401686	-3.381383912	0.000721217	0.008951431	TENT2	protein_coding
ENSG00000164330.17	850.21727	-0.135681585	0.109076452	-1.243912707	0.213531692	0.434268472	EBF1	protein_coding
ENSG00000164331.10	764.0523476	-0.07202639	0.049953071	-1.441881104	0.149335918	0.351344956	ANKRA2	protein_coding
ENSG00000164332.8	1657.226202	0.023936723	0.048497935	0.49356169	0.621615762	0.793071498	UBLCP1	protein_coding
ENSG00000164334.15	2.306342746	-0.487671891	0.281832855	-1.730358549	0.083566235	0.246885818	FAM170A	protein_coding
ENSG00000164338.10	997.0762149	-0.060724651	0.049387068	-1.229565815	0.218859738	0.440496415	UTP15	protein_coding
ENSG00000164342.13	982.2222376	0.036707753	0.111053239	0.330541934	0.740990513	0.866119398	TLR3	protein_coding
ENSG00000164344.16	30.09912384	-0.29703459	0.14391284	-2.063989496	0.039018712	0.151593076	KLKB1	protein_coding
ENSG00000164346.10	4927.641359	-0.154256592	0.064503157	-2.391458021	0.016781603	0.086508025	NSA2	protein_coding
ENSG00000164347.18	1285.996156	-0.109180586	0.0605064	-1.80444692	0.071161273	0.222038866	GFM2	protein_coding
ENSG00000164362.21	147.4070525	-0.430442698	0.18276986	-2.355107662	0.01851734	0.092210046	TERT	protein_coding
ENSG00000164363.10	5.510875062	0.186579693	0.266794207	0.699339373	0.484339967	0.692252377	SLC6A18	protein_coding
ENSG00000164366.4	1014.721593	0.003841244	0.048529582	0.079152628	0.936911224	0.970057508	CCDC127	protein_coding
ENSG00000164379.7	8733.643706	-0.288763198	0.134687578	-2.143948254	0.032037033	0.133099227	FOXQ1	protein_coding
ENSG00000164385.9	9.649956865	0.228721164	0.162257075	1.409622131	0.158651289	0.364196766	LINC01600	lncRNA
ENSG00000164393.8	32.52714094	-0.285545596	0.234854038	-1.21584282	0.224044804	0.446848845	ADGRF2	protein_coding

ENSG00000164398.15	92.36172623	-0.756293765	0.175027239	-4.321006087	1.55319E-05	0.00045825	ACSL6	protein_coding
ENSG00000164399.5	0.779491109	0.900927475	0.363443861	2.47886282	0.013180199		IL3	protein_coding
ENSG00000164400.6	48.64848349	0.595288085	0.210835993	2.823465175	0.004750759	0.035922924	CSF2	protein_coding
ENSG00000164402.14	3984.691483	0.034815552	0.046047867	0.756073062	0.449605392	0.664421262	SEPTIN8	protein_coding
ENSG00000164403.15	1674.237256	-0.050688672	0.088768564	-0.571020526	0.567985729	0.755413219	SHROOM1	protein_coding
ENSG00000164404.8	33.88440113	-0.317460633	0.115952729	-2.737845299	0.006184316	0.043181211	GDF9	protein_coding
ENSG00000164405.11	4216.574079	-0.215599106	0.076414011	-2.821460377	0.004780554	0.036110628	UQCRQ	protein_coding
ENSG00000164406.8	81.02345096	-0.929365621	0.123542821	-7.522619377	5.36896E-14	3.96547E-11	LEAP2	protein_coding
ENSG00000164411.12	45.06032393	0.711118999	0.239163064	2.973364643	0.002945543	0.025292202	GJB7	protein_coding
ENSG00000164414.18	605.8297582	0.108115024	0.076308998	1.416805705	0.156539741	0.361402921	SLC35A1	protein_coding
ENSG00000164418.21	33.83870217	0.529480706	0.166065056	3.188393262	0.001430658	0.015030158	GRIK2	protein_coding
ENSG00000164430.16	1159.920514	-0.141059572	0.081120135	-1.738897151	0.082052855	0.244028638	CGAS	protein_coding
ENSG00000164434.12	14.56352115	-1.247548301	0.254664083	-4.898799562	9.64239E-07	5.05032E-05	FABP7	protein_coding
ENSG00000164438.6	2.550540597	0.609907404	0.340864738	1.789294509	0.073567398	0.227091202	TLX3	protein_coding
ENSG00000164440.15	86.02727379	-0.195848623	0.142938192	-1.370163	0.170636025	0.380757135	TXLNB	protein_coding
ENSG00000164442.10	2825.168501	0.01668894	0.083255542	0.200454405	0.841125214	0.921982055	CITED2	protein_coding
ENSG00000164451.13	1.543209423	-0.05329651	0.314369946	-0.169534366	0.865376343	0.934687682	CALHM4	protein_coding
ENSG00000164458.10	4.481423472	1.040121211	0.287593477	3.616637002	0.000298455	0.004626749	TBXT	protein_coding
ENSG00000164463.12	2043.363866	-0.047983401	0.066941989	-0.716790789	0.473503196	0.684327699	CREBRF	protein_coding
ENSG00000164465.19	1662.023525	0.419899186	0.062069555	6.76497823	1.33329E-11	5.05225E-09	DCBLD1	protein_coding
ENSG00000164466.13	3193.067164	0.109678194	0.063858513	1.717518749	0.085884432	0.250877187	SFXN1	protein_coding
ENSG00000164483.17	92.17360895	-0.358027945	0.133547677	-2.680899846	0.007342449	0.048662217	SAMD3	protein_coding
ENSG00000164484.12	1074.908334	0.24858694	0.14220992	1.74802813	0.080459156	0.240900591	TMEM200A	protein_coding
ENSG00000164485.15	29.51980422	-0.531142951	0.180259163	-2.946551752	0.003213386	0.027058887	IL22RA2	protein_coding
ENSG00000164488.12	341.4838801	0.027430158	0.277599216	0.098812088	0.921287471	0.962915133	DACT2	protein_coding
ENSG00000164494.12	1361.595415	0.101336163	0.059017817	1.717043563	0.085971213	0.250996083	PDSS2	protein_coding
ENSG00000164500.7	1.845892695	-0.790098572	0.399381262	-1.978306563	0.047894133	0.173165243	SPATA48	protein_coding
ENSG00000164506.14	2221.297512	0.175279246	0.067065899	2.613537571	0.008961023	0.055904725	STXBP5	protein_coding
ENSG00000164508.5	0.236322056	-0.402846681	0.677539171	-0.594573271	0.552128751		H2AC1	protein_coding
ENSG00000164509.15	47.01127449	-0.637492326	0.175580477	-3.630769976	0.000282577	0.004445435	IL31RA	protein_coding
ENSG00000164512.18	23.11956567	-0.54846898	0.140555622	-3.902148991	9.53424E-05	0.001905843	ANKRD55	protein_coding
ENSG00000164520.11	16.44026106	0.801759442	0.154009507	5.205908756	1.93049E-07	1.3882E-05	RAET1E	protein_coding
ENSG00000164530.15	417.9084222	0.028724543	0.236567044	0.121422419	0.90335646	0.954055276	PI16	protein_coding
ENSG00000164532.11	8.871062399	0.113222561	0.313259973	0.361433222	0.717775617	0.852414783	TBX20	protein_coding
ENSG00000164535.15	1464.880333	-0.007854965	0.044036927	-0.17837223	0.858430657	0.931098983	DAGLB	protein_coding
ENSG00000164542.13	815.3894963	0.056325786	0.089967458	0.626068437	0.531270053	0.728922202	KIAA0895	protein_coding
ENSG00000164543.7	1986.595008	-0.162793316	0.054979478	-2.960983306	0.003066586	0.026120524	STK17A	protein_coding
ENSG00000164548.11	3423.520818	-0.161375167	0.044952651	-3.589892116	0.000330815	0.005001732	TRA2A	protein_coding
ENSG00000164556.7	4.208689761	-0.034334868	0.19449647	-0.176532088	0.859875942	0.931893212	FAM183BP	processed_pseudogene
ENSG00000164574.16	5098.123212	0.065329194	0.071908248	0.908507655	0.363610071	0.590970426	GALNT10	protein_coding
ENSG00000164576.12	2077.008642	0.016959658	0.049543571	0.342318048	0.732111555	0.860958534	SAP30L	protein_coding
ENSG00000164587.13	39901.71995	-0.155477424	0.06553006	-2.372612271	0.017662794	0.089387206	RPS14	protein_coding

ENSG00000164588.8	17.20848627	-0.025181471	0.23678187	-0.106348813	0.915305604	0.95992436	HCN1	protein_coding
ENSG00000164591.14	57.30585333	0.001091185	0.1606219	0.006793499	0.994579614	0.997556739	MYOZ3	protein_coding
ENSG00000164597.15	2802.557786	-0.028221889	0.051220548	-0.550987634	0.581642151	0.765238211	COG5	protein_coding
ENSG00000164600.7	0.762742219	-0.510369092	0.453939554	-1.124310687	0.260881246		NEUROD6	protein_coding
ENSG00000164603.12	628.951948	0.066384836	0.053513578	1.240523213	0.214781934	0.435864447	BMT2	protein_coding
ENSG00000164604.13	80.35499513	0.165301123	0.086124943	1.919317641	0.054944147	0.189048644	GPR85	protein_coding
ENSG00000164609.10	2868.667798	-0.033388885	0.044347318	-0.752895238	0.45151287	0.66618439	SLU7	protein_coding
ENSG00000164610.9	863.4105669	-0.221919202	0.06770634	-3.277672378	0.001046668	0.011874682	RP9	protein_coding
ENSG00000164611.13	2156.233295	-0.087537842	0.095956451	-0.91226635	0.361628524	0.589391385	PTTG1	protein_coding
ENSG00000164615.6	1751.880844	-0.133671828	0.051126845	-2.614513501	0.008935463	0.05583319	CAMLG	protein_coding
ENSG00000164616.16	3.524742207	0.174373977	0.370874387	0.47016991	0.63823363	0.804172529	FBXL21P	transcribed_unitary_pseudogene
ENSG00000164619.10	117.0552807	-0.070338914	0.169441786	-0.415121413	0.678053025	0.829354821	BMPER	protein_coding
ENSG00000164620.9	141.1128984	0.117880014	0.107739572	1.09411994	0.273902378	0.502462818	RELL2	protein_coding
ENSG00000164621.5	1.497873188	0.091260054	0.195482235	0.466845766	0.64061023	0.805462652	SMAD5-AS1	lncRNA
ENSG00000164626.9	2362.569094	-0.091401355	0.123991915	-0.737155768	0.461027629	0.673893371	KCNK5	protein_coding
ENSG00000164627.18	59.52029804	0.283360899	0.17423911	1.626276094	0.103890935	0.281773528	KIF6	protein_coding
ENSG00000164631.19	2145.301482	0.011430606	0.046701613	0.244758274	0.806643604	0.903947499	ZNF12	protein_coding
ENSG00000164638.10	957.4319624	-0.000554203	0.191610231	-0.002892346	0.997692245	0.998864405	SLC29A4	protein_coding
ENSG00000164645.3	0.319423989	-0.079152772	0.798537527	-0.09912217	0.92104127		TEX47	protein_coding
ENSG00000164647.9	1645.378438	-0.100694185	0.116277762	-0.865979725	0.386501281	0.611079251	STEAP1	protein_coding
ENSG00000164649.20	1276.683751	0.263475762	0.096996979	2.716329572	0.006601017	0.045185756	CDCA7L	protein_coding
ENSG00000164651.17	76.11294735	0.705382019	0.278263489	2.534942766	0.01124657	0.065576693	SP8	protein_coding
ENSG00000164654.16	1771.697271	-0.100611314	0.049079168	-2.049980041	0.040366379	0.154859202	MIOS	protein_coding
ENSG00000164659.15	633.0024177	0.244398079	0.157890812	1.547892984	0.121648064	0.310075906	ELAPOR2	protein_coding
ENSG00000164663.14	545.243025	0.126722948	0.069762453	1.816492129	0.069294914	0.218395308	USP49	protein_coding
ENSG00000164669.13	42.9030918	-0.323214506	0.151410714	-2.134687157	0.032786573	0.135067166	INTS4P1	transcribed_unprocessed_pseudogene
ENSG00000164674.16	280.8358034	-0.123666889	0.086971889	-1.421917943	0.155050074	0.359336992	SYTL3	protein_coding
ENSG00000164675.11	30.90726045	0.040898968	0.119681558	0.341731578	0.732552906	0.861000593	IQUB	protein_coding
ENSG00000164683.18	384.1788246	0.10628537	0.096510806	1.101279478	0.27077504	0.498949572	HEY1	protein_coding
ENSG00000164684.13	2406.668087	-0.074731564	0.127857843	-0.584489476	0.558891044	0.748866368	ZNF704	protein_coding
ENSG00000164687.11	2041.534187	0.119495068	0.131045772	0.911857485	0.361843745	0.589497945	FABP5	protein_coding
ENSG00000164690.8	832.1900023	0.08755997	0.158518008	0.552366071	0.580697571	0.764460374	SHH	protein_coding
ENSG00000164691.18	562.4034923	-0.417339517	0.121790212	-3.42670818	0.000610945	0.007907087	TAGAP	protein_coding
ENSG00000164692.18	131275.479	0.79837235	0.12041852	6.629979773	3.35733E-11	1.0679E-08	COL1A2	protein_coding
ENSG00000164694.17	4967.159844	0.99137731	0.172977911	5.731236449	9.97012E-09	1.18707E-06	FNDC1	protein_coding
ENSG00000164695.5	1419.82541	-0.189876932	0.095476842	-1.988722374	0.046731855	0.170278251	CHMP4C	protein_coding
ENSG00000164707.15	104.4666444	0.073863078	0.112678914	0.655518193	0.512134177	0.714767721	SLC13A4	protein_coding
ENSG00000164708.6	0						PGAM2	protein_coding
ENSG00000164713.10	6723.099949	-0.124000547	0.08675059	-1.429391394	0.152891771	0.356238276	BRI3	protein_coding
ENSG00000164715.6	6105.390389	0.007634319	0.08968539	0.085123331	0.932163342	0.967941304	LMTK2	protein_coding
ENSG00000164729.7	8.510964516	-0.55103534	0.227777643	-2.419180976	0.015555499	0.082115321	SLC35G3	protein_coding
ENSG00000164733.22	78080.82473	0.175643943	0.094592135	1.856855687	0.063331689	0.206665044	CTSB	protein_coding

ENSG00000164736.6	263.7235143	-0.007402516	0.109452503	-0.067632219	0.946078407	0.974726911	SOX17	protein_coding
ENSG00000164740.5	0.211331323	-0.311588552	0.495802575	-0.628452872	0.52970731		SEPTIN7P5	processed_pseudogene
ENSG00000164741.15	2762.00788	0.155451999	0.100129283	1.552512859	0.120539558	0.308386798	DLC1	protein_coding
ENSG00000164742.16	393.4908995	0.001535843	0.142513588	0.010776815	0.991401512	0.996078919	ADCY1	protein_coding
ENSG00000164743.5	21.28999086	0.284145395	0.118761402	2.392573599	0.016730672	0.086367781	C8orf48	protein_coding
ENSG00000164744.13	13.25623042	0.336880988	0.210059853	1.603738096	0.108771795	0.289235202	SUN3	protein_coding
ENSG00000164746.14	31.01638899	-0.127726239	0.17440239	-0.732365188	0.4639457	0.676143333	C7orf57	protein_coding
ENSG00000164749.13	3227.645848	-0.171461819	0.12189125	-1.40667865	0.159522699	0.365321113	HNF4G	protein_coding
ENSG00000164751.15	2492.322683	-0.06694907	0.054847117	-1.220648839	0.222219006	0.444795262	PEX2	protein_coding
ENSG00000164754.15	14389.9643	0.054476054	0.060568548	0.899411601	0.368433462	0.595345229	RAD21	protein_coding
ENSG00000164756.12	22.57751824	-0.546152404	0.230166523	-2.372857685	0.017651063	0.089356458	SLC30A8	protein_coding
ENSG00000164758.7	830.7496459	-0.145424267	0.063040421	-2.306841604	0.021063652	0.10062136	MED30	protein_coding
ENSG00000164761.9	881.5544222	0.994975139	0.181983376	5.467395758	4.56696E-08	4.26825E-06	TNFRSF11B	protein_coding
ENSG00000164764.11	621.1799131	0.260239822	0.176382745	1.47542676	0.140097845	0.337556331	SBSPON	protein_coding
ENSG00000164776.10	157.7672468	-0.108155612	0.102913885	-1.050933133	0.293289302	0.523261736	PHKG1	protein_coding
ENSG00000164778.4	130.4532705	0.348510445	0.288758883	1.20692545	0.227460855	0.45085566	EN2	protein_coding
ENSG00000164794.9	15.74491949	0.329772425	0.33773866	0.976413017	0.328859833	0.55831093	KCNV1	protein_coding
ENSG00000164796.18	21.34320494	-0.034180054	0.273322355	-0.125054	0.9004808	0.952509344	CSMD3	protein_coding
ENSG00000164808.17	2932.88042	-0.083140078	0.046647093	-1.782320656	0.074696956	0.229585926	SPIDR	protein_coding
ENSG00000164815.11	1179.98006	-0.091372375	0.063689528	-1.434653026	0.151385996	0.353823485	ORC5	protein_coding
ENSG00000164816.8	105.2110359	0.47291409	0.445831364	1.060746568	0.288805091	0.518530733	DEFA5	protein_coding
ENSG00000164818.16	3717.650957	-0.005264697	0.05551251	-0.094838029	0.924443481	0.96429705	DNAAF5	protein_coding
ENSG00000164821.5	1.974921738	-0.274027516	0.304791218	-0.89906631	0.368617342	0.595467881	DEFA4	protein_coding
ENSG00000164822.5	77.07036249	0.387071181	0.42074252	0.919971627	0.357587586	0.585358408	DEFA6	protein_coding
ENSG00000164823.11	1702.274705	0.054126535	0.055001879	0.984085206	0.32507362	0.554840214	OSGIN2	protein_coding
ENSG00000164825.4	328.6173122	-0.530228682	0.215823092	-2.456774557	0.014019063	0.076459163	DEFB1	protein_coding
ENSG00000164828.18	7887.468944	0.186208708	0.056743009	3.281614997	0.001032144	0.011755816	SUN1	protein_coding
ENSG00000164830.18	2893.300451	0.000325256	0.070374597	0.004621788	0.99631236	0.998266626	OXR1	protein_coding
ENSG00000164841.5	31.78295468	0.321878793	0.159922068	2.012722808	0.044143799	0.164288522	TMEM74	protein_coding
ENSG00000164845.16	40.57254012	0.083916306	0.09889018	0.848580784	0.396114603	0.619431842	FAM86FP	transcribed_unprocessed_pseudogene
ENSG00000164849.10	54.69787143	0.190599055	0.10047913	1.896901915	0.057840885	0.195314399	GPR146	protein_coding
ENSG00000164850.15	255.3703481	-0.345589902	0.167213938	-2.066752958	0.038757438	0.151013312	GPER1	protein_coding
ENSG00000164853.9	1.417693916	0.600792575	0.356473632	1.685377323	0.091915796	0.261484178	UNCX	protein_coding
ENSG00000164855.16	2193.897005	0.192513544	0.114774774	1.677315824	0.09348074	0.264279888	TMEM184A	protein_coding
ENSG00000164867.11	1182.99304	0.029110688	0.09626301	0.302407833	0.762341184	0.878594636	NOS3	protein_coding
ENSG00000164871.17	1.4050065	-0.460751981	0.43568534	-1.057533818	0.290268029	0.520064547	SPAG11B	protein_coding
ENSG00000164877.19	3552.87646	0.181928188	0.079433281	2.290327006	0.022002367	0.10354181	MICALL2	protein_coding
ENSG00000164879.7	128.8603089	-0.392480729	0.145679505	-2.69413827	0.007057088	0.047253647	CA3	protein_coding
ENSG00000164880.16	12287.02172	0.126903719	0.059657503	2.127204676	0.033403081	0.136586849	INTS1	protein_coding
ENSG00000164885.13	901.4023874	-0.274056556	0.068586305	-3.995791223	6.44785E-05	0.001407001	CDK5	protein_coding
ENSG00000164889.15	6051.291511	-0.1607753	0.074407865	-2.160729919	0.030716208	0.12956347	SLC4A2	protein_coding
ENSG00000164893.9	1.864052127	-0.654111612	0.371133509	-1.762469829	0.07798995	0.236239121	SLC7A13	protein_coding

ENSG00000164896.20	3812.723198	-0.176144989	0.054674942	-3.221676776	0.001274428	0.013811429	FASTK	protein_coding
ENSG00000164897.14	3640.533561	-0.330238623	0.07070604	-4.670585775	3.00342E-06	0.000124293	TMUB1	protein_coding
ENSG00000164898.13	103.9713392	-0.202867396	0.095870451	-2.116057597	0.034339909	0.139034677	FMC1	protein_coding
ENSG00000164900.4	3.388360595	0.242107679	0.304708509	0.794555031	0.426872386	0.645470625	GBX1	protein_coding
ENSG00000164902.14	1478.157053	0.030550519	0.040038177	0.763034699	0.445442702	0.661203064	PHAX	protein_coding
ENSG00000164904.18	2372.302902	-0.068660425	0.073785287	-0.930543584	0.352089709	0.580712813	ALDH7A1	protein_coding
ENSG00000164916.11	6165.809772	0.055907412	0.070447304	0.793606125	0.427424765	0.645992058	FOXK1	protein_coding
ENSG00000164919.11	6390.416366	-0.192650651	0.088973521	-2.165258268	0.030367912	0.128592218	COX6C	protein_coding
ENSG00000164920.9	678.5895037	0.13524544	0.108162576	1.250390342	0.211156991	0.431598459	OSR2	protein_coding
ENSG00000164924.18	66987.6612	0.069735162	0.058826909	1.185429641	0.235847618	0.460731235	YWHAZ	protein_coding
ENSG00000164929.17	42.4302459	-0.20563234	0.131229183	-1.566971121	0.117121442	0.302790324	BAALC	protein_coding
ENSG00000164930.12	2459.299162	0.353556197	0.090086911	3.924612274	8.68695E-05	0.00178058	FZD6	protein_coding
ENSG00000164932.13	2771.878981	0.869148311	0.129638204	6.70441494	2.02215E-11	6.99359E-09	CTHRC1	protein_coding
ENSG00000164933.12	18.03728459	0.104211629	0.099351385	1.048919741	0.294215059	0.524210192	SLC25A32	protein_coding
ENSG00000164934.14	3396.255663	0.027036898	0.075135377	0.359842444	0.718964961	0.853050793	DCAF13	protein_coding
ENSG00000164935.6	48.09848438	-0.235783414	0.164350454	-1.434638046	0.151390267	0.353823485	DCSTAMP	protein_coding
ENSG00000164938.14	2249.285181	-0.170033243	0.081390544	-2.089103177	0.036698436	0.145210911	TP53INP1	protein_coding
ENSG00000164941.14	2951.632293	-0.118599084	0.063327305	-1.872795369	0.061096648	0.202049679	INTS8	protein_coding
ENSG00000164944.12	4198.756598	-0.038705477	0.04700435	-0.823444568	0.410255227	0.63114072	VIRMA	protein_coding
ENSG00000164946.20	381.9392778	-0.135675059	0.200648119	-0.676184059	0.498923792	0.70456874	FREM1	protein_coding
ENSG00000164949.8	2895.992284	0.102694102	0.117745109	0.872172975	0.38311402	0.608084489	GEM	protein_coding
ENSG00000164951.16	2880.43335	-0.091530474	0.080084818	-1.142919179	0.253072149	0.479901002	PDP1	protein_coding
ENSG00000164953.16	603.3228809	0.015256739	0.076684041	0.198955861	0.842297272	0.922473549	TMEM67	protein_coding
ENSG00000164961.16	4476.146717	0.142017169	0.059268219	2.396177433	0.016567068	0.085772026	WASHC5	protein_coding
ENSG00000164967.10	1009.759377	-0.101685944	0.07657868	-1.327862334	0.18422359	0.39833339	RPP25L	protein_coding
ENSG00000164970.15	1387.670996	0.116740421	0.064734745	1.803365735	0.071330795	0.22239265	FAM219A	protein_coding
ENSG00000164972.13	89.003519	-0.084565931	0.15943109	-0.53042309	0.59581862	0.775345299	C9orf24	protein_coding
ENSG00000164975.15	2156.945739	0.113622908	0.054047741	2.102269341	0.035529689	0.142122016	SNAPC3	protein_coding
ENSG00000164976.9	5124.645271	-0.208226105	0.093678914	-2.222763854	0.026231728	0.116595267	MYORG	protein_coding
ENSG00000164978.18	681.0327491	-0.080061358	0.073500663	-1.089260346	0.276039094	0.504845578	NUDT2	protein_coding
ENSG00000164983.7	1607.860536	0.153973376	0.078159178	1.96999738	0.048838671	0.175255115	TMEM65	protein_coding
ENSG00000164985.15	3762.162668	-0.126250787	0.06455975	-1.955564987	0.050516421	0.179102818	PSIP1	protein_coding
ENSG00000164989.17	208.0854018	-0.131738855	0.076663715	-1.718399046	0.085723854	0.250670796	CCDC171	protein_coding
ENSG00000165006.14	3757.78523	0.133144563	0.053143904	2.505359107	0.012232712	0.069413732	UBAP1	protein_coding
ENSG00000165023.7	23.9037663	-0.861882655	0.224451125	-3.839956937	0.000123056	0.00231938	DIRAS2	protein_coding
ENSG00000165025.15	4208.065472	-0.17006176	0.085167866	-1.996783146	0.045848745	0.168330199	SYK	protein_coding
ENSG00000165028.12	146.3296362	-0.163487939	0.116454187	-1.403882016	0.160353986	0.366359968	NIPSNAP3B	protein_coding
ENSG00000165029.17	3316.610502	0.163992113	0.089713041	1.827962914	0.067555118	0.215074464	ABCA1	protein_coding
ENSG00000165030.4	1542.300497	0.168647821	0.069350505	2.431818214	0.015023245	0.080209257	NFIL3	protein_coding
ENSG00000165046.13	165.7248375	0.151732624	0.091136368	1.664896534	0.0959334	0.268302386	LETM2	protein_coding
ENSG00000165055.16	1484.87025	-0.150486125	0.05269245	-2.855933361	0.004291052	0.033337701	METTL2B	protein_coding
ENSG00000165059.8	3.235315736	-0.074884763	0.168279913	-0.445001196	0.65631889	0.815319427	PRKACG	protein_coding

ENSG00000165060.15	586.803749	-0.249352539	0.061134422	-4.078758468	4.52768E-05	0.001060842	FXN	protein_coding
ENSG00000165061.15	5.603000268	0.001479849	0.242059405	0.006113578	0.995122101	0.997709272	ZMAT4	protein_coding
ENSG00000165066.13	583.1973855	-0.139703356	0.281424658	-0.496414769	0.619601807	0.791730181	NKX6-3	protein_coding
ENSG00000165071.15	139.3286083	0.084643455	0.122205316	0.69263317	0.488539789	0.695972355	TMEM71	protein_coding
ENSG00000165072.10	351.4203889	-0.491776296	0.210108959	-2.340577473	0.019253943	0.094500495	MAMDC2	protein_coding
ENSG00000165076.14	5.146058637	-0.311652903	0.194985197	-1.598341349	0.10996703	0.29108912	PRSS37	protein_coding
ENSG00000165078.13	77.72992345	-0.982855144	0.242904932	-4.04625438	5.20437E-05	0.001189254	CPA6	protein_coding
ENSG00000165084.16	43.5867133	-0.032054249	0.123939617	-0.258627946	0.795922319	0.897879624	C8orf34	protein_coding
ENSG00000165091.18	26.96055446	0.039889354	0.148793011	0.268086208	0.788632962	0.893633101	TMC1	protein_coding
ENSG00000165092.13	15651.92337	-0.675923782	0.169111279	-3.996917212	6.41727E-05	0.001401908	ALDH1A1	protein_coding
ENSG00000165097.16	2718.439926	-0.014027595	0.070551743	-0.198827046	0.842398039	0.922488342	KDM1B	protein_coding
ENSG00000165102.15	6382.940214	0.147180516	0.06037024	2.437964722	0.014770215	0.079256455	HGSNAT	protein_coding
ENSG00000165105.10	3442.15006	0.122695672	0.116006175	1.057665008	0.290208194	0.520034654	RASEF	protein_coding
ENSG00000165113.13	260.0226	-0.298449556	0.075850959	-3.934683997	8.33062E-05	0.001721307	GKAP1	protein_coding
ENSG00000165115.15	387.5943339	-0.096604982	0.063535361	-1.520491593	0.12838747	0.320083573	KIF27	protein_coding
ENSG00000165118.15	1291.835926	-0.150247657	0.057492942	-2.613323492	0.008966639	0.055923746	C9orf64	protein_coding
ENSG00000165119.21	25093.55913	-0.042978885	0.03355687	-1.280777526	0.20027182	0.418733572	HNRNPK	protein_coding
ENSG00000165120.5	3.117493325	-0.228062698	0.2951128	-0.772798394	0.439641704	0.656215269	SSMEM1	protein_coding
ENSG00000165121.11	202.9130792	-0.146783682	0.06954719	-2.110562378	0.034809944	0.140260096	AL353743.1	transcribed_unprocessed_pseudogene
ENSG00000165124.18	1557.431561	0.17471381	0.153256254	1.140010966	0.254281732	0.481078399	SVEP1	protein_coding
ENSG00000165125.22	232.993639	-0.314107309	0.217832503	-1.441967131	0.149311646	0.351330027	TRPV6	protein_coding
ENSG00000165131.8	1.973004567	-0.395578539	0.224951408	-1.758506616	0.078661347	0.237680316	LLCFC1	protein_coding
ENSG00000165138.18	2465.214427	0.020355875	0.065488904	0.310829372	0.755930339	0.874745775	ANKS6	protein_coding
ENSG00000165140.11	2852.963027	-0.468412574	0.1214598	-3.856523516	0.000115011	0.00220107	FBP1	protein_coding
ENSG00000165152.9	2559.300538	0.009467922	0.094498185	0.100191571	0.920192237	0.962327805	PGAP4	protein_coding
ENSG00000165156.15	2933.960696	-0.056103257	0.051033432	-1.09934322	0.271618387	0.499837262	ZHX1	protein_coding
ENSG00000165164.14	59.24394151	-1.246745867	0.242794726	-5.134979199	2.82175E-07	1.87159E-05	CFAP47	protein_coding
ENSG00000165168.8	3925.767941	-0.264563665	0.122076502	-2.167195662	0.030219938	0.128177364	CYBB	protein_coding
ENSG00000165169.11	2750.086993	0.008964978	0.071064069	0.126153459	0.899610451	0.952113582	DYNLT3	protein_coding
ENSG00000165171.11	372.1023012	0.155275876	0.148808539	1.043460789	0.296734932	0.526563429	METTL27	protein_coding
ENSG00000165175.16	4125.627996	-0.05313339	0.069631095	-0.763069861	0.445421733	0.661203064	MID1IP1	protein_coding
ENSG00000165178.9	227.1650127	-0.377767902	0.128075265	-2.949577359	0.003182089	0.026865885	NCF1C	unprocessed_pseudogene
ENSG00000165181.16	75.22935207	0.299843013	0.121583323	2.466152467	0.013657319	0.075182539	SHOC1	protein_coding
ENSG00000165182.12	7.265177625	-0.217803745	0.130439107	-1.669773355	0.094964214	0.266537135	CXorf58	protein_coding
ENSG00000165185.15	1208.627473	0.094530706	0.078462439	1.204789285	0.228284657	0.451830881	KIAA1958	protein_coding
ENSG00000165186.12	216.1750008	-0.4500467	0.229963095	-1.957038798	0.050342907	0.178775153	PTCHD1	protein_coding
ENSG00000165188.13	229.7434701	0.329909442	0.167455851	1.97012789	0.048823715	0.175225749	RNF183	protein_coding
ENSG00000165192.14	8.186982587	-0.315167867	0.212951819	-1.479996124	0.138874281	0.335852409	ASB11	protein_coding
ENSG00000165194.15	112.534318	0.414406624	0.164279473	2.52257094	0.011650045	0.067197089	PCDH19	protein_coding
ENSG00000165195.16	851.7083747	0.123581724	0.060198891	2.052890364	0.040083212	0.154152866	PIGA	protein_coding
ENSG00000165197.5	45.0551328	-0.041978553	0.164807125	-0.254713215	0.798944609	0.8996049	VEGFD	protein_coding
ENSG00000165202.3	3.332716488	0.19197697	0.243893203	0.787135381	0.43120262	0.64918005	OR1Q1	protein_coding

ENSG00000165204.3	3.265655004	0.05642162	0.256698066	0.219797604	0.826028785	0.914180479	OR1K1	protein_coding
ENSG00000165209.19	2497.880436	-0.101714941	0.055792549	-1.82309185	0.068289481	0.216537054	STRBP	protein_coding
ENSG00000165215.6	10225.57548	0.19012275	0.183590027	1.035583214	0.300396626	0.529975052	CLDN3	protein_coding
ENSG00000165219.22	3817.925778	0.097808982	0.044572501	2.194379479	0.02820814	0.122259272	GAPVD1	protein_coding
ENSG00000165233.18	1386.976232	0.160432318	0.060584407	2.648079354	0.008095052	0.052171764	CARD19	protein_coding
ENSG00000165238.16	4799.628681	-0.016065508	0.144021715	-0.111549205	0.911180849	0.958066302	WNK2	protein_coding
ENSG00000165240.20	1406.533358	0.031027855	0.062501931	0.496430353	0.619590814	0.791730181	ATP7A	protein_coding
ENSG00000165244.7	1034.819845	-0.147926413	0.080096722	-1.846847286	0.064769276	0.209473858	ZNF367	protein_coding
ENSG00000165246.14	267.9464671	0.056059017	0.261236904	0.214590728	0.830086423	0.916390345	NLGN4Y	protein_coding
ENSG00000165259.14	105.5878462	0.196376781	0.119646345	1.641310316	0.100733016	0.276736616	HDX	protein_coding
ENSG00000165264.11	2173.506627	-0.201620835	0.06583888	-3.062336946	0.002196161	0.02046666	NDUFB6	protein_coding
ENSG00000165269.13	162.3486253	0.04818791	0.147450549	0.326807259	0.743813669	0.868238198	AQP7	protein_coding
ENSG00000165271.17	4855.948681	0.125129376	0.069273852	1.806300243	0.070871454	0.221515231	NOL6	protein_coding
ENSG00000165272.16	3212.387489	1.452800365	0.178143335	8.155232759	3.48508E-16	5.06231E-13	AQP3	protein_coding
ENSG00000165275.10	458.0579838	-0.067556351	0.056945096	-1.186341866	0.23548732	0.460316284	TRMT10B	protein_coding
ENSG00000165280.17	20207.2378	-0.003930427	0.056377443	-0.069716305	0.944419464	0.973966796	VCP	protein_coding
ENSG00000165282.14	2997.625991	0.051180246	0.074132897	0.690385083	0.489952055	0.69720931	PIGO	protein_coding
ENSG00000165283.16	4888.702605	-0.104856162	0.075562341	-1.387677518	0.165235265	0.373191759	STOML2	protein_coding
ENSG00000165288.11	2212.903493	-0.010120836	0.061119104	-0.165592025	0.868478024	0.936261174	BRWD3	protein_coding
ENSG00000165300.7	139.4674847	-0.132718974	0.216046091	-0.61430861	0.539011414	0.734773372	SLITRK5	protein_coding
ENSG00000165304.8	1778.016411	0.024825874	0.093271536	0.266167737	0.790110029	0.894604015	MELK	protein_coding
ENSG00000165309.14	57.67033482	-0.138679506	0.194443364	-0.713212851	0.475714056	0.685953191	ARMC3	protein_coding
ENSG00000165312.6	1310.19469	0.074570754	0.061420557	1.214100912	0.224709192	0.447682186	OTUD1	protein_coding
ENSG00000165322.18	4546.934756	-0.081300044	0.070204083	-1.158052933	0.246842436	0.473281124	ARHGAP12	protein_coding
ENSG00000165323.15	179.8296799	-0.275555954	0.202424951	-1.361274644	0.173426913	0.3844648	FAT3	protein_coding
ENSG00000165325.14	15.84636161	0.488352803	0.191102836	2.555445087	0.010605209	0.062861679	DEUP1	protein_coding
ENSG00000165338.17	656.8947263	-0.016450094	0.090332958	-0.182105115	0.855500234	0.929424191	HECTD2	protein_coding
ENSG00000165349.12	12.10457686	0.407132274	0.224174985	1.816136063	0.069349503	0.218513614	SLC7A3	protein_coding
ENSG00000165355.7	1328.590534	0.018904234	0.045300168	0.417310463	0.676451336	0.82859443	FBXO33	protein_coding
ENSG00000165359.16	578.4731981	-0.123012523	0.09361018	-1.314093436	0.188814763	0.404184355	INTS6L	protein_coding
ENSG00000165370.3	2.319784684	0.188174199	0.312989312	0.601216052	0.547696094	0.741100464	GPR101	protein_coding
ENSG00000165376.11	6457.247058	-0.458947641	0.225652814	-2.033866242	0.041965083	0.158890245	CLDN2	protein_coding
ENSG00000165379.13	52.19077001	0.103453777	0.148815456	0.695181667	0.48694146	0.69444456	LRFN5	protein_coding
ENSG00000165383.11	8.75603248	-0.251626393	0.190912786	-1.318017499	0.18749779	0.402551668	LRRC18	protein_coding
ENSG00000165389.7	3673.155562	0.07461917	0.071658515	1.04131617	0.297728834	0.527663	SPTSSA	protein_coding
ENSG00000165392.11	1342.010484	0.012310414	0.062413665	0.197239077	0.843640452	0.923338248	WRN	protein_coding
ENSG00000165406.16	2449.5939	0.009195975	0.06267561	0.146723346	0.883350391	0.943960371	MARCHF8	protein_coding
ENSG00000165409.18	39.50624917	-0.551674288	0.131526089	-4.194409586	2.73583E-05	0.000710485	TSHR	protein_coding
ENSG00000165410.15	2532.918387	-0.476374783	0.144860573	-3.28850544	0.001007208	0.011553335	CFL2	protein_coding
ENSG00000165416.15	4501.409485	-0.009232799	0.052382571	-0.176257072	0.860091985	0.931943739	SUGT1	protein_coding
ENSG00000165417.12	3053.437827	0.006250296	0.040536794	0.154188213	0.877461335	0.940945236	GTF2A1	protein_coding
ENSG00000165424.7	2532.096493	0.129814149	0.136022964	0.954354655	0.339904151	0.569758172	ZCCHC24	protein_coding

ENSG00000165434.8	1903.861271	0.24368077	0.078822445	3.09151497	0.001991379	0.019063783	PGM2L1	protein_coding
ENSG00000165443.12	191.3967793	-0.520082068	0.229105223	-2.270057662	0.023204085	0.107502062	PHYHIPL	protein_coding
ENSG00000165449.11	264.6495271	-0.082622409	0.188840333	-0.437525221	0.66173049	0.818815616	SLC16A9	protein_coding
ENSG00000165457.14	653.6996764	0.20076729	0.146448458	1.370907501	0.170403793	0.380421288	FOLR2	protein_coding
ENSG00000165458.14	7809.468884	0.217734375	0.059540398	3.65691837	0.000255266	0.004110758	INPPL1	protein_coding
ENSG00000165462.5	10.69401071	-0.391640189	0.29691277	-1.319041243	0.187155325	0.402093753	PHOX2A	protein_coding
ENSG00000165471.7	2.063577855	-0.208666422	0.341228391	-0.611515419	0.540858414	0.73617492	MBL2	protein_coding
ENSG00000165474.8	3728.188247	0.698500536	0.122454522	5.704162841	1.16916E-08	1.36959E-06	GJB2	protein_coding
ENSG00000165475.15	2231.745269	-0.242802136	0.091224267	-2.661595906	0.007777118	0.050734172	CRYL1	protein_coding
ENSG00000165476.14	5169.642784	0.110537273	0.064833071	1.704951982	0.08820338	0.255103119	REEP3	protein_coding
ENSG00000165478.7	8.678302079	0.112478588	0.237900662	0.472796449	0.636358408	0.802914666	HEPACAM	protein_coding
ENSG00000165480.16	1379.673394	0.037848379	0.098024854	0.38611003	0.699415182	0.841805651	SKA3	protein_coding
ENSG00000165487.14	2439.538765	0.094841797	0.058442722	1.622816207	0.1046287	0.283069775	MICU2	protein_coding
ENSG00000165490.13	688.8328118	0.093930876	0.094851757	0.990291376	0.322031721	0.551835482	DDIAS	protein_coding
ENSG00000165494.11	2378.6133	-0.114557867	0.041341596	-2.771007376	0.005588316	0.040258233	PCF11	protein_coding
ENSG00000165495.16	138.723461	-0.217193323	0.150597846	-1.442207366	0.149243883	0.351227724	PKNOX2	protein_coding
ENSG00000165496.5	7.050733528	-0.17378954	0.123769656	-1.40413689	0.16027809	0.366291082	RPL10L	protein_coding
ENSG00000165501.17	584.8443437	-0.105115368	0.062047932	-1.694099454	0.090246386	0.258650294	LRR1	protein_coding
ENSG00000165502.7	8105.279635	-0.0943586	0.063375321	-1.488885558	0.136517507	0.33247672	RPL36AL	protein_coding
ENSG00000165506.15	917.5543582	0.023014349	0.04640674	0.495926863	0.619946013	0.791903547	DNAAF2	protein_coding
ENSG00000165507.9	5526.544934	0.191777891	0.124998542	1.53424103	0.124970367	0.315043023	DEPP1	protein_coding
ENSG00000165509.13	2.02299828	0.915578131	0.412425732	2.219983042	0.026419919	0.117144974	MAGEC3	protein_coding
ENSG00000165511.6	168.8926746	0.086785898	0.072154746	1.202774626	0.229063546	0.452550309	ZNF22-AS1	lncRNA
ENSG00000165512.5	834.5437149	-0.138698223	0.06254133	-2.217705048	0.02657495	0.117640856	ZNF22	protein_coding
ENSG00000165516.11	1766.895601	0.031419658	0.053776543	0.584263253	0.559043211	0.748868034	KLHDC2	protein_coding
ENSG00000165521.15	48.53658486	-0.012260908	0.146571801	-0.083651205	0.933333754	0.968507013	EML5	protein_coding
ENSG00000165525.18	2403.768483	0.010555314	0.046689086	0.226076697	0.821141769	0.912084392	NEMF	protein_coding
ENSG00000165526.9	1312.527777	-0.058199311	0.051217312	-1.136321065	0.255822218	0.482890948	RPUSD4	protein_coding
ENSG00000165527.7	9140.609163	0.081536623	0.05498365	1.482924892	0.138094367	0.334653445	ARF6	protein_coding
ENSG00000165533.19	862.219331	0.07550517	0.049245955	1.533225838	0.12522022	0.315453898	TTC8	protein_coding
ENSG00000165548.11	112.4129577	0.521469346	0.191122319	2.728458657	0.006363107	0.044048466	TMEM63C	protein_coding
ENSG00000165553.4	5.671285849	-0.311265437	0.356607398	-0.872851876	0.38274382	0.607881645	NGB	protein_coding
ENSG00000165555.9	34.61300577	-0.024685226	0.090174547	-0.273749377	0.784277229	0.8905915	NOXRED1	protein_coding
ENSG00000165556.10	2587.381168	-0.013163535	0.194639942	-0.067630186	0.946080025	0.974726911	CDX2	protein_coding
ENSG00000165566.12	8.123020431	-0.390894623	0.231196064	-1.690749471	0.090884661	0.259754764	AMER2	protein_coding
ENSG00000165568.18	152.134026	0.010204914	0.148646119	0.068652409	0.945266301	0.974279498	AKR1E2	protein_coding
ENSG00000165572.8	1012.785159	-0.020050638	0.067236414	-0.298211001	0.765542126	0.880769596	KBTBD6	protein_coding
ENSG00000165583.15	2.66663879	0.03418151	0.328771266	0.103967449	0.91719518	0.960737744	SSX5	protein_coding
ENSG00000165584.16	3.678542547	0.302478948	0.404276337	0.748198498	0.454340437	0.668561938	SSX3	protein_coding
ENSG00000165588.18	3.4563803	0.103217833	0.411321941	0.250941713	0.801859174	0.901442541	OTX2	protein_coding
ENSG00000165591.6	449.8452808	0.136043637	0.09584185	1.419459634	0.155765057	0.360380881	FAAH2	protein_coding
ENSG00000165606.9	66.39688212	0.725163751	0.269174385	2.694029564	0.00705939	0.047256751	DRGX	protein_coding

ENSG00000165609.13	3101.630566	-0.057768709	0.058382181	-0.989492147	0.32242241	0.552324145	NUDT5	protein_coding
ENSG00000165617.15	1000.216316	0.114699806	0.124098425	0.924264796	0.355348467	0.583277253	DACT1	protein_coding
ENSG00000165621.8	22.93362725	0.097894047	0.239973406	0.407937065	0.68331988	0.832692541	OXGR1	protein_coding
ENSG00000165623.10	1.829610776	-0.290312631	0.38213941	-0.759703456	0.447431863	0.662772263	UCMA	protein_coding
ENSG00000165626.17	1178.638964	-0.098693283	0.100902932	-0.978101243	0.328024253	0.55756574	BEND7	protein_coding
ENSG00000165629.20	12417.08891	-0.159399822	0.061243998	-2.602701125	0.009249255	0.057219587	ATP5F1C	protein_coding
ENSG00000165630.14	349.0730159	-0.026869955	0.041738262	-0.643772733	0.519722817	0.720057267	PRPF18	protein_coding
ENSG00000165632.8	1823.22935	-0.122583748	0.058411636	-2.098618642	0.035850532	0.143062645	TAF3	protein_coding
ENSG00000165633.13	955.4873801	0.179000704	0.126924191	1.410296191	0.158452243	0.363950737	VSTM4	protein_coding
ENSG00000165637.13	7784.42194	-0.078614644	0.071349997	-1.101817065	0.27054121	0.498725593	VDAC2	protein_coding
ENSG00000165643.11	11.88927205	1.284636809	0.347409739	3.697757045	0.000217513	0.00361088	SOHLH1	protein_coding
ENSG00000165644.11	1848.942393	-0.131109244	0.108065888	-1.21323432	0.225040246	0.448014016	COMTD1	protein_coding
ENSG00000165646.14	103.223842	-0.056506329	0.126403698	-0.447030663	0.654852921	0.814538041	SLC18A2	protein_coding
ENSG00000165650.12	7343.209124	0.007130726	0.081380758	0.08762177	0.930177299	0.966852785	PDZD8	protein_coding
ENSG00000165655.17	2207.449873	0.018338518	0.120643012	0.152006466	0.879181836	0.941676462	ZNF503	protein_coding
ENSG00000165660.8	1552.281093	-0.033312796	0.045697509	-0.728984947	0.46601087	0.677659802	ABRAXAS2	protein_coding
ENSG00000165661.17	1949.012728	0.044948234	0.071707086	0.626831133	0.530769933	0.728713339	QSOX2	protein_coding
ENSG00000165669.14	1361.189372	-0.074018037	0.042785167	-1.729992934	0.083631538	0.246961545	FAM204A	protein_coding
ENSG00000165671.21	6471.353877	0.091783693	0.050559108	1.815374049	0.069466446	0.218690936	NSD1	protein_coding
ENSG00000165672.7	9847.802124	-0.159026889	0.059722501	-2.662763402	0.007750189	0.050603831	PRDX3	protein_coding
ENSG00000165675.16	1153.059209	0.040292626	0.055759754	0.722611273	0.469918741	0.68124851	ENOX2	protein_coding
ENSG00000165678.21	9794.489478	-0.042160127	0.052246516	-0.806946179	0.419697492	0.639690349	GHITM	protein_coding
ENSG00000165682.14	6.462283978	-0.142173973	0.172995648	-0.821835546	0.4111705	0.632080181	CLEC1B	protein_coding
ENSG00000165684.4	1459.180499	0.044588998	0.059621888	0.747862903	0.454542856	0.668724309	SNAPC4	protein_coding
ENSG00000165685.8	74.70803973	0.070748216	0.122114633	0.579359035	0.562346939	0.751208783	TMEM52B	protein_coding
ENSG00000165688.12	2800.10315	-0.007723399	0.053889318	-0.143319668	0.886037723	0.945485613	PMPCA	protein_coding
ENSG00000165689.17	2126.30393	-0.067887321	0.053882843	-1.259906074	0.207703247	0.427600025	ENTR1	protein_coding
ENSG00000165694.10	1.782786758	0.683778084	0.291383092	2.346663565	0.01894235	0.0935355	FRMD7	protein_coding
ENSG00000165695.10	129.0768094	-0.166293427	0.156209512	-1.064553785	0.287077903	0.516685684	AK8	protein_coding
ENSG00000165698.16	327.9960897	-0.014570277	0.089553589	-0.162698975	0.870755454	0.937557067	SPACA9	protein_coding
ENSG00000165699.15	2484.518746	-0.023893268	0.051724395	-0.46193422	0.644128493	0.807762325	TSC1	protein_coding
ENSG00000165702.14	55.00295888	-0.625506776	0.183666344	-3.405669005	0.000660021	0.008358546	GFI1B	protein_coding
ENSG00000165704.15	2562.764924	-0.159648533	0.074139276	-2.15335974	0.031290417	0.131186024	HPRT1	protein_coding
ENSG00000165714.11	720.2984689	-0.170779763	0.056734784	-3.010142119	0.002611255	0.023189453	BORCS5	protein_coding
ENSG00000165716.11	314.3345988	-0.026962742	0.121872737	-0.221236859	0.824908011	0.913501271	DIPK1B	protein_coding
ENSG00000165724.6	1197.902281	0.014126734	0.067771835	0.208445506	0.834881128	0.919104047	ZMYND19	protein_coding
ENSG00000165730.16	273.7447934	-0.040063809	0.127382475	-0.314515867	0.753129267	0.873173897	STOX1	protein_coding
ENSG00000165731.20	344.0267479	0.357135817	0.194893026	1.832470994	0.066881279	0.213671958	RET	protein_coding
ENSG00000165732.13	10407.82455	0.01326612	0.062303512	0.212927328	0.831383646	0.916928385	DDX21	protein_coding
ENSG00000165733.8	3521.853878	0.081901944	0.045820735	1.787442817	0.073865947	0.227722416	BMS1	protein_coding
ENSG00000165752.17	701.7128506	0.094449164	0.103993159	0.908224781	0.363759474	0.590990477	STK32C	protein_coding
ENSG00000165757.9	2411.962659	0.274203935	0.095188389	2.880644765	0.003968627	0.031587368	JCAD	protein_coding

ENSG00000165762.3	15.71200647	-0.310429251	0.292591074	-1.060966237	0.288705245	0.51847964	OR4K2	protein_coding
ENSG00000165775.18	2591.89675	-0.124939078	0.053646135	-2.328948334	0.019861803	0.096749138	FUNDC2	protein_coding
ENSG00000165782.11	1632.804803	-0.006496653	0.040641047	-0.15985447	0.872995715	0.938607117	PIP4P1	protein_coding
ENSG00000165792.18	935.4197821	-0.074276882	0.051004771	-1.456273233	0.145317102	0.345511528	METTL17	protein_coding
ENSG00000165794.10	11.24440871	-0.259587589	0.279319607	-0.929356845	0.352704185	0.581154394	SLC39A2	protein_coding
ENSG00000165795.23	2819.059368	-0.237351524	0.097298789	-2.439408809	0.014711315	0.079037724	NDRG2	protein_coding
ENSG00000165799.5	17.52688336	0.768797491	0.277271471	2.772724826	0.005558911	0.040099428	RNASE7	protein_coding
ENSG00000165801.10	2737.707881	-0.037359533	0.084565422	-0.441782608	0.658646525	0.816821094	ARHGEF40	protein_coding
ENSG00000165802.23	6560.408929	0.406186181	0.103767261	3.914396285	9.06307E-05	0.001837791	NSMF	protein_coding
ENSG00000165804.16	2799.034477	-0.190597985	0.086078682	-2.214229817	0.026812974	0.118385805	ZNF219	protein_coding
ENSG00000165805.10	3.937044778	-0.240935266	0.223104343	-1.0799219	0.28017696	0.508995347	C12orf50	protein_coding
ENSG00000165806.21	3159.591011	-0.238386018	0.073923716	-3.224756976	0.001260796	0.013684432	CASP7	protein_coding
ENSG00000165807.8	158.9670655	-0.203282021	0.126524912	-1.6066561	0.108129829	0.288264624	PPP1R36	protein_coding
ENSG00000165810.17	636.6343713	-0.05338999	0.125604202	-0.425065313	0.670789064	0.824526928	BTNL9	protein_coding
ENSG00000165813.20	3184.311751	0.054879373	0.067269035	0.81581923	0.414603533	0.63493861	CCDC186	protein_coding
ENSG00000165816.13	1499.717189	0.320036568	0.184638424	1.733315099	0.083039679	0.245830994	VWA2	protein_coding
ENSG00000165819.12	1323.684883	-0.082804516	0.04775402	-1.733980018	0.082921629	0.24562425	METTL3	protein_coding
ENSG00000165821.12	357.8067268	0.044947084	0.161386523	0.278505808	0.780624107	0.88874039	SALL2	protein_coding
ENSG00000165828.15	62.91966919	-0.900860702	0.217429838	-4.143224829	3.42456E-05	0.000848874	PRAP1	protein_coding
ENSG00000165832.6	1747.688117	-0.122135102	0.050250738	-2.430513588	0.015077441	0.080349937	TRUB1	protein_coding
ENSG00000165837.12	7.044791477	-0.072054656	0.135339474	-0.53239941	0.594449394	0.77430688	ERICH6B	protein_coding
ENSG00000165841.11	120.7060202	-0.63789168	0.177655853	-3.590603228	0.000329914	0.004993624	CYP2C19	protein_coding
ENSG00000165861.14	1284.369927	0.140282284	0.048884392	2.869674315	0.004108948	0.032355551	ZFYVE1	protein_coding
ENSG00000165863.17	29.28829733	-0.398174118	0.259476238	-1.534530177	0.124899275	0.31488202	C10orf82	protein_coding
ENSG00000165868.16	778.3001411	-0.024350251	0.100897611	-0.241336254	0.809294514	0.905498786	HSPA12A	protein_coding
ENSG00000165874.13	7.3319922	-0.116653666	0.120247579	-0.970112383	0.331990477	0.56154753	SHLD2P1	transcribed_unprocessed_pseudogene
ENSG00000165879.9	408.0848745	-0.194161515	0.074678254	-2.599973952	0.009323084	0.057528528	FRAT1	protein_coding
ENSG00000165886.5	759.3740349	0.386644487	0.082519437	4.685495949	2.79283E-06	0.000117474	UBTD1	protein_coding
ENSG00000165887.12	10.93153487	-0.032135474	0.175732362	-0.182865998	0.854903162	0.929168335	ANKRD2	protein_coding
ENSG00000165891.16	732.9818848	0.038935052	0.107652508	0.361673429	0.717596085	0.852340945	E2F7	protein_coding
ENSG00000165895.19	1972.78545	0.193724298	0.084710682	2.286893374	0.022202047	0.104222624	ARHGAP42	protein_coding
ENSG00000165898.14	658.2414765	-0.089051225	0.052432974	-1.698382112	0.089435666	0.257250034	ISCA2	protein_coding
ENSG00000165899.11	45.45424091	0.100032935	0.160751802	0.622281891	0.533756527	0.730980546	OTOGL	protein_coding
ENSG00000165905.18	2041.941752	-0.134946987	0.158647095	-0.850611144	0.394985397	0.618501514	LARGE2	protein_coding
ENSG00000165912.16	1530.818978	0.048342854	0.114969426	0.420484436	0.674131597	0.826897587	PACSIN3	protein_coding
ENSG00000165914.15	873.3019518	0.07548292	0.091048867	0.829037449	0.407083217	0.628212527	TTC7B	protein_coding
ENSG00000165915.14	2793.127459	0.253284665	0.053431721	4.740342619	2.13357E-06	9.46789E-05	SLC39A13	protein_coding
ENSG00000165916.8	5249.927948	-0.021707908	0.04581324	-0.473834802	0.635617715	0.802398925	PSMC3	protein_coding
ENSG00000165917.10	19.49235706	0.268674256	0.160340229	1.675650946	0.093806584	0.264703069	RAPSN	protein_coding
ENSG00000165923.16	123.0705234	-0.222741431	0.108995395	-2.043585697	0.040994493	0.156319307	AGBL2	protein_coding
ENSG00000165929.13	6751.506432	0.010934892	0.107646085	0.101581883	0.91908856	0.961796316	TC2N	protein_coding
ENSG00000165934.13	4433.077561	0.0527457	0.044048012	1.197459264	0.231127596	0.455121882	CPSF2	protein_coding

ENSG00000165935.9	30.53186407	0.2626429	0.124000046	2.118087114	0.034167691	0.138594941	SMCO2	protein_coding
ENSG00000165943.5	1382.744992	-0.005367064	0.053684088	-0.099974945	0.920364217	0.962460979	MOAP1	protein_coding
ENSG00000165948.11	385.3091574	-0.052283091	0.076112991	-0.686914154	0.492136827	0.699131101	IFI27L1	protein_coding
ENSG00000165949.12	18479.46853	0.048927146	0.118333301	0.413468951	0.679263066	0.830091888	IFI27	protein_coding
ENSG00000165953.10	2.976087538	-0.447921506	0.353371872	-1.267564119	0.204953652	0.42450273	SERPINA12	protein_coding
ENSG00000165959.12	3983.327106	0.066768865	0.079126193	0.843827596	0.398765752	0.621651171	CLMN	protein_coding
ENSG00000165966.16	232.3087644	-0.476175522	0.236566209	-2.012863643	0.044128976	0.164247386	PDZRN4	protein_coding
ENSG00000165970.12	3.9379744	-0.429427622	0.345472895	-1.243013933	0.213862699	0.434779325	SLC6A5	protein_coding
ENSG00000165972.13	9.886622148	-0.180913575	0.159538282	-1.133982219	0.256802007	0.483899552	CCDC38	protein_coding
ENSG00000165973.19	45.38420113	-0.408505717	0.212591473	-1.92155269	0.054662066	0.188390452	NELL1	protein_coding
ENSG00000165983.14	1715.206956	-0.121008217	0.069941538	-1.730133786	0.083606375	0.246941269	PTER	protein_coding
ENSG00000165985.10	98.97386987	-0.209320177	0.129528579	-1.616015394	0.106090985	0.28513962	C1QL3	protein_coding
ENSG00000165995.22	373.9281722	-0.200785472	0.137261723	-1.462792887	0.14352406	0.342703998	CACNB2	protein_coding
ENSG00000165996.14	241.7614071	-0.099732056	0.155889739	-0.639760235	0.522328488	0.722003024	HACD1	protein_coding
ENSG00000165997.5	4113.818785	0.025804865	0.072254186	0.357140073	0.720986946	0.854272885	ARL5B	protein_coding
ENSG00000166002.7	1164.602879	-0.115619827	0.072094136	-1.603734142	0.108772667	0.289235202	SMCO4	protein_coding
ENSG00000166004.15	1162.00343	-0.003041778	0.064119055	-0.047439535	0.96216292	0.98245363	CEP295	protein_coding
ENSG00000166006.14	3.414278508	-0.146246446	0.287107855	-0.509378073	0.610487243	0.785960842	KCNC2	protein_coding
ENSG00000166007.11	0.216760036	0.30656821	0.837334453	0.366123965	0.714272575		TRIM51HP	transcribed_unprocessed_pseudogene
ENSG00000166012.17	3277.989947	-0.096878398	0.065278804	-1.484071276	0.137790012	0.334193951	TAF1D	protein_coding
ENSG00000166013.11	0.116883937	-0.185308059	1.207741264	-0.153433574	0.87805637		TRIM53BP	unprocessed_pseudogene
ENSG00000166016.6	1556.471692	0.134886465	0.08121588	1.660838557	0.096745875	0.269765485	ABTB2	protein_coding
ENSG00000166024.14	941.5567089	-0.018078811	0.049058593	-0.368514663	0.712489514	0.849700764	R3HCC1L	protein_coding
ENSG00000166025.18	3574.424796	0.162150588	0.119876349	1.352648709	0.176167883	0.388269667	AMOTL1	protein_coding
ENSG00000166033.13	5522.177265	0.386973883	0.103403846	3.742354829	0.000182304	0.003178974	HTRA1	protein_coding
ENSG00000166035.11	209.2332424	-0.282517061	0.19979165	-1.414058398	0.157344757	0.362396466	LIPC	protein_coding
ENSG00000166037.11	2000.860288	-0.166281537	0.049191935	-3.380260128	0.000724173	0.008985434	CEP57	protein_coding
ENSG00000166046.11	654.4905567	0.050974097	0.085399622	0.596889026	0.550581475	0.743041155	TCP11L2	protein_coding
ENSG00000166049.11	8.986610969	2.961637329	0.469607976	6.306616331	2.85202E-10	6.50693E-08	PASD1	protein_coding
ENSG00000166068.13	3324.861758	0.104974737	0.057235103	1.834097127	0.066639578	0.213119686	SPRED1	protein_coding
ENSG00000166069.13	3.584999343	-0.667892688	0.329767453	-2.025344474	0.042832005	0.161067055	TMCO5A	protein_coding
ENSG00000166073.11	592.8607074	0.444772524	0.099838512	4.454919383	8.39247E-06	0.000281539	GPR176	protein_coding
ENSG00000166086.13	967.509775	0.075037047	0.133226581	0.563228793	0.573279099	0.758907015	JAM3	protein_coding
ENSG00000166090.8	0.444054852	0.154879982	0.426722738	0.36295226	0.716640547		IL25	protein_coding
ENSG00000166091.21	8.795365697	-0.316395125	0.217615473	-1.453918334	0.145968949	0.346415908	CMTM5	protein_coding
ENSG00000166104.15	7.019702627	1.204485205	0.280770393	4.28992955	1.7873E-05	0.00050872	AC126323.1	transcribed_unprocessed_pseudogene
ENSG00000166105.16	41.69967578	0.042686317	0.223112138	0.191322251	0.848273129	0.925634109	GLB1L3	protein_coding
ENSG00000166106.3	406.2796081	0.241683141	0.155705365	1.552182483	0.120618566	0.308480266	ADAMTS15	protein_coding
ENSG00000166111.10	27.85453887	0.104570237	0.203952635	0.512718244	0.608148426	0.784131253	SVOP	protein_coding
ENSG00000166118.8	0.563972217	-0.169368638	0.432340922	-0.391747875	0.695244512		SPATA19	protein_coding
ENSG00000166123.14	2760.398592	0.010624598	0.113003936	0.094019718	0.925093494	0.96471831	GPT2	protein_coding
ENSG00000166126.11	3319.495238	0.050336191	0.136005281	0.370104683	0.711304493	0.849078613	AMN	protein_coding

ENSG00000166128.13	1956.315544	-0.11525526	0.064427905	-1.788902796	0.073630471	0.22723761	RAB8B	protein_coding
ENSG00000166130.15	947.1195908	0.125971995	0.068168995	1.847936814	0.064611484	0.2091865	IKBIP	protein_coding
ENSG00000166133.18	528.2254526	-0.06076245	0.052080862	-1.16669441	0.243333801	0.46933816	RPUSD2	protein_coding
ENSG00000166135.14	2875.706368	-0.069016703	0.048273173	-1.429711353	0.15279988	0.35609179	HIF1AN	protein_coding
ENSG00000166136.16	652.2013899	-0.277328796	0.076754771	-3.613179902	0.000302465	0.004672281	NDUFB8	protein_coding
ENSG00000166140.17	1171.971704	-0.022512332	0.046198026	-0.487300726	0.626045233	0.796112208	ZFYVE19	protein_coding
ENSG00000166143.10	557.6438623	-0.483182431	0.176058002	-2.744450271	0.006061234	0.042629177	PPP1R14D	protein_coding
ENSG00000166145.14	16092.70855	0.14195932	0.082788786	1.71471678	0.086397166	0.251906591	SPINT1	protein_coding
ENSG00000166147.14	10282.58021	0.597763475	0.123852052	4.826431763	1.39001E-06	6.73027E-05	FBN1	protein_coding
ENSG00000166148.4	351.0990045	0.200588526	0.12661357	1.584257722	0.113135083	0.296617983	AVPR1A	protein_coding
ENSG00000166152.4	1.482633273	0.019153855	0.307145485	0.062360853	0.950275469	0.976776854	C16orf78	protein_coding
ENSG00000166153.16	60.14594076	-0.191444823	0.080449574	-2.379687215	0.017327339	0.08824044	DEPDC4	protein_coding
ENSG00000166159.11	4.509214793	0.516350502	0.242406051	2.130105662	0.033162891	0.136025913	LRTM2	protein_coding
ENSG00000166160.9	0						OPN1MW2	protein_coding
ENSG00000166164.16	3792.656979	-0.095622939	0.046353526	-2.062905385	0.039121618	0.151834945	BRD7	protein_coding
ENSG00000166165.13	6840.001103	-0.15484903	0.16379139	-0.94540397	0.344452676	0.574183835	CKB	protein_coding
ENSG00000166166.13	1442.686243	-0.037533354	0.069329099	-0.54137952	0.58824602	0.770136603	TRMT61A	protein_coding
ENSG00000166167.18	1387.218004	0.038531161	0.049814031	0.773500163	0.439226432	0.65575477	BTRC	protein_coding
ENSG00000166169.17	1091.826606	-0.055511131	0.045482435	-1.220496029	0.222276894	0.444813231	POLL	protein_coding
ENSG00000166170.10	3209.053585	0.110213923	0.042783312	2.576096079	0.009992288	0.060426578	BAG5	protein_coding
ENSG00000166171.13	655.3425903	-0.093468077	0.0740871	-1.261597181	0.207093771	0.427023056	DPCD	protein_coding
ENSG00000166173.11	515.3413714	0.39569904	0.130781338	3.025653713	0.002480963	0.022314326	LARP6	protein_coding
ENSG00000166181.13	6638.362107	0.027008774	0.043416151	0.62209048	0.533882375	0.731005852	API5	protein_coding
ENSG00000166183.16	118.7339672	0.853482371	0.199122525	4.2862171	1.81741E-05	0.000515273	ASPG	protein_coding
ENSG00000166188.2	977.5593576	-0.001090065	0.061094615	-0.017842251	0.985764699	0.99335557	ZNF319	protein_coding
ENSG00000166189.8	1224.326783	-0.006447368	0.05977702	-0.107856962	0.914109157	0.959368031	HPS6	protein_coding
ENSG00000166192.15	130.5433279	-0.064044369	0.059324163	-1.079566322	0.280335345	0.509155726	SENP8	protein_coding
ENSG00000166197.17	8694.102367	-0.010641563	0.063307827	-0.168092381	0.866510603	0.935276857	NOLC1	protein_coding
ENSG00000166199.13	880.0711585	-0.066508522	0.070684785	-0.940917085	0.346747351	0.576043968	ALKBH3	protein_coding
ENSG00000166200.15	3809.11895	-0.005139461	0.047111752	-0.109090857	0.913130426	0.95894649	COPS2	protein_coding
ENSG00000166206.15	751.0324062	0.299916758	0.211248901	1.419731688	0.155685809	0.360290417	GABRB3	protein_coding
ENSG00000166211.8	14.19972162	-0.940606082	0.22127267	-4.25089136	2.12921E-05	0.000586503	SPIC	protein_coding
ENSG00000166220.12	3.842390176	0.484900013	0.218309418	2.221159387	0.026340168	0.116898411	TBATA	protein_coding
ENSG00000166224.17	6002.691451	0.145559677	0.064681258	2.250415061	0.02442261	0.111219988	SGPL1	protein_coding
ENSG00000166225.9	2937.333985	0.090967811	0.083399135	1.090752447	0.275381826	0.504201044	FRS2	protein_coding
ENSG00000166226.13	9432.678478	0.122410174	0.081542623	1.501180228	0.133308952	0.327610891	CCT2	protein_coding
ENSG00000166228.9	6352.768339	-0.289278734	0.071615758	-4.039316818	5.36071E-05	0.001216053	PCBD1	protein_coding
ENSG00000166233.15	5083.935679	0.125407912	0.05326603	2.354369399	0.018554162	0.092318659	ARIH1	protein_coding
ENSG00000166246.14	54.04283703	0.229671998	0.088320533	2.600437188	0.009310506	0.057476119	C16orf71	protein_coding
ENSG00000166250.12	1497.80164	-0.03753994	0.154322671	-0.243256159	0.807806965	0.904605132	CLMP	protein_coding
ENSG00000166257.9	140.2758814	0.04589828	0.169007425	0.271575522	0.785948423	0.891719206	SCN3B	protein_coding
ENSG00000166260.13	2298.264916	-0.084439993	0.043656438	-1.93419336	0.053089359	0.18473808	COX11	protein_coding

ENSG00000166261.11	721.4328455	-0.141460712	0.04971623	-2.845362832	0.004436086	0.034190188	ZNF202	protein_coding
ENSG00000166262.16	130.2092538	-0.104342467	0.072398351	-1.441227123	0.149520528	0.351608443	FAM227B	protein_coding
ENSG00000166263.13	864.8894519	-0.103774246	0.069368771	-1.495979301	0.134659049	0.329850331	STXBP4	protein_coding
ENSG00000166265.13	896.6511893	-0.087766442	0.092250635	-0.951391194	0.341405829	0.571374215	CYYR1	protein_coding
ENSG00000166266.14	2012.085505	0.033472505	0.044146888	0.75820757	0.44832673	0.663431605	CUL5	protein_coding
ENSG00000166268.11	135.6167136	-0.164932317	0.195958682	-0.841668844	0.399973343	0.622728091	MYRFL	protein_coding
ENSG00000166272.18	3571.340735	0.048847325	0.058903375	0.829278878	0.40694662	0.628135196	WBP1L	protein_coding
ENSG00000166275.16	1040.727783	-0.098527646	0.060860425	-1.618911567	0.105466292	0.284304509	BORCS7	protein_coding
ENSG00000166278.15	1588.985465	-0.08782693	0.120365885	-0.729666295	0.465594188	0.677375627	C2	protein_coding
ENSG00000166289.6	861.240107	0.242198948	0.115493454	2.097079443	0.035986543	0.143435983	PLEKHF1	protein_coding
ENSG00000166292.12	210.5367921	-0.275919154	0.196184364	-1.406427854	0.159597114	0.365386331	TMEM100	protein_coding
ENSG00000166295.9	4966.341431	-0.173791499	0.05700263	-3.048832992	0.002297322	0.021120334	ANAPC16	protein_coding
ENSG00000166311.10	1857.545399	0.144952684	0.070054484	2.069142132	0.038532751	0.150326025	SMPD1	protein_coding
ENSG00000166313.19	931.3752907	0.064581607	0.122447887	0.527421159	0.597901153	0.777057517	APBB1	protein_coding
ENSG00000166317.12	27.42785436	-0.223469651	0.151823608	-1.471903176	0.141047026	0.338923245	SYNPO2L	protein_coding
ENSG00000166321.14	196.2791816	-0.141510443	0.08412073	-1.682230318	0.092524187	0.262649111	NUDT13	protein_coding
ENSG00000166323.13	42.83010706	-0.16832204	0.084352187	-1.995467413	0.045991925	0.168538293	C11orf65	protein_coding
ENSG00000166326.7	8610.687319	-0.01086841	0.05264068	-0.206464099	0.836428406	0.919722528	TRIM44	protein_coding
ENSG00000166329.3	0.622652323	-0.231943361	0.360981102	-0.642536023	0.520525208		CCDC182	protein_coding
ENSG00000166333.14	57.04104814	-0.183817373	0.099102348	-1.854823582	0.063621423	0.207315543	ILK	protein_coding
ENSG00000166337.10	384.0605832	-0.172795039	0.066970735	-2.580157442	0.009875528	0.05990338	TAF10	protein_coding
ENSG00000166340.17	15011.93814	-0.107998877	0.051844456	-2.083132604	0.037239138	0.146791106	TPP1	protein_coding
ENSG00000166341.8	2716.418936	0.209686569	0.109121312	1.921591339	0.054657199	0.188388576	DCHS1	protein_coding
ENSG00000166342.19	15.29695192	0.260121389	0.221861987	1.172446857	0.241017696	0.46660572	NETO1	protein_coding
ENSG00000166343.10	95.58360236	-0.201050913	0.092767795	-2.167249014	0.030215872	0.128173915	MSS51	protein_coding
ENSG00000166347.19	3296.353438	-0.342759436	0.06923255	-4.950842257	7.3893E-07	4.10195E-05	CYB5A	protein_coding
ENSG00000166348.18	3546.781186	-0.012351264	0.084904975	-0.14547162	0.884338521	0.944576199	USP54	protein_coding
ENSG00000166349.10	134.6157381	0.187550538	0.123593953	1.517473422	0.129147199	0.321444339	RAG1	protein_coding
ENSG00000166351.10	0.310199363	-0.124464513	0.412441873	-0.301774678	0.762823835		POTED	protein_coding
ENSG00000166352.17	402.8428287	0.025611671	0.079079028	0.323874381	0.746033141	0.869303793	IFTAP	protein_coding
ENSG00000166359.11	61.09971144	-0.042863162	0.103892018	-0.412574162	0.679918635	0.830460015	WDR88	protein_coding
ENSG00000166363.5	2.092509612	0.069551182	0.250458183	0.277695788	0.781245894	0.889172516	OR10A5	protein_coding
ENSG00000166368.2	0.796958645	0.26686851	0.30061267	0.88774871	0.374675938		OR2D2	protein_coding
ENSG00000166377.20	1581.953019	-0.134268561	0.057930274	-2.317761527	0.020462285	0.098670466	ATP9B	protein_coding
ENSG00000166387.13	2872.77205	-0.387928083	0.077562804	-5.001470588	5.68946E-07	3.29694E-05	PPFIBP2	protein_coding
ENSG00000166391.15	921.5624857	-0.598377997	0.234831255	-2.548119061	0.01083055	0.063899659	MOGAT2	protein_coding
ENSG00000166394.15	602.2651956	0.471958887	0.102799461	4.591063843	4.40992E-06	0.000169016	CYB5R2	protein_coding
ENSG00000166396.13	254.8385418	1.102019669	0.259971097	4.239008419	2.24509E-05	0.000611083	SERPINB7	protein_coding
ENSG00000166398.14	2332.919136	0.167614113	0.061015251	2.747085547	0.006012745	0.042390776	GARRE1	protein_coding
ENSG00000166401.15	1158.198696	0.244296295	0.084745468	2.882706312	0.003942748	0.03145033	SERPINB8	protein_coding
ENSG00000166402.9	261.6736687	0.066251975	0.155860722	0.425071651	0.670784443	0.824526928	TUB	protein_coding
ENSG00000166405.15	141.4494227	-0.382011811	0.191838393	-1.991320952	0.046445614	0.169681464	RIC3	protein_coding

ENSG00000166407.14	35.98985052	-0.775045248	0.294988845	-2.627371377	0.008604737	0.054397434	LMO1	protein_coding
ENSG00000166408.4	0.964379049	-0.230562171	0.400152956	-0.576185101	0.564490075		OR5P1P	unprocessed_pseudogene
ENSG00000166411.14	1639.312283	-0.12591172	0.066238878	-1.900873379	0.0573186	0.194281262	IDH3A	protein_coding
ENSG00000166415.15	1836.018725	0.350219233	0.200879075	1.743433122	0.081257987	0.242500986	WDR72	protein_coding
ENSG00000166426.8	128.0361127	0.552185803	0.294871664	1.872630946	0.061119365	0.202094132	CRABP1	protein_coding
ENSG00000166428.14	358.2846813	-0.557673377	0.178052981	-3.132064259	0.001735819	0.017297456	PLD4	protein_coding
ENSG00000166432.15	289.4819924	-0.248255985	0.139505628	-1.779541005	0.075151108	0.230623933	ZMAT1	protein_coding
ENSG00000166435.15	736.331211	0.083107986	0.080591046	1.031231014	0.3024325	0.531973079	XRRA1	protein_coding
ENSG00000166436.16	958.395157	-0.163035681	0.076609848	-2.12812954	0.033326345	0.136413876	TRIM66	protein_coding
ENSG00000166439.6	1478.109011	0.221359749	0.063759434	3.471796028	0.000516989	0.006970554	RNF169	protein_coding
ENSG00000166441.13	43770.35225	-0.262942557	0.066764243	-3.938373942	8.20357E-05	0.001701508	RPL27A	protein_coding
ENSG00000166444.19	3792.936266	0.017768039	0.076459477	0.23238505	0.816238959	0.909324753	DENND2B	protein_coding
ENSG00000166446.15	921.3573879	0.524130417	0.094707494	5.534202184	3.12648E-08	3.09642E-06	CDYL2	protein_coding
ENSG00000166448.15	181.3894904	-0.078902657	0.17699917	-0.445779816	0.655756303	0.81496385	TMEM130	protein_coding
ENSG00000166450.13	219.0625835	-0.211631703	0.148336396	-1.426701124	0.153666061	0.357365032	PRTG	protein_coding
ENSG00000166451.13	761.0168713	-0.079568122	0.073174524	-1.087374636	0.276871276	0.505751995	CENPN	protein_coding
ENSG00000166452.12	775.5473028	-0.077148643	0.046632243	-1.654405562	0.098045137	0.272238622	AKIP1	protein_coding
ENSG00000166454.10	3836.638318	0.030994455	0.037896471	0.817871805	0.413430391	0.634033298	ATMIN	protein_coding
ENSG00000166455.14	2.135565298	0.085612872	0.153880435	0.556359693	0.577964987	0.762358646	C16orf46	protein_coding
ENSG00000166471.11	2293.21282	0.033274579	0.059681868	0.557532462	0.577163687	0.761822537	TMEM41B	protein_coding
ENSG00000166473.17	64.52114075	0.42667396	0.177887133	2.398565609	0.016459428	0.085516936	PKD1L2	polymorphic_pseudogene
ENSG00000166477.13	1732.742551	0.062120365	0.052200966	1.190023289	0.234037239	0.458613218	LEO1	protein_coding
ENSG00000166478.10	1080.269509	-0.005727769	0.037348521	-0.153359985	0.878114399	0.941250336	ZNF143	protein_coding
ENSG00000166479.10	2470.613064	-0.012190576	0.057520789	-0.211933395	0.832158997	0.917376928	TMX3	protein_coding
ENSG00000166482.12	8781.530762	-0.020542755	0.162070907	-0.126751652	0.899136963	0.951977512	MFAP4	protein_coding
ENSG00000166483.11	3284.142916	0.097824604	0.076574465	1.277509465	0.201422441	0.420332617	WEE1	protein_coding
ENSG00000166484.20	1263.299107	0.11211292	0.051795469	2.16453143	0.030423587	0.128733721	MAPK7	protein_coding
ENSG00000166492.9	23.56969537	0.302879095	0.137172426	2.208017334	0.027243065	0.119650377	FAM86GP	unprocessed_pseudogene
ENSG00000166501.14	1642.745018	-0.716009888	0.148862072	-4.80988797	1.51015E-06	7.19211E-05	PRKCB	protein_coding
ENSG00000166503.9	1318.850908	0.148807283	0.117085417	1.270929257	0.203753817	0.423090636	HDGFL3	protein_coding
ENSG00000166507.19	24.46360245	-0.030890431	0.084303327	-0.366420071	0.714051645	0.850557998	NDST2	protein_coding
ENSG00000166508.18	8874.39981	0.005494742	0.090569455	0.060668818	0.951622966	0.977271197	MCM7	protein_coding
ENSG00000166509.12	7.003907812	-0.180112116	0.276957499	-0.650324026	0.515482941	0.717049557	CLEC3A	protein_coding
ENSG00000166510.14	1624.859584	-0.064598184	0.11451745	-0.564090312	0.572692671	0.758640216	CCDC68	protein_coding
ENSG00000166523.8	218.451504	0.024280121	0.157757374	0.153907995	0.87768228	0.94102964	CLEC4E	protein_coding
ENSG00000166526.18	2236.703405	-0.102029393	0.05929274	-1.720773783	0.085291882	0.249883308	ZNF3	protein_coding
ENSG00000166527.8	18.89515064	0.216181075	0.1712573	1.262317431	0.206834587	0.426698944	CLEC4D	protein_coding
ENSG00000166529.15	253.3430011	0.095428256	0.06848714	1.393374825	0.163506475	0.370703712	ZSCAN21	protein_coding
ENSG00000166530.8	2.544802864	-0.109040044	0.159533162	-0.683494533	0.494294404	0.701089647	HSBP1P2	processed_pseudogene
ENSG00000166532.16	803.712892	0.779229444	0.145762876	5.345870412	8.99836E-08	7.44063E-06	RIMKLB	protein_coding
ENSG00000166535.20	336.7816694	1.062406778	0.289509924	3.669673085	0.000242861	0.003940114	A2ML1	protein_coding
ENSG00000166546.14	111.2919642	0.894604809	0.156967889	5.699285484	1.20311E-08	1.40557E-06	BEAN1	protein_coding

ENSG00000166548.17	967.7155394	0.081005791	0.061724299	1.312380892	0.189391651	0.404862397	TK2	protein_coding
ENSG00000166557.14	6452.401798	0.121517335	0.074292779	1.63565473	0.101911869	0.278538228	TMED3	protein_coding
ENSG00000166558.11	6.028631966	0.449786453	0.26057596	1.726124134	0.084325083	0.248051447	SLC38A8	protein_coding
ENSG00000166562.9	2475.075494	-0.085911975	0.07917958	-1.085026909	0.277909737	0.506799156	SEC11C	protein_coding
ENSG00000166569.8	2.172713391	-0.40375993	0.260185036	-1.551818415	0.120705678	0.308594388	CPLX4	protein_coding
ENSG00000166573.6	11.77311925	-0.331155264	0.229185179	-1.444924426	0.148479115	0.350198873	GALR1	protein_coding
ENSG00000166575.17	1502.875274	0.067220723	0.061726508	1.089009005	0.276149915	0.504878743	TMEM135	protein_coding
ENSG00000166578.10	94.49840758	0.25485297	0.096202124	2.649140779	0.008069671	0.052087019	IQCD	protein_coding
ENSG00000166579.16	2377.754057	0.212965313	0.046408422	4.588936742	4.45509E-06	0.00017	NDEL1	protein_coding
ENSG00000166582.10	1734.00283	-0.31146388	0.083455037	-3.732116036	0.000189878	0.003273067	CENPV	protein_coding
ENSG00000166589.13	276.7719016	0.312394721	0.25661843	1.217351072	0.223470671	0.446152994	CDH16	protein_coding
ENSG00000166592.12	515.9559639	0.230430038	0.163501098	1.409348562	0.158732126	0.3642672	RRAD	protein_coding
ENSG00000166595.12	3326.145156	-0.117692568	0.070110011	-1.67868421	0.093213605	0.263844164	CIAO2B	protein_coding
ENSG00000166596.15	24.89544733	0.01435628	0.120694663	0.118947102	0.905317265	0.954975925	CFAP52	protein_coding
ENSG00000166598.15	45161.68839	-0.004292886	0.057601253	-0.074527646	0.940590544	0.971950254	HSP90B1	protein_coding
ENSG00000166603.5	2.794628555	-0.561935507	0.264578824	-2.123886937	0.033679599	0.137336512	MC4R	protein_coding
ENSG00000166619.15	4622.358938	0.069602986	0.057807045	1.204057152	0.228567489	0.452164767	BLCAP	protein_coding
ENSG00000166634.6	1.930819767	0.394303704	0.372174639	1.059458821	0.289390873	0.519093771	SERPINB12	protein_coding
ENSG00000166664.14	25.00059395	-0.280172001	0.152143344	-1.841500209	0.06554829	0.211020845	CHRFAM7A	protein_coding
ENSG00000166669.13	1045.027045	-0.299610705	0.10581576	-2.831437458	0.004633929	0.035268136	ATF7IP2	protein_coding
ENSG00000166670.10	321.479633	-0.228018674	0.204515269	-1.114922499	0.264883634	0.492714993	MMP10	protein_coding
ENSG00000166676.17	70.43265892	-0.198123821	0.094528548	-2.095915212	0.036089713	0.143702615	TVP23A	protein_coding
ENSG00000166681.14	2009.497967	0.322697031	0.128709772	2.507168074	0.012170283	0.069174695	BEX3	protein_coding
ENSG00000166682.13	121.573209	0.556177312	0.185561115	2.997272961	0.002724067	0.023877138	TMPRSS5	protein_coding
ENSG00000166685.12	1851.155814	0.106677464	0.045855604	2.326377904	0.019998399	0.097240599	COG1	protein_coding
ENSG00000166689.16	3635.800613	0.039367054	0.080013592	0.492004586	0.622716102	0.794048277	PLEKHA7	protein_coding
ENSG00000166693.7	0.505184444	-0.177674568	0.551776303	-0.32200471	0.747449126		OR5M13P	unprocessed_pseudogene
ENSG00000166704.11	399.1495529	-0.070595812	0.129027302	-0.547138561	0.584283542	0.767098013	ZNF606	protein_coding
ENSG00000166707.12	34.22975569	-0.178622921	0.116667352	-1.531044619	0.125758366	0.316216003	ZCCHC18	protein_coding
ENSG00000166710.21	179852.4126	-0.160994291	0.080252085	-2.006107274	0.044844804	0.16582113	B2M	protein_coding
ENSG00000166716.10	4457.672108	0.145435774	0.053059867	2.740975093	0.006125715	0.042909546	ZNF592	protein_coding
ENSG00000166734.20	6919.998676	0.136882106	0.047318605	2.892775621	0.00381854	0.030684217	GOLM2	protein_coding
ENSG00000166736.11	68.67680922	0.495849704	0.208511133	2.378049067	0.01740451	0.088498987	HTR3A	protein_coding
ENSG00000166741.8	3533.020843	0.697173056	0.12414192	5.61593582	1.95501E-08	2.08297E-06	NNMT	protein_coding
ENSG00000166743.9	60.7830953	-0.582822216	0.137788733	-4.229824912	2.33873E-05	0.000630272	ACSM1	protein_coding
ENSG00000166747.13	6753.380581	0.037346182	0.052891542	0.706089867	0.480132253	0.689289731	AP1G1	protein_coding
ENSG00000166750.10	4672.683992	0.202409849	0.088897906	2.276879813	0.022793404	0.106288727	SLFN5	protein_coding
ENSG00000166762.19	253.11812	-0.043266857	0.101836228	-0.424867042	0.670933602	0.824653736	CATSPER2	protein_coding
ENSG00000166763.8	27.92363443	0.060845199	0.135036134	0.450584573	0.652288987	0.812932292	STRCP1	transcribed_unprocessed_pseudogene
ENSG00000166770.11	241.0127222	-0.1683004	0.137786531	-1.221457556	0.221912826	0.444447487	ZNF667-AS1	lncRNA
ENSG00000166780.11	908.1024831	0.056355357	0.119793031	0.470439366	0.638041145	0.803985701	BMERB1	protein_coding
ENSG00000166783.22	5313.817155	-0.050239032	0.055339121	-0.907839352	0.363963104	0.591159339	MARF1	protein_coding

ENSG00000166787.3	1.083348814	-0.603419117	0.438675782	-1.37554691	0.168961953	0.378280673	SAA3P	transcribed_unprocessed_pseudogene
ENSG00000166788.10	711.4542859	-0.12323847	0.059314841	-2.077700432	0.037736959	0.148149862	SAAL1	protein_coding
ENSG00000166793.13	43.50145579	0.102359096	0.120321916	0.850710321	0.394930288	0.618437444	YPEL4	protein_coding
ENSG00000166794.5	4882.183495	0.089032217	0.064290831	1.384835374	0.166102812	0.374476808	PPIB	protein_coding
ENSG00000166796.12	41.07264106	-0.560240023	0.245594913	-2.281154836	0.022539286	0.105372097	LDHC	protein_coding
ENSG00000166797.11	2636.721241	-0.144137588	0.05679489	-2.537861924	0.011153198	0.065194219	CIAO2A	protein_coding
ENSG00000166800.10	27.61747688	-0.184383609	0.133994091	-1.376057767	0.168803749	0.378058006	LDHAL6A	protein_coding
ENSG00000166801.16	2720.516364	-0.042883373	0.061643552	-0.695666811	0.486637515	0.694147397	FAM111A	protein_coding
ENSG00000166803.14	938.200672	-0.316044971	0.093716727	-3.372343238	0.000745315	0.009187721	PCLAF	protein_coding
ENSG00000166813.16	366.9794356	0.435098109	0.115288572	3.773991657	0.000160656	0.00286944	KIF7	protein_coding
ENSG00000166816.15	717.42649	-0.147993786	0.143717807	-1.029752601	0.303126156	0.532529662	LDHD	protein_coding
ENSG00000166819.12	65.05991434	0.848395817	0.172347204	4.922596938	8.54032E-07	4.60596E-05	PLIN1	protein_coding
ENSG00000166821.9	577.7055733	-0.261013482	0.082337624	-3.17003904	0.001524185	0.015700326	PEX11A	protein_coding
ENSG00000166822.13	1727.686199	-0.09399047	0.053666205	-1.751390291	0.079878704	0.239937272	TMEM170A	protein_coding
ENSG00000166823.5	150.3552904	-0.386225886	0.166861923	-2.314643629	0.020632443	0.099227455	MESP1	protein_coding
ENSG00000166825.15	35194.41441	-1.133745637	0.239895383	-4.726000243	2.28985E-06	0.000100387	ANPEP	protein_coding
ENSG00000166828.3	44.10734051	-0.271921005	0.253224006	-1.073835805	0.282896279	0.511759358	SCNN1G	protein_coding
ENSG00000166831.9	1158.39354	-0.313916955	0.193862663	-1.619274959	0.105388116	0.284233332	RBPMS2	protein_coding
ENSG00000166833.22	2863.059564	0.000437279	0.103005949	0.004245183	0.996612844	0.998399915	NAV2	protein_coding
ENSG00000166839.17	198.0896561	0.057265658	0.079911604	0.716612551	0.473613198	0.684364705	ANKDD1A	protein_coding
ENSG00000166840.13	37.43197096	-0.729067373	0.209217342	-3.484736813	0.000492621	0.006727669	GLYATL1	protein_coding
ENSG00000166845.15	366.5203768	-0.191869243	0.078889624	-2.432122687	0.015010622	0.080151681	C18orf54	protein_coding
ENSG00000166847.10	4330.274203	0.017122213	0.043846514	0.390503393	0.696164347	0.839696017	DCTN5	protein_coding
ENSG00000166848.7	3004.799824	-0.045508017	0.047496339	-0.958137362	0.337993498	0.567844192	TERF2IP	protein_coding
ENSG00000166851.15	3602.556789	0.09976738	0.093354818	1.068690205	0.285209287	0.514576454	PLK1	protein_coding
ENSG00000166855.9	2997.506248	-0.025360245	0.040390922	-0.627869916	0.53008916	0.728097949	CLPX	protein_coding
ENSG00000166856.3	13.62601921	0.120331785	0.121783249	0.988081579	0.323112697	0.552939851	GPR182	protein_coding
ENSG00000166860.3	1008.311146	-0.026955295	0.063845999	-0.422192392	0.672884594	0.825971693	ZBTB39	protein_coding
ENSG00000166862.7	7.22256229	-0.373543195	0.186067242	-2.007570975	0.044688901	0.165486779	CACNG2	protein_coding
ENSG00000166863.13	94.42687315	-0.297514638	0.181328851	-1.640746277	0.100850094	0.276834303	TAC3	protein_coding
ENSG00000166866.13	2470.415482	-0.210406946	0.170368962	-1.235007502	0.216827712	0.438149836	MYO1A	protein_coding
ENSG00000166869.3	559.3486644	-0.659245535	0.285600991	-2.308274674	0.020983864	0.100396777	CHP2	protein_coding
ENSG00000166881.10	2140.414871	-0.048139399	0.073338458	-0.656400477	0.511566484	0.714435556	NEMP1	protein_coding
ENSG00000166884.2	0.674765406	-0.361366904	0.480585363	-0.751930731	0.452092716		OR4D6	protein_coding
ENSG00000166886.13	1355.706404	0.046998841	0.07945136	0.591542304	0.554157119	0.745394144	NAB2	protein_coding
ENSG00000166887.16	4325.495411	-0.04363549	0.038606289	-1.130268956	0.258362912	0.485532354	VPS39	protein_coding
ENSG00000166888.12	14728.25392	-0.068459711	0.04996763	-1.370081216	0.170661551	0.380775096	STAT6	protein_coding
ENSG00000166889.14	6256.663146	-0.012438469	0.047517139	-0.261768047	0.793500275	0.896435704	PATL1	protein_coding
ENSG00000166896.9	390.2954551	-0.297816603	0.063600223	-4.682634598	2.83211E-06	0.000118554	ATP23	protein_coding
ENSG00000166897.16	286.9546014	-0.199133141	0.214330626	-0.929093267	0.352840754	0.581231458	ELFN2	protein_coding
ENSG00000166900.17	3989.143152	-0.105969583	0.076398079	-1.3870713	0.165420022	0.373420447	STX3	protein_coding
ENSG00000166902.5	2368.796624	-0.202401716	0.063663457	-3.179244843	0.001476593	0.01535692	MRPL16	protein_coding

ENSG00000166908.18	3797.48241	0.141293222	0.066245366	2.132877052	0.032934813	0.135383488	PIP4K2C	protein_coding
ENSG00000166912.17	2185.961709	-0.121599818	0.064218797	-1.893523761	0.058288251	0.19623201	MTMR10	protein_coding
ENSG00000166913.13	35415.10411	-0.056579459	0.053485339	-1.057849871	0.290123892	0.519996035	YWHAB	protein_coding
ENSG00000166917.11	3.131455029	-0.828563253	0.261919327	-3.163429221	0.001559223	0.015964813	MIR202HG	lncRNA
ENSG00000166920.13	1782.215472	0.067545412	0.15657666	0.431388764	0.666185718	0.821598886	C15orf48	protein_coding
ENSG00000166922.8	261.2663948	0.323588029	0.128760711	2.513096017	0.011967676	0.068428969	SCG5	protein_coding
ENSG00000166923.12	21370.54161	0.218843452	0.167095846	1.309688167	0.190301348	0.405930875	GREM1	protein_coding
ENSG00000166924.9	61.51926871	0.992213922	0.162624139	6.101270859	1.05228E-09	1.87098E-07	NYAP1	protein_coding
ENSG00000166925.9	4029.690591	0.082133238	0.073692173	1.114544934	0.265045479	0.492868773	TSC22D4	protein_coding
ENSG00000166926.8	3.005000349	0.261758732	0.209187992	1.251308591	0.210821912	0.431192456	MS4A6E	protein_coding
ENSG00000166927.13	1435.437277	-0.128260444	0.118956233	-1.078215412	0.280937636	0.509845884	MS4A7	protein_coding
ENSG00000166928.11	123.2913925	-0.151993264	0.113976254	-1.333552023	0.182350715	0.395852323	MS4A14	protein_coding
ENSG00000166930.7	1.250547155	-0.845022675	0.372164109	-2.270564665	0.023173345	0.107428174	MS4A5	protein_coding
ENSG00000166938.13	1823.412403	-0.063037734	0.049610779	-1.270645934	0.203854638	0.423259651	DIS3L	protein_coding
ENSG00000166946.14	2273.192479	0.011687311	0.048170153	0.242625567	0.808295474	0.904966134	CCNDBP1	protein_coding
ENSG00000166947.15	3.116214027	-0.188357603	0.23933743	-0.786996012	0.4312842	0.649213154	EPB42	protein_coding
ENSG00000166948.10	2.356723014	0.086495647	0.341456758	0.253313618	0.800025873	0.900334118	TGM6	protein_coding
ENSG00000166949.16	5769.64249	0.23696717	0.068406252	3.464115658	0.000531978	0.007121536	SMAD3	protein_coding
ENSG00000166959.8	544.828085	-0.405507219	0.203648347	-1.991212912	0.046457485	0.169697614	MS4A8	protein_coding
ENSG00000166960.16	14.09378739	-0.017841803	0.179013805	-0.099667189	0.92060855	0.962643357	CCDC178	protein_coding
ENSG00000166961.15	47.97866163	-0.216176141	0.243892812	-0.886357165	0.375425094	0.601215162	MS4A15	protein_coding
ENSG00000166963.13	2223.022266	0.055997263	0.133541636	0.419324375	0.674979085	0.827445473	MAP1A	protein_coding
ENSG00000166965.12	1014.515525	0.030145242	0.069599927	0.433121747	0.664926325	0.820740053	RCCD1	protein_coding
ENSG00000166971.17	1047.967835	-0.10872646	0.054355087	-2.000299621	0.04546792	0.167330086	AKTIP	protein_coding
ENSG00000166974.13	4844.828068	-0.190502423	0.068118698	-2.796624553	0.00516395	0.038050194	MAPRE2	protein_coding
ENSG00000166979.13	880.4024915	0.082989123	0.105629013	0.785665984	0.432063196	0.650048954	EVA1C	protein_coding
ENSG00000166984.12	18.39222974	0.319944048	0.25938564	1.233468626	0.217400975	0.438819876	TCP10L2	protein_coding
ENSG00000166986.15	5363.699864	-0.01584131	0.05540256	-0.285931013	0.77493096	0.886050757	MARS1	protein_coding
ENSG00000166987.15	4990.882397	0.090116913	0.067960711	1.326014865	0.184834774	0.399253739	MBD6	protein_coding
ENSG00000166997.8	390.3393025	0.21629907	0.074512368	2.9028613	0.003697704	0.029989732	CNPY4	protein_coding
ENSG00000167004.13	36822.87954	-0.022918672	0.061330957	-0.373688475	0.708636101	0.847393996	PDIA3	protein_coding
ENSG00000167005.14	5888.392028	-0.119645585	0.048401575	-2.471935799	0.013438365	0.074258638	NUDT21	protein_coding
ENSG00000167011.9	8.43523014	0.183399827	0.210276988	0.872182107	0.383109039	0.608084489	NAT16	protein_coding
ENSG00000167014.11	2.734942421	-0.44757115	0.322300251	-1.388677634	0.164930798	0.372772489	TERB2	protein_coding
ENSG00000167034.10	270.9921464	0.549952262	0.132380189	4.154339613	3.26228E-05	0.000816077	NKX3-1	protein_coding
ENSG00000167037.19	374.4152665	-0.072965136	0.137777035	-0.529588519	0.596397256	0.775889755	SGSM1	protein_coding
ENSG00000167046.4	45.10380126	-0.151928055	0.150736583	-1.00790433	0.313500393	0.542808591	AL357033.1	lncRNA
ENSG00000167065.14	689.5576905	-0.135529419	0.067756776	-2.000234187	0.045474982	0.167341943	DUSP18	protein_coding
ENSG00000167074.15	1063.597912	-0.096821074	0.087702257	-1.103974718	0.269604105	0.497629636	TEF	protein_coding
ENSG00000167077.13	258.576257	-0.650723601	0.120102675	-5.418060842	6.02489E-08	5.43567E-06	MEI1	protein_coding
ENSG00000167080.9	75.13540321	0.132479154	0.254860785	0.519809881	0.603196092	0.781004162	B4GALNT2	protein_coding
ENSG00000167081.18	1770.707715	-0.084848666	0.100357242	-0.845466298	0.397850542	0.620890773	PBX3	protein_coding

ENSG00000167083.7	90.48052439	-0.321466452	0.103494817	-3.106111589	0.001895652	0.018393857	GNGT2	protein_coding
ENSG00000167085.13	4809.242308	-0.077921648	0.064126124	-1.215131114	0.224316089	0.447246623	PHB	protein_coding
ENSG00000167088.11	4584.186854	-0.167164468	0.081444951	-2.052484097	0.040122639	0.154236436	SNRPD1	protein_coding
ENSG00000167094.16	23.11056468	-0.117773977	0.133041267	-0.885243948	0.376025074	0.601852811	TTC16	protein_coding
ENSG00000167098.12	2.63516568	-0.121404263	0.407186621	-0.298153862	0.765585734	0.880796513	SUN5	protein_coding
ENSG00000167100.15	230.2871988	0.216778392	0.094707946	2.288914514	0.02208432	0.103804164	SAMD14	protein_coding
ENSG00000167103.12	76.85482216	0.408611973	0.123548574	3.307298166	0.000942006	0.010967167	PIP5KL1	protein_coding
ENSG00000167104.11	1.397700024	0.624137459	0.41362485	1.508945749	0.131312647	0.324643153	BPIFB6	protein_coding
ENSG00000167105.8	1018.658859	0.031372819	0.130460226	0.240478039	0.809959686	0.905832892	TMEM92	protein_coding
ENSG00000167106.12	9752.28451	-0.047620578	0.077117373	-0.617507784	0.536899849	0.733087411	FAM102A	protein_coding
ENSG00000167107.13	1339.767744	-0.164268469	0.102571075	-1.6015087	0.109264295	0.289966488	ACSF2	protein_coding
ENSG00000167110.18	5069.418735	0.186880779	0.048707205	3.836820054	0.000124638	0.002340085	GOLGA2	protein_coding
ENSG00000167112.10	2278.47274	-0.069505543	0.055490783	-1.252560139	0.210365829	0.430682094	TRUB2	protein_coding
ENSG00000167113.11	2714.077394	-0.043957273	0.052243555	-0.841391306	0.400128754	0.622795497	COQ4	protein_coding
ENSG00000167114.13	3919.214406	0.034844009	0.069400574	0.502070908	0.615617643	0.789115485	SLC27A4	protein_coding
ENSG00000167117.10	462.4549361	-0.427944686	0.176028643	-2.431108249	0.015052717	0.080312294	ANKRD40CL	protein_coding
ENSG00000167118.11	4455.052138	-0.041854551	0.055094036	-0.759692966	0.447438134	0.662772263	URM1	protein_coding
ENSG00000167123.19	3211.210742	0.506104257	0.113467605	4.460341405	8.18292E-06	0.00027557	CERCAM	protein_coding
ENSG00000167130.18	1362.843473	0.04569923	0.072496091	0.630368198	0.528453713	0.726723916	DOLPP1	protein_coding
ENSG00000167131.17	1.077682195	0.322157163	0.219257004	1.469312986	0.141747916	0.340046735	CCDC103	protein_coding
ENSG00000167136.7	400.2561086	-0.053625299	0.093978384	-0.57061312	0.568261923	0.755487872	ENDOG	protein_coding
ENSG00000167139.9	1.787564137	-0.319989166	0.337935655	-0.946893771	0.343692912	0.573292013	TBC1D21	protein_coding
ENSG00000167157.11	622.1827686	0.749725415	0.154396002	4.855860285	1.19865E-06	6.00905E-05	PRRX2	protein_coding
ENSG00000167165.19	164.8116134	-0.524318144	0.25083838	-2.090262839	0.036594195	0.14503819	UGT1A6	protein_coding
ENSG00000167173.19	4002.443681	0.223379073	0.060626953	3.684484553	0.000229166	0.003776991	C15orf39	protein_coding
ENSG00000167178.16	201.5577363	0.404857585	0.145699694	2.77871267	0.005457478	0.03954878	ISLR2	protein_coding
ENSG00000167182.15	3244.710431	0.049683678	0.037211496	1.335170114	0.181820676	0.39519176	SP2	protein_coding
ENSG00000167183.3	5671.214882	0.092799591	0.100932336	0.919423791	0.35787395	0.585554504	PRR15L	protein_coding
ENSG00000167186.11	970.2067427	-0.11648985	0.042120443	-2.765636858	0.005681176	0.040752034	COQ7	protein_coding
ENSG00000167191.12	4600.546408	-0.496897784	0.152206959	-3.264619349	0.001096113	0.012329712	GPRC5B	protein_coding
ENSG00000167193.8	5971.463564	0.11651607	0.037757286	3.085922839	0.002029215	0.019336779	CRK	protein_coding
ENSG00000167194.7	1.615635083	0.333954883	0.289508067	1.15352531	0.248694835	0.475365156	C16orf92	protein_coding
ENSG00000167195.7	0.598654975	-0.140638342	0.390144783	-0.36047731	0.718490222		GOLGA6C	protein_coding
ENSG00000167196.14	1653.968127	-0.006230107	0.046813881	-0.133082463	0.894128164	0.949630416	FBXO22	protein_coding
ENSG00000167202.11	3251.124825	0.063860159	0.066745933	0.956764793	0.338685986	0.568481597	TBC1D2B	protein_coding
ENSG00000167207.14	249.5565843	0.355308651	0.112714823	3.152279738	0.00162001	0.016432768	NOD2	protein_coding
ENSG00000167208.15	466.3923474	-0.44107383	0.118458166	-3.7234565	0.000196514	0.003341193	SNX20	protein_coding
ENSG00000167210.17	29.24039196	0.286845416	0.147804154	1.940712814	0.052293121	0.182916788	LOXHD1	protein_coding
ENSG00000167216.17	138.1217996	-0.222668917	0.109779254	-2.028333306	0.042526242	0.160280751	KATNAL2	protein_coding
ENSG00000167220.12	48.74583695	-0.063390992	0.078685104	-0.805628876	0.420456871	0.64027988	HDHD2	protein_coding
ENSG00000167232.14	1962.036366	-0.014476585	0.078772507	-0.183777125	0.854188302	0.928835187	ZNF91	protein_coding
ENSG00000167244.21	560.3435541	0.885938193	0.211065052	4.197465119	2.69919E-05	0.000702645	IGF2	protein_coding

ENSG00000167257.11	879.7979283	0.038259536	0.043748946	0.874524759	0.381832543	0.607107915	RNF214	protein_coding
ENSG00000167258.15	6421.624502	0.047064176	0.072796616	0.646515981	0.517945251	0.718988921	CDK12	protein_coding
ENSG00000167261.14	164.7386209	-0.194643062	0.113928481	-1.708467107	0.087549705	0.2539556	DPEP2	protein_coding
ENSG00000167264.18	643.9258889	-0.18670616	0.054269925	-3.440324657	0.000581017	0.007616034	DUS2	protein_coding
ENSG00000167272.11	1016.640768	-0.196911949	0.057314438	-3.435643035	0.000591149	0.007715719	POP5	protein_coding
ENSG00000167280.17	1457.398412	-0.06216244	0.076378955	-0.81386869	0.415720179	0.636111815	ENGASE	protein_coding
ENSG00000167281.19	112.0801474	-1.004676969	0.231707758	-4.335966032	1.45121E-05	0.000434935	RBFOX3	protein_coding
ENSG00000167283.8	2326.500733	-0.263780842	0.071437635	-3.692463253	0.000222092	0.003684097	ATP5MG	protein_coding
ENSG00000167286.10	562.7751106	-0.655836793	0.129970868	-5.046029178	4.51087E-07	2.73014E-05	CD3D	protein_coding
ENSG00000167291.16	5134.979745	-0.04388685	0.079218975	-0.553994162	0.579582841	0.763658618	TBC1D16	protein_coding
ENSG00000167302.11	496.6647893	0.014482121	0.052679938	0.274907716	0.783387139	0.890154816	TEPSIN	protein_coding
ENSG00000167306.20	6249.333348	0.004926945	0.093256392	0.052832248	0.957865567	0.980362821	MYO5B	protein_coding
ENSG00000167311.14	33.35145276	0.841458908	0.188007579	4.475664812	7.61739E-06	0.000262406	ART5	protein_coding
ENSG00000167315.18	4090.185421	-0.189800075	0.09764345	-1.943807541	0.051918664	0.182235975	ACAA2	protein_coding
ENSG00000167323.11	3522.164255	0.064166166	0.058362188	1.099447577	0.271572888	0.499802844	STIM1	protein_coding
ENSG00000167325.15	4176.420325	-0.087527295	0.063891082	-1.369945427	0.170703938	0.380825347	RRM1	protein_coding
ENSG00000167332.9	18.90025702	0.209001149	0.141369787	1.478400391	0.139300641	0.336472702	OR51E2	protein_coding
ENSG00000167333.13	772.3002282	0.044688644	0.063143403	0.70773259	0.479111335	0.688547256	TRIM68	protein_coding
ENSG00000167346.7	2.217761766	0.160862605	0.394491113	0.407772443	0.683440747	0.832796172	MMP26	protein_coding
ENSG00000167355.8	4.684958338	-0.441335777	0.245295328	-1.799201728	0.071986776	0.223749483	OR51B5	protein_coding
ENSG00000167359.8	0.430272659	0.059223574	0.538658419	0.109946437	0.912451854		OR51I1	protein_coding
ENSG00000167360.6	0.863361238	0.251944117	0.375912185	0.670220672	0.502717128		OR51Q1	protein_coding
ENSG00000167363.14	2108.975596	0.05928708	0.094646016	0.62640862	0.531046957	0.728903046	FN3K	protein_coding
ENSG00000167371.21	116.9066861	-0.043460078	0.098965019	-0.439145851	0.660555858	0.818067029	PRRT2	protein_coding
ENSG00000167377.18	39.96594444	-0.029359063	0.065639579	-0.447276828	0.654675196	0.814448957	ZNF23	protein_coding
ENSG00000167378.8	2403.600187	0.123616315	0.066017473	1.872478744	0.0611404	0.202146466	IRGQ	protein_coding
ENSG00000167380.16	721.0113321	0.026450944	0.065380981	0.404566338	0.685796306	0.834132296	ZNF226	protein_coding
ENSG00000167384.10	568.1915578	-0.066032662	0.056941299	-1.159662023	0.246186438	0.472664511	ZNF180	protein_coding
ENSG00000167390.8	1.132107007	0.088452942	0.359895747	0.245773792	0.805857346	0.903489903	POM121L3P	unprocessed_pseudogene
ENSG00000167393.18	691.3729076	0.036148769	0.073248963	0.493505539	0.621655427	0.793077311	PPP2R3B	protein_coding
ENSG00000167393.18_PAR_Y	0						PPP2R3B	protein_coding
ENSG00000167394.13	325.6613643	0.087816091	0.059404251	1.47827958	0.139332961	0.336515168	ZNF668	protein_coding
ENSG00000167395.10	2030.720832	0.154253019	0.056953981	2.708379935	0.006761257	0.045943443	ZNF646	protein_coding
ENSG00000167397.15	495.6858885	-0.059504147	0.089960989	-0.661443899	0.508327681	0.71196591	VKORC1	protein_coding
ENSG00000167414.4	6.923846924	-0.476021652	0.152653438	-3.118315954	0.001818877	0.017843585	GNG8	protein_coding
ENSG00000167419.11	20.40601173	0.231457627	0.209002243	1.107440874	0.268103362	0.496036183	LPO	protein_coding
ENSG00000167434.10	188.4949116	-0.421322317	0.267143762	-1.577137021	0.114764	0.299017689	CA4	protein_coding
ENSG00000167447.12	811.5862688	0.109523308	0.051532037	2.125344069	0.033557914	0.137043757	SMG8	protein_coding
ENSG00000167459.17	1.835388179	-0.284721851	0.392606836	-0.725208593	0.468324072	0.679824629	LINC00905	lncRNA
ENSG00000167460.17	36714.29241	0.158202517	0.054320391	2.912396515	0.00358667	0.029356936	TPM4	protein_coding
ENSG00000167461.12	2057.449216	-0.1114625	0.046368585	-2.403836553	0.016224025	0.084619312	RAB8A	protein_coding
ENSG00000167468.18	12335.74198	-0.086940848	0.074751267	-1.16306855	0.244801691	0.471085153	GPX4	protein_coding

ENSG00000167470.12	10181.74194	0.274855414	0.061051492	4.502026155	6.73087E-06	0.000238464	MIDN	protein_coding
ENSG00000167476.10	300.7418712	0.018311142	0.179441541	0.102045169	0.918720823	0.961619323	JSRP1	protein_coding
ENSG00000167483.19	170.9695535	-1.186268615	0.201248845	-5.894536275	3.75735E-09	5.54915E-07	NIBAN3	protein_coding
ENSG00000167487.12	821.1997428	-0.019045337	0.062263213	-0.305884268	0.759692764	0.876904431	KLHL26	protein_coding
ENSG00000167491.17	6809.789325	0.042348272	0.051904333	0.81589089	0.414562543	0.634898149	GATAD2A	protein_coding
ENSG00000167494.9	0.821195295	-0.151156917	0.330760881	-0.456997563	0.647672797		NOS2P2	transcribed_unprocessed_pseudogene
ENSG00000167508.12	1938.543512	0.010628986	0.079143807	0.134299656	0.893165625	0.949000419	MVD	protein_coding
ENSG00000167513.9	2237.523195	-0.110750621	0.102684656	-1.07855083	0.280788011	0.509701705	CDT1	protein_coding
ENSG00000167515.10	1899.532901	-0.110058179	0.06091402	-1.806779117	0.070796726	0.221404841	TRAPPC2L	protein_coding
ENSG00000167522.16	10986.39852	0.023833903	0.051826332	0.459880183	0.645602225	0.808749846	ANKRD11	protein_coding
ENSG00000167523.14	603.8960302	-0.00664141	0.073970684	-0.089784356	0.92845858	0.966080218	SPATA33	protein_coding
ENSG00000167524.16	311.4356326	-0.056215541	0.085019191	-0.661210026	0.508477633	0.712033606	RSKR	protein_coding
ENSG00000167525.14	134.2932434	-0.104747023	0.09154771	-1.144179612	0.252549156	0.479305544	PROCA1	protein_coding
ENSG00000167526.14	81152.15078	-0.206704101	0.074402028	-2.778205191	0.005466009	0.03959241	RPL13	protein_coding
ENSG00000167528.13	1293.202613	0.115685457	0.049819548	2.322089645	0.020228109	0.097996699	ZNF641	protein_coding
ENSG00000167531.6	0.94905382	-0.367026504	0.400087834	-0.917364819	0.358951501		LALBA	protein_coding
ENSG00000167535.8	1359.826497	0.379570634	0.068392308	5.549902364	2.85829E-08	2.86995E-06	CACNB3	protein_coding
ENSG00000167536.14	258.8137301	0.050831491	0.093644097	0.542815749	0.587256668	0.769296575	DHRS13	protein_coding
ENSG00000167543.16	2236.253439	0.152479269	0.069263637	2.201433188	0.027705372	0.120799645	TP53I13	protein_coding
ENSG00000167548.16	11032.01534	0.075161037	0.064126955	1.172066206	0.241170477	0.466839279	KMT2D	protein_coding
ENSG00000167549.18	96.49839449	-0.026120713	0.140657809	-0.185703968	0.852676916	0.927953301	CORO6	protein_coding
ENSG00000167550.11	80.6798269	-0.041065714	0.098844967	-0.415455793	0.677808271	0.829300333	RHEBL1	protein_coding
ENSG00000167552.14	7304.803625	0.0745052	0.120641102	0.617577252	0.536854044	0.733083854	TUBA1A	protein_coding
ENSG00000167553.16	11645.39858	0.083211036	0.072516409	1.147478711	0.251183827	0.477779612	TUBA1C	protein_coding
ENSG00000167554.15	87.20990015	-0.099477595	0.128357828	-0.775002173	0.438338376	0.655181327	ZNF610	protein_coding
ENSG00000167555.14	472.5101584	0.184511349	0.137677902	1.340166769	0.180191133	0.393159873	ZNF528	protein_coding
ENSG00000167562.13	549.4704364	0.120604663	0.095829753	1.258530466	0.208199976	0.428145849	ZNF701	protein_coding
ENSG00000167565.13	1180.443378	0.05917341	0.060556924	0.977153493	0.328493168	0.558014532	SERTAD3	protein_coding
ENSG00000167566.17	1685.262187	0.363116261	0.072190923	5.029943463	4.90624E-07	2.90094E-05	NCKAP5L	protein_coding
ENSG00000167578.18	74.36485363	-0.15485753	0.098340065	-1.574714548	0.115322348	0.299844986	RAB4B	protein_coding
ENSG00000167580.8	82.22387949	0.838679643	0.275892416	3.039879291	0.00236673	0.021567333	AQP2	protein_coding
ENSG00000167588.13	1292.997493	-0.465418848	0.187455123	-2.482828113	0.013034397	0.072734674	GPD1	protein_coding
ENSG00000167595.15	1313.832117	0.081894567	0.059027858	1.387388446	0.165323346	0.3732989	PROSER3	protein_coding
ENSG00000167600.14	13864.19711	-0.344256421	0.129031327	-2.668006523	0.007630278	0.050030787	CYP2S1	protein_coding
ENSG00000167601.12	4024.789764	0.423629582	0.107242832	3.950190189	7.80891E-05	0.001637579	AXL	protein_coding
ENSG00000167604.15	432.3796022	-0.183471497	0.066890218	-2.742874839	0.006090389	0.042765211	NFKBID	protein_coding
ENSG00000167608.12	5228.011615	0.026155142	0.09094416	0.287595623	0.773656295	0.885569225	TMC4	protein_coding
ENSG00000167612.13	16.99776487	1.229292665	0.265563029	4.629005289	3.67426E-06	0.000145939	ANKRD33	protein_coding
ENSG00000167613.16	1752.503403	-0.05911518	0.110709588	-0.533966216	0.593364908	0.773397244	LAIR1	protein_coding
ENSG00000167614.14	214.193696	0.345516582	0.173857886	1.987350644	0.046883553	0.170669539	TTYH1	protein_coding
ENSG00000167615.17	8048.900761	0.028926462	0.066638699	0.434079035	0.664231049	0.82051739	LENG8	protein_coding
ENSG00000167617.3	4355.202759	-0.197022274	0.14076012	-1.399702381	0.161602461	0.368255526	CDC42EP5	protein_coding

ENSG00000167618.10	69.88314079	-0.434204416	0.176509705	-2.459946411	0.013895776	0.076034184	LAIR2	protein_coding
ENSG00000167619.13	114.3698361	-0.316656021	0.169973947	-1.862967974	0.062466772	0.205040263	TMEM145	protein_coding
ENSG00000167625.11	1048.011125	0.064630755	0.048142933	1.342476485	0.17944155	0.392369131	ZNF526	protein_coding
ENSG00000167632.18	3685.972693	0.050082061	0.058758895	0.85233157	0.394030089	0.617656644	TRAPPC9	protein_coding
ENSG00000167633.18	9.750141321	0.1270674	0.199397051	0.637258169	0.523956694	0.723157203	KIR3DL1	protein_coding
ENSG00000167634.12	52.39979745	-0.722176181	0.196527541	-3.674681825	0.000238146	0.003889799	NLRP7	protein_coding
ENSG00000167635.12	5573.060125	0.078023494	0.051264915	1.521966708	0.128017423	0.319524605	ZNF146	protein_coding
ENSG00000167637.18	411.5160429	0.103972208	0.064545891	1.610826132	0.107217624	0.286956295	ZNF283	protein_coding
ENSG00000167641.11	969.4562255	-0.132860901	0.164198236	-0.809149385	0.418429225	0.638570011	PPP1R14A	protein_coding
ENSG00000167642.13	9294.696113	0.130153867	0.096445887	1.349501477	0.177175946	0.389426229	SPINT2	protein_coding
ENSG00000167644.12	0						C19orf33	protein_coding
ENSG00000167645.17	2195.80485	0.043508148	0.069104888	0.629595812	0.528959065	0.727165795	YIF1B	protein_coding
ENSG00000167646.14	66.2335429	0.470168834	0.145945395	3.22153936	0.001275039	0.013814211	DNAAF3	protein_coding
ENSG00000167653.5	9702.021362	0.369162196	0.278158073	1.32716693	0.184453469	0.398627694	PSCA	protein_coding
ENSG00000167654.18	22.62207184	-0.129385388	0.193425426	-0.668916132	0.503548979	0.708016665	ATCAY	protein_coding
ENSG00000167656.5	346.2152649	1.060149293	0.320320417	3.309652572	0.000934118	0.010889801	LY6D	protein_coding
ENSG00000167657.14	2815.993619	0.088218403	0.060332245	1.462209844	0.143683715	0.342953675	DAPK3	protein_coding
ENSG00000167658.16	153758.9901	-0.001758731	0.065100673	-0.027015556	0.978447326	0.990353068	EEF2	protein_coding
ENSG00000167664.8	45.0794822	-0.586644525	0.141305627	-4.151600592	3.30158E-05	0.000824015	TMIGD2	protein_coding
ENSG00000167670.16	1625.092747	-0.084599593	0.070039352	-1.207886571	0.227090896	0.450547295	CHAF1A	protein_coding
ENSG00000167671.12	3199.294739	0.021346937	0.050641858	0.421527521	0.67336992	0.82634303	UBXN6	protein_coding
ENSG00000167674.15	2520.720492	0.033722334	0.052967915	0.63665586	0.524349031	0.723512359	HDGFL2	protein_coding
ENSG00000167676.4	1550.948694	-0.306818633	0.240000455	-1.27840855	0.201105411	0.41981176	PLIN4	protein_coding
ENSG00000167680.17	1279.09913	0.307536682	0.099377439	3.094632787	0.001970567	0.01891024	SEMA6B	protein_coding
ENSG00000167685.15	2557.706999	-0.071857997	0.073710556	-0.974867117	0.329626179	0.559154502	ZNF444	protein_coding
ENSG00000167693.17	2318.205479	0.340369099	0.113004489	3.011996265	0.002595358	0.023085918	NXN	protein_coding
ENSG00000167695.15	2587.645612	-0.029081023	0.074983096	-0.387834385	0.698138598	0.841105493	TLCD3A	protein_coding
ENSG00000167699.13	3191.012083	-0.046146191	0.053348709	-0.864991701	0.387043344	0.611633063	GLOD4	protein_coding
ENSG00000167700.9	1268.285328	-0.074499579	0.08900085	-0.837065924	0.402555513	0.624453994	MFSD3	protein_coding
ENSG00000167701.14	417.9967773	-0.14537265	0.172430855	-0.843077942	0.399184854	0.622037489	GPT	protein_coding
ENSG00000167702.13	1114.243112	0.020661367	0.095307429	0.216786536	0.828374705	0.915567619	KIFC2	protein_coding
ENSG00000167703.15	3256.620056	-0.025620777	0.076771787	-0.333726469	0.738585974	0.864730329	SLC43A2	protein_coding
ENSG00000167705.12	893.1667238	-0.14982398	0.083660182	-1.790863666	0.073315176	0.226505169	RILP	protein_coding
ENSG00000167711.14	248.2450858	0.598470667	0.158507754	3.775655451	0.000159587	0.002858341	SERPINF2	protein_coding
ENSG00000167716.19	2533.381983	0.148244068	0.063002592	2.352983649	0.018623453	0.092515967	WDR81	protein_coding
ENSG00000167720.13	211.819546	-0.146504822	0.063424289	-2.309916665	0.020892768	0.100147856	SRR	protein_coding
ENSG00000167721.11	2139.562049	0.090588454	0.056104527	1.614637153	0.106389295	0.285686856	TSR1	protein_coding
ENSG00000167723.15	208.7535907	-0.078467467	0.155433745	-0.504829029	0.613678925	0.787956867	TRPV3	protein_coding
ENSG00000167733.14	207.9790717	0.437358142	0.108623384	4.02637192	5.66441E-05	0.001267135	HSD11B1L	protein_coding
ENSG00000167740.10	741.6565734	-0.003006358	0.05695449	-0.052785262	0.957903004	0.980370963	CYB5D2	protein_coding
ENSG00000167741.11	1727.254776	-0.238849398	0.214171348	-1.115225731	0.264753702	0.492557404	GGT6	protein_coding
ENSG00000167747.15	3750.935246	-0.148385641	0.090185971	-1.645329533	0.099901873	0.275237018	C19orf48	transcribed_unitary_pseudogene

ENSG00000167748.11	1410.077388	-0.061187725	0.208507814	-0.293455307	0.769174156	0.883098426	KLK1	protein_coding
ENSG00000167749.11	22.84712414	0.402210797	0.26124048	1.539618962	0.123653261	0.313066698	KLK4	protein_coding
ENSG00000167751.13	10.9798466	0.155846764	0.217061186	0.71798541	0.472766283	0.683648127	KLK2	protein_coding
ENSG00000167754.13	47.78126509	2.262869903	0.356405098	6.349151327	2.16506E-10	5.24149E-08	KLK5	protein_coding
ENSG00000167755.15	2231.717228	1.328048791	0.278778911	4.763806507	1.89975E-06	8.62347E-05	KLK6	protein_coding
ENSG00000167757.14	1739.18648	1.281949305	0.230984047	5.549947383	2.85756E-08	2.86995E-06	KLK11	protein_coding
ENSG00000167759.13	344.8477652	0.857149749	0.260099809	3.295464737	0.00098259	0.011327603	KLK13	protein_coding
ENSG00000167766.18	1639.804762	0.040808817	0.093804027	0.435043333	0.663530974	0.820042237	ZNF83	protein_coding
ENSG00000167767.14	2085.625637	0.739029417	0.171521288	4.308674604	1.64236E-05	0.000477127	KRT80	protein_coding
ENSG00000167768.4	20.07879115	-0.414031212	0.220643051	-1.876475194	0.060590058	0.200953874	KRT1	protein_coding
ENSG00000167769.5	7.495149251	-0.414631804	0.199277209	-2.080678499	0.037463343	0.147409328	ACER1	protein_coding
ENSG00000167770.11	7913.968152	0.043291031	0.052536324	0.824020948	0.409927655	0.630872751	OTUB1	protein_coding
ENSG00000167771.6	501.1468539	0.514137341	0.137347187	3.74334088	0.00018159	0.003168139	RCOR2	protein_coding
ENSG00000167772.12	767.2463697	0.56992609	0.147139448	3.87337385	0.000107339	0.00209191	ANGPTL4	protein_coding
ENSG00000167774.2	0.721234848	0.119416129	0.236241961	0.505482297	0.613220129		AC010323.1	protein_coding
ENSG00000167775.11	1931.080056	-0.136103582	0.083390629	-1.632120819	0.102654037	0.279874452	CD320	protein_coding
ENSG00000167778.9	3680.651974	0.070367012	0.056380159	1.248081116	0.212001353	0.43256932	SPRYD3	protein_coding
ENSG00000167779.9	1118.552984	0.593720179	0.128612385	4.616353068	3.90542E-06	0.000153183	IGFBP6	protein_coding
ENSG00000167780.12	45.17836153	0.109754417	0.257604379	0.426058041	0.670065554	0.82407492	SOAT2	protein_coding
ENSG00000167785.9	949.0864696	-0.001916018	0.072396237	-0.026465714	0.978885881	0.990569863	ZNF558	protein_coding
ENSG00000167791.14	1.011746434	0.131423305	0.334320162	0.393106128	0.694241098	0.838658951	CABP2	protein_coding
ENSG00000167792.13	6673.478956	-0.131238587	0.060360775	-2.174236298	0.029687394	0.12670789	NDUFV1	protein_coding
ENSG00000167797.8	7033.307937	-0.051807288	0.079124195	-0.654759116	0.512622857	0.715189067	CDK2AP2	protein_coding
ENSG00000167798.17	124.7511548	0.619149061	0.257216001	2.407117199	0.016079009	0.084064184	C3P1	transcribed_unprocessed_pseudogene
ENSG00000167799.10	804.6543926	-0.329948641	0.106508767	-3.09785429	0.001949272	0.018763739	NUDT8	protein_coding
ENSG00000167800.9	73.36628188	-0.172748551	0.215602605	-0.801235918	0.422995081	0.6421075	TBX10	protein_coding
ENSG00000167807.15	0.960167039	-0.120893184	0.209089331	-0.578189156	0.563136419		AC011511.1	protein_coding
ENSG00000167815.12	6604.889419	-0.060001686	0.078455612	-0.764785142	0.444399497	0.66033393	PRDX2	protein_coding
ENSG00000167822.3	0.598184885	0.107011466	0.476787025	0.224442907	0.822412687		OR8J3	protein_coding
ENSG00000167825.3	0.232726863	-0.20188639	0.965122788	-0.209182077	0.834306103		OR5I1	protein_coding
ENSG00000167840.13	480.1564308	-0.081484158	0.061208783	-1.331249424	0.183106954	0.396957949	ZNF232	protein_coding
ENSG00000167842.16	1000.938512	0.022407973	0.045465683	0.492854652	0.622115291	0.793430053	MIS12	protein_coding
ENSG00000167850.4	100.6719136	0.140239727	0.121188645	1.157201873	0.247189893	0.473777004	CD300C	protein_coding
ENSG00000167851.15	470.6691894	0.059546128	0.109224373	0.545172537	0.585634857	0.767963955	CD300A	protein_coding
ENSG00000167858.13	7.352635253	0.401231607	0.19540616	2.05332118	0.040041438	0.154087402	TEKT1	protein_coding
ENSG00000167861.16	9377.217696	0.091015975	0.108507378	0.838799877	0.40158162	0.623695175	HID1	protein_coding
ENSG00000167862.10	1397.752591	-0.044213018	0.059883258	-0.738320186	0.460319903	0.673427206	MRPL58	protein_coding
ENSG00000167863.12	3754.168781	-0.136381053	0.061474658	-2.21849225	0.026521287	0.117462968	ATP5PD	protein_coding
ENSG00000167874.7	253.4840608	-0.211746451	0.117085921	-1.808470643	0.070533282	0.220854266	TMEM88	protein_coding
ENSG00000167880.8	5815.855354	0.485527709	0.120437291	4.031373556	5.54518E-05	0.001244938	EVPL	protein_coding
ENSG00000167881.15	5031.21129	0.088394701	0.044017254	2.008183	0.044623849	0.165354886	SRP68	protein_coding
ENSG00000167889.13	89.53682145	1.318674182	0.189061945	6.974826066	3.06249E-12	1.53396E-09	MGAT5B	protein_coding

ENSG00000167895.15	1407.555301	-0.397546711	0.11168271	-3.559608372	0.000371408	0.00544578	TMC8	protein_coding
ENSG00000167900.12	4921.362244	-0.04193224	0.096563504	-0.434245216	0.664110382	0.82051739	TK1	protein_coding
ENSG00000167904.15	1594.565748	-0.142637794	0.062521075	-2.281435389	0.022522696	0.105330706	TMEM68	protein_coding
ENSG00000167910.4	7.713746088	-1.197841252	0.233289894	-5.134561252	2.82803E-07	1.8729E-05	CYP7A1	protein_coding
ENSG00000167912.6	111.3645634	-0.079440667	0.138469271	-0.573706109	0.566166694	0.754375356	AC090152.1	lncRNA
ENSG00000167914.12	179.7098776	-0.413425028	0.180834915	-2.286201353	0.022242482	0.104367462	GSDMA	protein_coding
ENSG00000167916.5	12.44039535	-0.569208006	0.360199216	-1.580258871	0.114047595	0.297899181	KRT24	protein_coding
ENSG00000167920.10	539.0129468	-0.24312841	0.111311839	-2.184209807	0.028946835	0.124584317	TMEM99	lncRNA
ENSG00000167925.16	2278.374506	-0.05651523	0.066805262	-0.845969739	0.397569626	0.620774855	GHDC	protein_coding
ENSG00000167930.17	7256.409413	0.02749628	0.074456339	0.369294015	0.711908586	0.849359082	FAM234A	protein_coding
ENSG00000167941.3	5.594164834	0.092609371	0.23432715	0.395214004	0.692684959	0.83773128	SOST	protein_coding
ENSG00000167962.14	3518.082184	0.028693641	0.064022453	0.448180912	0.654022638	0.814017055	ZNF598	protein_coding
ENSG00000167964.13	162.1897965	0.065457921	0.139824952	0.468141915	0.639683107	0.804951509	RAB26	protein_coding
ENSG00000167965.18	2455.527756	-0.058141284	0.061051678	-0.952329017	0.340930146	0.570939325	MLST8	protein_coding
ENSG00000167967.16	1473.778205	0.071441068	0.047801716	1.494529363	0.135037313	0.330386896	E4F1	protein_coding
ENSG00000167968.13	37.15063845	0.066301583	0.115014998	0.576460323	0.564304081	0.753067892	DNASE1L2	protein_coding
ENSG00000167969.13	2733.410959	-0.143665706	0.069659897	-2.062387572	0.039170852	0.151958355	ECI1	protein_coding
ENSG00000167971.16	69.74721584	0.753430949	0.201936759	3.731024273	0.000190703	0.003279061	CASKIN1	protein_coding
ENSG00000167972.14	1424.929317	0.322420152	0.145048674	2.222841086	0.026226518	0.116591722	ABCA3	protein_coding
ENSG00000167977.9	2939.432287	-0.029350252	0.061989922	-0.473468132	0.635879232	0.80257514	KCTD5	protein_coding
ENSG00000167978.17	57123.53342	0.021866004	0.051051509	0.428312593	0.668423551	0.822977105	SRRM2	protein_coding
ENSG00000167981.7	403.8252602	-0.160053003	0.090482279	-1.768887844	0.076912593	0.234084375	ZNF597	protein_coding
ENSG00000167984.18	626.0429889	-0.621222674	0.114010222	-5.448833117	5.07014E-08	4.66121E-06	NLRC3	protein_coding
ENSG00000167985.7	644.0524981	-0.038722903	0.054771435	-0.706990847	0.479572166	0.688864457	SDHAF2	protein_coding
ENSG00000167986.14	14272.25446	0.1484084	0.046922132	3.162865665	0.001562244	0.015984482	DDB1	protein_coding
ENSG00000167987.11	2339.229245	0.12122205	0.052428718	2.312130752	0.020770479	0.099693266	VPS37C	protein_coding
ENSG00000167992.13	138.2768561	0.248637071	0.169333928	1.468324005	0.142016233	0.340446771	VWCE	protein_coding
ENSG00000167994.13	772.9896742	0.368829665	0.104296457	3.536358542	0.000405684	0.005809556	RAB3IL1	protein_coding
ENSG00000167995.17	176.5377202	0.043717566	0.09300857	0.470038048	0.638327835	0.804244718	BEST1	protein_coding
ENSG00000167996.16	30927.28403	0.12761042	0.108387714	1.177351336	0.239055305	0.464427861	FTH1	protein_coding
ENSG00000168000.14	125.1961197	0.017061858	0.074730091	0.228313085	0.819402853	0.910988828	BSCL2	protein_coding
ENSG00000168002.12	2808.475001	-0.100893438	0.05184844	-1.945930066	0.051663141	0.181719645	POLR2G	protein_coding
ENSG00000168003.17	7762.315327	0.090993025	0.071699134	1.26909518	0.204407119	0.423862151	SLC3A2	protein_coding
ENSG00000168004.10	257.6028788	0.564734025	0.213343145	2.647068996	0.008119279	0.052316103	PLAAT5	protein_coding
ENSG00000168005.9	1290.865163	0.080720427	0.076088529	1.060875107	0.288746664	0.518510398	SPINDOC	protein_coding
ENSG00000168010.11	940.6158975	-0.070854221	0.073844741	-0.959502606	0.337305607	0.567038286	ATG16L2	protein_coding
ENSG00000168014.17	2425.523837	0.108445846	0.049166324	2.205693616	0.027405459	0.120085238	C2CD3	protein_coding
ENSG00000168016.15	2585.131056	-0.007361957	0.089322942	-0.082419553	0.934313088	0.969059612	TRANK1	protein_coding
ENSG00000168026.18	228.3171024	-0.017904994	0.076046698	-0.235447354	0.813861536	0.908061527	TTC21A	protein_coding
ENSG00000168028.14	36560.19435	-0.070516564	0.068045433	-1.0363159	0.300054791	0.529672188	RPSA	protein_coding
ENSG00000168032.10	312.0188292	0.458300565	0.190583767	2.404719833	0.016184869	0.084435295	ENTPD3	protein_coding
ENSG00000168036.18	22500.00002	0.117543929	0.071921488	1.634336718	0.102188168	0.278964413	CTNNB1	protein_coding

ENSG00000168038.11	315.0946241	-0.102412372	0.063271709	-1.618612396	0.105530686	0.28434494	ULK4	protein_coding
ENSG00000168040.5	551.1682276	0.269337295	0.088892793	3.029911485	0.002446254	0.02207048	FADD	protein_coding
ENSG00000168056.17	8671.781272	0.30028752	0.100731771	2.981060664	0.002872519	0.024831532	LTBP3	protein_coding
ENSG00000168060.16	302.417886	0.164974319	0.13665547	1.207228068	0.227344324	0.450702555	NAALADL1	protein_coding
ENSG00000168061.17	881.9400491	-0.024333339	0.079944742	-0.304376972	0.76084071	0.877538394	SAC3D1	protein_coding
ENSG00000168062.10	1609.038176	0.060531254	0.136159489	0.44456141	0.656636741	0.815516853	BATF2	protein_coding
ENSG00000168065.16	42.50080906	-0.982291509	0.254577239	-3.858520556	0.000114075	0.002190862	SLC22A11	protein_coding
ENSG00000168066.20	14691.68329	-0.03108665	0.034238437	-0.907945964	0.363906771	0.59114163	SF1	protein_coding
ENSG00000168067.12	1489.671491	0.043972552	0.075597632	0.58166573	0.560791859	0.750126676	MAP4K2	protein_coding
ENSG00000168070.12	15.01630475	-0.573807294	0.212007097	-2.706547577	0.006798684	0.04609713	MAJIN	protein_coding
ENSG00000168071.22	1271.108081	-0.281226068	0.106537983	-2.639678931	0.00829846	0.053056534	CCDC88B	protein_coding
ENSG00000168077.14	1592.028439	-0.075604968	0.145153846	-0.520860937	0.60246365	0.780438311	SCARA3	protein_coding
ENSG00000168078.10	1550.272594	-0.10913778	0.112919195	-0.96651221	0.33378794	0.563310506	PBK	protein_coding
ENSG00000168079.17	402.7665989	-0.054044404	0.203514664	-0.265555332	0.790581691	0.894859044	SCARA5	protein_coding
ENSG00000168081.9	55.68895928	-1.004185712	0.171758043	-5.846513456	5.01983E-09	6.8789E-07	PNOC	protein_coding
ENSG00000168090.10	5659.194891	-0.096343936	0.06474518	-1.488048001	0.136738235	0.332773488	COPS6	protein_coding
ENSG00000168092.14	6696.72494	0.129614422	0.04297882	3.01577435	0.002563239	0.02287493	PAFAH1B2	protein_coding
ENSG00000168096.15	648.0162233	0.033734117	0.054479616	0.619206211	0.535780521	0.732428795	ANKS3	protein_coding
ENSG00000168101.14	1059.077615	-0.060742109	0.059080112	-1.028131249	0.303888093	0.533284932	NUDT16L1	protein_coding
ENSG00000168116.14	331.5340502	0.371964047	0.090599908	4.105567619	4.03323E-05	0.000967288	KIAA1586	protein_coding
ENSG00000168118.12	2568.064861	-0.119066542	0.049335109	-2.413424118	0.015803418	0.083001754	RAB4A	protein_coding
ENSG00000168122.4	0.687024097	0.095882052	0.396974693	0.241531902	0.809142894		ZNF355P	unprocessed_pseudogene
ENSG00000168124.2	2.343551748	0.780555748	0.336690185	2.318320471	0.020431911	0.098592449	OR1F1	protein_coding
ENSG00000168126.3	5.7274191	-0.407750529	0.215879238	-1.888789922	0.058919984	0.197549906	OR2W6P	unprocessed_pseudogene
ENSG00000168129.8	0.439421199	-0.404384546	0.326682338	-1.237852492	0.215770761		DHFRP3	processed_pseudogene
ENSG00000168131.4	2.470866346	-0.287286089	0.263872377	-1.088731198	0.27627244	0.505032981	OR2B2	protein_coding
ENSG00000168135.4	7.935917577	-0.299474798	0.256163325	-1.169077572	0.242372379	0.468250628	KCNJ4	protein_coding
ENSG00000168137.18	6484.446355	0.098255849	0.045382251	2.165072185	0.030382158	0.128637281	SETD5	protein_coding
ENSG00000168140.5	1658.878171	0.588717378	0.098776752	5.960080344	2.52114E-09	3.93777E-07	VASN	protein_coding
ENSG00000168143.9	1396.444955	0.191189762	0.124401115	1.536881417	0.124322351	0.314008873	FAM83B	protein_coding
ENSG00000168148.4	4.798286962	0.190635626	0.154902304	1.230682964	0.218441462	0.44004362	H3-4	protein_coding
ENSG00000168152.13	258.7440147	-0.063394335	0.050921296	-1.244947404	0.213151084	0.433777836	THAP9	protein_coding
ENSG00000168158.4	5.330230405	-0.336945115	0.186691674	-1.804821334	0.071102645	0.221935388	OR2C1	protein_coding
ENSG00000168159.14	5756.130268	0.007098572	0.055393649	0.128147755	0.898032044	0.951552361	RNF187	protein_coding
ENSG00000168172.9	5714.129895	-0.021400746	0.074898919	-0.285728375	0.77508617	0.886111972	HOOK3	protein_coding
ENSG00000168175.15	8452.651669	0.085057698	0.041538796	2.047668852	0.040592458	0.155370886	MAPK1IP1L	protein_coding
ENSG00000168209.5	2340.262356	0.362687818	0.114847135	3.158004926	0.001588529	0.016185017	DDIT4	protein_coding
ENSG00000168214.20	6259.66598	-0.01284209	0.041431094	-0.309962605	0.756589394	0.875159823	RBPJ	protein_coding
ENSG00000168216.13	3443.43781	-0.025108904	0.050274681	-0.499434376	0.617473407	0.79029958	LMBRD1	protein_coding
ENSG00000168228.15	455.001121	-0.044472537	0.047002866	-0.946166503	0.344063668	0.573773008	ZCCHC4	protein_coding
ENSG00000168229.4	50.18471442	-0.13078717	0.139170351	-0.93976317	0.347339054	0.57656317	PTGDR	protein_coding
ENSG00000168234.13	1108.389985	-0.167740877	0.077135861	-2.17461599	0.029658905	0.126620894	TTC39C	protein_coding

ENSG00000168237.18	869.3396544	-0.239852582	0.097563877	-2.458415862	0.013955147	0.076272852	GLYCTK	protein_coding
ENSG00000168243.11	587.8388388	-0.194727179	0.234863674	-0.829107268	0.407043712	0.628185784	GNG4	protein_coding
ENSG00000168246.6	1806.956839	0.095392424	0.041509136	2.298106699	0.021555717	0.102179211	UBTD2	protein_coding
ENSG00000168255.20	61.04966172	-0.097049243	0.099698885	-0.973423552	0.330342838	0.559888452	POLR2J3	protein_coding
ENSG00000168256.18	3230.578901	0.135019628	0.052756514	2.559297752	0.010488387	0.062336664	NKIRAS2	protein_coding
ENSG00000168259.17	3182.259271	-0.02334488	0.042658978	-0.547244244	0.584210943	0.767080364	DNAJC7	protein_coding
ENSG00000168263.9	9.788763255	0.316777081	0.144385828	2.193962423	0.028238111	0.122341535	KCNV2	protein_coding
ENSG00000168264.10	13065.03585	0.109145534	0.050734959	2.151288503	0.031453435	0.131622133	IRF2BP2	protein_coding
ENSG00000168267.7	5.311935501	-1.442605288	0.283065757	-5.096361012	3.46245E-07	2.18671E-05	PTF1A	protein_coding
ENSG00000168268.11	3983.868499	0.219885237	0.082595463	2.662195103	0.007763287	0.050666578	NT5DC2	protein_coding
ENSG00000168269.10	20.60168401	0.131237761	0.275470407	0.476413284	0.633779972	0.801364608	FOXI1	protein_coding
ENSG00000168273.8	612.8260852	-0.139942234	0.073697976	-1.898861284	0.057582717	0.194679705	SMIM4	protein_coding
ENSG00000168275.16	1116.728291	-0.1316062	0.068716887	-1.915194427	0.055467713	0.190114561	COA6	protein_coding
ENSG00000168280.18	189.7400513	-0.141778157	0.126390517	-1.121746797	0.261970113	0.489501125	KIF5C	protein_coding
ENSG00000168282.6	49.62179878	0.124260634	0.108889659	1.141161013	0.253802925	0.480596622	MGAT2	protein_coding
ENSG00000168283.14	2796.367796	-0.019736296	0.058865672	-0.33527683	0.737416271	0.864009324	BMI1	protein_coding
ENSG00000168286.3	1354.830507	0.004869558	0.046549701	0.104609856	0.916685393	0.960457777	THAP11	protein_coding
ENSG00000168288.13	4266.617403	0.025862645	0.045288685	0.571061963	0.567957641	0.755398917	MMADHC	protein_coding
ENSG00000168291.13	1952.45771	-0.016330564	0.046294959	-0.352750371	0.724275593	0.856074577	PDHB	protein_coding
ENSG00000168297.16	1351.338973	-0.263214064	0.063047393	-4.17486038	2.98169E-05	0.000759398	PXK	protein_coding
ENSG00000168298.7	258.8462773	-0.289601716	0.14335794	-2.020130281	0.043369876	0.162358148	H1-4	protein_coding
ENSG00000168300.14	3469.768502	-0.042415933	0.060050869	-0.706333371	0.479980846	0.689186639	PCMTD1	protein_coding
ENSG00000168301.13	422.238023	-0.026442027	0.057797482	-0.457494453	0.647315689	0.809673823	KCTD6	protein_coding
ENSG00000168303.9	1669.094657	-0.094487839	0.057973064	-1.629857592	0.103131598	0.28062227	MPLKIP	protein_coding
ENSG00000168306.13	405.4316558	-0.324092388	0.136071777	-2.381775226	0.01722941	0.087895811	ACOX2	protein_coding
ENSG00000168309.18	521.5848368	-0.483922298	0.150370879	-3.21819159	0.001290016	0.013924953	FAM107A	protein_coding
ENSG00000168310.11	3144.181403	-0.126408144	0.047252405	-2.675168478	0.00746917	0.049241156	IRF2	protein_coding
ENSG00000168314.17	11.23684528	0.012939422	0.172486908	0.075016837	0.940201316	0.971745016	MOBP	protein_coding
ENSG00000168329.14	194.5017567	-0.229855789	0.160434383	-1.432709025	0.151941009	0.354716241	CX3CR1	protein_coding
ENSG00000168333.14	2.72443741	0.384289646	0.430821571	0.891992585	0.372396907	0.59890867	PPDPFL	protein_coding
ENSG00000168334.9	90.3431388	0.913958304	0.182933433	4.996125044	5.84937E-07	3.36721E-05	XIRP1	protein_coding
ENSG00000168348.4	2.959810286	0.100006403	0.204448402	0.489152285	0.624733888	0.795177508	INSM2	protein_coding
ENSG00000168350.8	2877.885738	-0.390627945	0.138493278	-2.820555272	0.004794061	0.036165683	DEGS2	protein_coding
ENSG00000168356.13	34.14879458	-0.358090424	0.167087263	-2.143134172	0.032102326	0.133235647	SCN11A	protein_coding
ENSG00000168367.11	1.270984364	0.298260328	0.362553134	0.822666526	0.410697656	0.631509236	LINC00917	lncRNA
ENSG00000168374.11	15468.87431	0.204595643	0.056847486	3.599027109	0.00031941	0.004873573	ARF4	protein_coding
ENSG00000168385.18	20932.74171	-0.019647765	0.038089302	-0.515834214	0.605970205	0.782749059	SEPTIN2	protein_coding
ENSG00000168386.18	6514.117035	-0.103651252	0.114233524	-0.907362813	0.364214968	0.591443847	FILIP1L	protein_coding
ENSG00000168389.18	845.9364135	0.165988852	0.118220678	1.404059373	0.16030117	0.366324615	MFSD2A	protein_coding
ENSG00000168393.13	1974.367321	-0.161058763	0.072353448	-2.225999836	0.026014192	0.115962566	DTYMK	protein_coding
ENSG00000168394.11	12051.26143	0.007105774	0.110382024	0.064374377	0.948672132	0.975927419	TAP1	protein_coding
ENSG00000168395.16	1433.318528	-0.163489558	0.054087548	-3.022683876	0.002505438	0.022474162	ING5	protein_coding

ENSG00000168397.17	2881.755703	-0.056475795	0.039971465	-1.412902805	0.157684304	0.362837096	ATG4B	protein_coding
ENSG00000168398.7	1103.281313	0.197473574	0.104053804	1.897802546	0.057722098	0.195019061	BDKRB2	protein_coding
ENSG00000168404.13	1651.360685	0.043671269	0.06535583	0.668207706	0.504001017	0.708250639	MLKL	protein_coding
ENSG00000168405.17	482.4967684	-0.172863104	0.110702464	-1.5615109	0.118403254	0.304818255	CMAHP	transcribed_unitary_pseudogene
ENSG00000168411.14	3085.655288	0.038736946	0.059241086	0.653886497	0.513184934	0.715588822	RFWD3	protein_coding
ENSG00000168412.6	24.9524889	0.584576934	0.237529728	2.461068513	0.013852391	0.075897176	MTNR1A	protein_coding
ENSG00000168418.7	5.148414423	-0.395282891	0.256580255	-1.540581879	0.123418581	0.312614015	KCNG4	protein_coding
ENSG00000168421.13	565.2269303	-0.68968354	0.127730293	-5.399529918	6.68157E-08	5.93422E-06	RHOH	protein_coding
ENSG00000168427.9	62.50669483	0.593590375	0.142479938	4.166133032	3.0981E-05	0.000781736	KLHL30	protein_coding
ENSG00000168434.13	1739.376154	0.077347283	0.041207176	1.877034319	0.06051339	0.200849555	COG7	protein_coding
ENSG00000168438.15	1585.430305	0.005709214	0.050125566	0.113898237	0.909318463	0.957088321	CDC40	protein_coding
ENSG00000168439.17	10285.69996	0.019612819	0.059517501	0.32953028	0.74175491	0.866756782	STIP1	protein_coding
ENSG00000168447.11	131.0882794	-0.107228553	0.19743684	-0.543103066	0.587058841	0.769137788	SCNN1B	protein_coding
ENSG00000168453.15	4274.889817	0.141265967	0.133097626	1.061371049	0.288521308	0.51832542	HR	protein_coding
ENSG00000168454.12	9.387064247	-0.183655667	0.160611208	-1.14347977	0.252839449	0.479688624	TXNDC2	protein_coding
ENSG00000168461.13	5136.91821	0.290101384	0.101742554	2.851327909	0.004353705	0.033698349	RAB31	protein_coding
ENSG00000168476.12	2642.573069	0.0141309	0.079903221	0.176850194	0.859626061	0.931723041	REEP4	protein_coding
ENSG00000168477.19	3288.07915	0.070841006	0.17441807	0.406156343	0.684627729	0.83356214	TNXB	protein_coding
ENSG00000168481.9	19.45776521	0.398622037	0.217199097	1.835284044	0.066463614	0.212821288	LGI3	protein_coding
ENSG00000168484.12	2.620173066	-0.89079528	0.325513206	-2.736587223	0.006208014	0.043284257	SFTPC	protein_coding
ENSG00000168487.20	3326.682395	0.469646066	0.069842475	6.724361741	1.76364E-11	6.29951E-09	BMP1	protein_coding
ENSG00000168488.18	11395.07534	0.06608969	0.049402052	1.337792421	0.180964113	0.394124008	ATXN2L	protein_coding
ENSG00000168490.14	139.4026376	0.488524003	0.133838628	3.650097213	0.000262141	0.00419307	PHYHIP	protein_coding
ENSG00000168491.10	69.0959573	0.110317836	0.14024023	0.786634735	0.431495719	0.649441818	CCDC110	protein_coding
ENSG00000168495.13	1643.648957	0.07999795	0.05122351	1.561742843	0.118348582	0.304695506	POLR3D	protein_coding
ENSG00000168496.4	2270.572447	0.036835649	0.073210135	0.503149577	0.614859113	0.788602281	FEN1	protein_coding
ENSG00000168497.5	1224.008007	-0.385047532	0.149632915	-2.573280971	0.010073939	0.0607938	CAVIN2	protein_coding
ENSG00000168502.17	2153.87791	0.036020662	0.116904996	0.308119096	0.757991711	0.876037788	MTCL1	protein_coding
ENSG00000168505.7	7.875377045	0.374551829	0.213331938	1.755723185	0.079135686	0.238580618	GBX2	protein_coding
ENSG00000168509.20	3.836894871	-0.372120342	0.220299489	-1.689156628	0.091189418	0.260259359	HJV	protein_coding
ENSG00000168515.3	0.477934045	0.850632302	0.701175546	1.213151694	0.225071829		SCGB1D1	protein_coding
ENSG00000168517.10	122.2014667	-0.091095672	0.072527796	-1.256010475	0.209112172	0.429128232	HEXIM2	protein_coding
ENSG00000168522.13	2888.113595	-0.04878171	0.058239468	-0.837605692	0.402252195	0.624183442	FNTA	protein_coding
ENSG00000168528.12	21038.9687	0.129410818	0.104776786	1.235109636	0.216789704	0.438149738	SERINC2	protein_coding
ENSG00000168530.16	1.065314473	-0.330568433	0.4245161	-0.778694689	0.436159586	0.65372932	MYL1	protein_coding
ENSG00000168538.16	2399.383403	-0.038696052	0.046628497	-0.829879879	0.406606702	0.627931636	TRAPPC11	protein_coding
ENSG00000168539.4	47.73014556	0.439928929	0.215223264	2.04405844	0.040947774	0.15619583	CHRM1	protein_coding
ENSG00000168542.16	143490.7567	0.759225802	0.124844561	6.081368652	1.19161E-09	2.06059E-07	COL3A1	protein_coding
ENSG00000168546.11	75.1545899	-0.10366934	0.155116571	-0.668331819	0.503921807	0.708250639	GFRA2	protein_coding
ENSG00000168556.7	549.8714453	0.031149872	0.056711021	0.549273697	0.582817636	0.766273063	ING2	protein_coding
ENSG00000168564.6	1632.155192	-0.001594899	0.058620418	-0.027207233	0.978294447	0.99033933	CDKN2AIP	protein_coding
ENSG00000168566.13	1324.343155	-0.012704799	0.063808033	-0.19910971	0.842176926	0.922423564	SNRNP48	protein_coding

ENSG00000168569.8	1024.68581	0.009156429	0.060166527	0.152184767	0.879041208	0.941618232	TMEM223	protein_coding
ENSG00000168575.10	3235.493823	0.124739882	0.06481238	1.924630463	0.054275606	0.187562892	SLC20A2	protein_coding
ENSG00000168582.5	1.51792083	-0.270559155	0.443792009	-0.609653058	0.542091657	0.737196609	CRYGA	protein_coding
ENSG00000168589.15	7.453130504	0.253990532	0.171554142	1.480526954	0.138732673	0.3356217	DYNLRB2	protein_coding
ENSG00000168591.16	2340.962823	0.127016977	0.043671749	2.908447214	0.003632285	0.029572658	TMUB2	protein_coding
ENSG00000168594.15	4.970239556	-0.668896256	0.252402127	-2.650121307	0.008046287	0.051984147	ADAM29	protein_coding
ENSG00000168610.16	17986.55693	0.042722844	0.048988032	0.872107798	0.383149572	0.608096621	STAT3	protein_coding
ENSG00000168612.4	1500.419008	-0.029625528	0.054225803	-0.546336358	0.584834748	0.767490556	ZSWIM1	protein_coding
ENSG00000168615.13	15341.31248	0.219555412	0.096531377	2.274446094	0.022939179	0.106684901	ADAM9	protein_coding
ENSG00000168619.16	3.241343195	0.195299506	0.352974267	0.553296724	0.580060244	0.764010435	ADAM18	protein_coding
ENSG00000168621.15	72.00667982	-0.622120938	0.152584429	-4.077224273	4.55765E-05	0.00106607	GDNF	protein_coding
ENSG00000168630.3	0.879938749	0.337129746	0.342331223	0.984805717	0.324719512		SPINT5P	processed_pseudogene
ENSG00000168631.13	16864.51736	0.075010233	0.297940779	0.251762223	0.801224858	0.900961878	MUCL3	protein_coding
ENSG00000168634.5	4.92886751	0.300171363	0.204099259	1.470712652	0.141368843	0.339417635	WFDC13	protein_coding
ENSG00000168646.13	2023.571121	-1.003150181	0.173256507	-5.789971172	7.03985E-09	8.89482E-07	AXIN2	protein_coding
ENSG00000168653.11	6358.644291	-0.189064079	0.06406073	-2.951325679	0.003164131	0.026742307	NDUFS5	protein_coding
ENSG00000168658.19	82.27299705	-0.126475366	0.137015732	-0.92307185	0.355969763	0.58391415	VWA3B	protein_coding
ENSG00000168661.15	230.0958094	-0.08059648	0.067764074	-1.189368875	0.234294543	0.458873003	ZNF30	protein_coding
ENSG00000168671.10	18.64962097	0.495465182	0.223309311	2.218739465	0.026504455	0.117424219	UGT3A2	protein_coding
ENSG00000168672.4	4402.348017	0.003750053	0.08590345	0.043654281	0.965179983	0.984173721	LRATD2	protein_coding
ENSG00000168675.19	1703.83187	-0.036240634	0.108752085	-0.333240823	0.738952505	0.864958949	LDLRAD4	protein_coding
ENSG00000168676.11	31.14763902	-0.062582488	0.156606389	-0.39961644	0.689439046	0.835893531	KCTD19	protein_coding
ENSG00000168679.18	505.5370997	0.56373227	0.11565133	4.874412325	1.09133E-06	5.57524E-05	SLC16A4	protein_coding
ENSG00000168685.15	2008.169767	-0.243870535	0.128855937	-1.892582834	0.058413368	0.196465207	IL7R	protein_coding
ENSG00000168701.19	1874.075404	-0.055832603	0.065205881	-0.856251032	0.391858941	0.615863132	TMEM208	protein_coding
ENSG00000168702.18	33.61835192	-0.630049603	0.20571877	-3.062674372	0.002193686	0.020447971	LRP1B	protein_coding
ENSG00000168703.5	7.612447876	0.279045448	0.226971947	1.229427035	0.218911739	0.440542941	WFDC12	protein_coding
ENSG00000168710.18	9494.876377	-0.029828153	0.039270827	-0.759549892	0.447523681	0.662783423	AHCYL1	protein_coding
ENSG00000168724.17	3548.614441	0.051369756	0.053811805	0.954618723	0.339770546	0.569643777	DNAJC21	protein_coding
ENSG00000168734.14	2155.913551	-0.100891399	0.098269769	-1.026677896	0.304572161	0.53402777	PKIG	protein_coding
ENSG00000168743.13	3304.648858	0.462007688	0.142634744	3.239096404	0.00119909	0.013195142	NPNT	protein_coding
ENSG00000168746.8	3.917934079	0.059366685	0.216748009	0.273897256	0.78416358	0.890519109	LINC01620	lncRNA
ENSG00000168748.14	13.80882305	-0.025105813	0.182456729	-0.137598722	0.890557566	0.947901515	CA7	protein_coding
ENSG00000168754.15	109.0632408	1.232125311	0.226094309	5.449607799	5.0481E-08	4.65076E-06	FAM178B	protein_coding
ENSG00000168757.12	0.230158599	0.183407442	0.939686845	0.195179322	0.845252566		TSPY2	protein_coding
ENSG00000168758.11	2444.482883	0.265348384	0.073878429	3.591689604	0.000328541	0.0049815	SEMA4C	protein_coding
ENSG00000168763.16	3912.611719	-0.036466695	0.050488616	-0.722275593	0.470125055	0.681456925	CNNM3	protein_coding
ENSG00000168765.17	3321.939495	-0.447321877	0.097484016	-4.588668959	4.46081E-06	0.000170069	GSTM4	protein_coding
ENSG00000168769.14	2147.709339	0.10124934	0.063467856	1.595285345	0.110648441	0.29235175	TET2	protein_coding
ENSG00000168772.11	225.5591197	0.026100284	0.154943152	0.168450708	0.866228718	0.935111573	CXXC4	protein_coding
ENSG00000168778.12	781.4192781	0.06851458	0.066058069	1.037187133	0.299648654	0.529463471	TCTN2	protein_coding
ENSG00000168779.20	91.26360009	0.823350138	0.169054066	4.870336189	1.11409E-06	5.66937E-05	SHOX2	protein_coding

ENSG00000168781.22	1058.748603	-0.049795348	0.053999464	-0.922145215	0.356452835	0.584392219	PPIP5K1	protein_coding
ENSG00000168785.8	796.5035302	0.375642316	0.110723991	3.392600943	0.000692324	0.008676851	TSPAN5	protein_coding
ENSG00000168792.5	1145.257298	0.00700896	0.055586682	0.126090634	0.899660181	0.95213662	ABHD15	protein_coding
ENSG00000168795.5	1400.49078	0.23766861	0.062955916	3.775159274	0.000159905	0.00286286	ZBTB5	protein_coding
ENSG00000168802.14	36.72910185	-0.054771017	0.07307644	-0.749503082	0.453554042	0.667970393	CHTF8	protein_coding
ENSG00000168803.16	368.5949743	-0.117395502	0.069229877	-1.695734661	0.089936141	0.258076335	ADAL	protein_coding
ENSG00000168806.8	685.5794229	0.030961387	0.060854646	0.508776053	0.610909206	0.78619479	LCMT2	protein_coding
ENSG00000168807.16	3300.841112	0.011997669	0.054988768	0.21818399	0.827285758	0.914944203	SNTB2	protein_coding
ENSG00000168811.7	38.5878155	-0.272124176	0.143366111	-1.898106705	0.057682027	0.194929019	IL12A	protein_coding
ENSG00000168813.17	1871.599817	-0.006839193	0.055951709	-0.122233852	0.902713817	0.953695609	ZNF507	protein_coding
ENSG00000168818.10	1676.736639	-0.044471751	0.053251651	-0.835124359	0.403647693	0.625280136	STX18	protein_coding
ENSG00000168824.14	317.4463515	0.185429421	0.170990695	1.084441589	0.278169052	0.50705985	NSG1	protein_coding
ENSG00000168826.16	256.3968217	-0.032677894	0.044927256	-0.727351205	0.467010838	0.678545322	ZBTB49	protein_coding
ENSG00000168827.15	3928.790613	-0.011731198	0.053197147	-0.220523069	0.825463808	0.913880144	GFM1	protein_coding
ENSG00000168828.5	1.782045387	0.583494547	0.256290842	2.276689027	0.022804802	0.106330502	OR13J1	protein_coding
ENSG00000168830.8	3.390403757	-0.117148056	0.356508899	-0.328597844	0.742459678	0.867217899	HTR1E	protein_coding
ENSG00000168843.14	17.28195084	-0.014801468	0.29432171	-0.050290099	0.959891214	0.981398795	FSTL5	protein_coding
ENSG00000168852.13	56.53403566	-0.141983995	0.107962857	-1.31511891	0.188469942	0.403797673	TPTE2P5	transcribed_unprocessed_pseudogene
ENSG00000168872.17	769.6542804	-0.017421288	0.048105761	-0.362145561	0.717243258	0.852280973	DDX19A	protein_coding
ENSG00000168874.13	298.8915509	-0.223145538	0.150247938	-1.485182036	0.137495609	0.333908056	ATOH8	protein_coding
ENSG00000168875.3	111.2957313	0.141304791	0.311736817	0.453282332	0.650345453	0.811608567	SOX14	protein_coding
ENSG00000168876.9	1055.644922	-0.229114235	0.067128153	-3.413087129	0.000642314	0.008203434	ANKRD49	protein_coding
ENSG00000168878.19	50.72709139	-0.202413776	0.223624283	-0.905151144	0.36538532	0.592594618	SFTPB	protein_coding
ENSG00000168883.20	3336.824197	-0.025550985	0.042964632	-0.594698103	0.55204529	0.743984136	USP39	protein_coding
ENSG00000168884.15	2031.716502	0.054005155	0.051912004	1.040321143	0.298190725	0.528050114	TNIP2	protein_coding
ENSG00000168887.11	1831.265175	-0.000178517	0.048124444	-0.003709496	0.997040258	0.998514351	C2orf68	protein_coding
ENSG00000168890.14	942.6963626	-0.039781316	0.061145381	-0.650602138	0.51530335	0.716920186	TMEM150A	protein_coding
ENSG00000168894.10	3182.462358	-0.079655814	0.06756855	-1.178888905	0.238442416	0.463845595	RNF181	protein_coding
ENSG00000168899.5	1006.738725	-0.010685585	0.093646044	-0.114106104	0.909153684	0.957009712	VAMP5	protein_coding
ENSG00000168903.9	387.4982171	-0.819288902	0.233229509	-3.512801204	0.000443409	0.006217001	BTNL3	protein_coding
ENSG00000168904.15	1200.239285	-0.177189185	0.056137724	-3.156330051	0.00159768	0.016266842	LRRC28	protein_coding
ENSG00000168906.13	7831.644926	-0.018281477	0.052745967	-0.346594778	0.72889577	0.858786864	MAT2A	protein_coding
ENSG00000168907.13	884.6383705	-0.309417055	0.172505649	-1.793663318	0.072866924	0.225664269	PLA2G4F	protein_coding
ENSG00000168913.7	19.13511241	-0.279923401	0.169626225	-1.650236578	0.098894558	0.273725112	ENHO	protein_coding
ENSG00000168916.15	1006.399772	-0.272938896	0.109104471	-2.50162887	0.012362344	0.069990109	ZNF608	protein_coding
ENSG00000168917.9	261.5717606	0.255951922	0.105574197	2.424379521	0.015334571	0.081254205	SLC35G2	protein_coding
ENSG00000168918.14	3371.265904	-0.190519452	0.105979582	-1.797699596	0.072224625	0.224173901	INPP5D	protein_coding
ENSG00000168924.15	4343.24298	0.057982703	0.068571029	0.845586018	0.397783728	0.620882181	LETM1	protein_coding
ENSG00000168925.12	15.67029398	-1.919498035	0.240934299	-7.966894046	1.62712E-15	1.96958E-12	CTRB1	protein_coding
ENSG00000168928.13	15.00901245	-2.53075294	0.289221771	-8.750215901	2.12945E-18	4.88395E-15	CTRB2	protein_coding
ENSG00000168930.13	0.745205469	-0.375642382	0.464213954	-0.809200969	0.418399558		TRIM49	protein_coding
ENSG00000168936.11	2649.0694	0.01142683	0.066701448	0.171313074	0.863977601	0.933873814	TMEM129	protein_coding

ENSG00000168938.6	1673.641998	0.45626009	0.083757476	5.447395433	5.11127E-08	4.68914E-06	PPIC	protein_coding
ENSG00000168939.12	180.4179054	0.01197652	0.082043687	0.14597735	0.883939269	0.944334923	SPRY3	protein_coding
ENSG00000168944.17	1579.480529	-0.144140267	0.051717831	-2.787051671	0.005319	0.038859912	CEP120	protein_coding
ENSG00000168952.15	908.9362613	-0.466498825	0.130641683	-3.570826811	0.000355856	0.005285325	STXBP6	protein_coding
ENSG00000168955.4	2209.554138	-0.737639021	0.258715718	-2.851156579	0.004356051	0.033710468	TM4SF20	protein_coding
ENSG00000168958.20	3139.669569	-0.10364118	0.041526627	-2.49577654	0.012568175	0.07079671	MFF	protein_coding
ENSG00000168959.14	3.283501462	-0.580401068	0.284886247	-2.037308131	0.041619174	0.158002493	GRM5	protein_coding
ENSG00000168961.17	7399.622346	-0.171223517	0.096020121	-1.783204548	0.074553012	0.229256695	LGALS9	protein_coding
ENSG00000168967.14	2.322015466	-0.74806544	0.344612087	-2.170746375	0.02995035	0.1274806	PMCHL1	transcribed_unprocessed_pseudogene
ENSG00000168970.22	129.0910956	-0.012442192	0.09871375	-0.126043151	0.899697767	0.95213662	JMJD7-PLA2G4B	protein_coding
ENSG00000168992.4	7.063656531	-0.175956994	0.176403878	-0.997466695	0.318538032	0.548046296	OR7E102P	unprocessed_pseudogene
ENSG00000168993.15	722.6678157	-0.328039432	0.139043923	-2.3592504	0.018311895	0.091535467	CPLX1	protein_coding
ENSG00000168994.14	1404.971044	-0.024915038	0.086627176	-0.287612265	0.773643555	0.885569225	PXDC1	protein_coding
ENSG00000168995.13	120.3748107	0.040935977	0.130379766	0.313974922	0.753540086	0.873394588	SIGLEC7	protein_coding
ENSG00000169006.7	3.078040431	0.695550784	0.283058971	2.45726458	0.013999954	0.076377467	NTSR2	protein_coding
ENSG00000169016.17	673.5075026	-0.062356582	0.045781775	-1.362039419	0.173185445	0.384144464	E2F6	protein_coding
ENSG00000169018.6	4442.394928	0.105362284	0.056001617	1.881415021	0.059915485	0.199750371	FEM1B	protein_coding
ENSG00000169019.11	1083.338606	-0.148014246	0.058736688	-2.519962406	0.011736737	0.067509475	COMMD8	protein_coding
ENSG00000169020.10	979.8381706	-0.268024007	0.096890822	-2.766247642	0.005670545	0.040695876	ATP5ME	protein_coding
ENSG00000169021.6	6737.97608	0.246019488	0.098926813	2.486883807	0.012886748	0.072123438	UQCRFS1	protein_coding
ENSG00000169026.13	363.3611989	0.127303307	0.104810322	1.214606575	0.22451618	0.447425063	SLC49A3	protein_coding
ENSG00000169031.20	176.2916352	0.130134015	0.175237114	0.742616742	0.45771375	0.671213894	COL4A3	protein_coding
ENSG00000169032.10	2663.07947	0.071023186	0.046085225	1.541127021	0.123285875	0.312386822	MAP2K1	protein_coding
ENSG00000169035.12	1036.482813	0.372876167	0.273789054	1.361910423	0.173226156	0.384200748	KLK7	protein_coding
ENSG00000169040.14	2.520329389	-0.138817667	0.273913033	-0.506794676	0.612298892	0.787117541	PMCHL2	transcribed_unprocessed_pseudogene
ENSG00000169045.17	11598.74534	-0.041707935	0.049295424	-0.84608128	0.397507403	0.620751188	HNRNPH1	protein_coding
ENSG00000169047.5	3988.342058	0.372393201	0.094175556	3.954244783	7.67768E-05	0.001616281	IRS1	protein_coding
ENSG00000169057.24	4397.365745	0.050220707	0.054679758	0.918451522	0.358382528	0.586056568	MECP2	protein_coding
ENSG00000169059.12	6.300207873	-0.724118664	0.469895074	-1.541022039	0.123311422	0.312415224	VCX3A	protein_coding
ENSG00000169062.15	2219.061978	-0.088819157	0.066681078	-1.33199942	0.182860379	0.396699693	UPF3A	protein_coding
ENSG00000169064.13	6.916870039	0.080777627	0.30210673	0.267381092	0.789175756	0.894080489	ZBBX	protein_coding
ENSG00000169067.4	35.24501513	0.738960353	0.21221791	3.482082886	0.00049753	0.006771034	ACTBL2	protein_coding
ENSG00000169071.15	1139.909898	0.194574801	0.15201027	1.280010893	0.200541305	0.419027493	ROR2	protein_coding
ENSG00000169075.7	7.035685237	-0.178072512	0.145793285	-1.221404067	0.221933068	0.444447487	BRD7P3	processed_pseudogene
ENSG00000169083.18	282.3557768	0.274836445	0.161887707	1.697698053	0.089564765	0.257452734	AR	protein_coding
ENSG00000169084.15	1429.379424	-0.143517844	0.078658424	-1.824570545	0.068065862	0.216093986	DHRSX	protein_coding
ENSG00000169084.15_PAR_Y	0						DHRSX	protein_coding
ENSG00000169085.13	5.67062406	-0.16591652	0.269104377	-0.616550802	0.537531054	0.733606121	VXN	protein_coding
ENSG00000169087.11	564.2101469	-0.00949628	0.051529247	-0.184289132	0.853786639	0.928650668	HSPBAP1	protein_coding
ENSG00000169093.16	1004.948239	-0.090326603	0.075168695	-1.201651873	0.229498435	0.45306031	ASMTL	protein_coding
ENSG00000169093.16_PAR_Y	0						ASMTL	protein_coding
ENSG00000169100.14	31965.5903	-0.207893829	0.072879374	-2.852574284	0.004336668	0.033602233	SLC25A6	protein_coding

ENSG00000169100.14_PAR_Y	0						SLC25A6	protein_coding
ENSG00000169105.8	1185.329871	0.205162849	0.068380417	3.000315861	0.002696998	0.023709314	CHST14	protein_coding
ENSG00000169116.11	2766.040973	0.47296571	0.134052551	3.528211202	0.000418378	0.005946401	PARM1	protein_coding
ENSG00000169118.18	1612.234547	0.078880179	0.047995459	1.64349256	0.100281063	0.276001082	CSNK1G1	protein_coding
ENSG00000169122.11	316.5959838	-0.285036426	0.138691216	-2.055187308	0.039860917	0.153669602	FAM110B	protein_coding
ENSG00000169126.16	36.22619664	-0.30650648	0.18759718	-1.63385441	0.102289424	0.279184909	ARMC4	protein_coding
ENSG00000169129.15	1506.452892	0.574665805	0.113286307	5.072685459	3.92241E-07	2.45232E-05	AFAP1L2	protein_coding
ENSG00000169131.13	521.0609357	-0.054721646	0.061747461	-0.886216938	0.375500638	0.601300529	ZNF354A	protein_coding
ENSG00000169136.12	1985.748623	-0.420920978	0.096668349	-4.354279154	1.33505E-05	0.000406553	ATF5	protein_coding
ENSG00000169139.12	3051.852681	-0.099471491	0.059544101	-1.670551558	0.094810288	0.266264727	UBE2V2	protein_coding
ENSG00000169154.7	2.584663984	-0.187270054	0.336633173	-0.556303029	0.578003717	0.762358646	GOT1L1	protein_coding
ENSG00000169155.10	1151.468474	0.001786162	0.059229983	0.030156382	0.975942334	0.989038119	ZBTB43	protein_coding
ENSG00000169164.7	0.26541698	0.250682743	0.687543992	0.364606114	0.71540545		AL159987.1	transcribed_unprocessed_pseudogene
ENSG00000169169.15	324.8802274	0.458346713	0.12930389	3.544724854	0.000393023	0.005660633	CPT1C	protein_coding
ENSG00000169174.11	1974.534591	0.705094529	0.171380812	4.114197615	3.88528E-05	0.000941129	PCSK9	protein_coding
ENSG00000169180.12	7343.60747	0.109937378	0.046447732	2.366905215	0.017937526	0.090188482	XPO6	protein_coding
ENSG00000169181.13	8.221615529	-0.253455705	0.176724351	-1.434186654	0.151519004	0.35401553	GSG1L	protein_coding
ENSG00000169184.6	1596.614477	0.321501604	0.154443171	2.081682224	0.037371505	0.147192524	MN1	protein_coding
ENSG00000169188.5	2621.770847	-0.074026866	0.054038268	-1.369897085	0.17071903	0.380825347	APEX2	protein_coding
ENSG00000169189.17	2550.224656	-0.033666172	0.057375264	-0.58677155	0.557357146	0.747781785	NSMCE1	protein_coding
ENSG00000169193.12	622.8027533	-0.186607786	0.05563297	-3.354266101	0.000795758	0.009641597	CCDC126	protein_coding
ENSG00000169194.9	5.278032555	-0.168428846	0.182808143	-0.921342142	0.356871826	0.584696094	IL13	protein_coding
ENSG00000169203.16	103.8270419	-0.126121585	0.109801921	-1.148628221	0.250709315	0.477393918	NPIPB12	protein_coding
ENSG00000169208.3	5.439089258	-0.070690418	0.3034182	-0.23298015	0.815776819	0.909044813	OR10G3	protein_coding
ENSG00000169213.7	1089.191151	0.507131411	0.172595571	2.938264337	0.003300555	0.027600897	RAB3B	protein_coding
ENSG00000169214.4	0.222993812	0.059651189	0.648893149	0.091927599	0.926755564		OR6F1	protein_coding
ENSG00000169217.9	2580.756341	0.003762415	0.047725511	0.078834453	0.9371643	0.970204278	CD2BP2	protein_coding
ENSG00000169218.14	72.99016546	-0.188879347	0.201589633	-0.936949704	0.34878443	0.577951902	RSPO1	protein_coding
ENSG00000169220.18	1159.101318	-0.130310416	0.080439462	-1.619981191	0.105236317	0.283960962	RGS14	protein_coding
ENSG00000169221.14	4200.865557	0.100539049	0.051590286	1.948798052	0.051319547	0.180934618	TBC1D10B	protein_coding
ENSG00000169223.15	8219.436516	-0.022485881	0.060759227	-0.370081753	0.711321577	0.849078613	LMAN2	protein_coding
ENSG00000169224.13	32.39693527	-0.317548872	0.154987665	-2.048865458	0.040475273	0.155208638	GCSAML	protein_coding
ENSG00000169228.14	132.9990798	-0.040447776	0.066661153	-0.606766821	0.54400567	0.738485875	RAB24	protein_coding
ENSG00000169230.10	3398.340358	-0.097110621	0.0725251	-1.338993264	0.180572866	0.39356846	PRELID1	protein_coding
ENSG00000169231.13	754.957008	0.256590228	0.070476693	3.640781334	0.000271812	0.004318173	THBS3	protein_coding
ENSG00000169239.13	580.6494012	-0.084103711	0.090349187	-0.930874015	0.351918737	0.580628609	CA5B	protein_coding
ENSG00000169241.19	2930.434516	0.153548779	0.078487333	1.956351083	0.05042381	0.178901797	SLC50A1	protein_coding
ENSG00000169242.12	4770.043122	0.470753909	0.089247262	5.27471542	1.32962E-07	1.01473E-05	EFNA1	protein_coding
ENSG00000169245.6	2921.84472	-0.842271525	0.187835807	-4.484083934	7.32278E-06	0.000253862	CXCL10	protein_coding
ENSG00000169246.16	232.0215064	-0.072434102	0.096792901	-0.748341053	0.454254468	0.668525733	NPIPB3	protein_coding
ENSG00000169247.14	361.4069548	0.03378644	0.151764065	0.22262477	0.823827557	0.913066113	SH3TC2	protein_coding
ENSG00000169248.13	1111.291224	-0.737154969	0.190250842	-3.874647612	0.000106779	0.002084728	CXCL11	protein_coding

ENSG00000169249.13	789.9141784	-0.230094884	0.064030124	-3.5935411	0.000326214	0.004947941	ZRSR2	protein_coding
ENSG00000169251.13	3497.854211	0.114191908	0.054881138	2.080713208	0.037460164	0.147409328	NMD3	protein_coding
ENSG00000169252.6	137.9279787	-0.216173979	0.151171781	-1.429988968	0.152720185	0.355944136	ADRB2	protein_coding
ENSG00000169253.3	46.03725458	-0.20844116	0.104461891	-1.995379922	0.046001459	0.168538293	AL669983.1	processed_pseudogene
ENSG00000169255.15	706.9909075	0.407431027	0.100463814	4.055500308	5.00271E-05	0.001151628	B3GALNT1	protein_coding
ENSG00000169258.7	719.379744	0.540691555	0.119858691	4.511075099	6.44999E-06	0.000230756	GPRIN1	protein_coding
ENSG00000169271.3	8.052562359	-0.186548681	0.310302115	-0.601184046	0.547717409	0.741100464	HSPB3	protein_coding
ENSG00000169282.18	206.2324706	0.085753126	0.08937057	0.959523098	0.337295289	0.567038286	KCNAB1	protein_coding
ENSG00000169288.18	1118.516695	-0.073157457	0.062788364	-1.165143532	0.243960898	0.470027592	MRPL1	protein_coding
ENSG00000169291.10	483.7865146	-0.081836423	0.105931361	-0.772541978	0.439793494	0.656354022	SHE	protein_coding
ENSG00000169297.8	1.571496728	-0.011560608	0.284548704	-0.040627871	0.967592564	0.984830373	NR0B1	protein_coding
ENSG00000169299.14	2317.149338	0.064422181	0.059712495	1.078872704	0.280644478	0.509526058	PGM2	protein_coding
ENSG00000169302.16	72.0368932	0.198592742	0.189738152	1.046667419	0.295252995	0.525146329	STK32A	protein_coding
ENSG00000169306.10	21.94561242	-0.148609278	0.179510045	-0.827860515	0.407749499	0.628802831	IL1RAPL1	protein_coding
ENSG00000169313.10	44.263576	-0.649252908	0.168170437	-3.860683955	0.00011307	0.002173469	P2RY12	protein_coding
ENSG00000169314.15	14.94472284	0.029617806	0.108133883	0.273899398	0.784161934	0.890519109	C22orf15	protein_coding
ENSG00000169325.10	1.079906945	0.317839621	0.512241884	0.620487373	0.534936965	0.731874921	GUSBP12	unprocessed_pseudogene
ENSG00000169327.5	3.004376447	0.536183619	0.274398978	1.954029212	0.050697763	0.179497595	OR5AU1	protein_coding
ENSG00000169330.9	174.2747826	0.282213561	0.097188767	2.903767281	0.003687021	0.029914228	MINAR1	protein_coding
ENSG00000169340.10	13.64729687	-0.274544252	0.321276372	-0.854542308	0.392804578	0.616850905	PDILT	protein_coding
ENSG00000169344.15	6.34546135	-0.896609944	0.273148157	-3.282504099	0.001028895	0.011734141	UMOD	protein_coding
ENSG00000169347.17	3284.901645	-0.58825626	0.271251017	-2.16867854	0.030107098	0.127930052	GP2	protein_coding
ENSG00000169359.16	1328.726585	-0.023675151	0.061845516	-0.382811118	0.701859817	0.843257382	SLC33A1	protein_coding
ENSG00000169371.14	559.0086871	0.101471689	0.053967798	1.880226588	0.060077203	0.19998622	SNUPN	protein_coding
ENSG00000169372.13	420.5284499	-0.204094549	0.050937239	-4.006784645	6.15509E-05	0.001356019	CRADD	protein_coding
ENSG00000169375.16	4949.597058	0.107179724	0.040961728	2.616582112	0.0088815	0.055567713	SIN3A	protein_coding
ENSG00000169379.16	836.1978062	0.288802269	0.055949012	5.16188327	2.44478E-07	1.66828E-05	ARL13B	protein_coding
ENSG00000169385.3	78.21749079	-0.070765919	0.148072839	-0.477912893	0.632712205	0.800592108	RNASE2	protein_coding
ENSG00000169393.9	3.662253607	-0.306475414	0.357546203	-0.857163106	0.391354749	0.615600549	ELSPBP1	protein_coding
ENSG00000169397.3	4.313219429	0.222934787	0.17831077	1.250259797	0.211204659	0.43162576	RNASE3	protein_coding
ENSG00000169398.19	7748.787189	0.044904652	0.048220473	0.931236249	0.351731371	0.580537877	PTK2	protein_coding
ENSG00000169402.15	4.938875637	-0.389620744	0.194207215	-2.006211481	0.044833689	0.165794101	RSPH10B2	protein_coding
ENSG00000169403.12	1967.698804	-0.169047382	0.107535695	-1.572011808	0.115947813	0.300924775	PTAFR	protein_coding
ENSG00000169410.10	3147.906611	0.086168963	0.043821472	1.966363959	0.049256583	0.176285655	PTPN9	protein_coding
ENSG00000169413.3	977.5723415	-0.172306121	0.120271245	-1.43264602	0.151959023	0.354739291	RNASE6	protein_coding
ENSG00000169418.10	513.8877248	0.107887164	0.118758361	0.908459524	0.36363549	0.590987347	NPR1	protein_coding
ENSG00000169427.8	129.466052	-0.005565372	0.174707304	-0.031855407	0.974587361	0.988147547	KCNK9	protein_coding
ENSG00000169429.11	7015.637188	0.161965209	0.194444667	0.83296298	0.404865608	0.626479739	CXCL8	protein_coding
ENSG00000169432.18	189.821383	0.498016691	0.170474673	2.921352971	0.003485147	0.02874069	SCN9A	protein_coding
ENSG00000169435.14	2915.561907	-0.14056194	0.127215511	-1.104911961	0.269197739	0.497250702	RASSF6	protein_coding
ENSG00000169436.17	487.4456478	-0.652372229	0.180960629	-3.605050621	0.000312092	0.004788745	COL22A1	protein_coding
ENSG00000169439.12	3193.845128	0.171720993	0.098721746	1.739444453	0.081956615	0.243828273	SDC2	protein_coding

ENSG00000169442.9	695.0162229	-0.67261549	0.134541291	-4.999323892	5.75317E-07	3.32061E-05	CD52	protein_coding
ENSG00000169446.5	2984.892707	0.048560708	0.05692612	0.853047924	0.393632728	0.617539563	MMGT1	protein_coding
ENSG00000169469.9	865.3158516	0.969476326	0.358726501	2.702550055	0.006880982	0.046441961	SPRR1B	protein_coding
ENSG00000169474.4	218.6961778	0.515985381	0.36205573	1.425154579	0.154112523	0.3580021	SPRR1A	protein_coding
ENSG00000169484.4	0.977198221	-1.010914861	0.475736079	-2.124948909	0.033590876	0.137071866	OR4K14	protein_coding
ENSG00000169488.6	0.403162054	0.21280199	0.627626534	0.339058307	0.734565806		OR4K15	protein_coding
ENSG00000169490.17	2112.161047	0.013943292	0.057534256	0.242347649	0.808510795	0.905167967	TM2D2	protein_coding
ENSG00000169495.5	53.89068203	-0.178748923	0.145645905	-1.227284236	0.219715778	0.441610372	HTRA4	protein_coding
ENSG00000169499.15	2908.025295	-0.085022637	0.074127364	-1.146980444	0.251389703	0.478028815	PLEKHA2	protein_coding
ENSG00000169504.15	11779.09441	0.025254916	0.099330627	0.254251044	0.799301618	0.899913877	CLIC4	protein_coding
ENSG00000169507.9	370.4837276	-0.310766892	0.189148621	-1.642977308	0.100387628	0.276156283	SLC38A11	protein_coding
ENSG00000169508.7	980.8689189	-0.237338052	0.116795264	-2.032086262	0.042144924	0.159422686	GPR183	protein_coding
ENSG00000169509.6	40.21741644	0.585713527	0.407788609	1.436316547	0.150912288	0.353258023	CRCT1	protein_coding
ENSG00000169515.8	411.1434237	0.500162481	0.15949675	3.135878823	0.0017134	0.017128888	CCDC8	protein_coding
ENSG00000169519.21	833.3808012	-0.05937668	0.047424691	-1.252020374	0.21056244	0.430825317	METTL15	protein_coding
ENSG00000169548.3	18.46657237	0.647503706	0.287622831	2.251224994	0.024371291	0.111055917	ZNF280A	protein_coding
ENSG00000169550.14	94.71935286	1.863835518	0.383669381	4.857920929	1.18625E-06	5.96918E-05	MUC15	protein_coding
ENSG00000169551.13	6.66223768	0.781415701	0.317092638	2.464313605	0.013727594	0.07535047	CT55	protein_coding
ENSG00000169554.22	2885.54767	-0.030380093	0.103198827	-0.294384095	0.768464419	0.882593801	ZEB2	protein_coding
ENSG00000169562.13	2936.533148	-0.218410132	0.144963869	-1.506652196	0.131899831	0.325469928	GJB1	protein_coding
ENSG00000169564.7	992.1824385	-0.042186153	0.098916021	-0.426484539	0.669754812	0.823906178	PCBP1	protein_coding
ENSG00000169567.13	9500.612706	-0.107869118	0.063678964	-1.693952162	0.090274374	0.258689182	HINT1	protein_coding
ENSG00000169570.10	870.9277226	-0.103714707	0.061904645	-1.675394582	0.093856839	0.264759158	DTWD2	protein_coding
ENSG00000169575.5	1.307781519	-0.864851085	0.337253432	-2.564395209	0.010335578	0.061816273	VPREB1	protein_coding
ENSG00000169583.13	963.0463702	0.775546028	0.161521724	4.801496709	1.57484E-06	7.42715E-05	CLIC3	protein_coding
ENSG00000169592.15	2018.846516	-0.079020749	0.055215717	-1.431127813	0.152393588	0.355449091	INO80E	protein_coding
ENSG00000169594.13	24.35422795	0.501064128	0.218895416	2.289057199	0.022076029	0.103798215	BNC1	protein_coding
ENSG00000169598.17	577.2733388	-0.11261425	0.065232699	-1.726346619	0.084285073	0.248034213	DFFB	protein_coding
ENSG00000169599.13	1439.538005	-0.124301614	0.058030853	-2.141991856	0.032194137	0.133522787	NFU1	protein_coding
ENSG00000169604.20	10909.07699	0.746348087	0.115762342	6.447244175	1.13902E-10	3.0451E-08	ANTXR1	protein_coding
ENSG00000169605.7	6112.614652	-1.745184743	0.376499921	-4.635285819	3.56445E-06	0.000142633	GKN1	protein_coding
ENSG00000169607.13	954.0598884	-0.011415056	0.087520027	-0.13042793	0.896227871	0.950842916	CKAP2L	protein_coding
ENSG00000169609.14	993.0654656	-0.032108511	0.044214002	-0.726206856	0.46771197	0.67922692	C15orf40	protein_coding
ENSG00000169612.4	252.2976264	0.059714448	0.067078346	0.890219444	0.373348066	0.599461631	RAMAC	protein_coding
ENSG00000169618.7	3.452506032	0.002314728	0.215722437	0.010730124	0.991438764	0.996078919	PROKR1	protein_coding
ENSG00000169621.10	170.3968131	0.002982216	0.077207055	0.038626217	0.9691884	0.98576178	APLF	protein_coding
ENSG00000169627.9	48.88310157	-0.164204477	0.112951286	-1.453763684	0.146011835	0.346446573	BOLA2B	protein_coding
ENSG00000169629.12	81.24026003	-0.041128007	0.106439067	-0.386399549	0.699200784	0.841761111	RGPD8	protein_coding
ENSG00000169635.10	853.5853456	0.099491984	0.097724976	1.018081432	0.308639259	0.538051807	HIC2	protein_coding
ENSG00000169641.13	3264.259274	0.103506575	0.056716271	1.824989077	0.068002678	0.216023068	LUZP1	protein_coding
ENSG00000169660.17	1053.263303	-0.068785981	0.060229098	-1.142072233	0.253423996	0.480192081	HEXD	protein_coding
ENSG00000169662.8	0.401980491	-0.145589094	0.248228732	-0.586511855	0.557531597		BCRP6	unprocessed_pseudogene

ENSG00000169668.11	3.228753649	0.014941813	0.167158756	0.089386959	0.928774387	0.966224252	BCRP2	transcribed_unprocessed_pseudogene
ENSG00000169676.6	5.200265901	-0.317939897	0.22262476	-1.428142569	0.153250827	0.356780173	DRD5	protein_coding
ENSG00000169679.15	2628.766383	-0.028569854	0.090632254	-0.31522833	0.752588297	0.872962499	BUB1	protein_coding
ENSG00000169682.18	38.18728131	0.009282164	0.085111902	0.109058352	0.913156208	0.958950455	SPNS1	protein_coding
ENSG00000169683.8	1493.589775	-0.135709888	0.075591901	-1.795296658	0.072606447	0.225129582	LRRC45	protein_coding
ENSG00000169684.13	185.3408572	0.288287645	0.094820333	3.04035681	0.00236298	0.021551191	CHRNA5	protein_coding
ENSG00000169688.11	0.639420154	-0.845115371	0.396120645	-2.133479742	0.032885392		MT1B	protein_coding
ENSG00000169689.15	2901.636544	-0.222340659	0.087116079	-2.552234468	0.010703446	0.063304027	CENPX	protein_coding
ENSG00000169692.13	8872.836316	-0.109183574	0.087113986	-1.253341504	0.21008145	0.430429234	AGPAT2	protein_coding
ENSG00000169696.16	2307.083723	-0.141074022	0.098559001	-1.431366192	0.152325292	0.35536588	ASPSCR1	protein_coding
ENSG00000169704.5	3.139805124	0.437758436	0.217660558	2.011197806	0.044304567	0.164633763	GP9	protein_coding
ENSG00000169710.9	24696.99929	0.229786759	0.089385634	2.57073479	0.010148301	0.061081839	FASN	protein_coding
ENSG00000169714.17	16264.5103	-0.061422911	0.038906105	-1.578747374	0.114394014	0.298435161	CNBP	protein_coding
ENSG00000169715.15	3630.724515	-0.566366807	0.150298403	-3.76828228	0.000164375	0.002916223	MT1E	protein_coding
ENSG00000169717.7	0.838231536	0.220647053	0.681627783	0.323706074	0.746160572		ACTRT2	protein_coding
ENSG00000169718.18	6110.69994	-0.102202802	0.060399619	-1.692110034	0.090624996	0.259250669	DUS1L	protein_coding
ENSG00000169727.12	5520.699835	0.032975458	0.05540999	0.595117558	0.551764893	0.743782557	GPS1	protein_coding
ENSG00000169733.12	2083.865916	-0.001424173	0.058000418	-0.024554536	0.980410283	0.991327585	RFNG	protein_coding
ENSG00000169738.8	3816.286212	-0.352703899	0.09381967	-3.759381148	0.000170334	0.003000264	DCXR	protein_coding
ENSG00000169740.14	813.45747	-0.053229916	0.065478393	-0.812938639	0.41625324	0.636704252	ZNF32	protein_coding
ENSG00000169744.13	648.845426	0.103264362	0.092894541	1.111630031	0.266297255	0.494268484	LDB2	protein_coding
ENSG00000169750.9	497.3265413	-0.352903646	0.150079863	-2.351439024	0.018700954	0.092763972	RAC3	protein_coding
ENSG00000169752.17	114.5956294	0.05742038	0.186568916	0.307770348	0.758257086	0.87627477	NRG4	protein_coding
ENSG00000169756.16	5155.298344	0.080162435	0.050651454	1.582628493	0.113506165	0.297108251	LIMS1	protein_coding
ENSG00000169758.13	103.3702913	-0.243029206	0.088597217	-2.74307947	0.006086595	0.042745817	TMEM266	protein_coding
ENSG00000169760.17	115.2011228	-0.138224017	0.200209421	-0.690397164	0.48994446	0.69720931	NLGN1	protein_coding
ENSG00000169762.17	1339.204031	-0.05498311	0.050482368	-1.089154744	0.276085652	0.504867162	TAPT1	protein_coding
ENSG00000169763.15	0						PRYP3	protein_coding
ENSG00000169764.16	5012.656631	0.023435266	0.039859352	0.58794899	0.556566532	0.747173281	UGP2	protein_coding
ENSG00000169777.6	1.129490506	0.06118294	0.372003584	0.164468684	0.869362198	0.93666114	TAS2R1	protein_coding
ENSG00000169783.13	655.7250318	0.390160114	0.138173701	2.823693016	0.004747383	0.03590363	LINGO1	protein_coding
ENSG00000169789.10	0.052645064	-0.185062121	1.315095589	-0.140721422	0.888090021		PRY	protein_coding
ENSG00000169800.14	0.022673509	-0.076732314	2.893054209	-0.026522944	0.978840234		RBMY1F	protein_coding
ENSG00000169807.10	0.036715567	-0.138692337	1.88860594	-0.073436355	0.941458888		PRY2	protein_coding
ENSG00000169811.8	0.010330592	-0.168982477	2.893054209	-0.058409717	0.953422275		RBMY1HP	unprocessed_pseudogene
ENSG00000169813.16	17136.4138	-0.026218272	0.049037076	-0.534662214	0.592883455	0.77310454	HNRNPF	protein_coding
ENSG00000169814.16	1923.747278	-0.16367364	0.068431534	-2.391786821	0.016766578	0.086477562	BTD	protein_coding
ENSG00000169826.8	1919.872082	0.175639388	0.06609716	2.65729101	0.007877141	0.051210233	CSGALNACT2	protein_coding
ENSG00000169836.5	3.46996295	0.51193087	0.267772016	1.911816169	0.055899777	0.191069463	TACR3	protein_coding
ENSG00000169840.5	1.421676531	0.228427443	0.400838585	0.569873888	0.568763236	0.755755325	GSX1	protein_coding
ENSG00000169849.8	0.282870267	0.562756804	0.764279044	0.73632374	0.461533702		TSPY14P	unprocessed_pseudogene
ENSG00000169851.15	3078.578544	-0.028611769	0.152127396	-0.18807769	0.85081575	0.926965412	PCDH7	protein_coding

ENSG00000169855.20	2726.083346	0.194323383	0.102600126	1.893987765	0.058226634	0.196145797	ROBO1	protein_coding
ENSG00000169856.9	107.6367369	-0.409561798	0.281803396	-1.45336005	0.146123812	0.346594675	ONECUT1	protein_coding
ENSG00000169857.9	624.2350082	-0.021613764	0.054592685	-0.395909528	0.692171772	0.837654883	AVEN	protein_coding
ENSG00000169860.7	306.7690731	0.435823181	0.135608476	3.213834373	0.001309752	0.014068293	P2RY1	protein_coding
ENSG00000169862.19	202.7963911	-0.628791405	0.228673123	-2.749738997	0.005964275	0.042147307	CTNND2	protein_coding
ENSG00000169871.13	8197.517875	-0.124992039	0.066104303	-1.890830613	0.058646959	0.196967903	TRIM56	protein_coding
ENSG00000169876.14	20906.31629	-0.097437948	0.250825282	-0.388469404	0.697668691	0.840683622	MUC17	protein_coding
ENSG00000169877.10	3.199795596	-0.17996721	0.203667053	-0.883634376	0.376893616	0.602667441	AHSP	protein_coding
ENSG00000169884.14	48.2066546	0.08652674	0.149927589	0.577123531	0.563856009	0.752654332	WNT10B	protein_coding
ENSG00000169885.10	9.043046078	0.041894048	0.155278023	0.269800237	0.78731394	0.892667419	CALML6	protein_coding
ENSG00000169891.18	1942.872795	-0.033433207	0.102413254	-0.326453911	0.744080954	0.868366785	REPS2	protein_coding
ENSG00000169894.18	17306.47873	0.293216716	0.187448045	1.56425593	0.117757477	0.303765914	MUC3A	protein_coding
ENSG00000169895.6	5738.600974	-0.123771821	0.057824188	-2.140485242	0.032315572	0.133807386	SYAP1	protein_coding
ENSG00000169896.18	834.5496149	-0.038836649	0.12645695	-0.307113602	0.758756905	0.876568815	ITGAM	protein_coding
ENSG00000169900.8	6.12699216	0.515559269	0.262218356	1.966144845	0.049281881	0.176347228	PYDC1	protein_coding
ENSG00000169902.15	823.8392386	0.36401016	0.099604575	3.654552615	0.000257631	0.004136617	TPST1	protein_coding
ENSG00000169903.7	2594.910383	1.370031137	0.259204356	5.285525119	1.25345E-07	9.68465E-06	TM4SF4	protein_coding
ENSG00000169905.13	5812.871476	0.245148185	0.047713448	5.137926425	2.77787E-07	1.84529E-05	TOR1AIP2	protein_coding
ENSG00000169906.5	11.00754985	-0.550351247	0.191002101	-2.881388449	0.003959274	0.031563996	S100G	protein_coding
ENSG00000169908.12	20663.61009	0.426977875	0.130043125	3.283356005	0.00102579	0.011704861	TM4SF1	protein_coding
ENSG00000169914.6	900.5411431	-0.011797904	0.080789539	-0.146032565	0.883895681	0.944321602	OTUD3	protein_coding
ENSG00000169918.10	137.1256666	-0.021144354	0.109128087	-0.193757211	0.846365992	0.924734	OTUD7A	protein_coding
ENSG00000169919.17	5164.162644	0.002865488	0.067680647	0.042338358	0.966228967	0.984499865	GUSB	protein_coding
ENSG00000169925.17	3085.724048	0.179686986	0.051802879	3.468667932	0.000523046	0.007034801	BRD3	protein_coding
ENSG00000169926.11	8766.792982	0.113964327	0.060079188	1.8969019	0.057840887	0.195314399	KLF13	protein_coding
ENSG00000169933.16	6.231824943	-0.240087593	0.217765973	-1.10250279	0.270243146	0.498376198	FRMPD4	protein_coding
ENSG00000169946.14	381.1903973	0.243917445	0.136324554	1.789240729	0.073576055	0.227101838	ZFPM2	protein_coding
ENSG00000169951.10	798.8003613	-0.080667437	0.067638096	-1.192633169	0.23301307	0.45744968	ZNF764	protein_coding
ENSG00000169953.12	0.659487942	0.412562575	0.322581102	1.278942174	0.20091742		HSFY2	protein_coding
ENSG00000169955.7	514.9095504	-0.10094837	0.059482182	-1.697119487	0.089674073	0.257673321	ZNF747	protein_coding
ENSG00000169957.10	2494.583546	-0.033465744	0.073049499	-0.458124205	0.64686321	0.809475293	ZNF768	protein_coding
ENSG00000169962.5	174.0438504	0.55203835	0.155154028	3.558002056	0.000373686	0.00546998	TAS1R3	protein_coding
ENSG00000169964.8	397.8633221	0.019021571	0.053571505	0.355068813	0.72253804	0.855233249	TMEM42	protein_coding
ENSG00000169967.16	5045.312353	0.061053026	0.043653053	1.398596915	0.161933892	0.36872156	MAP3K2	protein_coding
ENSG00000169972.12	596.7688759	0.021544487	0.077859833	0.276708615	0.782003856	0.889755938	PUSL1	protein_coding
ENSG00000169976.7	4145.296501	-0.042792103	0.060232818	-0.710444976	0.477428241	0.687151648	SF3B5	protein_coding
ENSG00000169981.11	531.3367817	0.0614296	0.044625729	1.37655119	0.16865105	0.377856392	ZNF35	protein_coding
ENSG00000169989.3	42.35694187	-0.197559477	0.097453761	-2.027212446	0.042640691	0.160573227	TIGD4	protein_coding
ENSG00000169991.11	2333.429117	0.050533527	0.098156378	0.514826725	0.606674108	0.783064412	IFFO2	protein_coding
ENSG00000169992.10	1655.620936	0.311867858	0.102261711	3.049703105	0.002290677	0.021077033	NLGN2	protein_coding
ENSG00000169994.18	4926.630522	0.082093714	0.184546642	0.444839925	0.65643544	0.815389435	MYO7B	protein_coding
ENSG00000170004.17	8991.624723	-0.019997358	0.071238186	-0.280711214	0.778931907	0.888232814	CHD3	protein_coding

ENSG00000170006.12	1007.552706	0.34061498	0.126674716	2.688894759	0.007168902	0.047811329	TMEM154	protein_coding
ENSG00000170011.14	231.3192307	-0.075332331	0.161901253	-0.465298009	0.64171806	0.806162013	MYRIP	protein_coding
ENSG00000170017.12	3788.781556	0.664503648	0.105851424	6.277701541	3.43615E-10	7.4496E-08	ALCAM	protein_coding
ENSG00000170027.7	11706.96086	0.086227868	0.055204374	1.561975291	0.11829381	0.304608483	YWHAG	protein_coding
ENSG00000170035.16	3393.221439	0.066444285	0.057647176	1.152602612	0.249073531	0.475755117	UBE2E3	protein_coding
ENSG00000170037.14	1581.161146	0.030382243	0.048409338	0.627611198	0.530258671	0.728172002	CNTROB	protein_coding
ENSG00000170043.12	3278.572627	-0.114792415	0.061985025	-1.851937882	0.064034743	0.208055623	TRAPPC1	protein_coding
ENSG00000170044.8	34.2569497	0.335339208	0.200721248	1.6706712	0.09478664	0.26623312	ZPLD1	protein_coding
ENSG00000170049.9	87.60629261	0.037893862	0.098853958	0.383331768	0.701473788	0.843017199	KCNAB3	protein_coding
ENSG00000170054.16	30.96162893	-2.812988866	0.292246083	-9.625411694	6.24563E-22	4.5361E-18	SERPINA9	protein_coding
ENSG00000170074.19	28.20899452	0.441933607	0.194811555	2.26851845	0.023297626	0.107720765	FAM153A	protein_coding
ENSG00000170075.9	119.3140506	0.284901845	0.116951632	2.436065579	0.014847992	0.079525637	GPR37L1	protein_coding
ENSG00000170085.18	1339.547902	-0.028914764	0.070867377	-0.408012338	0.683264617	0.832692541	SIMC1	protein_coding
ENSG00000170088.14	1344.82726	0.019357132	0.061042637	0.317108386	0.751161362	0.87228478	TMEM192	protein_coding
ENSG00000170089.15	498.7732361	-0.127390956	0.095255586	-1.337359434	0.181105339	0.394283458	AC106795.1	transcribed_unprocessed_pseudogene
ENSG00000170091.11	28.20629529	-0.610457988	0.218560355	-2.793086552	0.005220772	0.03836326	NSG2	protein_coding
ENSG00000170092.14	108.788496	-0.025030184	0.098492529	-0.254132814	0.799392953	0.899971214	SPDYE5	protein_coding
ENSG00000170099.6	61.62381283	-2.033151734	0.285334568	-7.125500942	1.03703E-12	5.72033E-10	SERPINA6	protein_coding
ENSG00000170100.14	509.3764168	0.072662406	0.063894203	1.137230027	0.255442138	0.482440699	ZNF778	protein_coding
ENSG00000170113.16	2342.114441	-0.050642069	0.06194149	-0.817579131	0.413597548	0.63413343	NIPA1	protein_coding
ENSG00000170122.5	35.85135843	0.023306374	0.141204362	0.165054208	0.868901315	0.936472513	FOXD4	protein_coding
ENSG00000170128.5	98.87449058	-0.776685811	0.165485147	-4.693386845	2.68719E-06	0.000114243	GPR25	protein_coding
ENSG00000170142.12	1078.161842	0.127091016	0.065963268	1.92669375	0.054017807	0.187013108	UBE2E1	protein_coding
ENSG00000170144.21	14275.74199	-0.08397907	0.047297621	-1.775545326	0.075807888	0.231806916	HNRNPA3	protein_coding
ENSG00000170145.5	4161.382301	-0.019403588	0.059979802	-0.323502026	0.746315073	0.869390824	SIK2	protein_coding
ENSG00000170152.5	0						AL391987.1	protein_coding
ENSG00000170153.11	1165.057044	-0.235765091	0.177123693	-1.331075972	0.183164014	0.396969725	RNF150	protein_coding
ENSG00000170160.17	45.51844714	-0.008573329	0.194643347	-0.04404635	0.964867458	0.984062284	CCDC144A	protein_coding
ENSG00000170161.7	31.8999094	0.038910174	0.097607227	0.3986403	0.690158263	0.83648625	AL512625.1	lncRNA
ENSG00000170162.14	1.783629135	0.813787477	0.331064056	2.458096743	0.013967554	0.076285084	VGLL2	protein_coding
ENSG00000170166.6	38.02670737	0.390143701	0.144694006	2.696336304	0.007010685	0.047087639	HOXD4	protein_coding
ENSG00000170175.11	400.0319879	0.174675215	0.105663508	1.653127166	0.098304985	0.272652441	CHRNB1	protein_coding
ENSG00000170178.7	10.09980397	0.522599305	0.27927251	1.871288031	0.061305169	0.20260089	HOXD12	protein_coding
ENSG00000170180.22	1.140048958	-0.257871712	0.387771134	-0.665010079	0.506044047	0.710171295	GYPA	protein_coding
ENSG00000170185.10	3155.25069	0.03796469	0.05588186	0.679374125	0.49690084	0.70314817	USP38	protein_coding
ENSG00000170190.16	2012.373463	0.011999517	0.105003426	0.114277384	0.90901791	0.957009712	SLC16A5	protein_coding
ENSG00000170191.5	647.6955982	-0.034573985	0.067319486	-0.513580645	0.607545215	0.783731301	NANP	protein_coding
ENSG00000170209.5	20.95966929	0.172124496	0.14860931	1.158234946	0.246768171	0.473281124	ANKK1	protein_coding
ENSG00000170214.5	38.28263869	0.81347219	0.159307414	5.10630467	3.2852E-07	2.10631E-05	ADRA1B	protein_coding
ENSG00000170222.12	365.9054529	-0.140630852	0.054156152	-2.596765974	0.009410602	0.057856347	ADPRM	protein_coding
ENSG00000170231.16	193.3763244	-0.057675534	0.200909731	-0.287071878	0.774057284	0.885664986	FABP6	protein_coding
ENSG00000170234.13	1405.764094	-0.102021513	0.041491787	-2.458836305	0.013938816	0.0762234	PWWP2A	protein_coding

ENSG00000170236.16	44.45062457	-0.022656362	0.09248284	-0.244979085	0.806472626	0.903818047	USP50	protein_coding
ENSG00000170242.19	3140.287225	-0.052290662	0.053624029	-0.975134891	0.329493354	0.558972705	USP47	protein_coding
ENSG00000170248.15	11542.55423	0.097978771	0.049703696	1.971257235	0.04869446	0.174964719	PDCD6IP	protein_coding
ENSG00000170255.7	0.489628959	-0.486765961	0.67909504	-0.716786212	0.473506021		MRGPRX1	protein_coding
ENSG00000170260.9	698.7601073	-0.134481523	0.047766087	-2.815418467	0.004871376	0.036549576	ZNF212	protein_coding
ENSG00000170262.13	3.423211548	0.076091312	0.154350549	0.492977268	0.622028649	0.793407745	MRAP	protein_coding
ENSG00000170264.13	262.0750452	-0.027217091	0.077795679	-0.349853507	0.726448641	0.857340819	FAM161A	protein_coding
ENSG00000170265.12	2629.642249	-0.108695791	0.050015591	-2.173238158	0.029762397	0.126933775	ZNF282	protein_coding
ENSG00000170266.16	5544.517317	0.034158826	0.06533693	0.522810391	0.601106208	0.779317066	GLB1	protein_coding
ENSG00000170270.5	649.3834345	-0.064560863	0.055297602	-1.167516506	0.243001847	0.469060318	GON7	protein_coding
ENSG00000170271.11	1122.897133	-0.186201469	0.140047151	-1.329562706	0.183662393	0.397588479	FAXDC2	protein_coding
ENSG00000170275.15	10241.19567	-0.017518364	0.065575508	-0.267147973	0.789355233	0.894190524	CRTAP	protein_coding
ENSG00000170276.6	4.771138302	0.166249398	0.181205745	0.91746207	0.35890056	0.586595518	HSPB2	protein_coding
ENSG00000170279.3	2.600782739	-0.770700169	0.394715129	-1.952547832	0.050873198	0.179899484	C7orf33	protein_coding
ENSG00000170289.12	30.1722314	0.291334282	0.183779674	1.585236686	0.112912568	0.296266317	CNGB3	protein_coding
ENSG00000170290.4	23.98851455	-0.01971758	0.224414514	-0.087862322	0.929986103	0.96680375	SLN	protein_coding
ENSG00000170291.15	1634.621988	-0.08096253	0.059000017	-1.372245865	0.169986912	0.379832404	ELP5	protein_coding
ENSG00000170293.9	1168.306473	-0.200492986	0.090672082	-2.211187624	0.027022847	0.119019063	CMTM8	protein_coding
ENSG00000170296.10	27.61205142	-0.048843376	0.097411231	-0.501414219	0.616079635	0.789459705	GABARAP	protein_coding
ENSG00000170298.15	190.0584503	-0.22043291	0.209295847	-1.053212059	0.292243813	0.521985506	LGALS9B	protein_coding
ENSG00000170310.15	1134.102997	-0.046470758	0.057308425	-0.810888755	0.417429559	0.637675696	STX8	protein_coding
ENSG00000170312.16	3196.343738	-0.117814094	0.091288676	-1.290566353	0.196854089	0.414071085	CDK1	protein_coding
ENSG00000170315.14	12508.44312	-0.05871187	0.063605388	-0.92306441	0.35597364	0.58391415	UBB	protein_coding
ENSG00000170322.14	2543.056955	0.02962716	0.051692238	0.573145246	0.566546354	0.754587421	NFRKB	protein_coding
ENSG00000170323.9	213.9716183	0.621823342	0.202534809	3.070204792	0.00213912	0.020078806	FABP4	protein_coding
ENSG00000170324.21	8.359395161	0.277876244	0.232792798	1.193663408	0.23260966	0.456966511	FRMPD2	protein_coding
ENSG00000170325.15	1036.670667	0.073829552	0.04693277	1.573091745	0.115697576	0.300641221	PRDM10	protein_coding
ENSG00000170340.11	2596.620585	-0.049681254	0.055115088	-0.901409307	0.367370735	0.594441889	B3GNT2	protein_coding
ENSG00000170345.10	19421.75167	0.213327478	0.12974227	1.644240367	0.100126562	0.275697109	FOS	protein_coding
ENSG00000170348.9	19972.80382	0.062760279	0.046245966	1.357097376	0.174750272	0.386145151	TMED10	protein_coding
ENSG00000170356.9	80.63800545	0.094196555	0.13031986	0.722810437	0.469796354	0.681184392	OR2A20P	transcribed_unprocessed_pseudogene
ENSG00000170364.12	602.1771144	-0.170173809	0.052356951	-3.250261981	0.001152987	0.012807477	SETMAR	protein_coding
ENSG00000170365.10	1331.409117	0.072906262	0.046286018	1.575124952	0.115227606	0.299688056	SMAD1	protein_coding
ENSG00000170367.5	8.981085464	0.004664233	0.21769685	0.021425357	0.982906346	0.992450592	CST5	protein_coding
ENSG00000170369.4	273.641174	0.81879633	0.19601639	4.17718298	2.95142E-05	0.000754437	CST2	protein_coding
ENSG00000170370.12	17.68921918	0.481773899	0.184359162	2.613235458	0.008968949	0.055930149	EMX2	protein_coding
ENSG00000170373.8	4502.403638	0.656895853	0.214040511	3.069025808	0.00214758	0.020130159	CST1	protein_coding
ENSG00000170374.6	3.722247704	0.025296703	0.197032906	0.128388216	0.897841756	0.951512688	SP7	protein_coding
ENSG00000170379.20	235.4761685	-0.163992361	0.110070088	-1.489890337	0.136253073	0.332149259	TCAF2	protein_coding
ENSG00000170381.14	524.3784814	-0.237593419	0.215452094	-1.102766811	0.270128444	0.498250858	SEMA3E	protein_coding
ENSG00000170382.12	355.2101449	0.737789421	0.146162207	5.047744125	4.47057E-07	2.71329E-05	LRRN2	protein_coding
ENSG00000170385.10	3574.98004	-0.00078127	0.061370805	-0.012730327	0.989842943	0.995404463	SLC30A1	protein_coding

ENSG00000170390.16	323.8137607	-0.108295064	0.137158249	-0.789562899	0.429783083	0.648054677	DCLK2	protein_coding
ENSG00000170396.9	55.74384896	-0.356378659	0.152453317	-2.337624819	0.019406719	0.094975416	ZNF804A	protein_coding
ENSG00000170409.7	14.47830187	-0.011610755	0.13050656	-0.088966834	0.929108268	0.96638703	AC091390.1	unprocessed_pseudogene
ENSG00000170412.18	3845.151781	0.201472923	0.116649504	1.727164847	0.084138062	0.247802401	GPRC5C	protein_coding
ENSG00000170417.16	388.8000123	-0.005426592	0.068098311	-0.079687616	0.936485709	0.969859815	TMEM182	protein_coding
ENSG00000170419.11	16.35470164	-0.695093267	0.247596918	-2.807358321	0.004994965	0.037191092	VSTM2A	protein_coding
ENSG00000170421.12	124647.5814	-0.073626865	0.100559737	-0.732170418	0.464064558	0.676236012	KRT8	protein_coding
ENSG00000170423.13	36.27332492	1.109715767	0.302103807	3.673292896	0.000239445	0.003905279	KRT78	protein_coding
ENSG00000170425.3	362.8606232	0.494359375	0.126337602	3.913002668	9.11555E-05	0.001847574	ADORA2B	protein_coding
ENSG00000170426.2	5.812811436	0.712622838	0.330780288	2.154369122	0.031211236	0.13100482	SDR9C7	protein_coding
ENSG00000170430.10	1154.213119	-0.363502767	0.087359753	-4.160986648	3.16876E-05	0.000796797	MGMT	protein_coding
ENSG00000170439.7	1758.876532	-0.435714553	0.122738601	-3.549939043	0.00038532	0.005584075	METTL7B	protein_coding
ENSG00000170442.12	51.58057566	0.596273304	0.133888252	4.453514735	8.44759E-06	0.000282518	KRT86	protein_coding
ENSG00000170445.16	2467.816783	-0.012079121	0.041026982	-0.294418939	0.768437796	0.882593801	HARS1	protein_coding
ENSG00000170448.12	781.9039843	0.079646123	0.06449777	1.234866302	0.216880266	0.438182953	NFXL1	protein_coding
ENSG00000170454.6	4.285004311	0.783032335	0.326178006	2.400628859	0.016366927	0.085242211	KRT75	protein_coding
ENSG00000170456.16	1651.219409	-0.02491981	0.084921264	-0.293446053	0.769181229	0.883098426	DENND5B	protein_coding
ENSG00000170458.14	4228.228309	0.199106219	0.107786234	1.847232356	0.064713472	0.209375506	CD14	protein_coding
ENSG00000170464.10	434.7979804	-0.002372256	0.08750797	-0.027109029	0.978372774	0.990349602	DNAJC18	protein_coding
ENSG00000170465.10	325.6407281	0.298625893	0.437369915	0.682776485	0.494748091	0.701377324	KRT6C	protein_coding
ENSG00000170468.8	728.237591	0.013877041	0.058049015	0.239057305	0.811061147	0.906502132	RIOX1	protein_coding
ENSG00000170469.11	108.2157621	0.070980328	0.061285708	1.15818729	0.246787614	0.473281124	SPATA24	protein_coding
ENSG00000170471.15	6500.850993	0.028441542	0.056005451	0.507835239	0.611568894	0.78667939	RALGAPB	protein_coding
ENSG00000170473.17	1582.110546	-0.14967151	0.058843629	-2.543546568	0.010973343	0.06442532	PYM1	protein_coding
ENSG00000170476.16	1348.593226	-0.879014014	0.16890134	-5.204304567	1.94724E-07	1.39335E-05	MZB1	protein_coding
ENSG00000170477.13	1212.500405	1.460459174	0.402538472	3.628123212	0.000285489	0.004479926	KRT4	protein_coding
ENSG00000170482.17	118.1788005	-0.489991688	0.155139657	-3.158390939	0.001586427	0.016167378	SLC23A1	protein_coding
ENSG00000170484.10	2.288137057	0.100777968	0.339688323	0.296677751	0.766712545	0.88160411	KRT74	protein_coding
ENSG00000170485.17	3888.422426	0.10100834	0.084381082	1.197049591	0.231287226	0.455291911	NPAS2	protein_coding
ENSG00000170486.11	2.820112829	-0.29650029	0.261553303	-1.133613248	0.256956814	0.484009694	KRT72	protein_coding
ENSG00000170498.9	49.93342972	0.15177529	0.182251338	0.832780114	0.404968752	0.626480291	KISS1	protein_coding
ENSG00000170500.13	455.2135352	0.05779109	0.196944914	0.293437842	0.769187504	0.883098426	LONRF2	protein_coding
ENSG00000170502.13	1358.667664	-0.112281584	0.054917858	-2.044536843	0.040900542	0.156111316	NUDT9	protein_coding
ENSG00000170509.12	24.81043995	-0.119111209	0.122001151	-0.976312177	0.328909788	0.558352233	HSD17B13	protein_coding
ENSG00000170515.14	7335.002531	-0.185567732	0.069779873	-2.659330321	0.007829616	0.050992552	PA2G4	protein_coding
ENSG00000170516.17	14.55053283	1.133574074	0.455529331	2.488476582	0.012829169	0.071922897	COX7B2	protein_coding
ENSG00000170522.10	2092.533027	-0.054671923	0.099828344	-0.547659322	0.583925848	0.766903278	ELOVL6	protein_coding
ENSG00000170523.3	6.239730438	0.941798264	0.291415276	3.23180815	0.001230096	0.01344467	KRT83	protein_coding
ENSG00000170525.21	4941.509	0.213008679	0.082863554	2.570595498	0.010152383	0.06109797	PFKFB3	protein_coding
ENSG00000170537.13	954.3982854	0.265394413	0.106169187	2.499731041	0.012428763	0.070238385	TMC7	protein_coding
ENSG00000170540.15	14465.82095	0.002231561	0.075745961	0.029461127	0.976496821	0.989380056	ARL6IP1	protein_coding
ENSG00000170542.6	2567.553251	0.048820666	0.106339777	0.459100698	0.646161855	0.808969321	SERPINB9	protein_coding

ENSG00000170545.17	2735.115847	-0.215539984	0.084537781	-2.549629069	0.010783759	0.063675318	SMAGP	protein_coding
ENSG00000170549.4	5.550143936	1.004443813	0.254260448	3.950452464	7.80036E-05	0.001636573	IRX1	protein_coding
ENSG00000170558.10	288.5587658	0.488689409	0.167205228	2.922692161	0.003470194	0.028656557	CDH2	protein_coding
ENSG00000170561.13	1353.53555	-0.094114074	0.235990169	-0.398805061	0.690036848	0.836404672	IRX2	protein_coding
ENSG00000170571.12	3413.059403	0.257322125	0.111900744	2.299556872	0.021473338	0.101966181	EMB	protein_coding
ENSG00000170577.8	714.414964	0.096911233	0.222219447	0.436105997	0.662759827	0.819437792	SIX2	protein_coding
ENSG00000170579.17	110.6722131	0.512649092	0.203417751	2.520178745	0.011729525	0.067476901	DLGAP1	protein_coding
ENSG00000170581.14	5658.744423	0.082322336	0.055857157	1.473801025	0.140535172	0.338328106	STAT2	protein_coding
ENSG00000170584.11	1388.288404	-0.126439742	0.050671054	-2.495305154	0.012584885	0.070881677	NUDCD2	protein_coding
ENSG00000170604.5	2869.433538	0.032331156	0.075679023	0.427214235	0.669223294	0.823639562	IRF2BP1	protein_coding
ENSG00000170605.6	0.732267531	0.951110557	0.509883124	1.86535014	0.062132341		OR9K2	protein_coding
ENSG00000170606.15	7098.008467	0.001892276	0.050672876	0.037342969	0.970211545	0.986050995	HSPA4	protein_coding
ENSG00000170608.3	2327.224524	-0.142717158	0.130297792	-1.095315246	0.27337855	0.50191772	FOXA3	protein_coding
ENSG00000170613.4	1.889323623	-0.019844422	0.448454226	-0.044250719	0.964704554	0.984034324	FAM71B	protein_coding
ENSG00000170615.15	22.60507543	0.101264453	0.217940997	0.464641597	0.642188139	0.806403842	SLC26A5	protein_coding
ENSG00000170619.10	1209.287221	0.038907497	0.057754252	0.673673288	0.500519048	0.705838599	COMMD5	protein_coding
ENSG00000170624.13	1455.448442	0.002017339	0.147420347	0.013684262	0.98908188	0.994972669	SGCD	protein_coding
ENSG00000170627.11	171.2985967	-0.471770433	0.215298944	-2.191234313	0.028434841	0.122932821	GTSF1	protein_coding
ENSG00000170629.14	166.0327102	0.0513382	0.119238549	0.430550353	0.666795347	0.822014732	DPY19L2P2	transcribed_unprocessed_pseudogene
ENSG00000170631.15	689.2457394	-0.094507452	0.050991985	-1.853378555	0.063828119	0.207756045	ZNF16	protein_coding
ENSG00000170632.14	2666.819616	-0.111132154	0.065379223	-1.699808429	0.089166966	0.256833159	ARMC10	protein_coding
ENSG00000170633.16	2063.584488	-0.106507981	0.041075508	-2.592980264	0.009514823	0.058345257	RNF34	protein_coding
ENSG00000170634.13	489.3104461	0.080909181	0.061294436	1.320008566	0.186832158	0.401808848	ACYP2	protein_coding
ENSG00000170638.10	3815.853608	0.022617916	0.065441673	0.34561946	0.729628719	0.859278707	TRABD	protein_coding
ENSG00000170653.19	2711.744385	0.017110823	0.039864557	0.429223966	0.667760243	0.822609491	ATF7	protein_coding
ENSG00000170667.15	143.9050092	-0.302638275	0.107167451	-2.823975687	0.004743198	0.035884436	RASA4B	protein_coding
ENSG00000170677.6	2687.609944	0.11221835	0.07292118	1.538899255	0.123828893	0.31327177	SOCS6	protein_coding
ENSG00000170681.7	36.40177861	0.345155631	0.101316799	3.406696957	0.000657541	0.008336823	CAVIN4	protein_coding
ENSG00000170683.6	2.212297901	-0.408856081	0.313590369	-1.303790299	0.192305091	0.408545191	OR10A3	protein_coding
ENSG00000170684.9	616.2133536	-0.289905242	0.093173734	-3.111448132	0.001861722	0.018159691	ZNF296	protein_coding
ENSG00000170688.4	0.502072966	0.192006637	0.380173516	0.505050006	0.613523714		OR5E1P	unprocessed_pseudogene
ENSG00000170689.10	1145.189058	0.635693371	0.177026365	3.590953087	0.000329471	0.004992126	HOXB9	protein_coding
ENSG00000170703.15	311.8479334	-0.404706703	0.17609309	-2.298254302	0.02154732	0.102172748	TTLL6	protein_coding
ENSG00000170734.12	1806.483677	0.008156514	0.066637563	0.12240114	0.902581336	0.953683147	POLH	protein_coding
ENSG00000170743.17	17.60009435	-0.123668659	0.210350181	-0.587918007	0.556587329	0.747173281	SYT9	protein_coding
ENSG00000170745.12	680.4676073	0.318718165	0.119956487	2.656948145	0.007885157	0.051237196	KCNS3	protein_coding
ENSG00000170748.7	1.944049898	-0.276852894	0.238864517	-1.15903734	0.246440965	0.47293297	RBMXL2	protein_coding
ENSG00000170759.11	18953.45865	-0.02431709	0.060451542	-0.402257564	0.687494482	0.835022772	KIF5B	protein_coding
ENSG00000170775.3	162.9130029	0.955845776	0.199664085	4.787269468	1.69066E-06	7.82155E-05	GPR37	protein_coding
ENSG00000170776.22	10668.05104	0.244701205	0.064604289	3.787692865	0.000152053	0.002757386	AKAP13	protein_coding
ENSG00000170777.10	1.868817723	0.100071862	0.348862047	0.286852247	0.774225456	0.885778642	TPD52L3	protein_coding
ENSG00000170779.11	1249.362689	0.072062564	0.07595623	0.948738023	0.34275387	0.572596719	CDCA4	protein_coding

ENSG00000170782.3	2.148273765	0.137390808	0.2182175	0.629604902	0.528953115	0.727165795	OR10A4	protein_coding
ENSG00000170786.12	1325.924406	0.585373228	0.179854461	3.254705086	0.001135102	0.012657197	SDR16C5	protein_coding
ENSG00000170788.14	2.395625452	-0.397739266	0.305180529	-1.303291753	0.192475176	0.408647539	DYDC1	protein_coding
ENSG00000170790.5	31.99264661	-0.054131631	0.185003642	-0.292597652	0.769829709	0.883509684	OR10A2	protein_coding
ENSG00000170791.18	1615.558582	-0.068592219	0.065506853	-1.0470999	0.295053505	0.525020088	CHCHD7	protein_coding
ENSG00000170801.10	4207.322412	0.665365594	0.125087472	5.319202506	1.04223E-07	8.31819E-06	HTRA3	protein_coding
ENSG00000170802.16	2095.510028	0.018131882	0.057384728	0.315970517	0.752024886	0.872682906	FOXN2	protein_coding
ENSG00000170807.12	4.505925403	-0.290143941	0.206219276	-1.406968093	0.15943685	0.365307893	LMOD2	protein_coding
ENSG00000170819.5	15.71491588	-0.719578198	0.151732391	-4.742416526	2.11184E-06	9.381E-05	BFSP2	protein_coding
ENSG00000170820.12	2.897026193	-0.343189549	0.318037513	-1.079085124	0.280549782	0.509417803	FSHR	protein_coding
ENSG00000170827.9	154.3894659	-1.077517181	0.286733706	-3.757902049	0.000171344	0.003015612	CELP	transcribed_unprocessed_pseudogene
ENSG00000170832.13	2831.265875	0.143422048	0.046280455	3.098976583	0.001941904	0.018722724	USP32	protein_coding
ENSG00000170835.16	1123.093253	-2.097048119	0.268814341	-7.801102081	6.13688E-15	5.84954E-12	CEL	protein_coding
ENSG00000170836.12	1171.279493	0.042303729	0.04381202	0.965573566	0.334257608	0.563657324	PPM1D	protein_coding
ENSG00000170837.3	144.2756794	0.40893638	0.206803092	1.977419078	0.047994279	0.173362625	GPR27	protein_coding
ENSG00000170846.17	720.2376708	0.145695401	0.058663868	2.483562795	0.01300754	0.072623903	AC093323.1	protein_coding
ENSG00000170848.16	3.849362352	-0.207019004	0.347258948	-0.596151676	0.551073905	0.743453229	PSG6	protein_coding
ENSG00000170852.11	3068.478846	0.057930275	0.043252318	1.339356534	0.180454632	0.393458997	KBTBD2	protein_coding
ENSG00000170854.18	1417.99927	0.144852765	0.052479204	2.760193645	0.005776711	0.041248948	RIOX2	protein_coding
ENSG00000170855.4	1303.050328	-0.212984245	0.055930134	-3.808041051	0.000140072	0.002576712	TRIAP1	protein_coding
ENSG00000170858.10	10.84557815	-0.38456848	0.197461261	-1.947564187	0.051467132	0.181220364	LILRP2	transcribed_processed_pseudogene
ENSG00000170860.4	3289.59157	-0.100106451	0.061901861	-1.617179993	0.105839434	0.284842206	LSM3	protein_coding
ENSG00000170871.12	3033.448177	0.06547462	0.056832536	1.15206225	0.249295494	0.47601217	KIAA0232	protein_coding
ENSG00000170873.19	1148.165518	0.147292338	0.123898272	1.188816723	0.234511796	0.459088973	MTSS1	protein_coding
ENSG00000170876.8	4762.513709	0.196681821	0.0561111	3.505221252	0.000456228	0.006353802	TMEM43	protein_coding
ENSG00000170881.5	1943.021718	0.04052756	0.050606673	0.80083432	0.423227567	0.642366314	RNF139	protein_coding
ENSG00000170889.14	34152.51699	-0.219257517	0.062649867	-3.499728389	0.000465732	0.006463447	RPS9	protein_coding
ENSG00000170890.14	31.65754693	-2.582524848	0.234222182	-11.02596189	2.8644E-28	6.24109E-24	PLA2G1B	protein_coding
ENSG00000170891.11	75.18753143	0.215501736	0.145473127	1.481385188	0.138503959	0.335198658	CYTL1	protein_coding
ENSG00000170892.12	2427.106871	0.096207861	0.062077496	1.549802536	0.121188918	0.309376069	TSEN34	protein_coding
ENSG00000170893.4	5.633513296	0.162900389	0.195600903	0.832820228	0.404946125	0.626479739	TRH	protein_coding
ENSG00000170899.11	947.4967755	-0.298020404	0.111653608	-2.669151581	0.007604312	0.04990559	GSTA4	protein_coding
ENSG00000170903.11	761.453639	0.070086712	0.06773935	1.03465286	0.300831058	0.530497043	MSANTD4	protein_coding
ENSG00000170906.16	4024.951435	-0.200524647	0.08689853	-2.307572377	0.021022932	0.100528511	NDUFA3	protein_coding
ENSG00000170909.13	317.5429553	0.229136266	0.116566794	1.965707876	0.049332365	0.176461433	OSCAR	protein_coding
ENSG00000170915.9	4184.61296	-0.182442142	0.103919994	-1.755601926	0.079156403	0.238580618	PAQR8	protein_coding
ENSG00000170917.14	106.2218667	0.078906925	0.080620344	0.97874707	0.327704968	0.557262132	NUDT6	protein_coding
ENSG00000170919.16	1057.05639	-0.014839664	0.092711884	-0.160062157	0.872832111	0.938543279	TPT1-AS1	lncRNA
ENSG00000170920.2	1.846643917	0.004000316	0.418954939	0.009548319	0.992381659	0.996465388	OR7G3	protein_coding
ENSG00000170921.15	2043.942068	0.766863496	0.105852988	7.244608882	4.33689E-13	2.69984E-10	TANC2	protein_coding
ENSG00000170923.3	5.104342503	0.098676058	0.249608626	0.39532311	0.692604446	0.837699816	OR7G2	protein_coding
ENSG00000170925.3	1.302132614	1.015687455	0.32690456	3.106984666	0.001890062	0.018364154	TEX13B	protein_coding

ENSG00000170927.15	219.1049504	0.596761939	0.201275216	2.964905221	0.003027762	0.025849342	PKHD1	protein_coding
ENSG00000170929.6	4.098540522	0.172227649	0.26362911	0.653295261	0.513565947	0.715782744	OR1M1	protein_coding
ENSG00000170935.8	2.184944342	-0.186467639	0.225376863	-0.827359282	0.408033452	0.629075855	NCBP2L	protein_coding
ENSG00000170946.15	670.5612832	0.003672418	0.042425595	0.086561383	0.931020162	0.967290504	DNAJC24	protein_coding
ENSG00000170948.3	1.965573791	-0.168174231	0.270000029	-0.622867455	0.533371626	0.730696826	MBD3L1	protein_coding
ENSG00000170949.17	1430.563523	-0.004465805	0.085350754	-0.052322972	0.958271349	0.980570859	ZNF160	protein_coding
ENSG00000170950.6	1.943993522	0.129084094	0.19979812	0.646072615	0.518232329	0.71911254	PGK2	protein_coding
ENSG00000170953.3	2.091866339	0.26449148	0.391039975	0.676379646	0.498799637	0.704439712	OR8B12	protein_coding
ENSG00000170954.11	232.8223944	-0.149716782	0.131319097	-1.140099084	0.254245024	0.481078399	ZNF415	protein_coding
ENSG00000170955.10	1202.959284	0.387131963	0.112899475	3.42899702	0.000605816	0.007857725	CAVIN3	protein_coding
ENSG00000170956.17	53.65674252	0.518295648	0.148273149	3.495546237	0.000473092	0.006533882	CEACAM3	protein_coding
ENSG00000170959.14	20.46144344	0.231301454	0.177326534	1.30438152	0.192103533	0.408296135	DCDC1	protein_coding
ENSG00000170961.7	417.3468552	0.552372243	0.122023854	4.526756242	5.9896E-06	0.000217145	HAS2	protein_coding
ENSG00000170962.13	825.1993272	0.152920298	0.115984919	1.318449844	0.187353104	0.40235607	PDGFD	protein_coding
ENSG00000170965.10	36.12853331	0.690304867	0.220566653	3.129688276	0.001749919	0.017386984	PLAC1	protein_coding
ENSG00000170967.5	3.360715057	0.013479114	0.217452246	0.061986546	0.950573546	0.97689181	DDI1	protein_coding
ENSG00000170983.4	1.239322646	-0.661340165	0.512278402	-1.290978035	0.196711294	0.413970544	LINC00208	lncRNA
ENSG00000170989.10	1992.860926	-0.12251178	0.104245059	-1.175228651	0.239903251	0.46533713	S1PR1	protein_coding
ENSG00000171004.18	242.9369785	0.767972793	0.238817117	3.215735966	0.001301105	0.014003024	HS6ST2	protein_coding
ENSG00000171014.2	1.127499047	-0.09358065	0.454246188	-0.206013067	0.836780704	0.919831527	OR4D5	protein_coding
ENSG00000171016.13	498.2509694	0.120535692	0.132575922	0.909182375	0.363253866	0.590587386	PYGO1	protein_coding
ENSG00000171017.11	43.58826907	0.403607463	0.095784069	4.21372223	2.51196E-05	0.000660216	LRRC8E	protein_coding
ENSG00000171033.13	292.6310521	0.232467605	0.145960796	1.59267154	0.111233895	0.293309903	PKIA	protein_coding
ENSG00000171044.11	228.1848759	0.086680352	0.153912229	0.563180406	0.573312044	0.758907015	XKR6	protein_coding
ENSG00000171045.16	1001.058646	-0.055525332	0.077879759	-0.712962298	0.475869087	0.686039211	TSNARE1	protein_coding
ENSG00000171049.9	167.9883913	0.383033943	0.178336929	2.147810578	0.031728806	0.132367066	FPR2	protein_coding
ENSG00000171051.9	559.9650322	0.469791664	0.150191647	3.127948012	0.001760313	0.017461681	FPR1	protein_coding
ENSG00000171053.10	0.859708896	0.4392654	0.397998328	1.103686545	0.269729134		PATE1	protein_coding
ENSG00000171054.8	1.088920725	0.379786223	0.306850596	1.237691004	0.215830656	0.437006435	OR13H1	protein_coding
ENSG00000171055.15	2027.793271	-0.045866273	0.057090913	-0.803390081	0.421749306	0.641183397	FEZ2	protein_coding
ENSG00000171056.8	456.7674017	-0.051664297	0.117534352	-0.439567633	0.660250287	0.817889783	SOX7	protein_coding
ENSG00000171060.11	20.2071346	0.667281016	0.208569993	3.199314565	0.001377548	0.014673527	C8orf74	protein_coding
ENSG00000171067.11	4155.14691	0.157459851	0.067662762	2.32712715	0.019958499	0.097122446	C11orf24	protein_coding
ENSG00000171084.15	154.3201461	0.061524282	0.085254504	0.72165433	0.470507027	0.681761147	FAM86JP	transcribed_unprocessed_pseudogene
ENSG00000171094.18	20.32979839	-0.37008356	0.168691216	-2.193851994	0.028246052	0.122341535	ALK	protein_coding
ENSG00000171097.14	20.10969451	-0.04714544	0.104521343	-0.451060414	0.651946007	0.812708504	KYAT1	protein_coding
ENSG00000171100.15	1216.703742	0.050519831	0.059504797	0.849004341	0.395878877	0.619144163	MTM1	protein_coding
ENSG00000171101.13	58.74418626	-0.24325048	0.15041305	-1.617216596	0.105831535	0.284842206	SIGLEC17P	transcribed_unprocessed_pseudogene
ENSG00000171102.14	10.47116344	-0.755965303	0.298644697	-2.531320027	0.011363412	0.066042063	OBP2B	protein_coding
ENSG00000171103.11	486.9039344	-0.09190103	0.045352718	-2.026362116	0.04272769	0.160845245	TRMT61B	protein_coding
ENSG00000171105.14	7730.573687	0.165173271	0.061888998	2.668863231	0.007610843	0.049933413	INSR	protein_coding
ENSG00000171109.19	2875.41526	0.098536759	0.055708481	1.768792798	0.076928459	0.234116312	MFN1	protein_coding

ENSG00000171115.4	700.6676674	-0.253762467	0.099782034	-2.543167921	0.010985242	0.064454544	GIMAP8	protein_coding
ENSG00000171116.7	3.033313476	-0.02965822	0.181893242	-0.163052895	0.870476788	0.937444814	HSFX1	protein_coding
ENSG00000171119.2	230.05992	0.10679864	0.140934847	0.757787315	0.448578317	0.663603078	NRTN	protein_coding
ENSG00000171121.16	114.3384991	0.37086453	0.119766681	3.096558474	0.001957812	0.018816849	KCNMB3	protein_coding
ENSG00000171124.13	3263.680834	0.49595069	0.169710625	2.922331414	0.003474216	0.028663862	FUT3	protein_coding
ENSG00000171126.8	36.62717556	0.528387039	0.195881868	2.697478048	0.006986689	0.047006168	KCNG3	protein_coding
ENSG00000171130.18	1102.900447	-0.132794045	0.106196042	-1.25046134	0.211131069	0.431598459	ATP6V0E2	protein_coding
ENSG00000171132.14	852.3483685	-0.018711591	0.064848905	-0.288541363	0.772932373	0.885135838	PRKCE	protein_coding
ENSG00000171133.3	0.879810416	0.725282106	0.385535763	1.88123172	0.059940405		OR2K2	protein_coding
ENSG00000171135.15	2663.180438	-0.060373069	0.057111339	-1.057111776	0.290460577	0.520323957	JAGN1	protein_coding
ENSG00000171136.7	1.117514218	-0.65168457	0.291853634	-2.232915724	0.025554502	0.114625688	RLN3	protein_coding
ENSG00000171148.13	4830.655382	0.041666054	0.050833514	0.819657164	0.412411576	0.6330947	TADA3	protein_coding
ENSG00000171150.9	1722.809464	0.063744559	0.050525704	1.261626341	0.207083273	0.427023056	SOCS5	protein_coding
ENSG00000171155.8	2291.758389	-0.078433328	0.059762665	-1.312413503	0.189380653	0.404862397	C1GALT1C1	protein_coding
ENSG00000171159.5	6238.833331	-0.01332635	0.096056806	-0.138734051	0.88966031	0.947311602	C9orf16	protein_coding
ENSG00000171160.18	244.0806911	0.022271996	0.090768218	0.245372188	0.806168261	0.903629249	MORN4	protein_coding
ENSG00000171161.13	2354.901607	0.013669022	0.052058047	0.262572699	0.792879945	0.896174888	ZNF672	protein_coding
ENSG00000171163.16	1074.937246	-0.124105187	0.059405042	-2.08913557	0.036695521	0.145210911	ZNF692	protein_coding
ENSG00000171169.9	656.352917	-0.02146767	0.053622142	-0.400350847	0.688898122	0.835661616	NAIF1	protein_coding
ENSG00000171174.15	274.2901538	-0.218732507	0.07449527	-2.93619323	0.003322673	0.027737952	RBKS	protein_coding
ENSG00000171180.3	1.800118143	-0.303745853	0.35786121	-0.84878116	0.396003076	0.61931618	OR2M4	protein_coding
ENSG00000171189.17	27.93837765	0.095410432	0.111298636	0.857247092	0.391308342	0.615575019	GRIK1	protein_coding
ENSG00000171195.11	3.302510161	-0.623218527	0.341557434	-1.824637572	0.06805574	0.216077593	MUC7	protein_coding
ENSG00000171199.11	1.052904182	-0.489251013	0.416763267	-1.173930265	0.240422959	0.465816415	OPRPN	protein_coding
ENSG00000171201.12	1.070023493	-0.366915117	0.479324035	-0.765484494	0.443983096	0.660029727	SMR3B	protein_coding
ENSG00000171202.7	1063.922101	-0.052033501	0.060963443	-0.853519715	0.393371159	0.617438572	TMEM126A	protein_coding
ENSG00000171204.13	1788.258942	0.091363257	0.06399489	1.427664874	0.153388341	0.356966822	TMEM126B	protein_coding
ENSG00000171206.15	7512.323938	0.134368183	0.05855517	2.294727925	0.021748721	0.102769902	TRIM8	protein_coding
ENSG00000171208.10	841.3758176	0.432759017	0.140048182	3.090072356	0.002001077	0.019144007	NETO2	protein_coding
ENSG00000171209.3	2.191522937	-0.529733778	0.334290177	-1.584652543	0.1130453	0.29648962	CSN3	protein_coding
ENSG00000171217.5	18.93428333	-0.029138545	0.098410892	-0.296090652	0.767160853	0.88185828	CLDN20	protein_coding
ENSG00000171219.9	2520.053658	0.273889863	0.092713826	2.954142601	0.003135391	0.026550899	CDC42BPG	protein_coding
ENSG00000171222.10	3695.439428	-0.196432073	0.072161533	-2.722116147	0.006486534	0.044626412	SCAND1	protein_coding
ENSG00000171223.6	13434.50593	0.243204451	0.091591606	2.655313751	0.007923467	0.051418089	JUNB	protein_coding
ENSG00000171224.9	269.7338546	-0.030538037	0.10720225	-0.284863769	0.775748514	0.886356674	FAM241B	protein_coding
ENSG00000171227.7	1396.426427	-0.201998852	0.126480181	-1.597079079	0.110248081	0.291663467	TMEM37	protein_coding
ENSG00000171234.14	335.8934581	-0.497634182	0.214650262	-2.31834882	0.020430371	0.098592449	UGT2B7	protein_coding
ENSG00000171236.10	2105.119997	-0.050960999	0.134166919	-0.379832818	0.704069519	0.844747727	LRG1	protein_coding
ENSG00000171241.9	808.9770984	0.072259484	0.081579305	0.885757534	0.375748198	0.601608525	SHCBP1	protein_coding
ENSG00000171243.8	442.3478092	-0.790615626	0.249162103	-3.173097421	0.001508219	0.015591342	SOSTDC1	protein_coding
ENSG00000171246.6	387.6974205	0.385559939	0.246391269	1.564827927	0.117623262	0.303509526	NPTX1	protein_coding
ENSG00000171262.11	1771.142957	-0.104116855	0.048520115	-2.145849319	0.031885002	0.132682654	FAM98B	protein_coding

ENSG00000171291.8	232.6583717	0.110652091	0.105698968	1.046860652	0.295163852	0.525045757	ZNF439	protein_coding
ENSG00000171295.13	1345.679763	0.171311921	0.066549793	2.57419165	0.01004746	0.060692843	ZNF440	protein_coding
ENSG00000171298.13	6115.517074	0.149533348	0.092283516	1.620368995	0.105153036	0.283836339	GAA	protein_coding
ENSG00000171302.17	9384.834544	0.177361984	0.074860104	2.369245754	0.017824405	0.08985818	CANT1	protein_coding
ENSG00000171303.7	410.7497979	-0.097614256	0.198921604	-0.490717216	0.623626468	0.794677186	KCNK3	protein_coding
ENSG00000171307.19	1977.347266	0.017061981	0.047474052	0.359395931	0.719298919	0.853200745	ZDHHC16	protein_coding
ENSG00000171310.11	1810.600927	0.198350844	0.101362527	1.956845887	0.05036559	0.178792734	CHST11	protein_coding
ENSG00000171311.13	1445.811101	-0.163815628	0.051541904	-3.178299911	0.001481414	0.015385031	EXOSC1	protein_coding
ENSG00000171314.9	3549.277713	0.047235007	0.066103039	0.714566337	0.474877052	0.685276816	PGAM1	protein_coding
ENSG00000171316.12	3910.258786	-0.095344795	0.074045295	-1.287655018	0.197866078	0.415317667	CHD7	protein_coding
ENSG00000171320.15	1084.820294	-0.150907735	0.096697886	-1.560610483	0.118615684	0.305094774	ESCO2	protein_coding
ENSG00000171345.13	63358.93417	0.149017409	0.133219911	1.118582109	0.263318459	0.490977216	KRT19	protein_coding
ENSG00000171346.16	640.7139057	-0.014450975	0.184011643	-0.078532938	0.937404131	0.970291207	KRT15	protein_coding
ENSG00000171357.6	36.87955635	0.247118829	0.11918045	2.073484603	0.038127203	0.14923822	LURAP1	protein_coding
ENSG00000171360.4	2.755524051	0.25184005	0.456260939	0.551964958	0.580972362	0.764703314	KRT38	protein_coding
ENSG00000171365.17	1666.630899	-0.318416285	0.085471533	-3.725407437	0.000195	0.003324536	CLCN5	protein_coding
ENSG00000171368.12	1660.825392	-0.21910025	0.131655908	-1.664188518	0.096074762	0.268513933	TPPP	protein_coding
ENSG00000171385.10	1405.585616	-0.009200827	0.140165638	-0.065642529	0.947662429	0.975449811	KCND3	protein_coding
ENSG00000171388.12	690.0341963	-0.128001331	0.15793746	-0.810455804	0.417678256	0.637898762	APLN	protein_coding
ENSG00000171396.12	0.851886461	-0.935392223	0.510528102	-1.832205161	0.06692086		KRTAP4-4	protein_coding
ENSG00000171401.15	1552.717613	-0.002252287	0.400730956	-0.005620447	0.995515556	0.997927076	KRT13	protein_coding
ENSG00000171402.15	1.246761174	-0.485153869	0.316557028	-1.532595474	0.125375557	0.315679762	XAGE3	protein_coding
ENSG00000171403.10	8.774092797	0.110237565	0.223288485	0.493700177	0.62151794	0.793022781	KRT9	protein_coding
ENSG00000171405.12	2.105386604	3.045188395	0.585657463	5.199606571	1.99711E-07	1.42435E-05	XAGE5	protein_coding
ENSG00000171408.14	354.3466348	-0.11331922	0.152849226	-0.74137909	0.458463619	0.671838757	PDE7B	protein_coding
ENSG00000171421.13	1400.595441	-0.11182787	0.07181454	-1.557175886	0.119428732	0.30660692	MRPL36	protein_coding
ENSG00000171425.10	1015.789315	-0.127327833	0.071158883	-1.789345592	0.073559176	0.227087578	ZNF581	protein_coding
ENSG00000171428.15	666.8853568	-0.107816573	0.100428533	-1.073565151	0.283017624	0.511920487	NAT1	protein_coding
ENSG00000171431.4	6400.762029	-0.48106502	0.298715101	-1.610447608	0.107300174	0.287089071	KRT20	protein_coding
ENSG00000171433.12	69.08774908	-0.39113516	0.131616935	-2.971769261	0.002960891	0.025385978	GLOD5	protein_coding
ENSG00000171435.14	154.1405405	0.460156618	0.147432025	3.121144252	0.001801498	0.017728966	KSR2	protein_coding
ENSG00000171443.7	1266.725158	-0.00613254	0.070733694	-0.08669899	0.930910779	0.967241624	ZNF524	protein_coding
ENSG00000171444.18	1102.97693	0.218223673	0.114170958	1.911376391	0.055956228	0.191202428	MCC	protein_coding
ENSG00000171446.7	4.768809156	-0.234697244	0.224069671	-1.04742977	0.294901407	0.524945972	KRT27	protein_coding
ENSG00000171448.9	719.164287	0.02607022	0.061584268	0.423325967	0.672057451	0.825522819	ZBTB26	protein_coding
ENSG00000171450.6	58.95707339	-0.093276725	0.2035874	-0.458165509	0.646833538	0.809461406	CDK5R2	protein_coding
ENSG00000171451.14	288.6822427	0.068028321	0.136390757	0.498775154	0.617937793	0.790670793	DSEL	protein_coding
ENSG00000171453.20	1652.971724	0.081831014	0.070546326	1.159961385	0.246064528	0.472576199	POLR1C	protein_coding
ENSG00000171456.20	5832.845752	-0.04795812	0.057617746	-0.832349812	0.405211522	0.626766851	ASXL1	protein_coding
ENSG00000171459.4	0.824577422	-0.029105664	0.281084034	-0.103547908	0.917528129		OR1L6	protein_coding
ENSG00000171462.15	144.3283402	0.344085335	0.1014834	3.39055783	0.000697505	0.008726727	DLK2	protein_coding
ENSG00000171466.10	2424.457401	0.058527958	0.05429794	1.077903839	0.281076672	0.510013247	ZNF562	protein_coding

ENSG00000171467.16	3459.430184	0.162227397	0.068470636	2.36929883	0.017821847	0.08985568	ZNF318	protein_coding
ENSG00000171469.11	1864.704628	-0.004856949	0.047678485	-0.101868782	0.918860829	0.961719668	ZNF561	protein_coding
ENSG00000171475.14	7622.180345	-0.173514042	0.082796385	-2.095671712	0.036111323	0.14376239	WIPF2	protein_coding
ENSG00000171476.22	1951.279014	0.614682827	0.153843947	3.995495687	6.4559E-05	0.001408053	HOPX	protein_coding
ENSG00000171478.7	0.397351009	0.112671828	0.313214595	0.35972726	0.719051104		SPACA5B	protein_coding
ENSG00000171481.4	1.37622396	-0.323981974	0.300826916	-1.076971365	0.281493058	0.510471203	OR1L3	protein_coding
ENSG00000171483.13	3.671653354	-1.297074222	0.364720014	-3.556356031	0.000376034	0.00549493	SSX6P	transcribed_unprocessed_pseudogene
ENSG00000171487.15	9.361423333	0.299501702	0.315105253	0.950481463	0.341867669	0.571708013	NLRP5	protein_coding
ENSG00000171488.15	1371.431724	0.076965354	0.076163538	1.010527555	0.312242605	0.541771689	LRRC8C	protein_coding
ENSG00000171489.10	0.628883805	0.007286381	0.262617055	0.027745268	0.977865319		SPACA5	protein_coding
ENSG00000171490.13	9908.345845	-0.058392464	0.051927944	-1.124490195	0.260805128	0.488010641	RSL1D1	protein_coding
ENSG00000171492.14	1973.894527	-0.210042441	0.049801609	-4.217583418	2.46934E-05	0.000650978	LRRC8D	protein_coding
ENSG00000171495.17	11.12055866	-0.132626048	0.267424198	-0.495938844	0.619937559	0.791903547	MROH2B	protein_coding
ENSG00000171496.4	7.539583318	-0.179663191	0.139823038	-1.284932685	0.198815808	0.416738343	OR1L8	protein_coding
ENSG00000171497.5	1624.318345	-0.046442557	0.056057573	-0.828479631	0.407398926	0.628463511	PPID	protein_coding
ENSG00000171501.10	1.264761028	0.268425103	0.347195152	0.77312457	0.439448662	0.656019127	OR1N2	protein_coding
ENSG00000171502.15	154.8810569	0.755641808	0.136930615	5.51842851	3.42045E-08	3.32707E-06	COL24A1	protein_coding
ENSG00000171503.12	1347.313291	-0.176096512	0.071582905	-2.460035827	0.013892315	0.076024789	ETFDH	protein_coding
ENSG00000171505.5	0.536623505	0.088760929	0.400382695	0.221690224	0.824555041		OR1N1	protein_coding
ENSG00000171509.16	27.09366892	0.040726337	0.153586278	0.265169114	0.790879187	0.895031374	RXFP1	protein_coding
ENSG00000171517.6	57.46962018	0.286575398	0.200839009	1.426891115	0.153611282	0.357332987	LPAR3	protein_coding
ENSG00000171522.6	1625.680026	0.066160062	0.114981168	0.575399112	0.565021405	0.753541861	PTGER4	protein_coding
ENSG00000171530.15	3258.002495	-0.18954374	0.060620483	-3.126727638	0.001767636	0.017506426	TBCA	protein_coding
ENSG00000171532.5	33.4303761	-0.470142546	0.182694283	-2.57338401	0.01007094	0.060784119	NEUROD2	protein_coding
ENSG00000171533.11	335.2006552	0.019174868	0.157022246	0.122115615	0.902807456	0.953759842	MAP6	protein_coding
ENSG00000171540.8	4.99763329	0.168071131	0.199717408	0.841544722	0.400042842	0.622728091	OTP	protein_coding
ENSG00000171551.12	225.4521327	1.131929423	0.193475138	5.850516179	4.9005E-09	6.78509E-07	ECEL1	protein_coding
ENSG00000171552.14	7658.656488	-0.031483674	0.078235759	-0.402420512	0.687374577	0.835012589	BCL2L1	protein_coding
ENSG00000171557.17	78.62284512	-1.565931558	0.365073636	-4.289358103	1.7919E-05	0.000509698	FGG	protein_coding
ENSG00000171560.16	266.2042822	-1.702737867	0.307832472	-5.531378329	3.17724E-08	3.12539E-06	FGA	protein_coding
ENSG00000171561.4	9.490338083	-0.456001566	0.244618952	-1.864130163	0.062303428	0.204658259	OR2AT4	protein_coding
ENSG00000171564.12	282.599803	-0.435832569	0.326355708	-1.335452571	0.181728269	0.395168301	FGB	protein_coding
ENSG00000171566.12	2332.301342	-0.055404309	0.041968046	-1.320154605	0.186783405	0.401730439	PLRG1	protein_coding
ENSG00000171570.11	0						RAB4B-EGLN2	protein_coding
ENSG00000171574.18	533.1941955	-0.051380057	0.053126122	-0.967133588	0.333477256	0.563044925	ZNF584	protein_coding
ENSG00000171587.15	15.07791982	0.361409	0.220658656	1.637864592	0.101449944	0.277835424	DSCAM	protein_coding
ENSG00000171595.14	9.851794424	-0.123274682	0.191621307	-0.643324499	0.520013562	0.720263082	DNAI2	protein_coding
ENSG00000171596.7	124.5101294	-0.220407086	0.135668059	-1.624605579	0.104246628	0.282351625	NMUR1	protein_coding
ENSG00000171603.18	16533.95539	0.199337532	0.056009831	3.558974	0.000372306	0.005455277	CLSTN1	protein_coding
ENSG00000171604.12	3802.983076	0.049698296	0.088385962	0.562287206	0.573920354	0.759309348	CXXC5	protein_coding
ENSG00000171606.18	1025.047005	0.031144882	0.071149609	0.437737921	0.661576277	0.818694537	ZNF274	protein_coding
ENSG00000171608.16	1440.649625	-0.255949372	0.104487836	-2.449561429	0.014303031	0.077513143	PIK3CD	protein_coding

ENSG00000171611.10	14.31803292	-0.13945081	0.148899034	-0.936546106	0.348992086	0.578164086	PTCRA	protein_coding
ENSG00000171612.7	935.174772	-0.068643698	0.070839821	-0.968998745	0.332545815	0.562072337	SLC25A33	protein_coding
ENSG00000171617.14	8533.247974	-0.259541339	0.090631831	-2.863688577	0.004187395	0.032746409	ENC1	protein_coding
ENSG00000171621.14	2961.770378	0.246900037	0.073692118	3.350426641	0.000806872	0.009748004	SPSB1	protein_coding
ENSG00000171631.15	588.6615812	-0.001469131	0.111508557	-0.013175052	0.989488134	0.995266467	P2RY6	protein_coding
ENSG00000171634.18	7154.740142	0.006407899	0.042929752	0.149264753	0.881344727	0.943042753	BPTF	protein_coding
ENSG00000171643.14	11.69553586	-0.327585069	0.151373851	-2.164079643	0.030458238	0.128811978	S100Z	protein_coding
ENSG00000171649.12	137.6309109	-0.24807323	0.130824066	-1.896235438	0.057928919	0.1955662	ZIK1	protein_coding
ENSG00000171657.6	115.1560079	-0.586714954	0.10789084	-5.438042311	5.38692E-08	4.92909E-06	GPR82	protein_coding
ENSG00000171658.8	56.58282175	0.133883193	0.18436355	0.726191227	0.467721549	0.67922692	NMRAL2P	transcribed_unprocessed_pseudogene
ENSG00000171659.15	502.8865774	-0.176226932	0.119138475	-1.47917734	0.139092924	0.336157304	GPR34	protein_coding
ENSG00000171671.6	4.896342628	0.064060232	0.197814728	0.323839545	0.746059515	0.869303793	SHANK2-AS3	lncRNA
ENSG00000171680.23	1832.156823	0.141499937	0.08932359	1.584127292	0.113164755	0.296668212	PLEKHG5	protein_coding
ENSG00000171681.12	6279.924985	-0.059701852	0.04853102	-1.230179199	0.218630007	0.440240277	ATF7IP	protein_coding
ENSG00000171695.10	7.297662365	0.296575374	0.197894585	1.498653308	0.133963596	0.32879367	LKAAEAR1	protein_coding
ENSG00000171700.14	1404.114964	-0.127510644	0.093429393	-1.364780827	0.172321947	0.382911298	RGS19	protein_coding
ENSG00000171703.17	1094.68397	-0.061704291	0.108638824	-0.567976428	0.570050974	0.756635841	TCEA2	protein_coding
ENSG00000171711.3	18.64746734	-0.070668959	0.408165395	-0.173138046	0.86254292	0.932958519	DEFB4A	protein_coding
ENSG00000171714.12	432.105756	0.085635818	0.172069909	0.497680379	0.61870934	0.791175604	ANO5	protein_coding
ENSG00000171720.10	1884.101556	-0.095596978	0.04319035	-2.213387417	0.026870947	0.118553739	HDAC3	protein_coding
ENSG00000171722.13	24.40625492	0.14559875	0.131663013	1.105843983	0.268794054	0.496828915	SPATA46	protein_coding
ENSG00000171723.16	1312.418899	-0.086453839	0.067158778	-1.287305125	0.197987958	0.415433419	GPHN	protein_coding
ENSG00000171724.3	248.3160566	-0.369550461	0.135644087	-2.724412613	0.006441597	0.044429486	VAT1L	protein_coding
ENSG00000171729.14	3165.46454	-0.039288238	0.061384396	-0.640036242	0.522149038	0.721812284	TMEM51	protein_coding
ENSG00000171735.19	2152.055875	-0.0789799	0.05454335	-1.448020692	0.147611264	0.348850591	CAMTA1	protein_coding
ENSG00000171747.9	31952.82703	-0.082656676	0.149512597	-0.552840879	0.580372373	0.764259849	LGALS4	protein_coding
ENSG00000171757.17	96.54395245	0.067594698	0.106440993	0.635043845	0.52539982	0.72434979	LRRC34	protein_coding
ENSG00000171759.10	87.60927471	-1.049520482	0.274292877	-3.826276832	0.000130096	0.002421697	PAH	protein_coding
ENSG00000171763.20	848.7718162	-0.132004837	0.059062001	-2.235021405	0.025415944	0.114274718	SPATA5L1	protein_coding
ENSG00000171766.17	5795.327525	-0.642247047	0.141664339	-4.533583041	5.79915E-06	0.000211295	GATM	protein_coding
ENSG00000171772.17	2.449528697	0.008498818	0.290558617	0.029249926	0.976665263	0.989448602	SYCE1	protein_coding
ENSG00000171773.3	0.473445898	-0.493806582	0.322854721	-1.529500885	0.12614032		NXNL1	protein_coding
ENSG00000171777.16	177.3005201	0.142552768	0.104536939	1.363659298	0.17267482	0.383382617	RASGRP4	protein_coding
ENSG00000171786.6	17.68133408	0.152488501	0.12117371	1.25842892	0.208236678	0.428196005	NHLH1	protein_coding
ENSG00000171790.15	32.49004719	0.157555743	0.096632185	1.630468597	0.103002497	0.280446099	SLFNL1	protein_coding
ENSG00000171791.14	950.8664552	-0.300266702	0.102377596	-2.932933712	0.003357756	0.02792384	BCL2	protein_coding
ENSG00000171792.11	1854.328209	-0.062195361	0.064462255	-0.96483378	0.334628073	0.564082146	RHNO1	protein_coding
ENSG00000171793.16	2092.83608	-0.039404323	0.061053061	-0.645411096	0.518660815	0.71943221	CTPS1	protein_coding
ENSG00000171794.4	3.424205258	0.093742177	0.201217285	0.465875368	0.641304713	0.806014521	UTF1	protein_coding
ENSG00000171798.18	133.6825706	0.287585941	0.207017557	1.38918624	0.164776125	0.372526547	KNDC1	protein_coding
ENSG00000171804.11	18.02055804	0.068109899	0.217996838	0.312435262	0.754709755	0.874032654	WDR87	protein_coding
ENSG00000171806.12	353.438063	-0.046035939	0.051876531	-0.887413592	0.374856268	0.600577653	METTL18	protein_coding

ENSG00000171811.14	200.9122239	0.174868381	0.214294276	0.81601984	0.414488789	0.634876043	CFAP46	protein_coding
ENSG00000171812.13	941.6756493	0.387491495	0.129830922	2.984585557	0.002839628	0.024600886	COL8A2	protein_coding
ENSG00000171813.14	1493.049325	0.142992202	0.07121353	2.007935887	0.044650105	0.165407978	PWWP2B	protein_coding
ENSG00000171815.6	17.42873502	0.008025197	0.221576269	0.036218667	0.971108002	0.98634098	PCDHB1	protein_coding
ENSG00000171817.17	67.8061766	-0.396659009	0.111446	-3.559203635	0.000371981	0.005452344	ZNF540	protein_coding
ENSG00000171819.5	54.72095186	-0.207601059	0.200157033	-1.037190933	0.299646883	0.529463471	ANGPTL7	protein_coding
ENSG00000171823.7	1952.466404	0.198906278	0.083464085	2.38313615	0.017165844	0.087663893	FBXL14	protein_coding
ENSG00000171824.14	3252.550918	0.003804052	0.051656147	0.073641801	0.941295409	0.972219864	EXOSC10	protein_coding
ENSG00000171827.11	280.4436493	-0.149096228	0.108531887	-1.373755053	0.169517739	0.379096507	ZNF570	protein_coding
ENSG00000171840.12	257.9213969	0.068342383	0.092573594	0.738249207	0.460363026	0.673444546	NINJ2	protein_coding
ENSG00000171843.17	1327.850768	0.058871976	0.082113043	0.71696254	0.473397211	0.684219909	MLLT3	protein_coding
ENSG00000171847.11	89.90507918	0.152516099	0.163495771	0.932844307	0.350900361	0.579914481	FAM90A1	protein_coding
ENSG00000171848.16	6073.055972	-0.159626167	0.091844596	-1.738002817	0.082210317	0.244321012	RRM2	protein_coding
ENSG00000171853.16	2616.267702	0.011812152	0.041403634	0.285292643	0.775419949	0.886214564	TRAPPC12	protein_coding
ENSG00000171855.7	0.861845382	-0.323357553	0.313330532	-1.032001418	0.302071453		IFNB1	protein_coding
ENSG00000171858.18	28979.80146	-0.252038317	0.07884338	-3.196696014	0.001390113	0.014771268	RPS21	protein_coding
ENSG00000171860.5	1121.092769	0.032221077	0.121426164	0.265355305	0.790735764	0.894940456	C3AR1	protein_coding
ENSG00000171861.11	471.6699953	-0.07194091	0.06110218	-1.177386972	0.239041087	0.464427861	MRM3	protein_coding
ENSG00000171862.11	6768.541594	-0.059104286	0.056545031	-1.045260484	0.295902596	0.525600106	PTEN	protein_coding
ENSG00000171863.15	28756.19497	-0.256175833	0.06278941	-4.079921025	4.5051E-05	0.001059464	RPS7	protein_coding
ENSG00000171864.5	111.2806299	0.245515918	0.176703259	1.389424962	0.164703564	0.372459119	PRND	protein_coding
ENSG00000171865.10	1761.000872	0.003725453	0.041409562	0.089966003	0.928314231	0.966035235	RNASEH1	protein_coding
ENSG00000171867.17	7027.583296	0.026501396	0.098434368	0.269229093	0.787753394	0.893026266	PRNP	protein_coding
ENSG00000171872.5	20.44573802	0.076595626	0.1932956	0.396261611	0.691912044	0.837573563	KLF17	protein_coding
ENSG00000171873.8	140.9280515	-0.504865554	0.192173845	-2.627129378	0.008610859	0.054405595	ADRA1D	protein_coding
ENSG00000171877.21	563.232617	-0.001630099	0.122552225	-0.013301258	0.989387445	0.995194819	FRMD5	protein_coding
ENSG00000171885.18	17.33521714	-0.708686197	0.280622733	-2.525405514	0.011556485	0.066843239	AQP4	protein_coding
ENSG00000171889.5	114.3388572	0.791256843	0.199311996	3.969940896	7.18905E-05	0.001536157	MIR31HG	lncRNA
ENSG00000171903.16	780.3548448	-0.183840369	0.185708652	-0.989939714	0.322203587	0.552064864	CYP4F11	protein_coding
ENSG00000171914.16	2881.634638	0.027505053	0.095992388	0.286533693	0.774469391	0.885888387	TLN2	protein_coding
ENSG00000171916.17	296.1235725	-0.787055333	0.20191777	-3.897900279	9.70304E-05	0.001933375	LGALS9C	protein_coding
ENSG00000171928.14	1563.465368	-0.039331757	0.061561762	-0.638899146	0.522888543	0.722356642	TVP23B	protein_coding
ENSG00000171931.13	26.08333108	-0.725005928	0.138820246	-5.222623842	1.76406E-07	1.2898E-05	FBXW10	protein_coding
ENSG00000171936.3	0.596810884	-0.131483329	0.558731791	-0.235324589	0.813956812		OR10H3	protein_coding
ENSG00000171940.13	4994.290193	-0.021681262	0.077872148	-0.278421264	0.780688998	0.888763375	ZNF217	protein_coding
ENSG00000171942.4	0.911173031	-0.330621655	0.389725025	-0.848345973	0.396245322		OR10H2	protein_coding
ENSG00000171943.12	473.2793555	0.183934741	0.056334632	3.265038452	0.001094492	0.012314662	SRGAP2C	protein_coding
ENSG00000171944.2	1.618071695	-0.078791619	0.438668866	-0.179615253	0.85745463	0.930410866	OR52A5	protein_coding
ENSG00000171951.5	257.8709931	0.305513101	0.177175936	1.724348733	0.084644907	0.248683607	SCG2	protein_coding
ENSG00000171953.16	688.4829111	-0.092271298	0.054059555	-1.706845287	0.087850812	0.254570323	ATPAF2	protein_coding
ENSG00000171954.13	34.07170863	0.155784797	0.154441312	1.008698999	0.313119012	0.54257709	CYP4F22	protein_coding
ENSG00000171956.7	13.14516863	0.193042858	0.169953753	1.135855222	0.256017161	0.48300578	FOXB1	protein_coding

ENSG00000171960.12	1319.891183	-0.170546828	0.059080589	-2.886681237	0.003893284	0.031153478	PPIH	protein_coding
ENSG00000171962.18	181.1686654	-0.031641789	0.073267792	-0.431864918	0.665839593	0.821294163	DRC3	protein_coding
ENSG00000171970.13	40.33820783	0.082239316	0.080199067	1.025439802	0.305155716	0.534648224	ZNF57	protein_coding
ENSG00000171984.15	324.2961739	-0.319542676	0.073986705	-4.318920251	1.56794E-05	0.000460969	SHLD1	protein_coding
ENSG00000171987.3	1.411566924	-0.336510674	0.459781086	-0.731893252	0.464233725	0.676372007	C11orf40	lncRNA
ENSG00000171988.19	5284.321807	0.050967026	0.057534501	0.88585153	0.375697538	0.601549515	JMJD1C	protein_coding
ENSG00000171989.6	25.15222803	0.349885608	0.1146894	3.050723148	0.00228291	0.021032211	LDHAL6B	protein_coding
ENSG00000171992.13	10056.75463	-0.089047732	0.106390223	-0.836991688	0.40259724	0.624474263	SYNPO	protein_coding
ENSG00000171999.7	0.620681531	0.046201745	0.318538083	0.145043079	0.884676859		OR52P2P	unitary_pseudogene
ENSG00000172000.7	81.96988288	0.216667378	0.181951689	1.190796185	0.233733607	0.458306758	ZNF556	protein_coding
ENSG00000172005.11	260.4264533	-0.282227534	0.227397514	-1.241119697	0.214561534	0.43557011	MAL	protein_coding
ENSG00000172006.12	304.6472614	-0.017334963	0.061690879	-0.280997172	0.778712569	0.888114256	ZNF554	protein_coding
ENSG00000172007.7	976.4380725	-0.016861757	0.050897143	-0.331290829	0.740424818	0.86582082	RAB33B	protein_coding
ENSG00000172009.15	2855.461289	-0.002516149	0.068924188	-0.036506038	0.970878865	0.98634098	THOP1	protein_coding
ENSG00000172014.12	13.87213119	-0.32138865	0.219291483	-1.465577442	0.142763441	0.341505322	ANKRD20A4P	protein_coding
ENSG00000172016.16	4459.557322	-1.111003478	0.335932732	-3.307220083	0.000942268	0.010967167	REG3A	protein_coding
ENSG00000172020.13	74.50841265	-0.01591536	0.192317975	-0.082755446	0.934045996	0.96886345	GAP43	protein_coding
ENSG00000172023.8	727.7585868	0.095092493	0.391756437	0.242733708	0.808211693	0.904918832	REG1B	protein_coding
ENSG00000172031.7	166.7533944	0.991145611	0.147296408	6.728919084	1.70928E-11	6.17859E-09	EPHX4	protein_coding
ENSG00000172037.14	12308.67837	0.292337291	0.08069606	3.622695958	0.000291548	0.004550432	LAMB2	protein_coding
ENSG00000172046.19	3692.36874	0.07083074	0.043071125	1.64450638	0.100071648	0.275632168	USP19	protein_coding
ENSG00000172053.18	10092.8491	-0.065223076	0.052553919	-1.241069672	0.214580012	0.435587328	QARS1	protein_coding
ENSG00000172057.10	4243.286851	-0.230975508	0.095255227	-2.424806638	0.015316542	0.081227816	ORMDL3	protein_coding
ENSG00000172058.16	5.870337261	-0.116509127	0.128204883	-0.908772933	0.363469997	0.590784485	SERF1A	protein_coding
ENSG00000172059.11	1327.323674	0.116038549	0.078655362	1.475278296	0.140137739	0.337623512	KLF11	protein_coding
ENSG00000172061.9	1103.021193	0.776182862	0.177743404	4.366872937	1.26038E-05	0.000389805	LRRC15	protein_coding
ENSG00000172062.17	234.2413562	-0.115803487	0.071764968	-1.61364924	0.10660353	0.285997785	SMN1	protein_coding
ENSG00000172071.14	1827.383067	0.063372801	0.057126222	1.109346968	0.26728053	0.495270812	EIF2AK3	protein_coding
ENSG00000172073.4	1.658215339	-0.051440484	0.369611269	-0.139174555	0.889312215	0.947172037	TEX37	protein_coding
ENSG00000172081.14	4114.83995	-0.11780922	0.059282077	-1.987265395	0.046892994	0.170672013	MOB3A	protein_coding
ENSG00000172086.9	1282.574683	-0.047119894	0.074828894	-0.629701865	0.528889662	0.727116914	KRCC1	protein_coding
ENSG00000172113.9	1416.799898	0.062972578	0.040234016	1.565157667	0.117545945	0.30341782	NME6	protein_coding
ENSG00000172115.9	13334.8432	-0.251575524	0.071947433	-3.496657385	0.000471126	0.006511348	CYCS	protein_coding
ENSG00000172116.23	298.1625366	-0.218225788	0.157086226	-1.38921021	0.164768838	0.372526547	CD8B	protein_coding
ENSG00000172123.13	306.4817213	-0.248024955	0.096840862	-2.561160124	0.010432326	0.062122093	SLFN12	protein_coding
ENSG00000172137.19	187.7388313	0.9552922	0.221200986	4.318661586	1.56978E-05	0.000460969	CALB2	protein_coding
ENSG00000172139.15	44.73376574	0.325300403	0.123645875	2.630903812	0.008515813	0.053961553	SLC9C1	protein_coding
ENSG00000172146.3	6.382596391	-0.100489539	0.272894837	-0.368235399	0.712697717	0.849811974	OR1A1	protein_coding
ENSG00000172148.3	1.03663868	-0.35793947	0.415600811	-0.861257871	0.389096033	0.613600652	OR7A2P	transcribed_unprocessed_pseudogene
ENSG00000172150.4	1.106096074	-0.102040064	0.479579373	-0.212769918	0.831506428	0.916936385	OR1A2	protein_coding
ENSG00000172154.10	0.187794877	0.136884807	0.883075444	0.155009187	0.876814075		OR8I2	protein_coding
ENSG00000172155.10	0.347030229	0.680292148	0.681135332	0.99876209	0.317909955		LCE1D	protein_coding

ENSG00000172156.4	863.8113318	-0.268151653	0.160479418	-1.67094108	0.094733316	0.266183026	CCL11	protein_coding
ENSG00000172159.16	428.9798271	0.15721544	0.121517118	1.293771952	0.195744204	0.41277257	FRMD3	protein_coding
ENSG00000172164.15	2466.315029	0.043303966	0.137195929	0.315635939	0.752278856	0.87286933	SNTB1	protein_coding
ENSG00000172167.8	546.7019546	0.042151909	0.081975389	0.514202001	0.607110769	0.783303212	MTBP	protein_coding
ENSG00000172171.11	339.1306499	-0.010429656	0.042153647	-0.24742001	0.80458319	0.902920905	TEFM	protein_coding
ENSG00000172172.8	2658.470728	-0.134520375	0.06959094	-1.933015627	0.053234273	0.18506501	MRPL13	protein_coding
ENSG00000172175.15	2202.330888	-0.100979547	0.067658107	-1.492497381	0.135568803	0.331166643	MALT1	protein_coding
ENSG00000172179.12	6.619447735	0.36751677	0.220653023	1.665586837	0.095795735	0.268007879	PRL	protein_coding
ENSG00000172183.15	2203.043444	0.236148187	0.104506801	2.259644191	0.023843343	0.10935149	ISG20	protein_coding
ENSG00000172186.7	2.136375145	-0.023256142	0.140230593	-0.165842141	0.868281181	0.936144516	HMGN1P35	processed_pseudogene
ENSG00000172188.6	0.844059611	-0.13152047	0.647700048	-0.203057682	0.839089947		OR4C11	protein_coding
ENSG00000172197.11	1776.556224	-0.198793918	0.089956347	-2.209893181	0.027112577	0.119281651	MBOAT1	protein_coding
ENSG00000172199.1	0.415467098	-0.014706488	0.651580851	-0.022570473	0.981992897		OR8U1	protein_coding
ENSG00000172201.12	1032.44628	0.009193124	0.125529086	0.07323501	0.941619107	0.972345757	ID4	protein_coding
ENSG00000172208.6	1.197990373	-0.330664833	0.437095548	-0.756504693	0.44934666	0.664156818	OR4X2	protein_coding
ENSG00000172209.6	9.285196267	-0.388077825	0.152843177	-2.539058874	0.011115112	0.065050124	GPR22	protein_coding
ENSG00000172215.6	504.6342796	-0.576443256	0.123715059	-4.659442939	3.17066E-06	0.000129371	CXCR6	protein_coding
ENSG00000172216.6	7352.414154	0.291051596	0.084531549	3.443112052	0.000575061	0.007566254	CEBPB	protein_coding
ENSG00000172232.10	6.506542446	0.265627209	0.134367923	1.976864737	0.048056922	0.173502608	AZU1	protein_coding
ENSG00000172236.18	1351.859958	-0.261406458	0.155792527	-1.677913977	0.093363894	0.264103758	TPSAB1	protein_coding
ENSG00000172238.6	245.94552	0.973504753	0.282770272	3.442740806	0.000575851	0.00757436	ATOH1	protein_coding
ENSG00000172239.14	3933.971979	0.020247977	0.057085016	0.354698628	0.722815379	0.855314485	PAIP1	protein_coding
ENSG00000172243.18	561.1868287	-0.150930831	0.110464266	-1.366331724	0.171834868	0.382394446	CLEC7A	protein_coding
ENSG00000172244.9	231.6290963	-0.085617037	0.092050596	-0.930108444	0.352314939	0.58090955	C5orf34	protein_coding
ENSG00000172247.4	37.18277496	-0.038811653	0.165506948	-0.234501653	0.81459555	0.908448632	C1QTNF4	protein_coding
ENSG00000172250.16	5.430316815	-0.126695285	0.173279345	-0.731162073	0.464680163	0.676647981	SERHL	transcribed_unitary_pseudogene
ENSG00000172260.15	895.626301	-0.368576473	0.16783319	-2.196088107	0.028085638	0.121925466	NEGR1	protein_coding
ENSG00000172262.11	2124.770756	-0.014013716	0.05852049	-0.239466824	0.810743619	0.906315816	ZNF131	protein_coding
ENSG00000172264.17	536.1871848	-0.434445382	0.171212486	-2.537463199	0.011165911	0.065259776	MACROD2	protein_coding
ENSG00000172269.19	3760.241169	-0.025643251	0.055913837	-0.458620847	0.646506463	0.809238012	DPAGT1	protein_coding
ENSG00000172270.21	41927.32729	0.044774315	0.066716572	0.671112346	0.502148963	0.707104807	BSG	protein_coding
ENSG00000172273.13	952.8657693	-0.015216827	0.047026506	-0.323579786	0.746256193	0.869345474	HINFP	protein_coding
ENSG00000172283.10	0						PRYP4	unprocessed_pseudogene
ENSG00000172288.7	0.005998911	-0.138205087	2.893054209	-0.047771344	0.961898475		CDY1	protein_coding
ENSG00000172289.3	0.689186008	-0.313439892	0.510662162	-0.613791103	0.539353377		OR10V1	protein_coding
ENSG00000172292.15	4195.083625	-0.141082741	0.083660467	-1.686372852	0.091724007	0.261126089	CERS6	protein_coding
ENSG00000172294.7	0.079054397	-0.047095386	1.020687401	-0.046140852	0.963197986		CSPG4P4Y	unprocessed_pseudogene
ENSG00000172296.13	796.907647	-0.387835998	0.138611738	-2.798002573	0.00514197	0.037939661	SPTLC3	protein_coding
ENSG00000172297.7	0.085959944	-0.200983455	0.833132125	-0.241238392	0.809370357		GOLGA2P3Y	unprocessed_pseudogene
ENSG00000172301.11	1691.29247	0.002943171	0.065330203	0.045050691	0.964066904	0.983475981	COPRS	protein_coding
ENSG00000172315.6	1532.549135	-0.109838703	0.055856992	-1.966427108	0.049249295	0.176285655	TP53RK	protein_coding
ENSG00000172318.5	33.42802206	-0.339198407	0.212440566	-1.596674369	0.110338312	0.291868989	B3GALT1	protein_coding

ENSG00000172320.3	6.890554843	-0.089810835	0.345207575	-0.260164729	0.79473671	0.897148396	OR5A1	protein_coding
ENSG00000172322.14	104.573365	-0.242807843	0.145874556	-1.66449756	0.096013038	0.268409043	CLEC12A	protein_coding
ENSG00000172324.5	1.071586412	0.002648921	0.335023134	0.00790668	0.993691448	0.997138741	OR5A2	protein_coding
ENSG00000172331.12	1087.928828	-0.035433524	0.059292226	-0.597608269	0.550101346	0.742874375	BPGM	protein_coding
ENSG00000172336.5	1452.269361	-0.024576478	0.071405779	-0.344180511	0.730710538	0.859971726	POP7	protein_coding
ENSG00000172339.10	675.9524834	-0.160082245	0.052197799	-3.066839013	0.002163353	0.020251869	ALG14	protein_coding
ENSG00000172340.15	4856.352425	-0.168178481	0.06385451	-2.633776074	0.008444116	0.053655472	SUCLG2	protein_coding
ENSG00000172342.5	0.067398213	-0.416017445	1.242974543	-0.334695065	0.737855125		CSPG4P3Y	unprocessed_pseudogene
ENSG00000172345.14	122.9617217	-0.209372422	0.088732482	-2.359591642	0.018295061	0.091500504	STARD5	protein_coding
ENSG00000172346.15	168.447621	0.471634519	0.148499798	3.175994354	0.001493239	0.015489373	CSDC2	protein_coding
ENSG00000172348.15	1615.127004	-0.162471859	0.141412299	-1.14892312	0.250587683	0.477350038	RCAN2	protein_coding
ENSG00000172349.17	920.1743487	-0.433130104	0.12836002	-3.374338097	0.000739934	0.009149863	IL16	protein_coding
ENSG00000172350.10	28.92880667	0.359254601	0.119473157	3.006990112	0.002638483	0.023369348	ABCG4	protein_coding
ENSG00000172352.5	0.007447867	-0.122857395	2.893054209	-0.04246633	0.966126952		CDY1B	protein_coding
ENSG00000172354.10	16065.62557	0.032605758	0.065338807	0.499025914	0.617761128	0.790560809	GNB2	protein_coding
ENSG00000172361.6	48.02294992	0.029800714	0.094776713	0.314430757	0.753193899	0.873205335	CFAP53	protein_coding
ENSG00000172362.3	1.494718385	-0.554270095	0.331790567	-1.670542054	0.094812166	0.266264727	OR5B12	protein_coding
ENSG00000172365.3	0.985540601	0.259216307	0.382256844	0.678120773	0.497695121	0.703700853	OR5B2	protein_coding
ENSG00000172366.20	2251.342708	-0.297794208	0.080326366	-3.707303385	0.000209478	0.003513634	MCRIP2	protein_coding
ENSG00000172367.16	182.541017	-0.250533921	0.166252984	-1.506943907	0.131825036	0.325375811	PDZD3	protein_coding
ENSG00000172375.14	1224.112519	-0.028940945	0.057402275	-0.504177662	0.614136536	0.788319265	C2CD2L	protein_coding
ENSG00000172377.3	0.628561852	0.400982646	0.480307262	0.834846102	0.403804365		OR9I1	protein_coding
ENSG00000172379.21	1108.720353	-0.364904175	0.16428183	-2.221208365	0.026336852	0.1168956	ARNT2	protein_coding
ENSG00000172380.6	10088.05019	0.079755361	0.066810869	1.193748296	0.232576442	0.456944023	GNG12	protein_coding
ENSG00000172382.10	118.2848325	0.552017202	0.152992771	3.608126039	0.000308417	0.004745718	PRSS27	protein_coding
ENSG00000172399.6	10.29084801	-0.692103286	0.203876519	-3.394717983	0.000686993	0.008634622	MYOZ2	protein_coding
ENSG00000172403.11	20809.53532	-0.725473882	0.234143387	-3.098417134	0.001945574	0.018732273	SYNPO2	protein_coding
ENSG00000172404.5	14.06665093	-0.090991887	0.102633688	-0.886569401	0.375310774	0.601129033	DNAJB7	protein_coding
ENSG00000172409.6	630.552366	-0.027349122	0.048822235	-0.560177586	0.575358314	0.760276221	CLP1	protein_coding
ENSG00000172410.4	0.777074633	-0.618105792	0.398738226	-1.55015434	0.121104476		INSL5	protein_coding
ENSG00000172421.10	4.557587602	0.060643778	0.340977856	0.177852539	0.858838785	0.93126207	EFCAB3	protein_coding
ENSG00000172425.12	10.0037421	-0.065504109	0.130823045	-0.500707721	0.616576838	0.789787444	TTC36	protein_coding
ENSG00000172426.16	46.73414713	0.290221712	0.078913429	3.677722728	0.000235326	0.003856631	RSPH9	protein_coding
ENSG00000172428.11	1799.27575	-0.161109025	0.064909527	-2.482055125	0.013062707	0.072838588	COPS9	protein_coding
ENSG00000172432.19	3617.010619	0.384930469	0.066890838	5.754606799	8.68437E-09	1.06904E-06	GTPBP2	protein_coding
ENSG00000172456.18	396.9953781	-0.221121915	0.081785737	-2.703673322	0.006857767	0.046353486	FGGY	protein_coding
ENSG00000172457.7	0.745406228	0.890740148	0.522205438	1.705727446	0.088058837		OR9G4	protein_coding
ENSG00000172458.4	148.7933419	0.046254918	0.102997458	0.449087957	0.653368208	0.813713243	IL17D	protein_coding
ENSG00000172459.4	0.323507665	0.05969662	0.956660365	0.062401059	0.950243451		OR5AR1	protein_coding
ENSG00000172460.17	64.90582911	0.229692914	0.16098221	1.426821723	0.153631288	0.357341374	PRSS30P	lncRNA
ENSG00000172461.11	561.865227	-0.840375741	0.274091521	-3.066040634	0.002169138	0.02028859	FUT9	protein_coding
ENSG00000172464.3	1.815836674	-0.643344771	0.463044553	-1.38937985	0.164717274	0.372470793	OR5AP2	protein_coding

ENSG00000172465.14	690.8524838	-0.069731679	0.08246626	-0.845578283	0.397788045	0.620882181	TCEAL1	protein_coding
ENSG00000172466.16	4486.69844	-0.043597596	0.056107446	-0.777037618	0.437136577	0.654136211	ZNF24	protein_coding
ENSG00000172468.14	0.233119318	0.059450797	0.388209831	0.153140884	0.878287175		HSFY1	protein_coding
ENSG00000172469.16	1862.155929	-0.109720801	0.067831411	-1.617551503	0.105759288	0.28473202	MANEA	protein_coding
ENSG00000172476.4	27.55170596	0.438859074	0.123709809	3.547488073	0.000388923	0.005621265	RAB40A	protein_coding
ENSG00000172478.18	844.8999988	0.051976795	0.196995559	0.263847545	0.791897397	0.895592145	MAB21L4	protein_coding
ENSG00000172482.5	37.37767366	0.068154817	0.201796976	0.337739537	0.735559477	0.862835483	AGXT	protein_coding
ENSG00000172487.4	0.996567285	-0.798775443	0.555901485	-1.436901078	0.150746104	0.353087133	OR8J1	protein_coding
ENSG00000172489.7	0.424781252	-0.097467299	0.658351341	-0.148047544	0.882305247		OR5T3	protein_coding
ENSG00000172493.22	5683.607453	-0.03041197	0.05998984	-0.506952006	0.612188494	0.787117541	AFF1	protein_coding
ENSG00000172497.9	3.681027545	-0.565293577	0.212760733	-2.656945055	0.007885229	0.051237196	ACOT12	protein_coding
ENSG00000172500.13	3240.562505	-0.007611569	0.054297303	-0.140183183	0.888515259	0.946808578	FIBP	protein_coding
ENSG00000172508.10	270.1628538	0.661124839	0.166411491	3.972831654	7.10232E-05	0.00152387	CARNS1	protein_coding
ENSG00000172519.9	11.27290439	-0.306687573	0.226976746	-1.351184995	0.17663618	0.388787943	OR10H5	protein_coding
ENSG00000172530.20	839.7033831	0.024228497	0.040446145	0.599031065	0.549152169	0.74205694	BANP	protein_coding
ENSG00000172531.16	8953.699701	-0.008237811	0.067179301	-0.122624247	0.902404653	0.953682208	PPP1CA	protein_coding
ENSG00000172534.14	13353.233	0.102497456	0.05859261	1.749323944	0.080235039	0.240484374	HCFC1	protein_coding
ENSG00000172538.7	1.092152952	0.070533491	0.291373736	0.242072232	0.808724194	0.905260061	FAM170B	protein_coding
ENSG00000172543.8	409.6773988	-0.262131357	0.14648401	-1.789487851	0.073536282	0.227043402	CTSW	protein_coding
ENSG00000172548.14	132.5873882	-0.592966562	0.20559935	-2.884087727	0.003925494	0.031367682	NIPAL4	protein_coding
ENSG00000172551.11	18.01403287	-0.012928219	0.254684329	-0.050761739	0.95951538	0.981202975	MUCL1	protein_coding
ENSG00000172554.12	16.79390463	-0.055591814	0.184130723	-0.30191493	0.762716914	0.878818482	SNTG2	protein_coding
ENSG00000172568.5	0.54060227	-0.903155483	0.341327049	-2.646012043	0.008144692		FNDC9	protein_coding
ENSG00000172572.7	1730.365572	0.293771412	0.143242606	2.050866153	0.040279983	0.154623047	PDE3A	protein_coding
ENSG00000172575.12	580.1377132	-0.345215118	0.116764592	-2.956505147	0.00311147	0.02640478	RASGRP1	protein_coding
ENSG00000172578.12	911.5034801	-0.721123874	0.129178649	-5.582376648	2.37254E-08	2.44996E-06	KLHL6	protein_coding
ENSG00000172586.8	1968.91276	-0.114071971	0.065148181	-1.75096173	0.079952502	0.24004044	CHCHD1	protein_coding
ENSG00000172590.18	2270.639607	-0.193619827	0.06969633	-2.778049109	0.005468635	0.03959241	MRPL52	protein_coding
ENSG00000172594.13	1570.294399	-0.149713252	0.094203428	-1.589254819	0.112002875	0.294588923	SMPDL3A	protein_coding
ENSG00000172602.11	748.8060032	0.224793195	0.130010699	1.729036125	0.08380263	0.247173747	RND1	protein_coding
ENSG00000172613.8	1000.177406	-0.007431645	0.060166746	-0.123517486	0.901697328	0.953280392	RAD9A	protein_coding
ENSG00000172638.13	2420.271741	0.351467871	0.106129542	3.311687452	0.000927351	0.010834094	EFEMP2	protein_coding
ENSG00000172640.3	4.355005076	0.044147781	0.190779765	0.231407041	0.816998596	0.909801385	OR10AD1	protein_coding
ENSG00000172650.14	10.90716187	-0.037947458	0.113337004	-0.334819672	0.73776112	0.864222476	AGAP5	protein_coding
ENSG00000172661.19	2145.065547	-0.10004938	0.043983646	-2.27469502	0.022924231	0.106647724	WASHC2C	protein_coding
ENSG00000172663.9	1752.370989	0.00554294	0.070875232	0.078207013	0.937663383	0.970438302	TMEM134	protein_coding
ENSG00000172667.11	3033.269511	0.021087507	0.076474458	0.27574575	0.782743354	0.889995724	ZMAT3	protein_coding
ENSG00000172671.20	544.2218645	-0.036478887	0.058624528	-0.622246156	0.533780021	0.730980546	ZFAND4	protein_coding
ENSG00000172673.11	230.4516608	-0.644952063	0.143221681	-4.5031734	6.69462E-06	0.00023776	THEMIS	protein_coding
ENSG00000172680.1	0.680045107	0.184761402	0.477553765	0.386891311	0.698836674		MOS	protein_coding
ENSG00000172687.13	394.9055939	0.018796367	0.10035267	0.187303111	0.851422984	0.927213779	ZNF738	protein_coding
ENSG00000172689.2	19.56412086	-0.486970542	0.242219384	-2.010452401	0.044383329	0.164762438	MS4A10	protein_coding

ENSG00000172716.16	1979.915797	0.068328333	0.102115213	0.669129808	0.503412677	0.707971801	SLFN11	protein_coding
ENSG00000172717.16	52.59146825	-0.094404359	0.108912192	-0.866793305	0.386055271	0.610639947	FAM71D	protein_coding
ENSG00000172724.12	841.3684931	-0.438689068	0.224114705	-1.957430984	0.050296819	0.178769554	CCL19	protein_coding
ENSG00000172725.14	8465.384342	0.019417322	0.063442255	0.306062928	0.759556733	0.876869785	CORO1B	protein_coding
ENSG00000172728.16	669.7482433	0.055452044	0.069103103	0.802453751	0.422290531	0.641570076	FUT10	protein_coding
ENSG00000172731.14	1135.627108	-0.140027055	0.078449095	-1.784941631	0.074270785	0.228589959	LRRC20	protein_coding
ENSG00000172732.12	1601.796752	-0.048337562	0.043615752	-1.108259283	0.267749854	0.495676767	MUS81	protein_coding
ENSG00000172733.11	14.06030276	0.194184359	0.147669751	1.314990768	0.188513005	0.403835967	PURG	protein_coding
ENSG00000172738.12	43.64471815	0.08320697	0.102110699	0.814870245	0.415146587	0.635546911	TMEM217	protein_coding
ENSG00000172742.6	6.341648492	-0.092317549	0.289080389	-0.319349056	0.749461837	0.871278202	OR4D9	protein_coding
ENSG00000172746.6	13.06450087	-0.284625834	0.09986309	-2.850160478	0.004369718	0.033783564	RPL12P13	unprocessed_pseudogene
ENSG00000172748.14	168.9397564	-0.063438938	0.079549496	-0.79747756	0.42517373	0.644065766	ZNF596	protein_coding
ENSG00000172752.14	57.73858223	0.381464294	0.181396356	2.102932508	0.03547167	0.141951631	COL6A5	protein_coding
ENSG00000172757.13	58089.91464	-0.065045991	0.061960796	-1.049792688	0.293813438	0.52387513	CFL1	protein_coding
ENSG00000172765.17	2030.104157	0.16762761	0.044563204	3.761569943	0.00016885	0.002977736	TMCC1	protein_coding
ENSG00000172766.19	1324.769157	-0.118051516	0.06010413	-1.964116548	0.049516578	0.17675163	NAA16	protein_coding
ENSG00000172769.4	0.261556127	-0.017404707	0.937830386	-0.018558481	0.985193325		OR5B3	protein_coding
ENSG00000172771.12	60.00727975	-0.054055126	0.138102083	-0.39141427	0.695491046	0.839377774	EFCAB12	protein_coding
ENSG00000172772.3	2.958070181	-0.404863969	0.305832627	-1.323808952	0.185566502	0.400179714	OR10W1	protein_coding
ENSG00000172775.17	3208.166202	-0.086605507	0.038877864	-2.227630254	0.025905181	0.11565107	PSME3IP1	protein_coding
ENSG00000172780.17	238.9021045	0.057481477	0.091744499	0.626538675	0.530961678	0.728884735	RAB43	protein_coding
ENSG00000172782.12	78.60711858	0.281759302	0.27000699	1.043525956	0.296704766	0.526552782	FADS6	protein_coding
ENSG00000172785.18	724.0073883	-0.204735698	0.083076593	-2.464420964	0.013723483	0.07533739	CBWD1	protein_coding
ENSG00000172789.3	15.30626841	-0.360317652	0.175395068	-2.054320325	0.039944699	0.153833352	HOXC5	protein_coding
ENSG00000172794.20	279.8767222	-0.100369997	0.126035522	-0.796362763	0.425821215	0.644530623	RAB37	protein_coding
ENSG00000172795.17	2112.028626	0.008806983	0.055201785	0.159541632	0.873242161	0.938725432	DCP2	protein_coding
ENSG00000172799.5	9.471559804	-0.281436857	0.148654439	-1.893228739	0.058327457	0.196308473	ZBTB8OSP2	processed_pseudogene
ENSG00000172803.18	53.80513375	-0.307258645	0.102386178	-3.000977786	0.002691142	0.023691292	SNX32	protein_coding
ENSG00000172809.13	29487.37741	-0.168567873	0.073447132	-2.295091278	0.021727894	0.102738328	RPL38	protein_coding
ENSG00000172817.4	895.9917247	0.119064378	0.124294821	0.957919065	0.338103572	0.567926271	CYP7B1	protein_coding
ENSG00000172818.10	1003.040867	0.318847004	0.143719817	2.218531942	0.026518584	0.117462934	OVOL1	protein_coding
ENSG00000172819.17	2585.287989	0.236085263	0.097107382	2.43117731	0.015049848	0.080312294	RARG	protein_coding
ENSG00000172824.16	136.0491136	0.117621619	0.130511393	0.90123641	0.367462636	0.594546441	CES4A	protein_coding
ENSG00000172828.13	1122.949578	-0.361061236	0.169002621	-2.136423889	0.032644879	0.134744197	CES3	protein_coding
ENSG00000172830.13	3965.538564	0.257108258	0.071774805	3.582151964	0.000340775	0.005111866	SSH3	protein_coding
ENSG00000172831.14	9977.899762	-0.320721468	0.133327876	-2.405509479	0.016149933	0.084323703	CES2	protein_coding
ENSG00000172840.7	1017.845495	-0.027382074	0.05683942	-0.481744421	0.629987516	0.79893388	PDP2	protein_coding
ENSG00000172845.17	6166.461108	0.063469273	0.040203182	1.578712673	0.114401977	0.298435161	SP3	protein_coding
ENSG00000172867.4	9.255460722	-0.365351858	0.221630357	-1.648473897	0.099255463	0.274420431	KRT2	protein_coding
ENSG00000172869.14	2658.67746	-0.135218863	0.064289467	-2.103281751	0.035441148	0.141872028	DMXL1	protein_coding
ENSG00000172878.14	489.8184928	-0.13463754	0.065111908	-2.067786745	0.038660081	0.150750862	METAP1D	protein_coding
ENSG00000172888.12	1677.427898	0.134758639	0.052730623	2.555604914	0.01060034	0.062847757	ZNF621	protein_coding

ENSG00000172889.16	1689.586499	-0.10551428	0.105187567	-1.003106004	0.315809718	0.544944963	EGFL7	protein_coding
ENSG00000172890.13	3598.453701	0.043968981	0.051539826	0.853106896	0.393600027	0.617539563	NADSYN1	protein_coding
ENSG00000172893.16	5261.787043	0.166595836	0.097367005	1.711009156	0.087079423	0.252935806	DHCR7	protein_coding
ENSG00000172900.12	3.126496929	-0.045502241	0.248732801	-0.182936229	0.854848056	0.929168335	ACTE1P	transcribed_unitary_pseudogene
ENSG00000172901.20	28.56286746	0.406135726	0.171976223	2.361580684	0.018197212	0.091147114	LVRN	protein_coding
ENSG00000172912.4	3.560962569	-0.309166293	0.190669802	-1.621474874	0.104915834	0.283512174	COX6B1P3	processed_pseudogene
ENSG00000172915.18	717.8968854	-0.000652119	0.17232986	-0.003784133	0.996980706	0.998504884	NBEA	protein_coding
ENSG00000172922.11	1465.07155	-0.039921361	0.065755245	-0.607120555	0.54377091	0.73835352	RNASEH2C	protein_coding
ENSG00000172927.8	2716.836782	0.252412019	0.153999853	1.639040649	0.101204794	0.277460178	MYEOV	protein_coding
ENSG00000172932.16	2065.006572	0.07934468	0.061021832	1.300267099	0.19350944	0.410084175	ANKRD13D	protein_coding
ENSG00000172935.9	1218.424205	-0.094204952	0.181461584	-0.519145433	0.603659327	0.781186153	MRGPRF	protein_coding
ENSG00000172936.16	6422.594479	0.167306115	0.057371729	2.916176974	0.003543494	0.029055126	MYD88	protein_coding
ENSG00000172938.3	3.253070148	-0.296131146	0.266759	-1.11010742	0.26695274	0.49508779	MRGPRD	protein_coding
ENSG00000172939.9	3350.253797	0.215876539	0.057221477	3.772648851	0.000161524	0.002880835	OXSR1	protein_coding
ENSG00000172940.13	18.99858983	0.073291488	0.117925969	0.621504222	0.534267917	0.731326937	SLC22A13	protein_coding
ENSG00000172943.20	3139.006156	0.078675824	0.054653716	1.439532942	0.149999583	0.352146881	PHF8	protein_coding
ENSG00000172954.14	1868.899462	0.022884297	0.056301853	0.406457263	0.684406653	0.833403846	LCLAT1	protein_coding
ENSG00000172955.17	729.5029252	-0.767728008	0.19910354	-3.855923445	0.000115294	0.002203574	ADH6	protein_coding
ENSG00000172965.17	1073.439748	0.285955906	0.065936869	4.336813516	1.44563E-05	0.000433859	MIR4435-2HG	lncRNA
ENSG00000172967.7	2.719676384	-0.160463386	0.249432302	-0.64331438	0.520020127	0.720263082	XKR3	protein_coding
ENSG00000172969.7	5.3600115	0.74450672	0.221422905	3.362374456	0.000772753	0.009462657	FRG2C	protein_coding
ENSG00000172971.7	6.764302696	0.424440633	0.132137487	3.212113709	0.001317622	0.014126701	UNC93B3	unprocessed_pseudogene
ENSG00000172974.11	118.4014553	-0.15744384	0.093655701	-1.681091888	0.092745068	0.263002006	VDAC2P5	processed_pseudogene
ENSG00000172977.13	898.1713928	-0.034123353	0.049514751	-0.689155287	0.490725551	0.697968122	KAT5	protein_coding
ENSG00000172985.11	981.089582	0.213765177	0.105306083	2.029941383	0.042362498	0.159940268	SH3RF3	protein_coding
ENSG00000172986.13	594.3403273	0.597662442	0.141776668	4.215520438	2.49203E-05	0.000656164	GXYLT2	protein_coding
ENSG00000172987.13	62.81694867	0.037690152	0.208334145	0.180912026	0.856436629	0.930000971	HPSE2	protein_coding
ENSG00000172992.12	1599.299611	-0.027046921	0.054828089	-0.493304092	0.621797737	0.793233086	DCAKD	protein_coding
ENSG00000172995.16	4.856933819	-0.242286016	0.273895795	-0.884591953	0.376376748	0.60224967	ARPP21	protein_coding
ENSG00000173011.12	1960.803921	0.005399001	0.04533013	0.119104016	0.905192949	0.954924815	TADA2B	protein_coding
ENSG00000173013.6	68.21272229	0.217976399	0.082061521	2.656255901	0.007901363	0.051314113	CCDC96	protein_coding
ENSG00000173020.11	10812.97039	0.014121547	0.061146418	0.230946432	0.817356418	0.909961882	GRK2	protein_coding
ENSG00000173039.19	7153.504054	0.139377062	0.04053138	3.438744546	0.000584418	0.007647857	RELA	protein_coding
ENSG00000173040.13	181.9586139	0.274061435	0.120688584	2.270814905	0.023158185	0.10736932	EVC2	protein_coding
ENSG00000173041.12	684.8154041	-0.035834006	0.076636964	-0.467581226	0.640084093	0.80515942	ZNF680	protein_coding
ENSG00000173064.13	5213.032851	-0.025400222	0.057843847	-0.439117095	0.660576693	0.818067029	HECTD4	protein_coding
ENSG00000173065.13	4405.208311	0.07754424	0.050284558	1.54210843	0.123047247	0.312151655	FAM222B	protein_coding
ENSG00000173068.18	976.8960609	0.042815285	0.152830492	0.28014884	0.779363315	0.888395435	BNC2	protein_coding
ENSG00000173077.16	19.69782866	-0.168232991	0.137919615	-1.219790178	0.222544426	0.445037788	DELEC1	lncRNA
ENSG00000173080.6	37.75064361	0.161034257	0.169352888	0.950879897	0.341665348	0.571479947	RXFP4	protein_coding
ENSG00000173083.15	998.1137931	0.195598943	0.103394432	1.891774436	0.058521041	0.196651094	HPSE	protein_coding
ENSG00000173085.15	830.6052691	0.023250522	0.065629555	0.354269077	0.72313724	0.855433359	COQ2	protein_coding

ENSG00000173093.13	4.519321216	-0.123137472	0.28205407	-0.436573994	0.662420326	0.819180729	CCDC63	protein_coding
ENSG00000173110.8	344.3180021	0.893747691	0.152767591	5.850374984	4.90466E-09	6.78509E-07	HSPA6	protein_coding
ENSG00000173113.7	3769.845216	-0.159188798	0.064255285	-2.477442892	0.013232758	0.073485907	TRMT112	protein_coding
ENSG00000173114.13	63.08853048	-0.106282117	0.138936958	-0.764966488	0.4442915	0.660240441	LRRN3	protein_coding
ENSG00000173120.15	8744.689038	0.073192214	0.04826779	1.51637798	0.129423804	0.321801957	KDM2A	protein_coding
ENSG00000173124.14	10.26600499	-0.640516971	0.178111342	-3.596160489	0.000322949	0.004912088	ACSM6	protein_coding
ENSG00000173137.12	1058.750771	-0.287454752	0.078260806	-3.673035916	0.000239686	0.003906054	ADCK5	protein_coding
ENSG00000173141.5	3513.212347	-0.044349569	0.071350039	-0.621577359	0.534219813	0.731285931	MRPL57	protein_coding
ENSG00000173145.11	1326.993564	-0.102792673	0.057934179	-1.774301021	0.076013372	0.23223969	NOC3L	protein_coding
ENSG00000173153.17	2953.954551	0.013443814	0.074666199	0.180052212	0.857111579	0.930223931	ESRRA	protein_coding
ENSG00000173156.7	1925.763455	0.375773074	0.141498939	2.655660016	0.007915337	0.051381892	RHOD	protein_coding
ENSG00000173157.17	26.00124348	0.254983695	0.320658951	0.795186582	0.426504976	0.645161321	ADAMTS20	protein_coding
ENSG00000173163.10	936.3299135	-0.158860012	0.05719566	-2.777483666	0.005478159	0.039641606	COMMD1	protein_coding
ENSG00000173166.18	769.5142712	0.130093102	0.079855884	1.629098523	0.103292164	0.280863258	RAPH1	protein_coding
ENSG00000173171.14	1213.108696	0.077563287	0.065360964	1.186691284	0.235349414	0.46014992	MTX1	protein_coding
ENSG00000173175.15	1508.152156	-0.128424453	0.192466112	-0.667257478	0.504607682	0.708683772	ADCY5	protein_coding
ENSG00000173193.15	9210.604478	0.029403676	0.078108623	0.376445965	0.70658538	0.846312246	PARP14	protein_coding
ENSG00000173198.6	248.153829	-0.351721557	0.122428046	-2.872883851	0.004067436	0.032162342	CYSLTR1	protein_coding
ENSG00000173200.13	416.1597037	-0.767705403	0.150091434	-5.114918161	3.13877E-07	2.03539E-05	PARP15	protein_coding
ENSG00000173207.13	1999.319545	0.048218682	0.077934567	0.618707264	0.536109224	0.732627686	CKS1B	protein_coding
ENSG00000173208.4	96.63122206	-0.668281091	0.140895904	-4.743083893	2.10489E-06	9.36924E-05	ABCD2	protein_coding
ENSG00000173209.23	1536.691581	-0.069656275	0.079531573	-0.875831724	0.381121521	0.60657385	AHSA2P	transcribed_unitary_pseudogene
ENSG00000173210.20	1221.949917	0.577342715	0.122831306	4.70028963	2.59793E-06	0.000111467	ABLIM3	protein_coding
ENSG00000173212.4	28.96546367	0.273723083	0.206687901	1.324330456	0.18539332	0.399893113	MAB21L3	protein_coding
ENSG00000173213.9	8.433706992	-0.34838086	0.205814139	-1.692696438	0.090513264	0.259106615	TUBB8B	protein_coding
ENSG00000173214.7	1597.428675	-0.017183454	0.071736191	-0.239536747	0.810689406	0.90629585	MFSD4B	protein_coding
ENSG00000173218.15	2715.484346	0.144846335	0.058496071	2.47617205	0.013279957	0.073654151	VANGL1	protein_coding
ENSG00000173221.14	2037.404593	0.133565635	0.089806285	1.487263785	0.136945155	0.333090799	GLRX	protein_coding
ENSG00000173226.17	1213.759503	-0.175110627	0.057310446	-3.055474886	0.002247044	0.020816209	IQCB1	protein_coding
ENSG00000173227.14	269.7161007	0.985299756	0.200014192	4.926149223	8.3866E-07	4.55122E-05	SYT12	protein_coding
ENSG00000173230.15	10871.42957	0.097701319	0.058196458	1.67881898	0.093187328	0.26379266	GOLGB1	protein_coding
ENSG00000173231.6	0.558309695	-0.106633941	0.235702696	-0.452408659	0.65097461		TERF1P1	processed_pseudogene
ENSG00000173237.4	255.3378072	-0.21617791	0.256066096	-0.844226991	0.398542574	0.621623424	C11orf86	protein_coding
ENSG00000173239.14	3.018230976	0.1742408	0.247722945	0.703369645	0.481825419	0.690629668	LIPM	protein_coding
ENSG00000173250.2	2.898135971	-0.05036576	0.276079736	-0.182431934	0.855243767	0.929364508	GPR151	protein_coding
ENSG00000173253.15	20.05115691	0.409017672	0.258906232	1.579790755	0.114154794	0.298089851	DMRT2	protein_coding
ENSG00000173258.13	526.8022307	-0.014517772	0.119921984	-0.121060136	0.903643404	0.954134104	ZNF483	protein_coding
ENSG00000173261.9	30.42158693	-0.154412028	0.104876927	-1.472316484	0.140935434	0.33882508	PLAC8L1	protein_coding
ENSG00000173262.12	33.75923664	-0.141012606	0.205713395	-0.685480914	0.4930405	0.700049718	SLC2A14	protein_coding
ENSG00000173264.15	1899.930513	0.060978973	0.061760856	0.987340148	0.323475915	0.553244238	GPR137	protein_coding
ENSG00000173267.14	325.0916405	0.464203291	0.164604407	2.820114597	0.00480065	0.036193414	SNCG	protein_coding
ENSG00000173269.14	1964.584587	0.013230347	0.097300888	0.135973549	0.891842192	0.948452928	MMRN2	protein_coding

ENSG00000173272.16	2326.414874	-0.151689872	0.074455506	-2.03732242	0.041617742	0.158002493	MZT2A	protein_coding
ENSG00000173273.16	3776.903895	0.070711506	0.080350179	0.880041671	0.378836736	0.604576592	TNKS	protein_coding
ENSG00000173275.13	452.1199562	-0.027218489	0.058217719	-0.467529295	0.640121238	0.805177323	ZNF449	protein_coding
ENSG00000173276.14	1557.64986	0.115691415	0.051942932	2.227279263	0.025928616	0.115684579	ZBTB21	protein_coding
ENSG00000173281.5	2636.576482	0.08109823	0.085210561	0.951739195	0.341229266	0.571239279	PPP1R3B	protein_coding
ENSG00000173285.4	2.634295962	-0.343062552	0.363833953	-0.942909668	0.345727109	0.575237963	OR10K1	protein_coding
ENSG00000173295.7	166.8785366	-0.179622385	0.105384992	-1.7044399	0.088298935	0.25524396	FAM86B3P	transcribed_unprocessed_pseudogene
ENSG00000173302.5	1.837619434	-0.499762137	0.343358688	-1.455510389	0.145528016	0.345859204	GPR148	protein_coding
ENSG00000173320.12	434.4992989	0.259235467	0.137394466	1.886797004	0.059187634	0.198142394	STOX2	protein_coding
ENSG00000173327.8	7774.331495	0.008579283	0.057867905	0.148256327	0.882140481	0.943441809	MAP3K11	protein_coding
ENSG00000173334.4	7781.398241	0.032823762	0.087398146	0.375565888	0.707239655	0.846703786	TRIB1	protein_coding
ENSG00000173335.5	1.128001771	0.52211743	0.388804808	1.34287802	0.179311475	0.392183103	CST9	protein_coding
ENSG00000173338.13	48.74293827	-0.068137946	0.132067011	-0.515934638	0.605900061	0.782714462	KCNK7	protein_coding
ENSG00000173349.6	0						SFT2D3	protein_coding
ENSG00000173357.12	0.218408672	0.275702101	0.727422117	0.379012535	0.704678555		AC007967.1	transcribed_unprocessed_pseudogene
ENSG00000173366.11	0						AC097637.1	protein_coding
ENSG00000173369.17	13219.4306	-0.092141883	0.135710757	-0.678957842	0.497164574	0.703311503	C1QB	protein_coding
ENSG00000173372.17	7446.304036	-0.098999019	0.130610101	-0.75797368	0.448466739	0.663571189	C1QA	protein_coding
ENSG00000173376.14	420.0402073	0.859458016	0.180000138	4.774763102	1.79919E-06	8.227E-05	NDNF	protein_coding
ENSG00000173389.16	2.550728462	0.558774139	0.249679996	2.23796118	0.025223587	0.113773754	IQCF1	protein_coding
ENSG00000173391.9	616.6581413	0.329236713	0.146265856	2.250947161	0.024388884	0.111089621	OLR1	protein_coding
ENSG00000173401.10	17.60349541	-0.009903458	0.118083979	-0.083867924	0.933161443	0.968383887	GLIPR1L1	protein_coding
ENSG00000173402.12	18420.81252	0.035067116	0.070111162	0.500164523	0.616959237	0.789907295	DAG1	protein_coding
ENSG00000173404.5	309.6144577	-0.612767963	0.241970043	-2.532412503	0.011328064	0.065915748	INSM1	protein_coding
ENSG00000173406.16	113.9650285	-0.61457214	0.190466672	-3.226664972	0.00125242	0.013617977	DAB1	protein_coding
ENSG00000173409.14	1210.50241	-0.119414119	0.063891737	-1.869007239	0.061621807	0.203200416	ARV1	protein_coding
ENSG00000173418.12	3224.698277	-0.214954663	0.059119508	-3.635934564	0.000276975	0.004371504	NAA20	protein_coding
ENSG00000173421.17	46.98573264	0.038297977	0.120171786	0.318693586	0.749958879	0.871631677	IHO1	protein_coding
ENSG00000173431.2	0.567146044	-0.119682247	0.312816146	-0.38259613	0.70201924		RNASE8	protein_coding
ENSG00000173432.12	2017.306343	0.285782148	0.246349252	1.160069073	0.246020685	0.47251282	SAA1	protein_coding
ENSG00000173436.15	809.9286915	-0.150718054	0.072587123	-2.076374545	0.037859324	0.148496467	MICOS10	protein_coding
ENSG00000173442.13	6641.711692	0.058090032	0.07051711	0.823772156	0.410069031	0.630942264	EHBP1L1	protein_coding
ENSG00000173451.7	292.0508229	-0.109867458	0.056263793	-1.952720422	0.050852732	0.179879241	THAP2	protein_coding
ENSG00000173452.14	3.151829213	-0.407756821	0.284914607	-1.431154498	0.152385941	0.355449091	TMEM196	protein_coding
ENSG00000173456.5	2407.414426	0.039398071	0.060965996	0.646230252	0.518130251	0.71911254	RNF26	protein_coding
ENSG00000173457.11	4916.618734	0.164485585	0.079999207	2.056090189	0.039773824	0.153532104	PPP1R14B	protein_coding
ENSG00000173464.14	0.151997989	-0.232253692	0.936400901	-0.248028052	0.804112702		RNASE11	protein_coding
ENSG00000173465.8	1242.089339	0.007949379	0.06357094	0.125047373	0.900486046	0.952509344	ZNRD2	protein_coding
ENSG00000173467.9	3799.62208	-0.362629593	0.184717199	-1.96316096	0.049627474	0.177074956	AGR3	protein_coding
ENSG00000173473.11	8839.745139	0.047319501	0.054393287	0.869951122	0.384327116	0.609211114	SMARCC1	protein_coding
ENSG00000173480.11	597.3308519	0.013083359	0.068368395	0.1913656	0.848239169	0.92562023	ZNF417	protein_coding
ENSG00000173482.17	2369.301243	0.182909164	0.096608767	1.893297775	0.058318281	0.196308473	PTPRM	protein_coding

ENSG00000173486.14	14.25849043	0.159038661	0.103250776	1.54031444	0.123483726	0.312706317	FKBP2	protein_coding
ENSG00000173511.10	4046.251586	0.072937763	0.073630862	0.990586844	0.321887364	0.551701529	VEGFB	protein_coding
ENSG00000173517.10	4946.90308	0.0797125	0.067232656	1.185621757	0.235771706	0.4606862	PEAK1	protein_coding
ENSG00000173530.6	836.4790276	0.170756829	0.105914355	1.612216108	0.106914921	0.286427611	TNFRSF10D	protein_coding
ENSG00000173531.15	327.9424726	0.015778403	0.129399132	0.121935926	0.902949763	0.953794585	MST1	protein_coding
ENSG00000173535.15	131.6910417	0.576729465	0.152601524	3.77931654	0.000157259	0.002830168	TNFRSF10C	protein_coding
ENSG00000173540.14	1338.314917	-0.055427219	0.066941335	-0.827996926	0.407672241	0.628720741	GMPPB	protein_coding
ENSG00000173542.9	1758.335442	0.086496953	0.061053942	1.41673002	0.156561877	0.361402921	MOB1B	protein_coding
ENSG00000173545.5	1526.259875	0.010328433	0.057675286	0.179079004	0.857875668	0.930682334	ZNF622	protein_coding
ENSG00000173546.7	3747.981345	-0.144503534	0.138026611	-1.046925172	0.295134091	0.525045757	CSPG4	protein_coding
ENSG00000173548.9	3126.253861	0.241843793	0.058218363	4.154080941	3.26598E-05	0.000816531	SNX33	protein_coding
ENSG00000173557.15	248.1011083	0.151582144	0.160863749	0.942301446	0.346038328	0.575414494	FAM166C	protein_coding
ENSG00000173559.14	1268.306895	-0.115984352	0.080598528	-1.4390381	0.150139728	0.352323045	NABP1	protein_coding
ENSG00000173567.15	48.36549069	-0.086112788	0.096134591	-0.895752371	0.370385033	0.596936056	ADGRF3	protein_coding
ENSG00000173572.11	5.853397268	-0.031720957	0.382565734	-0.082916357	0.933918048	0.968817523	NLRP13	protein_coding
ENSG00000173575.22	5616.475352	0.000681145	0.045699319	0.014904932	0.988108025	0.994383248	CHD2	protein_coding
ENSG00000173578.8	145.943573	-0.544903373	0.173364683	-3.143104838	0.00167166	0.016815768	XCR1	protein_coding
ENSG00000173581.8	669.9459984	-0.177966641	0.119193794	-1.493086462	0.135414556	0.33092404	CCDC106	protein_coding
ENSG00000173585.16	21.34571412	0.04546399	0.162984855	0.278946106	0.780286185	0.88874039	CCR9	protein_coding
ENSG00000173588.15	660.1266785	-0.143961707	0.059171299	-2.432965118	0.014975744	0.080004656	CEP83	protein_coding
ENSG00000173597.9	2707.18762	-0.348888188	0.187582832	-1.859915351	0.062897503	0.205864841	SULT1B1	protein_coding
ENSG00000173598.14	4525.590001	-0.125801553	0.073879462	-1.702794667	0.088606501	0.255861474	NUDT4	protein_coding
ENSG00000173599.15	1147.474632	0.254441715	0.099767604	2.550344042	0.010761666	0.06357071	PC	protein_coding
ENSG00000173610.12	2.93600121	0.629505871	0.29037218	2.167927627	0.030164193	0.128072975	UGT2A1	protein_coding
ENSG00000173611.18	908.3996502	-0.077617557	0.055646958	-1.394821192	0.163069767	0.37013549	SCAI	protein_coding
ENSG00000173612.10	4.486156083	-0.693171279	0.378163451	-1.832993847	0.066803486	0.213528023	GPRC6A	protein_coding
ENSG00000173614.14	561.3107372	-0.118001323	0.057425641	-2.054854263	0.039893083	0.153721259	NMNAT1	protein_coding
ENSG00000173621.9	2022.673736	0.031013058	0.102199414	0.303456323	0.761542128	0.878045279	LRFN4	protein_coding
ENSG00000173626.10	8.247193636	-0.186785043	0.18044933	-1.035110762	0.300617187	0.530256837	TRAPPC3L	protein_coding
ENSG00000173627.8	14.04266668	-0.454885343	0.17822641	-2.552289216	0.010701764	0.063302671	APOBEC4	protein_coding
ENSG00000173638.19	1562.096856	-0.106826964	0.081993371	-1.302873186	0.19261806	0.408871216	SLC19A1	protein_coding
ENSG00000173641.18	2384.423216	-0.416895777	0.232801351	-1.790779028	0.073328762	0.226518444	HSPB7	protein_coding
ENSG00000173653.8	1486.034835	0.013541211	0.064964666	0.208439637	0.83488571	0.919104047	RCE1	protein_coding
ENSG00000173660.12	7595.382726	-0.123336346	0.063244765	-1.950143157	0.051159059	0.180536331	UQCRH	protein_coding
ENSG00000173662.21	12.79480748	-0.267604044	0.16860406	-1.587174372	0.112473157	0.295486995	TAS1R1	protein_coding
ENSG00000173673.8	0.688484345	-0.356137789	0.482656861	-0.737869526	0.460593739		HES3	protein_coding
ENSG00000173674.11	6020.306336	-0.134359917	0.062866647	-2.137220986	0.032580022	0.134572475	EIF1AX	protein_coding
ENSG00000173678.14	9.129554684	-0.17583053	0.169450538	-1.037651053	0.299432541	0.529291628	SPDYE2B	protein_coding
ENSG00000173679.2	0.652118792	0.075706556	0.479645173	0.15783867	0.874583926		OR1L1	protein_coding
ENSG00000173681.16	842.9127815	-0.143356268	0.059542836	-2.40761573	0.016057072	0.083969643	BCLAF3	protein_coding
ENSG00000173692.14	10947.95191	-0.116238581	0.054437746	-2.135257046	0.032740019	0.135003012	PSMD1	protein_coding
ENSG00000173698.18	183.4000872	0.299948907	0.156303329	1.919018037	0.054982051	0.189138055	ADGRG2	protein_coding

ENSG00000173699.16	4.62656184	-0.0358358	0.267663896	-0.133883577	0.893494637	0.949245446	SPATA3	protein_coding
ENSG00000173702.7	33648.82429	0.315266827	0.149454073	2.109456233	0.03490522	0.140462164	MUC13	protein_coding
ENSG00000173705.9	132.1234769	0.256464945	0.159453782	1.608396749	0.107748313	0.287669908	SUSD5	protein_coding
ENSG00000173706.14	5911.392008	0.258712777	0.096324215	2.685853991	0.00723447	0.048160171	HEG1	protein_coding
ENSG00000173714.8	8.869878769	0.110301818	0.204119975	0.540377384	0.588936801	0.770555854	WFIKKN2	protein_coding
ENSG00000173715.18	823.3702982	-0.043966878	0.081407301	-0.540085196	0.589138279	0.770642095	C11orf80	protein_coding
ENSG00000173726.11	12097.60551	0.093535821	0.06348775	1.473289265	0.140673054	0.338511772	TOMM20	protein_coding
ENSG00000173727.12	158.4329766	0.201533343	0.120029569	1.679030797	0.093146042	0.263755707	AP000769.1	transcribed_unprocessed_pseudogene
ENSG00000173728.11	7.445486623	-0.284033368	0.139896449	-2.030311489	0.042324887	0.159861967	C1orf100	protein_coding
ENSG00000173744.18	6520.582698	-0.00352361	0.049710281	-0.070882916	0.943490941	0.973443884	AGFG1	protein_coding
ENSG00000173757.10	4086.317265	-0.06353314	0.059011451	-1.076623928	0.28164831	0.510540283	STAT5B	protein_coding
ENSG00000173762.8	1319.005915	-0.257316471	0.139384983	-1.846084607	0.064879922	0.20970719	CD7	protein_coding
ENSG00000173769.4	4.857134189	-0.159984509	0.348327694	-0.459293108	0.646023697	0.808900541	TOPAZ1	protein_coding
ENSG00000173786.17	4716.915911	-0.055384715	0.061389826	-0.902180672	0.3669609	0.5939993	CNP	protein_coding
ENSG00000173801.17	71498.75238	-0.065639744	0.130452558	-0.503169466	0.61484513	0.788602281	JUP	protein_coding
ENSG00000173805.16	62.46824436	0.125887607	0.172128019	0.731360345	0.46455908	0.676537247	HAP1	protein_coding
ENSG00000173809.18	36.61483452	-0.877874624	0.192789081	-4.55354949	5.27483E-06	0.000195221	TDRD12	protein_coding
ENSG00000173810.9	3.891517543	-0.221270958	0.13768348	-1.607098825	0.108032691	0.288076158	PPIAP7	processed_pseudogene
ENSG00000173811.11	6.614010166	-0.243288855	0.13085743	-1.859190225	0.06300018	0.206071654	CCDC13-AS1	lncRNA
ENSG00000173812.11	30883.86198	-0.177178603	0.071719191	-2.470448981	0.013494357	0.074519058	EIF1	protein_coding
ENSG00000173818.17	546.164234	0.025437806	0.062570598	0.406545668	0.684341709	0.83340018	ENDOV	protein_coding
ENSG00000173821.19	26305.41101	0.10567061	0.083562882	1.264563977	0.206027673	0.425803553	RNF213	protein_coding
ENSG00000173825.7	52.39494218	0.152599123	0.104577263	1.459199822	0.144510118	0.344266204	TIGD3	protein_coding
ENSG00000173826.15	47.06067598	-0.028841594	0.215438533	-0.133873887	0.8935023	0.949245446	KCNH6	protein_coding
ENSG00000173838.12	10.97146497	0.368895314	0.218992894	1.68450815	0.092083506	0.261756082	MARCHF10	protein_coding
ENSG00000173846.13	1502.93014	0.384802063	0.071212447	5.403578715	6.53243E-08	5.85728E-06	PLK3	protein_coding
ENSG00000173848.19	10708.42419	0.056023669	0.07374966	0.75964648	0.447465928	0.662772263	NET1	protein_coding
ENSG00000173852.15	3727.469949	0.030528594	0.077205282	0.395421052	0.692532175	0.837699816	DPY19L1	protein_coding
ENSG00000173862.3	2.23267776	-0.160062837	0.198835698	-0.805000503	0.420819389	0.640515258	AC008080.1	lncRNA
ENSG00000173867.10	0.569079344	-0.020687691	0.310995432	-0.066520885	0.946963131		AC013489.1	protein_coding
ENSG00000173868.12	72.70050845	-0.080486978	0.111642482	-0.720935046	0.470949479	0.682084733	PHOSPHO1	protein_coding
ENSG00000173875.14	1912.744974	-0.028257637	0.046279585	-0.610585354	0.541474121	0.736632672	ZNF791	protein_coding
ENSG00000173889.16	5365.456694	-0.005343093	0.052540789	-0.101694193	0.918999412	0.961746298	PHC3	protein_coding
ENSG00000173890.17	4029.547509	-0.320510721	0.106825061	-3.000332663	0.002696849	0.023709314	GPR160	protein_coding
ENSG00000173894.11	900.5958847	0.105918873	0.150961507	0.701628351	0.482910966	0.691139358	CBX2	protein_coding
ENSG00000173898.14	3709.169128	0.395806318	0.144456473	2.739969411	0.00614449	0.042972849	SPTBN2	protein_coding
ENSG00000173905.9	6458.071476	-0.04520018	0.077931258	-0.580000648	0.561914181	0.750933347	GOLIM4	protein_coding
ENSG00000173908.9	1.395635692	-0.246852649	0.443755461	-0.556280813	0.578018901	0.762358646	KRT28	protein_coding
ENSG00000173914.12	796.9320765	0.042167524	0.041966437	1.004791605	0.314997207	0.54421097	RBM4B	protein_coding
ENSG00000173915.16	4286.787275	-0.214708812	0.066801426	-3.214135152	0.001308381	0.014059825	ATP5MD	protein_coding
ENSG00000173917.10	697.8369713	0.260165328	0.127457083	2.041199456	0.041231006	0.156960211	HOXB2	protein_coding
ENSG00000173918.15	2971.764739	0.431805105	0.115770348	3.729841984	0.0001916	0.003285851	C1QTNF1	protein_coding

ENSG00000173926.6	809.1551389	0.038991094	0.09249515	0.421547448	0.673355371	0.82634303	MARCHF3	protein_coding
ENSG00000173928.4	126.9042766	0.009537449	0.062311102	0.153061792	0.878349546	0.941316258	SWSAP1	protein_coding
ENSG00000173930.9	33.9234566	0.420764816	0.181829417	2.314063496	0.020664239	0.09934748	SLCO4C1	protein_coding
ENSG00000173933.21	630.4699556	-0.053595487	0.064509981	-0.830809208	0.40608142	0.6275418	RBM4	protein_coding
ENSG00000173947.14	206.2351369	-0.011858396	0.186054245	-0.063736231	0.949180255	0.976210607	PIFO	protein_coding
ENSG00000173950.16	1406.255709	0.141503444	0.060002053	2.358310044	0.018358353	0.091701277	XXYLT1	protein_coding
ENSG00000173954.8	0.27486246	-0.419807837	0.528636937	-0.794132623	0.427118228		SNURFL	processed_pseudogene
ENSG00000173960.14	1450.774325	-0.003698846	0.044255726	-0.083578935	0.933391216	0.968507013	UBXN2A	protein_coding
ENSG00000173966.7	1.888300325	0.085444004	0.171091929	0.499404061	0.617494759	0.79029958	AC095040.1	processed_pseudogene
ENSG00000173976.16	1.373209036	0.135529396	0.236644771	0.572712404	0.566839437	0.754652569	RAX2	protein_coding
ENSG00000173988.13	13.16921327	-0.135961984	0.151387437	-0.898106121	0.369128976	0.595953605	LRRC63	protein_coding
ENSG00000173991.6	25.00300842	-0.266501904	0.15596978	-1.708676547	0.087510881	0.253909158	TCAP	protein_coding
ENSG00000173992.9	1377.27993	-0.135513696	0.049796511	-2.721349224	0.006501604	0.044692354	CCS	protein_coding
ENSG00000174004.6	372.7252035	-0.09719334	0.099191965	-0.979850935	0.327159705	0.556725964	NRROS	protein_coding
ENSG00000174007.9	117.0901267	0.025445722	0.076232535	0.333790846	0.738537392	0.864730329	CEP19	protein_coding
ENSG00000174010.9	904.6138885	-0.034648991	0.068363561	-0.506834206	0.612271154	0.787117541	KLHL15	protein_coding
ENSG00000174013.8	1631.104729	0.099668905	0.058707696	1.697714455	0.089561668	0.257452734	FBXO45	protein_coding
ENSG00000174015.10	23.76081307	0.260435706	0.271307502	0.95992814	0.337091382	0.566809352	CBY2	protein_coding
ENSG00000174016.11	6.296854727	-0.060396862	0.280168915	-0.215573029	0.829320581	0.9161251	TENT5D	protein_coding
ENSG00000174021.11	708.6611314	-0.083605616	0.090982464	-0.918920116	0.358137357	0.585787539	GNG5	protein_coding
ENSG00000174028.6	219.9429044	0.136407769	0.083162313	1.640259442	0.100951235	0.277012621	FAM3C2P	processed_pseudogene
ENSG00000174032.17	641.2738659	-0.102322977	0.06017264	-1.700490071	0.089038782	0.256548732	SLC25A30	protein_coding
ENSG00000174038.13	12.32696318	-0.13180134	0.14448456	-0.912217471	0.361654249	0.589394038	C9orf131	protein_coding
ENSG00000174059.17	3993.59811	0.008098246	0.089260243	0.09072624	0.92771012	0.965785707	CD34	protein_coding
ENSG00000174080.12	1396.492303	0.28301513	0.126825748	2.231527383	0.025646216	0.114883341	CTSF	protein_coding
ENSG00000174099.12	2765.59176	-0.116773197	0.158893645	-0.734914206	0.462391746	0.674881186	MSRB3	protein_coding
ENSG00000174106.3	2254.208035	0.219982752	0.066617649	3.302169244	0.000959402	0.011102413	LEMD3	protein_coding
ENSG00000174109.5	601.5347048	-0.076559437	0.06243055	-1.22631368	0.220080655	0.441997176	C16orf91	protein_coding
ENSG00000174123.11	278.3815018	-1.467349588	0.194077547	-7.560635461	4.01105E-14	3.06648E-11	TLR10	protein_coding
ENSG00000174125.8	487.126507	-0.156236635	0.118577836	-1.317587171	0.187641883	0.402816481	TLR1	protein_coding
ENSG00000174130.12	266.177867	-0.242869366	0.120275529	-2.01927498	0.043458647	0.162517428	TLR6	protein_coding
ENSG00000174132.9	588.1145005	0.137609437	0.072802321	1.890179249	0.058733991	0.197169026	FAM174A	protein_coding
ENSG00000174136.13	1099.444175	-0.07752664	0.103545415	-0.748721135	0.454025302	0.668391628	RGMB	protein_coding
ENSG00000174137.13	82.1613424	-0.28810891	0.098755465	-2.917397131	0.003529661	0.028999249	FAM53A	protein_coding
ENSG00000174145.8	42.85020339	0.61538923	0.196531113	3.131256011	0.001740604	0.017337207	NWD2	protein_coding
ENSG00000174151.15	838.969545	-0.060914527	0.068616569	-0.88775245	0.374673926	0.600417963	CYB561D1	protein_coding
ENSG00000174156.15	3.326545442	-0.753208594	0.27093713	-2.780012452	0.005435681	0.039445577	GSTA3	protein_coding
ENSG00000174165.8	1723.80839	-0.01684643	0.05370503	-0.313684403	0.753760748	0.873513322	ZDHHC24	protein_coding
ENSG00000174171.6	34.24297188	-0.13347832	0.179179523	-0.744941823	0.45630689	0.670286062	AC020659.1	lncRNA
ENSG00000174173.7	1380.854096	0.017613163	0.057670463	0.305410464	0.760053553	0.877118024	TRMT10C	protein_coding
ENSG00000174175.17	705.5252127	-0.113642637	0.161527507	-0.703549749	0.481713215	0.690581859	SELP	protein_coding
ENSG00000174177.13	722.1302461	-0.005086575	0.066337317	-0.076677434	0.938880157	0.970988717	CTU2	protein_coding

ENSG00000174197.16	3197.963847	0.100291437	0.063040223	1.590911835	0.111629419	0.29399705	MGA	protein_coding
ENSG00000174206.13	753.4193702	-0.004661222	0.069033838	-0.067520828	0.946167081	0.97474225	C12orf66	protein_coding
ENSG00000174225.15	22.19420024	-0.062904885	0.095389329	-0.659454107	0.509604207	0.71290602	ARL13A	protein_coding
ENSG00000174226.9	19.14600289	-0.089116068	0.185970396	-0.479194913	0.631799973	0.800107752	SNX31	protein_coding
ENSG00000174227.16	2212.497378	0.048635306	0.053428269	0.91029163	0.362668732	0.590080831	PIGG	protein_coding
ENSG00000174231.17	24791.03843	0.108735719	0.05366817	2.026074639	0.042757137	0.160914392	PRPF8	protein_coding
ENSG00000174233.11	2708.200478	0.136518856	0.067863554	2.011666759	0.044255077	0.164504784	ADCY6	protein_coding
ENSG00000174236.4	31.36815807	-0.388126822	0.201452748	-1.926639504	0.054024572	0.187021669	REP15	protein_coding
ENSG00000174238.15	5231.465157	0.096722255	0.045431164	2.128984723	0.033255524	0.136239164	PITPNA	protein_coding
ENSG00000174243.10	6921.571149	0.04799102	0.044780683	1.071690206	0.283859201	0.512819356	DDX23	protein_coding
ENSG00000174255.8	14.41187039	-0.564715746	0.177845277	-3.175320453	0.001496711	0.015501089	ZNF80	protein_coding
ENSG00000174276.7	381.6732749	0.002508996	0.070030583	0.035827153	0.971420182	0.986458933	ZNHIT2	protein_coding
ENSG00000174279.4	3.452331223	0.260863443	0.290539516	0.897858738	0.369260865	0.596083746	EVX2	protein_coding
ENSG00000174282.12	6054.779542	0.002467735	0.074387821	0.033173913	0.973535901	0.98764157	ZBTB4	protein_coding
ENSG00000174292.12	1137.368319	0.022292882	0.074159497	0.30060724	0.76371401	0.879571991	TNK1	protein_coding
ENSG00000174306.21	2325.673609	-0.067890962	0.070166625	-0.967567732	0.333260299	0.562819676	ZHX3	protein_coding
ENSG00000174307.7	2483.341175	0.346456643	0.115561976	2.998015917	0.002717435	0.023836082	PHLDA3	protein_coding
ENSG00000174325.6	5.809325778	0.381506089	0.228436473	1.6700752	0.094904486	0.266420975	DIRC1	lncRNA
ENSG00000174326.14	59.56629915	-0.257440691	0.143805994	-1.79019444	0.073422659	0.226724716	SLC16A11	protein_coding
ENSG00000174327.7	487.5854657	-0.011576403	0.089196232	-0.129785786	0.896735909	0.951012373	SLC16A13	protein_coding
ENSG00000174332.5	93.59847839	0.487027544	0.130471669	3.732822209	0.000189346	0.003268201	GLIS1	protein_coding
ENSG00000174339.2	0.784008236	-0.370544759	0.468283629	-0.791282751	0.428779012		OR2Y1	protein_coding
ENSG00000174343.6	5.137026621	0.102377722	0.203785421	0.502380011	0.615400237	0.789009241	CHRNA9	protein_coding
ENSG00000174348.14	3395.071308	0.316109698	0.151750074	2.083094192	0.037242639	0.146791106	PODN	protein_coding
ENSG00000174353.17	124.9442864	-0.09779907	0.074050956	-1.320699619	0.186601541	0.401517646	STAG3L3	transcribed_unprocessed_pseudogene
ENSG00000174358.16	1941.549847	0.412655478	0.272751803	1.512934005	0.130296423	0.323103039	SLC6A19	protein_coding
ENSG00000174365.20	576.2333176	-0.16905013	0.068929622	-2.452503372	0.014186605	0.077096027	SNHG11	lncRNA
ENSG00000174370.11	133.3502252	-0.038386436	0.11397807	-0.336787912	0.736276786	0.86325863	C11orf45	protein_coding
ENSG00000174371.17	838.0437337	0.142214144	0.092527924	1.536986215	0.124296685	0.313991643	EXO1	protein_coding
ENSG00000174373.16	1860.342997	0.061418572	0.053645524	1.144896483	0.252252041	0.478950251	RALGAPA1	protein_coding
ENSG00000174384.9	2.300646055	-0.346823631	0.233473795	-1.485492757	0.137413341	0.333819888	PMS2P6	unprocessed_pseudogene
ENSG00000174403.16	56.63767775	-0.652161742	0.236140705	-2.761750634	0.005749237	0.0410847	MIR1-1HG-AS1	lncRNA
ENSG00000174405.14	1010.324944	0.170098229	0.061628166	2.760072854	0.005778847	0.041248948	LIG4	protein_coding
ENSG00000174407.13	2.923880244	-0.683446288	0.313533926	-2.179816063	0.029271099	0.125412568	MIR1-1HG	lncRNA
ENSG00000174408.8	0.905524424	0.053743447	0.271645741	0.197843879	0.843167214		PPIAP24	processed_pseudogene
ENSG00000174417.3	4.360065419	-0.064522044	0.193474641	-0.333490959	0.738763713	0.864824884	TRHR	protein_coding
ENSG00000174418.7	4.553741261	-0.344637686	0.158284794	-2.177326559	0.029456211	0.12602978	RPL35P9	processed_pseudogene
ENSG00000174428.18	353.5478914	-0.111885194	0.070572781	-1.58538735	0.112878354	0.296194377	GTF2IRD2B	protein_coding
ENSG00000174429.4	12.48071111	0.119359366	0.174131562	0.685455089	0.493056792	0.70005004	ABRA	protein_coding
ENSG00000174437.17	28084.19437	0.032354041	0.04954119	0.653073542	0.513708869	0.715890354	ATP2A2	protein_coding
ENSG00000174442.12	1456.585609	0.041942721	0.063617811	0.659292116	0.509708204	0.71298284	ZWILCH	protein_coding
ENSG00000174444.15	76769.72046	-0.117717668	0.057113436	-2.061120397	0.039291555	0.152263949	RPL4	protein_coding

ENSG00000174446.13	931.1647227	-0.113736067	0.051318049	-2.216297556	0.02667113	0.117958779	SNAPC5	protein_coding
ENSG00000174448.8	2.672441235	-0.321138064	0.211427028	-1.518907336	0.128785822	0.320818445	STARD6	protein_coding
ENSG00000174450.12	8.077599845	0.048520815	0.242412259	0.200158254	0.841356815	0.92200346	GOLGA6L2	protein_coding
ENSG00000174453.10	3.318536905	-0.322956377	0.272618168	-1.184647302	0.236156926	0.461108091	VWC2L	protein_coding
ENSG00000174456.15	279.9771379	-0.084188533	0.052045894	-1.617582611	0.105752579	0.28473202	C12orf76	protein_coding
ENSG00000174460.4	27.37480217	0.277109831	0.173375241	1.598324128	0.10997086	0.29108912	ZCCHC12	protein_coding
ENSG00000174469.23	1297.03087	0.101020633	0.195988877	0.515440645	0.606245136	0.782881587	CNTNAP2	protein_coding
ENSG00000174473.16	54.66770998	0.611313146	0.220364116	2.77410477	0.005535385	0.039969422	GALNTL6	protein_coding
ENSG00000174482.10	13.95795335	0.199183407	0.220935344	0.901546142	0.367298012	0.594375432	LINGO2	protein_coding
ENSG00000174483.20	49.93717996	-0.040165412	0.082764273	-0.485298924	0.627464319	0.797051811	BBS1	protein_coding
ENSG00000174485.16	2421.500974	-0.036575028	0.0534884	-0.683793649	0.494105478	0.700959834	DENND4A	protein_coding
ENSG00000174495.12	2.751767699	-0.230485133	0.169739944	-1.357872096	0.174504273	0.385832498	PPIAP80	processed_pseudogene
ENSG00000174498.14	27.44996546	-0.107972514	0.214771226	-0.502732679	0.61515223	0.78882036	IGDCC3	protein_coding
ENSG00000174500.13	206.8023524	-0.41502083	0.112741233	-3.681180501	0.000232157	0.003815152	GCSAM	protein_coding
ENSG00000174501.14	626.640294	-0.144563077	0.10964781	-1.318431047	0.187359393	0.40235607	ANKRD36C	protein_coding
ENSG00000174502.19	1377.752524	-0.061801226	0.250950859	-0.246268239	0.805474594	0.903363893	SLC26A9	protein_coding
ENSG00000174514.13	1314.124034	-0.138563743	0.181434253	-0.763713251	0.445038142	0.660987134	MFSD4A	protein_coding
ENSG00000174516.15	556.3405993	0.036935115	0.081038591	0.455771933	0.648553989	0.810325474	PELI3	protein_coding
ENSG00000174521.7	19.41006114	-0.114449934	0.11514754	-0.993941635	0.320251283	0.549931905	TTC9B	protein_coding
ENSG00000174527.10	25.74787598	0.032836598	0.147440816	0.222710368	0.823760932	0.913038715	MYO1H	protein_coding
ENSG00000174529.7	99.82022469	0.060471871	0.06084373	0.993888298	0.320277252	0.549931905	TMEM81	protein_coding
ENSG00000174547.13	2226.71986	-0.130422827	0.065061866	-2.004597073	0.04500614	0.166333013	MRPL11	protein_coding
ENSG00000174562.13	24.94178866	0.140669424	0.276771517	0.508251087	0.611277268	0.786489991	KLK15	protein_coding
ENSG00000174564.13	115.6694853	0.377660183	0.141205732	2.674538621	0.007483216	0.049311085	IL20RB	protein_coding
ENSG00000174567.8	477.3631	-0.46293586	0.158045512	-2.929130061	0.003399122	0.028192524	GOLT1A	protein_coding
ENSG00000174572.3	10.92406619	-0.181441625	0.118712868	-1.52840739	0.126411421	0.317134744	RPL10P2	processed_pseudogene
ENSG00000174574.16	3278.287956	0.04137352	0.046103941	0.897396606	0.369507323	0.596255651	AKIRIN1	protein_coding
ENSG00000174576.10	17.19029702	-0.244814643	0.188953023	-1.295637612	0.195100365	0.41215283	NPAS4	protein_coding
ENSG00000174579.4	2521.408851	-0.045515771	0.0438932	-1.036966323	0.299751552	0.529481071	MSL2	protein_coding
ENSG00000174586.11	82.86913584	-0.055759775	0.083911723	-0.664505181	0.506367034	0.710412375	ZNF497	protein_coding
ENSG00000174599.5	50.13716596	0.27836396	0.139810026	1.991015731	0.046479158	0.169761316	TRAM1L1	protein_coding
ENSG00000174600.14	949.3079189	-0.236782272	0.117435702	-2.016271614	0.043771581	0.163279764	CMKLR1	protein_coding
ENSG00000174606.14	1564.758096	-0.024986569	0.041969119	-0.595356036	0.551605506	0.743701843	ANGEL2	protein_coding
ENSG00000174607.11	2701.874227	-0.046905019	0.127084595	-0.369085009	0.712064362	0.84949297	UGT8	protein_coding
ENSG00000174611.12	99.23964624	-0.071405604	0.170334484	-0.41920815	0.675064016	0.827445473	KY	protein_coding
ENSG00000174628.16	563.0878936	-0.12188548	0.070161291	-1.737218315	0.082348643	0.24449866	IQCK	protein_coding
ENSG00000174640.15	3697.355938	-0.110296459	0.115961424	-0.951147845	0.34152933	0.571448406	SLCO2A1	protein_coding
ENSG00000174652.19	1526.366994	-0.063890859	0.065754855	-0.971652347	0.331223531	0.560728095	ZNF266	protein_coding
ENSG00000174667.4	7.09950024	-0.19177116	0.260405999	-0.73643142	0.461468189	0.674094796	OR7D4	protein_coding
ENSG00000174669.12	2048.172767	-0.07661714	0.084080123	-0.911239636	0.362169124	0.589731649	SLC29A2	protein_coding
ENSG00000174672.16	355.538356	-0.041286817	0.17879249	-0.230920307	0.817376714	0.909961882	BRSK2	protein_coding
ENSG00000174677.4	0.701342235	-0.272236064	0.445551402	-0.61100933	0.541193404		VN1R6P	unitary_pseudogene

ENSG00000174678.9	1.005803674	0.276893978	0.327375406	0.84579957	0.397664565	0.620809285	FAM47DP	processed_pseudogene
ENSG00000174680.10	7.402286814	0.14526768	0.171302271	0.848019577	0.396427068	0.619712382	GRIK1-AS1	lncRNA
ENSG00000174684.7	1466.176248	-0.260272848	0.071720594	-3.628983425	0.00028454	0.004469855	B4GAT1	protein_coding
ENSG00000174695.10	4989.349401	0.105892737	0.051791973	2.044578145	0.040896466	0.156109434	TMEM167A	protein_coding
ENSG00000174697.5	17.8486371	0.136822105	0.173763521	0.787404078	0.43104536	0.649104228	LEP	protein_coding
ENSG00000174705.13	4576.544225	0.471512595	0.08865009	5.318805571	1.04451E-07	8.32111E-06	SH3PXD2B	protein_coding
ENSG00000174715.7	13.35876891	-0.190145511	0.107278329	-1.77245034	0.076319832	0.232914722	PPIAP72	processed_pseudogene
ENSG00000174718.12	5655.414415	-0.166371464	0.075818092	-2.194350451	0.028210225	0.122259272	RESF1	protein_coding
ENSG00000174720.16	1996.501336	-0.218428987	0.055318207	-3.948591201	7.86125E-05	0.001645387	LARP7	protein_coding
ENSG00000174721.10	100.1388131	0.058886858	0.083629519	0.704139614	0.481345832	0.69028227	FGFBP3	protein_coding
ENSG00000174738.13	2799.094716	0.014883108	0.062903484	0.236602292	0.812965342	0.907582894	NR1D2	protein_coding
ENSG00000174740.8	53.24057367	0.006874358	0.132471783	0.051893001	0.958613951	0.980760222	PABPC5	protein_coding
ENSG00000174744.14	2363.780925	-0.132513071	0.064419593	-2.05703055	0.039683287	0.153345625	BRMS1	protein_coding
ENSG00000174748.22	46375.33713	-0.045412991	0.055362632	-0.820282381	0.412055148	0.632813359	RPL15	protein_coding
ENSG00000174749.6	1131.007307	-0.081262431	0.070806919	-1.147662294	0.251108003	0.477777297	FAM241A	protein_coding
ENSG00000174775.17	1294.058782	0.039568474	0.067005374	0.590526876	0.554837473	0.745889864	HRAS	protein_coding
ENSG00000174776.11	12.51892938	-0.228616684	0.201413018	-1.135064092	0.256348465	0.483323705	WDR49	protein_coding
ENSG00000174780.17	7831.715224	0.026166678	0.043396961	0.602961068	0.546534589	0.740383074	SRP72	protein_coding
ENSG00000174788.10	17.00224802	0.103595728	0.140121973	0.739325356	0.459709456	0.673010285	PCP2	protein_coding
ENSG00000174791.11	1479.860858	-0.043769704	0.101927768	-0.429418838	0.667618447	0.822529007	RIN1	protein_coding
ENSG00000174792.11	8.092993452	0.138623003	0.168704471	0.821691339	0.41125259	0.632161773	ODAPH	protein_coding
ENSG00000174796.12	817.1431052	0.052694139	0.042796031	1.231285646	0.218216049	0.439816337	THAP6	protein_coding
ENSG00000174799.11	794.0013589	-0.042685113	0.055793613	-0.765053761	0.444239532	0.660208243	CEP135	protein_coding
ENSG00000174804.4	1637.356325	0.110255191	0.08355834	1.319499536	0.187002165	0.401962874	FZD4	protein_coding
ENSG00000174807.4	2964.664975	0.546188881	0.102130594	5.347945822	8.89581E-08	7.36982E-06	CD248	protein_coding
ENSG00000174808.12	469.3640563	0.150294878	0.113063939	1.329291014	0.183751978	0.397683632	BTC	protein_coding
ENSG00000174827.14	221.9016459	-0.748234316	0.178683274	-4.187489412	2.82057E-05	0.00072686	PDZK1	protein_coding
ENSG00000174837.15	89.24463892	-0.090886327	0.136625228	-0.665223603	0.505907486	0.710036733	ADGRE1	protein_coding
ENSG00000174839.13	1721.831643	0.062193311	0.042487216	1.463812345	0.143245229	0.342263259	DENND6A	protein_coding
ENSG00000174840.9	3157.687647	-0.047520116	0.051595066	-0.921020559	0.357039693	0.584782332	PDE12	protein_coding
ENSG00000174842.17	465.035591	-0.082679854	0.066271506	-1.247592798	0.212180217	0.432772166	GLMN	protein_coding
ENSG00000174844.14	70.94265643	-0.099696705	0.156825896	-0.63571583	0.524961656	0.724101133	DNAH12	protein_coding
ENSG00000174851.16	5219.414226	-0.002803389	0.06360094	-0.044077791	0.964842396	0.984062284	YIF1A	protein_coding
ENSG00000174871.11	81.42161851	0.113917481	0.120049663	0.948919627	0.342661492	0.572464339	CNIH2	protein_coding
ENSG00000174876.16	1.750423587	0.062043554	0.152431081	0.407026926	0.683988212	0.833144681	AMY1B	protein_coding
ENSG00000174885.13	339.3793803	-0.391262478	0.211282205	-1.851847762	0.064047687	0.208066281	NLRP6	protein_coding
ENSG00000174886.14	584.7801877	-0.054162468	0.071596246	-0.756498717	0.449350241	0.664156818	NDUFA11	protein_coding
ENSG00000174891.13	1583.336616	-0.02038794	0.050165768	-0.406411402	0.684440344	0.833403846	RSRC1	protein_coding
ENSG00000174898.16	14.48867271	-0.013721215	0.229295887	-0.059840649	0.952282551	0.977642998	CATSPERD	protein_coding
ENSG00000174899.12	125.9610865	-0.08358862	0.19646157	-0.42547059	0.670493657	0.824345505	SLC66A1L	transcribed_unitary_pseudogene
ENSG00000174903.16	5647.475093	0.05931303	0.061044684	0.971633005	0.331233157	0.560728095	RAB1B	protein_coding
ENSG00000174912.7	29.44220378	-0.001300237	0.103458254	-0.012567745	0.989972654	0.995414303	METTL15P1	processed_pseudogene

ENSG00000174914.3	1.167736093	0.763393846	0.443412444	1.721633789	0.085135878	0.249671441	OR9G1	protein_coding
ENSG00000174915.12	1579.583866	-0.124525331	0.055945013	-2.225852224	0.026024081	0.115968033	PTDSS2	protein_coding
ENSG00000174917.9	1847.297296	-0.126549814	0.071375226	-1.773021535	0.076225139	0.232719477	MICOS13	protein_coding
ENSG00000174928.16	57.78050879	-0.125471032	0.083263375	-1.506917433	0.131831822	0.325375811	C3orf33	protein_coding
ENSG00000174930.4	1.447608321	-0.455450842	0.433072793	-1.05167272	0.292949731	0.522848559	VN2R1P	unitary_pseudogene
ENSG00000174937.5	0.125626236	-0.15461246	1.205941603	-0.128208912	0.897983648		OR5M3	protein_coding
ENSG00000174938.14	6658.107797	0.117274509	0.11627242	1.008618455	0.313157653	0.54257709	SEZ6L2	protein_coding
ENSG00000174939.11	1128.247146	-0.021223878	0.152002017	-0.139628923	0.888953187	0.946992776	ASPHD1	protein_coding
ENSG00000174943.11	1297.464135	-0.022912354	0.058068417	-0.394575149	0.693156457	0.837965038	KCTD13	protein_coding
ENSG00000174944.9	347.6436771	-0.379247942	0.143131549	-2.649646033	0.008057614	0.052041893	P2RY14	protein_coding
ENSG00000174945.13	73.1793628	-0.084819136	0.103325352	-0.820893751	0.411706791	0.632547573	AMZ1	protein_coding
ENSG00000174946.7	221.3644758	-0.737563187	0.129964655	-5.675105966	1.38603E-08	1.57933E-06	GPR171	protein_coding
ENSG00000174948.6	2.041663781	0.460430149	0.375658039	1.225662973	0.220325529	0.442325882	GPR149	protein_coding
ENSG00000174950.11	31.14077275	0.136007276	0.176168157	0.772030988	0.440096075	0.656580733	CD164L2	protein_coding
ENSG00000174951.11	619.9333925	-0.206124191	0.129033804	-1.597443346	0.110166917	0.291501836	FUT1	protein_coding
ENSG00000174953.14	3358.238457	0.061583905	0.046530984	1.323503184	0.185668098	0.400339372	DHX36	protein_coding
ENSG00000174957.1	0.748101878	-0.205298484	0.542475279	-0.378447631	0.70509809		OR5J2	protein_coding
ENSG00000174963.18	12.3263018	0.244457586	0.334490876	0.73083484	0.46488004	0.676803338	ZIC4	protein_coding
ENSG00000174970.5	1.478201139	-0.971851702	0.499197787	-1.946826945	0.051555485	0.18147281	OR10AG1	protein_coding
ENSG00000174977.8	217.4249884	-0.090593512	0.076695292	-1.181213473	0.23751793	0.462766002	AC026271.1	processed_pseudogene
ENSG00000174982.4	0.275010279	-0.032852457	0.981234705	-0.033480733	0.97329123		OR4S2	protein_coding
ENSG00000174989.13	1252.200996	-0.008838488	0.048650555	-0.181672908	0.855839428	0.929653632	FBXW8	protein_coding
ENSG00000174990.8	4.791623638	-0.40916175	0.265360407	-1.541909566	0.123095571	0.31219507	CA5A	protein_coding
ENSG00000174992.8	60.73624575	-0.778998267	0.247278239	-3.150290422	0.001631082	0.01651433	ZG16	protein_coding
ENSG00000174996.12	2271.304823	0.156396179	0.064244155	2.434403243	0.014916367	0.079780867	KLC2	protein_coding
ENSG00000175003.15	28.29628302	-0.093212842	0.116155776	-0.802481332	0.422274583	0.641568214	SLC22A1	protein_coding
ENSG00000175018.13	1.751688141	-0.116489753	0.388945073	-0.29950181	0.764557191	0.880077891	TEX36	protein_coding
ENSG00000175029.17	6467.810691	0.172556739	0.059484928	2.900848101	0.003721542	0.03010755	CTBP2	protein_coding
ENSG00000175040.6	952.1217387	-0.329927331	0.113450714	-2.908111537	0.003636186	0.029584408	CHST2	protein_coding
ENSG00000175048.17	1219.85386	-0.030138153	0.068208333	-0.441854417	0.658594557	0.816810011	ZDHHC14	protein_coding
ENSG00000175054.16	2544.587726	-0.026784642	0.064594348	-0.414659228	0.678391383	0.829538438	ATR	protein_coding
ENSG00000175061.18	7681.857829	-0.044009006	0.085613016	-0.514045736	0.607220015	0.783397773	SNHG29	lncRNA
ENSG00000175063.17	4762.421769	-0.148809898	0.112422873	-1.323662107	0.185615289	0.400245308	UBE2C	protein_coding
ENSG00000175065.12	27.07155543	0.030971529	0.20282131	0.152703523	0.878632082	0.941457873	DSG4	protein_coding
ENSG00000175066.16	2368.222419	-0.054980366	0.063308729	-0.868448433	0.38514889	0.609827242	GK5	protein_coding
ENSG00000175073.8	2668.556872	0.001581438	0.047404524	0.033360485	0.97338712	0.987596986	VCPIP1	protein_coding
ENSG00000175077.6	1.825433316	-0.105848714	0.281374894	-0.376183934	0.706780159	0.846392013	RTP1	protein_coding
ENSG00000175084.12	52146.74031	-0.799553102	0.28526562	-2.802837235	0.005065523	0.037553637	DES	protein_coding
ENSG00000175087.10	962.0121306	-0.086494705	0.059694422	-1.448957903	0.147349339	0.348496182	PDIK1L	protein_coding
ENSG00000175093.5	22.70297572	0.580300733	0.151463943	3.831279724	0.000127478	0.002382131	SPSB4	protein_coding
ENSG00000175097.8	1.373611705	-0.558998655	0.325015448	-1.71991411	0.085448055	0.250154527	RAG2	protein_coding
ENSG00000175104.15	1571.610564	0.055876526	0.048126868	1.161025608	0.245631485	0.472160708	TRAF6	protein_coding

ENSG00000175105.8	1140.886178	0.116932588	0.048392301	2.41634691	0.015677117	0.08252739	ZNF654	protein_coding
ENSG00000175106.17	168.759152	-0.18654539	0.083102904	-2.244751748	0.024784074	0.112454768	TVP23C	protein_coding
ENSG00000175110.12	1419.398045	-0.054910723	0.054466032	-1.008164549	0.313375474	0.542678549	MRPS22	protein_coding
ENSG00000175115.13	8841.107587	0.061123494	0.061149834	0.999569261	0.317519005	0.546782071	PACS1	protein_coding
ENSG00000175121.11	8.480187015	1.357654218	0.275003106	4.936868676	7.93869E-07	4.35698E-05	WFDC5	protein_coding
ENSG00000175130.7	19468.74005	-0.084828599	0.072773814	-1.165647298	0.243757076	0.469717967	MARCKSL1	protein_coding
ENSG00000175137.11	1799.311255	0.127128787	0.048849301	2.602468895	0.009255521	0.057248823	SH3BP5L	protein_coding
ENSG00000175143.5	0.485090048	-0.029794028	0.6851636	-0.043484546	0.965315283		OR2T1	protein_coding
ENSG00000175147.13	413.8888995	0.024623148	0.100628506	0.244693569	0.806693708	0.903947499	TMEM51-AS1	lncRNA
ENSG00000175155.11	1653.390896	0.126716761	0.062411995	2.030327037	0.042323308	0.159861967	YPEL2	protein_coding
ENSG00000175161.14	74.49234002	-0.63696182	0.229359769	-2.777129673	0.005484129	0.039678219	CADM2	protein_coding
ENSG00000175164.15	2880.231046	-0.150544808	0.147477623	-1.020797629	0.307350328	0.536831346	ABO	protein_coding
ENSG00000175166.17	11741.84169	0.13180624	0.054598518	2.414099228	0.015774166	0.082918073	PSMD2	protein_coding
ENSG00000175170.16	51.30435284	0.147280311	0.134006484	1.099053622	0.271744678	0.499965798	FAM182B	lncRNA
ENSG00000175175.6	75.70152931	0.275943456	0.171424359	1.609709714	0.107461243	0.287346624	PPM1E	protein_coding
ENSG00000175182.15	901.4446163	0.023218494	0.070701382	0.328402264	0.742607531	0.867278751	FAM131A	protein_coding
ENSG00000175183.10	677.0661933	0.023482529	0.107541242	0.218358357	0.827149909	0.914910059	CSRP2	protein_coding
ENSG00000175189.4	13.07295555	-0.214996079	0.174604197	-1.23133397	0.218197982	0.439816337	INHBC	protein_coding
ENSG00000175193.14	410.6355546	-0.146390521	0.080963866	-1.808096977	0.070591409	0.220971692	PARL	protein_coding
ENSG00000175197.12	637.9831611	-0.074549967	0.091602325	-0.813843607	0.41573455	0.636111815	DDIT3	protein_coding
ENSG00000175198.17	989.2827701	0.230645183	0.083916906	2.748494842	0.005986958	0.042256828	PCCA	protein_coding
ENSG00000175202.6	2.607662011	-0.155011367	0.186823118	-0.82972262	0.406695629	0.627931636	HIGD2B	protein_coding
ENSG00000175203.17	5358.634969	0.058472344	0.052499111	1.113777792	0.265374527	0.493270165	DCTN2	protein_coding
ENSG00000175206.10	8.311891775	-0.049525267	0.137629588	-0.359844623	0.718963331	0.853050793	NPPA	protein_coding
ENSG00000175213.3	930.0962163	0.029516225	0.051884535	0.568882911	0.569435603	0.756283552	ZNF408	protein_coding
ENSG00000175215.11	14018.94222	0.062436882	0.054029268	1.155612205	0.247839814	0.474336011	CTDSP2	protein_coding
ENSG00000175216.15	9542.821563	0.054467812	0.057323921	0.950175966	0.342022848	0.571815478	CKAP5	protein_coding
ENSG00000175220.12	11697.65642	0.118860393	0.049720379	2.390576972	0.016821923	0.086659052	ARHGAP1	protein_coding
ENSG00000175221.15	4918.192844	0.010303759	0.057332235	0.179720171	0.857372257	0.930352956	MED16	protein_coding
ENSG00000175224.16	5876.008056	0.037994724	0.042661477	0.890609675	0.373138607	0.59941207	ATG13	protein_coding
ENSG00000175229.7	3.820486766	-0.558710369	0.244334077	-2.286665764	0.022215339	0.104273788	GAL3ST3	protein_coding
ENSG00000175262.14	36.8111433	-0.39597845	0.145991305	-2.712342703	0.006680948	0.04550417	C1orf127	protein_coding
ENSG00000175264.8	408.4271468	0.309030481	0.106747215	2.894974635	0.003791892	0.0305151	CHST1	protein_coding
ENSG00000175265.17	1537.243927	-0.074919898	0.124615372	-0.601209122	0.54770071	0.741100464	GOLGA8A	protein_coding
ENSG00000175267.15	23.96942033	0.403823238	0.178830583	2.258132982	0.02393737	0.109636105	VWA3A	protein_coding
ENSG00000175274.19	12150.56605	-0.067292489	0.089950063	-0.748109412	0.454394166	0.668606971	TP53I11	protein_coding
ENSG00000175279.22	115.9307416	-0.252119216	0.089463739	-2.81811626	0.004830632	0.036331451	CENPS	protein_coding
ENSG00000175283.8	1293.387947	0.080855803	0.054852589	1.474056276	0.14046644	0.338220027	DOLK	protein_coding
ENSG00000175287.19	295.7281429	-0.103133433	0.152306867	-0.677142371	0.498315628	0.704098444	PHYHD1	protein_coding
ENSG00000175294.6	43.38431295	0.900355216	0.147069095	6.121987856	9.24151E-10	1.68501E-07	CATSPER1	protein_coding
ENSG00000175302.5	0.925987922	0.717102504	0.442305477	1.621283348	0.104956885		ANKRD30BP1	unprocessed_pseudogene
ENSG00000175305.17	291.3678979	-0.018241805	0.089022101	-0.204913216	0.83763993	0.920088347	CCNE2	protein_coding

ENSG00000175309.15	2057.701268	-0.007708269	0.048901458	-0.157628615	0.874749454	0.939469056	PHYKPL	protein_coding
ENSG00000175311.7	1371.51466	-0.359548739	0.135242274	-2.658552895	0.007847703	0.051069801	ANKS4B	protein_coding
ENSG00000175315.3	77.03976303	1.087948963	0.222522317	4.889167869	1.01263E-06	5.24231E-05	CST6	protein_coding
ENSG00000175318.12	193.1160724	0.338357548	0.147570746	2.292849755	0.021856656	0.103078735	GRAMD2A	protein_coding
ENSG00000175319.3	0.625681333	-0.000553667	0.41361901	-0.001338591	0.998931959		NF1P5	processed_pseudogene
ENSG00000175322.12	284.5326077	-0.095949103	0.078851109	-1.216838981	0.223665486	0.446398775	ZNF519	protein_coding
ENSG00000175324.10	656.7666861	-0.002879713	0.063870313	-0.045086877	0.964038061	0.983471548	LSM1	protein_coding
ENSG00000175325.2	2.739209424	-0.165377372	0.276922167	-0.597198028	0.550375175	0.742906376	PROP1	protein_coding
ENSG00000175329.13	274.6123742	0.191228213	0.333884213	0.572738108	0.566822031	0.754652569	ISX	protein_coding
ENSG00000175334.8	7272.73312	-0.13024451	0.063676967	-2.045394371	0.040815993	0.155911514	BANF1	protein_coding
ENSG00000175336.10	5.658290795	0.136605905	0.228784465	0.597094321	0.550444409	0.742906376	APOF	protein_coding
ENSG00000175344.19	225.0060816	-0.018191861	0.16669486	-0.109132704	0.913097236	0.95894649	CHRNA7	protein_coding
ENSG00000175348.11	2830.055147	-0.062077084	0.049556284	-1.252658171	0.210330135	0.430680377	TMEM9B	protein_coding
ENSG00000175352.11	174.5341813	0.18340356	0.12288093	1.4925307	0.135560075	0.331163886	NRIP3	protein_coding
ENSG00000175354.20	2397.460037	0.01251494	0.054122726	0.231232623	0.817134087	0.909843627	PTPN2	protein_coding
ENSG00000175356.14	321.659665	-0.225997513	0.154268879	-1.464958545	0.142932228	0.341721496	SCUBE2	protein_coding
ENSG00000175376.9	1709.768175	0.014865648	0.04010319	0.37068492	0.710872223	0.848812642	EIF1AD	protein_coding
ENSG00000175387.16	4687.742672	0.081921408	0.049400072	1.658325665	0.097251751	0.270864091	SMAD2	protein_coding
ENSG00000175390.15	9858.508204	-0.150026772	0.043585014	-3.442164175	0.00057708	0.007582814	EIF3F	protein_coding
ENSG00000175395.16	637.7993909	-0.168107116	0.079611562	-2.111591745	0.034721481	0.140040914	ZNF25	protein_coding
ENSG00000175398.2	1.217242884	-0.352588519	0.432096725	-0.815994425	0.414503324	0.634876043	OR10P1	protein_coding
ENSG00000175414.7	490.6393026	0.214379368	0.09734051	2.202365369	0.02763951	0.120654043	ARL10	protein_coding
ENSG00000175416.16	7686.076835	0.050258829	0.087993565	0.571164824	0.56788792	0.755375352	CLTB	protein_coding
ENSG00000175426.11	154.5948371	0.721125967	0.181960909	3.9630818	7.39884E-05	0.00157201	PCSK1	protein_coding
ENSG00000175445.17	622.6484173	0.53046137	0.143085267	3.707309502	0.000209473	0.003513634	LPL	protein_coding
ENSG00000175449.14	35.69726643	-0.208938065	0.085114841	-2.454778309	0.01409715	0.076788936	RFESD	protein_coding
ENSG00000175455.16	2395.364728	0.025109357	0.071368967	0.351824582	0.724969822	0.856504444	CCDC14	protein_coding
ENSG00000175463.12	552.5557133	-0.659548638	0.130364483	-5.059266328	4.20873E-07	2.59779E-05	TBC1D10C	protein_coding
ENSG00000175467.15	4327.155094	0.03324095	0.050773748	0.654687731	0.512668826	0.71521864	SART1	protein_coding
ENSG00000175470.20	1730.761632	-0.025115071	0.037927546	-0.662185501	0.507852345	0.711703305	PPP2R2D	protein_coding
ENSG00000175471.19	765.2837773	0.087018216	0.095820258	0.908140074	0.36380422	0.590998261	MCTP1	protein_coding
ENSG00000175482.9	559.4649181	0.03933236	0.082604256	0.476154158	0.633964556	0.801388723	POLD4	protein_coding
ENSG00000175485.2	2.130767828	0.30889863	0.206708356	1.494369342	0.13507911	0.330452051	OR52W1	protein_coding
ENSG00000175489.10	541.0066776	0.030082036	0.10823932	0.277921521	0.781072603	0.889107173	LRRC25	protein_coding
ENSG00000175497.17	45.07684286	-0.239372631	0.2915199	-0.821119352	0.411578288	0.632436932	DPP10	protein_coding
ENSG00000175505.11	717.7629968	0.258193578	0.108986382	2.36904441	0.017834112	0.089867143	CLCF1	protein_coding
ENSG00000175509.8	9.968708619	-0.264765738	0.313156549	-0.845474058	0.397846211	0.620890773	ACRP1	processed_pseudogene
ENSG00000175513.9	5.061727205	-0.011540152	0.191318724	-0.06031899	0.951901577	0.977372445	TSGA10IP	protein_coding
ENSG00000175514.2	3.087815836	-0.214611705	0.206807683	-1.037735646	0.299393145	0.529291628	GPR152	protein_coding
ENSG00000175518.8	18.23902116	-0.043451993	0.134915415	-0.322068406	0.747400872	0.870027723	UBQLNL	protein_coding
ENSG00000175520.9	0.769652158	0.125402366	0.360005911	0.348334186	0.727589223		UBQLN3	protein_coding
ENSG00000175535.6	10.49445814	-2.036951676	0.394326609	-5.165646018	2.3961E-07	1.63916E-05	PNLIP	protein_coding

ENSG00000175536.7	283.3861174	-0.231357796	0.089345582	-2.589471027	0.009612351	0.058730913	LIPT2	protein_coding
ENSG00000175538.11	4716.22203	-0.247499443	0.135132912	-1.831526014	0.067022069	0.21393354	KCNE3	protein_coding
ENSG00000175544.14	130.3247076	0.885660482	0.151752203	5.836228161	5.33958E-09	7.19611E-07	CABP4	protein_coding
ENSG00000175548.9	678.9033907	0.174722172	0.09444	1.850086536	0.064301078	0.208627114	ALG10B	protein_coding
ENSG00000175550.8	3523.194573	-0.047610596	0.069185774	-0.688155859	0.491354637	0.698484343	DRAP1	protein_coding
ENSG00000175556.17	524.8082981	0.495264783	0.118637592	4.174602476	2.98507E-05	0.000759815	LONRF3	protein_coding
ENSG00000175564.13	57.59494748	-0.03655051	0.076791234	-0.475972426	0.634094023	0.80143614	UCP3	protein_coding
ENSG00000175567.11	8394.713461	-0.274482108	0.099975897	-2.745482811	0.006042193	0.042536455	UCP2	protein_coding
ENSG00000175573.7	2191.483437	0.145684948	0.06124733	2.378633446	0.017376946	0.08840686	C11orf68	protein_coding
ENSG00000175575.13	1001.670448	-0.16704414	0.053103997	-3.145603879	0.001657443	0.016707473	PAAF1	protein_coding
ENSG00000175581.14	1674.787019	-0.062182507	0.063930774	-0.972653745	0.330725421	0.560255858	MRPL48	protein_coding
ENSG00000175582.20	8784.621126	0.09481994	0.047400716	2.000390456	0.045458118	0.167309517	RAB6A	protein_coding
ENSG00000175591.12	464.7394236	0.59799484	0.143171688	4.176767405	2.95681E-05	0.000754747	P2RY2	protein_coding
ENSG00000175592.9	1985.693226	0.904016141	0.149017523	6.066508992	1.3072E-09	2.23388E-07	FOSL1	protein_coding
ENSG00000175595.15	541.763393	-0.089405368	0.049495988	-1.806315449	0.07086908	0.221515231	ERCC4	protein_coding
ENSG00000175600.16	144.3916029	0.410549668	0.123859243	3.314647011	0.000917589	0.010763333	SUGCT	protein_coding
ENSG00000175602.4	2135.219659	0.008717653	0.084124522	0.10362797	0.917464591	0.960881428	CCDC85B	protein_coding
ENSG00000175604.2	19.74728091	0.036214883	0.128520328	0.281783308	0.778109671	0.887635737	AC007601.1	lncRNA
ENSG00000175606.11	1816.883443	-0.234514628	0.060665463	-3.865702409	0.00011077	0.002141536	TMEM70	protein_coding
ENSG00000175611.12	234.6229498	-0.262463972	0.074464477	-3.524686982	0.000423983	0.006010386	LINC00476	lncRNA
ENSG00000175619.3	0.332008108	-0.304799392	0.534902933	-0.569821875	0.568798517		OR4B1	protein_coding
ENSG00000175634.15	2217.483279	-0.033165418	0.0635636	-0.521767454	0.601832252	0.779933506	RPS6KB2	protein_coding
ENSG00000175643.10	1069.328409	-0.00134358	0.087437294	-0.015366213	0.987740018	0.994152034	RMI2	protein_coding
ENSG00000175646.4	0.599130231	-0.120078056	0.400014723	-0.300184091	0.764036739		PRM1	protein_coding
ENSG00000175658.4	0.290825302	-0.157385846	0.3760194	-0.418557781	0.67553935		DRD5P2	processed_pseudogene
ENSG00000175662.18	3638.240832	0.008143339	0.076946404	0.10583132	0.915716187	0.960065544	TOM1L2	protein_coding
ENSG00000175664.10	4.996330539	-0.121022122	0.206973672	-0.584722304	0.558734454	0.748810879	TEX26	protein_coding
ENSG00000175676.15	1.150626905	-0.469375045	0.255356469	-1.838116915	0.066045179	0.211901839	GOLGA8DP	transcribed_unprocessed_pseudogene
ENSG00000175691.9	223.9305944	0.053607968	0.060313656	0.888819732	0.374099969	0.599983599	ZNF77	protein_coding
ENSG00000175697.11	28.77343719	0.320978922	0.170135834	1.886603864	0.059213627	0.198214181	GPR156	protein_coding
ENSG00000175699.15	1.327152206	0.217140714	0.312066275	0.69581602	0.486544055	0.694095015	CCDC197	protein_coding
ENSG00000175701.10	833.3970648	-0.271274201	0.083682382	-3.24171224	0.001188139	0.013104415	MTLN	protein_coding
ENSG00000175707.9	1238.47218	0.054994877	0.081334201	0.67615931	0.498939503	0.70456874	KDF1	protein_coding
ENSG00000175711.8	334.6422628	0.00185367	0.065613511	0.028251344	0.977461687	0.989933251	B3GNTL1	protein_coding
ENSG00000175718.10	1.097295193	0.063450897	0.31628082	0.200615698	0.840999082	0.921927096	RBMXL3	protein_coding
ENSG00000175727.14	12926.06341	0.012047288	0.068269734	0.17646601	0.859927849	0.931893212	MLXIP	protein_coding
ENSG00000175728.4	2.239278995	0.020412041	0.270297557	0.075516927	0.93980343	0.97152854	LINC02873	lncRNA
ENSG00000175730.8	27.40188129	0.010678289	0.070014649	0.152515063	0.878780711	0.941471389	BAK1P1	processed_pseudogene
ENSG00000175741.5	55.86973768	-0.082956234	0.063510381	-1.306183845	0.191490045	0.407528897	RWDD4P2	processed_pseudogene
ENSG00000175745.14	1654.427167	0.315046797	0.129026314	2.441725149	0.01461727	0.078693752	NR2F1	protein_coding
ENSG00000175746.6	6.409149604	-0.245822021	0.168416183	-1.459610456	0.144397164	0.344091169	C15orf54	lncRNA
ENSG00000175749.11	3.929114984	-0.15953237	0.129863892	-1.228458252	0.219274992	0.4410092	EIF3KP1	processed_pseudogene

ENSG00000175756.13	4370.25234	-0.155893451	0.071549615	-2.17881608	0.029345334	0.125641739	AURKAIP1	protein_coding
ENSG00000175764.14	327.3301381	-0.103967646	0.082660861	-1.257761467	0.208478035	0.428469902	TTLL11	protein_coding
ENSG00000175766.13	2.227345687	0.011231923	0.302794294	0.037094235	0.970409869	0.98613756	EIF4E1B	protein_coding
ENSG00000175768.13	102.3821635	-0.010310665	0.080232339	-0.128510089	0.897745315	0.951450551	TOMM5	protein_coding
ENSG00000175772.11	170.4690149	-0.344216362	0.094222759	-3.653218861	0.000258973	0.004152053	LINC01106	lncRNA
ENSG00000175773.13	20.4441502	-0.137771832	0.105034395	-1.311683021	0.189627108	0.405110246	ZBTB44-DT	lncRNA
ENSG00000175779.4	27.43481333	-0.52754012	0.124895062	-4.223866907	2.40146E-05	0.000640343	LINC02694	lncRNA
ENSG00000175782.11	1323.270201	0.01746947	0.082387107	0.212041311	0.832074805	0.917316703	SLC35E3	protein_coding
ENSG00000175785.13	554.0311093	-0.632816895	0.229528254	-2.757032671	0.005832852	0.041491708	PRIMA1	protein_coding
ENSG00000175787.17	229.1423581	-0.0821936	0.061507075	-1.336327562	0.181442232	0.394841798	ZNF169	protein_coding
ENSG00000175792.12	2566.508388	0.085114958	0.062301768	1.366172434	0.171884848	0.382469268	RUVBL1	protein_coding
ENSG00000175793.12	9542.633873	0.281382321	0.145571875	1.932944259	0.053243065	0.18508081	SFN	protein_coding
ENSG00000175800.5	3.265032231	-0.33479342	0.187567066	-1.784926462	0.074273246	0.228589959	OR52B3P	unitary_pseudogene
ENSG00000175806.15	828.9367769	0.011326653	0.093744383	0.12082487	0.903829751	0.954171989	MSRA	protein_coding
ENSG00000175809.6	1.513837632	-0.505987365	0.365037528	-1.386124234	0.165708972	0.373782279	CBLL2	protein_coding
ENSG00000175820.4	16.19211639	0.017639324	0.164211183	0.107418534	0.914456952	0.959547211	CCDC168	protein_coding
ENSG00000175826.12	2681.042733	-0.035707078	0.053237655	-0.670710954	0.502404683	0.707201156	CTDNEP1	protein_coding
ENSG00000175832.13	2786.507808	0.28816991	0.135404877	2.128209237	0.033319739	0.136412465	ETV4	protein_coding
ENSG00000175841.8	5.665337831	-0.508207607	0.175033165	-2.903493211	0.00369025	0.02993485	FAM172BP	processed_pseudogene
ENSG00000175854.14	797.4449774	0.006975356	0.064354682	0.108389249	0.913686929	0.959207287	SWI5	protein_coding
ENSG00000175857.8	226.1338833	-0.663376856	0.167405218	-3.962701182	7.41065E-05	0.001573752	GAPT	protein_coding
ENSG00000175866.15	2418.474558	0.474775051	0.099085206	4.791583617	1.6547E-06	7.6955E-05	BAIAP2	protein_coding
ENSG00000175868.14	7.302046532	-0.008986687	0.197696882	-0.045456896	0.963743131	0.983375587	CALCB	protein_coding
ENSG00000175874.10	226.4736727	0.552213941	0.191730399	2.88015851	0.003974753	0.031607263	CREG2	protein_coding
ENSG00000175877.4	16.93745467	0.199865063	0.189831267	1.052856392	0.292406815	0.522206737	TMEM270	protein_coding
ENSG00000175879.9	107.8245	0.153280578	0.088781916	1.726484225	0.084260335	0.248011659	HOXD8	protein_coding
ENSG00000175886.10	104.8245578	-0.225450915	0.079897845	-2.821739616	0.004776394	0.036097975	RPL7AP66	processed_pseudogene
ENSG00000175893.12	1468.423497	0.195527281	0.074865536	2.611712844	0.009008989	0.056131643	ZDHHC21	protein_coding
ENSG00000175894.18	89.64313181	-0.06442525	0.184350538	-0.349471452	0.726735399	0.857361493	TSPEAR	protein_coding
ENSG00000175895.4	1938.702169	-0.199899488	0.055024684	-3.632905697	0.000280247	0.004416761	PLEKHF2	protein_coding
ENSG00000175899.15	45414.02856	0.081711819	0.111469771	0.733040156	0.463533938	0.67581742	A2M	protein_coding
ENSG00000175906.5	241.6633515	0.533983483	0.165527216	3.225955793	0.001255527	0.013640519	ARL4D	protein_coding
ENSG00000175920.18	334.2971821	0.254598939	0.1592538	1.598699306	0.109887432	0.290949125	DOK7	protein_coding
ENSG00000175928.6	561.6027228	-0.018402471	0.224331952	-0.08203232	0.934621012	0.969183063	LRRN1	protein_coding
ENSG00000175931.13	2823.085548	0.029461792	0.051828765	0.568444804	0.569732975	0.756572668	UBE2O	protein_coding
ENSG00000175938.7	436.7415307	0.040669219	0.071313358	0.57028893	0.568481748	0.755609288	ORAI3	protein_coding
ENSG00000175946.8	38.0409286	0.226479642	0.162396433	1.394609707	0.163133566	0.3701766	KLHL38	protein_coding
ENSG00000175967.4	4.089146307	-0.18193948	0.190919442	-0.952964655	0.340607979	0.570595652	LINC02880	lncRNA
ENSG00000175970.11	1403.695659	-0.181286067	0.06505006	-2.786870102	0.005321981	0.038859912	UNC119B	protein_coding
ENSG00000175984.15	231.505819	0.506156958	0.129391318	3.911830918	9.1599E-05	0.001853363	DENND2C	protein_coding
ENSG00000175985.10	59.18457745	0.810417362	0.179509328	4.514625341	6.34288E-06	0.000227855	PLEKHD1	protein_coding
ENSG00000176007.10	0.914538757	-0.171328031	0.293339362	-0.584060828	0.559179388		FAM220BP	processed_pseudogene

ENSG00000176009.3	3.004531212	-0.193453243	0.243691243	-0.79384569	0.42728527	0.645915642	ASCL3	protein_coding
ENSG00000176014.13	3067.567975	0.272782089	0.109890491	2.482308412	0.013053424	0.07281478	TUBB6	protein_coding
ENSG00000176018.13	1613.057106	0.021575291	0.050209542	0.429704994	0.66741025	0.822410826	LYSMD3	protein_coding
ENSG00000176020.9	0.16420569	0.013333818	0.470828863	0.028319882	0.977407023		AMIGO3	protein_coding
ENSG00000176022.7	1606.615394	0.077741563	0.067303624	1.155087327	0.248054667	0.474584911	B3GALT6	protein_coding
ENSG00000176024.18	272.3189244	0.082433314	0.085366791	0.965636799	0.334225955	0.563657324	ZNF613	protein_coding
ENSG00000176029.14	12.88741887	0.064888732	0.148470507	0.437047961	0.662076566	0.818941542	C11orf16	protein_coding
ENSG00000176040.13	18.13872318	-0.276908649	0.150234605	-1.84317487	0.065303483	0.210667381	TMPRSS7	protein_coding
ENSG00000176043.5	3.727854613	-0.259143566	0.149054158	-1.73858663	0.0821075	0.244087955	AC007160.1	processed_pseudogene
ENSG00000176046.9	447.6835433	-0.05426358	0.141459519	-0.383597941	0.701276466	0.842917952	NUPR1	protein_coding
ENSG00000176049.16	105.4799105	-0.124537423	0.140187884	-0.888360818	0.374346694	0.600089913	JAKMIP2	protein_coding
ENSG00000176054.6	17.87070677	-0.258300021	0.087201632	-2.96210078	0.003055478	0.026051369	RPL23P2	processed_pseudogene
ENSG00000176055.10	507.6394725	-0.156781533	0.063882425	-2.454220112	0.014119053	0.076851772	MBLAC2	protein_coding
ENSG00000176058.13	2981.852178	-0.095034427	0.092949125	-1.022434877	0.30657512	0.536014445	TPRN	protein_coding
ENSG00000176076.7	11.24458503	0.232914966	0.116860626	1.993100445	0.046250449	0.169195419	KCNE5	protein_coding
ENSG00000176083.18	249.7328145	-0.813783964	0.18365144	-4.431133029	9.37392E-06	0.000308292	ZNF683	protein_coding
ENSG00000176087.15	7326.327705	-0.006451287	0.047656938	-0.135369322	0.892319879	0.948636822	SLC35A4	protein_coding
ENSG00000176092.16	1493.38923	0.324203968	0.146270447	2.216469385	0.026659372	0.117930714	CRYBG2	protein_coding
ENSG00000176095.12	3540.791283	0.112900443	0.043813897	2.576818106	0.009971441	0.060325624	IP6K1	protein_coding
ENSG00000176101.12	2681.398255	-0.074492358	0.067318671	-1.106563109	0.268482865	0.496443238	SSNA1	protein_coding
ENSG00000176102.13	1530.302804	-0.03170044	0.044104103	-0.718763971	0.47228636	0.683316934	CSTF3	protein_coding
ENSG00000176105.14	4408.623274	0.056750146	0.062848017	0.902974335	0.366539516	0.593647978	YES1	protein_coding
ENSG00000176108.9	735.7513937	0.056896308	0.060917333	0.933992102	0.350307967	0.579418897	CHMP6	protein_coding
ENSG00000176115.9	0.30710521	-0.041543656	0.358897004	-0.115753701	0.907847745		AQP7P4	unprocessed_pseudogene
ENSG00000176124.15	533.9634938	0.089352892	0.075089363	1.189954049	0.234064453	0.458645923	DLEU1	lncRNA
ENSG00000176125.7	114.943571	-0.026649212	0.096175464	-0.277089508	0.781711378	0.889555458	UFSP1	protein_coding
ENSG00000176134.5	0.32789954	0.14194137	0.443404694	0.320116978	0.748879656		AL445665.1	transcribed_unprocessed_pseudogene
ENSG00000176136.6	11.96942382	0.716032031	0.212812598	3.36461298	0.000766511	0.009409084	MC5R	protein_coding
ENSG00000176142.13	2154.846017	0.109865397	0.049604935	2.214807799	0.02677326	0.118290412	TMEM39A	protein_coding
ENSG00000176148.16	846.5716574	0.035459693	0.070555634	0.502577765	0.615261165	0.788890596	TCP11L1	protein_coding
ENSG00000176153.12	27564.35771	-0.419913561	0.127718928	-3.287794275	0.001009756	0.011579509	GPX2	protein_coding
ENSG00000176155.19	1522.338858	0.071555739	0.061181465	1.169565621	0.242175819	0.468033167	CCDC57	protein_coding
ENSG00000176160.11	13.74558306	-0.60842017	0.165279789	-3.681152864	0.000232182	0.003815152	HSF5	protein_coding
ENSG00000176165.12	3.33478386	0.01238796	0.255533244	0.048478859	0.961334613	0.982096569	FOXG1	protein_coding
ENSG00000176170.14	1192.630296	0.622496789	0.107513908	5.789918739	7.04205E-09	8.89482E-07	SPHK1	protein_coding
ENSG00000176171.11	1179.088675	-0.057411397	0.134524152	-0.426773896	0.669544022	0.823852035	BNIP3	protein_coding
ENSG00000176177.10	13.21943273	-0.380729362	0.159385694	-2.388729836	0.01690673	0.086962295	ENTHD1	protein_coding
ENSG00000176182.6	801.2180843	-0.006658686	0.065493187	-0.101669902	0.919018693	0.961746298	MYPOP	protein_coding
ENSG00000176183.8	15.30741864	-0.038878491	0.099504916	-0.390719301	0.69600473	0.839602429	AC233964.1	processed_pseudogene
ENSG00000176194.18	14.69330913	1.163256379	0.302809283	3.841547941	0.000122261	0.002307389	CIDEA	protein_coding
ENSG00000176198.3	2.34743823	-0.1750729	0.306693739	-0.570839499	0.568108445	0.755438092	OR11H4	protein_coding
ENSG00000176200.1	1.608847701	-0.627534378	0.442755429	-1.417338641	0.156383943	0.361218227	OR4D11	protein_coding

ENSG00000176204.13	13.19740342	-0.268568707	0.198524347	-1.352825036	0.176111533	0.388184737	LRRTM4	protein_coding
ENSG00000176208.9	898.7801319	-0.049223412	0.070107694	-0.70211141	0.482609687	0.690922399	ATAD5	protein_coding
ENSG00000176209.12	1258.295853	-0.1650958	0.073569744	-2.244071959	0.024827772	0.112570993	SMIM19	protein_coding
ENSG00000176219.3	0.563943814	0.065496002	0.431572157	0.151761417	0.879375114		OR11H6	protein_coding
ENSG00000176222.9	89.45326549	-0.130962515	0.10680855	-1.226142618	0.220145011	0.442065302	ZNF404	protein_coding
ENSG00000176225.15	787.0122425	-0.002318518	0.067745318	-0.034224036	0.9726985	0.987197115	RTTN	protein_coding
ENSG00000176230.7	1.0938795	-0.473902413	0.334830819	-1.415348845	0.15696624	0.361987609	OR4K17	protein_coding
ENSG00000176231.2	0.505289686	0.281827221	0.461019828	0.611312581	0.540992664		OR10H4	protein_coding
ENSG00000176232.10	0.003324219	-0.122775387	2.893054209	-0.042437984	0.966149549		AC092634.1	transcribed_unprocessed_pseudogene
ENSG00000176236.6	11.80557635	-0.301117547	0.126970294	-2.371559016	0.017713218	0.089567056	RPP38-DT	lncRNA
ENSG00000176239.7	0.325076604	0.200066421	0.615005474	0.325308358	0.744947706		OR51B6	protein_coding
ENSG00000176243.9	1.038145115	0.084349033	0.189164643	0.445902743	0.655667501	0.814876727	CDV3P1	processed_pseudogene
ENSG00000176244.7	299.3818427	-0.09626185	0.146955783	-0.655039555	0.512442287	0.714994158	ACBD7	protein_coding
ENSG00000176246.1	0.305241444	0.260175868	0.69176182	0.376106141	0.706837989		OR4L1	protein_coding
ENSG00000176248.9	2475.41252	0.070269345	0.049416237	1.421988998	0.155029445	0.359327595	ANAPC2	protein_coding
ENSG00000176253.4	2.598795703	0.050470866	0.298989995	0.168804531	0.865950394	0.93500911	OR4K13	protein_coding
ENSG00000176256.10	0.92925181	-0.523150623	0.435780612	-1.200490817	0.229948777		HMGB4	protein_coding
ENSG00000176261.15	1462.877318	-0.145510657	0.053445871	-2.722579972	0.006477435	0.044584932	ZBTB8OS	protein_coding
ENSG00000176268.5	17.48253188	-0.14244402	0.122936339	-1.158681166	0.246586171	0.473056816	CYCSP34	processed_pseudogene
ENSG00000176269.3	0.210231855	0.147681535	0.601935045	0.245344636	0.806189593		OR4F21	protein_coding
ENSG00000176273.15	685.0664312	-0.324587841	0.092544324	-3.507377077	0.000452547	0.00631263	SLC35G1	protein_coding
ENSG00000176281.4	0.781245208	-0.873694281	0.561036332	-1.557286456	0.11940249		OR4K5	protein_coding
ENSG00000176289.4	5.2931142	0.224753238	0.163656367	1.37332413	0.169651604	0.379298546	IDSP1	unprocessed_pseudogene
ENSG00000176290.8	0.662968663	-0.105875592	0.515006004	-0.205581277	0.837118004		OR4K3	polymorphic_pseudogene
ENSG00000176293.20	127.6644472	-0.100095948	0.130252972	-0.768473427	0.442205972	0.658601101	ZNF135	protein_coding
ENSG00000176294.6	2.678328175	-0.251390842	0.395022349	-0.636396505	0.524518018	0.723663817	OR4N2	protein_coding
ENSG00000176299.5	1.171406488	0.141861034	0.461941679	0.307097283	0.758769326	0.876568815	OR4M1	protein_coding
ENSG00000176302.13	2.101809652	-0.006295822	0.276481542	-0.022771219	0.981832766	0.992068024	FOXR1	protein_coding
ENSG00000176312.4	0.258093609	-0.094578449	0.780170595	-0.121227907	0.90351052		OR4H12P	unprocessed_pseudogene
ENSG00000176318.8	2.251879168	-0.170134608	0.176910905	-0.961696554	0.336202054	0.56572873	FOXN3P1	processed_pseudogene
ENSG00000176320.2	16.81649079	-0.529722673	0.19847849	-2.66891729	0.007609619	0.049932894	AL138899.1	lncRNA
ENSG00000176340.4	6013.325343	-0.156385251	0.077980316	-2.00544521	0.044915473	0.166054259	COX8A	protein_coding
ENSG00000176343.5	18.67073709	-0.303131863	0.11082091	-2.735330922	0.006231759	0.04340121	RPL37AP8	processed_pseudogene
ENSG00000176349.11	8.344427546	0.433052835	0.15948068	2.715393713	0.006619702	0.045263888	AC104129.1	lncRNA
ENSG00000176358.15	16.05073721	0.015609704	0.118713057	0.131491041	0.895386873	0.9503903	TAC4	protein_coding
ENSG00000176371.14	297.183574	0.076186759	0.064652052	1.178412074	0.238632367	0.464049384	ZSCAN2	protein_coding
ENSG00000176378.8	0.119299484	-0.278378994	0.787663095	-0.35342394	0.723770641		PFN1P10	processed_pseudogene
ENSG00000176381.6	28.9797342	0.821870154	0.254440186	3.230111433	0.00123742	0.013507776	PRR18	protein_coding
ENSG00000176383.9	75.06735668	0.273544901	0.137328531	1.991901456	0.046381871	0.16953387	B3GNT4	protein_coding
ENSG00000176386.9	488.8752928	-0.000875934	0.048187184	-0.018177731	0.985497068	0.993310157	CDC26	protein_coding
ENSG00000176387.7	2350.010316	-0.485075823	0.123853998	-3.91651324	8.98389E-05	0.001825132	HSD11B2	protein_coding
ENSG00000176390.12	1136.332308	-0.083178983	0.045505064	-1.827906065	0.067563651	0.215079349	CRLF3	protein_coding

ENSG00000176393.11	5709.145061	0.046608123	0.06128426	0.760523552	0.446941696	0.66237897	RNPEP	protein_coding
ENSG00000176396.11	688.2207396	-0.055344974	0.054926992	-1.007609764	0.31364184	0.542963896	EID2	protein_coding
ENSG00000176399.4	98.61686084	0.389012264	0.207576923	1.874063158	0.060921721	0.201670147	DMRTA1	protein_coding
ENSG00000176401.6	50.46626853	-0.041229365	0.086103117	-0.478837085	0.632054532	0.800245203	EID2B	protein_coding
ENSG00000176402.6	18.34005807	0.431516465	0.162274298	2.659179369	0.007833125	0.051000752	GJC3	protein_coding
ENSG00000176406.23	62.29334897	0.166222996	0.204810069	0.81159582	0.41702359	0.637390375	RIMS2	protein_coding
ENSG00000176407.18	4060.176048	0.031826073	0.039755513	0.800544887	0.423395168	0.642460233	KCMF1	protein_coding
ENSG00000176410.8	725.1751597	-0.202996506	0.053749834	-3.77669088	0.000158926	0.002851973	DNAJC30	protein_coding
ENSG00000176422.14	921.4113777	-0.087188237	0.059702644	-1.460374805	0.144187093	0.343665753	SPRYD4	protein_coding
ENSG00000176428.6	132.6513767	-0.11595535	0.125724066	-0.922300345	0.356371934	0.584333886	VPS37D	protein_coding
ENSG00000176435.7	1184.241313	-0.043615559	0.093717158	-0.465395666	0.641648137	0.80612065	CLEC14A	protein_coding
ENSG00000176438.12	756.5093088	-0.21583217	0.10700856	-2.016961726	0.043699508	0.163066745	SYNE3	protein_coding
ENSG00000176444.19	2541.886353	0.113544281	0.049572366	2.29047534	0.021993777	0.103512561	CLK2	protein_coding
ENSG00000176454.14	4334.869873	0.117194448	0.085026658	1.378325931	0.16810268	0.377249228	LPCAT4	protein_coding
ENSG00000176463.14	2119.462268	0.208657515	0.104810584	1.99080577	0.046502245	0.169788715	SLCO3A1	protein_coding
ENSG00000176472.10	232.8982359	-0.085821439	0.067042189	-1.280110941	0.200506121	0.419008932	ZNF575	protein_coding
ENSG00000176473.13	462.5702755	0.163115682	0.059396918	2.74619775	0.006029041	0.04247131	WDR25	protein_coding
ENSG00000176476.9	817.9333672	-0.197832155	0.061064662	-3.239715879	0.001196489	0.013169962	SGF29	protein_coding
ENSG00000176485.12	4061.806314	0.322644617	0.121962361	2.645444165	0.008158375	0.052467165	PLAAT3	protein_coding
ENSG00000176490.5	131.7690466	0.22764502	0.18512129	1.229707398	0.218806696	0.440412904	DIRAS1	protein_coding
ENSG00000176495.3	1.870955449	0.594168895	0.365350712	1.62629735	0.103886415	0.281773528	OR5AN1	protein_coding
ENSG00000176510.5	8.146524864	0.224206392	0.204992735	1.09372848	0.27407408	0.502598529	OR10AC1	polymorphic_pseudogene
ENSG00000176515.1	12.73603637	0.010737102	0.257053651	0.041769886	0.966682141	0.984499865	AL033381.1	lncRNA
ENSG00000176531.10	676.108712	0.089092733	0.087544313	1.017687273	0.308826597	0.538212042	PHLDB3	protein_coding
ENSG00000176532.4	3163.408957	-0.148349135	0.10543079	-1.40707601	0.159404851	0.365274503	PRR15	protein_coding
ENSG00000176533.13	478.3679028	-0.486257673	0.134204977	-3.623246195	0.000290929	0.004544014	GNG7	protein_coding
ENSG00000176540.3	0.991259761	-0.466307093	0.471299708	-0.98940671	0.322464193	0.552351546	OR4C5	protein_coding
ENSG00000176542.10	3037.434439	0.076842963	0.056816071	1.352486395	0.176219768	0.388334948	USF3	protein_coding
ENSG00000176547.10	0.524272344	0.008174248	0.567314649	0.014408667	0.988503945		OR4C3	protein_coding
ENSG00000176555.1	0.279046869	0.198832797	0.688255697	0.28889379	0.772662656		OR4S1	protein_coding
ENSG00000176563.10	100.1497349	-0.317620482	0.115525135	-2.749362562	0.00597113	0.042186109	CNTD1	protein_coding
ENSG00000176566.5	6.276325935	1.179415099	0.47122836	2.502852544	0.012319686	0.069793936	DCAF4L2	protein_coding
ENSG00000176567.1	0.467894038	0.076134596	0.592067388	0.128591099	0.89768121		OR4X1	protein_coding
ENSG00000176571.12	3.674292337	0.40731053	0.331756865	1.227738061	0.219545314	0.441389902	CNBD1	protein_coding
ENSG00000176584.18	4.527026052	0.633317652	0.261526826	2.421616407	0.015451651	0.08167596	DMBT1L1	transcribed_unitary_pseudogene
ENSG00000176593.8	170.1376265	-0.215558544	0.146417405	-1.472219399	0.14096164	0.338850695	AC008969.1	lncRNA
ENSG00000176595.4	1647.136554	0.148968783	0.129652688	1.148983372	0.250562837	0.477350038	KBTBD11	protein_coding
ENSG00000176597.12	2261.240188	0.247899746	0.081496095	3.041860419	0.002351209	0.021466296	B3GNT5	protein_coding
ENSG00000176601.13	21.33458397	-0.32344937	0.19305811	-1.675399028	0.093855967	0.264759158	MAP3K19	protein_coding
ENSG00000176605.9	1.272269466	0.052767196	0.455136486	0.115937083	0.907702406	0.956333677	C14orf177	lncRNA
ENSG00000176619.13	7436.408228	0.069126605	0.073981613	0.934375482	0.350110241	0.579201775	LMNB2	protein_coding
ENSG00000176623.12	2575.54014	-0.112378569	0.059943219	-1.87475031	0.060827082	0.201509941	RMDN1	protein_coding

ENSG00000176624.12	2427.777172	0.04116707	0.05823034	0.706969434	0.479585473	0.688864457	MEX3C	protein_coding
ENSG00000176635.18	4.246018893	-0.529281399	0.324514446	-1.630994874	0.102891401	0.280301237	HORMAD2	protein_coding
ENSG00000176641.11	923.5416952	0.242674271	0.093432139	2.597331856	0.009395111	0.057820382	RNF152	protein_coding
ENSG00000176654.12	8.731747593	-0.604525274	0.295433865	-2.046228772	0.040733868	0.155734318	NANOGP1	transcribed_unprocessed_pseudogene
ENSG00000176658.17	12731.2859	0.221410342	0.074461677	2.97348048	0.002944431	0.025292202	MYO1D	protein_coding
ENSG00000176659.9	11.36906422	0.059815823	0.13694103	0.436799865	0.662256497	0.819107943	C20orf197	lncRNA
ENSG00000176678.6	434.2890221	0.715479364	0.117365899	6.096143495	1.08658E-09	1.917E-07	FOXL1	protein_coding
ENSG00000176679.9	0.32915556	0.198375943	0.672444093	0.29500734	0.767988273		TGIF2LY	protein_coding
ENSG00000176681.14	52.38036429	-0.042445898	0.092205445	-0.460340477	0.645271852	0.80857521	LRRC37A	protein_coding
ENSG00000176692.8	172.5256378	0.896064936	0.148009271	6.054113581	1.41193E-09	2.38479E-07	FOXC2	protein_coding
ENSG00000176695.8	0.377071255	-0.245801313	0.534880038	-0.459544749	0.645843025		OR4F17	protein_coding
ENSG00000176697.20	90.52394688	0.712005993	0.134259977	5.303188682	1.13797E-07	8.91331E-06	BDNF	protein_coding
ENSG00000176700.20	130.0103118	-0.027612754	0.075833632	-0.364122792	0.715766318	0.851497034	SCAND2P	transcribed_unprocessed_pseudogene
ENSG00000176714.10	193.4123407	-0.153147288	0.05960236	-2.569483624	0.010185021	0.061225622	CCDC121	protein_coding
ENSG00000176715.17	1578.867416	-0.084477989	0.056789123	-1.487573399	0.136863433	0.332985195	ACSF3	protein_coding
ENSG00000176716.5	1.819311314	-0.309373047	0.215641706	-1.4346624	0.151383324	0.353823485	OR10AB1P	transcribed_unprocessed_pseudogene
ENSG00000176720.6	2575.339278	0.12465642	0.087394827	1.42635925	0.15376467	0.357502307	BOK	protein_coding
ENSG00000176723.10	59.22038894	0.18821217	0.108891309	1.72844069	0.083909246	0.247350092	ZNF843	protein_coding
ENSG00000176728.10	137.4866224	0.027061256	0.245192116	0.110367563	0.912117877	0.958582856	TTTY14	lncRNA
ENSG00000176731.12	1031.447631	-0.24253395	0.067086592	-3.615237286	0.000300073	0.00464356	RBIS	protein_coding
ENSG00000176732.7	31.2027659	-0.110175361	0.115849723	-0.951019635	0.341594409	0.571448406	PFN4	protein_coding
ENSG00000176742.3	0.884268894	0.277275148	0.480495309	0.577061092	0.563898187		OR51V1	protein_coding
ENSG00000176746.6	1.855601027	0.631835984	0.404380046	1.562480615	0.118174809	0.304409981	MAGEB6	protein_coding
ENSG00000176748.6	0.349842605	-0.250484823	0.831851524	-0.301117226	0.763325106		OR52Z1	unprocessed_pseudogene
ENSG00000176749.9	260.8295178	-0.127747492	0.106639918	-1.197933143	0.230943046	0.454922932	CDK5R1	protein_coding
ENSG00000176752.6	0.431498364	0.441903369	0.592666148	0.745619386	0.455897369		OR51A1P	unprocessed_pseudogene
ENSG00000176753.5	18.35765705	0.457393598	0.1315265	3.477577502	0.000505967	0.006851697	C15orf56	lncRNA
ENSG00000176754.13	4.084062166	-0.246154705	0.266195497	-0.924714008	0.355114691	0.583088069	LINC00303	lncRNA
ENSG00000176761.7	10.58395957	0.034954504	0.135165978	0.258604311	0.795940557	0.897879624	ZNF285B	unprocessed_pseudogene
ENSG00000176769.9	9.001625772	0.175515154	0.213002331	0.824005788	0.409936269	0.630872751	TCERG1L	protein_coding
ENSG00000176771.17	226.1174644	-0.015139993	0.129989338	-0.116471039	0.907279237	0.956073497	NCKAP5	protein_coding
ENSG00000176774.6	1.431140731	0.212673026	0.42669612	0.498417999	0.618189453	0.79087669	MAGEB18	protein_coding
ENSG00000176782.3	0.056645114	-0.15403913	1.605920793	-0.095919506	0.923584502		DEFB104A	protein_coding
ENSG00000176783.15	2187.297097	-0.015345545	0.039535665	-0.388144351	0.697909212	0.840875629	RUFY1	protein_coding
ENSG00000176787.2	0.5627216	0.236209637	0.54439979	0.433890021	0.664368306		OR52E2	protein_coding
ENSG00000176788.9	1932.522991	0.119439117	0.114767752	1.040702762	0.298013521	0.527883203	BASP1	protein_coding
ENSG00000176797.4	0.121875276	-0.093630993	0.912953122	-0.10255838	0.918313477		DEFB103A	protein_coding
ENSG00000176798.3	1.647839154	0.527931791	0.393603103	1.341279546	0.179829708	0.392832485	OR51L1	protein_coding
ENSG00000176809.10	249.6391326	0.221952546	0.08600184	2.580788356	0.009857499	0.059810673	LRRC37A3	protein_coding
ENSG00000176812.7	1.539896803	-0.285962424	0.200060654	-1.429378629	0.152895437	0.356238276	METTL1P1	processed_pseudogene
ENSG00000176826.15	201.3866567	0.279410868	0.131511175	2.124616919	0.033618591	0.137159193	FKBP9P1	transcribed_unprocessed_pseudogene
ENSG00000176834.14	2186.384516	-0.189721737	0.066933538	-2.834479431	0.004590041	0.035023679	VSIG10	protein_coding

ENSG00000176840.12	10.70491395	-0.98093214	0.206486667	-4.750583431	2.02831E-06	9.12152E-05	MIR7-3HG	lncRNA
ENSG00000176842.15	267.4399901	0.079480339	0.178801224	0.44451787	0.656668212	0.815516853	IRX5	protein_coding
ENSG00000176845.13	2209.059408	0.262404558	0.078002826	3.364039108	0.000768107	0.009423362	METRNL	protein_coding
ENSG00000176853.16	6309.682671	0.117035924	0.063848647	1.833021198	0.066799419	0.213528023	FAM91A1	protein_coding
ENSG00000176855.11	24.93822841	-0.046102687	0.115042423	-0.400745101	0.688607802	0.835577433	KRT18P28	processed_pseudogene
ENSG00000176857.5	6.405082868	0.148261679	0.1289657	1.149621013	0.2503	0.477073135	GJA1P1	processed_pseudogene
ENSG00000176868.2	0						AL358781.1	lncRNA
ENSG00000176871.9	5038.997785	0.051866022	0.054701317	0.948167713	0.343044081	0.572818022	WSB2	protein_coding
ENSG00000176882.5	0.589278058	-0.062323677	0.400917662	-0.15545256	0.876464551		AL135901.1	processed_pseudogene
ENSG00000176884.16	187.0685512	-0.101912862	0.158570195	-0.642698725	0.520419608	0.720588324	GRIN1	protein_coding
ENSG00000176887.7	100.4841329	0.784078698	0.155282688	5.04936325	4.43285E-07	2.70547E-05	SOX11	protein_coding
ENSG00000176890.16	722.8423501	-0.171260405	0.084693432	-2.022121453	0.043163806	0.161871703	TYMS	protein_coding
ENSG00000176893.5	1.194794596	0.176903428	0.485826908	0.36412851	0.715762048	0.851497034	OR51G2	protein_coding
ENSG00000176894.10	173.7857911	-0.323215311	0.086344722	-3.743312913	0.00018161	0.003168139	PXMP2	protein_coding
ENSG00000176895.9	1.928224682	-0.14327727	0.464360688	-0.308547373	0.757665859	0.875823787	OR51A7	protein_coding
ENSG00000176896.8	186.1352878	-0.217398656	0.057129715	-3.805351703	0.000141603	0.002599253	TCEANC	protein_coding
ENSG00000176900.2	0.354344024	-0.190602581	0.655294954	-0.290865326	0.771154321		OR51T1	protein_coding
ENSG00000176903.5	3041.343219	0.086126215	0.084985831	1.013418523	0.310860296	0.540341408	PNMA1	protein_coding
ENSG00000176904.3	0.301467753	-0.079413247	0.764494582	-0.103876795	0.917267122		OR51H1	transcribed_unprocessed_pseudogene
ENSG00000176907.5	2490.597166	0.006097336	0.131200778	0.046473324	0.962932996	0.982871458	TCIM	protein_coding
ENSG00000176909.12	169.8560551	-0.019354064	0.109285184	-0.177096874	0.859432297	0.931582539	MAMSTR	protein_coding
ENSG00000176912.5	68.09658693	-0.284559327	0.112267382	-2.534657203	0.011255741	0.065608806	TYMSOS	lncRNA
ENSG00000176915.15	4843.249468	0.099495114	0.04708339	2.113168012	0.03458639	0.139708113	ANKLE2	protein_coding
ENSG00000176919.13	182.4837124	-0.255510033	0.196853951	-1.297967511	0.194298499	0.411036735	C8G	protein_coding
ENSG00000176920.13	4634.362894	-0.227134249	0.111342446	-2.03996101	0.041354209	0.157237346	FUT2	protein_coding
ENSG00000176922.4	0.604589362	0.207781315	0.676033364	0.30735364	0.758574211		OR51S1	protein_coding
ENSG00000176923.5	2.671564209	-0.166493272	0.37103546	-0.448726039	0.653629298	0.813873262	OR7A15P	unprocessed_pseudogene
ENSG00000176925.8	0.718724489	-0.111185115	0.515977919	-0.215484251	0.82938979		OR51F2	protein_coding
ENSG00000176927.15	42.81881088	-0.207358593	0.131186513	-1.580639567	0.113960473	0.297743003	EFCAB5	protein_coding
ENSG00000176928.7	538.9731739	-0.960864228	0.154362519	-6.224724989	4.82401E-10	9.77749E-08	GCNT4	protein_coding
ENSG00000176933.5	36.2889842	0.467962682	0.134963468	3.467328516	0.000525659	0.00706559	TOB2P1	processed_pseudogene
ENSG00000176945.17	2633.97014	0.404284045	0.172202791	2.347720634	0.018888683	0.093345861	MUC20	protein_coding
ENSG00000176946.12	3600.180074	-0.027418605	0.053454804	-0.512930603	0.607999865	0.784088653	THAP4	protein_coding
ENSG00000176951.6	1.468764608	0.107134174	0.40322759	0.265691577	0.790476751	0.89480999	OR51N1P	unprocessed_pseudogene
ENSG00000176953.12	2699.99022	-0.017297613	0.044023606	-0.392916765	0.694380959	0.838711654	NFATC2IP	protein_coding
ENSG00000176956.13	43.05097008	0.212863238	0.16545128	1.286561445	0.198247189	0.415757148	LY6H	protein_coding
ENSG00000176970.8	1.378267273	0.48881209	0.210852334	2.318267388	0.020434794	0.098592449	RPL7L1P11	processed_pseudogene
ENSG00000176971.4	577.2297627	0.40871095	0.128862934	3.171671936	0.001515641	0.015643557	FIBIN	protein_coding
ENSG00000176973.8	305.9860106	-0.032350394	0.085687979	-0.377537134	0.705774473	0.845813279	FAM89B	protein_coding
ENSG00000176974.20	1906.169765	-0.105429113	0.074009826	-1.424528591	0.154293515	0.35829258	SHMT1	protein_coding
ENSG00000176978.14	6088.150127	-0.162725549	0.077878261	-2.089486174	0.036663981	0.145192379	DPP7	protein_coding
ENSG00000176979.14	2.854421659	-0.46285278	0.380474259	-1.216515361	0.223788663	0.446542358	TRIM60	protein_coding

ENSG00000176984.6	4.343393252	0.661880738	0.196183991	3.373775468	0.000741448	0.009158452	AP000679.1	lncRNA
ENSG00000176986.16	4479.373594	0.067934598	0.069235509	0.981210354	0.326489019	0.556264821	SEC24C	protein_coding
ENSG00000176988.9	1.922366601	1.750226783	0.391048727	4.475725557	7.61523E-06	0.000262406	FMR1NB	protein_coding
ENSG00000176994.11	3291.478688	0.049001314	0.059370556	0.825347061	0.409174581	0.6300343	SMCR8	protein_coding
ENSG00000176998.4	0.818746369	1.111440575	0.339102243	3.277597233	0.001046947		HCG4	unprocessed_pseudogene
ENSG00000177000.13	2075.366855	0.086025215	0.062538352	1.375559343	0.168958101	0.378280673	MTHFR	protein_coding
ENSG00000177023.2	0.038246459	-0.123182604	2.372338739	-0.051924543	0.958588818		DEFB104B	protein_coding
ENSG00000177025.3	29.45983786	-0.310401721	0.164598435	-1.885812107	0.059320279	0.198475097	C19orf18	protein_coding
ENSG00000177030.17	1772.168119	-0.087390283	0.055341453	-1.579110747	0.114310657	0.298318092	DEAF1	protein_coding
ENSG00000177034.17	1102.295581	0.042953994	0.081079052	0.529779188	0.596265037	0.775764062	MTX3	protein_coding
ENSG00000177042.14	1063.281621	0.005795907	0.063355914	0.091481698	0.927109848	0.965552934	TMEM80	protein_coding
ENSG00000177045.10	498.667209	0.247155851	0.079687263	3.101572853	0.001924955	0.018607756	SIX5	protein_coding
ENSG00000177047.6	0.962175094	0.002636656	0.306120275	0.008613136	0.993127796		IFNW1	protein_coding
ENSG00000177051.6	2090.773336	-0.005518555	0.057893041	-0.095323292	0.924058042	0.96412578	FBXO46	protein_coding
ENSG00000177054.14	1886.431592	0.028909618	0.060726443	0.476063085	0.634029435	0.801405446	ZDHHC13	protein_coding
ENSG00000177058.12	745.9932885	-0.005105159	0.054845412	-0.093082702	0.92583786	0.964911543	SLC38A9	protein_coding
ENSG00000177076.6	1001.702744	-0.188374033	0.151424586	-1.244012202	0.213495072	0.434214261	ACER2	protein_coding
ENSG00000177082.13	297.719334	0.039769371	0.050175859	0.792599707	0.428011079	0.646258596	WDR73	protein_coding
ENSG00000177084.18	4202.919244	-0.121913336	0.063803837	-1.910752417	0.056036405	0.19137135	POLE	protein_coding
ENSG00000177096.9	1070.579516	0.137781464	0.068941853	1.998516971	0.045660642	0.167883378	PHETA2	protein_coding
ENSG00000177098.9	265.5039768	0.044188941	0.149619602	0.295341925	0.767732692	0.882241701	SCN4B	protein_coding
ENSG00000177103.15	99.65558655	1.386315585	0.20824277	6.657208731	2.79077E-11	9.28346E-09	DSCAML1	protein_coding
ENSG00000177105.10	5163.696103	-0.045152975	0.056120707	-0.804568895	0.421068498	0.640718693	RHOG	protein_coding
ENSG00000177106.16	6193.668052	0.165607335	0.087968112	1.882583716	0.059756805	0.199404417	EPS8L2	protein_coding
ENSG00000177108.5	4.321717161	-0.29065498	0.249935625	-1.162919369	0.244862218	0.471160016	ZDHHC22	protein_coding
ENSG00000177112.8	8.952017427	-0.065377536	0.147827886	-0.442254418	0.658305111	0.816640908	IRAG1-AS1	lncRNA
ENSG00000177119.16	6507.778964	-0.044235705	0.066947958	-0.660747635	0.50877417	0.712293645	ANO6	protein_coding
ENSG00000177125.5	948.2148077	0.11262752	0.052738743	2.135574602	0.032714103	0.134934452	ZBTB34	protein_coding
ENSG00000177133.11	328.6756344	-0.480811839	0.17046211	-2.820637621	0.004792831	0.036165683	PRDM16-DT	lncRNA
ENSG00000177138.17	4.323251245	0.293611678	0.254880588	1.151957787	0.24933842	0.476052416	FAM9B	protein_coding
ENSG00000177143.5	0.777231508	-0.501099553	0.488987577	-1.024769496	0.305471963		CETN1	protein_coding
ENSG00000177144.7	4.253506343	-0.193697177	0.140071792	-1.382842139	0.166713275	0.375270643	NUDT4B	protein_coding
ENSG00000177150.13	956.3223955	0.055416932	0.056089597	0.988007298	0.323149074	0.552941756	FAM210A	protein_coding
ENSG00000177151.5	0.18155498	-0.033913789	0.559571244	-0.06060674	0.951672405		OR2T35	protein_coding
ENSG00000177156.11	10777.66856	-0.13819379	0.06002273	-2.302357614	0.021315013	0.101413291	TALDO1	protein_coding
ENSG00000177169.10	3485.189445	0.264299394	0.063137812	4.186071458	2.83824E-05	0.000730981	ULK1	protein_coding
ENSG00000177173.5	38.71028791	-0.209002106	0.124791874	-1.674805412	0.093972417	0.264879841	NAP1L4P1	processed_pseudogene
ENSG00000177174.1	0.184517756	0.013967457	1.068948517	0.013066538	0.989574707		OR14C36	protein_coding
ENSG00000177181.15	648.1304134	-0.025643184	0.141248955	-0.181546008	0.855939024	0.929727675	RIMKLA	protein_coding
ENSG00000177182.11	21.96825624	-0.475318387	0.175574987	-2.70721015	0.006785129	0.046033874	CLVS1	protein_coding
ENSG00000177186.3	0.856344962	-0.693216667	0.49198198	-1.409028573	0.158826719		OR2M7	protein_coding
ENSG00000177189.14	6568.667591	-0.017752284	0.061472034	-0.288786354	0.772744875	0.885000426	RPS6KA3	protein_coding

ENSG00000177191.2	415.3779591	-0.135686285	0.139368806	-0.97357715	0.330266536	0.559845465	B3GNT8	protein_coding
ENSG00000177192.14	1396.447351	-0.050148315	0.062193715	-0.80632449	0.420055773	0.639915064	PUS1	protein_coding
ENSG00000177197.7	11.63923351	-0.086505663	0.083176187	-1.040029189	0.29832634	0.528118578	PCNPP5	processed_pseudogene
ENSG00000177200.17	3622.031848	-0.036052494	0.058419955	-0.617126347	0.537151391	0.73329301	CHD9	protein_coding
ENSG00000177201.2	1.057647169	0.437814983	0.465074872	0.941386021	0.346507075	0.575908265	OR2T12	protein_coding
ENSG00000177202.3	114.6428065	-0.023459398	0.133588652	-0.175609217	0.860600961	0.93222468	SPACA4	protein_coding
ENSG00000177212.4	0.762791853	0.387086332	0.462750023	0.83649122	0.402878614		OR2T33	protein_coding
ENSG00000177225.17	3126.871103	-0.035288564	0.064484576	-0.547240386	0.584213594	0.767080364	GATD1	protein_coding
ENSG00000177233.2	0.300554712	-0.280541141	0.721674792	-0.388736234	0.697471275		OR2M1P	unprocessed_pseudogene
ENSG00000177234.7	8.681525812	0.943801341	0.218017018	4.329026008	1.4977E-05	0.000445993	LINC01561	lncRNA
ENSG00000177238.14	155.2456809	1.478095546	0.230376138	6.41600974	1.39893E-10	3.58594E-08	TRIM72	protein_coding
ENSG00000177239.15	3564.468718	0.121055893	0.053091239	2.280148191	0.022598901	0.105562472	MAN1B1	protein_coding
ENSG00000177243.3	0.180452622	-0.094123054	0.673621623	-0.139726889	0.88887578		DEFB103B	protein_coding
ENSG00000177257.3	1.150946722	-1.277945351	0.508659224	-2.512380179	0.011991982	0.068476639	DEFB4B	protein_coding
ENSG00000177261.8	0.306319625	0.026258393	0.423528194	0.061999162	0.950563499		ENSAP3	processed_pseudogene
ENSG00000177272.9	200.3350791	-0.742261864	0.157295172	-4.718910661	2.37111E-06	0.000103349	KCNA3	protein_coding
ENSG00000177275.4	0.869091936	-0.643606297	0.485366547	-1.32602113	0.184832699		OR2AJ1	protein_coding
ENSG00000177283.7	1184.068514	0.32425185	0.118608583	2.733797528	0.006260853	0.043568563	FZD8	protein_coding
ENSG00000177291.4	3.341342842	-0.054973024	0.182624318	-0.301016997	0.763401533	0.879281826	GJD4	protein_coding
ENSG00000177294.7	29.55468273	0.661915545	0.153394206	4.315127433	1.59511E-05	0.000466824	FBXO39	protein_coding
ENSG00000177300.7	0						CLDN22	protein_coding
ENSG00000177301.16	123.1518014	-0.529176121	0.184376617	-2.870082605	0.004103646	0.032331328	KCNA2	protein_coding
ENSG00000177302.15	2130.546832	-0.025342242	0.051934973	-0.487961007	0.625577461	0.795772806	TOP3A	protein_coding
ENSG00000177303.10	2911.734134	0.076775078	0.065115645	1.179057326	0.23837535	0.463801181	CASKIN2	protein_coding
ENSG00000177306.4	0.023370921	-0.200002945	2.291896187	-0.087265272	0.930460657		OR7E125P	unprocessed_pseudogene
ENSG00000177311.11	6626.116152	0.059456027	0.065135668	0.912802906	0.36134621	0.589146301	ZBTB38	protein_coding
ENSG00000177324.14	4.004835633	-0.317670673	0.284732464	-1.115681255	0.264558597	0.492345438	BEND2	protein_coding
ENSG00000177335.11	328.5823802	0.482206705	0.167582675	2.877425758	0.004009342	0.031824247	C8orf31	lncRNA
ENSG00000177337.8	468.7955054	-0.035021131	0.088869231	-0.394074877	0.693525759	0.838143324	DLGAP1-AS1	lncRNA
ENSG00000177338.14	2.00030035	0.497889399	0.330066787	1.508450467	0.131439275	0.324793268	LINC00469	lncRNA
ENSG00000177340.5	13.82023981	0.180000372	0.106678249	1.687320266	0.091541786	0.260828849	FLJ13224	lncRNA
ENSG00000177350.6	48.61101861	-0.361638595	0.111898895	-3.231833485	0.001229987	0.01344467	RPL13AP3	transcribed_processed_pseudogene
ENSG00000177352.10	1013.881144	0.0782404	0.052219136	1.498308974	0.134052994	0.328908845	CCDC71	protein_coding
ENSG00000177354.12	3.754739823	0.128448639	0.237197182	0.541526834	0.588144507	0.770026836	C10orf71	protein_coding
ENSG00000177359.20	101.4248484	0.555191652	0.182317446	3.045192131	0.002325317	0.021283416	AC024940.1	transcribed_unprocessed_pseudogene
ENSG00000177363.5	263.2198724	0.33171055	0.141437906	2.34527334	0.019013135	0.09375754	LRRN4CL	protein_coding
ENSG00000177369.9	20.03207682	-0.425008101	0.164100111	-2.589931827	0.009599494	0.058707383	FLJ40194	lncRNA
ENSG00000177370.5	1087.345568	-0.118851005	0.060895315	-1.951726591	0.050970674	0.180141854	TIMM22	protein_coding
ENSG00000177374.13	1634.85749	0.31711014	0.110439387	2.871350067	0.004087226	0.032260513	HIC1	protein_coding
ENSG00000177380.14	1074.840512	-0.159743368	0.095188446	-1.678180215	0.093311923	0.264018792	PPFIA3	protein_coding
ENSG00000177383.5	2441.708398	0.121352146	0.071480281	1.697701025	0.089564204	0.257452734	MAGEF1	protein_coding
ENSG00000177398.18	23.26119367	0.050656359	0.180873891	0.280064516	0.779428008	0.888395435	UMODL1	protein_coding

ENSG00000177400.6	0.362157571	0.063257506	0.463124119	0.136588667	0.891355935		OR7E8P	unprocessed_pseudogene
ENSG00000177406.5	384.8410004	0.05899594	0.089316898	0.660523833	0.508917729	0.712448853	NINJ2-AS1	lncRNA
ENSG00000177409.12	3742.530026	0.028322939	0.125038328	0.226514061	0.820801625	0.91184603	SAMD9L	protein_coding
ENSG00000177410.13	2613.644684	-0.279835126	0.093857923	-2.981475807	0.002868627	0.024812657	ZFAS1	lncRNA
ENSG00000177414.13	8.230052164	-0.157295941	0.310656949	-0.506333244	0.612622729	0.787221652	UBE2U	protein_coding
ENSG00000177418.2	5.676861133	0.080455156	0.125189536	0.642666783	0.520440339	0.720588324	AC006001.1	processed_pseudogene
ENSG00000177425.11	3974.651382	0.058623945	0.05583792	1.049894845	0.293766462	0.523834238	PAWR	protein_coding
ENSG00000177426.22	5031.767076	0.066317832	0.060379751	1.098345576	0.272053617	0.500254332	TGIF1	protein_coding
ENSG00000177427.13	623.5777937	0.104797817	0.05375674	1.949482363	0.051237847	0.180705055	MIEF2	protein_coding
ENSG00000177432.8	236.9983842	0.034504334	0.100049743	0.344871793	0.730190757	0.859563394	NAP1L5	protein_coding
ENSG00000177447.6	3.123242127	0.005215832	0.118388348	0.044056973	0.96485899	0.984062284	CBX3P1	processed_pseudogene
ENSG00000177452.7	5.299226785	-0.260722468	0.136215553	-1.914043306	0.055614623	0.190363554	AL161742.1	processed_pseudogene
ENSG00000177453.7	37.77543723	-0.112249946	0.107838995	-1.040903122	0.297920513	0.527829004	NIM1K	protein_coding
ENSG00000177455.15	264.8293845	-0.980940364	0.176625452	-5.553788289	2.79544E-08	2.82638E-06	CD19	protein_coding
ENSG00000177459.11	230.5533622	0.334003254	0.188138614	1.77530411	0.075847687	0.231863533	ERICH5	protein_coding
ENSG00000177462.7	0.77753214	0.218344573	0.408225211	0.534863031	0.592744574		OR2T8	protein_coding
ENSG00000177463.15	3020.494985	0.073262412	0.054206099	1.351552939	0.176518374	0.388654903	NR2C2	protein_coding
ENSG00000177464.5	700.1865944	0.093152655	0.082877435	1.123980913	0.261021123	0.488179936	GPR4	protein_coding
ENSG00000177465.4	282.8425992	0.19027346	0.103378723	1.840547589	0.065687884	0.211212447	ACOT4	protein_coding
ENSG00000177468.7	2.309785714	-0.035089055	0.437406927	-0.080220621	0.93606179	0.969767071	OLIG3	protein_coding
ENSG00000177469.13	12683.14545	0.184184698	0.118227514	1.557883541	0.119260858	0.30627525	CAVIN1	protein_coding
ENSG00000177476.3	1.19101763	0.057716512	0.40775526	0.141546947	0.887437875	0.94618631	OR2G3	protein_coding
ENSG00000177479.20	3019.559519	-0.00612187	0.039183212	-0.156237064	0.875846163	0.940021384	ARIH2	protein_coding
ENSG00000177483.11	47.40636263	0.031206682	0.117798393	0.264916025	0.791074153	0.895142757	RBM44	protein_coding
ENSG00000177485.7	2720.71625	0.012519034	0.056125763	0.223053244	0.823494068	0.913038715	ZBTB33	protein_coding
ENSG00000177489.1	0.708818213	-0.341296395	0.47963032	-0.711582192	0.476723538		OR2G2	protein_coding
ENSG00000177494.6	89.15116377	0.176960156	0.164108441	1.078312336	0.280894394	0.509788638	ZBED2	protein_coding
ENSG00000177504.10	2.949219701	-1.584739521	0.673968379	-2.351355895	0.018705133	0.092773725	VCX2	protein_coding
ENSG00000177508.12	248.0006534	0.499283138	0.197203435	2.531817649	0.011347299	0.065992845	IRX3	protein_coding
ENSG00000177511.6	11.23484348	-0.263026761	0.253667775	-1.036894658	0.299784954	0.529481071	ST8SIA3	protein_coding
ENSG00000177519.4	42.52091824	0.126274481	0.234370609	0.538781212	0.590037829	0.771184982	RPRM	protein_coding
ENSG00000177535.8	8.294607464	0.380906467	0.184285938	2.066931804	0.03874058	0.150961126	OR2B11	protein_coding
ENSG00000177542.11	2780.15327	0.047382506	0.079582616	0.595387644	0.551584383	0.743701843	SLC25A22	protein_coding
ENSG00000177548.13	1374.854791	-0.067211838	0.059745891	-1.124961684	0.260605271	0.487802696	RABEP2	protein_coding
ENSG00000177551.5	21.92108853	-0.096638863	0.276637163	-0.349334347	0.726838315	0.857405952	NHLH2	protein_coding
ENSG00000177553.6	0.563256116	-0.596715712	0.411382493	-1.45051314	0.146915478		C1orf167-AS1	lncRNA
ENSG00000177556.12	2219.998772	-0.226468229	0.066724218	-3.394093439	0.000688562	0.008646704	ATOX1	protein_coding
ENSG00000177558.3	2.767311484	-0.614259832	0.233096033	-2.635222164	0.008408223	0.053496374	FAM187B	protein_coding
ENSG00000177565.18	10206.56971	0.192852022	0.059894603	3.219856409	0.001282548	0.013868388	TBL1XR1	protein_coding
ENSG00000177570.15	2322.578873	0.078000212	0.08750333	0.891397068	0.37271619	0.599150974	SAMD12	protein_coding
ENSG00000177575.13	1957.574952	0.1184358	0.141184777	0.838870893	0.401541763	0.623695175	CD163	protein_coding
ENSG00000177576.12	200.2786383	-0.120794908	0.066097311	-1.827531358	0.067619916	0.215195579	C18orf32	protein_coding

ENSG00000177586.6	0.076430471	-0.154612537	1.239441982	-0.124743666	0.900726487		OR7E149P	unprocessed_pseudogene
ENSG00000177590.7	2.612478312	-0.512298284	0.220296684	-2.325492491	0.020045641	0.097394235	GIMAP3P	processed_pseudogene
ENSG00000177595.18	1193.023339	-0.059869533	0.064216468	-0.932308086	0.35117733	0.580126077	PIDD1	protein_coding
ENSG00000177596.1	0.909995536	-0.187343027	0.420606817	-0.445411296	0.65602255		AL355390.1	lncRNA
ENSG00000177599.13	57.77499362	-0.040147831	0.093599793	-0.428930766	0.66797361	0.822713321	ZNF491	protein_coding
ENSG00000177600.9	44318.89636	-0.281994237	0.069925138	-4.032802028	5.51157E-05	0.001241229	RPLP2	protein_coding
ENSG00000177602.5	407.9873839	-0.014760755	0.089547178	-0.164837744	0.869071695	0.936510232	HASPIN	protein_coding
ENSG00000177606.8	14303.16024	0.030466051	0.087401399	0.348576238	0.72740747	0.857813414	JUN	protein_coding
ENSG00000177613.9	1866.317247	0.045423144	0.060396901	0.752077398	0.452004515	0.666520058	CSTF2T	protein_coding
ENSG00000177614.11	314.1964467	0.42701458	0.153356625	2.784454725	0.005361781	0.039039317	PGBD5	protein_coding
ENSG00000177627.11	5.812278531	0.240806339	0.199687178	1.205917883	0.227849154	0.451304241	C12orf54	protein_coding
ENSG00000177628.16	3732.143383	0.184279578	0.064368052	2.862904367	0.004197772	0.032782496	GBA	protein_coding
ENSG00000177640.16	223.7248056	-0.260219939	0.08493258	-3.063841211	0.002185149	0.020403054	CASC2	lncRNA
ENSG00000177646.19	1974.395855	-0.059706651	0.043351935	-1.377254585	0.168433551	0.377757532	ACAD9	protein_coding
ENSG00000177663.14	2867.624128	0.15291713	0.051522443	2.96797126	0.002997724	0.025644248	IL17RA	protein_coding
ENSG00000177666.17	6862.971308	0.261141463	0.06252848	4.176360338	2.9621E-05	0.000755293	PNPLA2	protein_coding
ENSG00000177669.4	20.29565801	-0.506684317	0.145417379	-3.48434499	0.000493343	0.006732983	MBOAT4	protein_coding
ENSG00000177673.4	2.008268417	0.238114782	0.299442089	0.795194767	0.426500216	0.645161321	TEX44	protein_coding
ENSG00000177674.16	2328.276975	0.233014457	0.072099755	3.231834254	0.001229984	0.01344467	AGTRAP	protein_coding
ENSG00000177675.9	383.9979601	-0.149004011	0.137702096	-1.082075112	0.279219154	0.507909577	CD163L1	protein_coding
ENSG00000177679.16	275.8945001	0.618972542	0.146692219	4.219532201	2.4481E-05	0.00064655	SRRM3	protein_coding
ENSG00000177683.14	1168.398717	-0.016832434	0.050968997	-0.330248488	0.741212212	0.866294879	THAP5	protein_coding
ENSG00000177684.4	0.129564401	0.090952238	1.226197441	0.074174219	0.940871759		DEFB114	protein_coding
ENSG00000177685.17	2295.342004	0.000898733	0.101506738	0.008853926	0.992935682	0.996732584	CRACR2B	protein_coding
ENSG00000177688.7	18.80972825	-0.032982427	0.090576933	-0.364137161	0.715755588	0.851497034	SUMO4	protein_coding
ENSG00000177689.10	1.005145645	-0.089259901	0.462447711	-0.193016202	0.846946279	0.925089684	MAGEB10	protein_coding
ENSG00000177692.12	69.31560985	-0.051661471	0.076528911	-0.675058227	0.499638772	0.704985065	DNAJC28	protein_coding
ENSG00000177693.4	0.141396629	-0.216823738	0.879923005	-0.246412171	0.805363186		OR4F4	protein_coding
ENSG00000177694.16	905.1386492	-0.188307988	0.119154675	-1.580365924	0.114023091	0.297870884	NAALADL2	protein_coding
ENSG00000177697.19	13053.92175	0.031850574	0.066853109	0.476426225	0.633770755	0.801364608	CD151	protein_coding
ENSG00000177699.4	13.41882449	-0.526700011	0.168423329	-3.127239044	0.001764564	0.017489563	AC011944.1	lncRNA
ENSG00000177700.6	4392.297792	-0.079580771	0.073155009	-1.087837623	0.276666797	0.505526815	POLR2L	protein_coding
ENSG00000177706.9	3791.739853	0.421524363	0.103178202	4.085401335	4.40007E-05	0.001041509	FAM20C	protein_coding
ENSG00000177707.11	2299.637058	-0.318993474	0.113624077	-2.807446107	0.004993603	0.037191092	NECTIN3	protein_coding
ENSG00000177710.6	15.3754125	-0.302966831	0.151878053	-1.994803229	0.046064345	0.168684535	SLC35G5	protein_coding
ENSG00000177721.4	158.9483816	0.155245821	0.096095662	1.615534123	0.106195077	0.285341156	ANXA2R	protein_coding
ENSG00000177725.5	5.087398459	-0.126067744	0.160931267	-0.783363896	0.433413451	0.651336563	AC105206.1	lncRNA
ENSG00000177728.17	5404.517462	0.056576943	0.057424794	0.985235452	0.32450843	0.554292249	TMEM94	protein_coding
ENSG00000177731.16	9996.84164	0.079143039	0.051864436	1.525959717	0.127019897	0.318221574	FLII	protein_coding
ENSG00000177732.8	2477.128799	-0.124367991	0.103569902	-1.200812096	0.229824099	0.45349777	SOX12	protein_coding
ENSG00000177733.7	9740.305367	-0.03142152	0.051670661	-0.60811144	0.543113565	0.737918493	HNRNPA0	protein_coding
ENSG00000177736.4	3.184498006	-0.072696234	0.143057749	-0.508160057	0.6113411	0.786529118	FXNP2	processed_pseudogene

ENSG00000177738.4	274.5731001	0.084116218	0.089386494	0.941039456	0.34668464	0.575961744	AC025171.1	lncRNA
ENSG00000177752.14	2.368226947	0.043803035	0.230714976	0.189857788	0.849420571	0.92625018	YIPF7	protein_coding
ENSG00000177757.2	12.71819344	0.048270746	0.088130814	0.547717012	0.583886229	0.766903278	FAM87B	lncRNA
ENSG00000177770.11	1.376328584	-0.175155145	0.188928372	-0.927098154	0.353875574	0.582147315	CDKN2AIPNLP1	processed_pseudogene
ENSG00000177776.8	11.08562664	-0.402476733	0.120526739	-3.33931489	0.000839853	0.010043435	RPS2P2	processed_pseudogene
ENSG00000177788.6	950.9223534	0.099997293	0.083457052	1.198188661	0.230843579	0.454803808	AL162595.1	lncRNA
ENSG00000177791.12	20.69431919	-0.165279544	0.139364167	-1.185954377	0.235640317	0.460553377	MYOZ1	protein_coding
ENSG00000177800.2	4.382140727	-0.199355804	0.17870681	-1.115546772	0.264616187	0.492406678	TMEM78	lncRNA
ENSG00000177803.7	7.428794793	0.102542938	0.110170273	0.930767755	0.351973713	0.580641264	YWHAQP4	processed_pseudogene
ENSG00000177807.10	142.9754482	-0.583272283	0.164324403	-3.549517131	0.000385938	0.005591102	KCNJ10	protein_coding
ENSG00000177822.8	31.64486057	0.926946116	0.195588369	4.739270129	2.14489E-06	9.49879E-05	TENM3-AS1	lncRNA
ENSG00000177830.18	3690.74573	-0.02436517	0.051325917	-0.474714744	0.634990307	0.80203393	CHID1	protein_coding
ENSG00000177839.7	159.5115454	0.730427806	0.12489285	5.848435726	4.96217E-09	6.82134E-07	PCDHB9	protein_coding
ENSG00000177842.12	307.9746194	0.111316189	0.071999684	1.546064973	0.122088877	0.31077832	ZNF620	protein_coding
ENSG00000177853.14	2550.724412	-0.089282471	0.070725362	-1.262382671	0.206811122	0.426698944	ZNF518A	protein_coding
ENSG00000177854.8	543.3478886	-0.06854061	0.070599952	-0.970830829	0.331632528	0.561178466	TMEM187	protein_coding
ENSG00000177855.8	50.81635873	0.004139521	0.078748244	0.052566517	0.958077295	0.980464382	CACYBPP2	processed_pseudogene
ENSG00000177868.12	424.1569522	-0.065405291	0.063407428	-1.031508334	0.302302502	0.531903211	SVBP	protein_coding
ENSG00000177873.14	476.7342332	0.032791589	0.047534683	0.68984553	0.490291333	0.69757821	ZNF619	protein_coding
ENSG00000177875.5	63.95958805	0.481589929	0.112387424	4.285087372	1.82667E-05	0.000517225	CCDC184	protein_coding
ENSG00000177879.17	2660.41933	0.112137926	0.056626806	1.980297566	0.047670102	0.172649604	AP3S1	protein_coding
ENSG00000177885.15	7947.678463	0.02528973	0.040647945	0.622165043	0.53383335	0.730981916	GRB2	protein_coding
ENSG00000177888.8	1821.980171	0.133074529	0.065710723	2.025156965	0.042851249	0.16111552	ZBTB41	protein_coding
ENSG00000177889.10	5937.352518	-0.143544399	0.048413044	-2.964994282	0.003026886	0.025848049	UBE2N	protein_coding
ENSG00000177910.8	4.671639037	-0.249826245	0.252711384	-0.988583262	0.322867079	0.552736565	SPATA31C2	transcribed_unprocessed_pseudogene
ENSG00000177917.11	1458.65935	-0.059591876	0.057410755	-1.03799151	0.299274008	0.529160698	ARL6IP6	protein_coding
ENSG00000177932.7	171.9144374	-0.136226742	0.126435059	-1.07744437	0.281281791	0.510173004	ZNF354C	protein_coding
ENSG00000177938.5	6.487892643	-0.564053599	0.29652651	-1.902202939	0.057144629	0.193955252	CAPZA3	protein_coding
ENSG00000177943.14	648.8712579	0.136455478	0.112200324	1.216177219	0.22391742	0.446676558	MAMDC4	protein_coding
ENSG00000177946.6	672.779584	-0.072237131	0.07314744	-0.987555155	0.323370558	0.553150886	CENPBD1	protein_coding
ENSG00000177947.14	3.881546967	0.114262103	0.174565698	0.654550715	0.512757064	0.71531884	ODF3	protein_coding
ENSG00000177951.17	3103.03187	0.059132525	0.048633352	1.215884215	0.224029032	0.446848845	BET1L	protein_coding
ENSG00000177954.14	52005.04531	-0.212615699	0.074793219	-2.842713583	0.004473125	0.034414789	RPS27	protein_coding
ENSG00000177963.14	5153.869813	0.06345076	0.042961655	1.476916117	0.139698124	0.336947205	RIC8A	protein_coding
ENSG00000177971.8	1208.841214	-0.037571746	0.055043611	-0.682581424	0.494871375	0.7014966	IMP3	protein_coding
ENSG00000177981.11	1682.553043	-0.143600001	0.037766134	-3.802348444	0.000143331	0.002623239	ASB8	protein_coding
ENSG00000177984.7	20.53287904	0.64558203	0.285702451	2.2596307	0.023844181	0.10935149	LCN15	protein_coding
ENSG00000177989.13	1337.231574	-0.137180524	0.095427372	-1.437538521	0.150565036	0.352902999	ODF3B	protein_coding
ENSG00000177990.12	109.4878731	0.178854634	0.154084451	1.160757182	0.24574066	0.47224479	DPY19L2	protein_coding
ENSG00000177992.10	3.546894957	-0.255728837	0.304340026	-0.840273427	0.400755097	0.623236318	SPATA31E1	protein_coding
ENSG00000177993.3	17.81021081	-0.175956072	0.128080096	-1.373797161	0.169504663	0.379086717	ZNRF3-AS1	lncRNA
ENSG00000177994.16	4.919011605	0.204118479	0.217022611	0.940540146	0.346940567	0.576255111	C2orf73	protein_coding

ENSG00000178015.5	26.3816582	-0.051133471	0.121165583	-0.42201316	0.673015412	0.826064926	GPR150	protein_coding
ENSG00000178021.11	6.70448748	-0.099915011	0.146687459	-0.681142147	0.495781551	0.702179948	TSPYL6	protein_coding
ENSG00000178026.13	742.353248	-0.132837954	0.113836016	-1.16692378	0.243241151	0.469266801	LRRC75B	protein_coding
ENSG00000178028.14	1497.383599	-0.082435407	0.037993313	-2.169734627	0.030026955	0.127706874	DMAP1	protein_coding
ENSG00000178031.17	449.7370557	0.04477575	0.128019062	0.349758458	0.726519978	0.857355352	ADAMTSL1	protein_coding
ENSG00000178033.6	530.8496095	0.312643143	0.114793439	2.723527987	0.006458874	0.044488682	CALHM5	protein_coding
ENSG00000178035.13	7711.876994	-0.125183276	0.069121724	-1.811055457	0.070132268	0.219946299	IMPDH2	protein_coding
ENSG00000178038.17	1962.556296	0.605486341	0.114057961	5.308584659	1.1048E-07	8.7217E-06	ALS2CL	protein_coding
ENSG00000178053.20	524.3291392	0.030037833	0.165468811	0.181531688	0.855950263	0.929727675	MLF1	protein_coding
ENSG00000178055.13	4.508302961	0.124704998	0.221961663	0.561831249	0.574230999	0.759500539	PRSS42P	transcribed_unitary_pseudogene
ENSG00000178057.15	2301.99315	-0.072874416	0.066107862	-1.102356268	0.270306816	0.498441102	NDUFAF3	protein_coding
ENSG00000178074.6	1542.11182	-0.117630766	0.044528244	-2.641711291	0.008248834	0.052869089	C2orf69	protein_coding
ENSG00000178075.20	445.3931535	-0.088128149	0.101538924	-0.867924792	0.385435506	0.61005332	GRAMD1C	protein_coding
ENSG00000178078.12	2822.735474	-0.146498653	0.100571364	-1.456663681	0.14520924	0.345387843	STAP2	protein_coding
ENSG00000178081.12	2.209694697	0.019329967	0.256231873	0.075439356	0.939865147	0.971569292	ULK4P3	transcribed_unprocessed_pseudogene
ENSG00000178082.6	53.64332174	0.02494371	0.08765723	0.284559646	0.775981531	0.886383258	TWF1P1	processed_pseudogene
ENSG00000178084.2	7.076673915	0.052149855	0.265677976	0.196289719	0.844383408	0.923656294	HTR3C	protein_coding
ENSG00000178093.14	126.0809587	-0.191392998	0.080230139	-2.385549868	0.01705361	0.087367172	TSSK6	protein_coding
ENSG00000178096.9	477.6013393	0.070647008	0.07479066	0.944596658	0.344864834	0.574493477	BOLA1	protein_coding
ENSG00000178104.19	3095.939683	0.174796271	0.057632723	3.032934447	0.002421882	0.021908022	PDE4DIP	protein_coding
ENSG00000178105.11	1903.87361	-0.02923953	0.054884795	-0.532743714	0.594211002	0.774131748	DDX10	protein_coding
ENSG00000178107.2	0.547325984	-0.566787076	0.437126075	-1.296621519	0.194761445		AL161668.1	lncRNA
ENSG00000178115.11	1.683292812	0.202919361	0.21380379	0.949091504	0.342574075	0.572373023	GOLGA8Q	protein_coding
ENSG00000178125.14	4.897289774	-0.122899626	0.205024292	-0.599439336	0.548879952	0.741890251	PPP1R42	protein_coding
ENSG00000178127.13	85.92763962	0.119817822	0.0962245	1.245190375	0.21306178	0.433656851	NDUFV2	protein_coding
ENSG00000178130.9	0.609704429	0.135745711	0.687864285	0.19734374	0.843558553		FAM27E5	lncRNA
ENSG00000178146.9	17.21796845	-0.060795903	0.123865818	-0.490820664	0.623553294	0.794636113	GK4P	processed_pseudogene
ENSG00000178149.17	1130.254441	0.157344408	0.055017166	2.859914827	0.004237548	0.033001881	DALRD3	protein_coding
ENSG00000178150.10	77.55545488	0.518177578	0.193820861	2.673487133	0.007506716	0.049415256	ZNF114	protein_coding
ENSG00000178162.8	40.06167006	-0.225565032	0.232021198	-0.972174242	0.330963873	0.5604929	FAR2P2	transcribed_unprocessed_pseudogene
ENSG00000178163.8	855.0154874	0.123202525	0.123229999	0.999777047	0.317418416	0.546724993	ZNF518B	protein_coding
ENSG00000178171.11	22.24791583	-0.584593884	0.240416416	-2.431588875	0.01503276	0.080250223	AMER3	protein_coding
ENSG00000178172.7	2.581160502	0.934168842	0.254218116	3.674674552	0.000238153	0.003889799	SPINK6	protein_coding
ENSG00000178175.12	210.111923	-0.172535739	0.099744805	-1.729771676	0.083671077	0.247005803	ZNF366	protein_coding
ENSG00000178177.16	1243.407243	-0.145399603	0.060236795	-2.413800444	0.015787106	0.082946073	LCORL	protein_coding
ENSG00000178184.16	402.0543854	0.213535439	0.094235676	2.265972395	0.023453073	0.108206944	PARD6G	protein_coding
ENSG00000178187.8	42.45852638	0.01575891	0.126291421	0.124782113	0.900696049	0.952667784	ZNF454	protein_coding
ENSG00000178188.14	2260.988704	0.01984312	0.053440977	0.37130909	0.710407328	0.848559431	SH2B1	protein_coding
ENSG00000178199.14	588.0690382	-0.490025382	0.121990909	-4.016900821	5.89685E-05	0.001312396	ZC3H12D	protein_coding
ENSG00000178201.4	44.88780633	0.203967159	0.134957586	1.511342681	0.130701165	0.323760815	VN1R1	protein_coding
ENSG00000178202.13	3063.765276	-0.057123263	0.065366868	-0.873887098	0.382179741	0.607486653	POGLUT3	protein_coding
ENSG00000178209.16	77383.76009	0.359950297	0.081274253	4.428835488	9.47433E-06	0.000310657	PLEC	protein_coding

ENSG00000178217.14	11.26492897	-0.197815282	0.163134062	-1.212593368	0.225285326	0.448338067	SH2D4B	protein_coding
ENSG00000178222.13	144.8096645	0.588980017	0.184804332	3.187046597	0.001437336	0.015089087	RNF212	protein_coding
ENSG00000178226.11	152.0877204	0.045398046	0.088177877	0.514846219	0.606660485	0.783064412	PRSS36	protein_coding
ENSG00000178229.8	489.6562304	0.090744108	0.067702234	1.340341407	0.180134376	0.393075397	ZNF543	protein_coding
ENSG00000178233.18	11.57942135	-0.061595183	0.173579785	-0.354852282	0.722700258	0.855233249	TMEM151B	protein_coding
ENSG00000178234.13	1672.299996	-0.046261018	0.053519985	-0.864369038	0.387385196	0.611943909	GALNT11	protein_coding
ENSG00000178235.8	10.31879086	0.009884831	0.235634876	0.041949778	0.966538734	0.984499865	SLITRK1	protein_coding
ENSG00000178243.4	0.417314081	0.158337216	0.546087689	0.289948334	0.771855764		C9orf62	lncRNA
ENSG00000178248.11	6.284203193	-0.427920741	0.303673018	-1.409149699	0.158790908	0.364325351	FAM230I	lncRNA
ENSG00000178252.18	4964.910433	0.126545792	0.059362343	2.131751961	0.033027243	0.135673846	WDR6	protein_coding
ENSG00000178257.4	0.60007953	-0.241089905	0.395503672	-0.609576908	0.542142113		PRM3	protein_coding
ENSG00000178279.4	2.044672	-0.360934678	0.267513544	-1.3492202	0.177266248	0.389544809	TNP2	protein_coding
ENSG00000178287.18	1.637796505	-0.133278518	0.422600324	-0.315377227	0.752475255	0.872926584	SPAG11A	protein_coding
ENSG00000178295.15	1388.217228	-0.023499833	0.069810987	-0.336620837	0.736402746	0.86331471	GEN1	protein_coding
ENSG00000178297.14	48.9525466	-0.057919977	0.106095639	-0.545922319	0.585119335	0.767684635	TMPRSS9	protein_coding
ENSG00000178301.4	247.5269052	-0.310064266	0.0777374	-3.98861122	6.64612E-05	0.001443759	AQP11	protein_coding
ENSG00000178307.10	1421.644024	-0.042021661	0.056417657	-0.744831732	0.456373449	0.670308598	TMEM11	protein_coding
ENSG00000178338.11	392.0469234	-0.062501965	0.06096249	-1.025252832	0.305243905	0.534716742	ZNF354B	protein_coding
ENSG00000178342.4	32.48183642	0.445947656	0.115772328	3.851936494	0.000117187	0.002236827	KCNG2	protein_coding
ENSG00000178343.5	300.4110528	0.197929981	0.217105841	0.91167506	0.361939797	0.589506654	SHISA3	protein_coding
ENSG00000178358.4	1.70372941	-0.235099814	0.25372038	-0.926609892	0.354129117	0.582374871	OR2D3	protein_coding
ENSG00000178363.5	3.374441124	0.074055216	0.428396772	0.172865953	0.862756795	0.933027741	CALML3	protein_coding
ENSG00000178372.8	24.52712293	1.585710412	0.39089722	4.056591686	4.9794E-05	0.001147606	CALML5	protein_coding
ENSG00000178381.12	1083.390707	0.074487603	0.088101385	0.84547596	0.397845149	0.620890773	ZFAND2A	protein_coding
ENSG00000178385.15	1089.259936	0.050344825	0.066892509	0.752622766	0.451676633	0.66630713	PLEKHM3	protein_coding
ENSG00000178386.13	53.39482468	0.049442528	0.098162003	0.503682955	0.614484188	0.788520788	ZNF223	protein_coding
ENSG00000178394.5	1.530645718	-0.070962591	0.413527511	-0.17160307	0.863749593	0.933717578	HTR1A	protein_coding
ENSG00000178395.7	3.199705263	0.740618538	0.232406575	3.186736597	0.001438877	0.015099578	CCDC185	protein_coding
ENSG00000178397.13	851.0795786	0.012534484	0.047021353	0.266570035	0.789800229	0.89441592	FAM220A	protein_coding
ENSG00000178401.16	1904.694885	-0.088520892	0.090037905	-0.983151398	0.325532931	0.555215207	DNAJC22	protein_coding
ENSG00000178403.4	1.773005996	0.964702294	0.369268455	2.612468738	0.008989091	0.056023689	NEUROG2	protein_coding
ENSG00000178404.10	28.65284167	0.294065978	0.125578571	2.341689158	0.019196695	0.094353079	CEP295NL	protein_coding
ENSG00000178409.14	1052.086511	-0.017052463	0.072912546	-0.233875574	0.815081577	0.908711589	BEND3	protein_coding
ENSG00000178412.4	2.222562507	-0.001672277	0.174961164	-0.009557991	0.992373943	0.996465388	AC068473.1	lncRNA
ENSG00000178425.14	2203.530201	-0.10867019	0.050614	-2.147038168	0.031790243	0.132490764	NT5DC1	protein_coding
ENSG00000178429.9	111.8404396	-0.135030366	0.087297945	-1.546775989	0.121917272	0.310471538	RPS3AP5	processed_pseudogene
ENSG00000178440.7	20.66519846	-0.139890293	0.096474483	-1.450023769	0.147051891	0.348094968	TIMM23B-AGAP6	protein_coding
ENSG00000178445.10	410.8490374	-0.713561338	0.22348554	-3.192874755	0.00140864	0.014891877	GLDC	protein_coding
ENSG00000178449.9	1150.912274	-0.207412725	0.059844514	-3.465860305	0.000528538	0.007096936	COX14	protein_coding
ENSG00000178457.3	0.895715564	-0.435144761	0.362691271	-1.199766293	0.230230118		LINC00314	lncRNA
ENSG00000178458.5	169.7079008	0.301967663	0.142765204	2.115134883	0.034418453	0.139313851	H3P16	processed_pseudogene
ENSG00000178460.18	98.23070949	-0.268173917	0.124340547	-2.156769643	0.031023618	0.130481245	MCMDC2	protein_coding

ENSG00000178462.12	98.33855102	-0.205176063	0.228694513	-0.897162161	0.369632393	0.596285892	TUBAL3	protein_coding
ENSG00000178464.6	730.0754176	-0.131708012	0.087547369	-1.504419989	0.132473261	0.326330542	RPL10P16	processed_pseudogene
ENSG00000178467.18	1924.890023	0.079462597	0.058108065	1.367496871	0.171469618	0.381952232	P4HTM	protein_coding
ENSG00000178473.7	27.29635587	0.815412631	0.273860964	2.977469368	0.002906387	0.025049767	UCN3	protein_coding
ENSG00000178498.16	981.8214079	0.001793491	0.116083641	0.015449993	0.98767318	0.994152034	DTX3	protein_coding
ENSG00000178502.6	721.8859422	0.179499096	0.085899187	2.089648374	0.036649398	0.145148669	KLHL11	protein_coding
ENSG00000178503.6	1.28790144	-0.206663965	0.201811833	-1.02404285	0.305815036	0.535384265	NECAP1P1	processed_pseudogene
ENSG00000178522.14	2.94846269	-0.528007573	0.322795329	-1.635734861	0.10189509	0.278527308	AMBN	protein_coding
ENSG00000178531.6	477.586926	0.37349455	0.142874675	2.614141028	0.00894521	0.055853084	CTXN1	protein_coding
ENSG00000178537.10	1076.134142	-0.235209219	0.072186963	-3.258333762	0.001120685	0.012534932	SLC25A20	protein_coding
ENSG00000178538.10	1003.082816	0.002369606	0.175409245	0.013509012	0.989221696	0.995090347	CA8	protein_coding
ENSG00000178556.8	2.067324579	-0.141434932	0.165661614	-0.853758019	0.39323908	0.61733498	CKS1BP6	processed_pseudogene
ENSG00000178562.18	303.5382224	-0.365406781	0.128207096	-2.850129136	0.004370148	0.033783564	CD28	protein_coding
ENSG00000178567.8	2044.468061	-0.132014913	0.093282886	-1.415210428	0.157006808	0.362041356	EPM2AIP1	protein_coding
ENSG00000178568.15	36.53671129	-0.32347902	0.208158616	-1.554002545	0.12018381	0.30791051	ERBB4	protein_coding
ENSG00000178573.7	3047.150925	0.093921396	0.095620742	0.982228267	0.325987405	0.555770544	MAF	protein_coding
ENSG00000178585.15	1525.13551	-0.043555463	0.070958036	-0.613820016	0.539334269	0.735029537	CTNNBIP1	protein_coding
ENSG00000178586.5	0.560097478	-0.347413284	0.479289718	-0.724850276	0.468543889		OR6B3	protein_coding
ENSG00000178591.7	0.701459525	0.684599938	0.515924839	1.326937348	0.184529409		DEFB125	protein_coding
ENSG00000178596.10	1.20248457	0.014091176	0.250653773	0.056217688	0.95516839	0.979204931	GAPDHP29	processed_pseudogene
ENSG00000178597.7	441.0077502	-0.415795241	0.265318517	-1.567155001	0.117078466	0.30273629	PSAPL1	protein_coding
ENSG00000178602.8	0.502440925	0.240361476	0.465850785	0.515962372	0.60588069		OTOS	protein_coding
ENSG00000178605.13	2410.443395	-0.1602489	0.059324622	-2.701220772	0.006908546	0.046579427	GTPBP6	protein_coding
ENSG00000178605.13_PAR_Y	0						GTPBP6	protein_coding
ENSG00000178607.16	2490.415772	-0.16602114	0.06139834	-2.704000449	0.00685102	0.046343819	ERN1	protein_coding
ENSG00000178623.12	2493.777525	-0.0629062	0.092840741	-0.677571071	0.498043693	0.70391963	GPR35	protein_coding
ENSG00000178631.7	35.7499944	0.130224505	0.077106408	1.688893417	0.091239857	0.260298797	ACTG1P1	processed_pseudogene
ENSG00000178636.8	6.043378437	-0.269184629	0.128073456	-2.101798749	0.035570909	0.142210564	AC092656.1	transcribed_processed_pseudogene
ENSG00000178645.13	2.548141779	0.086110894	0.30351506	0.283712097	0.776631023	0.886761434	C10orf53	protein_coding
ENSG00000178654.11	6.15362511	-0.414853321	0.148588463	-2.791961861	0.005238953	0.038433983	PPIAP33	processed_pseudogene
ENSG00000178660.6	17.10660975	0.007702238	0.103440376	0.074460658	0.940643845	0.971959235	ARMC10P1	processed_pseudogene
ENSG00000178662.16	202.6504414	0.247041441	0.134279454	1.839756075	0.065804057	0.211413199	CSRNP3	protein_coding
ENSG00000178665.16	199.3171208	0.0345581	0.081516075	0.423942144	0.671608008	0.825156822	ZNF713	protein_coding
ENSG00000178685.14	4153.799864	-0.078188631	0.076746009	-1.018797356	0.308299181	0.53775581	PARP10	protein_coding
ENSG00000178690.3	7.031900015	-0.277677888	0.426748391	-0.650682918	0.515251192	0.716870509	DYNAP	protein_coding
ENSG00000178691.11	3843.384014	-0.025619814	0.05589841	-0.458328127	0.64671672	0.809384948	SUZ12	protein_coding
ENSG00000178694.10	1114.468895	0.045985514	0.049104427	0.936484088	0.349024001	0.578194979	NSUN3	protein_coding
ENSG00000178695.6	10988.42593	0.114423309	0.11721905	0.976149439	0.328990415	0.558445595	KCTD12	protein_coding
ENSG00000178700.9	551.9171031	-0.02800979	0.055640269	-0.503408595	0.61467703	0.788582476	DHFR2	protein_coding
ENSG00000178715.8	121.5572248	-0.085520468	0.102927627	-0.830879624	0.406041635	0.6275418	AL450998.1	processed_pseudogene
ENSG00000178718.7	2556.562556	0.020074991	0.084638584	0.237184864	0.812513378	0.90740378	RPP25	protein_coding
ENSG00000178719.17	13611.88397	-0.034032177	0.073494471	-0.46305765	0.643323045	0.807177689	GRINA	protein_coding

ENSG00000178722.13	8.239172729	-0.144691041	0.195388941	-0.740528302	0.458979495	0.67221044	C5orf64	lncRNA
ENSG00000178723.7	9.158011897	0.021481303	0.11887586	0.18070366	0.856600186	0.93008587	GLULP4	processed_pseudogene
ENSG00000178726.7	2601.756803	0.169230631	0.103892882	1.628895339	0.103335177	0.280878271	THBD	protein_coding
ENSG00000178732.5	21.09469241	0.249984602	0.141073865	1.772012141	0.076392542	0.23310025	GP5	protein_coding
ENSG00000178734.5	8.412016472	0.182049235	0.196976688	0.924217157	0.355373264	0.583280631	LMO7DN	lncRNA
ENSG00000178741.12	8040.286475	-0.187237666	0.065876664	-2.842245707	0.004479695	0.034456365	COX5A	protein_coding
ENSG00000178750.3	200.3285597	-0.035880319	0.153849815	-0.233216521	0.815593277	0.90895425	STX19	protein_coding
ENSG00000178752.16	170.5197927	0.849619198	0.143475438	5.921704875	3.18621E-09	4.79271E-07	ERFE	protein_coding
ENSG00000178761.15	547.2991984	0.07256948	0.057658374	1.258611274	0.208170773	0.428145849	FAM219B	protein_coding
ENSG00000178762.4	0.191323815	-0.063427449	0.689657942	-0.091969432	0.926722326		H2BC2P	unprocessed_pseudogene
ENSG00000178764.8	1878.876617	0.154266556	0.070759875	2.18014173	0.029246957	0.125368351	ZHX2	protein_coding
ENSG00000178772.7	8.323683278	-0.419077446	0.20629753	-2.031422505	0.042212153	0.159593875	CPN2	protein_coding
ENSG00000178773.15	564.7599507	-0.025417208	0.159610428	-0.159245287	0.873475625	0.938755898	CPNE7	protein_coding
ENSG00000178776.5	39.14882471	0.930589388	0.235153228	3.957374501	7.57781E-05	0.001600196	C5orf46	protein_coding
ENSG00000178789.9	54.96747339	0.072192055	0.129342223	0.55814763	0.576743578	0.761437117	CD300LB	protein_coding
ENSG00000178795.9	14.80647489	-0.259259844	0.155609964	-1.666087684	0.095695951	0.267831886	GDPD4	protein_coding
ENSG00000178796.13	23.182573	-0.628293943	0.194603241	-3.228589316	0.001244024	0.013562881	RIIAD1	protein_coding
ENSG00000178802.18	2025.547434	0.010416014	0.052855496	0.197065862	0.843775998	0.923352196	MPI	protein_coding
ENSG00000178803.12	32.67050483	-0.304869129	0.175353263	-1.738599692	0.082105201	0.244087955	ADORA2A-AS1	lncRNA
ENSG00000178804.7	0.79007333	0.450453558	0.35111094	1.282937976	0.199513807		H1-8	protein_coding
ENSG00000178809.11	10.30178376	-0.283794578	0.115260058	-2.462210956	0.013808342	0.075698343	TRIM73	protein_coding
ENSG00000178814.17	3073.398858	-0.148962664	0.108400188	-1.374191941	0.169382102	0.378929301	OPLAH	protein_coding
ENSG00000178821.13	384.5253054	-0.174040171	0.145565926	-1.195610643	0.231848536	0.455985181	TMEM52	protein_coding
ENSG00000178826.11	278.5417355	-0.451911262	0.130256777	-3.469387701	0.000521646	0.007018145	TMEM139	protein_coding
ENSG00000178828.7	403.8601993	-0.064786046	0.212580606	-0.304759907	0.760549019	0.877433944	RNF186	protein_coding
ENSG00000178836.6	0.911596427	-0.477513707	0.325490034	-1.467060917	0.142359485		AC114812.1	transcribed_processed_pseudogene
ENSG00000178852.16	201.1843736	-0.081189414	0.086090769	-0.943067586	0.345646334	0.575179681	EFCAB13	protein_coding
ENSG00000178860.8	596.4624087	0.19379176	0.10702747	1.810673094	0.07019147	0.220068616	MSC	protein_coding
ENSG00000178878.13	2012.113033	0.040056893	0.105006789	0.381469558	0.702854856	0.843911557	APOLD1	protein_coding
ENSG00000178882.15	3728.450787	-0.341498669	0.166035637	-2.056779342	0.039707456	0.153358899	RFLNA	protein_coding
ENSG00000178896.9	1016.268658	-0.141149269	0.078517042	-1.7976896	0.07222621	0.224173901	EXOSC4	protein_coding
ENSG00000178904.19	1712.039509	0.107131768	0.06464619	1.657201573	0.097478729	0.27130211	DPY19L3	protein_coding
ENSG00000178913.8	4866.086467	0.075275362	0.058325865	1.290600016	0.196842411	0.414071085	TAF7	protein_coding
ENSG00000178917.16	221.8647569	-0.033669112	0.06259517	-0.537886749	0.590655237	0.771575385	ZNF852	protein_coding
ENSG00000178919.9	16.89008009	0.413410357	0.291916518	1.416193778	0.156718777	0.361627437	FOXE1	protein_coding
ENSG00000178921.14	1937.629481	-0.016251993	0.063890071	-0.254374313	0.799206394	0.899829915	PFAS	protein_coding
ENSG00000178922.17	0						HYI	protein_coding
ENSG00000178927.18	3857.770143	0.018765042	0.051309983	0.365719118	0.714574679	0.850855339	CYBC1	protein_coding
ENSG00000178928.8	1.598668435	0.051297285	0.32742666	0.156668015	0.875506496	0.939862002	TPRX1	protein_coding
ENSG00000178934.5	96.49658467	1.295755562	0.277179956	4.674780898	2.94268E-06	0.000122243	LGALS7B	protein_coding
ENSG00000178935.5	550.3010329	-0.171554054	0.066769798	-2.569336135	0.010189357	0.061227511	ZNF552	protein_coding
ENSG00000178947.9	96.702398	0.212524168	0.154024716	1.379805611	0.167646505	0.376729153	SMIM10L2A	protein_coding

ENSG00000178950.17	8905.762671	0.030538574	0.062437678	0.489104895	0.624767436	0.795183438	GAK	protein_coding
ENSG00000178951.9	11387.64959	0.063065241	0.059220283	1.064926359	0.286909256	0.516491671	ZBTB7A	protein_coding
ENSG00000178952.11	9331.156451	-0.042355126	0.062128762	-0.681731366	0.49540883	0.701936201	TUFM	protein_coding
ENSG00000178965.14	34.77849254	-0.590108777	0.225641005	-2.615255044	0.008916085	0.055736447	ERICH3	protein_coding
ENSG00000178966.17	1074.023269	-0.040394448	0.064583792	-0.62545797	0.531670526	0.72921161	RMI1	protein_coding
ENSG00000178971.16	1780.036341	-0.070059642	0.053019085	-1.321404199	0.186366627	0.40121028	CTC1	protein_coding
ENSG00000178974.10	2516.837014	0.113800296	0.054291242	2.096107794	0.03607263	0.143647719	FBXO34	protein_coding
ENSG00000178977.3	157.9499481	-0.179516217	0.074474401	-2.410441915	0.015933208	0.083472158	LINC00324	lncRNA
ENSG00000178980.15	9069.601601	-0.053673271	0.074105557	-0.724281335	0.468893034	0.680259404	SELENOW	protein_coding
ENSG00000178982.10	8885.588167	-0.166704387	0.061209554	-2.723502729	0.006459368	0.044488682	EIF3K	protein_coding
ENSG00000178988.11	2316.53699	0.009855395	0.046003652	0.214230703	0.830367154	0.916560944	MRFAP1L1	protein_coding
ENSG00000178996.14	2278.704227	0.036308352	0.068039657	0.533635151	0.593593984	0.773559168	SNX18	protein_coding
ENSG00000178997.11	10.01270902	0.213504309	0.220793571	0.966986077	0.333550994	0.563116057	EXD1	protein_coding
ENSG00000178999.13	1438.019805	-0.178525707	0.097996876	-1.821748953	0.068493086	0.216881502	AURKB	protein_coding
ENSG00000179002.5	1.462376786	-0.21847611	0.321172576	-0.680245222	0.496349203	0.702572897	TAS1R2	protein_coding
ENSG00000179008.9	6.581886203	-0.253981141	0.269818602	-0.941303304	0.34654945	0.575921707	C14orf39	protein_coding
ENSG00000179010.14	8646.694638	0.069144341	0.046841124	1.476146051	0.139904689	0.337165503	MRFAP1	protein_coding
ENSG00000179021.10	1339.826632	-0.006931929	0.043102479	-0.160824367	0.872231733	0.938226491	C3orf38	protein_coding
ENSG00000179023.8	398.5516069	-0.283398906	0.175319409	-1.616471946	0.105992314	0.285130383	KLHDC7A	protein_coding
ENSG00000179028.4	0.078158842	-0.18555351	0.786039544	-0.236061292	0.81338511		AC245177.1	lncRNA
ENSG00000179029.15	446.6678249	-0.02273045	0.062795285	-0.361977016	0.717369206	0.852280973	TMEM107	protein_coding
ENSG00000179031.8	12.0358021	-0.122489521	0.13160538	-0.930733388	0.351991494	0.580641264	NSA2P3	processed_pseudogene
ENSG00000179038.8	49.22906324	-0.041367662	0.108039495	-0.382893884	0.701798447	0.843230145	AP001885.1	transcribed_processed_pseudogene
ENSG00000179041.4	1710.697587	-0.10008453	0.077974172	-1.283560016	0.199295948	0.417514519	RRS1	protein_coding
ENSG00000179044.16	268.8577027	-0.029745559	0.075199336	-0.395556144	0.692432495	0.837682505	EXOC3L1	protein_coding
ENSG00000179046.8	9.045580792	0.469158135	0.285834734	1.641361525	0.100722392	0.276727801	TRIML2	protein_coding
ENSG00000179051.14	10780.26898	-0.01408966	0.06315494	-0.223096724	0.823460228	0.913038715	RCC2	protein_coding
ENSG00000179055.7	1.996073534	-0.198093724	0.314066754	-0.630737641	0.528212083	0.726497918	OR13D1	protein_coding
ENSG00000179057.14	50.16402167	0.032229092	0.091634909	0.351711944	0.725054302	0.856523863	IGSF22	protein_coding
ENSG00000179058.7	47.88533107	0.157058364	0.135214328	1.161551195	0.245417816	0.471854056	C9orf50	protein_coding
ENSG00000179059.10	9.879040628	0.271211831	0.345287194	0.78546739	0.432179583	0.650179164	ZFP42	protein_coding
ENSG00000179066.8	130.6576164	0.502089844	0.183292763	2.739278055	0.006157427	0.043027936	AC020907.1	lncRNA
ENSG00000179071.5	37.31130498	0.083492566	0.111312163	0.750075857	0.453209019	0.667663605	CCDC89	protein_coding
ENSG00000179073.6	40.62066966	-0.45888855	0.184535351	-2.486724351	0.012892525	0.072138875	TAAR3P	transcribed_unprocessed_pseudogene
ENSG00000179082.3	15.76676478	0.145830947	0.16992794	0.858192874	0.39078597	0.61515299	C9orf106	lncRNA
ENSG00000179083.6	22.98382944	0.526472049	0.255020355	2.064431478	0.038976825	0.151515886	FAM133A	protein_coding
ENSG00000179085.8	1373.582916	0.07847286	0.082791418	0.947838093	0.343211885	0.572920242	DPM3	protein_coding
ENSG00000179088.15	27.41542677	-0.477269557	0.165004204	-2.892469076	0.003822268	0.030702853	C12orf42	protein_coding
ENSG00000179091.5	10565.7509	-0.217433643	0.078025904	-2.786685349	0.005325016	0.038875558	CYC1	protein_coding
ENSG00000179094.16	3734.923797	0.081267309	0.110706944	0.734075989	0.462902427	0.675262606	PER1	protein_coding
ENSG00000179097.6	14.05683078	0.288154241	0.131246505	2.195519342	0.028126365	0.122077949	HTR1F	protein_coding
ENSG00000179101.5	45.67631789	-0.036884833	0.095773566	-0.3851254	0.70014451	0.842148481	H3P47	processed_pseudogene

ENSG00000179104.9	1619.427862	-0.087952065	0.081024967	-1.085493369	0.277703199	0.506669363	TMTC2	protein_coding
ENSG00000179111.9	13.15227751	0.590273453	0.22752248	2.594352234	0.009476934	0.058165684	HES7	protein_coding
ENSG00000179115.11	3791.519578	-0.015474369	0.060512341	-0.255722537	0.798165092	0.899075217	FARSA	protein_coding
ENSG00000179119.15	2417.639583	0.064000981	0.048547665	1.318312253	0.187399141	0.402419177	SPTY2D1	protein_coding
ENSG00000179131.7	10.11102623	-0.344574984	0.107448305	-3.206890811	0.001341779	0.014352162	AC073335.1	processed_pseudogene
ENSG00000179133.15	14.5592949	-0.135138487	0.206084379	-0.655743475	0.511989191	0.714659438	C10orf67	protein_coding
ENSG00000179134.15	10305.21428	0.357256814	0.059658342	5.988379877	2.11942E-09	3.41627E-07	SAMD4B	protein_coding
ENSG00000179136.7	6.468182827	-0.341756538	0.193297199	-1.768036683	0.077054771	0.234369775	LINC00670	lncRNA
ENSG00000179141.9	3.414989914	0.178554359	0.235689833	0.757581932	0.4487013	0.663727259	MTUS2-AS1	lncRNA
ENSG00000179142.2	1.160085759	0.403833882	0.497366906	0.811943611	0.416823989	0.637203205	CYP11B2	protein_coding
ENSG00000179144.5	704.3845642	-0.492115322	0.110323091	-4.460673804	8.17024E-06	0.000275568	GIMAP7	protein_coding
ENSG00000179148.10	48.8945818	0.460300307	0.183900173	2.502990074	0.0123149	0.069775892	ALOXE3	protein_coding
ENSG00000179151.13	1568.774001	0.010053425	0.057063201	0.176180524	0.860152121	0.931968696	EDC3	protein_coding
ENSG00000179152.20	1744.105476	-0.025069339	0.048329463	-0.518717512	0.603957748	0.781333393	TCAIM	protein_coding
ENSG00000179157.4	10.36246284	-0.136993007	0.101456527	-1.350263121	0.176931597	0.389224505	RPS2P28	processed_pseudogene
ENSG00000179163.12	4625.478613	-0.141961331	0.070589407	-2.011085466	0.04431643	0.164649763	FUCA1	protein_coding
ENSG00000179165.11	5.671111818	-0.152373558	0.143973092	-1.058347476	0.289897056	0.51972041	PXT1	protein_coding
ENSG00000179168.15	58.93556758	0.160544884	0.092212483	1.741032006	0.081677969	0.243253201	GGN	protein_coding
ENSG00000179170.11	0.298977693	0.059191548	0.689143307	0.085891493	0.931552674		OR7E97P	unprocessed_pseudogene
ENSG00000179172.10	4.267100935	-0.173649626	0.108591586	-1.599107566	0.109796704	0.290787101	HNRNPCL1	protein_coding
ENSG00000179178.11	1341.690224	0.154894469	0.094348887	1.641720159	0.100648012	0.276645542	TMEM125	protein_coding
ENSG00000179195.16	8772.632583	-0.033227314	0.055065364	-0.603415868	0.546232069	0.740210179	ZNF664	protein_coding
ENSG00000179213.13	1.624673755	-0.151120053	0.394412905	-0.383151899	0.701607141	0.843070023	SIGLECL1	protein_coding
ENSG00000179218.14	45358.21653	0.005460082	0.059975102	0.09103914	0.927461491	0.965674498	CALR	protein_coding
ENSG00000179219.5	11.06083868	-0.033254166	0.168053623	-0.197878306	0.843140278	0.922991532	LINC00311	lncRNA
ENSG00000179222.18	9741.949675	0.323687129	0.090596261	3.572853077	0.000353113	0.005256818	MAGED1	protein_coding
ENSG00000179240.13	364.3797304	-0.048748214	0.097957145	-0.497648379	0.618731898	0.791175604	GVQW3	protein_coding
ENSG00000179241.13	953.5492949	0.266276411	0.099629901	2.672655577	0.007525347	0.049491707	LDLRAD3	protein_coding
ENSG00000179242.16	71.17020141	0.060179327	0.140870195	0.427197018	0.669235833	0.823639562	CDH4	protein_coding
ENSG00000179253.3	2.924310052	-0.012001031	0.221178372	-0.054259514	0.956728405	0.979774246	AL162457.1	lncRNA
ENSG00000179256.2	1.672628672	-0.249926859	0.185341975	-1.348463341	0.177509403	0.389788827	SMCO3	protein_coding
ENSG00000179262.10	5897.6528	0.06873999	0.054785858	1.254703176	0.209586533	0.429734735	RAD23A	protein_coding
ENSG00000179270.7	5.103525916	-0.184902138	0.257622493	-0.717725132	0.472926784	0.683766771	PCARE	protein_coding
ENSG00000179271.3	2786.305413	-0.103381654	0.068866912	-1.501180334	0.133308925	0.327610891	GADD45GIP1	protein_coding
ENSG00000179277.9	221.898388	0.587478983	0.125039996	4.698328548	2.62299E-06	0.000111896	MEIS3P1	processed_pseudogene
ENSG00000179284.5	19.5028221	-0.481881599	0.159358305	-3.023887577	0.002495492	0.022402008	DAND5	protein_coding
ENSG00000179292.5	382.2789892	0.647349298	0.215733678	3.000687256	0.002693711	0.023707107	TMEM151A	protein_coding
ENSG00000179295.18	8854.971786	-0.01647886	0.051376102	-0.320749517	0.748400219	0.870656104	PTPN11	protein_coding
ENSG00000179299.17	274.545533	0.15094091	0.138478614	1.089994374	0.275715622	0.504444524	NSUN7	protein_coding
ENSG00000179300.4	15.05042713	-0.155245768	0.222900691	-0.696479528	0.486128574	0.693789302	RTL3	protein_coding
ENSG00000179304.17	8.376265022	-0.036636412	0.089558937	-0.409076	0.682483888	0.832144779	FAM156B	protein_coding
ENSG00000179314.14	206.1400507	0.233364992	0.14651717	1.592748431	0.111216638	0.293282144	WSCD1	protein_coding

ENSG00000179331.3	58.40620958	0.17405545	0.151758891	1.146920942	0.251414297	0.478028815	RAB39A	protein_coding
ENSG00000179335.18	2585.366314	-0.03444884	0.0377078	-0.913573336	0.360941082	0.588660442	CLK3	protein_coding
ENSG00000179342.4	8.083327308	0.088764021	0.1442679	0.61527215	0.538375007	0.734356602	AC008267.1	processed_pseudogene
ENSG00000179344.16	9458.024879	-0.402961165	0.143341082	-2.811204997	0.004935633	0.036859985	HLA-DQB1	protein_coding
ENSG00000179348.12	460.0175855	0.19977368	0.108051483	1.848874947	0.064475872	0.20893911	GATA2	protein_coding
ENSG00000179361.18	1056.359996	0.012834903	0.077687231	0.165212511	0.868776718	0.936421752	ARID3B	protein_coding
ENSG00000179362.14	104.4003036	0.093221173	0.102609431	0.908504918	0.363611516	0.590970426	HMGN2P46	transcribed_processed_pseudogene
ENSG00000179363.7	2.312976387	-0.383649636	0.234738355	-1.634371321	0.102180906	0.278964413	TMEM31	protein_coding
ENSG00000179364.14	4746.907594	0.079501922	0.047545028	1.672139537	0.09449681	0.26572159	PACS2	protein_coding
ENSG00000179381.8	0.382397693	-0.616498735	0.54085093	-1.139868124	0.254341247		OR4K11P	unprocessed_pseudogene
ENSG00000179387.10	1541.915119	0.090796337	0.050279736	1.805823667	0.070945887	0.221684277	ELMOD2	protein_coding
ENSG00000179388.9	871.8745845	0.548068165	0.135259583	4.051972911	5.07875E-05	0.001164826	EGR3	protein_coding
ENSG00000179397.18	34.99108315	-0.133915798	0.10417186	-1.285527572	0.198607988	0.416432168	CATSPERE	protein_coding
ENSG00000179399.15	10.44487247	0.395636827	0.192876876	2.051240335	0.040243548	0.154537634	GPC5	protein_coding
ENSG00000179403.12	10162.11633	-0.033567932	0.092780207	-0.361800569	0.717501067	0.852340945	VWA1	protein_coding
ENSG00000179406.8	931.7060154	-0.098310715	0.09288657	-1.058395358	0.289875235	0.51970267	LINC00174	lncRNA
ENSG00000179407.3	1.155540972	-0.074637711	0.29087712	-0.256595332	0.797491179	0.898688211	DNAJB8	protein_coding
ENSG00000179409.11	2149.924957	0.085786273	0.065470304	1.310308147	0.190091613	0.405682071	GEMIN4	protein_coding
ENSG00000179412.11	1.467445663	-0.170339373	0.166931658	-1.020413835	0.307532235	0.537036948	HNRNPCL4	protein_coding
ENSG00000179420.11	2.77329892	-0.536626271	0.314132852	-1.708278099	0.087584753	0.25402031	OR6W1P	transcribed_unprocessed_pseudogene
ENSG00000179428.2	4.574777342	0.04700313	0.187679669	0.250443376	0.802244489	0.901619851	IL6-AS1	lncRNA
ENSG00000179431.7	479.9998229	0.292598783	0.097248408	3.008777108	0.002623015	0.023274916	FJX1	protein_coding
ENSG00000179443.5	0.602524324	-0.03324091	0.514168817	-0.064649797	0.948452835		OR13C6P	unprocessed_pseudogene
ENSG00000179447.2	10.76998978	0.165085422	0.166301718	0.992686208	0.320862898	0.550612055	SLC24A3-AS1	lncRNA
ENSG00000179452.2	0.34702331	-0.079640535	0.503708622	-0.158108342	0.874371428		LINC01699	lncRNA
ENSG00000179454.14	1096.549589	0.071860471	0.048762558	1.473681327	0.140567413	0.338361801	KLHL28	protein_coding
ENSG00000179455.10	104.4284293	0.63124943	0.257398849	2.452417454	0.014189993	0.077096027	MKRN3	protein_coding
ENSG00000179456.10	1751.956351	-0.133302661	0.073675199	-1.809328826	0.070399933	0.220595232	ZBTB18	protein_coding
ENSG00000179460.5	2.437677998	-0.270071851	0.203788729	-1.325254111	0.185086884	0.399620033	EEF1A1P27	processed_pseudogene
ENSG00000179468.5	0.762930494	-0.557684656	0.407227726	-1.36946632	0.170853556		OR9A2	protein_coding
ENSG00000179476.8	351.7081271	-0.075650078	0.063520177	-1.1909614	0.233668739	0.458262044	C14orf28	protein_coding
ENSG00000179477.11	52.7039231	0.321614206	0.205719203	1.563365023	0.117966762	0.304116761	ALOX12B	protein_coding
ENSG00000179520.11	15.13983524	-0.089874872	0.286895944	-0.313266444	0.754078242	0.873716234	SLC17A8	protein_coding
ENSG00000179523.5	376.1552594	0.025639364	0.057864414	0.443093817	0.657697877	0.81623788	EIF3J-DT	lncRNA
ENSG00000179526.17	4089.730139	-0.081755416	0.062187674	-1.314656276	0.188625448	0.403878299	SHARPIN	protein_coding
ENSG00000179528.16	125.2067032	-0.05448957	0.116571051	-0.467436556	0.640187574	0.805237521	LBX2	protein_coding
ENSG00000179532.13	604.1900875	-0.044523362	0.0897067	-0.496321482	0.619667612	0.791768232	DNHD1	protein_coding
ENSG00000179542.16	57.76245041	0.542495753	0.170214609	3.187128031	0.001436932	0.015088474	SLITRK4	protein_coding
ENSG00000179546.4	546.9407708	0.02916423	0.195295717	0.149333688	0.881290334	0.943007707	HTR1D	protein_coding
ENSG00000179562.3	1506.005683	-0.022373712	0.045982076	-0.486574647	0.626559794	0.796510871	GCC1	protein_coding
ENSG00000179564.4	7.682034815	0.243464998	0.118738868	2.050423783	0.040323094	0.154734015	LSMEM2	protein_coding
ENSG00000179571.10	3.467193252	-0.245264614	0.217795541	-1.126123213	0.26011337	0.487337003	NBPF17P	unprocessed_pseudogene

ENSG00000179577.3	2.649065301	-0.842015554	0.358294448	-2.350065871	0.018770089	0.09293764	AP003471.1	lncRNA
ENSG00000179580.10	2.045205619	-0.128315313	0.174383734	-0.73582157	0.461839293	0.674426824	RNF151	protein_coding
ENSG00000179583.19	3775.128593	-0.501824641	0.132826637	-3.778042192	0.000158066	0.002841603	CIITA	protein_coding
ENSG00000179588.9	4573.749598	-0.164703817	0.099258721	-1.659338503	0.097047601	0.27043377	ZFPM1	protein_coding
ENSG00000179593.16	184.6311325	0.2742878	0.157224264	1.744564056	0.081060782	0.242110055	ALOX15B	protein_coding
ENSG00000179598.6	198.0174632	-0.220704469	0.08134295	-2.713258735	0.006662506	0.045435374	PLD6	protein_coding
ENSG00000179600.4	1.167586138	-0.715428364	0.404150549	-1.77020263	0.076693391	0.233678361	GPHB5	protein_coding
ENSG00000179603.18	64.98128087	0.376374171	0.151271226	2.488075095	0.012843661	0.071976365	GRM8	protein_coding
ENSG00000179604.10	6286.284323	-0.004916881	0.073770618	-0.06665094	0.946859592	0.975098675	CDC42EP4	protein_coding
ENSG00000179611.3	191.3967589	-0.006288398	0.072949485	-0.086202095	0.931305765	0.967380072	DGKZP1	processed_pseudogene
ENSG00000179615.3	0.618296444	0.106675708	0.408977848	0.260834929	0.794219807		OR2AP1	protein_coding
ENSG00000179626.4	1.437335862	0.539411497	0.333792644	1.616007743	0.106092639	0.28513962	OR6C4	protein_coding
ENSG00000179627.10	862.803126	0.112012753	0.078147119	1.433357423	0.15175572	0.354433939	ZBTB42	protein_coding
ENSG00000179630.11	826.7322705	-0.00373023	0.070024771	-0.053270148	0.957516665	0.980171548	LACC1	protein_coding
ENSG00000179632.10	6937.899359	-0.098856112	0.056526827	-1.748835326	0.080319489	0.240687825	MAF1	protein_coding
ENSG00000179636.15	2.458629071	-0.425105125	0.220891714	-1.92449557	0.054292496	0.187576633	TPPP2	protein_coding
ENSG00000179639.10	106.9609968	-0.243847264	0.206083014	-1.18324776	0.23671097	0.461714066	FCER1A	protein_coding
ENSG00000179673.5	6.432393323	-0.396330137	0.209995704	-1.887324976	0.059116629	0.197951936	RPRML	protein_coding
ENSG00000179674.4	1208.70912	0.368212896	0.176987381	2.080447174	0.037484536	0.147464443	ARL14	protein_coding
ENSG00000179676.7	1.366422662	-0.263723552	0.415897792	-0.634106641	0.526011229	0.724920662	LINC00305	lncRNA
ENSG00000179695.3	1.271451433	-0.019328042	0.46163515	-0.041868652	0.966603406	0.984499865	OR6C2	protein_coding
ENSG00000179698.14	112.9866289	-0.098798825	0.138577977	-0.712947521	0.475878232	0.686039211	WDR97	protein_coding
ENSG00000179709.8	4.382229997	-0.06732856	0.331821369	-0.20290604	0.839208473	0.920933909	NLRP8	protein_coding
ENSG00000179715.13	679.7140357	-0.235668408	0.111669326	-2.110413091	0.03482279	0.140298882	PCED1B	protein_coding
ENSG00000179743.4	157.4335123	0.015296031	0.070134551	0.218095518	0.827354689	0.914973995	FLJ37453	lncRNA
ENSG00000179750.16	423.235644	-0.082707171	0.137418317	-0.601864239	0.54726451	0.740848452	APOBEC3B	protein_coding
ENSG00000179751.7	4.175838754	-1.449239078	0.356080938	-4.069970963	4.7019E-05	0.001092772	SYCN	protein_coding
ENSG00000179755.3	3.342662799	-0.113430435	0.188667811	-0.601217738	0.547694971	0.741100464	AC135776.1	lncRNA
ENSG00000179761.12	326.5261409	0.139966129	0.180693567	0.774604933	0.438573142	0.655312165	PIPOX	protein_coding
ENSG00000179766.19	94.98677034	-0.059668176	0.115804218	-0.515250454	0.606378016	0.782983727	ATP8B5P	transcribed_unprocessed_pseudogene
ENSG00000179772.8	350.7029386	0.241089599	0.129392097	1.86324826	0.062427346	0.204962646	FOXS1	protein_coding
ENSG00000179774.9	10.30511688	0.081195917	0.146906324	0.552705393	0.58046516	0.764316879	ATOH7	protein_coding
ENSG00000179776.19	3169.724141	0.021053211	0.091467013	0.230172717	0.817957561	0.910217994	CDH5	protein_coding
ENSG00000179796.12	14.37165436	-0.212041993	0.184782593	-1.14752147	0.251166165	0.477779612	LRRC3B	protein_coding
ENSG00000179799.9	63.34405868	0.516836462	0.22286799	2.319025099	0.020393675	0.098503125	OR7E22P	transcribed_unprocessed_pseudogene
ENSG00000179813.7	33.27095994	-0.55209741	0.188432362	-2.92995006	0.003390165	0.028123589	FAM216B	protein_coding
ENSG00000179817.5	0.868616153	0.138602078	0.45227012	0.306458623	0.759255479		MRGPRX4	protein_coding
ENSG00000179818.14	1113.203313	-0.157080588	0.064580179	-2.432334334	0.015001853	0.080134313	PCBP1-AS1	lncRNA
ENSG00000179820.16	14652.42598	0.031014952	0.097982106	0.316536905	0.751595018	0.872375074	MYADM	protein_coding
ENSG00000179826.7	2.232716272	0.644459527	0.279365316	2.306870215	0.021062056	0.10062136	MRGPRX3	protein_coding
ENSG00000179832.17	5352.318293	-0.006302858	0.06672471	-0.094460632	0.924743254	0.964540482	MROH1	protein_coding
ENSG00000179833.4	2267.048938	0.088019656	0.060870863	1.44600639	0.14817541	0.349687891	SERTAD2	protein_coding

ENSG00000179840.5	16.02942526	-0.802492709	0.159221858	-5.040091357	4.6531E-07	2.78145E-05	PIK3CD-AS1	lncRNA
ENSG00000179841.8	284.6218347	-0.17807869	0.094616289	-1.882114499	0.059820471	0.199559673	AKAP5	protein_coding
ENSG00000179846.10	49.30486572	0.591916556	0.151477994	3.907607581	9.32145E-05	0.001874485	NKPD1	protein_coding
ENSG00000179855.9	425.665716	0.000822335	0.080124567	0.010263201	0.991811294	0.996273665	GIPC3	protein_coding
ENSG00000179859.9	234.7466137	-0.082342055	0.066565774	-1.237002887	0.216086011	0.437279656	RNF227	protein_coding
ENSG00000179862.7	1616.969113	0.385480251	0.132048368	2.919235256	0.003508913	0.028877791	CITED4	protein_coding
ENSG00000179869.15	270.6177752	-0.273055656	0.187182377	-1.458767971	0.144628981	0.344481959	ABCA13	protein_coding
ENSG00000179873.14	29.28142492	-0.941647964	0.196404538	-4.794430783	1.63137E-06	7.60325E-05	NLRP11	protein_coding
ENSG00000179886.6	966.2269623	0.069638744	0.07716032	0.902520161	0.366780616	0.593829486	TIGD5	protein_coding
ENSG00000179889.19	10645.98823	0.074511726	0.061294021	1.215644269	0.224120463	0.446979286	PDXDC1	protein_coding
ENSG00000179899.8	78.04331101	0.120770248	0.103470688	1.167192858	0.243132495	0.46915754	PHC1P1	processed_pseudogene
ENSG00000179902.13	19.41056937	-0.202979399	0.144599897	-1.403731282	0.160398884	0.366413407	C1orf194	protein_coding
ENSG00000179909.15	348.3593339	-0.14902276	0.105106986	-1.417819742	0.156243399	0.361015321	ZNF154	protein_coding
ENSG00000179912.20	2295.561727	-0.076636434	0.051957149	-1.474993056	0.14021441	0.337762485	R3HDM2	protein_coding
ENSG00000179913.11	7203.500486	0.197439065	0.102244395	1.93105026	0.053476837	0.185582554	B3GNT3	protein_coding
ENSG00000179914.5	1447.234502	0.103401114	0.279831343	0.369512267	0.711745931	0.849244023	ITLN1	protein_coding
ENSG00000179915.24	130.5780681	-0.531375407	0.219388141	-2.422078992	0.015431996	0.081621565	NRXN1	protein_coding
ENSG00000179918.19	9822.438245	-0.354643078	0.084595851	-4.19220414	2.76257E-05	0.000715298	SEPHS2	protein_coding
ENSG00000179919.3	0.192341495	-0.278707204	0.722664597	-0.385666054	0.699744006		OR10A7	protein_coding
ENSG00000179921.15	362.1109365	-0.053889833	0.144479737	-0.372992323	0.709154158	0.847836998	GPBAR1	protein_coding
ENSG00000179922.6	438.8953294	-0.024372048	0.06089527	-0.400228915	0.68898792	0.835661616	ZNF784	protein_coding
ENSG00000179930.6	5.728106406	0.359957254	0.255858463	1.406860849	0.159468655	0.365307893	ZNF648	protein_coding
ENSG00000179933.6	2688.909249	-0.00372372	0.050386538	-0.073903067	0.941087515	0.972119954	C14orf119	protein_coding
ENSG00000179934.7	49.54240157	-0.094852263	0.133721667	-0.709326057	0.478122162	0.687773822	CCR8	protein_coding
ENSG00000179935.10	20.77794048	-0.095567911	0.122074794	-0.782863583	0.433707225	0.651522669	LINC00652	lncRNA
ENSG00000179938.12	0.764096178	-0.116873864	0.364630682	-0.320526685	0.748569105		GOLGA8J	protein_coding
ENSG00000179941.9	711.6553333	-0.020219053	0.079845963	-0.253225746	0.800093772	0.900334118	BBS10	protein_coding
ENSG00000179943.8	1322.06619	0.00866435	0.057444072	0.150831059	0.880108985	0.942252641	FIZ1	protein_coding
ENSG00000179950.14	8128.067773	-0.026210797	0.059030085	-0.444024389	0.657024951	0.815703028	PUF60	protein_coding
ENSG00000179954.16	2034.359535	0.0909328	0.160479736	0.566631046	0.570964878	0.757370027	SSC5D	protein_coding
ENSG00000179958.10	2564.567059	-0.151033637	0.074421455	-2.029436772	0.042413823	0.160106303	DCTPP1	protein_coding
ENSG00000179965.12	437.2139665	0.069486172	0.069874832	0.994437761	0.320009792	0.549710924	ZNF771	protein_coding
ENSG00000179967.11	249.8227135	0.080506952	0.087983873	0.91501941	0.360181441	0.588055778	PPP1R14BP3	processed_pseudogene
ENSG00000179978.11	50.0187285	-0.10024075	0.095148245	-1.053521792	0.292101912	0.521886101	NAIPP2	unprocessed_pseudogene
ENSG00000179981.11	2450.625601	-0.102999987	0.080488134	-1.279691581	0.200653627	0.419214737	TSHZ1	protein_coding
ENSG00000179988.15	219.4212716	-0.196923234	0.063049492	-3.12331197	0.001788281	0.017660636	PSTK	protein_coding
ENSG00000179994.11	7.987716565	-0.344085183	0.187317043	-1.836913381	0.066222684	0.212252566	SPDYE7P	transcribed_unprocessed_pseudogene
ENSG00000179997.8	1.312487167	-0.386385371	0.199056055	-1.941088252	0.052247574	0.182801993	RPA3P1	processed_pseudogene
ENSG00000180008.9	1800.89389	0.14030816	0.054336627	2.582202217	0.009817204	0.059665873	SOCS4	protein_coding
ENSG00000180011.7	2124.379945	0.010107723	0.058352813	0.173217404	0.862480544	0.932948383	ZADH2	protein_coding
ENSG00000180015.12	24.60355334	0.110178422	0.099822053	1.103748299	0.269702338	0.497747703	AC093909.1	processed_pseudogene
ENSG00000180016.2	0.790633877	0.19829849	0.407687641	0.48639809	0.626684944		OR1E1	protein_coding

ENSG00000180019.6	2.321017301	-0.165158605	0.169329792	-0.975366488	0.3293785	0.558829312	AC079741.1	transcribed_processed_pseudogene
ENSG00000180035.13	682.8991367	0.130801467	0.060342751	2.167641777	0.030185953	0.12810803	ZNF48	protein_coding
ENSG00000180042.5	0.794178181	-0.855501579	0.375953539	-2.275551338	0.022872877		OR1R1P	protein_coding
ENSG00000180043.11	1.390487277	0.869356429	0.355101077	2.448194289	0.014357421	0.077691959	FAM71E2	protein_coding
ENSG00000180044.5	232.8473074	0.195137774	0.114749587	1.700553178	0.089026923	0.256531522	C3orf80	protein_coding
ENSG00000180053.7	0.780062383	0.509224043	0.401345175	1.268793235	0.204514818		NKX2-6	protein_coding
ENSG00000180061.10	650.7317269	-0.313710667	0.140167761	-2.238108567	0.025213976	0.113742178	TMEM150B	protein_coding
ENSG00000180066.10	69.24640759	0.742858995	0.166427498	4.463559226	8.06093E-06	0.000272726	LINC02870	lncRNA
ENSG00000180068.10	0.500767436	-0.231192581	0.491776206	-0.470117461	0.6382711		OR3A4P	transcribed_unprocessed_pseudogene
ENSG00000180071.20	164.3152778	0.242861744	0.168753425	1.439151498	0.150107604	0.352323045	ANKRD18A	protein_coding
ENSG00000180083.11	0.857139918	0.245435664	0.434138339	0.56533976	0.571842689		WFDC11	protein_coding
ENSG00000180089.6	137.7193338	-0.541348477	0.12002005	-4.510483679	6.468E-06	0.000231029	TMEM86B	protein_coding
ENSG00000180090.5	2.043483971	-0.827033423	0.3156338	-2.620230856	0.008787026	0.055142891	OR3A1	protein_coding
ENSG00000180096.12	470.311723	-0.695064164	0.121335316	-5.728457206	1.01348E-08	1.20339E-06	SEPTIN1	protein_coding
ENSG00000180098.9	837.0598333	-0.079882384	0.045294257	-1.763631628	0.077794019	0.235791701	TRNAU1AP	protein_coding
ENSG00000180104.16	3596.830338	0.016580876	0.047128957	0.35181928	0.724973798	0.856504444	EXOC3	protein_coding
ENSG00000180105.11	3.25175932	-0.226801978	0.201109149	-1.127755643	0.259423131	0.486650098	ACER2P1	processed_pseudogene
ENSG00000180113.16	180.4442433	-0.208173121	0.120598957	-1.726160212	0.084318594	0.248049456	TDRD6	protein_coding
ENSG00000180116.15	6.001039488	-0.179566782	0.201888973	-0.889433333	0.373770237	0.59974172	C12orf40	protein_coding
ENSG00000180138.7	4.792855621	0.017666897	0.113469469	0.15569736	0.876271578	0.940196396	CSNK1A1L	protein_coding
ENSG00000180139.11	165.5867155	-0.21654342	0.17135631	-1.26370263	0.206336781	0.426139237	ACTA2-AS1	lncRNA
ENSG00000180150.5	0.760150098	-0.794432251	0.271091478	-2.930495112	0.003384223		HMGN2P9	processed_pseudogene
ENSG00000180152.3	14.41942962	0.2809593	0.217992644	1.28884762	0.197451067	0.414786211	XIAPP3	processed_pseudogene
ENSG00000180155.20	458.2720716	0.366305446	0.159407754	2.297914852	0.021566636	0.102208732	LYNX1	protein_coding
ENSG00000180172.7	39.63954901	-0.194487368	0.111046478	-1.751405102	0.079876155	0.239937272	RPS12P23	processed_pseudogene
ENSG00000180176.15	38.94792106	1.170383816	0.219766628	5.325575702	1.00634E-07	8.09099E-06	TH	protein_coding
ENSG00000180178.11	53.58274682	0.332053988	0.27092762	1.225618813	0.220342155	0.442338881	FAR2P1	transcribed_unprocessed_pseudogene
ENSG00000180182.11	6652.082074	-0.076082346	0.058505863	-1.300422607	0.193456166	0.410028807	MED14	protein_coding
ENSG00000180185.11	1947.183292	-0.116961073	0.061144542	-1.912862042	0.055765716	0.190730917	FAHD1	protein_coding
ENSG00000180189.10	17.08078508	-0.038882779	0.090635586	-0.429001242	0.667922321	0.822713321	HMGB1P14	processed_pseudogene
ENSG00000180190.12	201.7289473	0.089786056	0.134050963	0.669790459	0.502991376	0.707647512	TDRP	protein_coding
ENSG00000180198.16	3272.944323	-0.111490237	0.079270799	-1.406447745	0.159591211	0.365386331	RCC1	protein_coding
ENSG00000180205.4	1.225871154	-0.782043505	0.451904709	-1.730549582	0.083532131	0.246818528	WFDC9	protein_coding
ENSG00000180209.12	23.28469149	0.128575576	0.120533507	1.066720612	0.286098017	0.515539564	MYLPF	protein_coding
ENSG00000180210.15	37.38877345	0.121455786	0.230811099	0.526212937	0.598740266	0.777707371	F2	protein_coding
ENSG00000180211.5	206.7479188	-0.160480207	0.087206962	-1.840222429	0.065735588	0.211328642	FO393411.1	processed_pseudogene
ENSG00000180219.2	2.129564925	-0.490161852	0.318531348	-1.538818253	0.123848672	0.313284588	FAM71C	protein_coding
ENSG00000180221.6	9.268753373	-0.105779972	0.126471052	-0.83639671	0.402931763	0.62474853	TPT1P10	processed_pseudogene
ENSG00000180228.14	1723.20892	0.057138133	0.043759935	1.305717953	0.19164849	0.407786439	PRKRA	protein_coding
ENSG00000180229.12	219.1447795	-0.116610265	0.140580041	-0.829493747	0.406825073	0.628067293	HERC2P3	transcribed_unprocessed_pseudogene
ENSG00000180230.6	4.048083993	-0.244328703	0.133972134	-1.823727781	0.068193237	0.216372264	NACAP2	processed_pseudogene
ENSG00000180233.11	1902.477914	-0.088180001	0.053818233	-1.638478214	0.101321975	0.277639924	ZNRF2	protein_coding

ENSG00000180245.7	22.45532644	0.073718296	0.124225159	0.593424845	0.552896866	0.744505507	RRH	protein_coding
ENSG00000180251.5	566.6192857	-0.475413104	0.267308175	-1.778520632	0.075318386	0.230863438	SLC9A4	protein_coding
ENSG00000180257.14	562.9244055	0.043617382	0.080877013	0.539305062	0.589676377	0.771046989	ZNF816	protein_coding
ENSG00000180259.10	2.997474723	0.146731864	0.3350586	0.437928958	0.661437783	0.818639389	PRNT	lncRNA
ENSG00000180263.14	2866.209367	0.083032429	0.08011851	1.036370099	0.300029514	0.529649024	FGD6	protein_coding
ENSG00000180264.11	15.70503019	0.912304216	0.199006321	4.584297685	4.55515E-06	0.000173211	ADGRD2	protein_coding
ENSG00000180269.8	2.079695017	-0.079775266	0.333431552	-0.239255301	0.810907624	0.906400285	GPR139	protein_coding
ENSG00000180279.7	13.63434187	-0.254350465	0.13802996	-1.842719249	0.065370012	0.210723131	LINC01869	transcribed_unitary_pseudogene
ENSG00000180284.5	1.125857558	0.249692719	0.208839206	1.195621853	0.23184416	0.455985181	CNEP1R1P1	processed_pseudogene
ENSG00000180287.17	22.97349592	-0.369332834	0.244923752	-1.507950251	0.13156726	0.324988312	PLD5	protein_coding
ENSG00000180304.14	3033.356487	0.004170464	0.049068349	0.08499295	0.932266995	0.967967424	OAZ2	protein_coding
ENSG00000180305.5	3.138274946	-0.880967374	0.276452595	-3.186685127	0.001439134	0.015099578	WFDC10A	protein_coding
ENSG00000180316.12	34.39553156	-0.045563394	0.173713243	-0.262290852	0.793097214	0.896262994	PNPLA1	protein_coding
ENSG00000180318.4	11.34971993	0.42064691	0.429774497	0.978761917	0.327697631	0.557262132	ALX1	protein_coding
ENSG00000180329.14	1033.845598	0.003442657	0.052156198	0.066006668	0.947372517	0.975304216	CCDC43	protein_coding
ENSG00000180332.6	5.721882795	-0.13213253	0.171679332	-0.769647275	0.441509154	0.658086558	KCTD4	protein_coding
ENSG00000180336.18	44.17169389	0.068065243	0.138910359	0.489994005	0.624138141	0.794924329	MEIOC	protein_coding
ENSG00000180340.7	796.7687984	0.418570143	0.118139804	3.543006902	0.000395592	0.005689375	FZD2	protein_coding
ENSG00000180346.4	615.7316833	0.071767146	0.058600223	1.224690662	0.220691794	0.442816387	TIGD2	protein_coding
ENSG00000180347.13	62.45223052	-0.872365997	0.215388787	-4.050192246	5.11756E-05	0.001171259	ITPRID1	protein_coding
ENSG00000180353.11	2044.03029	-0.33146396	0.109066167	-3.039108909	0.002372791	0.021602254	HCLS1	protein_coding
ENSG00000180354.16	1410.809412	0.087417363	0.106895785	0.81778119	0.413482141	0.634068031	MTURN	protein_coding
ENSG00000180357.9	4102.750829	0.031135083	0.048429614	0.642893489	0.520293214	0.720503921	ZNF609	protein_coding
ENSG00000180370.10	9204.472355	0.106838412	0.042240627	2.529280911	0.011429651	0.066298022	PAK2	protein_coding
ENSG00000180376.17	721.29466	-0.135689307	0.058706629	-2.311311508	0.020815655	0.099855107	CCDC66	protein_coding
ENSG00000180383.3	3.85107665	-0.062556466	0.210511149	-0.297164624	0.766340829	0.881316222	DEFB124	protein_coding
ENSG00000180385.8	159.7113932	0.155573782	0.073865638	2.106172591	0.035189363	0.141299488	EMC3-AS1	transcribed_unprocessed_pseudogene
ENSG00000180386.7	0.267422667	0.090497714	0.632989079	0.142968839	0.886314791		KRTAP9-7	protein_coding
ENSG00000180389.7	71.07614563	-0.143422203	0.112749191	-1.2720464	0.203356634	0.422630916	ATP5F1EP2	processed_pseudogene
ENSG00000180398.13	5359.538265	0.08884008	0.04646553	1.911956688	0.05588175	0.191022827	MCFD2	protein_coding
ENSG00000180409.3	1.795344967	-0.100439557	0.396778062	-0.253137878	0.800161669	0.900334118	OR10AA1P	unprocessed_pseudogene
ENSG00000180422.3	4.804916879	0.015661662	0.177857004	0.088057606	0.929830891	0.96676374	LINC00304	lncRNA
ENSG00000180423.4	257.7596994	-0.024872234	0.04728764	-0.525977492	0.598903845	0.777782856	HARBI1	protein_coding
ENSG00000180424.7	1.889267538	-0.717903737	0.417907831	-1.717851844	0.085823642	0.25079334	DEFB123	protein_coding
ENSG00000180425.11	275.6652473	-0.142825613	0.076379011	-1.86995892	0.061489523	0.202994616	C11orf71	protein_coding
ENSG00000180432.6	10.01364429	0.066124791	0.174915034	0.378039492	0.705401257	0.845554012	CYP8B1	protein_coding
ENSG00000180433.5	0.27653165	-0.357765971	0.779642446	-0.458884676	0.646316984		OR6K6	protein_coding
ENSG00000180437.5	0.154300677	0.198613772	1.005834753	0.197461632	0.843466304		OR6K4P	unprocessed_pseudogene
ENSG00000180438.15	333.0208432	0.787704926	0.224936039	3.501906265	0.000461942	0.006421693	TPRXL	transcribed_processed_pseudogene
ENSG00000180440.4	5.950629534	-0.591429848	0.322156953	-1.835843812	0.06638076	0.212681006	SERTM1	protein_coding
ENSG00000180447.7	1091.842072	0.641364867	0.169287374	3.788616078	0.000151489	0.002749449	GAS1	protein_coding
ENSG00000180448.10	2451.938557	-0.266346757	0.091977015	-2.895796935	0.00378197	0.030457757	ARHGAP45	protein_coding

ENSG00000180458.2	4.110562251	-0.907936622	0.215438035	-4.214374776	2.50471E-05	0.000658707	AC022148.1	lncRNA
ENSG00000180475.4	3.427282706	-0.275072678	0.290160609	-0.948001452	0.343128715	0.57287614	OR10Q1	protein_coding
ENSG00000180479.14	218.4520571	-0.117722208	0.08133266	-1.447416185	0.147780396	0.349179218	ZNF571	protein_coding
ENSG00000180481.11	30.77500917	-0.22887549	0.117796101	-1.942980175	0.052018554	0.182454324	GLIPR1L2	protein_coding
ENSG00000180483.7	1.752882082	0.242669837	0.307388512	0.789456428	0.429845287	0.648099372	DEFB119	protein_coding
ENSG00000180488.15	1695.062935	0.015425807	0.054263798	0.284274364	0.77620013	0.886501719	MIGA1	protein_coding
ENSG00000180509.12	26.38920903	0.077800811	0.125345917	0.620688837	0.534804376	0.731808399	KCNE1	protein_coding
ENSG00000180525.13	754.6489754	-0.18215117	0.11132032	-1.636279609	0.101781082	0.278407771	PRR26	lncRNA
ENSG00000180530.11	2795.32204	0.074828452	0.069492874	1.076778782	0.281579107	0.510482981	NRIP1	protein_coding
ENSG00000180532.10	28.69337416	0.524022633	0.20019419	2.61757163	0.00885579	0.055430732	ZSCAN4	protein_coding
ENSG00000180535.4	479.1203175	-0.41681793	0.162551787	-2.564216227	0.01034091	0.061816273	BHLHA15	protein_coding
ENSG00000180537.13	45.1102012	-0.103568933	0.216203574	-0.479034322	0.631914212	0.800167663	RNF182	protein_coding
ENSG00000180539.8	33.97145812	-0.304266266	0.096298921	-3.159602037	0.001579848	0.016119181	C9orf139	lncRNA
ENSG00000180543.5	921.981493	-0.156589437	0.159917927	-0.97918626	0.327487956	0.557038255	TSPYL5	protein_coding
ENSG00000180549.8	15.41853756	-0.458175822	0.140958538	-3.250429725	0.001152307	0.012803187	FUT7	protein_coding
ENSG00000180573.9	2014.330641	-0.139419551	0.106599299	-1.307884311	0.190912552	0.406776661	H2AC6	protein_coding
ENSG00000180574.3	145.2015293	-0.110261256	0.106642903	-1.033929618	0.301169068	0.530779117	EIF2S3B	protein_coding
ENSG00000180581.7	47.31812219	0.129381858	0.069299651	1.866991486	0.061902777	0.20386467	SRP9P1	processed_pseudogene
ENSG00000180592.17	53.13389414	-0.291007412	0.126250501	-2.30500005	0.021166571	0.100938891	SKIDA1	protein_coding
ENSG00000180596.7	219.5733005	-0.077877653	0.132806347	-0.586400088	0.557606685	0.747932091	H2BC4	protein_coding
ENSG00000180610.10	41.12643359	0.007789428	0.097992051	0.079490404	0.936642564	0.969894566	ZBTB12BP	processed_pseudogene
ENSG00000180611.7	518.2065675	0.033057922	0.088001699	0.375650953	0.707176406	0.846685251	MB21D2	protein_coding
ENSG00000180613.11	1.569989353	-0.180392019	0.293683646	-0.614239237	0.539057249	0.734812867	GSX2	protein_coding
ENSG00000180616.9	214.9359955	0.045007964	0.116823307	0.385265276	0.700040885	0.842148481	SSTR2	protein_coding
ENSG00000180626.10	267.1897816	0.090384553	0.086391631	1.046218855	0.29546	0.525263561	ZNF594	protein_coding
ENSG00000180628.15	5569.22366	-0.069348798	0.053009259	-1.308239329	0.190792146	0.406601762	PCGF5	protein_coding
ENSG00000180638.19	31.76979813	-0.36564647	0.138356165	-2.642791296	0.00822257	0.052747673	SLC47A2	protein_coding
ENSG00000180644.8	684.8639154	-0.263057715	0.1303336	-2.018341505	0.043555707	0.162655501	PRF1	protein_coding
ENSG00000180658.4	1.811702703	-0.19835263	0.197707724	-1.003261915	0.315734507	0.544858332	OR2A4	protein_coding
ENSG00000180660.8	18.6223723	0.331645211	0.191721397	1.729828887	0.083660852	0.246997895	MAB21L1	protein_coding
ENSG00000180662.8	11.16175536	-0.328792024	0.114473423	-2.872212741	0.004076085	0.032217441	RPL21P8	processed_pseudogene
ENSG00000180663.5	0.222784897	0.213937324	0.657238327	0.325509508	0.744795488		VN1R3	unitary_pseudogene
ENSG00000180667.10	1534.957856	0.26016712	0.090618622	2.871011672	0.004091604	0.032283992	YOD1	protein_coding
ENSG00000180673.9	9.580403885	0.202707884	0.101498411	1.997153282	0.045808534	0.168256785	EXOC5P1	processed_pseudogene
ENSG00000180694.14	1776.173947	0.049182015	0.090759809	0.541892006	0.587892904	0.769850721	TMEM64	protein_coding
ENSG00000180697.9	1.903707108	0.387939949	0.309288433	1.254298278	0.20973361	0.42995538	C3orf22	protein_coding
ENSG00000180708.6	4.028640365	-0.329346752	0.398709632	-0.826031592	0.408786171	0.629737119	OR10K2	protein_coding
ENSG00000180712.4	52.56830001	-0.224234928	0.124691374	-1.798319492	0.072126393	0.223976842	LINC02363	lncRNA
ENSG00000180714.3	1.370360995	0.462066182	0.349615353	1.321641561	0.186287537	0.401143	OR5AZ1P	unprocessed_pseudogene
ENSG00000180720.7	31.38938206	0.106881953	0.164115931	0.651258853	0.514879405	0.716604913	CHRM4	protein_coding
ENSG00000180723.6	0.729307518	-0.419781934	0.419102489	-1.001621192	0.316526582		OR51A9P	unitary_pseudogene
ENSG00000180730.5	545.7498465	1.236883415	0.156187215	7.919235978	2.38974E-15	2.72862E-12	SHISA2	protein_coding

ENSG00000180739.14	75.78581795	0.340241075	0.1159718	2.933825925	0.003348119	0.027874286	S1PR5	protein_coding
ENSG00000180745.5	1229.148786	-0.170214182	0.164127883	-1.037082664	0.299697334	0.529481071	CLRN3	protein_coding
ENSG00000180747.15	232.5085229	-0.006687641	0.084667528	-0.078987084	0.937042896	0.970147738	SMG1P3	transcribed_unprocessed_pseudogene
ENSG00000180758.12	1232.872238	0.13494792	0.087342668	1.545040048	0.122336577	0.311082328	GPR157	protein_coding
ENSG00000180764.13	78.66853562	0.057732326	0.060885815	0.948206509	0.343024334	0.572813154	PIPSL	transcribed_processed_pseudogene
ENSG00000180767.10	323.7197862	-0.258286851	0.204416907	-1.263529788	0.206398848	0.426166435	CHST13	protein_coding
ENSG00000180769.10	104.9440502	0.150225061	0.08935152	1.681281533	0.092708243	0.262948914	WDFY3-AS2	lncRNA
ENSG00000180770.3	1.0740398	-0.355653973	0.364539872	-0.975624342	0.329250657	0.558767988	OR7E129P	unprocessed_pseudogene
ENSG00000180772.7	5.441132215	-0.248379433	0.38201623	-0.650180317	0.515575754	0.717092108	AGTR2	protein_coding
ENSG00000180773.15	806.7868086	0.489918177	0.096125418	5.096655907	3.45706E-07	2.18656E-05	SLC36A4	protein_coding
ENSG00000180776.16	9276.586038	0.140776804	0.065142817	2.161048774	0.030691572	0.129531245	ZDHHC20	protein_coding
ENSG00000180777.14	9.376874682	-0.243156091	0.262928576	-0.924799026	0.355070457	0.583088069	ANKRD30B	protein_coding
ENSG00000180785.10	122.7679376	-0.141602994	0.118602049	-1.193933794	0.232503867	0.456902864	OR51E1	protein_coding
ENSG00000180787.6	608.1795367	0.074404718	0.112047204	0.664047966	0.506659612	0.710657458	ZFP3	protein_coding
ENSG00000180801.14	631.4462307	0.240690621	0.106978326	2.249900802	0.024455243	0.111345327	ARSJ	protein_coding
ENSG00000180803.3	0.160506226	0.029151468	0.909588836	0.032049061	0.974432926		AL157366.1	processed_pseudogene
ENSG00000180806.5	237.4314457	0.021435783	0.164551834	0.130267664	0.896354663	0.950870411	HOXC9	protein_coding
ENSG00000180815.14	21.64267713	-0.1784605	0.159137701	-1.121421877	0.262108328	0.489558726	MAP3K15	protein_coding
ENSG00000180817.12	11549.39607	-0.087502501	0.065333862	-1.33931316	0.180468746	0.393470084	PPA1	protein_coding
ENSG00000180818.5	852.7551895	0.103372571	0.230380967	0.448702741	0.653646107	0.813873262	HOXC10	protein_coding
ENSG00000180822.12	921.9221513	-0.144744546	0.057571432	-2.514173102	0.011931186	0.068285431	PSMG4	protein_coding
ENSG00000180828.3	90.58007585	-0.159850671	0.144439255	-1.106698252	0.268424412	0.496424917	BHLHE22	protein_coding
ENSG00000180834.8	26.15641816	0.370585656	0.090964308	4.073967723	4.62189E-05	0.001078202	MAP6D1	protein_coding
ENSG00000180846.8	10.38980839	0.080771409	0.119174895	0.67775524	0.497926895	0.703881	CSNK1G2-AS1	lncRNA
ENSG00000180855.16	253.393096	-0.087972563	0.066097336	-1.330954737	0.183203905	0.396969725	ZNF443	protein_coding
ENSG00000180861.9	1074.805702	-0.201324615	0.174167513	-1.155925185	0.24771176	0.474229093	LINC01559	lncRNA
ENSG00000180867.11	728.3607089	0.051462859	0.062042254	0.8294808	0.406832397	0.628067293	PDIA3P1	transcribed_processed_pseudogene
ENSG00000180869.4	14.09874232	-0.706427718	0.249365908	-2.832896139	0.004612837	0.035143063	LINC01555	lncRNA
ENSG00000180871.8	235.5865369	0.460031472	0.191826513	2.398164182	0.016477478	0.085548496	CXCR2	protein_coding
ENSG00000180872.7	0.478361326	0.112277627	0.55000836	0.204138037	0.838245631		DEFB112	protein_coding
ENSG00000180875.5	592.8440023	0.106749952	0.214950372	0.49662604	0.619452786	0.791633012	GREM2	protein_coding
ENSG00000180878.3	18.73004038	0.110299691	0.12484044	0.883525335	0.3769525	0.602674359	C11orf42	protein_coding
ENSG00000180879.14	12033.49122	-0.152419555	0.078350294	-1.945360349	0.051731623	0.181872445	SSR4	protein_coding
ENSG00000180881.19	158.5973918	0.028441765	0.10205504	0.27869045	0.780482392	0.88874039	CAPS2	protein_coding
ENSG00000180884.10	611.7071092	0.161929375	0.065013614	2.490699485	0.01274919	0.071612718	ZNF792	protein_coding
ENSG00000180891.13	2308.095914	0.010205117	0.086503737	0.117973138	0.906088942	0.955375517	CUEDC1	protein_coding
ENSG00000180900.20	10081.76547	0.037299458	0.076811211	0.485599136	0.627251409	0.796963837	SCRIB	protein_coding
ENSG00000180901.11	2141.352531	0.0314378	0.048334268	0.650424663	0.51541795	0.717033841	KCTD2	protein_coding
ENSG00000180902.18	1134.898687	-0.13120062	0.068408667	-1.917894711	0.055124363	0.18939954	D2HGDH	protein_coding
ENSG00000180909.2	0.745253702	0.006740835	0.408574832	0.016498409	0.986836771		OR52B1P	unprocessed_pseudogene
ENSG00000180910.8	0.245510699	0.293719643	0.655998306	0.447744515	0.654337591		TTTY11	transcribed_unprocessed_pseudogene
ENSG00000180913.3	1.021356798	-0.24851946	0.372458341	-0.667240957	0.504618233	0.708683772	OR56B3P	unprocessed_pseudogene

ENSG00000180914.10	200.3920966	0.453397973	0.128900088	3.51743725	0.000435735	0.006139036	OXTR	protein_coding
[truncated: 561,058 more chars]
